# OT-55 reshapes tolerogenic BH3-mimetic-induced apoptosis toward immunogenic cell death in acute myeloid leukemia, potentiating PD-1/Tim-3 blockade

**DOI:** 10.1038/s41419-026-09000-9

**Published:** 2026-07-01

**Authors:** Yejin Lee, Eun-Ji Kwon, Sruthi Reddy Gajulapalli, Ji Yeon Paik, Barbora Orlikova-Boyer, Artur M. S. Silva, Hyuk-Jin Cha, Claudia Cerella, Marc Diederich

**Affiliations:** 1 Department of Pharmacy, College of Pharmacy, Seoul National University, Seoul, Korea; 2 Laboratoire de Biologie Moléculaire du Cancer (LBMCC), Luxembourg, Luxembourg; 3 Department of Chemistry & REQUIMTE, University of Aveiro, Aveiro, Portugal; 4 Present Address: Department of Cancer Research, Luxembourg Institute of Health (LIH), Luxembourg, Luxembourg

**Keywords:** Acute myeloid leukaemia, Acute myeloid leukaemia

## Abstract

BH3 mimetics are apoptogenic but rarely cause immunogenic cell death (ICD), limiting durable antitumor immunity. We hypothesized that an ICD-inducing immunoadjuvant could convert BH3-mimetic-triggered tolerogenic apoptosis into immunogenic priming, enhancing checkpoint immunotherapy and overcoming immune evasion in AML. We in vivo evaluated the hydroxycoumarin OT-55, combined with the Bcl-xL inhibitor A-1331852, to enhance PD-1/Tim-3 blockade in Bcl-xL-dependent murine prophylactic and bilateral AML vaccination models. To define the clinical and immunological context, we derived a nine-gene AML ICD score (*ATG5, CALR, CD8A, CD8B, IFNGR1, IL1B, PDIA3, PIK3CA, TLR4*) by screening 34 ICD-associated genes in transcriptomes of three AML patient cohorts (TARGET-AML, BEAT-AML, GSE37642), retaining genes consistently associated with favorable prognosis (HR < 1, Cox regression). These cohorts were dichotomized by median ICD score to infer immune composition (CIBERSORTx) and profile driver mutations, and to assess blast maturation. High ICD scores were associated with an immune-activated state, increased CD8⁺ T cells, activated dendritic cells, and higher *HAVCR2* (Tim-3) expression, consistent with a survival advantage. Bone marrow scRNA-seq from AML and healthy donors revealed ICD-related and progenitor-to-intermediate exhausted T cells, alongside T cell depletion in myelomonocytic AML. Experimentally, we used murine C1498 myelomonocytic AML cells to evaluate OT-55 combined with A-1331852 by prophylactic whole-cell vaccination for DAMP release, dependency testing (CRT neutralization and apyrase), antigen-specific CD8⁺ responses, and synergy with anti-PD-1/anti-Tim-3 therapy in a bilateral tumor model, while monitoring hematologic and serum parameters. OT-55 reduced C1498 viability, induced CRT exposure and ATP release, and conferred CRT/ATP-dependent, but HMGB1-independent, vaccine protection. While A-1331852 was cytotoxic yet weakly immunogenic, its combination with OT-55 enhanced DAMP release, increased CD8⁺ effector functions, and, with PD-1/Tim-3 blockade, achieved local and distant tumor control with low toxicity. These findings identify OT-55 as an immunogenic adjuvant converting tolerogenic BH3 mimetic-driven apoptosis into ICD, providing a proof-of-concept immunogenic treatment for myelomonocytic AML.

## Introduction

Acute myeloid leukemia (AML) is a highly aggressive hematologic malignancy with a poor prognosis. The National Cancer Institute reports a 5-year overall survival rate of about 32%, with limited effective treatments [1]. Immune system alterations play a key role in AML development and progression, often leading to immune evasion and resistance to current immunotherapies [2]. Improving immune recognition of AML cells could create new therapeutic strategies. Enhancing antigen presentation may improve outcomes alone or combined with existing targeted and immune treatments.

Immunogenic cell death (ICD) is a programmed form of cell death that triggers immune recognition by releasing damage-associated molecular patterns (DAMPs), such as ATP, high-mobility group box 1 (HMGB1), and calreticulin (CRT). These signals alert the immune system, recruit innate immune cells, facilitate tumor antigen uptake by antigen-presenting cells, and promote cross-presentation to T cells, leading to cytokine release and tumor eradication [3]. Strategies that induce ICD hallmarks and activate adaptive immune effectors, such as antigen-presenting cells and cytotoxic T lymphocytes, could enhance antitumor immunity [4].

Although BH3 mimetics exhibit clinical activity in AML, they are poorly immunogenic and susceptible to resistance, especially in genetically altered subtypes. The BCL-2 family inhibits ICD [5, 6]. Apoptosis induced by the BH3 mimetics ABT-263 (navitoclax, targeting Bcl-2, Bcl-xL, and Bcl-w) or ABT-737 (targeting Bcl-2, Bcl-xL, and Bcl-w) increases caspase activation but reduces interferon (IFN) production and weakens anticancer immunity [6, 7]. BH3 mimetics, such as venetoclax, bind to BCL-2, lowering leukemia cell apoptosis thresholds and enhancing chemotherapy sensitivity. As monotherapy, BH3 mimetics show synergistic, durable responses with NK cells [8] or adoptive T cell therapies [9]. However, their inability to trigger true ICD limits their potential to prime durable adaptive antitumor immunity.

To address this limitation, we turned to coumarin derivatives with established ER stress-inducing and ICD-promoting properties. Compounds that induce ICD and DAMP release are under investigation [10]. Coumarins, with various pharmacological effects including anticancer, antiseptic, antiviral, anticoagulant, and anti-inflammatory activities, are of particular interest. The hydroxycoumarin derivative OT52, combined with subtoxic doses of B-cell lymphoma-extra large (Bcl-xL)- or myeloid leukemia 1 (Mcl-1)-targeting BH3 mimetics, induces synergistic ICD [11]. OT-55 induces ER stress, leading to caspase-dependent apoptosis and DAMP release, and enhances macrophage phagocytosis of chronic myeloid leukemia (CML) cells [12]. These properties make OT-55 a compelling candidate to convert BH3-mimetic-triggered apoptosis into ICD in AML, where canonical mitochondria-mediated apoptosis is typically immunologically silent.

To address these shortcomings, we hypothesize that combining OT-55, an ER stress-inducing ICD adjuvant, with BH3 mimetics will convert tolerogenic apoptosis into ICD, generating the danger signals required to activate antigen-presenting cells and prime cytotoxic T lymphocytes in the context of BH3-mimetic-induced dying cells. In this model, OT-55-mediated DAMP release activates antigen-presenting cells and promotes cross-priming of tumor-reactive CD8⁺ T cells, functioning as an in vivo vaccine.

Immune checkpoint inhibitors (ICIs) targeting Hepatitis A virus cellular receptor 2 (HAVCR2), also known as T cell immunoglobulin and mucin-domain containing-3 (Tim-3) and PD-1 (Programmed death-1) and PD-L1 (Programmed death-ligand 1), can reinvigorate progenitor-exhausted T cells [13]. PD-1/PD-L1 interactions promote immune evasion in leukemia models [14], and Tim-3 is elevated in refractory AML [15, 16]. PD-1 and Tim-3 are coexpressed in AML with disseminated disease, marking deeply exhausted CD8⁺ T cells [17]. Emerging immunotherapies, including ICIs and immunomodulatory agents, aim to counteract immune evasion in AML by restoring T cell effector functions, reprogramming the immunosuppressive microenvironment, and synergizing with chemotherapy [18, 19]. This checkpoint-exhausted landscape, particularly pronounced in myelomonocytic AML, which displays a cold tumor microenvironment poor in T cells and characterized by high expression of galectins and *HAVCR2* (Tim-3), as well as *CD274* (PD-L1) upregulation in adult forms, provides a rational basis for combining ICD-inducing vaccination with PD-1 and Tim-3 blockade to sustain and amplify vaccine-primed effector responses [20].

Using bioinformatic profiling, we developed an ICD score that classifies AML cases into high- and low-ICD groups, with upregulation of *HAVCR2* (Tim-3) and *PDCD1* (PD-1) in high-ICD AMLs. We tested whole-cell vaccination with endoplasmic reticulum (ER) stress inducer OT-55 in mice, then evaluated the BH3 mimetic A-1331852, which was cytotoxic against Bcl-xL-overexpressing C1498 cells in vitro but had limited immunogenicity in vivo. OT-55 plus A-1331852-treated C1498 cells enhanced alarmin release and anti-leukemic effects, enabling prophylactic CD8⁺-dependent and antigen-specific vaccination in immunocompetent mice. Using a bilateral tumor assay, a syngeneic two-flank assay used to assess systemic immune control, we further confirmed systemic efficacy.

Altogether, AML is intrinsically immunosuppressive and often immunotherapy-refractory; yet the widely used BH3-mimetic regimens, although cytotoxic, primarily induce canonical mitochondria-mediated apoptosis, a mode of cell death that is typically immunologically silent. Here, OT-55 plus A-1331852 vaccination provides a proof-of-concept for an efficient immunogenic combination treatment that durably primes anti-AML immunity in a myelomonocytic AML context.

## Results

### Prognostic and immunological significance of ICD-related gene signatures in AML patients

**Fig. 1:**
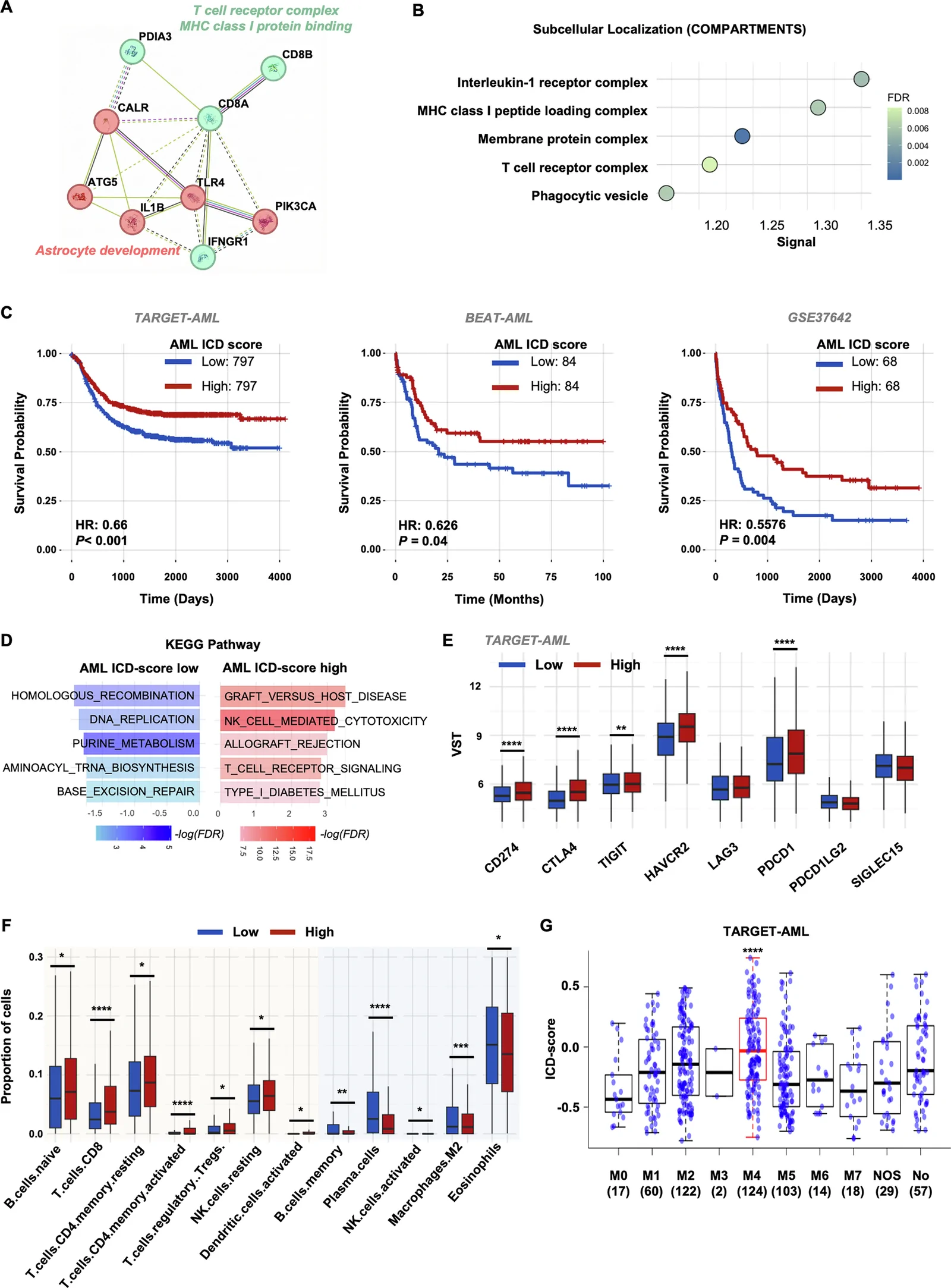
Identification and characterization of an ICD-related gene signature in AML patients. **A** Protein–protein interaction network of the nine ICD-related genes was constructed using STRING. **B** The subcellular compartments associated with the nine identified ICD-related genes were determined using the COMPARTMENTS, subcellular localization database. **C** Kaplan–Meier survival curves for patients stratified by high vs low AML ICD scores in the TARGET-AML, BEAT-AML, and GSE37642 cohorts. **D** KEGG pathway enrichment analysis of differentially expressed genes between AML ICD-score high and low groups. **E** Expression levels of immune checkpoint genes were compared between AML ICD-score high and low groups across the TARGET-AML cohort. **F** Differences in immune cell infiltration between AML ICD-score high and low groups were analyzed using CIBERSORTx. **G** Distribution of AML ICD scores across different French-American-British (FAB) subtypes in the TARGET-AML cohort. Each FAB subtype was compared against all other subtypes. Statistical significance for (**F**) was assessed using unpaired two-tailed Student’s *t*-tests. ^*^P < 0.05, ^**^P < 0.01, ^***^P < 0.001, and ^****^P < 0.0001. Statistical significance for (**E**, **G**) was assessed using unpaired one-tailed Student’s *t*-tests. ^*^P < 0.05, ^**^P < 0.01, ^***^P < 0.001, and ^****^P < 0.0001.

Because AML immune evasion varies with leukemic differentiation and the microenvironment [20, 21], we created a patient-derived transcriptional framework of ICD for mechanistic studies. We examined 34 ICD-related genes [22] across three AML cohorts (TARGET-AML, BEAT-AML, GSE37642). Nine genes consistently associated with better survival (hazard ratio < 1 in all datasets), including *ATG5, CALR, CD8A, CD8B, IFNGR1, IL1B, PDIA3, PIK3CA*, and *TLR4*, present in all cohorts (Supplemental Fig. 1A). These genes are involved in membrane-associated immune complex assembly, such as MHC class I, interleukin-1 receptor, and T cell receptor complexes, which are relevant to immune recognition and antigen presentation (Fig. 1A, B).

Based on evidence linking AML differentiation states to specific immune microenvironments [20], we developed an AML ICD score by constructing an ICD-related gene signature. Briefly, the score was derived by evaluating the prognostic associations of 34 previously reported ICD-related genes across three independent AML cohorts. Genes consistently associated with favorable overall survival (hazard ratio < 1) in all three cohorts were retained, yielding the nine shared prognostically favorable genes listed above. A composite AML ICD score was then calculated for each patient using gene set variation analysis (GSVA) on TPM-normalized expression values of these nine genes, and patients were stratified into ICD-high and ICD-low groups based on cohort-specific median GSVA scores (Supplemental Fig. 1B). Patients with higher scores had significantly improved overall survival in three AML cohorts (TARGET-AML, BEAT-AML, and GSE37642) (Fig. 1C). These findings suggest that elevated ICD gene expression indicates increased ICD activity, potentially leading to better clinical outcomes in AML.

To identify ICD-score-specific differentially expressed genes (DEGs) and assess whether the scores reflect distinct immune microenvironment states, we performed differential expression analysis between ICD-high and ICD-low groups using DESeq2, with DEGs defined as genes with FDR-adjusted *P* < 0.05 and absolute log2 fold-change > 1 (Supplemental Fig. 1C). Gene set enrichment analysis (GSEA) was then performed using fgsea on DESeq2-ranked test statistics to identify pathways enriched in each direction. The gene list of the DEGs driving pathway enrichment is provided in Supplemental Table 1.

KEGG pathway enrichment analysis of DEGs (log2 fold-change > 1) showed that the high AML ICD-score group was enriched in immune signaling pathways like NK cell cytotoxicity, T cell receptor signaling, and graft-versus-host disease, indicating ICD-related immune activation (Fig. 1D). The low ICD-score group was enriched in pathways linked to proliferation and metabolism, such as homologous recombination, DNA replication, purine metabolism, and base excision repair, which are associated with survival and replication rather than immunity. This suggests that reduced ICD-related immune activity in the low-score group may impair immune clearance and increase cellular stress.

The immune dysfunction and editing observed in AML and other malignancies indicate that targeting these immune alterations could render immunotherapy effective for various AML subtypes. Since immune activation can coexist with inhibitory circuits, and our AML microenvironment classification highlighted lineage-associated checkpoint programs, we compared immune checkpoint gene expression across AML ICD score levels (Fig. 1E and Supplemental Fig. 1D). In the TARGET-AML, TCGA-LAML, and BEAT-AML cohorts, *HAVCR2* was consistently upregulated in patients with high AML ICD scores, especially in those with poor prognosis [16]. Bulk RNA-seq analysis also showed increased expression of *CTLA4* and *TIGIT* in high-score groups, indicating adaptive immune reactivity. Additionally, *PDCD1* and *CD274* (PD-L1) were consistently elevated in the TARGET-AML and BEAT-AML cohorts.

Since checkpoint transcripts can reflect changes in immune cell abundance and myeloid-T cell interactions, and given our previous identification of AML-driven immunosuppressive niches with diverse immune infiltration [20], we inferred the immune cell composition associated with the AML ICD score. Comparing tumor-infiltrating immune cells between high and low AML ICD-score groups in TARGET-AML using CIBERSORTx (Fig. 1F), the high-score group showed significantly increased CD8⁺ T cells, resting and activated CD4⁺ memory T cells, and activated dendritic cells, indicating enhanced T cell priming and antigen presentation. Notably, the high-score group also had more resting NK cells, whereas activated NK cells were enriched in the low-score group, suggesting that ICD-high tumors exhibit adaptive immune dominance, whereas ICD-low tumors rely more on innate NK responses. Increased regulatory T cells imply a hyperactivated/exhausted microenvironment. The low-score group also exhibited higher infiltration of M2 macrophages, plasma cells, and memory B cells, associated with immunosuppression and a myeloid-driven suppressive niche, similar to previously described ML/Mo-High AML microenvironment (AME) [20]. Together, these findings suggest that high ICD scores reflect an inflamed, immune-engaged tumor microenvironment, whereas low scores correspond to an immune-cold or suppressive niche.

We evaluated whether AML ICD scores reflect genetic backgrounds by comparing driver mutations between low- and high-ICD-score groups in the TARGET-AML, TCGA-LAML, and BEAT-AML cohorts (Supplemental Fig. 1E), using the updated WHO 2022 classification [23]. Transcription factor mutations were more frequent in the low-score groups across all cohorts. Additionally, TCGA-LAML and BEAT-AML showed higher mutation rates in DNA-methylation regulators within the low-score groups. These mutations disrupt epigenetic programming, impair antigen presentation, and reduce immune activation [24, 25]. Overall, AML cases with lower ICD signatures are enriched for mutations affecting transcription and epigenetic regulation, typically associated with immune-cold or immunosuppressive tumor phenotypes.

Given that blast maturation is a clinical feature contributing to resistance against BH3 mimetics [26, 27], we next examined whether specific AML differentiation lineages are more likely to be associated with distinct ICD-linked immune states. We evaluated AML ICD scoring across French-American-British (FAB) morphological blast subtypes (M0–M7), which remains a consolidated framework for assessing blast differentiation in experimental studies, consistently available on all our investigated public cohorts, although it is no longer routinely used in clinical patient evaluation (Fig. 1G and Supplemental Fig. 1F). AML ICD scores were higher in patients with the M4 myelomonocytic subtype, suggesting that blasts arrested at this stage of myeloid differentiation are more responsive to ICD.

To determine whether the prognostic value of the AML ICD score is confounded by immune cell infiltration, we performed several independent analyses. First, Pearson correlations between the ICD score and the CIBERSORTx-estimated CD8⁺ T cell fraction were weak and non-significant in BEAT-AML (*r* = 0.107, *P* = 0.17) and GSE37642 (*r* = 0.072, *P* = 0.40); in the TARGET-AML cohort, while the correlation reached statistical significance due to the large sample size (*n* = 1594), the effect size remained modest (*r* = 0.19) (Supplemental Fig. 1G). Second, multivariate Cox proportional hazards regression simultaneously including the ICD score and the CD8⁺ T cell fraction confirmed that the ICD score remained a highly significant independent predictor of overall survival in all three cohorts, whereas the CD8⁺ T cell fraction was not significantly associated with survival (*P* = 0.769, 0.507, and 0.838 in TARGET-AML, BEAT-AML, and GSE37642, respectively) (Supplemental Fig. 1G). Third, extending this analysis to all 22 CIBERSORTx-inferred immune cell populations revealed no consistent or reproducible correlation patterns across cohorts (Supplemental Fig. 1H–J). No consistent, reproducible pattern was observed across cohorts, with correlations varying in direction and magnitude across cell types without a unifying signature, indicating that the ICD score does not reflect overall immune infiltration density. These analyses collectively support the interpretation that the ICD score reflects a tumor-intrinsic immunogenic transcriptional program rather than being driven primarily by immune infiltration. Fourth, single-cell resolution analysis of individual M4 and M5 AML patients from the scPCA bone marrow scRNA-seq resource demonstrated that every individual patient examined showed near-complete absence of Naive T cells and other lymphoid populations, with malignant myeloid progenitors (Early GMP, Late GMP, Pro-Monocyte) dominating the cellular composition without exception across patients, while healthy controls showed clearly delineated T cell clusters, confirming that CD8⁺ T cells do not constitute a major cellular component of the myelomonocytic AML bone marrow microenvironment (Supplemental Fig. 2A).

To illustrate differences in T cell exhaustion and ICD-related molecular profiles across malignant and immune cell populations, we analyzed publicly available bone marrow scRNA-seq datasets from myelomonocytic (M4) and monocytic (M5) AML patients (including pediatric and adult cases) and healthy controls (Supplemental Fig. 2 and Supplemental Table 2). Both AML subtypes showed diverse malignant and immune cell populations. Single-cell RNA-seq (scRNA-seq) revealed reduced NK and T cell proportions in M4 and M5 AML samples compared to controls (Supplemental Fig. 2A). To characterize the functional state of the residual CD8⁺ T cells present in AML bone marrow, we assessed expression of markers spanning the four-stage T cell exhaustion hierarchy [28] across all seven AML patients and both healthy controls in the scPCA dataset (Supplemental Fig. 2B–G). Application of the Beltra framework to the T cell clusters revealed that AML bone marrow CD8⁺ T cells occupy a progenitor-to-intermediate exhaustion transition rather than a terminally exhausted state (Supplemental Fig. 2B). The progenitor marker *TCF7/TCF-1* was retained in approximately half of T cells across the AML cohort (mean pct.1 = 0.506 ± 0.201), comparable to healthy bone marrow (0.487), and was co-expressed with *SLAMF6/Ly108* (mean pct.1 = 0.144 ± 0.052). In parallel, effector and intermediate exhaustion markers were significantly upregulated: *KLRG1* (mean pct.1 = 0.312 ± 0.164), *PRF1* (0.407 ± 0.230), *GZMB* (0.301 ± 0.225), and the transcription factor *ZNF683/Hobit* (0.120 ± 0.098), which has recently been identified as a marker of intermediate-exhausted CD8⁺ effector/effector-memory T cells that predicts anti-PD-1 response in Richter syndrome bone marrow [29]. Critically, canonical terminal exhaustion markers were uniformly absent across all seven AML patients: *ENTPD1/CD39* (mean pct.1 = 0.009 ± 0.011), *TOX2* (0.015 ± 0.016), and *BTLA* (0.014 ± 0.020) (Supplemental Fig. 2B). This marker profile places the residual AML bone marrow CD8⁺ T cells at the Texprog2/Texint transition, the exhaustion subsets that are preferentially expanded by PD-1/PD-L1 blockade [28].

We analyzed the distribution of ICD/ER-stress mediators across monocyte blast clusters to identify potential danger signals in the microenvironment of M4 and M5 AML patients (Supplemental Fig. 2D–G). We focused on genes involved in ICD/ER stress signaling, such as *CALR, HMGB1, HSPA1A, HSPA1B, HSPA5, HSP90B1*, and *IL1B*, which were reliably detected in the scRNA-seq dataset. ICD gene expression varied among blast-like subpopulations; in some M4 and M5 cases, these genes were more highly expressed in non-monocytic blast populations, indicating widespread ICD signaling (e.g., M4_SCPCS000219, M4_SCPCS000217, M5a_SCPCS000241). In others, ICD expression was enriched in monocytic-like blast compartments (e.g., M5a_SCPCS000236, M5b_SCPCS000227).

These findings indicate that myelomonocytic M4/M5 AML features an immunosuppressive microenvironment with a progenitor-to-intermediate exhausted T cell compartment permissive to checkpoint blockade, characterizing a leukemia subtype resistant to immune therapies but potentially responsive to strategies that induce ICD-related danger signals. Accordingly, we investigated whether the ICD-inducing synthetic coumarin derivative OT-55 promotes DAMP release and cytotoxicity in murine AML.

### Coumarin derivative OT-55 induced cytotoxic activity via the release of DAMPs in murine AML

Our ICD-score analysis linked favorable outcomes with immune-activated transcriptional programs and a checkpoint-rich environment, notably *HAVCR2*/Tim-3, in ICD-high AML (Fig. 1). Consistent with prior AME profiling, scRNA-seq of M4/M5 AML confirmed T cell depletion and revealed a progenitor-to-intermediate exhaustion state permissive to checkpoint blockade (Supplemental Fig. 2). These results suggest the microenvironment needs robust ICD-associated danger signals for effective T cell priming. We therefore used a myelomonocytic AML model to test whether pharmacologic ER-stress induction can trigger functional ICD and circumvent immune restriction.

OT-55 was selected as the lead compound for this study on the basis of previously established evidence that coumarin derivatives display anti-leukemic activity, induce ER stress-coupled caspase-dependent apoptosis, promote DAMP release, including calreticulin surface exposure and HMGB1 secretion, and enhance macrophage phagocytosis of leukemia cells, properties consistent with ICD induction and with the capacity to potentiate BH3-mimetic-driven apoptosis into an immunostimulatory signal [10, 11].

**Fig. 2:**
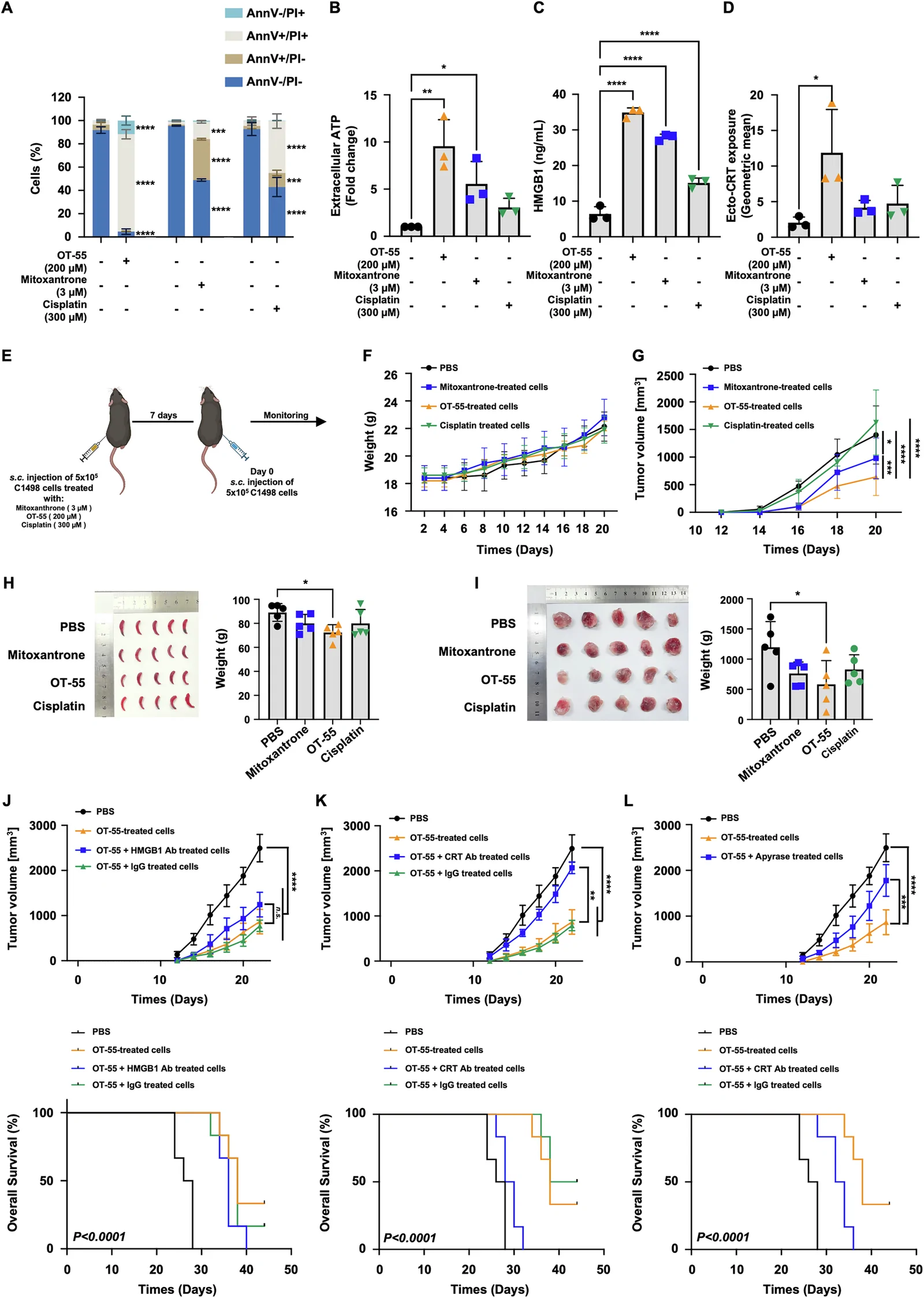
OT-55 triggered cell death via DAMP release in C1498 cells, and vaccination with OT-55-treated C1498 cells elicited an in vivo antitumor effect that depended on CRT or ATP. **A** The effect of OT-55 on the C1498 cell line. Flow cytometry analysis of apoptosis (early and late) and necrosis in C1498 cells after 24-h treatment with OT-55 (200 μM), mitoxantrone (3 μM), and cisplatin (300 μM). Under the same 24-h treatment conditions, mitoxantrone was used as a bona fide ICD inducer, and cisplatin was used as an ICD-negative control. **B** Levels of extracellular ATP and **C** HMGB1 release in the cell culture supernatants. **D** Evaluation of ectopic calreticulin exposure by flow cytometry. Quantification of ectopic calreticulin exposure after indicated concentrations of OT-55, mitoxantrone, and cisplatin for 24 h. **E** Experimental design: C57BL/6 mice were injected into the left flank with 5 × 10⁵ treated C1498 cells in vitro using OT-55 (200 μM), mitoxantrone (3 μM), and cisplatin (300 μM) for 24 h. One week later, they were re-challenged with 5 × 10⁵ C1498 cells in the right flank (*n* = 5 mice per group). Created with BioRender.com. **F** Body weight of mice in each treatment group was recorded every two days and shown as mean ± SD. **G** Tumor volumes were calculated using the formula L × W × W × 0.52 mm³ and shown as means ± SD. **H**, **I** When the tumor volume in the PBS group reached approximately 2500 mm³, the mice were sacrificed, and the spleen and tumor weights were recorded. **J**–**L** Mice were injected with OT-55 (200 μM)-treated C1498 cells in the presence or absence of blocking reagents against HMGB1, CRT, or ATP (*n* = 6 mice per group). Tumor volume (upper panel) and survival graph (lower panel). Data analysis was performed using one-way and two-way ANOVA followed by Tukey’s post-hoc test. ^*^P < 0.05, ^**^P < 0.01, ^***^P < 0.001, and ^****^P < 0.0001.

We next examined whether the coumarin derivative OT-55 induces canonical DAMPs in C1498 cells, a murine FAB-M4 model. C1498 cells treated with various OT-55 concentrations for 24, 48, and 72 h showed dose- and time-dependent reductions in proliferation and viability (Supplemental Fig. 3A, B). Annexin-V/PI staining confirmed a 27.0-fold increase in apoptotic cells at 24 h (Fig. 2A). Mitoxantrone served as a positive control, while cisplatin was a non-ICD inducer.

Since cytotoxicity alone does not confirm immunogenicity, and AML apoptosis can be poorly inflammatory, we tested whether OT-55-induced cell death is associated with hallmark ICD danger signals. We measured ICD-related DAMP release, including extracellular ATP, HMGB1, and calreticulin exposure. After 24 h, OT-55 increased extracellular ATP secretion by 9.6-fold in C1498 cells compared to control, surpassing mitoxantrone (5.6-fold) and cisplatin (3.0-fold) (Fig. 2B). OT-55 at 200 μM also significantly increased nuclear HMGB1 release by 5.4-fold, nearly matching mitoxantrone’s 4.4-fold increase, and was 2.2 times higher than cisplatin (Fig. 2C). Additionally, OT-55 elevated CRT exposure at 200 μM by 5.8-fold (Fig. 2D and Supplemental Fig. 3C). Based on these results, we conducted in vivo functional ICD validation via protective vaccination, assessing whether OT-55-treated cells stimulate antitumor immunity and which DAMPs are essential.

### Prophylactic vaccination with OT-55-treated cells enhanced antitumor immunity that is mediated by CRT- or ATP-dependent mechanisms

DAMPs emission by dying cells is key for recruiting immune effectors. Vaccination in immunocompetent mice is the standard test for cell death immunogenicity. C57BL/6 mice were vaccinated with dying cancer cells treated with 200 µM OT-55, 3 µM mitoxantrone, or 300 µM cisplatin for 24 h, then injected into the left flank. Controls received PBS. One week later, mice were rechallenged with live C1498 cells in the right flank; tumor growth was monitored until tumors exceeded 2000 mm³ in controls (Fig. 2E). Body weight changes were minimal and nonsignificant (Fig. 2F). OT-55-treated cells significantly reduced tumor weight compared to PBS (−54.3%), mitoxantrone (−34.5%), or cisplatin (−60.6%) (Fig. 2G and Supplemental Fig. 3D). The OT-55 group also had lower spleen (−18.7%, −9.4%, −9.5%) and tumor (−51.3%, −23.4%, -29.7%) weights than other groups (Fig. 2H, I). These findings support the immunogenicity of the coumarin derivative OT-55.

To link vaccine protection to observed in vitro DAMP signals, we neutralized specific DAMP pathways during vaccination using HMGB1 and CRT blockade or ATP degradation with apyrase. OT-55-treated C1498 cells were incubated with anti-HMGB1, anti-CRT antibodies, or apyrase. Mice vaccinated with these treated cells showed no significant decrease in efficacy in the HMGB1-depleted group (Fig. 2J). However, CRT- (−58%) and ATP- (−51.2%) depleted groups exhibited marked reductions in efficacy, indicating CRT- and ATP-signaling are crucial for tumor control (Fig. 2K, L). These groups also had shorter survival (32 and 37 days) than the over 48-day survival in the vaccinated group. Overall, vaccination with OT-55-treated C1498 cells enhances long-term antitumor effects via CRT- and ATP-dependent DAMP signaling. As the immunostimulatory activity of HMGB1 is highly sensitive to the oxidative microenvironment in which it is released, we assessed whether OT-55 induces an oxidative environment. Our results show that OT-55 (200 µM) induced a time-dependent and progressive increase in intracellular ROS, rising significantly from 1 h and peaking at 24 h, as measured by DCFH-DA geometric mean fluorescence intensity (Supplemental Fig. 3E). This oxidative response was partially suppressed by the antioxidant N-acetylcysteine (NAC, 10 mM), confirming its specificity.

Analysis of AML patients shows that higher CRT expression correlates with long-term survival (Supplemental Fig. 3F). Our in vivo studies support this, indicating CRT is key to vaccine-induced immunity and survival outcomes.

Persistent tumors in some mice suggest priming alone may be insufficient due to inhibitory circuits. ICD-high AML cases showed increased *HAVCR2* (Tim-3) and variable *PDCD1* (PD-1) expression (Fig. 1E). scRNA-seq indicated a checkpoint-expressing progenitor-to-intermediate exhausted T cell state in myelomonocytic AML (Supplemental Fig. 2). Next, we tested whether PD-1 and Tim-3 blockade could enhance OT-55 vaccination.

### Prophylactic vaccination with OT-55-treated cells enhanced antitumor immunity and was potentiated by immune checkpoint inhibitors in a syngeneic mouse model

**Fig. 3:**
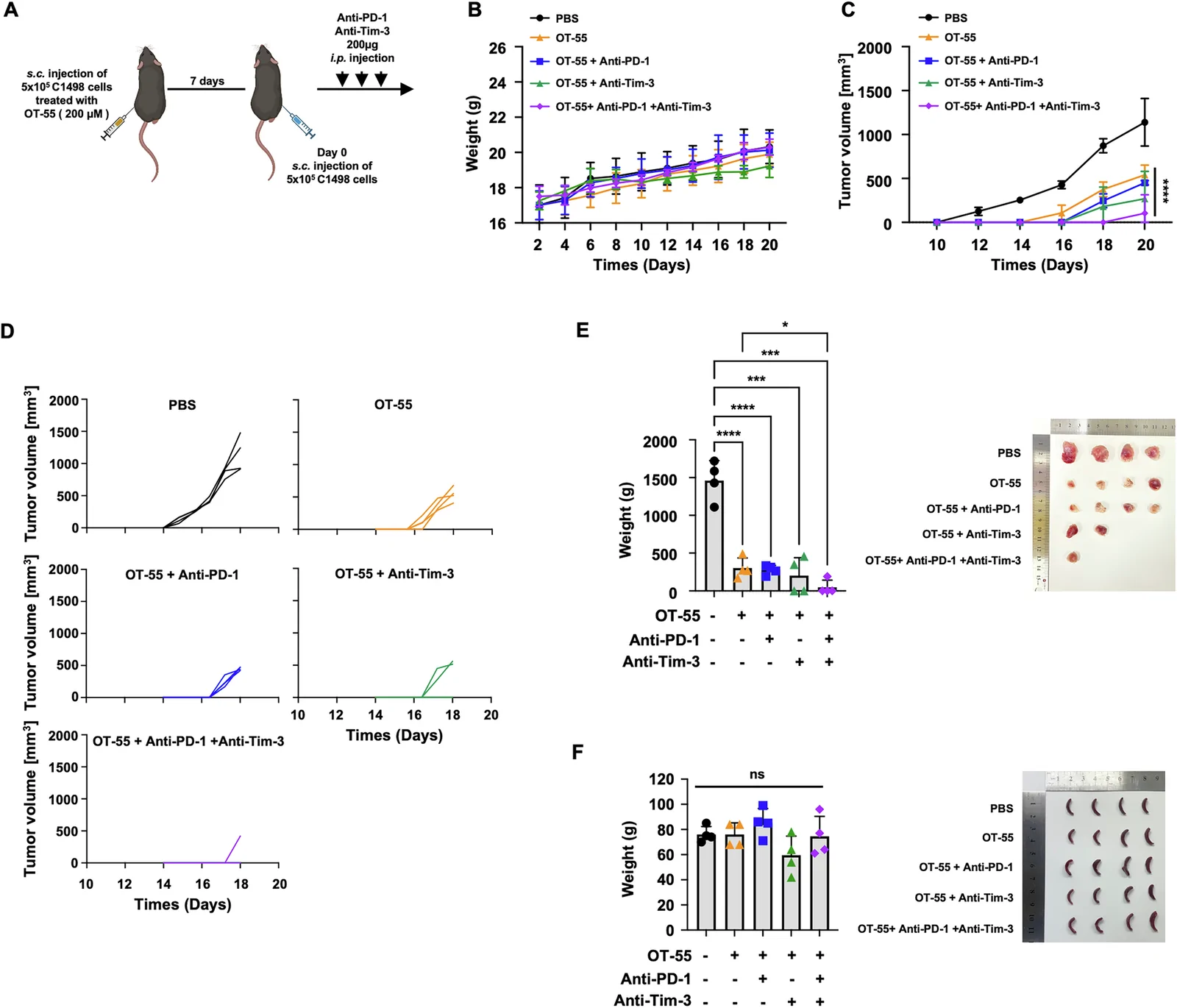
Vaccination with OT-55-treated C1498 cells synergizes with ICIs. **A** Experimental design: C57BL/6 mice were injected into the left flank with 5 × 10⁵ treated C1498 cells in vitro with OT-55 (200 μM) for 24 h, and one week later, re-challenged with 5 × 10⁵ C1498 cells in the right flank (*n* = 4 mice per group). After re-challenge, ICIs were administered by *i.p*. injection of 200 µg of anti-PD-1 or anti-Tim-3, alone or in combination, every 3 days for 3 doses. Created with BioRender.com. **B**, **C** Body weight and tumor volume were measured every two days and represented as means ± SD. **D** Individual tumor growth curves of mice in each treatment group. Tumor volumes were measured using the formula L × W × W × 0.52 mm³. **E**, **F** When the tumor volume in the PBS group reached approximately 2500 mm³, the mice were sacrificed, and the tumor and spleen weights were measured. All data analysis was done using one-way ANOVA and two-way ANOVA; Tukey’s post-hoc test. ^**^P < 0.01, and ^***^P < 0.001.

While OT-55 vaccination limited tumor growth, high *HAVCR2* (Tim-3) and *PDCD1* (PD-1) expression in ICD-high AML (Fig. 1E) suggested ongoing immune suppression (Fig. 2J–L). To test whether blocking PD-1 and Tim-3 improves vaccine efficacy, we combined OT-55 with anti-PD-1 and anti-Tim-3 antibodies, injected intraperitoneally on days 2, 5, and 8 after re-challenging with C1498 cells. Tumor progression and body weight were monitored every two days (Fig. 3A). Body weight remained stable across groups (Fig. 3B). OT-55-treated cells reduced tumor size significantly compared to non-vaccinated controls. Adding anti-PD-1 decreased tumor size by 60%, anti-Tim-3 by 53% (Fig. 3C, D), and combined ICIs reduced it by 63.2% (Fig. 3C), with tumor rejection in 3 of 4 mice. Tumor weight correlated with tumor volume, while spleen weight was unaffected. The combination therapy enhanced antitumor effects, leading to reduced tumor burden, with no significant change in spleen weight (Fig. 3E, F). Of note, anti-PD-1 and anti-Tim-3 administered as monotherapies in the absence of prior vaccination had no significant effect on primary or distant (abscopal) tumor growth in the bilateral tumor model (Fig. 7B), confirming that the tumor control observed upon combination with OT-55 vaccination is dependent on prior immunogenic priming rather than on checkpoint blockade alone.

### Synergistic and immunogenic effects of OT-55 in combination with BH3 mimetic A-1331852

Having established that OT-55 induces functional ICD in vivo and that checkpoint blockade enhances vaccine-mediated tumor rejection, we explored whether OT-55 could serve as an immunogenic adjuvant with BH3 mimetics in AML therapy, which typically triggers strong apoptosis but limited immunogenicity. We used the Bcl-xL inhibitor A-1331852, given the high basal Bcl-xL expression in C1498 cells (Supplemental Fig. 4A), to test this combination.

**Fig. 4:**
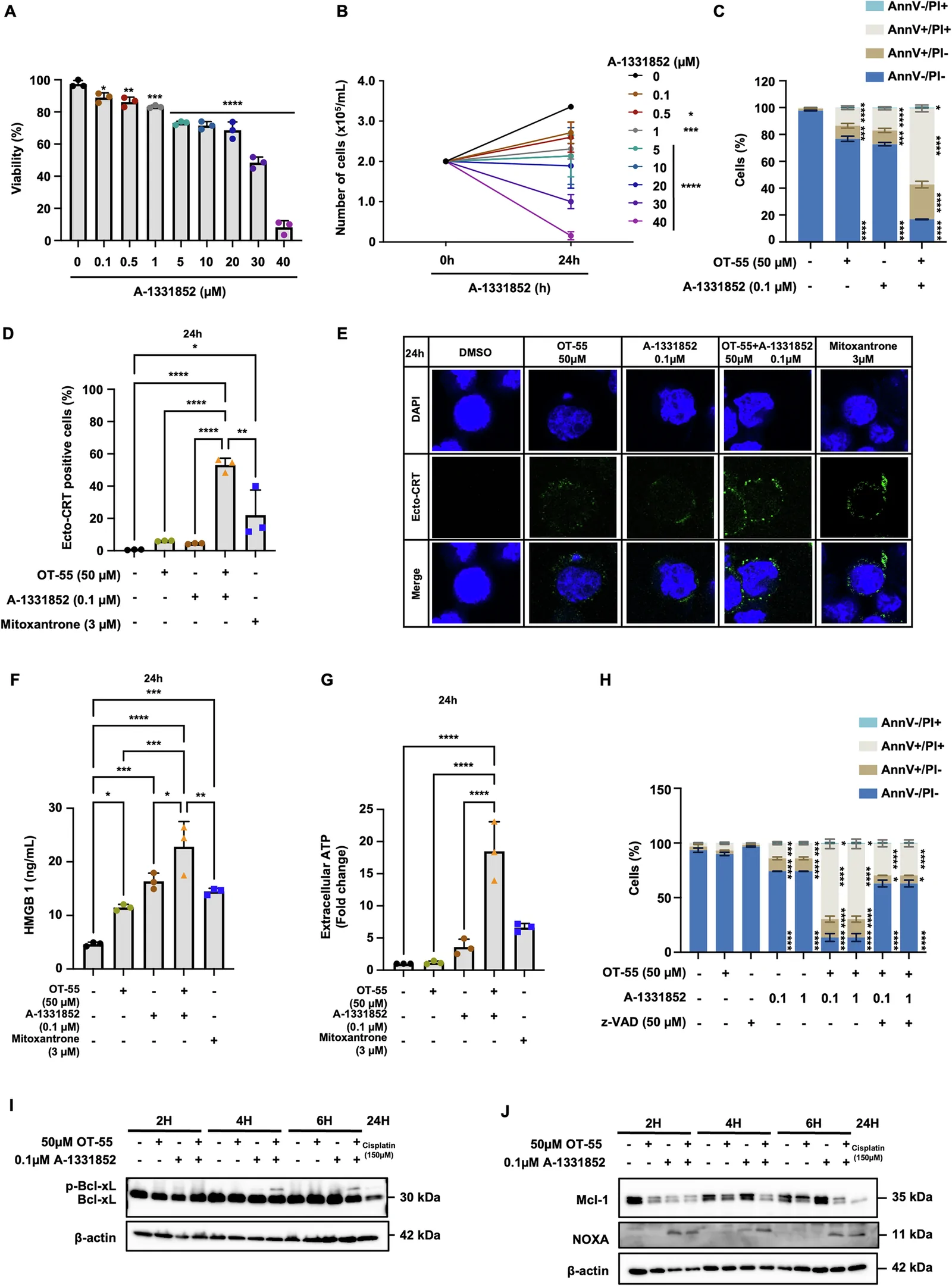
OT-55 enhances sensitivity to selective BH3 protein inhibitors, promoting induction of apoptotic ICD. **A**, **B** C1498 cells were treated with various doses of A-1331852 (0, 0.1, 0.5, 1, 5, 10, 20, 30, 40 µM) for 24 h. Cell viability and proliferation were assessed by the trypan blue staining. **C** Flow cytometry analysis of apoptosis and necrosis in C1498 cells after 24 h of treatment with 50 µM of OT-55 and 0.1 µM of A-1331852 in single and combination treatments. **D**, **E** C1498 cells were treated with 50 µM of OT-55 and 0.1 µM of A-1331852 for 24 h, alone or in combination, and mitoxantrone was used as a bona fide ICD inducer. Ectopic calreticulin exposure was quantified by flow cytometry and confirmed by confocal microscopy. **F** Release of HMGB1 after 24 h of treatment with 50 µM of OT-55 and 0.1 µM of A-1331852 in single and combination treatments, assessed by ELISA in C1498 cells supernatants. Mitoxantrone treatment at 3 µM for 24 h served as the control. **G** Extracellular release of ATP into the supernatant induced by 50 µM of OT-55 and 0.1 µM of A-1331852 in single and combination treatments after 24 h in C1498 cells was measured in a luciferase-based assay. **H** Cells were pretreated with 50 μM z-VAD for 1 h before OT-55 and/or A-1331852 treatment. After treatments, apoptotic and necrotic cells with caspase-dependent cell death were assessed by Annexin V/PI staining using FACS analysis. **I**, **J** Total proteins were isolated from OT-55- and A-1331852-treated C1498 cells. Western Blots for apoptosis-related protein expression. Cisplatin (150 µM) was used as a positive control of apoptosis induction. All data represent the mean ± SD of at least three independent experiments. Data analysis was done using one-way ANOVA and Tukey’s post-hoc test. ^*^P < 0.05, ^**^P < 0.01, ^***^P < 0.001, and ^****^P < 0.0001.

Western blot confirmed high Bcl-xL expression in C1498 cells relative to other anti-apoptotic proteins and cancer types (Supplemental Fig. 4A). C1498 cells treated with various concentrations of A-1331852 for 24 h showed sensitivity (Fig. 4A, B and Supplemental Fig. 4B), with an IC50 of 24.78 ± 0.85 µM. Since 50 µM OT-55 was cytostatic (Supplemental Fig. 3A), we combined it with 0.1 µM A-1331852, a subtoxic dose, which increased cell death 3.5-fold over OT-55 alone (Fig. 4C).

We assessed ICD-related DAMPs: CRT exposure, HMGB1 release, and extracellular ATP, under the same conditions. CRT surface exposure increased with the combination at 6, 12, and 24 h (Fig. 4D and Supplemental Fig. 4C, D). Confocal microscopy confirmed time-dependent CRT surface re-localization (Fig. 4E and Supplemental Fig. 4E, F). HMGB1 release was significantly higher with the combination at 24 h (Fig. 4F), with a time-dependent increase (Supplemental Fig. 4G, H). The combination also caused an 18.5-fold increase in ATP secretion at 24 h (Fig. 4G). These results suggest enhanced ICD with the combination, prompting further investigation into the underlying cell death mechanisms.

Pre-treatment with 50 µM z-VAD-FMK for 1 h reduced cell death induced by 50 µM OT-55 and 0.1 µM A-1331852 by 30%, indicating caspase-dependent cell death (Fig. 4H). We examined modulation of the BCL-2 family proteins and caspase processing by measuring levels of pro- and anti-apoptotic proteins Bcl-xL, Mcl-1, and NOXA, and caspases. The combination treatment caused Bcl-xL phosphorylation, reduced Mcl-1 (0.7-fold at 4 h, 0.3-fold at 6 h), and increased NOXA (5.9-fold at 4 h, 5.4-fold at 6 h) at 4 and 6 h, respectively. These changes decreased anti-apoptotic capacity and increased pro-apoptotic signals, suggesting inactivation of BCL-2 family components (Fig. 4I, J and Supplemental Fig. 4I, J).

To clarify the mechanistic basis for synergistic apoptosis induction, we examined Bim protein expression and caspase-3 processing under combination treatment conditions. Bcl-xL phosphorylation induced by OT-55 and A-1331852 (Fig. 4I) reduces Bcl-xL binding affinity for pro-apoptotic BH3-only proteins, most critically Bim. Consistent with this, the combination induced a time-dependent increase in Bim protein levels, reaching statistical significance at 6 h (Supplemental Fig. 4K, L), consistent with reduced Bim sequestration by phospho-Bcl-xL. Concurrently, OT-55-mediated ER stress reduced Mcl-1 (Fig. 4J), a second anti-apoptotic barrier capable of sequestering Bim, through a mechanism likely involving integrated stress response (ISR)-mediated translational suppression, though direct measurement of ISR activation was not performed in this context, and induced NOXA, which competitively displaces Bim from residual Mcl-1. The convergence of these two mechanisms, Bim liberation from Bcl-xL and from Mcl-1, drove robust caspase-3 cleavage at 6 h with the combination treatment (Supplemental Fig. 4M, N), supporting a mechanistic model in which Bcl-xL phosphorylation and Mcl-1 downregulation converge to drive caspase-dependent ICD.

Because enhanced DAMP emission and apoptotic engagement are necessary but insufficient for immunogenicity, we tested whether vaccination with dual-treated cells elicits protective antitumor immunity in vivo.

### Prophylactic vaccination with OT-55-treated cells demonstrates functional ICD in vivo and requires antigen-specific CD8^+^ T cell responses

To assess the role of immune cells in vaccine-induced anticancer responses, mice vaccinated with OT-55 and A-1331852-treated C1498 cells underwent antibody-mediated depletion of CD4⁺ or CD8⁺ T cells.

**Fig. 5:**
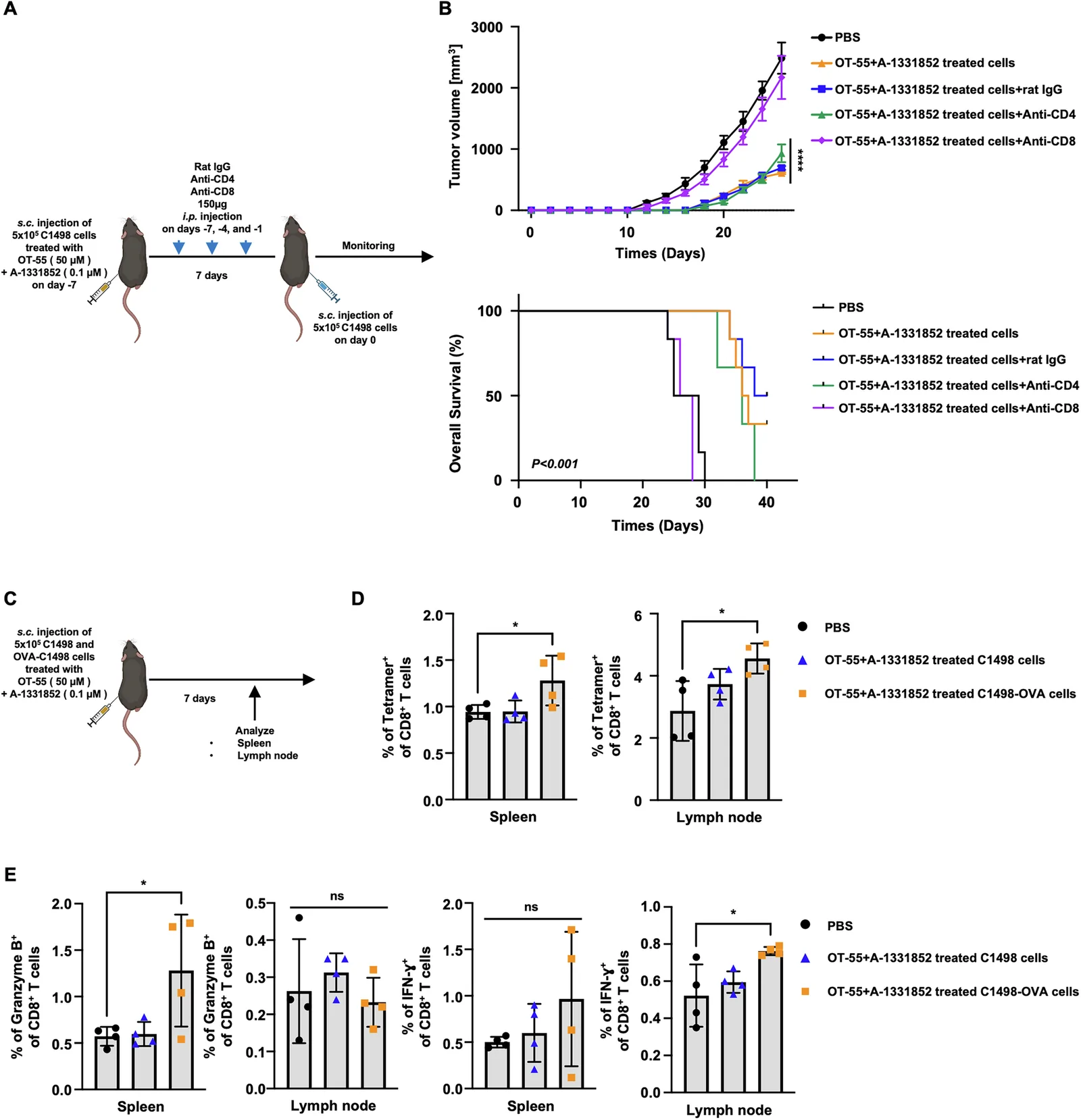
Prophylactic vaccination with OT-55 and A-1331852-treated C1498 cells induced C1498 antigen-specific CD8^+^ T cells in vivo. **A** Experimental design of the vaccination assay with depletion of T cell subsets. C57BL/6 mice were injected into the left flank with 5 × 10⁵ treated C1498 cells in vitro with OT-55 (50 μM) and A-1331852 (0.1 μM) in combination for 24 h. After vaccination, 150 μg of rat IgG-, anti-CD4-, and anti-CD8-specific antibodies were administered via intraperitoneal (*i.p*.) injection three times. One week later, the mice were re-challenged with 5 × 10⁵ C1498 cells in the right flank (*n* = 6 mice per group). Created with BioRender.com. **B** (Upper) Tumor volume kinetics in mice vaccinated with OT-55 (50 μM) and A-1331852 (0.1 μM)-treated C1498 cells, with or without antibody-mediated depletion of CD4⁺ or CD8⁺ T cells, and in PBS controls. Tumor volumes are shown as mean ± SD. (Lower) Overall survival in C57BL/6 mice vaccinated with OT-55- and A-1331852-treated C1498 cells and subsequently treated with 150 µg of rat IgG, anti-CD4, and anti-CD8. **C** Experimental design for the study in (**D**, **E**). OT-55 and A-1331852-treated C1498 and C1498-OVA cells. After treatment, the cells were injected into C57BL/6 mice, and their organs were analyzed. Created with BioRender.com. **D** Frequencies of OVA-Tet⁺CD8⁺ T cells in the spleen and lymph nodes. **E** Frequencies and proportions of effector CD8⁺ T cells in the spleen and lymph nodes (*n* = 4 mice per group). All data analysis was performed using one-way ANOVA followed by Tukey’s post-hoc test. ^*^P < 0.05, and ^****^P < 0.0001.

To generate T cell subset-depleted mice, we injected anti-CD4 and anti-CD8 antibodies intraperitoneally on days −7, −4, and −1, starting on the day of vaccination (−7 days). Flow cytometry on day 0 confirmed efficient CD4⁺ and CD8⁺ T cell depletion (Supplemental Fig. 5A). One week post-vaccination, mice were rechallenged with C1498 cells (Fig. 5A). Vaccination with treated C1498 cells delayed tumor growth from day 12 and reduced tumor formation by 72.4% at day 26 versus PBS (Fig. 5B). Both vaccination alone and with rat IgG, a negative control, showed similar delays (70.7% reduction). Vaccinated groups had extended survival over 40 days. CD4⁺ T cell depletion reduced tumor growth by 73.5% at day 26, suggesting CD4⁺ T cells are not essential for vaccine efficacy. CD8⁺ T cell depletion decreased tumor reduction to 15.4%, and overall vaccine efficacy was reduced by 67.4%. CD8⁺ T cell depletion shortened survival to levels similar to those with PBS, indicating that CD8⁺ T cells are critical for immunity and survival.

Given the key role of CD8⁺ T cells in vaccine efficacy, we examined antigen-specific responses induced by vaccination. Mice were vaccinated with 50 µM OT-55 and 0.1 µM A-1331852-treated C1498 or OVA-C1498 cells, which are C1498 leukemia cells engineered to express ovalbumin. No significant differences in viability or proliferation were found between C1498 and OVA-C1498 cells after treatment (Supplemental Fig. 5B). OVA-specific CD8⁺ T cells in the spleen and lymph nodes were quantified using H-2Kb OVA257-264 tetramers (Fig. 5C). Vaccination with OT-55 and A-1331852-treated OVA-C1498 cells significantly increased OVA-specific tetramer⁺CD8⁺ T cells in the spleen (1.36-fold) and lymph nodes (1.59-fold) compared to PBS (Fig. 5D).

To further examine the functional profile of vaccine-induced CD8⁺ T cells, spleen and lymph node cells were analyzed for granzyme B and IFN-γ expression (Fig. 5E). In vaccinated mice treated with OT-55 and A-1331852 against OVA-C1498 cells, the proportion of granzyme B⁺CD8⁺ cells was significantly higher in the spleen (2.21-fold versus PBS), but slightly lower in lymph nodes. Conversely, IFN-γ⁺CD8⁺ T cells were more abundant in lymph nodes, with a 1.46-fold increase compared to PBS. These findings suggest tissue-specific differentiation of vaccine-induced CD8⁺ T cells, with spleen cells exhibiting a cytolytic phenotype and lymph node cells displaying a primed or early effector phenotype characterized by cytokine production, especially IFN-γ [30, 31]. Vaccination with OT-55 and A-1331852-treated C1498 cells effectively triggered CD8⁺ T cell-dependent antitumor responses, demonstrating the vaccine’s capacity to induce antigen-specific and effector CD8⁺ T cell proliferation and infiltration in multiple organs, including spleen and lymph nodes.

### Enhanced immunogenicity and vaccination efficacy are induced by the combination of OT-55, A-1331852, and ICIs in vivo

Since vaccine efficacy primarily relies on CD8⁺ T cells, and intermediate-exhausted CD8⁺ cells in myelomonocytic AML exhibit inhibitory receptor programs, we tested whether blocking PD-1 and/or Tim-3 could boost vaccine-induced CD8⁺ responses. Considering the in vitro immunogenic and apoptotic effects of OT-55 and A-1331852, we examined whether their combination with ICIs could improve antitumor immunity in a prophylactic mouse vaccination model.

**Fig. 6:**
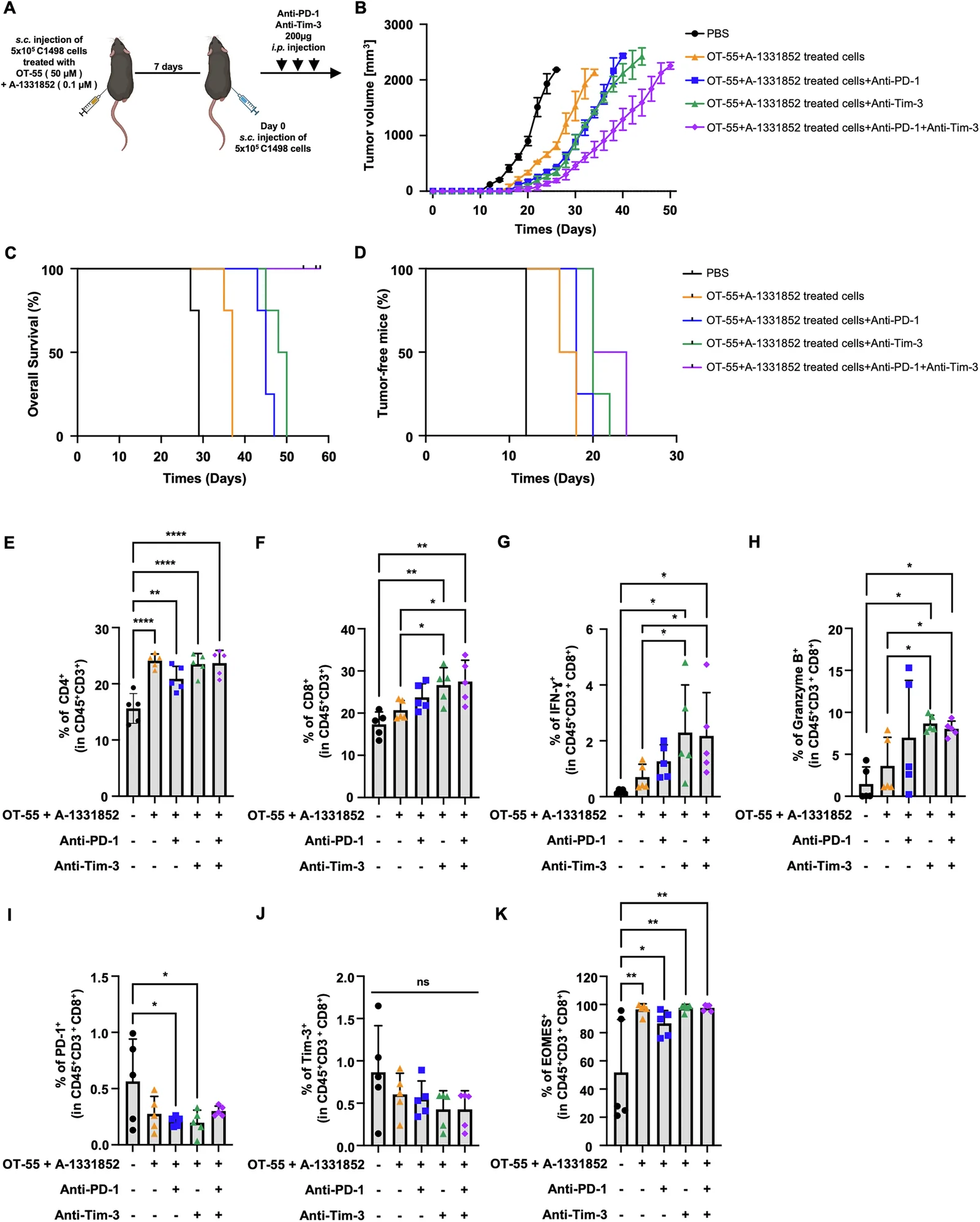
Vaccination with OT-55 and A-1331852-treated C1498 synergizes with ICIs and enhances immunogenicity. **A** Experimental design of vaccination assay with ICIs injection. C57BL/6 mice were injected into the left flank with 5 × 10⁵ treated C1498 cells in vitro with OT-55 (50 μM) and A-1331852 (0.1 μM) in combination for 24 h, and one week later, re-challenged with 5 × 10⁵ C1498 cells in the right flank (*n* = 5 mice in each group). After re-challenge, ICIs were administered by intraperitoneal injection of 200 µg every 3 days for 3 doses, using anti-PD-1 and anti-Tim-3, either alone or in combination. Created with BioRender.com. **B** Kinetic analysis of tumor volume in syngeneic immunocompetent C57BL/6 mice. **C**, **D** Overall survival and tumor-free survival in C57BL/6 mice vaccinated with OT-55- and A-1331852-treated C1498 cells, allocated to receive 200 µg of anti-PD-1 and anti-Tim-3, both alone and in combination. **E**–**K** Immune infiltration after the vaccination assay with ICIs. Percentage of different immune cell subsets in live CD45⁺CD3⁺ cells. Data analysis was done using one-way ANOVA followed by Tukey’s post-hoc test. ^*^P < 0.05, ^**^P < 0.01, ^***^P < 0.001, and ^****^P < 0.0001.

Before testing the prophylactic mouse model, we measured HMGB1 release into the supernatant prior to injection of treated cells to monitor ICD induction (Supplemental Fig. 6A). Syngeneic C57BL/6 mice were vaccinated with dying cancer cells treated with 50 μM OT-55 and 0.1 μM A-1331852 for 24 h, then subcutaneously injected into the left flank. Controls received PBS only. After a week, mice were re-challenged with live C1498 cells in the right flank. Anti-PD-1 and anti-Tim-3 antibodies were administered on days 2, 5, and 8 post-rechallenge via *i.p*. injection (Fig. 6A). The combination of OT-55 and A-1331852-treated cells with anti-PD-1 and anti-Tim-3 delayed tumor formation more than controls (92.9% reduction at day 24) or treated cells alone (79.1%). This combination significantly improved overall and tumor-free survival (Fig. 6B–D).

To evaluate the impact of combination therapy on antitumor immune responses, flow cytometry was used to analyze immune infiltration in the tumor-draining lymph node (Fig. 6E–K). Vaccination with OT-55 and A-1331852-treated C1498 cells increased the proportions of CD4⁺ (1.55-fold) (Fig. 6E) and CD8⁺ (1.24-fold) (Fig. 6F) T cells among CD45⁺CD3⁺ cells. Anti-Tim-3 injections enhanced CD8⁺ T cell responses, with a 1.55-fold increase compared with the non-vaccinated group and a 1.25-fold increase compared with the vaccination-alone group. Combining anti-PD-1 and anti-Tim-3 with treated cells further increased CD8⁺ cytotoxic T lymphocytes (1.62-fold vs non-vaccinated; 1.31-fold vs vaccination alone). Vaccination combined with anti-Tim-3 also boosted CD8⁺ T cell function, elevating IFN-γ⁺ cells (12.40-fold vs non-vaccinated, 5.31-fold vs vaccination) and granzyme B⁺ cells (4.86-fold vs non-vaccinated, 2.07-fold vs vaccination) (Fig. 6G, H). Frequencies of IFN-γ⁺ and granzyme B⁺ CD8⁺ T cells were significantly higher in the combination group (10.81- and 4.38-fold vs non-vaccinated; 4.63- and 1.87-fold vs vaccination). A moderate reduction in exhaustion markers PD-1 and Tim-3 was observed, particularly in the OT-55/A-1331852 + anti-PD-1/anti-Tim-3 group (Fig. 6I, J). The proportion of EOMES⁺CD8⁺ T cells was increased in vaccinated mice and remained high with combination therapy (Fig. 6K). Overall, these findings indicate that combining vaccination with immune checkpoint inhibitors enhances the differentiation, functionality, and persistence of CD8⁺ T cells, amplifying antitumor immunity.

To address whether the combination regimen exerts a sustained effect on tumor growth kinetics beyond delaying tumor onset, we calculated tumor growth slopes from log-transformed tumor volumes after tumor establishment. The full combination of OT-55, A-1331852, anti-PD-1, and anti-Tim-3 significantly reduced the post-establishment growth slope by approximately 48% compared to PBS controls (0.0963 vs 0.0504), with a stepwise reduction in growth rate observed upon sequential addition of each immune component (Supplemental Fig. 6B). These data demonstrate that the combination achieves sustained suppression of tumor expansion after establishment, rather than a transient delay in onset.

### Distant tumor (abscopal) antitumor efficacy of OT-55 and A-1331852 combined with immune checkpoint inhibitors

To assess if the improved local tumor control from OT-55/A-1331852 vaccination plus checkpoint blockade induces systemic immunity capable of restraining an untreated distant lesion and to evaluate tolerability in immunocompetent hosts, we tested the regimen in a bilateral (abscopal) C1498 model with blood counts and serum chemistry profiling.

**Fig. 7:**
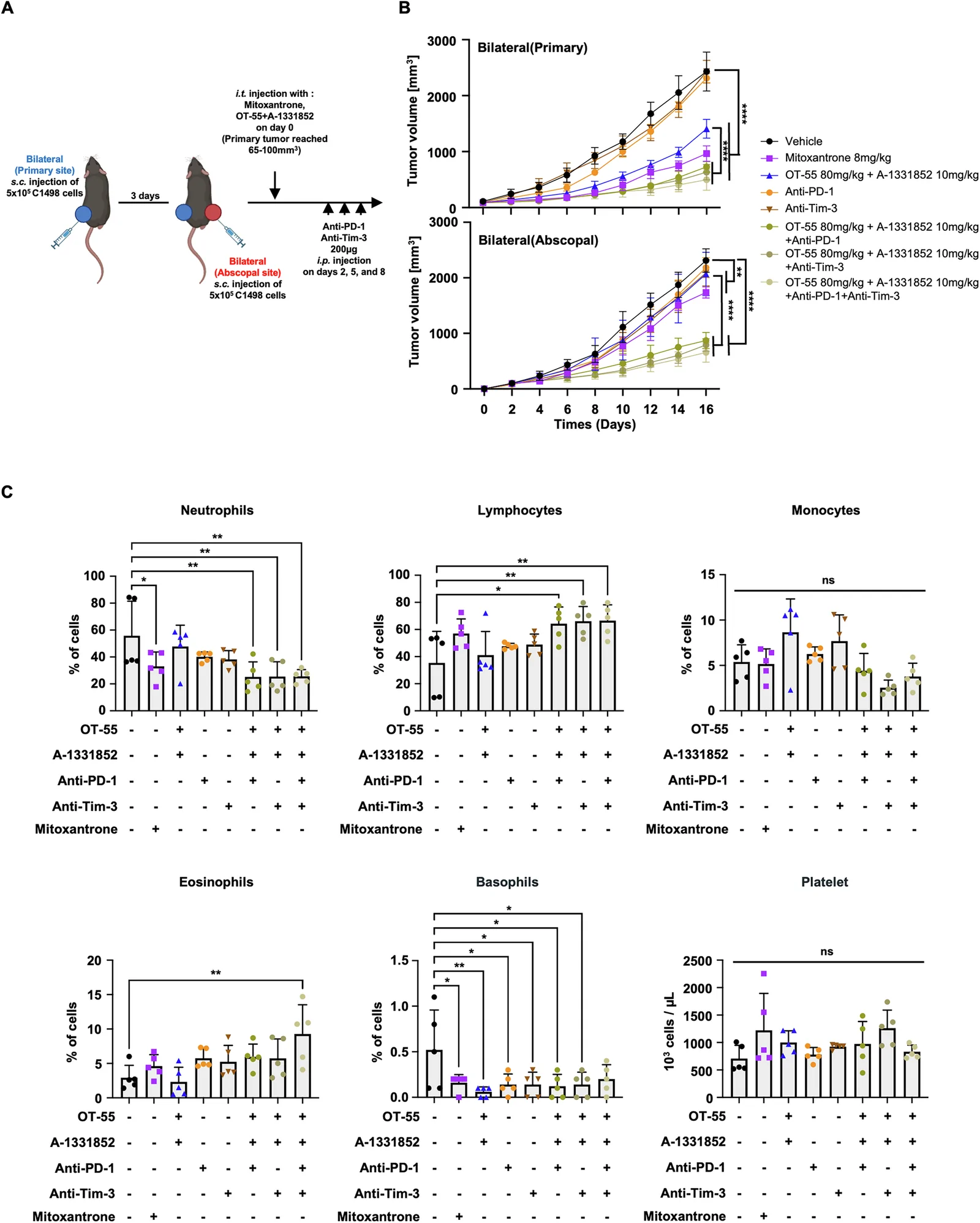
Combination treatment with OT-55 and A-1331852 induced systemic antitumor effects and modulated peripheral immune cell profiles without hematologic toxicity. **A** Experimental design of the bilateral tumor (abscopal) assay with ICIs injection. C57BL/6 mice were injected into the left flank with 5 × 10⁵ C1498 cells on day 0, followed 3 days later by injection of 5 × 10⁵ C1498 cells into the right flank (*n* = 5 mice per group). When the primary tumors reached 65–100 mm³ (day 0 of treatment), the drugs were administered via *i.t*. injection, and on days 2, 5, and 8, ICIs were administered via *i.p*. injection, as indicated for the treatment groups. Tumor incidence and growth were monitored on a routine basis. Created with BioRender.com. **B** Kinetic analysis of tumor volume in syngeneic immunocompetent C57BL/6 mice. Primary site tumor volume (upper panel) and distant (abscopal) site tumor volume (lower panel). **C** Complete blood count (CBC) analysis was performed on EDTA-treated whole blood obtained from each mouse in the following eight groups. Data analysis was done using one-way ANOVA and two-way ANOVA, and Tukey’s post-hoc test. ^*^P < 0.05, ^**^P < 0.01, ^***^P < 0.001, and ^****^P < 0.0001.

To evaluate the systemic antitumor efficacy of the vaccination assay, a bilateral tumor model was established by subcutaneous injection of C1498 cells into the left flank on day 0, followed by injection of C1498 cells into the right flank on day 3 (Fig. 7A). We determined the optimal doses for intratumoral injection of A-1331852 (10 and 20 mg/kg) (Supplemental Fig. 7A), which are below the systemic doses (25 mg/kg daily for 10 days [32] or 14 days [33]). For mitoxantrone, doses of 4 and 8 mg/kg were tested, both below systemic levels (6 mg/kg biweekly for three weeks [34], and 20 or 40 mg/kg twice daily [35]). OT-55 was tested at 40 and 80 mg/kg. Blood cytotoxicity was assessed to ensure systemic safety (Supplemental Fig. 7B). Based on these findings, doses that did not cause cytotoxicity were selected. Mitoxantrone served as a positive control.

We monitored primary and distant (abscopal) tumor growth after intratumoral injections at the primary site (Fig. 7B). Tumor volumes at the endpoint are in Supplemental Tables 3–6. The combination of OT-55 and A-1331852, when injected into the primary tumor, significantly reduced distant (abscopal) tumor size compared with vehicle, similar to mitoxantrone (Supplemental Table 3). Systemic administration of anti-PD-1 and/or anti-Tim-3 enhanced the effect of OT-55/A-1331852 at the primary site, with a 74% reduction in distant (abscopal) tumor size in the OT-55/A-1331852/anti-PD-1/anti-Tim-3 group versus vehicle (Supplemental Table 3).

Intratumoral injection of OT-55/A-1331852 at the primary site, with or without anti-PD-1 and/or anti-Tim-3, significantly reduced tumor size compared to the vehicle (Supplemental Tables 3 and 4). Anti-Tim-3 or anti-PD-1 alone had no effect on AML growth, but combined with OT-55/A-1331852 enhanced efficacy, reducing tumor volumes at both distant (abscopal) and primary sites.

To verify systemic immunomodulation, we performed complete blood count (CBC) tests (Fig. 7C). The combination of OT-55, A-1331852, and ICIs (anti-PD-1 and/or anti-Tim-3) significantly reduced neutrophils by 42.2–44.5% and increased lymphocytes by approximately 1.49–1.55-fold compared to non-vaccinated controls, indicating enhanced adaptive immunity. Eosinophils increased 1.83–3.20-fold with combination therapy. Platelet counts remained stable. Liver and kidney functions, assessed via biochemical analyses, showed normal serum levels of AST, ALT, BUN, and creatinine (Supplemental Fig. 7C).

Collectively, these findings suggest a stepwise strategy of ICD induction followed by CD8⁺ T cell reinforcement with checkpoint inhibitors, converting tolerogenic cell death into protective antitumor immunity in AML, with evidence of systemic effects at local and distant sites, supporting translational development.

## Discussion

AML is accompanied by profound immune dysregulation within the bone marrow niche, with impaired antigen presentation, expansion of immunosuppressive myeloid populations and regulatory T cells, and progressive T cell dysfunction/exhaustion, which collectively blunt endogenous anti-leukemic immunity and likely contribute to the limited efficacy of current immunotherapies in AML [36–38]. In this context, vaccination is attractive because it can deliver leukemia antigens along with adjuvant stimuli that promote dendritic cell activation and CD8⁺ T cell priming. AML vaccination has been clinically feasible and can boost CD8⁺ T cells targeting leukemia-associated antigens [39, 40]. Combination strategies that pair vaccination with immune modulation can further enhance vaccine-induced responses [41].

An apparent paradox emerges from our single-cell analysis: the AML ICD score identifies a tumor-intrinsic immunogenic transcriptional program, yet M4/M5 AML bone marrow shows markedly reduced T cell infiltration compared to healthy controls. However, two features of the residual T cell compartment resolve this paradox and provide the biological foundation for our combination strategy. First, the CD8⁺ T cells that do infiltrate M4/M5 AML bone marrow occupy a TCF7⁺ progenitor-to-intermediate exhaustion transition (Texprog2/Texint), a state that retains both self-renewal capacity and cytotoxic effector potential (KLRG1⁺, PRF1⁺, GZMB⁺, ZNF683⁺) while lacking terminal exhaustion commitment (CD39^−^, TOX2^−^, BTLA^−^). This phenotype corresponds to the exhaustion subsets that are preferentially expanded by PD-1/PD-L1 blockade [28], and that predict an anti-PD-1 response in hematologic malignancies [29]. Second, M4/M5 AML blasts derive from the monocytic lineage and constitutively express MHC class I and II molecules, preserving the antigen-presentation machinery required for CD8⁺ T cell recognition, a critical distinction from solid tumors, where MHC-I downregulation frequently limits immunotherapy efficacy. Together, these observations suggest that the determinant of checkpoint blockade responsiveness in this context is not the frequency of infiltrating T cells but rather their functional state: a small pool of progenitor-exhausted, checkpoint-responsive T cells confronting MHC-competent tumor cells may represent a more favorable therapeutic substrate than abundant but terminally exhausted infiltrates. OT-55 + A-1331852-induced ICD is proposed as our approach to exploit this configuration by providing the DAMP and antigen context through calreticulin surface exposure and HMGB1 release, which converts the latent immunogenic potential captured by the ICD score into active immune engagement, while concurrent PD-1/Tim-3 blockade releases the checkpoint restraint on the progenitor-to-intermediate exhausted, yet primed T cell reservoir. Our results are consistent with the emerging principle, established in melanoma and chronic infection models, that the differentiation state of tumor-infiltrating CD8⁺ T cells, specifically the presence of a TCF1⁺ stem-like progenitor subset, is a stronger predictor of checkpoint blockade response than overall T cell infiltration density [42, 43].

For whole-cell vaccines, the immunological outcome is strongly shaped by the mode of leukemia cell death used for vaccine preparation. ICD is characterized by stress-coupled regulated cell death, with the emission/exposure of DAMPs (e.g., calreticulin, ATP, HMGB1) that activate antigen-presenting cells for cross-priming [44, 45]. Synthetic coumarin derivatives, including OT52 and OT-55, induce ER stress signaling pathways mechanistically linked to ICD and immunostimulatory alarmin release [11, 12]. In line with this paradigm, other ICD-inducing agents, such as the platinum pyrophosphate PT-112, promote DAMP release and can synergize with immune checkpoint blockade [46]. Together, these data support ER-stress-driven ICD as a rational adjuvant principle to counter AML-associated immunosuppression by promoting inflammatory antigen presentation rather than tolerogenic clearance [22].

The differential functional contribution of individual DAMPs to OT-55-mediated ICD can be mechanistically explained by the redox context of OT-55-induced apoptosis. OT-55 generates a sustained, progressive oxidative stress in AML cells, peaking at 24 h and partially suppressed by the antioxidant NAC, without NAC rescuing cell viability (Supplemental Fig. 3E). HMGB1 immunostimulatory activity is critically dependent on its redox state: whereas the reduced and C23–C45 disulfide forms of HMGB1 engage pattern recognition receptors, the fully oxidized sulfonyl form, favored under conditions of sustained ROS, is immunologically inert. The oxidative microenvironment induced by OT-55 thus provides a mechanistic basis for HMGB1’s functional redundancy in this context. CRT translocation and ATP release, driven by ER stress and autophagy-dependent pathways that appear to operate through mechanisms distinct from the oxidative conversion of HMGB1, may not be equivalently impaired, positioning them as the dominant immunogenic effectors of OT-55-driven ICD. These findings show that the DAMP hierarchy in drug-induced ICD is not merely stimulus-dependent but is also shaped by the dying cell’s redox state.

BH3 mimetics that target antiapoptotic BCL-2 family proteins (e.g., venetoclax, now a widely used therapeutic backbone in AML) primarily trigger intrinsic mitochondria-mediated apoptosis [47–49]. Canonical apoptosis, when not coupled to ICD-associated stress and DAMP release, is generally immunologically silent and may fail to generate the danger signals required for durable antitumor immunity [44, 50]. Our results are consistent with this concept: vaccination with BH3 mimetic-treated cells alone was insufficient, whereas adding OT-55 during vaccine preparation substantially improved tumor rejection, indicating that the immune benefit is primarily provided by OT-55-driven adjuvanticity rather than by apoptotic priming per se. Moreover, resistance to BH3-mimetic-based therapy remains common (e.g., in FLT3-ITD⁺ or TP53-mutated AML) [51, 52], underscoring the need for immune-engaging strategies that could act across molecular subtypes.

The synergistic apoptotic and immunogenic effects of OT-55 and A-1331852 can be mechanistically rationalized through dual-axis convergence on the BCL-2 rheostat. A-1331852-mediated Bcl-xL inhibition and its associated phosphorylation reduce the sequestration of Bim, the principal executioner BH3-only protein in this cellular context, as confirmed by Bim upregulation at 6 h under combination treatment (Supplemental Fig. 4K, L). OT-55-induced ER stress simultaneously suppresses Mcl-1, the residual anti-apoptotic brake that would otherwise buffer free Bim, through ISR-mediated translational repression, and induces NOXA to competitively displace Bim from Mcl-1. The net result is a convergent increase in total Bim levels that, in the context of reduced Bcl-xL and Mcl-1 activity, is consistent with exceeding the remaining buffering capacity of anti-apoptotic proteins (a model that co-immunoprecipitation of Bim with Bcl-xL and Mcl-1 would directly corroborate in future studies), producing the robust caspase-3 cleavage observed at 6 h (Supplemental Fig. 4M, N). This mechanism positions OT-55 not merely as an ICD inducer but as a BCL-2 network sensitizer that dismantles the Mcl-1-dependent escape route left open by selective Bcl-xL inhibition, providing a mechanistic rationale for the observed immunogenic synergy.

The use of A-1331852, a selective Bcl-xL inhibitor, in this study warrants contextualization within the current AML therapeutic landscape. Venetoclax combined with azacitidine has become a standard-of-care backbone for newly diagnosed AML patients ineligible for intensive chemotherapy, exploiting Bcl-2 dependence across a broad range of AML subtypes. The choice of A-1331852 in the present study was dictated by the molecular profile of the C1498 model system, which exhibits high basal Bcl-xL and low Bcl-2 expression, rendering venetoclax mechanistically inappropriate for this cellular context. Importantly, in the prophylactic vaccination models that constitute the primary experimental framework of this study, A-1331852 is used ex vivo exclusively, and animals are never exposed to the drug systemically, thereby precluding any in vivo thrombocytopenic effect. In the bilateral tumor model, sub-systemic intratumoral dosing was employed, and platelet counts remained stable (Fig. 7C); however, it should be noted that murine models are known to underpredict Bcl-xL inhibitor-induced thrombocytopenia in humans due to species differences in platelet Bcl-xL versus Mcl-1 dependency, and murine tolerability data should not be extrapolated directly to clinical settings [53, 54]. From a translational perspective, the immunogenic adjuvant principle demonstrated by OT-55 is not inherently tied to Bcl-xL inhibition: in AML subtypes with predominant Bcl-2 dependence, an analogous strategy pairing OT-55 with venetoclax could be envisioned, and whether the ICD-converting capacity of OT-55 extends to other BH3 mimetics, including venetoclax, remains to be determined in appropriate Bcl-2-dependent model systems. Future studies should formally test this combination in Bcl-2-dependent AML models and explore whether the immunogenic adjuvant effect of OT-55 can enhance the emerging immunological dimension of venetoclax-based regimens.

Across prophylactic, therapeutic, and distant (abscopal) models, vaccination with OT-55- and A-1331852-treated cells combined with PD-1 and Tim-3 blockade yielded the most consistent tumor rejection compared with monotherapy or dual combinations, outperforming checkpoint inhibition alone. Vaccination increased the frequencies of CD4⁺, CD8⁺, cytotoxic, and effector T cells in draining lymph nodes, supporting improved priming. This is mechanistically coherent with AML biology, as PD-1 and Tim-3 co-expression marks dysfunctional/exhausted T cells in AML, and Tim-3/galectin-9 signaling has been implicated in immune escape and leukemia stem cell programs [15, 17, 55]. Consistent with other vaccine platforms, checkpoint blockade can augment vaccine-driven expansion and differentiation of tumor-reactive T cells [56, 57].

We also note that dedicated anti-PD-1 and anti-Tim-3 monotherapy arms were not included in the prophylactic vaccination model (Fig. 3); however, both agents showed no significant single-agent activity in the bilateral tumor model (Fig. 7B), consistent with the well-documented limited efficacy of checkpoint monotherapy in AML, where prior immunogenic priming appears necessary to generate a sufficient antigen-specific effector pool [58].

Formal kinetic analysis of tumor growth slopes after establishment confirmed that the combination regimen produces durable suppression of tumor expansion, reducing post-establishment growth rates by approximately 48% compared to untreated controls. The stepwise reduction in growth slope with the addition of anti-PD-1 and anti-Tim-3 to the OT-55/A-1331852 vaccination backbone is consistent with checkpoint blockade augmenting the vaccine-primed CD8⁺ effector T cell response, rather than simply shifting the timing of tumor appearance. We note that tumors eventually progressed in all groups, indicating that the combination achieved sustained growth suppression rather than durable eradication.

Future studies should define the antigen specificity, breadth, and durability of the T cell response induced by OT-55-based whole-cell vaccination, including memory formation and the contribution of cross-presenting dendritic cells [59]. Overall, these data position ER-stress-driven ICD as the central feature enabling whole-cell AML vaccination to counter AML-associated immunosuppression, while checkpoint blockade sustains and amplifies the vaccine-primed effector response.

Several limitations of this study merit explicit discussion. The preclinical functional data, including DAMP dependency testing, vaccination efficacy, CD8⁺ T cell depletion, antigen-specific tetramer responses, and distant (abscopal) tumor control, were generated exclusively in the C1498 murine cell line, a syngeneic FAB-M4 model. AML is a genetically and immunologically heterogeneous disease, and the immunogenic properties of OT-55-induced cell death, as well as the specific DAMP hierarchy and checkpoint dependencies described here, may not be conserved across AML subtypes. In particular, AML subtypes enriched for transcription factor mutations or DNA-methylation regulator alterations, which were overrepresented in the ICD-low patient group in our bioinformatic analysis, may exhibit distinct immunological barriers that require different or additional therapeutic strategies. The selection of C1498 was motivated by the identification of myelomonocytic M4 AML as the subtype with the highest ICD transcriptional scores and the most pronounced T cell exhaustion signature in our patient data; nevertheless, validation in additional murine AML models representing other FAB subtypes and genetic backgrounds, as well as in primary AML patient samples, will be required before broader clinical translation can be considered. These findings should therefore be interpreted as a proof-of-concept demonstration that ER-stress-driven ICD induction can convert tolerogenic apoptosis into immunogenic priming in a myelomonocytic AML context, rather than as evidence of universal applicability across the full AML disease spectrum.

## Materials and methods

### Acquisition of gene expression and clinical data

All analyses were conducted using publicly available human AML datasets in accordance with the guidelines of the Institutional Review Board (IRB) of Seoul National University, Seoul, South Korea. This study was exempt from IRB review (IRB No. E2503/004-005).

The results of The Cancer Genome Atlas (TCGA) AML cohorts are based in whole or in part on data generated by the TCGA Research Network (https://www.cancer.gov/tcga) and by the Therapeutically Applicable Research to Generate Effective Treatments (TARGET) initiative (https://www.cancer.gov/ccg/research/genome-sequencing/target) [60].

Pediatric patients with de novo AML enrolled in Children’s Oncology Group (COG) clinical trials CCG-2961, AAML03P1, and AAML0531, with written informed consent and available clinical and/or molecular annotations, were included in the TARGET-AML cohort. This cohort represents an aggregated dataset derived from multiple COG trials and genomic profiling platforms. For the present study, harmonized gene expression data provided by the TARGET Data Coordinating Center were used to enable cross-study comparability (dbGaP accession phs000465.v23.p8).

RNA sequencing data and mutation data for the TARGET-AML and TCGA-LAML cohorts were retrieved from the Genomic Data Commons (GDC) using the GDCquery and GDCdownload functions of the TCGAbiolinks R package. For both cohorts, raw read counts, transcript-per-million (TPM) expression matrices for protein-coding genes, and simple nucleotide variation (SNV) profiles processed through the GDC harmonized pipelines were downloaded. Clinical metadata, including age, sex, vital status, and follow-up duration, were obtained through the same framework and integrated with the molecular data. To ensure biological consistency within the TARGET-AML cohort, only RNA-seq and SNV samples derived from primary bone marrow were retained, which corresponded to patients whose sample type codes were annotated as “09” in the GDC barcode system.

Gene expression and mutation data for the BEAT-AML cohort [61] were retrieved from the Vizome database (http://www.vizome.org/aml2/). Additionally, cBioPortal [61] was used to access processed data, including raw read counts, RPKM-normalized expression values, high-confidence SNV calls, and comprehensive clinical annotations such as diagnostic category, treatment history, and survival outcomes. To match the inclusion criteria applied to the other cohorts, only samples collected at the time of initial diagnosis and derived from bone marrow aspirates were included; samples meeting all the following conditions were retained:

DISEASE_STAGE_AT_SPECIMEN_COLLECTION = “Initial Diagnosis,” SPECIMEN_TYPE = “Bone Marrow Aspirate,” and SPECIMEN_GROUPS = “Initial Acute Leukemia Diagnosis.”

For the GSE37642 cohort, microarray-based gene expression data generated on the GPL570 (Affymetrix Human Genome U133 Plus 2.0) platform were downloaded directly from the Gene Expression Omnibus (GEO), along with the corresponding clinical metadata. The clinical variables were harmonized with the expression matrices and incorporated into the downstream analytical pipeline. Across all cohorts, only bone marrow-derived samples collected at initial diagnosis were used to minimize biological heterogeneity and ensure comparability among datasets.

### AML ICD signature scoring and stratification

To identify ICD-related transcriptional markers relevant to AML, we evaluated the prognostic associations of 34 previously reported ICD-associated genes across three independent AML cohorts (TARGET-AML, BEAT-AML, and GSE37642). For each gene, patients were dichotomized into high- and low-expression groups using cohort-specific median values. Univariate Cox proportional hazards regression was performed separately within each cohort to assess overall survival, and genes consistently associated with a favorable prognosis (hazard ratio < 1) across all three datasets were defined as prognostically favorable; the HR < 1 criterion was applied independently in each cohort, and the intersection of the three resulting gene sets defined the final signature. This process yielded nine shared ICD-related genes (*ATG5, CALR, CD8A, CD8B, IFNGR1, IL1B, PDIA3, PIK3CA* and *TLR4*). An AML ICD score was calculated for each patient by applying GSVA to log₂(TPM + 1)-transformed expression matrices, using the nine retained prognostically favorable genes as the input gene set. GSVA was implemented with the *GSVA* R package (version 2.4.8) (method = “gsva”, kcdf = “Gaussian”), yielding a single per-sample enrichment score. Patients were then stratified into AML ICD-high and AML ICD-low groups based on cohort-specific median GSVA scores. These groups were utilized in downstream analyses, including survival modeling, immune profiling, and characterization of the mutational landscape.

### Survival analysis

Survival analyses were performed to investigate the prognostic relevance of the AML ICD score and each ICD-related gene. Across the three AML cohorts (TARGET-AML, BEAT-AML, and GSE37642), patients were stratified into high- and low-expression groups based on the median expression value of each gene or the median AML ICD score. Kaplan–Meier survival curves were generated, and differences between groups were evaluated using the log-rank test. Effect sizes were estimated using Cox proportional hazards regression models, yielding hazard ratios (HRs) and corresponding *P*-values. All survival analyses were conducted in R (v4.3.0) using the survival package (v3.4-0).

### Differential expression analysis and functional enrichment

Differential expression analysis was performed between the high and low AML ICD score groups using the DESeq2 R package (v3.15), which employs Wald statistical tests. Differentially expressed genes (DEGs) were defined based on a false discovery rate (FDR)-adjusted *P*-value < 0.05 and an absolute log2 fold-change > 1.

For functional annotation of DEGs, GSEA was performed using the fgsea R package with the following parameters: minSize = 10, maxSize = 500, and nperm = 100,000. Genes were ranked using test statistics from the DESeq2 analysis, and pathways enriched in either direction were identified.

### Immune cell infiltration analysis using CIBERSORTx

Immune cell composition was estimated using CIBERSORTx (https://cibersortx.stanford.edu), a computational algorithm that estimates the relative proportions of immune cell types from bulk tissue gene expression profiles [62]. CIBERSORTx infers the relative proportions of immune cell subsets by deconvoluting bulk transcriptomic profiles against reference signatures derived from purified immune populations. In this study, the relative abundance of tumor-infiltrating immune subsets was inferred using CIBERSORTx with the LM22 leukocyte gene signature matrix and 1000 permutations, following the procedure described by Newman et al. [62].

For each cohort, inferred immune cell fractions were obtained for all patient samples and compared between the AML ICD-high and AML ICD-low groups. Statistical significance was assessed using two-sided Student’s *t*-tests, and resulting *P*-values were adjusted for multiple comparisons using the FDR method.

### Association between AML ICD score and blast maturation stage (FAB classification)

AML subtypes were stratified using the FAB classification system, a well-established morphological framework widely used in AML research [63], to ensure consistent and comparable subtype assignment throughout the study.

FAB classification data were extracted from the clinical metadata of the three AML cohorts to examine differences in AML ICD scores across blast maturation stages. AML ICD score distributions were compared across FAB subtypes, and pairwise statistical comparisons were performed to identify subtypes exhibiting significantly higher scores. One-sided Student’s *t*-tests (greater alternative) were used, and *P*-values were corrected for multiple testing using the FDR method.

### Categorization of somatic mutations and integration with clinical features

Somatic mutation data from AML patients were obtained from the GDC and cBioPortal and processed after harmonizing patient identifiers and clinical annotations. Genes were grouped into seven biologically relevant functional categories: DNA methylation, RNA splicing, epigenetic modifiers, transcription factors, activated signaling pathways, tumor suppressors, and the cohesin complex, following the AML molecular classification proposed by Saygin et al. [64].

For each sample, mutation status was summarized at the gene level and converted into a presence-absence matrix restricted to the predefined driver categories. Clinical features, including AML ICD scores, age, and gender, were integrated as sample-level annotations. The mutation landscape was visualized using the ComplexHeatmap package (version 2.22.0), which enabled simultaneous representation of gene categories, mutation frequencies, and clinical covariates across patients.

### scRNA-seq data analysis

scRNA-seq data were obtained from the Single Cell Pediatric Cancer Atlas (scPCA), an open-access resource provided by Alex’s Lemonade Stand Foundation for Childhood Cancer (https://scpca.alexslemonade.org). Data were accessed and analyzed according to the scPCA guidelines. scRNA-seq datasets were analyzed using Seurat in R. To characterize cellular heterogeneity within individual AML patient samples, scRNA-seq data were annotated using the BoneMarrowMap package in R. This approach enabled the classification of blast-like cell populations based on transcriptional similarity to normal bone marrow lineages. For downstream analyses, blast-like subpopulations were grouped according to analysis-relevant cellular identities, and gene expression was compared between the cell types of interest and all other blast-like compartments within each patient. Patient information and blast-specific gene expression comparison datasets are provided separately in Supplemental Table 2.

### Single-cell T cell exhaustion phenotyping

To classify the exhaustion state of bone marrow CD8⁺ T cells, we assessed gene expression of markers spanning the four-stage Tex developmental hierarchy defined by Beltra et al. [28]: progenitor markers (*TCF7/TCF-1, SLAMF6/Ly108*), intermediate-exhausted/effector markers (*KLRG1, ZNF683/Hobit, GZMB, PRF1, CX3CR1, CD244/2B4, IFNG-AS1*), checkpoint receptors (*LAG3, CD96*), and terminal exhaustion markers (*ENTPD1/CD39, TOX, TOX2, BTLA*). Marker expression was quantified as the fraction of cells expressing each gene (pct.1) in the T cell cluster relative to all other cell types (pct.2), with statistical significance assessed by the Wilcoxon rank-sum test and Bonferroni-adjusted *P*-values, as implemented in Seurat’s FindMarkers function. Log₂ fold-change and adjusted *P*-values were computed across 7 AML patients (2 M4, 5 M5, including 1 FLT3-ITD) and 2 healthy bone marrow controls from the scRNA-seq dataset described in Supplemental Table 2. T cell exhaustion staging was assigned based on concordance with published marker profiles: Texprog1/2 (TCF7⁺, SLAMF6⁺, CD39^−^, TOX2^−^), Texint (TCF7^−^, KLRG1⁺, CX3CR1⁺, GZMB⁺, PRF1⁺, ZNF683⁺), and Texterm (TCF7^−^, CD39⁺, TOX₍hi₎, TOX2⁺, BTLA⁺) [28, 29].

### Compounds

3,3’-[3-(5-chloro-2-hydroxyphenyl)-3-oxopropane-1,1-diyl] bis(4-hydroxycoumarin) OT-55 was synthesized according to a previously reported procedure [65]. Cisplatin and mitoxantrone were purchased from Sigma-Aldrich (St. Louis, MO, USA). A-1331852 was purchased from Selleckchem (Houston, TX). Caspase inhibitor 1 (zVAD-FMK, 627610) was obtained from Calbiochem (San Diego, CA). The InvivoMAb anti-mouse CD4, InvivoMAb anti-mouse CD8, InVivoPlus anti-mouse PD-1 (CD279; BP0273), and InVivoPlus anti-mouse Tim-3 (CD366; BP0115) antibodies were purchased from Bio X Cell (Lebanon, New Hampshire, USA).

### Cell culture

The murine acute myeloid cell line, C1498, was purchased from the American Type Culture Collection (ATCC, Manassas, VA, USA). Ovalbumin-expressing C1498 were kindly provided by Prof. Yeonseok Chung (Seoul National University, Korea). C1498 and Ovalbumin-expressing C1498 were cultured in DMEM medium (Gibco, Grand Island, NY, USA) supplemented with 10% (*v*/*v*) fetal bovine serum (FBS) (Biowest, Riverside, MO, USA) and 1% (*v*/*v*) penicillin–streptomycin (GenDEPOT, Baker, USA) at 37 °C and 5% CO₂.

After 48 h, cell viability was assessed, and all living cells were resuspended in 30 mL of fresh, complete cell culture medium in a 75 cm² flask. Cells were counted and seeded at a density of 2 × 10⁵ cells/mL in 30 mL of fresh complete growth medium. Cells were cultured according to standard protocols, and mycoplasma testing was performed monthly using the MycoAlert™ PLUS Mycoplasma Detection Kit (Lonza, Walkersville, MD, USA).

### Cell proliferation and viability

Cell proliferation and viability were measured by Trypan Blue staining (Lonza, Walkersville, MD, USA). The number of cells per milliliter was counted, and the fraction of trypan blue-positive cells was estimated using a Malassez cell counting chamber (Marienfeld, Lauda-Königshofen, Germany). Apoptosis was evaluated by detecting phosphatidylserine exposure and quantified using flow cytometry (FACSLyric, BD Biosciences) after annexin V and propidium iodide (PI) staining. The Annexin V-Fluorescein Isothiocyanate (APC) Apoptosis Detection Kit I (BD Biosciences, San Jose, CA, USA) was used according to the manufacturer’s protocol.

### Measurement of extracellular ATP content

Extracellular ATP levels in the supernatant were assessed using the ENLITEN® ATP Assay System bioluminescence detection kit (Promega, Madison, WI, USA), following the manufacturer’s protocol. Briefly, C1498 cells were seeded at 2 × 10⁵ cells/mL and treated with the indicated concentrations of OT-55, mitoxantrone, cisplatin, or A-1331852 for the indicated time periods. After treatment, the supernatants were collected by centrifugation, and 100 µL of reconstituted rluciferase/luciferin (rL/L) reagent was added to 100 µL of the supernatant. Light output was measured immediately using a SpectraMax i3x microplate reader (Molecular Devices, San Jose, CA, USA).

### Analysis of ectopic calreticulin

After treatment, cells were collected and washed twice with 1× phosphate-buffered saline (PBS). Cells were incubated for 40 min with the primary antibody for calreticulin (CRT) (Abcam,2907, Cambridge, UK), diluted in buffer (2% FBS in 1× PBS), followed by washing and incubation with the Alexa488-conjugated monoclonal secondary antibody (Invitrogen, Grand Island, NY, USA) in the buffer (for 40 min) and fixed in fixation buffer (Biolegend, San Diego, CA, USA) for 10 min at 4 °C. After washing twice in PBS, each sample was analyzed using fluorescence-activated cell sorting (FACS; FACSLyric, BD Biosciences) and confocal microscopy (Leica Microsystems, Wetzlar, Germany). Isotype-matched IgG antibodies (Abcam) were used as the controls.

### Detection of HMGB1 release

Cells were seeded in 1 mL of medium. After treatment, the cells were centrifuged, and the supernatant was collected. HMGB1 release into the supernatant was quantified using an enzyme-linked immunosorbent assay kit (Shino-Test-Corporation, Tokyo, Japan) according to the manufacturer’s instructions.

### Protein extraction and Western blots

Whole-cell extracts were prepared using M-PER® (Thermo Fisher Scientific, San Jose, CA, USA) supplemented with 1% PhosStop (Roche, Basel, Switzerland), 1 mM of sodium orthovanadate (JUNSEI, Tokyo), 0.5% Complete (Sigma-Aldrich, St. Louis, MO, USA), 5 mM sodium fluoride (JUNSEI, Tokyo), 1 mM of phenylmethylsulfonyl fluoride (PMSF). Proteins were resolved by SDS-PAGE and transferred to PVDF membranes (GE Healthcare, Waukesha, WI, USA). Membranes were incubated with selected primary antibodies (Supplemental Table 7). The chemiluminescence signal was detected with an ECL Plus Western Blotting Detection System (GE Healthcare) and quantified with an ImageQuant LAS-4000 mini system (GE Healthcare).

### Prophylactic vaccination of mouse models

Wild-type C57BL/6 mice (4–6 weeks old) were purchased from Orient Bio (Orient Bio Inc., Seongnam, Korea). Animals were maintained at the College of Pharmacy Animal Center, Seoul National University, and the experiments were conducted in accordance with the Guidelines for the Care and Use of Laboratory Animals. All mice had unlimited access to water and food and were housed in pathogen-free cages containing wood shavings and bedding, with a 12-h light/dark cycle at a controlled room temperature of 25 °C. Animal experiments were approved by the Institutional Animal Care and Use Committee (IACUC) of Seoul National University (SNU-191106-1-1, SNU-210305-1-1, and SNU-220510-1).

C1498 cells were treated in vitro with OT-55, mitoxantrone, cisplatin, or A-1331852 for 24 h, washed with 1× PBS, and resuspended in 5 × 10⁵ cells/100 μL PBS for subcutaneous injection into the left flank of 7-week-old female C57BL/6 mice. One week later, the mice received live 5 × 10⁵ C1498 cells *s.c*. in the right flank. Tumor incidence and growth were monitored routinely using a digital Vernier caliper (SD500-150PRO, Sincon, South Korea). The mice were sacrificed when the tumor size exceeded the ethical limits.

### Immunodepletion

C1498 cells were treated with OT-55 in vitro for 24 h. After treatment, cells were incubated with 10 μg/10⁶ cells of HMGB1 antibody (Abcam, USA), 6 μg/10⁶ cells of CRT antibody (Abcam, USA), or 13 units/10⁶ cells of apyrase (Sigma-Aldrich, Germany). Control groups were treated with isotype control antibodies for CRT and HMGB1. For survival experiments, mice were monitored for tumor growth and euthanized before tumors reached 2500 mm³. Kaplan–Meier curves were used to calculate survival.

### Analysis of ROS generation

The DCFDA/H2DCFDA-Cellular ROS assay kit (Abcam, USA) was used according to the manufacturer’s instructions to measure cellular ROS levels. Samples were analyzed with FACSLyric (BD Biosciences), and data were analyzed using FlowJo software (BD Biosciences).

### Combination of prophylactic vaccination with immune checkpoint antibodies

C1498 cells were treated in vitro with drug treatments for 24 h, washed with 1× PBS, and resuspended in 5 × 10⁵ cells/100 μL PBS for subcutaneous injection into the left flank of 7-week-old female C57BL/6 mice. One week later, the mice received untreated C1498 cells *s.c*. in the right flank. Immune checkpoint antibodies were injected *i.p*. at 200 μg for anti-PD-1 (CD279) and/or anti-Tim-3 (CD366). Tumor incidence and growth were monitored routinely using a digital Vernier caliper (SD500-150PRO, Sincon, South Korea). The mice were sacrificed when the tumor size exceeded the ethical limits. Antibodies were purchased from BioXCell (West Lebanon, NH, USA). For survival experiments, mice were monitored for tumor growth and euthanized before tumors reached 2500 mm³. Kaplan–Meier curves were used to calculate survival.

### T cell subset depletion

Anti-CD4 (GK1.5) and anti-CD8 (2.43) antibodies (BioXCell, USA) were administered intraperitoneally at 150 μg/200 μL of PBS every 3 days, starting on the day of vaccination, to deplete the targeted cell populations in vivo. For survival experiments, mice were monitored for tumor growth and euthanized before tumors reached 2500 mm³. Kaplan–Meier curves were used to calculate survival.

### Tetramer-based quantification of OVA-specific CD8^+^ T cells by flow cytometry

Mice were vaccinated with C1498 and OVA-C1498 cells treated with OT-55 and A-1331852. After one week, mice were sacrificed, and cells were isolated from the spleen and lymph nodes. For detection of OVA-specific CD8⁺ T cells, cells were stained with PE-conjugated H-2Kb OVA257-264 (SIINFEKL) tetramers (NIH Tetramer Core Facility), followed by surface staining with anti-CD3 and anti-CD8 antibodies. PE-conjugated H-2Kb OVA257-264 (SIINFEKL) tetramers were obtained from the NIH Tetramer Core Facility (NIH Contract 75N93020D00005, RRID:SCR_026557).

### Flow cytometry

The cells were isolated from the spleen and lymph nodes. Cells were obtained and stained with an appropriate panel of antibodies listed in Supplemental Table 7. LIVE/DEAD fixable dye was used. For intracellular staining, cells were incubated for 3 h at 37 °C in complete media containing PMA, ionomycin, brefeldin A, and monesin, using the Cytofix/Cytoperm kit (BioLegend). An appropriate panel of antibody staining followed this. Samples were analyzed with FACSLyric (BD Biosciences), and data were analyzed using FlowJo software (BD Biosciences).

### Bilateral tumor (abscopal) assay

Tumors were established by subcutaneous injection of 5 × 10⁵ C1498 cells into the left flank of 7-week-old female C57BL/6 mice on day 0, followed by injection of 5 × 10⁵ C1498 cells into the right flank on day 3, to generate a bilateral tumor model. Tumor incidence and growth were monitored routinely using a digital Vernier caliper SD500-150PRO (Sincon, South Korea). When the primary tumors reached 65-100 mm³ (day 0 of treatment), mice were injected with the following treatment groups: (1) 100 µL vehicle *i.t*. on day 0; (2) mitoxantrone 8 mg/kg *i.t*. on day 0; (3) OT-55 80 mg/kg and A-1331852 10 mg/kg *i.t*. on day 0; (4) anti-PD-1 (CD279), 200 µg/mouse in 100 µL *i.p*., on days 2, 5 and 8; (5) anti-Tim-3 (CD366), 200 µg/mouse in 100 µL *i.p*., on days 2, 5 and 8; (6) OT-55 80 mg/kg and A-1331852 10 mg/kg *i.t*. on day 0, and anti-PD-1 (CD279), 200 µg/mouse in 100 µL *i.p*., on days 2, 5 and 8; (7) OT-55 80 mg/kg and A-1331852 10 mg/kg *i.t*. on day 0, and anti-Tim-3 (CD366), 200 µg/mouse in 100 µL *i.p*., on days 2, 5 and 8; (8) OT-55 80 mg/kg and A-1331852 10 mg/kg *i.t*. on day 0, and anti-PD-1 (CD279) and anti-Tim-3 (CD366), 200 µg/mouse in 100 µL *i.p*., on days 2, 5 and 8. Tumor incidence and growth were monitored routinely using a digital Vernier caliper SD500-150PRO (Sincon, South Korea).

### CBC

Blood samples were collected from the mice by eye bleeding and stored in ethylenediaminetetraacetic acid (EDTA)-coated tubes. According to the manufacturer’s instructions, a blood cell analyzer measured the following cell types: neutrophils, lymphocytes, monocytes, eosinophils, basophils, and platelets (XN-V, Sysmex, Japan). The normal reference ranges for CBC parameters were sourced from publicly available data provided by Charles River Laboratories (USA).

### Biochemical analysis

Blood samples were collected from the mice’s eyes. Serum was obtained by centrifugation at 4000 rotations per minute (rpm) for 15 min. The levels of alanine aminotransferase (ALT), aspartate aminotransferase (AST), blood urea nitrogen (BUN), and creatinine (CRE) were measured using a FUJI DRI-CHEM 3500 s automated clinical chemistry analyzer (Fujifilm, Japan) according to the manufacturer’s instructions.

### Statistical analysis

All data are expressed as mean ± SD. Statistical significance was evaluated using the *t*-test and one-way or two-way ANOVA, as indicated in the Figure legends. Tukey’s test was performed using GraphPad Prism 10 software (La Jolla, CA, USA). Statistical significance is reported as follows: ^*^P < 0.05, ^**^P < 0.01, ^***^P < 0.001, and ^****^P < 0.0001.

### Use of generative AI and AI-assisted technologies in the writing process

During the preparation of this work, the author(s) used Grammarly.com and Claude to improve language and readability. After using these tools, the author(s) reviewed and edited the content as needed and took full responsibility for the publication’s content.

## Reporting summary

Further information on research design is available in the Nature Research Reporting Summary linked to this article.

## Supplementary information

Supplementary figures, legends, tables

Reporting Summary

Lee_Original Western blots

## Data Availability

The data supporting this study’s findings are available from the corresponding author upon request.
