# Supplementary material for: OT-55 reshapes tolerogenic BH3-mimetic-induced apoptosis toward immunogenic cell death in acute myeloid leukemia, potentiating PD-1/Tim-3 blockade
Source: Cell Death Dis. 2026 Jul 1;17(1):821. doi: 10.1038/s41419-026-09000-9 (PMC13594238; doi:10.1038/s41419-026-09000-9)
Supplement: Supplementary file 1 [file 41419_2026_9000_MOESM1_ESM.pdf]

Supplemental Figures

OT-55 reshapes tolerogenic BH3-mimetic-induced apoptosis toward immunogenic cell death in acute myeloid leukemia, potentiating PD-1/Tim-3 blockade

Yejin Lee[1], Eun-Ji Kwon[1], Sruthi Reddy Gajulapalli[1], Ji Yeon Paik[1], Barbora Orlikova-Boyer[2,&], Artur M.S. Silva[3], Hyuk-Jin Cha[1], Claudia Cerella[2,&], and Marc Diederich[1]*

[1] Department of Pharmacy, College of Pharmacy, Seoul National University, 1 Gwanak-ro, Gwanak-gu, Seoul, 08626, Korea

[2] Laboratoire de Biologie Moléculaire du Cancer (LBMCC), BAM3 Pavillon 2, 6A rue Nicolas-Ernest Barblé, L-1210 Luxembourg, Luxembourg

[3] Department of Chemistry & REQUIMTE, University of Aveiro, 3810-193, Aveiro, Portugal

***Corresponding author:**

Marc Diederich, Research Institute of Pharmaceutical Sciences & Natural Products Research Institute, College of Pharmacy, Seoul National University, Seoul 08826, Republic of Korea. Email: marcdiederich@snu.ac.kr

Present address: [&]Department of Cancer Research, Luxembourg Institute of Health (LIH), BAM3 Pavillon 2, 6A rue Nicolas-Ernest Barblé, L-1210 Luxembourg, Luxembourg.

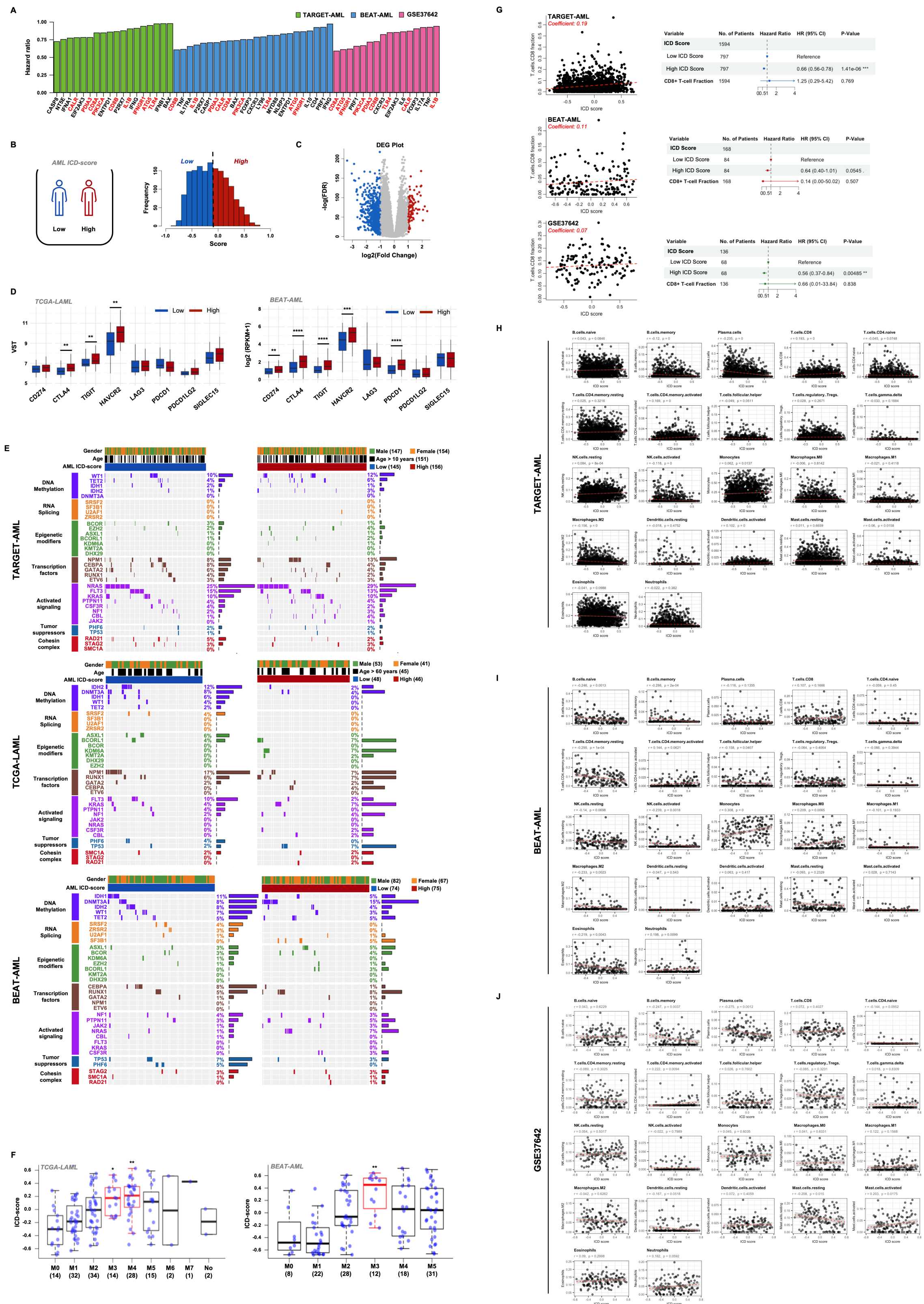
A
TARGET-AML
BEAT-AML
GSE37642
Hazard ratio
B
AML ICD-score
Low
High
Frequency
Score
C
DEG Plot
-log(FDR)
log2(Fold Change)
D
TCGA-LAML
BEAT-AML
VST
log2 (RPKM+1)
CD274
CTLA4
TIGIT
HAVCR2
LAG3
PDCD1
PDCD1LG2
SIGLEC15
E
TARGET-AML
TCGA-LAML
BEAT-AML
Gender
Age
AML ICD-score
DNA Methylation
RNA Splicing
Epigenetic modifiers
Transcription factors
Activated signaling
Tumor suppressors
Cohesin complex
F
ICD-score
G
Coefficient: 0.19
Coefficient: 0.11
Coefficient: 0.07
T cells CD8 fraction
ICD score
Variable
No. of Patients
Hazard Ratio
HR (95% CI)
P-Value
ICD Score
Low ICD Score
High ICD Score
CD8+ T-cell Fraction
Reference
H
I
J

Supplemental Figure 1. Integrated clinical, transcriptomic, and genomic characterization of AML cohorts stratified by ICD-score across TARGET-AML, TCGA-LAML, and BEAT-AML, and independence of the ICD score from immune cell infiltration. (A) Bar plot showing hazard ratios (HRs) for 34 previously reported ICD-related genes across the TARGET-AML (n = 1594), BEAT-AML (n = 168), and GSE37642 (n = 136) cohorts. Nine genes (*ATG5, CALR, CD8A, CD8B, IFNGR1, IL1B, PDIA3, PIK3CA* and *TLR4*) consistently exhibited HR < 1 across all three cohorts. **(B)** Stratification of TARGET-AML patients into AML ICD-high and AML ICD-low score groups based on the cohort-specific median AML ICD score. **(C)** Identification of differentially expressed genes (DEGs) between AML ICD-high and AML ICD-low score groups in the TARGET-AML cohort. **(D)** Comparison of immune checkpoint gene expression between AML ICD-high and AML ICD-low score groups in the TCGA-LAML and BEAT-AML cohorts. **(E)** Integrated oncoprint showing driver gene mutations in TARGET-AML (n = 301), TCGA-LAML (n = 94), and BEAT-AML (n = 149) cohorts, annotated with AML ICD score, sex, and age. **(F)** Distribution of AML ICD scores across French-American-British (FAB) subtypes in the TCGA-LAML and BEAT-AML cohorts. Each subtype was statistically compared against all other subtypes. Statistical significance for panels was assessed using unpaired one-tailed Student's *t*-tests. $^{*}P < 0.05$, $^{**}P < 0.01$, $^{***}P < 0.001$, and $^{****}P < 0.0001$. **(G)** Forest plots from multivariate Cox proportional hazards regression, including the AML ICD score and the CIBERSORTx-estimated $CD8^{+}$ T cell fraction as co-variables. Hazard ratios (HR) with 95% confidence intervals are shown for each cohort. The ICD score retained independent prognostic significance across all three cohorts; the $CD8^{+}$ T cell fraction was not significantly associated with overall survival in any cohort. **(H-J)** Pearson correlation scatter plots between the AML ICD score (x-axis) and each of the 22 CIBERSORTx-estimated immune cell fractions (y-axis) in the TARGET-AML **(H)**, BEAT-AML **(I)**, and GSE37642 **(J)** cohorts. Pearson *r* and associated p-values are indicated for each cell type. No consistent directional pattern is observed across cohorts, indicating that the ICD score does not reflect overall immune infiltration density or a single dominant immune subtype signature.

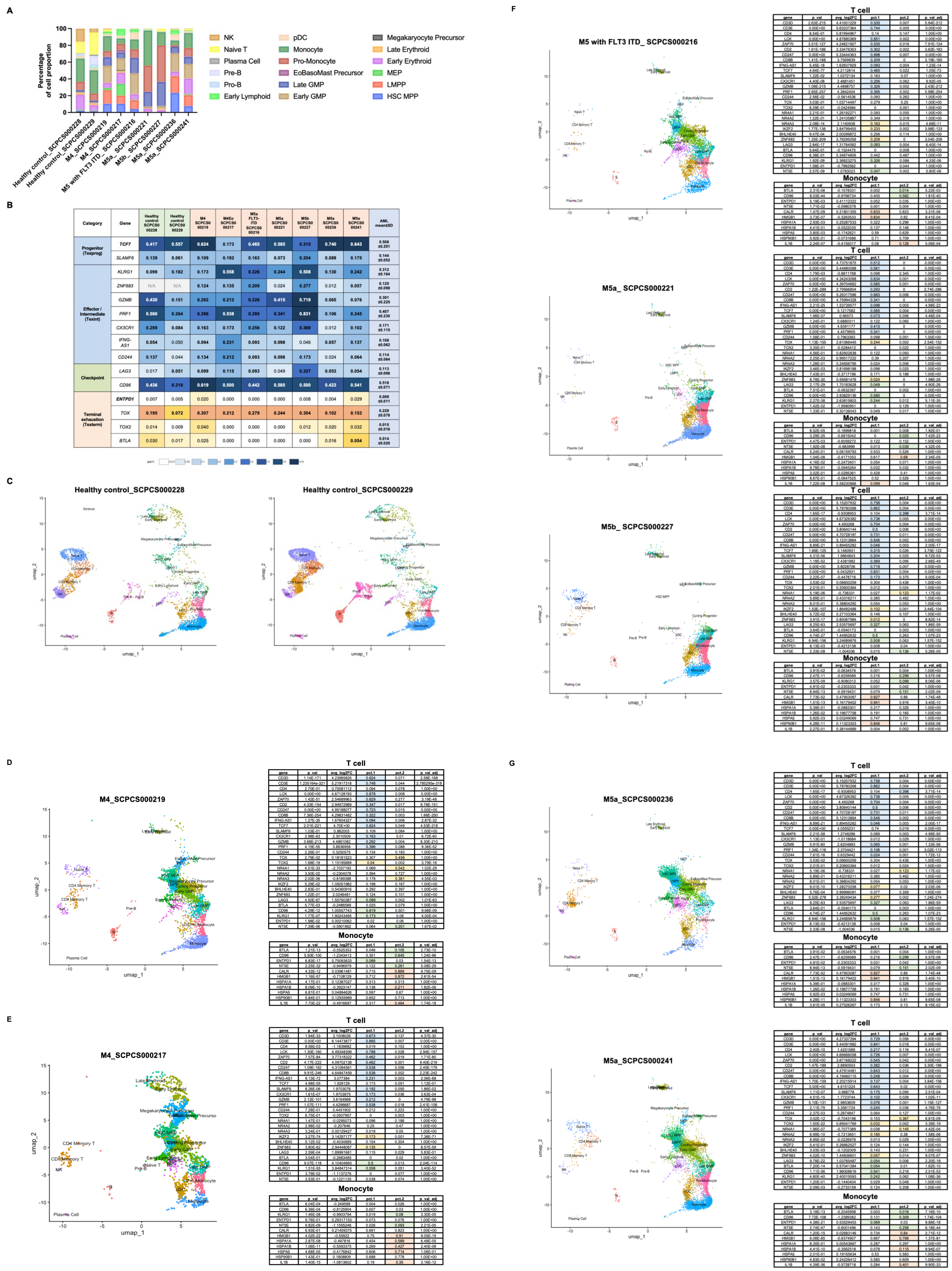
A
Percentage of cell proportion
NK
Naive T
Plasma Cell
Pre-B
Pro-B
Early Lymphoid
pDC
Monocyte
Pro-Monocyte
EoBasoMast Precursor
Late GMP
Early GMP
Megakaryocyte Precursor
Late Erythroid
Early Erythroid
MEP
LMPP
HSC MPP
Healthy control_SCPCS000228
Healthy control_SCPCS000229
M4_SCPCS000219
M4_SCPCS000217
M5 with FLT3 ITD_SCPCS000216
M5a_SCPCS000221
M5b_SCPCS000227
M5a_SCPCS000236
M5a_SCPCS000241
B
Category
Gene
Progenitor (Texprog)
Effector / Intermediate (Texint)
Checkpoint
Terminal exhaustion (Texterm)
TCF7
SLAMF6
KLRG1
ZNF683
GZMB
PRF1
CX3CR1
IFNG-AS1
CD244
LAG3
CD96
ENTPD1
TOX
TOX2
BTLA
C
Healthy control_SCPCS000228
Healthy control_SCPCS000229
D
M4_SCPCS000219
T cell
Monocyte
E
M4_SCPCS000217
T cell
Monocyte
F
M5 with FLT3 ITD_ SCPCS000216
M5a_ SCPCS000221
M5b_ SCPCS000227
T cell
Monocyte
G
M5a_ SCPCS000236
M5a_ SCPCS000241
T cell
Monocyte
umap_1
umap_2

Supplemental Figure 2. Single-cell resolution hematopoietic cell composition and T cell exhaustion staging of individual M4 and M5 AML patients and healthy controls from publicly available bone marrow scRNA-seq data (scPCA resource). Cell populations were annotated using the BoneMarrowMap reference. **(A)** Stacked bar plots showing hematopoietic cell composition for each individual sample (two healthy controls, two M4, one M5 with FLT3-ITD, and three M5a/M5b patients). Every individual AML patient showed near-complete absence of Naive T cells and other lymphoid populations, with the composition overwhelmingly dominated by malignant myeloid progenitor states (Early GMP, Late GMP, Pro-Monocyte), without exception across patients. Both healthy controls retained substantial Naive T, B cell, and NK cell fractions. **(B)** Heatmap summarizing pct.1 (fraction of T cells expressing each marker) and $\log_2$ fold-change across all nine samples (two healthy controls, seven AML) for the T cell exhaustion staging panel. Markers are grouped by functional category: progenitor (*TCF7, SLAMF6*), effector/intermediate (*KLRG1, ZNF683, GZMB, PRF1, CX3CR1, IFNG-AS1, CD244*), checkpoint (*LAG3, CD96*), and terminal exhaustion (*ENTPD1, TOX, TOX2, BTLA*). T cell exhaustion staging was assigned according to the four-stage Tex developmental hierarchy defined by Beltra *et al.* [1]: Texprog1/2 (TCF7^{+}, SLAMF6^{+}, CD39^{-}), Texint (TCF7^{-}, KLRG1^{+}, CX3CR1^{+}, GZMB^{+}, ZNF683^{+}), and Texterm (TCF7^{-}, CD39^{+}, TOX$_{(hi)}$, TOX2^{+}). AML bone marrow CD8^{+} T cells uniformly displayed retention of the progenitor marker *TCF7* (mean pct.1 = 0.506 ± 0.201) alongside significant expression of effector/intermediate markers (*KLRG1* mean = 0.312; *PRF1* = 0.407; *GZMB* = 0.301; *ZNF683* = 0.120), with near-complete absence of terminal exhaustion markers (*ENTPD1* mean = 0.009; *TOX2* = 0.015; *BTLA* = 0.014), consistent with a Texprog2 → Texint transition state that retains both self-renewal capacity and effector potential without terminal epigenetic commitment. This profile mirrors the ZNF683^{+} intermediate-exhausted CD8^{+} effector/effector-memory population associated with anti-PD-1 response in Richter syndrome bone marrow [2]. Values represent pct.1 (fraction of T cells expressing each marker vs all other cell types, Wilcoxon rank-sum test, Seurat FindMarkers). Sample headers: green = healthy control; orange = AML. Blue intensity scale: higher pct.1 = darker blue (progenitor/effector/checkpoint markers). Warm/amber scale (terminal markers): white = not expressed, amber = expressed. Staging per Beltra *et al.* [1]. **(C)** UMAP projections of single-cell transcriptomes from the two healthy control samples (Healthy control_SCPCS000228 and Healthy control_SCPCS000229), with cell type annotations. Clearly delineated Naive T, CD4 Memory T, CD8 Memory T, and NK

cell clusters are visible in healthy bone marrow, confirming that the annotation approach has sufficient resolution to detect lymphoid populations when genuinely present, and that their absence in AML patients reflects true cellular depletion rather than a technical limitation. **(D-G)** UMAP projections for each AML patient sample (M4_SCPCS000219, M4_SCPCS000217, M5 with FLT3-ITD_SCPCS000216, M5a_SCPCS000221, M5b_SCPCS000227, M5a_SCPCS000236, and M5a_SCPCS000241) with cell type annotations and corresponding T cell and monocyte marker expression tables. For each patient, differential gene expression analysis (Wilcoxon rank-sum test with Bonferroni correction, Seurat FindMarkers) was performed to compare the T cell cluster with all other cell types. Marker panels include T cell identity genes (*CD3D, CD3E, CD8B, LCK, ZAP70*), progenitor exhaustion markers (*TCF7, SLAMF6*), effector/intermediate exhaustion markers (*KLRG1, ZNF683, GZMB, PRF1, CX3CR1, IFNG-AS1, CD244*), terminal exhaustion markers (*ENTPD1/CD39, TOX, TOX2, BTLA*), checkpoint receptors (*LAG3, CD96*), and ICD/ER-stress markers (*CALR, HMGB1, HSPA1A, HSPA1B, HSPA5, HSP90B1*).

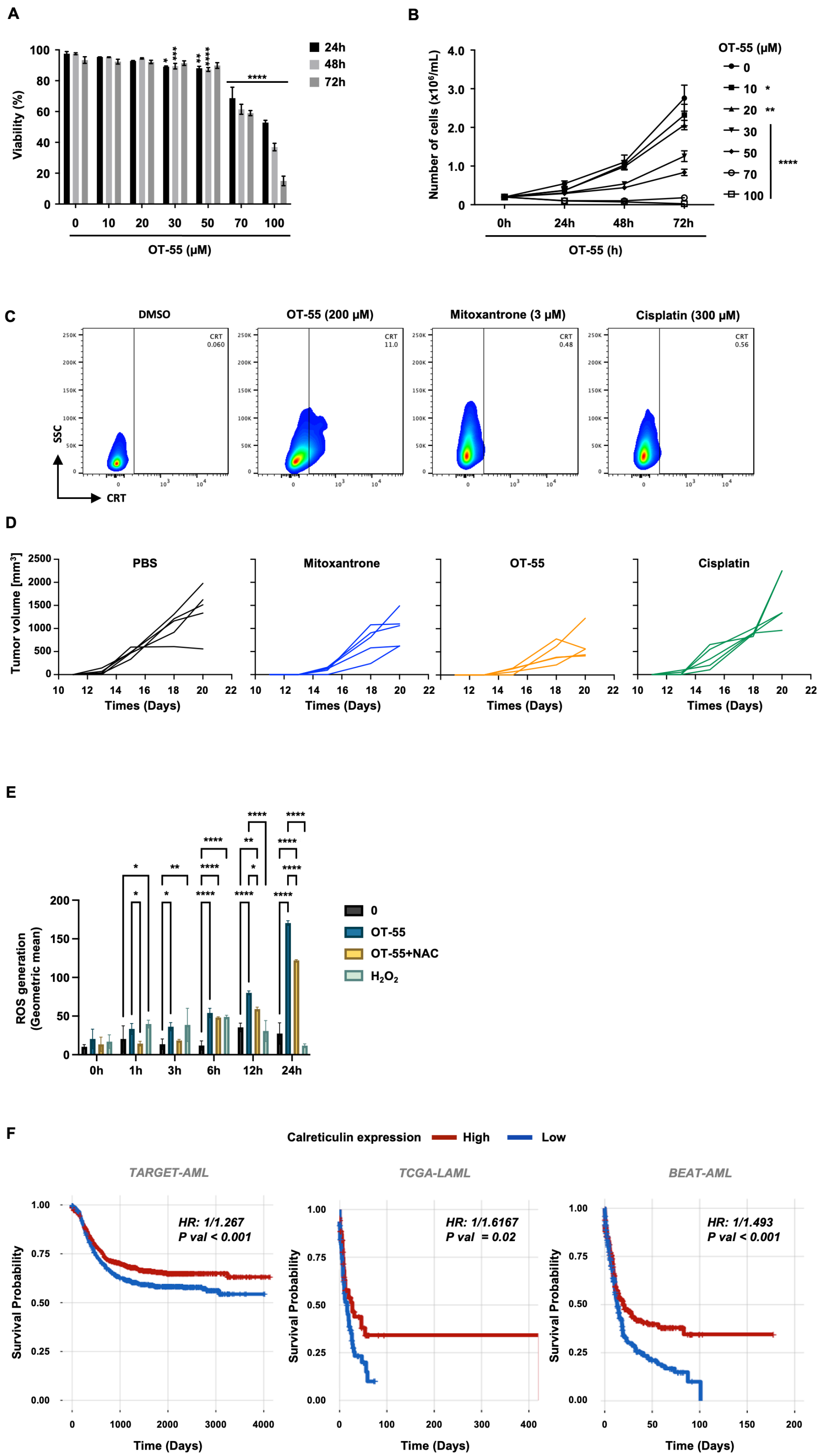
A
Viability (%)
24h
48h
72h
OT-55 (μM)
B
Number of cells (x10⁶/mL)
OT-55 (μM)
OT-55 (h)
C
DMSO
OT-55 (200 μM)
Mitoxantrone (3 μM)
Cisplatin (300 μM)
SSC
CRT
D
PBS
Mitoxantrone
OT-55
Cisplatin
Tumor volume [mm³]
Times (Days)
E
ROS generation (Geometric mean)
0
OT-55
OT-55+NAC
H2O2
F
Calreticulin expression High Low
TARGET-AML
TCGA-LAML
BEAT-AML
HR: 1/1.267
P val < 0.001
HR: 1/1.6167
P val = 0.02
HR: 1/1.493
P val < 0.001
Survival Probability
Time (Days)

Supplemental Figure 3. Effect of OT-55 on murine AML cells and induction of progressive oxidative stress independently of cell death rescue by antioxidant treatment. (A-B) C1498 cells were treated with increasing concentrations of OT-55. Viability and proliferation were determined after 24, 48, and 72 h. **(C)** CRT expression was analyzed by flow cytometry based on dot plot data. **(D)** Each mouse showed tumor volumes calculated using the formula L × W × W × 0.52 mm^3, and these values are presented as means ± SD. **(E)** DCFH-DA geometric mean fluorescence intensity measured at 0, 1, 3, 6, 12, and 24h in C1498 cells treated with vehicle (0), OT-55 (200 µM), OT-55 + NAC (10 mM), or H_2O_2 (200 µM) as a positive control. OT-55 induced a time-dependent increase in intracellular ROS, which was partially suppressed by NAC. Data analysis was done using one-way ANOVA using Tukey's test. $^*P < 0.05$, $^{**}P < 0.01$, and $^{****}P < 0.0001$. **(F)** Kaplan-Meier survival analysis based on high vs. low expression of the CALR gene in the TARGET-AML, TCGA-LAML, and BEAT-AML cohorts.

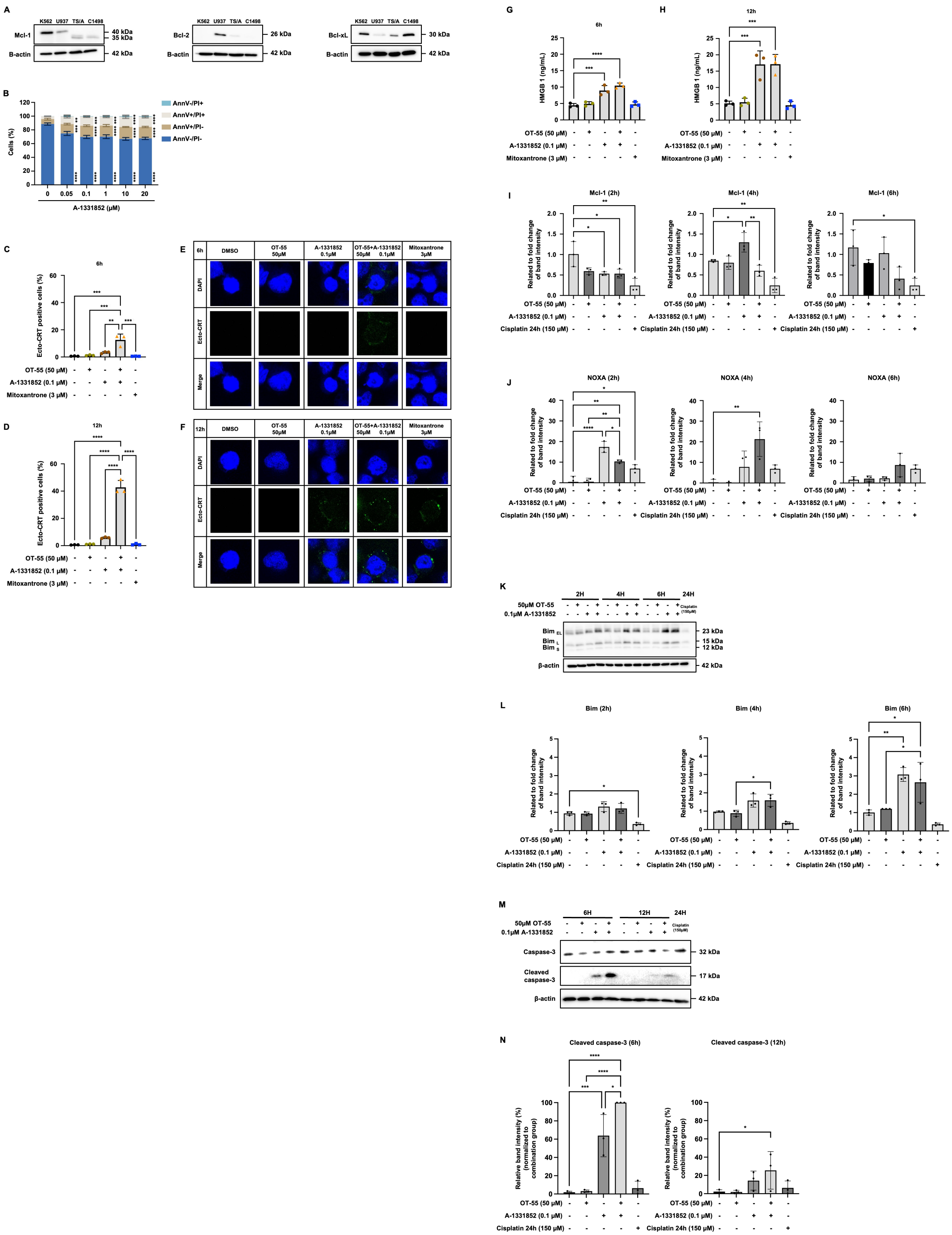
A
K562 U937 TS/A C1498
Mcl-1
B-actin
40 kDa
35 kDa
42 kDa
Bcl-2
26 kDa
Bcl-xL
30 kDa
B
AnnV-/PI+
AnnV+/PI+
AnnV+/PI-
AnnV-/PI-
Cells (%)
A-1331852 (μM)
C
6h
Ecto-CRT positive cells (%)
OT-55 (50 μM)
A-1331852 (0.1 μM)
Mitoxantrone (3 μM)
D
12h
E
DMSO
OT-55 50μM
A-1331852 0.1μM
OT-55+A-1331852 50μM 0.1μM
Mitoxantrone 3μM
DAPI
Ecto-CRT
Merge
F
G
HMGB 1 (ng/mL)
H
I
Mcl-1 (2h)
Mcl-1 (4h)
Mcl-1 (6h)
Related to fold change of band intensity
Cisplatin 24h (150 μM)
J
NOXA (2h)
NOXA (4h)
NOXA (6h)
K
2H
4H
6H
24H
50μM OT-55
0.1μM A-1331852
Bim EL
Bim L
Bim S
β-actin
23 kDa
15 kDa
12 kDa
L
Bim (2h)
Bim (4h)
Bim (6h)
M
12H
Caspase-3
Cleaved caspase-3
32 kDa
17 kDa
N
Cleaved caspase-3 (6h)
Cleaved caspase-3 (12h)
Relative band intensity (%) (normalized to combination group)

Supplemental Figure 4. OT-55 was sensitized by the Bcl-xL inhibitor, and the combination treatment of OT-55 and A-1331852 induced apoptotic immunogenic cell death; combination treatment induces Bim upregulation and caspase-3 cleavage, establishing the mechanistic link between Bcl-xL phosphorylation, Mcl-1 downregulation, and apoptotic execution. (A) Expression of basal level of anti-apoptotic proteins in human and mouse, hematological and solid cancer cell types. **(B)** After 24 hours of A-1331852 treatment with different doses, apoptotic and necrotic cell death was assessed by Annexin V/PI staining using FACS analysis. **(C-D)** C1498 cells were treated with 50 μM of OT-55 and 0.1 μM of A-1331852 for 6 and 12 hours, alone or in combination, and mitoxantrone was used as a *bona fide* ICD inducer. Evaluation of ectopic calreticulin exposure by flow cytometry and **(E-F)** observed by confocal microscopy. **(G-H)** Release of HMGB1 after 6 and 12 hours of treatment with 50 μM of OT-55 and 0.1 μM of A-1331852 in single and combination treatments, and 3 μM of mitoxantrone, assessed by ELISA in C1498 cells supernatants. **(I-J)** Western Blots for apoptosis-related protein expression in C1498 cells. After 2, 4, and 6 hours of treatment with 50 μM of OT-55 and 0.1 μM of A-1331852 in single and combination treatments, and 150 μM of cisplatin for 24 hours as a positive control. Band intensity was quantified using ImageJ. **(K-L)** Western blot analysis of Bim protein expression (Bim_{EL} ~23 kDa; Bim_{L} ~15 kDa) in C1498 cells treated with OT-55 (50 μM), A-1331852 (0.1 μM), or their combination at 2, 4, and 6h. Cisplatin (150 μM, 24h) served as a positive control. β-actin (42 kDa) was used as a loading control. Quantification shows fold change in band intensity relative to vehicle control (mean ± SD, n = 3 independent experiments). **(M-N)** Western blot analysis of caspase-3 processing showing pro-caspase-3 (32 kDa) and cleaved caspase-3 (17 kDa) in C1498 cells treated with OT-55 (50 μM), A-1331852 (0.1 μM), or their combination at 6, 12, and 24h. Cisplatin (150 μM, 24h) served as a positive control. β-actin (42 kDa) was used as a loading control. Quantification of cleaved caspase-3 band intensity percentage is shown at 6h and 12h (mean ± SD, n = 3 independent experiments). All data analysis was done using one-way ANOVA using Tukey's test. $^*P < 0.05$, $^{**}P < 0.01$, $^{***}P < 0.001$, and $^{****}P < 0.0001$.

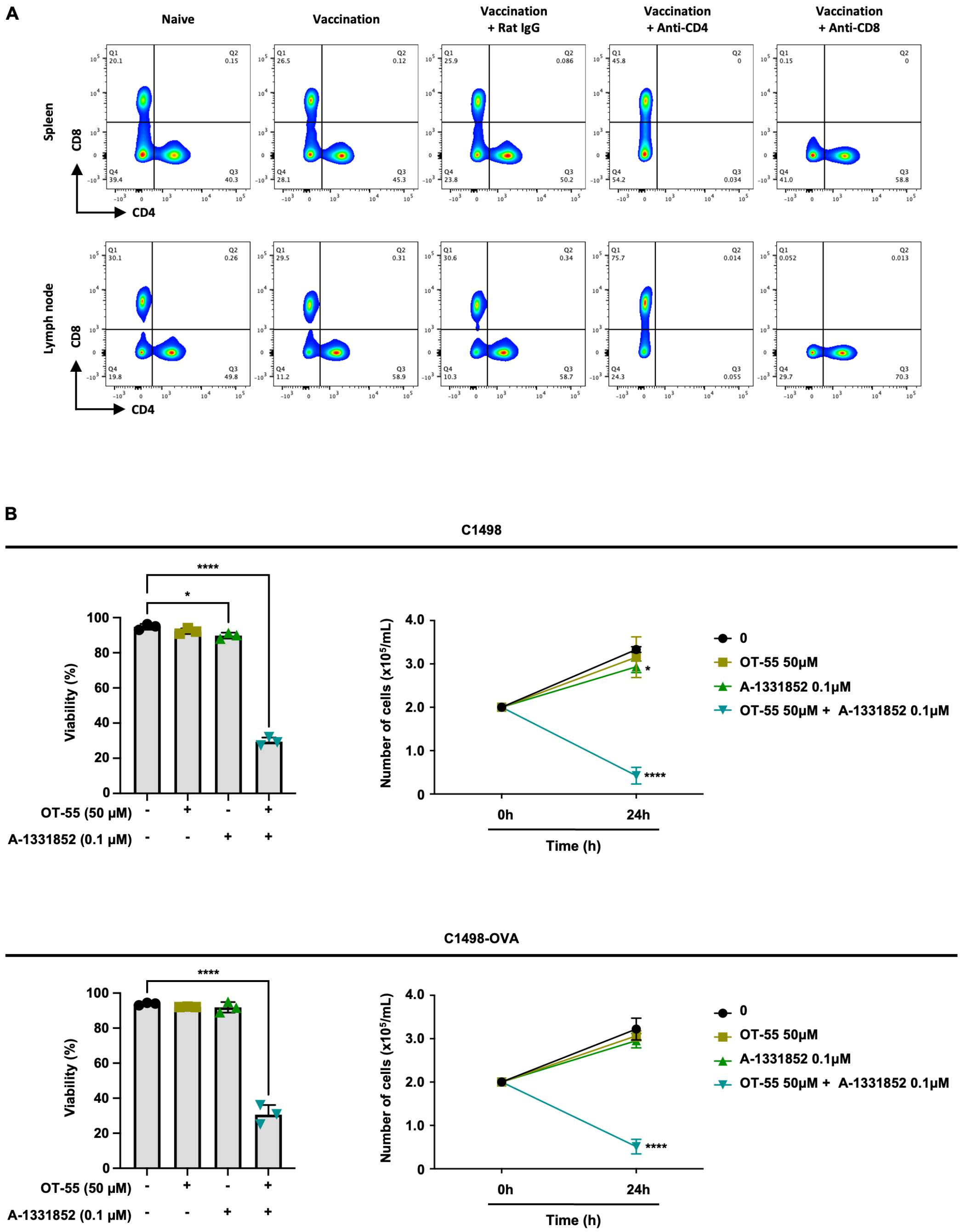
A
Naive
Vaccination
Vaccination + Rat IgG
Vaccination + Anti-CD4
Vaccination + Anti-CD8
Spleen
Lymph node
CD8
CD4
B
C1498
C1498-OVA
Viability (%)
OT-55 (50 μM)
A-1331852 (0.1 μM)
Number of cells (x10^5/mL)
Time (h)
0h
24h
0
OT-55 50μM
A-1331852 0.1μM
OT-55 50μM + A-1331852 0.1μM

Supplemental Figure 5. Validation of $CD4^+$ and $CD8^+$ T cells depletion *in vivo* and *in vitro* assessment of C1498 and OVA-C1498 cells viability and proliferation after OT-55 and A-1331852 treatment. (A) Flow cytometry analysis of spleen and lymph node T cell subsets confirming antibody-mediated depletion efficiency in vaccinated mice. **(B)** Viability and proliferation of C1498 and OVA-C1498 cells after treatment with 50 µM of OT-55 and 0.1 µM of A-1331852 for 24 hours. Data analysis was done using one-way ANOVA using Tukey's test. **P < 0.05,* and *****P < 0.0001*.

A

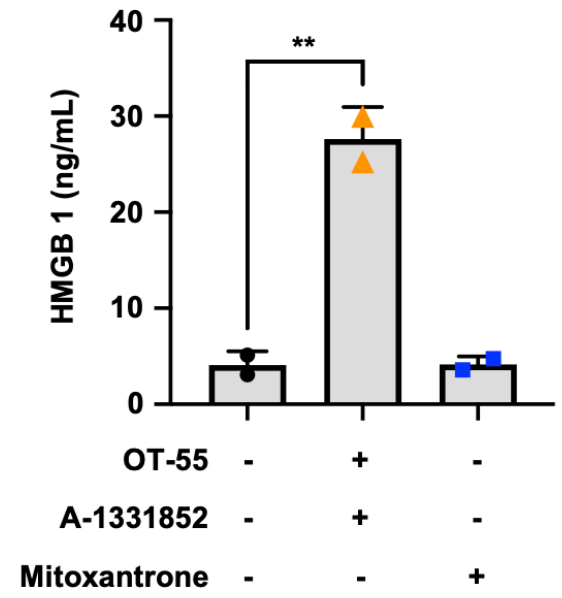

B

	PBS	OT-55+A-1331852 treated cells	OT-55+A-1331852 treated cells +Anti-PD-1	OT-55+A-1331852 treated cells +Anti-Tim-3	OT-55+A-1331852 treated cells +Anti-PD-1+Anti-Tim-3
**Slope**	0.0963	0.07885	0.062	0.06054	0.05037

Supplemental Figure 6. OT-55- and A-1331852-treated C1498 cells released HMGB1 prior to injection. (A) Release of HMGB1 after 24 hours of treatment with 50 µM of OT-55 and 0.1 µM of A-1331852 in single and combination treatments, and 3 µM of mitoxantrone, assessed by ELISA in C1498 cells supernatants. These cells are prepared for vaccination injection. Data analysis was done using one-way ANOVA using Tukey's test. $^{**}P < 0.01$. **(B)** Slopes of tumor growth curves from mice vaccinated with OT-55- and A-1331852-treated C1498 cells, and injected with anti-PD-1, anti-Tim-3, or both. Slope values were calculated by linear regression of individual tumor growth curves. Statistical significance was determined by comparison to the PBS control group. Statistical comparisons indicated $^{*}P < 0.05$, and $^{****}P < 0.0001$.

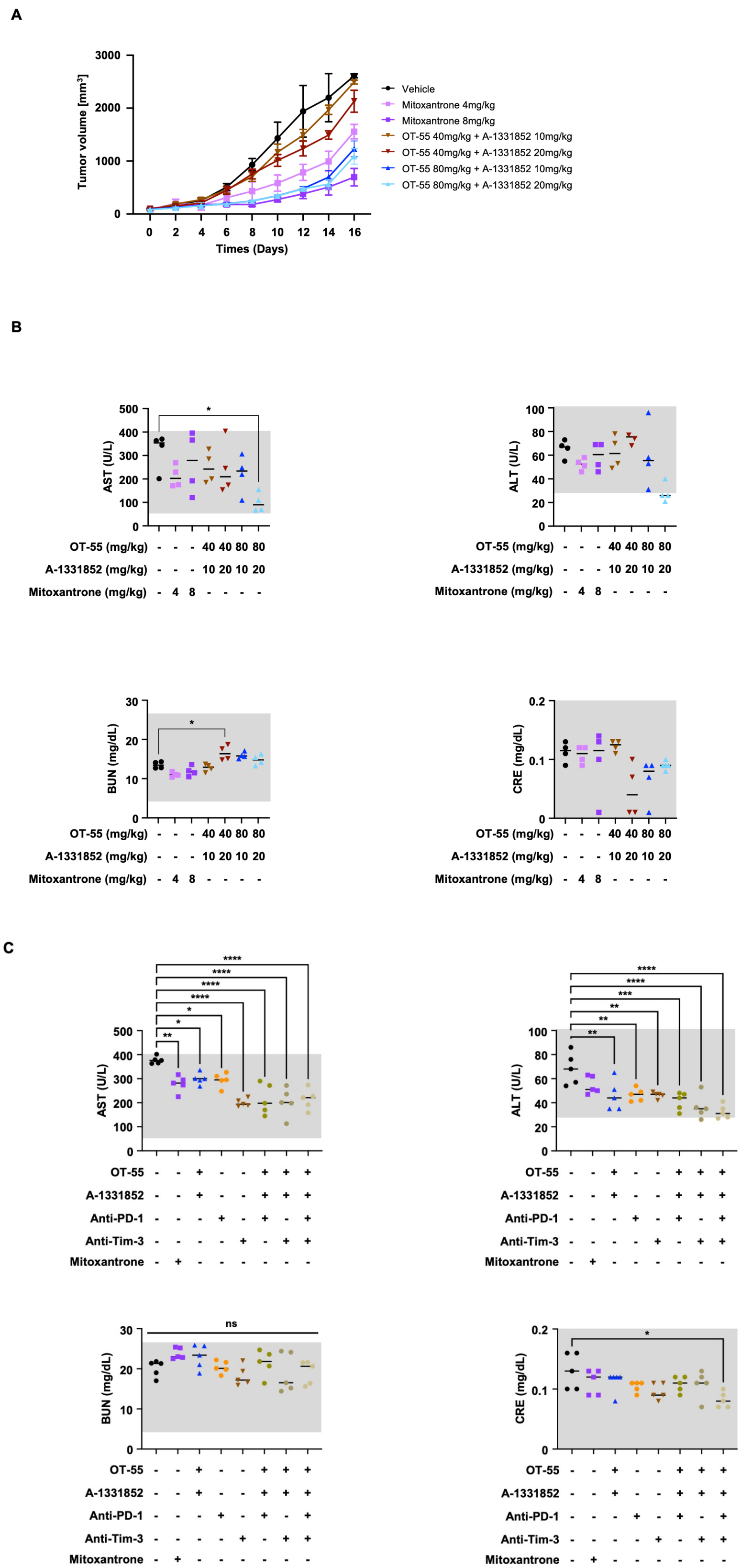
A
Tumor volume [mm³]
Times (Days)
Vehicle
Mitoxantrone 4mg/kg
Mitoxantrone 8mg/kg
OT-55 40mg/kg + A-1331852 10mg/kg
OT-55 40mg/kg + A-1331852 20mg/kg
OT-55 80mg/kg + A-1331852 10mg/kg
OT-55 80mg/kg + A-1331852 20mg/kg
B
AST (U/L)
ALT (U/L)
BUN (mg/dL)
CRE (mg/dL)
OT-55 (mg/kg)
A-1331852 (mg/kg)
Mitoxantrone (mg/kg)
C
AST (U/L)
ALT (U/L)
BUN (mg/dL)
CRE (mg/dL)
OT-55
A-1331852
Anti-PD-1
Anti-Tim-3
Mitoxantrone
ns

Supplemental Figure 7. Evaluation of hepatic and renal toxicity following treatments with OT-55, A-1331852, mitoxantrone, and immune checkpoint inhibitors. (A) Kinetic analysis of tumor volume in syngeneic immunocompetent C57BL/6 mice. C57BL/6 mice bearing established C1498 tumors were treated intratumorally with vehicle, mitoxantrone (4 or 8 mg/kg), or combinations of OT-55 (40 or 80 mg/kg) and A-1331852 (10 or 20 mg/kg). **(B)** Serum levels of aspartate aminotransferase (AST), alanine aminotransferase (ALT), blood urea nitrogen (BUN), and creatinine (CRE) were measured. **(C)** Serum levels of AST, ALT, BUN, and CRE were measured. Mice were treated with OT-55 (80 mg/kg), A-1331852 (10 mg/kg), anti-PD-1, anti-Tim-3, or mitoxantrone (8 mg/kg), alone or in combination, as indicated. The gray areas indicate the normal age range for 8- to 10-week-old mice. Data analysis was done using one-way ANOVA using Tukey's test. *$^{*}P < 0.05$, $^{**}P < 0.01$, $^{***}P < 0.001$,* and *$^{****}P < 0.0001$.*

References

1. Beltra JC, Manne S, Abdel-Hakeem MS, Kurachi M, Giles JR, Chen Z*, et al.* Developmental Relationships of Four Exhausted CD8(+) T Cell Subsets Reveals Underlying Transcriptional and Epigenetic Landscape Control Mechanisms. *Immunity* 2020, **52**(5)**:** 825–841 e828.

2. Parry EM, Lemvigh CK, Deng S, Dangle N, Ruthen N, Knisbacher BA*, et al.* ZNF683 marks a CD8(+) T cell population associated with anti-tumor immunity following anti-PD-1 therapy for Richter syndrome. *Cancer Cell* 2023, **41**(10)**:** 1803–1816 e1808.

Supplemental Tables

OT-55 reshapes tolerogenic BH3-mimetic-induced apoptosis toward immunogenic cell death in acute myeloid leukemia, potentiating PD-1/Tim-3 blockade

Yejin Lee[1], Eun-Ji Kwon[1], Sruthi Reddy Gajulapalli[1], Ji Yeon Paik[1], Barbora Orlikova-Boyer[2,&], Artur M.S. Silva[3], Hyuk-Jin Cha[1], Claudia Cerella[2,&], and Marc Diederich[1]*

[1] Department of Pharmacy, College of Pharmacy, Seoul National University, 1 Gwanak-ro, Gwanak-gu, Seoul, 08626, Korea

[2] Laboratoire de Biologie Moléculaire du Cancer (LBMCC), BAM3 Pavillon 2, 6A rue Nicolas-Ernest Barblé, L-1210 Luxembourg, Luxembourg

[3] Department of Chemistry & REQUIMTE, University of Aveiro, 3810-193, Aveiro, Portugal

***Corresponding author:**

Marc Diederich, Research Institute of Pharmaceutical Sciences & Natural Products Research Institute, College of Pharmacy, Seoul National University, Seoul 08826, Republic of Korea. Email: marcdiederich@snu.ac.kr

Present address: [&]Department of Cancer Research, Luxembourg Institute of Health (LIH), BAM3 Pavillon 2, 6A rue Nicolas-Ernest Barblé, L-1210 Luxembourg, Luxembourg.

Supplemental Table 1_Complete list of statistically significant DEGs (FDR < 0.05, log2FC>1)

gene	baseMean	log2FoldChange	lfcSE	stat	pvalue	FDR
TLR10	78.65953789	-0.1431672	0.073338513	-1.952142118	0.050921334	0.066766028
IHO1	91.33446222	-0.133884929	0.068553807	-1.952990436	0.050820728	0.066638582
CLU	1807.79493	0.210408924	0.107708532	1.953502855	0.050760039	0.066563463
HORMAD1	49.42729203	0.178928632	0.091565931	1.95409613	0.05068985	0.066475875
KATNAL1	653.6552319	-0.07119378	0.03643091	-1.954213623	0.050675959	0.066462111
ALDH1A2	36.32632202	0.167204633	0.085500837	1.955590605	0.050513401	0.066253353
TBC1D8	936.7337033	0.121838161	0.062301819	1.955611617	0.050510923	0.066253353
DKK4	9.644819169	-0.135315584	0.069192187	-1.955648335	0.050506595	0.066253306
FAM217B	455.7137905	-0.078305566	0.040025284	-1.956402512	0.050417756	0.066141203
NELFA	1116.001816	0.053010625	0.027094101	1.956537476	0.050401872	0.066124797
PIGT	3797.098515	0.080361907	0.041056889	1.957330634	0.050308608	0.066006863
RPL7	27876.70676	0.106856568	0.054582204	1.957718107	0.050263099	0.065951575
AGT	34.52436452	0.290074527	0.148135503	1.958170194	0.050210045	0.065886378
ZNF626	54.54092769	-0.178530493	0.091172073	-1.95817082	0.050209972	0.065886378
AKT1	3560.806513	-0.060482701	0.030884828	-1.958330514	0.050191243	0.065870537
E2F6	236.0562263	0.072876685	0.037208514	1.958602397	0.050159369	0.06583312
AL133500.1	3.376037865	-0.14651945	0.074793658	-1.958982271	0.050114863	0.065779118
MTHFR	2385.682217	0.083660664	0.042700831	1.959228028	0.050086088	0.065745758
IQCF2	0.31766399	-0.42650332	0.217618574	-1.959866346	0.050011414	0.06565214
NDUFA9	311.499092	0.064205603	0.032749474	1.960507936	0.049936451	0.06555813
KLK1	17.22468131	-0.228118577	0.116351486	-1.960598738	0.04992585	0.065548609
TBC1D4	285.6697517	-0.156507742	0.079825516	-1.960622995	0.049923018	0.065548609
ZBTB38	513.0102225	0.124293339	0.063394717	1.960626145	0.04992265	0.065548609
PDCD10	1021.392312	0.067612403	0.034481571	1.960827258	0.049899177	0.065526776
H1-7	7.570882711	-0.1527786	0.077910719	-1.960944563	0.04988549	0.065513198
TBC1D3D	2.521099677	0.357743169	0.18242916	1.960997737	0.049879287	0.065509447
SLC25A30	670.6880687	0.078994876	0.040268415	1.96170807	0.049796482	0.065405082
RALY	5822.897087	0.050066452	0.025499965	1.963392967	0.04960053	0.065152082
MFSD6	1219.825701	0.073042475	0.037191696	1.963945779	0.04953638	0.065072186
PRSS41	2.887463569	-0.203157907	0.103427493	-1.964254382	0.049500599	0.065029547
AL772284.1	0.661275138	-0.386490563	0.196741524	-1.96445852	0.049476942	0.065002832
MED28	3702.332341	0.054515594	0.027730124	1.965934025	0.049306232	0.064782901
TBX18	11.52887941	-0.346948766	0.176324092	-1.967676467	0.049105275	0.064523198
H3C14	18.62740987	0.177788536	0.090347746	1.967824805	0.049088199	0.06450509

MUC13	5.188005024	-0.251418313	0.127726255	-1.968415282	0.049020275	0.064420159
FHAD1	23.84384576	0.148551849	0.075464241	1.968506485	0.049009791	0.064410705
CPEB4	4528.864381	0.120240521	0.061020683	1.970487963	0.048782473	0.06411626
RAB19	48.86705635	-0.189483769	0.096155759	-1.970592002	0.048770562	0.06410491
PGPEP1L	10.3171377	-0.258901099	0.131322011	-1.971498127	0.048666927	0.063972986
SYS1-DBNDD2	3.371834475	0.172424736	0.087454857	1.971585591	0.048656933	0.063964145
CLTB	974.0658022	0.078016607	0.039562579	1.9719798	0.048611912	0.063909253
MLXIPL	47.04998437	0.177172179	0.089826734	1.972376938	0.048566593	0.063853961
CKMT1A	3.846574316	0.227667099	0.11540718	1.972729074	0.048526438	0.063805452
TAPBP	11816.08336	0.075317037	0.038169273	1.973237385	0.048468523	0.063733584
MAGOH	573.135164	0.08747463	0.044329496	1.973282746	0.048463358	0.063731073
FUT9	0.535667713	-0.31686577	0.160514783	-1.974059733	0.048374951	0.06361909
TXNDC8	3.170400133	0.236170483	0.119617984	1.974372712	0.048339378	0.063576579
SELENOO	1382.822544	-0.06820984	0.034537041	-1.974976363	0.04827083	0.06349069
UBE2U	0.363660966	-0.52557981	0.266079093	-1.975276615	0.048236766	0.063450149
CNPY3	3506.893596	-0.08836648	0.044735374	-1.97531557	0.048232347	0.063448601
OR52B4	0.958633856	-0.242531303	0.122780887	-1.975318059	0.048232065	0.063448601
KLHL14	36.29550638	0.194903868	0.098658431	1.975541949	0.048206679	0.06342336
PTPN18	2334.91665	-0.099714103	0.05046398	-1.975946089	0.048160883	0.063367368
HNRNPC	16884.08909	0.048141011	0.024341588	1.977726793	0.047959536	0.063106689
PDLIM2	799.2536365	-0.083327014	0.042131384	-1.977789633	0.047952444	0.063101599
GIT1	1998.248045	-0.075584887	0.038200171	-1.978653113	0.047855075	0.062977704
ACTR1A	3068.299986	0.047658815	0.024072757	1.979782189	0.047728009	0.062814706
Z82206.1	43.28107611	-0.160077776	0.080852138	-1.979883031	0.047716674	0.062804011
DISC1	728.1377814	-0.110540668	0.055789157	-1.981400568	0.04754637	0.062584069
TRPV4	17.01909556	-0.203149647	0.102495849	-1.982028053	0.047476101	0.062495778
FAM193A	2884.31224	-0.053608842	0.027046306	-1.982113294	0.047466562	0.062487424
KRT24	0.471945247	-0.433422225	0.218628308	-1.982461601	0.047427601	0.062440333
FOXI2	0.695696409	-0.278384478	0.140402618	-1.982758456	0.047394417	0.062400842
SYNJ2	908.7905267	0.119095114	0.060064095	1.982800444	0.047389724	0.062398861
PPID	372.5403542	-0.063762947	0.032142365	-1.983766479	0.047281879	0.062261048
DIRAS3	5.334487674	0.341490935	0.172084376	1.98443893	0.04720693	0.062166537
ANKRD42	444.0911814	0.074499955	0.037531015	1.985023694	0.047141835	0.062084992
OR2Y1	0.298969163	-0.374827266	0.188808752	-1.985221885	0.04711979	0.062060134
AFM	0.510635158	-0.475833578	0.239681497	-1.985274557	0.047113933	0.062056596
POTEM	2.203058731	-0.194308617	0.097826984	-1.986247662	0.047005831	0.061918375
SPDEF	13.31339571	-0.16955435	0.085347157	-1.9866432	0.04696195	0.061864736

PTK2B	11211.72335	-0.069375776	0.034875954	-1.989215128	0.046677463	0.061494109
GSTM1	196.8639678	-0.349371993	0.175632846	-1.989217849	0.046677163	0.061494109
ARHGAP12	866.4635767	0.104403365	0.052469706	1.989783696	0.046614768	0.061419782
PTPN14	901.6582137	0.201649673	0.101311395	1.990394792	0.046547463	0.061335229
AGAP9	349.0130604	-0.107973761	0.054241535	-1.990610347	0.046523742	0.061308099
ACOT1	29.56434997	-0.107948445	0.054212818	-1.991197805	0.046459145	0.061227097
CNN1	10.24001692	-0.16057976	0.080629162	-1.991584143	0.046416705	0.061175285
TEX46	2.757978186	-0.226461001	0.113701211	-1.991720219	0.046401764	0.061159712
ALX4	52.40238518	-0.155516727	0.078075385	-1.991879103	0.046384324	0.061140843
TNNC2	27.29803999	-0.109413867	0.054928483	-1.991933167	0.046378391	0.06113714
SLC41A1	425.1092037	0.131339631	0.065935437	1.991942984	0.046377314	0.06113714
SLC7A8	93.07923773	0.162000479	0.081310346	1.992372272	0.046330227	0.061081877
SLC25A51	533.2784923	0.067582815	0.033920114	1.992411193	0.04632596	0.061080365
ASH1L	6728.873255	-0.066935686	0.033586099	-1.992958036	0.046266042	0.061005474
EIF3D	8340.164169	0.063023058	0.031621058	1.99307236	0.046253524	0.060993076
FBXO25	417.5546567	-0.079209732	0.039723904	-1.994006721	0.04615132	0.060862403
ABCC2	190.5254025	-0.106927126	0.053616105	-1.994309852	0.046118203	0.060822828
TMEM181	1309.078257	0.068484423	0.034318479	1.995555329	0.045982346	0.06064774
DNAJB1	5879.404263	0.126998532	0.063632182	1.995822356	0.045953262	0.060613465
UBE2F-SCLY	9.027253501	-0.133230561	0.066747445	-1.996039849	0.045929586	0.060586317
CYP19A1	11.35832995	-0.20640947	0.10335203	-1.997149646	0.045808929	0.060431231
OR52I1	6.325991631	-0.151015311	0.075613681	-1.997195608	0.045803938	0.060428719
U2AF1	935.8971242	0.144448296	0.072306639	1.997718299	0.045747209	0.060357946
DPF2	1862.758629	0.048685399	0.024369537	1.997797498	0.045738619	0.06035068
WNT8A	3.894812191	0.248971104	0.124622619	1.99780029	0.045738316	0.06035068
RALGAPA1	1496.725624	0.067146499	0.033606813	1.998002593	0.045716379	0.060329469
FAM193B	2725.491218	-0.064775651	0.032417201	-1.998187664	0.045696318	0.060307062
BCAR3	226.4428901	0.140740213	0.070425443	1.998428503	0.045670224	0.060276689
CFB	38.18145158	0.137816497	0.068949237	1.998811056	0.045628801	0.060226079
HOXA1	85.23234118	-0.201967159	0.101041652	-1.99885053	0.045624529	0.060224501
SEMA7A	805.6810141	0.13682537	0.068437772	1.999266864	0.045579487	0.060169104
FAM168A	2246.035016	-0.052951294	0.02647548	-2.000012623	0.045498901	0.060066774
RPS18	41792.05101	-0.159185934	0.079574374	-2.000467295	0.045449828	0.060006036
CCDC97	1684.559403	-0.054113364	0.027035178	-2.00159083	0.045328756	0.059850226
PRKD2	2939.702429	0.078096484	0.039014302	2.001739864	0.045312717	0.059833084
NPAS2	22.02800955	0.137184665	0.068521433	2.002069396	0.045277268	0.05979031
ANKRD50	1169.455636	-0.090361904	0.045111813	-2.003065229	0.045170288	0.059653062

TBC1D2B	3602.88161	0.085419589	0.042633215	2.003592474	0.045113733	0.059582395
HLA-DQB1	4568.092569	0.1748257	0.08725584	2.003598832	0.045113051	0.059582395
ST14	354.1489982	0.171881291	0.08577824	2.003786642	0.04509292	0.059562946
DAG1	738.0568137	-0.095432148	0.047624854	-2.003830789	0.045088189	0.059560716
TEX261	1160.943931	-0.068610132	0.034227547	-2.004529653	0.045013354	0.059465873
REPIN1	3396.86818	-0.079083683	0.039449274	-2.004693006	0.044995877	0.059446797
CCAR2	2681.044722	0.052566764	0.02621214	2.005435763	0.044916482	0.059345909
NT5DC3	1386.31405	0.144895207	0.072239711	2.00575564	0.044882326	0.059304784
CCAR1	2897.299022	-0.052977329	0.026410827	-2.005894347	0.044867521	0.059289225
TSSK6	83.15216338	-0.092127718	0.045920207	-2.006256594	0.044828878	0.05924216
FAM184A	20.46332434	-0.168923604	0.084182237	-2.006641892	0.044787807	0.059191881
CNOT7	2592.652836	0.051652264	0.025734417	2.007127846	0.044736052	0.059127473
TXNL1	2506.548848	0.059489894	0.029636668	2.007307081	0.044716976	0.059106251
MC4R	0.420305937	-0.555126408	0.276468592	-2.007918531	0.04465195	0.059024287
AL121758.1	2.888802612	-0.213669752	0.106311988	-2.009836859	0.044448458	0.058759265
FCHSD1	910.6646952	0.090550493	0.045038198	2.010526553	0.044375489	0.058666764
MED31	81.84025077	0.093988881	0.046738498	2.010952126	0.044330514	0.058611263
SHTN1	256.7787158	0.227289871	0.113014125	2.011163385	0.044308202	0.058585721
SLC22A24	0.494762891	-0.312793345	0.15552099	-2.011261279	0.044297866	0.058576012
CATSPER4	2.726557176	-0.213720818	0.106228298	-2.011900983	0.044230376	0.05849072
CLEC1A	8.157818252	-0.193172053	0.095994682	-2.012320356	0.044186178	0.05843622
KCNAB2	10125.13984	-0.073185805	0.036364109	-2.012583512	0.044158463	0.058403513
ADAT3	78.62061115	-0.087260134	0.043337482	-2.013502649	0.044061777	0.058279575
CFHR4	0.369168145	-0.798839451	0.396695136	-2.013736439	0.044037212	0.058251021
PPCS	602.1096303	-0.070507533	0.035012243	-2.013796528	0.0440309	0.058246608
GOSR1	1329.914526	0.052936914	0.026286742	2.013825552	0.044027852	0.058246512
RAB36	38.43770819	-0.113002784	0.056110621	-2.013928606	0.04401703	0.058236131
SELPLG	1149.881516	-0.146551517	0.072763921	-2.014068443	0.044002348	0.058220642
PPL	299.0453492	0.145134497	0.071958969	2.016906276	0.043705295	0.057831513
C15orf39	6246.963811	0.117344526	0.058139892	2.018313467	0.043558625	0.057641334
UBE2K	2024.312923	0.049065857	0.024310285	2.018316794	0.043558279	0.057641334
RCAN1	318.157273	-0.12856247	0.063689648	-2.018577175	0.043531185	0.057612813
RPS4Y2	1.575099607	-0.323642882	0.160271387	-2.01934287	0.043451595	0.057511365
OMG	304.5618677	-0.112182313	0.055477079	-2.022138045	0.043162093	0.057132051
ZNF705A	1.646525327	-0.229170598	0.113312864	-2.022458796	0.043128976	0.057092077
OTOR	1.136766878	-0.261126038	0.12911241	-2.022470482	0.04312777	0.057092077
RPL27A	26767.44042	-0.157256014	0.077751243	-2.02255306	0.043119248	0.057086921

MTRNR2L3	62.06808455	-0.112903895	0.05581721	-2.022743414	0.043099608	0.05706478
ARID1A	11940.81179	-0.050230101	0.024824764	-2.023386824	0.043033282	0.056980816
ANP32B	5861.019696	-0.110748875	0.054711163	-2.024246421	0.042944803	0.056867508
ZBTB32	55.74717052	-0.103316911	0.051026388	-2.024774151	0.04289056	0.056799522
SESN3	2823.072457	0.130926148	0.064650829	2.025127152	0.042854309	0.056755355
ZFYVE1	1423.987274	0.069034209	0.034088054	2.025173078	0.042849595	0.056752952
PIAS2	1327.539015	0.069200872	0.034144103	2.026729843	0.042690049	0.056545464
LDHC	19.76457586	-0.179827718	0.088721612	-2.026876133	0.042675082	0.056529465
ZNF43	1561.992151	-0.099791307	0.049213671	-2.02771518	0.042589325	0.056419686
PELI3	98.20923712	0.111555498	0.054995989	2.028429706	0.042516411	0.056326906
CASKIN1	38.12241215	-0.152055843	0.074958996	-2.028520262	0.042507177	0.056318485
SCT	30.16215886	-0.126913818	0.062558037	-2.028737221	0.042485063	0.056292996
BCAP31	2982.725693	-0.072367066	0.035667627	-2.028928526	0.042465571	0.056270978
KRTAP12-4	0.297280963	-0.505470009	0.249027944	-2.029772241	0.042379696	0.056160988
API5	1399.462779	0.05632719	0.027750418	2.029778072	0.042379103	0.056160988
PKNOX2	7.29243654	-0.267691652	0.131864548	-2.030050193	0.042351437	0.056131141
GCNT4	66.33095073	0.181436752	0.089365808	2.030270374	0.042329064	0.056105287
HPRT1	903.8591803	-0.078703179	0.03875434	-2.03082232	0.042273022	0.0560348
AC012309.1	7.637493739	-0.116554926	0.057390288	-2.030917258	0.042263389	0.056025825
NTF3	0.43944328	-0.407310046	0.200440976	-2.032069759	0.042146594	0.055874783
RNF133	0.252348609	-0.620880653	0.305394339	-2.03304572	0.042047904	0.055747723
RRAD	148.2114053	0.220948086	0.108677775	2.033056765	0.042046788	0.055747723
MAGEB16	0.246748112	-0.679214175	0.334004359	-2.033548834	0.041997105	0.055687917
IFIT1	349.2422109	0.200270697	0.098481104	2.033595168	0.041992429	0.055685489
HDX	50.32051192	0.223666309	0.109963039	2.03401354	0.04195023	0.055633299
MRPS36	354.3201484	-0.115863102	0.056941782	-2.034764255	0.041874599	0.055536762
SPACA3	0.47459283	-0.404510328	0.198760085	-2.035168817	0.04183389	0.05548653
MCU	895.5583067	-0.100657993	0.049459172	-2.03517343	0.041833425	0.05548653
SLAMF8	4.06480157	0.218567049	0.107373408	2.035578951	0.041792653	0.055439349
FLVCR1	617.60907	-0.071129094	0.034926086	-2.036560695	0.041694085	0.055312344
GPR89A	243.3688734	-0.066727302	0.032752873	-2.037296123	0.041620376	0.055218303
STX7	1816.81575	-0.083204473	0.040837749	-2.037440239	0.041605945	0.055202899
RAD54L2	3130.194297	-0.064108942	0.031463784	-2.037547122	0.041595245	0.055192443
ZNF704	466.2670371	0.204540683	0.100345085	2.038372713	0.041512673	0.055086613
EVPLL	0.709033908	-0.29745276	0.145882304	-2.038991375	0.041450888	0.055008356
EIF4G1	9805.032666	-0.049503735	0.024277089	-2.039113307	0.04143872	0.054995937
LYNX1-SLURP2	0.828072727	-0.348912987	0.171044847	-2.039891835	0.0413611	0.054896644

CIRBP	5928.931112	0.076430347	0.03746681	2.039948085	0.041355497	0.054892929
LOXL4	210.9035975	0.147822695	0.072452935	2.040258196	0.041324616	0.05485566
JADE1	1717.491015	0.05488172	0.02689451	2.040629144	0.041287703	0.054810378
UTP4	1293.675738	-0.053855885	0.026390688	-2.040715421	0.041279122	0.054802703
GAPVD1	6165.119976	0.084149746	0.041222145	2.04137234	0.041213832	0.054719735
NCMAP	5.995988862	-0.17529081	0.085868776	-2.041380104	0.04121306	0.054719735
SOCS1	372.5827516	0.156264329	0.076547826	2.04139474	0.041211607	0.054719735
VPS35	3322.337618	-0.055136526	0.026998339	-2.042219211	0.041129792	0.054619269
MTRNR2L5	0.930971611	-0.404916126	0.198253105	-2.042420092	0.041109878	0.054596529
GOLGA2	1859.287636	0.063492333	0.031075436	2.043167861	0.041035824	0.054501879
RNF19A	4417.838535	0.07121895	0.03483586	2.044414858	0.040912581	0.05434188
TNPO1	5228.685997	0.081743022	0.039973969	2.044906327	0.040864094	0.054281161
OR9G1	0.299961796	-0.658064009	0.321764138	-2.04517512	0.040837596	0.054249645
RAET1E	4.505497634	-0.194495865	0.095091301	-2.045359186	0.04081946	0.054229233
POLR2J2	85.18028861	-0.096813997	0.047328439	-2.04557766	0.040797941	0.054204324
TLE2	37.82565111	0.141798056	0.069317054	2.04564459	0.040791351	0.054199247
TLDC2	45.80607331	-0.151220554	0.073888004	-2.046618482	0.040695559	0.05407564
BATF	156.9115475	-0.127789327	0.062422563	-2.047165643	0.040641824	0.054007904
CHEK1	735.0617961	-0.062725289	0.030638547	-2.047267083	0.040631868	0.05399834
RDX	1434.996356	0.080669324	0.03939127	2.047898518	0.040569944	0.053919706
STOX2	161.3692633	-0.24611566	0.120178187	-2.047922894	0.040567555	0.053919706
FEZF2	0.323215117	-0.625572517	0.305342126	-2.048759292	0.040485658	0.053814994
MBOAT4	34.36578422	0.107620671	0.052521691	2.049070935	0.040455179	0.053778132
RPL18	26569.52447	-0.110573248	0.05396163	-2.049108751	0.040451482	0.05377687
RPTN	0.609130595	-0.360859112	0.176085524	-2.049340019	0.040428878	0.053750471
SIAH2	1819.404385	-0.104108919	0.050785733	-2.049963889	0.040367955	0.053673119
ZNF436	320.4206651	0.075978644	0.037051992	2.050595416	0.040306363	0.053594867
ZNF552	634.0665798	-0.088407157	0.043102592	-2.05108677	0.040258497	0.053534858
RETREG2	3188.749328	-0.061429242	0.029948528	-2.051160639	0.040251306	0.053528931
SYNJ2BP-COX16	0.270916644	-0.312953482	0.152548222	-2.051505276	0.040217766	0.053487962
HERC5	828.7498328	0.135176181	0.065867269	2.052251186	0.040145257	0.053395156
OR6B2	0.586771406	-0.328293569	0.159965496	-2.052277383	0.040142713	0.053395156
MAB21L4	10.12360451	-0.364383983	0.177372855	-2.054339056	0.039942887	0.053133215
SLC1A3	254.2527712	0.147624675	0.071856176	2.05444658	0.039932489	0.053122993
LIN52	358.7748158	-0.067647169	0.032910641	-2.055480133	0.039832653	0.052993781
SYNPO	91.88978976	-0.127658044	0.062086754	-2.056123674	0.039770597	0.052914818
KIF25	1.666321521	-0.350459014	0.170394183	-2.056754559	0.039709841	0.052837574

L3MBTL4	103.7410448	-0.229539051	0.111595627	-2.056882128	0.039697566	0.052824832
HSDL1	348.0732037	0.079645011	0.038708457	2.057561003	0.039632293	0.052741561
FARP2	1035.147526	0.085052862	0.041332102	2.057791824	0.039610121	0.052715639
PATZ1	1644.459101	-0.059169205	0.028745998	-2.058345824	0.039556948	0.052648453
ASMT	21.61070781	-0.16724547	0.081252064	-2.058353487	0.039556213	0.052648453
SEMA4C	560.1738024	-0.127629902	0.061958401	-2.059928912	0.039405337	0.052453799
CD69	6032.138088	0.140926015	0.068411536	2.059974433	0.039400985	0.052451573
MED9	588.038124	0.07372273	0.03573932	2.062790523	0.039132535	0.052097749
PFDN2	760.6156069	-0.134804866	0.065330803	-2.063419696	0.03907277	0.052021722
ZNRF4	0.981441512	-0.281651972	0.136486518	-2.063588237	0.039056774	0.052003962
CMKLR1	58.87568095	-0.191751619	0.092867959	-2.064776921	0.038944113	0.051857481
LMBR1	1727.273215	0.074693984	0.036166137	2.065301697	0.038894463	0.051794892
ATN1	2458.951992	0.120045838	0.058118422	2.065538507	0.038872076	0.051768602
NKX2-2	0.341998003	-0.329230457	0.159379943	-2.06569566	0.038857225	0.051752346
ONECUT2	29.71802523	-0.266382267	0.128946129	-2.065841515	0.038843447	0.051737515
KRT18	424.5308235	0.259372526	0.125482803	2.066996592	0.038734475	0.051595881
RAB2B	1208.244686	0.071113157	0.034398337	2.06734287	0.038701857	0.051555942
S100A6	5788.490107	0.171445829	0.082929935	2.067357571	0.038700473	0.051555942
FGF4	0.477716657	-0.310970122	0.150418267	-2.067369398	0.03869936	0.051555942
NACC1	1605.506326	0.077937121	0.037679486	2.06842315	0.03860025	0.051431089
CEP135	1046.111043	0.069178236	0.033431379	2.06926062	0.038521637	0.051329839
CLIP3	398.2679096	-0.228027613	0.110056666	-2.071910973	0.038273744	0.051002996
PSMB4	4092.269432	-0.070062985	0.033813765	-2.072025557	0.038263058	0.050992227
VCX	0.988385345	-0.395826461	0.191010203	-2.072279154	0.038239415	0.050964189
DUSP22	1303.664712	-0.072443793	0.034928175	-2.074078988	0.038071976	0.050744486
METAP1	1256.160528	-0.050960371	0.024568007	-2.07425743	0.038055409	0.05072586
MISP3	33.23878321	-0.145590225	0.070151007	-2.075383258	0.037951029	0.050590171
OR2T34	0.299545552	0.380940786	0.183546831	2.075441912	0.037945597	0.050586376
OR2T1	0.278348918	0.61791428	0.297632026	2.076101445	0.03788457	0.050508459
GTF2IRD1	141.9779598	-0.153438114	0.073892819	-2.076495608	0.037848137	0.050463323
SH2D4A	30.80685512	-0.190558261	0.091684591	-2.07841097	0.037671523	0.050231264
CCT6B	157.7349263	0.084901697	0.040832235	2.079281133	0.037591518	0.050128
RPL13A	51721.85671	-0.113933864	0.054789306	-2.079490916	0.037572252	0.050105722
DCAF12L2	1.440587218	-0.538538743	0.258961597	-2.079608518	0.037561455	0.050094737
MAP1LC3B2	96.16217422	0.110376423	0.053075136	2.079625826	0.037559866	0.050094737
COX4I1	9899.394174	0.11764814	0.056555374	2.080229191	0.037504516	0.050025616
RNF24	5819.094437	-0.066007625	0.031716428	-2.08118092	0.037417349	0.04991275

RAB29	462.2120504	0.105650937	0.050743109	2.082074561	0.03733566	0.049807175
CELSR1	360.8860683	0.168908884	0.081082597	2.08317062	0.037235674	0.049677176
MMP25	500.7088981	0.189577055	0.090993296	2.083417812	0.037213156	0.049650518
GRHPR	2092.025024	-0.071187419	0.034167183	-2.08350272	0.037205424	0.049643586
ZYG11B	1131.086458	0.067683125	0.032481538	2.083741369	0.037183699	0.049617981
AC010547.4	0.686816405	-0.230538454	0.110580225	-2.084807249	0.0370868	0.049492053
HSF1	2909.020943	-0.068387863	0.032800608	-2.084957178	0.037073188	0.049477261
IGSF3	64.82762815	0.211573906	0.101433127	2.085846243	0.036992553	0.049373013
PDZD9	38.5961913	0.135365877	0.064895422	2.085907963	0.036986961	0.049368916
PDE8B	22.40660387	-0.100826683	0.048336601	-2.085928263	0.036985122	0.049368916
HCLS1	4291.229935	-0.085956945	0.041200339	-2.086316428	0.03694997	0.049326269
TAS2R38	2.218016479	-0.278669608	0.133567638	-2.086355741	0.036946411	0.049324882
LPL	119.1366047	-0.243147178	0.116466989	-2.087691796	0.036825645	0.049167009
JUN	24781.04313	0.131817703	0.063128292	2.088092347	0.036789505	0.049122108
MEX3D	81.66348419	-0.127565423	0.061066898	-2.088945515	0.036712628	0.049022805
NPPA	24.60756721	0.13800786	0.066058068	2.089190058	0.036690618	0.048996757
NBPF4	2.304954732	-0.356055367	0.170351505	-2.090121635	0.036606875	0.048888261
PLPP3	93.22249059	0.177943636	0.085130652	2.090241671	0.036596096	0.048877201
RHOF	221.6198294	-0.084345116	0.040341069	-2.090800202	0.036545978	0.048813595
H1-4	10433.00985	-0.152799276	0.073051011	-2.091679136	0.036467228	0.048711735
MCMBP	1501.032865	0.062939876	0.030083759	2.092154638	0.036424685	0.048658228
EFCAB10	29.30508741	-0.106035165	0.050677339	-2.092358556	0.036406453	0.048637193
ADIPOR1	6520.955112	0.107192067	0.051220398	2.092761295	0.036370469	0.048592436
GOLGA7B	20.90742352	0.169221874	0.080856435	2.092868349	0.036360908	0.048582979
OR2T10	0.966129083	0.288665875	0.137911572	2.093122938	0.036338182	0.048555928
RPL41	12310.0123	-0.190173919	0.090833605	-2.093651572	0.03629103	0.048496233
MADD	6218.151918	-0.068494798	0.032708353	-2.094107201	0.036250432	0.048445289
FSHR	0.44707437	-0.562466757	0.268543754	-2.094506939	0.036214846	0.048401036
ERCC3	1754.880781	-0.050674239	0.024188611	-2.094962707	0.036174308	0.048350159
BEX4	669.6745215	0.138957709	0.066326308	2.095061724	0.036165506	0.048341695
MIOX	5.933292059	-0.163580691	0.077995371	-2.097312796	0.035965895	0.048078163
HAO1	0.904545019	-0.313320803	0.149341472	-2.09801603	0.035903729	0.04799834
CCDC66	646.1030154	-0.075365493	0.035916242	-2.098367979	0.035872652	0.047960069
DDX50	949.1122258	-0.045632669	0.021739643	-2.09905332	0.035812201	0.04788252
PEBP1	3236.740839	-0.097656563	0.046521714	-2.099160918	0.035802718	0.047873111
TUBB2A	407.3517848	-0.177906705	0.084731105	-2.099662283	0.03575856	0.047817332
SHE	56.99470472	-0.192045244	0.091459046	-2.099794967	0.035746881	0.047804981

PRELID1	631.6608584	-0.125359759	0.059694854	-2.100009483	0.035728007	0.047783005
CAMK1G	19.73856269	0.268070367	0.127615148	2.100615574	0.035674726	0.047715007
LILRA1	400.5151174	0.187770861	0.08938163	2.100776886	0.035660556	0.047699315
TMED9	2484.34196	0.076080633	0.0362123	2.100961092	0.035644382	0.047680938
COL6A2	258.4434457	0.186295369	0.08865341	2.101389777	0.035606765	0.047633874
INA	4.988735656	-0.225424385	0.10726188	-2.101626268	0.035586027	0.047609385
SMARCD2	2540.677621	-0.048083611	0.022876568	-2.101871682	0.035564518	0.047583861
DOK1	822.0615165	-0.086627856	0.04121226	-2.101992348	0.035553946	0.047572969
HNF1B	3.003069666	-0.204817817	0.097412654	-2.102579157	0.035502573	0.047507477
GNAI1	500.988265	0.19830242	0.094275866	2.103427194	0.035428443	0.047411522
PCSK5	120.1535337	-0.158089911	0.075149933	-2.103660028	0.035408114	0.047387556
GRAMD1A	3109.184343	-0.065831794	0.03128925	-2.103974819	0.035380644	0.04735403
MPV17L2	193.6010324	-0.119317214	0.056708663	-2.104038575	0.035375082	0.047349825
ANKRD10	5344.370838	0.056374659	0.026790458	2.104281263	0.03535392	0.047324734
OLA1	2017.892505	0.072046058	0.034231274	2.104685246	0.035318716	0.047280844
WWP1	1053.677594	0.070242758	0.033357116	2.105780297	0.035223441	0.047156526
KLHL38	0.85388199	-0.319503288	0.151713986	-2.10595803	0.035207998	0.047139076
BAG3	457.6955631	0.180494668	0.085697379	2.106186566	0.03518815	0.047115724
MYH11	1206.428831	0.09896352	0.046973421	2.106798218	0.035135074	0.047047876
FAM151B	77.73718552	0.136396754	0.064738633	2.106883434	0.035127685	0.0470412
RUNX1T1	767.510792	0.413150987	0.196095245	2.106889369	0.03512717	0.0470412
SLC49A3	190.0583376	-0.126008294	0.059765744	-2.108369871	0.034999008	0.046875297
FZD1	178.2476563	-0.155545981	0.073760427	-2.108799893	0.034961858	0.046828745
AP2A1	4113.687271	-0.073136892	0.034676759	-2.109103987	0.034935607	0.046796786
MID2	33.75263057	0.201772615	0.09562301	2.110084333	0.034851093	0.046686773
CYB5R2	73.58523489	0.226727514	0.107437979	2.110310678	0.034831604	0.04666386
DPRX	0.617095419	-0.291214142	0.137955742	-2.110924399	0.03477881	0.046596321
UTP15	518.3531793	0.061548385	0.029141815	2.11202988	0.034683886	0.046472324
WNT16	11.38077686	0.176479087	0.083507228	2.113339072	0.034571757	0.046325255
GPRIN1	39.06367539	-0.179104193	0.084739441	-2.11358713	0.034550546	0.046300003
EMILIN1	564.0309973	0.216282494	0.102323668	2.113709344	0.0345401	0.046289173
AMDHD1	19.75120124	-0.129977976	0.06147999	-2.114150911	0.03450238	0.046241789
CABP5	1.820499599	-0.301757533	0.14272274	-2.114291891	0.034490345	0.046228824
COPE	3057.928158	0.081047391	0.038328375	2.114553268	0.03446804	0.046202092
KMT2D	18862.55985	-0.080933069	0.038270956	-2.114738605	0.034452232	0.046184065
CORO1B	1708.289397	-0.073625977	0.034806796	-2.115275944	0.034406436	0.046125833
PSTPIP1	1286.398705	-0.13057458	0.061669968	-2.117312256	0.034233355	0.045896943

C7orf61	33.29068102	-0.131262201	0.061949495	-2.118858279	0.034102446	0.045724563
IL15RA	383.9481214	0.118476164	0.055901178	2.119385814	0.034057875	0.04566793
NAPA	1849.97815	0.075992826	0.03582893	2.12099062	0.033922592	0.045489647
PIP5K1A	2515.067666	0.073454813	0.034631787	2.121022893	0.033919876	0.045489122
H4C15	15.02332028	0.174389803	0.082194936	2.12166115	0.033866203	0.045420255
MAP3K7CL	148.4844826	-0.122575074	0.057760062	-2.122142369	0.033825784	0.045369155
RPS14	33854.31799	-0.140930215	0.066406748	-2.122227318	0.033818654	0.045362699
GALNT14	244.7557862	-0.189409038	0.089230335	-2.122697816	0.033779182	0.045312859
EIF1AY	528.7132246	-0.369248515	0.173944451	-2.122795602	0.033770984	0.045304966
SALL2	139.6747076	-0.194409604	0.091569648	-2.123079083	0.033747225	0.045276197
FAP	11.86356088	-0.277418175	0.13065136	-2.123347019	0.033724783	0.04524919
AKAP12	121.3283016	0.189373781	0.089180125	2.123497595	0.033712177	0.045235376
WFDC1	105.8484283	-0.188592595	0.088807655	-2.123607404	0.033702986	0.045226144
RPS23	8269.41443	-0.123511388	0.058159813	-2.123655183	0.033698988	0.04522388
ZNF354A	146.9617365	-0.088845365	0.041830293	-2.123947929	0.033674498	0.045194113
DBR1	477.1264669	0.069912528	0.03290968	2.124375821	0.03363873	0.045149206
PAFAH1B1	4483.03076	0.068635333	0.032302337	2.12477921	0.03360504	0.045107081
RASSF1	3470.202953	-0.06950822	0.032690956	-2.126221667	0.033484807	0.044948777
RAB33A	152.9314128	-0.161621076	0.076008224	-2.126363002	0.033473046	0.044936072
AL445524.2	0.831139204	-0.329217667	0.154766375	-2.127191171	0.033404202	0.044846728
CLASRP	2739.874392	-0.061526003	0.02891912	-2.127519861	0.033376913	0.044813165
CTCF	2278.021133	-0.0478893	0.022508889	-2.127572771	0.033372522	0.044810343
SESTD1	1310.992715	0.155369683	0.072998611	2.12839232	0.033304569	0.044722169
FAM156B	2.912866523	0.178436473	0.083833253	2.128468922	0.033298224	0.044716716
AL110118.1	4.542194924	0.156294828	0.073422766	2.128697076	0.033279331	0.044694411
ESX1	0.258221113	-0.489030759	0.229523632	-2.130633588	0.03311934	0.044482594
SSBP1	1938.409456	0.069676849	0.032702198	2.130647251	0.033118214	0.044482594
ZNF35	368.6989385	0.098200838	0.046082381	2.130984447	0.033090423	0.044449856
SLC43A3	1076.067599	-0.08058376	0.037811095	-2.131219923	0.033071028	0.044426852
UPF1	4593.199923	0.071084396	0.033347491	2.131626491	0.033037564	0.044384944
LRRC72	1.430607883	-0.328083398	0.153904459	-2.13173419	0.033028704	0.044376087
SEMA4A	1923.708392	0.125249028	0.058745703	2.132054279	0.033002385	0.044343768
TEX14	408.2564151	0.189242043	0.088753089	2.132230482	0.032987904	0.044327354
KLHDC2	1323.035399	0.053311159	0.024993967	2.132961121	0.032927916	0.044249783
IL17REL	39.20433102	-0.230992966	0.108286456	-2.133165812	0.032911127	0.044230258
TMTC3	199.9052049	-0.082105685	0.03847631	-2.133928263	0.032848654	0.04414933
SOX15	136.1948693	0.115926525	0.054293556	2.135180184	0.032746295	0.04401478

MYL1	0.285932306	-0.543663249	0.254588405	-2.13545958	0.032723488	0.043987146
OR4D5	0.306198654	-0.665055425	0.31130946	-2.136316145	0.032653654	0.043896289
GNPDA1	430.0071362	-0.118453128	0.055431888	-2.13691309	0.032605061	0.043833976
CDT1	1819.322958	-0.094408785	0.044158832	-2.137936648	0.032521885	0.043725159
SUDS3	2043.03669	0.06615591	0.030938326	2.138315751	0.032491125	0.043686803
CTSV	25.97954625	-0.268875309	0.125714556	-2.138776261	0.032453793	0.043639605
SUCLG2	549.9654934	0.146524967	0.068476211	2.139793745	0.032371439	0.043531857
OR10H2	0.348391774	-0.403232845	0.188405439	-2.140239938	0.032335381	0.043486355
PRRG4	543.2178461	0.127903928	0.059745532	2.140811608	0.032289233	0.043427277
PHC1	666.7172286	-0.082693977	0.038626893	-2.140839447	0.032286988	0.043427241
SPCS1	1447.691931	-0.143121156	0.066835222	-2.141403164	0.03224154	0.043369092
DAPK3	926.041263	-0.077431859	0.036148342	-2.142058388	0.032188784	0.043301104
PRAC1	0.276813471	-0.790975779	0.369254379	-2.142089095	0.032186313	0.043300757
PPIA	10551.93846	-0.100039076	0.046672186	-2.143440992	0.032077705	0.04315761
TECRL	0.350294394	-0.769150299	0.358785246	-2.14376234	0.032051934	0.043125903
KLHL20	725.5931351	0.061664246	0.028762451	2.143914848	0.03203971	0.043112419
OR5B2	1.055435471	-0.32086495	0.14950901	-2.146124502	0.031863047	0.04287765
MMP12	0.368028169	-0.549411055	0.255982122	-2.146286823	0.031850102	0.042863177
PHYHD1	81.75218396	-0.126390676	0.058884089	-2.146431723	0.031838551	0.042850578
SEC61B	1781.758056	0.117332732	0.054656907	2.146713732	0.031816079	0.042823278
RASAL2	365.4081657	-0.176033617	0.081993494	-2.146921775	0.03179951	0.04280392
UCK1	636.7157904	0.06353849	0.029594838	2.146945028	0.031797658	0.04280392
ERFL	89.94788552	0.127838982	0.059524639	2.147664984	0.031740379	0.042730204
ZNF695	43.57804272	-0.14963202	0.069665754	-2.147856175	0.031725183	0.042712684
RBIS	379.4233788	-0.098225967	0.045716137	-2.148606002	0.031665646	0.04263546
AGPAT2	1256.561697	0.097865074	0.045535557	2.149201217	0.031618453	0.042574847
CAMKK2	3421.10403	-0.078649003	0.036591635	-2.149371091	0.031604995	0.042559654
SIX4	9.162103737	-0.256344506	0.119196612	-2.150602288	0.031507605	0.042431426
ZNF587B	1271.86933	-0.088938274	0.041353198	-2.150698798	0.031499981	0.042424079
EID1	1543.629467	-0.105259729	0.048942092	-2.150699437	0.031499931	0.042424079
DPEP3	89.04748012	-0.22649788	0.105286072	-2.151261565	0.03145556	0.042370083
FOLR2	46.51942574	-0.184810923	0.085905795	-2.151320781	0.031450889	0.042366707
EIF3J	1871.356279	0.057534831	0.02673607	2.151955402	0.031400867	0.042302235
SPTLC2	6110.092658	-0.077688517	0.036093758	-2.152408644	0.031365183	0.042257072
FAM71D	22.3186529	-0.147209957	0.068356285	-2.153568699	0.031274011	0.042137139
ZNF878	114.2577908	0.124959513	0.058010729	2.154075888	0.031234221	0.042086425
UBA52	21091.38207	-0.144921561	0.06724936	-2.154987971	0.031162776	0.041993047

GPC3	15.46668345	-0.32052385	0.148735651	-2.154990068	0.031162611	0.041993047
SFTPB	33.53434711	-0.135944024	0.063077251	-2.155198919	0.031146271	0.041976587
CHD5	129.0564569	-0.195230803	0.09056617	-2.155670305	0.031109419	0.041929806
ASCL1	0.280698479	-0.448577046	0.208035831	-2.156248967	0.03106423	0.041871784
CDRT15L2	0.389453791	-0.388765734	0.18022928	-2.157062016	0.031000833	0.041789208
CASD1	683.5169881	-0.075591005	0.035035221	-2.157571812	0.030961138	0.041738574
UBE2W	1052.581964	0.081378037	0.037705842	2.158234168	0.03090963	0.041672007
OR2T6	0.383997027	-0.449772025	0.208339729	-2.158839444	0.030862625	0.041611501
AP5Z1	2331.674576	0.068676678	0.03181131	2.158876112	0.03085978	0.041610531
ADAMTS5	15.46089255	-0.286779855	0.132768144	-2.160005004	0.030772282	0.04149541
CECR2	41.39438738	-0.211692078	0.097969985	-2.160785044	0.030711948	0.041416905
DPP9	2195.974105	0.053304051	0.024661608	2.161418294	0.030663042	0.041353802
HOXC13	0.326426987	-0.331194785	0.15314408	-2.162635245	0.030569245	0.041230143
SMPD2	468.1454046	-0.065977476	0.030502375	-2.163027536	0.030539062	0.041192272
SPATA6	145.8218695	-0.183917242	0.085025885	-2.163073549	0.030535523	0.041190337
CTNNA1	3664.244896	0.091972486	0.04251925	2.16307872	0.030535125	0.041190337
NAA20	596.1885537	-0.093561439	0.04324976	-2.163282275	0.030519476	0.041174366
RPL35A	15807.54764	-0.151235215	0.069907959	-2.163347599	0.030514455	0.041170429
LTBP2	126.6141962	-0.120234868	0.055575238	-2.163461139	0.03050573	0.041161495
CST9L	0.391452038	-0.582741382	0.26922058	-2.164549911	0.03042217	0.041051578
FAM200B	139.9543942	-0.096632053	0.044624358	-2.165455336	0.030352832	0.040960837
IFITM10	224.2340324	0.191620644	0.088488789	2.165479336	0.030350996	0.040960837
DUSP7	681.3718437	-0.132570931	0.06117416	-2.16710669	0.03022672	0.040796276
CCL22	17.65545553	0.222709258	0.102763201	2.167208255	0.030218978	0.040788639
ARHGEF40	2363.75188	-0.11132398	0.051361249	-2.167470254	0.030199015	0.040764506
FNDC4	8.58945138	-0.202916882	0.093586389	-2.168230708	0.030141137	0.040689184
ERVFRD-1	176.3724804	0.122231848	0.056369964	2.16838612	0.030129321	0.040676038
ARNTL2	285.596411	-0.133947057	0.061759174	-2.168860891	0.030093247	0.040630138
PCMT1	1092.689726	-0.078676027	0.036255175	-2.170063374	0.030002045	0.040509798
CARHSP1	949.3712204	0.072934933	0.033593809	2.171082553	0.029924933	0.040408465
SALL3	2.760832945	0.257682485	0.118680472	2.171229021	0.029913865	0.040396307
FAM216B	1.132809026	-0.361067613	0.166232585	-2.172062787	0.029850927	0.040314097
HSP90AA1	26821.74496	-0.100664437	0.046329063	-2.172814037	0.029794316	0.040240419
AZU1	22940.98898	0.192065421	0.088366833	2.173501236	0.029742613	0.04017336
OR2G6	1.582054952	-0.263141995	0.121055734	-2.173725987	0.02972572	0.040153314
DUSP10	5347.627928	0.102605189	0.047170623	2.175192588	0.029615687	0.040007444
GFER	126.6140188	-0.077858967	0.0357904	-2.175414856	0.029599042	0.039987718

SRGAP2	1644.940353	-0.10817172	0.049723062	-2.17548386	0.029593876	0.039983498
GPR157	107.267842	-0.135986156	0.062490674	-2.176103211	0.029547544	0.039923656
ESYT1	5385.337059	-0.076470881	0.03513312	-2.176603776	0.029510143	0.039875874
HDAC11	111.0405878	-0.091237315	0.041910752	-2.176942914	0.029484827	0.039844416
PRRC2B	11269.14595	0.071917146	0.033034743	2.17701543	0.029479416	0.039839855
CDC25B	1021.440397	-0.089690547	0.041186235	-2.177682589	0.029429676	0.03977538
TEX12	9.749570602	0.116061684	0.053283385	2.178196521	0.029391409	0.039726403
MTCL1	182.8331937	-0.194120606	0.089096686	-2.17876348	0.029349243	0.03967215
AHSP	2019.374792	-0.270060141	0.123936308	-2.179023596	0.029329915	0.039648763
SPATA5L1	362.6501481	0.074831474	0.034331938	2.179646107	0.029283704	0.039589028
ALYREF	1262.13803	-0.071347983	0.032732776	-2.179710715	0.029278912	0.039585283
ZNF280D	654.491631	-0.067218226	0.030833508	-2.180038248	0.029254627	0.039555181
PRDM13	0.656430691	-0.280755544	0.128733571	-2.180903875	0.029190527	0.039471239
C11orf86	0.748383848	0.365968214	0.167698416	2.182299762	0.029087417	0.039334531
DCAF4L2	0.361794013	-0.617147303	0.28277754	-2.182448095	0.029076478	0.039322456
LPAR1	70.52928924	0.231618499	0.10612633	2.182479126	0.02907419	0.039322079
GEN1	708.6524435	-0.076069602	0.034848428	-2.182870368	0.029045358	0.039285798
BLID	0.595419271	0.573983575	0.262895291	2.1833163	0.029012525	0.039244102
HDAC3	1436.79763	0.060924343	0.027895348	2.184032371	0.028959869	0.039175584
CCL15	0.463258405	-0.346737859	0.158722697	-2.184551206	0.028921769	0.039126747
KCNJ18	0.303390026	-0.410163837	0.187684761	-2.185386996	0.028860484	0.039046537
CHD4	1508.330348	-0.0849065	0.038846984	-2.185665163	0.028840111	0.039021671
HSBP1	1525.107981	0.103639101	0.047410621	2.185989106	0.028816402	0.038992288
APBB1IP	2402.200226	-0.089796292	0.041078058	-2.185991666	0.028816215	0.038992288
DNAJC15	571.2077991	0.106947628	0.04891992	2.186177477	0.028802623	0.038979033
AFF1	8992.977317	-0.075852823	0.034690532	-2.186556936	0.028774884	0.038944185
DESI1	894.3863078	-0.081956012	0.037474187	-2.186999047	0.028742594	0.038903173
C6orf15	0.33354586	-0.424114937	0.193909192	-2.187183244	0.02872915	0.038887666
TFDP3	0.330753144	-0.494521246	0.226040087	-2.18775905	0.028687158	0.038833512
STYXL2	73.91011069	-0.290329958	0.132679151	-2.188210852	0.028654247	0.038791643
MLLT10	3658.76921	-0.061878162	0.028272274	-2.188651745	0.028622162	0.038750887
HELB	896.852162	0.103689886	0.047336224	2.190497593	0.028488169	0.038572146
TENT5A	9367.771909	0.134938846	0.061591276	2.190875956	0.02846077	0.038537714
GOLGA5	577.2372312	0.061191345	0.027916759	2.191921555	0.028385171	0.038438007
MRNIP	1423.300965	0.088896981	0.040547345	2.192424229	0.028348889	0.038391531
CABP4	124.8145747	-0.151193963	0.068959448	-2.192505416	0.028343032	0.038386256
MORN5	1.1380167	-0.347728298	0.158557738	-2.193070501	0.0283023	0.038333743

EIF3F	8353.702957	0.088317068	0.040267923	2.193236233	0.028290363	0.038320227
SLC25A46	935.2892501	-0.067129672	0.030599874	-2.193789177	0.028250569	0.038268973
TBCC	1020.989379	-0.075640874	0.034477205	-2.193938677	0.028239818	0.038257057
AP4E1	718.3591725	0.068760618	0.031334968	2.194373342	0.028208581	0.038217384
LIPT2	51.66996134	-0.090616868	0.041252892	-2.196618558	0.0280477	0.038002051
TEX44	3.29130797	-0.23611065	0.107456952	-2.197258026	0.028002024	0.03794279
CNDP2	2406.236694	-0.071618439	0.032580155	-2.198222798	0.027933233	0.037852199
C3orf85	0.353509622	-0.698241684	0.317599842	-2.198495062	0.027913846	0.037828547
NKX1-1	1.624685791	-0.244555794	0.11121041	-2.199036884	0.0278753	0.037778925
ZNF90	56.18942106	-0.200995807	0.091394791	-2.199204188	0.027863407	0.037765422
RPL13	46833.44871	-0.142753561	0.0649024	-2.199511267	0.027841589	0.037738464
GMNC	0.743299954	-0.458783982	0.208425421	-2.201190141	0.027722566	0.037579734
NSG1	49.09651197	0.125727949	0.05711453	2.201330378	0.027712644	0.037568886
SOCS2	1658.211048	-0.237507563	0.107891605	-2.201353509	0.027711008	0.037568886
SENP6	4423.017631	-0.090066885	0.040913815	-2.201380781	0.027709078	0.037568886
RSPH9	95.46589473	0.106777452	0.048491484	2.20198358	0.027666468	0.037514083
ABCA5	421.386564	-0.163406274	0.074188724	-2.202575602	0.027624675	0.037460009
SYT11	696.0954134	0.095384927	0.043296402	2.203068188	0.027589943	0.037415503
BLZF1	578.6133986	0.09345156	0.042418589	2.20308037	0.027589085	0.037415503
KLC1	2162.483807	0.073053096	0.033152204	2.203566794	0.027554825	0.037373057
SMG6	2158.215986	-0.075824201	0.03439104	-2.204766162	0.027470508	0.037261278
AL163195.3	0.299249023	-0.510037538	0.231326206	-2.20484115	0.027465243	0.03725672
PDCL2	0.461055577	-0.368418541	0.167063343	-2.20526259	0.027435673	0.037219187
ZNF100	750.7930593	0.080931131	0.036688481	2.205900262	0.027390984	0.037161137
SMAD3	3568.058332	0.12695233	0.057527343	2.206817195	0.027326833	0.037076675
H2AC19	76.56648879	-0.205839142	0.093267242	-2.206982183	0.027315304	0.037063601
KIAA1143	790.4060176	-0.068652716	0.031101037	-2.207409246	0.027285481	0.037025702
FRMPD3	13.31987892	0.178219635	0.080730702	2.207581884	0.027273433	0.037011919
ADH7	0.447878386	-0.491678548	0.222612153	-2.208677919	0.027197051	0.036910823
EZH2	2743.038804	-0.094120836	0.042603763	-2.209214165	0.027159748	0.036862752
MMP1	1.141220037	0.453810642	0.205359661	2.209833418	0.027116726	0.036806912
CIB4	0.28687037	-0.430136435	0.194632258	-2.209995611	0.027105467	0.036794182
MMP15	108.0596725	-0.213613456	0.096657671	-2.210000033	0.02710516	0.036794182
TAS2R42	4.930944601	-0.167543234	0.075805106	-2.210184025	0.027092393	0.036781537
B9D1	63.15127226	-0.133932775	0.060586951	-2.2105878	0.027064394	0.036746073
TTLL10	41.60910221	-0.20418517	0.092350098	-2.210990295	0.027036509	0.036710759
ANKK1	6.071307898	-0.200495754	0.090675575	-2.211132974	0.02702663	0.036699891

MSLNL	16.71558696	-0.20393702	0.092223871	-2.211325744	0.027013288	0.036684319
TMEM204	60.17610457	-0.147338483	0.066591271	-2.212579531	0.026926648	0.036569197
FAM111B	86.60218988	-0.168314258	0.076039061	-2.213523632	0.026861566	0.036483341
ZNF143	795.2321624	0.092630969	0.041815752	2.215217113	0.026745166	0.036327768
DMRTB1	0.393719983	-0.311119044	0.140429613	-2.215480329	0.026727113	0.036305766
OR4X1	0.554470763	-0.591702413	0.267041821	-2.215766843	0.026707474	0.036281607
EPS15	5558.853862	0.07191951	0.032447805	2.21646761	0.026659494	0.03621894
MRPL22	666.1790186	0.099220475	0.04475262	2.217087512	0.026617112	0.036163871
SSU72P5	0.219093068	-0.790143239	0.356378445	-2.217146545	0.026613079	0.036160902
NPBWR1	18.92008517	0.284614162	0.128312806	2.218127482	0.026546141	0.036072454
PRR20G	0.355098295	-0.49158986	0.221594998	-2.218415874	0.02652649	0.036048253
KCNQ5	943.3007375	-0.135780947	0.061125035	-2.2213639	0.026326324	0.035778721
ARHGAP26	3654.937664	0.113168398	0.050943364	2.221455143	0.02632015	0.035772814
MRGPRX3	0.672742772	-0.249667195	0.112359393	-2.222041146	0.026280525	0.035721439
ZNF683	31.23863842	0.204191146	0.091885237	2.222241067	0.026267018	0.03570556
KRTAP10-7	0.320629032	-0.601124324	0.270448906	-2.222690905	0.02623665	0.035666755
PLXND1	7412.318201	-0.11462479	0.051554722	-2.223361621	0.026191425	0.03560775
NBPF12	1070.174969	-0.092190643	0.041459216	-2.223646552	0.026172234	0.03558413
AKR1C4	10.82081639	-0.248179835	0.111592405	-2.223984997	0.026149454	0.035555627
MPPE1	642.4421927	-0.064826014	0.029148522	-2.223989745	0.026149134	0.035555627
AANAT	34.26816183	0.139743271	0.062825826	2.224296584	0.026128496	0.035532068
SLC38A8	4.219514476	-0.297141	0.133448212	-2.22663905	0.025971407	0.035320897
GGT7	236.2395409	-0.116411155	0.052280818	-2.226651342	0.025970585	0.035320897
PNPT1	795.8554488	-0.086039093	0.038631723	-2.227161706	0.025936468	0.035278283
SDF2	834.8609097	0.058649069	0.026331492	2.227335559	0.025924856	0.035264939
C4orf47	9.631468533	0.157856781	0.07084994	2.22804397	0.025877583	0.035203081
YIPF3	1675.363919	-0.065661807	0.029450115	-2.229594266	0.025774391	0.035065139
SERPINA11	0.497865268	-0.4295959	0.19266448	-2.229761815	0.02576326	0.035052432
RBPMS	673.08123	-0.167910748	0.075292332	-2.230117522	0.025739642	0.035022733
CCDC85C	2445.912423	0.106745245	0.047855057	2.230594904	0.025707975	0.034982077
PFN3	0.383338483	-0.349702426	0.156741289	-2.231080456	0.0256758	0.034940724
ANO6	2067.331758	0.082702665	0.037067637	2.231128624	0.02567261	0.034938812
C11orf94	35.23580808	0.144496961	0.064754912	2.231444012	0.025651732	0.034912826
ANO5	19.28046806	-0.329961152	0.147842848	-2.231837095	0.025625732	0.034879864
HNRNPDL	9733.424409	0.070464437	0.031565717	2.232309095	0.025594541	0.034839833
RPL7L1	2373.365803	-0.055183109	0.024705685	-2.233619868	0.025508096	0.034724576
SRI	1183.574802	-0.087505791	0.039173113	-2.23382275	0.025494738	0.034708806

KCNQ1	487.3212923	0.173223266	0.077520706	2.234541921	0.025447437	0.034646821
LHX8	0.457018287	-0.742568089	0.332252315	-2.234952343	0.025420478	0.034612522
WNK2	9.235614023	-0.181837189	0.0813526	-2.235173676	0.025405949	0.034595147
ATP5MPL	1925.996693	-0.159411528	0.071290714	-2.236077043	0.025346725	0.034516903
PRKY	735.987087	-0.32251646	0.144165308	-2.237129474	0.025277879	0.034425545
H2AC17	24.95241249	-0.173366337	0.077488647	-2.237312732	0.025265908	0.034411635
NNAT	208.6504647	-0.121728395	0.054402386	-2.23755619	0.025250011	0.034392378
TUBB3	30.75792884	-0.214610085	0.095909129	-2.237639796	0.025244554	0.034387338
TLK1	3071.705594	0.062519663	0.027930849	2.238373207	0.025196728	0.034324579
ERP27	305.5299734	0.122613825	0.054774148	2.238534569	0.025186216	0.034312647
RTN4R	194.0273378	0.196892168	0.087946928	2.238761179	0.025171459	0.034294931
SLC25A6	21109.37967	-0.114248914	0.051023032	-2.23916356	0.025145276	0.034261642
RPL31	27879.53695	-0.156122856	0.069723467	-2.239172313	0.025144707	0.034261642
SNRPG	969.2454938	-0.095996704	0.042859853	-2.239781479	0.025105113	0.034211681
SLC20A1	3323.012659	0.080018725	0.035725628	2.239812966	0.025103068	0.034211276
RAB4A	1005.284089	0.074470616	0.033245862	2.239996509	0.02509115	0.034197415
PHF21A	3139.858244	0.048986966	0.021868381	2.240082004	0.0250856	0.034192231
ANKUB1	32.72508331	0.15497255	0.069175242	2.24028925	0.025072151	0.03417628
ZNF709	57.15836947	-0.12824539	0.057244729	-2.240300437	0.025071425	0.03417628
SDK2	584.2640081	-0.213927341	0.095488762	-2.240340501	0.025068826	0.03417628
ZMAT2	1225.162131	-0.074985465	0.033469616	-2.240404107	0.0250647	0.034173263
GPI	8741.229589	0.08985469	0.040098645	2.240841058	0.025036372	0.03413702
F8A2	0.255972578	0.426747473	0.190406433	2.241245036	0.025010207	0.034103719
CCDC74A	28.28216468	-0.22319779	0.099458868	-2.244121565	0.024824581	0.033852959
METRN	190.8416584	0.14000487	0.062386467	2.244154478	0.024822464	0.033852431
GABRE	149.3944551	-0.422829044	0.188395112	-2.244373751	0.024808364	0.033835559
HORMAD2	10.52333717	-0.258715238	0.115255976	-2.244701295	0.024787315	0.033809206
PUM2	7049.02941	0.074048965	0.032967253	2.246136916	0.024695239	0.033685966
LAIR1	4377.799375	-0.111700853	0.049726906	-2.246285997	0.024685695	0.033675293
NFRKB	2283.599736	-0.061704039	0.027468452	-2.246360277	0.024680941	0.033671154
PTBP1	9420.95528	0.064367884	0.028643941	2.24717272	0.024628991	0.033602624
TM4SF19	14.52309409	0.145511514	0.064752879	2.247182165	0.024628387	0.033602624
LRCH4	1639.365549	-0.078209979	0.034801638	-2.247307382	0.024620389	0.033595572
IGFL2	0.903905842	-0.323037019	0.143676068	-2.248370397	0.024552581	0.03350538
NPIPB8	6.970991752	-0.163781456	0.072844018	-2.248385811	0.024551599	0.03350538
MYDGF	1948.589811	0.119451288	0.053060269	2.2512379	0.024370474	0.033261508
C18orf32	169.9315637	0.080652232	0.03582522	2.251269665	0.024368463	0.033261083

CTC1	2081.652516	-0.074481176	0.033045322	-2.253909796	0.024201843	0.033035963
HRCT1	0.409720724	-0.339603971	0.150667517	-2.253995943	0.024196423	0.033030868
C4BPA	5.587779746	0.329203431	0.146016363	2.254565333	0.024160625	0.032984301
KANSL1	8755.772783	0.066893358	0.029668134	2.254720756	0.024150862	0.032973272
BEAN1	100.1931695	-0.167457116	0.074267523	-2.254782546	0.024146981	0.032970273
WT1	604.3064354	-0.227962319	0.101073954	-2.255401232	0.024108156	0.032919557
CYB5R3	3039.744943	-0.102421768	0.045403385	-2.255817893	0.024082038	0.032886188
SERTAD1	649.2060161	0.135310842	0.059951167	2.25701765	0.024006972	0.032785966
ZNF586	1596.756189	0.093965338	0.041627506	2.257289655	0.023989981	0.032765048
SCAND1	1687.003543	-0.11808476	0.052303789	-2.25767123	0.023966164	0.032734803
NETO2	333.608578	0.143142681	0.063377499	2.258572581	0.023909985	0.032660349
CDC23	739.702037	-0.057484459	0.025449525	-2.258763561	0.023898096	0.032646387
EDARADD	12.33908795	-0.205527981	0.090967613	-2.259353339	0.023861414	0.032598553
EPM2AIP1	2349.447994	-0.072369791	0.032022805	-2.259945399	0.02382464	0.032550585
ZNF492	63.44201204	0.17127975	0.075785974	2.2600455	0.023818427	0.032544368
OTOP1	0.421395831	-0.344995756	0.152605278	-2.260706573	0.023777433	0.032490624
APOBEC3G	606.1005454	-0.084278304	0.03727673	-2.260882462	0.023766536	0.032478002
THEG	1.135796462	-0.33622573	0.148702405	-2.261064509	0.023755263	0.032464862
HM13	3602.368397	0.055821288	0.024673489	2.262399442	0.023672735	0.032354336
AC008878.1	0.646505096	-0.355252946	0.157002766	-2.262717758	0.023653093	0.032329748
MS4A18	0.465762575	-0.475170731	0.209881775	-2.26399234	0.023574586	0.032224692
PRR9	1.166108773	-0.473525869	0.209149135	-2.264058466	0.023570519	0.032221384
AC005832.4	0.318750889	0.3474996	0.153484381	2.264071421	0.023569722	0.032221384
UBR7	707.0689327	-0.070876277	0.031300919	-2.264351333	0.023552515	0.032201269
XAGE5	0.339505886	-0.449418382	0.198435974	-2.26480296	0.023524774	0.032165588
ZHX2	801.8096795	0.133419692	0.05888495	2.265768962	0.023465533	0.032086829
DMXL2	5654.095754	0.160833228	0.070980228	2.265887743	0.023458257	0.032079122
CDH22	8.57322048	0.238536172	0.105261814	2.266122567	0.02344388	0.032061701
OR6M1	0.314984087	-0.691191289	0.304863009	-2.267219271	0.023376834	0.031972243
TULP2	233.6428485	-0.181475966	0.080042334	-2.267249813	0.023374969	0.031971927
PRCP	1251.795849	0.096763086	0.0426724	2.267580112	0.023354811	0.031946587
GRM2	65.30756111	-0.135962065	0.059927329	-2.268782323	0.023281566	0.031848623
CDR2L	29.15741301	-0.202143305	0.089055751	-2.269851207	0.023216612	0.031761987
TOMM20L	37.98503617	-0.130712661	0.057582925	-2.269989956	0.023208192	0.031752688
XKR6	44.10166269	-0.197670719	0.087062786	-2.270438701	0.023180979	0.031717672
TENM1	538.9448468	0.17740553	0.0781328	2.270564101	0.023173379	0.03170949
C10orf62	0.526094317	-0.352046751	0.155002781	-2.271228605	0.023133143	0.031656645

TOP1	4587.216267	0.098111615	0.043194919	2.271369361	0.023124628	0.031647206
SFMBT1	389.7204595	-0.131301368	0.057774501	-2.272652558	0.023047126	0.031543346
SMYD1	0.998991832	-0.302396877	0.132983657	-2.273940159	0.022969585	0.031439418
PEX13	695.0207295	0.069006444	0.030343214	2.274196948	0.022954148	0.031420486
AIG1	403.8264981	-0.160037893	0.070363654	-2.274439753	0.02293956	0.031402714
DEFB134	0.409994034	-0.568519979	0.249923127	-2.27477939	0.022919167	0.031376992
RPLP1	64024.25362	-0.175345849	0.077069796	-2.275156504	0.022896543	0.031348212
RERE	4730.061686	-0.077853001	0.034214725	-2.275423826	0.022880517	0.031328462
TSGA10IP	15.08088814	-0.136027306	0.059779367	-2.275489216	0.022876599	0.031325288
PCNX1	6954.495807	0.084214995	0.037006146	2.275702916	0.022863797	0.031309948
GFM1	978.3375595	-0.056589504	0.024855198	-2.276767388	0.02280012	0.031224933
HACL1	586.6714269	-0.048667198	0.021369479	-2.277416232	0.022761382	0.031174062
TSPAN13	388.1759153	-0.145951849	0.064049909	-2.278720622	0.022683678	0.031069813
GH1	0.915203323	-0.353528458	0.155072497	-2.279762461	0.022621781	0.030987201
HBA2	23307.47625	0.331243824	0.145291423	2.279858075	0.022616107	0.030981598
GNL2	1571.603095	0.04851985	0.021279881	2.280080909	0.02260289	0.030965659
EPN2	219.9922303	0.167858641	0.073587812	2.281065791	0.022544554	0.030887901
CIAO3	632.5382858	-0.106331754	0.046607915	-2.281409791	0.022524209	0.030862187
KRT14	0.764721669	-0.445666175	0.195339036	-2.281500847	0.022518827	0.030856972
FOXO3	5434.780929	0.124612972	0.054610618	2.281845129	0.022498485	0.030831258
PLBD2	1309.147542	0.138654315	0.06076104	2.281960857	0.022491651	0.030824051
PRKCG	91.57183499	-0.173391613	0.075971902	-2.282312377	0.022470904	0.030797774
PKIG	571.4535619	-0.124356425	0.054484013	-2.282438808	0.022463446	0.030789708
FBXO30	1176.784991	-0.123412202	0.054067917	-2.282540334	0.022457459	0.030783658
CD70	162.0555779	-0.251982371	0.110370888	-2.283051035	0.022427362	0.030744555
ANK3	179.519868	0.192920589	0.084466687	2.283984324	0.022372452	0.03067143
NLRP13	0.746586726	-0.420103099	0.183898286	-2.284431838	0.022346164	0.030637537
SEPHS2	1792.939005	0.076015339	0.033258762	2.28557332	0.022279232	0.03054791
SGSM2	2364.769464	-0.065784642	0.028779422	-2.285822234	0.02226466	0.030530068
EZHIP	1.079792583	0.306009669	0.133864162	2.285971583	0.022255921	0.030520223
STAT5A	6582.58811	0.074921564	0.032763264	2.286755193	0.022210116	0.030459543
ATP2B2	38.89834021	-0.185847734	0.081248424	-2.2874011	0.022172422	0.03040998
H2BC17	1430.589721	-0.197762294	0.086449968	-2.287592452	0.022161266	0.030396809
SLC27A3	559.5919262	-0.122313045	0.053461112	-2.287888148	0.022144036	0.030375304
RGMB	25.75502076	0.187416733	0.081897434	2.288432297	0.022112359	0.030333978
PSG11	0.559230344	-0.524714503	0.229272535	-2.288606015	0.022102254	0.030322242
THBS4	205.6744113	-0.181682331	0.079380271	-2.288759275	0.022093343	0.030312141

CGB7	0.517192737	-0.437760028	0.19123583	-2.289110928	0.022072908	0.030286227
COMMD9	1576.319749	0.073148056	0.031953563	2.289198769	0.022067806	0.030281349
TFEC	1362.077887	-0.149876867	0.06545788	-2.289668804	0.022040523	0.030246032
TOMM22	1523.164543	-0.093257764	0.040685587	-2.292157274	0.021896569	0.030050593
CADM3	2.491756542	-0.300068932	0.130892655	-2.292481053	0.0218779	0.030027076
DOCK6	331.4046306	-0.210916059	0.091964169	-2.293459089	0.021821588	0.029951889
PRG4	25.59169604	-0.138396481	0.060340504	-2.293591708	0.021813962	0.029943522
PPP4R3B	3347.288176	0.068508234	0.029868899	2.293631061	0.0218117	0.029942516
H1-8	6.027143295	-0.224822415	0.097952501	-2.295218732	0.021720592	0.029819538
PHGDH	355.4220416	-0.19392775	0.084480609	-2.295529746	0.021702784	0.029797179
INTS10	1785.809393	-0.054595575	0.023782777	-2.295592896	0.021699169	0.029794307
HSPA8	19956.27413	-0.10750527	0.04682123	-2.29607959	0.021671331	0.029758171
CCR10	28.6211376	-0.127728601	0.055627248	-2.296151699	0.021667209	0.029754598
RGS18	626.5739302	-0.140029494	0.060976511	-2.296449767	0.021650178	0.029733296
RTN3	4764.586815	0.075273297	0.032745952	2.298705426	0.021521672	0.029558886
QSOX2	1060.438423	-0.093546128	0.040678001	-2.299673671	0.021466715	0.029485474
TFF1	0.789646592	-0.42688249	0.185468427	-2.301645063	0.021355197	0.029334358
PEAK1	1886.366795	0.105023199	0.045607967	2.302737985	0.02129359	0.029251785
MAJIN	29.42771035	-0.223952122	0.097236882	-2.303160255	0.021269828	0.029221194
PRKCD	3188.023805	-0.10622456	0.046107581	-2.303841555	0.02123154	0.029170639
CREBL2	350.4987392	-0.105719069	0.045884504	-2.304025537	0.02122121	0.029158494
TEX13C	0.456597022	-0.470310592	0.204071329	-2.304638253	0.021186842	0.029113315
NELFCD	2559.139257	-0.047182591	0.020467791	-2.30521171	0.021154719	0.029071215
MYF6	1.831254939	0.472953775	0.205048845	2.306542009	0.021080366	0.028971071
NPTX2	140.0493168	0.315117296	0.136593853	2.306965428	0.021056748	0.028940645
OR2A7	6.030403415	-0.222681497	0.096517927	-2.307151677	0.021046366	0.028928407
ZNF548	961.4257497	0.075905135	0.032896436	2.307396949	0.021032701	0.028911655
CR589904.2	3.505220944	0.275244469	0.119250867	2.30811295	0.020992855	0.028858909
MMP10	0.672295328	0.507741213	0.21988385	2.309133718	0.020936162	0.028782995
OVCH2	3.72648143	-0.345755357	0.149728152	-2.309220754	0.020931334	0.028778379
MCPH1	999.5113656	0.047438316	0.020538199	2.309760235	0.020901432	0.028739285
IL34	21.7981032	-0.131703073	0.057010018	-2.310174211	0.020878511	0.028709786
HADHA	6776.103597	0.058969668	0.02552602	2.310178754	0.02087826	0.028709786
BBC3	679.0487807	0.128219692	0.055495759	2.310441265	0.020863737	0.028693502
PSMC3	1609.476507	-0.074200593	0.032107105	-2.311033441	0.020831008	0.028650504
MUSK	6.121863973	-0.26223521	0.113443169	-2.31159983	0.020799746	0.028609517
DIPK1C	7.629448862	-0.460619431	0.199204907	-2.312289577	0.020761731	0.028559235

MORN3	144.8102066	0.110865493	0.047944711	2.312361255	0.020757784	0.028555812
PRSS33	23.85622227	0.23416495	0.101233818	2.313109936	0.020716596	0.028501155
COG5	1648.243596	-0.075932687	0.032816023	-2.313890628	0.020673722	0.02844417
OR56A5	0.290673045	-0.64767133	0.279872433	-2.31416622	0.020658606	0.028425371
KAT8	2225.70237	0.048241398	0.020843859	2.314417751	0.020644818	0.028408396
DYNAP	3.723576893	-0.453497239	0.195852721	-2.31550135	0.020585511	0.028328777
SPRED1	88.39023235	-0.251647646	0.108671206	-2.31567914	0.020575794	0.028317397
CA11	73.70379948	-0.119307282	0.051519955	-2.315748959	0.02057198	0.028314138
AL928654.3	39.28707124	0.213902599	0.092368633	2.31574933	0.020571959	0.028314138
CYP11B1	2.065568604	-0.287804225	0.124272281	-2.315916485	0.020562829	0.028305524
ITGA9	1865.670166	-0.206555938	0.089186757	-2.315993369	0.020558631	0.028301736
GGTA1	120.9101459	0.208253555	0.089884645	2.316898007	0.020509288	0.028235795
C10orf105	21.343904	-0.207948738	0.089750714	-2.316959144	0.020505957	0.028233195
ABCF1	3086.185287	-0.069577109	0.030028399	-2.317043533	0.02050136	0.028228852
ZBTB24	608.9744803	0.068127099	0.029400484	2.317210144	0.020492287	0.028218344
C9orf50	16.36267282	-0.181098455	0.07810828	-2.318556435	0.0204191	0.028119541
SLC2A12	12.62778581	0.201842774	0.087053518	2.318605607	0.020416431	0.028117844
BTF3	13434.4686	0.121690007	0.052483071	2.318652555	0.020413883	0.028116314
ANTXR2	3613.094684	0.101887462	0.043926997	2.319472492	0.020369431	0.028057064
RMDN2	62.37429884	-0.162263677	0.069952542	-2.319625159	0.020361164	0.02804765
AATK	382.1833962	0.172773382	0.074461911	2.320292078	0.020325082	0.027999918
SARNP	352.9877469	-0.136972464	0.059012071	-2.321092304	0.020281863	0.027942345
ZSWIM8	2597.27838	-0.077900419	0.033539944	-2.322616236	0.020199778	0.027831216
SOX5	68.61346342	-0.271585829	0.116923574	-2.32276366	0.020191852	0.027822255
MECP2	4313.145016	0.076049794	0.032740156	2.322829317	0.020188323	0.027819351
HNRNPA1	37528.55906	0.088422287	0.038064898	2.32293506	0.020182641	0.027813479
SDC1	31.59328771	-0.203422118	0.087567103	-2.323042674	0.02017686	0.02780747
BUD31	808.0115113	0.099489921	0.042821151	2.323382704	0.020158602	0.027784263
RBM43	396.6434477	0.080181087	0.034493972	2.324495612	0.020098946	0.027703991
CLCN7	4013.674527	-0.089835641	0.038645346	-2.324617294	0.020092433	0.027696964
PLD1	1899.392542	-0.125002826	0.053770292	-2.324756312	0.020084994	0.02768866
OR5AN1	5.875133023	-0.282158312	0.121369928	-2.324779428	0.020083758	0.02768866
ZNF296	266.9947924	-0.140361569	0.060326324	-2.326705151	0.019980964	0.027549126
PLPPR2	693.0050388	0.118457361	0.050908241	2.326879849	0.019971661	0.02753824
JPH2	5.286797736	-0.222462671	0.095588737	-2.32728957	0.019949859	0.027510116
ADRB1	144.1415965	0.259085408	0.111301752	2.327774746	0.019924068	0.027476487
LSR	177.254083	0.108644291	0.046667603	2.328045223	0.019909702	0.027458611

MIS12	416.3687312	0.060932117	0.026165307	2.32873693	0.019873006	0.027409933
SUSD1	1739.897646	-0.083124085	0.035694329	-2.328775703	0.019870951	0.027409029
ALKBH8	212.4530344	-0.096743356	0.041542568	-2.328776506	0.019870909	0.027409029
ISG20L2	1106.529958	0.060698423	0.026063144	2.328898703	0.019864433	0.027403901
KRTAP12-2	0.370739371	-0.934486113	0.401230831	-2.329048621	0.01985649	0.027394874
DIO3	0.797747956	-0.330629179	0.141853193	-2.330784187	0.019764742	0.027270217
NCR3	40.56652876	-0.15175861	0.065070822	-2.332206736	0.019689818	0.027168756
YARS1	4422.529619	0.072740002	0.031184141	2.332595957	0.019669361	0.027142443
EGLN2	1124.853386	0.116774668	0.050040217	2.333616358	0.019615819	0.027070467
OR10J1	0.500898652	-0.323866927	0.138772059	-2.333805015	0.019605934	0.027058733
DNAJC10	2064.074419	-0.085129827	0.036464766	-2.334577603	0.019565498	0.027004829
MOXD1	6.727458474	-0.204294365	0.087480755	-2.335306375	0.019527421	0.026954176
ZCWPW2	9.341579878	-0.124941321	0.053499482	-2.335374431	0.019523869	0.026951173
INAVA	64.72709037	-0.238690858	0.102196681	-2.335602841	0.01951195	0.02693662
CDK11A	227.1085944	0.098204191	0.042029855	2.336534114	0.019463422	0.026871521
IFI35	472.8329187	-0.091757819	0.039255163	-2.337471358	0.019414689	0.02680613
SMIM41	4.668287688	-0.226318248	0.096819569	-2.33752589	0.019411856	0.02680411
PRKAG3	2.536756817	0.309279889	0.132301932	2.337682342	0.019403733	0.026794784
TMEM132A	183.1789221	0.210846641	0.090167245	2.338395067	0.019366763	0.026745619
TRIM17	67.7239672	-0.13988732	0.059814369	-2.338690907	0.019351436	0.026726338
AMY1B	0.582333549	-0.315494873	0.134872352	-2.339210871	0.019324523	0.026691051
GNAS	28996.55674	0.087785491	0.037527338	2.339241081	0.01932296	0.026690776
KMT2C	19245.42336	0.102162853	0.043665626	2.339663102	0.019301142	0.02666252
GPIHBP1	4.779049534	-0.281645216	0.120345479	-2.34030574	0.019267959	0.026618561
KCNE1B	9.298684914	-0.216088203	0.092326536	-2.340477736	0.019259087	0.026608181
LRFN5	0.635015579	-0.473506846	0.202282123	-2.340823991	0.019241236	0.026585395
ZNF333	1437.360253	0.071423145	0.030509104	2.341043669	0.019229918	0.026571633
TUBA3E	9.465647076	-0.143019879	0.061077903	-2.341597734	0.019201398	0.026534098
AL512506.3	0.693337158	-0.263451384	0.112500327	-2.341783274	0.019191856	0.026522784
MESP2	4.38052981	-0.219890476	0.093859619	-2.342759081	0.019141739	0.026455392
OR8H2	0.50059057	-0.659775831	0.281594012	-2.343003764	0.01912919	0.026439915
IVNS1ABP	5847.952506	-0.140849985	0.060101402	-2.343539104	0.01910176	0.026403866
ZNF726	188.6922637	-0.139276822	0.05942771	-2.343634319	0.019096885	0.026398992
PPP1R15A	20097.07936	0.109840424	0.046867263	2.34364922	0.019096122	0.026398992
CSNK2A1	4061.04747	0.049195457	0.020989874	2.343771018	0.019089887	0.026393047
PRR23B	0.488675705	-0.499705532	0.213157733	-2.344299341	0.019062865	0.026357548
C1orf43	2657.155692	0.077040194	0.032858134	2.344630826	0.019045927	0.02633599

OR52N1	6.916129445	-0.267983635	0.114269748	-2.345184438	0.019017669	0.026298774
SLC26A6	902.2299047	-0.120349141	0.051303591	-2.345822919	0.018985125	0.026255624
VRK2	650.8596666	-0.067957709	0.028954032	-2.347089636	0.018920702	0.02616838
SYNGR1	2044.744807	-0.146813413	0.062548792	-2.347182232	0.018916001	0.026163726
TMEM151A	22.37004454	-0.233107624	0.099285786	-2.347844873	0.018882384	0.026119074
TTN	5526.355595	-0.17579704	0.074870325	-2.34802024	0.018873496	0.026108625
GMPPB	680.1299688	0.098273438	0.041846554	2.348423678	0.018853063	0.026082202
CGGBP1	3054.046624	0.055550263	0.023638225	2.350018415	0.018772482	0.025972559
FABP3	57.22985719	-0.13400941	0.057016737	-2.350352132	0.018755658	0.025951117
RIC8A	2437.375902	-0.061241495	0.026053346	-2.350619146	0.018742206	0.025934337
PTK2	717.6098654	-0.199627692	0.08491146	-2.351010007	0.01872253	0.025908943
ZNF276	1607.186541	-0.082905697	0.035259649	-2.351291035	0.018708395	0.025891212
CNGA3	0.854842264	-0.284154822	0.120828241	-2.351725229	0.018686573	0.025862841
ANO9	230.1164741	0.138855839	0.059043186	2.351767374	0.018684456	0.025861739
H1-10	9910.4482	0.171527146	0.07291419	2.352452178	0.018650088	0.025815995
HTR2B	174.6205798	0.149150014	0.063379515	2.353284255	0.018608403	0.025760115
BRMS1	848.3440595	0.077017057	0.032717408	2.354008518	0.018572185	0.025711797
CYP3A7-CYP3A51P	1.388510058	-0.205089684	0.087123385	-2.354014171	0.018571903	0.025711797
CAMK2N1	9.18914854	0.186796662	0.07934504	2.354232361	0.018561005	0.025699953
HAUS8	294.8542285	-0.073164175	0.031076208	-2.354346947	0.018555283	0.025693849
CCDC130	1009.776268	-0.069693131	0.029598717	-2.354599728	0.018542667	0.025678196
NXPH4	7.378389004	0.207899029	0.088285889	2.354838714	0.018530747	0.025663504
PRPS2	834.910357	0.079583343	0.033778159	2.356059247	0.018469972	0.025581146
NXPH1	0.302188541	-0.572342038	0.242868909	-2.356588336	0.01844368	0.02554654
IQSEC2	473.0425423	0.164330033	0.069719121	2.357029615	0.018421778	0.025518008
DDX43	62.38198474	0.14863128	0.063057635	2.357070316	0.018419759	0.025517017
CPEB2	1504.982796	-0.17076145	0.07240639	-2.358375434	0.018355119	0.025429271
C12orf4	367.6059218	-0.073614128	0.031210011	-2.358670317	0.018340541	0.025410874
NUPR2	1.800914133	-0.25376718	0.107581453	-2.358837633	0.018332275	0.025401219
DUSP1	22480.89842	0.141284069	0.059884148	2.359289947	0.018309943	0.025372072
SEC31A	6690.415099	0.053141819	0.022515962	2.360184281	0.018265859	0.025312777
WDR91	1620.626819	-0.104387657	0.044221985	-2.360537588	0.018248469	0.025290468
MVD	617.417006	-0.081945945	0.034711034	-2.360803943	0.018235368	0.025274102
CMTM4	747.1053882	-0.138866641	0.058821035	-2.360832997	0.01823394	0.025273911
VPS13C	8192.9692	-0.109042211	0.046185445	-2.360964808	0.01822746	0.025266719
MTMR1	1035.445818	0.079949892	0.033848686	2.361979171	0.018177663	0.025199476
KCTD11	75.96750586	-0.12502916	0.052930783	-2.362125639	0.018170483	0.025191306

C7orf26	869.3560896	-0.090009116	0.038104522	-2.362163672	0.018168619	0.025190506
ETNK2	28.85544483	0.188539505	0.079793477	2.362843573	0.018135323	0.025146123
RHOT1	1380.722075	0.057125865	0.024170487	2.363455255	0.018105413	0.025106429
PRAME	1314.868963	0.340968665	0.1440837	2.36646244	0.017958996	0.024905161
DEFB135	0.396272819	-0.631837327	0.266949909	-2.366875981	0.017938943	0.024879114
PTPRK	61.43012197	-0.213787875	0.090285473	-2.36790999	0.017888887	0.024811451
PIAS3	507.5465224	-0.069432246	0.029314322	-2.368543514	0.01785828	0.024770754
HIPK2	4190.518712	0.104086471	0.043922405	2.369780805	0.017798634	0.02468977
DST	1400.464242	-0.220316427	0.092928025	-2.370828695	0.017748255	0.024621631
CACNA1H	159.0885755	0.261735438	0.110385906	2.371094719	0.017735485	0.02460566
MYOD1	0.596473834	-0.39207364	0.165256696	-2.372512883	0.017667547	0.024513142
TMEM259	3945.065224	-0.082620514	0.034813043	-2.373263233	0.017631693	0.02446513
CD200R1L	1.317287156	-0.347488429	0.146414458	-2.373320467	0.017628961	0.024463074
RPL27	18016.35738	-0.207979698	0.087627422	-2.373454496	0.017622564	0.024455931
RFWD3	3328.495804	0.075405319	0.031761084	2.374141864	0.017589791	0.02441218
LMAN1L	10.93150666	-0.24409105	0.102801391	-2.374394431	0.017577762	0.024397216
BPIFA1	0.360603616	-0.451903252	0.190289302	-2.374822162	0.017557407	0.024370692
IRAK1	4339.208937	0.078647013	0.03311245	2.37514933	0.017541852	0.024350827
APOM	168.9649363	0.089731411	0.037777417	2.375265921	0.017536311	0.024344863
ZNF682	155.0355178	0.142095452	0.059815048	2.37558032	0.017521379	0.024325858
SAV1	432.4452963	0.096100298	0.040449759	2.37579408	0.017511232	0.024313496
NSA2	1906.939239	0.138359158	0.058229884	2.376085077	0.017497428	0.024296053
PDLIM7	2089.31226	0.125645639	0.052834463	2.378100071	0.017402103	0.024165403
HOXA10	710.0603288	-0.309494011	0.130120463	-2.378519128	0.017382335	0.024139665
LRPPRC	4674.941048	-0.069557249	0.029240995	-2.378757976	0.017371077	0.024125743
TMEM233	12.89753634	-0.291376621	0.122487705	-2.37882341	0.017367994	0.024123173
APC	1691.922508	0.086199048	0.036229175	2.379271611	0.017346889	0.024095569
BEND4	201.8346069	-0.267263043	0.112327981	-2.379309601	0.017345101	0.024094795
SAMD4A	105.5427356	0.151345204	0.063601335	2.379591648	0.017331833	0.024078072
DCAF4L1	61.84002727	-0.18988363	0.079791181	-2.379757109	0.017324053	0.024068973
KRTAP9-6	0.434451871	-0.318455918	0.133805468	-2.379991814	0.017313023	0.024055355
KAAG1	0.413664461	-0.513338463	0.215660954	-2.380303221	0.017298397	0.024036741
BTD	1386.428953	-0.092409068	0.038817788	-2.380585613	0.017285144	0.02402003
APOBEC3A	488.1857641	0.229214143	0.096279822	2.380708012	0.017279402	0.024013756
DNAJB13	102.3385647	0.133719486	0.05616516	2.380826249	0.017273857	0.024007755
THAP9	172.6030625	0.084771068	0.035579196	2.382602117	0.017190763	0.023893965
TFF2	1.446096935	-0.348730971	0.14633797	-2.383051859	0.017169775	0.023866488

ASB17	0.30421295	-0.682375884	0.286286984	-2.383537922	0.017147118	0.023836686
RFX5	2082.602197	-0.060173594	0.025231883	-2.384823767	0.017087305	0.023755225
ABHD4	1291.458357	-0.134910866	0.056538033	-2.386196643	0.017023646	0.023668406
DCTN2	2724.017641	0.058624889	0.024559035	2.387100625	0.016981842	0.023611963
HABP2	1.175355351	-0.304503535	0.127543761	-2.387443587	0.016966006	0.02359162
MON2	3085.366732	-0.088607896	0.037093847	-2.388749147	0.016905841	0.023509629
MEMO1	362.7293353	-0.090142475	0.037732659	-2.388977565	0.016895334	0.023496687
STARD8	723.7095908	0.174183864	0.072902245	2.389279827	0.016881439	0.023479031
ZNF44	988.3948147	0.094678496	0.039612621	2.390109334	0.016843358	0.023427732
ACTL10	2.007779281	-0.199319229	0.083380039	-2.390490945	0.016825864	0.023405063
FCGR3B	307.0010473	-0.306829126	0.128337943	-2.390790426	0.016812146	0.023387643
RBM5	6037.343105	0.074263395	0.031047485	2.391929524	0.01676006	0.023316843
FGD4	898.1358959	-0.181716479	0.075949906	-2.392583336	0.016730228	0.023276994
ASDURF	0.234088404	0.36592923	0.1529291	2.392803132	0.01672021	0.023264709
TMEM203	294.233664	-0.082887196	0.034639979	-2.39281889	0.016719492	0.023264709
TNFSF11	4.011381166	-0.365090494	0.15256468	-2.39302107	0.016710281	0.023254201
PON2	288.41209	-0.197907409	0.082672246	-2.393879653	0.016671218	0.023201489
PRSS22	5.027896668	0.279584678	0.116768791	2.394344202	0.016650115	0.023173769
KARS1	3416.361869	0.063528897	0.026530563	2.394555211	0.016640538	0.023162086
SPINT1	304.7594768	-0.200736852	0.083820089	-2.394853719	0.016626997	0.023144884
PEX11B	373.8652725	0.062261698	0.025991323	2.395480158	0.016598613	0.023107016
SPAG4	179.6006316	0.108281051	0.045199084	2.395647049	0.016591058	0.023098142
C3orf62	725.1360184	0.108558601	0.04530661	2.396087494	0.016571134	0.023072045
RAP1GAP2	2112.274754	-0.114239525	0.047665002	-2.396717096	0.016542691	0.023034081
ANKHD1	453.9974979	0.10044206	0.041903587	2.396980038	0.016530824	0.023019196
CLMP	72.86617856	0.153574134	0.064069633	2.396987887	0.01653047	0.023019196
TNIK	719.5921881	-0.178404191	0.074423166	-2.397159376	0.016522735	0.023011207
PBDC1	401.4301798	0.108557426	0.045283959	2.397260073	0.016518195	0.02300652
TK2	615.9433108	-0.098429792	0.041056807	-2.397404939	0.016511665	0.022999062
FCRL4	1.103600637	-0.375573694	0.156653456	-2.397481066	0.016508234	0.02299592
ARHGAP11B	107.3337518	-0.117227111	0.048890328	-2.397756701	0.016495818	0.022980259
AL391628.1	24.05226301	-0.115535541	0.048184718	-2.397763128	0.016495529	0.022980259
RPS9	24309.48528	-0.125434284	0.05230814	-2.39798787	0.016485411	0.022969031
CLSTN1	3191.036255	-0.096778648	0.040346613	-2.39868086	0.016454248	0.022927245
RNF111	1732.991978	0.082073812	0.034212875	2.398915934	0.016443689	0.022914163
OR4F6	0.367086818	-0.450343665	0.187557964	-2.401090597	0.016346289	0.022780058
VSTM2A	0.49557989	-0.573237682	0.238721301	-2.401284176	0.016337643	0.022769631

N4BP2	2101.157998	0.083936519	0.034951628	2.40150526	0.016327774	0.022757497
BLCAP	1439.00538	0.064249715	0.026753718	2.401524725	0.016326906	0.022757497
GTF2E2	459.5820644	-0.061743149	0.025709318	-2.401586385	0.016324154	0.022755692
CCM2L	3.386641069	-0.258192334	0.107507352	-2.40162491	0.016322435	0.022754917
MARVELD2	34.86544721	-0.154245573	0.064201739	-2.402513963	0.016282813	0.022701296
LACRT	0.902633209	-0.534922591	0.222625218	-2.402794243	0.016270339	0.022685521
CRYBA2	0.751730327	-0.454918127	0.189327853	-2.402806137	0.01626981	0.022685521
PRPF38B	2840.952164	0.069742821	0.029020907	2.403192289	0.016252639	0.022664071
DENND4A	5953.566904	0.103635549	0.043120355	2.403402038	0.016243318	0.022652688
ZSCAN12	347.6966946	0.104924157	0.043653091	2.403590526	0.016234947	0.022642626
ENO4	6.027387233	-0.320899091	0.133504903	-2.403650225	0.016232296	0.022640542
CXorf66	0.407445395	-0.422472079	0.17575571	-2.403745961	0.016228046	0.022636228
CCDC3	49.78599308	0.270141305	0.112350475	2.404451833	0.016196741	0.02259417
ZNF7	656.1069322	-0.073524585	0.030574134	-2.404796948	0.016181454	0.022574455
LRRC37A2	470.940404	-0.091603484	0.038086778	-2.405125546	0.016166911	0.022555773
HTR3A	4.174661234	0.261211133	0.108590623	2.405466738	0.016151822	0.022536329
NKPD1	120.2212993	-0.148711233	0.061795014	-2.406524806	0.01610511	0.022472754
FGD1	27.11236132	0.155248766	0.064506628	2.406710292	0.016096934	0.022462946
PRSS57	5928.562705	-0.183980537	0.07643222	-2.40710706	0.016079455	0.022440155
TINF2	1435.353882	0.055408011	0.023018184	2.407140898	0.016077966	0.022439676
MMD2	0.7696394	-0.328884652	0.136562118	-2.408315405	0.016026329	0.022369203
GAPT	845.0603552	-0.150226722	0.062356322	-2.409165881	0.015989029	0.022318732
ADCYAP1R1	1.046945535	-0.332189022	0.137848766	-2.409807725	0.01596093	0.022281098
STEAP1	2.002778192	-0.476145466	0.197583106	-2.409849082	0.015959121	0.022280162
ZNF507	762.2849656	-0.11479094	0.047621729	-2.410474019	0.015931806	0.022243615
HID1	26.08934135	0.172288246	0.071470793	2.410610516	0.015925845	0.022236879
IFITM2	3246.607563	-0.197292379	0.081832011	-2.410943793	0.0159113	0.022218155
OXA1L	4611.708517	0.090535492	0.037551875	2.410944654	0.015911263	0.022218155
TMEM89	5.205680514	0.199105338	0.08255631	2.411751907	0.01587608	0.022172139
CTAGE8	0.768247833	-0.312042282	0.129344888	-2.412482521	0.015844297	0.02212933
NAT14	246.2622099	0.115339697	0.047762445	2.414861643	0.015741188	0.021986889
RCC2	7503.482433	0.075619388	0.031289687	2.416751197	0.015659717	0.021874653
OR9I1	0.344983638	-0.540832469	0.223756207	-2.4170613	0.015646382	0.021857586
PTPRU	57.38816986	0.218108208	0.090203843	2.417948055	0.015608305	0.021805949
GPR82	35.23234118	-0.179944577	0.074405883	-2.418418661	0.01558813	0.021779318
KCNU1	0.761061923	-0.696681464	0.288050634	-2.418607642	0.015580035	0.021769562
NPIPA3	1.000925951	-0.273541785	0.113074334	-2.419132403	0.015557576	0.021739733

EMB	7743.121455	0.127016848	0.052503637	2.419200925	0.015554646	0.02173719
TNKS	2685.579526	-0.087627174	0.03621736	-2.419479913	0.015542719	0.021722074
PML	3769.715834	0.081184722	0.033546978	2.420030825	0.015519191	0.021690741
MFAP2	17.28356542	-0.235121407	0.097130441	-2.420676822	0.015491643	0.021653783
SNX20	1054.336513	0.104349945	0.043084642	2.421975444	0.015436394	0.021578098
SLC35G1	40.47168071	-0.237507797	0.098050216	-2.422307749	0.015422284	0.021559914
RSL1D1	4614.16722	0.103401425	0.042679279	2.422754702	0.015403324	0.021534947
VSIG2	71.13960379	-0.184300717	0.076061939	-2.423034698	0.015391456	0.021519893
H2AJ	1077.369995	0.187843811	0.077523293	2.423062853	0.015390264	0.021519762
PLRG1	688.8615693	-0.060044682	0.024780037	-2.423107075	0.01538839	0.02151868
PIFO	14.88368853	-0.176147	0.072687778	-2.42333724	0.015378643	0.021506586
SLC35E3	967.1261452	-0.082479886	0.034034857	-2.423394486	0.015376219	0.021504733
PBLD	647.7053971	-0.10243865	0.042242199	-2.425031152	0.015307073	0.021409557
OTUD7A	32.23121076	0.181168429	0.074705865	2.425089777	0.015304601	0.02140763
ACER1	4.404556024	-0.168581302	0.069433334	-2.427959187	0.015184052	0.021240526
IL20RB	49.5345312	-0.140119047	0.057702779	-2.428289388	0.015170233	0.021222713
ASB15	41.60207125	-0.260711439	0.107337518	-2.428893871	0.015144965	0.021188878
ADORA3	13.85573725	0.174337855	0.071735001	2.430303936	0.015086166	0.021108122
PHEX	32.72392804	-0.173334176	0.071271137	-2.432038874	0.015014096	0.021008786
TUB	16.60431435	-0.214979328	0.0883813	-2.432407389	0.014998827	0.020988921
DGKE	955.3126549	-0.114175178	0.046903653	-2.434249181	0.014922718	0.02088391
EIF5A	5665.582802	-0.105362145	0.043282954	-2.434264188	0.014922099	0.02088391
AC010980.1	5.691743088	0.319144095	0.131099246	2.434370178	0.01491773	0.020879915
CEP57	1338.421484	0.067479613	0.027716973	2.434595285	0.014908454	0.020868424
PLEKHH1	105.1743032	-0.184499933	0.075776995	-2.434774993	0.014901052	0.020859555
PKP3	19.86287653	-0.241200577	0.099032631	-2.435566689	0.014868484	0.020815452
GPR33	0.43125164	-0.45809081	0.188082263	-2.435587504	0.014867628	0.020815452
ATP6V0A1	2807.047633	-0.141442279	0.058055869	-2.436313172	0.014837832	0.020775512
HTRA4	24.9611441	-0.202374188	0.083043519	-2.436965485	0.014811093	0.020739557
SEC14L6	4.698931048	-0.259883612	0.106641095	-2.436993095	0.014809962	0.020739457
OR7A5	0.685036168	-0.44435065	0.182328501	-2.437088271	0.014806065	0.020735482
DLGAP3	88.03818005	-0.225863546	0.092677179	-2.437099932	0.014805587	0.020735482
RAPGEF6	606.5119278	0.098034986	0.040213363	2.437870843	0.014774052	0.02069361
ARL5C	20.43087465	-0.222040845	0.091064468	-2.438281898	0.014757261	0.020671571
EPOP	56.52631656	-0.190049629	0.077909855	-2.439352883	0.014713592	0.020611877
TAF4B	840.1157104	-0.103929127	0.04260398	-2.439422938	0.01471074	0.020609356
CHORDC1	2104.358842	0.108094473	0.044292855	2.440449406	0.014669001	0.020552351

RHCG	5.861414053	-0.370572051	0.151841179	-2.440524062	0.014665969	0.020549575
PITPNM3	53.67073388	-0.144062465	0.059020159	-2.440902683	0.014650602	0.020529513
STMN2	1.004070568	-0.61221135	0.250731884	-2.44169724	0.0146184	0.020485856
MAGED4	0.340888956	-0.393486445	0.161084618	-2.442731344	0.014576584	0.020428718
GPR22	171.7448045	0.177482656	0.072642194	2.443244706	0.014555864	0.02040114
AC004687.2	63.08238019	0.0964495	0.039454868	2.444552555	0.014503195	0.020328776
ATXN3	1636.894941	0.068127636	0.027866749	2.444764386	0.01449468	0.020318296
OR5B12	0.967900514	-0.353014761	0.144331673	-2.445857886	0.014450795	0.02025823
CKM	125.1741909	-0.158428109	0.064744761	-2.446964165	0.014406516	0.020197603
NSMAF	2529.422635	0.066394193	0.027126738	2.44755532	0.014382904	0.020165945
USP48	3725.376589	-0.071817574	0.029334948	-2.448191666	0.014357526	0.020131804
OR7E24	0.357630811	-0.482811545	0.197148082	-2.448979163	0.014326174	0.020089282
CSPG4	464.1043984	-0.290591744	0.118630801	-2.449547179	0.014303597	0.02005906
IGBP1P2	3.042539991	-0.250000238	0.102021273	-2.450471661	0.014266919	0.020009058
NAV1	858.4490311	-0.196558593	0.080186057	-2.451281434	0.014234861	0.019965527
ZCCHC3	920.0302609	0.085656069	0.034927079	2.452425747	0.014189666	0.019903564
ARHGAP30	4942.576228	0.092213407	0.037593192	2.452928331	0.014169857	0.019877202
PHOSPHO1	468.7552901	-0.242173886	0.098712813	-2.453317654	0.014154528	0.019857123
APOBEC2	74.03572777	-0.139853585	0.056982399	-2.454329525	0.014114757	0.019802748
SIPA1L2	996.3672644	0.22351211	0.091010676	2.455888922	0.014053659	0.019718442
ISG15	780.9727103	-0.2081642	0.084750562	-2.456198465	0.014041559	0.019702877
TRAPPC8	1578.619035	-0.073459007	0.029906266	-2.456308219	0.01403727	0.019698272
TOMM5	131.6402201	-0.111239202	0.045285547	-2.456395274	0.01403387	0.019694912
FGF13	36.80832097	0.330223551	0.134424465	2.456573288	0.014026919	0.019686568
ATP6V1G3	0.460870896	-0.381571159	0.155277677	-2.457347162	0.013996735	0.019645615
SMAD5	1315.068205	0.093108351	0.037884158	2.457712022	0.013982525	0.019627077
ADO	475.1003279	0.066242071	0.026950593	2.457907719	0.013974908	0.019617792
ADAM15	2843.748174	0.10980335	0.044660119	2.458644364	0.013946269	0.019578994
SH3BP5	259.5585681	0.180782551	0.073522981	2.458857733	0.013937984	0.019568765
CEP152	877.8046052	-0.103105474	0.041920541	-2.459545389	0.013911311	0.019532718
ARMC12	29.45996222	-0.123418523	0.050169913	-2.460010718	0.013893287	0.01950881
SPEG	41.35539777	0.194443324	0.079039882	2.460065953	0.013891149	0.019507207
TMEM37	7.083194552	-0.239544168	0.097366458	-2.460232953	0.013884686	0.019499531
AC068831.6	305.7104808	-0.121225667	0.049264065	-2.460732101	0.013865386	0.019473823
FAN1	2615.74533	-0.095910515	0.038974257	-2.46086832	0.013860123	0.019467828
ABI2	890.5330953	-0.130339077	0.05295135	-2.461487318	0.013836229	0.019435662
TET3	5568.214134	0.076124693	0.030925899	2.461519189	0.013835	0.01943533

HIVEP3	1220.947472	-0.145704383	0.059178304	-2.462125009	0.013811652	0.019403923
DHPS	217.3433919	-0.094452273	0.038330896	-2.464129033	0.013734666	0.019297151
DHX35	847.8795392	0.071305209	0.028932946	2.464498777	0.013720503	0.019278637
MPHOSPH10	426.1063228	0.093838079	0.038070474	2.46485186	0.013706991	0.019261033
CLN3	225.2987244	-0.102158766	0.041444054	-2.464980052	0.013702088	0.019255526
GPT2	899.3864897	0.18265689	0.074093023	2.4652374	0.01369225	0.019243082
AC098588.2	2.295290351	0.176914644	0.071745721	2.465856362	0.013668613	0.019211243
IFT20	566.4563219	0.084709769	0.034347726	2.466240982	0.013653944	0.019192003
RPL23A	8755.046787	-0.221996359	0.08999164	-2.466855345	0.013630541	0.019160484
HTR1B	2.527112095	-0.744212558	0.301541736	-2.468025046	0.013586081	0.019099359
MAMDC2	510.0231102	0.284753282	0.115376663	2.468031887	0.013585822	0.019099359
IGF2	7.824673433	-0.297777156	0.120647955	-2.468149215	0.013581369	0.019095477
ZNF189	519.1853857	-0.101882621	0.041273604	-2.468469231	0.013569232	0.019079783
MATN1	58.41316448	-0.168175897	0.068119043	-2.468852904	0.013554692	0.019060708
FCHO1	882.9840667	-0.09562095	0.03872569	-2.469186483	0.013542063	0.019044316
ARL2-SNX15	0.866806877	0.303828598	0.123045329	2.469241213	0.013539991	0.019042772
MARCHF3	555.3778001	-0.157359239	0.063716009	-2.469697039	0.013522752	0.019019893
ANKRD37	78.31865866	0.138374264	0.056026996	2.469778398	0.013519677	0.019016935
PCDH12	135.9751554	-0.130433191	0.052775365	-2.471478721	0.013455556	0.018928101
RHOU	414.3760268	0.201102743	0.081357493	2.471840467	0.013441949	0.018910318
CAMK2B	27.64974481	-0.195720919	0.079164957	-2.47231763	0.013424019	0.018886452
MUC21	0.587958105	-0.350299698	0.141685421	-2.47237645	0.01342181	0.018884702
ARMCX5-GPRASP2	0.42033757	-0.348188398	0.140823428	-2.472517547	0.013416513	0.018878606
SCAMP2	2623.856936	0.081643668	0.033012051	2.47314742	0.013392888	0.018846718
RPL23	29287.78984	-0.158547463	0.064094862	-2.473637629	0.013374528	0.018822234
GLMP	241.5530975	-0.121934066	0.049277442	-2.474439869	0.013344528	0.018781366
GALNT4	15.05618196	-0.133540576	0.053956877	-2.47495005	0.013325481	0.018755907
SLC25A13	782.0694397	-0.077323124	0.031241625	-2.475003235	0.013323497	0.018754463
ATOH1	0.233444516	-0.620422628	0.250645541	-2.47529888	0.013312472	0.018740291
RSBN1	2043.472346	0.080859527	0.03266499	2.475418694	0.013308006	0.018735352
MINDY2	607.0195713	-0.089340949	0.036085731	-2.475797159	0.013293908	0.018716851
CHRNB1	429.5655723	0.104991666	0.042399341	2.476257015	0.013276796	0.018694104
SPATA22	3.453326775	-0.422066248	0.170413327	-2.476720894	0.013259555	0.01867117
PHACTR1	1506.919897	0.10138393	0.040930552	2.476974387	0.013250141	0.018659257
SFR1	228.1137694	0.079399516	0.032054336	2.477028845	0.013248119	0.018657752
MRPL32	505.7055801	0.082828357	0.033429512	2.477701658	0.013223166	0.01862395
AWAT2	0.321350136	-0.515720151	0.208121517	-2.477976129	0.013212998	0.018610969

NDST1	2712.548926	0.100425791	0.040520231	2.478411094	0.0131969	0.01858963
SCAF4	4708.914603	0.080328121	0.032409073	2.478568948	0.013191061	0.018582744
IL18	990.0318741	-0.143741608	0.057985423	-2.478926594	0.013177842	0.018565458
CCDC62	132.5293741	-0.100833594	0.04067137	-2.479227868	0.013166716	0.018551118
ZNF131	2505.876341	0.065197348	0.02629719	2.479251524	0.013165843	0.018551118
PRPF3	2807.067473	0.081392993	0.032828723	2.479322583	0.01316322	0.018548863
UCHL1	10.45335609	-0.329765689	0.132994093	-2.479551398	0.013154777	0.0185383
PHKA2	2280.179653	-0.086460772	0.034867048	-2.479727351	0.013148288	0.01853049
TRPC5OS	3.331863165	-0.30155867	0.121605376	-2.479813641	0.013145107	0.01852734
HOXD10	0.446238703	-0.67786011	0.273302886	-2.48025229	0.013128945	0.018505894
UBR5	10221.99631	0.103542442	0.041743191	2.480463054	0.013121186	0.018496289
CLEC12A	1772.97762	-0.186154548	0.075029986	-2.481068677	0.013098913	0.018466222
TMEM202	0.662360515	-0.350067318	0.141094128	-2.481090629	0.013098107	0.018466222
ZNF414	569.8481639	-0.095917441	0.038651109	-2.48162199	0.013078593	0.018440233
ZNF641	702.9364001	-0.099803137	0.04021665	-2.481637232	0.013078034	0.018440233
CLDND2	123.2306698	-0.152181468	0.061322395	-2.48166216	0.013077119	0.018440233
UNC5B	231.2582107	-0.254731798	0.102644837	-2.481681555	0.013076408	0.018440233
CAPN6	2.633872517	-0.553633995	0.222973736	-2.482956082	0.013029715	0.018376611
TTC9B	19.5251749	-0.138851337	0.055921102	-2.48298641	0.013028606	0.018376371
ATG9B	42.23546042	-0.160903471	0.064800457	-2.483060767	0.013025886	0.01837386
RAB28	279.4566148	-0.064418366	0.025936016	-2.483741793	0.013001004	0.018340083
CHP2	0.530170832	-0.431858978	0.173753572	-2.485468202	0.012938115	0.018252682
TINCR	86.09027121	-0.170485471	0.068569994	-2.486298456	0.012907966	0.018211463
RFLNA	49.13629216	-0.188523061	0.075813447	-2.486670494	0.012894477	0.018193742
KLK4	0.773981474	-0.404320384	0.162538545	-2.48753539	0.012863165	0.018150871
HARBI1	183.4525326	-0.085621327	0.034412053	-2.488120252	0.01284203	0.018122355
ZNF614	332.2945775	0.09529337	0.038294231	2.488452384	0.012830042	0.018106742
ADPRS	415.734839	-0.067877231	0.027260585	-2.489940351	0.012776454	0.018032415
SBNO1	5147.644233	0.085341038	0.034269582	2.490285374	0.012764056	0.018016216
SAA1	6.492319718	-0.232686529	0.093434037	-2.490382908	0.012760554	0.018012571
PRR11	927.3637356	-0.113669194	0.045635983	-2.490780015	0.012746301	0.017993751
INSM1	4.75571819	-0.267357839	0.107310842	-2.491433617	0.012722874	0.017961974
C15orf61	154.6607201	-0.110773137	0.044460051	-2.491520688	0.012719756	0.017958868
IL12RB1	418.2796233	-0.129167315	0.051837942	-2.491752343	0.012711464	0.017948455
PSMD3	2477.651189	-0.062848504	0.025212877	-2.492714459	0.012677075	0.017901189
TUSC1	48.92341503	-0.334779983	0.134286076	-2.493035708	0.012665611	0.017886292
SFRP2	15.61500839	-0.270679715	0.108431105	-2.49632902	0.012548615	0.017722349

CD47	4967.231086	0.098423271	0.039414914	2.497107351	0.012521105	0.017684773
RNF10	12425.00696	0.09793833	0.039216318	2.497387194	0.012511226	0.017672096
PWP1	951.9285737	0.058272181	0.023331581	2.497566769	0.012504891	0.017664422
WAS	2431.933101	-0.139303405	0.055772239	-2.497719451	0.012499507	0.017658091
TSLP	17.49817781	0.386065538	0.154566765	2.497726708	0.012499251	0.017658091
HCAR2	408.3307844	0.209994874	0.084067067	2.497944581	0.012491572	0.017649429
APOC2	9.235471327	-0.255718316	0.102316093	-2.499297116	0.012443993	0.017583474
CES1	879.145221	0.391739309	0.156703341	2.499878477	0.012423591	0.017555913
BAP1	3975.751555	0.055423716	0.022165235	2.500479551	0.012402529	0.017527416
AGBL1	3.21802944	-0.42935969	0.171627981	-2.501688178	0.012360274	0.017468961
SLC36A1	1282.018543	-0.139452621	0.055742213	-2.501741721	0.012358405	0.01746758
RPP21	23.53820857	0.126875726	0.05069198	2.502875736	0.012318879	0.017412971
C12orf42	44.21480516	0.211884536	0.084639328	2.503381599	0.012301283	0.017389355
MRPL16	1158.467505	-0.097644896	0.038983715	-2.50476118	0.01225341	0.017322932
STX16-NPEPL1	116.2941559	0.115960706	0.046292878	2.504936192	0.012247349	0.017315614
SMARCC1	7481.944298	-0.060333332	0.024064043	-2.50719841	0.012169238	0.017206422
GGA1	3425.996228	0.0756988	0.030187191	2.507646355	0.012153824	0.017185869
CCT4	2467.356512	-0.070242422	0.028005938	-2.508126041	0.012137336	0.017163795
TICAM2	3.2408188	0.224017274	0.089316095	2.508140041	0.012136856	0.017163795
STXBP5	3214.312024	-0.136241926	0.05429952	-2.509081597	0.012104552	0.017119907
LIPA	1758.226891	0.128442186	0.051176401	2.509793261	0.012080186	0.017086681
MTUS1	46.87873067	0.233893657	0.093188588	2.509895927	0.012076675	0.017082948
LIPF	1.625413085	0.370476501	0.147571866	2.510481911	0.01205665	0.017055855
TREML1	35.82974336	0.222259934	0.08852046	2.5108312	0.012044728	0.017040221
TMEM38A	79.48318175	0.209270314	0.083347009	2.510831709	0.01204471	0.017040221
CSTL1	0.810001746	-0.398210409	0.158552689	-2.511533623	0.012020783	0.017008805
LRRC75A	457.7503748	-0.152820319	0.060839564	-2.511857579	0.012009755	0.016994428
STK35	1533.208846	0.077827857	0.030978199	2.512342849	0.011993251	0.016972301
TUG1	3450.27346	-0.054143929	0.021548515	-2.512652492	0.011982731	0.01695864
GGA3	2667.532041	-0.052915486	0.021056637	-2.513007496	0.011970679	0.016942809
YJEFN3	133.2962287	-0.128152	0.050963162	-2.514600662	0.011916728	0.016867668
TFAP2D	0.608117087	-0.879232261	0.349543693	-2.515371552	0.0118907	0.016832044
APOBEC1	0.609044819	-0.471346146	0.187336991	-2.516033509	0.01186839	0.016801678
HTR2C	0.455391093	-0.625468721	0.248566059	-2.516307831	0.011859155	0.016789819
MRPL54	586.2991139	-0.159342819	0.063322412	-2.516373152	0.011856957	0.016787921
C12orf71	42.76079842	0.158313552	0.062905615	2.516683953	0.011846504	0.016774334
POU2F2	1622.237433	0.168451683	0.066910525	2.517566301	0.011816873	0.016733588

RPL32	38176.59114	-0.155823445	0.061880156	-2.518148878	0.011797344	0.016707143
FAM167A	59.78409794	0.23193448	0.092065558	2.519231779	0.011761121	0.016657049
NCBP3	3222.739185	-0.072739995	0.02886805	-2.519740523	0.011744137	0.016634199
RHOB	9300.350091	0.196673554	0.078048835	2.519878168	0.011739546	0.016628899
CHIT1	149.9806228	-0.283242202	0.112376955	-2.520465182	0.011719983	0.016602391
TRRAP	8687.801402	-0.08440014	0.033485092	-2.52052885	0.011717863	0.016600589
TMEM88	101.7621428	0.138038801	0.054765595	2.520538707	0.011717535	0.016600589
METTL21C	28.01529017	-0.269788748	0.106947894	-2.522618623	0.011648466	0.016504663
USP19	2474.143422	-0.071123926	0.028176903	-2.524192489	0.011596441	0.01643214
ANHX	1.331606278	-0.325577019	0.12898251	-2.524195101	0.011596355	0.01643214
NUDT3	155.7404723	-0.117740982	0.046620941	-2.525495627	0.011553522	0.016373694
MBNL1	16323.19746	0.080181822	0.031741677	2.526073933	0.01153452	0.016347948
KCNG3	3.243310356	0.267365505	0.105830621	2.526352979	0.011525362	0.016336151
FCGRT	2725.022344	0.156996077	0.062130423	2.526879257	0.011508106	0.016312874
MARK2	2831.900148	0.06639384	0.026269128	2.527447438	0.011489502	0.016287682
MAPK13	669.7275208	0.142334412	0.056303977	2.527963735	0.01147262	0.016264928
AXIN1	2004.090673	-0.076267537	0.030159472	-2.528808758	0.011445037	0.016226998
SCG2	17.52177428	0.331334554	0.131010512	2.529068453	0.011436572	0.016216171
DDX42	4092.090237	0.065087979	0.025733727	2.529286905	0.011429456	0.016207254
CD36	2724.900922	-0.221719566	0.087632074	-2.530118884	0.011402388	0.016170044
AL031847.1	0.414222219	-0.365420181	0.144406125	-2.530503329	0.0113899	0.016153505
FOXD4L6	0.458151844	-0.340909895	0.134711417	-2.530668162	0.01138455	0.016147086
PTPRQ	1.713412941	-0.357938729	0.141385572	-2.531649615	0.011352737	0.016103133
DDX1	1624.528406	-0.067398749	0.0266161	-2.532254906	0.011333157	0.016076524
BRAT1	2042.871514	0.096552874	0.038121396	2.532773816	0.011316395	0.01605391
UPK3B	30.59448402	-0.162911149	0.064309005	-2.533255643	0.01130085	0.01603302
PC	173.4069389	-0.168543936	0.06646809	-2.535712051	0.011221896	0.015922159
CALR3	2.592255586	-0.297740705	0.117390336	-2.536330632	0.011202091	0.015895211
LEP	6.612078189	-0.295086749	0.116341071	-2.5363936	0.011200077	0.015893505
TSPAN4	711.8573218	-0.123632169	0.048719915	-2.537610514	0.011161212	0.015839502
DDIT4	1339.483272	0.181369707	0.071470201	2.537696902	0.011158458	0.015836741
GRIP2	37.76406462	-0.21801754	0.085867265	-2.539006465	0.011116777	0.015778729
CTXN2	0.36223798	-0.561877311	0.221216978	-2.539937557	0.011087226	0.015737927
C2orf66	12.06866186	-0.354914173	0.139642755	-2.541586732	0.011035056	0.01566501
ZNF804A	121.0523381	-0.24490281	0.096356817	-2.541624129	0.011033876	0.01566447
HOXA4	55.46299931	-0.325543431	0.128084506	-2.541630067	0.011033688	0.01566447
AIRE	20.12805212	0.193306	0.076044201	2.542021574	0.011021337	0.015648939

ASCC1	651.3276818	-0.070823389	0.027842068	-2.543754676	0.010966808	0.015572644
PPM1N	72.4265066	-0.144315562	0.056727548	-2.544011985	0.010958732	0.015562306
ZDHHC6	813.4056277	-0.056713874	0.022286848	-2.544723877	0.010936418	0.015531745
SLC25A34	119.309126	0.137351915	0.05396602	2.545155553	0.010922907	0.015513682
IL26	1.346411791	0.324751874	0.127519519	2.546683645	0.010875197	0.015447042
TUSC2	427.7881541	0.082518819	0.032395803	2.547207062	0.010858898	0.01542501
TRIM64B	0.70567882	-0.776286047	0.304717584	-2.547559078	0.010847948	0.015410574
FIP1L1	592.2219705	0.056430209	0.022146182	2.548078463	0.010831811	0.015388766
RP9	268.5339088	0.123622	0.04851532	2.548102329	0.01083107	0.015388766
DNAJB12	2220.525572	0.065848435	0.025829663	2.549333892	0.010792891	0.0153357
C1QTNF12	55.8248026	0.152198684	0.059674939	2.550462347	0.010758014	0.015287253
H3C4	5.671524276	-0.213519316	0.083703048	-2.550914485	0.010744068	0.015268544
TMEM229A	0.37428927	-0.83469427	0.327188362	-2.551112348	0.010737971	0.015260986
PDLIM5	1122.793225	-0.143057001	0.056041556	-2.552695029	0.010689305	0.015192924
MFN2	4976.953421	0.086473054	0.033869983	2.553088192	0.010677246	0.015176887
NTN1	300.6014199	-0.206747638	0.080976672	-2.553175274	0.010674576	0.015174194
MUC2	5.108715618	-0.306895771	0.120115335	-2.555009081	0.010618503	0.015095581
NAF1	646.8198803	0.059832251	0.023416588	2.555122532	0.010615042	0.015091757
LYPD2	3.550568092	0.387862436	0.151779613	2.555431715	0.010605617	0.015079452
ADGRL3	77.8610169	-0.387467908	0.15162479	-2.555439041	0.010605394	0.015079452
FGF19	0.458331031	-0.459424538	0.179743833	-2.555996106	0.01058843	0.015057203
HNMT	145.283242	0.283361677	0.110854171	2.556166132	0.010583258	0.015050941
H2AZ1	3238.094674	-0.108538819	0.042460589	-2.556224996	0.010581467	0.015049489
EEF1D	2721.496768	-0.071830003	0.028099318	-2.556289865	0.010579495	0.015047777
EIF4A3	2724.401244	-0.116663189	0.045635851	-2.556393425	0.010576346	0.015044392
ADAM10	4023.754473	0.083986692	0.032839726	2.557472368	0.010543593	0.014998892
ACTR1B	2691.089658	0.083793704	0.03275927	2.557862387	0.010531776	0.01498317
NCBP2L	4.410568408	0.264005754	0.10319896	2.558221067	0.010520919	0.014968811
C12orf43	394.7919528	0.062127163	0.024280582	2.558718054	0.010505891	0.014948517
PHACTR4	2876.37616	-0.091631321	0.035803946	-2.55925202	0.010489766	0.014926659
UGP2	1562.502046	-0.095805785	0.037428868	-2.559676292	0.01047697	0.014909535
TRPS1	1720.349531	-0.232228177	0.090724898	-2.559696202	0.01047637	0.014909535
MCM9	1403.111587	0.075055176	0.029320058	2.559857738	0.010471502	0.01490392
RPS15A	4870.723908	-0.144132142	0.056268708	-2.561497274	0.010422206	0.014834836
CPXM1	3006.365757	-0.189820484	0.074086024	-2.562163215	0.010402242	0.014807497
CHAT	1.980867641	0.402974338	0.157270577	2.562299606	0.010398157	0.014802759
TH	0.905310179	-0.311520896	0.121574227	-2.56239258	0.010395374	0.014799873

CISH	546.839871	0.144223087	0.056272304	2.562949746	0.010378707	0.014777219
CDK14	961.2974033	0.180880004	0.070562638	2.563396268	0.010365367	0.014759299
TMEM200B	14.83679673	-0.201505956	0.078587177	-2.564107327	0.010344155	0.014730167
MSANTD1	71.41493106	-0.190470894	0.074280426	-2.564213816	0.010340982	0.01472672
LRRC37A	109.1743146	-0.12037602	0.046929275	-2.565051752	0.010316042	0.014692271
SRPK2	1186.040154	0.073750536	0.028746872	2.565515135	0.010302273	0.014673729
GC	0.441280662	-0.752029895	0.293070214	-2.566040006	0.010286696	0.014652609
RPS6	56415.34535	0.134838161	0.052546797	2.566058625	0.010286144	0.014652609
GHITM	3285.723659	0.077331016	0.030130435	2.566541637	0.010271829	0.014633562
MAOB	6.675276782	-0.357086689	0.139101915	-2.567086803	0.010255694	0.014611639
EBF4	80.16453346	-0.284347902	0.110687911	-2.568915599	0.01020173	0.014535813
RETREG3	1433.695792	-0.092137663	0.035859275	-2.56942346	0.010186789	0.014515581
ASTN1	3.972013335	-0.456743694	0.177760701	-2.569430085	0.010186594	0.014515581
OR1G1	0.603336303	-0.402152773	0.156510486	-2.569494107	0.010184712	0.014514735
IPO7	3922.63332	-0.078081879	0.030384152	-2.569822554	0.010175062	0.014502038
C11orf52	0.582174889	-0.342572076	0.133304782	-2.569840867	0.010174524	0.014502038
ZNF787	1208.214018	0.079812102	0.031055352	2.569995059	0.010169997	0.01449693
GLMN	346.4912497	-0.088619528	0.034477697	-2.570343593	0.010159769	0.014483406
FEZ1	120.2723393	0.245050837	0.095330294	2.570545272	0.010153855	0.01447603
CPPED1	1497.144696	0.131168784	0.051025716	2.570640711	0.010151058	0.014473096
SPATS1	5.302094217	-0.220509476	0.085759072	-2.571267046	0.010132716	0.014447997
LAMC3	128.2434612	0.290276838	0.112890987	2.571302156	0.010131689	0.014447584
CDKN2AIPNL	430.1943122	-0.081365379	0.031643373	-2.571324505	0.010131035	0.014447584
C1QTNF9B	0.984841176	-0.325629368	0.126584408	-2.57242872	0.010098775	0.014402749
DMRT1	2.21036115	-0.310619961	0.12071126	-2.573247596	0.01007491	0.01436976
TWIST2	4.514791156	-0.334682882	0.130060914	-2.573277954	0.010074027	0.014369547
DCUN1D1	2412.775736	0.111354292	0.043270453	2.573448742	0.010069056	0.014363504
PHETA1	199.2694743	-0.13444533	0.052241101	-2.573554665	0.010065974	0.014360154
CD99	10490.76924	0.172792518	0.067138507	2.57367232	0.010062552	0.014356319
LOXL3	497.1851458	-0.137319774	0.053354454	-2.573726523	0.010060976	0.014355117
FBXO45	566.6640639	0.09375091	0.03642105	2.574085857	0.010050533	0.014341261
PNMA5	26.73981134	-0.240547783	0.093436133	-2.574462101	0.010039608	0.014326717
ZCRB1	959.703732	0.120368133	0.046752991	2.574554688	0.010036922	0.014323928
C8orf58	216.9299966	-0.08251277	0.032046771	-2.574760801	0.010030943	0.014316439
ENOPH1	621.4744275	-0.066318418	0.025753887	-2.57508382	0.01002158	0.014304119
PPP2R1B	1105.691418	-0.087767064	0.034064597	-2.576489161	0.009980933	0.014247142
NIPSNAP3A	1082.863006	-0.099666374	0.038668438	-2.57746054	0.009952924	0.014208197

TCERG1	4201.878282	0.071541628	0.027754961	2.577615843	0.009948453	0.01420285
MYH15	44.30647185	-0.220499579	0.085538924	-2.577768916	0.009944047	0.014197596
LIF	100.3037967	0.231912013	0.089940074	2.578517019	0.009922541	0.014167924
TWF2	724.2007322	-0.101238437	0.039234965	-2.58031163	0.009871119	0.014095529
RPS25	16113.81541	-0.198117452	0.076764538	-2.580846035	0.009855853	0.014074756
CNR1	25.0517254	0.255999296	0.099148161	2.581987329	0.009823319	0.01402932
DUSP13	34.8142558	-0.296772694	0.114930493	-2.582192817	0.009817472	0.014021992
LGALS14	1.521501716	-0.453870027	0.175766449	-2.582233579	0.009816312	0.014021359
SI	0.749617326	-0.869359908	0.336668984	-2.58223938	0.009816147	0.014021359
MPHOSPH6	189.0711531	-0.141954721	0.054965256	-2.582626389	0.009805144	0.014007451
COX16	65.18742366	-0.096230512	0.037250353	-2.583344929	0.009784744	0.013979328
HSPBAP1	661.3503323	-0.103662544	0.040125128	-2.583481938	0.009780859	0.013974797
MME	176.1457211	-0.311709681	0.12063427	-2.583923144	0.009768356	0.013957952
CSNKA2IP	0.51099	-0.595237723	0.230333124	-2.584247163	0.009759183	0.013945863
SLC35A1	89.97177892	-0.112099061	0.043365429	-2.584986783	0.009738273	0.013916999
EIF5AL1	73.98279476	-0.189065742	0.073127892	-2.585412162	0.009726265	0.013900853
TM9SF4	2176.298625	-0.052585487	0.020337959	-2.585583236	0.00972144	0.013894971
MFSD14B	1196.305761	-0.094507564	0.036547864	-2.585857353	0.009713712	0.01388494
DNM2	8663.358558	0.061512099	0.023779226	2.586799863	0.009687184	0.013848032
PEX19	740.8261543	0.063123764	0.02440191	2.586837051	0.009686139	0.013847549
GJA9	1.698505888	-0.210547272	0.081376757	-2.587314621	0.009672723	0.013829379
KCNJ15	68.1773443	-0.284554815	0.109971784	-2.587525682	0.009666799	0.013821919
TMEM92	38.89164786	-0.208476075	0.080568812	-2.587553041	0.009666031	0.013821832
SLC7A6OS	393.3454841	0.079692894	0.030794687	2.587878043	0.009656916	0.013809807
PSMC3IP	170.369095	0.092510623	0.035726163	2.58943628	0.009613321	0.013748468
THAP4	1126.297395	0.099491016	0.038421627	2.589453507	0.00961284	0.013748468
LSAMP	21.75245341	0.43220143	0.166908045	2.589458341	0.009612705	0.013748468
CACYBP	1330.01438	-0.104460628	0.040336652	-2.589719868	0.009605406	0.01374016
XRCC3	851.6919585	-0.083027361	0.03204592	-2.59088711	0.009572889	0.013694646
METTL22	1019.894068	-0.072437051	0.027947066	-2.591937629	0.009543707	0.013653898
DSC2	407.9070209	0.327924537	0.126496321	2.592364226	0.00953188	0.013637974
BIRC6	16564.5765	0.115936266	0.04471716	2.592657161	0.009523766	0.013627361
AC069288.1	0.665515323	-0.281328229	0.108490301	-2.593118714	0.009510993	0.01361008
RGS19	1308.084314	-0.14780111	0.056993073	-2.593317093	0.009505509	0.013603226
FAM71B	0.731084155	-0.340696786	0.131340545	-2.593995527	0.009486772	0.013577405
SLC22A6	1.767597806	-0.390409755	0.150476542	-2.594489139	0.009473161	0.013558916
FUCA1	311.7581747	-0.148061825	0.057065128	-2.594611277	0.009469795	0.01355509

GKN1	1.191508764	0.533463025	0.205586191	2.594838797	0.009463529	0.013547112
PBX3	2273.588679	-0.219977298	0.084743065	-2.595814757	0.009436693	0.013509683
IARS2	1520.796946	-0.090193583	0.034744896	-2.595880045	0.0094349	0.013508104
TRIT1	607.8242165	-0.098055997	0.037770679	-2.596087751	0.009429198	0.013500929
OSCAR	822.5741079	0.209557082	0.080716946	2.596196877	0.009426204	0.013497629
ELP2	3044.533703	0.082608246	0.031817547	2.596310919	0.009423075	0.013494136
FAU	6687.378125	-0.151029333	0.058162348	-2.596685638	0.009412803	0.013480412
C5orf22	467.7845525	0.082227274	0.031647038	2.598261296	0.009369716	0.013419688
GSTA1	0.426627352	-0.820872213	0.315854838	-2.598890734	0.009352554	0.013396087
KCNN1	68.55021823	0.221173649	0.085101325	2.598944848	0.00935108	0.013394956
FNDC3A	3164.076155	0.087130586	0.033523242	2.599109782	0.009346588	0.013389502
B4GALT7	723.2121871	-0.068126468	0.026198735	-2.600372464	0.009312263	0.013341306
REPS1	2601.459862	0.069316579	0.026653709	2.600635361	0.00930513	0.013332063
ARSA	1006.530792	-0.128517829	0.049401684	-2.601486818	0.009282063	0.013299987
PWWP3B	0.472046912	-1.009901495	0.388180296	-2.601629975	0.00927819	0.013295411
NLRP6	114.4573902	0.203407102	0.078166209	2.602238303	0.009261747	0.01327282
METTL11B	0.599018268	-0.606382854	0.233023459	-2.602239518	0.009261714	0.01327282
CSF3R	14732.6092	0.142681449	0.05482382	2.602544833	0.009253472	0.013262903
RAB13	151.9617301	0.21360852	0.082070532	2.602743208	0.009248119	0.013256202
PSG9	0.530256593	-0.610052698	0.234325903	-2.603436878	0.009229426	0.013230376
LHFPL2	895.3334244	0.166343601	0.063887389	2.603700098	0.009222342	0.013221189
FASTKD2	889.8606044	-0.065913805	0.025312431	-2.604009228	0.009214028	0.013210237
CLTA	1562.839623	-0.09450817	0.03629327	-2.60401361	0.00921391	0.013210237
AC098650.1	1.549576229	-0.422124972	0.162088743	-2.604283093	0.009206668	0.013201619
PIANP	11.26637631	-0.165973364	0.063723394	-2.604590748	0.009198406	0.013190739
CCDC32	368.2326095	-0.078759681	0.03023722	-2.604726257	0.009194769	0.01318649
NOTCH2NLR	4.403486257	-0.236967357	0.090968409	-2.604941197	0.009189003	0.013179186
OR8G1	0.934355236	0.428355395	0.164351814	2.606332018	0.00915177	0.013126748
KCNJ5	29.11093735	-0.209945495	0.080536497	-2.606836673	0.009138294	0.013108378
FPR3	20.72767515	-0.199605388	0.076541444	-2.607807973	0.009112406	0.013072202
ZBED3	660.0981919	-0.138831243	0.053236486	-2.60782131	0.009112051	0.013072202
IPMK	541.8188262	0.107392639	0.041165009	2.608833088	0.009085154	0.013035019
NBPF14	536.3951273	-0.112368557	0.043044445	-2.610524025	0.009040362	0.012971704
ETV3L	3.05176182	-0.314622621	0.120508309	-2.610796082	0.009033174	0.01296234
RAB8A	1505.600371	-0.06612148	0.025324149	-2.611004986	0.009027658	0.012955374
CTAGE6	1.237380711	0.299128508	0.114552374	2.611281618	0.009020358	0.012945847
ABI3	144.276336	0.196360423	0.075194418	2.61136968	0.009018035	0.012943463

CATSPER1	99.78096476	0.207404258	0.079420646	2.611465262	0.009015515	0.012940794
MBOAT7	6067.436461	0.118792977	0.045473544	2.61235363	0.008992119	0.012908159
ZNF770	880.7778837	-0.094958782	0.036349337	-2.612393776	0.008991063	0.012907589
XPC	654.9017272	-0.072114637	0.027593526	-2.613462174	0.008963	0.012868247
NKAP	509.0769563	0.077154884	0.029521396	2.613524283	0.008961372	0.012866852
GRM8	1.901109373	-0.290269746	0.111039798	-2.614105488	0.008946141	0.012845926
CDH24	201.5540149	0.173791007	0.066453892	2.615211873	0.008917212	0.012805326
STAG1	1873.792781	-0.07617566	0.029121559	-2.61578233	0.008902329	0.012784891
SF3A2	2731.09335	-0.143812694	0.054971852	-2.616115153	0.008893656	0.012773373
GPKOW	400.8091238	-0.061545247	0.023523628	-2.616316114	0.008888423	0.012766794
INO80C	393.9872047	-0.090155821	0.034455132	-2.616615175	0.00888064	0.012756551
CD7	670.6853931	-0.215447884	0.082335473	-2.616707927	0.008878227	0.012754021
TTPAL	818.1678919	-0.114674425	0.043823537	-2.616731401	0.008877617	0.012754021
ATP6V1FNB	74.34499675	0.173863386	0.066442335	2.61675613	0.008876974	0.012754021
HMGB4	0.312847228	-0.766874066	0.293043996	-2.616924686	0.008872592	0.012748733
CWF19L2	574.3456757	-0.09690151	0.037017748	-2.61770409	0.008852353	0.012720587
SLC5A7	0.582740226	-0.411878517	0.157314473	-2.618185776	0.008839866	0.012703576
HRK	29.11796608	-0.259503083	0.099089235	-2.618882692	0.008821828	0.012678584
DHX34	1200.971393	-0.113470806	0.043309021	-2.620027069	0.008792279	0.012637045
ZNF441	121.2718412	-0.142855746	0.054519691	-2.620259626	0.008786285	0.012629357
CCDC144A	230.9678478	-0.308862502	0.117812383	-2.621647192	0.008750597	0.012578983
ITGAX	6024.202746	0.180771997	0.068928727	2.622593004	0.008726345	0.012545042
METTL18	59.19052613	0.12606909	0.048056594	2.623346357	0.008707071	0.012518253
SSU72P2	0.293425	-0.718178267	0.273674065	-2.624210177	0.008685017	0.012487464
LRRC70	50.93154235	0.144176034	0.054931788	2.624637566	0.008674124	0.012472718
HSPH1	2851.425193	-0.186900823	0.071207899	-2.624720357	0.008672016	0.012470602
NMI	1125.695317	-0.101653974	0.038727505	-2.624852167	0.00866866	0.012466692
NSUN3	724.3655553	-0.089645144	0.034152242	-2.624868467	0.008668245	0.012466692
NPIPA9	3.176737014	-0.200685339	0.076430598	-2.625719844	0.008646595	0.012436788
TMEM106B	834.5709597	0.102411766	0.039001712	2.625827428	0.008643863	0.012433771
CPE	6.600579413	-0.323038876	0.123012278	-2.626070188	0.0086377	0.01242582
GPC4	96.84628354	0.395258302	0.150490935	2.626459212	0.008627833	0.012412538
FSD2	18.26204918	-0.163944283	0.062419857	-2.626476452	0.008627396	0.012412538
TNFAIP6	119.3714114	0.254852314	0.097019632	2.626811793	0.008618899	0.012401508
HTR3D	0.924351567	-0.408090879	0.155345344	-2.626991376	0.008614352	0.012395876
GPN1	742.6708492	0.065651405	0.024980934	2.628060436	0.008587326	0.012357895
P2RX5-TAX1BP3	96.6154541	-0.134596307	0.051212976	-2.628168065	0.008584609	0.012354894

C2CD4B	46.46144461	0.482561369	0.18358254	2.628579873	0.008574222	0.012340852
BRF2	215.6130126	0.079509515	0.030243777	2.628954546	0.008564781	0.01232817
HOXA7	107.7617434	-0.40699701	0.154805951	-2.629078589	0.008561657	0.01232458
TNFRSF11B	2.103680055	-0.358665526	0.136402544	-2.629463601	0.008551969	0.012311539
PDCD1LG2	13.46063597	-0.189375179	0.072016006	-2.629626254	0.008547879	0.012306556
XKRX	10.09952975	-0.200339268	0.076183737	-2.629685507	0.008546389	0.012305316
PDXK	2556.662208	0.112082072	0.042613343	2.630210747	0.008533195	0.012287223
RHOD	1.577792058	-0.264716536	0.100621626	-2.630811559	0.008518125	0.012266426
TM6SF1	110.4325904	0.188164258	0.071509165	2.631330656	0.008505124	0.012248605
MTRNR2L7	0.923196486	-0.6594965	0.250582924	-2.631849327	0.008492151	0.012230823
NR4A2	2913.133683	0.174365484	0.066248309	2.631999003	0.008488411	0.012226335
UGT2B4	0.657630808	-0.555211067	0.210945426	-2.632012822	0.008488066	0.012226335
ZCCHC2	2546.877841	0.127113782	0.048293153	2.632128438	0.008485178	0.012223477
SPESP1	43.43325414	-0.141436139	0.053715614	-2.633054485	0.008462077	0.012191096
ARHGAP10	612.3994233	-0.198803978	0.075493385	-2.633396001	0.008453572	0.01217974
PLEKHH3	317.6578962	-0.204800835	0.077758037	-2.633822079	0.008442972	0.012165363
MYZAP	5.58217158	-0.332512378	0.126245267	-2.633860161	0.008442025	0.012164894
TSBP1	14.00430465	-0.287842582	0.109273582	-2.634146112	0.008434918	0.012155548
NETO1	19.91016895	0.339414365	0.128844596	2.634292591	0.00843128	0.012151199
NUDT13	502.1315751	0.123260521	0.0467765	2.635095007	0.008411374	0.012123403
KCNH3	110.3697366	0.172715634	0.06552372	2.635925347	0.008390819	0.012094668
MAPKAPK3	5307.254757	0.093665606	0.035518099	2.637123299	0.008361243	0.012052925
TXNDC17	316.6159632	-0.141098272	0.053501488	-2.637277513	0.008357443	0.012048334
OR2J2	0.345516628	-0.442572766	0.167813925	-2.637282723	0.008357315	0.012048334
PSMB1	3218.516308	0.117738421	0.044643717	2.637289835	0.008357139	0.012048334
POPDC3	3.766765179	-0.689091079	0.261281449	-2.637351717	0.008355615	0.012048334
EPN3	8.693496529	-0.213909884	0.08108691	-2.638032246	0.008338865	0.012025095
WASHC5	1868.330431	0.064299376	0.024362306	2.6392976	0.008307802	0.011981182
MBTD1	1955.736072	-0.079375029	0.030072086	-2.63949194	0.00830304	0.011975197
SMCO4	86.41880138	0.195523929	0.074059288	2.640100036	0.008288156	0.011954611
PPP2R2B	21.40844564	0.216191188	0.0818672	2.640754641	0.00827216	0.011932419
EFCAB3	1.459653979	-0.306549451	0.116068018	-2.641119015	0.008263269	0.011920471
ARHGAP11A	789.7334199	-0.135002345	0.051113543	-2.641224552	0.008260695	0.011917637
WNT10A	23.15868271	0.191732643	0.072587406	2.641403706	0.008256327	0.011912214
PRSS21	706.0371674	-0.349301325	0.132240398	-2.641411633	0.008256134	0.011912214
RNF187	1923.881126	-0.109031107	0.041276274	-2.641495848	0.008254082	0.01191073
VPS37A	1245.49982	0.081027471	0.030669996	2.641913292	0.008243916	0.011896938

FKBP15	2087.561854	0.104569809	0.039580788	2.641933482	0.008243424	0.011896938
DLGAP4	1371.420031	0.102436807	0.038768789	2.642249354	0.00823574	0.011886891
LGALS12	450.6948341	0.229513841	0.0868478	2.642713344	0.008224463	0.011871491
GSTO1	1682.286103	0.145807004	0.055130518	2.644760269	0.008174881	0.011800793
RBM34	228.3047026	0.091904514	0.034742526	2.64530313	0.008161776	0.011782745
GFAP	23.44894304	-0.180994705	0.068382943	-2.646781469	0.008126185	0.011732228
ZNF280C	368.9393437	-0.079822612	0.030155311	-2.647049844	0.008119738	0.011723786
SSTR2	65.6907521	-0.229044885	0.086526683	-2.647101172	0.008118506	0.011722871
UCP2	6350.417639	-0.146996593	0.055520391	-2.647614518	0.00810619	0.011705951
CRIPT	1654.935398	0.095790367	0.036158054	2.649212469	0.008067959	0.011651602
ANO7	314.4250797	-0.264797169	0.099941136	-2.649531298	0.008060351	0.011641473
UXT	1460.389678	-0.153841304	0.058049138	-2.650191033	0.008044627	0.011619621
ZNF383	407.6357198	-0.091334218	0.034454976	-2.650828118	0.008029469	0.011598583
VCX3B	0.390320045	-0.455635423	0.17185403	-2.65129321	0.00801842	0.011583477
C20orf202	4.194413532	-0.245425156	0.092566634	-2.651334998	0.008017428	0.011582899
NPTXR	59.94796019	-0.222680516	0.083934848	-2.653016255	0.007977603	0.011526214
IRF5	1900.178256	-0.111933887	0.042189709	-2.653108769	0.007975417	0.011523906
SEL1L3	1683.281721	-0.197545024	0.074448935	-2.653429828	0.007967834	0.011513799
LRIG1	344.9195629	0.168943164	0.0636677	2.653514495	0.007965835	0.011511761
PHLPP2	600.5748431	-0.169670529	0.063920163	-2.654413279	0.007944646	0.011481987
SLC30A8	2.619364981	-0.40793596	0.153563327	-2.656467323	0.00789641	0.011413118
IRAG2	2693.88394	0.132804872	0.049986181	2.656831719	0.007887881	0.011401631
LAMA5	726.7941957	-0.217943928	0.082018159	-2.657264319	0.007877765	0.011387851
IL36A	1.5103515	-0.332773631	0.125218711	-2.657539191	0.007871344	0.011379409
EXOSC1	527.6971687	-0.077843683	0.029288067	-2.657863428	0.007863775	0.011369307
PSTK	363.8442981	0.086598454	0.032572295	2.658653742	0.007845355	0.011343512
RNGTT	937.9651411	-0.085145837	0.032008501	-2.660100712	0.007811729	0.011295728
C1QTNF9	1.582678172	0.248872418	0.093529477	2.660898211	0.007793251	0.011269842
PCNX2	523.0241094	-0.186636337	0.070127173	-2.661398251	0.007781686	0.011253948
GALNT12	64.21528716	-0.180402026	0.067783237	-2.66145488	0.007780377	0.011252887
RTL5	136.9244211	-0.191560112	0.071975485	-2.661463289	0.007780182	0.011252887
SELE	13.57277828	-0.341871793	0.128413781	-2.662267168	0.007761625	0.011227424
YEATS4	538.0570356	0.097373817	0.036570618	2.662624297	0.007753393	0.011216346
GNAT3	0.341771644	-0.649248215	0.243780893	-2.663244881	0.007739107	0.011196507
HIPK4	22.54397054	-0.203694747	0.076476035	-2.663510837	0.007732992	0.011188487
SLC22A8	3.246928129	-0.30292681	0.113718432	-2.663832111	0.007725611	0.011178634
LINC02218	2.586870637	-0.400790553	0.150436761	-2.664179618	0.007717634	0.011167918

CCNDBP1	2610.905318	-0.099942081	0.037505506	-2.664730928	0.007704994	0.011150452
TMPRSS13	68.95490964	-0.178767728	0.067084245	-2.664824332	0.007702855	0.011148179
REM2	169.3671014	0.171532694	0.064354134	2.665449508	0.007688548	0.011128296
ELP5	888.563799	-0.095206459	0.035710097	-2.666093544	0.007673834	0.01110782
JPH1	7.394307203	-0.377709829	0.14166773	-2.666167019	0.007672157	0.011106214
AKAP7	307.7634772	-0.14430059	0.054122806	-2.666169771	0.007672094	0.011106214
GPSM3	3115.551445	0.13410484	0.050275152	2.667417878	0.007643657	0.011066595
KIAA0895	186.7104366	0.152007707	0.05698615	2.667450032	0.007642925	0.011066354
OR52I2	8.187260159	-0.235005223	0.088028977	-2.669634842	0.007593378	0.010995427
KCNA4	0.557753948	-0.695366907	0.260472217	-2.669639453	0.007593273	0.010995427
SNX5	3149.224831	-0.072545135	0.027167979	-2.670244085	0.007579613	0.010977119
KLF2	7762.732832	0.206180484	0.077196328	2.670858683	0.007565749	0.010957852
ATP6V0E1	1398.787813	-0.139404097	0.052192051	-2.670983322	0.00756294	0.010954594
FABP4	16.16215684	-0.379955485	0.142252897	-2.670985917	0.007562882	0.010954594
ADSS2	1087.108121	-0.080700853	0.030210147	-2.671316176	0.007555444	0.010945356
ZNF823	97.38079065	0.136037329	0.050914769	2.671863794	0.007543126	0.01092832
KLHL6	2255.412462	-0.105439816	0.039460748	-2.672017673	0.007539668	0.010924119
P2RY2	1190.920553	0.221654399	0.082935628	2.672607709	0.007526421	0.010905733
ANKRD28	10990.70576	0.121735511	0.045542976	2.672981031	0.00751805	0.01089441
CD207	2.065185388	-0.409154469	0.153069598	-2.672996306	0.007517708	0.01089441
NEXMIF	5.430623824	-0.255024129	0.095398933	-2.673238801	0.007512275	0.010887654
GSC	2.775442002	-0.424702063	0.158847046	-2.67365415	0.007502978	0.010874985
SNX21	157.4600996	0.118299177	0.044237416	2.674188219	0.00749104	0.010858485
PSAPL1	0.596617081	-0.38690987	0.144520214	-2.677202443	0.007423976	0.010762071
NLRP2	218.9175312	-0.260339978	0.097238847	-2.677324815	0.007421265	0.010758938
DCLRE1B	336.3477644	-0.092275912	0.034463514	-2.677495717	0.00741748	0.010754247
LMF2	1644.051734	-0.074959769	0.027987817	-2.678299998	0.007399691	0.01072925
CHPF2	2765.17754	0.094149831	0.035147124	2.678735035	0.007390085	0.010716116
NACA2	36.01264711	-0.240729179	0.089794744	-2.680882727	0.007342824	0.010648374
DDX52	2065.669888	0.085603641	0.031928697	2.681087811	0.007338326	0.010642638
CDAN1	1271.695392	-0.094995824	0.035431814	-2.681088343	0.007338314	0.010642638
NCAPH2	1708.823308	-0.078037976	0.029105561	-2.681205015	0.007335756	0.010640488
UHRF2	5493.931659	0.085759473	0.031981953	2.681495782	0.007329384	0.010632034
PSMD2	4878.830541	0.063203924	0.023563793	2.682247524	0.007312933	0.010608956
BRIX1	629.8003656	0.085552563	0.031892421	2.68253589	0.007306631	0.0106006
SMIM19	380.4549348	-0.115404596	0.043019065	-2.682638418	0.007304392	0.010598136
GGTLC2	0.992788027	-0.326647612	0.121763165	-2.682647192	0.0073042	0.010598136

PTCHD1	4.944795489	-0.250885309	0.093520473	-2.682677914	0.007303529	0.010598136
TMC3	27.99210056	-0.235474697	0.087745079	-2.68362281	0.007282922	0.010569336
HEATR9	50.92585115	0.22101183	0.082351133	2.683774003	0.00727963	0.010565341
TOPORS	1853.382545	0.1161559	0.043277044	2.684007278	0.007274552	0.010558755
AMN1	159.5303053	0.129338026	0.048186687	2.684102911	0.007272472	0.010556518
ZNF266	2021.930228	0.087159819	0.032459485	2.685187997	0.007248902	0.010523085
ENSA	3371.068569	0.074765293	0.027829239	2.68657339	0.007218909	0.010480322
OR1E2	0.328580283	-0.752792445	0.280154265	-2.68706402	0.007208314	0.010465716
JAML	693.5799004	-0.234162251	0.087139052	-2.687225139	0.007204837	0.010461445
INHBA	367.8972746	0.278769013	0.103738602	2.687225461	0.00720483	0.010461445
KRTAP10-4	0.316051967	-0.623345604	0.231910109	-2.687875951	0.00719081	0.010442627
CSNK2A3	8.643896862	0.12171683	0.045282931	2.687918582	0.007189893	0.010442069
RNF217	465.0155268	-0.264846665	0.098528604	-2.688018048	0.007187751	0.010439734
KDM5A	7287.630869	0.098502797	0.036639382	2.688440478	0.007178663	0.010427308
NOL3	52.55352852	-0.14333927	0.053311284	-2.688722914	0.007172593	0.010419264
RUNDC3B	32.95214017	-0.220715519	0.082079451	-2.68904723	0.007165628	0.010409919
CT45A5	4.201280123	-0.68460865	0.254557943	-2.689402041	0.007158015	0.010399631
DUSP2	2226.759753	0.178616262	0.066386024	2.690570248	0.007133002	0.010364059
GATC	680.6358828	-0.071245782	0.026478738	-2.690678916	0.007130679	0.010361453
INHBE	20.20052978	-0.237178453	0.088138245	-2.690982256	0.007124198	0.010352805
SIGLEC11	17.64106961	0.251937311	0.093603327	2.691542257	0.007112249	0.010336208
STIM2	1033.632126	0.110255591	0.040957663	2.691940484	0.007103762	0.010324641
RAB6B	341.6279312	-0.195892067	0.072760086	-2.692301193	0.007096083	0.010314245
TMEM251	72.21273397	-0.119067374	0.044217595	-2.692760051	0.007086325	0.010300827
UGT2A3	0.62568712	-0.538494633	0.199945907	-2.693201577	0.007076947	0.010287959
HOXA2	93.14173542	-0.284451929	0.105580946	-2.694159685	0.007056635	0.010259193
PRLR	305.0670564	0.321168321	0.119180898	2.694796944	0.007043154	0.010240354
CBLL2	1.01248226	-0.509281137	0.188977322	-2.694932558	0.007040288	0.010236948
ABTB1	1972.888298	-0.11468489	0.042552947	-2.695110394	0.007036531	0.010232246
IFNA2	0.507308637	-0.573025792	0.212606219	-2.695244737	0.007033695	0.010228881
KIF18A	441.6477781	-0.130379828	0.048352059	-2.69646896	0.007007893	0.010192115
MUC20	62.31165983	-0.192650642	0.071442385	-2.696587485	0.007005399	0.010189246
PRR7	599.9488393	0.127906123	0.047390606	2.698976331	0.006955312	0.010117147
KLHL12	784.8375949	-0.071451059	0.026459744	-2.700368513	0.006926271	0.010075652
KIDINS220	3600.255487	-0.081078076	0.030024154	-2.700428366	0.006925025	0.010074588
SFXN5	944.7536211	-0.103300811	0.038248881	-2.700753815	0.006918253	0.010065484
TM4SF1	173.0252751	0.387444977	0.143456994	2.700774408	0.006917824	0.010065484

CLDN24	1.019101274	-0.508986804	0.188390832	-2.701759946	0.006897354	0.010036571
SLC22A1	27.45514952	-0.27959503	0.103443977	-2.702864285	0.006874481	0.010004031
DSTN	1448.341198	0.148836896	0.055065172	2.702922589	0.006873275	0.01000302
PAGE2B	6.277708029	-0.272219422	0.100699108	-2.70329528	0.006865573	0.009992553
APOC4-APOC2	1.80965769	-0.303649666	0.112317772	-2.703487267	0.006861608	0.009987525
OR52N4	17.52850175	0.214944703	0.079505911	2.703505925	0.006861222	0.009987525
TPBGL	6.150262564	-0.226484367	0.083752548	-2.70420867	0.006846728	0.009967349
MOSPD1	202.5812278	-0.145868309	0.053935608	-2.704489938	0.006840934	0.009959655
SLC9A7	2642.384345	-0.125062867	0.046240272	-2.704630906	0.006838032	0.009956171
RIF1	5466.869847	0.100399018	0.037120825	2.70465479	0.006837541	0.009956171
MAP3K13	442.6436796	-0.11116176	0.041092681	-2.705147432	0.006827408	0.009942181
AC091057.5	24.39700395	-0.183123855	0.067673916	-2.705973973	0.006810438	0.009918208
CALHM4	0.63494622	-0.436326034	0.16119717	-2.706784708	0.00679383	0.009894757
ZFP36	16969.96342	0.212095519	0.07835641	2.706804973	0.006793415	0.009894757
TMEM11	884.1455609	0.090871024	0.03356918	2.70697778	0.00678988	0.009890476
KCP	143.8021722	-0.181314743	0.066954481	-2.708029999	0.00676839	0.009859907
SMIM38	27.13609381	-0.232308789	0.085773158	-2.708408954	0.006760666	0.009849387
TAS2R43	34.3960954	-0.242663362	0.089573469	-2.709098624	0.006746628	0.009829667
NRXN3	29.70251722	-0.281215651	0.103803991	-2.709102492	0.00674655	0.009829667
CNPY4	172.8390163	-0.100199282	0.036979417	-2.709596053	0.00673652	0.009816401
DERA	547.7217309	-0.107160357	0.039547262	-2.709678289	0.00673485	0.009814698
SLC1A1	7.97078055	-0.269879597	0.099586223	-2.71000938	0.006728131	0.009805637
KRT12	0.735771962	-0.369897512	0.136466421	-2.710538674	0.006717401	0.009790729
GOLGA6L6	0.636840952	-0.335126099	0.123595011	-2.711485645	0.006698244	0.009763534
LRCH1	1757.16523	0.105527112	0.038885046	2.713822448	0.00665118	0.009695655
HDHD5	653.5372805	-0.147961843	0.0545021	-2.714791608	0.006631749	0.009668048
CHML	1229.588696	0.118636006	0.043694325	2.715135342	0.006624869	0.009658738
ANKRD29	11.35681003	-0.225909626	0.083182897	-2.715818196	0.006611221	0.009639558
HTD2	46.43090677	0.135281874	0.04979919	2.716547671	0.006596669	0.009619058
ZNF254	1100.010422	0.117798486	0.043361693	2.716648674	0.006594657	0.009616839
PRSS3	6.981209967	0.238966904	0.087936386	2.717497441	0.006577766	0.009592923
APPL2	1499.166899	0.099231945	0.036512343	2.717764335	0.006572463	0.009585903
AKTIP	265.0140791	0.15400818	0.056663414	2.717947423	0.006568828	0.009581315
ZNF394	4297.627772	0.080928498	0.029775242	2.717979513	0.00656819	0.009581099
MEOX1	4.48744595	0.271605901	0.099912146	2.718447265	0.006558911	0.009568276
TXNDC9	340.9513706	0.071694602	0.026372529	2.71853345	0.006557203	0.009566497
CLIP4	479.0088729	0.196475481	0.072255564	2.719174412	0.006544509	0.00954869

VRTN	1.440582754	-0.283351661	0.104199848	-2.71930973	0.006541832	0.009545496
YWHAG	4602.483667	-0.089252696	0.032820538	-2.71941603	0.00653973	0.009543139
GRIP1	146.0103756	-0.21030664	0.077329907	-2.719602927	0.006536035	0.009538459
CLPSL2	2.305960865	-0.411149646	0.151175154	-2.719690603	0.006534302	0.009536642
MTHFS	65.50443482	-0.143985018	0.052932296	-2.720173299	0.006524771	0.009523441
XIRP1	113.9636564	0.333073965	0.122434338	2.720429347	0.00651972	0.009516779
CENPN	305.0547568	-0.090736943	0.033340449	-2.721527319	0.006498101	0.009485929
AC113348.2	0.535632995	-0.469047922	0.172337547	-2.721681551	0.00649507	0.009482211
TMEM247	0.399871492	-0.425385486	0.156277343	-2.721990772	0.006488995	0.00947405
SPDYE17	8.625547269	-0.156065595	0.05732377	-2.722528454	0.006478445	0.009459352
PTCH1	174.6450465	-0.288115101	0.105762737	-2.724164568	0.006446438	0.009413319
DOP1A	1135.111059	-0.126138338	0.046291315	-2.724881299	0.006432461	0.00939361
OBI1	142.0174871	-0.12029344	0.044137501	-2.725424814	0.00642188	0.009378858
MRPS15	1190.117906	-0.105403369	0.038668825	-2.725797032	0.006414643	0.009368988
GPR37L1	186.6299672	0.161833773	0.059368339	2.725927236	0.006412113	0.009365992
SGK3	338.4416727	0.098861979	0.036256211	2.726759831	0.006395957	0.009343091
OXT	12.93001661	0.200049123	0.073360598	2.726928736	0.006392684	0.009339007
CCND2	8596.610465	0.161049115	0.059040208	2.727787068	0.006376075	0.009315438
SMAD4	2371.805472	0.098266538	0.036011045	2.728788805	0.00635674	0.009287882
APCDD1	23.68310922	-0.271737066	0.099574534	-2.728981554	0.006353026	0.009283148
TIGD3	56.83183888	-0.159000796	0.058256454	-2.729324979	0.006346413	0.009274178
NGB	0.871232761	-0.437060937	0.160131507	-2.729387516	0.006345209	0.009273111
RHNO1	394.1794295	-0.085775766	0.031426211	-2.729433898	0.006344317	0.009272499
CGB5	0.565982409	-0.523832769	0.191891163	-2.729843108	0.006336447	0.009261689
SPRYD7	145.215802	-0.1099741	0.04024277	-2.732766698	0.00628048	0.00918057
S100A4	5721.657644	-0.247436916	0.090525643	-2.733335089	0.006269651	0.009165425
TRIAP1	552.0064234	0.108794219	0.039802192	2.733372551	0.006268938	0.009165067
SART3	1613.510754	0.082787458	0.030276258	2.734401934	0.006249371	0.009137143
SLC36A3	40.78365257	0.263638071	0.096384577	2.735272378	0.006232868	0.009113695
PCDH1	29.57363519	-0.258665466	0.094524905	-2.736479507	0.006210046	0.009081004
TPSD1	445.9408813	0.376274428	0.137495321	2.736634419	0.006207123	0.009077407
HOMER3	832.5217144	0.176368339	0.06444593	2.736686988	0.006206131	0.009076635
KCTD8	0.441410277	-0.750947796	0.274326219	-2.737426265	0.0061922	0.009056937
SCEL	0.6309084	-0.599179789	0.21884293	-2.737944466	0.006182451	0.009043354
ZNF408	501.1113187	0.118357959	0.043227423	2.738029466	0.006180854	0.009041693
CLDN7	33.11690784	-0.160069573	0.05845898	-2.738152015	0.006178551	0.009039
PSMB2	3229.437434	0.096515475	0.035230445	2.739547393	0.006152384	0.009001392

PHF5A	573.2874886	0.08795969	0.032097434	2.740396314	0.006136514	0.008978843
IGDCC4	34.96770146	0.266967429	0.097410167	2.740652619	0.00613173	0.008972514
CHRNA7	95.00379471	-0.183513655	0.066918031	-2.742364831	0.006099855	0.008926539
PLEKHD1	38.74155937	-0.182260382	0.066456491	-2.742551974	0.00609638	0.008922121
LAMA1	4.544802636	-0.311536871	0.113589907	-2.742645705	0.00609464	0.008920242
ANKRD61	270.4158667	-0.186289063	0.067922765	-2.742660177	0.006094372	0.008920242
LCE5A	0.741631348	-0.628026986	0.228920522	-2.743428074	0.006080137	0.008900345
DNASE1L1	298.6061625	0.088787215	0.032350763	2.744516864	0.006060004	0.008871538
GNA15	6669.163989	0.167282135	0.060945864	2.744766012	0.006055406	0.008865469
ARFGEF1	5980.226889	0.111818514	0.040736939	2.744892382	0.006053074	0.008862719
PIP5K1C	1240.665735	-0.109487247	0.039885446	-2.745042548	0.006050305	0.008859327
VGLL4	1184.728774	0.093421581	0.034031997	2.745110198	0.006049058	0.008858164
IMPDH1	2345.38336	0.116859086	0.042565384	2.745401897	0.006043683	0.008850955
RUNDC1	981.8841377	0.089167704	0.032476585	2.745599733	0.006040041	0.008846282
IFNA21	0.923515005	-0.314091387	0.11439568	-2.745657764	0.006038972	0.00884538
LIMS1	2074.446972	-0.104508691	0.038061338	-2.745796571	0.006036418	0.0088423
VGLL1	1.80982646	-0.32740378	0.119207565	-2.746501698	0.006023457	0.008823975
TBX1	99.45656174	-0.217983754	0.07936414	-2.746627801	0.006021142	0.008821244
TRIOBP	1903.218882	-0.078404222	0.028539557	-2.747212344	0.006010421	0.008806195
CLEC2L	27.90064853	0.389392202	0.141634654	2.749272086	0.005972778	0.008751699
HNRNPH1	28104.53636	0.083349892	0.030315222	2.749440222	0.005969715	0.008747865
LRRC55	0.668662271	-0.418196005	0.152050628	-2.750373418	0.005952739	0.008723641
CCDC38	45.8996924	0.155740891	0.056622254	2.750524407	0.005949996	0.008720275
ARG1	1107.43338	-0.233633248	0.084937513	-2.750648553	0.005947742	0.008717624
GPX5	0.455657925	-0.538050026	0.195562477	-2.751294809	0.00593602	0.008701094
MPDU1	1032.298102	-0.104121251	0.037837461	-2.751803329	0.00592681	0.008688246
GIPC3	202.754686	-0.211062433	0.076698496	-2.751845786	0.005926042	0.00868777
ZHX1-C8orf76	23.81840663	0.13645465	0.049582001	2.752100511	0.005921434	0.008681666
STX4	1079.804559	-0.086746808	0.031516642	-2.752412768	0.00591579	0.00867404
SYNGR3	35.1908713	0.182576585	0.066323562	2.752816325	0.005908504	0.008664005
LY6G6C	81.87814538	-0.248562248	0.090245946	-2.754276064	0.005882213	0.0086261
KIF21A	52.73115132	-0.272977175	0.099098927	-2.754592649	0.005876525	0.008618404
ITPK1	2031.791292	0.104219974	0.037832713	2.754758099	0.005873554	0.008614693
CAMTA2	901.1177867	-0.10443762	0.037897293	-2.755806893	0.005854755	0.008587764
TMEM248	2794.957912	0.057130664	0.02072965	2.755987914	0.005851516	0.008583656
CKLF-CMTM1	0.314270454	-0.383440297	0.139084158	-2.756894111	0.005835324	0.008560546
MEGF10	123.1813102	-0.332923795	0.120760445	-2.756894403	0.005835319	0.008560546

RHOV	12.44498553	-0.281398155	0.102051802	-2.757405068	0.005826213	0.00854846
CUZD1	0.849823369	-0.354845859	0.128617076	-2.758932718	0.005799047	0.00850924
CR2	43.77099759	0.226031945	0.081917544	2.759261755	0.005793211	0.008501314
UNC45B	5.224090601	-0.283759947	0.102838062	-2.759289123	0.005792726	0.008501239
ATP6V0D2	8.335568392	0.343551666	0.124502634	2.75939276	0.005790889	0.00849918
TNFSF13B	1145.88951	0.191309021	0.069314352	2.760020346	0.005779776	0.008483507
ARHGAP29	17.64622771	-0.293651702	0.106362969	-2.760845281	0.005765198	0.008462744
TMEM159	412.7245871	-0.097848771	0.035428002	-2.761904857	0.005746522	0.008435962
PANX1	378.2781551	0.121050821	0.043827386	2.761990474	0.005745015	0.008434383
XRCC6	6153.582125	0.073941756	0.026765647	2.762561863	0.005734969	0.008420265
NFKBID	2828.452634	0.139090331	0.050345981	2.762689872	0.005732721	0.008417595
PDGFC	926.4513878	0.217962556	0.078876797	2.763329211	0.005721503	0.008401753
YWHAE	7793.646429	-0.069320476	0.025077499	-2.764249955	0.005705382	0.008378709
MRPS18A	169.4422454	-0.094661264	0.034241746	-2.764498789	0.005701032	0.008372949
RPAP3	686.231701	-0.081253431	0.029386076	-2.765031679	0.005691727	0.008359911
RASSF6	10.80882257	0.282583527	0.102167508	2.765884511	0.005676864	0.008338705
KMT2A	6096.68786	-0.102916891	0.037208716	-2.765935056	0.005675984	0.008338039
PCGF1	367.3435433	0.137572492	0.049724532	2.766692559	0.005662813	0.008319316
KDM6A	7144.774979	0.128483701	0.04641141	2.768364526	0.00563384	0.008277372
CWH43	0.433688996	-0.400702366	0.144738919	-2.768449352	0.005632374	0.008275839
RPRM	0.897147965	-0.497575415	0.179714493	-2.768699433	0.005628053	0.008270111
HGD	42.29197582	0.174829677	0.063143377	2.768772988	0.005626783	0.008268865
ZNF526	485.7136291	-0.094226129	0.034019129	-2.769798353	0.005609101	0.0082435
FERMT2	52.29100626	0.242276267	0.087465573	2.769961463	0.005606293	0.008239991
MTNR1B	1.627228545	-0.42213591	0.152369	-2.770484219	0.005597301	0.008227394
LILRA2	1460.164327	0.192177617	0.06934632	2.77127347	0.005583751	0.008208093
USP29	0.664632141	-0.371528933	0.134056621	-2.771432916	0.005581017	0.00820469
RBM24	2.440766506	-0.324099451	0.116904648	-2.772340173	0.005565484	0.00818247
FAM222A	117.9664543	-0.208151066	0.075070232	-2.772751081	0.005558462	0.00817276
C4BPB	6.425418487	0.253247671	0.091310445	2.773479754	0.005546029	0.008155092
ACO2	2899.58219	-0.067381583	0.024293142	-2.773687408	0.005542491	0.008150501
SERAC1	312.0772907	-0.11728113	0.042282476	-2.773752686	0.005541379	0.008149478
EVC	103.390955	-0.379705601	0.136861034	-2.774387939	0.005530568	0.008134191
CRK	879.9467735	0.093763289	0.033789965	2.774885606	0.005522113	0.008122365
PLEKHS1	27.42596852	0.249378918	0.089844245	2.775680482	0.005508632	0.008103145
SPAG5	1382.177056	-0.1120871	0.04037778	-2.775959987	0.005503898	0.008096791
MMP17	176.1674501	0.231858374	0.083508383	2.776468245	0.0054953	0.00808475

FIBIN	0.44615623	-0.829165578	0.298511237	-2.777669562	0.005475027	0.008055528
TEX45	122.6971605	0.185179433	0.066661968	2.777887303	0.005471359	0.008050738
MX1	1735.3417	0.233885788	0.084188294	2.778127195	0.005467321	0.008045401
GIGYF2	3014.626201	-0.086067528	0.030977192	-2.778416063	0.005462462	0.008038855
C10orf67	1.287387938	-0.388320426	0.139752732	-2.778624938	0.005458952	0.008034293
ARL4D	66.74350202	-0.195917748	0.070506191	-2.778731133	0.005457167	0.008032271
CHD9	5079.505607	-0.090181849	0.032433506	-2.780514947	0.005427276	0.007988875
COL14A1	147.5888974	-0.276237314	0.099330179	-2.781000861	0.005419159	0.007977528
SMCO2	7.498238078	-0.209370347	0.075222286	-2.783355271	0.005379986	0.007920456
TUBGCP3	2325.806718	-0.076205074	0.027378384	-2.783402954	0.005379195	0.007919888
TP63	67.28804743	-0.33961881	0.122009775	-2.783537713	0.005376961	0.007917194
TMEM272	34.07956818	-0.240932703	0.086547964	-2.783805569	0.005372523	0.007911254
TMEM174	0.271948502	-0.75835004	0.272340792	-2.7845628	0.005359994	0.007893399
FCRL5	144.3920362	0.240487051	0.086364309	2.784565209	0.005359954	0.007893399
ADCY3	1388.459944	-0.118602863	0.042577896	-2.785550109	0.005343698	0.007870585
ELOA3	0.403145429	-0.551692986	0.198023659	-2.785995319	0.005336364	0.007860375
IK	2003.719152	0.105546909	0.0378836	2.786084468	0.005334897	0.007858804
ENDOD1	596.3979913	-0.178599117	0.064099857	-2.786263866	0.005331945	0.007855047
ADNP	5038.222328	0.0807044	0.028959368	2.786814985	0.005322886	0.007842292
FMR1NB	0.661087323	-0.374674519	0.134403692	-2.787680249	0.005308691	0.007821967
KBTBD6	288.899801	-0.115666993	0.041489923	-2.787833373	0.005306183	0.00781886
SRP9	2219.311202	0.078262331	0.028057781	2.789327163	0.005281768	0.00778347
RNASE9	1.407390176	-0.397839965	0.142622224	-2.789466848	0.00527949	0.007780699
AC021072.1	0.697768332	-0.328493326	0.117707349	-2.790763083	0.005258395	0.007750193
HSD11B1L	109.0336544	-0.169641035	0.060761683	-2.791908105	0.005239823	0.007723403
MTHFD2L	762.5143171	-0.114278007	0.040924198	-2.792431201	0.005231359	0.007711507
LIMS4	0.335672306	0.482578831	0.172785469	2.792936426	0.005223196	0.007700053
SUN2	5681.939596	0.092484628	0.033102317	2.793901966	0.005207626	0.007677679
SMAP1	355.4375572	0.071733425	0.025674456	2.79396089	0.005206678	0.007676859
PALLD	238.3840209	-0.226149968	0.080929248	-2.794415763	0.005199358	0.007666645
CLPX	1435.867257	0.061064434	0.021847451	2.795036991	0.005189378	0.007652504
ZNF304	424.2955269	0.119584005	0.042784009	2.795063133	0.005188958	0.007652462
IL36G	1.161033529	-0.419597524	0.150113981	-2.79519284	0.005186877	0.007649969
NGLY1	2346.701527	0.076566208	0.027389345	2.795474268	0.005182363	0.007643887
NGEF	34.59831418	-0.206299224	0.073787395	-2.795859985	0.005176182	0.007635346
BMP1	965.3278258	-0.130389239	0.046630418	-2.796227109	0.005170305	0.007627253
IRX1	131.3811984	0.56608241	0.202440104	2.796295785	0.005169207	0.007626207

TRIM59	370.2219863	-0.112176245	0.040112221	-2.796560326	0.005164977	0.007620541
GOSR2	1176.283714	-0.087543541	0.03128595	-2.798174302	0.005139237	0.007583135
ZNF575	139.3449465	-0.104735411	0.037425524	-2.798502188	0.005134022	0.007576011
DNHD1	2570.853264	-0.122653644	0.043827788	-2.798536016	0.005133484	0.007575789
ITGB1BP2	144.7811059	-0.141805006	0.050668556	-2.798678656	0.005131217	0.007573015
CCKBR	2.06823929	-0.347389389	0.124117652	-2.798871737	0.00512815	0.007569059
GJC2	67.78830407	-0.225280365	0.080472274	-2.799478039	0.00511853	0.007555429
PI15	11.9149623	-0.55640956	0.198731733	-2.799802289	0.005113391	0.007548413
PPP3CA	1778.296933	-0.083386056	0.029778155	-2.800242522	0.005106423	0.007538694
ZNF432	490.7102129	0.115163347	0.041121627	2.800554242	0.005101493	0.007531985
GPATCH2	426.5875679	-0.093043478	0.033217758	-2.801016176	0.005094197	0.007521779
FSTL3	463.9743329	-0.157578766	0.056252525	-2.801274552	0.005090119	0.007516326
ZNF467	693.7911513	0.1785083	0.063707009	2.802019803	0.005078376	0.007499551
RTN4RL2	49.75382287	-0.154248466	0.05503236	-2.802868441	0.005065033	0.007480411
IL10	41.20069919	0.253981722	0.090538554	2.805232813	0.005028025	0.007426315
MTERF4	666.4746853	0.07336818	0.026148367	2.805841743	0.005018533	0.007412856
ZIC4	0.930365476	-0.594969412	0.211987431	-2.806625897	0.005006335	0.007395395
REC8	1580.334375	0.152012642	0.054160531	2.806705142	0.005005103	0.007394134
AP2M1	6317.501513	-0.093705152	0.033384558	-2.806841209	0.00500299	0.00739157
NKX1-2	0.43619702	-0.551378768	0.196388587	-2.807590688	0.004991362	0.007374948
PARP4	4656.229958	-0.096161413	0.034242702	-2.80823087	0.00498145	0.007360858
RNFT2	227.1697867	-0.214772588	0.076458135	-2.809022064	0.004969224	0.007343346
MATN4	32.27367603	-0.392081772	0.139481924	-2.810986259	0.00493899	0.007299218
TUBB8B	1.996535328	-0.345903089	0.123046014	-2.811168586	0.004936191	0.007295634
DVL3	2499.394498	0.091470006	0.032532972	2.811609322	0.004929434	0.007286196
RGP1	1295.307855	0.092834988	0.033016102	2.811809474	0.004926367	0.007282214
LRRC42	326.643917	0.073515227	0.026138832	2.812490923	0.004915941	0.00726735
ZXDC	3298.962314	0.07875862	0.027995081	2.813302112	0.004903556	0.007249588
OTX2	0.524819841	-0.600202526	0.213249741	-2.814552197	0.004884524	0.007221997
AP001931.2	9.200089753	-0.200760578	0.071328005	-2.814610871	0.004883633	0.007221224
DENND3	13948.89477	0.123626807	0.0439173	2.814991047	0.004877859	0.007213232
CYP51A1	82.6834304	-0.132254144	0.046972847	-2.815544592	0.004869464	0.007201361
EIF4E1B	1.485198869	-0.257224449	0.091358484	-2.81555077	0.00486937	0.007201361
PEF1	911.6604263	0.079476324	0.028221965	2.816115928	0.004860812	0.007189653
MALSU1	244.7732518	-0.094064633	0.033396555	-2.816596866	0.00485354	0.00717944
IL18R1	226.1068787	0.233972501	0.083064633	2.816752367	0.004851191	0.007176507
POLK	1027.379812	0.093911057	0.03333942	2.816817388	0.004850209	0.007175597

ADCY4	412.0669909	-0.176600563	0.062690595	-2.817018433	0.004847174	0.007171649
XDH	9.806494636	-0.318772051	0.113152353	-2.817193299	0.004844536	0.007168287
TMEM51	80.15055722	0.263046232	0.093371144	2.817211174	0.004844266	0.007168287
STX16	3114.128219	0.064525604	0.022900721	2.817623188	0.004838055	0.00715978
MOS	0.828749185	-0.471456032	0.167249413	-2.818880033	0.004819153	0.007132346
IFT52	644.0745064	0.091092174	0.032312237	2.819123084	0.004815505	0.007127486
L3MBTL3	682.2761562	0.139147782	0.049346754	2.819796058	0.004805418	0.007113094
AGO1	5635.979622	0.091620539	0.03248869	2.82007491	0.004801244	0.007107453
TXN	3266.837188	-0.186798507	0.066213247	-2.821165162	0.004784956	0.007083878
CPNE7	226.8207956	-0.303745868	0.107646841	-2.821688626	0.004777153	0.007072861
BMP3	105.8696349	0.441795684	0.156559577	2.821901374	0.004773986	0.007068706
LINGO4	1.589285805	-0.332608812	0.117862945	-2.821996439	0.004772571	0.007067145
SLC16A4	291.0514113	-0.155831685	0.055218359	-2.822099169	0.004771042	0.007065416
PTGER4	7008.018244	0.154402342	0.054699399	2.822742918	0.004761474	0.00705178
RPAIN	1560.80979	0.087760774	0.031080485	2.82366168	0.004747847	0.007032131
PTGES3	5537.759639	0.080666311	0.02856304	2.824150031	0.004740619	0.007021956
RTP4	54.84671409	-0.17351417	0.061433883	-2.82440508	0.004736848	0.007016901
CCNYL1	687.0925629	0.096745928	0.034241997	2.825358789	0.00472277	0.006996577
SEC13	2489.699886	0.061010987	0.021593253	2.825465246	0.004721201	0.006994782
NAB1	748.4218401	0.07977394	0.028229514	2.825905509	0.004714717	0.006985704
DPH3	566.4661744	0.10198053	0.036086921	2.825969265	0.004713779	0.006984843
SLC37A2	287.5621734	0.21456782	0.075892081	2.827275492	0.004694592	0.006956939
FGL1	2.957500057	-0.380352254	0.134494367	-2.828016245	0.004683743	0.006941387
DNLZ	37.75484849	-0.211466564	0.074774937	-2.828040679	0.004683385	0.006941383
CHRM4	65.48968497	0.154390417	0.054585705	2.828403855	0.004678075	0.006934037
RPL37A	36037.17594	-0.223480442	0.07900538	-2.828673714	0.004674133	0.006928718
SLC52A1	9.551669233	-0.218600275	0.077269819	-2.829051221	0.004668623	0.006921075
OXSM	137.9171434	-0.0927012	0.032766647	-2.829132977	0.00466743	0.006919831
RB1	1908.597014	-0.110531553	0.039063563	-2.82953078	0.004661632	0.006911758
SPEM1	1.035716519	-0.391485636	0.138349054	-2.829695074	0.004659239	0.006908733
DHRS12	305.5343356	-0.14027357	0.04955887	-2.830443252	0.004648356	0.006893118
PSMB3	2253.032968	-0.153876448	0.05435968	-2.830709255	0.004644492	0.00688791
TMIGD2	227.9979934	-0.267475165	0.094455448	-2.831760052	0.004629257	0.006865837
C1orf50	268.2530681	0.107561641	0.037983034	2.831833809	0.00462819	0.006864774
NRP1	228.6317406	0.31478444	0.111125643	2.832689466	0.00461582	0.006846946
MSRB3	1991.149178	-0.204613938	0.072232659	-2.832706733	0.004615571	0.006846946
SMAD1	341.6593916	-0.212946947	0.075173282	-2.832747756	0.004614979	0.006846735

PLLP	112.4261001	-0.104099938	0.036741975	-2.833270086	0.004607444	0.006836075
RNF44	3595.304193	-0.083718539	0.029536847	-2.834376314	0.004591523	0.006812969
SOWAHA	3.739971842	-0.291146527	0.102718919	-2.83440022	0.004591179	0.006812969
SDK1	63.21981084	-0.300638484	0.106028703	-2.835444326	0.004576199	0.006791261
ADAMTSL2	30.57718429	-0.304898529	0.107504619	-2.836143539	0.004566192	0.006776924
TIMM17A	1326.064025	0.09734633	0.034322657	2.836211969	0.004565214	0.006775986
SMPD4	3122.412159	0.076601663	0.027007106	2.836352116	0.004563211	0.006773526
RNFT1	382.9559721	0.08735516	0.030798214	2.836370924	0.004562942	0.006773526
SLC7A2	30.86022521	-0.357937736	0.126179949	-2.836724359	0.004557894	0.006766661
LCN8	16.04635154	-0.359490593	0.126710365	-2.83710486	0.004552466	0.006759115
FARP1	242.4488052	-0.181148701	0.063835865	-2.837726113	0.004543615	0.006746486
MC5R	1.733542612	-0.384507161	0.135435514	-2.839042363	0.004524915	0.006719229
DYDC1	0.867989703	-0.39381095	0.138696309	-2.839375859	0.004520188	0.006712719
GAD2	1.164873007	-0.370778756	0.13055238	-2.840076568	0.004510271	0.0066985
ALOX5	2164.294559	0.218692794	0.077001865	2.840097347	0.004509977	0.0066985
CPLX1	7.092026704	-0.27101914	0.09541116	-2.840539207	0.004503734	0.006689806
ITGAV	892.0833536	0.23073021	0.081222986	2.840700907	0.004501451	0.006686923
NANS	1035.723195	0.100791793	0.03547628	2.841103793	0.004495768	0.006678988
RGS4	0.636964686	-0.606963816	0.213618053	-2.841350755	0.004492287	0.006674324
NOBOX	1.757656028	-0.351875257	0.123837995	-2.841415968	0.004491369	0.006673465
TPPP2	2.54212009	-0.347713221	0.1223624	-2.841667229	0.004487831	0.006668715
DSP	21.30433356	-0.262334445	0.09226276	-2.843340526	0.004464334	0.006634304
EID2B	58.05758767	-0.10454692	0.036765128	-2.843643558	0.004460091	0.006628502
SPATA32	39.45236015	-0.178026311	0.062595577	-2.844071717	0.004454102	0.006620103
CABP2	1.036440297	-0.364139969	0.128025021	-2.844287514	0.004451086	0.006616123
POMGNT2	275.5673763	-0.173836795	0.061098463	-2.845190984	0.00443848	0.006597887
H3C1	778.5283755	-0.191194914	0.067183209	-2.845873487	0.004428979	0.006584263
NBPF26	441.2339495	-0.132363142	0.046489148	-2.84718364	0.004410791	0.006557722
HBE1	1.231503935	-0.566843755	0.198999929	-2.848462094	0.004393109	0.006531929
TM4SF5	1.127963567	-0.382186776	0.134149393	-2.848963881	0.004386186	0.006522132
SLC27A6	43.64569484	-0.39418533	0.138327165	-2.849659565	0.004376605	0.006508379
RUBCN	1112.795688	0.10315658	0.036187772	2.850592168	0.00436379	0.006489816
C11orf24	295.2857437	-0.151062398	0.052973506	-2.851659429	0.004349167	0.00646856
DRC3	319.5614121	-0.141531991	0.049614938	-2.85260843	0.004336202	0.006449766
GTF2A1L	0.474127125	-0.623396786	0.218472932	-2.853428022	0.004325032	0.006433642
WDR44	801.2163988	0.077167878	0.027035305	2.854337236	0.004312672	0.006415744
PAWR	95.20603653	-0.275747163	0.096598628	-2.854566029	0.004309567	0.006411611

SMIM26	257.3171249	-0.179632612	0.062922544	-2.854821198	0.004306106	0.00640695
MEPE	1.454318494	0.412190646	0.144373893	2.855022026	0.004303384	0.006403386
TMEM208	335.1129485	-0.118497676	0.041503315	-2.855137627	0.004301818	0.006401543
TGDS	176.0977382	-0.098059157	0.03434273	-2.855310448	0.004299478	0.006398546
FBL	3739.594391	-0.128096121	0.044861634	-2.855360127	0.004298805	0.006398032
IRAK4	1042.83743	-0.078957386	0.027643139	-2.856310377	0.004285959	0.006379398
TMEM212	11.98966982	-0.372749635	0.130492849	-2.856475566	0.00428373	0.006376564
PUM1	7658.673989	0.094304464	0.033013325	2.856557567	0.004282623	0.006375402
SCUBE3	245.1533899	-0.203927698	0.071387602	-2.856626254	0.004281697	0.006374508
CNR2	215.2686898	0.206985041	0.072449822	2.856943385	0.004277421	0.006368627
TSPAN7	143.8965155	-0.332556386	0.116401421	-2.856978757	0.004276945	0.006368401
ANKRD1	5.015844695	-0.267943341	0.093759297	-2.857778898	0.004266175	0.006352849
LRP5L	1441.247109	0.161457248	0.056495954	2.85785507	0.004265151	0.006351808
ARMH1	3124.872824	-0.188279197	0.065855582	-2.858970982	0.004250177	0.006329988
C1QTNF1	19.81940352	-0.307217946	0.107425443	-2.859824791	0.004238751	0.006313452
AC005833.1	2.452802793	-0.247227165	0.086432046	-2.860364609	0.004231542	0.006303194
FAM86B2	4.086308236	-0.24266195	0.084823335	-2.860792386	0.004225837	0.006295175
FUT1	86.69009935	-0.25366845	0.088661054	-2.86110347	0.004221692	0.00628948
SERINC2	253.9972562	0.263025248	0.091867097	2.863106118	0.0041951	0.006250339
ARSH	0.571586217	-0.460328111	0.160747221	-2.863676954	0.004187548	0.006239562
DLX2	33.37148055	-0.315930471	0.110310809	-2.864002839	0.004183243	0.006233621
SYNE2	2052.965639	0.244262805	0.08528655	2.864024921	0.004182951	0.006233621
LILRA4	34.00527962	-0.233166444	0.081401929	-2.864384738	0.004178202	0.006227057
NT5E	272.1204326	0.263552746	0.092006814	2.864491599	0.004176792	0.006225431
LIMD2	5652.434403	0.10490926	0.036620177	2.864793888	0.004172807	0.006219965
ARHGAP31	471.5779335	0.202625561	0.070720581	2.865156914	0.004168027	0.006213312
PMM2	367.5694266	-0.090693991	0.031653022	-2.865255314	0.004166731	0.006211854
DARS1	1721.89073	-0.092171609	0.032164779	-2.865606804	0.004162108	0.006205435
CASP1	920.9930289	0.160269904	0.055911797	2.866477406	0.004150678	0.006188864
OGT	19917.38372	-0.106207664	0.037044279	-2.867046297	0.004143224	0.006178221
CERS6	2345.354467	0.096920783	0.03379585	2.86783085	0.004132964	0.006163391
SMYD2	477.8640383	-0.105339807	0.036727295	-2.868161344	0.004128649	0.006157426
CACNA1D	194.0101627	-0.166300575	0.057971773	-2.868647383	0.004122311	0.006148441
RPL29	26192.33878	-0.215915437	0.075264616	-2.868750916	0.004120962	0.006146897
NRP2	126.1441546	0.289571082	0.100892953	2.870082343	0.004103649	0.00612154
THOC1	2180.718669	0.106119995	0.036973575	2.870157808	0.00410267	0.006120546
FLOT2	3830.966848	-0.115893437	0.040377337	-2.870259558	0.00410135	0.006119043

NECAB3	269.9203112	-0.124236721	0.043283905	-2.870275244	0.004101146	0.006119043
TRIM32	101.0181637	-0.225734256	0.078643079	-2.870363915	0.004099996	0.006117956
OXCT1	406.2179783	-0.129901091	0.045245569	-2.871023487	0.004091451	0.006105671
CELSR3	491.2570443	-0.188906863	0.065783207	-2.871657867	0.004083248	0.006093893
HROB	401.3511159	-0.093993788	0.032726931	-2.872062425	0.004078024	0.006086561
MAEA	1728.238548	-0.07786855	0.027105918	-2.872750932	0.004069148	0.006073776
TRAPPC3L	1.657336582	-0.417186726	0.145198457	-2.873217348	0.004063145	0.006065279
IGLL5	19.34307931	-0.324355484	0.112867389	-2.873775031	0.004055978	0.006055042
HKDC1	6.56836634	-0.225536408	0.078477783	-2.873888621	0.004054519	0.006053326
PIGX	384.0721216	-0.082085486	0.028554508	-2.87469448	0.004044186	0.00603836
CMYA5	79.58388348	-0.235316407	0.081857423	-2.874710675	0.004043979	0.00603836
AC000120.2	0.413764885	-0.430477971	0.14974067	-2.874823332	0.004042536	0.006036817
EGR4	17.83214981	-0.29074408	0.101105526	-2.875649738	0.004031969	0.006021496
CSHL1	0.382553876	-0.591332871	0.205613364	-2.875945711	0.00402819	0.006016311
NFATC3	3483.28575	-0.090232967	0.031374511	-2.875995937	0.004027549	0.006015813
HSP90AB1	35025.14714	-0.104533023	0.036330641	-2.877268904	0.004011336	0.005992054
KLHL17	157.1060795	-0.157456695	0.054716126	-2.877701789	0.004005836	0.005984295
OR6N2	8.924973436	-0.390713005	0.135753092	-2.878114972	0.004000593	0.005976918
GRIN3B	65.39448825	-0.174787594	0.06072414	-2.878387296	0.003997141	0.005972217
GNPTG	933.3707767	-0.101936375	0.03541026	-2.878724306	0.003992872	0.005966294
ZFP57	65.16526027	-0.552326093	0.191862202	-2.878764482	0.003992364	0.00596599
POSTN	7.746279789	-0.530484135	0.184267696	-2.878877567	0.003990932	0.005964306
OR10P1	0.607243828	-0.468142446	0.162539724	-2.880172516	0.003974576	0.005940316
OOSP2	0.382278774	-0.646967052	0.224624498	-2.880215902	0.003974029	0.005939952
DUSP15	23.50782557	-0.27557879	0.095664152	-2.880690263	0.003968054	0.005931474
NKX2-3	287.5170778	-0.558972785	0.193918769	-2.88250997	0.003945207	0.005897772
B3GNT2	2858.12425	0.119440885	0.041420555	2.883613821	0.003931405	0.005877589
RTL8A	123.7013741	-0.267445954	0.092717223	-2.884533685	0.003919938	0.005860893
COX8A	1128.926894	-0.20857624	0.072305646	-2.884646646	0.003918532	0.005859238
KRT8	228.9401746	-0.275257888	0.095418242	-2.884751209	0.003917231	0.00585774
COX6A2	0.363191876	-0.470794069	0.163172584	-2.885252272	0.003911001	0.005848871
VPS29	1223.748237	-0.109203693	0.037848189	-2.885308314	0.003910305	0.005848277
PKP1	1.208773685	-0.341230402	0.118217899	-2.886452933	0.00389611	0.005827492
LCN6	18.82705655	0.470147949	0.162874425	2.886567056	0.003894697	0.005825824
EXT1	288.1287651	0.220153298	0.076257152	2.88698558	0.003889521	0.005818525
CACNA2D2	230.9314563	0.278839189	0.096579909	2.887134523	0.00388768	0.005816216
ZNF260	309.2819864	-0.114502378	0.039657263	-2.887299025	0.003885648	0.00581362

DDOST	6037.439758	0.1160687	0.040197474	2.88746254	0.003883628	0.005811044
TOMM70	1387.13197	0.067887578	0.023511012	2.887480087	0.003883412	0.005811044
MVB12B	930.7737911	0.172376345	0.059697827	2.887481055	0.0038834	0.005811044
TKTL2	6.54715135	0.264981924	0.091764907	2.887617189	0.00388172	0.00580952
ARPC3	4041.645666	0.153533813	0.053168199	2.887700123	0.003880697	0.005808433
CDK11B	1676.62136	0.096821833	0.033522537	2.888260864	0.003873785	0.005798531
PRSS58	0.570456038	-0.551384764	0.190805981	-2.889766662	0.003855279	0.005771271
PPP1R1B	29.32963928	0.228156614	0.078944666	2.890082703	0.003851405	0.005765913
BRMS1L	137.680798	0.135252011	0.046797052	2.890182268	0.003850185	0.005764528
SCIMP	214.1440728	0.256997286	0.088920473	2.890192508	0.00385006	0.005764528
ETV5	561.338929	0.190200604	0.065798003	2.89067441	0.003844162	0.00575639
NYX	4.031466075	-0.318036869	0.110003512	-2.891151951	0.003838325	0.00574809
BHLHE23	16.02756393	0.541169158	0.18716768	2.891360073	0.003835784	0.005744724
DSPP	0.444849803	-0.462390259	0.159883984	-2.892036138	0.003827539	0.005732815
SNRPC	1714.049491	-0.172892064	0.059766687	-2.89278315	0.003818448	0.005719636
SCML2	591.7203772	0.176068345	0.060854149	2.893284158	0.003812362	0.005710957
PLAAT4	344.63954	-0.19055129	0.065853636	-2.893557612	0.003809044	0.005706423
CENPH	364.9498305	-0.127380863	0.0440205	-2.893671416	0.003807664	0.005704792
SHISA2	30.37136755	0.326625472	0.112842529	2.894524559	0.003797332	0.005689748
TFRC	17591.94221	-0.160940977	0.055586194	-2.895340802	0.003787471	0.005675407
MIS18BP1	4442.889879	0.107984022	0.037288536	2.895904057	0.00378068	0.005665664
POMK	1986.339648	0.097718535	0.033740427	2.896185514	0.00377729	0.005661019
TCEAL6	0.652783644	0.574535405	0.198311255	2.897139678	0.00376582	0.005644261
VASN	428.9996933	0.160386109	0.055357863	2.897259756	0.003764379	0.005642533
MAGEA4	0.446269471	-1.553743346	0.536146439	-2.89798315	0.003755708	0.005629966
FAM160A2	1926.149047	0.103150211	0.035574849	2.89952633	0.00373727	0.005602756
ZCCHC24	347.8345686	0.185584315	0.063998262	2.899833678	0.003733607	0.005597694
TSTD3	353.0828847	0.130176792	0.04486654	2.9014226	0.003714725	0.005569811
GABRD	19.8746241	-0.283622434	0.09770751	-2.902770051	0.003698781	0.00554633
OR4X2	0.375836826	-0.595985244	0.20531339	-2.902807472	0.003698339	0.005546092
FAAH2	34.78976399	0.189982245	0.065430786	2.90356048	0.003689457	0.005533196
PSMB7	2904.828623	0.079766507	0.027471236	2.903637404	0.003688551	0.005532261
XCL1	23.76352629	0.275011848	0.094705633	2.90385946	0.003685936	0.005528762
ATP6V0B	5365.89178	0.135443381	0.046635196	2.90431672	0.003680556	0.005521116
COL17A1	248.1704177	-0.236711164	0.08149944	-2.904451414	0.003678973	0.005519164
ZFP30	228.9604173	-0.159088918	0.054737046	-2.906421331	0.003655888	0.005484953
PAEP	0.945600109	-0.443681232	0.15261483	-2.907196061	0.003646845	0.005471806

ELAVL4	14.88671664	-0.484944172	0.166784318	-2.907612518	0.003641993	0.005464944
ALDOB	15.68304528	-0.278799326	0.09584268	-2.908926667	0.003626719	0.005442442
TP53INP1	4059.256798	0.17447283	0.059962105	2.909718241	0.003617547	0.005429095
LAMB4	14.50211397	-0.339495573	0.116651981	-2.910328406	0.003610492	0.005418921
DEF8	952.5735475	-0.136335215	0.04683445	-2.911002809	0.003602708	0.005407653
C20orf144	25.76244124	-0.187937365	0.064551558	-2.911430366	0.003597781	0.005400672
TMEM109	1395.646511	-0.138541991	0.047571349	-2.912298971	0.003587791	0.005386089
EDRF1	1128.555459	0.093043231	0.031946475	2.9124725	0.003585798	0.00538351
TRPM6	96.20935594	-0.325284288	0.111666224	-2.913005163	0.003579687	0.005374748
RALGAPA2	4389.992714	-0.126046189	0.043266724	-2.913236269	0.003577038	0.005371183
MKRN1	6874.684512	0.124003949	0.042560787	2.913572724	0.003573186	0.00536581
MVP	2039.850198	0.123530835	0.042391587	2.914041291	0.003567827	0.005358174
ABCD4	1517.787912	-0.081650638	0.028018338	-2.91418561	0.003566178	0.005356108
LGALS7B	1.305343213	-0.358065402	0.122832394	-2.915073051	0.003556053	0.005341311
SEC14L5	15.47148752	-0.303093431	0.103923589	-2.916502734	0.003539796	0.005317301
RAVER1	1329.054808	-0.115230956	0.039507709	-2.916670139	0.003537897	0.005314856
DUOXA2	6.047405243	-0.303629204	0.104097177	-2.916786136	0.003536582	0.005313288
ZDHHC21	752.9513651	-0.121479689	0.04163996	-2.917382443	0.003529827	0.005303546
MMP20	0.765620183	-0.508617551	0.174329695	-2.917561182	0.003527804	0.005300915
ZNF286A	79.4403011	-0.171044589	0.058621601	-2.917774126	0.003525396	0.005297703
LENG9	85.70832619	-0.152848713	0.052363955	-2.918968081	0.003511922	0.00527786
TRAPPC2B	116.9078692	-0.146537144	0.050194309	-2.919397587	0.003507086	0.005270997
IKBIP	219.4708298	-0.118649983	0.040630508	-2.92021902	0.003497855	0.005257526
FAM187B	1.176088591	-0.338072264	0.115767995	-2.920256718	0.003497431	0.005257294
PRSS12	11.60831498	-0.320732785	0.10982278	-2.920457711	0.003495176	0.005254308
FAM172A	660.2933337	-0.087340302	0.029898328	-2.921243649	0.003486371	0.005241473
SNRPB2	980.3093611	0.13605531	0.046568444	2.921620267	0.003482158	0.005235542
NCALD	124.9122985	-0.24659986	0.084404854	-2.921631266	0.003482035	0.005235542
USP24	9088.075843	0.115847613	0.039647648	2.921928989	0.003478708	0.005231159
PVALB	1.752284633	-0.422424776	0.144496605	-2.923423533	0.003462052	0.005206512
SLC44A2	2583.492748	0.138757792	0.047463791	2.923445192	0.003461812	0.005206512
HOXA6	450.7379158	-0.466710587	0.159642557	-2.923472263	0.003461511	0.005206497
SLCO2A1	11.52149664	-0.320770122	0.109703538	-2.923972449	0.003455953	0.005198538
AP4S1	847.1165136	-0.094815287	0.032421233	-2.924481198	0.003450309	0.005190447
MROH2B	0.997595372	-0.534730369	0.182676135	-2.927204316	0.003420241	0.005145609
CYP46A1	59.5505737	-0.323923081	0.110645395	-2.927578522	0.003416128	0.005139816
NAP1L5	204.0040678	0.093419337	0.031907515	2.927816147	0.003413518	0.005136285

YIPF7	1.97481901	-0.269979157	0.092208182	-2.927930603	0.003412262	0.005134789
SHMT2	1750.69083	-0.1080873	0.036911382	-2.928291911	0.003408299	0.00512922
LATS1	2106.865367	0.088766129	0.030312041	2.928411467	0.003406988	0.005127642
FCN1	4647.922071	0.29005697	0.099040144	2.928680819	0.003404038	0.005123595
HYLS1	77.17967657	-0.126077855	0.043048386	-2.928747536	0.003403307	0.00512289
PTMA	32487.35347	-0.158828415	0.054225635	-2.929028228	0.003400235	0.005118659
FKBP5	3191.806966	0.170344781	0.058153084	2.929247611	0.003397836	0.005115441
EXOG	392.7393652	-0.103220093	0.035236533	-2.929348771	0.003396731	0.00511417
GMPS	2440.727033	-0.067700026	0.023107261	-2.929816153	0.003391626	0.005106877
TCEAL3	209.0124911	0.189481889	0.064666778	2.930127276	0.003388232	0.005102159
CFAP53	43.76784038	-0.166055483	0.056665649	-2.930443486	0.003384786	0.005097362
ITM2A	3368.234921	0.272229017	0.092886672	2.930765102	0.003381284	0.005092479
PPAN-P2RY11	1.038731951	-0.297387957	0.101456367	-2.931190678	0.003376655	0.005085899
FNDC10	236.8793528	-0.235567123	0.080360055	-2.931395735	0.003374426	0.005082934
ECHDC3	115.1394804	0.197039672	0.06719831	2.932211734	0.003365572	0.005069987
SLC26A3	12.39813984	-0.270827381	0.092347093	-2.932711504	0.00336016	0.005062223
HGSNAT	2254.168938	-0.100878639	0.034379766	-2.934244465	0.003343607	0.005037674
ATG9A	1350.570699	0.145065141	0.049434932	2.934466265	0.003341219	0.005034463
C19orf47	463.2531054	-0.081089401	0.027633228	-2.934488888	0.003340975	0.005034463
MTHFD1L	1123.201116	-0.12069294	0.041117082	-2.935347908	0.003331739	0.005020952
MBTPS2	337.8702443	-0.090589655	0.030861079	-2.935401433	0.003331164	0.005020473
SMDT1	1305.238081	-0.142738307	0.04862239	-2.935649778	0.003328499	0.005016842
HRH2	1212.016452	0.219807694	0.074856581	2.936384357	0.003320626	0.005005361
HSPA4L	69.9094352	-0.268567572	0.09145468	-2.936619224	0.003318112	0.005001957
ENPP4	549.6329456	0.204546921	0.069653243	2.936646054	0.003317825	0.00500191
MRPS5	947.8518161	-0.087474357	0.029777678	-2.937581527	0.003307832	0.004987228
RPS6KA3	4197.40004	0.097743042	0.033271939	2.937702017	0.003306547	0.004985674
MEGF11	172.8422909	-0.258776369	0.088079304	-2.9379929	0.003303446	0.004981383
SETD9	293.2006668	0.113582008	0.03865899	2.938049067	0.003302847	0.004980864
PPP1R3A	0.577914738	-1.173260695	0.399288444	-2.938378789	0.003299336	0.004975953
NUDT19	530.584496	-0.111617399	0.037982802	-2.938629897	0.003296665	0.004972306
PRKRA	1887.953511	0.07136938	0.024284243	2.938917209	0.00329361	0.004968082
CPVL	724.5206521	0.285225213	0.097019348	2.93987971	0.003283397	0.004953058
ITM2C	4510.335916	0.194905295	0.066276021	2.940811661	0.003273535	0.004938562
THEM5	18.43998118	-0.311828435	0.106030365	-2.940935215	0.00327223	0.004936973
NLGN3	531.5896478	0.153405341	0.052152683	2.941465951	0.003266628	0.004928901
NQO1	115.4780191	-0.15093194	0.051309372	-2.941605678	0.003265154	0.004927057

POU5F1B	5.466216682	-0.265677967	0.090296097	-2.942297336	0.00325787	0.004916444
UQCRB	2481.591839	-0.16832983	0.057203009	-2.942674409	0.003253904	0.004910839
BTBD19	456.9687043	0.165641079	0.056275954	2.943372207	0.003246578	0.00490016
HSD17B3	0.480996844	-0.48723938	0.165506568	-2.943927767	0.003240756	0.00489175
HCAR3	299.3164114	0.246497942	0.083723174	2.944202046	0.003237885	0.004887794
AP2A2	2073.498168	0.095980798	0.032595572	2.944596199	0.003233764	0.004881949
DDX19A	579.8506513	-0.090210098	0.030635131	-2.944661745	0.003233079	0.004881291
CACNA1G	12.62598599	-0.249671532	0.084760236	-2.945621	0.003223071	0.004866556
ATP6AP1	4266.447926	0.104258496	0.035386098	2.946312279	0.003215876	0.004856066
PSMB6	1121.096472	-0.183168341	0.062165437	-2.9464659	0.003214279	0.004854029
CARM1	1148.03941	-0.108178985	0.03670849	-2.946974512	0.003208997	0.004846427
BTG2	6426.682488	0.139263098	0.047254569	2.947082199	0.003207879	0.004845113
ZDHHC12	700.1752696	-0.163347147	0.055414174	-2.947750275	0.003200955	0.004835028
GALR2	36.83800423	-0.169194037	0.057393049	-2.947988308	0.003198492	0.00483168
CYLC2	0.638373258	-0.660513594	0.224011344	-2.948572076	0.003192457	0.004822936
GAREM2	70.75598194	-0.177413815	0.060158222	-2.949119996	0.003186802	0.004814765
MBP	2226.20514	-0.104683049	0.035492914	-2.949406988	0.003183844	0.004810667
ESAM	316.1125136	-0.260232901	0.088218944	-2.949852832	0.003179253	0.004804101
IKZF2	973.6323284	-0.255772335	0.086690623	-2.950403708	0.00317359	0.004795913
AC069444.2	0.292489667	-0.446822979	0.151415499	-2.950972529	0.003167751	0.00478746
MTMR3	1025.886864	0.128929108	0.043684482	2.951370882	0.003163668	0.004781658
SPTBN1	14081.08694	0.167600229	0.056770678	2.952232286	0.003154855	0.004768706
ANP32E	2288.401596	-0.126441518	0.042825376	-2.952490527	0.003152217	0.004765087
C11orf91	5.454469893	-0.286024264	0.096864986	-2.95281376	0.003148919	0.004760469
DSG4	0.42698206	-0.573978624	0.19434471	-2.95340493	0.003142894	0.004751727
NKAIN1	8.421833668	-0.300905891	0.10186153	-2.954068044	0.003136148	0.004741895
SNF8	719.3123551	0.080689073	0.027314103	2.954117617	0.003135645	0.0047415
RABEP1	1409.397548	-0.08113117	0.027460497	-2.954468349	0.003132083	0.00473648
GABRA6	0.46958849	-0.989585089	0.334908357	-2.954793655	0.003128782	0.004731854
TEF	99.31770548	-0.157993536	0.053468514	-2.954889223	0.003127813	0.004730754
NTSR2	0.315250302	-0.672074131	0.2274198	-2.955213802	0.003124524	0.004726145
RPL38	15152.75559	-0.246167387	0.08329874	-2.955235413	0.003124306	0.004726145
RAB5B	2342.648411	-0.11421208	0.038641022	-2.955721019	0.003119391	0.00471911
IP6K1	2922.180204	0.129562282	0.043829453	2.956055193	0.003116013	0.004714364
PDE6A	37.05050635	0.255559508	0.086452095	2.956082296	0.003115739	0.004714314
TNMD	0.308472321	-0.58344294	0.197282556	-2.957397517	0.003102479	0.004694613
PTGFRN	122.2415765	0.297192234	0.100478085	2.957781632	0.003098615	0.004689129

BIRC2	2228.721511	0.09973712	0.033719701	2.957829272	0.003098137	0.004688767
IBA57	824.5740447	-0.114395789	0.038669801	-2.958271978	0.00309369	0.0046824
PYGL	4572.819073	-0.14979526	0.050603465	-2.960177906	0.003074614	0.004653888
APOF	14.23564026	-0.22019148	0.074379858	-2.960364348	0.003072754	0.004651432
LACC1	80.80312611	-0.132344895	0.044703088	-2.960531397	0.003071088	0.00464927
HBQ1	136.6233356	-0.313035886	0.105720303	-2.960981749	0.003066601	0.004642836
CAPG	3110.57827	-0.218275177	0.073716821	-2.960995507	0.003066464	0.004642836
MEGF6	2638.366507	-0.198205324	0.066926288	-2.961546636	0.003060981	0.004635045
S100A2	14.59910498	0.222199882	0.075028259	2.961549224	0.003060956	0.004635045
KIF2A	2228.512443	0.094830922	0.032008184	2.962708591	0.003049451	0.0046183
PGLYRP1	864.3424556	-0.361021578	0.121795237	-2.96416827	0.003035023	0.004596804
PRSS45P	0.296103941	-0.53770443	0.181370035	-2.964681731	0.003029962	0.004589495
HNRNPD	7559.092763	0.092896548	0.031319243	2.966117253	0.003015855	0.00456848
GPR32	18.05675766	-0.353896147	0.119296458	-2.966526855	0.003011841	0.004562752
GANAB	9716.441853	-0.104197623	0.035121398	-2.966784601	0.003009317	0.004559282
PTF1A	0.444684444	-0.547858096	0.184620614	-2.967480623	0.003002512	0.004549324
ARL2BP	39.19791302	-0.111526154	0.037570308	-2.96846522	0.00299291	0.004535126
KDSR	912.4717331	0.096282991	0.032420938	2.969778097	0.002980149	0.00451614
CDCP1	189.0260732	-0.276161699	0.09298848	-2.9698485	0.002979466	0.004515455
CYB5B	692.044254	-0.105779039	0.035615515	-2.970026954	0.002977736	0.004513182
TCFL5	282.618919	-0.118968695	0.040053548	-2.970241124	0.002975661	0.004510386
STX18	851.2373916	0.086575001	0.029144983	2.970494131	0.002973211	0.004507021
VPS54	1021.410531	-0.08174176	0.027501445	-2.972271416	0.002956052	0.004481358
FAM170B	0.717309197	-0.498966978	0.167797974	-2.973617423	0.002943118	0.004462095
DBNL	4601.652389	-0.092405971	0.031074597	-2.973681997	0.002942499	0.004461502
CFAP99	16.27370971	0.227643687	0.076543389	2.974047641	0.002938994	0.004456534
AMOTL1	655.4582022	-0.229966551	0.077310392	-2.974587836	0.002933824	0.004449039
KRTAP5-1	3.692903462	0.307174921	0.103248296	2.975108862	0.002928846	0.004441833
GRB14	1.859810164	-0.398010057	0.133746235	-2.975859883	0.002921683	0.004431314
RCAN2	4.67001159	-0.292714048	0.098352083	-2.976185545	0.002918582	0.004426954
UTS2	23.44986014	-0.407693507	0.136959214	-2.976751225	0.002913203	0.004419137
NUGGC	16.08896035	-0.214003978	0.071880956	-2.977199952	0.002908942	0.004413016
KIF20B	1167.959737	0.123783913	0.0415745	2.97739993	0.002907045	0.00441048
RPL5	36132.1836	0.135025043	0.045341688	2.977944783	0.002901883	0.004402988
SKA1	242.2046284	-0.165771021	0.055632264	-2.979764038	0.002884705	0.004377264
NOB1	879.1927706	-0.118507115	0.039768971	-2.979888891	0.00288353	0.00437582
HMG20B	1597.255305	-0.10715498	0.035957919	-2.980010645	0.002882384	0.00437442

NF1	3723.718128	-0.114430877	0.03839503	-2.980356456	0.002879131	0.004369823
RAB6D	2.682386818	0.268522087	0.090093813	2.98047199	0.002878046	0.004368514
APOBEC3B	126.6861072	-0.193552182	0.064929772	-2.980946584	0.002873589	0.004362088
CRKL	5559.522898	0.096319636	0.032303403	2.981717898	0.00286636	0.004351451
EIF5A2	127.8838341	0.220932003	0.074057858	2.983235139	0.002852188	0.004330272
KDM2B	2641.944811	0.077549392	0.025986765	2.984187971	0.00284332	0.004317145
CHTOP	2974.218032	0.067031486	0.022456176	2.98499118	0.002835865	0.004306159
DPP10	82.18694683	-0.520549495	0.174352782	-2.985610489	0.002830129	0.004297782
FBXO47	6.882064572	-0.347186706	0.116282454	-2.985718764	0.002829127	0.004296594
ELOA	1460.343311	0.098763932	0.033062753	2.987165978	0.002815768	0.004276638
AADACL4	0.476972168	-0.528949682	0.177059057	-2.987419508	0.002813434	0.004273424
CDK15	49.03805478	-0.340195344	0.113828285	-2.988671441	0.002801933	0.004256285
RAB7A	5058.733447	0.090247641	0.030190689	2.989254125	0.002796594	0.004248505
CHD8	6017.167599	0.07914917	0.026476112	2.989455859	0.002794748	0.00424603
ETFB	268.1780339	-0.141893994	0.047459186	-2.98981094	0.002791502	0.004241427
TACC2	30.1937684	-0.336070208	0.112399268	-2.989967938	0.002790067	0.004239577
WRN	1659.962252	-0.128361736	0.042927598	-2.990191443	0.002788026	0.004236805
GNAZ	42.75593487	-0.269960341	0.090265413	-2.990739548	0.002783028	0.004229536
CDCA5	771.0118498	-0.133755961	0.044710007	-2.991633643	0.002774891	0.004217497
SLC47A1	41.23609008	0.330949956	0.110603985	2.992206434	0.002769689	0.004209919
TLR5	97.89151245	0.288653493	0.096458887	2.992502822	0.002767001	0.00420616
AC091959.1	0.940969378	-0.3435848	0.114778268	-2.993465627	0.002758286	0.004193237
DPPA5	1.018547512	-0.366829278	0.122525917	-2.993891301	0.002754441	0.004187717
STXBP1	117.2017728	-0.236817098	0.079093577	-2.994138163	0.002752213	0.004184655
CD9	1200.01692	0.269387311	0.089948961	2.994890733	0.002745432	0.004174669
ECSCR	1.700025467	-0.295589519	0.098652342	-2.996274712	0.002733001	0.00415609
H1-0	5420.571761	0.253717726	0.084669859	2.996553064	0.002730507	0.00415262
CDH26	141.5972197	-0.190733501	0.063643592	-2.996900333	0.002727399	0.004148215
HHLA2	31.7959265	0.237527218	0.079249422	2.997210731	0.002724623	0.004144315
ZSWIM2	0.701209902	-0.499329644	0.166585711	-2.997433824	0.00272263	0.004141605
KCNK2	0.540079734	-0.583059163	0.19441245	-2.999083468	0.002707931	0.004119566
INSYN1	2.874007489	-0.279686416	0.09325482	-2.999163113	0.002707223	0.004118809
MRPS24	41.72308372	0.160391543	0.053477311	2.999244697	0.002706498	0.004118027
CCDC110	1.838787282	-0.337122113	0.112388608	-2.999611073	0.002703245	0.004113397
IFIH1	1593.538932	0.161191987	0.053717529	3.000733487	0.002693302	0.004098585
RSPO3	1.899365776	-0.415360434	0.138416061	-3.000810967	0.002692617	0.004097861
TMEM200C	0.780362959	-0.569238945	0.189540965	-3.003250224	0.002671127	0.004065472

YTHDF2	3857.159332	0.080887811	0.026933016	3.003295757	0.002670727	0.00406518
MOB3C	623.9613238	-0.128099204	0.042647658	-3.003663247	0.002667504	0.004060589
GMCL2	5.956989305	-0.265249226	0.088302617	-3.003865966	0.002665727	0.0040582
KLK3	0.940294582	-0.53660926	0.178609566	-3.004370212	0.002661313	0.004051795
NOP10	1290.441943	-0.204861551	0.068169388	-3.005183968	0.002654203	0.004041284
CLASP2	3322.742426	-0.114600534	0.038109624	-3.007128478	0.002637283	0.004015834
SPO11	0.541481524	-0.495394675	0.164717449	-3.007542171	0.002633696	0.004010685
FAM126A	716.0389326	0.126303167	0.041994276	3.007628151	0.002632951	0.004009862
KSR1	1950.418457	0.149771158	0.049792987	3.007876532	0.0026308	0.004006898
ANAPC2	1604.625017	-0.097662991	0.032460611	-3.008661526	0.002624013	0.003996872
SLAMF9	19.33579994	-0.310132418	0.103052101	-3.009472046	0.002617022	0.003986533
TRPV2	1282.413037	-0.126912504	0.042166596	-3.009787772	0.002614303	0.003982702
TECPR1	1147.777143	-0.09935107	0.032998678	-3.010759067	0.002605955	0.003970294
DCLK1	4.870879307	-0.370133785	0.122917006	-3.011249599	0.002601749	0.003964194
H2AC18	131.5247704	0.249446758	0.082831552	3.011494437	0.002599652	0.003961306
SMC6	770.2289201	-0.108312567	0.035962523	-3.011817822	0.002596884	0.003957397
CYBA	8879.441271	-0.182680015	0.060636077	-3.01272812	0.002589108	0.003945854
PRKAB1	1500.884784	0.103041655	0.034199897	3.012922939	0.002587446	0.003943629
NUP153	6482.215884	0.106540471	0.035360635	3.012968216	0.00258706	0.003943348
PATL2	428.8642262	-0.120685229	0.04005325	-3.013119493	0.002585771	0.003941689
ROPN1L	90.89275347	-0.186240834	0.061806131	-3.013306778	0.002584175	0.003939564
NPHS2	1.276386225	0.551504616	0.182981007	3.013999241	0.002578285	0.00393089
BRF1	1423.934083	-0.107953798	0.035815817	-3.014137534	0.002577109	0.003929404
GPR155	176.7532647	0.180499295	0.05987903	3.01439909	0.002574889	0.003926324
RWDD3	51.18658371	0.133219102	0.044173196	3.015835703	0.002562721	0.003908074
IL22RA1	1.844695439	-0.423467773	0.140405789	-3.016027864	0.002561097	0.003905903
SDC2	215.4247266	0.41883093	0.138856593	3.01628407	0.002558934	0.003902908
M6PR	2006.575658	-0.095323819	0.031581674	-3.018326957	0.002541745	0.003876993
MTSS2	503.4809917	0.168318679	0.055764825	3.018366475	0.002541414	0.00387679
SACM1L	1760.506331	-0.102327421	0.033897569	-3.018724477	0.002538413	0.003872514
AC004076.1	2.243916476	-0.241010302	0.079838138	-3.018736502	0.002538312	0.003872514
BCAP29	77.69305441	0.116468596	0.038566886	3.019911856	0.002528483	0.003857966
POU3F1	10.96278747	-0.276360696	0.09150268	-3.020247027	0.002525686	0.003853999
TPH1	40.30405412	-0.142171402	0.047064815	-3.020757713	0.00252143	0.003847806
RPS26	9574.738883	-0.291347398	0.096434466	-3.021195756	0.002517785	0.003842543
GPATCH2L	5528.171195	0.097634706	0.032314717	3.02136964	0.00251634	0.003840636
GBX2	0.277321803	-0.757522501	0.250702971	-3.021593634	0.002514479	0.003838095

PARP14	5639.08112	0.151884984	0.050248386	3.022683832	0.002505439	0.003824594
SETD3	1858.311103	0.065221951	0.02156058	3.025055536	0.002485875	0.003795026
BMP6	102.1498417	0.245232593	0.081066898	3.025064479	0.002485801	0.003795026
PRR35	7.399755079	-0.277435405	0.091694809	-3.025639163	0.002481082	0.0037883
ISY1	559.1557192	0.098775242	0.032641311	3.026080723	0.002477462	0.003783067
VIPR2	10.32531138	-0.280964818	0.092837126	-3.026427363	0.002474623	0.003779027
CLVS2	2.24526413	-0.681295561	0.225047645	-3.027339212	0.002467169	0.003767939
C18orf63	0.730980808	-0.552729427	0.182551768	-3.027795532	0.002463447	0.003762548
PEAR1	262.1815597	-0.254464702	0.084020202	-3.028613308	0.002456789	0.003752672
OR6C75	0.964429573	-0.403722796	0.133286233	-3.028990978	0.00245372	0.003748276
CCNH	1784.955089	0.101255653	0.033420828	3.029717116	0.002447829	0.003739569
MANSC4	9.082289066	-0.167928782	0.055425469	-3.029812538	0.002447056	0.003738679
DPEP2NB	12.70560877	-0.199117632	0.065711387	-3.030184581	0.002444043	0.003734368
LDLR	4265.254504	0.202938535	0.066955829	3.030931537	0.002438005	0.003725433
PNISR	7952.049459	0.099333076	0.032772071	3.03102838	0.002437223	0.003724529
IFNB1	10.89156036	-0.318321746	0.105011737	-3.031296821	0.002435057	0.00372151
CHRNE	114.3114261	0.175470398	0.057877602	3.031749607	0.002431408	0.003716223
SLC46A2	29.11996019	0.224709239	0.074114478	3.031920937	0.002430028	0.003714404
MCHR2	0.641103557	-0.543489939	0.179253471	-3.031963267	0.002429688	0.003714173
NOL12	133.6967318	-0.116793167	0.038507716	-3.032980883	0.002421509	0.003701961
C1orf54	146.6434197	-0.258876567	0.085342668	-3.033377939	0.002418325	0.003697382
HIGD2B	5.803568443	-0.262821893	0.08663352	-3.033720602	0.00241558	0.003693473
CYP2A6	45.64813673	-0.23441957	0.077256771	-3.03429157	0.002411013	0.003686778
CERS4	455.6224458	0.218603765	0.07203541	3.034670958	0.002407982	0.003682431
CAMSAP1	1585.286689	0.097973413	0.032275841	3.035502991	0.002401348	0.003672573
PHB2	4472.686719	-0.12587365	0.041462378	-3.035852212	0.002398569	0.003668609
AC092042.3	0.701193638	-0.52631701	0.173348712	-3.036174904	0.002396003	0.003664971
PABPC4L	27.73211665	-0.644629946	0.212275294	-3.036763883	0.002391327	0.003658104
RPL4	61945.12824	0.128284855	0.042232941	3.037554413	0.002385063	0.003648808
XPR1	1157.177513	0.085828543	0.028255087	3.037631529	0.002384453	0.003648159
NUPR1	15.92456405	-0.227711998	0.074962351	-3.037684853	0.002384031	0.003647799
SLC25A21	19.20180522	-0.368347797	0.121218515	-3.038709032	0.002375942	0.003635706
TUBA1B	3965.637009	-0.160105818	0.052682633	-3.039062534	0.002373156	0.003631726
FOXO6	28.32461208	-0.240605215	0.079159413	-3.039502283	0.002369694	0.003626712
PDE6C	24.39239351	-0.173522214	0.057086517	-3.039635689	0.002368645	0.00362539
CBLC	1.507290434	-0.352469935	0.11594829	-3.039889023	0.002366653	0.003622625
NXF1	13772.34842	0.120526005	0.039635482	3.040861362	0.002359024	0.00361123

SBSPON	28.8090698	-0.185185214	0.06089308	-3.041153684	0.002356735	0.003608007
ZFP82	141.6943251	-0.156525383	0.051465061	-3.041391225	0.002354876	0.003605444
TSKU	18.26100787	-0.293853677	0.096613521	-3.04153782	0.00235373	0.003603971
SERINC5	1877.705677	0.157334104	0.051713859	3.042397301	0.002347019	0.003593976
CNKSR2	218.6918624	0.249026706	0.081805294	3.044139246	0.002333471	0.00357351
HIGD2A	1354.52906	-0.198507678	0.065205216	-3.044352749	0.002331816	0.003571254
POC1B	735.5548226	-0.098848239	0.032467764	-3.044504054	0.002330643	0.003569737
KRT13	5.935922401	-0.376279371	0.123589197	-3.044597582	0.002329918	0.003568907
MS4A12	0.634317207	-0.518990664	0.170441527	-3.044977789	0.002326975	0.003564677
KANK3	81.32881762	-0.181314237	0.059541838	-3.045156875	0.00232559	0.003562834
MMP14	1021.362091	0.313147117	0.102833018	3.04520009	0.002325256	0.003562601
DBX2	0.451749464	-0.540919958	0.177568922	-3.046253554	0.002317123	0.003550419
NT5C2	2894.973246	0.08875684	0.029133303	3.046576586	0.002314635	0.003546884
CD244	1114.875673	-0.177937985	0.058403393	-3.046706302	0.002313637	0.003545632
EVPL	468.5745308	-0.290030086	0.095189416	-3.046873267	0.002312352	0.00354394
ZNF274	1464.150109	0.108594573	0.035632584	3.047619971	0.002306614	0.003535424
HMGN4	1053.563743	0.114713583	0.037623536	3.048984643	0.002296162	0.003519679
EXOC5	1307.572299	0.085561589	0.028061411	3.049083676	0.002295405	0.003518795
FTO	1690.817929	-0.178897229	0.058670052	-3.049208635	0.002294451	0.003517607
FAM124B	299.66265	-0.225617571	0.07398973	-3.049309284	0.002293682	0.003516704
PRRX2	2.941069213	-0.384681456	0.126121585	-3.050084226	0.002287772	0.003507917
ABHD12B	197.2450207	-0.192834037	0.063221605	-3.050128759	0.002287433	0.003507672
AL049697.1	28.68682013	-0.159503815	0.05228918	-3.050417214	0.002285237	0.003504579
C1QL1	3.505839085	-0.326413078	0.106977194	-3.051239854	0.002278984	0.003495264
GNAQ	5693.44545	-0.086280766	0.028274918	-3.051494864	0.002277049	0.00349257
ADCY8	1.692429778	-0.37904916	0.12419211	-3.052119496	0.002272316	0.003485583
GPRC6A	0.845497124	-0.654164149	0.214323039	-3.052234379	0.002271447	0.003484523
NUTM2G	148.6689423	-0.171294763	0.056117521	-3.052429272	0.002269972	0.003482534
TARDBP	2217.909175	0.096053843	0.031452025	3.053979581	0.002258275	0.003464859
HGFAC	16.0320883	-0.270805567	0.088662442	-3.054343658	0.002255535	0.003460928
SNX3	3558.770507	0.122300905	0.040033704	3.054948556	0.002250991	0.003454226
ADGRD2	27.29289255	-0.299062555	0.097893327	-3.054984057	0.002250725	0.003454088
CSRP1	1601.448295	-0.152191719	0.04981478	-3.055151857	0.002249466	0.003452427
OR7C2	0.602429136	-0.542404245	0.177507861	-3.055663229	0.002245633	0.003446815
PTPN9	980.7723083	-0.109297122	0.035765026	-3.055977747	0.002243279	0.003443471
BTBD3	984.860247	-0.1143716	0.037416746	-3.056695549	0.002237915	0.003435506
OSR1	1.238248657	-0.327050327	0.106976047	-3.057229506	0.002233932	0.003429661

EVX1	0.869991556	-0.393410573	0.128662299	-3.057698915	0.002230436	0.003424562
SLC5A6	1041.19101	-0.129051037	0.042204096	-3.057784681	0.002229797	0.003423851
COX7A1	108.8421636	0.165203698	0.054021666	3.058100753	0.002227447	0.00342051
ZNF629	1135.937832	0.118080458	0.038609808	3.058302153	0.00222595	0.00341848
OAT	1305.049058	-0.18972807	0.062031348	-3.058583713	0.002223859	0.003415537
KLRC2	5.991988064	0.352499558	0.115183578	3.06032825	0.002210945	0.003395969
CPEB3	318.9494584	-0.10763808	0.03517171	-3.060359587	0.002210714	0.00339588
BZW2	2057.272164	-0.107306959	0.03506016	-3.060652266	0.002208554	0.003392829
DHX29	1314.851745	0.09934805	0.032457013	3.060911632	0.002206642	0.003390157
CCNE2	225.6149108	-0.125475031	0.040988987	-3.061188904	0.0022046	0.003387285
ANKRD34C	0.758268586	-0.443835256	0.144974483	-3.061471563	0.002202519	0.003384354
NCF1	1145.193348	0.249444943	0.081470718	3.061774199	0.002200294	0.0033812
PDCD6IP	3831.844043	-0.066450943	0.021697805	-3.062565158	0.002194487	0.003372542
OR52E5	0.438468258	-0.846303763	0.276320823	-3.06275782	0.002193075	0.003370636
MUSTN1	2.926944018	-0.251696235	0.082134182	-3.064451725	0.002180694	0.00335187
DDR1	109.4198958	0.265983206	0.086707367	3.067596392	0.002157879	0.003317062
PXYLP1	177.5822397	0.154408149	0.050323208	3.068328815	0.002152596	0.003309202
GTF2IRD2	198.3266206	-0.130453521	0.042513983	-3.068485014	0.002151471	0.003307733
TMEM219	1612.187793	-0.146349161	0.047684179	-3.069134568	0.002146799	0.003300808
SNAP25	6.836358646	-0.618029632	0.201351409	-3.069408035	0.002144834	0.003298047
GNG11	79.47453467	-0.37622999	0.122559681	-3.069769651	0.002142239	0.003294316
CIZ1	3363.85597	-0.102780373	0.033480636	-3.069845285	0.002141697	0.00329374
CDK2AP2	652.4281191	-0.176988315	0.057647831	-3.070164365	0.00213941	0.003290482
AP000356.5	7.603731306	-0.175066661	0.057019597	-3.070289312	0.002138515	0.003289364
C1orf210	2.688595791	-0.238904324	0.077766065	-3.072089672	0.002125659	0.003269846
SPDYE3	153.2181861	-0.160732165	0.052312667	-3.072528595	0.002122535	0.003265297
RAB11A	2304.12938	0.087846862	0.028581981	3.073504967	0.002115602	0.003254887
AF196969.1	11.16090316	0.166736237	0.054249483	3.073508344	0.002115578	0.003254887
PEX5L	38.90705142	-0.255943805	0.083271914	-3.073590975	0.002114992	0.00325446
CLPTM1	2169.88644	0.094899851	0.030874997	3.073679646	0.002114363	0.003253749
ARFGAP1	2553.299863	0.108845753	0.035399569	3.074776212	0.002106606	0.003242066
HEBP1	790.4506807	-0.123042368	0.039995475	-3.076407251	0.002095115	0.003224635
CD2AP	1721.188623	0.145647441	0.047319518	3.077956974	0.00208425	0.003208165
BRWD1	6917.559563	0.120706047	0.039210204	3.078434587	0.002080912	0.003203279
HELQ	436.1871434	-0.087614543	0.028451713	-3.079411919	0.002074097	0.003193039
CHGA	42.72384681	-0.244145987	0.079276642	-3.079671144	0.002072293	0.003190512
TREM2	27.97420481	-0.343978067	0.111671574	-3.080265231	0.002068163	0.003184405

RPS11	40811.25961	-0.233260972	0.075722585	-3.080467623	0.002066758	0.003182492
CDA	761.0276291	0.293418351	0.095248899	3.080543226	0.002066234	0.003181935
PGAM4	42.46008113	0.170517859	0.055340059	3.081273559	0.002061172	0.00317439
LIN54	884.4378669	0.089135032	0.028926702	3.081410076	0.002060227	0.003173184
ZNF264	1821.103126	0.123722572	0.040144656	3.081918833	0.002056709	0.003168015
SEC24C	2684.584197	0.09533941	0.030928706	3.082554076	0.002052324	0.00316151
IGSF8	503.1500047	-0.130915351	0.042465327	-3.082876339	0.002050103	0.003158337
NFE2L3	868.4872654	-0.206233531	0.066870794	-3.084059866	0.002041965	0.003146047
OR6T1	0.422369305	-0.928146042	0.300944558	-3.084109739	0.002041623	0.003145767
GTF3C6	564.245202	-0.162726104	0.052760565	-3.084237323	0.002040747	0.003144666
BBX	2794.963779	0.124684816	0.040426414	3.08424127	0.00204072	0.003144666
LUM	26.570097	-0.422295941	0.13691633	-3.084335818	0.002040072	0.00314412
SP4	936.6749313	0.108461954	0.035164438	3.084421624	0.002039483	0.003143461
BMP4	8.011994273	-0.448609908	0.145432472	-3.084661228	0.002037841	0.003141177
KIF26A	272.6392849	-0.292005887	0.094621789	-3.086032189	0.002028469	0.003126976
KCNN2	65.79470195	-0.718892614	0.232943279	-3.086127309	0.00202782	0.003126222
PPP2R2D	1877.205424	0.082136175	0.026613942	3.086208532	0.002027266	0.003125614
CAMSAP2	65.01290197	0.247824131	0.080272268	3.087294494	0.002019874	0.003114463
SLC25A53	106.74349	-0.107934585	0.034958237	-3.087529454	0.002018278	0.003112247
FABP5	250.7983614	-0.187527563	0.060728155	-3.087983867	0.002015194	0.003107737
FBRSL1	3392.060385	-0.109613259	0.035488269	-3.088718134	0.002010221	0.003100311
SULT1C4	133.1772364	-0.272104479	0.088080886	-3.08925683	0.002006579	0.003094939
NR3C2	90.60400301	0.213639053	0.069148058	3.089588609	0.002004339	0.003091727
LDHAL6B	6.021340976	-0.299779348	0.097027521	-3.089632151	0.002004045	0.003091518
COL15A1	28.94178763	-0.294587981	0.095343475	-3.089755012	0.002003217	0.003090483
SNX33	546.2095665	0.191299327	0.061909456	3.089985581	0.002001662	0.003088328
UBE2V1	953.035614	0.124476651	0.040277599	3.090468528	0.00199841	0.003083553
DACH1	1674.241411	-0.325034816	0.105165897	-3.090686492	0.001996944	0.003081534
ABCC8	11.95511033	-0.348677528	0.112807215	-3.090915133	0.001995407	0.003079405
FILIP1L	354.4918497	0.193147845	0.062487235	3.090996836	0.001994858	0.0030788
TCF25	4081.953637	0.07185909	0.023242915	3.091655693	0.001990436	0.003072218
PKN2	2495.96461	0.104999868	0.033954309	3.092387137	0.001985537	0.003064899
ICA1L	645.780075	0.119711807	0.038711459	3.092412695	0.001985366	0.003064877
TP53I11	878.6220154	0.2149651	0.069512834	3.09245198	0.001985103	0.003064713
BAZ1B	3618.266205	-0.07367808	0.023824748	-3.092501948	0.001984769	0.003064439
ARHGAP23	1406.991522	0.15505365	0.050129755	3.093046214	0.001981133	0.003059065
FGL2	1282.836016	0.273432039	0.088398811	3.093164208	0.001980345	0.003058091

GABARAPL2	2189.1925	-0.11828256	0.038234884	-3.093577064	0.001977592	0.00305408
RARS2	837.6572559	0.08713883	0.028166406	3.093714849	0.001976674	0.003052903
SYCP3	54.30866284	-0.176377742	0.05700577	-3.094033144	0.001974554	0.00304987
CELF3	16.01343275	-0.257850526	0.083316147	-3.094844588	0.00196916	0.003041779
P3H3	69.79976945	-0.281635931	0.090983658	-3.095456226	0.001965104	0.003035752
GRK2	20361.17716	0.100585081	0.032486969	3.096166964	0.001960399	0.003028723
CNTN6	0.674051272	-0.981335976	0.3167206	-3.098428007	0.001945502	0.003005945
SIVA1	2102.798659	-0.120513645	0.03888351	-3.099350948	0.001939451	0.002996833
MED8	413.2258212	0.091548151	0.02953427	3.099726164	0.001936996	0.002993276
PLPP6	98.90359201	-0.132794414	0.042838237	-3.099903812	0.001935835	0.002991717
USP9X	9948.01088	0.131382976	0.042375694	3.100432447	0.001932383	0.002986618
RPL6	23731.32674	0.159794888	0.05153843	3.100499717	0.001931944	0.002986175
PCNX3	3347.516712	-0.104568101	0.033726003	-3.100518598	0.001931821	0.002986175
CNIH4	957.259596	0.138826462	0.044772951	3.100677064	0.001930787	0.002984859
PHLDA3	108.8687933	-0.255315878	0.082322542	-3.101409061	0.00192602	0.002977725
DEFB136	0.514552018	-0.82882395	0.267027589	-3.103888827	0.001909951	0.002953114
ARHGAP21	1492.932372	0.239715862	0.077229042	3.103960059	0.001909491	0.002952636
DRG2	948.8937899	-0.07366185	0.023728586	-3.104350656	0.001906972	0.002948974
GAA	2116.584206	0.202803384	0.065328138	3.104380298	0.001906781	0.002948911
MARVELD1	794.006162	-0.239484293	0.077141036	-3.104499298	0.001906014	0.002947958
CHST10	266.5773315	-0.164624674	0.053025914	-3.104607971	0.001905314	0.002947108
NDUFB4	1481.46873	-0.192328616	0.061923318	-3.105915842	0.001896907	0.002934336
DKK2	15.04326437	-0.437365115	0.140806163	-3.106150367	0.001895403	0.002932242
SERTM2	12.81565699	-0.471401268	0.151669587	-3.108080394	0.001883068	0.00291339
FBXW7	1979.426581	0.092310334	0.0296982	3.108280396	0.001881794	0.002911649
NLRP14	3.148040959	-0.466147812	0.149960324	-3.10847429	0.00188056	0.002909969
TAC4	69.14382873	-0.218281512	0.070211897	-3.108896378	0.001877876	0.002906045
ASB14	284.4562127	0.170108906	0.054711228	3.109213798	0.001875859	0.002903154
SHISA7	87.13166736	-0.263466401	0.0847314	-3.109430524	0.001874484	0.002901255
NIFK	439.058046	-0.089305926	0.028719588	-3.1095824	0.00187352	0.002899993
ZNF724	98.75082478	-0.128919424	0.041456837	-3.109726466	0.001872607	0.002898808
SRSF2	4475.894159	-0.10568518	0.033982879	-3.109953719	0.001871167	0.002896808
INSR	2819.146764	-0.154903649	0.049806658	-3.110099214	0.001870245	0.00289561
CELA2B	35.64896478	0.219069612	0.070436409	3.110175766	0.00186976	0.002895089
AL365232.1	0.669915118	-0.649746618	0.208907735	-3.110208531	0.001869553	0.002894997
ADAM18	0.496052724	-0.790849864	0.254196737	-3.111172368	0.001863461	0.002885792
ZNF275	871.2049406	0.116894799	0.037566594	3.111668785	0.001860331	0.002881172

PSG7	0.442383872	-0.716975217	0.230373872	-3.112224539	0.001856832	0.002875981
AEBP2	1364.847455	0.091842077	0.029502178	3.113060925	0.001851578	0.00286807
AC008073.3	5.290655733	-0.278045637	0.089287432	-3.114051216	0.001845375	0.002858687
CYP27C1	5.980304102	-0.348499452	0.111904698	-3.114252203	0.001844118	0.002856967
PIGR	20.48228782	-0.283095696	0.090850947	-3.116045613	0.001832939	0.002839873
CCDC126	195.2901977	0.1387809	0.044534919	3.116226641	0.001831814	0.002838355
OSBPL2	2354.794676	0.117937354	0.037843028	3.116488296	0.001830189	0.002836062
IRAG1	113.0639048	-0.232738214	0.074672286	-3.116795091	0.001828286	0.002833337
CALB2	3.324401226	-0.452239784	0.145080154	-3.117171941	0.00182595	0.002829941
NYAP1	11.39500899	-0.236685153	0.075925087	-3.117351108	0.001824841	0.002828446
BRPF3	1464.606747	0.108894627	0.034915523	3.118802715	0.001815875	0.002814772
MYO1G	9937.251813	0.137832851	0.044189965	3.119098455	0.001814053	0.002812171
NSUN7	39.45824434	0.281745773	0.09032598	3.119210813	0.001813362	0.002811321
GUCA2A	1.952347483	-0.483727204	0.15503787	-3.120058378	0.001808152	0.002803466
GJA8	0.418878944	-0.526984617	0.168703564	-3.123731391	0.001785734	0.002768927
LUC7L	2753.946626	-0.090114202	0.028839384	-3.124692336	0.001779911	0.002760117
CNGA1	8.684324564	-0.281266439	0.090011532	-3.124782259	0.001779367	0.002759492
ZNF367	493.6124371	-0.139052318	0.044462635	-3.127397169	0.001763615	0.00273528
ASMTL	1149.47436	-0.09128869	0.029187718	-3.127640534	0.001762156	0.002733233
MS4A7	703.5162127	0.247657761	0.079178458	3.12784269	0.001760944	0.002731571
ABHD14B	1375.0685	-0.149260252	0.047713967	-3.128229762	0.001758626	0.002728192
NACA	15368.31746	0.17117046	0.054713575	3.128482461	0.001757115	0.002726063
BABAM1	536.6487253	-0.083292432	0.0266225	-3.12864806	0.001756125	0.002724743
STX6	644.8937099	-0.119732168	0.038269166	-3.128685034	0.001755904	0.002724616
SFMBT2	3036.102759	0.151173757	0.048314349	3.128961889	0.001754251	0.002722266
CLIC3	34.92934171	0.220104836	0.070331947	3.129514306	0.001750955	0.002717368
ZNF670	65.04135041	-0.170624076	0.054519507	-3.129596846	0.001750464	0.002716821
DCTN5	2172.818987	0.072420491	0.02313104	3.13087915	0.001742839	0.002705201
SCAMP4	1047.796917	-0.13068022	0.041720511	-3.132277558	0.001734558	0.002692561
FOXC1	772.3660059	-0.435389013	0.138975382	-3.132849913	0.001731179	0.00268753
MANSC1	99.35672588	-0.244691667	0.078097285	-3.133164837	0.001729323	0.002684861
UBE4A	2318.779244	0.084133832	0.026852496	3.133184771	0.001729205	0.002684861
QPRT	514.3158484	-0.180029529	0.057456099	-3.133340607	0.001728288	0.002683679
BPIFC	1.683699129	-0.445343925	0.142059308	-3.134915479	0.001719036	0.002669525
FAM20A	187.6561681	0.255077526	0.081352455	3.135461945	0.001715837	0.002664768
SND1	6922.705746	-0.083036898	0.026473434	-3.136612305	0.00170912	0.002654547
C11orf42	20.79118534	0.22279197	0.070999038	3.137957582	0.001701295	0.002642603

SF3A3	2827.526076	0.096708382	0.030811024	3.138759122	0.001696649	0.002635595
ACTBL2	0.470652465	-0.629813902	0.20064895	-3.138884614	0.001695922	0.002634676
OTOP3	1.070362293	-0.382249033	0.121763027	-3.139286559	0.001693597	0.002631273
AC040162.1	439.594724	0.126144699	0.040175509	3.139840703	0.001690397	0.00262651
RCN1	431.6538029	0.117569821	0.037439567	3.140255912	0.001688003	0.002622998
TMEM62	474.7307474	0.088202585	0.028086292	3.140413962	0.001687093	0.002621791
NEURL1	139.2297339	-0.199943887	0.063665044	-3.140559961	0.001686252	0.002620693
CYP39A1	0.41698732	-0.674313871	0.214675692	-3.141081616	0.001683251	0.002616237
LEMD1	0.549722092	-0.579575535	0.184513745	-3.1410968	0.001683164	0.002616237
LAIR2	23.37734941	0.222609063	0.070833937	3.142689423	0.001674034	0.002602324
CCDC25	357.8235608	-0.126258567	0.040172899	-3.142879181	0.001672949	0.002600844
ADPRM	425.9343001	0.120919785	0.038470544	3.143178446	0.001671239	0.002598393
PTPRM	330.3639984	0.343955442	0.109428235	3.14320562	0.001671084	0.002598358
ABRA	17.5374725	-0.273678945	0.087019539	-3.145028659	0.001660706	0.002582426
GPR182	16.58075033	-0.191452216	0.060868595	-3.14533654	0.001658959	0.002579914
PRKAG1	772.0715728	-0.08023564	0.025506859	-3.145649566	0.001657184	0.00257736
SARM1	207.6574551	-0.182176907	0.057912066	-3.145750415	0.001656613	0.002576676
ATG4D	1230.750464	0.128818938	0.040942266	3.146355846	0.001653187	0.002571552
KDM4A	909.8480253	-0.108434409	0.034460612	-3.146618737	0.001651702	0.002569445
TTC32	452.285695	0.098431513	0.031252333	3.149573254	0.001635091	0.002543807
ZNF45	432.0260818	-0.094563192	0.030023591	-3.149629605	0.001634776	0.002543519
KHDRBS3	79.40684138	-0.291073357	0.092381093	-3.150789286	0.001628299	0.002533643
LRP2BP	91.07539477	0.239388286	0.075961199	3.151454833	0.001624593	0.002528077
FOXI3	0.950434824	-0.342257724	0.108599893	-3.15154752	0.001624077	0.002527476
ANKRA2	461.928314	0.081058984	0.025719789	3.151619339	0.001623678	0.002527055
TUBA1C	1409.682153	-0.176281381	0.055927679	-3.151952402	0.001621827	0.002524375
LYVE1	24.12219444	-0.310651063	0.098483312	-3.154352322	0.001608548	0.002503905
NUP210L	293.3005945	-0.232718977	0.073773833	-3.154492134	0.001607777	0.002502905
SLC36A2	9.01987337	-0.314346624	0.099614554	-3.155629489	0.001601522	0.002493365
H1-6	20.74341266	-0.250419215	0.079354523	-3.155701842	0.001601124	0.002492945
STMP1	1764.877573	-0.192488236	0.060983354	-3.156406171	0.001597263	0.00248713
TMEM74	4.243592328	-0.395476344	0.125282738	-3.156670661	0.001595815	0.002485073
ABHD12	1320.286758	0.100005296	0.031679782	3.156754524	0.001595356	0.002484556
TARS1	2933.02049	0.102640427	0.032510779	3.157119891	0.001593358	0.002481642
WDR5B	344.328925	-0.1473141	0.04665757	-3.157346191	0.001592122	0.002479914
SNX13	1600.923033	0.127972514	0.040530616	3.157428286	0.001591674	0.002479414
FCER2	423.7305719	0.293073058	0.092819668	3.157445669	0.001591579	0.002479414

TCP11	19.03830286	-0.205868092	0.065190474	-3.157947493	0.001588842	0.002475396
DIPK2A	1552.499277	-0.169353071	0.053625564	-3.158066046	0.001588196	0.002474586
SLA2	902.7754199	0.182044535	0.05761661	3.159584267	0.001579944	0.002461925
NTNG2	2141.870629	0.265690591	0.084088434	3.159656764	0.001579551	0.002461509
MFN1	1379.207196	0.108994877	0.034477498	3.161333684	0.001570485	0.002447575
SPATA2L	390.0741803	0.135800106	0.042951713	3.16169248	0.001568551	0.002444756
CRAT	629.8433991	-0.148705716	0.047030864	-3.161875079	0.001567568	0.002443419
KLHDC8A	73.50045124	-0.385531945	0.121885569	-3.163064721	0.001561176	0.002433649
CLPS	0.988771024	-0.455464679	0.143984385	-3.163292174	0.001559957	0.002431942
NODAL	34.33827756	-0.225213079	0.071176532	-3.164147944	0.001555377	0.002424996
BTNL8	28.11229915	-0.322943566	0.102060957	-3.16422239	0.001554979	0.002424569
VDAC3	1985.669668	-0.087632871	0.027689113	-3.164885463	0.00155144	0.002419243
QRSL1	492.038036	-0.080473539	0.025425886	-3.165024029	0.001550701	0.002418284
EPHA3	9.976091429	-0.591559693	0.186895651	-3.165187051	0.001549833	0.002417122
EPB41L1	38.46735039	0.256410846	0.080991796	3.165886652	0.00154611	0.002411508
FCRL2	92.31602536	0.282188975	0.089127492	3.166127173	0.001544832	0.002409707
KLHL11	868.7238489	0.130844523	0.041322614	3.166414442	0.001543307	0.00240752
AC013394.1	707.0750583	0.187290005	0.059148259	3.166450021	0.001543119	0.002407418
MYOT	39.72992917	-0.256076418	0.080866386	-3.166660844	0.001542	0.002405865
KCNK17	268.2195382	-0.361985085	0.114291545	-3.16720792	0.001539102	0.002401535
RPL12	22960.9394	-0.228079511	0.07199604	-3.167945241	0.001535204	0.002395643
KDM4B	3350.160603	-0.12527533	0.039541815	-3.168173487	0.001534	0.002393954
TAS2R46	35.84098966	-0.237411584	0.074921518	-3.168803712	0.001530677	0.00238896
MKLN1	3030.883357	0.131614888	0.041532756	3.168941858	0.00152995	0.002388015
TMEM245	2041.803391	-0.098893574	0.031199994	-3.169666412	0.00152614	0.002382259
GALNT17	16.20045794	-0.600246529	0.189315079	-3.170621859	0.00152113	0.002374628
ZNF460	2308.409335	-0.151638468	0.047824249	-3.170744387	0.001520489	0.002373816
DICER1	4196.756615	0.115094935	0.036298031	3.17083133	0.001520034	0.002373295
MAP1LC3A	392.7390401	0.236517119	0.07456435	3.171986601	0.001514	0.002364062
LAMTOR5	1496.144622	0.119901526	0.037798631	3.17211296	0.001513341	0.002363222
TARM1	24.99242957	-0.354096511	0.111610185	-3.172618259	0.00151071	0.002359302
DLEC1	466.4210918	0.194594013	0.061332635	3.17276457	0.001509949	0.002358301
GTPBP2	2296.03191	-0.111599897	0.035162657	-3.173818657	0.001504477	0.002349942
ZNF469	444.0736264	-0.207198902	0.065277451	-3.174126715	0.001502881	0.002347636
ZNF354B	242.1349234	-0.119817983	0.037746145	-3.174310507	0.001501929	0.002346337
PSTPIP2	2258.255617	-0.143743726	0.04528124	-3.174465358	0.001501128	0.002345273
OR4B1	0.440167948	-0.66725926	0.210192671	-3.174512493	0.001500884	0.002345079

TUBA3C	0.96149644	-0.480267759	0.151273386	-3.174833136	0.001499227	0.002342677
IGBP1	1736.693889	0.101662064	0.03201949	3.175005749	0.001498335	0.00234147
ZBTB25	905.3900554	0.114024534	0.035909583	3.175323269	0.001496697	0.002339096
TMEM220	292.2996965	-0.113811132	0.035841493	-3.17540156	0.001496293	0.002338652
NCOR1	10804.97247	-0.094665625	0.029808313	-3.175812877	0.001494173	0.002335525
ALKAL2	32.27455269	0.22382995	0.070478945	3.175841392	0.001494026	0.002335482
TIAM1	272.9501016	0.282029269	0.088791552	3.176307466	0.001491628	0.002331919
MTF2	2333.637917	0.109298178	0.034395684	3.177671306	0.00148463	0.002321164
HDAC4	2163.356292	-0.167864291	0.052824225	-3.177789963	0.001484022	0.0023204
TEX30	254.5020311	-0.180052495	0.05665209	-3.178214531	0.001481851	0.00231719
CDK1	404.7365327	-0.200462713	0.063069198	-3.178456679	0.001480613	0.00231544
C11orf97	9.3795502	-0.325169893	0.102302734	-3.178506394	0.00148036	0.002315228
THNSL2	67.46483665	-0.352335501	0.110820676	-3.179330016	0.001476159	0.002308843
TTC8	210.9435987	-0.193218759	0.060772605	-3.179372674	0.001475942	0.002308688
RAI14	35.62132582	-0.358834449	0.112836607	-3.180124418	0.001472118	0.002302891
IKBKG	227.4252149	-0.102094629	0.032099114	-3.180605829	0.001469674	0.002299252
XCL2	22.54883692	0.292671001	0.092000253	3.181197786	0.001466675	0.002294742
FOSL1	906.5692333	0.223070091	0.070101605	3.182096754	0.00146213	0.002287814
OR11A1	0.537757296	-0.713890851	0.22430624	-3.182661582	0.001459281	0.002283539
CRIP3	16.44092699	-0.280368424	0.088089453	-3.182769486	0.001458737	0.00228287
SMIM9	1.462226169	-0.322065356	0.101119485	-3.184997996	0.00144755	0.002265544
LRRK1	2748.96961	-0.154674322	0.048546274	-3.186121359	0.001441941	0.002256946
PARP12	1429.881385	0.133981715	0.042036445	3.187275117	0.001436201	0.002248141
C6	0.467441476	-0.691950294	0.217091856	-3.187361827	0.00143577	0.002247647
DSCAML1	18.50068937	-0.259382674	0.081375889	-3.187463487	0.001435266	0.002247037
CETN2	304.5793345	-0.185924329	0.058306746	-3.188727562	0.001429005	0.002237414
ELOA3D	0.498500357	-0.609107672	0.19100776	-3.188915849	0.001428074	0.002236136
C1QTNF4	1865.223207	-0.319391659	0.100141362	-3.189407973	0.001425645	0.002232511
CATIP	37.86299418	0.215026474	0.067413601	3.189660114	0.001424402	0.002230743
PFN2	32.66908075	0.208093122	0.065228613	3.190212253	0.001421683	0.002226663
ATP5F1E	4320.769588	-0.261657096	0.082009733	-3.190561491	0.001419966	0.002224152
POGZ	4944.047246	0.074409681	0.023318186	3.191057853	0.001417529	0.002220512
TTLL9	36.02855307	-0.201167349	0.063040247	-3.191093898	0.001417352	0.002220412
TNFAIP8L1	767.6026516	0.138116847	0.043276938	3.191465338	0.001415531	0.002217737
ACOT2	260.7524647	-0.128366548	0.040218052	-3.191764474	0.001414066	0.002215619
ETV6	7608.616265	-0.106576546	0.03339028	-3.191843419	0.001413679	0.002215191
AGAP6	56.95445867	-0.162877474	0.051027334	-3.191965218	0.001413083	0.002214434

MAPKAPK5	1445.272368	-0.065652874	0.020560257	-3.193193218	0.001407088	0.002205215
GTF2E1	178.8329054	-0.124730092	0.039059672	-3.193321539	0.001406462	0.002204411
PRDM7	9.74085363	0.335047951	0.104878587	3.19462686	0.001400117	0.002194642
GPR42	1.036461307	0.473923917	0.148244419	3.196909001	0.001389087	0.002177527
SIDT1	445.6456546	0.155436595	0.048617025	3.197163876	0.001387861	0.002175778
GJB7	48.32907825	-0.233859237	0.073131517	-3.197790061	0.001384851	0.002171234
ZFAND1	918.5166214	0.117267349	0.036666394	3.198224197	0.001382767	0.002168141
FAM131A	433.1338139	0.126000117	0.039388863	3.198876735	0.001379641	0.002163413
OR52M1	1.037726116	0.627657901	0.196168	3.199593715	0.001376214	0.002158212
SPTBN5	164.4158996	-0.200096199	0.062505056	-3.201280227	0.001368184	0.002145791
MSGN1	0.855031654	-0.682870272	0.213237279	-3.202396298	0.001362894	0.002137665
SPRR2F	0.442356397	-0.871455129	0.272065302	-3.203110147	0.00135952	0.002132544
EMC6	22.1020403	-0.172615957	0.05386698	-3.204485499	0.001353041	0.002122551
PLCD4	65.96257422	-0.200860573	0.062677615	-3.204662005	0.001352212	0.00212142
LRIG2	1229.385976	-0.107820216	0.033641896	-3.204938741	0.001350912	0.002119552
GPR68	79.24649434	-0.230982893	0.072069724	-3.204992075	0.001350662	0.002119329
ZNF396	36.15214274	-0.163763334	0.05108076	-3.205968988	0.001346085	0.002112316
ZBTB41	486.8472836	0.123445253	0.038500849	3.206299504	0.00134454	0.002110061
IL2RA	142.0311875	-0.325791159	0.101607928	-3.206355695	0.001344277	0.002109818
CNOT4	1500.960443	0.103066363	0.03213874	3.206919869	0.001341644	0.002105853
ARHGAP1	3337.258798	-0.106474588	0.0331976	-3.207297761	0.001339882	0.002103257
HNRNPA2B1	25794.42089	-0.087174051	0.02717629	-3.207724444	0.001337896	0.002100308
ARL6IP6	582.5645415	-0.107808291	0.033607591	-3.207855368	0.001337287	0.002099521
NSDHL	265.3872332	-0.089707866	0.027961167	-3.208301977	0.001335212	0.002096431
RAB27A	2150.438143	0.150635618	0.046933873	3.209528833	0.001329527	0.002087672
PPDPF	2642.636662	-0.308073121	0.095971538	-3.210046721	0.001327134	0.002084082
KRTAP12-3	0.723946581	-0.595780571	0.185555405	-3.210796102	0.001323678	0.002078822
GRAMD1C	48.23022551	-0.230054651	0.071650335	-3.210796597	0.001323676	0.002078822
MS4A4A	152.4530109	0.33967393	0.105784765	3.210991002	0.001322781	0.002077746
VDAC2	3062.669094	0.100985804	0.031449786	3.211017186	0.00132266	0.002077723
PHRF1	2327.148602	-0.113201547	0.035251914	-3.211217012	0.001321741	0.002076445
CCND1	145.2707744	-0.297558563	0.092657807	-3.211370654	0.001321034	0.002075502
PTGDR2	95.68608595	-0.262309233	0.081661614	-3.212148543	0.001317462	0.002070056
LRRC37B	1666.291441	0.099242443	0.03088576	3.213210359	0.001312601	0.002062584
PTHLH	2.21410525	-0.415164889	0.129187724	-3.213655883	0.001310566	0.002059552
ZBTB7B	2126.784404	0.172556149	0.053689816	3.213945637	0.001309245	0.00205764
RBBP7	3334.910384	0.063656959	0.019801438	3.214764469	0.001305516	0.002051944

SUMO3	3010.1082	0.137916177	0.042897195	3.215039491	0.001304266	0.002050144
ABLIM1	1840.334885	0.233849967	0.072691032	3.217040137	0.001295205	0.002036065
LMO2	2966.598366	-0.132019702	0.041018783	-3.218518243	0.001288548	0.002025763
UQCRC1	3282.428976	-0.114830678	0.035677843	-3.218543184	0.001288436	0.002025749
GNG8	4.656396157	0.301014565	0.093523659	3.218592682	0.001288213	0.002025562
OVOL1	11.79009965	0.318069598	0.098810812	3.218975667	0.001286494	0.002023021
VCX2	0.417982016	-0.447105443	0.138892156	-3.219083461	0.00128601	0.002022424
PUS3	109.9661563	-0.115479434	0.035864166	-3.219911328	0.001282303	0.002016754
ZNF728	0.565982321	-0.636444615	0.197623221	-3.220495096	0.001279694	0.002012813
ATP5MGL	42.94637921	-0.185519807	0.057568415	-3.222597095	0.001270341	0.001998263
SLC22A7	4.749085275	-0.294493753	0.091369695	-3.223100959	0.001268108	0.001994911
SEPTIN8	348.4730018	-0.174951552	0.054274099	-3.223481462	0.001266425	0.001992423
PRL	27.84395719	-0.659853829	0.204687882	-3.223707351	0.001265426	0.001991012
CHMP6	354.9573321	-0.131662798	0.040838073	-3.224020835	0.001264042	0.001988994
NSMCE2	485.3156052	-0.083117951	0.025780766	-3.224029554	0.001264004	0.001988994
EMD	1835.971336	0.118418634	0.036710702	3.225725137	0.00125654	0.001977507
B4GALT3	1076.150653	-0.094632221	0.029332831	-3.226153682	0.00125466	0.001974707
DLX4	33.03376635	-0.27254617	0.084468289	-3.226609325	0.001252664	0.001971724
ZGRF1	844.0250213	-0.129664039	0.040182271	-3.226896736	0.001251406	0.001969903
MS4A5	0.9411149	-0.44341016	0.137407693	-3.226967494	0.001251097	0.001969574
TMEM184C	530.9478201	-0.086690051	0.026863937	-3.227004764	0.001250934	0.001969476
AP2S1	1278.918709	-0.1898178	0.058815387	-3.227349328	0.001249428	0.001967264
FZD2	147.7100545	-0.281250982	0.087139275	-3.227602968	0.001248321	0.001965679
ARL1	595.9233173	0.088096834	0.027291995	3.227936746	0.001246865	0.001963545
TMUB2	1631.766855	0.07311611	0.022643086	3.229069949	0.001241935	0.001955938
ZNF689	540.5826995	0.093446441	0.028935491	3.229474816	0.001240178	0.001953328
MICA	420.545559	-0.09929417	0.030741161	-3.230007192	0.001237871	0.001949851
SMTNL2	3.447902436	-0.290237587	0.089852768	-3.230146318	0.001237269	0.001949059
OR8G5	0.899662123	0.620259383	0.192012539	3.23030666	0.001236575	0.001948123
UGT1A8	0.46721872	-0.93478376	0.289370821	-3.230400901	0.001236167	0.001947638
ODF3L2	1.138546966	-0.343718007	0.106378333	-3.231090356	0.001233189	0.001943102
EZH1	1818.87279	-0.109452965	0.033873195	-3.231255971	0.001232475	0.001942133
MIF4GD	868.1521709	-0.111698917	0.034541982	-3.233714789	0.001221914	0.001925646
TNRC6A	3364.806879	0.119717749	0.03701946	3.233913973	0.001221062	0.001924459
RHBDF2	1540.382995	0.16117282	0.049831259	3.234371812	0.001219106	0.001921531
HES3	2.616293217	0.459972069	0.142195027	3.234797158	0.001217292	0.001918825
CTSW	3319.171811	-0.273318756	0.084490065	-3.234921828	0.00121676	0.001918142

ARSJ	3.221618186	-0.374062417	0.115591039	-3.236084908	0.001211813	0.001910497
IRS4	0.552705648	-0.560150745	0.17307884	-3.236390676	0.001210516	0.001908605
HOOK1	89.72093448	-0.299074445	0.092396771	-3.236849544	0.001208571	0.001905693
TMEM126B	354.8748578	-0.094838826	0.029287311	-3.238222347	0.00120277	0.001896699
PAQR7	555.8971055	-0.133115778	0.041103589	-3.238543931	0.001201415	0.001894714
TMTC2	592.8953855	-0.308313051	0.095199537	-3.238598222	0.001201187	0.001894506
RAB11FIP1	8484.973831	0.161871876	0.049975121	3.23904922	0.001199289	0.001891666
TDRD1	5.977806765	-0.427497717	0.131974314	-3.239249389	0.001198447	0.001890491
MBD1	2804.827993	0.072395401	0.02234562	3.239802761	0.001196124	0.001886978
ATF7IP	5082.169156	-0.09928352	0.030644737	-3.239822877	0.00119604	0.001886978
SYPL2	4.890991706	-0.309513797	0.095522603	-3.240215274	0.001194395	0.001884554
MS4A8	0.773520394	-0.474056824	0.146277643	-3.240801641	0.001191941	0.001880834
STX2	377.5905853	0.165408684	0.051035412	3.241057121	0.001190873	0.0018793
G6PC2	2.892136916	-0.463890944	0.143116181	-3.24135915	0.001189612	0.001877461
PPP6R1	9521.882877	-0.102190857	0.031524354	-3.241647969	0.001188407	0.001875711
KRT3	1.263375884	-0.530179065	0.163551386	-3.241666599	0.00118833	0.001875711
TMEM216	308.6182997	-0.143854544	0.044375736	-3.241738739	0.001188029	0.001875416
ACADS	581.7279119	-0.106281963	0.032779453	-3.242334906	0.001185546	0.001871648
ARHGAP19	766.2157672	0.115810158	0.035715525	3.242571947	0.00118456	0.001870243
BUD13	584.1283168	0.09525443	0.029374139	3.242799023	0.001183617	0.001868904
EPHA1	431.2858439	0.174475196	0.053803634	3.242814358	0.001183553	0.001868904
GKAP1	172.4952458	-0.141439805	0.043604114	-3.243726136	0.001179771	0.001863132
EPHB2	68.57694408	0.364423006	0.112346489	3.243741814	0.001179706	0.001863132
ZBED6CL	462.6739179	-0.166359192	0.051276772	-3.244338257	0.001177239	0.001859433
IFI44	1334.460802	0.243988349	0.075201333	3.244468415	0.001176701	0.001858733
SBF2	1135.113782	0.160628636	0.049506721	3.244582399	0.00117623	0.001858139
MRPL9	565.3898177	0.122526297	0.037758908	3.244963975	0.001174655	0.001855801
EXOC1	886.918962	-0.109220324	0.033656752	-3.245123677	0.001173996	0.00185491
RNF130	4696.31675	0.126824002	0.039056857	3.247163529	0.001165614	0.001841815
OR5H14	0.519497108	-0.550748574	0.169586146	-3.247603572	0.001163813	0.001839118
RAB6A	1983.920076	0.087683834	0.026993969	3.248274964	0.00116107	0.001834931
KLK7	0.642496134	-0.509914633	0.156938476	-3.24913716	0.001157556	0.001829526
CIAO2B	603.2240746	-0.204864559	0.063033649	-3.25008247	0.001153715	0.001823603
UQCRH	2741.041615	-0.192563703	0.059245015	-3.250293767	0.001152859	0.001822395
PDLIM1	3767.484151	-0.238440366	0.073355109	-3.25049433	0.001152046	0.001821258
GOLGA8G	1.180154388	-0.393915013	0.121162795	-3.251121873	0.001149506	0.001817389
CREB3L3	13.52530423	-0.328318755	0.100976577	-3.25143478	0.001148241	0.001815536

SETD5	6186.836205	0.104065872	0.032004623	3.251588691	0.00114762	0.0018147
PSMA2	345.773287	0.150685284	0.046340143	3.251722468	0.00114708	0.001813993
TRIM25	4001.769708	0.127412344	0.039180311	3.25194821	0.001146169	0.001812699
KIAA1586	498.742631	-0.140007547	0.043052747	-3.25200029	0.001145959	0.001812514
CD46	4571.145492	0.084907658	0.026103875	3.252684118	0.001143205	0.001808304
INPP5K	1634.729219	0.097757504	0.030051057	3.253047155	0.001141745	0.001806141
AOC3	81.97716854	-0.227897957	0.070052016	-3.253267632	0.00114086	0.001804886
CRMP1	27.30382738	-0.442489051	0.135977937	-3.254123881	0.001137427	0.0017996
GPR151	9.074528548	-0.291202121	0.089475101	-3.254560394	0.00113568	0.001796982
IL36B	0.765656522	-0.688655306	0.211542011	-3.255406819	0.0011323	0.001791779
NLGN2	219.868881	-0.231630993	0.071149574	-3.255549962	0.00113173	0.001791021
MARF1	5399.702896	0.107976508	0.033155121	3.256706829	0.001127128	0.001783882
SLC7A11	415.8158844	0.305532845	0.093803833	3.257146717	0.001125382	0.001781264
GCFC2	321.6595333	-0.10240781	0.031439732	-3.2572736	0.00112488	0.001780612
SMIM22	11.72652556	-0.230656157	0.070809336	-3.257425767	0.001124277	0.001779802
SEPTIN9	5694.491298	0.123534347	0.03792253	3.257544968	0.001123805	0.001779198
INPP5A	753.4173972	-0.136367107	0.041856708	-3.257951091	0.001122197	0.001776798
ADGRE2	3154.554616	0.178828539	0.054880596	3.258502144	0.00112002	0.001773494
SLX1B	1.26009443	-0.308540654	0.094680641	-3.258751225	0.001119037	0.001772081
TRIP10	201.180536	0.228293979	0.070052122	3.258915958	0.001118388	0.001771196
ADH1C	0.437981981	-0.65764478	0.201795515	-3.25896628	0.00111819	0.001771025
PIK3AP1	4099.054985	0.148447657	0.045548132	3.259138228	0.001117512	0.001770095
FUBP3	1372.522408	0.087520156	0.026853591	3.259160197	0.001117426	0.001770095
CBX3	3266.326999	0.085318697	0.02617736	3.259255205	0.001117051	0.001769652
UTRN	8581.027272	-0.162285657	0.049790751	-3.25935345	0.001116665	0.001769182
MED27	500.2272169	-0.082136714	0.025197677	-3.259693834	0.001115325	0.001767203
GALP	0.993116446	-0.582974829	0.178799671	-3.260491612	0.001112193	0.001762382
SERTM1	1.890785048	-0.506092096	0.155191381	-3.261083783	0.001109872	0.001758848
LSM8	1316.910936	0.108933935	0.033396318	3.261854639	0.001106859	0.001754214
ZNF525	282.0178046	-0.152754984	0.046821027	-3.262529544	0.001104227	0.001750184
BRINP2	4.451666104	0.821002299	0.251537838	3.263931605	0.001098777	0.001741688
ATL2	2034.116521	0.107369387	0.032886137	3.2648829	0.001095093	0.00173599
ANP32D	1.274341496	-0.275032836	0.084233117	-3.265138995	0.001094104	0.001734561
SIGLEC12	182.5474246	-0.340069145	0.104134365	-3.265676454	0.001092029	0.001731413
MAP3K9	154.1258836	0.230581818	0.070599543	3.266052571	0.00109058	0.001729255
MEIS1	2783.855024	-0.40019999	0.122531955	-3.266086704	0.001090449	0.001729187
HOXD12	0.847526188	0.909319419	0.278402749	3.266201294	0.001090007	0.001728627

ALDH2	620.3527177	-0.259519122	0.079409118	-3.268127516	0.001082616	0.001717044
PXDN	894.2531877	0.48443372	0.148170257	3.269439689	0.001077607	0.001709239
ZNF366	13.6798532	-0.356140132	0.108929532	-3.269454359	0.001077551	0.001709239
TMA7	520.3170418	-0.196022689	0.059954967	-3.269498717	0.001077382	0.001709159
NPLOC4	5336.890129	0.07449359	0.022784115	3.269540723	0.001077222	0.001709044
NTSR1	124.2252914	0.297160796	0.090882172	3.269736946	0.001076475	0.001707997
SAFB	4061.913282	0.093894317	0.028712911	3.270107921	0.001075065	0.001705897
GRM5	0.518333314	-0.562368797	0.171962895	-3.270291513	0.001074367	0.001704928
UNC93A	0.401079222	-0.833994744	0.254849906	-3.272493826	0.001066032	0.001691839
RDH10	153.3413099	0.166473135	0.050870343	3.272498743	0.001066013	0.001691839
TCHHL1	1.126962238	-0.45650584	0.139476496	-3.272994747	0.001064145	0.001689117
CDK5RAP1	1147.192851	0.079235313	0.024201758	3.273948617	0.001060559	0.001683562
CDC34	1087.368991	-0.137664758	0.042044532	-3.274260696	0.001059388	0.00168184
FBXO40	53.15589517	0.177079997	0.054078797	3.274481079	0.001058562	0.001680665
AKAP17A	4563.795037	0.123848087	0.037818501	3.274801566	0.001057362	0.001678896
GTSF1	783.3965024	-0.300626451	0.091776761	-3.275627167	0.001054276	0.001674132
ORC1	912.6012729	-0.125237494	0.038228096	-3.276058909	0.001052666	0.001671711
SNX22	326.3113991	-0.189017574	0.057696249	-3.276080801	0.001052584	0.001671711
TMEM114	0.67864423	-0.526824178	0.160770693	-3.276867	0.001049658	0.001667204
PARM1	22.23613989	0.243088675	0.07418167	3.276937194	0.001049397	0.001666925
STAT2	3535.815462	-0.122050635	0.037241207	-3.277300716	0.001048047	0.001664915
TIMM44	1031.848038	-0.094318603	0.028769226	-3.278454626	0.001043771	0.001658258
ZNF428	415.8748353	-0.138198915	0.042143376	-3.279255912	0.001040812	0.001653691
PGRMC1	682.7427631	0.140440131	0.042825746	3.279338812	0.001040506	0.001653339
SLC13A1	0.615915508	-0.664074321	0.202485759	-3.27961	0.001039507	0.001651885
ZNF740	1156.495261	-0.116115002	0.035404744	-3.279645344	0.001039377	0.001651812
ZNF865	1091.01028	0.114465476	0.03489366	3.280409011	0.001036567	0.001647481
LZTS2	1154.746441	-0.160725738	0.048975084	-3.281785892	0.001031519	0.001639591
EYS	118.2660809	-0.258381656	0.078690277	-3.283527102	0.001025168	0.001629628
DDHD1	2237.688725	0.132297034	0.040284138	3.284097421	0.001023096	0.001626466
DOT1L	3652.183363	0.118476583	0.036071167	3.284523171	0.001021551	0.001624142
CEP112	25.67213156	-0.280994499	0.085549206	-3.284595076	0.00102129	0.00162386
RIMS4	1.47224866	-0.364362075	0.110924475	-3.284776198	0.001020634	0.001622948
LONRF2	48.87943847	-0.303173496	0.092276226	-3.285499513	0.001018017	0.001618919
CRYBA1	80.42495553	-0.270931471	0.082436806	-3.286535268	0.001014281	0.001613108
ZNF836	95.56084339	-0.132732529	0.040377863	-3.287259879	0.001011674	0.001609093
LDLRAP1	493.8305788	-0.159547333	0.048524028	-3.288006746	0.001008994	0.001604961

SNTB2	174.8837797	-0.203897257	0.062008674	-3.288205412	0.001008282	0.001603959
AC079447.1	15.96821332	-0.125245558	0.038086215	-3.28847482	0.001007318	0.001602555
CWC27	494.7190312	-0.101787308	0.030937541	-3.290090451	0.001001552	0.001593511
ZNF502	31.37810211	-0.27816708	0.08451261	-3.291426936	0.000996805	0.001586088
MYOZ2	1.569419824	-0.316939149	0.096280212	-3.291841007	0.000995339	0.001583883
SRSF9	1642.934655	0.071504763	0.021718136	3.292398685	0.000993367	0.001580874
SPP1	54.37661484	0.371298063	0.112772376	3.292455802	0.000993165	0.001580681
PLCG1	1511.115732	0.22693927	0.068919498	3.292816648	0.000991891	0.001578783
PHF13	681.0225253	0.177125174	0.053780554	3.293479905	0.000989554	0.001575191
ATP9A	467.7130909	-0.308631023	0.093702332	-3.293738981	0.000988643	0.001573868
FAT1	179.8374407	-0.444140907	0.134830651	-3.294064836	0.000987497	0.001572172
CERS3	5.892586983	-0.277454353	0.084223137	-3.29427713	0.000986752	0.001571113
HEXA	1864.89435	0.102450395	0.031089106	3.295379302	0.000982889	0.00156509
MT3	1.251249477	-0.436784735	0.132481972	-3.296937152	0.000977454	0.001556562
BEX2	127.1383741	-0.323102105	0.097983429	-3.297517847	0.000975435	0.001553473
A3GALT2	19.51763943	0.2328898	0.070625646	3.297524514	0.000975412	0.001553473
MOCS3	144.4561052	-0.128802895	0.039055634	-3.297933747	0.000973991	0.001551426
ME2	1964.363569	-0.090344539	0.02739182	-3.298230603	0.000972962	0.001549913
ACVRL1	63.15113821	0.335251423	0.101627629	3.298821635	0.000970916	0.00154678
CHCHD10	401.9272718	-0.272025789	0.082423663	-3.300336085	0.000965691	0.001538581
ADRB2	651.3803046	-0.244634416	0.074116432	-3.300677183	0.000964518	0.001536837
SMIM23	4.802309483	-0.324330942	0.098261497	-3.300692052	0.000964467	0.001536837
KRT222	0.550042343	-0.489661418	0.148340235	-3.300934636	0.000963633	0.001535678
ADD1	7825.791866	-0.070762129	0.021433965	-3.301401725	0.00096203	0.001533248
DNAJC21	1663.495149	0.071925594	0.021785758	3.30149598	0.000961707	0.001532858
ZFP37	8.411988685	-0.314714473	0.09532387	-3.301528489	0.000961596	0.001532805
APH1B	702.6652012	-0.09733585	0.029475521	-3.302260553	0.000959089	0.001528934
PTPN4	770.4461124	-0.136707426	0.04138651	-3.303188057	0.000955923	0.00152401
ALDH1L2	65.51805029	-0.241246347	0.073009413	-3.304318413	0.000952076	0.001518002
GPS2	56.67372066	-0.110705286	0.033498819	-3.304751874	0.000950605	0.00151578
PIGBOS1	155.3086163	-0.134555747	0.040706932	-3.305475001	0.000948156	0.001511997
FBLN2	234.7230555	-0.33922606	0.102613306	-3.305868138	0.000946826	0.00151
PPP4R4	7.29872577	0.381416	0.115315419	3.307588891	0.000941028	0.001500876
NCAPG	670.8950862	-0.173704794	0.052507687	-3.308178358	0.00093905	0.001497842
CNOT3	1482.996482	-0.116551435	0.035217028	-3.309519278	0.000934563	0.001490807
ADGRA1	0.770198733	-0.468909746	0.14168297	-3.30957028	0.000934393	0.001490657
IPO5	5611.121781	0.090793922	0.02741511	3.311820436	0.00092691	0.00147884

MAPK15	24.47238622	-0.306798244	0.092622515	-3.312350629	0.000925155	0.001476161
EFNA3	11.16469644	-0.212113571	0.064023432	-3.313061544	0.000922807	0.001472533
GNL1	2657.874035	0.113791191	0.034342564	3.313415748	0.000921639	0.00147079
ITGA7	606.7074417	-0.320452783	0.09669168	-3.314171231	0.000919152	0.001466941
UGT1A10	0.518781963	-1.019463766	0.307405117	-3.31635262	0.000912007	0.001455656
RNF41	1667.091453	0.078140023	0.023561629	3.316410001	0.00091182	0.001455476
NDC80	452.8381185	-0.141067104	0.042534896	-3.31650285	0.000911517	0.001455111
ABRACL	725.4680199	-0.177640306	0.053562344	-3.316514812	0.000911478	0.001455111
POLD3	589.6670858	-0.124195283	0.037439775	-3.317201599	0.00090924	0.001451713
KDM4F	0.433495222	-0.634110906	0.191049269	-3.319096215	0.000903093	0.001442017
PIK3R4	1801.422549	0.126237464	0.038030368	3.319385811	0.000902157	0.001440639
CC2D2B	75.23982164	-0.19363481	0.058328052	-3.319754461	0.000900967	0.001438856
NBPF6	1.278402171	-0.651356618	0.196176685	-3.320254986	0.000899353	0.001436396
DLL4	36.08132772	-0.302830096	0.09119901	-3.320541493	0.00089843	0.001435039
PRDX3	1700.398282	-0.1546105	0.046559954	-3.320675521	0.000897999	0.001434467
MSX2	8.195528938	-0.667438369	0.200921016	-3.321894251	0.000894086	0.001428333
KTN1	3486.414562	0.124750942	0.037546929	3.322533828	0.000892039	0.001425179
EFNA1	123.1721938	0.239513218	0.072085914	3.322607784	0.000891802	0.001424918
NLRP10	0.644316171	-0.479781768	0.144376571	-3.323127596	0.000890142	0.001422381
VPS26B	1717.27975	-0.084251399	0.025346708	-3.323958191	0.000887495	0.001418267
YTHDC2	1477.043599	0.109315802	0.03288009	3.324680805	0.000885198	0.001414712
DAO	3.293882004	-0.310098854	0.093225078	-3.326345891	0.000879927	0.001406402
FOXD2	8.906995067	-0.239833387	0.072093918	-3.326679882	0.000878873	0.001404833
NDUFA12	806.9691835	-0.203377794	0.061128434	-3.32705713	0.000877684	0.001403047
FRMD6	49.33339457	-0.307932441	0.092547226	-3.3273006	0.000876917	0.001401936
LONP2	3240.43992	-0.087139099	0.026179319	-3.328547249	0.000873002	0.00139579
SYCE3	4.176388523	0.327221914	0.098302854	3.328712236	0.000872485	0.001395078
SMC4	3376.232895	-0.129609052	0.038935992	-3.328772307	0.000872297	0.001394891
APP	2315.425657	0.339538389	0.101957166	3.330206208	0.000867817	0.001387841
SNU13	2366.591095	-0.134922935	0.040510462	-3.330570159	0.000866683	0.001386141
GIMAP1	89.03252674	0.195593169	0.058726302	3.330588875	0.000866625	0.001386141
ODF1	0.508977665	-0.5093908	0.152925284	-3.330978276	0.000865414	0.001384336
AQP4	2.481029955	-0.358787149	0.107691948	-3.331606094	0.000863464	0.00138133
COIL	753.8418671	0.107226251	0.032182328	3.331836336	0.00086275	0.001380301
OTOF	129.7946241	0.427863896	0.128410409	3.332003221	0.000862233	0.001379586
UNC119B	1210.52816	0.129162768	0.038763817	3.332044614	0.000862104	0.001379494
MAN1A2	2355.134532	0.086558291	0.025977099	3.33210001	0.000861933	0.001379332

THOC7	714.2097438	-0.150514237	0.045153739	-3.333372574	0.000858	0.00137315
NRIP1	4472.256432	0.201113966	0.060333249	3.333385298	0.00085796	0.00137315
ARSB	1191.94123	0.118563742	0.03556527	3.333694427	0.000857008	0.001371787
URI1	1475.589626	-0.087682701	0.026301516	-3.333750835	0.000856834	0.001371621
AFAP1L1	61.29392097	-0.329559857	0.098848868	-3.33397705	0.000856137	0.001370618
HIF1AN	2488.544604	-0.102823353	0.030837023	-3.33441244	0.000854798	0.001368586
SRPRB	667.1029806	-0.112974249	0.033880953	-3.334447252	0.000854691	0.001368527
HOXD3	0.349416095	-0.592013478	0.177537741	-3.33457819	0.000854289	0.001367994
ATRAID	1434.374976	-0.190241472	0.057041829	-3.33512225	0.000852619	0.001365432
C5orf52	0.559999076	-0.534056711	0.160121985	-3.33531158	0.000852039	0.001364615
MS4A3	2411.328756	0.293084438	0.087867534	3.335525909	0.000851382	0.001363675
CUL4B	1535.68694	0.086036601	0.025792857	3.335675444	0.000850924	0.001363053
TRH	2531.888433	0.484036643	0.145105047	3.335767098	0.000850644	0.001362715
KRT23	36.87388105	-0.457356884	0.137083889	-3.336328486	0.000848928	0.001360077
ZNF775	125.4263773	-0.14159808	0.042430114	-3.337207141	0.000846249	0.001355896
GPM6B	360.2237626	-0.268964994	0.080551029	-3.339063415	0.000840614	0.001346978
N4BP3	614.3141741	0.307408476	0.092028782	3.340351445	0.000836724	0.001340855
SLC35C2	1755.103273	-0.09881363	0.029580601	-3.340487517	0.000836314	0.001340308
PLEKHM3	1740.687537	0.106820648	0.031968451	3.341439577	0.000833452	0.001335829
PORCN	221.5199182	0.113468014	0.033935518	3.343635838	0.000826882	0.001325408
ZNF433	483.6746535	0.152986226	0.045746235	3.344236442	0.000825094	0.00132265
LIPT1	117.995775	0.118196536	0.035339982	3.344555667	0.000824145	0.001321237
SPDYE6	176.1135863	-0.161345933	0.048230136	-3.345334414	0.000821834	0.001317641
EDN1	38.67505612	0.316111265	0.094479849	3.345806203	0.000820437	0.001315509
MAP4	4224.348372	-0.101796596	0.030424576	-3.345867412	0.000820256	0.001315326
PPP1CA	3185.349896	-0.119695093	0.035772725	-3.345987599	0.000819901	0.001314864
MIP	9.106207764	-0.190997003	0.057072043	-3.346594797	0.000818107	0.001312095
LILRB3	879.0303398	0.23190144	0.069294275	3.346617608	0.00081804	0.001312095
EFHD1	5.58951173	-0.263517066	0.078731204	-3.34704732	0.000816773	0.00131017
CLEC2B	3082.902221	0.230952326	0.068993657	3.347442897	0.000815608	0.001308409
ZNF804B	0.395571676	-1.05932251	0.316414728	-3.347892553	0.000814286	0.001306395
CRP	0.53286866	-0.617645633	0.184443712	-3.348694434	0.000811933	0.001302727
SLC35D1	1135.189502	0.132804674	0.03965375	3.349107539	0.000810723	0.001300892
GDPD5	574.5296374	-0.199340098	0.059506574	-3.349883623	0.000808455	0.001297359
KCTD15	536.0123003	-0.216453489	0.064604029	-3.350464232	0.000806762	0.001294749
AL049634.2	1.8324767	-0.39539976	0.117992595	-3.351055708	0.000805041	0.001292093
P2RY11	207.0558328	-0.134659449	0.04016464	-3.352686568	0.000800313	0.001284609

LY6G6F	5.557934798	-0.394132614	0.117519489	-3.353763863	0.000797204	0.001279723
FGF21	0.550248818	-0.506942078	0.151130457	-3.354334323	0.000795562	0.001277192
COX5A	2155.28403	-0.205662304	0.061302844	-3.354857475	0.000794059	0.001274884
SCGB1D1	0.368208281	-0.788516126	0.234994825	-3.355461666	0.000792326	0.001272207
CLEC4D	240.1917437	0.379175499	0.112981449	3.356086365	0.000790539	0.001269441
CDC42EP5	6.194137295	-0.317941321	0.094716176	-3.356779543	0.00078856	0.001266367
TBC1D25	704.4120045	0.08478439	0.025255713	3.357038122	0.000787822	0.001265287
RPN1	7332.037258	0.092871007	0.027651745	3.358594816	0.000783398	0.001258285
GPR148	0.421621603	-0.648637595	0.193101702	-3.359046501	0.000782119	0.001256333
JUP	6588.083882	0.249784408	0.074351734	3.359496742	0.000780846	0.00125439
CTXND1	67.91161101	-0.241830431	0.07198079	-3.359652357	0.000780406	0.001253787
CIP2A	578.8824986	-0.136866449	0.040735749	-3.359860903	0.000779817	0.001252944
RPL37	35765.79615	-0.275352915	0.081951672	-3.359942599	0.000779587	0.001252676
RBSN	1495.045382	0.105869648	0.031505839	3.36031836	0.000778527	0.001251076
QSER1	1551.901651	-0.11244446	0.03345838	-3.360726401	0.000777378	0.001249332
ZNF34	240.3665343	-0.123878254	0.036856261	-3.3611183	0.000776276	0.001247663
TMEM239	1.653189499	-0.334262961	0.099446714	-3.361226806	0.000775971	0.001247276
MREG	138.0859081	0.26783062	0.079668704	3.361804669	0.000774349	0.001244771
ZNF600	308.5326359	-0.129889714	0.038632996	-3.362144442	0.000773397	0.001243342
AP003108.2	272.6996806	0.162115307	0.048217547	3.36216416	0.000773341	0.001243342
SLC16A11	12.18785115	-0.219547938	0.065285128	-3.362908903	0.000771258	0.001240108
VWA5B2	14.29168644	-0.26237437	0.078012134	-3.363250795	0.000770303	0.001238675
TNK2	3895.055553	-0.118243989	0.035156241	-3.363385411	0.000769928	0.001238173
SYNCRIP	5627.1381	0.085636302	0.025453634	3.364403732	0.000767092	0.001233714
PSCA	17.12391039	0.329515309	0.097940496	3.364443948	0.000766981	0.001233636
AC001226.2	258.1570148	-0.191478045	0.056905099	-3.364866215	0.000765808	0.00123185
GNMT	133.2353506	-0.157964354	0.04694126	-3.365149412	0.000765022	0.001230688
RBM44	188.8639564	0.181651612	0.053967841	3.365923273	0.000762879	0.001227341
GSAP	722.3655401	-0.184484972	0.054806149	-3.366136362	0.00076229	0.001226494
FBN2	651.1982149	0.361229159	0.107295423	3.366678191	0.000760794	0.001224188
NCAPG2	1508.193039	-0.137693958	0.040881719	-3.368105911	0.000756865	0.001217966
PLEKHG1	277.6359314	0.272723577	0.080961828	3.368545192	0.00075566	0.001216127
HARS2	610.767658	0.093243426	0.02768045	3.368566125	0.000755603	0.001216127
ZAR1L	15.39867726	-0.226083228	0.067034863	-3.3726216	0.000744562	0.001198463
DEPP1	368.3621625	-0.284291233	0.08427427	-3.373404873	0.000742447	0.001195157
ERV3-1	1142.738027	-0.111776253	0.033133997	-3.37346121	0.000742295	0.001195011
TDRD5	5.268197515	-0.50588477	0.149953125	-3.373619387	0.000741869	0.001194423

ARMCX5	245.4330535	0.113565151	0.033659809	3.373909558	0.000741087	0.001193262
BCAS1	9.017204752	-0.26393143	0.078225993	-3.373960759	0.000740949	0.001193139
SASH3	3138.241386	0.148220926	0.043921421	3.374684219	0.000739004	0.001190105
CNST	3028.686157	-0.131661089	0.039006853	-3.375332272	0.000737266	0.001187403
UPK3A	44.14835296	-0.447711774	0.132628311	-3.375687819	0.000736314	0.001185967
LGALS1	9906.1597	-0.318810308	0.094386166	-3.377722822	0.000730887	0.001177323
ATG16L1	1840.281996	0.11165364	0.033049854	3.378339859	0.000729249	0.001174781
CHRNB3	1.25744601	-0.437855212	0.129575416	-3.379153426	0.000727094	0.001171406
PHLDB2	77.78197176	0.264049633	0.078138858	3.379235888	0.000726876	0.001171151
ANKRD30BL	138.7473609	-0.425152527	0.125807076	-3.379400755	0.00072644	0.001170545
TMED4	2585.53704	0.064876564	0.019196457	3.379611304	0.000725884	0.001169746
TMSB4X	40225.95053	0.171737096	0.050801989	3.380519129	0.00072349	0.001165984
IRF9	656.9044525	0.190095744	0.056228235	3.380788012	0.000722783	0.00116494
C6orf89	1583.518481	-0.115079576	0.034036398	-3.381073916	0.000722031	0.001163824
C13orf46	107.8620444	0.243678116	0.072057178	3.381732714	0.000720302	0.001161132
RBP3	0.614219303	-0.449357286	0.132873716	-3.38183729	0.000720028	0.001160786
RPP30	696.34124	0.101377702	0.029965124	3.383189782	0.000716491	0.001155179
ESRRA	727.3049883	-0.126427147	0.037369105	-3.383199729	0.000716465	0.001155179
MOSPD2	409.8428122	-0.154911495	0.045786589	-3.383337742	0.000716105	0.001154747
HILPDA	578.548318	0.279494314	0.082597712	3.383802117	0.000714895	0.001152891
PCP2	64.53947003	-0.156957555	0.046382786	-3.38396131	0.000714481	0.001152318
EFCAB14	2656.857869	0.076553719	0.022621705	3.384082591	0.000714165	0.001151904
EPG5	5927.469602	0.161951627	0.047831195	3.385899701	0.000709453	0.001144398
NOTCH2NLC	245.4469076	0.1944658	0.057431402	3.386053484	0.000709056	0.001143851
CYP4F11	23.42062815	-0.35898092	0.106007764	-3.386364408	0.000708253	0.00114265
CLDN14	7.232287891	-0.353552174	0.104387586	-3.386917816	0.000706826	0.001140442
CNIH2	119.7553165	0.174612121	0.051551166	3.387161389	0.000706198	0.001139523
HNRNPA1L2	175.4283547	0.12972491	0.038274654	3.389316311	0.000700671	0.001130698
SLC35D2	130.3946552	0.155042003	0.045737776	3.38980194	0.000699431	0.00112879
UBOX5	523.6045278	0.100162438	0.029546833	3.389955156	0.000699041	0.001128253
DUSP28	485.9174583	0.118783167	0.035032133	3.390691791	0.000697165	0.001125318
FCN2	2.433995401	-0.402896293	0.118780723	-3.391933343	0.000694013	0.001120323
HNRNPU	23858.0658	-0.097035883	0.0286068	-3.392056487	0.000693701	0.001119912
ZC2HC1A	14.24472772	-0.335521239	0.098910262	-3.392178224	0.000693393	0.001119507
GABBR2	2.610411821	-0.3488678	0.102809707	-3.393335213	0.000690471	0.001114881
RETSAT	706.884643	-0.101973514	0.030049061	-3.393567351	0.000689886	0.001114028
STK10	5793.270639	-0.114926078	0.033865738	-3.393579604	0.000689855	0.001114028

ABCC3	93.03682395	0.296743114	0.087441603	3.393614761	0.000689767	0.001114019
CCDC121	62.37104304	-0.189178358	0.055744058	-3.39369548	0.000689563	0.001113783
VTCN1	3.201293367	-0.400482587	0.117970113	-3.394780064	0.000686838	0.001109472
TEX48	3.007409328	-0.359872798	0.105991209	-3.395307971	0.000685514	0.001107426
RASA4	92.40558847	0.160267341	0.047199006	3.395566051	0.000684868	0.001106474
OR2J3	0.386707205	-0.547570707	0.161245605	-3.39587989	0.000684084	0.001105297
STEAP1B	13.43059654	0.310257031	0.091350832	3.396324075	0.000682974	0.001103596
RIPK4	6.751151451	-0.389982751	0.114807688	-3.396834816	0.000681701	0.001101629
CATSPERD	37.97302014	-0.243417352	0.071648007	-3.397405763	0.00068028	0.001099424
ZNF268	326.4208594	-0.136449109	0.040152886	-3.398239148	0.000678211	0.001096171
ENTHD1	2.883845789	-0.421645708	0.124073216	-3.398362055	0.000677906	0.001095769
SPON1	94.87926297	-0.295872287	0.087056433	-3.398626353	0.000677252	0.001094801
TULP4	1139.868105	0.17987813	0.052894963	3.400666499	0.000672218	0.001086753
RTN4RL1	10.22759409	-0.314233018	0.092395956	-3.400939074	0.000671548	0.00108576
GDAP2	1047.730536	-0.123817594	0.036406833	-3.400943828	0.000671536	0.00108576
SLC22A4	809.8856591	0.184757248	0.054323986	3.401025241	0.000671336	0.001085597
ALG5	498.5357125	0.117521819	0.034553926	3.401113398	0.00067112	0.001085337
WASHC4	3010.868474	0.120431489	0.035404604	3.40157708	0.000669982	0.001083587
CAPN5	110.8108063	-0.240823573	0.070792399	-3.401828089	0.000669367	0.001082681
ANXA3	876.4483654	-0.354505305	0.104204694	-3.402008992	0.000668924	0.001082054
SMARCA1	73.10689513	0.397125554	0.116720115	3.402374589	0.00066803	0.001080697
AC013489.1	0.89190303	-0.342435515	0.100639824	-3.4025846	0.000667517	0.001079956
RSPH10B2	8.40983383	-0.209553524	0.061584112	-3.40272057	0.000667185	0.001079508
SLFN11	1016.334903	-0.186142276	0.054699922	-3.402971482	0.000666572	0.001078606
BST1	1037.870774	0.239473493	0.070354561	3.403809054	0.000664532	0.001075393
HTRA1	5.381738349	-0.403915971	0.118649117	-3.404289722	0.000663364	0.001073591
PPBP	550.7213507	0.419819204	0.123320007	3.40430733	0.000663321	0.001073591
PARD6G	41.85477214	-0.256877153	0.075449727	-3.404613409	0.000662578	0.001072497
UPK2	9.616999807	0.254796243	0.074818641	3.40551817	0.000660386	0.001069038
ZSCAN32	254.9384215	-0.11251494	0.033026806	-3.406776241	0.00065735	0.001064211
NEUROG3	7.581377283	-0.599282908	0.175883677	-3.407268478	0.000656165	0.001062381
COX19	1219.11247	0.092044777	0.02700961	3.407852881	0.000654762	0.001060196
HOXA13	37.55609739	-0.61951106	0.181762026	-3.408363519	0.000653538	0.001058302
FCRL1	266.6939318	0.316029819	0.09270244	3.409077679	0.000651829	0.001055622
IGSF6	514.0234683	0.176705509	0.05183189	3.409204427	0.000651526	0.001055219
LYPD4	6.788628001	-0.397328552	0.116505554	-3.410382909	0.000648717	0.001050757
EIF2AK4	2494.207691	-0.07768017	0.022774084	-3.410902045	0.000647483	0.001048845

SKP2	814.5558186	-0.10631282	0.031167606	-3.411003751	0.000647242	0.001048541
GAPDH	52365.59526	0.173833788	0.050961811	3.411059853	0.000647109	0.001048412
EPB41	9369.630577	-0.195192908	0.057213645	-3.411649607	0.000645711	0.001046233
ARHGEF17	1134.668779	-0.212976457	0.062425698	-3.411679249	0.00064564	0.001046206
AL160269.1	3.169149987	-0.33997965	0.099631883	-3.41235797	0.000644035	0.00104369
SMPX	0.64591879	-0.89357485	0.261863742	-3.412365696	0.000644017	0.00104369
GATD3A	78.63952675	-0.245704447	0.071995505	-3.412774843	0.00064305	0.001042268
NUTM2B	73.21549692	-0.239680292	0.070222445	-3.413157885	0.000642147	0.00104089
PARL	141.4468946	-0.135694072	0.039755477	-3.413217033	0.000642008	0.00104075
MFAP3L	92.1615134	-0.310936519	0.091096482	-3.413265921	0.000641893	0.00104065
PACSIN2	2443.806977	0.091919266	0.026929231	3.413363999	0.000641662	0.001040362
CCT8L2	0.543236239	-0.614910114	0.180142193	-3.413470796	0.00064141	0.00104004
RBM14	6767.870606	-0.142841606	0.041839494	-3.414037584	0.000640077	0.001037965
SMS	1177.575744	0.109071115	0.031945751	3.414260481	0.000639554	0.001037202
HNRNPCL3	0.543773331	-0.503681688	0.147516066	-3.414419197	0.000639181	0.001036684
TFF3	39.96213497	-0.37453325	0.109640375	-3.416015777	0.000635446	0.00103071
LTK	1131.494856	-0.275665844	0.080695238	-3.416135186	0.000635167	0.001030344
GBP3	548.715496	0.143701251	0.042053463	3.417108619	0.0006329	0.001026751
SERPINE2	243.5135196	-0.298261732	0.087271921	-3.417613924	0.000631726	0.001024932
TCEAL9	82.42520293	-0.406349465	0.118870484	-3.418421887	0.000629854	0.001021979
ABCD1	878.2871499	-0.162577781	0.047558993	-3.418444581	0.000629801	0.001021978
PRKCA	1153.842533	0.252090743	0.073743765	3.418468571	0.000629746	0.001021973
CARS1	1298.013233	0.124003573	0.036272717	3.418645807	0.000629336	0.001021392
ORM1	122.1034782	-0.37806918	0.110579988	-3.418965642	0.000628597	0.001020277
CTSG	7001.117215	0.369315847	0.108002376	3.419515949	0.000627327	0.0010183
NANOGNB	1.187311794	-0.541464143	0.158344992	-3.419521733	0.000627313	0.0010183
IL31RA	231.1026484	0.390968943	0.114327253	3.419735292	0.000626821	0.001017648
C7orf25	14.15002868	-0.194908958	0.05697904	-3.42071325	0.000624571	0.00101408
RESP18	0.803873593	-0.666869563	0.194944603	-3.420815716	0.000624336	0.001013782
TAFA2	440.2353625	-0.172301985	0.050361884	-3.421277576	0.000623277	0.001012146
DPYS	6.37127394	-0.359944068	0.105176623	-3.422282024	0.000620979	0.001008498
UNC50	734.1919369	0.088326923	0.02580699	3.422596826	0.00062026	0.001007414
TMBIM4	490.9236146	-0.10566792	0.030862578	-3.423820274	0.000617474	0.001002973
SLC1A2	216.7904033	0.255081053	0.074497622	3.424016027	0.00061703	0.001002334
PSMC5	1521.629057	-0.112298932	0.03279526	-3.424242754	0.000616515	0.001001581
ZNF506	1110.636099	0.143646699	0.041944832	3.424657861	0.000615574	0.001000135
DAAM2	38.99618822	-0.389259972	0.113654282	-3.424947691	0.000614918	0.000999152

GPR88	38.23877417	-0.3716591	0.108514478	-3.424972455	0.000614862	0.000999144
KCNH6	9.967396465	-0.252900172	0.073810338	-3.426351644	0.000611748	0.000994166
SLC22A10	6.4277139	-0.570010362	0.166355198	-3.426465586	0.000611491	0.000993832
BUB1B-PAK6	1.556284878	-0.314977195	0.091913237	-3.426897003	0.000610521	0.000992337
FAM83F	180.6137092	-0.300197074	0.087589224	-3.427328835	0.00060955	0.000990842
PAK1IP1	237.3284697	-0.159754007	0.046609207	-3.427520386	0.000609121	0.000990226
PLG	1.114260096	-0.48375293	0.141049209	-3.429674879	0.000604305	0.000982479
VEZT	1822.278856	0.113472066	0.033084729	3.429741382	0.000604157	0.000982319
IRF1	9274.108355	0.16463187	0.047983487	3.431010969	0.000601336	0.000977815
CCM2	1154.000677	-0.126967085	0.037004835	-3.431094446	0.000601151	0.000977595
ULK2	484.5999558	-0.20968809	0.061113434	-3.431129217	0.000601074	0.000977551
CAVIN1	528.0769079	-0.392765189	0.114459515	-3.43147695	0.000600304	0.00097638
CYB561D1	510.0509441	-0.138854302	0.040461146	-3.431793635	0.000599604	0.000975321
MAP1A	1052.139068	-0.291738574	0.085009718	-3.43182614	0.000599532	0.000975286
RPL39L	54.38355614	-0.324946547	0.094662277	-3.432693137	0.000597618	0.000972253
LYZL6	0.549546469	-0.512360847	0.149233593	-3.433280911	0.000596324	0.000970228
RCSD1	3530.470021	-0.163371217	0.04758403	-3.43332031	0.000596237	0.000970168
SMURF2	1427.438571	0.119269815	0.034729531	3.434247784	0.000594201	0.000966935
COL7A1	335.8336447	0.277029304	0.080652468	3.434852172	0.000592877	0.000964861
BANF2	0.677070741	-0.560186155	0.163075571	-3.435132258	0.000592265	0.000963945
TSC22D1	3585.065409	0.209188794	0.060896469	3.435154735	0.000592216	0.000963945
CRISP3	511.4968938	-0.531051415	0.154593122	-3.435155511	0.000592214	0.000963945
NAAA	866.4038094	0.202614684	0.058961835	3.436370059	0.000589565	0.00095979
PRPF39	1178.2044	0.114200299	0.033226553	3.437019115	0.000588154	0.000957573
CELA3B	1.067892604	-0.454313481	0.132169848	-3.437345869	0.000587445	0.000956498
SHROOM2	7.07818572	0.395347176	0.115012818	3.437418379	0.000587288	0.000956321
DCD	1.327571426	-0.632769992	0.184077278	-3.437523624	0.00058706	0.000956029
GLYR1	3113.44406	-0.068810872	0.020013645	-3.438197928	0.000585599	0.000953731
SLC15A5	1.059548951	-0.465493764	0.13536997	-3.438678185	0.000584562	0.00095212
TGFA	97.02496328	-0.297049957	0.08638119	-3.438826873	0.000584241	0.000951676
CASS4	443.5097809	0.21556609	0.062682036	3.439040954	0.000583779	0.000951003
H2BC10	757.2175175	-0.330815444	0.096193579	-3.439059506	0.000583739	0.000951003
COL13A1	5.437583684	-0.362141209	0.105287531	-3.439545092	0.000582693	0.000949392
PEG10	30.70122457	0.305784287	0.088891562	3.439969767	0.000581779	0.000947982
IL6R	2164.583893	-0.20826157	0.060538313	-3.440161435	0.000581367	0.00094739
GNAT2	75.06368422	-0.262264197	0.07621619	-3.441056252	0.000579448	0.000944341
LRRC15	3.375840577	-0.424846522	0.123461003	-3.441139407	0.00057927	0.00094413

DRD4	69.39188074	-0.167573096	0.048695626	-3.441235078	0.000579065	0.000943874
ITGB7	938.3782921	0.235921964	0.068550801	3.441563939	0.000578362	0.000942806
CBL	5589.92965	0.127345937	0.036995238	3.442225104	0.00057695	0.000940583
TDRD7	980.6017997	-0.169681199	0.049280364	-3.443180687	0.000574915	0.000937344
TMOD3	2640.798298	0.093055351	0.027024292	3.44339645	0.000574457	0.000936674
UBASH3A	124.5968328	0.262476437	0.07621737	3.44378766	0.000573626	0.000935398
RSPH14	6.515746004	-0.227172784	0.065948849	-3.444681539	0.000571733	0.000932388
PDIA4	2602.396349	0.157257893	0.045645562	3.445195723	0.000570646	0.000930694
KXD1	736.1029158	-0.109551488	0.031793409	-3.445729546	0.00056952	0.000928935
SLC25A2	11.81208904	-0.25272829	0.073343269	-3.445827997	0.000569313	0.000928674
PI4KA	7505.227507	-0.09585169	0.027806859	-3.447052075	0.000566739	0.000924553
OTUD3	640.451446	0.120614077	0.034986541	3.44744218	0.000565922	0.000923296
MTRNR2L11	4.554713629	-0.793271515	0.230071105	-3.447940641	0.000564878	0.000921671
STPG4	72.92164428	0.21498325	0.062349213	3.448050755	0.000564648	0.000921372
ELOA3B	0.550609899	-0.621261095	0.180163884	-3.448310955	0.000564104	0.000920561
GLRX5	1223.648902	-0.219193869	0.063561642	-3.448524347	0.000563659	0.000919911
GUSB	4282.541553	0.129649002	0.037590381	3.448994063	0.000562679	0.000918389
LHFPL5	442.9061119	0.200602981	0.058152207	3.449619374	0.000561377	0.000916341
ENDOU	25.44321389	-0.225264843	0.065296697	-3.449865812	0.000560865	0.000915581
UNC5CL	92.07354984	-0.165523143	0.047940704	-3.452663996	0.00055508	0.000906212
ZNF417	676.3148003	-0.116204112	0.033645469	-3.453781893	0.000552784	0.00090254
CDC26	160.5655574	-0.122481315	0.035460926	-3.45397955	0.000552379	0.000901954
FNTA	1495.541217	0.092440641	0.026761876	3.454191359	0.000551946	0.000901321
ETAA1	310.3828956	0.134024386	0.038798025	3.454412582	0.000551493	0.000900657
PHIP	8370.056368	0.134400657	0.038905351	3.45455452	0.000551203	0.000900258
RAB3C	26.85914246	-0.337748499	0.097761971	-3.454804527	0.000550692	0.000899499
CEP55	152.7083626	-0.211908184	0.061332311	-3.455082323	0.000550125	0.000898647
CD300A	690.332743	-0.216114625	0.062542391	-3.455490312	0.000549293	0.000897363
FSD1L	196.2532755	-0.16565814	0.04793757	-3.455705842	0.000548854	0.000896721
SH3RF1	788.0451491	-0.189958004	0.054945248	-3.457223516	0.000545772	0.00089176
ZNF282	1021.583963	-0.103401798	0.029897349	-3.458560749	0.00054307	0.000887419
OOSP4B	1.50006315	-0.486010651	0.140508324	-3.458945603	0.000542294	0.000886226
SDC3	82.88409469	0.270902257	0.078307856	3.459451867	0.000541276	0.000884636
C11orf95	593.4901314	-0.167618216	0.0484445	-3.460005085	0.000540165	0.000882894
TEKT2	72.65634806	-0.202247727	0.058407227	-3.462717519	0.000534749	0.000874115
ABHD1	67.45044333	-0.198665135	0.057363184	-3.463286415	0.00053362	0.000872342
FCGR1B	89.73132784	0.276274855	0.079757736	3.463925517	0.000532354	0.000870344

CASR	8.896031171	-0.298303462	0.086112273	-3.464122484	0.000531964	0.00086978
ABHD14A-ACY1	1.310603979	-0.320298274	0.092457309	-3.464282893	0.000531647	0.000869334
ZSWIM1	426.8456887	-0.103390189	0.029843805	-3.464376938	0.000531461	0.000869103
COX6A1	588.6011759	-0.206122323	0.059484227	-3.4651593	0.000529917	0.000866651
TMEM14B	855.3983319	-0.169672975	0.048958917	-3.465619422	0.000529012	0.000865241
APLN	37.19008857	0.41007784	0.118322688	3.465758319	0.000528738	0.000864867
REEP1	13.83523306	-0.238726859	0.068870453	-3.46631754	0.00052764	0.000863142
THAP7	340.9524165	-0.121530369	0.03505768	-3.466583324	0.000527118	0.000862361
SH3D19	982.7911521	0.183024042	0.052788179	3.467140659	0.000526027	0.000860647
SLCO1B3	0.477363397	-1.138853408	0.328389114	-3.467999876	0.000524348	0.000857971
ZC3H12C	675.330534	-0.309862293	0.089322422	-3.469031481	0.000522338	0.000854755
IFNL3	1.29375715	-0.435904478	0.125655537	-3.469043122	0.000522316	0.000854755
ESS2	856.3949989	0.101946169	0.029385431	3.469275965	0.000521863	0.00085412
OR10K2	0.859897678	-0.842874035	0.242946815	-3.469376769	0.000521667	0.000853871
GPX4	4016.982707	-0.236648421	0.068208792	-3.469470924	0.000521485	0.000853644
COL5A3	20.24046806	-0.271298369	0.078194989	-3.469510953	0.000521407	0.000853588
RBFOX3	132.7281623	0.222557914	0.064131826	3.470319328	0.00051984	0.000851094
ESYT2	9993.749194	-0.107307925	0.030919417	-3.47056756	0.00051936	0.000850379
PDE6G	230.3480123	-0.238162327	0.068616407	-3.470923927	0.000518671	0.000849322
OR2T3	0.471134506	0.623575239	0.179532344	3.4733309	0.000514041	0.000841811
INTS6L	1381.762503	-0.115513251	0.033256866	-3.473365507	0.000513975	0.000841773
ANGPTL6	157.1665859	-0.189893615	0.054665003	-3.473769425	0.000513202	0.000840577
LDHAL6A	21.3838665	-0.211967086	0.061009746	-3.474315149	0.000512159	0.00083894
ARHGAP17	4049.705542	0.10963668	0.031544016	3.475672862	0.000509573	0.000834774
CCK	2.23786242	-0.463115227	0.133195753	-3.476951903	0.000507149	0.000830872
AC010323.1	1.199111951	0.318351221	0.091539947	3.477729996	0.000505679	0.000828533
BSG	10872.47942	-0.18712096	0.053783759	-3.479135029	0.000503035	0.00082427
POLR2E	2213.472221	-0.130303232	0.037451505	-3.479252255	0.000502815	0.000823978
PDHB	809.70537	-0.079283276	0.022785924	-3.47948474	0.000502379	0.000823333
PDE4DIP	594.2032981	-0.216063738	0.062090218	-3.479835404	0.000501722	0.000822325
SPDYE16	18.46241504	-0.179667337	0.051617669	-3.480733244	0.000500043	0.000819642
CYB561A3	1015.902394	-0.103142122	0.029616068	-3.48264059	0.000496494	0.000813893
C1R	150.4663145	-0.175339097	0.050336661	-3.483327946	0.000495221	0.000811874
ADH5	1455.694244	0.085748509	0.024602494	3.485358329	0.000491478	0.000805806
ZNF532	523.200738	-0.373384447	0.107125417	-3.485488834	0.000491239	0.00080548
INSL3	53.88946002	0.224472188	0.064355828	3.487985395	0.000486675	0.000798063
HNRNPUL1	9670.908612	0.109922407	0.031511402	3.488337604	0.000486034	0.000797079

YES1	906.5967738	0.252803979	0.072452898	3.489218328	0.000484435	0.000794524
CAVIN3	13.16159344	0.531491515	0.152300248	3.489761328	0.000483452	0.000792978
NBPF3	233.9719915	-0.235927679	0.067604717	-3.48981093	0.000483362	0.000792897
TRIM36	42.99631873	0.292637674	0.083841744	3.490357654	0.000482375	0.000791343
MPG	608.8737668	-0.182031822	0.052152604	-3.490368796	0.000482354	0.000791343
WIPI1	491.127421	0.186689687	0.05348684	3.490385452	0.000482324	0.000791343
TRIM33	3816.976816	0.105578351	0.030248043	3.490419207	0.000482263	0.000791343
TMED1	154.7410077	-0.111658191	0.031982489	-3.491228933	0.000480804	0.000789031
KRT85	2.509952653	-0.572884127	0.164054546	-3.492034451	0.000479356	0.000786722
ROR2	37.72048882	-0.403992712	0.115688087	-3.49208569	0.000479264	0.000786637
EPPIN	0.752998979	-0.529397267	0.151597177	-3.492131438	0.000479182	0.000786568
PLK2	796.6875373	-0.271007179	0.077596951	-3.492497769	0.000478526	0.000785556
CSNK1D	6851.384162	0.145546703	0.041670694	3.49278331	0.000478014	0.000784782
BGN	25.11932885	-0.422828166	0.121045135	-3.493144661	0.000477368	0.000783787
ZBTB8OS	403.9989432	-0.122118854	0.0349583	-3.49327214	0.00047714	0.000783478
BCAM	188.8102208	-0.298311629	0.085391121	-3.493473608	0.00047678	0.000782953
SLX1A	1.378628944	-0.329862148	0.094394675	-3.49449954	0.000474951	0.000780015
TBC1D20	1754.360265	0.102320585	0.029272133	3.495494716	0.000473184	0.000777178
HTRA3	317.2977091	-0.346452695	0.099098983	-3.496026725	0.000472241	0.000775695
KRT27	0.593678289	-0.834800859	0.238775749	-3.496171047	0.000471986	0.000775341
TRIM11	1948.46543	0.141040886	0.040340656	3.496246682	0.000471852	0.000775186
ZNF888	554.7837879	0.146501107	0.041884553	3.497735932	0.000469225	0.000770935
NXF3	252.3190692	-0.381848046	0.109153261	-3.498274279	0.000468279	0.000769445
HBM	1822.161726	-0.403723292	0.115397408	-3.498547314	0.0004678	0.000768722
ZMAT4	1.812517035	-0.593761908	0.169659892	-3.499718765	0.000465749	0.000765416
RAB42	34.06566274	-0.19495399	0.05570339	-3.499858617	0.000465505	0.000765079
TBC1D3L	50.41471421	-0.25179985	0.071938508	-3.500209488	0.000464893	0.000764137
ETDA	0.962928218	-0.363568364	0.103868746	-3.500267184	0.000464792	0.000764036
ABLIM3	31.18315196	0.346123023	0.09887948	3.500453526	0.000464467	0.000763566
GGT5	451.0766636	-0.312527502	0.089279998	-3.500532116	0.00046433	0.000763405
GNB2	5121.732024	0.141191497	0.040329658	3.50093469	0.000463629	0.000762317
DYNC1H1	23784.5441	0.131196311	0.037474325	3.500965296	0.000463576	0.000762293
DOCK5	6174.444014	-0.158820722	0.045357793	-3.501509044	0.000462631	0.000760803
SMIM10	52.7939023	-0.314167618	0.089705324	-3.50221819	0.000461402	0.000758845
CCDC17	444.0795263	-0.191792861	0.054758551	-3.50251894	0.000460881	0.000758052
IZUMO1R	0.366463396	-0.753677709	0.215081594	-3.504147867	0.000458071	0.000753494
PRR29	145.4971819	-0.165867397	0.047334356	-3.504165058	0.000458041	0.000753494

TMEM235	2.035131741	-0.376893954	0.107541674	-3.504631651	0.000457239	0.000752252
SLC32A1	3.056576359	-0.509321512	0.145299562	-3.505320351	0.000456058	0.000750372
RERGL	0.801817784	-0.982756501	0.280348127	-3.505486245	0.000455774	0.000749968
HTR1A	0.406455295	-0.948410566	0.27053587	-3.505674006	0.000455453	0.000749502
SORBS1	362.4913616	-0.267680518	0.076355275	-3.505723974	0.000455367	0.000749424
SMIM12	784.4632307	0.089301597	0.025471181	3.505985704	0.00045492	0.000748751
TEX50	0.578127828	-0.661924188	0.188772175	-3.506471166	0.000454091	0.000747449
SOX2	0.730751277	-0.545955727	0.155662307	-3.507308466	0.000452664	0.000745164
NSUN2	1962.659845	0.094695758	0.026997439	3.507582966	0.000452197	0.000744458
NSUN4	1565.001692	-0.087580168	0.024967767	-3.507729304	0.000451949	0.000744111
DEFB132	0.689044942	-0.461971832	0.131695902	-3.507867925	0.000451713	0.000743786
ITGB3	566.9922675	-0.421715917	0.120216512	-3.507970008	0.00045154	0.000743563
NECAP2	1673.110852	0.095698984	0.027280274	3.507991981	0.000451503	0.000743563
GPR21	70.92124275	-0.261011902	0.074404695	-3.508003115	0.000451484	0.000743563
FTSJ1	874.0031222	-0.095350122	0.027175999	-3.508615122	0.000450446	0.000741949
BCAS4	321.1088528	-0.197836356	0.056376729	-3.509184736	0.000449483	0.000740424
AC093155.3	4.765273388	-0.266149358	0.075839963	-3.509355066	0.000449195	0.000740013
SPINK8	8.383712281	-0.299186912	0.08524938	-3.509549408	0.000448867	0.000739534
CYP7A1	2.560766041	-0.346036186	0.098562732	-3.51082178	0.000446724	0.000736066
EFNB3	7.688264632	-0.435940827	0.124154477	-3.511277544	0.000445959	0.000734867
OTOA	37.42794564	-0.272746264	0.077673618	-3.51144017	0.000445686	0.000734479
HPGD	222.4561946	-0.382978413	0.109065436	-3.511455391	0.00044566	0.000734479
TENT5C	8990.280039	-0.305293203	0.08692831	-3.512011252	0.000444729	0.000733026
PTP4A1	212.6082097	-0.246279499	0.070122845	-3.512115062	0.000444555	0.000732801
CPNE2	1305.198913	-0.168999863	0.048109682	-3.51280357	0.000443405	0.000730967
EPAS1	292.590532	0.284514637	0.080985687	3.513147183	0.000442832	0.000730084
AMDHD2	475.5424756	-0.17443495	0.049646957	-3.513507352	0.000442232	0.000729156
SCGB1D4	0.725294154	-0.748028877	0.212890039	-3.513686601	0.000441934	0.000728726
MOB2	532.5672926	-0.101790134	0.028966986	-3.514005005	0.000441404	0.000727914
GPR39	0.42662774	-0.692221463	0.196982555	-3.514125722	0.000441204	0.000727644
LTA4H	3144.817382	0.146385492	0.041653322	3.514377336	0.000440786	0.000727017
PTGR1	89.69662441	-0.268042103	0.076263762	-3.514671935	0.000440298	0.000726272
MRPS35	869.7124477	0.120602949	0.034313995	3.514686896	0.000440273	0.000726272
TTC12	96.99941494	-0.251531847	0.071561797	-3.514890014	0.000439936	0.000725798
CROCC2	195.5213528	0.462386682	0.131549791	3.51491766	0.00043989	0.000725784
TUBB4B	5434.980262	-0.170965439	0.048612742	-3.516885354	0.000436642	0.000720486
PDCD1	521.7835069	0.337025719	0.09581894	3.517318392	0.000435931	0.000719372

LBHD2	0.65475577	-0.496704787	0.141167564	-3.518547549	0.000433916	0.000716108
OR10G4	0.449685962	-0.699270642	0.198718143	-3.518906887	0.000433329	0.000715199
TNFSF12	63.49494955	0.285732916	0.081188485	3.519377347	0.000432561	0.000713992
ATE1	926.8842807	-0.08535029	0.024250146	-3.519578454	0.000432233	0.000713511
ZNF346	837.1743785	-0.098132861	0.027873392	-3.520664463	0.000430467	0.000710655
TMPRSS2	4.804415901	-0.396767485	0.112690853	-3.520849082	0.000430167	0.000710221
OR10D3	0.619141376	-0.804353758	0.228449802	-3.520921228	0.00043005	0.000710087
RRP15	372.0868839	-0.125668944	0.035690327	-3.521092522	0.000429773	0.000709689
NCEH1	474.2824402	-0.228103824	0.064762491	-3.522159523	0.000428046	0.000706898
HMGCS2	0.650707006	-0.554460539	0.157407971	-3.522442589	0.00042759	0.000706203
TANC1	611.9902508	-0.355516853	0.100912986	-3.523003992	0.000426685	0.000704768
NDUFA1	1203.935485	-0.283318578	0.080413244	-3.523282516	0.000426237	0.000704087
ZNF561	841.9246213	0.099725576	0.028304051	3.523367601	0.0004261	0.000703921
FAM13B	3237.433646	0.111161964	0.031547099	3.523682637	0.000425594	0.000703144
CEMIP	307.7937646	0.253111136	0.07181015	3.524726471	0.00042392	0.000700438
APBA2	568.3509769	-0.308126902	0.087411814	-3.525002945	0.000423478	0.000699767
MCTP1	2331.215178	0.17952322	0.05090979	3.526300542	0.000421408	0.000696406
PLA2G10	17.59632241	-0.195438941	0.055405801	-3.527409379	0.000419647	0.000693554
SREBF2	6313.865989	0.124926365	0.035410965	3.527900656	0.000418869	0.000692326
LSM3	875.4102782	-0.221703621	0.062830601	-3.528593047	0.000417775	0.000690576
GREM1	47.29174013	-0.414423562	0.117396137	-3.530129466	0.000415356	0.000686636
MARCHF7	3574.423965	0.11457986	0.032446732	3.531322044	0.000413488	0.000683605
LEFTY1	2.708290831	-0.319971737	0.090604313	-3.531528766	0.000413165	0.000683129
PSMB8	1886.481173	-0.146626231	0.041511714	-3.532165166	0.000412172	0.000681544
SPATA1	121.0397208	-0.15224266	0.043098393	-3.532443993	0.000411737	0.000680883
EMP2	42.57722144	-0.341836495	0.096766289	-3.532598995	0.000411496	0.000680542
P4HA2	206.0485494	0.196322644	0.055527481	3.535594254	0.000406859	0.00067293
SLC30A1	1702.07396	0.196732058	0.055635901	3.536063149	0.000406138	0.000671793
PPP1R1C	7.480093244	-0.389778957	0.110171317	-3.537934977	0.000403269	0.000667105
SF3A1	5653.785026	0.106489968	0.030088989	3.539167338	0.000401391	0.000664055
MSMB	5.509333862	-0.344501372	0.097339164	-3.539185632	0.000401364	0.000664055
GLT1D1	314.9766012	0.375624093	0.106132023	3.539215429	0.000401318	0.000664046
DSG1	0.590131912	-0.659900371	0.186449646	-3.539295384	0.000401197	0.000663901
SLC30A9	1022.997574	-0.100781668	0.028472964	-3.539556641	0.0004008	0.0006633
CRH	0.978475669	-0.532636919	0.150448913	-3.540317509	0.000399646	0.000661447
CFAP97	679.6701147	-0.172043877	0.048595161	-3.540349991	0.000399597	0.000661421
NHS	31.09780812	-0.316429875	0.089373594	-3.540529826	0.000399324	0.000661027

CYP8B1	7.753656673	-0.328797998	0.092864576	-3.540618107	0.000399191	0.000660861
FAM90A26	0.626635096	-0.486385251	0.137337819	-3.541524503	0.000397822	0.000658651
SLC39A6	892.6169359	0.100267239	0.028302876	3.542652016	0.000396125	0.000655897
POM121L2	0.679390201	-0.464910777	0.131216871	-3.543071657	0.000395495	0.000654909
SPPL2C	0.694659239	-0.498070294	0.140492035	-3.545185284	0.000392337	0.000649735
FBXL12	993.2943163	0.116093768	0.032743612	3.545539413	0.00039181	0.000648917
GPR6	0.503045731	-0.504557824	0.142286136	-3.546078599	0.00039101	0.000647646
DDIAS	126.3758349	-0.153208681	0.043202365	-3.546303093	0.000390677	0.000647149
IER2	3902.291364	-0.168933557	0.047632533	-3.546600352	0.000390236	0.000646474
ANKRD55	31.6389216	-0.308851023	0.087062841	-3.547449416	0.00038898	0.000644448
ASPDH	59.58928673	-0.215422054	0.060722731	-3.547634488	0.000388707	0.00064405
ACSM3	2298.349916	0.281501192	0.079345348	3.547797065	0.000388467	0.000643707
FRMPD1	13.49813895	-0.287716863	0.081072028	-3.548904226	0.000386838	0.000641061
NPIPB3	222.2213366	-0.169568958	0.047761048	-3.550360893	0.000384703	0.000637578
STRIT1	0.571152338	-0.701861421	0.197577496	-3.552334839	0.000381829	0.000632867
DUOX2	31.22745974	-0.327914805	0.092307063	-3.552434602	0.000381684	0.000632681
LRATD1	21.38553005	-0.604418299	0.170115959	-3.55297822	0.000380896	0.000631429
SEC22B	1212.777983	0.099763458	0.028078338	3.553039952	0.000380807	0.000631334
SLC25A47	20.71308754	-0.350656743	0.098666714	-3.553951755	0.000379489	0.000629202
ID2	2596.594438	0.269709628	0.075884455	3.554214455	0.00037911	0.000628627
VAMP5	321.3707491	-0.242711076	0.068287094	-3.554274497	0.000379023	0.000628537
ZFR2	13.53254572	-0.268628174	0.075577447	-3.554343074	0.000378925	0.000628427
ZNF879	89.73705079	-0.184781444	0.051968672	-3.555631469	0.000377072	0.000625408
TAS2R41	22.36267947	-0.331020273	0.093092182	-3.555833208	0.000376783	0.000624981
LIPK	1.633183227	0.551287908	0.154981786	3.557114177	0.000374951	0.000621995
IQCF1	0.602350883	-0.638657833	0.179512015	-3.557744222	0.000374053	0.000620558
TCOF1	5620.116784	0.180515056	0.05073748	3.557824653	0.000373939	0.000620421
SP5	1.077000345	-0.510499713	0.143484816	-3.557865759	0.00037388	0.000620376
AKAP11	1977.689563	-0.135624784	0.038118568	-3.557971592	0.00037373	0.000620179
LGALS3BP	2293.291428	-0.280861198	0.078934998	-3.558132689	0.000373501	0.000619851
EIF3M	4059.076263	0.100117627	0.028134843	3.558492483	0.000372989	0.000619056
YBX2	34.1717201	-0.374363803	0.105199026	-3.558624229	0.000372802	0.000618798
RPS21	16694.4546	-0.265038967	0.074472186	-3.558898692	0.000372413	0.000618204
ZNF493	920.4464519	-0.182847129	0.051376109	-3.558991413	0.000372282	0.000618038
ITPRID1	0.822067101	-0.602445476	0.169273674	-3.559002776	0.000372266	0.000618038
ARPC1A	216.3775365	0.133163862	0.037414124	3.559186987	0.000372005	0.000617683
GXYLT2	96.13250961	0.385289768	0.108251759	3.559201002	0.000371985	0.000617683

ATP5MC3	2715.513593	-0.177634233	0.049906847	-3.559315911	0.000371822	0.000617485
LMCD1	29.95508403	-0.341024759	0.095780751	-3.5604728	0.000370188	0.000614822
CAGE1	27.16347921	-0.223050484	0.062646003	-3.560490247	0.000370163	0.000614822
HAAO	293.551122	-0.211083544	0.059279906	-3.560794205	0.000369735	0.000614175
KIR3DL3	0.82411965	0.479952059	0.134755984	3.561638203	0.000368548	0.000612255
FNDC3B	7199.451026	0.171892533	0.048258923	3.56188085	0.000368207	0.000611741
HERC6	460.9326881	-0.170563443	0.047872961	-3.562834646	0.000366872	0.000609574
ODF3B	832.0511304	0.242858167	0.068146306	3.56377596	0.000365558	0.000607443
MRFAP1L1	330.4711763	-0.148727946	0.04173124	-3.56394743	0.000365319	0.000607097
GRPR	5.646363274	-0.232447746	0.065194574	-3.565446191	0.000363238	0.000603689
SPP2	0.744407838	-0.453160782	0.127048037	-3.566845998	0.000361304	0.000600526
TENT5D	0.522101275	-1.073927381	0.301065811	-3.567085145	0.000360974	0.00060003
PIWIL2	48.12739866	-0.21577446	0.060463209	-3.568690192	0.00035877	0.000596417
CHST1	6.28810035	-0.356305687	0.099841085	-3.568728109	0.000358718	0.000596381
SYCP2	146.3535774	-0.305395425	0.085563142	-3.569240408	0.000358018	0.000595267
GET3	1033.577438	-0.148604116	0.041633083	-3.569375743	0.000357833	0.00059501
THSD7A	327.9807406	0.539780575	0.151225382	3.569378157	0.00035783	0.00059501
FSBP	60.34301397	-0.163111642	0.045697056	-3.569412467	0.000357783	0.00059501
CLPSL1	0.788352516	-0.601140366	0.16840928	-3.569520426	0.000357635	0.000594833
ATP6V0E2	155.4083829	-0.221576262	0.062059153	-3.570404203	0.000356431	0.00059288
OMP	2.711921512	-0.366830301	0.10267858	-3.572607861	0.000353444	0.000587962
DNAJA4	1447.992892	-0.257370496	0.072026497	-3.573275196	0.000352544	0.000586514
FAM81A	38.01192396	-0.373250788	0.104452177	-3.573413193	0.000352358	0.000586255
REEP2	27.6563619	-0.295696622	0.082741379	-3.573745383	0.000351911	0.000585561
UBR1	2384.356029	-0.139757615	0.039105699	-3.573842612	0.00035178	0.000585393
NEK7	992.5901064	-0.141452185	0.039567168	-3.574988825	0.000350243	0.000582884
AL139011.2	3.57304575	-0.299476766	0.083761894	-3.575334236	0.000349781	0.000582165
WFDC8	0.529378436	-0.521052108	0.145706334	-3.576042957	0.000348834	0.000580639
LETMD1	1633.495826	-0.090683468	0.025353349	-3.576784515	0.000347847	0.000579044
INPP5D	12056.993	-0.10177976	0.028450038	-3.57749113	0.000346908	0.000577531
H4C7	1.946871564	-0.541424166	0.151299323	-3.578496943	0.000345576	0.000575362
FOXG1	0.635497326	-0.895763852	0.250285474	-3.578968601	0.000344953	0.000574374
LY6G6E	8.000039082	-0.426484572	0.119093599	-3.581087265	0.000342167	0.000569784
NKX2-5	1.982559002	-0.479352505	0.133786426	-3.582968152	0.000339712	0.000565744
USP6	66.00615652	-0.359891233	0.100385504	-3.58509165	0.00033696	0.000561208
BRWD3	2340.350165	0.117019258	0.032639282	3.585227727	0.000336784	0.000560963
COX6C	1221.780631	-0.193108228	0.0538595	-3.585406992	0.000336553	0.000560626

LY6G6D	3.288802556	-0.463457308	0.129237016	-3.586103443	0.000335656	0.000559179
FAM131C	2.408052154	-0.372773856	0.103929617	-3.586791393	0.000334772	0.000557754
IQCC	161.8704329	-0.126350584	0.035209453	-3.588541553	0.000332533	0.000554071
ZNF680	370.4284901	-0.15223072	0.042408053	-3.589665419	0.000331103	0.000551734
GNG3	30.51541254	-0.228092983	0.063531279	-3.590246998	0.000330365	0.000550552
AC138894.1	2.813289119	-0.274144076	0.076355254	-3.590376069	0.000330201	0.000550326
HOXB5	271.7999148	-0.66136776	0.184149921	-3.591463718	0.000328826	0.000548081
DAP3	2160.947494	-0.104796894	0.029178649	-3.591560823	0.000328703	0.000547923
HSPA6	329.8869434	-0.390105071	0.108561676	-3.593395794	0.000326396	0.000544123
AFAP1	61.78755657	0.319305068	0.088772018	3.596911239	0.000322018	0.000536871
CAVIN4	13.04970631	-0.292171721	0.08122611	-3.597017256	0.000321887	0.000536698
IBSP	2.780095559	-0.640502654	0.178027842	-3.597766776	0.000320961	0.0005352
FGF23	8.868676008	-0.347563218	0.096561436	-3.599399855	0.000318952	0.000531895
CD79B	185.4544667	-0.26372796	0.073232909	-3.601221966	0.000316725	0.000528226
CCDC13	6.134418928	-0.217530501	0.060396523	-3.60170571	0.000316136	0.000527289
ANLN	971.7504346	-0.186731114	0.051843433	-3.6018277	0.000315988	0.000527086
NKX6-2	12.70447612	-0.332177924	0.092224651	-3.601834427	0.00031598	0.000527086
KLRF1	67.37964349	0.257036665	0.07134656	3.602649721	0.00031499	0.000525511
USP49	1091.877857	-0.142932627	0.039672963	-3.60277167	0.000314842	0.000525309
MYL2	2.11525737	0.470089148	0.13047309	3.602958644	0.000314616	0.000524976
CRPPA	214.1731421	-0.223188169	0.061941687	-3.603198081	0.000314326	0.000524537
MAT2B	1096.996147	-0.135167943	0.037504608	-3.60403559	0.000313314	0.000522894
MS4A13	0.818040811	-1.021817781	0.283477652	-3.604579669	0.000312659	0.000521844
RDH13	634.3621618	-0.211218406	0.058594932	-3.60472139	0.000312488	0.000521604
KRTCAP2	157.460637	-0.17494759	0.048523653	-3.605408515	0.000311662	0.00052027
SULT1A1	440.2443743	-0.184760458	0.051238302	-3.605905192	0.000311067	0.00051932
GPR160	761.9557575	0.22473343	0.062303342	3.607084656	0.000309657	0.00051701
FABP6	4.108409545	-0.477574947	0.132389842	-3.607338289	0.000309354	0.000516549
MRPS28	81.8071957	-0.106658537	0.029560393	-3.608156906	0.00030838	0.000514966
RTP3	4.601992008	-0.548706603	0.152001062	-3.609886653	0.000306331	0.000511588
UBE2E2	610.7869626	0.196242782	0.054361748	3.609942435	0.000306265	0.000511522
TAF2	1654.779636	0.100494506	0.027823788	3.611819763	0.000304056	0.000507876
SLC39A8	1975.406338	0.149803237	0.041474138	3.61196745	0.000303883	0.00050763
IWS1	968.5019954	-0.094083967	0.026045201	-3.612334095	0.000303453	0.000506955
HOXB3	1067.325498	-0.495655596	0.137209865	-3.612390369	0.000303387	0.000506889
WNT8B	31.47658345	-0.24470041	0.067727182	-3.613031048	0.000302638	0.000505681
H3C11	700.7010632	-0.303608553	0.084021552	-3.613460417	0.000302137	0.000504887

IRX4	0.980431892	-0.464293526	0.128461249	-3.614269119	0.000301196	0.000503356
RNF223	10.19067393	0.325119266	0.089950306	3.614432007	0.000301007	0.000503083
CCDC88A	5370.505029	0.130575877	0.036118856	3.615172016	0.000300148	0.000501691
PELO	275.5663972	-0.166165115	0.045958755	-3.615526913	0.000299737	0.000501047
RBMXL2	0.683968759	-0.570685742	0.157817257	-3.616117478	0.000299055	0.000499949
MYMK	0.637990094	-0.51897749	0.143477289	-3.617140351	0.000297876	0.00049802
ZFYVE26	2563.650859	-0.130299318	0.03599759	-3.61966786	0.000294981	0.000493223
AC020765.6	3.390600544	-0.288001689	0.079517238	-3.621877408	0.000292473	0.00048907
HEATR1	2195.616626	-0.117702663	0.032493205	-3.622377785	0.000291907	0.000488167
VMP1	10098.31581	0.206159905	0.056902212	3.623056054	0.000291143	0.00048693
SMN2	147.3221623	0.152001603	0.041952068	3.623220733	0.000290957	0.000486661
ZNF484	462.3281965	0.134171307	0.037022892	3.624009395	0.000290071	0.00048522
OTOP2	2.014290998	-0.402808778	0.11113393	-3.624534617	0.000289482	0.000484277
KCNK4	2.383471539	-0.402792571	0.111091241	-3.625781529	0.000288089	0.000481987
LYRM1	673.5258217	0.123500577	0.034057414	3.626246464	0.000287571	0.000481161
LPAR3	53.24826909	-0.595221349	0.164122824	-3.626682341	0.000287086	0.000480391
GINS4	309.9116892	-0.143949788	0.039688895	-3.626953768	0.000286785	0.000479928
RNF113B	0.560068734	-0.684548081	0.188666375	-3.628352337	0.000285236	0.000477377
ILDR2	307.4249137	-0.316017856	0.087032212	-3.631044761	0.000282276	0.000472464
TMPRSS7	1.501080241	-0.419097851	0.115416571	-3.631175735	0.000282133	0.000472265
PIWIL4	1382.520732	-0.217192069	0.059808754	-3.631442795	0.000281841	0.000471816
ZBTB47	291.5821018	-0.198963821	0.054784258	-3.631769929	0.000281484	0.000471259
MGAT2	7.613811973	-0.191307629	0.052671185	-3.632111767	0.000281111	0.000470675
GAS2L1	267.7373704	0.286650385	0.07891969	3.632178309	0.000281039	0.000470594
TIGIT	65.16404496	0.239735961	0.066002097	3.632247612	0.000280963	0.000470508
PRR15	20.40456492	-0.454533463	0.125038414	-3.635150582	0.000277818	0.000465281
UCHL3	75.2442447	-0.177882441	0.048932473	-3.635263689	0.000277696	0.000465117
MYO1H	36.47103989	0.207915813	0.057193841	3.635283293	0.000277675	0.000465117
NAT8L	64.91490588	-0.320847039	0.088249868	-3.635665948	0.000277263	0.000464471
RAPGEFL1	377.1016247	0.164070198	0.045112383	3.63692156	0.000275916	0.000462253
OR11H6	0.908110752	-0.547400479	0.150492771	-3.63738719	0.000275418	0.000461458
EBLN2	475.195552	0.150132677	0.041270551	3.63776769	0.000275011	0.000460816
KAT2B	1690.37784	-0.195777738	0.053811412	-3.638219674	0.000274529	0.000460048
ZNF107	881.4161202	0.143444302	0.039425185	3.638392617	0.000274345	0.000459779
FKBP8	6712.523832	-0.186550065	0.051258521	-3.639396199	0.000273278	0.00045803
BANK1	586.1375099	-0.24404844	0.067040312	-3.64032372	0.000272295	0.000456422
SLC30A5	596.2902737	-0.087870986	0.024131076	-3.641403587	0.000271156	0.00045455

THRA	1420.062607	0.125607498	0.03449188	3.641654206	0.000270892	0.000454147
CA10	0.793033041	-0.553582766	0.151986721	-3.642310093	0.000270202	0.00045303
PPP2R5B	668.831439	-0.204653767	0.056184508	-3.642530187	0.000269971	0.000452681
H2AC8	3153.789645	-0.274892271	0.075453156	-3.643217648	0.000269251	0.000451512
CCR3	20.72314957	-0.328869941	0.090268568	-3.643238693	0.000269229	0.000451512
SLCO4A1	308.40216	0.241715742	0.066339877	3.643596495	0.000268855	0.000450925
HECTD1	7808.306957	0.119815666	0.032877396	3.644317387	0.000268102	0.000449702
SETD1B	4606.567801	0.127810909	0.035056694	3.645834628	0.000266525	0.000447095
EPHB6	938.2255463	-0.193031904	0.052921511	-3.647513084	0.000264791	0.000444223
SRF	2352.946966	0.104461574	0.028631162	3.64852728	0.000263748	0.000442511
STMN3	723.2648464	-0.16480351	0.04516333	-3.649055767	0.000263206	0.00044164
CYP3A5	180.6084121	-0.191108872	0.05237092	-3.649141005	0.000263119	0.000441531
COPRS	227.0507096	-0.204642684	0.056062737	-3.65024424	0.000261991	0.000439677
ANKRD30A	0.759382512	-0.733105819	0.200832671	-3.650331471	0.000261902	0.000439565
PPP1R13B	169.2094135	-0.274305203	0.075130775	-3.651036489	0.000261184	0.000438398
ZCCHC17	348.9620505	-0.127534926	0.034926485	-3.651524823	0.000260688	0.000437602
TTLL3	1504.185375	-0.125201697	0.034280229	-3.652300507	0.000259901	0.00043632
EFCAB1	2.599853102	-0.5581567	0.152799147	-3.652878367	0.000259317	0.000435376
DCN	41.33529601	-0.457172352	0.125136101	-3.653400959	0.00025879	0.000434528
CHST13	211.3873187	-0.341140822	0.093366405	-3.653785545	0.000258402	0.000433914
PPP1R14A	39.97372639	-0.389069772	0.106476279	-3.654051187	0.000258135	0.000433503
AP000471.1	2.721668205	-0.300670465	0.082282938	-3.654104651	0.000258081	0.000433449
PITRM1	2252.192439	-0.089956825	0.024614822	-3.654579503	0.000257604	0.000432685
HCFC1	8776.99558	-0.117493035	0.032149141	-3.654624397	0.000257559	0.000432647
GBX1	1.846155291	-0.510400361	0.139646992	-3.654932712	0.000257249	0.000432164
KIF1C	578.7663087	0.203616924	0.055705618	3.655231373	0.00025695	0.000431698
SHKBP1	4251.826726	0.133257066	0.036451317	3.655754479	0.000256427	0.000430856
E2F2	2074.149097	-0.285667243	0.078119193	-3.656812505	0.000255371	0.000429119
IL10RB	134.8114595	0.145686226	0.039835069	3.657235419	0.00025495	0.000428449
TM2D1	659.6720927	-0.106921511	0.029232809	-3.657585899	0.000254602	0.0004279
NEUROG1	1.779215903	-0.635842086	0.173840644	-3.657614649	0.000254573	0.000427889
KRIT1	198.4762013	-0.130216354	0.035600128	-3.657749651	0.000254439	0.0004277
VSIG10L2	0.554417781	-0.577326326	0.157827338	-3.657961507	0.000254229	0.000427384
GTF2F1	2381.388218	0.129464928	0.035385676	3.658681831	0.000253516	0.000426221
COL4A5	570.3603241	-0.586824141	0.16035203	-3.659599077	0.00025261	0.000424735
MRFAP1	2294.236569	0.110425071	0.030173853	3.659627797	0.000252582	0.000424724
HSPA1L	105.9176575	-0.195758346	0.053482973	-3.660199397	0.000252019	0.000423814

CARD17	5.815091912	-0.430234734	0.117536332	-3.660440361	0.000251782	0.000423452
ZNF215	19.85045929	-0.456176902	0.124621593	-3.660496484	0.000251727	0.000423395
SYT12	8.967545978	-0.275962444	0.075386512	-3.660634223	0.000251592	0.000423204
HSPA1B	2089.181641	-0.382791874	0.104564719	-3.660812928	0.000251416	0.000422945
PURG	13.43010634	0.515943473	0.140929883	3.660994116	0.000251239	0.000422683
HNRNPCL4	0.668153812	0.590758739	0.161318481	3.662064844	0.000250191	0.000420956
ZNF792	733.5472011	-0.213775329	0.058361965	-3.662922075	0.000249354	0.000419585
MTCH2	1153.458162	-0.093542057	0.025535902	-3.663158576	0.000249124	0.000419234
OR9Q1	0.920636757	-0.709758041	0.193699681	-3.664218952	0.000248094	0.000417537
ASL	425.21091	-0.136572718	0.037271421	-3.664274525	0.000248041	0.000417482
DEF6	2905.041467	-0.100930612	0.027541798	-3.664634067	0.000247692	0.000416932
CASK	360.3858006	-0.196825135	0.053709253	-3.664641053	0.000247686	0.000416932
HEXIM2	244.5849817	-0.173803043	0.047406059	-3.666262192	0.000246122	0.000414359
COL9A2	869.02918	-0.239889233	0.065428566	-3.666429636	0.000245961	0.000414124
LRMDA	852.1908028	-0.24438288	0.066637222	-3.667362946	0.000245065	0.000412651
SERPINF1	486.3827231	-0.268030536	0.073084268	-3.667417677	0.000245012	0.000412598
FUOM	210.210791	-0.206212956	0.056223879	-3.667711263	0.000244731	0.00041216
NAT2	0.650855824	-0.69629085	0.189837854	-3.667818794	0.000244628	0.000412022
GNB1	10643.17528	0.096705052	0.02635679	3.669075449	0.000243429	0.000410038
GDE1	2111.613703	0.139107137	0.03791156	3.669253815	0.000243259	0.000409787
TXNL4B	331.2680912	-0.085823698	0.023387961	-3.66956739	0.000242961	0.00040932
KCNMB1	101.0633896	-0.272266326	0.074191488	-3.669778492	0.000242761	0.000409017
KCNA1	1.38188754	-0.627451583	0.170947015	-3.670444806	0.000242129	0.000407988
TMEM63A	3620.787213	-0.153575833	0.04183837	-3.670693479	0.000241893	0.000407626
SAMD9L	1009.347548	-0.216987631	0.059112739	-3.670742275	0.000241847	0.000407583
THG1L	351.005736	-0.132894362	0.036201063	-3.671007152	0.000241597	0.000407196
MANBA	3244.288276	0.108911202	0.029658785	3.672139707	0.000240528	0.00040543
ALKBH6	59.09107991	-0.130521626	0.035541829	-3.672338496	0.000240341	0.00040515
C11orf65	55.87295306	-0.220428534	0.060011977	-3.67307571	0.000239648	0.000404017
ZNF578	18.97695567	-0.344714597	0.093846271	-3.673183738	0.000239547	0.000403881
LIG3	2962.655435	-0.127059593	0.034590591	-3.673241437	0.000239493	0.000403825
RFC3	609.6978324	-0.125405266	0.034134689	-3.673836447	0.000238936	0.00040292
MCEE	153.3357473	-0.134402539	0.036580749	-3.674133092	0.000238658	0.000402487
C6orf201	15.21180117	0.208715058	0.05680525	3.674221279	0.000238576	0.000402382
STK38	2119.916546	-0.138276203	0.037621452	-3.675461619	0.00023742	0.000400467
COLGALT1	3758.997693	-0.156212349	0.042492637	-3.676221618	0.000236714	0.000399311
ENHO	7.443938361	0.583372476	0.158677843	3.676458318	0.000236494	0.000398975

VASP	4312.889945	0.143640859	0.039068839	3.676609374	0.000236355	0.000398773
C10orf82	1.337032215	-0.589836985	0.160408965	-3.67708242	0.000235917	0.000398069
MS4A4E	1283.053245	-0.346790807	0.094283586	-3.678167339	0.000234916	0.000396414
SMIM10L1	466.5390616	-0.11663464	0.031709673	-3.678203749	0.000234882	0.000396392
PDS5B	2809.412308	0.107325177	0.029175358	3.678624098	0.000234496	0.000395773
OR8D1	0.56551865	-0.799537078	0.217320452	-3.679069635	0.000234086	0.000395117
RRBP1	2888.633911	0.140153541	0.03809413	3.679137472	0.000234024	0.000395046
SPATA7	154.9635074	-0.13450418	0.036553568	-3.679645765	0.000233558	0.000394293
RRM2	1688.152347	-0.207358044	0.056341748	-3.680362258	0.000232903	0.000393221
CDC42EP2	4.53195763	-0.264258835	0.071799582	-3.680506604	0.000232771	0.000393032
CNN3	58.5983241	-0.342046605	0.092928029	-3.680768972	0.000232532	0.000392662
ATG14	2571.896444	0.16144612	0.043861081	3.680851386	0.000232456	0.000392569
SLC25A52	2.891191638	-0.287468355	0.07809749	-3.680891103	0.00023242	0.000392541
PABPC1	80648.6246	0.145110067	0.039421111	3.681024321	0.000232299	0.00039237
IL17RE	314.745941	0.374136331	0.101631806	3.681291756	0.000232055	0.000391993
TGFB1I1	140.2326695	-0.25925663	0.070412159	-3.68198667	0.000231424	0.000390959
TEX10	1386.092994	-0.116724382	0.031699549	-3.682209508	0.000231221	0.000390651
YLPM1	5679.245797	-0.080254404	0.021795007	-3.682238095	0.000231195	0.000390641
IQCJ-SCHIP1	36.60001875	-0.387009812	0.105070251	-3.683343364	0.000230195	0.000388984
RPL18A	30934.91914	-0.255078633	0.0692502	-3.683435314	0.000230112	0.000388877
SVOP	4.187093516	-0.286898671	0.077869908	-3.684333	0.000229302	0.000387543
FFAR2	222.2659515	0.36255478	0.098385713	3.685034847	0.000228671	0.00038651
IFIT3	1273.034106	0.360828465	0.097909739	3.685317392	0.000228418	0.000386115
KLC2	292.5905762	-0.192010334	0.052093736	-3.68586225	0.00022793	0.000385323
DES	35.18249537	0.507511757	0.137687027	3.685980946	0.000227823	0.000385176
ETFDH	458.6155865	-0.110376372	0.029941656	-3.686381664	0.000227465	0.000384604
H2BC15	1540.573946	-0.225215585	0.06108964	-3.686641193	0.000227233	0.000384245
TP53TG5	144.7130321	0.239903607	0.065063313	3.687233181	0.000226706	0.000383386
PGC	8.622339738	-0.335441262	0.090937245	-3.688711518	0.000225393	0.000381198
ATP6V1G2	18.09639842	-0.194147865	0.052630333	-3.688896745	0.000225229	0.000380954
IL27RA	760.3419582	0.182235205	0.049391447	3.689610542	0.000224598	0.00037992
MAGED1	1446.96728	-0.226647656	0.061419018	-3.690186939	0.000224089	0.000379092
ODR4	1068.402628	0.12628636	0.03422153	3.690260442	0.000224025	0.000379016
UNC5A	131.4969215	-0.389887205	0.105647387	-3.69045763	0.000223851	0.000378755
ZSWIM4	499.2444537	0.184192799	0.04990489	3.690876752	0.000223482	0.000378164
STAU1	3413.494549	0.111814077	0.030293704	3.691000548	0.000223374	0.000378012
METTL7B	188.1953345	-0.457083031	0.123832428	-3.691141633	0.00022325	0.000377836

ZNF737	349.8659734	-0.206113283	0.05583624	-3.691389006	0.000223033	0.000377501
TPT1	50749.08429	0.202405516	0.054820143	3.692174187	0.000222345	0.000376369
ELOVL5	3055.342347	0.120573947	0.032654687	3.692393284	0.000222154	0.000376078
TESK1	635.5189439	0.158125456	0.042822515	3.692577517	0.000221993	0.000375838
NMS	0.634589362	-0.863953689	0.233956905	-3.692789873	0.000221807	0.000375557
TMEM210	1.640614809	-0.44084414	0.119375783	-3.69291099	0.000221702	0.00037541
AGXT2	4.313241367	-0.414932296	0.112328291	-3.693925126	0.000220819	0.000373948
C1QA	178.2713795	0.420243255	0.113735959	3.694902289	0.000219971	0.000372545
TANC2	1298.881852	-0.223159309	0.060395514	-3.694964959	0.000219917	0.000372485
IGFLR1	50.69262056	-0.174817285	0.047311863	-3.694998941	0.000219888	0.000372468
OAZ1	12219.74433	-0.140630955	0.038053589	-3.695602952	0.000219366	0.000371615
GLUD2	23.32837218	0.185273476	0.050121565	3.696482276	0.000218608	0.000370363
MOGAT1	3.885502734	-0.478143669	0.12934917	-3.696534506	0.000218563	0.000370319
STRA8	2.630092454	-0.495695449	0.134082278	-3.696949785	0.000218205	0.000369746
LRRC75B	156.7282688	-0.184915349	0.050016544	-3.697083732	0.00021809	0.000369583
SEMG1	2.114750202	-0.555419099	0.150214901	-3.697496684	0.000217736	0.000369014
HS6ST1	765.3234764	-0.191987992	0.051923446	-3.69752024	0.000217716	0.000369012
GCA	1723.053422	0.228730557	0.061858791	3.697624094	0.000217627	0.000368893
CACNA1E	23.45764225	-0.426425416	0.115320628	-3.697737541	0.00021753	0.00036876
TXLNA	2167.37481	-0.106785082	0.028874378	-3.698264298	0.000217079	0.000368028
PI4KB	3439.921354	0.083265153	0.022512636	3.69859636	0.000216795	0.000367579
CXCL9	16.93805672	0.498763707	0.134749249	3.701421061	0.000214395	0.000363542
CDC73	2663.097972	0.119069148	0.032168248	3.70144955	0.000214371	0.000363532
CNPY1	0.917551562	-0.518087581	0.139964778	-3.701556845	0.000214281	0.00036341
OR56B1	11.73713248	0.361469689	0.097636652	3.702192601	0.000213744	0.000362532
TRAPPC4	702.7665473	0.113897355	0.030764553	3.70222686	0.000213715	0.000362514
TFEB	592.7171175	0.206854287	0.055870061	3.702417395	0.000213555	0.000362273
SDS	51.62765421	0.279331007	0.075443314	3.702528325	0.000213462	0.000362146
LONRF1	1846.304254	0.144709676	0.039066973	3.704143577	0.000212106	0.000359878
PPP1R26	274.5195002	-0.287200681	0.077519722	-3.70487244	0.000211497	0.000358876
AGA	401.5039004	0.131303328	0.035434317	3.705541349	0.00021094	0.000357961
FRZB	3.026042728	-0.552418774	0.149056719	-3.70609778	0.000210477	0.000357207
ECE2	0.857814417	-0.524793635	0.141593392	-3.706342706	0.000210274	0.000356893
DNAJC5	2569.026285	0.151125358	0.04077358	3.706452988	0.000210182	0.000356768
CAMK1	38.3083291	-0.259631188	0.07003891	-3.706956452	0.000209765	0.000356091
AFP	0.71234794	-0.703913405	0.1898171	-3.708377206	0.000208592	0.00035413
IGF1R	2152.173751	-0.276027431	0.074431554	-3.708473319	0.000208513	0.000354027

GLS	4171.026735	0.152396512	0.04108904	3.708933419	0.000208134	0.000353415
CTIF	566.5227396	-0.169188374	0.045616111	-3.708960908	0.000208112	0.000353407
DCBLD2	214.1378812	-0.263103414	0.070927958	-3.70944576	0.000207713	0.000352761
GJA3	21.67026715	-0.481311226	0.129725787	-3.710220141	0.000207079	0.000351715
ADSL	524.8094915	-0.146552111	0.039486462	-3.711452055	0.000206074	0.000350037
TREML4	79.58067488	0.328637628	0.088530826	3.712126543	0.000205525	0.000349136
KCNG4	2.291336961	-0.330777217	0.089099945	-3.712428972	0.00020528	0.000348749
PLEKHN1	25.76084456	0.298032865	0.080271681	3.712802105	0.000204977	0.000348265
GPR25	10.20428971	-0.488834032	0.131652355	-3.713067139	0.000204763	0.000347931
DHRS11	451.8571421	-0.189692664	0.051083534	-3.713381802	0.000204508	0.000347529
SELENOI	1221.531785	0.146569059	0.039453962	3.714938884	0.000203253	0.000345426
COPB2	2015.361723	-0.110685611	0.029793236	-3.715125471	0.000203103	0.000345201
PIGH	440.586593	0.124566899	0.033522457	3.715923942	0.000202462	0.000344142
TMEM87B	890.9141635	-0.104115093	0.028015569	-3.716329789	0.000202138	0.00034362
ZNF324	537.3487235	-0.112471386	0.030262843	-3.716484509	0.000202014	0.00034344
SOWAHD	163.300445	-0.292308639	0.078631069	-3.717469984	0.000201228	0.000342133
LIN7A	395.5396192	0.437095214	0.117577483	3.717507843	0.000201198	0.000342111
DMRT3	0.526094264	-0.625542366	0.168214671	-3.718714685	0.000200239	0.000340511
BCL2L14	13.43127201	-0.35488677	0.095387672	-3.720467899	0.000198854	0.000338185
H2AW	185.0488279	-0.432008527	0.116108993	-3.720715476	0.000198659	0.000337883
MRPL19	688.8434448	-0.110076664	0.029578729	-3.721480546	0.000198058	0.00033689
FRG1	783.5319889	0.148615389	0.039930352	3.721865203	0.000197757	0.000336406
KLRK1	21.35544554	0.311535944	0.083702907	3.721925034	0.00019771	0.000336356
CD38	3358.564046	-0.227068807	0.060998216	-3.722548331	0.000197222	0.000335555
AC004706.3	69.79101955	-0.176392843	0.047382196	-3.72276633	0.000197052	0.000335295
RASGEF1A	195.9428412	-0.208683052	0.056020771	-3.725101372	0.000195237	0.000332235
TGFB2	18.90730788	0.318980785	0.085628596	3.725166605	0.000195186	0.000332178
ZDHHC22	2.008980265	-0.404195533	0.108457805	-3.726753754	0.000193962	0.000330123
TCEAL4	415.7533501	-0.273754469	0.073444217	-3.727379508	0.000193481	0.000329333
ADPRH	380.2984387	0.176486986	0.047334952	3.72847081	0.000192645	0.000327939
PRMT9	665.9098733	0.135433513	0.036323241	3.72856361	0.000192574	0.000327847
LRRN3	81.74141107	-0.282008995	0.07562259	-3.729163393	0.000192117	0.000327096
BCL2L1	1888.794432	-0.277454685	0.074398369	-3.729311361	0.000192004	0.000326933
F2RL1	68.49590569	0.361510649	0.09692788	3.729686935	0.000191718	0.000326474
DLL3	31.15662803	0.472152934	0.126558068	3.730721743	0.000190932	0.000325164
MBNL3	2536.556765	-0.203220212	0.054468843	-3.730944203	0.000190763	0.000324905
PGA3	1.623870026	-0.570268824	0.152844479	-3.731039733	0.000190691	0.000324811

HS3ST6	2.218527858	-0.387721575	0.103910483	-3.731303749	0.000190491	0.000324498
CASKIN2	119.2665255	-0.276965645	0.07421007	-3.732184112	0.000189827	0.000323394
COLEC12	46.8336412	0.386577042	0.103560041	3.732878402	0.000189304	0.000322532
CEP76	359.5391997	-0.12222701	0.032741245	-3.7331204	0.000189122	0.00032225
WASHC2C	2152.287577	0.091283723	0.024442619	3.734612961	0.000188004	0.000320373
AL627230.1	2.442796445	-0.43474967	0.116407946	-3.734707854	0.000187933	0.00032028
CPLX4	3.215933749	-0.465467613	0.124625857	-3.734920067	0.000187775	0.000320038
OSMR	151.3710926	0.227119786	0.060805613	3.735177941	0.000187583	0.000319738
PWWP2B	809.3024342	-0.144007789	0.038551413	-3.735473692	0.000187362	0.00031939
DUS3L	1119.967973	-0.123864841	0.033153154	-3.736140484	0.000186866	0.000318573
USP32	3126.439623	-0.142343611	0.038073254	-3.738677346	0.000184991	0.000315403
NIPA2	1251.198051	0.090367706	0.024169704	3.738883393	0.000184839	0.000315172
MTRES1	131.8169882	-0.114201321	0.030539112	-3.73951021	0.000184379	0.000314415
TMEM238	77.6375488	-0.239149794	0.063952139	-3.739512036	0.000184378	0.000314415
KPNB1	6771.013122	-0.083690282	0.022379263	-3.739635299	0.000184287	0.000314313
NAT9	1090.090122	-0.102902572	0.027515634	-3.73978558	0.000184177	0.000314153
TLCD3A	73.27081841	-0.192696361	0.051516902	-3.740449325	0.000183692	0.000313352
DCLK2	73.15733039	-0.362562479	0.096913996	-3.741074505	0.000183235	0.0003126
FBXO21	672.6341147	0.188511101	0.05038799	3.741191106	0.00018315	0.000312482
FAM240A	0.558747684	-0.75487943	0.201752606	-3.741609324	0.000182846	0.00031199
SLC35G6	56.54740047	-0.245666875	0.065651485	-3.74198506	0.000182572	0.000311551
RSAD2	762.5136134	-0.329153581	0.087941209	-3.742882153	0.000181922	0.000310467
ABTB2	592.1506925	-0.236501214	0.063183697	-3.743073363	0.000181783	0.000310258
AL139142.2	0.334263439	-0.832356869	0.22233989	-3.743623649	0.000181385	0.000309606
PNPLA2	4188.45289	0.15805485	0.042200105	3.74536628	0.000180131	0.000307492
CRLF2	293.9994599	0.393726936	0.105112972	3.745750182	0.000179856	0.000307049
RABAC1	129.5830451	-0.202840532	0.05414107	-3.746518679	0.000179306	0.000306137
NPIPB7	5.519555923	-0.265317042	0.070813594	-3.74669644	0.000179179	0.000305946
RPF2	462.4176429	-0.114737821	0.030612771	-3.74803769	0.000178223	0.000304342
OR5A2	1.776412083	-0.469454972	0.125231599	-3.74869422	0.000177758	0.000303573
GCN1	5848.540425	-0.117947053	0.031456443	-3.749535645	0.000177162	0.000302583
MTMR11	447.7205574	0.36317346	0.096843176	3.750119277	0.00017675	0.000301906
MRGPRE	15.57484063	-0.306101276	0.081624408	-3.750119421	0.00017675	0.000301906
IL25	0.350315271	-0.63906698	0.17038925	-3.750629693	0.000176391	0.000301344
AGRP	28.9459902	-0.295691185	0.078828687	-3.751060618	0.000176088	0.000300853
TAGLN3	2.228545078	-0.397301109	0.105916663	-3.751072771	0.00017608	0.000300853
SYNJ1	1664.809579	0.172410518	0.045956427	3.751608442	0.000175704	0.000300249

CSDC2	16.23565858	-0.378347649	0.100847249	-3.751690326	0.000175646	0.000300177
BHLHA15	28.53374621	0.233252604	0.062171748	3.751745947	0.000175607	0.000300136
ACP3	447.4937046	0.28043181	0.074742265	3.751984379	0.00017544	0.000299877
CCDC88C	4675.688304	0.123400128	0.03288304	3.752698219	0.000174941	0.00029905
DENND2C	100.7905134	-0.375596219	0.100085617	-3.75274922	0.000174906	0.000299016
NANOS3	29.95016855	-0.240973556	0.064195889	-3.753722574	0.000174228	0.000297882
ACSM1	690.6828802	0.357980598	0.095349515	3.754403983	0.000173754	0.000297099
ZNF365	2.484062882	0.408107676	0.108685839	3.75492963	0.00017339	0.000296502
USP17L1	0.313427464	-0.773248482	0.205907155	-3.755325938	0.000173116	0.000296059
SFXN1	922.2363975	0.175797764	0.046812823	3.755333538	0.000173111	0.000296059
TMEM225	0.758239824	-1.087613786	0.289616267	-3.755361529	0.000173091	0.000296059
CHD7	2955.535742	0.23236554	0.061875052	3.755399485	0.000173065	0.00029605
WDR33	2982.715325	-0.103278714	0.027497529	-3.75592706	0.000172701	0.000295453
WNT4	170.2385013	-0.355650637	0.094677221	-3.756454112	0.000172338	0.000294857
COMMD6	2116.46193	-0.266907457	0.071052738	-3.756469689	0.000172327	0.000294857
FOXO4	1259.988499	-0.19211916	0.051128859	-3.757548346	0.000171586	0.000293623
ADGRF1	7.107308794	-0.469008837	0.124793447	-3.758280978	0.000171085	0.00029279
DHX30	2999.396276	-0.081716357	0.021738908	-3.758990798	0.0001706	0.000291986
ACP1	1902.052508	0.131140775	0.034884195	3.759317798	0.000170377	0.000291631
ZNF550	386.4231841	-0.145679167	0.038745888	-3.759861365	0.000170008	0.000291023
CMTR2	364.9626307	-0.155943944	0.041467699	-3.760612424	0.000169498	0.000290176
MYO9B	12181.13122	0.116248487	0.030911451	3.760693296	0.000169443	0.000290108
JAG1	1589.240273	0.305908792	0.081335208	3.761086977	0.000169177	0.000289677
PTCD1	222.8607786	-0.139080676	0.036976098	-3.761367009	0.000168987	0.000289378
PLEKHB1	174.3006271	-0.233442132	0.062063072	-3.761369253	0.000168986	0.000289378
LEPROT	2474.236832	0.128188748	0.034072226	3.762265174	0.000168381	0.000288391
PAPOLG	668.8071641	0.114212152	0.030356373	3.762378022	0.000168305	0.000288286
LEFTY2	5.529058767	-0.49350773	0.131159858	-3.762643076	0.000168127	0.000288006
CCDC107	32.64027708	-0.216837452	0.057619362	-3.763274096	0.000167703	0.000287305
DNAH9	14.47367356	-0.230111539	0.061138471	-3.763776457	0.000167367	0.000286753
SYCP2L	86.00768722	-0.436920806	0.116076461	-3.764077586	0.000167165	0.000286433
C5orf51	534.8121061	-0.15518162	0.041219888	-3.764726886	0.000166731	0.000285714
NMUR2	0.849015192	-1.027258767	0.272853625	-3.764871248	0.000166635	0.000285574
TMX2	1143.311242	-0.107181688	0.028461552	-3.765841288	0.000165989	0.000284493
ZNF28	239.7822738	-0.163336946	0.043371283	-3.766016037	0.000165873	0.000284319
PTPRG	96.8993818	-0.516708476	0.137198209	-3.766145923	0.000165787	0.000284196
SMIM30	286.4727257	-0.202261878	0.053702901	-3.766311931	0.000165677	0.000284032

CDC42BPG	297.587418	-0.219532592	0.058279604	-3.76688548	0.000165297	0.000283405
PRSS38	1.109680722	-0.588346224	0.156182559	-3.767041774	0.000165193	0.000283253
BCL7A	1030.970595	-0.211779137	0.05619495	-3.768650677	0.000164132	0.000281458
NMBR	0.38119804	-0.821347931	0.217882364	-3.769685238	0.000163454	0.000280319
PCBP1	417.1315686	0.175924409	0.046667995	3.769701491	0.000163443	0.000280319
PRKD3	1581.149419	0.209027319	0.05543987	3.770342894	0.000163023	0.00027963
GRB7	4.792049624	-0.366207728	0.097107763	-3.771147814	0.000162498	0.000278754
UQCRFS1	2726.366634	0.149378019	0.039602796	3.771905912	0.000162005	0.000277932
HMGCLL1	0.676144286	-0.74952231	0.198686224	-3.772391944	0.00016169	0.000277416
SLMAP	3687.868496	0.136170987	0.036096052	3.772462109	0.000161645	0.000277362
PHF20	2448.181506	0.102549829	0.027182863	3.772591125	0.000161561	0.000277243
RGPD6	11.2583292	0.291306388	0.077213214	3.772753061	0.000161456	0.000277087
HELZ2	3288.707097	0.253676027	0.06723169	3.773161518	0.000161192	0.000276658
C10orf99	1.89818272	-0.516773338	0.136940578	-3.773704958	0.000160841	0.00027608
AC021660.3	2.880024692	-0.5179714	0.137252511	-3.773857359	0.000160743	0.000275936
BAX	1366.159198	-0.140563399	0.037246319	-3.773886971	0.000160724	0.000275927
GPAM	234.5191056	-0.161971903	0.042911807	-3.774530053	0.00016031	0.00027524
DUOXA1	12.10097977	-0.365311325	0.096736177	-3.776367189	0.000159132	0.000273243
SEMA3B	183.0200179	-0.287286094	0.076071539	-3.77652532	0.000159031	0.000273094
SH3RF2	7.376688765	-0.354452202	0.093850373	-3.776779899	0.000158869	0.000272839
NOSTRIN	141.4539384	-0.239257541	0.06334885	-3.776825341	0.00015884	0.000272813
GSN	7235.672251	-0.209510645	0.055468437	-3.777114657	0.000158656	0.00027252
CRYBG2	35.71291959	0.275607935	0.07296685	3.777166427	0.000158623	0.000272488
OTUD7B	257.4952284	-0.284734445	0.075358638	-3.778391627	0.000157845	0.000271174
RAD18	720.0351116	-0.109155576	0.028886757	-3.778741072	0.000157623	0.000270818
PNMA1	711.2434998	-0.191061002	0.050543557	-3.780125779	0.000156749	0.00026934
H2BE1	3.910491008	-0.268011539	0.070867525	-3.781866796	0.000155657	0.000267486
SOX18	21.3105235	-0.313472931	0.082882237	-3.782148533	0.000155481	0.000267207
SERP1	4973.960449	0.124844261	0.033006874	3.782371491	0.000155341	0.000266991
FAM107B	5743.381918	0.221733749	0.05859072	3.784451705	0.000154048	0.000264791
CHIA	2.258947954	0.556734406	0.147094392	3.784878531	0.000153784	0.00026436
ALAS2	8950.556299	-0.438011292	0.115724157	-3.7849599	0.000153733	0.000264297
C20orf27	1244.634473	-0.223503667	0.059048117	-3.785110843	0.00015364	0.00026416
ZNF316	2216.743672	-0.110226472	0.029114254	-3.785996763	0.000153094	0.000263244
ART5	3.381764792	-0.415906577	0.109844353	-3.786326431	0.000152891	0.000262918
PARVB	1434.589945	-0.171840826	0.045377419	-3.786923789	0.000152524	0.00026231
KIF3A	138.3515204	-0.174298652	0.046019343	-3.787508451	0.000152166	0.000261717

CAMK2A	223.3341266	0.463269698	0.122315087	3.787510665	0.000152164	0.000261717
RARRES1	25.26319741	-0.234253554	0.061842769	-3.787889138	0.000151933	0.000261362
EBLN1	2.770041264	-0.626572997	0.165408598	-3.788031608	0.000151846	0.000261235
PERCC1	33.96336242	-0.27973428	0.073834071	-3.78868831	0.000151445	0.000260569
NEXN	111.7232326	-0.208419575	0.054999615	-3.789473326	0.000150967	0.000259769
OR2H1	0.829993776	-0.610636649	0.16113837	-3.789517356	0.00015094	0.000259746
ECT2L	99.45571	-0.241733845	0.063765273	-3.790995208	0.000150045	0.000258228
SOX13	78.51961947	0.353591211	0.093269809	3.791057507	0.000150007	0.000258186
UEVLD	213.4865423	-0.143781653	0.037924456	-3.791264703	0.000149882	0.000257993
HAT1	1570.491126	0.095416844	0.025166951	3.791354876	0.000149828	0.000257922
TFB2M	693.5957857	0.13844526	0.036508883	3.792097916	0.00014938	0.000257174
C19orf85	3.09228583	-0.392609563	0.103516226	-3.792734514	0.000148997	0.000256538
LAMTOR4	1473.380116	-0.238745364	0.062946713	-3.792817033	0.000148948	0.000256476
CEBPZOS	981.2084055	0.097972521	0.02583106	3.79281847	0.000148947	0.000256476
MARCOL	0.413203944	-0.681344866	0.179609529	-3.793478387	0.000148552	0.000255838
FSCN1	4066.452231	0.275061226	0.072503476	3.793766074	0.000148379	0.000255564
BEND3	224.4122292	-0.152784791	0.040264902	-3.794490591	0.000147947	0.000254842
HAUS1	381.9928573	-0.138657977	0.036541208	-3.7945647	0.000147903	0.000254788
CHRNA6	24.84659633	0.430972351	0.113561758	3.795048233	0.000147615	0.000254314
PUF60	2702.482447	-0.141758326	0.037349668	-3.795437338	0.000147383	0.000253938
EPN1	3483.884018	0.129644472	0.034153718	3.795910952	0.000147102	0.000253476
MB21D2	23.56904336	0.392076582	0.103253205	3.797234018	0.00014632	0.00025215
ADCK2	414.4245775	-0.131515534	0.034609229	-3.800013345	0.000144688	0.00024936
HYAL1	86.64545839	-0.217304751	0.057157681	-3.801846884	0.000143621	0.000247543
DUOX1	198.5299969	-0.224937396	0.059159157	-3.802241429	0.000143393	0.000247171
CYP27A1	126.3754213	0.424166695	0.111554335	3.8023327	0.00014334	0.000247102
FOXA1	9.609899279	0.440879933	0.115948626	3.802373069	0.000143317	0.000247083
IFT22	121.6194246	-0.196643271	0.051714588	-3.802471984	0.000143259	0.000247006
CD5L	35.49011095	-0.466170882	0.122582008	-3.802930699	0.000142994	0.000246571
EGLN1	453.0923835	0.222209805	0.058425786	3.803283104	0.000142791	0.000246242
PEPD	731.8364387	-0.181231571	0.047651142	-3.803299611	0.000142781	0.000246242
LSMEM1	44.05035558	-0.335355301	0.088167891	-3.803598994	0.000142609	0.000245972
JKAMP	708.2000508	0.121837714	0.032029554	3.80391546	0.000142427	0.000245679
PFDN4	515.2936214	-0.252324024	0.066321248	-3.804572899	0.000142049	0.000245049
TLL1	1.41472427	-0.678420383	0.178309094	-3.80474359	0.000141951	0.000244902
NUTM2F	4.883503153	-0.399035281	0.104844111	-3.805986595	0.00014124	0.000243696
RAI2	4.653504912	-0.573143428	0.150585269	-3.806105547	0.000141172	0.000243601

ADD3	2379.650174	0.185868125	0.048829875	3.806442745	0.00014098	0.00024329
PDGFA	88.20106902	0.376620038	0.09893877	3.806597119	0.000140892	0.00024316
RPP38	103.6165361	-0.137200687	0.036026915	-3.808282942	0.000139935	0.00024153
SNCAIP	11.33627693	-0.583454939	0.153157086	-3.809519732	0.000139237	0.000240346
MLLT11	199.3743936	-0.213041612	0.055896807	-3.811337754	0.000138217	0.000238606
RPS5	26448.9901	-0.250597817	0.065742449	-3.811811391	0.000137952	0.00023817
C12orf65	425.5393492	-0.141771208	0.037192256	-3.811847501	0.000137932	0.000238157
METTL24	19.71944103	-0.29058475	0.076226219	-3.812136482	0.000137771	0.000237899
CNGB1	6.20461672	-0.344691132	0.09041839	-3.812179493	0.000137747	0.000237879
NPFFR1	7.140571516	-0.355261445	0.093179302	-3.812664819	0.000137476	0.000237433
HMGXB3	3418.141223	0.110631388	0.029010218	3.813531752	0.000136995	0.000236622
RNPS1	5905.207255	0.110195851	0.028895058	3.813657413	0.000136925	0.000236523
GHR	15.74866233	-0.408327196	0.107062677	-3.813907952	0.000136786	0.000236304
GDAP1	151.5293149	-0.200022836	0.052445363	-3.813927962	0.000136775	0.000236304
KCNE5	607.0376891	-0.561854495	0.14730925	-3.814115521	0.000136672	0.000236147
KLHDC8B	161.0895598	-0.25119898	0.065860174	-3.81412567	0.000136666	0.000236147
CEP97	653.1867931	-0.130397393	0.034187298	-3.814205864	0.000136622	0.000236102
BRS3	3.410808398	-0.363573723	0.095306505	-3.814783915	0.000136302	0.000235571
ADAMTS7	119.4493925	-0.372527335	0.09761702	-3.816212955	0.000135516	0.000234232
GALNT11	927.6308076	-0.142569875	0.037344365	-3.817707883	0.000134697	0.000232838
GLP2R	8.42686625	-0.400902935	0.105006688	-3.817880029	0.000134603	0.000232697
IL27	32.73513355	0.266317886	0.069739011	3.818779225	0.000134114	0.000231871
AQP1	518.3637044	-0.35896019	0.093943347	-3.821028334	0.000132896	0.000229786
URM1	1152.411141	-0.151193659	0.039563762	-3.821518794	0.000132632	0.00022935
VSNL1	9.815435514	-0.307303583	0.080403146	-3.822034295	0.000132355	0.000228891
UPK1A	4.730868095	-0.30827893	0.080641428	-3.822835702	0.000131926	0.000228168
CYP2A7	11.03120913	-0.332137717	0.086869245	-3.823421256	0.000131613	0.000227647
ATP5PB	2804.939938	0.153750977	0.040211725	3.823535992	0.000131551	0.000227561
ZNF451	7806.420516	0.172856586	0.045208304	3.823558274	0.000131539	0.000227561
MKX	5.598003944	-0.402936333	0.105358426	-3.824433875	0.000131073	0.000226774
FLRT3	8.303924068	-0.607032258	0.158722624	-3.824484759	0.000131046	0.000226747
NHLRC3	1003.211987	-0.172986083	0.045225948	-3.82492995	0.000130809	0.000226358
XAB2	2930.280121	0.121242359	0.031697806	3.824944829	0.000130801	0.000226358
NR2C2	3106.780665	-0.129276408	0.033796761	-3.825112322	0.000130712	0.00022623
ASB3	1033.788183	-0.120604796	0.031520493	-3.826234401	0.000130118	0.000225222
ASB6	915.3713785	0.132453125	0.034612099	3.826786809	0.000129827	0.000224737
HMSD	9.088752529	-0.498512649	0.130242409	-3.82757547	0.000129412	0.000224038

PLCZ1	0.831646867	-0.607745062	0.15877469	-3.827720036	0.000129336	0.000223926
CD59	1198.229227	-0.290648491	0.075890192	-3.829855775	0.000128218	0.000222012
DDX5	41644.72288	0.103174445	0.026934465	3.830573359	0.000127845	0.000221385
ARPIN	124.6442687	-0.333433033	0.087040147	-3.830795844	0.000127729	0.000221204
NCKAP5L	2121.766254	0.152901141	0.039910414	3.831108883	0.000127567	0.000220942
TRPM4	952.64097	-0.517748482	0.13510379	-3.832227674	0.000126988	0.000219959
PFKP	3163.797363	-0.18012547	0.046993296	-3.833003518	0.000126588	0.000219286
PTH1R	43.45479377	-0.391676343	0.102168337	-3.833637268	0.000126262	0.000218741
PDZD7	153.0402902	-0.310620327	0.081024019	-3.833682073	0.000126239	0.00021872
HSD17B7	183.6022493	-0.162711055	0.042433794	-3.834468655	0.000125836	0.000218041
MICOS10	319.4403077	-0.179082121	0.046700851	-3.83466509	0.000125735	0.000217886
HMX1	0.406269187	-0.881507084	0.229853767	-3.835077819	0.000125525	0.00021754
STAC3	334.0630377	-0.233884091	0.060980439	-3.8353953	0.000125363	0.000217278
PZP	104.8806419	-0.338223434	0.088172368	-3.835934555	0.000125088	0.000216821
ANGPT4	8.972912854	-0.321240751	0.083732288	-3.836521828	0.000124789	0.000216322
PPIAL4F	0.706813406	-0.857896502	0.223591792	-3.836887279	0.000124604	0.00021602
ZC2HC1C	126.8871263	-0.185561063	0.048362119	-3.836909242	0.000124592	0.00021602
DNAH17	382.1699943	0.18965499	0.04942731	3.837048574	0.000124522	0.000215917
FICD	369.9483826	0.180529538	0.047045882	3.83730798	0.00012439	0.000215708
C1orf167	22.03622213	-0.356147437	0.092811332	-3.837327064	0.000124381	0.000215708
MAMDC4	761.7323915	-0.195185745	0.05086345	-3.837446045	0.000124321	0.000215625
PDCD6	498.2185272	-0.142771717	0.037198396	-3.838114896	0.000123982	0.000215058
SEMG2	0.680543053	-0.889206175	0.231655123	-3.838491305	0.000123793	0.000214747
C4orf17	2.576966382	-0.398708277	0.103862698	-3.838801456	0.000123636	0.000214495
EXOSC6	1539.605157	-0.108333645	0.028219491	-3.838965168	0.000123554	0.000214371
TBC1D22B	1286.072485	-0.14802803	0.038553968	-3.839501764	0.000123284	0.000213922
ZNF423	19.55988581	-0.421691283	0.109806165	-3.840324292	0.000122872	0.000213226
KCNB2	0.368331734	-0.81884888	0.213210368	-3.840567825	0.00012275	0.000213033
EPHB4	416.6296467	-0.248824236	0.064787984	-3.840592367	0.000122738	0.000213031
DAZL	2.755117411	-0.52649521	0.137084854	-3.840651947	0.000122708	0.000212998
MT1A	0.679476419	-1.066045777	0.277562356	-3.840743368	0.000122662	0.000212937
ARMC1	559.5650412	-0.112612311	0.029319398	-3.840880671	0.000122594	0.000212837
ZNF667	160.519393	-0.37055453	0.096463733	-3.841387001	0.000122341	0.000212417
PUS10	559.6315442	0.158094344	0.041150593	3.841848472	0.000122111	0.000212037
BNIP5	5.732366619	-0.437255891	0.113810609	-3.841960719	0.000122055	0.000211959
WASL	748.3374858	0.146285657	0.038072121	3.842330123	0.000121872	0.000211659
TRIM46	343.761158	-0.229277665	0.059655487	-3.843362589	0.00012136	0.000210789

IL1R2	214.409029	0.462376459	0.120295817	3.843662	0.000121212	0.00021055
MLEC	3331.617257	-0.123844064	0.032217134	-3.844043459	0.000121024	0.000210242
SEMA5A	18.8531512	-0.546883131	0.142235552	-3.844911645	0.000120596	0.000209517
ACCSL	1.511017647	-0.4380558	0.113926087	-3.845087744	0.000120509	0.000209386
INAFM2	1213.093266	0.163674185	0.042555935	3.846095391	0.000120015	0.000208545
MC2R	0.466832199	-0.727064445	0.18901172	-3.846663285	0.000119737	0.000208081
PPIB	1442.834474	0.16731082	0.043487412	3.847339049	0.000119408	0.000207527
NDE1	2474.334978	0.107782168	0.02801448	3.847373452	0.000119391	0.000207516
TLCD2	71.52228257	-0.265687301	0.069045387	-3.848009417	0.000119081	0.000206997
PLXNB3	105.9040134	-0.316972831	0.082370122	-3.848152971	0.000119012	0.000206894
MYO1D	262.4451991	-0.357651711	0.0929377	-3.848295276	0.000118943	0.000206792
AP000781.2	12.96558336	0.26595758	0.069082732	3.849841655	0.000118194	0.000205509
PQBP1	937.2783091	-0.151758056	0.039410839	-3.850667962	0.000117796	0.000204835
PRR33	485.1417277	-0.271909662	0.070607979	-3.850976416	0.000117648	0.000204595
CLDN1	2.744823106	0.542763116	0.140936861	3.851108313	0.000117584	0.000204503
C9orf152	32.03005278	0.329664043	0.085590839	3.851627645	0.000117335	0.000204088
CPA5	7.708568096	-0.376712873	0.097801242	-3.851820943	0.000117243	0.000203945
SLF1	390.9214144	-0.143378089	0.03721719	-3.852469531	0.000116933	0.000203424
ERCC6L2	1121.522428	-0.134984602	0.035032466	-3.853128786	0.000116618	0.000202895
FBXL17	827.3304531	-0.122167186	0.031702953	-3.853495452	0.000116443	0.000202609
S100B	233.745448	-0.370531677	0.096131025	-3.854444248	0.000115993	0.000201843
NEU1	1574.38	0.160508095	0.041633242	3.855286966	0.000115594	0.000201167
ALOX15B	20.89710675	0.415053154	0.107657067	3.85532659	0.000115575	0.000201152
PLSCR3	4.371050836	-0.220319893	0.057136857	-3.856003015	0.000115256	0.000200614
CXCR2	135.809523	-0.372459906	0.096561837	-3.857216442	0.000114686	0.000199639
R3HCC1	575.9622082	-0.14607954	0.037868769	-3.857520169	0.000114543	0.000199409
RABGGTA	714.2316579	-0.137751005	0.035708891	-3.857610875	0.000114501	0.000199353
MUC1	221.4059892	-0.229210878	0.059396481	-3.8589976	0.000113853	0.000198243
GALNT2	2105.200591	-0.135092031	0.035003434	-3.859393661	0.000113669	0.000197939
HPX	22.22460243	-0.278473428	0.072145644	-3.859878589	0.000113443	0.000197564
HSD3B2	0.409437503	-0.8627898	0.223515208	-3.860094392	0.000113343	0.000197408
SPINT4	0.336090919	-0.694206037	0.179832652	-3.860289169	0.000113253	0.000197268
TMEM263	670.5501828	0.184791979	0.047862822	3.860866751	0.000112986	0.00019682
ZNF449	103.7963351	0.154932085	0.040125449	3.861192563	0.000112835	0.000196575
TPD52L3	0.502774242	-0.543700133	0.140809957	-3.861233565	0.000112816	0.000196559
TMEM176A	243.0285232	0.459727853	0.119053766	3.861514586	0.000112686	0.000196351
THAP6	310.9392725	-0.121457547	0.031452349	-3.861636732	0.00011263	0.00019627

INPPL1	5480.87861	-0.124433156	0.032221648	-3.861787403	0.00011256	0.000196167
GHRH	4.712433087	-0.397879325	0.10302958	-3.861797015	0.000112556	0.000196167
NUTF2	1899.20042	-0.169916512	0.043991128	-3.86251776	0.000112224	0.000195616
HIGD1A	421.8889808	-0.196460606	0.050863187	-3.862530397	0.000112219	0.000195616
PRKX	1749.947422	0.142571125	0.03689935	3.86378418	0.000111644	0.000194638
NDUFB9	1801.838847	0.188327846	0.048736378	3.86421506	0.000111447	0.000194313
PPP1R14C	13.23260577	-0.540109819	0.139766497	-3.864372592	0.000111375	0.000194204
SRD5A1	191.92043	0.209963054	0.054319587	3.865328629	0.00011094	0.000193463
PRM2	0.931584421	-0.588108441	0.152138975	-3.865600118	0.000110816	0.000193265
AL590132.1	0.544434384	0.573113816	0.148229811	3.866387021	0.00011046	0.00019266
CISD2	513.4686544	-0.172186192	0.044530319	-3.866718153	0.00011031	0.000192415
MCOLN2	221.6449052	-0.443984082	0.114816459	-3.866902772	0.000110226	0.000192287
BUB3	2454.066963	-0.145252371	0.037556975	-3.867520466	0.000109948	0.000191818
TYW1B	354.4050921	-0.165422084	0.042771461	-3.867580843	0.00010992	0.000191787
CDK12	7960.406061	0.115213059	0.029782519	3.868479323	0.000109516	0.000191099
S1PR2	195.8871921	0.259378266	0.067044821	3.868729333	0.000109404	0.000190921
ZPLD1	1.531392185	-0.539801524	0.13952741	-3.86878481	0.000109379	0.000190894
CCDC152	20.20529594	-0.367348762	0.094940887	-3.869236655	0.000109177	0.000190558
AC244197.3	55.43363887	-0.187007744	0.04832942	-3.869439033	0.000109086	0.000190417
PAK6	171.6369316	-0.228856968	0.059143303	-3.869533112	0.000109044	0.00019036
ZBTB11	2900.279295	0.144209813	0.037266324	3.869708502	0.000108966	0.00019024
TSR2	702.1606178	0.127706412	0.032998865	3.870024345	0.000108824	0.000190011
NXPH2	2.16246468	-0.579070128	0.149601409	-3.870753155	0.0001085	0.000189461
RAB41	10.20869857	-0.16073533	0.041504394	-3.872730444	0.000107623	0.000187946
CRELD2	644.6140982	0.151170811	0.039026924	3.873500564	0.000107283	0.00018737
HAND2	0.50616444	-0.626698495	0.161766569	-3.874091531	0.000107023	0.000186932
RNF207	57.51997661	-0.282585801	0.072930907	-3.874705714	0.000106754	0.000186478
AL049779.1	10.02069194	0.251552865	0.064900041	3.876004702	0.000106186	0.000185503
AKAP4	0.989764151	-0.813379345	0.209801784	-3.876894319	0.000105798	0.000184842
PAK1	1916.147883	-0.192656351	0.049690501	-3.877126343	0.000105697	0.000184683
ZNF707	285.960379	-0.137206531	0.035382106	-3.87785094	0.000105383	0.00018415
C1QBP	2524.999154	-0.197434677	0.050903324	-3.878620522	0.00010505	0.000183585
SLC25A16	682.0199279	0.096273289	0.024819581	3.878924798	0.000104919	0.000183372
RPL34	27311.99783	-0.284640326	0.073369864	-3.879526403	0.00010466	0.000182935
SF3B5	1821.797097	-0.266148238	0.068591701	-3.880181336	0.000104379	0.00018246
WDR13	1476.399026	-0.108505315	0.027963003	-3.880317017	0.00010432	0.000182374
PTPN7	4678.425208	-0.109911895	0.028319394	-3.881152734	0.000103963	0.000181765

MAP3K11	3808.382248	-0.128493979	0.033097662	-3.882267532	0.000103487	0.00018095
RAB17	34.47491458	0.389178953	0.100236342	3.882613283	0.00010334	0.000180709
RNF165	61.72459164	-0.358949805	0.092444869	-3.882852671	0.000103238	0.000180547
RPRML	2.597147186	-0.394422127	0.101563268	-3.883511563	0.000102959	0.000180074
BCHE	0.702647418	-0.978829125	0.252028598	-3.883801813	0.000102836	0.000179875
HOXC5	0.462167976	-0.984684344	0.253511235	-3.884184246	0.000102674	0.000179608
DNAJC30	149.1251152	-0.127743611	0.032883874	-3.884688637	0.000102461	0.000179252
AC073283.3	0.784312854	-0.589750738	0.151809587	-3.884805629	0.000102412	0.000179182
HS3ST3A1	11.98631904	-0.457342689	0.117693696	-3.885872426	0.000101963	0.000178413
OR1R1P	0.715693501	-0.634134357	0.163143389	-3.886975508	0.000101501	0.00017762
RPS27L	1354.052841	-0.138647807	0.035669548	-3.887007686	0.000101488	0.000177612
MICU3	52.54370722	-0.323531875	0.083232613	-3.887080612	0.000101457	0.000177575
TPI1	7411.281793	-0.176288934	0.045349886	-3.887307136	0.000101362	0.000177425
SLC5A1	1.855305898	-0.375696605	0.096625924	-3.888155364	0.000101009	0.000176822
NPIPB5	593.8176298	-0.170605154	0.043846988	-3.890920748	9.98645E-05	0.000174834
CYP4F12	21.72005246	-0.391790744	0.100687244	-3.891165654	9.97638E-05	0.000174674
MAP2K3	8031.851118	0.190203673	0.048880664	3.891184318	9.97561E-05	0.000174674
ZCCHC12	15.59674786	0.409400133	0.105187403	3.892102286	9.93793E-05	0.000174032
PARD6A	47.66520127	-0.194239056	0.049905696	-3.892121999	9.93713E-05	0.000174032
LEO1	472.0321665	-0.104887691	0.026948586	-3.892140745	9.93636E-05	0.000174032
MAD2L1	440.6595109	-0.17540071	0.04505978	-3.892622445	9.91664E-05	0.000173705
FEV	5.801006975	-0.691553164	0.177638367	-3.893039424	9.89961E-05	0.000173423
NDUFB8	276.1934431	-0.173091233	0.044455584	-3.893576859	9.87769E-05	0.000173054
HOXB8	95.51005836	-0.824245626	0.211669589	-3.894020057	9.85965E-05	0.000172753
MKRN2OS	108.9328571	-0.208884109	0.053638467	-3.894296792	9.8484E-05	0.000172572
GATAD2B	4612.040544	0.120647231	0.030973502	3.895175583	9.81276E-05	0.000171963
FANCB	207.7727539	-0.146054144	0.037495313	-3.895264028	9.80918E-05	0.000171915
CTDP1	3030.78227	0.167957096	0.043106355	3.896341892	9.76565E-05	0.000171168
SMIM18	57.82432362	0.221528411	0.056854925	3.896380294	9.7641E-05	0.000171156
SGCZ	1.285223299	-1.033979756	0.26533459	-3.896890176	9.74358E-05	0.000170812
BCL2L2	990.3969796	0.12646359	0.032452183	3.8969209	9.74234E-05	0.000170805
PLXDC1	72.784521	0.25607198	0.065710092	3.896996213	9.73931E-05	0.000170767
SSR4	3318.113198	-0.235488098	0.060417441	-3.897684058	9.7117E-05	0.000170298
ATP1B3	2295.345972	0.137926596	0.035384203	3.897970943	9.70021E-05	0.000170112
CEP126	22.58875636	-0.339282131	0.087040608	-3.897975179	9.70004E-05	0.000170112
PNLDC1	34.03097687	-0.287203949	0.07366707	-3.898674812	9.67206E-05	0.000169649
SINHCAF	2697.752053	0.110987	0.028464064	3.899197286	9.65121E-05	0.000169298

CLEC4A	14.30651782	0.25440978	0.06524394	3.899362581	9.64463E-05	0.000169198
CT55	0.879035152	-0.61373217	0.157334922	-3.900800664	9.58751E-05	0.000168211
OGFOD1	552.1784098	-0.096277749	0.024680295	-3.900996735	9.57974E-05	0.00016809
CCDC7	380.9033086	-0.193337385	0.049558284	-3.901212244	9.57122E-05	0.000167955
PSMF1	3292.30872	-0.157894129	0.040466729	-3.901825824	9.54698E-05	0.000167545
RAPGEF4	41.80431037	-0.336168746	0.086153416	-3.901978137	9.54098E-05	0.000167455
SNTB1	1789.905757	-0.17270749	0.044258525	-3.902242307	9.53057E-05	0.000167287
MOK	298.9735771	-0.208617438	0.053455685	-3.902623948	9.51555E-05	0.000167038
TNFSF15	30.48586987	0.384090794	0.098400015	3.903361118	9.4866E-05	0.000166545
LYRM2	612.8205728	0.109846894	0.028140746	3.903482	9.48186E-05	0.000166477
SLCO3A1	1517.971456	0.181562595	0.046506142	3.904056241	9.45938E-05	0.000166097
MTM1	752.4854293	0.106700398	0.027327533	3.904501621	9.44198E-05	0.000165806
LHX5	0.968612712	-0.571833162	0.146440712	-3.904878313	9.42728E-05	0.000165563
OR5K1	1.302818377	-0.590351141	0.151179322	-3.904972792	9.4236E-05	0.000165513
GP5	29.97613845	-0.371573106	0.095125492	-3.90613598	9.37837E-05	0.000164734
KCNJ3	3.214510614	-0.616349893	0.157763466	-3.906797363	9.35275E-05	0.000164299
OTUD6A	0.928341538	-0.420503534	0.107625094	-3.907114209	9.3405E-05	0.000164098
FAM114A1	102.4399951	0.246527597	0.063093943	3.907310071	9.33294E-05	0.00016398
LSM10	437.4161434	-0.174805036	0.04473352	-3.907696845	9.31801E-05	0.000163732
NBPF9	243.7698821	0.189603432	0.048519718	3.907760403	9.31556E-05	0.000163704
FAM217A	114.0294123	0.200024815	0.051178672	3.908362751	9.29237E-05	0.000163311
OOSP3	2.265888345	-0.409818898	0.104830315	-3.909354838	9.25429E-05	0.000162657
CLEC12B	97.02395476	-0.30985514	0.079259465	-3.909377117	9.25344E-05	0.000162656
HELZ	7017.895779	0.121830411	0.031163025	3.909453903	9.2505E-05	0.000162619
PCMTD1	1656.760974	-0.12456199	0.031861077	-3.909534778	9.2474E-05	0.000162579
MACO1	2704.99745	0.175090901	0.044782391	3.909815799	9.23665E-05	0.000162405
TIMM17B	680.6775973	-0.162498884	0.041558798	-3.910095864	9.22595E-05	0.000162231
JAG2	61.25971123	-0.348214701	0.08904925	-3.910360835	9.21583E-05	0.000162068
SIX6	0.520394325	-0.616660648	0.157694162	-3.910484954	9.2111E-05	0.000161999
UBP1	3398.998502	0.110043436	0.028129111	3.912083718	9.15032E-05	0.000160945
LYPLA2	912.9820252	-0.105815316	0.027044247	-3.912673775	9.12798E-05	0.000160566
TOR4A	926.6759684	-0.177153573	0.045266204	-3.913594661	9.09322E-05	0.000159969
COPZ1	1931.691466	-0.134926424	0.034468896	-3.914440004	9.06143E-05	0.000159424
NUDT21	2514.689411	0.099987926	0.025542956	3.914500944	9.05914E-05	0.000159398
MAP2K2	3045.200558	-0.128999629	0.032952955	-3.914660463	9.05315E-05	0.000159307
ZNF852	439.1081328	-0.151947274	0.038814899	-3.914663591	9.05303E-05	0.000159307
PBXIP1	2886.758069	-0.14577857	0.037236072	-3.914982435	9.04108E-05	0.000159123

STS	344.6100388	0.242108658	0.061840786	3.915032041	9.03922E-05	0.000159105
TAF9	1673.205166	0.158935051	0.040593569	3.915276629	9.03006E-05	0.000158958
TBRG1	1350.153272	0.090581658	0.023134554	3.915426965	9.02444E-05	0.000158873
NELFE	1121.826094	-0.147259073	0.037603847	-3.916064045	9.00063E-05	0.000158468
THAP1	184.1229552	0.149338127	0.038132689	3.91627584	8.99274E-05	0.000158344
AKAP8	2742.888266	0.110467606	0.028206498	3.916388531	8.98853E-05	0.000158284
CDC14A	1904.38799	0.162088636	0.041378726	3.917197329	8.95844E-05	0.000157768
MFSD10	5273.814656	0.166169183	0.042415437	3.917658135	8.94134E-05	0.000157481
JMJD1C	19391.65505	0.17942752	0.045797988	3.917803561	8.93595E-05	0.0001574
UGDH	225.3478604	-0.179513042	0.045814472	-3.918260654	8.91902E-05	0.000157116
TMEM74B	7.404732991	-0.297176571	0.075841555	-3.918387131	8.91435E-05	0.000157048
ANKRD53	28.58718413	-0.225513556	0.057544208	-3.918961875	8.89312E-05	0.000156688
NCR2	20.04777157	-0.807853974	0.206124408	-3.919254301	8.88234E-05	0.000156512
POLR1H	322.162459	-0.171090504	0.043652908	-3.919338094	8.87925E-05	0.000156472
PANX3	2.719581683	-0.400768731	0.102222505	-3.920552845	8.83461E-05	0.000155699
ARID3B	2465.838591	0.194192875	0.049519833	3.921517133	8.79932E-05	0.000155091
PITX1	19.52809918	-0.487773397	0.124364291	-3.922133865	8.77682E-05	0.000154709
AAR2	849.1924811	0.125268594	0.031931197	3.923078607	8.74246E-05	0.000154117
TRIM71	741.3596121	0.428544031	0.109211885	3.923968808	8.7102E-05	0.000153562
FGF11	18.53328468	-0.253728095	0.064659819	-3.924045832	8.70742E-05	0.000153527
OLFML1	17.73541011	-0.298553406	0.076066566	-3.924896584	8.6767E-05	0.000152999
DAPP1	873.3420805	0.186961273	0.047632594	3.92507013	8.67045E-05	0.000152903
KIFBP	221.925546	-0.135954758	0.03463219	-3.925676053	8.64865E-05	0.000152532
CRISPLD1	113.5093742	-0.345609675	0.088025785	-3.92623223	8.62869E-05	0.000152194
NBEAL2	12943.13077	-0.203267509	0.051769608	-3.926386859	8.62314E-05	0.00015211
PCGF5	1676.07774	0.204179158	0.051999727	3.926542909	8.61755E-05	0.000152025
OXGR1	0.931333207	-0.720203923	0.183383048	-3.927320052	8.58976E-05	0.000151548
NRF1	1036.51697	0.119596689	0.03044999	3.927642977	8.57824E-05	0.000151358
CYB5R1	669.3293317	-0.13644438	0.034739345	-3.927661283	8.57759E-05	0.000151358
MGAT4A	1008.828574	0.181155779	0.046117777	3.928111651	8.56155E-05	0.000151091
ZNF280A	0.830234432	-0.715490229	0.182144615	-3.928143745	8.5604E-05	0.000151085
C17orf64	17.74108417	-0.216021461	0.054987034	-3.928589058	8.54457E-05	0.000150819
BRD1	3728.254435	0.141506437	0.036011835	3.929442513	8.5143E-05	0.000150298
PLA2G2C	33.31949527	0.438275032	0.111512516	3.930276596	8.48482E-05	0.000149791
CFAP45	191.5607813	0.252419369	0.064203782	3.931534277	8.44055E-05	0.000149023
RNF125	1884.087102	0.192149669	0.048870168	3.931839707	8.42983E-05	0.000148847
DNAJC14	509.8960662	0.104617183	0.026597429	3.933356924	8.37677E-05	0.000147924

PLAGL1	2010.802638	-0.179376499	0.045603017	-3.933434874	8.37405E-05	0.000147889
CTAG2	0.644869233	-0.670601606	0.170485233	-3.933487932	8.3722E-05	0.00014787
SAMD12	33.00014439	-0.359787398	0.091443919	-3.934514202	8.33651E-05	0.000147253
TMEM41A	1067.945481	-0.132014131	0.033550813	-3.934752042	8.32826E-05	0.00014712
SLC25A43	234.1808957	-0.196817361	0.050011817	-3.935417145	8.30523E-05	0.000146727
VAC14	1481.912553	-0.111910917	0.028429172	-3.936481818	8.26849E-05	0.000146091
GTPBP8	303.3773107	0.110993638	0.028195017	3.936640255	8.26303E-05	0.000146007
C1GALT1C1	186.2781244	-0.172391478	0.043789514	-3.936821	8.25681E-05	0.000145911
SCRT2	4.157822675	-0.425204612	0.108005	-3.936897485	8.25418E-05	0.000145877
PRM1	0.543437401	-0.656110592	0.166604957	-3.938121663	8.2122E-05	0.000145148
LRG1	493.180635	0.272920525	0.069300937	3.938193859	8.20973E-05	0.000145118
S100A5	12.51891887	0.282406081	0.071708456	3.938253542	8.20768E-05	0.000145095
AC011005.1	12.79576181	0.201237417	0.051090566	3.938837086	8.18775E-05	0.000144756
C11orf49	1251.763224	-0.156077611	0.039609499	-3.940408655	8.13429E-05	0.000143823
DPP7	2783.778314	-0.177255113	0.044982684	-3.940518857	8.13056E-05	0.00014377
SETD1A	3404.404043	0.154402566	0.03918048	3.940803312	8.12092E-05	0.000143613
HBD	4979.751674	-0.448051834	0.113669131	-3.941719538	8.08995E-05	0.000143078
ADAM20	25.51997517	-0.310771951	0.078831176	-3.942246816	8.07218E-05	0.000142777
ALDH8A1	72.18528868	-0.340999618	0.086488895	-3.942698281	8.057E-05	0.000142521
MYL5	413.5814296	-0.140583044	0.035656289	-3.94272784	8.05601E-05	0.000142516
LBX2	54.35124333	-0.2223317	0.056384337	-3.943146446	8.04195E-05	0.000142281
ZNF669	412.7436108	0.131756415	0.033388682	3.946140029	7.94212E-05	0.000140527
CDO1	8.701025165	-0.340300164	0.086228985	-3.946470695	7.93116E-05	0.000140346
KRT7	3.601254641	0.428422806	0.108537687	3.947226236	7.90618E-05	0.000139917
IRF2BP2	13154.05723	0.13042429	0.033037374	3.947780196	7.88792E-05	0.000139606
SLC5A3	2382.537328	0.240402303	0.060891455	3.948046596	7.87915E-05	0.000139463
AHCY	2587.729562	-0.171576388	0.043450471	-3.948780878	7.85502E-05	0.000139049
PFN1	11450.15375	-0.251881197	0.063754738	-3.950783993	7.78956E-05	0.000137903
RTP5	75.89300674	0.419256694	0.106108866	3.951193795	7.77623E-05	0.000137679
NRG4	356.9842963	-0.331115324	0.08379848	-3.951328539	7.77185E-05	0.000137614
AMER2	2.868375163	-0.453729778	0.114804166	-3.952206573	7.74339E-05	0.000137122
F8A3	6.651591895	0.370147228	0.093646836	3.952586572	7.73109E-05	0.000136917
FSTL5	3.389802168	-0.71362336	0.18049206	-3.953765946	7.69307E-05	0.000136256
ZNF773	198.1610635	0.164571947	0.041622867	3.953883015	7.6893E-05	0.000136202
JMJD7	42.44537856	-0.216824933	0.054834158	-3.954194661	7.67929E-05	0.000136036
TMEM126A	457.6196347	0.154122699	0.038965998	3.955312449	7.64347E-05	0.000135414
KRT20	0.572739246	-0.750743505	0.189786957	-3.955717072	7.63054E-05	0.000135197

OR6K2	0.421751378	-0.757882333	0.191578874	-3.955980723	7.62213E-05	0.000135061
USP53	555.0134572	0.23484715	0.059355482	3.956621028	7.60174E-05	0.000134711
TMEM86A	81.27212187	0.259746033	0.065602362	3.95940061	7.51381E-05	0.000133165
ZMPSTE24	600.5605985	0.11206099	0.028289167	3.961268571	7.45526E-05	0.00013214
ACBD7	34.21344515	-0.273948074	0.069144941	-3.961939516	7.43434E-05	0.000131781
ANKRD40	787.6241812	0.077012081	0.019436696	3.962200322	7.42622E-05	0.000131649
COX14	312.6636812	-0.177943312	0.044904166	-3.962735087	7.4096E-05	0.000131366
INIP	429.7195054	-0.130540879	0.032939971	-3.962993141	7.40159E-05	0.000131236
RRAGA	932.3291887	0.127182088	0.032091588	3.963097322	7.39836E-05	0.000131191
PECR	67.77220391	-0.324246489	0.081812846	-3.963271137	7.39297E-05	0.000131107
OR2F1	0.795502528	-0.641559864	0.161860667	-3.963655129	7.38109E-05	0.000130908
LCN15	5.44991571	-0.344584676	0.086921699	-3.964311342	7.36081E-05	0.00013056
ZNF844	340.6425863	-0.206753253	0.052145228	-3.964950584	7.34112E-05	0.000130223
TNK1	67.32952449	-0.199437498	0.050286689	-3.966009746	7.30859E-05	0.000129657
TPRG1L	793.0885646	-0.170755814	0.043038186	-3.967542083	7.26177E-05	0.000128838
SLC23A1	220.8746995	-0.203040246	0.051170693	-3.967901055	7.25084E-05	0.000128656
TOGARAM2	136.4734965	0.251277228	0.063324576	3.968083887	7.24528E-05	0.000128569
CAND1	4140.815998	0.113625613	0.028633992	3.968207246	7.24153E-05	0.000128514
RNF112	10.70397753	0.344040438	0.08668095	3.969043246	7.21618E-05	0.000128076
FAM210B	2024.042775	-0.268140349	0.067556105	-3.969150518	7.21293E-05	0.00012803
TCF7	3470.341231	0.241632562	0.060877558	3.969156587	7.21275E-05	0.00012803
GUCA1C	0.664813283	-0.702548612	0.176986296	-3.969508527	7.2021E-05	0.000127861
CNPPD1	2126.928686	-0.143566014	0.036166977	-3.969533145	7.20136E-05	0.000127859
DLX5	1.599668249	-0.661729671	0.166694592	-3.969712907	7.19593E-05	0.000127775
ROBO4	133.4300079	-0.40332876	0.10159987	-3.969776361	7.19401E-05	0.000127752
POTEH	0.521727246	-1.029373477	0.25923393	-3.970828495	7.16231E-05	0.000127201
CDH9	30.60854147	-0.826459357	0.208128747	-3.97090439	7.16003E-05	0.000127172
TRIM69	418.4868885	-0.21515895	0.054182795	-3.970982865	7.15767E-05	0.000127141
BOD1	279.3176182	0.120867994	0.030433262	3.971575327	7.13989E-05	0.000126837
AMD1	8277.335718	0.13459107	0.033888316	3.971606954	7.13894E-05	0.000126832
CENPVL3	0.341116994	-1.179369966	0.296887003	-3.972454007	7.1136E-05	0.000126393
SPTSSB	1.793794069	-0.479610499	0.120714893	-3.973084731	7.09478E-05	0.00012607
FAM237A	0.4009538	-0.934716737	0.235260977	-3.973105735	7.09415E-05	0.00012607
PCBP4	108.1119686	0.256969465	0.064636878	3.975585946	7.02062E-05	0.000124775
NARF	3338.641247	-0.168514041	0.042371356	-3.977074513	6.97683E-05	0.000124008
TTC29	2.439379071	-0.775071458	0.194837171	-3.978047183	6.94836E-05	0.000123513
RTF2	1898.874249	0.094978339	0.023874138	3.978293898	6.94115E-05	0.000123396

PROK1	0.715408612	-0.608967426	0.153057812	-3.978675876	6.93001E-05	0.000123209
HOMER2	683.5334643	0.264719652	0.06653401	3.978711842	6.92897E-05	0.000123202
CXorf65	9.905688502	0.229409385	0.057654241	3.979054791	6.91898E-05	0.000123036
RPS6KB2	1194.585743	0.112544081	0.028264836	3.981770132	6.84039E-05	0.000121649
CFP	270.623887	0.254160403	0.063817343	3.982622766	6.81589E-05	0.000121224
IRS1	115.8072021	0.310726169	0.078020077	3.982643728	6.81529E-05	0.000121224
EIF3A	9372.769553	0.096079006	0.024117268	3.983826315	6.78144E-05	0.000120634
BCL7C	330.7032618	-0.160430506	0.040269418	-3.983929117	6.77851E-05	0.000120592
TMEM140	811.6991925	0.17545017	0.044026356	3.985116793	6.74469E-05	0.000120002
SMIM29	483.8928397	0.165772396	0.041584876	3.986362655	6.70939E-05	0.000119385
LRP1B	20.07873084	-0.588643186	0.147624773	-3.987428214	6.67934E-05	0.000118861
ALS2	1416.627341	-0.196294579	0.049226633	-3.987568684	6.67539E-05	0.000118801
SLC18B1	160.9821078	-0.295896028	0.074199912	-3.987821794	6.66827E-05	0.000118685
C5orf46	0.785433548	-0.836896228	0.209856309	-3.98794887	6.6647E-05	0.000118632
SLC4A11	48.17326507	-0.301294238	0.075536327	-3.988732943	6.64272E-05	0.000118252
PCLAF	397.7589904	-0.217367678	0.054477545	-3.990041748	6.60617E-05	0.000117612
CHKA	2021.896715	0.169557154	0.042487121	3.9907894	6.58537E-05	0.000117252
LALBA	1.469862629	-0.599098349	0.15008681	-3.991678884	6.56072E-05	0.000116824
NEMF	2946.304607	0.127108449	0.031837076	3.99246618	6.53897E-05	0.000116447
SBSN	1.686045975	-0.41469145	0.103867219	-3.992515193	6.53761E-05	0.000116434
BNIP1	452.3648421	0.147526019	0.036950422	3.99253951	6.53694E-05	0.000116432
NIP7	596.8535917	0.106395251	0.026646371	3.992860888	6.52809E-05	0.000116285
ASIC1	77.95036115	0.32620027	0.08169579	3.992865115	6.52797E-05	0.000116285
NMB	126.4719357	-0.232111189	0.058125825	-3.993254091	6.51727E-05	0.000116114
PMP2	0.796377975	-0.715307928	0.17910754	-3.993734312	6.50407E-05	0.000115889
AC099489.1	908.6125226	-0.337342571	0.084463936	-3.993924373	6.49886E-05	0.000115807
FGF16	3.966601191	-0.492637982	0.123345058	-3.993982326	6.49727E-05	0.000115789
NOMO2	678.7643009	0.152676395	0.038212236	3.995484458	6.45621E-05	0.000115068
GFM2	593.4449433	-0.09862207	0.02468207	-3.995696886	6.45042E-05	0.000114975
USP25	2145.890432	0.131519875	0.032913568	3.995916634	6.44444E-05	0.000114879
TRIM23	505.3595421	0.17320485	0.043339812	3.996437536	6.43028E-05	0.000114637
SLC6A5	0.622720645	-0.870754007	0.217768625	-3.998528279	6.37376E-05	0.00011364
ELANE	16190.44005	0.436935477	0.109273441	3.998551448	6.37313E-05	0.000113639
VGLL3	23.7881694	-0.479335712	0.119874398	-3.998649591	6.37049E-05	0.000113602
RO60	2286.086043	0.140337969	0.035093579	3.998964302	6.36203E-05	0.000113461
PCDHGC3	136.3892211	-0.301343685	0.075354638	-3.999006491	6.36089E-05	0.000113452
NACAD	45.16065137	0.321442832	0.080321078	4.001973597	6.28163E-05	0.000112048

GSDMD	2138.015211	-0.168278396	0.042043556	-4.002477735	6.26826E-05	0.00011182
AAK1	5403.880158	0.148529212	0.037108491	4.002566779	6.2659E-05	0.000111788
CRYZL1	794.0836662	-0.123086827	0.030751256	-4.002660177	6.26342E-05	0.000111754
ALAS1	2801.447854	0.194403603	0.048562719	4.003144924	6.2506E-05	0.000111535
CRY1	675.5378305	0.229183732	0.057231849	4.004478902	6.21543E-05	0.000110918
ZNF524	243.4355708	-0.183384159	0.045791836	-4.0047348	6.20871E-05	0.000110808
TFAM	1141.229045	0.135635114	0.033847935	4.007190234	6.14454E-05	0.000109673
APCDD1L	5.657561438	-0.353440368	0.088193335	-4.007563244	6.13484E-05	0.00010951
CDK2AP1	1525.409478	-0.197929183	0.04938173	-4.008145977	6.11973E-05	0.00010925
WASF2	12672.78715	0.104211008	0.025997497	4.008501566	6.11052E-05	0.000109095
THRSP	7.732940388	0.242304703	0.060446953	4.008551135	6.10924E-05	0.000109082
UBE2I	3184.222357	0.105745294	0.026379339	4.008640719	6.10692E-05	0.000109051
HNRNPM	9433.794059	0.114329651	0.028514515	4.009524722	6.08411E-05	0.000108654
JAK2	2280.060657	-0.162329595	0.040484706	-4.009652316	6.08082E-05	0.000108605
IQCM	0.551744752	-0.647608532	0.161511911	-4.009664231	6.08051E-05	0.000108605
B3GNT8	241.3340823	-0.215150604	0.053649684	-4.010286473	6.06451E-05	0.000108333
C1D	593.7278827	0.169276792	0.042210019	4.010346297	6.06298E-05	0.000108316
BPGM	1701.291117	-0.34070814	0.084940471	-4.011140242	6.04262E-05	0.000107962
DPYSL2	1995.270592	-0.363079782	0.090516408	-4.01120404	6.04099E-05	0.000107943
CREBZF	2392.42802	-0.134339484	0.033491037	-4.011207002	6.04091E-05	0.000107943
RTCB	2210.009411	0.11165933	0.027835448	4.011407659	6.03578E-05	0.000107869
DYNLRB2	4.261333226	-0.334519837	0.083389955	-4.011512381	6.0331E-05	0.000107831
SPNS2	1074.847499	-0.298804852	0.07448423	-4.011652529	6.02952E-05	0.000107777
TIMM22	386.8197897	-0.124768664	0.031100223	-4.011825402	6.02511E-05	0.000107708
ZNF230	217.5552897	0.168879164	0.042092952	4.012053236	6.01929E-05	0.000107614
F3	162.9578943	0.376792477	0.093911567	4.012205197	6.01542E-05	0.000107554
RBBP6	6679.378691	0.126133832	0.031436993	4.012274094	6.01366E-05	0.000107533
FUBP1	7716.066437	0.10704405	0.026672796	4.013229398	5.98937E-05	0.000107108
MPP1	4674.042512	-0.201256752	0.050145568	-4.013450418	5.98376E-05	0.000107018
SACS	2089.563148	0.18585306	0.046306713	4.013523091	5.98191E-05	0.000106994
MCHR1	6.771533105	-0.318781436	0.079415497	-4.01409608	5.9674E-05	0.000106745
FGR	3957.269552	0.307223695	0.076534901	4.014164669	5.96567E-05	0.000106723
C19orf12	312.8168175	-0.135099517	0.033644153	-4.015542167	5.93093E-05	0.000106112
TMEM45A	27.77345505	-0.422426931	0.105194787	-4.015664128	5.92786E-05	0.000106066
TP53I3	448.6653667	-0.120868309	0.030098066	-4.015816458	5.92403E-05	0.000106008
PIK3R2	18.48652176	-0.195977364	0.048793552	-4.016460265	5.90788E-05	0.000105728
USP22	10469.44705	0.129106698	0.032143296	4.016598029	5.90443E-05	0.000105676

E2F7	241.6456446	-0.269142027	0.067001382	-4.0169623	5.89532E-05	0.000105523
CDK5R2	1.121779231	-0.532687218	0.132608538	-4.016990369	5.89461E-05	0.00010552
NEK2	225.6710725	-0.223487342	0.055629974	-4.017390742	5.88461E-05	0.00010535
H4C5	2086.221815	-0.289170204	0.071947533	-4.019181626	5.84006E-05	0.000104562
SCYL1	1848.060767	0.117255844	0.029171061	4.019594831	5.82983E-05	0.000104389
BASP1	945.8039984	0.371621964	0.092446632	4.019854007	5.82342E-05	0.000104283
LIMD1	1732.319111	-0.167075458	0.041556393	-4.020451432	5.80867E-05	0.000104029
NEK6	1696.393645	-0.161862943	0.040241082	-4.022330782	5.76251E-05	0.000103211
GATA2	3628.096384	-0.331913816	0.082515833	-4.022425797	5.76018E-05	0.000103179
NFATC2	1357.823547	-0.1931599	0.048015711	-4.022847848	5.74986E-05	0.000103004
EMX1	39.66426034	-0.315865534	0.078511506	-4.023175057	5.74188E-05	0.00010287
SOCS7	1160.34041	-0.13571173	0.033724588	-4.02411828	5.71892E-05	0.000102468
MFF	1448.610146	0.106453485	0.026449357	4.024804255	5.70227E-05	0.000102179
TMEM19	260.1043641	-0.15402664	0.038268108	-4.024934833	5.69911E-05	0.000102132
SHC3	8.823290123	-0.332562624	0.082617247	-4.025341399	5.68927E-05	0.000101965
ARL3	303.0539748	-0.169827986	0.042185483	-4.025744706	5.67953E-05	0.0001018
CHPF	92.4314462	-0.329916379	0.0819432	-4.026159348	5.66953E-05	0.00010163
ATP2C2	92.52601609	-0.44334694	0.110107307	-4.026498807	5.66135E-05	0.000101493
TRIM62	312.729603	0.153008326	0.037988053	4.027801237	5.63009E-05	0.000100941
CPB2	22.66509518	-0.31303585	0.077712504	-4.028127169	5.62229E-05	0.000100811
PMAIP1	3314.943404	0.229429127	0.056955311	4.028230628	5.61982E-05	0.000100776
GPANK1	543.8061128	-0.120008721	0.02979013	-4.028472524	5.61404E-05	0.000100681
KCTD1	209.7023947	-0.25967102	0.064454244	-4.028765275	5.60706E-05	0.000100565
LRP8	1376.135874	-0.176403351	0.043781562	-4.029169901	5.59742E-05	0.000100402
SP140L	868.9037692	0.141920171	0.035223122	4.029176335	5.59726E-05	0.000100402
PLXNB1	739.7978265	-0.300054833	0.07446433	-4.029510969	5.5893E-05	0.000100274
NIPA1	813.4007253	-0.311783156	0.077372731	-4.029625855	5.58657E-05	0.000100235
CYHR1	1240.503682	-0.111617341	0.02769795	-4.029805193	5.58231E-05	0.000100167
GSTA2	0.432521948	-1.301376625	0.32292389	-4.029979395	5.57817E-05	0.000100102
SON	28166.92121	0.113261739	0.028098018	4.030951121	5.55516E-05	9.96985E-05
SPDYE1	135.4754273	0.155938791	0.038683301	4.031165604	5.55009E-05	9.96166E-05
TTI2	284.7492332	0.122405455	0.030345017	4.033790905	5.48842E-05	9.85187E-05
HMBOX1	2240.173884	-0.130087624	0.032244357	-4.034430759	5.47348E-05	9.82596E-05
EGFL7	1907.390239	-0.349014174	0.086489135	-4.03535281	5.45203E-05	9.78835E-05
FZD6	158.4179495	-0.356386537	0.088307468	-4.03574631	5.4429E-05	9.77285E-05
RPGR	1151.211037	0.172142788	0.042649675	4.036204005	5.4323E-05	9.75471E-05
CYFIP1	1888.441458	-0.202766261	0.050234262	-4.036413697	5.42745E-05	9.7469E-05

IGSF9B	120.8927312	0.372041128	0.092156548	4.037055809	5.41262E-05	9.72116E-05
CADM2	7.660670533	-0.774034161	0.19171091	-4.037507094	5.40222E-05	9.70337E-05
GLYATL1B	4.950858017	-0.367433847	0.090988429	-4.038248041	5.38519E-05	9.67367E-05
TSKS	49.25018057	-0.394941589	0.097787621	-4.038768753	5.37325E-05	9.65311E-05
BGLAP	15.93427421	-0.248451594	0.061515099	-4.038871706	5.37089E-05	9.64976E-05
NDUFB5	893.5960728	-0.197526109	0.048900317	-4.03936255	5.35967E-05	9.63047E-05
FBXL18	574.2374044	-0.144036412	0.035642498	-4.041142412	5.31914E-05	9.55854E-05
AC005520.1	32.74208397	0.19387799	0.047975032	4.041226867	5.31723E-05	9.55597E-05
VENTX	176.1136933	-0.446059503	0.110364925	-4.041678131	5.307E-05	9.53848E-05
FAM220A	156.5334207	-0.157378713	0.038936811	-4.041900386	5.30198E-05	9.53031E-05
TP53TG3D	3.601801746	-0.528539708	0.130758784	-4.04209714	5.29753E-05	9.52319E-05
HSPD1	6825.013329	-0.200097548	0.049502616	-4.042161075	5.29608E-05	9.52147E-05
CD34	6558.077433	-0.479590285	0.118621397	-4.043033542	5.27641E-05	9.48697E-05
VWA7	220.8032861	0.283380808	0.070073259	4.044064929	5.25324E-05	9.44617E-05
TMEM253	7.449577603	-0.302131526	0.074645467	-4.047553595	5.17557E-05	9.30738E-05
TYW1	936.1496718	-0.093474072	0.023079534	-4.050084968	5.1199E-05	9.20811E-05
POU6F1	218.5745625	0.197386399	0.048732676	4.050391169	5.11321E-05	9.19692E-05
BEND5	204.8855699	-0.19322124	0.047685919	-4.051955875	5.07912E-05	9.13645E-05
A2M	281.6476806	-0.304703879	0.075170708	-4.053492224	5.04587E-05	9.07746E-05
CDCA7L	1216.924117	-0.18154051	0.044785366	-4.053567605	5.04424E-05	9.07537E-05
SYNDIG1L	1.868225596	-0.440215761	0.108582957	-4.05418838	5.03087E-05	9.05214E-05
TAF1C	1436.162861	-0.141686672	0.034940915	-4.055036064	5.01266E-05	9.0202E-05
MEF2D	12051.96225	0.167612581	0.04132568	4.055894074	4.99429E-05	8.98798E-05
SMIM42	0.77830178	-1.002474743	0.247129893	-4.056468969	4.98202E-05	8.96672E-05
FAM13A	678.9809683	-0.199075116	0.049072681	-4.056740191	4.97624E-05	8.95714E-05
PRR15L	21.74741537	-0.235721984	0.058104434	-4.056867423	4.97353E-05	8.95308E-05
ZAP70	1072.052428	0.275650935	0.067944334	4.057011356	4.97047E-05	8.94839E-05
CENATAC	1063.507959	0.116077085	0.028600592	4.058555345	4.93772E-05	8.89026E-05
RABGAP1	1416.523208	-0.13474085	0.033185777	-4.060198778	4.9031E-05	8.82873E-05
SMIM27	259.926968	-0.151021921	0.037191663	-4.060639064	4.89386E-05	8.81291E-05
CALCOCO2	5824.892677	0.125013725	0.030768816	4.063000872	4.84458E-05	8.72498E-05
NOX3	2.101376696	-0.67957377	0.167249041	-4.063244654	4.83953E-05	8.71667E-05
CALCA	2.276327946	-0.493330226	0.121411976	-4.063274837	4.8389E-05	8.71634E-05
ZNF443	147.0716534	-0.217552645	0.053529332	-4.064176353	4.82024E-05	8.68353E-05
PDC	12.09571461	-0.319556932	0.078617905	-4.064683867	4.80976E-05	8.66545E-05
EXTL1	7.968369734	-0.347520595	0.085492199	-4.064939263	4.8045E-05	8.65676E-05
NDUFS5	1845.328726	-0.316719171	0.077912287	-4.065073459	4.80173E-05	8.65258E-05

SYDE1	35.47315812	-0.314235744	0.077288282	-4.06576178	4.78758E-05	8.62787E-05
AFG3L2	1529.501681	0.091817123	0.022565302	4.068951709	4.72251E-05	8.5114E-05
VIPR1	108.4585249	0.305905789	0.075152081	4.070489925	4.69144E-05	8.45617E-05
TCP1	7116.752284	0.115169863	0.028289623	4.071099201	4.67918E-05	8.43485E-05
SNX12	883.8132149	0.096715118	0.023749693	4.072268124	4.65575E-05	8.39339E-05
FAM162B	0.848223642	-0.67446188	0.165577676	-4.073386565	4.63344E-05	8.35394E-05
CGB3	1.451551743	-0.561585421	0.137844921	-4.074037824	4.6205E-05	8.33137E-05
GNG12	13.07003894	-0.402411719	0.098767177	-4.074346684	4.61437E-05	8.32109E-05
C1orf232	0.745949437	-0.508268798	0.124720962	-4.075247566	4.59654E-05	8.2897E-05
MPP2	76.1910009	0.381594541	0.093626524	4.075709797	4.58742E-05	8.27401E-05
ITPKB	2732.674554	0.171476502	0.042070084	4.075972466	4.58224E-05	8.26544E-05
BLK	231.1302733	0.307721806	0.075484837	4.076604211	4.56982E-05	8.24378E-05
KLK15	0.627587186	-0.680502128	0.166910586	-4.077045939	4.56115E-05	8.2289E-05
CPNE8	522.535652	-0.487776659	0.119629241	-4.077403287	4.55415E-05	8.21703E-05
AC108488.2	3.068692123	-0.32443175	0.079552554	-4.078206575	4.53844E-05	8.18945E-05
TPP2	7005.71517	0.138223979	0.033891769	4.078393764	4.53479E-05	8.18361E-05
IER5L	160.7387081	0.314521824	0.077106497	4.079057339	4.52187E-05	8.16104E-05
GPR158	4.438005504	-0.807959521	0.198063697	-4.079291332	4.51732E-05	8.15359E-05
IL1R1	289.5261501	0.372046088	0.091190991	4.079855733	4.50637E-05	8.13457E-05
SLC2A4	137.5023045	-0.413978196	0.101464675	-4.08002289	4.50313E-05	8.12947E-05
TRIM60	1.19916715	-0.549950806	0.134789981	-4.080057006	4.50247E-05	8.12903E-05
CYP26B1	4.002294672	-0.529846087	0.129853987	-4.080322066	4.49733E-05	8.12051E-05
GALNTL6	30.78730818	-0.422973296	0.10364342	-4.081043407	4.4834E-05	8.09609E-05
IFI6	1412.935173	-0.353937422	0.086710367	-4.08183515	4.46815E-05	8.0693E-05
TMEM209	441.7797765	-0.136036759	0.033319859	-4.082753103	4.45053E-05	8.03822E-05
RDH16	13.16031729	-0.227224146	0.055644399	-4.083504363	4.43616E-05	8.01301E-05
ATP6V1A	2491.334383	0.14007583	0.034285785	4.08553662	4.39751E-05	7.94392E-05
CCDC47	2369.748162	0.129408774	0.031671611	4.085954918	4.38959E-05	7.93035E-05
EIF2S3B	104.5765711	0.28108125	0.068785882	4.086321788	4.38266E-05	7.91856E-05
AHNAK	50406.84455	0.267615921	0.065485388	4.086650916	4.37645E-05	7.90807E-05
HMX2	26.26800119	-1.274894115	0.311948887	-4.086868618	4.37235E-05	7.90139E-05
ODAPH	8.311877025	-0.319912787	0.078277544	-4.086903737	4.37168E-05	7.90092E-05
DRAP1	1139.346874	-0.24681019	0.060380306	-4.087594217	4.3587E-05	7.87818E-05
CTR9	1363.048261	-0.148497546	0.036328369	-4.087646889	4.35771E-05	7.87712E-05
CHAC1	81.76737299	-0.411356361	0.100631942	-4.087731506	4.35612E-05	7.87497E-05
AC003002.2	3.560491651	-0.320706389	0.078454938	-4.087778252	4.35524E-05	7.87411E-05
LGALS9	3321.790549	-0.240337126	0.058783625	-4.088504689	4.34163E-05	7.85022E-05

PLEK2	82.0277475	-0.445574297	0.108976999	-4.088700393	4.33797E-05	7.84433E-05
OR5BS1P	0.684459794	-0.735155693	0.179789999	-4.088968781	4.33295E-05	7.83598E-05
NUDT11	10.19360177	-0.505837761	0.123685395	-4.089712933	4.31907E-05	7.81161E-05
ZNF534	9.502681063	-0.342930503	0.083851589	-4.089731699	4.31872E-05	7.81161E-05
CCDC187	23.42271421	-0.373307567	0.091250309	-4.091027957	4.29465E-05	7.76888E-05
ADGRB1	233.7925541	-0.300643338	0.07347776	-4.091623625	4.28363E-05	7.74966E-05
EXOSC9	1064.359527	0.124204144	0.030353626	4.091904677	4.27845E-05	7.74099E-05
EML6	195.7415646	-0.270337938	0.066064803	-4.092011578	4.27647E-05	7.73813E-05
LHX6	64.47142188	-0.488001383	0.119251941	-4.092188177	4.27322E-05	7.73296E-05
CEP295NL	592.0453721	0.279056792	0.068178007	4.093061748	4.25714E-05	7.70458E-05
TRIM50	6.936038149	-0.333457201	0.081437749	-4.094626916	4.22849E-05	7.65343E-05
SLC9A1	1556.67966	0.186230416	0.045465014	4.0961258	4.20122E-05	7.60477E-05
OCLN	158.9493097	-0.435802743	0.10638751	-4.096371324	4.19677E-05	7.59742E-05
UST	74.55860542	-0.315186513	0.076933741	-4.096856696	4.18798E-05	7.58222E-05
CLEC18A	29.93483022	-0.298074715	0.072755964	-4.096911067	4.187E-05	7.58114E-05
GDF9	62.44424903	-0.316866296	0.077338899	-4.097114103	4.18333E-05	7.57519E-05
ZFYVE27	1679.184291	0.114528015	0.02795043	4.097540363	4.17563E-05	7.56196E-05
SIKE1	1092.782818	0.098010937	0.023919449	4.097541666	4.17561E-05	7.56196E-05
LCMT1	680.1380877	-0.110596127	0.026989306	-4.097775962	4.17139E-05	7.55567E-05
PSMA7	3522.585088	-0.170117896	0.041505868	-4.098646864	4.15573E-05	7.528E-05
RHOXF2B	1.273564233	-0.836550778	0.204079569	-4.099140266	4.14688E-05	7.51266E-05
ZNF766	819.6175945	0.131980616	0.032196388	4.099236765	4.14515E-05	7.51023E-05
NOL7	1045.755625	-0.222715306	0.0543254	-4.099653351	4.13769E-05	7.49742E-05
AC008397.2	2.710621987	-0.495753445	0.12092283	-4.099750591	4.13596E-05	7.49496E-05
MEFV	565.7377906	0.380157776	0.092722288	4.099961125	4.1322E-05	7.48884E-05
RANBP3	1886.602684	0.120392995	0.029356137	4.101118485	4.11158E-05	7.45217E-05
OLIG1	208.5981335	0.39196017	0.095539317	4.102605943	4.08523E-05	7.40509E-05
UGT3A2	93.55641534	0.536450209	0.130730441	4.10348352	4.06975E-05	7.37773E-05
UBE2QL1	6.064849416	-0.534052998	0.13013208	-4.103930391	4.0619E-05	7.36416E-05
AC025283.2	1.503281293	-0.409035177	0.0996607	-4.104277603	4.0558E-05	7.35379E-05
ST6GALNAC4	918.763787	-0.230655912	0.056196129	-4.104480477	4.05224E-05	7.34803E-05
MTRNR2L13	0.971079524	-1.014974216	0.247281069	-4.104536672	4.05126E-05	7.34692E-05
SHLD2	727.2508162	0.100084697	0.024381875	4.104881069	4.04523E-05	7.33666E-05
ADAMTS17	57.15202202	-0.290347312	0.070717584	-4.105730088	4.0304E-05	7.31044E-05
AMY2B	208.723193	-0.163826722	0.039898907	-4.106045311	4.02491E-05	7.30116E-05
VPS11	2347.888211	0.085525428	0.020828606	4.106152249	4.02304E-05	7.29846E-05
CDKAL1	1093.92822	-0.142233177	0.034637109	-4.106381335	4.01906E-05	7.2919E-05

UGT1A1	0.806034454	-0.507827198	0.123666612	-4.106421189	4.01836E-05	7.29132E-05
SFRP1	8.957339839	-0.53955897	0.131393278	-4.106442712	4.01799E-05	7.29132E-05
SRGAP1	276.7599968	-0.350001056	0.085205584	-4.107724413	3.99576E-05	7.25166E-05
SMARCD1	2467.107651	0.130719521	0.031821461	4.107904401	3.99265E-05	7.24668E-05
NR4A1	2318.599869	0.323388619	0.078719824	4.108096317	3.98934E-05	7.24133E-05
TAF1	3297.180555	-0.147918964	0.036005864	-4.108190907	3.9877E-05	7.23904E-05
PIGW	110.0961349	-0.153880417	0.037456529	-4.108240187	3.98685E-05	7.23817E-05
CMA1	3.925738659	-0.462096999	0.112471098	-4.108584393	3.98092E-05	7.22806E-05
GPR34	53.54808286	0.319807206	0.077828599	4.109121966	3.97166E-05	7.21193E-05
CLEC10A	242.7346778	0.611909057	0.148907926	4.109311522	3.9684E-05	7.20668E-05
RFTN1	1310.150616	0.177635917	0.043227297	4.109345969	3.96781E-05	7.20627E-05
ADCY9	2182.122638	-0.426091128	0.103676535	-4.109812581	3.9598E-05	7.19239E-05
SLC3A2	4717.487653	0.143325666	0.034861827	4.11124944	3.93524E-05	7.14844E-05
SERPINB6	2921.48586	-0.205425802	0.049962319	-4.111614672	3.92902E-05	7.1378E-05
SLC25A31	0.568161418	-0.77931134	0.189448446	-4.113580001	3.8957E-05	7.07793E-05
C4orf48	192.1213929	-0.376985169	0.091639569	-4.113781581	3.8923E-05	7.0724E-05
RDH5	50.80253321	-0.183724032	0.044639306	-4.115745725	3.8593E-05	7.01309E-05
OR2A25	0.694042748	-0.586976877	0.142571482	-4.117070745	3.83718E-05	6.97355E-05
CDCA4	1004.077392	0.141130921	0.034270419	4.118155705	3.81917E-05	6.94146E-05
TCL1B	0.938880267	-0.677399643	0.164485865	-4.118284833	3.81703E-05	6.93821E-05
MAFF	3116.335309	0.261769575	0.063556428	4.118695487	3.81023E-05	6.92651E-05
RPS6KA5	954.8747913	-0.209246194	0.050802847	-4.118788764	3.80869E-05	6.92435E-05
DTL	1158.037159	-0.161378467	0.039160021	-4.121000561	3.77231E-05	6.85883E-05
ZNF713	155.0069934	-0.170925064	0.041471722	-4.121484619	3.76439E-05	6.84507E-05
FAM155B	2.912570226	-0.726155737	0.176156892	-4.122210205	3.75255E-05	6.82417E-05
FOXD4L5	0.543990242	-0.620919069	0.150621822	-4.122371255	3.74992E-05	6.82004E-05
SAP30L	660.7025175	-0.179116023	0.043436193	-4.123658387	3.72902E-05	6.78265E-05
ZNF503	398.6041144	0.412985371	0.100148529	4.123728786	3.72788E-05	6.7812E-05
AGO4	3884.202915	0.129126628	0.03131303	4.123734708	3.72778E-05	6.7812E-05
ZNF880	232.0588578	-0.208847009	0.050641414	-4.124035869	3.72291E-05	6.77342E-05
LIN28B	54.4608337	-1.241102561	0.300939078	-4.124099042	3.72189E-05	6.77219E-05
PTRHD1	294.3397636	-0.282130604	0.068399223	-4.124763299	3.71116E-05	6.75331E-05
MAOA	54.46452234	-0.403178335	0.097743419	-4.124864271	3.70953E-05	6.75097E-05
H3-3A	4713.384509	0.113562667	0.027531175	4.124875406	3.70936E-05	6.75097E-05
WNT3A	4.718373225	-0.384322682	0.093138556	-4.126354319	3.68559E-05	6.70865E-05
LRRC4B	33.66759396	-0.274341011	0.066485052	-4.126356299	3.68556E-05	6.70865E-05
SMLR1	3.469499103	-0.505735944	0.122552334	-4.126693694	3.68016E-05	6.70001E-05

ZHX3	747.704421	-0.163570164	0.039631963	-4.12722843	3.67162E-05	6.68507E-05
MDM4	12394.2227	0.137528349	0.03331816	4.127729385	3.66363E-05	6.67115E-05
PRKCZ	138.3123881	-0.305021645	0.073887698	-4.128179023	3.65647E-05	6.65874E-05
B4GALT5	3065.692465	0.195045151	0.047240086	4.128806029	3.64652E-05	6.64123E-05
TMEM35A	2.713173661	-0.584405382	0.141513454	-4.129680703	3.63267E-05	6.61663E-05
HDAC7	2854.746384	-0.11899424	0.028813739	-4.129774346	3.63119E-05	6.61455E-05
SKOR2	0.926939004	-0.677059699	0.163943953	-4.129824166	3.63041E-05	6.61374E-05
CES4A	67.37519273	-0.340046297	0.08233103	-4.130232519	3.62397E-05	6.60261E-05
MSMO1	417.7708003	0.225163237	0.054513099	4.130442768	3.62065E-05	6.59719E-05
MSL1	3826.533134	0.10403249	0.025173215	4.13266597	3.5858E-05	6.53429E-05
IFNAR1	1475.004897	0.113742638	0.027521358	4.132886074	3.58236E-05	6.52864E-05
MED29	1569.559134	0.117171844	0.028343589	4.133980471	3.56534E-05	6.49822E-05
C16orf96	73.76449869	-0.227200913	0.054951859	-4.134544628	3.55659E-05	6.48288E-05
CNIH3	83.99280019	-0.227790539	0.055092805	-4.13466948	3.55466E-05	6.47996E-05
BMPR1A	59.75855559	-0.407714784	0.098586448	-4.13560679	3.54018E-05	6.45417E-05
JMJD6	2562.834895	0.145980879	0.035290111	4.136594524	3.52498E-05	6.42706E-05
BLOC1S1	211.7261548	-0.239656079	0.057934213	-4.136693448	3.52346E-05	6.42489E-05
CERK	2204.638902	0.151998004	0.036739748	4.137154216	3.5164E-05	6.4126E-05
OR8B8	0.48431048	-0.665692138	0.160884927	-4.137691149	3.50818E-05	6.39821E-05
SPTLC1	1392.678114	0.075638588	0.018276215	4.138635256	3.49378E-05	6.37254E-05
XPO1	6575.551933	-0.121632974	0.029384929	-4.139297835	3.4837E-05	6.35476E-05
TMEM169	6.807554342	-0.404572636	0.097739321	-4.139302691	3.48363E-05	6.35476E-05
IQCJ	1.206702047	-0.777669071	0.187843803	-4.139977254	3.4734E-05	6.33715E-05
EIF3H	9703.0538	0.183758985	0.04437656	4.140901964	3.45943E-05	6.31224E-05
RTL3	9.631877055	-0.370959267	0.089581897	-4.141007036	3.45784E-05	6.30994E-05
CCDC192	9.814851923	-0.375184895	0.090575075	-4.142253204	3.4391E-05	6.27632E-05
ATF7IP2	1065.200073	-0.188092944	0.045406484	-4.142424776	3.43653E-05	6.27221E-05
TSFM	30.32425238	-0.175381831	0.042333216	-4.142889394	3.42957E-05	6.2601E-05
DPP8	2112.877082	0.108910265	0.026279959	4.144232718	3.40953E-05	6.2241E-05
QPCT	265.094163	0.374641737	0.090378854	4.145236645	3.39463E-05	6.19747E-05
TNS3	2807.63092	-0.285647587	0.068902421	-4.145682909	3.38802E-05	6.18599E-05
VTA1	825.3265697	-0.119460174	0.028813718	-4.145947854	3.38411E-05	6.17942E-05
OSTN	1.808099808	-0.689572063	0.16630378	-4.146460554	3.37654E-05	6.16618E-05
FFAR4	5.760936101	0.421231485	0.101576077	4.146955632	3.36925E-05	6.15344E-05
PKD1	3481.32678	-0.177039722	0.042685191	-4.147567736	3.36026E-05	6.13759E-05
ZNF287	30.31076765	-0.282797231	0.068173686	-4.148187498	3.35118E-05	6.12157E-05
CHMP1B	2086.076194	0.117839699	0.028396806	4.149751914	3.32836E-05	6.08045E-05

PLSCR2	10.83044766	-0.418150968	0.100760052	-4.149967779	3.32522E-05	6.07529E-05
ZNF675	453.3365439	0.16196402	0.039017289	4.151083382	3.30905E-05	6.04631E-05
OR10G3	3.592201236	-0.456817566	0.110036642	-4.151504048	3.30297E-05	6.03577E-05
NECAB1	60.18795615	-0.451634974	0.108775399	-4.151995563	3.29589E-05	6.02338E-05
NSMCE3	529.6756618	0.177487678	0.042747316	4.152019202	3.29555E-05	6.02332E-05
CASC3	4249.459775	0.086134114	0.020744399	4.152162377	3.29348E-05	6.02011E-05
PINX1	113.027417	-0.135720188	0.032684539	-4.152427726	3.28967E-05	6.0137E-05
CD84	3182.945391	-0.224387124	0.054034757	-4.152644264	3.28655E-05	6.00857E-05
SLFN12L	67.7484209	0.255641105	0.061557912	4.152855377	3.28352E-05	6.00359E-05
NFKBIA	19411.3029	0.22427462	0.054003812	4.152940495	3.2823E-05	6.00191E-05
GOLGA6D	1.292837722	-0.659888546	0.158895054	-4.15298354	3.28168E-05	6.00134E-05
FRMD5	210.4575137	-0.276070209	0.066445795	-4.154818387	3.25546E-05	5.95395E-05
SNX32	328.1172944	-0.243711102	0.058646342	-4.155606208	3.24426E-05	5.93402E-05
SLC25A1	1173.45746	-0.21466328	0.051649106	-4.156185777	3.23605E-05	5.91955E-05
OMA1	408.3715687	-0.198930847	0.047861591	-4.156377681	3.23333E-05	5.91513E-05
MNS1	61.16079765	-0.267292486	0.064301798	-4.156843134	3.22675E-05	5.90365E-05
IRAK3	5240.434126	0.216911144	0.052165959	4.158097536	3.20909E-05	5.87188E-05
SLC12A7	1765.271032	-0.233192205	0.056073273	-4.158705101	3.20057E-05	5.85684E-05
ADAM12	29.35064278	-0.253999158	0.061070131	-4.159138921	3.1945E-05	5.84627E-05
PLXNA4	138.8441745	-0.39113364	0.094041285	-4.159169442	3.19407E-05	5.84604E-05
GPX1	11434.61542	-0.277659675	0.06675426	-4.15943006	3.19043E-05	5.83992E-05
TOP2B	7448.859552	-0.123739846	0.029745615	-4.159935745	3.18337E-05	5.82755E-05
SLC35G3	2.271618756	-0.555076818	0.133420646	-4.160351738	3.17758E-05	5.81749E-05
OR2A1	8.409341199	-0.428363876	0.10296116	-4.16044142	3.17633E-05	5.81575E-05
SNRNP200	13232.87862	-0.09358249	0.022492876	-4.160539134	3.17497E-05	5.8138E-05
TSN	1663.102963	0.110994068	0.026671468	4.161528206	3.16125E-05	5.78922E-05
FCF1	778.930992	-0.096031211	0.023069248	-4.162736869	3.14456E-05	5.75918E-05
BORCS8-MEF2B	3.041138337	-0.311756257	0.074891994	-4.16274476	3.14445E-05	5.75918E-05
DRD2	2.532606	-0.426695128	0.102493057	-4.163161301	3.13871E-05	5.74956E-05
SCARF1	923.6766714	-0.239375125	0.057478937	-4.164571192	3.11938E-05	5.71468E-05
SYT17	60.57356162	-0.458997479	0.110191871	-4.165438635	3.10755E-05	5.69353E-05
AGO2	5703.180941	0.175306038	0.042082961	4.16572493	3.10365E-05	5.68692E-05
SLC9A4	2.591031055	-0.539932957	0.129611675	-4.165774084	3.10298E-05	5.68623E-05
HCRTR2	0.829064639	-0.742288333	0.178144624	-4.166773706	3.08941E-05	5.66189E-05
KIFAP3	588.1125393	-0.191075249	0.045849035	-4.167486771	3.07976E-05	5.64474E-05
FPR2	130.9274585	0.443312146	0.106339537	4.168836527	3.06158E-05	5.61195E-05
CAPN13	5.960855125	-0.499001245	0.119697034	-4.168868918	3.06115E-05	5.61168E-05

KIAA1191	1388.597211	0.117139511	0.028097589	4.169023574	3.05907E-05	5.6084E-05
BMP8B	115.7174905	-0.283584087	0.068016289	-4.169355498	3.05462E-05	5.60076E-05
TPSB2	735.4142519	0.55728739	0.133662324	4.169367799	3.05446E-05	5.60076E-05
ABCA13	1769.711755	-0.463014687	0.111048093	-4.169496952	3.05273E-05	5.59833E-05
SOHLH1	0.729498286	-0.726079103	0.1741399	-4.169516022	3.05247E-05	5.59833E-05
GLRA3	1.064045106	-0.636212346	0.152549293	-4.170536183	3.03884E-05	5.57391E-05
TSC22D3	19151.95454	0.254774294	0.061087373	4.170653988	3.03727E-05	5.57155E-05
C9orf116	12.85300318	-0.228781578	0.054840485	-4.17176428	3.0225E-05	5.54498E-05
RPE	812.9964477	-0.181599959	0.043530584	-4.171778624	3.02231E-05	5.54498E-05
SLC38A1	6462.487837	0.247970226	0.059439703	4.171794523	3.0221E-05	5.54498E-05
RALGDS	835.7447194	0.203142221	0.048688605	4.172274407	3.01574E-05	5.53414E-05
ZNF654	1282.364755	0.171261501	0.04104572	4.172456938	3.01333E-05	5.53022E-05
NMUR1	228.4948941	-0.458617789	0.10989965	-4.173059592	3.00536E-05	5.51613E-05
GATA6	5.500815232	-0.52932555	0.126840065	-4.173173119	3.00387E-05	5.51389E-05
HSFX1	0.641906084	-0.481536383	0.115378475	-4.173537414	2.99906E-05	5.5056E-05
ZNF727	30.38444764	-0.627316273	0.150302035	-4.173704444	2.99686E-05	5.50208E-05
DCLK3	3.914146932	-0.537156955	0.12867452	-4.174540174	2.98589E-05	5.48243E-05
WIF1	1.929870639	-0.561715459	0.134535308	-4.175227051	2.97689E-05	5.46643E-05
STAT1	6416.726388	0.21285873	0.050980462	4.175300154	2.97593E-05	5.46519E-05
ERBB4	9.349450401	-0.683668199	0.163678908	-4.176886377	2.95527E-05	5.42774E-05
LEMD3	2281.016105	0.133011226	0.031841235	4.177326153	2.94956E-05	5.41777E-05
PTCD3	2915.706418	-0.124788737	0.02987263	-4.177360196	2.94912E-05	5.41747E-05
TEX55	3.212795801	-0.480455202	0.114936004	-4.180197562	2.91256E-05	5.35081E-05
RETREG1	102.9444574	0.354883927	0.084891918	4.180420642	2.9097E-05	5.34606E-05
SPON2	525.6130367	-0.273544842	0.065425632	-4.181004221	2.90224E-05	5.33286E-05
PIK3C3	1835.017164	-0.150339029	0.035947277	-4.182209082	2.8869E-05	5.30517E-05
UGT3A1	1.362007219	-0.564314625	0.134924898	-4.182435072	2.88403E-05	5.30039E-05
KANSL3	4053.139448	0.103852764	0.024817454	4.184666364	2.85585E-05	5.24909E-05
DDX49	1415.504393	-0.10011433	0.023917916	-4.185746368	2.84231E-05	5.22469E-05
FBXL22	63.56148194	-0.311556408	0.074423796	-4.18624723	2.83604E-05	5.21367E-05
ZNF579	397.9238026	0.195579114	0.046715841	4.186569435	2.83202E-05	5.20676E-05
ZNF236	1547.81073	-0.116621231	0.027849675	-4.187525745	2.82012E-05	5.18537E-05
RIIAD1	3.148828047	-0.464300017	0.11086149	-4.188109127	2.81288E-05	5.17254E-05
MARCHF8	4739.567081	-0.280420798	0.066953186	-4.188311462	2.81038E-05	5.16842E-05
TRAFD1	1268.121947	0.122375345	0.029216758	4.188532614	2.80764E-05	5.16387E-05
CKAP2L	327.5447391	-0.239755647	0.057224747	-4.189719654	2.79299E-05	5.13742E-05
TTYH3	4075.93363	0.180700479	0.043126989	4.189962818	2.79E-05	5.1324E-05

ZKSCAN2	428.4315691	0.143658146	0.034285433	4.190063699	2.78876E-05	5.1306E-05
GADD45B	4343.158293	0.275502271	0.065747962	4.190278489	2.78612E-05	5.12623E-05
MASP2	480.9582778	0.217951426	0.052006996	4.190809748	2.77961E-05	5.11472E-05
CYREN	699.1086909	0.155859766	0.037188753	4.19104581	2.77672E-05	5.10988E-05
ALKBH5	3429.504879	0.134369666	0.032054423	4.191922814	2.766E-05	5.09064E-05
FAM136A	861.541928	-0.127129075	0.030315975	-4.193468157	2.74722E-05	5.05654E-05
C8orf89	0.882488051	-0.542325991	0.129311866	-4.193938312	2.74153E-05	5.04654E-05
PDILT	0.881954464	-0.573520092	0.136742628	-4.194157294	2.73888E-05	5.04214E-05
CCDC54	0.532497493	-0.61049257	0.145510102	-4.195533919	2.7223E-05	5.01209E-05
ESM1	7.892088543	-0.583803725	0.139128371	-4.19615152	2.71489E-05	4.99892E-05
C1orf131	159.9465504	-0.178695971	0.042584734	-4.196244844	2.71377E-05	4.99733E-05
NR2C1	1286.598204	0.118133377	0.028150476	4.196496637	2.71075E-05	4.99225E-05
DBN1	1084.717655	-0.303993852	0.072433286	-4.19688062	2.70616E-05	4.98426E-05
U2SURP	4994.3906	0.119833098	0.028550504	4.197232278	2.70197E-05	4.977E-05
TTC6	1.797940844	-0.58240468	0.13874252	-4.197737511	2.69595E-05	4.96638E-05
ZC3HAV1	9518.374118	0.144579722	0.034434137	4.19873223	2.68413E-05	4.94508E-05
H3C2	2979.121927	-0.403884425	0.096139835	-4.201010192	2.65727E-05	4.89604E-05
NDUFA13	221.6294265	-0.179231423	0.042657547	-4.201634564	2.64995E-05	4.88302E-05
FBXO31	697.0513432	0.151397396	0.036031154	4.201846933	2.64746E-05	4.8789E-05
PAX9	1.94758455	-0.608936474	0.14490025	-4.202452893	2.64038E-05	4.86631E-05
RGSL1	7.311990731	-0.348014234	0.082797854	-4.203179402	2.63192E-05	4.85117E-05
URGCP	598.2575471	0.139443038	0.033172752	4.20354142	2.62771E-05	4.84387E-05
SCUBE2	27.41037089	-0.305020806	0.07254296	-4.20469202	2.61438E-05	4.81975E-05
PCYOX1	484.3652414	-0.161873086	0.038497787	-4.204737383	2.61385E-05	4.81923E-05
CWC15	1106.360132	0.187013881	0.044467873	4.205595367	2.60396E-05	4.80144E-05
CCPG1	584.9015258	0.164755039	0.039173764	4.205749471	2.60218E-05	4.79862E-05
RNPC3	1078.498433	-0.157438479	0.03740833	-4.208647677	2.56904E-05	4.73794E-05
ASPHD1	17.6057353	-0.224888836	0.053425353	-4.209402903	2.56046E-05	4.72258E-05
PBOV1	1.844955418	-0.425738302	0.101128405	-4.209878542	2.55508E-05	4.71309E-05
GLG1	3934.751896	-0.148219358	0.035202639	-4.210461512	2.5485E-05	4.70139E-05
HOXB4	371.9005054	-0.560157325	0.133028538	-4.210805693	2.54462E-05	4.69467E-05
LRRC71	12.56336836	-0.40985999	0.097332371	-4.210931931	2.54319E-05	4.69249E-05
ZNF827	1211.117614	-0.313894633	0.074538688	-4.211163914	2.54058E-05	4.68811E-05
GBA	982.2977336	-0.180141988	0.04277477	-4.211407494	2.53784E-05	4.6835E-05
USP50	168.1476912	0.181130736	0.042988438	4.21347565	2.51471E-05	4.64124E-05
TATDN3	360.7811981	0.134535047	0.031924853	4.214116424	2.50758E-05	4.62852E-05
TMEM31	0.835745007	-0.506184314	0.120084812	-4.215223442	2.49531E-05	4.60631E-05

RNASE4	2.708548944	0.586928166	0.139209625	4.216146448	2.48512E-05	4.58794E-05
CYC1	2164.296831	-0.17275144	0.040970505	-4.21648309	2.48142E-05	4.58153E-05
OGDHL	17.59174947	0.495097302	0.117409131	4.216855172	2.47733E-05	4.57441E-05
TAPT1	4347.697815	0.195260842	0.04630183	4.21713013	2.47431E-05	4.56927E-05
SOX30	7.593420351	-0.369232419	0.087546498	-4.217557826	2.46962E-05	4.56105E-05
CLTRN	3.114875423	-0.308143873	0.073057238	-4.217841828	2.46652E-05	4.55574E-05
SLC35F3	9.010218219	-0.491609654	0.116549401	-4.218036732	2.46439E-05	4.55223E-05
PSORS1C2	0.616358747	-0.747423559	0.177186162	-4.218295324	2.46156E-05	4.54744E-05
AVPR1A	14.03458899	-0.499830701	0.118471484	-4.218995878	2.45393E-05	4.53377E-05
POC1B-GALNT4	12.89730219	-0.198134004	0.046950786	-4.220035949	2.44263E-05	4.51333E-05
CHRDL2	8.552620062	-0.370360463	0.087755227	-4.220380674	2.4389E-05	4.50686E-05
REV3L	2159.88051	-0.162730766	0.038549733	-4.221320162	2.42876E-05	4.48853E-05
PGBD1	189.8193588	-0.17394134	0.041195699	-4.222317993	2.41803E-05	4.46912E-05
HECW2	62.71160182	-0.272452072	0.064524402	-4.22246569	2.41644E-05	4.46662E-05
MOG	8.647554651	-0.587209508	0.139066378	-4.222512429	2.41594E-05	4.46611E-05
PRKAA1	1770.484109	0.124935589	0.029587339	4.222603091	2.41497E-05	4.46474E-05
POLR2A	16099.67139	0.141396271	0.033481688	4.223092667	2.40973E-05	4.45547E-05
ERVW-1	112.1497197	-0.287141359	0.067988833	-4.223360633	2.40686E-05	4.45059E-05
RTP1	0.653808688	-0.848350425	0.200853516	-4.223727026	2.40295E-05	4.44378E-05
DHRS2	8.325359769	-0.419530757	0.099325648	-4.223790799	2.40227E-05	4.44294E-05
SCAF11	7397.973019	0.120103606	0.02843476	4.22383049	2.40185E-05	4.44258E-05
CXCL12	555.4459834	-0.559644924	0.132489498	-4.224070069	2.39929E-05	4.43827E-05
SESN1	2124.921312	0.207972488	0.049232537	4.224289461	2.39696E-05	4.43437E-05
OOEP	4.808570915	-0.343527619	0.081316718	-4.224563233	2.39405E-05	4.4294E-05
RAP1GDS1	988.1904634	-0.131123113	0.031034672	-4.225052343	2.38885E-05	4.42021E-05
LDOC1	15.09739875	-0.469830028	0.111183634	-4.225712114	2.38186E-05	4.4077E-05
SNX7	5.417906875	-0.666624762	0.157743435	-4.22600638	2.37875E-05	4.40236E-05
CLIC1	13592.86538	0.175651904	0.041557233	4.226746856	2.37094E-05	4.38832E-05
TBC1D2	1011.309836	0.230835078	0.054607885	4.227138196	2.36682E-05	4.38111E-05
TMED8	2538.175775	0.134618513	0.031838899	4.228114584	2.35658E-05	4.36256E-05
ARHGEF18	1361.882522	-0.154904134	0.036633765	-4.228452444	2.35304E-05	4.35642E-05
GOLM2	1726.515206	0.093776719	0.022176751	4.228604982	2.35145E-05	4.35388E-05
MPZL1	1215.11085	-0.204176332	0.048282899	-4.22875045	2.34993E-05	4.35148E-05
TBL1Y	25.48503631	-1.003646713	0.237312246	-4.229224277	2.34498E-05	4.34274E-05
GDI2	6464.015646	0.128745276	0.030440728	4.229375702	2.34341E-05	4.34023E-05
SF1	19411.83045	0.125394949	0.029641798	4.230342135	2.33336E-05	4.32203E-05
MUC17	3.933222136	-0.44816661	0.105920997	-4.231140409	2.32509E-05	4.30712E-05

NFIC	3061.703895	0.1768453	0.041793057	4.231451667	2.32188E-05	4.30157E-05
OPN3	70.10229118	-0.249534228	0.0589679	-4.231696036	2.31936E-05	4.29731E-05
RHEX	388.1047749	-0.412615308	0.097495131	-4.232163235	2.31454E-05	4.2888E-05
DHRS9	294.5483324	-0.390403551	0.092240465	-4.232454276	2.31155E-05	4.28365E-05
EGFL6	2.483035777	-0.48356932	0.114245127	-4.232734746	2.30867E-05	4.27872E-05
IL12A	47.41542989	-0.301671534	0.07127034	-4.232778088	2.30822E-05	4.2783E-05
MIA	3.78850049	-0.400387407	0.094575771	-4.2335093	2.30073E-05	4.26481E-05
ZWILCH	359.515607	-0.11052876	0.02610384	-4.234195457	2.29371E-05	4.25221E-05
TRIB1	7610.621641	0.264919458	0.062564087	4.234369416	2.29194E-05	4.24933E-05
HSD17B13	103.9881689	0.231794656	0.0547344	4.234899028	2.28654E-05	4.23972E-05
OR6K6	1.828663022	-0.6708242	0.158392125	-4.235211829	2.28336E-05	4.23423E-05
ARFGEF3	21.60978896	-0.394221867	0.093066791	-4.235902655	2.27635E-05	4.22163E-05
GAB1	1071.941984	-0.213919646	0.050501175	-4.235934018	2.27604E-05	4.22144E-05
AIMP1	1735.448853	0.109108527	0.025757267	4.236028903	2.27508E-05	4.22006E-05
SOCS4	1171.737096	0.124529222	0.029397247	4.236084472	2.27451E-05	4.21941E-05
CMTM8	30.0781412	0.349691816	0.082544416	4.236407917	2.27124E-05	4.21374E-05
MYBPC3	112.1001821	-0.238078013	0.05619462	-4.236669182	2.2686E-05	4.20924E-05
SDE2	4104.426874	0.161186978	0.038045358	4.236705526	2.26823E-05	4.20896E-05
HARS1	1310.591276	-0.079881559	0.018849657	-4.237825705	2.25695E-05	4.18842E-05
HHLA1	1.285007307	-0.620009352	0.146284479	-4.238380965	2.25137E-05	4.17847E-05
TNP2	1.815739259	-0.506714531	0.119541368	-4.238821579	2.24696E-05	4.17068E-05
GDF5	6.052630213	-0.306378452	0.07226747	-4.239507107	2.24011E-05	4.15835E-05
TIPRL	749.9084475	-0.146571063	0.034569264	-4.239924348	2.23595E-05	4.15103E-05
FCMR	438.2012254	0.269303252	0.063512633	4.240152531	2.23368E-05	4.1472E-05
RC3H2	3237.966207	0.135339551	0.031904294	4.242048205	2.21489E-05	4.11271E-05
KNDC1	16.38893744	-0.43772378	0.103182644	-4.242222938	2.21317E-05	4.1099E-05
BTBD9	1341.045676	0.147402354	0.034745921	4.24229229	2.21248E-05	4.10902E-05
CAPN11	136.7880174	-0.303650333	0.071569	-4.24276338	2.20784E-05	4.10079E-05
RPS27AP5	82.31977682	-0.486440206	0.114623895	-4.243794059	2.19772E-05	4.08237E-05
TBC1D26	1.012871275	-0.534923048	0.126025678	-4.244555991	2.19027E-05	4.06892E-05
MORN2	71.06661561	-0.285760688	0.06731852	-4.244904474	2.18687E-05	4.06298E-05
BOD1L2	1.587103143	-0.719352314	0.16944716	-4.245289874	2.18311E-05	4.05639E-05
ZHX1	1191.244387	0.122434297	0.028835748	4.245920635	2.17698E-05	4.04538E-05
SNORC	69.78907608	-0.261119831	0.061494982	-4.24619736	2.17429E-05	4.04077E-05
HP1BP3	6601.674956	-0.072402827	0.017050707	-4.246323943	2.17306E-05	4.03887E-05
WDR47	843.5506854	0.140710814	0.033134627	4.246639468	2.17001E-05	4.03358E-05
RAB39A	22.16904548	0.425738268	0.100237554	4.247293059	2.16369E-05	4.02221E-05

AC091167.6	0.689328437	-0.519659126	0.12234374	-4.247533421	2.16137E-05	4.01828E-05
DDX59	673.5306586	0.09419802	0.022164687	4.249914336	2.13852E-05	3.97619E-05
PI16	16.84222595	-0.353679804	0.083220386	-4.249917865	2.13849E-05	3.97619E-05
SETBP1	334.2577978	-0.351402862	0.082678567	-4.250229221	2.13552E-05	3.97135E-05
VCAM1	112.8730224	-0.471839894	0.110975579	-4.25174527	2.12111E-05	3.94493E-05
PRKAR2B	1464.408589	-0.335677323	0.078942371	-4.252181934	2.11698E-05	3.93762E-05
MAPK11	222.7413636	-0.2699459	0.063481005	-4.252388597	2.11502E-05	3.93436E-05
OR52K2	13.65635405	-0.533920699	0.125547275	-4.252746205	2.11165E-05	3.92846E-05
CCL17	1.770280345	-0.406684929	0.095620774	-4.253102243	2.10829E-05	3.92259E-05
NCK2	858.184173	0.169921196	0.039942928	4.254099616	2.09892E-05	3.90552E-05
FAM53A	72.56536262	-0.243022381	0.057123223	-4.254353477	2.09654E-05	3.90146E-05
GRIN3A	80.0557883	-0.287115337	0.067476268	-4.255056582	2.08996E-05	3.88959E-05
SP140	478.2297308	0.262892428	0.0617822	4.25514836	2.0891E-05	3.88836E-05
GPR75	117.0180355	-0.234218993	0.055035971	-4.25574382	2.08355E-05	3.8784E-05
OS9	4414.698763	0.129174051	0.030346178	4.256682705	2.07482E-05	3.86252E-05
GAST	3.750808333	-0.709751314	0.166722923	-4.257070953	2.07123E-05	3.85619E-05
MAGI3	155.0141892	-0.331774126	0.07793467	-4.257080012	2.07114E-05	3.85619E-05
RAMP1	116.6504746	-0.493978875	0.116022501	-4.257612708	2.06621E-05	3.84759E-05
WEE2	7.138349233	-0.302693391	0.07109351	-4.257679658	2.0656E-05	3.8468E-05
BCL2L15	157.7080439	-0.298481782	0.070094683	-4.25826566	2.06019E-05	3.8371E-05
RADX	17.17344559	0.341448957	0.080181799	4.258434741	2.05863E-05	3.83457E-05
TRAM1L1	1.743708901	-0.782890482	0.183806726	-4.25931358	2.05056E-05	3.81989E-05
ZNF569	173.4812386	-0.151649772	0.035603787	-4.259371927	2.05002E-05	3.81925E-05
SLC22A13	24.68517699	-0.30252118	0.071010054	-4.260258438	2.04191E-05	3.8045E-05
CPQ	746.9472379	-0.223314855	0.05241438	-4.260564654	2.03911E-05	3.79965E-05
REEP3	784.7080185	-0.314595234	0.073835245	-4.260773204	2.03721E-05	3.79647E-05
MSL2	4794.393122	0.133725339	0.031375083	4.262150974	2.02469E-05	3.77349E-05
MEI4	4.561152461	-0.671324983	0.157498381	-4.262424661	2.02221E-05	3.76923E-05
EPHA8	6.707333739	-0.46194758	0.10836942	-4.262711549	2.01961E-05	3.76475E-05
PRRX1	4.349655293	-0.54929499	0.128850486	-4.263041675	2.01663E-05	3.75955E-05
PLD5	2.233117116	-0.517437946	0.121358584	-4.26371114	2.01059E-05	3.74865E-05
ELP1	1989.394395	-0.133619475	0.031331055	-4.264761503	2.00116E-05	3.73142E-05
AVIL	148.9258061	-0.227962055	0.053434315	-4.266210847	1.98821E-05	3.70762E-05
AC139491.7	4.139778598	0.414543974	0.097148962	4.267096259	1.98034E-05	3.6933E-05
ZNHIT1	1367.673417	-0.176372114	0.041320912	-4.268349952	1.96924E-05	3.67295E-05
FKRP	396.6297259	-0.113032142	0.026475685	-4.269281056	1.96104E-05	3.658E-05
JPH3	37.38972534	-0.298570524	0.069928576	-4.26964969	1.9578E-05	3.65231E-05

SYT8	2.669168338	-0.460107894	0.107761445	-4.269689332	1.95745E-05	3.65201E-05
HEXIM1	2349.839406	-0.200616084	0.046976981	-4.270518859	1.95019E-05	3.6388E-05
CETP	174.1183343	-0.372673578	0.087231292	-4.27224648	1.93514E-05	3.61106E-05
IGSF10	1089.38132	-0.418465307	0.097936077	-4.272841237	1.92998E-05	3.60178E-05
IER5	4229.661642	0.21590285	0.050526986	4.273020589	1.92843E-05	3.59923E-05
MAP3K5	1928.430947	-0.18300847	0.042827746	-4.27312865	1.92749E-05	3.59783E-05
MAP4K4	6677.16087	0.138703369	0.032453146	4.273957554	1.92034E-05	3.58481E-05
PFKFB4	565.1823008	0.251785125	0.058908241	4.274191911	1.91832E-05	3.58138E-05
SLFN13	744.0973603	-0.258388866	0.060448502	-4.274528862	1.91542E-05	3.57631E-05
ERAP1	3031.659785	0.118823232	0.027795719	4.27487528	1.91244E-05	3.5711E-05
MRPL3	1002.49073	-0.140869749	0.032951596	-4.275050868	1.91094E-05	3.56863E-05
SMPD1	490.5155917	0.171007778	0.039999077	4.275293052	1.90886E-05	3.56509E-05
EDN3	1.575703929	0.634684828	0.148414736	4.276427304	1.89917E-05	3.54732E-05
GCNT3	12.02338773	-0.286244674	0.066925267	-4.277079279	1.89361E-05	3.53729E-05
VSIG8	1.578618394	-0.463931443	0.108429998	-4.278626319	1.8805E-05	3.51313E-05
C19orf71	136.8886704	-0.196739584	0.045962392	-4.280447045	1.86518E-05	3.48484E-05
IQCA1L	13.20711404	-0.411730894	0.096150568	-4.28214729	1.85098E-05	3.45865E-05
USP45	345.5724396	-0.222632875	0.051977219	-4.283277941	1.8416E-05	3.44144E-05
TAF5L	1119.797641	0.137157138	0.03201821	4.28372291	1.83792E-05	3.43489E-05
KPNA7	2.752631964	-0.422882475	0.098708797	-4.284141715	1.83446E-05	3.42875E-05
SV2A	1065.336679	-0.296916672	0.069304109	-4.284257805	1.8335E-05	3.42729E-05
RAB11FIP5	213.131402	0.425053828	0.099207709	4.284483855	1.83164E-05	3.42413E-05
ADGRE5	12377.20733	0.1926884	0.044971704	4.284658583	1.8302E-05	3.42177E-05
LBX1	0.668115218	-0.651614183	0.152056601	-4.285339666	1.8246E-05	3.41163E-05
HMG20A	653.1296619	-0.110308271	0.025739404	-4.285579833	1.82263E-05	3.40827E-05
PLA2G4C	75.45681519	-0.304044895	0.070943241	-4.285748587	1.82125E-05	3.40601E-05
NFKBIE	1915.360141	0.197572222	0.046098418	4.28587857	1.82018E-05	3.40434E-05
TAF4	736.0447873	-0.123959362	0.028922698	-4.285885213	1.82013E-05	3.40434E-05
POLDIP2	1521.590068	-0.126130787	0.029426309	-4.286327143	1.81651E-05	3.39813E-05
FAM161B	160.145087	-0.12705822	0.029641	-4.286569999	1.81453E-05	3.39474E-05
DEUP1	2.918680467	-0.484172096	0.112933638	-4.287226598	1.80918E-05	3.38505E-05
ACTL7A	8.596613098	-0.337731402	0.078773722	-4.287361243	1.80808E-05	3.38332E-05
RIPPLY1	0.394439826	-0.868051162	0.202454818	-4.287629068	1.8059E-05	3.37957E-05
DNAJC4	855.9561411	-0.234597076	0.054714509	-4.287657537	1.80567E-05	3.37946E-05
MYL12B	4102.155099	0.173023453	0.040351312	4.287926322	1.80349E-05	3.3757E-05
KLRC4	10.10491842	0.423261798	0.098699569	4.288385467	1.79977E-05	3.36905E-05
NFE2L1	2648.286411	0.116557985	0.02717675	4.288886143	1.79571E-05	3.36179E-05

YIPF2	526.1657376	-0.136313351	0.031779673	-4.289325165	1.79217E-05	3.35547E-05
GNGT1	0.785631183	-0.756754245	0.176427328	-4.289325548	1.79217E-05	3.35547E-05
PROM1	2404.871253	-0.461710222	0.107637993	-4.289472617	1.79098E-05	3.35389E-05
PPIH	768.5683441	-0.108409867	0.025272276	-4.289675598	1.78934E-05	3.35114E-05
TLX1	1.105302466	-0.614972554	0.143353142	-4.28991332	1.78743E-05	3.34788E-05
ZDHHC24	1164.789577	-0.138760864	0.03233561	-4.291270927	1.77653E-05	3.32779E-05
SAXO1	11.92309184	-0.287718501	0.067041762	-4.291630988	1.77366E-05	3.32272E-05
SH2D3C	1140.957762	-0.226552272	0.052786468	-4.291862708	1.7718E-05	3.31957E-05
MYO1E	253.0038066	-0.29662512	0.069093136	-4.293119933	1.7618E-05	3.30114E-05
WHRN	485.6636829	0.299284218	0.069710668	4.293234094	1.76089E-05	3.29975E-05
B9D2	369.840185	-0.156452785	0.036433235	-4.294232618	1.75299E-05	3.28525E-05
YIPF6	837.1408579	-0.149187114	0.034741166	-4.294246009	1.75288E-05	3.28525E-05
FYB2	0.69533552	-1.144673541	0.266533029	-4.294678016	1.74947E-05	3.27929E-05
MED13	8639.655844	0.163149857	0.037985635	4.295040928	1.74661E-05	3.27425E-05
RCOR3	2865.669183	0.124961755	0.029093943	4.295112382	1.74605E-05	3.27351E-05
KLHL36	1760.289632	0.111877235	0.026043286	4.295818748	1.7405E-05	3.26341E-05
H3-5	79.59628148	0.382652154	0.08907452	4.295865474	1.74013E-05	3.26304E-05
RTRAF	3148.121623	0.161556218	0.03759679	4.297074791	1.73067E-05	3.2456E-05
SHISA6	0.852213618	-0.634172505	0.147580967	-4.297115793	1.73035E-05	3.24531E-05
E2F4	2144.383561	-0.145896761	0.033951317	-4.297234258	1.72942E-05	3.24389E-05
LRRC19	45.62807245	-0.227012186	0.052814941	-4.298256903	1.72147E-05	3.22927E-05
PTK7	1111.543791	-0.30344443	0.070595459	-4.298356216	1.72069E-05	3.22814E-05
RSKR	568.9353039	-0.155409481	0.03614583	-4.299513401	1.71174E-05	3.21164E-05
ACACB	852.0288249	-0.259723455	0.060407638	-4.299513498	1.71173E-05	3.21164E-05
DDAH1	50.82454586	-0.381091675	0.088625352	-4.300030044	1.70775E-05	3.20477E-05
WDR81	2187.553122	-0.185058245	0.043032636	-4.300416201	1.70478E-05	3.1995E-05
LGI2	11.67964869	-0.414378974	0.096342174	-4.301117103	1.69939E-05	3.1897E-05
EWSR1	15103.5175	0.101584302	0.023617674	4.301198414	1.69877E-05	3.18884E-05
AKT1S1	862.2406923	-0.140959767	0.032768839	-4.301640607	1.69538E-05	3.18278E-05
RAD21L1	3.397396068	-0.577686534	0.134292613	-4.30170001	1.69493E-05	3.18224E-05
RELL2	109.8447539	-0.271788103	0.063180351	-4.301782119	1.6943E-05	3.18136E-05
ZNF487	282.7210888	0.169483541	0.039392843	4.302394227	1.68962E-05	3.17289E-05
FXYD7	23.74498832	0.262571948	0.061023937	4.302769742	1.68676E-05	3.16782E-05
CKMT1B	1.393156967	-0.515400585	0.11976021	-4.303604538	1.68042E-05	3.1562E-05
CERS5	1600.428184	0.088593574	0.020581602	4.30450332	1.67361E-05	3.14372E-05
NKIRAS2	1714.92753	0.129214976	0.030018044	4.304576843	1.67305E-05	3.14297E-05
FRMD1	0.767364501	-0.686004763	0.159350092	-4.305016421	1.66973E-05	3.13704E-05

CWC25	2614.70918	0.14552777	0.033793317	4.306406842	1.65928E-05	3.1177E-05
ADORA2B	154.7712804	0.23097983	0.053629262	4.306973911	1.65503E-05	3.11002E-05
CNTNAP4	0.678476232	-1.287585739	0.298922754	-4.307419624	1.6517E-05	3.10406E-05
RPS17	385.6382707	-0.133450987	0.030973778	-4.30851502	1.64354E-05	3.08902E-05
FA2H	7.578059309	-0.40222592	0.093352817	-4.30866399	1.64244E-05	3.08724E-05
NUF2	228.832271	-0.228792003	0.05309931	-4.308756621	1.64175E-05	3.08624E-05
KIAA2013	1148.044761	-0.175685851	0.040769991	-4.309195281	1.6385E-05	3.08042E-05
BSPRY	392.159195	-0.261370318	0.060650275	-4.309466287	1.63649E-05	3.07695E-05
ZNF134	1185.966629	0.165223387	0.038337296	4.309729803	1.63454E-05	3.07358E-05
DGKA	2531.101255	0.167015874	0.038746071	4.310524099	1.62868E-05	3.06285E-05
SLTM	3982.196327	0.112801719	0.026161741	4.311705327	1.62E-05	3.04682E-05
EBP	399.0601292	-0.187456819	0.043465877	-4.312735231	1.61247E-05	3.03295E-05
GPR176	17.27954769	-0.378157797	0.087670267	-4.313409898	1.60756E-05	3.024E-05
HOXB13	0.658247887	-0.632191009	0.146541131	-4.314085767	1.60265E-05	3.01505E-05
RPL28	40635.99455	-0.332477485	0.077049726	-4.315102765	1.59529E-05	3.00149E-05
ARAF	2754.441355	0.108553789	0.025152854	4.315764293	1.59052E-05	2.99281E-05
NOMO3	330.0761714	0.193888416	0.044916187	4.316671286	1.584E-05	2.98083E-05
ZFC3H1	6786.653319	0.171389928	0.039702583	4.316845754	1.58275E-05	2.97876E-05
TTLL13P	10.17838952	-0.226154225	0.052385617	-4.317105339	1.58089E-05	2.97555E-05
APLF	280.7674071	-0.205833156	0.047678473	-4.317108785	1.58086E-05	2.97555E-05
PDCD7	708.6715678	-0.119294651	0.027632648	-4.317163273	1.58047E-05	2.97534E-05
RAMP3	9.156855886	-0.552378995	0.127915047	-4.318326959	1.57216E-05	2.95998E-05
GAR1	349.9662716	-0.197442702	0.045717446	-4.318760515	1.56908E-05	2.95446E-05
FRY	3885.621215	-0.258406801	0.059829779	-4.31903321	1.56714E-05	2.95109E-05
ZMIZ2	2613.540508	-0.126706902	0.029334074	-4.319444475	1.56422E-05	2.94588E-05
BEST2	11.8922531	-0.323802276	0.07494523	-4.320518835	1.55663E-05	2.93186E-05
PENK	31.3761127	-0.902853878	0.208936711	-4.321183547	1.55195E-05	2.92332E-05
EI24	1029.129574	-0.15607142	0.03611129	-4.321956407	1.54652E-05	2.91338E-05
RHOH	2681.691767	0.259027902	0.059910776	4.323561108	1.53531E-05	2.89253E-05
COL4A4	58.32893308	-0.300921366	0.069599281	-4.323627489	1.53484E-05	2.89194E-05
TMEM59L	3.452769835	-0.406117547	0.093915125	-4.32430398	1.53014E-05	2.88336E-05
TAS1R1	102.5756373	0.339931291	0.078608399	4.324363495	1.52973E-05	2.88286E-05
CDKN2C	1195.787804	-0.280709706	0.064906509	-4.324831375	1.52649E-05	2.87703E-05
NDUFS7	255.7264138	-0.18742814	0.043334739	-4.325124469	1.52446E-05	2.87348E-05
BHLHE41	92.19899431	0.442104823	0.102208875	4.325503277	1.52184E-05	2.86882E-05
TKT	22094.27659	0.190657952	0.044076909	4.325574455	1.52135E-05	2.86817E-05
ALDH3A1	9.081444897	-0.401583029	0.092825251	-4.326226142	1.51686E-05	2.85998E-05

AK8	19.21852517	-0.312903391	0.072324687	-4.326370494	1.51586E-05	2.85838E-05
EIF4EBP3	42.24374587	-0.197742386	0.045697556	-4.327198332	1.51018E-05	2.84794E-05
NBPF20	75.12974866	-0.203785711	0.047089593	-4.327616726	1.50731E-05	2.84281E-05
ASNS	34.31108222	-0.271117851	0.062628994	-4.328951078	1.49821E-05	2.82591E-05
FAM81B	67.21615377	-0.274708949	0.06345157	-4.32942714	1.49498E-05	2.82008E-05
TMC4	164.3452976	-0.219085692	0.050599037	-4.329839154	1.49218E-05	2.81508E-05
TBC1D3B	4.317612557	-0.353230744	0.081569188	-4.330443322	1.48809E-05	2.80764E-05
FAM205A	6.182129396	-0.516945577	0.119370366	-4.330602262	1.48702E-05	2.80589E-05
UGGT2	202.6866515	-0.513187029	0.118494888	-4.330879057	1.48515E-05	2.80263E-05
FAM174B	114.2164867	-0.255014415	0.058875693	-4.331404066	1.48161E-05	2.79623E-05
EML5	28.14164637	-0.344034271	0.079418549	-4.331913357	1.47819E-05	2.79003E-05
GSTK1	4490.860533	-0.214809736	0.04958742	-4.331940118	1.47801E-05	2.78996E-05
BTBD6	535.6233412	-0.137247994	0.03168143	-4.332127448	1.47675E-05	2.78786E-05
AL591806.2	10.91682507	-0.235078959	0.0542558	-4.332789461	1.47232E-05	2.77975E-05
TNFRSF1A	2851.829048	0.161904374	0.037360045	4.333623648	1.46675E-05	2.7695E-05
PCK2	502.6674284	-0.177916667	0.041051409	-4.333996609	1.46426E-05	2.76508E-05
LMNB1	2078.152914	-0.17564815	0.040527614	-4.334036273	1.464E-05	2.76485E-05
MTX3	363.2179684	0.318882347	0.0735635	4.334790315	1.45899E-05	2.75565E-05
DUSP18	173.4433431	-0.194333511	0.044825715	-4.335313143	1.45553E-05	2.74938E-05
OR13C3	0.377161385	-0.767996277	0.177114101	-4.336166765	1.44989E-05	2.73899E-05
TMEM61	0.447325542	-0.738579542	0.170304743	-4.336811346	1.44565E-05	2.73124E-05
HMOX2	518.4348929	-0.153827541	0.035470129	-4.336819293	1.44559E-05	2.73124E-05
AMZ2	794.2003023	0.128193638	0.029559146	4.336851925	1.44538E-05	2.73124E-05
PPP6R3	5543.145196	0.125656446	0.028972645	4.337071889	1.44393E-05	2.72879E-05
SLC45A4	725.0321112	-0.246773561	0.056889416	-4.337776279	1.43932E-05	2.72033E-05
PSG8	0.687860349	-0.790006044	0.182106201	-4.338161126	1.4368E-05	2.71583E-05
ATRNL1	8.056982034	-0.667830267	0.153905007	-4.339236777	1.42978E-05	2.70284E-05
HSD11B2	60.99790088	0.315466332	0.072696319	4.339509026	1.42801E-05	2.69975E-05
ADM5	110.7814638	-0.278797915	0.064235948	-4.340216398	1.42342E-05	2.69133E-05
YY1AP1	4658.435258	0.133257923	0.03070181	4.340393116	1.42228E-05	2.68943E-05
AMPH	43.19498058	-0.379589954	0.087437148	-4.341289272	1.41649E-05	2.67874E-05
INO80	3485.955782	0.142659534	0.032860907	4.34131456	1.41633E-05	2.67869E-05
NPR3	2089.301357	-0.436846622	0.100579133	-4.343312677	1.4035E-05	2.65469E-05
FOXD3	0.700666019	-0.701677617	0.161546489	-4.343502749	1.40229E-05	2.65265E-05
RWDD1	940.8048556	0.189706816	0.043672735	4.34382729	1.40022E-05	2.64899E-05
PRRC1	1863.26548	0.118333777	0.027241427	4.343890472	1.39981E-05	2.64848E-05
TOPBP1	3392.513161	0.114672314	0.02639635	4.344248891	1.39753E-05	2.64442E-05

TBC1D13	1438.039885	-0.11361902	0.026152553	-4.344471521	1.39611E-05	2.64199E-05
RAB40C	700.2636083	-0.155435633	0.035768428	-4.345609817	1.38889E-05	2.62858E-05
METTL16	1422.39647	0.102796239	0.023655041	4.34563775	1.38872E-05	2.6285E-05
TBC1D5	3939.575713	-0.162197601	0.037312992	-4.346947114	1.38045E-05	2.61312E-05
PTGIR	267.0169945	0.354159124	0.081472506	4.346977188	1.38027E-05	2.61301E-05
TMEM230	1414.215011	-0.182102511	0.041891159	-4.347039239	1.37988E-05	2.61253E-05
NIPBL	8699.792945	0.155349031	0.035732244	4.347586813	1.37644E-05	2.60627E-05
CEACAM7	0.621639463	-0.709450162	0.16315735	-4.348257443	1.37223E-05	2.59856E-05
MCC	230.0941588	0.26352306	0.060597648	4.348734127	1.36926E-05	2.59317E-05
TCEAL5	1.417296368	-0.742393421	0.170711943	-4.348807743	1.3688E-05	2.59255E-05
TUBGCP5	751.9593241	-0.14451777	0.033215825	-4.350870957	1.35598E-05	2.56852E-05
SPI1	7151.14959	0.244237434	0.056133501	4.351010143	1.35512E-05	2.56714E-05
RBMXL3	0.509514143	-1.148909204	0.264052097	-4.35107018	1.35475E-05	2.56669E-05
TMEM237	180.8390299	-0.17260311	0.039667916	-4.351201838	1.35393E-05	2.5654E-05
MRPL21	749.3770622	-0.19850488	0.045620255	-4.351244378	1.35367E-05	2.56515E-05
EED	668.1321313	0.151500184	0.034812464	4.351894886	1.34966E-05	2.55779E-05
AMER3	1.536075652	-0.487213318	0.111906888	-4.35373841	1.33835E-05	2.53661E-05
POLE4	327.02111	-0.280899443	0.064516319	-4.353928563	1.33719E-05	2.53466E-05
SPATA18	12.51023182	-0.545524601	0.125292622	-4.354004171	1.33673E-05	2.53403E-05
UBTD1	222.9480003	0.251501706	0.057761393	4.354148922	1.33585E-05	2.5326E-05
LRRC3	107.7137121	-0.230703992	0.052961623	-4.356059711	1.32425E-05	2.51085E-05
AAGAB	1168.178376	0.110434848	0.025345644	4.357152938	1.31765E-05	2.49859E-05
NOL4L	2088.489305	-0.206711633	0.047434639	-4.357820295	1.31364E-05	2.49122E-05
RNF225	5.377622361	-0.410532433	0.094204043	-4.357906732	1.31312E-05	2.49048E-05
SURF1	702.6463853	-0.157715142	0.036188795	-4.358120879	1.31184E-05	2.48829E-05
ZIC3	0.676417791	-0.847753945	0.194485327	-4.358960958	1.30681E-05	2.479E-05
NGDN	1176.79145	0.143582609	0.032934882	4.359590758	1.30306E-05	2.47211E-05
NPIPB4	670.9761342	-0.161835793	0.037118393	-4.359989186	1.30069E-05	2.46785E-05
MED10	623.8447872	0.178124165	0.040852886	4.360136604	1.29981E-05	2.46643E-05
BMP8A	134.6860929	-0.247890811	0.056842519	-4.361010328	1.29463E-05	2.45684E-05
HYAL3	573.3625902	-0.250171299	0.057356438	-4.361695168	1.29059E-05	2.4494E-05
ZYX	12543.51549	0.257549748	0.059048026	4.361699506	1.29056E-05	2.4494E-05
FGFRL1	981.9749817	0.304205658	0.069714991	4.363561636	1.27962E-05	2.42905E-05
SCO1	1125.205031	-0.112247469	0.025714982	-4.365061219	1.27087E-05	2.41268E-05
LPA	0.716085097	-0.728236729	0.166805304	-4.365788803	1.26665E-05	2.4049E-05
DCUN1D4	691.5758119	-0.158316316	0.036258532	-4.366318957	1.26358E-05	2.39931E-05
DHX58	429.3532919	-0.182536948	0.041804433	-4.366449525	1.26282E-05	2.3981E-05

UNC79	106.3568316	-0.418075284	0.095734266	-4.367039098	1.25942E-05	2.39188E-05
ICE1	2364.963548	0.137171769	0.031398345	4.368757902	1.24955E-05	2.37336E-05
AURKAIP1	842.9590037	-0.20157848	0.046140707	-4.368777427	1.24944E-05	2.37336E-05
MRPL33	1078.764932	-0.375864666	0.086027418	-4.369126413	1.24745E-05	2.36982E-05
PNMA6A	16.33166622	-0.38102312	0.087202605	-4.369400658	1.24588E-05	2.36708E-05
NFKBIZ	11762.02301	0.208963004	0.047820399	4.369746165	1.24391E-05	2.36356E-05
ZUP1	579.5305754	0.168395855	0.038533852	4.370075881	1.24203E-05	2.36023E-05
CCNB2	661.1911219	-0.220197242	0.050385776	-4.370226279	1.24118E-05	2.35883E-05
TAOK1	3991.300976	-0.164404068	0.03761326	-4.370907223	1.23731E-05	2.35171E-05
ETS2	7081.981895	0.226982965	0.051917838	4.371964887	1.23133E-05	2.34057E-05
AKAP14	23.01722814	0.322655592	0.07379782	4.372156116	1.23026E-05	2.33875E-05
TNRC6C	794.5283351	-0.207687497	0.04749638	-4.372701639	1.22718E-05	2.33314E-05
TSPYL1	3406.034248	0.132608846	0.030325658	4.372826647	1.22648E-05	2.33203E-05
NPEPPS	3050.74772	0.129456289	0.029602893	4.373095882	1.22497E-05	2.32938E-05
USP42	2261.337779	0.174213056	0.039835457	4.373316321	1.22373E-05	2.32725E-05
CEBPZ	1014.939606	0.130828896	0.029913039	4.373641046	1.22191E-05	2.32402E-05
TMPRSS11F	6.212741679	-0.760692668	0.173916759	-4.373889401	1.22052E-05	2.3216E-05
ISL1	0.588144237	-0.896561724	0.204967015	-4.374175634	1.21892E-05	2.31878E-05
DNAJA1	5527.442877	0.151560771	0.034643799	4.374831122	1.21527E-05	2.31205E-05
GNLY	550.023016	0.38653114	0.088327364	4.376119973	1.20811E-05	2.29865E-05
SVEP1	25.28158908	-0.425653303	0.097263434	-4.376293179	1.20715E-05	2.29705E-05
TG	111.5063306	-0.28764919	0.065715908	-4.377162212	1.20234E-05	2.28814E-05
LYST	10932.77551	-0.286251541	0.065392587	-4.377431058	1.20086E-05	2.28554E-05
CCNE1	290.6386441	-0.204533528	0.046699865	-4.37974558	1.18818E-05	2.26162E-05
UBE2D4	280.3365905	-0.132976127	0.030360034	-4.379972929	1.18694E-05	2.25948E-05
NFATC1	1034.933173	0.195076322	0.044529583	4.380825304	1.18231E-05	2.25088E-05
PRDX5	1390.503843	-0.263159813	0.060067804	-4.381046044	1.18111E-05	2.24882E-05
DNAJC2	1781.3892	0.145723959	0.033260686	4.381267446	1.17991E-05	2.24675E-05
RNF152	80.50400151	-0.414401692	0.094568637	-4.382020338	1.17584E-05	2.23922E-05
SLC35G2	134.8195441	0.36610867	0.08354245	4.382307064	1.17429E-05	2.23649E-05
ZMYM2	7088.698447	0.149122025	0.034017094	4.383737969	1.1666E-05	2.22206E-05
ANKDD1A	516.0764028	0.2332225	0.053194993	4.384294248	1.16362E-05	2.2166E-05
GAL3ST2	125.849157	0.447001309	0.101955114	4.384295109	1.16362E-05	2.2166E-05
ADI1	1052.040273	-0.165847769	0.037826904	-4.384386592	1.16313E-05	2.2161E-05
HES4	146.1366656	0.408602701	0.093190501	4.384596019	1.16201E-05	2.21418E-05
GALM	312.5737367	-0.297677415	0.067875159	-4.385660675	1.15634E-05	2.20359E-05
TKTL1	516.3453379	0.579699826	0.132178652	4.38572958	1.15598E-05	2.20311E-05

FAM9B	1.950247527	-0.448468883	0.102251641	-4.385933366	1.15489E-05	2.20126E-05
LILRB5	33.96020425	-0.367851547	0.083868076	-4.386073506	1.15415E-05	2.20006E-05
LAYN	28.73026701	0.355302018	0.080987311	4.387131958	1.14855E-05	2.1896E-05
PTS	497.1376698	0.137843536	0.031413895	4.387979753	1.14408E-05	2.18129E-05
IGFBP7	1939.277114	-0.305426786	0.069591014	-4.388882506	1.13935E-05	2.17247E-05
CNGA4	17.52941743	-0.303319452	0.069073725	-4.39124211	1.12705E-05	2.14923E-05
FZD7	92.37447179	-0.335302184	0.076349864	-4.39165396	1.12492E-05	2.14538E-05
SPACA5	0.433783514	-0.564009686	0.128408141	-4.392320328	1.12147E-05	2.13902E-05
SELENOF	2104.764916	0.134458537	0.030611832	4.392371403	1.12121E-05	2.13872E-05
HBG2	370.1774898	-0.602156606	0.137089721	-4.392427105	1.12092E-05	2.13838E-05
CYP4F8	3.098670338	-0.334847348	0.076223889	-4.392944923	1.11825E-05	2.1335E-05
HGS	3766.252169	0.153066706	0.034835568	4.393977582	1.11295E-05	2.12359E-05
CCDC105	0.776819803	-0.648230004	0.147501178	-4.394744601	1.10903E-05	2.11632E-05
SMIM20	252.9089925	-0.214413298	0.048787747	-4.394818636	1.10865E-05	2.1158E-05
PRX	336.7255525	-0.33335847	0.075831179	-4.396060774	1.10233E-05	2.10394E-05
IMMT	2306.040796	-0.080015082	0.018198639	-4.396761739	1.09878E-05	2.09737E-05
TRA2A	5767.859239	0.152873642	0.034764849	4.397362483	1.09574E-05	2.09178E-05
NIM1K	27.67308304	-0.226712305	0.051552459	-4.397701073	1.09404E-05	2.08872E-05
GALE	367.7809182	-0.191056917	0.043433267	-4.398861255	1.0882E-05	2.07779E-05
ELOVL4	5.974515365	0.434140815	0.098689624	4.399052265	1.08725E-05	2.07616E-05
PPP3R2	4.659226105	-0.491047897	0.111617107	-4.399396396	1.08552E-05	2.07308E-05
CCDC9	910.2326549	0.15965383	0.03628623	4.399846184	1.08328E-05	2.06899E-05
LRIT2	0.927392421	-0.758784495	0.172441757	-4.400236383	1.08133E-05	2.06547E-05
MAN2B1	5547.227725	0.169319871	0.038478796	4.400342206	1.0808E-05	2.06466E-05
TMEM156	274.7471594	-0.259800891	0.059038303	-4.400548057	1.07978E-05	2.06291E-05
KCNK5	698.605603	-0.338483419	0.076893205	-4.401993872	1.07261E-05	2.04941E-05
ZCCHC18	43.00646136	0.318949008	0.072431579	4.403452372	1.06542E-05	2.03587E-05
CACHD1	135.3151434	-0.39908869	0.090612637	-4.404338103	1.06107E-05	2.02777E-05
LAMP5	833.6142751	-0.809571259	0.183809901	-4.404394175	1.0608E-05	2.02744E-05
OR8S1	0.569222124	-0.958428194	0.217528637	-4.405986288	1.05304E-05	2.0128E-05
SDR16C5	0.992252081	-0.653865669	0.14840205	-4.406042012	1.05277E-05	2.01248E-05
RNF166	1961.054224	0.157623124	0.035761922	4.407568532	1.04538E-05	1.99855E-05
ANXA6	3740.497961	0.253383043	0.057480091	4.408187952	1.04239E-05	1.99304E-05
GALNT7	1239.587248	-0.153363414	0.034789408	-4.408336459	1.04168E-05	1.99187E-05
OR3A1	3.996095053	-0.809736829	0.1836814	-4.408376827	1.04148E-05	1.99169E-05
GCHFR	30.15794673	-0.207202273	0.046997676	-4.408776963	1.03956E-05	1.98821E-05
MT1G	37.44555672	-0.665168135	0.15086988	-4.408886221	1.03904E-05	1.9874E-05

ACTR8	673.9540533	-0.101846202	0.023099711	-4.40898168	1.03858E-05	1.98672E-05
FNIP2	1137.640681	0.259206127	0.058788513	4.409128813	1.03787E-05	1.98556E-05
RARB	74.38106251	-0.314795009	0.07138968	-4.409531023	1.03595E-05	1.98207E-05
ATR	2069.515756	-0.164818426	0.037377643	-4.409545729	1.03588E-05	1.98207E-05
PPEF1	10.46769979	-0.386002189	0.087536418	-4.409618293	1.03553E-05	1.98166E-05
VPS33A	323.2548299	-0.124536024	0.028230327	-4.411426943	1.02692E-05	1.96537E-05
CLEC5A	664.213972	0.445825523	0.101036163	4.412534191	1.02168E-05	1.95553E-05
BEND7	19.5055288	-0.527886158	0.119629727	-4.412667084	1.02105E-05	1.95452E-05
TM2D2	328.2306555	0.139097103	0.03152189	4.412714553	1.02083E-05	1.95429E-05
HGF	1849.415757	0.452181223	0.102455862	4.413424623	1.01748E-05	1.94807E-05
HOXC4	10.89895537	-0.532206673	0.12057432	-4.413930543	1.01511E-05	1.94372E-05
EMC9	120.3724642	-0.180161152	0.040799364	-4.415783303	1.00645E-05	1.92733E-05
PRM3	0.627256883	-0.71312941	0.161431787	-4.417527809	9.98362E-06	1.91203E-05
MVK	215.1907172	-0.182380603	0.041282648	-4.417851416	9.96869E-06	1.90936E-05
PAPOLB	3.855453645	-0.440796304	0.099759989	-4.418568077	9.9357E-06	1.90322E-05
OR10H1	12.44074002	-0.419511102	0.094932818	-4.419031366	9.91443E-06	1.89934E-05
PPP1R14B	1132.125306	-0.260805984	0.059015948	-4.419245857	9.90459E-06	1.89764E-05
ADAM22	240.6247147	-0.264278693	0.059792675	-4.419917538	9.87386E-06	1.89193E-05
ZNF776	1027.880323	0.180675492	0.040862922	4.421502005	9.80172E-06	1.87829E-05
BARHL2	0.720499644	-0.753409162	0.170380424	-4.421923277	9.78262E-06	1.87482E-05
FBXW4	994.9746555	-0.121867152	0.027559059	-4.422036022	9.77752E-06	1.87402E-05
PRSS1	3.600934862	-0.457584198	0.103478075	-4.422040095	9.77733E-06	1.87402E-05
SLC22A18	251.623582	-0.233744495	0.052844439	-4.423256227	9.72243E-06	1.86383E-05
H3C13	565.8808287	-0.361149256	0.081647658	-4.423265318	9.72202E-06	1.86383E-05
TRPM8	7.225196794	-0.566958648	0.128165252	-4.423653349	9.70457E-06	1.86077E-05
RDH8	1.144756479	-0.565098897	0.1277408	-4.423793308	9.69828E-06	1.85975E-05
LYPD1	1.221547573	-0.695179294	0.157143444	-4.423851713	9.69566E-06	1.85943E-05
HOXD8	0.599059282	-0.647068067	0.146265804	-4.423918982	9.69264E-06	1.85903E-05
CLN8	1846.712012	0.246109764	0.055631409	4.423935468	9.6919E-06	1.85903E-05
LYPD6B	8.823821907	-0.647701716	0.146393135	-4.424399522	9.67109E-06	1.85526E-05
MAP7D2	38.54730787	-0.370088344	0.083646496	-4.424433298	9.66958E-06	1.85515E-05
GHSR	9.723230344	-0.544678845	0.123105885	-4.424474458	9.66773E-06	1.85498E-05
STYX	764.2366282	0.124092306	0.028028721	4.427326772	9.54081E-06	1.83081E-05
CCDC63	2.417103165	-0.440800199	0.099555147	-4.427698744	9.52438E-06	1.82783E-05
AC073082.1	0.778307591	-0.525286135	0.11863372	-4.427797877	9.52E-06	1.82717E-05
CELA1	17.40399742	-0.350303355	0.079112917	-4.427890747	9.51591E-06	1.82657E-05
S100A14	2.122368408	-0.482459982	0.108949697	-4.428282005	9.49867E-06	1.82343E-05

SCML4	153.2351373	0.356064713	0.080391808	4.429116886	9.46197E-06	1.81657E-05
FAM71E2	6.113320955	0.619884078	0.139951878	4.429265874	9.45544E-06	1.81549E-05
JTB	198.1047001	-0.236580051	0.053397989	-4.430504911	9.40127E-06	1.80527E-05
GATA4	1.356285202	-0.530835125	0.119807337	-4.430739709	9.39104E-06	1.80348E-05
SUCLA2	734.2027859	-0.119554689	0.026982745	-4.430783082	9.38915E-06	1.8033E-05
CFHR5	0.562365467	-1.148317821	0.259166482	-4.430811474	9.38791E-06	1.80323E-05
DMGDH	19.20782117	-0.316403286	0.071387348	-4.432203922	9.32747E-06	1.7918E-05
RBM8A	1412.915941	-0.134638125	0.030375127	-4.432512303	9.31414E-06	1.78942E-05
NPTX1	51.95899821	0.512901242	0.115711336	4.432592854	9.31066E-06	1.78892E-05
MTIF2	654.2554921	-0.127645276	0.02879619	-4.432714012	9.30543E-06	1.78809E-05
SLC25A26	602.9977704	0.120624528	0.027211775	4.432806336	9.30144E-06	1.7875E-05
DELE1	2337.259042	-0.115609646	0.026080285	-4.432836705	9.30013E-06	1.78743E-05
ZNF528	388.0085526	-0.234467918	0.052883755	-4.433647282	9.26522E-06	1.78089E-05
MIEN1	353.4152211	-0.269951154	0.060882817	-4.43394649	9.25236E-06	1.77859E-05
PRSS23	29.58774517	-0.380458184	0.085800588	-4.434214197	9.24087E-06	1.77656E-05
RASAL3	2219.976488	-0.153030322	0.034499511	-4.435724423	9.17632E-06	1.76432E-05
FAM9C	5.39377856	-0.388086936	0.087484481	-4.436066028	9.16178E-06	1.7617E-05
OPN4	1.087731219	-0.57804946	0.130299386	-4.436317611	9.15108E-06	1.75982E-05
ATG4A	409.4152853	-0.191870292	0.043242898	-4.437035942	9.12061E-06	1.75413E-05
HECTD2	79.42924044	-0.327982153	0.07390576	-4.437842915	9.08649E-06	1.74774E-05
KIT	3134.753559	-0.34278386	0.077222431	-4.438915708	9.04132E-06	1.73922E-05
SERPINA10	2.412338511	-0.521633945	0.117505392	-4.439234131	9.02796E-06	1.73682E-05
ZBTB26	586.8505058	0.163475963	0.036818632	4.440033592	8.99448E-06	1.73055E-05
PNMA8B	11.41277977	-0.466286132	0.105015816	-4.440151501	8.98956E-06	1.72977E-05
ZNF808	738.5451524	-0.161996815	0.036471218	-4.441771423	8.92213E-06	1.71697E-05
CYBB	6429.043319	0.37632125	0.084720473	4.441916289	8.91612E-06	1.71598E-05
MDH2	3416.106756	-0.21873588	0.049239914	-4.442247368	8.90241E-06	1.71351E-05
FITM1	29.66689667	-0.21821994	0.049122668	-4.442347085	8.89829E-06	1.71289E-05
SPRY3	112.9634194	-0.22477661	0.050592542	-4.442880317	8.87625E-06	1.70881E-05
POFUT1	1085.602944	-0.135492238	0.030490527	-4.443748686	8.84049E-06	1.70209E-05
SERPINB5	0.926231054	-0.572803432	0.128893389	-4.444009383	8.82978E-06	1.7002E-05
PCDHA3	1.66501327	-0.756666396	0.170254961	-4.444313355	8.8173E-06	1.69796E-05
HCAR1	28.78902185	-0.404273809	0.090950973	-4.444964079	8.79066E-06	1.693E-05
NGFR	19.12044319	-0.416069749	0.093599906	-4.445194107	8.78125E-06	1.69135E-05
CLPTM1L	2289.5443	-0.119056121	0.02678229	-4.445330077	8.7757E-06	1.69045E-05
TBX20	3.434004635	-0.869823438	0.195657301	-4.445647739	8.76274E-06	1.68812E-05
TRAM2	895.3361356	-0.183468356	0.041266494	-4.445939956	8.75084E-06	1.68599E-05

UBL4A	534.4844492	-0.169252065	0.038066727	-4.446194323	8.74049E-06	1.68417E-05
FAM117B	1158.040117	0.144162044	0.032409045	4.44820396	8.65913E-06	1.66865E-05
CGB2	0.585767146	-0.840768706	0.188986512	-4.448829168	8.63397E-06	1.66397E-05
SMIM15	318.549604	0.19717288	0.04431512	4.44933651	8.6136E-06	1.66021E-05
MSTN	4.632057914	-0.390500187	0.087764285	-4.449420255	8.61024E-06	1.65972E-05
TTL	1155.650011	-0.1372502	0.030842141	-4.450086668	8.58356E-06	1.65474E-05
TNRC18	12188.67545	-0.182402625	0.040983837	-4.450599043	8.56311E-06	1.65096E-05
FCGBP	160.6935282	0.358287756	0.080487571	4.451466872	8.52857E-06	1.64447E-05
ST8SIA1	45.31957967	-0.389411918	0.087473508	-4.451769758	8.51655E-06	1.64231E-05
CPNE6	23.87157635	-0.327812827	0.073632916	-4.451987565	8.50791E-06	1.64081E-05
LSM5	582.2205534	-0.259939167	0.058375585	-4.452874763	8.47282E-06	1.6342E-05
SH2D6	12.31204494	-0.325575952	0.073115196	-4.452917704	8.47112E-06	1.63403E-05
CIAO2A	647.9903188	-0.223889961	0.050278687	-4.45297949	8.46868E-06	1.63372E-05
USP38	3008.13679	0.152528071	0.034250604	4.453295792	8.45621E-06	1.63148E-05
TMEM190	2.025632376	-0.432751741	0.097160315	-4.453996883	8.42863E-06	1.62632E-05
GBP4	1932.234134	0.325451179	0.073065709	4.454225986	8.41964E-06	1.62474E-05
TSPAN1	9.368573124	-0.321838898	0.072252411	-4.454368951	8.41403E-06	1.62382E-05
PTPN13	80.27028861	-0.416666123	0.093539271	-4.454451254	8.4108E-06	1.62336E-05
AC105052.1	0.692536191	0.630845464	0.141609874	4.454812685	8.39665E-06	1.62078E-05
MAGED4B	0.483882189	-0.701500381	0.157467861	-4.454879722	8.39402E-06	1.62044E-05
DNTT	475.5549658	-0.630551151	0.141533976	-4.455122133	8.38454E-06	1.61877E-05
SLC2A13	176.0840668	-0.26239117	0.058896509	-4.455122663	8.38452E-06	1.61877E-05
ZBTB17	2136.041457	0.151631438	0.034033482	4.455360737	8.37522E-06	1.61729E-05
MT-CYB	281765.2137	-0.488065651	0.109530153	-4.455993494	8.35056E-06	1.61268E-05
ZNF33A	1431.773734	-0.154708623	0.034710827	-4.457071021	8.30871E-06	1.60476E-05
TXNL4A	1262.786557	-0.154235302	0.03460017	-4.457645769	8.28647E-06	1.60062E-05
FCER1A	444.2033971	-0.491958395	0.110347512	-4.458264485	8.26259E-06	1.59617E-05
UBB	5081.537942	-0.232132815	0.052063438	-4.458653222	8.24762E-06	1.59343E-05
CLPP	729.3004181	-0.201625857	0.045198228	-4.460923924	8.16071E-06	1.5768E-05
ADARB2	65.52700065	-0.454946362	0.101973076	-4.461436096	8.14122E-06	1.57319E-05
ALPK3	459.0322767	-0.440144738	0.098640678	-4.462101706	8.11597E-06	1.56846E-05
ZCCHC4	277.7556941	-0.119740581	0.026823659	-4.463991298	8.04468E-06	1.55484E-05
PAGE2	0.710719905	-0.726331034	0.162638857	-4.465913294	7.97279E-06	1.5411E-05
CACFD1	201.1689768	0.232978966	0.05216288	4.466374645	7.95562E-06	1.53793E-05
TCEAL7	0.573682672	-1.148173663	0.257056733	-4.466615792	7.94667E-06	1.53635E-05
MSANTD3	352.154982	0.197169959	0.044139293	4.466994052	7.93263E-06	1.53379E-05
NRBP2	148.8370424	-0.269794953	0.060396706	-4.467047491	7.93065E-06	1.53356E-05

RBBP8NL	9.392992334	-0.554237466	0.124062273	-4.467413452	7.9171E-06	1.53109E-05
FAM72A	28.58584033	-0.250876362	0.056134689	-4.469185961	7.85178E-06	1.51861E-05
IP6K3	1.63563894	-0.491677005	0.110004461	-4.469609709	7.83625E-06	1.51575E-05
SYCP1	2.245516882	-0.730268435	0.163309243	-4.47169076	7.76036E-06	1.50122E-05
KATNA1	583.5535918	0.107814308	0.024102141	4.473225265	7.70485E-06	1.49063E-05
CHRNA2	87.43340007	-0.365504478	0.081706029	-4.47340889	7.69824E-06	1.4895E-05
UGT2B7	0.800924484	-0.903088652	0.201840716	-4.474264016	7.6675E-06	1.4837E-05
MYL7	0.843617801	-0.640865275	0.143232577	-4.47429829	7.66627E-06	1.48361E-05
F13A1	2904.426562	0.437666874	0.097797912	4.475216936	7.63338E-06	1.47739E-05
DNM1L	3147.757511	0.11301707	0.025243139	4.477140103	7.56496E-06	1.46429E-05
LYSMD2	461.3137313	0.185720562	0.04148129	4.477212814	7.56239E-06	1.46394E-05
MRPL55	923.8150637	-0.205471941	0.045883794	-4.478094006	7.53124E-06	1.45805E-05
COMMD3	562.8951927	-0.262900714	0.058706651	-4.478210033	7.52715E-06	1.4574E-05
ANKRD52	2375.950876	-0.133751753	0.029858196	-4.479565789	7.4795E-06	1.44832E-05
PHYHIPL	0.565741063	-1.015120404	0.22657464	-4.480291373	7.45412E-06	1.44355E-05
MRC2	1663.798368	-0.348616316	0.077806133	-4.480576316	7.44418E-06	1.44177E-05
TSNAX-DISC1	11.37730163	-0.327093692	0.072998313	-4.48083907	7.43502E-06	1.44013E-05
HEATR3	495.153423	-0.200570404	0.04475321	-4.4816987	7.40512E-06	1.43449E-05
BICRA	846.8292883	0.166968414	0.037240145	4.483559733	7.3408E-06	1.42217E-05
CDON	18.18067622	-0.346544348	0.077273011	-4.484675075	7.30251E-06	1.41489E-05
NRCAM	65.68178669	0.406173161	0.090558012	4.485226146	7.28366E-06	1.41138E-05
CEACAM3	154.7178664	-0.498972243	0.111245627	-4.485320043	7.28046E-06	1.41089E-05
ZNF628	704.8583339	0.177190735	0.039504489	4.485331664	7.28006E-06	1.41089E-05
KLF12	536.8991932	0.347088578	0.077376524	4.4857091	7.26718E-06	1.4086E-05
DDX24	7411.049418	0.131011584	0.029200428	4.486632324	7.23578E-06	1.40265E-05
HEBP2	1727.399795	0.174686055	0.038934333	4.486684101	7.23402E-06	1.40245E-05
VNN2	590.5017607	0.433215651	0.096552826	4.486825165	7.22924E-06	1.40166E-05
FTL	60099.95857	0.319340795	0.07116475	4.487345131	7.21162E-06	1.39839E-05
WSCD1	25.84164213	-0.692636207	0.154333764	-4.48791107	7.19249E-06	1.39482E-05
CLCN6	2046.238384	0.190951605	0.042537622	4.489005173	7.15566E-06	1.38781E-05
ZC3H6	630.1602782	-0.177835434	0.039610723	-4.489578058	7.13644E-06	1.38422E-05
SLC6A19	167.3179024	-0.573447253	0.127708023	-4.490299338	7.11232E-06	1.37968E-05
PPAN	154.373193	-0.166204151	0.037003854	-4.491536171	7.07113E-06	1.37182E-05
PHLPP1	2576.333936	-0.180510844	0.040185701	-4.491917237	7.05849E-06	1.36951E-05
PIN1	1090.995469	-0.147492435	0.032834425	-4.492006071	7.05554E-06	1.36907E-05
HOMER1	102.7485281	-0.363445214	0.080897404	-4.492668426	7.03363E-06	1.36495E-05
FRAT2	891.7700798	-0.209944823	0.046723322	-4.493362501	7.01073E-06	1.36064E-05

DEFB1	84.28427584	0.704528099	0.156780378	4.493726231	6.99876E-06	1.35846E-05
ACVR1C	12.06168747	0.341533086	0.075998814	4.493926507	6.99218E-06	1.35731E-05
IFIT2	1181.99467	0.398770313	0.088709003	4.495263144	6.9484E-06	1.34895E-05
ZSCAN23	20.87981153	-0.507047136	0.112781466	-4.495837435	6.92967E-06	1.34545E-05
BBS4	306.450768	-0.133268099	0.029642159	-4.495897119	6.92772E-06	1.3452E-05
CALHM5	4.843732532	-0.50754164	0.112889179	-4.495928163	6.92671E-06	1.34514E-05
PPP1R7	786.5672196	-0.192283896	0.042758647	-4.496959286	6.89322E-06	1.33877E-05
SAE1	1405.468689	-0.121408125	0.026995665	-4.497319243	6.88156E-06	1.33664E-05
RETN	504.9970059	0.496156174	0.110294018	4.498486713	6.84389E-06	1.32945E-05
SLC30A4	97.96928871	0.385468593	0.0856764	4.499122215	6.82346E-06	1.32561E-05
S100A3	7.021060852	-0.386271569	0.085846306	-4.49957126	6.80907E-06	1.32295E-05
MRPS30	1111.300886	-0.143960473	0.03199079	-4.500059966	6.79343E-06	1.32004E-05
MED25	2026.049747	-0.176694714	0.039255308	-4.50116741	6.75812E-06	1.31331E-05
PAK3	6.045514555	-0.509999275	0.113299704	-4.501329294	6.75298E-06	1.31244E-05
POFUT2	755.4513479	0.142023984	0.031548101	4.50182359	6.73729E-06	1.30952E-05
FUT4	5311.288591	0.247730951	0.055019647	4.502590689	6.71301E-06	1.30493E-05
MUC12	21.69253322	0.431033393	0.095726095	4.502778422	6.70708E-06	1.30391E-05
TUBE1	243.8988372	-0.164936138	0.036627935	-4.50301491	6.69962E-06	1.30259E-05
IDS	1173.802032	0.178706385	0.039678007	4.503915358	6.67128E-06	1.29721E-05
ZNF718	367.2186038	-0.302422928	0.067141397	-4.504269255	6.66017E-06	1.29518E-05
MED1	3079.453806	0.112793548	0.025030437	4.506255683	6.59816E-06	1.28324E-05
SERP2	50.32314817	-0.490317464	0.108798864	-4.506641387	6.58618E-06	1.28104E-05
SUMO4	57.72467948	0.251227667	0.055731608	4.507812994	6.54993E-06	1.27412E-05
DDTL	101.4786288	-0.229726877	0.050959679	-4.508012602	6.54377E-06	1.27305E-05
ITGB6	2.518364529	-0.592413071	0.131364524	-4.509688413	6.49229E-06	1.26316E-05
ITGB1BP1	742.888076	-0.1613092	0.035768141	-4.509856937	6.48714E-06	1.26228E-05
TMEM242	584.9434363	0.140808683	0.031220556	4.510127362	6.47887E-06	1.2608E-05
TLR6	423.9216456	0.252831972	0.056052711	4.510610981	6.46412E-06	1.25805E-05
LURAP1	17.50945812	-0.447948457	0.09930974	-4.510619558	6.46386E-06	1.25805E-05
H3C6	83.4441907	-0.372162933	0.082506887	-4.510689292	6.46173E-06	1.25784E-05
PTPRD	74.34706201	-0.579303832	0.128428291	-4.51071822	6.46085E-06	1.25779E-05
LEXM	5.699132471	-0.434016244	0.096192231	-4.511967752	6.4229E-06	1.25053E-05
SH3D21	850.620119	-0.247752745	0.054909394	-4.512028419	6.42106E-06	1.25029E-05
MYOM3	3.175466383	-0.407321137	0.090257771	-4.512865013	6.39577E-06	1.24549E-05
H2AC16	682.1386029	-0.366276046	0.08115805	-4.513120327	6.38808E-06	1.24412E-05
EOLA2	732.49154	0.149267498	0.033072141	4.51339078	6.37993E-06	1.24265E-05
BDKRB1	0.827741298	-0.658972915	0.145991829	-4.513765745	6.36866E-06	1.24058E-05

APELA	0.794731159	-0.854432839	0.189292431	-4.513824637	6.36689E-06	1.24036E-05
SIT1	45.20570788	0.326895319	0.072407657	4.514651214	6.34211E-06	1.23566E-05
ACTN1	6063.883568	-0.214804979	0.047575718	-4.51501285	6.33129E-06	1.23367E-05
EPRS1	2844.17592	-0.13662703	0.030256742	-4.515589582	6.31408E-06	1.23044E-05
CHIC1	108.506138	-0.340336481	0.07536389	-4.515909149	6.30457E-06	1.22871E-05
GABRA2	4.947849961	-0.866269498	0.191812808	-4.516223432	6.29522E-06	1.22701E-05
SERPINB1	14539.21487	0.219540366	0.048599063	4.517378533	6.26099E-06	1.22046E-05
CAPN7	1699.380157	0.165386514	0.036611035	4.517395248	6.2605E-06	1.22046E-05
CLSTN2	1054.877429	-0.708302809	0.156780849	-4.517789102	6.24887E-06	1.21834E-05
NUDT16L1	599.9675135	-0.14460467	0.03200042	-4.518836701	6.21803E-06	1.21245E-05
AC068896.1	34.11886717	-0.271242139	0.060008198	-4.520084689	6.18149E-06	1.20544E-05
CP	76.07373523	-0.350961506	0.07763154	-4.520862338	6.15882E-06	1.20114E-05
SLC2A5	1258.665965	-0.311568491	0.068905508	-4.521677607	6.13515E-06	1.19664E-05
L1CAM	38.20135548	0.367522684	0.081270341	4.522223974	6.11933E-06	1.19368E-05
RNF141	952.639648	0.153817329	0.033995844	4.52459214	6.05121E-06	1.18051E-05
RRN3	2107.028151	0.153427641	0.033908094	4.524808717	6.04502E-06	1.17942E-05
TMSB15B	2.442903042	-0.407344666	0.090022926	-4.524899178	6.04243E-06	1.17903E-05
SDHC	859.204243	-0.168687664	0.03727919	-4.524982026	6.04006E-06	1.17869E-05
HOXB6	169.0206056	-0.855899161	0.189112696	-4.525868314	6.0148E-06	1.17387E-05
MYH9	42535.94022	0.148895682	0.032897011	4.526115791	6.00777E-06	1.17262E-05
HEG1	385.4131019	0.323982055	0.071574172	4.526521848	5.99624E-06	1.17048E-05
TYW3	533.5283765	0.154029676	0.034027308	4.526648838	5.99264E-06	1.1699E-05
CAMK2D	416.0180989	0.35017665	0.077357468	4.526733633	5.99024E-06	1.16955E-05
ZDHHC3	3063.63721	0.128818091	0.028455777	4.526957517	5.9839E-06	1.16842E-05
PAIP2	1849.638903	0.164126241	0.036254875	4.52701162	5.98237E-06	1.16824E-05
MYOCD	1.479585141	-0.786524882	0.173739242	-4.527042215	5.9815E-06	1.16819E-05
ANKRD20A3P	2.282464261	-0.624981676	0.138051974	-4.527147704	5.97852E-06	1.16772E-05
CBWD3	134.1086464	-0.208150111	0.045969995	-4.527955923	5.9557E-06	1.16338E-05
LYPLAL1	208.3568364	-0.154995957	0.034228637	-4.528253835	5.94731E-06	1.16186E-05
TSPAN8	0.736253028	-0.784854855	0.173219457	-4.530985527	5.87092E-06	1.14705E-05
EIF3E	14825.72603	0.165926874	0.036616162	4.53152016	5.85607E-06	1.14426E-05
MRGBP	366.8824735	0.14448661	0.031883853	4.531654562	5.85235E-06	1.14365E-05
DOP1B	2877.895353	-0.1755497	0.038738278	-4.53168575	5.85149E-06	1.1436E-05
VIM	31557.3233	0.288702924	0.06369216	4.532785854	5.82108E-06	1.13777E-05
ASIC5	0.485545841	-0.967044737	0.213321053	-4.53328315	5.80739E-06	1.1352E-05
NPL	391.5028517	-0.361521212	0.079736814	-4.53393097	5.7896E-06	1.13184E-05
ZNF219	1015.74151	0.237949104	0.052475067	4.534517352	5.77354E-06	1.12881E-05

LRRC66	2.895045946	-0.508294153	0.112092798	-4.534583503	5.77173E-06	1.12857E-05
C3orf20	88.61035709	-0.350501779	0.077290971	-4.534834703	5.76486E-06	1.12734E-05
DENND2B	292.5774682	-0.312485901	0.068886828	-4.536221336	5.72711E-06	1.12007E-05
MS4A15	0.581004509	-0.630887833	0.139059969	-4.536804075	5.71131E-06	1.11709E-05
LITAFD	26.66973507	-0.316685332	0.069795401	-4.5373381	5.69687E-06	1.11438E-05
MAP6D1	14.22099615	-0.242467643	0.053438044	-4.537360022	5.69628E-06	1.11438E-05
PLK1	674.888896	-0.222081344	0.048941415	-4.537697638	5.68717E-06	1.11271E-05
CPN1	1.075050129	-0.668999515	0.147399459	-4.538683669	5.66065E-06	1.10763E-05
PKMYT1	519.9827556	-0.22970438	0.050601585	-4.539470022	5.63958E-06	1.10361E-05
SMG8	491.8742029	0.136160357	0.029989194	4.540314055	5.61705E-06	1.09932E-05
BID	1824.366885	0.189113885	0.04165186	4.540346657	5.61618E-06	1.09926E-05
NR0B2	3.234307739	-0.364633153	0.08030094	-4.540832955	5.60324E-06	1.09683E-05
PAGE1	1.098789913	-0.863629822	0.190170565	-4.541343305	5.58969E-06	1.09429E-05
HAP1	46.30050619	-0.501375836	0.110388247	-4.541931315	5.57412E-06	1.09135E-05
INMT	6.932516784	-0.378806887	0.083385003	-4.542865889	5.54946E-06	1.08663E-05
CELF6	4.057355079	-0.385773577	0.084884577	-4.544683997	5.50177E-06	1.0774E-05
CA12	22.92242524	-0.476383596	0.104820783	-4.544743725	5.50021E-06	1.0772E-05
MSH3	858.2542248	-0.140633247	0.030939196	-4.545471905	5.48123E-06	1.07359E-05
OR51D1	1.157549841	-0.614731642	0.135172842	-4.547745191	5.42237E-06	1.06217E-05
ANKLE2	5939.25348	0.119050892	0.026173391	4.548546685	5.40177E-06	1.05824E-05
TSPAN33	891.9940652	0.26519083	0.058302203	4.54855592	5.40153E-06	1.05824E-05
LGALS9B	10.06264851	-0.323998577	0.07122173	-4.549153393	5.38622E-06	1.05541E-05
KCNJ9	38.95705191	0.303140736	0.066616799	4.550514885	5.35148E-06	1.0487E-05
GLRX2	286.1002479	-0.132674485	0.029151501	-4.551206002	5.33393E-06	1.04537E-05
GUCD1	4344.296719	0.136481264	0.029984137	4.55178226	5.31934E-06	1.04261E-05
NUDT15	270.43849	0.180650215	0.03968643	4.551939137	5.31537E-06	1.04194E-05
KRT81	3.649834225	0.694266661	0.152501809	4.552514258	5.30086E-06	1.0392E-05
RHOBTB1	241.7449551	-0.332809204	0.073099965	-4.552795699	5.29377E-06	1.03791E-05
NDUFAB1	569.3111733	-0.262055677	0.057547258	-4.553747403	5.26986E-06	1.03333E-05
MMP2	685.6678625	0.538995258	0.11835901	4.553901379	5.26601E-06	1.03268E-05
YIPF4	1616.612758	-0.171914701	0.037748042	-4.554268012	5.25683E-06	1.03098E-05
PDPR	2127.742787	-0.129285762	0.028385908	-4.554575463	5.24915E-06	1.02958E-05
C2CD2L	761.1330054	-0.149667344	0.032859318	-4.554791515	5.24376E-06	1.02862E-05
FUT5	22.03069292	0.390764938	0.085786799	4.55507076	5.23679E-06	1.02736E-05
PPM1L	705.8124935	-0.233242448	0.051190092	-4.556398345	5.20382E-06	1.02099E-05
BMP7	1.684796862	-0.529338084	0.116152763	-4.557257797	5.18258E-06	1.01693E-05
KLC3	32.34089505	-0.387975806	0.085125704	-4.557681004	5.17215E-06	1.01498E-05

TPBG	72.51614669	-0.546621871	0.119934207	-4.557681119	5.17215E-06	1.01498E-05
SLC6A8	1128.547412	-0.404341635	0.088692213	-4.558930477	5.14148E-06	1.00917E-05
ZNF335	3303.657736	0.188438944	0.041332516	4.55909685	5.13741E-06	1.00847E-05
SOAT2	6.826953045	-0.413760099	0.090746356	-4.559523044	5.12699E-06	1.00653E-05
PCDHA13	0.569334045	-0.881483762	0.193318283	-4.559753716	5.12136E-06	1.00552E-05
PGAM1	1836.046554	0.19321475	0.042370266	4.560149516	5.11172E-06	1.00373E-05
TMED10	4516.427906	0.152087332	0.033346245	4.560853342	5.09462E-06	1.00047E-05
CDKN3	206.7704833	-0.231965634	0.050859636	-4.560898418	5.09352E-06	1.00036E-05
EDDM3A	0.754023746	-0.681098556	0.149309275	-4.561662738	5.07501E-06	9.96821E-06
MT2A	1183.743838	0.474111817	0.103933551	4.561682092	5.07454E-06	9.96821E-06
ARMH2	6.810337386	-0.406615654	0.08913366	-4.561864221	5.07014E-06	9.96065E-06
FLT4	470.1595144	-0.570178801	0.124986447	-4.56192503	5.06867E-06	9.95876E-06
CDH4	110.8208268	-0.473492715	0.103788177	-4.562106463	5.0643E-06	9.95116E-06
CSF1	1661.676545	-0.417859088	0.091578322	-4.562860297	5.04614E-06	9.91647E-06
BBIP1	1066.121333	0.206897416	0.045339836	4.563259055	5.03656E-06	9.89864E-06
FXYD1	3.8682991	0.331692738	0.07267459	4.564081281	5.01686E-06	9.86092E-06
EHBP1L1	4956.399485	-0.194339417	0.042572297	-4.564926749	4.99669E-06	9.82225E-06
CABLES2	377.3156854	-0.168888221	0.036992969	-4.565414044	4.98509E-06	9.80045E-06
HJURP	583.8618216	-0.282667817	0.061896857	-4.566755619	4.95331E-06	9.73894E-06
DYNC1I1	39.66281438	-0.723631581	0.158397003	-4.568467619	4.91303E-06	9.66071E-06
ERBIN	3962.447613	0.158662796	0.034729654	4.568510699	4.91202E-06	9.6597E-06
METTL7A	2298.271134	-0.209258229	0.04579989	-4.568967901	4.90132E-06	9.63962E-06
HLA-G	32.62194287	0.27165928	0.059449884	4.569551088	4.8877E-06	9.6138E-06
KIF13B	1805.257341	0.193572119	0.042355446	4.570182513	4.873E-06	9.58585E-06
NLGN1	26.5772685	-0.880105573	0.192533096	-4.571191102	4.8496E-06	9.54078E-06
PARK7	1801.610792	-0.208097923	0.045517607	-4.571811547	4.83526E-06	9.51352E-06
CHCHD3	1068.89272	-0.136724702	0.029901551	-4.572495325	4.8195E-06	9.48347E-06
ZNF12	1194.262966	0.154628924	0.03381074	4.573366986	4.79948E-06	9.44503E-06
NEIL3	216.5350306	-0.273395185	0.059765262	-4.574483202	4.77397E-06	9.39577E-06
SDHD	757.4312614	0.194755039	0.042566814	4.575278785	4.75586E-06	9.36107E-06
TMIGD3	33.50845557	0.397221487	0.086808835	4.575818674	4.74361E-06	9.3379E-06
RPL26L1	215.2065468	-0.238434953	0.052105474	-4.57600585	4.73937E-06	9.33049E-06
GRIK1	50.25714033	-0.329078376	0.071910829	-4.576200544	4.73497E-06	9.32275E-06
UGT2A1	1.707865905	-1.083762753	0.236812803	-4.576453386	4.72925E-06	9.31244E-06
DNALI1	39.83338399	-0.301570418	0.065889141	-4.576936547	4.71834E-06	9.2919E-06
TMEM144	196.1025965	-0.280662038	0.061316902	-4.577237744	4.71156E-06	9.27947E-06
ZNF480	398.8274319	-0.159322001	0.034804229	-4.577662106	4.70201E-06	9.2616E-06

H4C12	161.4671551	-0.297913553	0.065066181	-4.578623617	4.68046E-06	9.22006E-06
RAP1GAP	568.8456211	-0.475094009	0.103720757	-4.580510454	4.63842E-06	9.13819E-06
DRAM2	1075.170943	0.149215641	0.03257016	4.581360334	4.61961E-06	9.10204E-06
OR14J1	1.141019548	-0.508151911	0.110903747	-4.581918331	4.6073E-06	9.07869E-06
NXNL1	1.16965102	-0.43844229	0.095674728	-4.582634298	4.59155E-06	9.04856E-06
TIGD2	158.3871013	-0.193947249	0.042317262	-4.583170997	4.57977E-06	9.02627E-06
SAGE1	183.6396475	-0.984259433	0.214751367	-4.583251067	4.57802E-06	9.02372E-06
CENPS-CORT	2.205533457	-0.334424455	0.072950192	-4.584284766	4.55543E-06	8.9801E-06
PEX14	469.2890981	-0.118509949	0.025844644	-4.585474222	4.52957E-06	8.93003E-06
KLRC1	10.90277817	0.417485696	0.09103577	4.585952259	4.51922E-06	8.91051E-06
UROC1	7.482653908	-0.418251612	0.091182404	-4.586977246	4.4971E-06	8.86779E-06
OR5AS1	0.714712268	-0.65864799	0.143536489	-4.588714643	4.45984E-06	8.7952E-06
ARL6IP5	3098.315451	0.155803093	0.03395288	4.58880348	4.45794E-06	8.79234E-06
DENND1B	2190.939535	0.193253164	0.042113392	4.588876727	4.45637E-06	8.79015E-06
PSG1	0.619218712	-0.978789354	0.213294483	-4.588910793	4.45565E-06	8.7896E-06
RAG1	75.04472183	-0.505643123	0.110178021	-4.589328407	4.44674E-06	8.77292E-06
TPRX1	1.540928961	-0.688456516	0.150004447	-4.589574052	4.44151E-06	8.76348E-06
G6PD	2123.116157	-0.19647595	0.042809011	-4.589593344	4.4411E-06	8.76348E-06
WDR17	36.09524971	-0.493378839	0.107487464	-4.590105884	4.43021E-06	8.74295E-06
NLGN4Y	2.380979084	-0.781877366	0.170286035	-4.591553073	4.3996E-06	8.6834E-06
UAP1	987.9789252	0.215849853	0.047009261	4.591645298	4.39765E-06	8.68044E-06
C9	3.429037842	-0.513189373	0.111763123	-4.59175941	4.39525E-06	8.67657E-06
TCTEX1D1	321.709437	-0.415589898	0.090507614	-4.591767264	4.39508E-06	8.67657E-06
NUTM2E	12.72011809	-0.444678669	0.096835243	-4.592116023	4.38774E-06	8.6635E-06
RTN2	147.7798783	-0.256275217	0.055801041	-4.592660131	4.37631E-06	8.64181E-06
ACOX1	1602.592437	-0.127835007	0.027829466	-4.593512829	4.35846E-06	8.60742E-06
NLRP7	14.2709657	-0.42255738	0.0919886	-4.593584208	4.35697E-06	8.60535E-06
ASZ1	0.659523942	-0.752709841	0.163825327	-4.59458776	4.33606E-06	8.5649E-06
ZNF354C	61.01785387	-0.378222629	0.082312713	-4.594947909	4.32857E-06	8.55099E-06
ADGRF4	2.845453324	-0.696659675	0.151567728	-4.596358902	4.29938E-06	8.49417E-06
STARD3	1637.262459	0.106065246	0.023072748	4.596992343	4.28633E-06	8.46925E-06
DCAF12	3340.829844	-0.362435287	0.078822357	-4.598128	4.26304E-06	8.42408E-06
PPP1R35	318.3436381	-0.178997289	0.038905506	-4.600821469	4.20828E-06	8.31671E-06
TBXA2R	195.0095137	-0.364338847	0.079179643	-4.601420684	4.19619E-06	8.29365E-06
LRRC53	0.470724996	-1.028669691	0.223538814	-4.601749795	4.18956E-06	8.28139E-06
C16orf46	3.681691833	-0.301087692	0.065413091	-4.602865986	4.16716E-06	8.23795E-06
CBX6	2288.027568	0.235418202	0.051145244	4.602934417	4.16579E-06	8.23607E-06

AREG	1802.424312	0.541710432	0.11768744	4.602958746	4.16531E-06	8.23594E-06
TRPC4AP	4996.768392	0.131097239	0.028480177	4.603104856	4.16239E-06	8.23099E-06
MB	3.039602353	-0.623681313	0.135476497	-4.603612634	4.15224E-06	8.21177E-06
ZNF839	421.4781945	-0.138049439	0.029985502	-4.603872797	4.14706E-06	8.20234E-06
FZR1	3029.328664	0.180924252	0.039296324	4.60410124	4.14251E-06	8.19417E-06
ILKAP	2572.266844	0.156077639	0.033898224	4.604301329	4.13853E-06	8.18713E-06
VPS37B	1926.061127	0.167560217	0.036384556	4.605256661	4.11957E-06	8.15045E-06
RAB34	1487.795858	0.353996668	0.076861741	4.605629033	4.11221E-06	8.1367E-06
KLHL29	256.0875686	-0.307674789	0.066803036	-4.605700672	4.11079E-06	8.13472E-06
UBAP1L	367.0356577	-0.234353197	0.050876882	-4.6062806	4.09935E-06	8.1129E-06
MRE11	1631.219874	-0.149167198	0.03238084	-4.606650074	4.09208E-06	8.09933E-06
BTNL2	3.38177853	-0.496792953	0.10782871	-4.607241942	4.08045E-06	8.07713E-06
FGF5	4.687942663	-0.510041648	0.110701033	-4.607379308	4.07776E-06	8.07262E-06
PRND	9.721176713	-0.415334749	0.090087366	-4.610355114	4.01982E-06	7.95872E-06
TTC33	181.951303	-0.192326865	0.041711133	-4.610924054	4.00883E-06	7.93777E-06
ZNF845	302.5956961	0.199912763	0.043347719	4.611840463	3.99119E-06	7.90364E-06
DTX3	114.7136678	-0.410044039	0.088849811	-4.615024341	3.93049E-06	7.78423E-06
RTP2	0.754338045	-0.75812505	0.164229045	-4.616266559	3.90705E-06	7.73859E-06
SLC17A1	0.640746087	-0.761059728	0.164863228	-4.616309748	3.90624E-06	7.73776E-06
TBXAS1	2070.176658	-0.21483383	0.046537328	-4.616376507	3.90498E-06	7.73605E-06
AGR3	1.075478371	-0.918900946	0.199046747	-4.61650822	3.90251E-06	7.73193E-06
TMEFF1	4.371639874	-0.661847561	0.143345328	-4.61715473	3.89037E-06	7.70867E-06
ATP2B3	13.64026815	-0.332898462	0.072098747	-4.617257267	3.88845E-06	7.70564E-06
TLE5	4005.056415	0.197999414	0.042880376	4.617483193	3.88422E-06	7.69804E-06
GABBR1	973.9519309	-0.427184963	0.092508076	-4.617812645	3.87806E-06	7.68661E-06
GLYCTK	436.5977948	-0.182320934	0.039463375	-4.620003575	3.83733E-06	7.60666E-06
GAL	21.11742232	-0.496756277	0.10751691	-4.620261852	3.83256E-06	7.59796E-06
BRD9	2005.542983	-0.152558074	0.033017714	-4.620491659	3.82832E-06	7.59032E-06
P4HA1	1140.398842	0.15918011	0.034444561	4.621342433	3.81265E-06	7.56002E-06
S100P	1235.969204	0.468439483	0.10136107	4.621493058	3.80988E-06	7.5553E-06
NDRG2	305.9484033	-0.349659866	0.075647109	-4.622250242	3.796E-06	7.52853E-06
GJB1	7.981065021	-0.406710476	0.08798783	-4.622349176	3.79419E-06	7.5257E-06
SOX1	1.379535318	-0.690760143	0.149417172	-4.623030496	3.78174E-06	7.50178E-06
IRF4	651.9951148	0.40784813	0.08821548	4.623317038	3.77652E-06	7.49217E-06
USO1	1370.748681	-0.159164663	0.034420639	-4.624105464	3.76218E-06	7.46449E-06
H2BC3	544.0560372	-0.421922364	0.09123433	-4.624600917	3.7532E-06	7.44743E-06
DACH2	0.682545681	-0.785613104	0.169865037	-4.624925287	3.74733E-06	7.43654E-06

CRLS1	237.117119	-0.15273335	0.033021496	-4.625270505	3.7411E-06	7.42492E-06
DOCK9	276.2628076	0.365664161	0.079057292	4.625305912	3.74046E-06	7.4244E-06
ZFAND2A	1376.14601	0.211015303	0.045611877	4.62632359	3.72214E-06	7.38878E-06
ZP1	21.75072605	-0.409935501	0.088584112	-4.627641357	3.69854E-06	7.34268E-06
YOD1	3130.167558	0.247720554	0.053528783	4.627800925	3.69569E-06	7.33777E-06
HR	51.58532715	0.468868436	0.101301195	4.628459071	3.68397E-06	7.31524E-06
NUDCD2	964.9061757	0.173172471	0.037412054	4.628788161	3.67812E-06	7.30436E-06
OR10A5	0.975996579	-0.942491813	0.203574068	-4.629724307	3.66153E-06	7.27216E-06
MRPS18C	168.5416638	-0.176419326	0.038102321	-4.630146458	3.65407E-06	7.25808E-06
SCGB2A2	1.051508185	-0.882603301	0.190617883	-4.630222964	3.65272E-06	7.25614E-06
MARCHF6	11488.7564	0.1539128	0.033240718	4.63024899	3.65226E-06	7.25596E-06
BSDC1	5616.21263	0.185789079	0.040121684	4.630640136	3.64537E-06	7.243E-06
TCEAL2	0.641222143	-0.724335048	0.156397078	-4.631384785	3.63228E-06	7.21773E-06
SGCG	2.871920461	-0.463506307	0.100074469	-4.63161395	3.62826E-06	7.21048E-06
OXER1	109.670026	-0.258290154	0.055761404	-4.632059751	3.62046E-06	7.19569E-06
TRIM34	6.449155759	-0.299719838	0.064698923	-4.632532074	3.6122E-06	7.18002E-06
PITPNC1	746.800756	0.205364102	0.044314938	4.634195885	3.58328E-06	7.12324E-06
MICAL1	7035.48428	-0.20513179	0.044260648	-4.634631468	3.57574E-06	7.10898E-06
ASF1A	433.9605135	-0.13331144	0.028763762	-4.634701079	3.57454E-06	7.10731E-06
NKTR	10888.07759	0.171402928	0.036977985	4.635269496	3.56473E-06	7.08853E-06
NBL1	270.6238024	0.411448066	0.088760486	4.635486832	3.56098E-06	7.08181E-06
G2E3	990.1551537	0.17169642	0.037031162	4.63653888	3.54292E-06	7.04659E-06
OR4K13	0.959572188	-0.572016015	0.123356424	-4.637099513	3.53332E-06	7.02823E-06
ADGRG4	1.698695104	-0.567183613	0.122305468	-4.637434629	3.5276E-06	7.01756E-06
C19orf53	839.8432595	-0.3682281	0.079396936	-4.637812474	3.52116E-06	7.00546E-06
MDGA1	192.5489522	-0.428857839	0.092461367	-4.638238131	3.51392E-06	6.99176E-06
GRB10	1702.981291	-0.333465662	0.071893634	-4.638319746	3.51253E-06	6.98971E-06
LYAR	289.1504021	-0.253729402	0.054699105	-4.638639012	3.50711E-06	6.97963E-06
RPL36A	284.0236189	-0.179500651	0.03869117	-4.639318274	3.4956E-06	6.95744E-06
IL17RA	7055.536873	0.28386834	0.061166123	4.640940548	3.46827E-06	6.90374E-06
PTPRN2	153.2950536	-0.402613032	0.086747833	-4.641188344	3.46411E-06	6.89616E-06
OR2AE1	3.373075033	-0.394136504	0.084918697	-4.641339522	3.46158E-06	6.89182E-06
SMIM40	2.316694029	-0.461188407	0.099340654	-4.642494153	3.44228E-06	6.8541E-06
KITLG	22.209282	-0.48068062	0.103536616	-4.642614752	3.44027E-06	6.8508E-06
ACTR2	9370.405713	0.150241014	0.032358922	4.642954833	3.43461E-06	6.84022E-06
AC107871.1	364.91807	0.202549385	0.043622548	4.6432268	3.43009E-06	6.83192E-06
PPIF	3266.997335	0.207374713	0.044661703	4.643233479	3.42998E-06	6.83192E-06

SNX16	347.378941	0.174867481	0.037657109	4.643677752	3.42261E-06	6.8184E-06
SCYGR4	0.991547021	-0.60954407	0.131252332	-4.644062786	3.41624E-06	6.80639E-06
TMEM273	105.3353605	-0.400313817	0.086186081	-4.644761823	3.40469E-06	6.78408E-06
OR2A14	0.672437045	-0.767070721	0.165111471	-4.645774849	3.38802E-06	6.75156E-06
ABCB6	50.53193418	-0.405193336	0.087215714	-4.645875369	3.38637E-06	6.74896E-06
HDGFL1	0.399624462	-0.992523687	0.213614793	-4.646324702	3.37901E-06	6.73497E-06
SLC17A9	2747.453126	-0.25717562	0.055326937	-4.648289478	3.34699E-06	6.67182E-06
CHRM1	4.534755718	-0.600820764	0.129250336	-4.648504451	3.3435E-06	6.66555E-06
HUWE1	17032.17711	-0.128540363	0.027649303	-4.648954972	3.33621E-06	6.65169E-06
PDCD2	1287.112221	-0.121784244	0.02619437	-4.64925264	3.3314E-06	6.64278E-06
ASGR2	164.6225201	0.481377625	0.103538079	4.64928104	3.33094E-06	6.64254E-06
FRG2B	0.599761684	-0.888237105	0.191047144	-4.64930848	3.3305E-06	6.64233E-06
RIMKLB	1246.34578	-0.207968682	0.044724693	-4.649974535	3.31976E-06	6.62159E-06
CRB2	144.6782879	-0.330724762	0.071122816	-4.650051552	3.31852E-06	6.61979E-06
CENPW	261.6785253	-0.268529132	0.057718959	-4.652355787	3.28164E-06	6.54689E-06
TEX54	21.77397698	-0.352227972	0.07570828	-4.652436596	3.28036E-06	6.545E-06
RTL8C	223.3284039	-0.471123636	0.101262809	-4.652484352	3.2796E-06	6.54415E-06
GPR101	2.384902012	-0.496510829	0.106716998	-4.652593679	3.27786E-06	6.54134E-06
NUCKS1	5287.682838	-0.144598017	0.03107864	-4.652649453	3.27697E-06	6.54024E-06
SLC30A2	9.970171412	-0.351438493	0.075523325	-4.653376865	3.26543E-06	6.51787E-06
CPAMD8	121.526584	0.437187013	0.093944042	4.653695985	3.26037E-06	6.50844E-06
ADRA2A	47.9015477	-0.496186275	0.106603266	-4.654512876	3.24748E-06	6.48336E-06
H2BC12	4123.517803	-0.231920825	0.049827001	-4.654521048	3.24735E-06	6.48336E-06
HACE1	598.9683029	-0.211276612	0.045391638	-4.654527218	3.24725E-06	6.48336E-06
INTS12	351.9947686	0.148270054	0.031845579	4.655907045	3.22557E-06	6.4416E-06
INF2	2611.315549	-0.215698844	0.046327211	-4.655985982	3.22434E-06	6.43979E-06
NCL	10482.76223	-0.157749875	0.033878502	-4.65634155	3.21878E-06	6.42934E-06
TAF12	388.7037336	-0.172932506	0.037137246	-4.656578672	3.21507E-06	6.4226E-06
PPT1	3561.570586	0.245943521	0.05281374	4.656809359	3.21148E-06	6.41607E-06
RBX1	1133.981937	-0.258723321	0.055519309	-4.660060206	3.16117E-06	6.31621E-06
KRT74	0.716941724	-0.736536281	0.157953336	-4.662999195	3.11634E-06	6.22727E-06
CLOCK	598.987454	-0.177011457	0.037956612	-4.663521049	3.10844E-06	6.21213E-06
PLEKHG3	702.3823591	-0.387376345	0.08306322	-4.663632643	3.10676E-06	6.20939E-06
HDDC2	443.9903389	-0.241029996	0.051671669	-4.66464508	3.0915E-06	6.17953E-06
ZP3	133.2261864	-0.232196278	0.049776941	-4.664735758	3.09014E-06	6.17744E-06
MMP27	2.814534063	-0.764102259	0.163784367	-4.665294203	3.08176E-06	6.16131E-06
MXD3	502.488722	-0.176344148	0.037795987	-4.665684482	3.07591E-06	6.15026E-06

CSPP1	2366.971686	-0.182399778	0.039084071	-4.666857186	3.05842E-06	6.1159E-06
PGLS	1316.433164	-0.230396325	0.049368124	-4.666904559	3.05771E-06	6.11511E-06
HS6ST3	1.18744156	-0.799527331	0.171275359	-4.668081463	3.04025E-06	6.08082E-06
OR2K2	1.085704718	-0.674296243	0.144396569	-4.66975253	3.01563E-06	6.03218E-06
DEPDC5	1284.05308	-0.201070637	0.043048747	-4.670766287	3.00078E-06	6.0031E-06
IFI27	553.9552316	-0.572038897	0.122442799	-4.671886805	2.98445E-06	5.97104E-06
CDK3	5.869178171	-0.247826719	0.053027817	-4.673522953	2.96077E-06	5.92426E-06
PCDHA6	0.833500976	-0.902324258	0.193071163	-4.673532006	2.96064E-06	5.92426E-06
SSTR3	34.22586851	0.376759507	0.080612506	4.673710382	2.95806E-06	5.92006E-06
CPXM2	2.394783053	-0.560789134	0.119986903	-4.673752904	2.95745E-06	5.91944E-06
RPL26	1891.370081	-0.35420413	0.075780332	-4.674090512	2.95259E-06	5.91032E-06
WFDC10B	1.055231744	1.026274123	0.219563727	4.674151499	2.95171E-06	5.90916E-06
SLC18A2	152.7999092	-0.362308112	0.077488533	-4.675635218	2.93045E-06	5.8672E-06
SUPV3L1	1449.684191	0.142516261	0.030471795	4.676989373	2.91117E-06	5.8292E-06
OR56B4	0.744772659	-0.618680816	0.132263122	-4.677651707	2.90179E-06	5.811E-06
CD320	283.2712753	-0.243486268	0.052046224	-4.678269568	2.89306E-06	5.79411E-06
APAF1	2400.471276	-0.200374153	0.042829885	-4.678372481	2.89161E-06	5.7918E-06
MAP1LC3C	1.302313509	-0.542254104	0.11589916	-4.678671558	2.8874E-06	5.78395E-06
ACSM2A	1.991685171	-0.458807713	0.098049504	-4.67934762	2.87789E-06	5.7655E-06
NRDE2	1423.49989	-0.143781033	0.030721836	-4.680092371	2.86746E-06	5.74519E-06
COL1A2	121.5425085	-0.577482841	0.123372554	-4.680804768	2.85751E-06	5.72584E-06
KIF16B	491.5441427	-0.318513109	0.068045851	-4.680860079	2.85674E-06	5.72488E-06
GALNT9	11.34953447	0.446899572	0.095469707	4.681061539	2.85393E-06	5.71985E-06
ZC3H12A	2798.461187	0.259732506	0.055485407	4.681095794	2.85346E-06	5.71948E-06
ST3GAL4	1754.62224	-0.272416767	0.058191163	-4.681411261	2.84907E-06	5.71127E-06
PTPN20	16.23801681	-0.604199311	0.129050066	-4.681898503	2.8423E-06	5.69829E-06
RPIA	1105.697587	-0.182490914	0.038951783	-4.685046536	2.79896E-06	5.61198E-06
DOCK7	1108.880296	-0.215375443	0.045964829	-4.685657437	2.79063E-06	5.59583E-06
RANGAP1	2891.914141	-0.135460383	0.028905322	-4.686347513	2.78124E-06	5.57758E-06
PTPN3	6.721257236	-0.466707151	0.099586539	-4.686448143	2.77987E-06	5.57541E-06
PCDHA7	0.917239786	-0.858226251	0.18312292	-4.686612953	2.77764E-06	5.5715E-06
CDK5	260.1945197	-0.244043852	0.052057318	-4.687983591	2.7591E-06	5.53489E-06
ALKBH7	1092.362362	-0.278631165	0.059432166	-4.688221645	2.75589E-06	5.52902E-06
MRPL11	564.0143376	-0.274778557	0.058610095	-4.688246229	2.75556E-06	5.52892E-06
GINS3	108.6836041	-0.244820059	0.052216052	-4.688597654	2.75084E-06	5.52E-06
WDR36	1083.677025	-0.16157692	0.034459747	-4.68885976	2.74732E-06	5.5135E-06
TPST1	277.1423611	0.362853677	0.077361795	4.690347174	2.72742E-06	5.47414E-06

OR8A1	1.3434391	-0.660140412	0.140723872	-4.691033613	2.71828E-06	5.45636E-06
SLC39A5	25.49329317	-0.30125825	0.064196856	-4.692725907	2.69589E-06	5.41196E-06
FAM205C	0.726139523	-0.848406664	0.180781354	-4.692998725	2.69229E-06	5.4053E-06
TGM7	1.785813695	-0.534590813	0.11387348	-4.694603299	2.67125E-06	5.36359E-06
AP002373.1	10.32049774	0.331460401	0.070602181	4.694761529	2.66918E-06	5.35999E-06
PTH	0.483614164	-0.681928756	0.145236261	-4.695306471	2.66207E-06	5.34627E-06
DHX36	2134.632812	0.164962904	0.035133258	4.695348915	2.66152E-06	5.34571E-06
CTXN1	21.43218905	-0.383954299	0.081768764	-4.695610892	2.65811E-06	5.33941E-06
GOLGA8A	1307.351526	-0.351523257	0.074859026	-4.695803266	2.65561E-06	5.33493E-06
ZNF227	264.0444692	-0.152662285	0.032509626	-4.695910231	2.65422E-06	5.33269E-06
SYCN	0.451403995	-0.800463245	0.170449862	-4.696180085	2.65072E-06	5.3262E-06
REXO1	2059.305902	0.168373547	0.03584122	4.697762678	2.63027E-06	5.28565E-06
PSMB10	571.5864612	-0.193400361	0.041166733	-4.697976867	2.62751E-06	5.28065E-06
MDH1	1530.836051	-0.161294502	0.034332666	-4.697989472	2.62735E-06	5.28065E-06
HSPB1	2960.702174	-0.458426658	0.097560985	-4.698872773	2.61601E-06	5.25863E-06
CNP	3068.982306	0.145770121	0.031021895	4.698943126	2.61511E-06	5.25735E-06
MGAT4D	0.712771716	-0.715711318	0.152304316	-4.699218899	2.61158E-06	5.2508E-06
STARD10	257.4486605	0.276954411	0.058936026	4.69923794	2.61134E-06	5.2508E-06
HSDL2	536.0479849	-0.161410305	0.034344516	-4.699740227	2.60493E-06	5.23849E-06
PPM1F	3251.396551	-0.189148965	0.040243374	-4.700126867	2.6E-06	5.22912E-06
U52112.1	2.632230569	-0.340182189	0.07234789	-4.70203332	2.57583E-06	5.18105E-06
SYT1	8.756207475	-0.58498042	0.124408034	-4.702111266	2.57485E-06	5.17961E-06
ZNF512B	1217.04571	-0.178844003	0.03803121	-4.702558816	2.56921E-06	5.16879E-06
FAM183A	0.738897035	-0.664663889	0.141321268	-4.703212036	2.561E-06	5.15281E-06
TMEM52B	93.52590231	0.395055841	0.08399684	4.703222634	2.56087E-06	5.15281E-06
MICAL3	1422.072517	-0.244233897	0.051926457	-4.703457751	2.55792E-06	5.14766E-06
MAP10	128.8053193	-0.278343792	0.059175137	-4.703728712	2.55453E-06	5.14136E-06
ST6GALNAC2	45.11876247	0.317153693	0.067418874	4.704227092	2.54829E-06	5.12935E-06
C1orf116	173.7113574	-0.431893929	0.091785913	-4.705448973	2.53308E-06	5.09924E-06
IQSEC3	260.0609735	-0.42663554	0.090668087	-4.705465309	2.53288E-06	5.09924E-06
TGFB3	106.7966521	-0.186560179	0.039643813	-4.705909009	2.52737E-06	5.0888E-06
CBLIF	10.6124214	-0.499265447	0.106084117	-4.706316654	2.52233E-06	5.07917E-06
PDAP1	1278.551197	-0.114495415	0.024325664	-4.70677451	2.51667E-06	5.0683E-06
PRSS16	2.948341053	-0.570933968	0.121251854	-4.708661758	2.49348E-06	5.02212E-06
C4orf54	1.419750492	-0.604576319	0.128382636	-4.709175169	2.48721E-06	5.01E-06
LYPD8	38.26344771	-0.410415531	0.087126495	-4.710570909	2.47024E-06	4.97633E-06
CYP4F3	749.423407	-0.522773027	0.110967912	-4.711028792	2.46469E-06	4.96567E-06

CFD	10250.41714	0.455087454	0.096566415	4.712688714	2.4447E-06	4.92588E-06
RAPGEF5	63.63621657	-0.51059319	0.108323235	-4.713607293	2.43369E-06	4.90422E-06
SLC5A8	1.599796499	-0.7251327	0.153814726	-4.714325583	2.42513E-06	4.88746E-06
LY6G5B	230.1683833	0.197172176	0.041820372	4.714739861	2.4202E-06	4.87803E-06
HES6	402.8885917	-0.359768531	0.076302542	-4.715026819	2.41679E-06	4.87166E-06
NMT2	345.4135205	0.282006551	0.059806895	4.715284927	2.41373E-06	4.86599E-06
CALHM6	60.6990409	-0.347910168	0.073781876	-4.715387925	2.41251E-06	4.86403E-06
SYT9	6.920795541	-0.424798959	0.090077145	-4.715946074	2.4059E-06	4.85121E-06
CD300LB	430.8360354	0.427485309	0.090589549	4.718925249	2.37094E-06	4.78121E-06
FPR1	1071.800496	0.489324759	0.103691929	4.71902455	2.36978E-06	4.77937E-06
MTFR1	401.4703294	-0.1663073	0.035238387	-4.719492481	2.36434E-06	4.76888E-06
RPS28	16558.74724	-0.345240113	0.073151596	-4.719515782	2.36407E-06	4.76882E-06
SP100	4350.545609	0.137283609	0.029083769	4.720282644	2.35517E-06	4.75137E-06
CNOT6	1707.336036	-0.145108204	0.03073549	-4.721193815	2.34464E-06	4.73062E-06
CILK1	379.9537493	-0.212475284	0.04498387	-4.723365992	2.31973E-06	4.68083E-06
OVCH1	13.6553984	-0.697157639	0.147586358	-4.723726835	2.31561E-06	4.67301E-06
CES5A	0.718943128	-1.076887614	0.227963653	-4.723944376	2.31314E-06	4.66849E-06
PRUNE1	394.4099748	-0.155972091	0.033010933	-4.724861594	2.30272E-06	4.64795E-06
SRY	0.880760696	-0.948618423	0.200770548	-4.72488834	2.30242E-06	4.64782E-06
AVPI1	155.6044928	0.269428992	0.057022636	4.724948053	2.30174E-06	4.64693E-06
XKR9	3.949741778	-0.387241955	0.081954954	-4.725058517	2.30049E-06	4.64488E-06
C4orf50	6.56612386	-0.437054778	0.092490917	-4.725380542	2.29685E-06	4.63801E-06
SCMH1	1684.428619	-0.156413762	0.033098213	-4.72574647	2.29272E-06	4.63014E-06
PARN	1559.880622	-0.111169617	0.023523684	-4.725859105	2.29144E-06	4.62805E-06
ATG2B	2523.462886	-0.200138098	0.042348839	-4.725940628	2.29053E-06	4.62667E-06
ABCG1	1102.377252	0.299543982	0.063380631	4.726112326	2.28859E-06	4.62324E-06
C7orf50	457.427217	-0.239438639	0.050662256	-4.726174028	2.2879E-06	4.62231E-06
AKR1B10	0.739877442	-0.864234918	0.182842419	-4.7266653	2.28237E-06	4.61163E-06
SLC28A1	12.55229015	-0.546909927	0.11570469	-4.72677405	2.28115E-06	4.60963E-06
PHETA2	134.5095299	-0.231970114	0.049073887	-4.726956221	2.2791E-06	4.60598E-06
SKAP2	1448.534843	-0.294778096	0.06235301	-4.727568	2.27225E-06	4.5926E-06
RALBP1	2247.311382	-0.134133177	0.028369691	-4.728045082	2.26692E-06	4.5823E-06
SPIC	2.773551761	-0.661606572	0.139920686	-4.728440022	2.26251E-06	4.57387E-06
TMEM121B	222.5637955	0.471154316	0.09962832	4.729120369	2.25495E-06	4.55904E-06
NPSR1	0.638531349	-1.327825202	0.280761359	-4.729373043	2.25214E-06	4.55384E-06
XPO6	5801.293406	0.143517194	0.030342187	4.729955543	2.24569E-06	4.54126E-06
ZNF121	2409.319294	-0.168353424	0.035571691	-4.732792268	2.21452E-06	4.4787E-06

TYK2	5577.507271	-0.157192364	0.033210152	-4.73326247	2.20939E-06	4.46879E-06
PHF21B	5.173936323	-0.602031443	0.127187131	-4.733430488	2.20757E-06	4.46555E-06
AMPD3	1762.782804	-0.273929209	0.057859221	-4.734408872	2.19694E-06	4.44453E-06
ASB2	105.1973208	0.352227721	0.074381634	4.735412509	2.1861E-06	4.42304E-06
RAB31	1487.666815	0.373339218	0.078834033	4.735761985	2.18234E-06	4.41589E-06
SPEGNB	0.482858253	-0.708508803	0.149607669	-4.735778651	2.18216E-06	4.41589E-06
RNF157	60.69802768	0.356106525	0.075182931	4.736534232	2.17404E-06	4.40001E-06
CLEC17A	89.94488694	0.342748045	0.072351773	4.737244559	2.16644E-06	4.38507E-06
EHF	3.282647702	-0.553200694	0.116750059	-4.738333317	2.15483E-06	4.36204E-06
ZNF142	1350.469257	-0.129052307	0.027234644	-4.738534828	2.15269E-06	4.35815E-06
MED13L	22753.74076	0.157385432	0.033205553	4.739732318	2.14001E-06	4.33292E-06
ZNF319	745.0580181	0.168969774	0.035626742	4.742779358	2.10806E-06	4.26868E-06
FH	785.3165813	-0.183876673	0.038764886	-4.74338232	2.10179E-06	4.25643E-06
GOLGA6C	1.630903652	-0.6660061	0.140383669	-4.744185011	2.09347E-06	4.24002E-06
RAN	4368.712955	-0.161543932	0.034049409	-4.744397573	2.09128E-06	4.23601E-06
TBC1D10A	474.9112121	0.225511507	0.047529645	4.744649478	2.08868E-06	4.23118E-06
CCDC170	35.96147591	-0.45141939	0.095136131	-4.744983699	2.08523E-06	4.22464E-06
MDM2	3600.218381	0.156114523	0.032891107	4.746405234	2.07064E-06	4.1955E-06
TRPM5	54.57442219	-0.462586872	0.097452186	-4.746808567	2.06651E-06	4.18758E-06
LARP1B	755.5797062	-0.132070303	0.027820544	-4.747222125	2.06229E-06	4.17947E-06
AP1M1	2170.931853	-0.149404398	0.031470521	-4.747439555	2.06008E-06	4.17541E-06
C6orf52	12.98349416	-0.246219754	0.051854962	-4.748239051	2.05195E-06	4.15937E-06
SYN3	31.8069298	-0.367374107	0.077351684	-4.749400266	2.04021E-06	4.13599E-06
HOXD9	0.765674553	-0.949266422	0.199861236	-4.7496275	2.03792E-06	4.13177E-06
RPS19	30476.33059	-0.280480214	0.05904624	-4.75017911	2.03237E-06	4.12095E-06
LAP3	1539.957886	0.200718742	0.042254224	4.750264559	2.03151E-06	4.11963E-06
RABL6	2414.467923	-0.112783475	0.023741604	-4.750457202	2.02957E-06	4.11614E-06
NAP1L2	5.370218358	0.539413581	0.113541039	4.750824772	2.02589E-06	4.10909E-06
ANKRD34A	38.48116947	-0.249276823	0.052468298	-4.750998848	2.02414E-06	4.10597E-06
VSIG10L	71.8080315	-0.285270952	0.060035394	-4.751712797	2.01701E-06	4.09192E-06
SMG7	5379.106713	0.105865317	0.022276509	4.752329738	2.01086E-06	4.07988E-06
ID1	288.1181508	0.390591301	0.082187828	4.752422705	2.00994E-06	4.07842E-06
ZNF362	1094.948875	-0.167829379	0.035313052	-4.75261602	2.00801E-06	4.07495E-06
YIF1B	1031.647771	-0.243692534	0.051275055	-4.752652795	2.00765E-06	4.07463E-06
GPAT2	66.49127902	-0.413367505	0.086969275	-4.753029204	2.00391E-06	4.06747E-06
C1orf185	0.60553523	-0.674014323	0.141804431	-4.753125962	2.00295E-06	4.06594E-06
KCNRG	35.04813331	-0.182989847	0.038495287	-4.753564979	1.99861E-06	4.05754E-06

USF2	3199.892416	0.135059479	0.028410535	4.753852045	1.99577E-06	4.0522E-06
RAB11FIP4	842.5672604	0.285877824	0.060121947	4.754966145	1.9848E-06	4.03034E-06
REN	26.93547725	-0.832225101	0.175020147	-4.755024582	1.98422E-06	4.02959E-06
SPEM2	2.283099432	-0.535686556	0.112652322	-4.755219849	1.98231E-06	4.02612E-06
SLCO2B1	28.51000198	-0.449881867	0.094606908	-4.755275021	1.98176E-06	4.02543E-06
AGAP1	114.3841179	-0.429208928	0.090246465	-4.755963889	1.97502E-06	4.01215E-06
CACTIN	583.795024	-0.165429572	0.034780007	-4.756456027	1.97021E-06	4.0028E-06
SPARC	2124.4182	0.480893242	0.101098632	4.756674075	1.96809E-06	3.99889E-06
GPX8	4.319366036	-0.460744059	0.096861316	-4.756739608	1.96745E-06	3.99801E-06
PSMD7	1351.963121	0.147224306	0.030946861	4.757325992	1.96174E-06	3.98683E-06
WNT2	0.729837052	-0.884285447	0.18585477	-4.757937853	1.95581E-06	3.97518E-06
PRDM9	0.946258413	-1.371073716	0.288145597	-4.758267109	1.95262E-06	3.96912E-06
WSCD2	3.078142407	-0.638121978	0.134104624	-4.758389092	1.95144E-06	3.96713E-06
PTTG1	659.7400694	-0.245174687	0.051520573	-4.758772489	1.94774E-06	3.96002E-06
H2AC6	3792.212803	-0.234991649	0.049379836	-4.758858381	1.94691E-06	3.95874E-06
IZUMO4	79.31616262	-0.203226748	0.042704499	-4.75890717	1.94644E-06	3.9582E-06
ICA1	366.6085348	-0.370719939	0.07786618	-4.760987883	1.92648E-06	3.91801E-06
SKIDA1	770.8580463	-0.565127776	0.11869517	-4.761169094	1.92475E-06	3.9149E-06
ANKRD13C	914.0419931	0.142536515	0.029937104	4.761199149	1.92446E-06	3.91472E-06
SPEN	10106.62608	0.136729704	0.028710944	4.762285194	1.91413E-06	3.89411E-06
GH2	0.453406314	-1.015359591	0.213173327	-4.763070519	1.90669E-06	3.87938E-06
COL26A1	8.057636429	-0.508957058	0.106854251	-4.763096023	1.90645E-06	3.8793E-06
ANKRD9	759.8746649	-0.342936959	0.071986407	-4.763912699	1.89875E-06	3.86402E-06
FBXL13	143.7575708	-0.29210979	0.061317153	-4.763916397	1.89871E-06	3.86402E-06
RASA3	1774.607646	0.209025902	0.043863835	4.765335817	1.88539E-06	3.83765E-06
RAD51AP2	1.036391669	-1.158191124	0.24304021	-4.765430059	1.88451E-06	3.83625E-06
STON1-GTF2A1L	0.94835514	-0.590045364	0.123735793	-4.768590792	1.85519E-06	3.77696E-06
PIM2	2697.779651	0.231305114	0.048505283	4.768658135	1.85457E-06	3.77609E-06
ECPAS	5705.918006	0.146878051	0.030798918	4.768935397	1.85202E-06	3.77129E-06
CRB3	5.368893882	0.31074776	0.065160727	4.768942492	1.85196E-06	3.77129E-06
CELSR2	184.9770715	-0.358963634	0.075265101	-4.769323777	1.84845E-06	3.76481E-06
MRPL37	1653.720999	-0.167472442	0.03511445	-4.769331202	1.84839E-06	3.76481E-06
EIF2S1	1036.144091	-0.145118444	0.030427311	-4.769348314	1.84823E-06	3.76481E-06
SYNPR	0.5364841	-0.969968992	0.20336771	-4.769532934	1.84654E-06	3.76207E-06
ATP5MC2	7221.034828	-0.309228202	0.064833538	-4.769571593	1.84618E-06	3.76174E-06
CST7	3057.086145	0.437905894	0.091806404	4.769883977	1.84332E-06	3.75631E-06
MAP2K7	1385.416397	0.134327644	0.028161393	4.769921832	1.84297E-06	3.75599E-06

GAPDHS	4.466664704	-0.347415761	0.07283001	-4.7702281	1.84017E-06	3.75067E-06
C2orf72	2.953789425	-0.532295232	0.111578679	-4.770581956	1.83694E-06	3.74448E-06
OPLAH	199.5592372	-0.375163626	0.078638614	-4.770730425	1.83559E-06	3.74211E-06
KIF1A	24.12942658	-0.488685162	0.10243138	-4.770854034	1.83446E-06	3.74021E-06
AKNAD1	13.77033273	-0.315302006	0.066080535	-4.771480832	1.82876E-06	3.72897E-06
C1QTNF7	30.10846936	-0.425172082	0.089099112	-4.771900343	1.82496E-06	3.7216E-06
PAM16	243.6640718	-0.172574359	0.036162171	-4.772234416	1.82193E-06	3.71582E-06
MOB3B	418.54812	0.296728021	0.062176191	4.772373751	1.82067E-06	3.71363E-06
H2BC11	1099.971186	-0.317452587	0.066518703	-4.77238092	1.82061E-06	3.71363E-06
ATF1	383.6380536	-0.148442097	0.031101202	-4.772873336	1.81616E-06	3.7052E-06
NOG	156.6077688	-0.443815588	0.092986902	-4.772882816	1.81607E-06	3.7052E-06
TMPRSS11B	3.102324952	-0.696149905	0.145853842	-4.772928135	1.81567E-06	3.70497E-06
SAT2	759.9654148	0.205081087	0.042962059	4.773539515	1.81016E-06	3.69412E-06
PTN	0.939304905	-0.967809791	0.202723775	-4.774032001	1.80574E-06	3.68547E-06
GOLGA8N	663.9433321	-0.334117788	0.069982205	-4.774324934	1.80311E-06	3.6805E-06
POU2AF1	202.4702702	0.370011257	0.077500152	4.774329458	1.80307E-06	3.6805E-06
GYS2	0.533797142	-0.717607318	0.150292188	-4.774747956	1.79932E-06	3.67354E-06
EP300	15508.27758	0.17839676	0.03735456	4.775769279	1.79022E-06	3.65532E-06
TMEM217	84.53017247	-0.263100684	0.055079809	-4.776717476	1.7818E-06	3.63851E-06
CMBL	342.3851344	-0.34452285	0.072121513	-4.776977547	1.7795E-06	3.63419E-06
HYOU1	3514.067451	0.200970429	0.042070004	4.777048034	1.77887E-06	3.6333E-06
IFIT1B	613.4001864	-0.597613923	0.125069804	-4.778243075	1.76834E-06	3.61215E-06
OR2D2	1.212954785	-0.956041109	0.200067835	-4.778584771	1.76533E-06	3.60639E-06
ZIM3	1.121806455	-0.522954954	0.109430175	-4.778891697	1.76264E-06	3.60127E-06
GSC2	5.115679552	-0.45392252	0.094978787	-4.779198957	1.75995E-06	3.59615E-06
COL4A6	30.61585412	-0.447175909	0.093566684	-4.779221524	1.75975E-06	3.59612E-06
ZNF57	14.09522788	-0.250394771	0.05237778	-4.780553319	1.74813E-06	3.57275E-06
SERPINH1	376.602538	-0.344067172	0.07195797	-4.781501935	1.7399E-06	3.5563E-06
SDR42E2	43.18450174	-0.318105933	0.066525685	-4.781700993	1.73818E-06	3.55315E-06
H3C7	1153.901964	-0.490084469	0.102468804	-4.782767543	1.72898E-06	3.53471E-06
H2BC18	2048.865484	-0.34739855	0.072617197	-4.783970772	1.71866E-06	3.51397E-06
CTSF	257.7151329	-0.356174374	0.074443538	-4.784490138	1.71422E-06	3.50527E-06
TPM4	13344.24669	0.225410381	0.047103669	4.785410226	1.70638E-06	3.48961E-06
H1-5	2381.15648	-0.41791216	0.08731241	-4.786400444	1.69799E-06	3.47281E-06
REST	4076.908405	0.150855558	0.031516522	4.78655474	1.69669E-06	3.4705E-06
C15orf65	6.867398531	-0.341670656	0.071372008	-4.787180065	1.69141E-06	3.46007E-06
LRP12	681.0521392	0.356455434	0.074456841	4.787410096	1.68947E-06	3.45647E-06

ZMYM5	1144.563656	0.14949788	0.031225903	4.787623944	1.68768E-06	3.45315E-06
EBI3	29.13479305	0.474491219	0.099094045	4.788291956	1.68207E-06	3.44204E-06
OLIG2	30.54649121	0.585451084	0.122231564	4.789688228	1.67041E-06	3.41853E-06
PDPK1	1200.568917	0.137910692	0.028792877	4.789750289	1.66989E-06	3.41783E-06
CPA4	2.144143419	-0.459612478	0.095955205	-4.789865019	1.66894E-06	3.41624E-06
PARP11	519.0839051	-0.17135067	0.035760355	-4.791637899	1.65425E-06	3.38654E-06
ZIC5	1.044256168	-0.648685379	0.135368436	-4.791998775	1.65128E-06	3.3808E-06
MANEA	184.3479767	-0.210406766	0.043906362	-4.792170342	1.64987E-06	3.37827E-06
B3GALNT2	490.0106737	-0.180038164	0.037565253	-4.792678054	1.6457E-06	3.37008E-06
PHF24	36.08293271	-0.541355909	0.112919505	-4.794175384	1.63345E-06	3.34536E-06
HTR3B	3.312714133	-0.44884195	0.093610601	-4.794776936	1.62856E-06	3.33568E-06
SLC23A2	2041.620006	0.159819316	0.033329875	4.795077036	1.62612E-06	3.33104E-06
MAP4K2	2282.089615	-0.16031319	0.033431643	-4.795253091	1.6247E-06	3.32847E-06
SUCO	4420.522757	0.201671226	0.042054288	4.795497332	1.62272E-06	3.32476E-06
AC068946.1	6.580839991	-0.404164044	0.084276346	-4.795699654	1.62108E-06	3.32175E-06
CNTF	95.0116452	0.294222578	0.061328607	4.797476977	1.60677E-06	3.29277E-06
ZNF337	160.9826987	-0.183390617	0.038224183	-4.797764253	1.60446E-06	3.2884E-06
TRIM37	740.3861985	-0.136136016	0.028368903	-4.798776207	1.59638E-06	3.27217E-06
LST1	2385.193949	-0.41627574	0.086744806	-4.798854917	1.59575E-06	3.27123E-06
MED21	1616.786173	0.155747848	0.032453111	4.799165364	1.59328E-06	3.2665E-06
GJC3	2.968465687	-0.319472607	0.066566323	-4.79931281	1.59211E-06	3.26444E-06
KCNK16	14.14045594	-0.593719563	0.123707962	-4.799364184	1.5917E-06	3.26395E-06
MTX1	458.9245765	-0.16538319	0.034458164	-4.799535766	1.59034E-06	3.26149E-06
SFSWAP	3057.570367	0.107788192	0.022457754	4.799598105	1.58984E-06	3.26082E-06
AGBL3	68.22221262	-0.202283227	0.042139265	-4.800350122	1.58388E-06	3.24894E-06
INHBC	36.2930574	-0.300450584	0.062588542	-4.800408715	1.58342E-06	3.24833E-06
RBM20	22.27410378	-0.405145292	0.084388166	-4.800972817	1.57897E-06	3.23953E-06
GLIS3	104.9280723	0.569459789	0.118600935	4.801478079	1.57499E-06	3.2317E-06
NYAP2	6.098767778	-0.718473393	0.149634797	-4.801512796	1.57471E-06	3.23148E-06
ZSCAN1	6.913473653	-0.61545119	0.128168182	-4.801903083	1.57165E-06	3.22553E-06
TMEM68	244.5059518	-0.149737654	0.031175625	-4.803036176	1.56278E-06	3.20766E-06
GDF2	2.489778272	-0.789124909	0.164280807	-4.803512504	1.55906E-06	3.20037E-06
HOATZ	1.074894528	-0.528432581	0.109995598	-4.804124802	1.5543E-06	3.19093E-06
SNTA1	120.7289086	-0.291091213	0.060584934	-4.804679835	1.54999E-06	3.18242E-06
PRDX4	470.4026923	-0.333277064	0.069360207	-4.805018305	1.54737E-06	3.17738E-06
NPC1L1	34.40413884	-0.448474987	0.09332717	-4.805406448	1.54437E-06	3.17155E-06
CLGN	29.39782667	-0.541082946	0.112590967	-4.805740299	1.5418E-06	3.16659E-06

OBSCN	1988.807334	-0.308058766	0.064100151	-4.805897674	1.54059E-06	3.16444E-06
CCDC153	34.58888167	-0.236101633	0.049124813	-4.806158388	1.53858E-06	3.16065E-06
UPK3BL2	0.528084086	0.722575645	0.150334095	4.806465515	1.53622E-06	3.15613E-06
CHCHD7	586.1206377	-0.205210731	0.042693743	-4.806576249	1.53537E-06	3.15471E-06
ZNF684	149.1515159	0.191974511	0.039939075	4.806683911	1.53454E-06	3.15335E-06
GARRE1	559.6413096	0.243564219	0.050670038	4.806868648	1.53313E-06	3.15076E-06
HELLS	1305.312857	-0.224019263	0.046593862	-4.807913605	1.52514E-06	3.13467E-06
AHI1	838.8757568	-0.191431865	0.039815482	-4.807975615	1.52466E-06	3.13403E-06
GRIA2	0.768302765	-0.91846404	0.191024325	-4.808099911	1.52372E-06	3.13241E-06
ANKAR	236.271616	-0.17317442	0.036012914	-4.808675609	1.51934E-06	3.12373E-06
MAPK1IP1L	4861.960152	0.209183542	0.043498493	4.808983699	1.517E-06	3.11925E-06
PCDHGB2	119.9785905	-0.500130588	0.103977936	-4.809968399	1.50954E-06	3.10425E-06
CPO	2.09877997	-0.508425264	0.105693223	-4.810386601	1.50639E-06	3.09809E-06
TRAPPC1	918.385232	-0.274890432	0.057142284	-4.810630839	1.50455E-06	3.09463E-06
KCNK1	13.4369578	-0.844752405	0.175592793	-4.810860354	1.50282E-06	3.0914E-06
MRPL40	487.1684295	-0.174509465	0.036265073	-4.812053283	1.49388E-06	3.07333E-06
RBAK	485.6651841	0.17459761	0.036276111	4.813018944	1.48667E-06	3.05883E-06
CIDEA	1.743216256	-0.547782111	0.113800437	-4.81353257	1.48285E-06	3.05129E-06
BEX5	7.922982919	-0.569957308	0.118394923	-4.814035043	1.47913E-06	3.04395E-06
COPS9	532.5884749	-0.341666189	0.070959854	-4.814922402	1.47257E-06	3.03077E-06
PFKM	450.7503406	-0.269749502	0.056019705	-4.815260991	1.47008E-06	3.02595E-06
SLC25A37	14571.73192	-0.367192415	0.076248702	-4.815720196	1.4667E-06	3.01932E-06
TRIM22	4188.687352	0.202977072	0.042144638	4.816201587	1.46317E-06	3.01237E-06
SCYGR2	0.467033054	-1.015200192	0.210786739	-4.81624316	1.46286E-06	3.01206E-06
ABHD15	543.3120839	-0.256954889	0.053344973	-4.816852913	1.4584E-06	3.00319E-06
NENF	731.3730875	-0.279299347	0.057983006	-4.816917365	1.45793E-06	3.00253E-06
NDUFS2	1867.947014	-0.127566264	0.026481835	-4.817123282	1.45643E-06	2.99975E-06
SEC61A2	691.9650341	0.185346184	0.038475016	4.817312746	1.45505E-06	2.99722E-06
BOD1L1	4868.447134	0.173172091	0.035940715	4.818270649	1.44808E-06	2.98319E-06
SPOCK1	71.72438968	-0.578402481	0.120028399	-4.818880235	1.44366E-06	2.9744E-06
MDFIC	698.5126828	0.295903689	0.061402487	4.819083122	1.44219E-06	2.97169E-06
DPY19L1	476.0709305	-0.237122568	0.049187787	-4.820761073	1.43012E-06	2.94711E-06
LENEP	5.148676611	-0.387935986	0.08047104	-4.820814888	1.42973E-06	2.94663E-06
FDX2	1.340539215	-0.500040363	0.103714939	-4.821295446	1.42629E-06	2.93984E-06
TMEM131	3277.493407	-0.126720934	0.026279052	-4.822127304	1.42035E-06	2.92792E-06
TUBGCP2	3723.692219	0.125238504	0.025970088	4.822413601	1.41831E-06	2.92402E-06
VSIR	8893.151341	0.283929632	0.058876592	4.822453589	1.41803E-06	2.92374E-06

NUDC	1271.286832	-0.199695787	0.041399171	-4.823666372	1.40943E-06	2.90632E-06
LCOR	7070.431427	0.152264224	0.03155377	4.825547705	1.39619E-06	2.87932E-06
FAM149A	3.987522948	-0.537047916	0.111282907	-4.825969509	1.39324E-06	2.87354E-06
B3GAT1	17.79040019	0.449982106	0.093239148	4.826107033	1.39228E-06	2.87186E-06
SH3TC1	1768.518284	0.28208205	0.05843909	4.826941193	1.38646E-06	2.86016E-06
TMPRSS3	13.90989679	-0.436934886	0.090514545	-4.82723399	1.38442E-06	2.85626E-06
MED4	738.6023244	0.169072166	0.035023453	4.827398566	1.38328E-06	2.8542E-06
TNFSF13	152.7611135	-0.270445758	0.056020649	-4.827608432	1.38182E-06	2.8515E-06
NMU	11.43751823	-0.602751338	0.124818884	-4.829007598	1.37215E-06	2.83184E-06
ZNF527	159.5747175	-0.197915226	0.040983239	-4.829174758	1.371E-06	2.82976E-06
RPS6KA4	1662.322276	-0.229912552	0.047608002	-4.829283748	1.37025E-06	2.82851E-06
IGFALS	51.17575348	-0.272417457	0.056407311	-4.829470732	1.36896E-06	2.82615E-06
SEBOX	2.808410391	0.381127193	0.078916151	4.829520803	1.36862E-06	2.82574E-06
CGAS	932.2363795	0.192272868	0.039796222	4.831435215	1.35552E-06	2.79899E-06
GTF3C4	1599.237594	0.15941451	0.032994917	4.831486964	1.35517E-06	2.79856E-06
SCTR	1.161695344	-0.604656228	0.125137345	-4.83194068	1.35209E-06	2.79248E-06
COX7A2	2062.86024	-0.362424662	0.074996964	-4.832524443	1.34813E-06	2.7846E-06
RGR	1.21012544	-0.755518333	0.156334275	-4.832710745	1.34686E-06	2.78229E-06
TXK	405.438351	-0.295229614	0.06107883	-4.833583342	1.34097E-06	2.77041E-06
SF3B2	5097.810349	0.126977628	0.026269417	4.833667563	1.3404E-06	2.76953E-06
TMEM94	2157.071014	-0.182899217	0.037827351	-4.83510512	1.33075E-06	2.74988E-06
XRCC1	622.0180606	-0.119192821	0.024645842	-4.836224229	1.32329E-06	2.73474E-06
TRAK1	3431.672497	0.115545991	0.023891639	4.83625225	1.3231E-06	2.73464E-06
IGFBP3	38.20131167	-0.566460147	0.117101671	-4.837336157	1.31591E-06	2.72006E-06
RIMBP2	4.41031541	-0.786880423	0.162658651	-4.837618039	1.31404E-06	2.71649E-06
TMCO2	7.00668029	-0.461095036	0.095311321	-4.83777823	1.31299E-06	2.71459E-06
P2RX3	9.39612722	-0.492478046	0.101793697	-4.838001389	1.31151E-06	2.71183E-06
ZNF418	160.8608457	-0.396142744	0.081856463	-4.83948037	1.30179E-06	2.69202E-06
ABL2	1676.108976	0.165401162	0.0341772	4.83951767	1.30155E-06	2.6918E-06
CFAP77	4.455527659	-0.313247131	0.064722925	-4.839817298	1.29959E-06	2.68803E-06
FGF10	3.869711226	-1.070305189	0.221134837	-4.840056874	1.29802E-06	2.68507E-06
MINDY4B	0.538078179	-0.957536116	0.19781552	-4.840551021	1.2948E-06	2.67869E-06
ADAM23	162.0563752	-0.734023909	0.151636831	-4.840670333	1.29402E-06	2.67736E-06
GPR141	266.4682893	-0.377919441	0.078069456	-4.840810477	1.29311E-06	2.67576E-06
HNF4G	2.818120733	-1.158831422	0.239334594	-4.841888523	1.28611E-06	2.66156E-06
DTX2	360.6880514	-0.186485274	0.038500767	-4.843676871	1.27458E-06	2.63798E-06
GADL1	6.369337727	-0.5544568	0.114448356	-4.844602572	1.26865E-06	2.62599E-06

SNAP29	1034.322944	0.156488666	0.032297355	4.845247057	1.26454E-06	2.61776E-06
MAP3K20	1256.271605	0.351250749	0.072473381	4.846617387	1.25584E-06	2.60002E-06
RNF103	1639.014767	0.221869379	0.045761116	4.848425896	1.24445E-06	2.57671E-06
GPC6	32.37148555	-0.940549539	0.193971636	-4.848902432	1.24146E-06	2.5708E-06
ZFP1	294.0001391	0.184416992	0.038031072	4.84911373	1.24014E-06	2.56833E-06
H4C11	129.1437427	-0.334907903	0.069058583	-4.849620241	1.23698E-06	2.56206E-06
CAPNS2	19.32708366	-0.351273687	0.072429606	-4.849863263	1.23547E-06	2.55919E-06
EDDM3B	0.51995276	-0.764020955	0.157526753	-4.850102844	1.23397E-06	2.55637E-06
MZT1	479.7158772	0.218935542	0.045127337	4.851505885	1.22528E-06	2.53862E-06
GPN3	349.4365046	-0.213122952	0.043928887	-4.851544528	1.22504E-06	2.53839E-06
CEP170	3649.569944	0.163294313	0.033652256	4.85240321	1.21974E-06	2.52769E-06
TMEM164	2960.510475	-0.176187761	0.036306439	-4.852796599	1.21733E-06	2.52295E-06
ATP6V1C1	774.4267814	-0.201911137	0.041600811	-4.853538499	1.21278E-06	2.51379E-06
RPS15	22399.20646	-0.345569967	0.071177305	-4.855058289	1.20351E-06	2.49485E-06
KRTAP10-11	0.727685193	-1.128861834	0.232502539	-4.855266693	1.20225E-06	2.49249E-06
LARS2	1131.331771	-0.138194749	0.028454589	-4.85667708	1.19372E-06	2.47507E-06
POLR2C	1200.024112	0.1220901	0.025133626	4.857639772	1.18793E-06	2.46333E-06
REPS2	481.7116826	-0.214320003	0.044119759	-4.857687512	1.18765E-06	2.463E-06
OSBPL10	288.966637	-0.347793757	0.071580915	-4.858749781	1.18129E-06	2.45008E-06
KRT2	4.085864438	-0.492594896	0.101369045	-4.859421315	1.17729E-06	2.44205E-06
CPA3	1576.210043	0.570320691	0.11736106	4.859539369	1.17659E-06	2.44085E-06
FAM24A	0.591377727	-0.73299908	0.150831118	-4.859733784	1.17544E-06	2.43871E-06
CD22	492.0771149	0.329248026	0.067734526	4.860859709	1.16877E-06	2.42514E-06
TAZ	1178.785434	-0.147238496	0.030284951	-4.861770944	1.1634E-06	2.41426E-06
OR2C3	5.021015376	-0.635183438	0.130632072	-4.862385078	1.1598E-06	2.40703E-06
RAE1	1551.964283	0.130736172	0.026881978	4.863339092	1.15422E-06	2.39571E-06
ACOT7	456.0692853	-0.26382606	0.054237931	-4.864235321	1.149E-06	2.38513E-06
TMEM47	2.108883364	-0.783331837	0.161035609	-4.864339266	1.1484E-06	2.38413E-06
TMEM236	39.18594303	-0.396798634	0.081571185	-4.864446136	1.14778E-06	2.3831E-06
GOLGA8B	879.0311122	-0.368070062	0.075662184	-4.864650246	1.14659E-06	2.38089E-06
NR1H4	0.485495858	-0.888110812	0.182524399	-4.865710092	1.14047E-06	2.36842E-06
RENBP	381.6515791	-0.215048497	0.044184192	-4.86709129	1.13253E-06	2.35218E-06
ATP5PF	724.3615439	-0.329218183	0.067634268	-4.867623919	1.12948E-06	2.3461E-06
AC012651.1	13.81328633	-0.389445714	0.080005733	-4.867722598	1.12892E-06	2.34518E-06
RAC3	98.83321576	-0.298170832	0.061254038	-4.867774332	1.12862E-06	2.34481E-06
WDR20	1051.961415	0.171154355	0.035158892	4.868024708	1.12719E-06	2.3421E-06
SCPEP1	2883.804322	0.347856633	0.07144771	4.868688326	1.12341E-06	2.33449E-06

PCCA	445.6708578	-0.356106519	0.073139591	-4.868861241	1.12243E-06	2.3327E-06
FADS3	615.4960965	0.259743849	0.053347899	4.868867419	1.1224E-06	2.3327E-06
FBXO9	1969.290257	-0.197393168	0.040540724	-4.869009452	1.12159E-06	2.33145E-06
NPIPB13	88.73660726	-0.256016613	0.052580283	-4.869061157	1.1213E-06	2.33108E-06
SUPT16H	3048.693748	-0.125856849	0.025846778	-4.86934382	1.11969E-06	2.328E-06
GGCX	1448.343159	-0.185057029	0.03800243	-4.869610395	1.11818E-06	2.32511E-06
ADAMTS4	68.00035338	-0.394141276	0.080932728	-4.869986281	1.11606E-06	2.32094E-06
POLG	2959.127538	-0.15280756	0.031370389	-4.871076347	1.10992E-06	2.30841E-06
ABCC1	7251.517592	-0.213256063	0.043778736	-4.871224716	1.10909E-06	2.30692E-06
TAP2	1030.798097	0.187914402	0.038575433	4.871349117	1.10839E-06	2.30572E-06
CTF1	41.26226926	-0.303970261	0.062380354	-4.872852498	1.09998E-06	2.28848E-06
USF1	2200.264616	0.149066857	0.030589155	4.873192985	1.09809E-06	2.28478E-06
PLEKHH2	28.09690902	-0.418520501	0.085880787	-4.873272784	1.09764E-06	2.2841E-06
SMKR1	7.680956286	-0.398700494	0.081804997	-4.873791433	1.09477E-06	2.27835E-06
TNFAIP1	491.6210617	0.144370771	0.02962119	4.873901829	1.09415E-06	2.27732E-06
LY6K	7.834825681	0.669992398	0.137442265	4.87471883	1.08964E-06	2.26815E-06
SSRP1	2650.704035	-0.15650362	0.032105123	-4.87472416	1.08961E-06	2.26815E-06
NEDD4	696.1599654	-0.290658348	0.059622011	-4.875017534	1.08799E-06	2.2652E-06
TMC6	4076.521154	-0.161301239	0.033085083	-4.875346336	1.08618E-06	2.26168E-06
PCDHA2	1.634604389	-0.871970085	0.178843285	-4.875609857	1.08473E-06	2.2589E-06
SRBD1	794.7179016	-0.190002794	0.038965592	-4.876168555	1.08166E-06	2.25275E-06
PLN	95.5094284	0.358538629	0.073517716	4.876901057	1.07765E-06	2.24465E-06
ITSN2	5520.690119	0.162598122	0.033338905	4.87712849	1.07641E-06	2.2423E-06
DPEP1	32.89021388	-0.517356371	0.106061036	-4.877911736	1.07215E-06	2.23365E-06
ARIH1	4876.661951	0.188910659	0.038725164	4.878240362	1.07036E-06	2.23017E-06
C11orf58	3325.538243	0.15165234	0.031081889	4.879122352	1.06559E-06	2.22046E-06
SLC25A14	178.120114	-0.136737799	0.028021566	-4.879734323	1.06229E-06	2.21382E-06
CCDC188	64.86125891	-0.284357159	0.058253589	-4.881367235	1.05353E-06	2.1958E-06
PIKFYVE	7610.988749	0.164155323	0.033612212	4.883800007	1.04061E-06	2.16909E-06
AFF2	2144.949183	0.415123205	0.084992991	4.88420516	1.03847E-06	2.16487E-06
RFNG	752.4700465	-0.154568351	0.031646451	-4.884223921	1.03837E-06	2.16487E-06
AL136295.5	24.97523037	-0.255033789	0.052198256	-4.885868028	1.02974E-06	2.14713E-06
P2RX5	316.3386565	-0.305860624	0.062600757	-4.885893378	1.02961E-06	2.14708E-06
CDRT4	32.81681072	-0.397721046	0.081401496	-4.885918152	1.02948E-06	2.14704E-06
SCRT1	12.18791365	-0.403577855	0.082583412	-4.886911826	1.0243E-06	2.13647E-06
EPHA2	38.56248461	-0.544575322	0.111411302	-4.887971975	1.0188E-06	2.12522E-06
ANXA9	186.4667941	-0.204450115	0.041824699	-4.888262683	1.0173E-06	2.12232E-06

SOX4	11468.00595	-0.285718067	0.058445986	-4.888583219	1.01564E-06	2.11909E-06
ZNF615	277.9613459	0.211403487	0.043242456	4.888794645	1.01455E-06	2.11704E-06
SYNRG	1792.239021	-0.185458611	0.037932899	-4.88912302	1.01286E-06	2.11374E-06
PCDHA8	0.803505822	-1.018408899	0.208300438	-4.889134706	1.0128E-06	2.11374E-06
ABHD8	268.573981	-0.143575666	0.029363945	-4.889522427	1.01081E-06	2.1099E-06
KATNB1	1230.682726	-0.190250104	0.03890965	-4.889535178	1.01074E-06	2.1099E-06
BACH2	798.4072044	0.389744776	0.0797031	4.889957564	1.00858E-06	2.10569E-06
FRG2	0.498139001	-1.096845892	0.224271234	-4.890711451	1.00472E-06	2.09787E-06
ADORA1	5.896690425	-0.528067972	0.107962308	-4.891225299	1.0021E-06	2.09262E-06
PAMR1	1.314854551	-0.743076474	0.151902189	-4.891808874	9.99134E-07	2.08665E-06
KBTBD4	285.5269017	0.158694035	0.032437038	4.892371277	9.96283E-07	2.08091E-06
RUFY4	67.01346972	-0.56525216	0.115535531	-4.892453029	9.95869E-07	2.08027E-06
CCIN	75.03752106	0.34028453	0.06954727	4.89285242	9.93849E-07	2.07627E-06
ZNF318	1841.085324	0.197301505	0.040317825	4.893654532	9.89805E-07	2.06805E-06
NCR3LG1	326.577511	0.295388974	0.060360194	4.893771132	9.89219E-07	2.06704E-06
GNG13	3.950622242	-0.35779307	0.073108998	-4.893967628	9.88231E-07	2.0652E-06
PPIG	2289.921872	0.175888845	0.035936602	4.894420558	9.85958E-07	2.06067E-06
TLR7	139.9706674	0.411377238	0.084039351	4.895054915	9.82783E-07	2.05425E-06
CCDC87	9.117026374	-0.320689716	0.065511342	-4.895178564	9.82165E-07	2.05318E-06
NOCT	2293.389339	0.242114522	0.049451734	4.895976347	9.78188E-07	2.04508E-06
PARP6	2724.579854	0.128038135	0.026149396	4.896408863	9.76039E-07	2.04081E-06
MEOX2	0.574954893	-0.892150806	0.182185511	-4.896936106	9.73425E-07	2.03556E-06
SLC9B1	41.72475495	-0.206142985	0.042090921	-4.897564187	9.70319E-07	2.02928E-06
TIRAP	152.1546126	-0.16430542	0.033545823	-4.897939754	9.68467E-07	2.02563E-06
IRGM	136.4588324	-0.297811446	0.0607799	-4.89983442	9.59175E-07	2.0064E-06
POLR2G	1199.68437	-0.20584771	0.04200872	-4.900118596	9.57788E-07	2.00372E-06
RAPGEF2	4313.629608	-0.25985773	0.05302984	-4.90021715	9.57308E-07	2.00293E-06
BBOX1	1.318697056	-1.218332829	0.248620941	-4.900362871	9.56598E-07	2.00166E-06
MAPK8IP1	176.1902973	0.281990555	0.057539062	4.900854187	9.54209E-07	1.99687E-06
SRSF7	5096.054218	-0.175458185	0.035800978	-4.900932756	9.53827E-07	1.99628E-06
KLHL40	2.771577319	-0.587864761	0.119940956	-4.90128459	9.5212E-07	1.99293E-06
ATF6	3279.848036	0.130217477	0.026567396	4.901401521	9.51553E-07	1.99195E-06
SNURF	23.24484204	0.368452379	0.075161528	4.90214061	9.47979E-07	1.98468E-06
NR1D1	184.5324469	0.270236791	0.055123349	4.902401551	9.46721E-07	1.98226E-06
DPP4	73.16831951	0.404334357	0.082476603	4.902412816	9.46666E-07	1.98226E-06
CXorf56	604.8624933	0.116135802	0.023687351	4.902861477	9.44506E-07	1.97805E-06
AARD	1.567391215	-0.583427419	0.118958645	-4.904455833	9.36867E-07	1.96226E-06

EYA1	14.31495745	-0.525508158	0.10713935	-4.904903377	9.34733E-07	1.958E-06
RFX4	5.218378847	-0.397382446	0.081010852	-4.905298964	9.32851E-07	1.95427E-06
CEACAM21	200.6858253	-0.258546376	0.052703934	-4.905637136	9.31245E-07	1.95111E-06
SPDYA	177.4095547	0.231865934	0.047240258	4.908227512	9.19032E-07	1.92573E-06
H2AC21	981.5106219	-0.42316093	0.086214355	-4.908242153	9.18963E-07	1.92573E-06
AC006030.1	4.533035032	-0.25668214	0.052294976	-4.90835179	9.1845E-07	1.92492E-06
KIR2DL4	12.11939323	0.468666005	0.095466706	4.909208919	9.14445E-07	1.91673E-06
ACAD11	52.00063118	-0.201222471	0.040978488	-4.910441574	9.08715E-07	1.90492E-06
LHX2	2.565935978	-0.594388128	0.121029297	-4.911109475	9.05625E-07	1.89865E-06
FXYD6	61.8109847	0.448134773	0.091236758	4.911778795	9.02538E-07	1.89238E-06
APOA2	130.7975564	0.39921319	0.081270494	4.912154091	9.00812E-07	1.88896E-06
SAPCD2	162.071423	-0.355725087	0.072391861	-4.913882319	8.92904E-07	1.87258E-06
UGT1A7	0.720260011	-0.888349354	0.180778908	-4.914009952	8.92322E-07	1.87156E-06
MRPL46	63.1164842	-0.214891791	0.043727833	-4.91430232	8.90992E-07	1.86897E-06
TMOD2	454.8630274	-0.318407779	0.06478912	-4.914525461	8.89978E-07	1.86705E-06
SCYL2	2432.640569	0.137970294	0.028072809	4.914730554	8.89046E-07	1.86529E-06
GABRP	1.289590362	-0.572538055	0.116493077	-4.914781821	8.88814E-07	1.865E-06
NSD1	6284.043959	-0.146337929	0.029767075	-4.916100418	8.82851E-07	1.85269E-06
UBR3	1735.035661	-0.181017835	0.036807683	-4.917936168	8.74614E-07	1.8356E-06
DNAAF6	0.540350328	-0.769571643	0.156445956	-4.919089385	8.69478E-07	1.82502E-06
CBWD2	553.127488	-0.165999496	0.033740445	-4.919896448	8.659E-07	1.8177E-06
CPTP	266.0984193	-0.187091873	0.038019026	-4.921006473	8.61003E-07	1.80762E-06
RP1L1	12.70799983	-0.371557814	0.075495096	-4.921615235	8.58328E-07	1.80219E-06
NTS	0.941217394	-1.116025749	0.22675155	-4.921799877	8.57519E-07	1.80069E-06
EFNA4	58.91205978	-0.231296709	0.046993581	-4.921878809	8.57173E-07	1.80016E-06
MZT2B	539.7085999	-0.265056088	0.053850814	-4.922044195	8.56449E-07	1.79883E-06
CTSZ	2640.151737	0.31228128	0.063443284	4.922211791	8.55715E-07	1.79748E-06
NEGR1	550.3365431	-0.565490209	0.114876076	-4.922610759	8.53972E-07	1.79401E-06
ASPHD2	152.9524625	0.214961246	0.043664728	4.922995165	8.52296E-07	1.79068E-06
UBE2Q1	1477.265905	0.145503842	0.02955401	4.923319772	8.50882E-07	1.7879E-06
H2BC5	4381.140355	-0.301144517	0.061161615	-4.923750261	8.49012E-07	1.78416E-06
CCDC85A	2.622931664	-0.828695618	0.168305062	-4.923771206	8.48921E-07	1.78416E-06
OR52A5	12.85989892	-0.89213016	0.181160326	-4.924533857	8.45617E-07	1.77741E-06
MAB21L3	28.01795349	-0.652154957	0.132421476	-4.924842845	8.44282E-07	1.7748E-06
EIF1B	2994.064162	0.243206464	0.049368592	4.92633986	8.37842E-07	1.76145E-06
PNPLA6	2427.481364	-0.18234864	0.037013457	-4.926549753	8.36943E-07	1.75975E-06
ZNF229	28.40126821	-0.546562755	0.110939375	-4.926679605	8.36387E-07	1.75877E-06

ZBTB37	1596.28564	-0.217953979	0.044235663	-4.927110024	8.34548E-07	1.75509E-06
PLA2G4B	20.48662138	-0.259048976	0.052572491	-4.927462438	8.33044E-07	1.75211E-06
PCDHA5	2.498357278	-0.637839679	0.129425976	-4.928219978	8.29822E-07	1.74552E-06
ARID4B	5071.550707	0.186396113	0.037810502	4.929744488	8.23372E-07	1.73214E-06
CAP1	9263.469546	0.155519628	0.031545002	4.930087732	8.21927E-07	1.72929E-06
SLCO5A1	546.1990773	0.590783442	0.119816233	4.930746249	8.19161E-07	1.72365E-06
DSTYK	2234.985687	0.175622417	0.03560666	4.932291285	8.12706E-07	1.71026E-06
NHSL2	629.3919758	-0.458854933	0.093030516	-4.932305582	8.12646E-07	1.71026E-06
KCNJ14	157.0846008	-0.228402446	0.046299007	-4.933203968	8.08916E-07	1.70265E-06
ARHGAP15	2813.247708	-0.17070679	0.034599853	-4.933743259	8.06685E-07	1.69813E-06
LPIN2	2580.121858	-0.25146347	0.050958438	-4.934677714	8.02832E-07	1.6902E-06
MTERF2	136.7740067	-0.263985883	0.053488385	-4.93538702	7.9992E-07	1.68425E-06
GOLGA8K	54.83685883	-0.302906786	0.061373712	-4.935448317	7.99669E-07	1.68391E-06
SMIM32	6.942157533	-0.496912492	0.100658387	-4.936622834	7.9487E-07	1.67398E-06
VMA21	2319.371535	0.131521463	0.026640484	4.936902134	7.93733E-07	1.67177E-06
PACSIN3	48.05682261	-0.270704993	0.05481772	-4.938275335	7.88165E-07	1.66022E-06
SLC12A1	13.01717646	-0.516771615	0.10464564	-4.938300496	7.88063E-07	1.66018E-06
GCSAML	396.2928485	-0.500289564	0.101281195	-4.939609597	7.82791E-07	1.64925E-06
GOLGA6L10	16.98631636	-0.32239173	0.065246565	-4.941129562	7.76713E-07	1.63662E-06
UBL5	1825.197904	-0.279031836	0.056467266	-4.941479502	7.7532E-07	1.63386E-06
ZCCHC10	383.8179999	0.214269808	0.043357197	4.941966352	7.73386E-07	1.62996E-06
TFAP2A	6.436344706	-0.533429297	0.107930299	-4.942349831	7.71866E-07	1.62694E-06
XRCC4	137.1716503	-0.232351512	0.047012244	-4.942361636	7.71819E-07	1.62694E-06
NDUFB11	1665.33199	-0.337377947	0.068253267	-4.943030005	7.69176E-07	1.62162E-06
LRRC36	21.54708788	-0.353062837	0.071393107	-4.945335051	7.6013E-07	1.60272E-06
NUP205	2428.943244	-0.153458856	0.031024411	-4.946390705	7.56021E-07	1.59423E-06
GALNTL5	4.318085168	-0.695681904	0.140633435	-4.946774588	7.54533E-07	1.59126E-06
PPP1R10	2627.357098	-0.221218581	0.044705559	-4.9483462	7.48467E-07	1.57864E-06
NFKBIL1	180.7471138	-0.18194714	0.036768517	-4.948449232	7.48071E-07	1.57797E-06
SNRPA	1368.962009	-0.221378543	0.044733984	-4.948777734	7.4681E-07	1.57548E-06
C11orf1	369.3025015	-0.323865683	0.065442384	-4.948867473	7.46465E-07	1.57492E-06
PROCR	57.74511612	-0.266008746	0.053743729	-4.949577358	7.43748E-07	1.56936E-06
ECI2	229.7275755	-0.196130916	0.039624997	-4.949676517	7.43369E-07	1.56873E-06
SNRPD1	1226.87002	-0.255354939	0.051589082	-4.949786406	7.4295E-07	1.56801E-06
MTERF3	220.8792496	-0.176139417	0.035580317	-4.950473562	7.40331E-07	1.56266E-06
STATH	0.48309255	-1.334428558	0.269547517	-4.950624565	7.39757E-07	1.56161E-06
BFSP2	3.211317459	-0.489545567	0.098872382	-4.951287275	7.37242E-07	1.55647E-06

SHISA9	8.070247614	-0.374164058	0.075563642	-4.951641414	7.35901E-07	1.55381E-06
ZNF611	601.7782598	-0.158380878	0.031984919	-4.951736049	7.35543E-07	1.55322E-06
PCDHGC5	40.87961692	-0.433855514	0.087595009	-4.952970712	7.3089E-07	1.54356E-06
DGKZ	6777.034652	-0.163148199	0.032939102	-4.953025184	7.30685E-07	1.54329E-06
G3BP1	4786.524887	-0.127059843	0.025651276	-4.953353738	7.29452E-07	1.54086E-06
GCNT7	448.1950183	0.270146915	0.054537015	4.953459823	7.29054E-07	1.54018E-06
COL23A1	866.9767144	-0.403167998	0.081382193	-4.954007573	7.27004E-07	1.53602E-06
GPR84	347.2253688	0.417039349	0.084166726	4.954919485	7.23602E-07	1.52899E-06
TCEAL1	145.3983595	-0.243603615	0.049163461	-4.954972838	7.23404E-07	1.52874E-06
JAK3	4592.363279	0.23161498	0.046740569	4.95533074	7.22074E-07	1.52609E-06
MCAT	337.6133549	-0.152004448	0.030674748	-4.955360925	7.21961E-07	1.52602E-06
MEAK7	153.5213984	-0.260045561	0.052472116	-4.955880972	7.20033E-07	1.52211E-06
PCDHA12	0.636654361	-0.866570259	0.174856909	-4.955882303	7.20028E-07	1.52211E-06
CDC42EP1	122.2354117	0.552376437	0.111456421	4.955985784	7.19645E-07	1.52162E-06
PLA2G12B	3.378551627	-0.585641618	0.118166079	-4.95608912	7.19262E-07	1.52097E-06
GCK	4.033595877	-0.431947683	0.087148542	-4.956453326	7.17916E-07	1.51829E-06
UPP2	11.22925566	-0.515563174	0.104017882	-4.95648598	7.17795E-07	1.5182E-06
SLC35F1	4.475141213	-0.522437958	0.105404771	-4.95649253	7.17771E-07	1.5182E-06
CNBD2	14.67100022	-0.37630852	0.075909825	-4.957309791	7.1476E-07	1.5121E-06
PUDP	157.2751863	-0.288475631	0.058185439	-4.95786635	7.12716E-07	1.50794E-06
EDF1	2355.804142	-0.261172671	0.052667636	-4.95888353	7.08995E-07	1.50023E-06
CRTAC1	2.166729115	-0.562420031	0.113389984	-4.960050353	7.04749E-07	1.49141E-06
LIFR	43.19419788	-0.594763716	0.119908536	-4.960144943	7.04406E-07	1.49085E-06
TMEM82	3.166966685	-0.59494354	0.119916336	-4.961321871	7.00151E-07	1.482E-06
DMAC2	1004.112624	-0.171243715	0.034511902	-4.961874217	6.98162E-07	1.47795E-06
VAX2	1.171173278	-0.661095525	0.13321672	-4.962556708	6.95712E-07	1.47292E-06
TNIP3	8.551611059	0.453167461	0.091316864	4.962582394	6.9562E-07	1.47289E-06
ELFN2	21.00705951	-0.488791621	0.098484718	-4.963121477	6.93692E-07	1.46896E-06
GPR85	39.34804298	-0.511433386	0.103037424	-4.963569228	6.92094E-07	1.46574E-06
ASB13	301.0479062	-0.260077979	0.052391974	-4.964080529	6.90273E-07	1.46204E-06
ATF7	833.6905176	-0.137344061	0.027666029	-4.964357537	6.89289E-07	1.46011E-06
EIF2B1	1237.539635	-0.117200786	0.023606262	-4.964817734	6.87656E-07	1.45681E-06
OR2V2	2.179200729	-0.599779926	0.120769645	-4.966313556	6.82376E-07	1.44578E-06
DUSP26	3.676177476	-0.538583691	0.108443471	-4.966492529	6.81747E-07	1.44461E-06
SPATA12	26.39335612	0.431942284	0.086963846	4.966917887	6.80254E-07	1.4416E-06
NHLRC2	1529.881125	-0.164630083	0.033143703	-4.967160238	6.79404E-07	1.43995E-06
PLA2G4A	438.3580956	-0.317214281	0.063861607	-4.967214221	6.79215E-07	1.43971E-06

IDH2	4476.766412	-0.175754634	0.035381269	-4.967448506	6.78396E-07	1.43813E-06
SYNGR4	6.456188659	-0.401545377	0.080833892	-4.967537318	6.78085E-07	1.43763E-06
HTR3C	0.564603724	-0.864168197	0.173943425	-4.968099243	6.76124E-07	1.43362E-06
GCH1	783.3222808	0.290016406	0.058367545	4.968795726	6.737E-07	1.42864E-06
SMIM7	659.183212	-0.156309417	0.031457312	-4.968937531	6.73208E-07	1.42775E-06
FAM177A1	915.8401054	0.155888356	0.031371445	4.969116221	6.72587E-07	1.42659E-06
CRYBB2	1.750033579	-0.667230402	0.134270989	-4.969281941	6.72013E-07	1.42552E-06
HES7	4.961438918	-0.454803613	0.091521698	-4.96935288	6.71767E-07	1.42516E-06
TPMT	507.0146184	0.166330201	0.033466451	4.970057996	6.69329E-07	1.42014E-06
EXOC3	2360.776162	-0.124086345	0.024965442	-4.970324366	6.6841E-07	1.41834E-06
MARK4	1691.004807	0.165247499	0.033242634	4.970950903	6.66253E-07	1.41392E-06
NRN1	6.154464296	-0.557681794	0.112151048	-4.972595484	6.60624E-07	1.40212E-06
NEK3	351.9796485	0.220258749	0.044293517	4.972708496	6.60239E-07	1.40146E-06
ABHD17B	1453.964543	0.174829141	0.035153334	4.973330354	6.58124E-07	1.39712E-06
ZNF350	724.7618192	0.162134793	0.032596154	4.974046662	6.55695E-07	1.39212E-06
PRAF2	76.9650469	-0.221282205	0.044481518	-4.974699895	6.53488E-07	1.38758E-06
TEX13D	1.174957277	-0.664117112	0.133497303	-4.974760514	6.53284E-07	1.3873E-06
TVP23A	192.2828175	0.244603087	0.049163208	4.975328065	6.51372E-07	1.38339E-06
SMIM2	2.77824214	-0.770261678	0.154813196	-4.975426506	6.51041E-07	1.38284E-06
SENP5	3627.355849	0.177001297	0.035572498	4.975790447	6.49819E-07	1.38039E-06
TACC3	3351.512099	-0.140539061	0.028240772	-4.976459548	6.47578E-07	1.37578E-06
RNF103-CHMP3	37.10687606	-0.236094799	0.047438989	-4.976809205	6.4641E-07	1.37345E-06
ERAP2	2090.97959	0.241893864	0.048602809	4.976952319	6.45932E-07	1.37258E-06
TRAF4	1381.473319	0.26854905	0.053918998	4.980601608	6.33869E-07	1.34709E-06
GCKR	9.789068898	-0.528994159	0.106196025	-4.981299055	6.31588E-07	1.34239E-06
OR2A42	10.26656479	-0.497634351	0.099891959	-4.981725826	6.30197E-07	1.33958E-06
FAM153A	29.34424879	0.403609978	0.081017758	4.98174706	6.30128E-07	1.33958E-06
SUGP2	7677.441995	-0.158778093	0.031871108	-4.981881821	6.29689E-07	1.33879E-06
USP41	17.78766963	-0.407006749	0.081696669	-4.981925892	6.29545E-07	1.33863E-06
MUC19	202.4509093	-0.488671137	0.098067457	-4.983010182	6.26027E-07	1.33129E-06
CCL23	212.6241853	-0.650043667	0.13044462	-4.983292291	6.25114E-07	1.3295E-06
KHNYN	3283.1778	0.197450366	0.039614776	4.984260618	6.21992E-07	1.323E-06
ARHGEF1	8209.59728	-0.169370099	0.033980261	-4.984367172	6.21649E-07	1.32242E-06
PCOLCE	61.51645098	-0.268693607	0.053904932	-4.984583013	6.20956E-07	1.32108E-06
TMEM69	399.3917478	-0.211985383	0.04252664	-4.984766833	6.20366E-07	1.31997E-06
AC090004.1	73.07617641	0.219396058	0.044011041	4.985023107	6.19544E-07	1.31837E-06
RPA3	364.3579203	-0.280287926	0.056225935	-4.985029134	6.19525E-07	1.31837E-06

UNC5C	261.3928535	-0.909860688	0.182517475	-4.985060682	6.19424E-07	1.31837E-06
FAM71F2	203.4454881	-0.375926046	0.07541001	-4.985094774	6.19314E-07	1.31831E-06
CTSH	1825.444979	0.296620273	0.059494469	4.985678169	6.17448E-07	1.31448E-06
TBC1D28	0.776499224	-0.595114938	0.119355334	-4.986077452	6.16175E-07	1.31191E-06
ZNF154	557.709208	-0.300983307	0.060364089	-4.986131824	6.16001E-07	1.31168E-06
ELOA3C	0.463486446	-0.816100956	0.163654166	-4.986741116	6.14063E-07	1.3077E-06
DPY19L2	389.8517333	-0.460527277	0.092344525	-4.987055567	6.13064E-07	1.30571E-06
SNX24	62.22048269	-0.322189217	0.064581994	-4.988839736	6.0743E-07	1.29385E-06
ANAPC10	154.3425728	0.182266875	0.036532933	4.989111419	6.06577E-07	1.29218E-06
RHOG	6585.455852	0.265181629	0.053151028	4.989209811	6.06268E-07	1.29166E-06
KY	70.59383061	-0.39041788	0.078226955	-4.990835704	6.01186E-07	1.28097E-06
TUBB	11836.38427	-0.217445367	0.043565091	-4.991275436	5.99819E-07	1.2782E-06
SLC10A5	24.44604919	-0.403850163	0.080907366	-4.991512902	5.99082E-07	1.27677E-06
CAPZB	7929.107859	0.123900316	0.024813002	4.99336269	5.9337E-07	1.26473E-06
IL32	157.450125	0.355974385	0.071275697	4.994330434	5.90402E-07	1.25854E-06
IDH1	1032.683703	-0.249835501	0.050023235	-4.994389145	5.90223E-07	1.2583E-06
H2AC14	1120.688732	-0.464560688	0.093002303	-4.995152509	5.87893E-07	1.25347E-06
C10orf53	0.835632803	-0.652045298	0.13050793	-4.996212107	5.84674E-07	1.24674E-06
EIF5B	2388.99309	-0.229497589	0.045933862	-4.996261526	5.84524E-07	1.24656E-06
UCHL5	630.5924563	-0.152752328	0.030569594	-4.996871284	5.82679E-07	1.24276E-06
ELK1	1096.524739	0.167369398	0.033487607	4.997950381	5.79429E-07	1.23596E-06
ATP1A4	23.08724881	-0.481791316	0.09636902	-4.999441894	5.74965E-07	1.22657E-06
ARC	277.9835786	0.568497197	0.113699675	4.999989621	5.73334E-07	1.22323E-06
GOLGA6L22	1.190656812	-0.585069706	0.117011834	-5.000090034	5.73035E-07	1.22272E-06
GRXCR2	0.834382548	-0.862390714	0.172453722	-5.000708037	5.71202E-07	1.21894E-06
PTGER3	67.48713144	-0.606016704	0.121183319	-5.000826113	5.70852E-07	1.21833E-06
MLLT3	619.1817714	-0.372825455	0.074544999	-5.001347609	5.6931E-07	1.21517E-06
TMEM191B	17.71381215	-0.3564319	0.071266583	-5.001388949	5.69187E-07	1.21504E-06
FAM50A	612.88571	-0.209436759	0.041870849	-5.001970629	5.67472E-07	1.21151E-06
PABPC5	0.503107658	-1.231834817	0.246222887	-5.002925729	5.64667E-07	1.20565E-06
ONECUT3	15.8073446	-0.467607524	0.093465613	-5.002989985	5.64479E-07	1.20538E-06
SPDYE21	72.80707966	-0.219322349	0.043822495	-5.004789136	5.59232E-07	1.19431E-06
REP15	0.548103726	-0.752783312	0.150381998	-5.005807341	5.56284E-07	1.18814E-06
KRTAP10-3	1.10620566	-0.725133165	0.144838337	-5.006500224	5.54286E-07	1.18401E-06
COX20	224.3659372	-0.28015228	0.055957023	-5.006561544	5.5411E-07	1.18376E-06
NLRP4	4.346194381	-0.658973242	0.131587271	-5.007879839	5.50329E-07	1.17581E-06
HSBP1L1	281.5854259	0.229713019	0.045863613	5.008611493	5.48241E-07	1.17148E-06

PHF23	519.7320286	-0.163861036	0.032714706	-5.008788213	5.47738E-07	1.17053E-06
AADAC	1.824139966	-0.746921999	0.149079147	-5.01023793	5.43628E-07	1.16187E-06
GOLGA6L7	139.3079246	-0.705374352	0.140780249	-5.010463877	5.4299E-07	1.16064E-06
TLX3	0.53739175	-0.745825205	0.14884599	-5.01071748	5.42275E-07	1.15923E-06
FAS	549.0431196	0.298850785	0.05963555	5.011285838	5.40675E-07	1.15594E-06
SYNE1	3427.109423	-0.347471318	0.069334411	-5.011527679	5.39996E-07	1.15461E-06
JADE3	392.1260279	-0.183310467	0.036562896	-5.013565289	5.34306E-07	1.14257E-06
GPATCH3	634.8197108	0.161296054	0.032171463	5.013637504	5.34106E-07	1.14227E-06
KIAA2026	1944.886348	-0.119143767	0.023759725	-5.014526319	5.31643E-07	1.13713E-06
SYNGR2	2889.123794	0.197263426	0.039335022	5.014956518	5.30455E-07	1.13471E-06
AC113554.1	0.956579882	-0.660899844	0.131764731	-5.015756789	5.28251E-07	1.13012E-06
GRK1	11.7548944	0.537613247	0.107184272	5.01578483	5.28174E-07	1.13008E-06
MYL10	1.436715916	-0.517454035	0.103161601	-5.01595582	5.27705E-07	1.1292E-06
IL20RA	1.721062972	-0.798593248	0.159196611	-5.016396022	5.26497E-07	1.12674E-06
JMJD8	307.9216039	-0.19003731	0.037872158	-5.017863241	5.22493E-07	1.11829E-06
METTL23	529.011576	-0.196371252	0.03913394	-5.017927003	5.2232E-07	1.11804E-06
TCTEX1D4	249.2281423	0.334113885	0.066583391	5.017976445	5.22186E-07	1.11788E-06
ZSCAN18	411.3095411	-0.294257208	0.058639896	-5.018037679	5.22019E-07	1.11764E-06
IQCE	516.1045983	-0.21987483	0.0438149	-5.01826614	5.21399E-07	1.11644E-06
EPGN	352.0932985	-0.572605643	0.114102557	-5.018341919	5.21194E-07	1.11612E-06
ANAPC16	1210.720848	-0.212035784	0.042249957	-5.018603494	5.20485E-07	1.11472E-06
PSG4	1.761098156	-0.970698966	0.193390016	-5.019385117	5.18371E-07	1.11032E-06
KCTD5	583.4941137	0.195869101	0.039018188	5.01994351	5.16867E-07	1.10722E-06
LRRC3B	6.06089741	-0.763004361	0.151989221	-5.020121546	5.16388E-07	1.10631E-06
FLT3	5039.456806	-0.35571055	0.0708347	-5.021699082	5.12164E-07	1.09738E-06
IMPA2	890.9349121	-0.260528414	0.051879131	-5.021834562	5.11803E-07	1.09673E-06
TXNDC16	412.8066874	-0.221909454	0.044188111	-5.021926742	5.11557E-07	1.09632E-06
NOTCH1	4781.438536	-0.237933536	0.047368683	-5.023013521	5.0867E-07	1.09025E-06
ADCY1	80.31934885	-0.402785399	0.08018368	-5.023284036	5.07953E-07	1.08884E-06
RPS6KC1	1059.53031	-0.199468689	0.03970778	-5.023415803	5.07605E-07	1.08821E-06
DHCR7	683.7444906	0.274076437	0.054558064	5.023573338	5.07188E-07	1.08744E-06
KCNK13	9.330105919	0.547223442	0.108903555	5.024844604	5.0384E-07	1.08038E-06
PRELID2	247.4780977	-0.280480174	0.055807389	-5.025860852	5.01179E-07	1.07479E-06
CAT	10811.93634	-0.286444819	0.05699167	-5.026082235	5.00601E-07	1.07367E-06
ZFX	2877.984659	0.16669225	0.033161401	5.026695014	4.99005E-07	1.07036E-06
NID1	1483.991768	0.508260745	0.101112025	5.026709182	4.98968E-07	1.07036E-06
SPTY2D1OS	32.99441042	-0.225314165	0.04482129	-5.026945087	4.98355E-07	1.0692E-06

AC008687.4	16.33572359	-0.42208084	0.083932434	-5.028816882	4.93515E-07	1.05893E-06
IGFBPL1	5.881630883	-0.521648248	0.103721041	-5.029338726	4.92174E-07	1.05617E-06
OR4K17	1.035304256	-0.570721001	0.113473436	-5.02955599	4.91617E-07	1.05509E-06
ATP4A	4.099560683	-0.461346746	0.091718932	-5.030005634	4.90465E-07	1.05273E-06
TBCB	949.3751617	-0.243450737	0.048399463	-5.03002975	4.90404E-07	1.05272E-06
NFKBIB	1194.293665	0.205659371	0.040883875	5.030329759	4.89637E-07	1.05119E-06
HLX	504.7220941	0.345197363	0.068596462	5.032291083	4.84653E-07	1.0406E-06
ADAM32	65.20124173	-0.46251117	0.09190087	-5.032718094	4.83574E-07	1.0384E-06
CNOT11	1285.482137	0.094314379	0.018740189	5.032733577	4.83535E-07	1.0384E-06
DHX15	9723.326946	0.166101986	0.032998812	5.033574674	4.81417E-07	1.03399E-06
MSC	17.72975517	0.584710595	0.116151587	5.034030169	4.80274E-07	1.03165E-06
OR6C4	1.36139356	-0.620512878	0.123238022	-5.03507658	4.77658E-07	1.02614E-06
PIGG	1798.158309	-0.137660014	0.027335482	-5.035946003	4.75495E-07	1.02161E-06
CDKL4	18.7525483	-0.474079816	0.094115186	-5.037229762	4.72318E-07	1.01489E-06
SPATS2	767.1366553	-0.170529871	0.033852132	-5.0374928	4.71669E-07	1.01361E-06
NPDC1	277.9805112	-0.446837211	0.088702234	-5.037496687	4.7166E-07	1.01361E-06
AUP1	3620.264887	-0.161409045	0.032041336	-5.037525504	4.71589E-07	1.01361E-06
GPR19	17.52109278	-0.252300267	0.050082869	-5.037655987	4.71267E-07	1.01308E-06
BAG4	1891.897061	0.156636248	0.031092844	5.037694437	4.71173E-07	1.01299E-06
PROX1	6.297976724	-0.734697672	0.145820282	-5.038377806	4.69494E-07	1.00949E-06
GCNT1	901.7891393	-0.291330019	0.057820575	-5.038518234	4.6915E-07	1.00886E-06
GOLGA8M	683.7721176	-0.739992272	0.146862654	-5.038668789	4.68781E-07	1.00818E-06
SLC22A9	2.791605456	-0.506019761	0.100426724	-5.038696263	4.68714E-07	1.00815E-06
CYP3A7	4.399287452	-0.360473939	0.071540496	-5.038739735	4.68607E-07	1.00803E-06
OR6P1	1.348413982	-0.846139573	0.167912185	-5.039179108	4.67533E-07	1.00583E-06
USP18	98.13378113	-0.415048722	0.082357394	-5.03960485	4.66494E-07	1.0037E-06
MOSPD3	309.7749614	-0.325769044	0.064623094	-5.041062306	4.62955E-07	9.96197E-07
SULT1C3	0.745362072	-0.646530229	0.128238868	-5.041608982	4.61634E-07	9.93464E-07
UPF2	3256.637055	-0.182119037	0.036119754	-5.042089654	4.60475E-07	9.9108E-07
BACE2	220.6271358	-0.454205984	0.090078287	-5.042347025	4.59856E-07	9.89857E-07
OST4	1699.174698	-0.349810066	0.069364195	-5.04309271	4.58067E-07	9.86114E-07
KLK11	1.383723206	-0.703383814	0.139447079	-5.044091417	4.55681E-07	9.81086E-07
TRIM52	593.1170123	-0.144392317	0.028623881	-5.044470294	4.54779E-07	9.79252E-07
FGFR4	53.77554501	-0.315292467	0.062500729	-5.044620622	4.54422E-07	9.7859E-07
FAM163B	1.870521141	-0.679282124	0.134645614	-5.044962887	4.53609E-07	9.76947E-07
BCL2L12	373.6855911	-0.177988588	0.03527468	-5.045788834	4.51654E-07	9.72843E-07
PANK3	2915.485603	0.169925177	0.03367638	5.04582671	4.51565E-07	9.72757E-07

MBNL2	1373.902326	0.26172097	0.051865911	5.046107656	4.50901E-07	9.71436E-07
PCDHGC4	41.51593954	-0.448152899	0.088799039	-5.046821481	4.49221E-07	9.67921E-07
PLTP	1035.109753	-0.287642159	0.056992839	-5.046987745	4.4883E-07	9.67186E-07
PRAP1	6.598474221	-0.361257965	0.071578293	-5.047032423	4.48725E-07	9.67067E-07
SYCE1	2.430520987	-0.62628587	0.124077906	-5.047521262	4.47579E-07	9.64702E-07
CFI	5.982394964	-0.571835932	0.113282348	-5.04788206	4.46735E-07	9.62989E-07
AP3B1	2804.531365	0.139262974	0.027586072	5.048307496	4.45741E-07	9.60953E-07
RILPL2	2521.81974	0.228071504	0.045170741	5.049098161	4.43901E-07	9.5709E-07
RTL9	12.21818653	-0.721167522	0.142812179	-5.049762057	4.42361E-07	9.53875E-07
KHDC3L	1.740090654	-0.547006952	0.108307246	-5.050511129	4.40629E-07	9.50247E-07
AMBP	7.356075157	-0.435583574	0.086236656	-5.051025773	4.39444E-07	9.47794E-07
SLC35F2	554.8567939	0.301320913	0.059650726	5.051420681	4.38536E-07	9.45941E-07
DYNC1LI2	3164.615385	0.190739652	0.037756803	5.051795671	4.37676E-07	9.44189E-07
SLC2A14	221.6128406	-0.426608492	0.084444633	-5.051931402	4.37365E-07	9.43622E-07
SLC5A11	20.96591777	-0.343215969	0.067932158	-5.052334233	4.36443E-07	9.41737E-07
CLCN5	269.7978386	-0.318124521	0.062964513	-5.052441577	4.36198E-07	9.41312E-07
NOD1	908.0351997	-0.184829953	0.036560149	-5.055503336	4.29257E-07	9.26436E-07
PER2	1722.21827	0.181510871	0.035900566	5.055933371	4.28291E-07	9.24453E-07
SEPTIN2	5898.204611	0.118906236	0.023517613	5.056050432	4.28028E-07	9.23988E-07
CACNA2D3	149.822367	-0.52572764	0.103962314	-5.056905928	4.26113E-07	9.19955E-07
SLC7A1	2425.189546	-0.21197847	0.041916979	-5.057102734	4.25674E-07	9.19108E-07
OTP	1.848587252	-0.58357827	0.115392302	-5.057341419	4.25142E-07	9.1806E-07
TEX49	1.131284677	-0.720208444	0.142395162	-5.0578154	4.24087E-07	9.15883E-07
KCNK18	0.753355471	-1.137914886	0.224981371	-5.057818257	4.2408E-07	9.15883E-07
ACHE	218.6622204	-0.501074002	0.099065483	-5.058007956	4.23659E-07	9.15161E-07
PSG5	1.018803985	-0.842356922	0.166526135	-5.058406722	4.22774E-07	9.1335E-07
EIF2AK2	4337.419518	0.206463927	0.040813168	5.05875767	4.21997E-07	9.11772E-07
SMIM31	1.106725148	-0.673450528	0.133111437	-5.059298775	4.20801E-07	9.09289E-07
NECTIN4	84.82998995	0.450223903	0.088984308	5.059587602	4.20164E-07	9.08013E-07
AIFM1	973.1344496	-0.123375141	0.024375263	-5.061489689	4.15993E-07	8.99098E-07
BAIAP2L1	189.7501739	0.301976234	0.059660142	5.061607676	4.15736E-07	8.98641E-07
GPR18	426.1055984	0.241498945	0.047708336	5.061986331	4.14911E-07	8.96957E-07
ISOC1	266.834403	-0.241662036	0.047740459	-5.061996529	4.14889E-07	8.96957E-07
MAFA	62.64774625	-0.565625503	0.11171372	-5.063169548	4.12343E-07	8.91603E-07
IL17F	0.58946664	-0.915117906	0.180739495	-5.063187262	4.12304E-07	8.91603E-07
RBKS	182.9509811	-0.303903925	0.060021105	-5.063284375	4.12094E-07	8.91262E-07
C2orf50	25.9193622	-0.347816392	0.068689508	-5.063602923	4.11406E-07	8.89872E-07

TMBIM6	14080.10526	0.164725471	0.032526501	5.064346552	4.09804E-07	8.86504E-07
NKX3-2	4.189993428	0.786769102	0.1553442	5.064682813	4.09081E-07	8.85038E-07
GTF2A2	700.3850714	-0.247951751	0.048954708	-5.064921454	4.08569E-07	8.84028E-07
EIF3L	9075.082128	0.174602675	0.034470125	5.065333383	4.07686E-07	8.82216E-07
EIF4E	1878.635262	0.158495039	0.031287133	5.065821772	4.06642E-07	8.80054E-07
LEKR1	46.8723011	-0.311145956	0.061415902	-5.066211635	4.05811E-07	8.78352E-07
FSTL1	109.9026294	-0.501554904	0.098998074	-5.066309709	4.05602E-07	8.77996E-07
SEZ6L	117.5256989	-0.572476245	0.112910567	-5.070174214	3.97452E-07	8.60449E-07
TUBD1	184.5847028	-0.213106968	0.042030777	-5.070260015	3.97273E-07	8.60157E-07
ZNF562	1743.590343	-0.190989304	0.037664878	-5.070753291	3.96244E-07	8.58025E-07
AC010325.1	28.31477172	-0.264385663	0.052137158	-5.0709642	3.95805E-07	8.57169E-07
CUX2	88.38662595	-0.442296029	0.087207823	-5.071747145	3.9418E-07	8.53744E-07
ITGAE	1320.613142	-0.295776439	0.058314842	-5.072061065	3.9353E-07	8.52431E-07
YAP1	6.375133475	-0.624574566	0.123120119	-5.072887932	3.91823E-07	8.48828E-07
PRTFDC1	29.42247772	-0.572670993	0.11287546	-5.073476499	3.90613E-07	8.46299E-07
CORO7	1172.068911	0.167836964	0.033080055	5.073660325	3.90235E-07	8.45575E-07
CPSF3	1261.293219	-0.112434471	0.022159582	-5.073853414	3.89839E-07	8.4481E-07
EPYC	0.904364384	-1.391083611	0.274155031	-5.074076547	3.89382E-07	8.43913E-07
FBLN1	46.42397631	0.65590159	0.129246925	5.074794541	3.87915E-07	8.40826E-07
NECTIN1	1011.8129	-0.374389116	0.073766385	-5.075335003	3.86814E-07	8.38532E-07
PSME1	4880.191472	-0.29311737	0.057751537	-5.075490404	3.86498E-07	8.3794E-07
NR4A3	1323.257357	0.448857562	0.088433603	5.075644854	3.86184E-07	8.37352E-07
LCA5	78.58124281	-0.474050087	0.093395542	-5.075724978	3.86021E-07	8.37092E-07
WDSUB1	709.8884187	0.15741551	0.031006066	5.07692618	3.8359E-07	8.31912E-07
UPF3B	526.7475701	-0.192340521	0.037884021	-5.077088285	3.83263E-07	8.31295E-07
FBXO22	1037.04271	-0.179689309	0.03538875	-5.077582857	3.82267E-07	8.29226E-07
FBN1	781.2000583	-0.456834486	0.08996998	-5.0776324	3.82167E-07	8.29102E-07
VAMP2	2615.90912	0.244320916	0.048113549	5.078006514	3.81416E-07	8.27563E-07
DDN	36.42477384	-0.297632987	0.058607468	-5.078414037	3.80599E-07	8.25882E-07
SPRED2	879.6408622	0.292279132	0.057518437	5.081485983	3.74494E-07	8.12725E-07
NAP1L1	17412.57312	0.213913274	0.042095242	5.081649746	3.74171E-07	8.12114E-07
ZNF574	1248.798136	-0.183964578	0.036194424	-5.082677345	3.72152E-07	8.07821E-07
SMIM36	1.452272795	-0.561293417	0.110430919	-5.082755989	3.71997E-07	8.07576E-07
C4orf36	2.093737395	-0.453470836	0.089206757	-5.083368708	3.70799E-07	8.05063E-07
PDZK1IP1	115.7657473	-0.581795821	0.11436529	-5.087171276	3.63444E-07	7.89181E-07
MT1H	6.649997695	-1.088072714	0.213864518	-5.087672908	3.62484E-07	7.87185E-07
C19orf81	41.34434426	-0.491597917	0.096605855	-5.088696903	3.60532E-07	7.83034E-07

ANKRD36C	2774.792154	-0.269193487	0.052896579	-5.089052852	3.59856E-07	7.81652E-07
SULT1A3	3.848177987	-0.357110914	0.070170539	-5.089185848	3.59604E-07	7.81191E-07
H2BC13	772.1710837	-0.423902778	0.083291215	-5.089405606	3.59188E-07	7.80373E-07
CREB3L2	3988.167204	0.153844015	0.030224695	5.090010479	3.58044E-07	7.77974E-07
BIRC5	664.4692381	-0.288984305	0.056772347	-5.090229976	3.5763E-07	7.7716E-07
ZIM2	0.667529966	-1.113202788	0.218585514	-5.09275646	3.52895E-07	7.66957E-07
GMEB1	1434.391874	0.142707591	0.02802112	5.092858301	3.52705E-07	7.6663E-07
CLIC2	223.5495393	-0.461048457	0.090527499	-5.092910567	3.52608E-07	7.66503E-07
PKP2	102.795695	-0.624799835	0.122671473	-5.093277357	3.51926E-07	7.65106E-07
C11orf54	303.0846975	-0.180335182	0.035403099	-5.093768325	3.51016E-07	7.63211E-07
ZFYVE9	346.6676638	0.306702633	0.060209549	5.093920153	3.50735E-07	7.62685E-07
CBFB	1808.190792	0.136402073	0.026770162	5.09530243	3.48185E-07	7.57225E-07
TLE1	538.2642902	0.359092633	0.070463511	5.09615014	3.4663E-07	7.53928E-07
C2orf76	105.9484534	-0.201085152	0.039443271	-5.098085065	3.43107E-07	7.46347E-07
THPO	4.432366071	-0.551773871	0.108230211	-5.098150197	3.42989E-07	7.46173E-07
FOXP4	958.6423247	0.294753266	0.057800853	5.099462233	3.4062E-07	7.41102E-07
FBP1	368.7948512	0.497203421	0.09749032	5.100028602	3.39602E-07	7.3897E-07
LRRC57	1091.169011	0.173323944	0.033969481	5.102343065	3.35474E-07	7.30068E-07
STON2	1147.388692	-0.440804519	0.086385869	-5.102738715	3.34773E-07	7.28624E-07
DNAAF1	86.53120636	0.344970565	0.067603346	5.102862256	3.34555E-07	7.28229E-07
ATP11C	687.5860864	-0.180248744	0.035320034	-5.103300458	3.3378E-07	7.26625E-07
HOXB9	403.0892248	-1.067963729	0.209263632	-5.103436843	3.3354E-07	7.26182E-07
CTSD	3424.496356	0.307361506	0.060223089	5.103715385	3.33049E-07	7.25194E-07
SNX19	2992.405939	-0.1594104	0.031227779	-5.10476259	3.3121E-07	7.2127E-07
GJA10	1.484867052	-0.739808072	0.144903032	-5.105538939	3.29853E-07	7.18395E-07
XRN1	5322.930029	0.222894343	0.043650398	5.106353088	3.28436E-07	7.15388E-07
UBQLN2	2043.720449	0.123560892	0.024192221	5.107463711	3.26512E-07	7.11276E-07
ACKR3	120.7148285	0.478479204	0.093676133	5.107802671	3.25927E-07	7.10081E-07
RABGEF1	568.7815684	0.252377951	0.049403989	5.108452878	3.24807E-07	7.07721E-07
OSBPL9	4503.938626	0.195317492	0.038227557	5.1093376	3.2329E-07	7.04493E-07
PRSS54	0.619211892	-0.816531128	0.159801921	-5.109645257	3.22764E-07	7.03426E-07
H2BC7	2034.986875	-0.387132788	0.07575768	-5.110145784	3.2191E-07	7.01643E-07
H3C10	2702.511024	-0.369166498	0.07223624	-5.110544224	3.21232E-07	7.00242E-07
GPR108	1202.357544	-0.165679364	0.032415726	-5.111079832	3.20323E-07	6.98337E-07
PTPRF	273.6308871	-0.421764561	0.082518028	-5.111180797	3.20151E-07	6.98042E-07
FGF9	21.10179511	0.512793732	0.100320685	5.111545385	3.19534E-07	6.96774E-07
MOGS	1292.625428	-0.125455445	0.024541376	-5.111997221	3.18771E-07	6.95186E-07

RMND1	482.5691768	-0.138706888	0.0271305	-5.112581275	3.17786E-07	6.93117E-07
CABP1	12.93135118	-0.465058761	0.090958869	-5.112846771	3.1734E-07	6.9222E-07
POTEI	4.444223441	0.438907535	0.085830615	5.113647781	3.15996E-07	6.89366E-07
TRIM14	1571.774884	-0.194083642	0.037948379	-5.114411911	3.1472E-07	6.86658E-07
RGS16	142.9301241	-0.563535629	0.110178785	-5.114738097	3.14177E-07	6.85549E-07
CELF2	11502.95162	0.132558598	0.02591054	5.116010689	3.12065E-07	6.81019E-07
PITPNM1	2246.110611	0.228718237	0.044696237	5.117169846	3.10154E-07	6.76924E-07
SLC6A16	99.44762973	-0.274510416	0.053638794	-5.117758877	3.09188E-07	6.74889E-07
H2AC11	1833.215627	-0.356210764	0.069602264	-5.11780426	3.09113E-07	6.74802E-07
MTHFD2	1336.345868	0.182920143	0.035739425	5.118161338	3.08529E-07	6.73601E-07
PAX8	84.68709398	-0.466691688	0.091175832	-5.11858984	3.07829E-07	6.72147E-07
ZGLP1	25.29499588	-0.325640956	0.06361265	-5.119122618	3.0696E-07	6.70326E-07
OLAH	15.0545269	0.595691129	0.116348157	5.119901702	3.05695E-07	6.67638E-07
KLF18	0.614525042	-0.825139206	0.161158384	-5.120051387	3.05452E-07	6.67182E-07
HNRNPA3	9145.688214	0.109627329	0.021410331	5.120300549	3.05049E-07	6.66376E-07
EXO1	328.8464636	-0.250377654	0.048897537	-5.120455344	3.04799E-07	6.65903E-07
AC007192.1	16.17880954	0.476658309	0.093087827	5.120522455	3.0469E-07	6.65741E-07
PKM	17650.32517	0.229365739	0.044783977	5.121602705	3.0295E-07	6.62011E-07
COA8	324.2756418	-0.163706545	0.031956836	-5.122739398	3.01128E-07	6.58105E-07
SEC16A	8398.805975	0.162664575	0.031747673	5.123669193	2.99647E-07	6.54939E-07
KDM4E	1.653959825	-0.851068954	0.166104933	-5.123682594	2.99625E-07	6.54939E-07
H2BC8	2294.204204	-0.389038111	0.07589336	-5.126115237	2.95782E-07	6.46636E-07
PSMA3	2071.206731	0.119630346	0.023335127	5.126620615	2.94989E-07	6.44976E-07
CLEC2A	26.22910176	-1.033546785	0.201592498	-5.126910941	2.94535E-07	6.44054E-07
CCDC90B	569.0506129	-0.142098431	0.027715244	-5.127085734	2.94262E-07	6.43529E-07
CUTC	416.3233529	-0.180327288	0.035169653	-5.127354718	2.93842E-07	6.42682E-07
SDHA	607.4825037	0.217327101	0.042380008	5.128057051	2.92748E-07	6.40361E-07
GOLGA6L9	39.81917185	-0.326496666	0.063667129	-5.128182641	2.92553E-07	6.40006E-07
ADM	835.1521753	0.492375229	0.096004949	5.128644269	2.91836E-07	6.3851E-07
CDK5RAP2	2835.885803	-0.15661083	0.030532688	-5.129284114	2.90846E-07	6.36415E-07
SLC39A7	1187.55049	0.178936237	0.034874282	5.130893796	2.8837E-07	6.31066E-07
VSX1	2.018895804	-0.48046235	0.093639329	-5.130988793	2.88224E-07	6.30818E-07
CCNL1	28217.9747	0.203024594	0.039565913	5.131300625	2.87747E-07	6.29845E-07
LCTL	14.49827685	-0.277474781	0.054074805	-5.131313572	2.87727E-07	6.29845E-07
GLB1L3	19.04997426	0.447517532	0.087207956	5.131613574	2.87269E-07	6.28939E-07
DNASE2	399.8122937	0.279667139	0.054495972	5.131886436	2.86853E-07	6.28098E-07
USP15	7463.528324	0.201310338	0.039225072	5.132185285	2.86397E-07	6.27171E-07

ITGA4	8774.915072	-0.214220355	0.041739329	-5.132338256	2.86165E-07	6.26732E-07
AKR1B1	866.8605078	-0.282991326	0.055131839	-5.13299267	2.85171E-07	6.24625E-07
HBG1	50.87542309	-0.879052665	0.171226328	-5.133863916	2.83853E-07	6.21809E-07
BORCS6	101.8825301	-0.196416908	0.038258713	-5.133913112	2.83779E-07	6.21716E-07
CCDC197	8.002082036	-0.647396837	0.12608702	-5.134524064	2.82859E-07	6.19769E-07
AC069368.1	21.82052936	0.389672333	0.075871428	5.13595624	2.80713E-07	6.15136E-07
SGCA	7.481402395	-0.332117012	0.064661675	-5.13622655	2.8031E-07	6.14321E-07
HCST	459.7766741	-0.432866942	0.084274291	-5.136405622	2.80043E-07	6.13805E-07
MZT2A	622.5272735	-0.259010332	0.050423187	-5.136730685	2.79559E-07	6.12813E-07
CDC20B	5.30456223	-0.504130402	0.098141266	-5.136783132	2.79481E-07	6.12711E-07
PROSER3	448.5320298	-0.172466991	0.033574614	-5.136827154	2.79416E-07	6.12636E-07
ST8SIA4	1094.776397	0.20592015	0.040079216	5.137828823	2.77931E-07	6.09449E-07
PPP1R42	2.852114754	-0.487268907	0.09482564	-5.138577586	2.76826E-07	6.07094E-07
KYAT1	28.77226376	-0.269076547	0.052359801	-5.138991024	2.76218E-07	6.05828E-07
ZMAT1	711.3981934	-0.25779092	0.050151056	-5.140288928	2.74316E-07	6.01725E-07
SLC25A3	13976.61788	0.144863016	0.028173756	5.141771598	2.7216E-07	5.97062E-07
RHBDL3	129.6277834	0.545573313	0.106098259	5.142151396	2.7161E-07	5.95922E-07
YY1	6441.131969	0.150776781	0.029319185	5.14259801	2.70965E-07	5.94574E-07
CA9	4.904965785	-0.461782081	0.089790594	-5.142878119	2.70561E-07	5.93754E-07
ERCC8	512.3854138	-0.15347997	0.02984167	-5.143142801	2.7018E-07	5.92984E-07
OR6N1	8.323574244	-0.695108205	0.135143756	-5.143472594	2.69706E-07	5.9201E-07
PRG2	73.48852806	0.410481302	0.079800042	5.143873234	2.69131E-07	5.90815E-07
ERI1	730.6361781	0.179654312	0.034925224	5.143970129	2.68992E-07	5.90576E-07
CA5A	2.974754267	-0.513180091	0.099763325	-5.143975396	2.68985E-07	5.90576E-07
HASPIN	239.2522482	-0.215138047	0.041820645	-5.144302411	2.68517E-07	5.89664E-07
MMRN1	1325.873753	-0.571969081	0.111184665	-5.144316252	2.68497E-07	5.89664E-07
ATP6V1G1	2175.139791	0.234758831	0.04563028	5.14480368	2.67801E-07	5.88224E-07
VWA5B1	3.405197689	-0.420370512	0.081705907	-5.144921939	2.67632E-07	5.87919E-07
BSX	0.509125702	-0.93498623	0.181709515	-5.14549956	2.6681E-07	5.86179E-07
ATP8B2	1543.289016	-0.240941088	0.046820985	-5.146006362	2.6609E-07	5.84664E-07
AC005726.1	6.777257744	-0.277092739	0.053844557	-5.146160593	2.65872E-07	5.84249E-07
ZNF101	776.8201248	0.193047148	0.037511751	5.146311364	2.65658E-07	5.83846E-07
SGPL1	1042.618275	0.203380926	0.039515508	5.146863509	2.64878E-07	5.82196E-07
PSME4	10363.7402	0.242578121	0.047123428	5.147718068	2.63674E-07	5.79616E-07
ZADH2	1151.282155	-0.148366302	0.028818177	-5.148358257	2.62776E-07	5.77706E-07
ARHGEF16	24.25162885	-0.451049486	0.087605564	-5.148639733	2.62382E-07	5.76905E-07
PCDHA11	1.05043122	-0.865632485	0.168103921	-5.149389012	2.61336E-07	5.7467E-07

MRAP2	2.650142163	-0.752243411	0.146080802	-5.149502214	2.61179E-07	5.74388E-07
SELENOK	1208.91618	0.234791683	0.045593408	5.149684826	2.60925E-07	5.73893E-07
CAMSAP3	33.14226891	-0.404974696	0.078634161	-5.150111513	2.60332E-07	5.72654E-07
PBRM1	3393.312362	-0.12389021	0.024051481	-5.151042897	2.59042E-07	5.69881E-07
FAM83D	280.4113328	-0.302074885	0.058634905	-5.151792902	2.58008E-07	5.6767E-07
TBC1D9B	5239.217589	-0.157514451	0.030568529	-5.152830557	2.56584E-07	5.646E-07
RAG2	11.31749047	-0.680388706	0.132031971	-5.153211763	2.56063E-07	5.63516E-07
ITSN1	567.5537841	-0.279523346	0.054241044	-5.153354797	2.55867E-07	5.6315E-07
EFNA5	21.99480089	-0.631293682	0.122491001	-5.153796415	2.55265E-07	5.61887E-07
PGD	8557.117469	0.197809363	0.038362509	5.156319777	2.51851E-07	5.54434E-07
FOXJ1	14.54595318	-0.454656804	0.088173423	-5.156392815	2.51752E-07	5.5428E-07
BEX3	248.2097483	-0.571235696	0.110779465	-5.156512483	2.51592E-07	5.53989E-07
TMED7-TICAM2	3.820143346	0.312716707	0.060643131	5.156671548	2.51378E-07	5.53581E-07
PAQR4	192.6356111	-0.277499689	0.053806011	-5.157410527	2.50388E-07	5.51463E-07
SLC44A1	2487.173616	-0.366368325	0.071037151	-5.157418597	2.50378E-07	5.51463E-07
LRRC43	74.37429377	-0.316606196	0.061382329	-5.157937194	2.49685E-07	5.50038E-07
TAS1R3	85.8709673	-0.28563396	0.055356246	-5.159922855	2.47052E-07	5.44298E-07
ASB4	0.71782979	-0.948598756	0.183833509	-5.160097101	2.46822E-07	5.43853E-07
CAPN8	2.95014768	-0.533461093	0.10333713	-5.162337034	2.43886E-07	5.37444E-07
RAC2	7225.327516	-0.295468428	0.057218402	-5.163870666	2.41895E-07	5.33117E-07
HPGDS	312.2718584	-0.641476126	0.124210599	-5.164423393	2.41181E-07	5.31604E-07
CD3G	185.8206796	0.447209471	0.086589752	5.164692835	2.40834E-07	5.30898E-07
CALML6	7.048717675	-0.331358634	0.064157989	-5.164729139	2.40787E-07	5.30855E-07
NIT1	587.7052907	-0.138810693	0.026871328	-5.165754836	2.39471E-07	5.28011E-07
GOLGA8J	3.484106542	-0.557226044	0.107865615	-5.165928418	2.39248E-07	5.27581E-07
C8orf76	159.0677285	0.172231196	0.033322451	5.168623258	2.35825E-07	5.2009E-07
OR2V1	0.567290384	-0.948961163	0.183596955	-5.16871952	2.35703E-07	5.19881E-07
MYL9	123.3392321	-0.399403496	0.077265215	-5.169253643	2.35031E-07	5.18456E-07
GBP5	1590.700993	0.347220922	0.067167082	5.169510312	2.34708E-07	5.17802E-07
TRIL	2.95214181	-0.595116622	0.115114392	-5.169784682	2.34364E-07	5.17101E-07
AIFM3	259.5501105	-0.379575135	0.073404825	-5.170983484	2.32865E-07	5.13852E-07
TSPAN2	316.7414789	-0.464475695	0.089819688	-5.17120141	2.32594E-07	5.13311E-07
MYH10	1263.336204	-0.299083768	0.057795306	-5.174879921	2.28058E-07	5.03357E-07
KCNC3	299.4328891	-0.613303479	0.118477179	-5.176553688	2.26022E-07	4.98921E-07
HNRNPUL2	1020.915423	-0.12981048	0.025075928	-5.176696913	2.25849E-07	4.98594E-07
LRRC26	7.004812991	-0.652961412	0.126129766	-5.176901801	2.25601E-07	4.98103E-07
OGN	132.6257821	-0.311729235	0.060207762	-5.177558923	2.24808E-07	4.96409E-07

OR56A4	1.086818138	-0.563352133	0.108800054	-5.177866315	2.24438E-07	4.95648E-07
TSHZ3	119.927242	0.527113192	0.101794353	5.178216465	2.24017E-07	4.94774E-07
OR52B2	1.198863071	-0.600501665	0.115945906	-5.179153679	2.22895E-07	4.92351E-07
AP3B2	17.56538252	-0.465436114	0.089853285	-5.17995656	2.21938E-07	4.90292E-07
UBE2H	5171.539222	0.261652896	0.050507052	5.180522035	2.21266E-07	4.88863E-07
EGR1	7367.103819	-0.447816491	0.086440082	-5.18065788	2.21105E-07	4.88562E-07
SPCS2	929.7561768	0.176085073	0.033980432	5.1819551	2.19572E-07	4.8523E-07
GOLGA8F	1.065367043	-0.644518728	0.124372319	-5.182171844	2.19317E-07	4.84721E-07
NBDY	661.4896622	-0.35385292	0.068260109	-5.183890368	2.17305E-07	4.80328E-07
MAGEL2	1.331518628	-0.840871049	0.162207137	-5.183933734	2.17254E-07	4.80271E-07
CLK1	11659.01106	0.193995277	0.037419471	5.184340482	2.16781E-07	4.79278E-07
PARP16	437.2759899	-0.167358294	0.032281282	-5.184375724	2.1674E-07	4.79241E-07
OR52K1	16.11731568	-0.613193555	0.118273393	-5.184543521	2.16545E-07	4.78864E-07
C7orf57	5.585244936	-0.414555415	0.079929529	-5.186511418	2.1427E-07	4.73888E-07
PRELID3A	65.77756196	-0.33811183	0.065189716	-5.186582324	2.14188E-07	4.73761E-07
TIMM10B	1260.156569	0.136475094	0.026313061	5.186591408	2.14178E-07	4.73761E-07
PREP	988.5210753	-0.159881884	0.030821029	-5.187428462	2.13218E-07	4.71721E-07
OR51E1	0.830470658	-0.89820998	0.173150833	-5.187442448	2.13202E-07	4.71721E-07
MRM3	263.1028383	-0.159243189	0.03069374	-5.188132487	2.12413E-07	4.70047E-07
KLHL21	3056.648578	0.277016957	0.053393339	5.188230618	2.12302E-07	4.69853E-07
UGGT1	4832.370824	-0.154750198	0.029822997	-5.18895532	2.11477E-07	4.68081E-07
CPN2	1.44827544	-0.786188468	0.151498379	-5.189418337	2.10952E-07	4.66972E-07
SAMD4B	3678.161127	0.160140903	0.030858911	5.18945411	2.10911E-07	4.66935E-07
DPEP2	740.9964048	-0.260716465	0.050224815	-5.190989105	2.0918E-07	4.63154E-07
COPS3	2652.179217	0.148805713	0.028663654	5.191442563	2.08671E-07	4.62079E-07
SH2D2A	181.6845496	0.32076026	0.061779682	5.192002415	2.08044E-07	4.60744E-07
CD63	6821.097899	0.265274243	0.051090618	5.192230029	2.0779E-07	4.60233E-07
RASA1	1433.420434	-0.187002241	0.036015565	-5.192261725	2.07755E-07	4.60206E-07
HSPB8	0.892327283	-0.824063982	0.158676439	-5.193360704	2.06531E-07	4.57548E-07
BRSK1	47.45387314	-0.381099015	0.073367997	-5.194349455	2.05437E-07	4.55175E-07
WWTR1	28.31825155	-0.398181034	0.076655471	-5.194424206	2.05354E-07	4.55044E-07
CLCN1	48.44736157	-0.386412487	0.074387796	-5.194568268	2.05195E-07	4.54743E-07
MAB21L1	6.487729856	-0.710164988	0.136711487	-5.194625584	2.05132E-07	4.54654E-07
POR	999.9106406	0.191387919	0.036842386	5.194775411	2.04967E-07	4.5434E-07
LIPJ	4.43483686	-0.4119888	0.079307165	-5.194849672	2.04885E-07	4.5421E-07
KIF11	987.0150553	-0.236566859	0.045537991	-5.194934051	2.04792E-07	4.54056E-07
PNMA8A	21.02229116	-0.711102817	0.136870665	-5.195436274	2.0424E-07	4.52883E-07

MNT	1474.29352	0.15964657	0.030724829	5.196011732	2.03609E-07	4.51535E-07
PRAG1	484.897476	0.357268261	0.06875152	5.196514325	2.0306E-07	4.50367E-07
IRGQ	5164.885584	0.252380517	0.048562473	5.197027666	2.025E-07	4.49177E-07
HIP1R	1576.637383	0.318415253	0.0612547	5.198217534	2.01209E-07	4.46362E-07
PLCH1	84.33226038	-0.537248213	0.103342291	-5.198725568	2.0066E-07	4.45195E-07
GTF2A1	690.9848569	-0.193390854	0.037195893	-5.199252896	2.00091E-07	4.43984E-07
TUT4	4388.541804	0.187285852	0.036021267	5.199313272	2.00026E-07	4.4389E-07
SCNN1G	1.077957842	-0.655545929	0.126057161	-5.200386254	1.98875E-07	4.41385E-07
GRINA	4173.610815	0.278347861	0.053521303	5.200692867	1.98547E-07	4.40708E-07
XPA	485.5937689	0.162671051	0.031272813	5.201676298	1.97499E-07	4.38431E-07
H3-4	3.242970931	-0.459697045	0.088374672	-5.20168317	1.97492E-07	4.38431E-07
AACS	625.3859328	-0.196973258	0.037860981	-5.202539732	1.96583E-07	4.36497E-07
EIF6	941.8799649	-0.266211483	0.051167299	-5.202766019	1.96344E-07	4.36016E-07
GZF1	1763.038551	0.203024258	0.039018296	5.203309241	1.95771E-07	4.34792E-07
SLC38A9	456.5647485	-0.184373939	0.035431218	-5.20371439	1.95344E-07	4.33894E-07
PLD4	1898.961982	-0.469039624	0.090131949	-5.203921881	1.95126E-07	4.33458E-07
LIN37	24.64240785	-0.237788788	0.045677426	-5.205827227	1.93134E-07	4.29083E-07
CCDC6	2741.67103	0.185733915	0.035677495	5.205912505	1.93046E-07	4.28934E-07
CERS1	14.68994825	-0.410669496	0.078876063	-5.206516174	1.92419E-07	4.2759E-07
STRC	10.90984065	-0.380131328	0.072996643	-5.207517967	1.91383E-07	4.25337E-07
EIF4B	15796.22991	0.142917982	0.027441604	5.208076823	1.90808E-07	4.24107E-07
ABCB4	62.84625971	0.395042731	0.075843338	5.208667522	1.90202E-07	4.22807E-07
MYCN	721.6379906	-0.56629894	0.108700545	-5.209715765	1.8913E-07	4.20473E-07
PKN3	410.8396055	-0.271711864	0.052152848	-5.209914219	1.88928E-07	4.20071E-07
OR10X1	1.39420918	-0.979877951	0.188078649	-5.209937207	1.88905E-07	4.20067E-07
ZNF331	1646.33944	0.301545155	0.057877572	5.210051943	1.88788E-07	4.19855E-07
FOXR1	4.375172177	-0.425099457	0.081581822	-5.210712973	1.88116E-07	4.18409E-07
C2CD4C	3.999120709	0.755497447	0.144972009	5.211333232	1.87488E-07	4.1706E-07
STUM	29.1771083	-0.508749598	0.097556199	-5.214938705	1.83878E-07	4.09075E-07
AATF	1477.396227	-0.132473935	0.025402223	-5.215052853	1.83765E-07	4.0887E-07
PFDN1	1063.454333	0.183942453	0.0352689	5.215429261	1.83392E-07	4.08087E-07
PLBD1	1083.775142	0.560016919	0.107344667	5.216998044	1.81846E-07	4.04693E-07
H4C13	150.8718437	-0.475361891	0.091094707	-5.218326129	1.80547E-07	4.01848E-07
MAP3K4	1276.682415	-0.165919641	0.031792271	-5.218867264	1.80021E-07	4.00722E-07
TENM4	33.58773722	-0.533162622	0.102135073	-5.220171759	1.78757E-07	3.97955E-07
GDF15	152.8126723	-0.487448817	0.093373677	-5.220409338	1.78528E-07	3.9749E-07
PINLYP	164.6798313	-0.314711681	0.060282772	-5.220590702	1.78353E-07	3.97146E-07

DHH	68.08351118	-0.505965311	0.096908085	-5.221084589	1.77878E-07	3.96133E-07
NDUFC2	238.3011802	-0.285894639	0.054756451	-5.221204733	1.77763E-07	3.95921E-07
OR13C2	1.442280317	-0.66406836	0.127184196	-5.221311942	1.7766E-07	3.95737E-07
AL121594.1	4.470231406	-0.323993279	0.062048711	-5.221595678	1.77388E-07	3.95176E-07
BIN2	3728.433956	-0.230538449	0.044144131	-5.222403151	1.76616E-07	3.93501E-07
DGKH	366.328251	-0.406355844	0.077805059	-5.222743236	1.76292E-07	3.92823E-07
GBP6	5.29955103	-0.430424097	0.082390653	-5.22418599	1.74923E-07	3.89817E-07
PRB3	25.26241136	-0.366006372	0.0700485	-5.225042225	1.74115E-07	3.88062E-07
SORCS1	49.07584957	-0.693866998	0.13274921	-5.226901158	1.72375E-07	3.84226E-07
ALG13	1385.762729	-0.158972337	0.030411112	-5.227442428	1.71871E-07	3.83147E-07
LYRM4	631.471049	-0.243770967	0.046629142	-5.227867343	1.71476E-07	3.82311E-07
TLN1	23448.62469	-0.184460791	0.035280246	-5.228444093	1.70943E-07	3.81164E-07
CCR6	7.441677491	0.401109964	0.076714627	5.228598232	1.708E-07	3.8089E-07
TEX2	1877.495706	-0.197550684	0.037772118	-5.230066376	1.69449E-07	3.7792E-07
ASCL5	4.549647247	-0.473713497	0.090572565	-5.230209607	1.69318E-07	3.77671E-07
MPIG6B	805.4874137	-0.410615015	0.078500081	-5.230759113	1.68815E-07	3.76593E-07
ELF2	3942.688054	0.129734678	0.02479071	5.233197262	1.66603E-07	3.71699E-07
FAM241B	13.75336254	-0.60387327	0.115378537	-5.233844041	1.66021E-07	3.70442E-07
RHBDF1	278.4140419	0.458981796	0.087688083	5.234255129	1.65652E-07	3.69661E-07
LRFN4	386.072896	-0.279141529	0.053329362	-5.234293405	1.65617E-07	3.69627E-07
ESF1	282.7981162	-0.225764612	0.043127494	-5.234818754	1.65147E-07	3.68619E-07
BTNL9	18.36658443	-0.503572704	0.096161048	-5.236763894	1.63417E-07	3.64798E-07
AVEN	249.9978047	-0.195842023	0.03739562	-5.237031036	1.6318E-07	3.64312E-07
RPS10	280.6839297	-0.205634695	0.039258346	-5.237986721	1.62338E-07	3.62473E-07
C11orf96	186.972384	0.467336303	0.089214883	5.238322179	1.62043E-07	3.61856E-07
UBTD2	196.8709018	-0.251246443	0.047955127	-5.239198748	1.61275E-07	3.60183E-07
VPS25	586.9331173	-0.255076351	0.048685503	-5.239267017	1.61216E-07	3.60091E-07
ST6GALNAC3	38.75447194	-0.487271485	0.092996121	-5.239696893	1.60841E-07	3.59294E-07
CDK16	3433.043967	0.169183499	0.032279748	5.241165445	1.59566E-07	3.56486E-07
SMARCE1	736.9332682	0.221954346	0.042348248	5.241169472	1.59562E-07	3.56486E-07
UHRF1BP1	3812.284195	0.174386494	0.033270284	5.241509043	1.59269E-07	3.55904E-07
CLDN19	55.39849199	-0.488948822	0.093280461	-5.241706762	1.59098E-07	3.55564E-07
SLC66A1	815.1364115	-0.235886136	0.044996123	-5.242365818	1.58531E-07	3.54336E-07
PSMC6	2296.402185	0.137892344	0.02630247	5.242562616	1.58362E-07	3.53999E-07
GPA33	234.8868671	-0.489557524	0.093373078	-5.2430265	1.57964E-07	3.5315E-07
LRIF1	201.0798687	-0.217159785	0.041415985	-5.243380904	1.57661E-07	3.52512E-07
HSD17B12	614.1339971	-0.165137758	0.031489516	-5.244213963	1.5695E-07	3.50963E-07

SNX17	2552.145538	-0.152638184	0.029104293	-5.24452466	1.56686E-07	3.50412E-07
EN1	0.516535383	-0.995681079	0.189847611	-5.244633168	1.56594E-07	3.50246E-07
KCNMB4	96.40675137	-0.427198487	0.081452195	-5.244775605	1.56473E-07	3.50016E-07
RHOC	508.7658389	0.321471249	0.061292043	5.244909955	1.56359E-07	3.49801E-07
ACKR1	81.4449225	-0.616869038	0.117565541	-5.247022476	1.54577E-07	3.45855E-07
CCT5	2238.192201	-0.205604594	0.039172456	-5.248703167	1.53174E-07	3.42754E-07
MYLIP	4030.260033	0.220862747	0.042048145	5.252615744	1.49954E-07	3.35588E-07
BAG2	119.0751633	-0.33261657	0.063323545	-5.252652354	1.49924E-07	3.3556E-07
COPG1	3377.899871	0.128799081	0.024519013	5.253028723	1.49618E-07	3.34913E-07
PRKACB	2165.641824	-0.218337683	0.041561615	-5.25334928	1.49358E-07	3.34368E-07
KCTD18	550.7873685	0.132054244	0.025134777	5.253845803	1.48956E-07	3.33506E-07
SKOR1	71.66645499	-0.302940772	0.057651484	-5.25469169	1.48273E-07	3.32015E-07
TTC14	1112.863264	-0.239868392	0.045645661	-5.255009697	1.48017E-07	3.31479E-07
SMARCB1	2448.058309	-0.163905414	0.031184916	-5.255919627	1.47287E-07	3.29882E-07
RFLNB	7797.567803	0.264798939	0.050380898	5.25593926	1.47271E-07	3.29882E-07
ACTL8	2.296990386	-0.831400591	0.158153497	-5.256921946	1.46486E-07	3.28165E-07
ZNF277	678.0054347	0.187681851	0.035701176	5.257021519	1.46407E-07	3.28025E-07
ARL4A	2047.18021	0.303461355	0.057724587	5.257055442	1.4638E-07	3.28002E-07
NSMF	1222.227851	0.251515896	0.047842473	5.257167589	1.46291E-07	3.2784E-07
CNTNAP3C	12.00957851	-0.825158553	0.156953666	-5.257338507	1.46155E-07	3.27573E-07
TATDN2	1057.186967	0.124165337	0.023616661	5.257531488	1.46002E-07	3.27267E-07
PGK2	0.808895194	-0.807202964	0.153490506	-5.258976491	1.44859E-07	3.24743E-07
ZNF75A	374.1715957	-0.185739818	0.035318403	-5.25900951	1.44833E-07	3.24722E-07
DCAF13	775.096136	-0.15349279	0.029184753	-5.259348699	1.44567E-07	3.24161E-07
SERPINB13	1.640422572	-0.688122913	0.130832077	-5.259588676	1.44378E-07	3.23775E-07
CA14	285.8009153	0.294040546	0.055905198	5.259628028	1.44347E-07	3.23743E-07
ORAI1	1641.633458	0.265206123	0.050408529	5.261135892	1.43168E-07	3.21136E-07
OR56A1	1.96929587	-0.61267332	0.116443009	-5.261572367	1.42829E-07	3.20411E-07
OR2A12	0.754930167	-0.788359143	0.149825287	-5.26185637	1.42608E-07	3.19953E-07
CDKN2D	1617.171453	0.29730655	0.056500822	5.261986301	1.42507E-07	3.19764E-07
JAGN1	597.3316169	-0.19790919	0.037609252	-5.262247511	1.42305E-07	3.19346E-07
FCRL3	135.6512509	0.434422551	0.082549486	5.262571251	1.42055E-07	3.18821E-07
GIN1	45.85089702	-0.259627338	0.049330982	-5.262967133	1.41749E-07	3.18171E-07
ARL11	367.2917229	-0.266360898	0.050609472	-5.263064153	1.41674E-07	3.1804E-07
PGLYRP3	0.959094638	-0.991351237	0.188357518	-5.263136005	1.41619E-07	3.17952E-07
MASP1	4.109608457	-0.647668559	0.123040409	-5.263868715	1.41055E-07	3.16723E-07
PARP9	1503.013439	0.251600194	0.047785022	5.265252267	1.39997E-07	3.14383E-07

MARS1	3628.780078	-0.133070569	0.025272639	-5.26540069	1.39884E-07	3.14165E-07
KRT73	9.899193201	-0.542722423	0.103070049	-5.265568698	1.39756E-07	3.13914E-07
STX3	1578.19674	0.240013051	0.045581105	5.265625946	1.39712E-07	3.13852E-07
SLC4A5	57.55821953	-0.225219391	0.042762597	-5.266737964	1.38869E-07	3.11993E-07
POU2F3	8.88213608	-0.470266928	0.089265172	-5.268201651	1.37767E-07	3.09552E-07
CHRD	7.113763022	-0.496040852	0.094148041	-5.268732607	1.37369E-07	3.08694E-07
CEP192	3535.689734	-0.207069398	0.039300492	-5.268875457	1.37262E-07	3.08489E-07
ARHGDIA	5327.371239	0.207459389	0.03936949	5.269547288	1.36761E-07	3.07397E-07
DCAF17	597.6933616	-0.151086429	0.028664571	-5.270842182	1.35799E-07	3.05271E-07
H2BU1	115.1278422	-0.595945978	0.113058398	-5.271134117	1.35583E-07	3.04821E-07
MTTP	3.391518571	-0.515484659	0.097765942	-5.272640425	1.34475E-07	3.02364E-07
TDRD15	5.462533987	-0.922801009	0.174992102	-5.273386624	1.33929E-07	3.01171E-07
CFHR2	0.74982555	-0.808181847	0.153213095	-5.274887545	1.32838E-07	2.98751E-07
PTPRN	30.45237942	0.552734492	0.104780922	5.275144368	1.32652E-07	2.98367E-07
CLDN12	136.6570843	0.304153252	0.057652658	5.275615412	1.32311E-07	2.97636E-07
MYBPC1	4.69666481	-0.537295543	0.101844764	-5.275632468	1.32299E-07	2.97636E-07
WASH6P	370.3049815	-0.251670582	0.047696897	-5.276456045	1.31706E-07	2.96342E-07
CENPQ	78.95489857	-0.241640375	0.045795903	-5.276462763	1.31701E-07	2.96342E-07
CCDC138	282.4738048	-0.260479928	0.049344745	-5.278777438	1.30049E-07	2.9268E-07
LNX2	483.89869	-0.226491329	0.042897272	-5.279853958	1.29287E-07	2.91E-07
SLC25A24	791.4926116	0.247061391	0.046777959	5.281576972	1.28077E-07	2.88309E-07
KCNA3	451.6351431	0.38782667	0.073417848	5.282457605	1.27462E-07	2.86959E-07
ARHGAP32	485.1536334	-0.454910906	0.086115393	-5.282573618	1.27382E-07	2.8681E-07
EPB41L4B	3.74793772	-0.554550438	0.104976456	-5.282617275	1.27351E-07	2.86775E-07
GOLGA8H	143.6730272	-0.283793151	0.053721171	-5.282705934	1.2729E-07	2.86669E-07
CCNI2	10.89880681	0.388738892	0.073579719	5.283234282	1.26923E-07	2.85876E-07
TBX6	88.55102198	-0.293934628	0.055623618	-5.284349309	1.26152E-07	2.84173E-07
SERPINE1	435.0253456	0.494902997	0.093653845	5.284385243	1.26128E-07	2.8415E-07
PCDH18	11.63438896	-0.767812189	0.14529801	-5.284395753	1.2612E-07	2.8415E-07
ACP4	86.59546545	-0.275544552	0.052141889	-5.284514231	1.26039E-07	2.84015E-07
AL138752.2	7.574162516	0.442408125	0.083706718	5.285216484	1.25556E-07	2.8296E-07
HINT3	612.4000734	0.142956479	0.027048286	5.285232472	1.25545E-07	2.8296E-07
TPM3	19414.44805	0.175177726	0.033143582	5.285419209	1.25417E-07	2.82712E-07
PLAAT1	4.641988785	-1.274734226	0.241170251	-5.285619683	1.2528E-07	2.82435E-07
TOP1MT	2133.30136	-0.31672112	0.05991785	-5.285922613	1.25073E-07	2.82001E-07
NEU4	1236.528595	0.55858906	0.105653274	5.287001876	1.24337E-07	2.80375E-07
CSF2	4.105428146	0.904404692	0.171055597	5.287197303	1.24205E-07	2.80108E-07

HBS1L	3422.392669	0.197147453	0.037285147	5.287559982	1.23959E-07	2.79586E-07
MACC1	434.1634404	-0.482703463	0.091288968	-5.28764287	1.23903E-07	2.79491E-07
TPST2	1958.546699	-0.218786331	0.041372499	-5.288206807	1.23521E-07	2.78663E-07
SIX2	2.509949216	-0.611399169	0.11561508	-5.288230309	1.23505E-07	2.7866E-07
PMEPA1	192.2226008	0.474533128	0.089715305	5.289321889	1.22771E-07	2.77034E-07
HEATR6	663.5232509	-0.171857407	0.03248924	-5.289671503	1.22536E-07	2.76537E-07
OPRD1	37.46166115	-0.315727548	0.05967991	-5.290348966	1.22083E-07	2.75546E-07
CCDC166	1.492328729	-0.591530619	0.111795681	-5.29117598	1.21532E-07	2.74334E-07
APOA5	4.15635176	-0.638551254	0.120673869	-5.291545366	1.21287E-07	2.73812E-07
NUMA1	5932.531016	-0.131666948	0.024882345	-5.291581153	1.21263E-07	2.7379E-07
ZBP1	125.9393549	0.391867453	0.074050159	5.291919113	1.21039E-07	2.73316E-07
CRYBB1	13.57301574	-0.430672087	0.08133753	-5.294875386	1.19098E-07	2.68962E-07
TERB2	1.795108931	-0.654098935	0.123526186	-5.295224909	1.1887E-07	2.68479E-07
CRCP	1200.864084	0.134285308	0.025357091	5.295769492	1.18516E-07	2.67711E-07
FCGR2B	142.7119903	0.555071582	0.104809919	5.295983315	1.18378E-07	2.67429E-07
HAGHL	348.5418282	-0.326884807	0.061718213	-5.29640755	1.18103E-07	2.6684E-07
FAM210A	413.9131961	0.184956026	0.034917021	5.297016211	1.1771E-07	2.65983E-07
TTC28	853.8181359	-0.361184309	0.068162374	-5.298881036	1.16515E-07	2.63311E-07
NEMP2	519.6507618	-0.162448074	0.030654547	-5.299314187	1.16238E-07	2.62718E-07
ALOX12	213.9054772	-0.205385605	0.038752829	-5.299886732	1.15875E-07	2.61925E-07
IL18BP	438.9666151	-0.181642681	0.034269667	-5.300392432	1.15554E-07	2.61231E-07
OGA	8923.243968	0.176566448	0.033310898	5.300560984	1.15447E-07	2.6102E-07
CYB561D2	388.0367151	-0.160550815	0.030285935	-5.301167508	1.15064E-07	2.60184E-07
LSMEM2	35.84273079	-0.370881543	0.069957264	-5.301544446	1.14827E-07	2.59678E-07
FHL3	2080.242528	0.284444291	0.053652621	5.30159166	1.14797E-07	2.59641E-07
FTH1	9353.298119	0.454238065	0.085669442	5.302218058	1.14404E-07	2.58781E-07
AMBN	0.804519125	-0.817941977	0.154226923	-5.303496713	1.13605E-07	2.57004E-07
MFSD11	652.208732	-0.13036786	0.024575672	-5.304752691	1.12826E-07	2.5527E-07
JOSD2	192.7615526	-0.291386971	0.054917433	-5.305910299	1.12112E-07	2.53684E-07
MARCKS	2309.232235	0.472395022	0.089031478	5.305932594	1.12098E-07	2.53683E-07
PPM1G	2699.147129	-0.174290927	0.032842629	-5.306850714	1.11535E-07	2.52438E-07
PPP1R11	972.1574422	-0.194791518	0.036696633	-5.308157831	1.10739E-07	2.50664E-07
MFSD2A	555.051555	0.365918659	0.068934129	5.308236484	1.10691E-07	2.50585E-07
CCDC190	2.160805835	-0.755488278	0.142318336	-5.308439504	1.10568E-07	2.50335E-07
PASD1	0.422009731	-1.09224289	0.205737756	-5.308908332	1.10284E-07	2.49721E-07
ZNF736	2058.669251	0.208045437	0.039187488	5.308976047	1.10243E-07	2.49657E-07
ZFP42	0.843263047	-0.68775825	0.129519917	-5.310057847	1.0959E-07	2.48208E-07

PPP2R2A	2619.675378	0.19250536	0.036252214	5.310168365	1.09524E-07	2.48086E-07
AKAP10	1132.655656	-0.169229795	0.031868612	-5.310234243	1.09484E-07	2.48025E-07
CCNA2	586.401764	-0.2878793	0.054205036	-5.310932702	1.09066E-07	2.47105E-07
PODN	9.002733208	-0.473120375	0.089070704	-5.311739501	1.08584E-07	2.46042E-07
NLRP8	1.071169854	-0.623889744	0.117449509	-5.311982547	1.08439E-07	2.45742E-07
GAL3ST3	2.228268301	-0.469197096	0.088319898	-5.312473252	1.08147E-07	2.4511E-07
SRPX	8.071595975	-0.573862717	0.107989601	-5.314055352	1.07212E-07	2.43018E-07
TRIM40	4.76540176	-0.669601738	0.12600106	-5.314254787	1.07095E-07	2.4278E-07
ZNF672	916.2963773	-0.167916786	0.031594875	-5.31468431	1.06842E-07	2.42236E-07
DYNLRB1	243.6207677	-0.230814668	0.043425416	-5.315197648	1.06542E-07	2.41582E-07
CDH1	159.3766148	-0.52550478	0.098865016	-5.315376477	1.06437E-07	2.41373E-07
NDUFC1	609.7680021	-0.275442549	0.051816131	-5.315768375	1.06208E-07	2.40882E-07
CRTAP	3928.852354	0.237691683	0.044711169	5.316158929	1.05981E-07	2.40394E-07
SPIN4	656.854201	-0.24142414	0.045413074	-5.316181465	1.05967E-07	2.40392E-07
AC010422.8	116.7546065	-0.205542748	0.038661812	-5.316428254	1.05824E-07	2.40094E-07
SLC22A2	1.770196014	-0.79890018	0.150267404	-5.316523467	1.05769E-07	2.39996E-07
CLMN	717.7506393	0.346350831	0.065140265	5.317000656	1.05492E-07	2.39396E-07
VPS37D	9.317267539	0.430747877	0.081006834	5.317426354	1.05245E-07	2.38864E-07
ARRDC4	1029.189401	0.283678582	0.053347221	5.317588786	1.05151E-07	2.38679E-07
SPECC1L-ADORA2A	6.002364775	-0.358555901	0.067426573	-5.317723899	1.05073E-07	2.38529E-07
GLYATL3	1.316044287	-1.077835686	0.202576586	-5.320633084	1.03407E-07	2.34773E-07
RASL12	9.347189932	0.77512692	0.145672076	5.321039848	1.03176E-07	2.34276E-07
DYNC2H1	224.3770056	-0.406896104	0.076458035	-5.321822682	1.02733E-07	2.33297E-07
SNCA	1167.974734	-0.499459132	0.093849767	-5.321900598	1.02689E-07	2.33224E-07
DLGAP2	75.73163589	-0.671804239	0.12619952	-5.323350179	1.01873E-07	2.31399E-07
AGAP3	879.6468117	0.361564911	0.067920164	5.323381001	1.01856E-07	2.31387E-07
MRPL28	797.5121898	-0.227008661	0.042639018	-5.323965456	1.01529E-07	2.30671E-07
PODXL	230.2321554	-0.334552274	0.06283471	-5.324322688	1.0133E-07	2.30245E-07
MICALL2	1270.353243	-0.308168177	0.057875492	-5.324674823	1.01134E-07	2.29826E-07
MYO1B	112.4847566	-0.465696148	0.087457673	-5.324817528	1.01054E-07	2.29672E-07
HINT2	5.98145309	-0.345072915	0.064799945	-5.325203793	1.0084E-07	2.29211E-07
FNIP1	4413.139956	0.22405407	0.042071585	5.3255439	1.00651E-07	2.2881E-07
ENPEP	20.80157063	-0.70111676	0.131643058	-5.325892377	1.00459E-07	2.28398E-07
TRIM5	566.8325095	-0.191913475	0.036032102	-5.326180317	1.003E-07	2.28063E-07
MFNG	1851.217371	-0.249979159	0.046931804	-5.326433995	1.0016E-07	2.27771E-07
KANSL2	2457.465092	0.158234596	0.029704878	5.326889228	9.99091E-08	2.27227E-07

PNMA6E	2.675179518	-0.624890271	0.117305545	-5.327030981	9.98311E-08	2.27077E-07
UQCRHL	127.7800479	-0.288037484	0.054065395	-5.327575697	9.95323E-08	2.26423E-07
C19orf84	21.99239657	-0.478422238	0.089793806	-5.328009337	9.9295E-08	2.2591E-07
GDF6	1.604500556	-0.654918106	0.122915996	-5.328176387	9.92038E-08	2.25728E-07
DLL1	135.1287542	0.526046362	0.09872191	5.328567522	9.89904E-08	2.25269E-07
CYBRD1	1046.260688	-0.284517901	0.053392577	-5.328791326	9.88685E-08	2.25018E-07
ARID1B	8994.433515	-0.163245713	0.030634458	-5.328826547	9.88493E-08	2.25E-07
NCKAP5	5.609594985	-0.679074614	0.127427773	-5.329094273	9.87038E-08	2.24695E-07
PNP	3522.780812	-0.286757105	0.053808056	-5.329259682	9.86139E-08	2.24517E-07
PLXNC1	2274.789178	-0.386946705	0.072596384	-5.330109912	9.81533E-08	2.23494E-07
PTGER2	1347.651682	0.351125965	0.065873791	5.33028323	9.80597E-08	2.23307E-07
IL31	9.389008509	-0.62437389	0.117133159	-5.330462335	9.79631E-08	2.23113E-07
KLKB1	10.21105285	-0.534827874	0.10029817	-5.332379203	9.69343E-08	2.20795E-07
RILP	461.0010838	-0.274946693	0.051560485	-5.332507868	9.68656E-08	2.20665E-07
OR51L1	0.619335575	-0.781602554	0.146572125	-5.332545702	9.68454E-08	2.20644E-07
TDRD3	546.4999663	-0.1431501	0.026843677	-5.332730679	9.67467E-08	2.20445E-07
CEP95	3443.986556	0.193068968	0.036204083	5.332795437	9.67122E-08	2.20392E-07
GK3P	147.9366165	0.359746348	0.067458718	5.332836982	9.66901E-08	2.20367E-07
FBXW12	7.005039754	-0.406888931	0.076282374	-5.333983603	9.60812E-08	2.19005E-07
C3orf14	15.90656502	-0.836700119	0.156848985	-5.334431189	9.58445E-08	2.18491E-07
CAPN3	419.0317958	-0.355341953	0.066596239	-5.335766093	9.5142E-08	2.16915E-07
ELAVL3	12.66875641	-0.376170077	0.070494213	-5.336183807	9.49232E-08	2.16441E-07
RBM19	2200.340211	-0.16429722	0.030789259	-5.336186256	9.49219E-08	2.16441E-07
STK40	2334.626342	0.199000539	0.037290353	5.33651534	9.47499E-08	2.16096E-07
FAM229A	526.7430498	0.27108005	0.050791799	5.337083022	9.44538E-08	2.15446E-07
EPPK1	105.1355467	-0.57801469	0.108298648	-5.337229058	9.43778E-08	2.15298E-07
PRPH2	29.24704365	-0.44036213	0.082496249	-5.33796548	9.39953E-08	2.14451E-07
LRRC59	3075.120545	0.175580415	0.032887458	5.338825966	9.35504E-08	2.1346E-07
SIGLEC14	422.3758962	0.443357386	0.083042351	5.338931061	9.34962E-08	2.13361E-07
GHRHR	1.269817593	-0.7197094	0.134802715	-5.338982955	9.34694E-08	2.13325E-07
RNF208	32.05465003	-0.369581566	0.069219408	-5.33927662	9.33182E-08	2.13005E-07
IYD	2.314321165	-0.471877894	0.088354264	-5.340748381	9.25637E-08	2.11307E-07
GPR26	0.749500555	-0.867418211	0.162408454	-5.340967105	9.2452E-08	2.11077E-07
RFX6	0.98795491	-1.111964717	0.208193391	-5.341018319	9.24259E-08	2.11042E-07
ZFAT	660.2663385	-0.236168662	0.044217657	-5.34104871	9.24104E-08	2.11031E-07
ARL5B	2732.690914	0.273651629	0.051231558	5.341466108	9.21979E-08	2.1057E-07
PCDHB7	1.410370061	-0.839318657	0.15709916	-5.342604372	9.16206E-08	2.09276E-07

NPIPA5	57.60660829	-0.33002462	0.061765256	-5.343208193	9.13158E-08	2.08605E-07
AVPR1B	44.41910465	-0.506853757	0.094852196	-5.343616464	9.11102E-08	2.08159E-07
AIF1	2998.700352	-0.410601902	0.076837065	-5.343799929	9.1018E-08	2.07973E-07
HHEX	2438.551026	0.26067716	0.048776698	5.344297002	9.07686E-08	2.07427E-07
CCDC74B	13.19076081	-0.4964403	0.092884785	-5.344689093	9.05724E-08	2.07003E-07
PCNA	1983.349944	-0.251594123	0.047047851	-5.347622039	8.91173E-08	2.03701E-07
LAMP3	17.75981118	0.406670762	0.076046748	5.347641695	8.91076E-08	2.03701E-07
TMEM151B	8.174163061	-0.437594375	0.081819325	-5.348300963	8.87837E-08	2.02986E-07
FASN	5653.275674	-0.237074243	0.04432325	-5.348755799	8.85609E-08	2.025E-07
MEPCE	1242.804149	-0.158097768	0.029555276	-5.349223261	8.83325E-08	2.02002E-07
RINT1	739.8416847	0.172161644	0.032183045	5.349451668	8.82211E-08	2.0177E-07
SH3BGRL2	107.8185686	-0.509432898	0.095227012	-5.349667991	8.81157E-08	2.01553E-07
PEX26	2664.691366	-0.158056633	0.02954265	-5.350117	8.78974E-08	2.01077E-07
APOBEC3C	2379.397181	-0.222592717	0.041603903	-5.350284481	8.78161E-08	2.00915E-07
OR10A4	1.037352263	-1.051695672	0.196549186	-5.350801466	8.75655E-08	2.00365E-07
BLOC1S5	263.977229	-0.198815063	0.037155047	-5.350957083	8.74903E-08	2.00216E-07
CARD19	2984.661689	0.292882789	0.054728577	5.351551303	8.72034E-08	1.99583E-07
MLH3	700.0041989	-0.216148795	0.040375411	-5.353476029	8.62805E-08	1.97494E-07
FAM219B	600.9794388	-0.151827272	0.028356982	-5.354140755	8.59639E-08	1.96792E-07
FGFR2	19.48084259	-0.660966549	0.123440628	-5.354530004	8.57791E-08	1.96392E-07
CIITA	5860.552184	-0.366797552	0.068493111	-5.355247349	8.54395E-08	1.95637E-07
ZNF597	242.8152087	0.238570083	0.044548168	5.355328689	8.5401E-08	1.95572E-07
GRN	14147.29735	0.375281677	0.070075866	5.355362698	8.5385E-08	1.95558E-07
FAM170A	0.769022926	-0.679281536	0.126834191	-5.355665759	8.5242E-08	1.95253E-07
EMID1	1296.173407	-0.397204965	0.074160426	-5.356023224	8.50736E-08	1.94891E-07
ANO8	563.57995	-0.320697997	0.059873449	-5.356263998	8.49603E-08	1.94654E-07
ULK1	5281.945241	0.258485126	0.048255647	5.356577762	8.4813E-08	1.94339E-07
FAM78A	2665.849534	-0.211019924	0.039390747	-5.357093719	8.45713E-08	1.93808E-07
TASP1	372.8749519	-0.201548452	0.037620121	-5.357464234	8.43981E-08	1.93434E-07
PEX3	203.0131585	-0.21676637	0.040459585	-5.357602367	8.43336E-08	1.93309E-07
RSRC2	6895.311873	0.171667554	0.032037488	5.358333758	8.3993E-08	1.9255E-07
PPFIBP1	720.2565158	-0.342149276	0.063850832	-5.35857194	8.38823E-08	1.92319E-07
CD151	1361.117868	0.27374152	0.051080782	5.358992396	8.36874E-08	1.91895E-07
LANCL2	758.0055532	0.194929006	0.03637097	5.359466835	8.34679E-08	1.91414E-07
SNX25	169.0375812	0.425352501	0.079356195	5.360041559	8.32028E-08	1.90828E-07
CYP20A1	1143.637198	-0.154294842	0.028785857	-5.36009197	8.31796E-08	1.90797E-07
C2orf78	1.702734859	-0.78281756	0.146043414	-5.360170235	8.31436E-08	1.90737E-07

SH2D4B	29.92935018	-0.531360848	0.099127293	-5.360388981	8.30429E-08	1.90529E-07
CASP9	722.3122315	0.176531696	0.032932236	5.360452742	8.30136E-08	1.90484E-07
ATP10A	1130.560044	-0.422905672	0.078891913	-5.360570645	8.29595E-08	1.90382E-07
WNK4	38.58716577	-0.329679197	0.061494012	-5.361159368	8.26895E-08	1.89784E-07
KCNN3	21.88428076	-0.373972154	0.069729353	-5.363195532	8.17624E-08	1.87679E-07
ELOVL7	99.14612364	-0.517480322	0.09646888	-5.364220287	8.12996E-08	1.86638E-07
CD58	765.558756	0.240054126	0.044746592	5.364746629	8.10629E-08	1.86117E-07
CYP1A2	6.877123882	-0.456004733	0.084993934	-5.365144432	8.08845E-08	1.85729E-07
ADAM28	1609.303317	0.336429278	0.062705767	5.365204747	8.08574E-08	1.85688E-07
GFRA3	12.47320624	0.605067653	0.112771105	5.365449379	8.07479E-08	1.85459E-07
PLCL1	154.298875	0.447351345	0.083373324	5.365641201	8.06621E-08	1.85283E-07
PRR18	1.55451771	-0.69633176	0.129773822	-5.365733627	8.06208E-08	1.8521E-07
AC055839.2	89.54739672	0.313761513	0.058461442	5.366982077	8.0065E-08	1.83955E-07
KCNS1	144.1562244	-0.492846031	0.091817525	-5.367668417	7.9761E-08	1.83278E-07
FP565260.1	112.6303157	-0.279790426	0.052124472	-5.367736385	7.9731E-08	1.83231E-07
RASGRF2	92.77845638	0.385350624	0.071787967	5.367899915	7.96587E-08	1.83086E-07
NCF2	3333.143356	0.352477196	0.065661532	5.368092772	7.95736E-08	1.82912E-07
ANKRD13A	2837.771791	0.121916234	0.022708017	5.368862991	7.92346E-08	1.82154E-07
PDK4	112.7393609	0.58584497	0.109100253	5.369785616	7.88303E-08	1.81246E-07
FRA10AC1	332.3162277	-0.21306296	0.039677531	-5.369864443	7.87958E-08	1.81188E-07
FPGS	2612.932857	-0.157738018	0.02936728	-5.371216546	7.82072E-08	1.79855E-07
TRIM38	3765.926519	0.205932648	0.038339871	5.371239951	7.81971E-08	1.79853E-07
PLA2G2A	1.350732184	-1.019793163	0.189826998	-5.372224042	7.77714E-08	1.78895E-07
EXO5	98.62971888	-0.184549807	0.034352577	-5.372225982	7.77706E-08	1.78895E-07
SPEF2	267.0374621	-0.33452657	0.062266627	-5.372485804	7.76585E-08	1.78678E-07
FAM167B	10.1700379	-0.410114912	0.076335901	-5.372503719	7.76508E-08	1.78678E-07
LILRB2	938.9883386	0.463990516	0.086360581	5.372711854	7.75612E-08	1.78496E-07
RFC5	486.5390261	-0.146824995	0.02732451	-5.373380687	7.72739E-08	1.77855E-07
OXCT2	8.216403356	-0.484034377	0.090053006	-5.374994109	7.65852E-08	1.76291E-07
NTM	2.415993309	-1.027485172	0.191133659	-5.375741649	7.62681E-08	1.75582E-07
BCO1	1.247309692	-0.740035178	0.13766027	-5.375807969	7.624E-08	1.75538E-07
HLA-DOB	103.258451	0.305602336	0.056847387	5.375837837	7.62274E-08	1.75529E-07
ASPN	84.12742517	-0.384566006	0.071528551	-5.37639863	7.59905E-08	1.75004E-07
ARMCX6	295.7430927	-0.169388254	0.031492953	-5.378608163	7.50639E-08	1.72891E-07
AKR7A3	60.36092654	-0.312664087	0.058114389	-5.38014922	7.44241E-08	1.71437E-07
ROGDI	310.0478241	-0.302880587	0.056289679	-5.380748144	7.41769E-08	1.70888E-07
MICU1	1136.272632	-0.166687504	0.030975299	-5.381304162	7.39481E-08	1.70381E-07

MYPOP	168.123669	-0.223085599	0.041452836	-5.38167274	7.37968E-08	1.70052E-07
TRIM2	41.32822329	-0.41004579	0.076167334	-5.383486155	7.30569E-08	1.68367E-07
PPP2R5A	2740.07449	0.136604983	0.02537383	5.383695909	7.29717E-08	1.68191E-07
HAND1	0.720521544	-0.825961064	0.153405146	-5.384180953	7.27753E-08	1.67758E-07
KCNK15	1.081609146	-0.647431748	0.120227301	-5.385064324	7.24187E-08	1.66955E-07
LRFN1	251.118018	-0.248777384	0.046193291	-5.38557397	7.22138E-08	1.66503E-07
EIF4E3	1298.007189	0.363478687	0.067466119	5.387573717	7.14152E-08	1.64681E-07
BORCS7	343.2178958	0.239193799	0.044382615	5.389357952	7.07098E-08	1.63073E-07
TTC39A	201.9983852	-0.398427812	0.073918104	-5.390124888	7.04087E-08	1.62398E-07
BTBD17	1.42071155	-0.56467739	0.104729304	-5.391780201	6.97631E-08	1.60928E-07
RGS7	1.287387291	-0.898047413	0.166557108	-5.391828812	6.97442E-08	1.60903E-07
ATXN7	4690.539086	0.145311565	0.026949607	5.391973388	6.96881E-08	1.60793E-07
SELENBP1	1727.609596	-0.602314967	0.111699428	-5.392283368	6.95679E-08	1.60534E-07
CCDC134	1743.359173	0.192584545	0.035712647	5.392614725	6.94397E-08	1.60258E-07
TUBA4A	1400.157231	0.35826031	0.066421929	5.393705269	6.90194E-08	1.59306E-07
HOXB7	54.28906643	-0.789727827	0.146335049	-5.396710043	6.78739E-08	1.56681E-07
MANF	1028.806846	0.251059474	0.046509102	5.398071896	6.73609E-08	1.55515E-07
DAB2IP	58.73822102	-0.518319315	0.096007714	-5.398725701	6.71159E-08	1.54967E-07
KRT86	3.226720026	0.660810108	0.122386724	5.399361023	6.68787E-08	1.54438E-07
KLRG2	28.56931514	-0.571545477	0.105831242	-5.40053644	6.64419E-08	1.53447E-07
CBWD1	415.0979799	-0.18606714	0.034451896	-5.400780921	6.63514E-08	1.53257E-07
EID2	192.3591594	0.19440104	0.035985974	5.402133553	6.58529E-08	1.52123E-07
INPP5J	43.08382031	-0.646760402	0.11971686	-5.402417009	6.57489E-08	1.51901E-07
ADAMTS2	353.6269303	0.652128893	0.120705625	5.402638824	6.56676E-08	1.51731E-07
RELCH	1995.897633	-0.196333888	0.036338894	-5.402858067	6.55874E-08	1.51563E-07
ACTL7B	4.451694008	-0.461177682	0.085351786	-5.403257572	6.54414E-08	1.51244E-07
TMCO3	2544.913794	0.188348459	0.034855741	5.40365669	6.52959E-08	1.50925E-07
SUSD4	11.27595428	0.477140978	0.088292257	5.404108981	6.51313E-08	1.50563E-07
LONRF3	611.3342679	0.387389685	0.07168262	5.404234434	6.50858E-08	1.50475E-07
RORC	15.74625671	0.418268887	0.077393072	5.404474515	6.49987E-08	1.50292E-07
RFTN2	55.75093857	-0.384866736	0.071212595	-5.404475654	6.49983E-08	1.50292E-07
TCIM	2.539394514	-1.182353763	0.218761423	-5.40476353	6.48939E-08	1.50085E-07
SH2D7	5.401986275	-0.443717084	0.082089611	-5.405276957	6.47083E-08	1.49673E-07
MIF	1.495901896	-0.573778266	0.106134999	-5.406117408	6.44056E-08	1.48991E-07
GSTP1	7154.501056	-0.34183915	0.063227428	-5.406500991	6.42679E-08	1.4869E-07
TRDMT1	538.1287375	-0.219009696	0.040508128	-5.406561814	6.42461E-08	1.48657E-07
KDM7A	2197.731608	0.308346788	0.057029217	5.40682134	6.41531E-08	1.48459E-07

TAF7	2749.85263	0.189737158	0.03509182	5.406877113	6.41331E-08	1.48431E-07
GPATCH11	983.9938054	0.188295677	0.034823437	5.407153675	6.40342E-08	1.48219E-07
SLC50A1	774.201401	-0.17964683	0.033223275	-5.407258295	6.39968E-08	1.4815E-07
SLC39A13	760.7683048	0.171109728	0.031629299	5.409848873	6.3078E-08	1.4604E-07
EEF1E1	160.967384	-0.240403812	0.044425196	-5.411429381	6.25237E-08	1.44774E-07
HS3ST4	56.35729694	-1.022480603	0.188944239	-5.411546865	6.24826E-08	1.44696E-07
MGRN1	5166.787663	0.158904362	0.029359371	5.412389931	6.21891E-08	1.44033E-07
APOD	4.798109441	-0.509502454	0.094122959	-5.413158053	6.19228E-08	1.43434E-07
CD226	231.2023442	0.419418657	0.077475354	5.413575255	6.17786E-08	1.43117E-07
EIF2S3	10197.33061	0.136053583	0.025125731	5.414910437	6.13194E-08	1.4207E-07
RTCA	1202.657332	0.126271907	0.023318244	5.415155116	6.12356E-08	1.41892E-07
MNX1	9.075686302	-0.780920625	0.144204939	-5.415352814	6.1168E-08	1.41752E-07
CTDSP2	6570.482954	0.177669619	0.032803288	5.416213772	6.08743E-08	1.41089E-07
P2RX1	4401.973718	-0.190508936	0.035168537	-5.417027676	6.0598E-08	1.40465E-07
RASGEF1C	6.423591453	-0.450450104	0.083136215	-5.41821763	6.01961E-08	1.3955E-07
ZNF606	301.2262573	-0.255520512	0.047159152	-5.418259297	6.01821E-08	1.39534E-07
C11orf68	520.1135202	-0.171322067	0.031614581	-5.419083904	5.99052E-08	1.38908E-07
HEMGN	1819.201673	-0.584316002	0.107820909	-5.419319925	5.98262E-08	1.38741E-07
EFNB2	20.94584955	-0.489411626	0.090302716	-5.419677794	5.97065E-08	1.3848E-07
PCDHAC2	0.805817161	-0.834068529	0.153891467	-5.419849095	5.96494E-08	1.38364E-07
RGS7BP	0.923467504	-1.012248902	0.186759207	-5.420074949	5.95741E-08	1.38206E-07
ANKRD34B	8.878580564	-0.668770163	0.123384844	-5.420196995	5.95334E-08	1.38128E-07
VAX1	1.737720786	-0.840023571	0.154966488	-5.420678905	5.93731E-08	1.37772E-07
F5	411.4570117	0.499079445	0.092057276	5.421401396	5.91336E-08	1.37233E-07
JAKMIP3	35.50910192	-0.34246606	0.063169155	-5.421412676	5.91299E-08	1.37233E-07
ANO10	652.7629753	-0.213797088	0.039427461	-5.422542584	5.87572E-08	1.36392E-07
CPT2	196.2808672	-0.188202436	0.034702037	-5.423382948	5.84815E-08	1.35768E-07
EVA1A	1.329846916	-0.837208497	0.154357644	-5.423822725	5.83377E-08	1.3545E-07
KBTBD3	75.64439466	-0.241027149	0.044427604	-5.425166501	5.79005E-08	1.34451E-07
POU5F1	25.82916061	-0.236270367	0.043544313	-5.425975298	5.76389E-08	1.33859E-07
RGS1	1302.751654	0.434655675	0.080103697	5.426162456	5.75786E-08	1.33735E-07
EHD2	219.8051997	-0.539277752	0.09936977	-5.426979965	5.73156E-08	1.3314E-07
MPP3	44.92057423	-0.453210757	0.083509515	-5.4270553	5.72914E-08	1.331E-07
BLVRB	2235.487181	-0.447940834	0.082536283	-5.427199034	5.72453E-08	1.33008E-07
ZBTB4	2245.493898	0.196653448	0.036232727	5.427508832	5.7146E-08	1.32793E-07
UXS1	1419.981966	0.145819052	0.02686618	5.427606503	5.71148E-08	1.32737E-07
H3C8	475.3382046	-0.528797401	0.09742675	-5.427640772	5.71038E-08	1.32727E-07

OGDH	2390.565962	-0.242111921	0.044601834	-5.428295253	5.68949E-08	1.32257E-07
FGFBP1	0.803329798	-0.980428503	0.180602821	-5.428644452	5.67837E-08	1.32014E-07
OXNAD1	860.2189311	0.242521835	0.044665627	5.429719679	5.64426E-08	1.31237E-07
ZP2	2.404317422	-0.615935432	0.113433962	-5.429903208	5.63846E-08	1.31118E-07
ITIH6	1.269869796	-0.614174374	0.113093688	-5.430668876	5.61432E-08	1.30572E-07
BCO2	64.53952301	-0.299819038	0.055201036	-5.431402383	5.59129E-08	1.30051E-07
NNT	2904.720281	-0.15315425	0.028196389	-5.4316973	5.58206E-08	1.29852E-07
DDC	8.465553178	-0.477014529	0.087816307	-5.431958423	5.57389E-08	1.29678E-07
SNAI2	7.786675606	-0.806690378	0.148489206	-5.432653307	5.55222E-08	1.29189E-07
GIMAP6	288.576382	-0.417021862	0.076760862	-5.432740762	5.5495E-08	1.29141E-07
SNRPD2	3147.34394	-0.345476449	0.063585278	-5.433277341	5.53283E-08	1.28768E-07
OLFM3	7.127313851	-1.660556109	0.305616603	-5.43346171	5.52711E-08	1.28651E-07
GNL3	1472.388518	-0.140488218	0.0258559	-5.433507162	5.52571E-08	1.28633E-07
MTBP	290.3269234	-0.201426113	0.037064184	-5.434521773	5.49436E-08	1.27919E-07
OR3A2	13.63716898	-0.652909783	0.120089576	-5.436856409	5.42288E-08	1.26269E-07
LURAP1L	2.012554085	-1.011177853	0.185961506	-5.437565399	5.40135E-08	1.25783E-07
RFXAP	161.8187172	-0.209729688	0.038568744	-5.437814829	5.3938E-08	1.25622E-07
ADGRG2	51.74079625	-0.307029015	0.056461368	-5.437860026	5.39243E-08	1.25605E-07
SLC35D3	4.574379775	-0.588828506	0.108281162	-5.437958855	5.38944E-08	1.25551E-07
ID4	10.19295973	-0.728854	0.133954869	-5.441041498	5.297E-08	1.23412E-07
PSENEN	110.8750746	-0.419111452	0.077016482	-5.441841027	5.27327E-08	1.22874E-07
NXPE3	1389.232905	0.206490244	0.037942923	5.442127973	5.26478E-08	1.2269E-07
C6orf163	105.0297596	-0.305895867	0.056205336	-5.442470213	5.25467E-08	1.22469E-07
DHX9	9228.702518	0.148833632	0.027340707	5.443664425	5.21955E-08	1.21665E-07
SCNN1B	4.830725468	-0.505513855	0.092856057	-5.444059005	5.208E-08	1.2141E-07
PSMD4	1829.987982	-0.200880885	0.036895301	-5.444619815	5.19161E-08	1.21043E-07
ZNF622	599.0907025	0.190145182	0.034914663	5.445997858	5.15158E-08	1.20124E-07
SNRPE	1279.873804	-0.401293206	0.073683443	-5.44617881	5.14634E-08	1.20016E-07
LRRC8A	3065.525023	0.22946246	0.042131496	5.446340182	5.14168E-08	1.19921E-07
PTOV1	1290.791013	-0.146390781	0.026876133	-5.446869101	5.12642E-08	1.1958E-07
OR14A16	0.86616294	-0.872687842	0.160214271	-5.44700441	5.12252E-08	1.19503E-07
SLC13A2	1.19875056	-0.715887313	0.13141816	-5.447400195	5.11114E-08	1.19252E-07
OMD	64.20371368	-0.385024039	0.070665936	-5.448509715	5.07936E-08	1.18524E-07
NMT1	2149.494122	-0.133140947	0.024433105	-5.449202963	5.0596E-08	1.18078E-07
NDUFS6	604.7004453	-0.381056834	0.06992706	-5.449347265	5.0555E-08	1.17996E-07
STXBP3	1540.383374	0.155209682	0.028478095	5.450142783	5.03294E-08	1.17483E-07
C20orf173	6.317154115	-0.422408183	0.07750183	-5.450299446	5.02851E-08	1.17394E-07

HRH3	4.286392601	-0.493721541	0.090581401	-5.450584093	5.02047E-08	1.1722E-07
RBM11	14.74262946	0.484189235	0.088812949	5.451786471	4.98663E-08	1.16444E-07
GGCT	183.8648861	-0.284411705	0.05216791	-5.451851584	4.98481E-08	1.16415E-07
RPL36	14282.05393	-0.448064959	0.082158998	-5.453632222	4.93512E-08	1.15269E-07
AC013470.2	6.253629055	-0.821714914	0.150620316	-5.455538385	4.88247E-08	1.14052E-07
CACNG7	2.440948964	-0.667863369	0.122415638	-5.455703038	4.87795E-08	1.1396E-07
SCHIP1	99.90082051	-0.638113303	0.116947649	-5.456401305	4.85881E-08	1.13527E-07
STARD13	86.37656301	-0.440140468	0.080655898	-5.457015284	4.84205E-08	1.13149E-07
NFAM1	1890.010435	0.444062008	0.081373235	5.457101551	4.8397E-08	1.13107E-07
DUSP9	3.048763376	-0.588181955	0.107762077	-5.458153479	4.81112E-08	1.12453E-07
TP53I13	1044.260821	-0.220933765	0.040477488	-5.458188671	4.81016E-08	1.12444E-07
ARSI	225.1427046	0.658265461	0.120588984	5.458752843	4.79491E-08	1.12101E-07
PLPP2	19.63683336	-0.426176308	0.078058274	-5.459719825	4.76887E-08	1.11505E-07
RBPJL	3.492604822	-0.629226087	0.115247865	-5.459763507	4.76769E-08	1.11491E-07
C8orf74	2.396425528	-0.545332612	0.099878699	-5.459949082	4.76271E-08	1.11388E-07
GUCA2B	1.005180838	-1.081071166	0.197995925	-5.460067759	4.75953E-08	1.11327E-07
MRPL47	652.0064187	0.230751898	0.04226002	5.460288466	4.75362E-08	1.11202E-07
UBE2D2	2668.081644	0.163380308	0.029916916	5.461134644	4.73101E-08	1.10686E-07
FOXN1	3.085053119	-0.586027455	0.107286795	-5.462251491	4.70133E-08	1.10005E-07
NDST4	1.427334286	-1.202214264	0.220085532	-5.462486588	4.69511E-08	1.09873E-07
LGALS4	45.78610306	-0.296002873	0.054187063	-5.462611498	4.69181E-08	1.09808E-07
FSIP1	15.37625202	-0.305193565	0.055863862	-5.463166244	4.67716E-08	1.09479E-07
ATP5PD	641.5945334	-0.345205241	0.063183811	-5.463507738	4.66817E-08	1.09281E-07
GPX3	359.1674777	0.320674182	0.058692445	5.463636431	4.66478E-08	1.09215E-07
CCDC85B	819.2003132	-0.287344101	0.052583991	-5.464478761	4.64269E-08	1.08711E-07
PGS1	2693.03165	0.196780011	0.036009993	5.464594501	4.63966E-08	1.08653E-07
DDX58	579.0653823	-0.258041312	0.047218784	-5.4648021	4.63424E-08	1.08539E-07
SIN3A	5622.681449	0.141512135	0.025894741	5.464898529	4.63172E-08	1.08493E-07
TECPR2	748.8491964	-0.229717708	0.042030877	-5.465451237	4.61731E-08	1.08168E-07
PTPRA	2273.093492	-0.117151852	0.021434187	-5.4656542	4.61203E-08	1.08057E-07
ALPK2	16.52994261	0.514338839	0.094069716	5.467634655	4.56081E-08	1.0687E-07
NKIRAS1	564.5426707	0.217487648	0.039774279	5.468047494	4.5502E-08	1.06634E-07
H2AX	1441.565213	-0.268553319	0.049111123	-5.468278969	4.54426E-08	1.06508E-07
WNT10B	63.92001727	-0.519602791	0.095019793	-5.468363771	4.54209E-08	1.0647E-07
OR51E2	1.562172637	-0.623511317	0.114015108	-5.46867277	4.53418E-08	1.06297E-07
CIB1	2028.796878	0.216197915	0.039533218	5.468765859	4.5318E-08	1.06254E-07
VTN	61.20123869	0.35683778	0.065246675	5.469056965	4.52436E-08	1.06092E-07

GPR55	36.11981733	-0.347825023	0.063588552	-5.469931514	4.5021E-08	1.05583E-07
TMEM63C	36.64975332	-0.543469069	0.099340291	-5.4707819	4.48054E-08	1.0509E-07
AL117339.4	40.25102294	-0.359031355	0.065623335	-5.471092769	4.47269E-08	1.04918E-07
DKK1	1.565499296	-0.901204545	0.164715898	-5.471266326	4.46831E-08	1.04828E-07
H2AC4	361.1828523	-0.453263348	0.082830359	-5.472188643	4.44511E-08	1.04297E-07
ACAT1	612.2258262	-0.200351594	0.036610155	-5.472568875	4.43558E-08	1.04085E-07
HIVEP2	1299.084737	0.347710948	0.063530096	5.473168947	4.42058E-08	1.03746E-07
FAM221A	167.2868598	-0.359133649	0.06555349	-5.478482492	4.28989E-08	1.00691E-07
LAMTOR1	2048.885185	-0.270052758	0.049290305	-5.478821016	4.28169E-08	1.0051E-07
DEFB118	1.584433211	-1.293688808	0.236103092	-5.479338697	4.26918E-08	1.00229E-07
TAOK2	1711.700538	-0.145597266	0.026571237	-5.47950642	4.26514E-08	1.00146E-07
SEPTIN12	9.675221691	-0.431975265	0.078830857	-5.479773821	4.2587E-08	1.00007E-07
USHBP1	43.67471426	-0.273596421	0.049927548	-5.479868978	4.25641E-08	9.99648E-08
LRFN3	33.09123104	-0.466593586	0.085139347	-5.480351937	4.24481E-08	9.97043E-08
IL2	1.186876266	0.758481884	0.138370156	5.481542439	4.21634E-08	9.90474E-08
TSGA13	1.987588922	-0.497197231	0.090699567	-5.4818038	4.21011E-08	9.89131E-08
GARS1	4230.782994	0.181435584	0.03309571	5.482148169	4.20192E-08	9.87325E-08
ITGB2	13574.76531	0.347321428	0.063349743	5.482602002	4.19115E-08	9.84913E-08
EVA1C	51.70776143	-0.433314794	0.079029877	-5.482923777	4.18353E-08	9.83241E-08
SPIDR	2207.280335	-0.201744525	0.036792095	-5.483365989	4.17308E-08	9.80903E-08
SPINK9	2.24889885	-0.559771044	0.102085286	-5.483366574	4.17307E-08	9.80903E-08
ITGA1	49.52159525	-0.492826354	0.089872031	-5.483645471	4.16649E-08	9.79589E-08
WFIKKN2	3.135704319	-0.659562274	0.120273723	-5.483843491	4.16183E-08	9.7861E-08
SHANK3	2142.556579	-0.519918005	0.094801696	-5.484269023	4.15183E-08	9.76375E-08
SMO	106.4733722	-0.43205774	0.07875347	-5.486205712	4.10659E-08	9.65852E-08
BTAF1	7915.263318	0.240423149	0.043817138	5.486965993	4.08896E-08	9.61822E-08
RAB3IL1	130.4589463	-0.503207288	0.091700942	-5.487482186	4.07703E-08	9.59131E-08
SYT13	1.82428551	-0.679226486	0.123773228	-5.487668828	4.07273E-08	9.58234E-08
H6PD	2879.196482	-0.265920316	0.048452665	-5.488249588	4.05936E-08	9.55204E-08
CAMKV	38.45122855	-0.517972217	0.094359463	-5.489351058	4.03413E-08	9.49381E-08
FBXO46	613.7418271	-0.13186868	0.024018755	-5.490237912	4.01393E-08	9.4474E-08
PMPCB	2142.212306	0.112369502	0.020466552	5.490397264	4.01031E-08	9.44002E-08
PPIAL4H	0.935019589	-0.584986737	0.106521014	-5.491749632	3.97971E-08	9.36913E-08
LGSN	28.85854188	-0.794107929	0.144596271	-5.491897696	3.97638E-08	9.3624E-08
NLRX1	705.3736126	-0.195611334	0.035616405	-5.492169562	3.97026E-08	9.34912E-08
WDR38	33.07960253	-0.38777459	0.07060263	-5.4923533	3.96613E-08	9.34052E-08
SLC6A2	0.937894865	-0.945952258	0.1722258	-5.492511922	3.96257E-08	9.33325E-08

TEDDM1	7.456473992	-0.427852441	0.077897205	-5.492526197	3.96225E-08	9.33325E-08
ISLR	5.3852742	-0.678911494	0.123602942	-5.492680678	3.95878E-08	9.32658E-08
VWDE	46.62610751	-0.769161909	0.140021661	-5.493163706	3.94797E-08	9.30222E-08
NME8	100.4507307	-0.407702067	0.074168925	-5.496939159	3.8644E-08	9.10642E-08
TAFA3	3.430381037	-0.597787442	0.108747778	-5.49700833	3.86288E-08	9.10394E-08
FBXO10	86.2851887	-0.242674508	0.044140256	-5.497804774	3.84548E-08	9.06402E-08
FOXF1	2.864359526	-0.690680521	0.125627058	-5.497864343	3.84418E-08	9.06205E-08
ATF4	14926.51005	0.203912794	0.03708933	5.497882997	3.84378E-08	9.06205E-08
H4C4	801.988688	-0.500000344	0.090921055	-5.499280063	3.81345E-08	8.99177E-08
DIPK2B	29.64196168	-0.617422256	0.112248891	-5.500475348	3.78769E-08	8.9321E-08
CHRM3	15.2013174	-0.529856263	0.096328858	-5.500493568	3.7873E-08	8.9321E-08
LRRC31	2.890142263	-0.60012298	0.10909226	-5.50105921	3.77516E-08	8.90471E-08
PWP2	60.46738595	-0.426087208	0.07744689	-5.501669681	3.76211E-08	8.875E-08
CABYR	54.91198535	-0.520917814	0.094677711	-5.502011097	3.75483E-08	8.85889E-08
RIPK3	393.3304354	-0.298267127	0.054184119	-5.504696491	3.69805E-08	8.72597E-08
IL12RB2	464.2082269	-0.556681223	0.101118666	-5.505227158	3.68693E-08	8.70077E-08
DVL1	730.0592182	-0.183842144	0.033376459	-5.508138084	3.62649E-08	8.55918E-08
GUCY2F	5.208230375	-0.514236545	0.093358459	-5.508194455	3.62533E-08	8.55747E-08
DAW1	2.966364928	-0.802191945	0.145613135	-5.509063059	3.60749E-08	8.51638E-08
ZNF207	12753.30119	0.123049407	0.022333591	5.509611375	3.59627E-08	8.49092E-08
SLC4A9	11.7695121	-0.373706612	0.067826028	-5.509781747	3.59279E-08	8.48373E-08
PLGRKT	213.0565623	-0.264679466	0.048029324	-5.510788865	3.57229E-08	8.43635E-08
TMEM107	1108.72984	-0.36576076	0.066369205	-5.511001031	3.56799E-08	8.4272E-08
PRC1	182.0265726	-0.285327509	0.051766972	-5.51176743	3.55248E-08	8.39159E-08
MIB1	2721.314819	-0.20127944	0.036514835	-5.512264762	3.54245E-08	8.36892E-08
IER3IP1	358.7276295	0.263801491	0.047854689	5.512552598	3.53666E-08	8.35625E-08
CEACAM1	429.5219132	-0.523094737	0.094889007	-5.512701138	3.53368E-08	8.3502E-08
FBXO34	1430.415055	0.189737743	0.03441771	5.51279397	3.53182E-08	8.3468E-08
KL	16.38544465	-0.627382959	0.113767385	-5.514611761	3.49551E-08	8.26199E-08
RUNX3	3222.103174	0.39997626	0.072492774	5.517463842	3.43927E-08	8.13005E-08
PNPLA5	1.480702491	-0.594806156	0.107796897	-5.517841148	3.43189E-08	8.1136E-08
PRDM2	8524.769245	0.265202836	0.048057894	5.518403142	3.42094E-08	8.08867E-08
ELOVL2	17.0404694	-0.692302288	0.125424058	-5.51969295	3.39593E-08	8.0305E-08
BAD	133.0570618	-0.219446334	0.039754513	-5.520035701	3.38931E-08	8.01582E-08
ETV1	17.56410008	-0.784265098	0.142075541	-5.520057088	3.3889E-08	8.01581E-08
ZNF77	120.99381	-0.219282715	0.039724643	-5.520067607	3.38869E-08	8.01581E-08
CADPS	5.644996378	-0.880642966	0.159511366	-5.520879087	3.37308E-08	7.98033E-08

OR2Z1	0.596466408	-0.889031594	0.161028396	-5.520961609	3.37149E-08	7.97755E-08
STRIP2	194.4742752	-0.430315835	0.077928126	-5.521957933	3.35243E-08	7.93339E-08
MED12	5277.453867	-0.155780525	0.028210412	-5.522093255	3.34985E-08	7.92824E-08
RNF146	435.2418033	-0.199289278	0.036085678	-5.522669603	3.33887E-08	7.90322E-08
ZNF813	167.3504615	-0.249210089	0.04511454	-5.523941709	3.31477E-08	7.84713E-08
LZTFL1	481.3367215	-0.17068845	0.030898325	-5.524197479	3.30995E-08	7.83666E-08
MIEF1	1610.084193	0.152168143	0.027543111	5.524726077	3.3E-08	7.81405E-08
GTSF1L	2.596538599	-0.691784617	0.125174352	-5.526568393	3.26555E-08	7.73341E-08
TRABD2B	2.723606267	-0.753709721	0.136347979	-5.527839316	3.24199E-08	7.67854E-08
KRTAP5-4	0.670348215	-0.851962658	0.154116131	-5.528056361	3.23798E-08	7.66997E-08
MAP7D3	556.6026096	-0.224398265	0.040590669	-5.528321468	3.23309E-08	7.65932E-08
ASB5	1.705081803	-0.8742201	0.15812396	-5.52870103	3.22611E-08	7.6437E-08
SRD5A2	2.774486502	-0.532779614	0.096348122	-5.529735344	3.20714E-08	7.59969E-08
TMEM270	0.809120969	-0.74607169	0.134919818	-5.529741321	3.20703E-08	7.59969E-08
PPP2R5D	737.51503	-0.118824387	0.021485426	-5.530464674	3.19384E-08	7.56999E-08
TMEM256-PLSCR3	3.365758716	-0.385239369	0.069651802	-5.530931828	3.18534E-08	7.55077E-08
RPH3AL	98.54420857	-0.380728501	0.068828507	-5.531552529	3.17409E-08	7.525E-08
FAM71F1	13.46860766	-0.715260038	0.129244271	-5.534172095	3.12702E-08	7.41431E-08
NDUFS1	2097.280906	-0.14000546	0.02529489	-5.534930691	3.11351E-08	7.38319E-08
TAS2R39	1.208549903	-0.597621132	0.107953375	-5.53591894	3.09601E-08	7.34256E-08
PLXNA2	181.8997796	-0.407860813	0.073673855	-5.536031892	3.09401E-08	7.33872E-08
NUP107	1204.794011	-0.174086151	0.03144391	-5.536402754	3.08747E-08	7.32409E-08
RECK	223.7134726	-0.37281412	0.067313637	-5.53846346	3.05137E-08	7.23933E-08
MPP5	1198.370419	0.197743626	0.03570243	5.538660062	3.04795E-08	7.23208E-08
SFI1	1685.308244	-0.198741267	0.035879649	-5.539108522	3.04015E-08	7.21446E-08
MIXL1	11.58774053	-0.449461788	0.081140616	-5.539294726	3.03692E-08	7.20767E-08
ATAD3B	1408.095719	0.23803182	0.042960992	5.540650011	3.01351E-08	7.15297E-08
CCDC80	75.89145221	-0.349452107	0.063050862	-5.542384291	2.98381E-08	7.08333E-08
GPR146	60.61418013	-0.366844288	0.066180506	-5.543086752	2.97186E-08	7.05581E-08
DTNBP1	629.2970872	-0.141772843	0.025575488	-5.543309303	2.96808E-08	7.0477E-08
DUSP16	723.8113402	0.244250759	0.044059698	5.54363218	2.96261E-08	7.03557E-08
KLRC4-KLRK1	11.81631059	0.538446812	0.097124712	5.543870373	2.95858E-08	7.02685E-08
TYMP	1510.564553	0.376608498	0.067910307	5.545675133	2.92822E-08	6.95559E-08
STT3A	2591.963263	-0.167081176	0.030123402	-5.546557339	2.91349E-08	6.92144E-08
UIMC1	2750.212849	0.177879238	0.032059545	5.548401729	2.88293E-08	6.84967E-08
DENND2A	11.62323618	-0.401339551	0.072325784	-5.549052221	2.87222E-08	6.82506E-08
SLC39A11	1203.711601	-0.296974544	0.053513152	-5.549561808	2.86386E-08	6.80603E-08

GK5	1093.972859	0.218690041	0.039394146	5.551333385	2.83499E-08	6.73822E-08
TRIML2	1.289834487	-1.236044055	0.222649498	-5.551524091	2.8319E-08	6.73169E-08
FGF6	2.081544868	-0.840099329	0.151327434	-5.551533545	2.83174E-08	6.73169E-08
MYADML2	2.875670168	-0.481745231	0.086772257	-5.551834737	2.82687E-08	6.72137E-08
OR13A1	6.557182226	-0.538882206	0.097030493	-5.55374078	2.7962E-08	6.64926E-08
CCRL2	214.7529517	0.339554658	0.061134607	5.554213506	2.78864E-08	6.6321E-08
E2F3	2145.639944	0.166385126	0.029955942	5.554328006	2.78682E-08	6.62856E-08
ANXA2R	616.9184809	-0.346297669	0.062341548	-5.554845492	2.77857E-08	6.60975E-08
DDX54	2014.506826	-0.151094558	0.027199332	-5.555083481	2.77479E-08	6.60155E-08
TM6SF2	1.654090561	-0.633884431	0.114048844	-5.558008366	2.7287E-08	6.49269E-08
SLC7A4	3.987090159	-0.477083817	0.085825346	-5.558775335	2.71674E-08	6.46502E-08
ALKAL1	1.012521102	-0.891263697	0.160332256	-5.558854589	2.71551E-08	6.46287E-08
PRR32	1.821444343	-2.039095432	0.3668093	-5.5590069	2.71314E-08	6.45802E-08
PYGB	2081.06474	0.185722744	0.033404306	5.559844332	2.70015E-08	6.42789E-08
FHL2	384.0599759	-0.397692016	0.071525428	-5.560148674	2.69545E-08	6.41747E-08
OSBP2	1449.36153	-0.550659027	0.099031383	-5.560449713	2.6908E-08	6.40719E-08
H2BC9	1324.865543	-0.486033883	0.087402701	-5.560856531	2.68454E-08	6.39305E-08
KIAA2012	20.57828646	-0.362170887	0.065124138	-5.561238889	2.67866E-08	6.37983E-08
TTC37	2402.449476	-0.186250445	0.033489707	-5.561423509	2.67583E-08	6.37386E-08
NTRK2	15.81816694	-0.608532656	0.109417978	-5.561541769	2.67402E-08	6.37032E-08
FXYD2	24.08551239	0.481539505	0.086581405	5.561696561	2.67165E-08	6.36544E-08
DEFB124	1.007392034	-0.735232846	0.132183456	-5.562215343	2.66371E-08	6.34732E-08
BCLAF3	378.594539	-0.202274664	0.036361965	-5.56280886	2.65467E-08	6.32653E-08
DHCR24	463.4825382	-0.458739219	0.082461933	-5.563042302	2.65112E-08	6.31884E-08
TIMM50	1393.527729	-0.176683872	0.031759944	-5.563103923	2.65018E-08	6.31737E-08
TNKS1BP1	946.3796497	0.262665785	0.047214257	5.563272622	2.64762E-08	6.31204E-08
COL4A1	40.78565051	-0.562127327	0.101039356	-5.563449232	2.64494E-08	6.30642E-08
C9orf43	175.9638043	-0.392862946	0.070599546	-5.56466673	2.62654E-08	6.26331E-08
KLHL22	481.0948664	-0.195832423	0.035187921	-5.565330932	2.61655E-08	6.24026E-08
CLECL1	289.222774	-0.491919876	0.088388425	-5.565433197	2.61502E-08	6.23736E-08
MTRNR2L1	38.80055898	-0.836357333	0.150255061	-5.566250667	2.60279E-08	6.20894E-08
IFI27L2	267.3203741	-0.464576107	0.083449272	-5.56716792	2.58913E-08	6.17711E-08
APBB3	622.7098611	-0.209425036	0.037615113	-5.567576972	2.58306E-08	6.16338E-08
CLDN5	41.96341939	-0.524877234	0.094264306	-5.568144045	2.57467E-08	6.14411E-08
AP2B1	7034.467007	-0.157561607	0.028296064	-5.568322473	2.57203E-08	6.13857E-08
HIP1	2404.018916	-0.328807853	0.059049352	-5.568356644	2.57153E-08	6.13812E-08
CFAP300	0.96374754	-0.773165122	0.138848324	-5.568415212	2.57067E-08	6.1368E-08

AP3M1	1692.820895	0.122184103	0.021941054	5.568743523	2.56583E-08	6.126E-08
ACOT8	517.065029	-0.142633344	0.025608945	-5.569668976	2.55224E-08	6.0943E-08
SLC26A7	8.598523453	-0.575192879	0.103272154	-5.569680278	2.55207E-08	6.0943E-08
PRR36	18.96037016	-0.457292945	0.082083368	-5.571079202	2.53166E-08	6.04664E-08
ACRBP	320.5491559	-0.320583596	0.057542994	-5.571201186	2.52989E-08	6.04315E-08
CFAP94	23.20015733	-0.29603054	0.053135733	-5.571213992	2.5297E-08	6.04315E-08
NKX2-4	2.136072128	-0.59528232	0.10684525	-5.571443951	2.52637E-08	6.0362E-08
COPA	7614.189111	0.149848781	0.026893718	5.571887894	2.51994E-08	6.02157E-08
HSPB6	14.44271104	-0.569204582	0.102152232	-5.572120854	2.51657E-08	6.01426E-08
STAP1	331.8367234	0.353235559	0.063384102	5.572936237	2.50481E-08	5.9869E-08
GSTM2	435.4794339	-0.347242706	0.062305522	-5.573225206	2.50066E-08	5.9777E-08
NOC2L	1699.032358	-0.178724453	0.032066055	-5.573633935	2.4948E-08	5.96441E-08
ZNRD2	528.7288804	-0.271007293	0.048621321	-5.573836553	2.4919E-08	5.95821E-08
MRPS27	1741.728267	-0.157350523	0.028223296	-5.575200122	2.47245E-08	5.91244E-08
SRRM4	2.61613226	-0.522386595	0.093685633	-5.575952006	2.4618E-08	5.88768E-08
SERGEF	214.8310471	-0.188676657	0.033836662	-5.576101324	2.45969E-08	5.88335E-08
GPHB5	0.773642763	-1.020675582	0.183037928	-5.576306463	2.45679E-08	5.87714E-08
KCNC4	686.0817418	-0.320561955	0.057482603	-5.57667779	2.45155E-08	5.86533E-08
MAX	3427.268049	0.164071856	0.029420618	5.576764324	2.45033E-08	5.86313E-08
ASIC2	1.893356403	-0.765645272	0.137284315	-5.57707757	2.44593E-08	5.8533E-08
TAF8	1730.386414	-0.158871705	0.028483954	-5.577586141	2.43879E-08	5.83693E-08
TMEM234	184.8143952	-0.195296477	0.035013097	-5.577812126	2.43562E-08	5.83007E-08
AC119396.1	874.8631803	-0.313268614	0.056153204	-5.578819895	2.42156E-08	5.79711E-08
APTX	800.4626443	-0.120929972	0.021668931	-5.580800086	2.39415E-08	5.73219E-08
ZNF676	1.429932507	-0.737648618	0.132146789	-5.582039665	2.37714E-08	5.69217E-08
CDKN2AIP	976.8718077	0.200415507	0.035901277	5.582406103	2.37214E-08	5.68088E-08
TMEM120A	447.1269566	0.273676249	0.049005768	5.584572149	2.34276E-08	5.61122E-08
FGD5	180.1388855	-0.679410135	0.121624834	-5.586113586	2.32207E-08	5.56235E-08
PAK4	392.626367	-0.189915417	0.033995799	-5.586437866	2.31774E-08	5.55266E-08
SMAD9	27.78983976	-0.402869249	0.072107172	-5.587089885	2.30906E-08	5.53253E-08
SLC35A5	438.9117738	-0.229371339	0.041051562	-5.587396168	2.30499E-08	5.52347E-08
DCAF5	3010.227384	0.145399952	0.026021851	5.587609904	2.30216E-08	5.51735E-08
TBC1D1	4425.510291	0.166916903	0.029872612	5.587623223	2.30198E-08	5.51735E-08
PDE8A	787.7821914	0.202012194	0.036147979	5.588478262	2.29068E-08	5.49118E-08
SLITRK6	4.197238498	-1.458084414	0.260892501	-5.588832203	2.28602E-08	5.48067E-08
USP39	2572.895297	0.143635077	0.025696712	5.589628525	2.27556E-08	5.45627E-08
BTBD16	0.865569422	-1.211417666	0.216722473	-5.589718735	2.27438E-08	5.4541E-08

FP565260.6	50.16926541	0.269315902	0.048175736	5.590280968	2.26702E-08	5.43714E-08
MPST	1226.346807	-0.232883523	0.041636935	-5.593195649	2.22928E-08	5.34726E-08
MEF2A	2045.774136	0.222012415	0.03968452	5.594433725	2.21343E-08	5.3099E-08
EIF3B	5678.057032	-0.133351225	0.023834699	-5.594835719	2.20831E-08	5.29826E-08
FBXO4	206.923615	-0.184679226	0.033008035	-5.594977849	2.2065E-08	5.29457E-08
GAGE10	0.849977748	-0.670345815	0.119809302	-5.595106613	2.20486E-08	5.29129E-08
MAP3K15	66.80228792	-0.427320353	0.076370316	-5.595372299	2.20149E-08	5.28384E-08
FBXO39	20.99503277	-0.509192921	0.090992513	-5.595987014	2.1937E-08	5.2658E-08
TTBK2	1459.908302	-0.169180847	0.030229665	-5.596517461	2.187E-08	5.25036E-08
PPDPFL	1.543408975	-1.832669873	0.327457707	-5.596661295	2.18519E-08	5.24665E-08
NDNF	8.854821973	-1.028107273	0.183685635	-5.597102207	2.17964E-08	5.23398E-08
BHLHB9	1.425356122	-0.607163574	0.108469857	-5.597532743	2.17424E-08	5.22164E-08
FAT2	71.89178576	-0.419306412	0.074903247	-5.597973805	2.16872E-08	5.20901E-08
SPACA5B	0.68217624	-0.652367893	0.11653361	-5.59810935	2.16702E-08	5.20558E-08
ZSCAN31	334.8908268	-0.34374863	0.06139717	-5.598769968	2.15878E-08	5.18643E-08
CDCA8	542.7185006	-0.289292582	0.051660099	-5.599923092	2.14447E-08	5.15268E-08
IQGAP1	8416.701903	0.262596262	0.046888784	5.600406784	2.13849E-08	5.13895E-08
ITPR1	6978.247893	-0.26665577	0.047609841	-5.600854019	2.13298E-08	5.12634E-08
SLA	2292.572126	0.362887508	0.064786947	5.601244141	2.12819E-08	5.11544E-08
RSPH6A	20.14097223	-0.37403198	0.066771849	-5.601641771	2.12331E-08	5.10434E-08
GRM3	4.563216511	-0.775727643	0.138481055	-5.601687847	2.12275E-08	5.10361E-08
CASP4	2156.434329	0.235374449	0.042012667	5.602463847	2.11326E-08	5.08143E-08
CDH5	29.45428297	-0.542936009	0.096908518	-5.6025623	2.11206E-08	5.07917E-08
VPS53	2385.281696	-0.17562608	0.031347197	-5.602608801	2.11149E-08	5.07843E-08
PPP1R8	730.7176335	0.16413291	0.029295331	5.602698493	2.1104E-08	5.07643E-08
FBXO42	2321.884661	0.188348102	0.033611796	5.603630951	2.09907E-08	5.0498E-08
GHRL	267.9770676	-0.265062895	0.047290672	-5.604972073	2.08289E-08	5.01147E-08
CENPV	388.9766979	-0.26606361	0.047467964	-5.60511952	2.08111E-08	5.00782E-08
CLSTN3	1654.563831	-0.239937798	0.042803944	-5.605506763	2.07647E-08	4.99725E-08
SGCE	48.04292733	-0.367623564	0.065577788	-5.605915869	2.07157E-08	4.98607E-08
PREPL	611.8858416	-0.249932145	0.044580448	-5.606317457	2.06677E-08	4.97514E-08
DIDO1	4579.470149	-0.188621305	0.033643374	-5.606491933	2.06469E-08	4.97074E-08
FAM166C	1.421548329	-0.680816007	0.121423996	-5.60693132	2.05945E-08	4.95875E-08
OR4D1	6.850387746	-0.395660978	0.070557943	-5.60760364	2.05147E-08	4.94014E-08
IL2RG	853.0305704	-0.401053863	0.071518937	-5.607659739	2.05081E-08	4.93915E-08
TSC1	1979.914435	-0.172706138	0.030794594	-5.608326579	2.04292E-08	4.92076E-08
GUCY1A2	3.825045562	-0.763639613	0.136155793	-5.608572335	2.04002E-08	4.91439E-08

ELMOD1	1.748861807	-0.619565279	0.110456208	-5.609148572	2.03324E-08	4.89866E-08
ZNF621	1204.856921	-0.194872124	0.034739355	-5.609549245	2.02854E-08	4.88793E-08
ARHGAP20	9.570644289	-0.470693118	0.08390333	-5.609945647	2.0239E-08	4.87735E-08
LMNTD2	50.56928637	-0.298435925	0.053194912	-5.610234377	2.02053E-08	4.86982E-08
TENT4A	1869.526604	0.206596886	0.036824253	5.610348244	2.0192E-08	4.86721E-08
PRRT4	400.5541888	0.551994086	0.098381725	5.610738007	2.01466E-08	4.85686E-08
C11orf98	1.739509172	-0.46391436	0.082675229	-5.611286069	2.00828E-08	4.8421E-08
OR2M2	0.399728697	-1.104257659	0.196771139	-5.611888335	2.00131E-08	4.82587E-08
TMEM179B	438.5351862	-0.264377875	0.04710879	-5.612071051	1.99919E-08	4.82137E-08
AC022415.2	16.15383243	-0.315239309	0.056169379	-5.612298255	1.99657E-08	4.81563E-08
PADI3	15.82300782	-0.753382672	0.134235903	-5.61237834	1.99565E-08	4.814E-08
TNR	25.82575083	-0.650833411	0.115963138	-5.612416341	1.99521E-08	4.81353E-08
FAM228B	637.4755692	-0.174389935	0.031066777	-5.613390058	1.98401E-08	4.7871E-08
LILRA6	337.6161316	0.442268241	0.078786941	5.613471413	1.98307E-08	4.78544E-08
DHX37	1554.543607	-0.1691278	0.030123426	-5.614494148	1.97138E-08	4.75781E-08
POLR2J	712.273158	-0.382561149	0.068130352	-5.615135369	1.96408E-08	4.74078E-08
DQX1	23.60848861	-0.367761259	0.065483875	-5.616058256	1.95363E-08	4.71613E-08
NT5C1A	4.255798268	0.661706688	0.117800793	5.617166696	1.94114E-08	4.68656E-08
HAUS3	808.6308725	0.209350549	0.037269497	5.617208856	1.94067E-08	4.686E-08
SLC25A39	5595.647912	-0.409648764	0.072925427	-5.617365282	1.93891E-08	4.68234E-08
PERP	14.14566199	0.488474234	0.086956296	5.617468297	1.93776E-08	4.68012E-08
AJM1	204.1007116	-0.253360651	0.045101377	-5.617581243	1.93649E-08	4.67764E-08
ZNF85	163.780867	-0.26409935	0.047012049	-5.617694967	1.93522E-08	4.67514E-08
IL13RA1	1217.447469	0.427136379	0.076030158	5.617986217	1.93196E-08	4.66785E-08
AP3D1	3870.516723	-0.138384165	0.024629499	-5.618635012	1.92472E-08	4.65093E-08
SIRT1	1263.421986	0.199086199	0.035431294	5.61893672	1.92136E-08	4.64339E-08
GRAMD2B	134.5843308	0.312634307	0.05563462	5.6194202	1.91599E-08	4.63099E-08
AIFM2	109.9013106	-0.370631523	0.065953398	-5.619597061	1.91403E-08	4.62682E-08
ATP6V1B1	1.586929904	-0.580614674	0.103318299	-5.619669321	1.91323E-08	4.62546E-08
EPHX3	15.96771351	-0.467777277	0.083238915	-5.619694525	1.91295E-08	4.62535E-08
ANGPTL2	26.11520848	-0.409667475	0.072878165	-5.621264934	1.89564E-08	4.58406E-08
ZNF395	1605.15096	0.277900494	0.049435262	5.621503477	1.89303E-08	4.5783E-08
TMEM134	403.5349118	-0.192616926	0.034256076	-5.622854318	1.87828E-08	4.5432E-08
EIF4ENIF1	941.3535278	-0.134160701	0.023858386	-5.623209545	1.87442E-08	4.53442E-08
ST8SIA3	1.433973492	-0.845974376	0.150439403	-5.62335638	1.87282E-08	4.53112E-08
FHL5	0.668527712	-1.113365839	0.197940308	-5.624755499	1.85771E-08	4.49511E-08
GYPA	442.4295945	-0.65669328	0.116749059	-5.624827195	1.85694E-08	4.4938E-08

PRXL2C	633.2945241	0.297608439	0.052901407	5.625718765	1.84737E-08	4.4712E-08
CILP2	224.0323467	0.413670549	0.073530736	5.625818167	1.84631E-08	4.46918E-08
CFAP97D2	6.954113464	0.500637368	0.088975435	5.626691974	1.83698E-08	4.44716E-08
SURF2	159.540405	-0.272073685	0.048351902	-5.626948945	1.83425E-08	4.44109E-08
LRRC37A3	319.8530691	-0.225131117	0.040003979	-5.627718038	1.82609E-08	4.42189E-08
GAP43	1.405814528	-1.188167495	0.211103574	-5.628362753	1.81928E-08	4.40594E-08
DEXI	362.0429397	-0.285211722	0.050671861	-5.628601715	1.81676E-08	4.40039E-08
LDLRAD4	2086.607823	-0.353754074	0.062849362	-5.628602474	1.81676E-08	4.40039E-08
PLA2G5	2.304319023	-0.656777776	0.116680212	-5.62887028	1.81394E-08	4.39463E-08
COX18	553.0722079	-0.158525848	0.028162357	-5.628997869	1.8126E-08	4.39192E-08
CHL1	25.92631034	-0.664332836	0.117995567	-5.630150823	1.80052E-08	4.3632E-08
BST2	1731.746283	-0.380716174	0.067618181	-5.630381751	1.79811E-08	4.35791E-08
GK	454.0549445	0.306267077	0.054379043	5.632079191	1.7805E-08	4.31575E-08
MDK	485.8609848	-0.456817314	0.081108228	-5.63219448	1.77931E-08	4.3134E-08
GATA5	3.797401698	-0.864137096	0.153413241	-5.632741268	1.77367E-08	4.30028E-08
CD8B2	7.077822734	0.512152728	0.090923478	5.632788574	1.77319E-08	4.29963E-08
TMEM14C	1088.404959	-0.315318344	0.055976045	-5.633094354	1.77005E-08	4.29254E-08
GCNA	105.0889406	-0.33570541	0.059595068	-5.633107253	1.76991E-08	4.29254E-08
TMEM132E	6.389430804	-0.69048266	0.122575072	-5.63314098	1.76957E-08	4.29244E-08
TEX43	1.042667159	-0.748816209	0.132922345	-5.633486287	1.76603E-08	4.28439E-08
CLTC	11932.11744	0.186628825	0.033128312	5.633514453	1.76574E-08	4.28422E-08
MYO1C	637.8423143	0.376783991	0.066870686	5.63451657	1.7555E-08	4.25991E-08
COL3A1	40.93119473	-0.760789422	0.13501449	-5.634872401	1.75188E-08	4.25165E-08
SPEM3	5.377738432	-0.600017224	0.106482574	-5.634886534	1.75174E-08	4.25165E-08
ZEB1	1455.68837	0.358177997	0.063553866	5.635817581	1.7423E-08	4.22944E-08
RRM1	1430.189303	-0.1709375	0.03033017	-5.635890001	1.74157E-08	4.22819E-08
WDR26	13086.00835	0.234226701	0.041551625	5.637004541	1.73034E-08	4.20145E-08
A4GNT	3.155657872	-0.611307778	0.10843256	-5.637677279	1.72359E-08	4.18559E-08
CKLF	29.02752823	-0.341095185	0.06050229	-5.637723577	1.72313E-08	4.18498E-08
ZNF780A	210.5859484	-0.343323496	0.060887042	-5.638695612	1.71343E-08	4.16195E-08
OR2H2	10.54033128	-0.598165204	0.106081782	-5.638717534	1.71321E-08	4.16194E-08
OR2M5	0.389387785	-1.131402124	0.200636928	-5.63905227	1.70989E-08	4.15437E-08
C1orf216	427.8134393	-0.245095451	0.043459355	-5.639647699	1.70398E-08	4.14054E-08
LMO1	3.285353699	-0.777763023	0.137900331	-5.640037407	1.70013E-08	4.1317E-08
MT-ATP6	135497.0037	-0.64393184	0.114170612	-5.640083998	1.69967E-08	4.13109E-08
ORC5	243.5530672	-0.18189749	0.032249794	-5.640268331	1.69785E-08	4.12718E-08
NUMBL	390.8235933	-0.260444734	0.046170123	-5.640979913	1.69085E-08	4.11067E-08

DNAJC5B	60.51862128	-0.671809559	0.119078549	-5.64173452	1.68346E-08	4.0932E-08
OR13J1	1.022930332	-0.77928676	0.138112777	-5.642394409	1.67701E-08	4.07805E-08
SLC25A38	1047.781209	-0.177602129	0.031475179	-5.642609113	1.67492E-08	4.07347E-08
SRP72	4623.730144	0.154500897	0.027380971	5.642637592	1.67465E-08	4.0733E-08
EIF2AK1	5421.979345	-0.276591532	0.048999002	-5.644840149	1.65335E-08	4.022E-08
APH1A	3004.040893	0.195188084	0.034570162	5.646143232	1.64087E-08	3.99214E-08
TECTB	1.203019105	-0.781766774	0.138450004	-5.646563762	1.63686E-08	3.98289E-08
KNL1	967.3132642	-0.286102849	0.050667402	-5.646684768	1.63571E-08	3.98058E-08
INTS2	804.0020882	-0.197534852	0.0349793	-5.647192845	1.63089E-08	3.96933E-08
IQCK	40.01946404	-0.262626377	0.046502711	-5.647549886	1.62751E-08	3.96159E-08
LHX9	8.951134975	0.768468565	0.136066153	5.647756966	1.62555E-08	3.95732E-08
GOLGA8O	177.7365872	-0.440083519	0.077920139	-5.647879048	1.62439E-08	3.955E-08
CNOT10	1172.919343	-0.103595842	0.018341938	-5.648031324	1.62296E-08	3.95199E-08
EEF1A1	279923.5248	0.211292228	0.037409009	5.64816422	1.6217E-08	3.94943E-08
CACNG3	2.361221088	-0.72102037	0.127652375	-5.648311454	1.62031E-08	3.94654E-08
DYRK1A	9320.592652	0.187360828	0.033166453	5.649106637	1.61284E-08	3.92882E-08
NISCH	3960.286146	-0.180209333	0.031891907	-5.650628942	1.59862E-08	3.89467E-08
PLS3	33.26847378	-0.745639067	0.131923733	-5.652046432	1.58549E-08	3.86316E-08
APBA1	79.88970767	-0.726512322	0.128530633	-5.652444929	1.58181E-08	3.85469E-08
CDK7	582.7693018	0.146433251	0.025905262	5.652645096	1.57997E-08	3.85068E-08
FBXO41	1679.070042	-0.26077287	0.046122366	-5.653935264	1.56815E-08	3.82235E-08
BNC1	0.993571006	-0.771218393	0.136393527	-5.654362143	1.56426E-08	3.81334E-08
TMEM192	567.8831778	-0.166037237	0.029363747	-5.654497669	1.56303E-08	3.8108E-08
COX8C	2.562181614	-0.789545455	0.139630015	-5.654553963	1.56252E-08	3.81003E-08
BICRAL	1155.752274	-0.174471802	0.030848754	-5.655716367	1.55198E-08	3.7848E-08
ZNF322	81.27054274	-0.260170716	0.045998326	-5.65609099	1.54859E-08	3.77703E-08
REV1	1765.95937	-0.143806359	0.025419727	-5.657273856	1.53796E-08	3.75156E-08
NUDT5	1004.956107	-0.223651382	0.039529883	-5.657780019	1.53344E-08	3.74099E-08
CNTN3	0.991093919	-2.105311143	0.372039398	-5.658839245	1.524E-08	3.71844E-08
WFDC12	3.742793904	-0.876332245	0.154844739	-5.659425361	1.51881E-08	3.70622E-08
VAV1	2996.807356	-0.218246788	0.038551854	-5.661122989	1.50386E-08	3.6702E-08
CNMD	11.23143648	-0.424435684	0.074972568	-5.661213069	1.50307E-08	3.66873E-08
SPPL2B	2171.441355	-0.182759039	0.032280321	-5.661623909	1.49947E-08	3.66041E-08
DYNLT3	419.3974706	0.299149213	0.052830661	5.662416573	1.49256E-08	3.64399E-08
SLC6A11	3.135001745	-0.598136516	0.105619673	-5.663116531	1.48648E-08	3.6296E-08
FBLN5	189.9146433	0.625990158	0.110530083	5.663527442	1.48292E-08	3.62137E-08
DNAJC7	4349.559024	0.172807508	0.030506016	5.664702518	1.4728E-08	3.59709E-08

CNTN2	53.09750154	-0.400969818	0.07077143	-5.665701812	1.46424E-08	3.57663E-08
C1GALT1C1L	2.9070107	-0.683912667	0.120708199	-5.665834414	1.46311E-08	3.57431E-08
SHISA4	49.08452897	-0.573038729	0.101126843	-5.666534325	1.45715E-08	3.5602E-08
ZG16	5.745379651	-0.421030618	0.074297634	-5.666810599	1.4548E-08	3.55491E-08
GAS1	27.03751197	-0.711581285	0.125564476	-5.667058925	1.45269E-08	3.5502E-08
SMIM10L2B	3.486595026	-0.579457863	0.102235892	-5.667851615	1.44599E-08	3.53426E-08
RAB3A	63.5718081	-0.347390327	0.061284597	-5.668476979	1.44072E-08	3.52183E-08
CA2	2529.550287	-0.512885998	0.090477489	-5.668658601	1.4392E-08	3.51854E-08
AK6	147.3289089	-0.267183953	0.047129456	-5.669149948	1.43508E-08	3.5089E-08
PTPA	1568.889322	-0.160158642	0.028247787	-5.66977665	1.42984E-08	3.49653E-08
PEX11G	135.3544619	-0.284186448	0.050120789	-5.670031453	1.42771E-08	3.49177E-08
CIB2	60.77518397	-0.42734983	0.075343542	-5.6720167	1.41126E-08	3.45197E-08
SFPQ	32364.52012	0.199603986	0.035190121	5.672159696	1.41008E-08	3.44952E-08
FAM83H	215.6151333	-0.451185431	0.079535152	-5.672780117	1.40499E-08	3.43747E-08
TNNI1	3.625221268	-0.521949497	0.092003235	-5.673164617	1.40183E-08	3.43019E-08
DDB1	7093.138908	-0.142009216	0.025030223	-5.673509793	1.39901E-08	3.42371E-08
RNF14	580.8272963	-0.234812316	0.04138211	-5.674247118	1.393E-08	3.40943E-08
DNAJC13	2479.057921	-0.263563595	0.046443497	-5.674930062	1.38745E-08	3.39628E-08
ARHGEF7	5345.37692	0.264391751	0.046587019	5.675223647	1.38508E-08	3.39088E-08
RNF4	3391.021345	0.153885239	0.027107679	5.676813536	1.37227E-08	3.35995E-08
ACTR10	1016.007159	0.146529112	0.025809703	5.677287839	1.36847E-08	3.35107E-08
TRIM55	1.676293271	-0.709114924	0.12489822	-5.677542267	1.36644E-08	3.34651E-08
ACO1	582.1625744	-0.194184861	0.034201752	-5.677629054	1.36574E-08	3.34523E-08
AZIN2	85.64915777	-0.420079431	0.073985462	-5.677864539	1.36387E-08	3.34105E-08
PRXL2A	136.8844511	-0.546285465	0.096211674	-5.67795406	1.36315E-08	3.33972E-08
SLAMF6	211.1185541	-0.375135595	0.066068616	-5.677969601	1.36303E-08	3.33972E-08
SHH	18.34166751	-0.455495619	0.080214165	-5.678493592	1.35886E-08	3.33004E-08
NUDT2	246.6877628	-0.211974298	0.037329121	-5.678523711	1.35862E-08	3.32987E-08
MPEG1	4449.476766	0.598734666	0.105431183	5.678914425	1.35552E-08	3.32269E-08
SLC7A5	6063.35923	0.371075725	0.06533946	5.679197926	1.35328E-08	3.3176E-08
USP12	2300.509491	0.238735318	0.042036273	5.679269318	1.35271E-08	3.31663E-08
GLCCI1	449.2588334	-0.284805479	0.050146171	-5.679506052	1.35084E-08	3.31246E-08
PTPRJ	2249.400846	0.375512772	0.066112629	5.679894669	1.34778E-08	3.30536E-08
C10orf71	1.66675473	-0.751348746	0.132275641	-5.680174687	1.34557E-08	3.30036E-08
DNTTIP2	2045.134001	0.249470274	0.043912698	5.681050964	1.3387E-08	3.28391E-08
SLC2A1	5643.408446	-0.435568506	0.076669559	-5.681114037	1.3382E-08	3.28311E-08
SCARA3	33.72758742	-0.749135277	0.131838993	-5.682198108	1.32975E-08	3.26277E-08

LYPD3	54.05208235	0.449635713	0.079126112	5.682519972	1.32724E-08	3.25704E-08
PHACTR3	205.567118	-0.909446958	0.16003406	-5.682833744	1.32481E-08	3.25148E-08
TC2N	429.8026176	0.452854339	0.079661767	5.684713745	1.31032E-08	3.21631E-08
SOX8	17.63816021	0.597843855	0.105151212	5.68556315	1.30382E-08	3.20077E-08
ZNF195	1247.494497	-0.176828659	0.031087148	-5.688159623	1.28416E-08	3.15289E-08
TRIM58	3717.50568	-0.496964073	0.087366092	-5.688294659	1.28314E-08	3.15079E-08
TBC1D17	790.4897787	-0.19046975	0.033480904	-5.688907037	1.27855E-08	3.13991E-08
TAF5	178.6183475	-0.20366826	0.035798631	-5.689275034	1.2758E-08	3.13354E-08
ATP6V0A2	3064.690177	-0.206613287	0.036308716	-5.690459746	1.26698E-08	3.11227E-08
MYO18B	1123.316042	-0.853529766	0.149987245	-5.690682354	1.26533E-08	3.1086E-08
LRRN4CL	15.57530557	-0.493923388	0.086791244	-5.690935709	1.26345E-08	3.10438E-08
ZBTB44	3005.085253	-0.166030888	0.029172663	-5.691317542	1.26063E-08	3.09784E-08
LGI3	2.716725431	-0.554389962	0.097405503	-5.691567147	1.25879E-08	3.0937E-08
SIGLEC9	247.7654632	0.486894039	0.085527328	5.692847508	1.24938E-08	3.07097E-08
FANCD2OS	15.22298734	-0.320697326	0.056332179	-5.692968539	1.24849E-08	3.06918E-08
SERBP1	5891.988904	-0.158538642	0.027847741	-5.693052236	1.24788E-08	3.06806E-08
ZNF624	178.391535	0.237162234	0.041650927	5.694044498	1.24065E-08	3.05065E-08
EVL	1910.310149	-0.275128588	0.048305358	-5.695612243	1.2293E-08	3.02313E-08
TK1	1089.56494	-0.290078457	0.050925368	-5.696148442	1.22544E-08	3.01402E-08
GM2A	2356.281531	0.265885411	0.046677581	5.696212345	1.22498E-08	3.01327E-08
SDR9C7	0.95056587	-0.711109907	0.124833932	-5.696447232	1.2233E-08	3.0095E-08
DPPA2	0.875882348	-1.237393102	0.217209008	-5.696785396	1.22087E-08	3.00392E-08
OR56A3	0.982516504	-1.213400279	0.212987835	-5.697040307	1.21905E-08	2.99981E-08
EEA1	644.3631798	-0.26430804	0.046388203	-5.697742548	1.21404E-08	2.98786E-08
TMEM215	1.283812078	-0.833453902	0.146271811	-5.697980324	1.21235E-08	2.98407E-08
DDX39B	7411.33918	0.170069071	0.029838903	5.699575145	1.20106E-08	2.95666E-08
NPC2	1824.799776	0.352361242	0.061821156	5.699687088	1.20028E-08	2.95509E-08
DLGAP1	14.14199772	-0.412424954	0.072358669	-5.699731081	1.19997E-08	2.9547E-08
GLS2	19.13776274	-0.313498319	0.054985668	-5.701455172	1.18789E-08	2.92534E-08
IGSF21	2.240035565	-0.723990714	0.126980906	-5.701571505	1.18708E-08	2.92371E-08
LINGO3	701.8976275	-0.306863235	0.053810093	-5.702707796	1.17919E-08	2.90464E-08
PLEKHG5	69.9837998	-0.497258424	0.087191997	-5.703028275	1.17697E-08	2.89955E-08
FOXA2	1.002645725	-1.238062005	0.217073731	-5.703416984	1.17429E-08	2.89331E-08
ENTPD2	7.291129627	-0.550887627	0.096585135	-5.703648138	1.1727E-08	2.88975E-08
MXD1	6702.280903	0.27993917	0.049080506	5.703673303	1.17253E-08	2.88968E-08
CHCHD1	231.5777615	-0.357614283	0.062698083	-5.70375144	1.17199E-08	2.88872E-08
IL1RL2	7.95120954	0.612670284	0.107386767	5.705268	1.1616E-08	2.86348E-08

RASGRF1	41.77221277	-0.474774843	0.083205546	-5.706048051	1.15629E-08	2.85075E-08
BHMT	3.138890528	-0.622322638	0.109062315	-5.706119851	1.15581E-08	2.84991E-08
STK39	523.3960579	-0.20963908	0.036736134	-5.706617934	1.15243E-08	2.84195E-08
KCNJ10	6.502728204	-0.45080313	0.078972422	-5.708361496	1.14069E-08	2.81335E-08
ST6GAL1	1746.912724	-0.269954123	0.047290822	-5.708383	1.14055E-08	2.81335E-08
CLK4	1233.180652	0.202113149	0.035403412	5.708860668	1.13735E-08	2.80582E-08
GLT8D1	536.9814876	-0.189100276	0.033119892	-5.709568053	1.13263E-08	2.79453E-08
GRAMD4	1762.534426	-0.268012413	0.046940479	-5.7096225	1.13227E-08	2.79399E-08
WNT1	17.22812422	0.485982145	0.085114372	5.70975424	1.13139E-08	2.79218E-08
CHSY1	4075.458729	0.216154175	0.037852525	5.710429529	1.12691E-08	2.78147E-08
VIL1	37.75926179	-0.417274483	0.073069966	-5.710615546	1.12568E-08	2.77879E-08
LHFPL6	119.2503044	-0.557033765	0.097538409	-5.710917045	1.12369E-08	2.77422E-08
SPATA4	1.25845767	-0.842374456	0.147489397	-5.711423834	1.12035E-08	2.76631E-08
STOML3	4.54022351	-0.856059898	0.149856342	-5.712537011	1.11304E-08	2.74862E-08
COL28A1	9.377239806	-0.500036903	0.087504094	-5.714440082	1.10066E-08	2.71838E-08
TMEM179	4.672940307	-0.679039599	0.118820827	-5.714819663	1.0982E-08	2.71266E-08
CEACAM16	2.929228696	-0.677272644	0.118475241	-5.716575352	1.08692E-08	2.68514E-08
DAP	2891.555926	0.225384368	0.039422902	5.717092276	1.08362E-08	2.67732E-08
ECHDC1	632.9407196	-0.223376763	0.039071346	-5.717150401	1.08325E-08	2.67675E-08
SQOR	469.3146272	0.275933661	0.048261694	5.717446707	1.08137E-08	2.67242E-08
S100A11	2255.797991	0.544312241	0.095200698	5.717523655	1.08088E-08	2.67155E-08
MYO5A	3912.694263	0.219983785	0.03846545	5.718996802	1.07155E-08	2.64883E-08
NCAPH	618.2227153	-0.245445283	0.042913977	-5.719471828	1.06856E-08	2.64177E-08
OAF	726.9363751	0.394599451	0.068988527	5.7197837	1.0666E-08	2.63726E-08
AHR	3166.342268	0.349904904	0.061165326	5.720641536	1.06123E-08	2.6243E-08
H4C8	1062.547636	-0.353065542	0.0617109	-5.72128329	1.05722E-08	2.61474E-08
PCDH9	256.9071942	-0.662805703	0.115834839	-5.721989239	1.05284E-08	2.60423E-08
MRPS17	139.7855803	-0.255140638	0.04457861	-5.723387031	1.04421E-08	2.58321E-08
MAN2C1	3361.237311	-0.19920009	0.034801681	-5.723864001	1.04128E-08	2.57629E-08
SMARCAD1	1005.883677	-0.193743506	0.033844462	-5.72452618	1.03723E-08	2.56658E-08
HSPB7	24.18058762	-0.571736824	0.09986623	-5.72502662	1.03418E-08	2.55935E-08
LEF1	833.2013633	0.481765725	0.08414822	5.725203971	1.0331E-08	2.557E-08
PRIMA1	0.736180351	-0.893437121	0.156047806	-5.725406507	1.03186E-08	2.55428E-08
PLCXD3	0.918375414	-1.078430916	0.188351002	-5.725644698	1.03042E-08	2.55102E-08
SLC36A4	1156.412747	0.29988214	0.052374644	5.725712254	1.03001E-08	2.55033E-08
ABCF3	1778.697177	0.171413353	0.029934769	5.726229399	1.02687E-08	2.54289E-08
STXBP5L	53.13896355	-0.707445421	0.123538698	-5.726508625	1.02519E-08	2.53903E-08

TERB1	7.928659564	-0.375415819	0.065554038	-5.726814568	1.02334E-08	2.53478E-08
SLFNL1	176.8966588	-0.360681735	0.062978432	-5.727067526	1.02182E-08	2.53132E-08
STBD1	43.35672985	-0.34254982	0.059807801	-5.727510737	1.01915E-08	2.52504E-08
STH	0.769831533	-1.135020834	0.198098377	-5.7295817	1.00679E-08	2.49472E-08
SEMA3A	30.90607484	-0.661968984	0.115522369	-5.730223435	1.00298E-08	2.48562E-08
UCN2	18.43511678	0.538515382	0.093977993	5.730228569	1.00295E-08	2.48562E-08
RSF1	2731.554327	0.189899817	0.033137533	5.730656375	1.00043E-08	2.47991E-08
CMTM2	45.1421607	-0.511043732	0.089154748	-5.732097797	9.9196E-09	2.45923E-08
GPHN	261.8829075	-0.388432805	0.067751248	-5.733219877	9.85418E-09	2.44332E-08
RUNDC3A	198.1238893	-0.544840483	0.095031337	-5.7332718	9.85116E-09	2.44288E-08
NUFIP2	11277.37741	0.200728202	0.035010346	5.733396697	9.8439E-09	2.44139E-08
AP1AR	286.4203046	-0.182955052	0.03190249	-5.734820364	9.76157E-09	2.42128E-08
RNF144B	692.2501467	0.459131325	0.080059352	5.734886864	9.75775E-09	2.42064E-08
POLR1C	467.9961008	-0.186399782	0.032502586	-5.734921661	9.75574E-09	2.42045E-08
MYC	1887.276201	-0.307420158	0.053603698	-5.735055056	9.74807E-09	2.41885E-08
PPP1R15B	12045.76581	0.225932898	0.039391574	5.735564061	9.71883E-09	2.4119E-08
NRAP	1.651282212	-0.752496932	0.131181298	-5.736312581	9.676E-09	2.40158E-08
PSME2	1649.751128	-0.27768336	0.04840633	-5.736509225	9.66478E-09	2.3991E-08
ECD	2174.75189	0.152802545	0.026632667	5.737410559	9.6135E-09	2.38667E-08
ADAMTS6	225.5889065	-0.463939725	0.080844521	-5.73866623	9.54251E-09	2.36935E-08
STING1	4213.415461	-0.245110889	0.042711061	-5.738815306	9.53411E-09	2.36756E-08
TEX15	13.44870524	-0.835717211	0.145579056	-5.740641797	9.43184E-09	2.34246E-08
SKI	3978.290341	0.298749113	0.052040746	5.740676992	9.42988E-09	2.34227E-08
LPAR6	912.7949975	0.399546565	0.069595393	5.740991589	9.41238E-09	2.33822E-08
ANKZF1	1049.622967	-0.196792023	0.034273524	-5.741808753	9.36706E-09	2.32726E-08
CSH2	0.654839488	-1.10724423	0.192817174	-5.742456485	9.33129E-09	2.31867E-08
IL13RA2	1.32566063	-1.343626875	0.23397589	-5.742586877	9.3241E-09	2.31717E-08
LRRC10B	3.66711161	-0.61156731	0.106493532	-5.742764831	9.3143E-09	2.31503E-08
TRMT44	712.8789755	-0.222073029	0.038668906	-5.742935417	9.30492E-09	2.313E-08
UBE2E1	1594.871754	0.185859445	0.032361654	5.743199765	9.2904E-09	2.30968E-08
VWCE	294.1269138	-0.346214889	0.060274095	-5.744008123	9.24613E-09	2.29897E-08
HEPHL1	10.07634786	-0.516231414	0.089869685	-5.744221904	9.23446E-09	2.29636E-08
HMGCR	1300.325755	0.207415829	0.036108173	5.744290255	9.23073E-09	2.29572E-08
GGA2	6260.663875	0.243073284	0.042314513	5.744442435	9.22244E-09	2.29395E-08
ZFAND5	11438.46512	0.196856915	0.034267384	5.744731416	9.2067E-09	2.29032E-08
ZNF136	1335.886771	0.214668049	0.037366389	5.744950335	9.1948E-09	2.28765E-08
HNRNPH3	4126.082258	0.185944959	0.032359079	5.746299563	9.12176E-09	2.26977E-08

PSG6	0.767145921	-1.13657371	0.197731472	-5.748066803	9.02696E-09	2.24647E-08
STX5	2813.521168	0.190780807	0.033189089	5.74829891	9.01458E-09	2.24367E-08
CDK20	81.35606537	-0.470299255	0.081807609	-5.748844928	8.98552E-09	2.23672E-08
NXNL2	5.368605101	-0.536862225	0.09338564	-5.748873432	8.984E-09	2.23663E-08
TFPT	177.5564992	-0.231403415	0.040250338	-5.749104919	8.97171E-09	2.23386E-08
CD302	421.5669091	0.289795405	0.050406311	5.749188874	8.96726E-09	2.23303E-08
BIN3	1420.041225	0.228002979	0.039658199	5.749201506	8.96659E-09	2.23303E-08
KRT15	2.633925656	-0.492048994	0.085577874	-5.74972213	8.93902E-09	2.22657E-08
MYCBP	277.5830239	-0.217085016	0.037754814	-5.749863214	8.93157E-09	2.22499E-08
BARHL1	7.055511021	-0.387456799	0.067352534	-5.75266851	8.78456E-09	2.18865E-08
CKS2	835.6418794	-0.356856671	0.062015262	-5.754336239	8.69829E-09	2.16743E-08
GABRA3	0.725131108	-1.50530151	0.261499582	-5.756420333	8.59163E-09	2.14113E-08
CES3	70.20985239	-0.407518771	0.070770599	-5.758306128	8.49622E-09	2.11762E-08
ZSCAN2	282.5166422	-0.217682989	0.037802693	-5.758398964	8.49155E-09	2.11672E-08
LAX1	353.1745838	0.353621267	0.06140765	5.758586569	8.48212E-09	2.11464E-08
MRPL39	300.4636743	0.17230732	0.029915813	5.759740453	8.42434E-09	2.1005E-08
BMP5	5.461879017	-0.959665619	0.166603763	-5.76016771	8.40304E-09	2.09546E-08
RCC1	1618.00162	-0.206733374	0.035884796	-5.761029608	8.36024E-09	2.08505E-08
CBLL1	1576.611179	0.216802253	0.037628744	5.761612823	8.33139E-09	2.07812E-08
ZEB2	20094.68111	0.258259283	0.044823216	5.761730326	8.32559E-09	2.07694E-08
PNMT	49.5737681	-0.567970835	0.098569491	-5.762136234	8.30559E-09	2.07222E-08
GMPR	742.8168596	-0.4560116	0.079134435	-5.76249264	8.28807E-09	2.06811E-08
KCTD16	3.32196194	-0.560365568	0.097236502	-5.762913663	8.26741E-09	2.06322E-08
ZC3H12B	80.51665565	-0.37183627	0.064522215	-5.762918507	8.26717E-09	2.06322E-08
MYH4	1.441221335	-1.019168018	0.176838835	-5.763259045	8.2505E-09	2.05952E-08
KCNH5	2.93355308	-0.795162012	0.137969454	-5.763319269	8.24756E-09	2.05905E-08
PDZK1	31.26355352	-0.287403009	0.049864353	-5.763696766	8.22912E-09	2.05471E-08
ZDHHC9	647.284638	-0.202649383	0.035144449	-5.766184662	8.10862E-09	2.02488E-08
ZNF20	66.99069834	-0.262371228	0.045498701	-5.766565191	8.09034E-09	2.02057E-08
MFSD13A	421.4670705	-0.236774609	0.041057703	-5.766874271	8.07553E-09	2.01713E-08
CAPSL	1.85897558	-0.912793105	0.158233287	-5.76865413	7.99071E-09	1.9962E-08
GATA3	232.0210567	0.48846395	0.08467017	5.76902056	7.97336E-09	1.99212E-08
MED14	2870.521202	0.158195934	0.027420076	5.769346982	7.95793E-09	1.98852E-08
TAF7L	7.798360434	-0.442025695	0.076603528	-5.770304637	7.91284E-09	1.9775E-08
VRK3	1220.615256	-0.133181117	0.023079959	-5.770422567	7.9073E-09	1.97637E-08
COPS8	1128.92359	0.156955064	0.027199658	5.77047935	7.90464E-09	1.97596E-08
FBXW11	1852.572607	0.178460048	0.030923265	5.771061045	7.87739E-09	1.9694E-08

COL20A1	101.3262042	0.611314316	0.105924714	5.771215161	7.87019E-09	1.96785E-08
AK4	297.335019	0.480267903	0.08320942	5.771797241	7.84305E-09	1.96131E-08
GNB4	2207.953314	0.177548981	0.030757951	5.772457921	7.81235E-09	1.95389E-08
POLR3GL	184.9155389	-0.329109515	0.05701344	-5.772490039	7.81086E-09	1.95376E-08
CDK8	404.115583	-0.198621649	0.034403763	-5.773253696	7.77552E-09	1.94517E-08
CPHXL	8.977278509	-0.480053072	0.083135413	-5.774351225	7.72502E-09	1.93278E-08
IL1RL1	471.8620396	0.654281972	0.113304465	5.774547121	7.71603E-09	1.93078E-08
VHL	1917.459638	0.151688285	0.026267372	5.774779613	7.70539E-09	1.92837E-08
FOXK1	4698.964223	0.21064972	0.03647635	5.774967008	7.69682E-09	1.92647E-08
ATP6V1E1	1759.952466	0.163253172	0.028268867	5.775016366	7.69456E-09	1.92615E-08
SEPTIN11	2230.348536	-0.29634479	0.051313593	-5.775171328	7.68748E-09	1.92462E-08
BTC	2.297760636	-1.051831177	0.182118588	-5.775528958	7.67117E-09	1.92079E-08
DNAH14	445.359076	-0.335679665	0.058110613	-5.776563801	7.62416E-09	1.90926E-08
FOXS1	1.242723885	-0.737024839	0.12758243	-5.776852168	7.61111E-09	1.90623E-08
H4C2	1027.363205	-0.522454728	0.090437826	-5.776949226	7.60672E-09	1.90538E-08
KIZ	666.431586	-0.203351372	0.035197188	-5.77748913	7.58236E-09	1.89952E-08
CD300LG	5.12022883	-0.538223911	0.093150684	-5.777992073	7.55974E-09	1.8941E-08
MED22	898.1143891	-0.157614056	0.027278092	-5.778045476	7.55734E-09	1.89374E-08
LHB	7.632799134	-0.467262717	0.080864065	-5.778372848	7.54265E-09	1.8903E-08
AKAP5	142.7987576	0.343883723	0.059506975	5.778880957	7.51991E-09	1.88484E-08
RTF1	3822.492008	0.18726388	0.032401306	5.779516509	7.49156E-09	1.87798E-08
DUXB	21.32550366	-0.420079134	0.072684069	-5.779521417	7.49134E-09	1.87798E-08
SLC4A2	1133.405581	-0.225811271	0.039064822	-5.780424907	7.45122E-09	1.86834E-08
ZDHHC11	77.98155645	-0.36324183	0.062838972	-5.780518326	7.44708E-09	1.86754E-08
FOXD4L3	0.695454078	-0.768677545	0.132974499	-5.780638783	7.44175E-09	1.86645E-08
WFIKKN1	49.51311613	-0.323488258	0.055954715	-5.781251127	7.41471E-09	1.8599E-08
ANKS1A	1412.017874	-0.24481458	0.042344797	-5.781456036	7.40568E-09	1.85788E-08
PYY	2.049031789	-0.616204574	0.106582913	-5.781457432	7.40562E-09	1.85788E-08
TMPRSS15	1.023676574	-1.152144125	0.199279732	-5.78154193	7.4019E-09	1.8574E-08
AMELX	1.677873278	-1.124878133	0.194559708	-5.781660254	7.3967E-09	1.85633E-08
CCNK	1546.926041	0.178837465	0.030929095	5.782175894	7.37405E-09	1.85089E-08
CHST9	1.523937965	-0.681441182	0.117851978	-5.782178563	7.37394E-09	1.85089E-08
H3C3	952.325047	-0.52407974	0.090632042	-5.782499547	7.35988E-09	1.8478E-08
OAZ3	13.40000622	-0.297928414	0.051522264	-5.782517938	7.35907E-09	1.8478E-08
FBLIM1	21.79263286	-0.403235774	0.069732605	-5.782600174	7.35547E-09	1.84717E-08
OPA3	802.1947128	-0.193317164	0.033425829	-5.783466481	7.31768E-09	1.83792E-08
TFDP1	3053.907674	-0.298088978	0.051513766	-5.786588714	7.18302E-09	1.80433E-08

SBNO2	3388.026968	0.235225186	0.040645443	5.787246238	7.15497E-09	1.79751E-08
FAM240B	1.181579791	-0.680263568	0.117529638	-5.788017217	7.12221E-09	1.78951E-08
STAB1	13957.91487	0.658549525	0.113737571	5.790079017	7.03533E-09	1.76791E-08
C2orf68	1521.429866	-0.182317315	0.031484937	-5.790620264	7.0127E-09	1.76245E-08
DDX46	2069.364325	-0.139956896	0.024169519	-5.790636328	7.01203E-09	1.76245E-08
ADAMTS15	23.20049995	0.780097864	0.134676252	5.792393639	6.93903E-09	1.74438E-08
TAS2R40	2.436126291	-0.50041018	0.086372759	-5.79361118	6.88888E-09	1.732E-08
ENTPD6	915.9402989	-0.17067348	0.029458723	-5.7936483	6.88736E-09	1.73184E-08
EEF1AKMT3	152.5594907	-0.244775027	0.04224653	-5.793967551	6.87427E-09	1.72877E-08
RNF183	10.62766236	-0.380390332	0.065650498	-5.794172861	6.86587E-09	1.72688E-08
ATF2	2441.123346	0.17731857	0.030595132	5.795646553	6.80585E-09	1.712E-08
YTHDC1	7030.375459	0.227177587	0.039194598	5.796145382	6.78564E-09	1.70714E-08
PSEN1	2844.528519	0.159674248	0.02754324	5.79722105	6.74228E-09	1.69645E-08
TMEM171	1.083202344	-0.813851905	0.140348126	-5.798808505	6.67877E-09	1.68068E-08
SWT1	382.1583039	-0.270227167	0.046591234	-5.799957237	6.63318E-09	1.66943E-08
AL391987.2	7.757285261	-0.631417744	0.108864842	-5.800015229	6.63089E-09	1.66906E-08
PPM1J	23.39032776	-0.482425031	0.083174995	-5.800120932	6.62671E-09	1.66823E-08
CHDH	28.334402	-0.57701818	0.099463718	-5.801293072	6.58055E-09	1.65682E-08
C1orf52	847.5091187	0.169789511	0.029264882	5.801817637	6.55999E-09	1.65185E-08
C1orf74	50.63635108	-0.271759979	0.04684025	-5.801847321	6.55883E-09	1.65177E-08
HHIPL1	24.49926081	-0.68973224	0.118875432	-5.802142863	6.54727E-09	1.64908E-08
STRADB	1811.905589	-0.426617316	0.073522649	-5.802529174	6.5322E-09	1.64549E-08
SELENOW	668.7800981	-0.297838417	0.051327114	-5.802750088	6.5236E-09	1.64354E-08
GTDC1	728.1005764	-0.178580664	0.030775098	-5.802764983	6.52302E-09	1.64354E-08
VAMP4	558.5455663	0.175744257	0.030285982	5.802825074	6.52068E-09	1.64323E-08
SCN3B	29.17945725	-0.500658733	0.086217847	-5.806903652	6.36387E-09	1.60392E-08
CYB561	383.1380471	-0.28675438	0.049371467	-5.808099267	6.31861E-09	1.59271E-08
CLCA1	1.534177126	-1.177026991	0.202608219	-5.809374362	6.27067E-09	1.58083E-08
TIGAR	228.1937034	0.2978942	0.051273563	5.809898588	6.25107E-09	1.57609E-08
PLPPR4	1.612661601	-0.911800661	0.156855296	-5.813005274	6.13611E-09	1.54731E-08
TRIM4	652.7667738	-0.158536044	0.02726228	-5.815215899	6.05557E-09	1.5272E-08
ERLIN2	1407.98468	-0.189071741	0.032512957	-5.815273692	6.05348E-09	1.52686E-08
ZZZ3	1629.064276	-0.151219979	0.02599954	-5.816255904	6.01803E-09	1.51812E-08
CCDC73	30.38286609	-0.366338592	0.062975651	-5.817146563	5.98607E-09	1.51025E-08
RCN3	127.9625013	-0.401580325	0.069028365	-5.817613131	5.96939E-09	1.50624E-08
KHDC1L	0.85173384	-0.688562735	0.118353164	-5.817865052	5.9604E-09	1.50416E-08
TTC13	408.058397	-0.263558856	0.045300102	-5.818063126	5.95334E-09	1.50258E-08

ITPRIP	5664.524689	0.283017002	0.048641821	5.818388344	5.94177E-09	1.49985E-08
PLAGL2	3522.678873	0.211684111	0.036378637	5.81891265	5.92317E-09	1.49535E-08
PRSS48	1.281088231	-1.054437754	0.181154606	-5.820651068	5.86188E-09	1.48007E-08
TRIM39	309.5030637	0.186034743	0.031955123	5.821750127	5.82346E-09	1.47055E-08
ATP5MG	788.2004502	-0.434484311	0.074614594	-5.82304729	5.77842E-09	1.45937E-08
SOX9	6.872096809	-0.78812807	0.135344652	-5.823119423	5.77593E-09	1.45893E-08
POLR2D	913.321672	-0.13779899	0.023662339	-5.823557479	5.7608E-09	1.45529E-08
UVSSA	1428.410083	-0.198306135	0.034051475	-5.823716462	5.75532E-09	1.4541E-08
RSU1	1508.431583	-0.261197538	0.044840149	-5.825081892	5.70846E-09	1.44244E-08
CAPN15	2217.781103	-0.186686426	0.032047696	-5.825268323	5.70209E-09	1.44102E-08
CTDSPL	365.2550538	-0.410316553	0.070427986	-5.826044124	5.67566E-09	1.43453E-08
TAP1	2330.290997	0.255122178	0.043789047	5.826164186	5.67158E-09	1.43368E-08
LRRFIP2	3970.662733	0.19966408	0.034267845	5.826572489	5.65773E-09	1.43036E-08
SELENOV	2.332057046	-0.653537206	0.112157039	-5.826983426	5.64382E-09	1.42703E-08
CRNKL1	1080.462037	0.145637072	0.024991395	5.827488612	5.62677E-09	1.4229E-08
C9orf16	1361.176602	-0.450226733	0.077248245	-5.828310215	5.59914E-09	1.4161E-08
INPP5B	2051.241344	-0.191011605	0.032772656	-5.828383339	5.59669E-09	1.41566E-08
LIPI	2.663074795	-0.60886032	0.104459272	-5.828686201	5.58654E-09	1.41328E-08
PSMB9	636.8510751	-0.338167439	0.058008765	-5.829592092	5.5563E-09	1.40581E-08
CCDC88B	3851.935615	-0.298500949	0.051203377	-5.829712126	5.55231E-09	1.40498E-08
RAD23A	2389.17296	-0.230289842	0.039498722	-5.830311172	5.53241E-09	1.40013E-08
PSMG2	1503.509133	0.199938445	0.034290221	5.830771615	5.51717E-09	1.39645E-08
RCN2	1204.248745	0.180884294	0.031020324	5.831154212	5.50453E-09	1.39343E-08
C16orf91	127.6058561	-0.274403676	0.047045728	-5.832701256	5.45372E-09	1.38075E-08
FAR1	2504.776676	0.265932068	0.045585453	5.833704575	5.42101E-09	1.37264E-08
OR7C1	16.80875325	-0.377226931	0.064662271	-5.833802669	5.41782E-09	1.37201E-08
PRR30	1.769221609	-0.798675653	0.136894773	-5.834230454	5.40394E-09	1.36868E-08
PICALM	6291.848064	0.177128992	0.030354578	5.835330495	5.3684E-09	1.35985E-08
ATAT1	523.2325769	-0.191888278	0.032881606	-5.835733219	5.35545E-09	1.35675E-08
MIA2	1194.734908	0.144029544	0.024678764	5.836173301	5.34133E-09	1.35335E-08
ARF4	3104.428283	0.184041904	0.031534613	5.836187185	5.34089E-09	1.35335E-08
ENTPD8	17.7078637	-0.448808186	0.076895153	-5.836625182	5.32687E-09	1.35003E-08
IL23A	127.8329775	0.31815075	0.054507245	5.836852479	5.31962E-09	1.34837E-08
OR51B2	1.517721191	-0.741344917	0.127008685	-5.83696239	5.31611E-09	1.34765E-08
DMTN	2686.686373	-0.599839028	0.102756156	-5.837499653	5.299E-09	1.34349E-08
ZNF598	2136.293076	0.229288935	0.039275608	5.837947448	5.28478E-09	1.34006E-08
TMEM102	127.3423801	-0.287495118	0.049244558	-5.83810944	5.27965E-09	1.33893E-08

CDC6	425.9589048	-0.257218387	0.044055555	-5.838500662	5.26727E-09	1.33596E-08
IGDCC3	12.91994459	-0.480891115	0.082358571	-5.838992922	5.25173E-09	1.33219E-08
CRABP1	9.615050362	-0.994196941	0.170252337	-5.839549459	5.23422E-09	1.32792E-08
ZNF710	838.8282977	-0.206803579	0.035407378	-5.840691743	5.19845E-09	1.31902E-08
ZIC1	0.982026986	-1.136055781	0.194473604	-5.841696545	5.16719E-09	1.31126E-08
TRAPPC5	146.0864545	-0.256078449	0.043834041	-5.841999586	5.15779E-09	1.30904E-08
SMU1	2869.064311	0.123403899	0.021119654	5.843083414	5.12433E-09	1.30072E-08
SUSD5	33.46394261	-0.350713321	0.06001455	-5.843804932	5.10218E-09	1.29526E-08
B3GNT6	6.095677774	-0.575289113	0.098418195	-5.845353221	5.05494E-09	1.28344E-08
KAT7	6611.060617	0.16262146	0.027820137	5.845458673	5.05174E-09	1.28279E-08
HMHB1	2.44967127	-1.024028164	0.175168887	-5.845947754	5.03692E-09	1.2792E-08
LSM12	547.7992666	0.193720144	0.033136788	5.846074797	5.03308E-09	1.27839E-08
DTNB	395.6803405	-0.209814303	0.035887789	-5.846398172	5.02331E-09	1.27607E-08
ESR1	64.40699504	-0.440084285	0.075265139	-5.847119787	5.00157E-09	1.27071E-08
TWIST1	18.08312237	0.855878702	0.146366247	5.847514178	4.98973E-09	1.26787E-08
ERAS	6.175462212	-0.556194138	0.095113447	-5.847691937	4.98441E-09	1.26668E-08
SCN3A	37.56558057	-0.601485219	0.102840207	-5.848736007	4.95322E-09	1.25892E-08
LHFPL1	1.264084304	-0.988888246	0.169045648	-5.849829652	4.92077E-09	1.25083E-08
TBX5	1.458365838	-1.377919564	0.235534244	-5.850187804	4.91018E-09	1.24831E-08
SLC7A3	10.74423662	-0.819619584	0.140061783	-5.851843134	4.86155E-09	1.2361E-08
LEMD2	2203.587128	0.194935277	0.03330912	5.852309453	4.84794E-09	1.2328E-08
SLC25A23	112.4934981	-0.455780514	0.077875033	-5.85271683	4.83607E-09	1.22994E-08
GABRR2	98.00650142	-0.401824768	0.068656061	-5.852721011	4.83595E-09	1.22994E-08
UBE2N	2277.710723	0.169125973	0.028893075	5.853512464	4.81298E-09	1.22439E-08
ZNF385B	16.91371995	-0.777768453	0.132871708	-5.853529447	4.81249E-09	1.22439E-08
GDA	12.3190836	-0.652908791	0.111484226	-5.856512762	4.72687E-09	1.2028E-08
RWDD4	320.9508433	0.171192865	0.029230515	5.856648965	4.723E-09	1.20197E-08
RBM23	3115.905264	0.138415138	0.023633149	5.85682162	4.71809E-09	1.20087E-08
HCRTR1	89.82539858	0.425301464	0.072614749	5.856956992	4.71425E-09	1.20005E-08
TNFRSF25	449.1178365	0.363328405	0.062023877	5.857879584	4.68814E-09	1.19356E-08
TOPAZ1	1.454863151	-0.694373346	0.118522233	-5.858591475	4.66809E-09	1.18861E-08
LRRN4	11.5154194	-0.441097946	0.075289207	-5.858714172	4.66465E-09	1.18789E-08
IL9R	54.08608231	-0.588883681	0.100510238	-5.858942231	4.65825E-09	1.18641E-08
C15orf62	110.3606128	-0.252299172	0.04305763	-5.859569469	4.64069E-09	1.18209E-08
FAM76A	389.4623868	0.173796605	0.029659495	5.85972912	4.63623E-09	1.18111E-08
COPS7B	946.0267538	-0.123052093	0.020995434	-5.86089781	4.60371E-09	1.17298E-08
SPATA31E1	13.86116296	-0.885345344	0.151057975	-5.860963918	4.60188E-09	1.17267E-08

GPR89B	173.5064805	-0.218994827	0.037352202	-5.862969658	4.54661E-09	1.15874E-08
SAMD15	43.59818882	-0.22171671	0.037812715	-5.863549067	4.53077E-09	1.15485E-08
ING3	982.2564796	0.26839936	0.04577416	5.863556198	4.53057E-09	1.15485E-08
PALM2AKAP2	3140.986428	0.369896245	0.063080736	5.863854288	4.52244E-09	1.15303E-08
CHGB	7.974938104	-0.49355161	0.084155078	-5.864787042	4.49709E-09	1.14671E-08
ITGA5	10946.78062	0.279833757	0.04771204	5.865055388	4.48983E-09	1.14501E-08
XKR8	403.3988273	0.257250611	0.043859899	5.865280555	4.48374E-09	1.1436E-08
PIPOX	138.8138176	-0.292789658	0.049914146	-5.865865333	4.46796E-09	1.13973E-08
SLFN12	174.8562186	-0.339782787	0.057923394	-5.866071824	4.46241E-09	1.13846E-08
SIAH1	1215.440787	0.232190248	0.039580613	5.86626201	4.45729E-09	1.1373E-08
COX7C	4579.962041	-0.443612188	0.075602465	-5.867694728	4.41896E-09	1.12767E-08
C4B	15.21909415	-0.429065586	0.073108083	-5.868921291	4.3864E-09	1.11951E-08
ERFE	231.6317304	-0.581952808	0.099157523	-5.868972805	4.38503E-09	1.1193E-08
UNC5D	2.54400601	-1.24513706	0.21214973	-5.869142797	4.38054E-09	1.1183E-08
NDUFA2	362.975983	-0.425856822	0.07254997	-5.869841471	4.36212E-09	1.11375E-08
GOLGA6B	3.366832065	-0.604858758	0.103039727	-5.870151029	4.35398E-09	1.11181E-08
ATOH7	2.398377696	-0.51301054	0.087389482	-5.870392289	4.34765E-09	1.11034E-08
DPY19L3	1272.644847	-0.19608167	0.033399636	-5.870772725	4.33769E-09	1.10794E-08
EGR2	160.6589242	0.483734569	0.08236537	5.873033367	4.27892E-09	1.09307E-08
SRSF11	11109.83911	0.197677238	0.03365121	5.87429812	4.24639E-09	1.0849E-08
RFX3	940.4415263	-0.239906962	0.040838655	-5.874506879	4.24104E-09	1.08368E-08
FNBP4	7135.952763	0.186137763	0.031674403	5.876598881	4.18781E-09	1.07022E-08
MAGEC3	1.155374608	-2.555500902	0.434830379	-5.877006361	4.17752E-09	1.06773E-08
VIP	0.778199762	-1.550359091	0.263765759	-5.877787529	4.15786E-09	1.06284E-08
SLC38A6	133.8111282	-0.213432582	0.036311003	-5.877903844	4.15494E-09	1.06223E-08
METTL14	709.1799922	0.173476742	0.029508633	5.878847201	4.13134E-09	1.05634E-08
ADGRL1	700.6779875	-0.297172515	0.050549397	-5.878853761	4.13117E-09	1.05634E-08
SRSF4	5805.133104	0.181570868	0.030874805	5.880874881	4.08104E-09	1.04375E-08
IREB2	4060.746325	0.184441494	0.031362178	5.881016705	4.07754E-09	1.04299E-08
GALNT8	9.413280935	-0.455780101	0.077491587	-5.881672018	4.06143E-09	1.03901E-08
MAS1L	0.592868836	-1.307218787	0.222248791	-5.881781322	4.05874E-09	1.03845E-08
PROB1	259.3595647	-0.317612908	0.053999215	-5.881806015	4.05814E-09	1.03844E-08
MAP3K19	33.93945131	-0.480637202	0.081671123	-5.885032396	3.97976E-09	1.01851E-08
ERICH6	10.61488128	-0.360988177	0.06133894	-5.885138802	3.9772E-09	1.01799E-08
ALOX5AP	1671.149218	0.511733101	0.086951068	5.885299796	3.97333E-09	1.01713E-08
TFCP2L1	83.89567199	0.625125654	0.106204986	5.886029239	3.95584E-09	1.01279E-08
NQO2	680.1600818	-0.240742701	0.040899288	-5.886232119	3.95099E-09	1.01168E-08

PRRT1	17.19745306	-0.421131501	0.07154249	-5.886452959	3.94572E-09	1.01046E-08
GJD2	1.323021395	-1.193763165	0.202797449	-5.886480196	3.94507E-09	1.01043E-08
CAVIN2	249.6892297	-0.655735596	0.111387591	-5.886971708	3.93336E-09	1.00756E-08
ILRUN	3459.062192	0.16334487	0.027740966	5.888218515	3.90381E-09	1.00012E-08
EFCAB7	123.893772	-0.218731113	0.037146966	-5.888263169	3.90275E-09	9.99981E-09
SPEF1	5.832620246	-0.42116902	0.071522305	-5.888638837	3.89389E-09	9.97842E-09
CXCL10	38.07490749	0.762435713	0.129474496	5.888694171	3.89259E-09	9.97639E-09
DIO1	44.73463468	-0.312247877	0.053021082	-5.889126796	3.88241E-09	9.95161E-09
CARMIL3	64.35072314	-0.311700811	0.05291392	-5.890714787	3.84529E-09	9.85773E-09
KDM6B	6725.022914	0.256620102	0.04356205	5.890909679	3.84075E-09	9.8474E-09
GATD3B	81.3826881	-0.258685923	0.043901468	-5.892420724	3.80579E-09	9.75903E-09
FO393400.1	11.63344823	-0.457780316	0.077688213	-5.892532445	3.80321E-09	9.75371E-09
PRICKLE1	117.7278756	-0.504073518	0.085485981	-5.896563542	3.7115E-09	9.51974E-09
TUFM	3148.035909	-0.20860649	0.035376787	-5.896705442	3.70831E-09	9.51281E-09
SLC22A25	3.354151872	-0.886058496	0.150257751	-5.89692372	3.70341E-09	9.50149E-09
GLIPR2	1344.211627	0.368663688	0.062511009	5.897580211	3.68871E-09	9.46501E-09
EPS8L1	166.0447575	-0.364378933	0.061784352	-5.897592532	3.68843E-09	9.46501E-09
CLN6	756.6547173	-0.223779936	0.03793156	-5.899571073	3.64448E-09	9.35397E-09
GSDMA	4.854575882	-0.429501324	0.072801613	-5.899612712	3.64356E-09	9.35283E-09
OR8D4	1.298387513	-0.96311805	0.163249063	-5.899684999	3.64196E-09	9.34996E-09
CCDC78	147.2148924	-0.329500326	0.055845345	-5.900229069	3.62997E-09	9.32041E-09
ADGRD1	50.43974338	-0.57430333	0.097332885	-5.900403847	3.62613E-09	9.31176E-09
KCNE4	4.520044577	-0.646437498	0.109544453	-5.90114313	3.60992E-09	9.27134E-09
EFCAB2	461.5501605	-0.302171026	0.051198423	-5.90195959	3.59209E-09	9.22677E-09
SEL1L2	7.572060484	-0.766181286	0.129806169	-5.902502884	3.58028E-09	9.19764E-09
CCNT1	4093.581295	0.209678209	0.035523265	5.90256021	3.57903E-09	9.19565E-09
POLR2B	3930.345317	0.179865073	0.030463735	5.904235685	3.54285E-09	9.10388E-09
ATAD3C	30.47078224	-0.472468812	0.080016786	-5.904621207	3.53458E-09	9.08381E-09
CARD14	61.4502401	0.465224863	0.078789296	5.904670934	3.53351E-09	9.08227E-09
FOXM1	790.3862675	-0.258878884	0.04384226	-5.904779568	3.53119E-09	9.07748E-09
MRPL1	340.3809644	-0.258097146	0.04370726	-5.905132102	3.52364E-09	9.05928E-09
TDGF1	1.408602001	-0.823661281	0.139478537	-5.905290493	3.52026E-09	9.05176E-09
DENND4B	4215.688496	-0.168495443	0.028526343	-5.906661081	3.49111E-09	8.97799E-09
COQ2	377.6780305	-0.219171547	0.037102174	-5.907242638	3.47881E-09	8.94754E-09
CBARP	49.18258698	-0.501915319	0.084965596	-5.90727712	3.47808E-09	8.94684E-09
MBOAT2	1582.877543	-0.480927227	0.081406543	-5.907722001	3.46871E-09	8.9239E-09
NIPSNAP3B	305.6064486	-0.256382742	0.04339659	-5.907900616	3.46495E-09	8.9154E-09

IFNK	76.94372154	0.495477943	0.083862836	5.908194433	3.45878E-09	8.90069E-09
NDUFB10	868.1106257	-0.37572709	0.063593388	-5.908272871	3.45713E-09	8.89762E-09
CCDC124	533.3087011	-0.293452892	0.04966731	-5.90837097	3.45507E-09	8.8935E-09
OR1D5	1.88439527	-0.700222676	0.118479304	-5.910084308	3.41933E-09	8.80264E-09
EPHA5	0.89580589	-1.567873643	0.265257673	-5.910756973	3.40539E-09	8.76792E-09
E2F8	270.8432668	-0.360106803	0.060908804	-5.912229141	3.37509E-09	8.69103E-09
CCL21	2.133795276	-0.719603562	0.121703259	-5.912771505	3.36399E-09	8.66359E-09
HAPLN2	6.691143755	-0.420190861	0.071061688	-5.913043639	3.35843E-09	8.65043E-09
ZNF330	734.4603708	0.164791141	0.027868094	5.91325485	3.35413E-09	8.64047E-09
ART1	6.879724443	-0.693500747	0.117272473	-5.913585107	3.3474E-09	8.62429E-09
ZKSCAN4	238.9779126	-0.241204223	0.040764145	-5.917068106	3.27731E-09	8.44481E-09
OR51V1	1.436512501	-1.146331901	0.193702297	-5.918008814	3.25863E-09	8.39777E-09
ZBTB39	895.2174401	0.220491595	0.037254735	5.918485128	3.2492E-09	8.37459E-09
ZNF618	1322.1594	-0.258343467	0.043629119	-5.921354149	3.19301E-09	8.23085E-09
PRRG2	28.46994538	-0.370074523	0.062482865	-5.922816211	3.16474E-09	8.15906E-09
COMMD7	791.7575341	-0.241341261	0.040745564	-5.923129753	3.15871E-09	8.14458E-09
OR6V1	0.625523029	-0.764418874	0.129046863	-5.923575805	3.15015E-09	8.12358E-09
EMP3	5674.702152	0.378402506	0.06387941	5.923700666	3.14776E-09	8.11848E-09
CCDC200	622.9039044	-0.289682392	0.048901753	-5.923762979	3.14657E-09	8.11647E-09
IGF1	3.967780649	-0.652853463	0.110173792	-5.92566938	3.11028E-09	8.02392E-09
SAT1	20461.75559	0.385554552	0.065058578	5.92626772	3.09897E-09	7.99581E-09
RBBP9	215.9789146	-0.231228812	0.039015701	-5.926557925	3.0935E-09	7.98275E-09
MRPL23	658.0243043	-0.401168996	0.067688251	-5.926715355	3.09054E-09	7.97615E-09
ZNF146	1047.431142	-0.224822316	0.037932934	-5.926836968	3.08825E-09	7.9713E-09
RTL8B	22.39189904	-0.830303768	0.140063099	-5.928069365	3.06517E-09	7.91276E-09
NXF5	1.583910279	-0.586251865	0.098890591	-5.928287599	3.0611E-09	7.9033E-09
CA3	40.76328654	-0.604297988	0.101931872	-5.928449836	3.05808E-09	7.89654E-09
GOLGA7	336.5390418	0.164224884	0.027698403	5.929037931	3.04715E-09	7.86935E-09
PPA2	1357.523278	-0.149030938	0.025135374	-5.929131589	3.04541E-09	7.8659E-09
E4F1	1156.917551	-0.170749253	0.028793931	-5.930043084	3.02855E-09	7.82339E-09
ADAMTS9	33.74829065	-0.863003851	0.145509986	-5.930890908	3.01295E-09	7.78413E-09
IPO8	1234.678023	-0.189711098	0.031986646	-5.930946854	3.01193E-09	7.7825E-09
ZNF670-ZNF695	9.692518097	-0.444495668	0.074944153	-5.931025318	3.01049E-09	7.77981E-09
RBL1	742.082493	-0.237566364	0.040045471	-5.932415197	2.98511E-09	7.71524E-09
ADGRF5	7.954484886	-0.866710425	0.1460784	-5.933186715	2.97111E-09	7.68007E-09
HS3ST2	5.055360201	-0.63189122	0.106480955	-5.934312083	2.9508E-09	7.62859E-09
POMC	76.91362638	-0.614305928	0.103504525	-5.935063503	2.93732E-09	7.59473E-09

ERVV-1	0.60003673	-1.162777052	0.195891685	-5.935816284	2.92387E-09	7.56096E-09
KLK13	2.742370118	-1.298644661	0.218775463	-5.935970345	2.92113E-09	7.55486E-09
DNAL1	235.2603313	-0.182363015	0.030718068	-5.936669429	2.9087E-09	7.52373E-09
MAP3K10	311.6227799	-0.247544486	0.041696553	-5.936809325	2.90622E-09	7.51831E-09
ZNF16	175.4147566	-0.187090852	0.031509591	-5.937584283	2.89252E-09	7.48386E-09
ACAP2	4742.504154	0.167807194	0.028260174	5.9379392	2.88627E-09	7.46866E-09
SNRPA1	2828.669638	0.182792691	0.030780976	5.938495558	2.87649E-09	7.44435E-09
CENPA	104.9580878	-0.327116827	0.055084022	-5.938506538	2.8763E-09	7.44435E-09
TCEA3	28.53159483	0.420996779	0.070866286	5.940720223	2.83773E-09	7.34596E-09
PLA2G3	2.45895244	-0.779268615	0.13116406	-5.94117485	2.82987E-09	7.32658E-09
KLHL35	150.039934	-0.352893809	0.059393978	-5.941575568	2.82296E-09	7.30966E-09
ADAR	14199.17478	0.161438645	0.027167981	5.942239404	2.81154E-09	7.28107E-09
PCMTD2	1403.224326	-0.183306336	0.030840102	-5.943765476	2.78548E-09	7.21453E-09
ADAM19	681.9152438	0.427760598	0.071939538	5.946112668	2.74585E-09	7.11283E-09
TMEM196	4.02206245	-1.060812818	0.17835946	-5.947611724	2.72083E-09	7.04895E-09
AC004233.4	12.48768543	-0.506179387	0.085090349	-5.948728548	2.70233E-09	7.00196E-09
LRRTM1	1.043643046	-1.53963487	0.258782721	-5.949527332	2.68918E-09	6.96879E-09
KIF17	164.1285171	-0.536564483	0.09017766	-5.950082151	2.68008E-09	6.94613E-09
PCBD1	594.3021889	-0.394045513	0.066223527	-5.950234447	2.67759E-09	6.94059E-09
DSN1	431.4223219	-0.168585618	0.028332264	-5.950305245	2.67643E-09	6.93851E-09
IDO1	3.430216014	-0.76523745	0.128594878	-5.950761522	2.66898E-09	6.92011E-09
GYPB	287.7246491	-0.689251338	0.115821197	-5.95099477	2.66518E-09	6.91117E-09
SIX3	28.34238036	1.07100191	0.179966412	5.951121103	2.66312E-09	6.90675E-09
ZKSCAN8	3183.450026	0.24288424	0.040811042	5.951434451	2.65802E-09	6.89445E-09
MRPS2	312.7999227	-0.293700529	0.049340114	-5.952570923	2.63963E-09	6.84764E-09
ENPP2	246.6079719	-0.53455258	0.089797948	-5.952837344	2.63533E-09	6.8374E-09
NRK	0.698073351	-1.433780221	0.24082615	-5.95359027	2.62323E-09	6.80691E-09
SEC14L3	31.26218244	-0.568720094	0.095523546	-5.953716326	2.62121E-09	6.80256E-09
PGM3	409.1492534	0.298099746	0.050064963	5.954258776	2.61253E-09	6.78094E-09
SNAI3	125.4019435	-0.277960239	0.046680222	-5.95456118	2.60771E-09	6.76931E-09
ZNF558	465.2988639	-0.206252417	0.034636027	-5.954852049	2.60307E-09	6.75818E-09
C1orf146	6.728555254	-0.396112972	0.066512632	-5.955454753	2.5935E-09	6.73421E-09
CD160	82.48244393	-0.297610564	0.049960087	-5.95696654	2.56963E-09	6.67312E-09
COL9A1	4.902579636	-0.592282101	0.099426134	-5.957006237	2.569E-09	6.67239E-09
NABP1	3391.499836	0.36309531	0.060940062	5.958236683	2.54974E-09	6.62323E-09
ENKUR	8.934861223	-0.583521185	0.097931602	-5.958456421	2.54632E-09	6.61521E-09
LTBP1	1341.030399	-0.645609601	0.108339858	-5.959114358	2.53609E-09	6.58951E-09

ANXA4	794.4892762	-0.289476003	0.04857045	-5.959920139	2.52361E-09	6.55797E-09
NEDD4L	461.6855635	-0.47688683	0.080001236	-5.960993272	2.50709E-09	6.51591E-09
CHMP3	1135.473451	0.135622594	0.022749786	5.961488779	2.4995E-09	6.49704E-09
SDHAF3	139.5287732	-0.326470221	0.054761818	-5.961639596	2.49719E-09	6.49191E-09
ZIC2	1.165294598	-0.819707969	0.137480923	-5.962339712	2.48652E-09	6.46501E-09
ASAP3	19.16225754	-0.472602458	0.079258381	-5.96280738	2.47941E-09	6.44738E-09
ZFP90	592.559448	-0.204369149	0.034267435	-5.96394655	2.46217E-09	6.40342E-09
MTCP1	26.05667215	0.199943801	0.033520599	5.964804016	2.44928E-09	6.37073E-09
NEURL1B	386.3943614	-0.512623219	0.085932324	-5.965429468	2.43991E-09	6.34721E-09
RPRD1A	1176.001037	-0.184698788	0.030961456	-5.965442627	2.43972E-09	6.34721E-09
SNAP91	1.742199857	-0.827392423	0.138674225	-5.966447059	2.42475E-09	6.30945E-09
C1orf189	7.632918231	0.395596299	0.066297732	5.966965778	2.41706E-09	6.29027E-09
HEPACAM	1.084211046	-0.669327452	0.112168481	-5.967161573	2.41416E-09	6.28357E-09
OSGIN1	116.4690481	-0.288427334	0.048329553	-5.967928912	2.40284E-09	6.25493E-09
TMEM132C	5.058736069	-0.850512412	0.14251331	-5.967950722	2.40252E-09	6.25492E-09
ZBTB2	979.7033679	0.249714797	0.04184183	5.968065927	2.40082E-09	6.25134E-09
SOD3	1.92044102	-0.659253968	0.110395009	-5.971773313	2.34688E-09	6.11171E-09
RNF151	13.08150463	-0.649616708	0.108779735	-5.971854112	2.34572E-09	6.10949E-09
H2BC6	645.8844963	-0.448333861	0.075069344	-5.97226292	2.33985E-09	6.09501E-09
USP20	2608.514342	-0.251631558	0.042130561	-5.972660957	2.33415E-09	6.08096E-09
SLC12A4	1359.328517	-0.281119399	0.047065731	-5.972910485	2.33058E-09	6.07247E-09
STX19	5.756261139	-0.449561301	0.075265216	-5.973028765	2.32889E-09	6.06888E-09
SLC5A9	24.36000879	-0.603358289	0.10099952	-5.973872838	2.31686E-09	6.03835E-09
RMDN1	805.9234371	-0.174125894	0.029147254	-5.974006824	2.31496E-09	6.03419E-09
MT-ND2	195872.2187	-0.719581702	0.120443381	-5.97443958	2.30882E-09	6.019E-09
PPIC	6.654050087	-0.76478494	0.127994186	-5.975153748	2.29873E-09	5.99349E-09
LHCGR	1.347709271	-0.892843061	0.149419465	-5.975413327	2.29508E-09	5.98475E-09
OR2W3	212.5137275	-0.479175835	0.080189766	-5.975523536	2.29352E-09	5.9815E-09
GPR179	31.67549058	-0.355945771	0.059566699	-5.975583246	2.29268E-09	5.98011E-09
SETMAR	399.6495133	-0.181996442	0.030451242	-5.976650856	2.27772E-09	5.94186E-09
SULT6B1	2.313428041	-0.666635568	0.111539063	-5.976700447	2.27702E-09	5.94084E-09
TAF15	2841.903523	-0.231121502	0.038661603	-5.978063058	2.25806E-09	5.89216E-09
WDR35	448.3301406	-0.363001626	0.060719309	-5.978355655	2.25401E-09	5.88237E-09
DUS2	479.5003511	-0.185918012	0.031092467	-5.979519394	2.23797E-09	5.84128E-09
ATMIN	1464.032648	0.180935199	0.030257006	5.979943961	2.23214E-09	5.82685E-09
S100A10	4081.306117	0.626577138	0.104772478	5.980360014	2.22645E-09	5.81277E-09
MYADM	10217.29425	0.343175414	0.057378607	5.98089484	2.21915E-09	5.79448E-09

TMEM50A	2259.511624	0.191384671	0.031994923	5.981719999	2.20794E-09	5.76597E-09
ADA2	3245.758814	0.464117824	0.077588514	5.981785159	2.20705E-09	5.76443E-09
YEATS2	2459.2067	-0.154118841	0.025761642	-5.982492839	2.19748E-09	5.7402E-09
POTEE	11.15927739	0.485307054	0.08110919	5.98337936	2.18555E-09	5.70979E-09
TMEM205	239.5562146	-0.339985656	0.05681981	-5.983576046	2.18291E-09	5.70366E-09
CD27	44.97150927	0.405888259	0.067825872	5.984268966	2.17364E-09	5.68019E-09
BOLA2B	16.89641126	-0.501183719	0.083749307	-5.984332751	2.17279E-09	5.67872E-09
COPG2	957.2042659	-0.25312862	0.04229843	-5.984350236	2.17255E-09	5.67872E-09
SEPTIN6	8170.321274	-0.264373643	0.044145921	-5.988631229	2.11614E-09	5.53216E-09
RPUSD1	525.0640215	-0.228830695	0.038206435	-5.989323456	2.10716E-09	5.5094E-09
ETNK1	2092.808259	-0.204366169	0.034120966	-5.989460295	2.10539E-09	5.5055E-09
DMC1	57.17986788	-0.351569814	0.058686921	-5.990599046	2.0907E-09	5.46782E-09
MMUT	408.9769966	-0.195216628	0.032580027	-5.99191115	2.07389E-09	5.4246E-09
CD300C	398.5818211	0.51817591	0.086468911	5.992626742	2.06478E-09	5.4015E-09
PHLDA2	136.6325049	0.579839716	0.096755345	5.992844304	2.06202E-09	5.395E-09
VWA3B	6.750719242	-0.46716626	0.077950863	-5.993086471	2.05895E-09	5.38769E-09
EBPL	884.3384542	-0.31622938	0.052762669	-5.993430381	2.0546E-09	5.37702E-09
RANBP17	165.9704914	-0.581780397	0.097063346	-5.993821774	2.04966E-09	5.3648E-09
FIGNL1	324.780955	-0.238614841	0.039809785	-5.993874157	2.049E-09	5.36379E-09
GJD3	10.98529009	-0.407754628	0.068025237	-5.994166994	2.04531E-09	5.35485E-09
PER3	123.0999845	-0.419074808	0.069903986	-5.995005855	2.03478E-09	5.328E-09
APOL5	0.896613354	-1.058344953	0.176533515	-5.995150274	2.03297E-09	5.32398E-09
MATR3	6.574904067	-0.411838072	0.068675491	-5.996871169	2.01156E-09	5.26859E-09
EPHA6	0.79265115	-1.135423366	0.189334235	-5.996925834	2.01088E-09	5.26752E-09
AK1	46.43343046	-0.320227601	0.053394633	-5.997374292	2.00533E-09	5.2537E-09
GPR173	31.41036194	-0.649818114	0.108340203	-5.997940705	1.99835E-09	5.23612E-09
R3HDM1	1621.38523	-0.175490206	0.029257766	-5.998072612	1.99673E-09	5.23257E-09
NKX6-1	2.200751941	-0.928664239	0.15481813	-5.998420457	1.99246E-09	5.22207E-09
FERMT1	35.32117959	-0.770853087	0.128504949	-5.998625693	1.98994E-09	5.21618E-09
STRN3	1082.330013	-0.233618385	0.038940849	-5.999314191	1.98153E-09	5.1948E-09
DTHD1	26.28893344	0.510086253	0.085020496	5.999568059	1.97843E-09	5.18738E-09
F11	2.228959367	-0.836502396	0.139420709	-5.999843231	1.97508E-09	5.1793E-09
CLEC18C	4.452729574	-0.481787933	0.080296064	-6.0001438	1.97143E-09	5.17041E-09
MEF2C	3072.414866	-0.388849755	0.064794624	-6.001265731	1.95785E-09	5.13549E-09
ZC3HC1	296.4070078	-0.144131765	0.024014168	-6.001947139	1.94965E-09	5.11467E-09
ZNF529	1022.304872	0.257269815	0.04286397	6.002006221	1.94894E-09	5.11349E-09
TLE4	5077.65762	0.231218374	0.038523489	6.002010174	1.94889E-09	5.11349E-09

HSPA5	51980.2985	0.334555929	0.055733504	6.002779358	1.93968E-09	5.09056E-09
TRAF3IP3	1240.637218	-0.291918849	0.048615926	-6.004593022	1.91812E-09	5.03466E-09
FZD5	635.3767988	-0.406217321	0.067616461	-6.007669027	1.8821E-09	4.94075E-09
HERC4	1729.321099	-0.212027699	0.035290279	-6.008104912	1.87704E-09	4.92815E-09
PRDM5	36.07606213	-0.805240619	0.134015516	-6.008562597	1.87175E-09	4.91492E-09
COA5	418.8532331	-0.181836326	0.030260791	-6.008974624	1.867E-09	4.90311E-09
ZNF646	1288.162909	-0.219455772	0.036509894	-6.010857482	1.84545E-09	4.84714E-09
FMOD	15.79817977	-0.551325984	0.091721672	-6.010858433	1.84544E-09	4.84714E-09
CFAP73	16.63885725	-0.35418025	0.058906324	-6.012601484	1.82569E-09	4.79655E-09
RNF212	35.08381802	-0.577536825	0.096051573	-6.012778431	1.8237E-09	4.79196E-09
ATRN	1646.542189	-0.232554523	0.038669857	-6.013844879	1.81174E-09	4.76117E-09
PTAR1	3657.043914	0.180581037	0.030027342	6.013886951	1.81127E-09	4.76057E-09
SPRY2	1186.257586	0.487026365	0.080981118	6.014073101	1.80919E-09	4.75574E-09
RUSC2	1312.781816	0.405451866	0.067406884	6.014992006	1.79896E-09	4.72948E-09
SUGT1	1643.785308	-0.165979686	0.027592408	-6.015411413	1.7943E-09	4.71788E-09
PTPN5	2.155579514	-0.701954172	0.116666144	-6.01677701	1.77924E-09	4.6789E-09
HSF2BP	9.320374075	-0.366539279	0.060918396	-6.016889827	1.778E-09	4.67627E-09
PSMG1	573.1851417	-0.165563568	0.027512427	-6.017774015	1.76832E-09	4.65143E-09
KCNMB2	3.208178627	-0.674378162	0.112048763	-6.018613205	1.75918E-09	4.62801E-09
EMSY	1001.851892	-0.171128945	0.028429653	-6.019382054	1.75084E-09	4.6067E-09
AARSD1	76.2501117	-0.244190269	0.040567068	-6.019421204	1.75042E-09	4.6062E-09
TNFRSF21	710.7436576	0.488200107	0.081101121	6.019646841	1.74798E-09	4.60041E-09
TMEM168	506.3657008	-0.224222772	0.037245493	-6.020131651	1.74275E-09	4.58726E-09
RREB1	10805.76428	0.17959815	0.029832596	6.020198495	1.74203E-09	4.58599E-09
CRISP1	0.687550942	-1.404979425	0.233366727	-6.020478756	1.73902E-09	4.57867E-09
ATF3	3974.194815	0.398026381	0.066101497	6.021442768	1.72869E-09	4.55209E-09
KIRREL3	202.7954229	-0.482322679	0.080096187	-6.021793226	1.72495E-09	4.54285E-09
AL049844.1	2.058931771	-0.679250485	0.112763908	-6.023651503	1.70525E-09	4.49158E-09
MGME1	562.8662323	-0.162342852	0.02694953	-6.023958547	1.70202E-09	4.48367E-09
FOXB1	3.619729854	-0.770295751	0.127854394	-6.02478902	1.6933E-09	4.4613E-09
FAM20C	755.3859154	0.56395967	0.093605702	6.024843112	1.69274E-09	4.46041E-09
CFAP299	1.851965795	-0.741947653	0.123114893	-6.026465482	1.67584E-09	4.41648E-09
CBX4	2351.263613	0.278199989	0.046160503	6.026797182	1.67241E-09	4.40803E-09
HSCB	109.9302007	-0.241720149	0.040102524	-6.02755453	1.66459E-09	4.38802E-09
TRMU	780.6328853	-0.187004601	0.031015234	-6.029443483	1.64525E-09	4.33762E-09
DDX3X	23671.14662	0.185726784	0.030802993	6.029504401	1.64463E-09	4.33657E-09
POLR3G	113.9749968	-0.267510137	0.044365183	-6.029731359	1.64232E-09	4.33107E-09

RBBP8	813.2003749	0.193700791	0.032123047	6.029963131	1.63997E-09	4.32545E-09
NF2	1527.648223	-0.170955632	0.028349441	-6.030299975	1.63656E-09	4.31702E-09
TEKT1	6.003215711	-0.56281207	0.093316825	-6.031196097	1.62751E-09	4.29373E-09
CDKL3	19.59379769	-0.266778362	0.044232384	-6.031290621	1.62655E-09	4.2918E-09
AP1S2	1439.561643	0.321063679	0.053215864	6.03323251	1.60712E-09	4.24109E-09
DMXL1	2447.720064	-0.169154012	0.028021547	-6.036569326	1.57425E-09	4.15491E-09
RBM39	24490.64704	0.178446121	0.029556246	6.037509664	1.56511E-09	4.13134E-09
MRPL18	683.4148833	-0.282092609	0.046723286	-6.037516488	1.56504E-09	4.13134E-09
HYDIN	41.66061362	0.575278819	0.095280532	6.037737284	1.5629E-09	4.12663E-09
CPNE9	55.30983944	-0.357255201	0.059163128	-6.038477219	1.55575E-09	4.10831E-09
TMEM9	697.1040044	-0.255361576	0.042281002	-6.039629307	1.54469E-09	4.07963E-09
CDH13	11.30327199	-0.557523081	0.092296966	-6.040535313	1.53604E-09	4.05734E-09
PAQR9	126.1784391	-0.68504331	0.11339345	-6.04129525	1.52882E-09	4.03881E-09
YPEL4	416.7272943	-0.567526522	0.093932006	-6.041886512	1.52323E-09	4.02458E-09
FANCM	466.1475642	-0.203452306	0.033668334	-6.04283863	1.51426E-09	4.00143E-09
GASK1A	11.0569868	-0.639710589	0.105855577	-6.043239331	1.5105E-09	3.99204E-09
FCHSD2	2518.530859	-0.18371386	0.030397877	-6.043641239	1.50674E-09	3.98265E-09
TBCA	855.26398	-0.400796727	0.066316693	-6.043677851	1.5064E-09	3.98228E-09
HSD17B10	666.386816	-0.284311191	0.047041676	-6.043815044	1.50512E-09	3.97943E-09
RDH11	827.615366	0.193962093	0.032091546	6.04402453	1.50317E-09	3.9748E-09
DAPK2	292.4680094	-0.46859191	0.077527217	-6.044224593	1.5013E-09	3.97041E-09
TLCD5	68.63815282	-0.730861549	0.120910421	-6.044653109	1.49732E-09	3.96041E-09
LRTM1	1.486732738	-1.007556053	0.166673896	-6.045074099	1.49341E-09	3.95061E-09
SRP19	483.6136027	0.221979232	0.03671866	6.045406686	1.49034E-09	3.943E-09
EGFLAM	2.246541157	-0.814382539	0.134699969	-6.045899977	1.48578E-09	3.93149E-09
PLA2G7	16.84832925	0.73179131	0.121003223	6.047700976	1.46927E-09	3.88833E-09
SLC11A1	2459.634268	0.532483563	0.088043803	6.047939182	1.4671E-09	3.88311E-09
FOXI1	14.24594121	-0.721875212	0.11935073	-6.048351902	1.46335E-09	3.8737E-09
ZNF592	3887.725024	0.16977461	0.028066406	6.049032696	1.45718E-09	3.85789E-09
CXCL8	22511.45273	0.603295224	0.099717512	6.050042887	1.44807E-09	3.8343E-09
PPP2R5E	1775.406364	0.116334789	0.019228621	6.050085002	1.44769E-09	3.83381E-09
NPIPB9	9.880723695	-0.436807197	0.072196506	-6.050253965	1.44618E-09	3.83031E-09
SLC6A15	1.238279589	-1.592569071	0.263119973	-6.052634676	1.42496E-09	3.77462E-09
RTL6	724.7928232	-0.300681619	0.049674919	-6.052986584	1.42185E-09	3.76689E-09
DHRS7	1246.080797	0.235659412	0.038929427	6.053503274	1.41729E-09	3.75533E-09
RNF114	3787.110855	0.163159312	0.026950856	6.053956635	1.41331E-09	3.74528E-09
CFAP97D1	9.027469646	-0.374414043	0.061845603	-6.054012294	1.41282E-09	3.74449E-09

PODNL1	34.74771154	-0.268627943	0.044369097	-6.054392823	1.40948E-09	3.73616E-09
SLC35A4	1111.108992	-0.272981253	0.045083061	-6.055073626	1.40353E-09	3.7209E-09
H4C9	715.8815895	-0.281335161	0.046452212	-6.056442707	1.39165E-09	3.68988E-09
STEAP3	690.8234382	-0.362980917	0.059917339	-6.058028046	1.378E-09	3.6542E-09
RBP5	185.0509042	-0.293657976	0.048471939	-6.058308782	1.3756E-09	3.64833E-09
DDRGK1	699.2634058	-0.187937069	0.031018672	-6.058836658	1.3711E-09	3.63687E-09
OPHN1	185.5393642	-0.468702224	0.077356524	-6.058987657	1.36981E-09	3.63395E-09
SFTPD	8.594453388	-0.616094208	0.101648751	-6.061011075	1.35269E-09	3.58901E-09
FETUB	1.15139968	-0.925107432	0.152623132	-6.061384159	1.34955E-09	3.58118E-09
RAX	0.992450278	-0.843316741	0.13912204	-6.061704824	1.34686E-09	3.57453E-09
SLC25A10	80.05465918	-0.260245989	0.04292232	-6.063185525	1.33452E-09	3.54224E-09
OR2D3	0.861727694	-1.199451755	0.197821023	-6.063317931	1.33342E-09	3.5398E-09
ACTA2	129.7740276	-0.340540407	0.056149397	-6.064898721	1.32037E-09	3.50564E-09
C11orf80	143.1667781	-0.279561853	0.04609365	-6.065083935	1.31885E-09	3.50208E-09
ARRDC2	2423.797439	0.260279032	0.042910009	6.065695116	1.31384E-09	3.48926E-09
RPL22L1	1356.619464	-0.396977798	0.065444744	-6.065846889	1.3126E-09	3.48644E-09
PRSS53	68.19077243	-0.278782191	0.045959257	-6.065855113	1.31253E-09	3.48644E-09
GABRA1	1.408068978	-1.624721666	0.267838843	-6.066041989	1.31101E-09	3.48315E-09
CAND2	122.1631907	-0.378098191	0.062328544	-6.066212501	1.30962E-09	3.47993E-09
IRF2BP1	616.613898	-0.24044358	0.039635329	-6.06639542	1.30813E-09	3.47644E-09
MAGED2	1570.880569	-0.253268722	0.041748241	-6.066572224	1.30669E-09	3.47309E-09
ZNF17	144.593532	-0.257150877	0.042379686	-6.067786215	1.29685E-09	3.44742E-09
MDGA2	12.25852968	-0.941633307	0.155185289	-6.067800077	1.29674E-09	3.44742E-09
AADACL3	0.862537586	-1.022016456	0.16841957	-6.068276138	1.2929E-09	3.43785E-09
HEPH	12.9055843	-0.621178867	0.102340568	-6.069722677	1.28131E-09	3.40749E-09
SPINK7	2.165182806	-0.699339917	0.115211636	-6.0700459	1.27874E-09	3.4011E-09
MYOZ1	5.091655064	-0.481381187	0.079293487	-6.070879309	1.27212E-09	3.38396E-09
KLHL34	6.243010947	-0.786536263	0.129556023	-6.071012733	1.27106E-09	3.38161E-09
DGCR6L	562.1825223	-0.311683244	0.051338321	-6.071161624	1.26988E-09	3.37893E-09
NSF	608.5886105	-0.231425907	0.038118734	-6.071185524	1.26969E-09	3.37889E-09
MAN2A2	7656.598247	-0.225691052	0.037170215	-6.071825364	1.26464E-09	3.36591E-09
FZD3	101.9542021	-0.563090458	0.09271149	-6.07357793	1.25091E-09	3.32981E-09
LY9	638.1856221	0.338417664	0.055712929	6.07431106	1.24521E-09	3.31509E-09
ANKMY2	736.9602087	0.178553474	0.029391397	6.075025024	1.23968E-09	3.30082E-09
PPP1CC	3539.766133	0.164441068	0.027065949	6.075570041	1.23548E-09	3.29008E-09
RIMS3	184.3372963	-0.453916313	0.074700529	-6.076480536	1.22849E-09	3.2719E-09
CFAP74	18.51835107	-0.487019489	0.080118504	-6.07873915	1.21131E-09	3.22659E-09

GPATCH8	3544.935697	0.167733517	0.027590569	6.079378775	1.20649E-09	3.21419E-09
UBE2B	2194.50234	0.260090098	0.042778698	6.079897416	1.20259E-09	3.20425E-09
OSBPL1A	611.1847395	-0.411870386	0.067738847	-6.080268659	1.19981E-09	3.19727E-09
ABCA3	154.773694	-0.433342236	0.071265292	-6.080691216	1.19666E-09	3.18929E-09
ZNF358	130.3831642	-0.433164748	0.071224736	-6.081661649	1.18943E-09	3.17047E-09
RPTOR	2008.608145	-0.201190697	0.033081357	-6.081694241	1.18919E-09	3.17026E-09
PRR5	377.9457545	-0.325117496	0.053454366	-6.082150412	1.18581E-09	3.16168E-09
CRACDL	38.0281736	-0.60082429	0.098756743	-6.083881202	1.17308E-09	3.12815E-09
OR1C1	2.073283617	-0.9198778	0.151189104	-6.084286323	1.17011E-09	3.12067E-09
TMEM160	298.9723678	-0.367595333	0.060415759	-6.084427941	1.16908E-09	3.11834E-09
H3C12	175.3636343	-0.52848047	0.08684321	-6.085455271	1.16161E-09	3.09883E-09
TEKT5	6.10918538	-0.460941923	0.075737723	-6.086028232	1.15746E-09	3.08819E-09
SMIM28	1.241051663	-0.847090746	0.139167408	-6.086847205	1.15156E-09	3.07286E-09
SHOX2	20.54156882	-0.81939964	0.134605246	-6.087427239	1.14739E-09	3.06217E-09
MAGEB2	1.569103409	-1.192853488	0.195942119	-6.087784972	1.14483E-09	3.05576E-09
RD3	7.459733024	-0.674929889	0.110864667	-6.087871889	1.14421E-09	3.05451E-09
MYT1L	12.63322464	-0.799636203	0.131317682	-6.089326212	1.13387E-09	3.02731E-09
PALB2	268.2852021	-0.183671055	0.030158935	-6.090104033	1.12837E-09	3.01305E-09
PCDHGB7	58.35293512	-0.530481757	0.087105002	-6.090141183	1.12811E-09	3.01276E-09
QRFP	32.16374696	-0.340489943	0.055903477	-6.090675594	1.12435E-09	3.00313E-09
ZSWIM5	31.60433838	-0.420269201	0.068996527	-6.09116459	1.12092E-09	2.99438E-09
TIGD4	4.818837578	-0.402121606	0.066016919	-6.091190163	1.12074E-09	2.99431E-09
WDR76	616.7663191	-0.221832283	0.036415237	-6.091743531	1.11688E-09	2.98438E-09
GABRA4	19.11754812	-1.233924177	0.202491738	-6.093701357	1.10329E-09	2.94849E-09
DVL2	1205.599459	-0.180472045	0.029610313	-6.094904968	1.09502E-09	2.9268E-09
SRGN	41906.44461	0.42819012	0.070245494	6.095624055	1.09011E-09	2.91407E-09
TADA1	676.0951283	0.176715469	0.028986195	6.096538984	1.0839E-09	2.89784E-09
ZBED9	1.633065535	-0.724866381	0.118895156	-6.09668556	1.0829E-09	2.89558E-09
ABHD2	7941.286587	0.226506706	0.037140928	6.098574207	1.07019E-09	2.86197E-09
CREG1	4229.811623	0.269376472	0.044167585	6.098963168	1.06759E-09	2.85541E-09
CCDC71	322.6962652	-0.222902856	0.036545449	-6.099332809	1.06512E-09	2.8492E-09
BROX	1859.468159	0.197582758	0.032391003	6.099927192	1.06117E-09	2.83902E-09
UPB1	380.9046617	0.424129651	0.069529936	6.09995741	1.06097E-09	2.83887E-09
SPATA20	1257.683192	-0.308850724	0.050630393	-6.100105265	1.05999E-09	2.83663E-09
FAM3D	1.334835193	-0.787893737	0.129139423	-6.101109313	1.05335E-09	2.81925E-09
SRSF5	12541.26292	0.161596433	0.026480322	6.102510058	1.04416E-09	2.79503E-09
SLC29A4	90.14685294	-0.494075613	0.080960652	-6.102663451	1.04315E-09	2.79273E-09

OR10K1	1.1642166	-0.766024614	0.125518702	-6.102872344	1.04179E-09	2.78946E-09
CKAP4	1310.04039	0.507017107	0.083065044	6.103856454	1.03539E-09	2.77271E-09
TTC4	23.03151928	-0.279545105	0.045795799	-6.104164767	1.0334E-09	2.76774E-09
CLEC20A	4.997968268	-0.800351447	0.131100532	-6.104868032	1.02886E-09	2.75596E-09
CORO1C	4799.207464	0.218168999	0.0357362	6.104985905	1.0281E-09	2.75431E-09
SOWAHB	1.84103451	-0.665232761	0.108958654	-6.105368743	1.02564E-09	2.74809E-09
PPP1R17	2.763084139	1.068390129	0.174982189	6.105707877	1.02346E-09	2.74263E-09
STAT6	6360.559252	-0.197210154	0.032292822	-6.106934707	1.01563E-09	2.72202E-09
UTP23	453.0824364	0.205087511	0.033570725	6.109117756	1.00183E-09	2.68541E-09
MT-ND4	483969.4941	-0.719729505	0.117767676	-6.111435091	9.87392E-10	2.64706E-09
TLCD4	425.2231737	-0.67944236	0.111169618	-6.111763007	9.85364E-10	2.64199E-09
TSPAN31	509.1951134	-0.148838574	0.024350781	-6.112271059	9.82232E-10	2.63395E-09
ABCC6	319.141549	-0.397810322	0.065081934	-6.112453911	9.81106E-10	2.6313E-09
FRMPD2	2.549381592	-0.700003978	0.114509097	-6.113086172	9.77226E-10	2.62125E-09
PBK	64.74691705	-0.500424953	0.081861204	-6.113090589	9.77199E-10	2.62125E-09
ZNF625	8.75227814	-0.474637234	0.077634193	-6.113765288	9.73074E-10	2.61083E-09
DHRS13	163.7263122	-0.313893803	0.051341139	-6.113884698	9.72346E-10	2.60923E-09
SLC4A1	20197.18898	-0.694208961	0.113543522	-6.114034086	9.71435E-10	2.60714E-09
MAP3K8	3166.563737	0.316526239	0.05176542	6.114627139	9.6783E-10	2.59782E-09
NDUFA11	432.8540279	-0.257085854	0.042009551	-6.119700163	9.37516E-10	2.5168E-09
ABCB1	594.8052114	0.574746911	0.093917425	6.11970472	9.37489E-10	2.5168E-09
LY6G5C	125.8694763	-0.282419752	0.046147349	-6.119956189	9.36011E-10	2.51345E-09
GALNT10	1585.252244	-0.240151204	0.039240217	-6.120027497	9.35592E-10	2.51267E-09
SLC22A3	9.877466391	-0.825314721	0.134850109	-6.120237706	9.34359E-10	2.5097E-09
GRIA3	11.09160189	-0.923745334	0.150926218	-6.120509401	9.32767E-10	2.50577E-09
TULP3	138.1773887	-0.222794543	0.036399905	-6.120745232	9.31387E-10	2.50241E-09
DRC1	12.08220438	-0.475519636	0.077684779	-6.121142927	9.29065E-10	2.49651E-09
PEBP4	2.468605158	-0.644518301	0.105289399	-6.121397858	9.2758E-10	2.49286E-09
MROH6	801.1134624	-0.312394571	0.051027655	-6.122064024	9.23709E-10	2.4828E-09
ELAVL1	2117.032268	-0.138332514	0.022594371	-6.122432526	9.21574E-10	2.4774E-09
UNC119	1112.244157	0.296791679	0.048473042	6.12281932	9.19339E-10	2.47173E-09
SNX10	663.2000282	0.364529385	0.059532259	6.123224443	9.17004E-10	2.46579E-09
SLC35C1	312.7551725	-0.26853502	0.043852813	-6.123552913	9.15114E-10	2.46105E-09
CNTNAP2	22.79978148	-0.567628207	0.092695695	-6.12356601	9.15039E-10	2.46105E-09
ACAT2	347.2310033	-0.173708851	0.028367239	-6.123572631	9.15001E-10	2.46105E-09
NKAPL	6.505597816	-0.503000356	0.082138065	-6.123839875	9.13467E-10	2.45763E-09
PRLHR	2.528708107	-0.709930052	0.115921236	-6.124245042	9.11146E-10	2.45173E-09

GNAI2	16421.70549	0.238692248	0.03897312	6.12453525	9.09487E-10	2.4476E-09
CHCHD5	377.8201323	-0.425807485	0.069497775	-6.126922559	8.95951E-10	2.4115E-09
KCTD9	1190.032297	0.239652804	0.039114379	6.126974502	8.95658E-10	2.41105E-09
ZDHHC8	1254.373677	-0.229569511	0.037465159	-6.127546655	8.92444E-10	2.40273E-09
SYN2	23.39809937	0.50080026	0.081714995	6.128621338	8.86438E-10	2.38688E-09
HMX3	273.3556912	-1.088947725	0.177679211	-6.128728969	8.85839E-10	2.3856E-09
ASRGL1	202.7547628	-0.444897865	0.072589156	-6.12898526	8.84413E-10	2.38209E-09
ACP7	2.38727364	-0.557211993	0.09091058	-6.129231521	8.83045E-10	2.37873E-09
SUB1	5951.542691	0.19523942	0.031851087	6.129756861	8.80135E-10	2.37122E-09
CCDC58	128.6103843	-0.228499792	0.037277035	-6.129773713	8.80042E-10	2.37122E-09
FER	702.2704052	-0.298520874	0.048690877	-6.130940571	8.7361E-10	2.35429E-09
GOLGA1	917.8835395	-0.19764184	0.032227083	-6.132787144	8.63527E-10	2.32743E-09
KIAA0232	2807.270804	0.231678141	0.03777557	6.133015102	8.6229E-10	2.32442E-09
MAP4K3	1274.844352	0.274020752	0.04467497	6.1336528	8.58838E-10	2.31543E-09
GNA14	53.34747486	-0.623092401	0.101584716	-6.133721935	8.58465E-10	2.31475E-09
CRELD1	81.38172441	-0.234305113	0.038196152	-6.13425957	8.55567E-10	2.30725E-09
GADD45GIP1	699.5377498	-0.493653569	0.080471209	-6.134536519	8.54078E-10	2.30355E-09
DGKD	6484.36796	0.222696292	0.036300551	6.134790893	8.52713E-10	2.30019E-09
COL9A3	73.7255444	-0.459866854	0.07495242	-6.135450361	8.49183E-10	2.29098E-09
REM1	2.021635344	-0.579285131	0.094407604	-6.136000772	8.46247E-10	2.28337E-09
IL5	5.123884032	-0.463617981	0.075549028	-6.136650516	8.42795E-10	2.27437E-09
PGA5	5.900271074	-0.629780712	0.10261574	-6.137272017	8.39506E-10	2.26581E-09
ANXA13	2.430954562	-0.73887661	0.120383295	-6.137700524	8.37245E-10	2.26002E-09
ADAMTS20	1.760267506	-1.040415775	0.169500877	-6.138114392	8.35067E-10	2.25445E-09
DIO2	6.007621676	-0.676542388	0.110207517	-6.138804402	8.31449E-10	2.24499E-09
ABCB10	1855.54846	-0.298677519	0.048638471	-6.140766987	8.2124E-10	2.21773E-09
PURB	2913.682801	0.155846047	0.025373126	6.142169671	8.14018E-10	2.19853E-09
TOMM20	6856.984305	0.198668906	0.032343695	6.142430783	8.12681E-10	2.19523E-09
DUSP12	542.4941143	0.254405082	0.04141333	6.143072322	8.09404E-10	2.18668E-09
TFAP2C	2.513590761	-0.885422127	0.144123059	-6.143514668	8.07152E-10	2.18089E-09
AICDA	3.808190246	-0.606707394	0.09874841	-6.143971245	8.04834E-10	2.17493E-09
H2AC12	1136.988761	-0.491224656	0.079941345	-6.144813482	8.00575E-10	2.16372E-09
BCL2A1	1438.830994	0.557151945	0.090669474	6.144867974	8.00301E-10	2.16328E-09
STRA6	9.948830946	-0.739827185	0.120384877	-6.145515994	7.9704E-10	2.15476E-09
KRTAP10-6	0.863447433	-1.19775493	0.194897889	-6.145551063	7.96864E-10	2.15458E-09
SHC1	1890.393569	0.178286144	0.029009951	6.145689317	7.9617E-10	2.153E-09
TIFA	573.9789079	0.273744715	0.044542201	6.145738378	7.95924E-10	2.15263E-09

PVR	331.3436784	-0.418973865	0.068167114	-6.14627551	7.93235E-10	2.14566E-09
SKAP1	184.5640674	0.415806408	0.067647956	6.146621917	7.91505E-10	2.14128E-09
DECR1	736.9734985	-0.245955737	0.040012617	-6.146954578	7.89847E-10	2.13709E-09
PIK3CG	5980.958285	0.265796417	0.043235824	6.147596882	7.86657E-10	2.12875E-09
GNRH2	20.16646848	-0.421109382	0.068497208	-6.147832783	7.85488E-10	2.12588E-09
CCDC40	36.98332879	-0.454118612	0.073841241	-6.149932054	7.75161E-10	2.09822E-09
PPM1K	1144.308328	-0.256801421	0.041755159	-6.150172255	7.73988E-10	2.09534E-09
OSBPL5	2411.709508	-0.323448524	0.052588679	-6.150535251	7.72219E-10	2.09083E-09
KCNMA1	47.07023581	-0.624620603	0.101554069	-6.150621126	7.71801E-10	2.08999E-09
ADCY7	2339.974954	-0.35613547	0.057901345	-6.150728832	7.71277E-10	2.08886E-09
THEM4	416.6408363	-0.375085317	0.060981651	-6.150789748	7.70981E-10	2.08835E-09
DISP1	193.890735	-0.308325643	0.050121759	-6.151532793	7.67377E-10	2.07887E-09
SPZ1	1.177709936	-1.026047465	0.166786412	-6.151864848	7.65771E-10	2.07481E-09
GYPE	176.7829511	-0.657944011	0.106941062	-6.152398351	7.63199E-10	2.06813E-09
GNAO1	26.07151142	-0.449605664	0.073065571	-6.153454469	7.58131E-10	2.05468E-09
GLIPR1L1	10.36087186	-0.454774779	0.073891234	-6.154651325	7.52428E-10	2.03951E-09
TCTN1	307.2941042	-0.252660925	0.041048454	-6.155187401	7.49888E-10	2.0329E-09
TARS3	378.3517255	-0.229075748	0.037215869	-6.155324404	7.4924E-10	2.03143E-09
C19orf38	716.4846971	0.452146217	0.073436246	6.156989797	7.41406E-10	2.01047E-09
DAD1	1538.939063	-0.414257287	0.067270366	-6.158094692	7.36253E-10	1.99677E-09
MN1	1922.697847	0.651580137	0.105774365	6.160095007	7.27013E-10	1.97198E-09
PTBP2	3248.236423	0.269902053	0.043814152	6.160156911	7.26729E-10	1.97149E-09
ACTRT3	21.50374729	-0.378183866	0.061383031	-6.161049066	7.22646E-10	1.96068E-09
LHFPL4	22.9387468	-0.475541346	0.077171514	-6.162135755	7.17703E-10	1.94754E-09
IGF2BP2	3353.90624	-0.330649501	0.053637826	-6.164483699	7.07135E-10	1.91913E-09
EBF3	52.7825324	0.672884089	0.109139553	6.165354997	7.03252E-10	1.90885E-09
H2AC13	1068.731509	-0.501235504	0.081298374	-6.165381642	7.03133E-10	1.9088E-09
C15orf40	407.9985679	-0.173170833	0.028084364	-6.166094089	6.99974E-10	1.90049E-09
KIAA1217	253.4686414	-0.854125709	0.138512675	-6.16640831	6.98586E-10	1.89698E-09
CCDC70	1.944731773	-0.907912276	0.147227638	-6.166724452	6.97191E-10	1.89345E-09
DGCR2	1594.460463	-0.212692391	0.034485949	-6.167508652	6.93743E-10	1.88435E-09
TCAP	69.41041575	0.392326948	0.06360628	6.16805371	6.91357E-10	1.87813E-09
SEPTIN14	2.858147985	-0.614680275	0.099646028	-6.168638027	6.88807E-10	1.87146E-09
MPO	154350.9309	0.650135728	0.105383607	6.169230146	6.86233E-10	1.86473E-09
SLC5A12	0.939411361	-1.517322362	0.245908208	-6.170279455	6.81694E-10	1.85265E-09
PPP3CB	1091.094307	-0.17188104	0.027852793	-6.171052115	6.78371E-10	1.84388E-09
WDR64	4.63667354	-0.566907159	0.091851277	-6.172011708	6.74265E-10	1.83297E-09

ID3	166.8869462	0.531877173	0.086168114	6.172552124	6.71964E-10	1.82697E-09
IMPA1	671.1795347	0.178970524	0.028990659	6.173385854	6.68428E-10	1.81761E-09
APOA4	1.482334282	-1.217716014	0.197215782	-6.174536336	6.63579E-10	1.80467E-09
SH3GL1	1980.118893	0.234521186	0.037976765	6.175386048	6.6002E-10	1.79524E-09
PHF20L1	2657.983933	0.205339186	0.033237164	6.17799962	6.49189E-10	1.76603E-09
SPINK1	4.619721961	1.123095574	0.181776417	6.178444884	6.47361E-10	1.7613E-09
SAMD1	873.4984732	-0.253810706	0.041079972	-6.178453723	6.47325E-10	1.7613E-09
GEMIN2	138.6249253	-0.194269796	0.03144209	-6.178654041	6.46504E-10	1.75946E-09
LLPH	507.769613	0.180734508	0.029248645	6.179243683	6.44094E-10	1.75314E-09
CPA2	2.80176829	-0.645191522	0.104389301	-6.180628795	6.38468E-10	1.73807E-09
ZNF700	636.8944291	-0.249690737	0.04039519	-6.181199775	6.36162E-10	1.73204E-09
CENPS	12.89099319	-0.372096595	0.060195042	-6.181515716	6.3489E-10	1.72881E-09
SKIL	2774.297912	0.40510291	0.065533377	6.181627234	6.34442E-10	1.72783E-09
PF4V1	5.190913712	0.915730793	0.148125008	6.182148489	6.3235E-10	1.72238E-09
SLC46A3	257.4936949	-0.323553939	0.052329755	-6.182982157	6.29018E-10	1.71354E-09
HSPB11	450.9390586	-0.249135162	0.040278313	-6.185342461	6.19678E-10	1.68833E-09
PCDHAC1	1.886076872	-0.892310655	0.144259995	-6.185433823	6.19319E-10	1.68759E-09
RPS6KA1	3110.391889	-0.249790605	0.040383295	-6.185493443	6.19085E-10	1.68718E-09
BBS1	39.8262032	-0.242885496	0.039261192	-6.186401505	6.15531E-10	1.67773E-09
AGBL5	812.1025935	-0.163715185	0.026463546	-6.18644163	6.15375E-10	1.67754E-09
HIPK1	15656.37208	0.185869741	0.030044611	6.186458655	6.15308E-10	1.67754E-09
DEK	3494.926875	0.204962954	0.033128247	6.186954371	6.13377E-10	1.67256E-09
RPRD2	2757.238467	0.167246624	0.027030894	6.187239859	6.12268E-10	1.66977E-09
NAA11	3.975511662	-0.642899396	0.103900806	-6.187626673	6.10768E-10	1.66591E-09
CALN1	234.2006297	-0.697480891	0.112703836	-6.188617141	6.06943E-10	1.65571E-09
PADI4	2024.799801	-0.538087724	0.086937576	-6.189357348	6.041E-10	1.64818E-09
HAO2	0.547090636	-1.033123768	0.166810139	-6.193411109	5.88759E-10	1.60655E-09
IGSF23	1.824830704	-0.711580204	0.114881673	-6.194027149	5.86461E-10	1.6005E-09
HUS1	943.807277	0.154835296	0.024997188	6.194108576	5.86158E-10	1.5999E-09
NPY1R	1.532351439	-1.106529983	0.178635378	-6.194349617	5.85262E-10	1.59768E-09
NLRC3	978.0724318	-0.338179723	0.054593843	-6.194466373	5.84828E-10	1.59671E-09
SEC14L1	4871.87467	0.23641235	0.03815905	6.195446364	5.81201E-10	1.58703E-09
GPR132	958.4307974	0.359293518	0.05798332	6.196497889	5.77333E-10	1.57669E-09
NWD1	12.36004921	-0.53448194	0.086243156	-6.197383851	5.74094E-10	1.56806E-09
ZBTB46	516.6241058	-0.433671601	0.069975264	-6.197498583	5.73676E-10	1.56714E-09
ZNF14	549.0894097	0.325796577	0.052557823	6.198821791	5.68874E-10	1.55424E-09
LTB4R2	169.7948905	-0.276831164	0.044655291	-6.199291504	5.67179E-10	1.54982E-09

PPP1R32	108.1494941	-0.214935488	0.034667249	-6.199958006	5.64782E-10	1.54349E-09
RAB3IP	331.9518901	-0.364334117	0.058762672	-6.200094447	5.64293E-10	1.54237E-09
FAM50B	37.2813934	-0.480813623	0.077530789	-6.201582957	5.5898E-10	1.52806E-09
FKBPL	94.06935968	-0.336804327	0.054297139	-6.202984821	5.54022E-10	1.51472E-09
KIF2C	547.1177404	-0.281315118	0.045346433	-6.203687937	5.51551E-10	1.50817E-09
SPHK2	326.5122083	-0.190232335	0.0306643	-6.203707132	5.51484E-10	1.50817E-09
SLC6A20	21.23750973	-0.618316236	0.099664958	-6.203948181	5.50639E-10	1.5061E-09
ZMAT5	170.5191395	-0.237579518	0.038292994	-6.204255448	5.49565E-10	1.50337E-09
TNFAIP8L3	11.42201514	0.620201216	0.099936467	6.205954968	5.43657E-10	1.48742E-09
SPNS1	133.8947442	0.268348422	0.043238868	6.206185247	5.42862E-10	1.48545E-09
HSF5	63.78649209	-0.354911278	0.057176524	-6.207290237	5.3906E-10	1.47525E-09
GNA12	3023.53619	0.295025668	0.047528264	6.207373128	5.38776E-10	1.47468E-09
STUB1	323.2805544	-0.264916562	0.04267705	-6.20747128	5.38439E-10	1.47397E-09
KCNAB1	320.0414949	0.407002818	0.065564066	6.207711712	5.37617E-10	1.47192E-09
PVALEF	3.309382453	-0.557598563	0.089809505	-6.208680984	5.34312E-10	1.46308E-09
C16orf74	121.8078044	-0.431185869	0.06944347	-6.209163681	5.32673E-10	1.4588E-09
EPHA7	1.393812438	-1.31248607	0.211333942	-6.210484035	5.28216E-10	1.44679E-09
EOGT	818.9435922	0.200027485	0.032204899	6.211088652	5.26188E-10	1.44144E-09
NCCRP1	3.152591489	-0.565959897	0.091118771	-6.211232758	5.25705E-10	1.44032E-09
NPIPA2	2.583509764	-0.689933956	0.111076163	-6.21135931	5.25282E-10	1.43936E-09
C16orf87	945.6064724	0.225731168	0.036335929	6.21234057	5.22011E-10	1.4306E-09
MTRF1	383.5996519	-0.156599197	0.025207742	-6.212345272	5.21996E-10	1.4306E-09
CYP4V2	382.6204551	-0.415665993	0.066909455	-6.212365552	5.21928E-10	1.4306E-09
PSMA1	757.4410891	0.198262792	0.031911086	6.21297538	5.19906E-10	1.42543E-09
UBAP1	4408.413981	0.261759417	0.042126233	6.213691479	5.17541E-10	1.41914E-09
F12	93.71093456	-0.446455551	0.071847711	-6.213914775	5.16806E-10	1.41732E-09
MICALL1	1618.809593	0.247018046	0.039750698	6.214181388	5.15929E-10	1.41512E-09
CRADD	301.8818507	-0.327682317	0.052720943	-6.215410746	5.11906E-10	1.40428E-09
ZNF746	2360.86352	0.204000254	0.032818985	6.215921997	5.10241E-10	1.39991E-09
OCSTAMP	8.916665032	-0.748735003	0.120438161	-6.216758849	5.07529E-10	1.39266E-09
SCN4B	22.4593693	0.563918582	0.090707911	6.216862171	5.07195E-10	1.39194E-09
SNAPC3	692.4014156	-0.223141711	0.035890219	-6.217340393	5.05652E-10	1.3879E-09
KCNK6	513.7084502	-0.448218583	0.072084151	-6.21799077	5.03561E-10	1.38236E-09
CEP70	713.4514376	-0.329781739	0.053035439	-6.218139179	5.03085E-10	1.38124E-09
THSD7B	2.589831625	-0.86004002	0.138309637	-6.218221961	5.0282E-10	1.38071E-09
FAM122B	1036.209306	-0.178024366	0.028628951	-6.218333529	5.02463E-10	1.37992E-09
KLF11	1471.731803	0.431573532	0.069403181	6.218353756	5.02398E-10	1.37992E-09

HPR	14.69999163	-0.388692785	0.062501072	-6.218977905	5.00404E-10	1.37465E-09
ELOB	2237.188509	-0.430890467	0.069285861	-6.219024498	5.00255E-10	1.37444E-09
HS3ST5	8.107182545	-0.714248199	0.114840122	-6.219500506	4.9874E-10	1.37047E-09
TCTN3	671.2676684	-0.211542248	0.034011512	-6.219724972	4.98027E-10	1.3687E-09
MATK	913.3925702	-0.337512379	0.054264242	-6.219793452	4.9781E-10	1.3683E-09
GLYATL1	11.85735757	-0.592825534	0.095289079	-6.221337628	4.92934E-10	1.35508E-09
CLN5	1079.991471	0.261032659	0.04195696	6.221438793	4.92616E-10	1.3544E-09
CTSO	377.2187894	0.31197011	0.050132332	6.222932336	4.87948E-10	1.34175E-09
TTC17	3798.780355	-0.164395365	0.026417148	-6.223055015	4.87566E-10	1.34089E-09
ZNF703	314.0796322	0.619673091	0.09956468	6.223824428	4.8518E-10	1.33452E-09
DPPA3	1.114042115	-1.012472972	0.162663972	-6.224322192	4.83642E-10	1.33048E-09
GIPR	129.8708864	-0.600665782	0.096501928	-6.224391495	4.83429E-10	1.33007E-09
SPAST	1132.410312	0.15463232	0.02483855	6.225496952	4.80032E-10	1.32091E-09
PATE1	0.733970645	-0.898392223	0.144294676	-6.226094033	4.78207E-10	1.31608E-09
CCDC174	809.716906	0.172171048	0.027651599	6.226440848	4.7715E-10	1.31335E-09
PCDHB5	2.448376853	-1.053808309	0.16924549	-6.226507487	4.76947E-10	1.31298E-09
ARRDC1	1357.172856	-0.210738072	0.033843693	-6.226804823	4.76044E-10	1.31068E-09
STK24	2228.827376	0.259291276	0.041630975	6.228325799	4.71446E-10	1.2982E-09
ESPN	172.6871496	-0.675281814	0.108411277	-6.228889009	4.69754E-10	1.29372E-09
PPP1R14D	1.825522562	-0.552474793	0.08868917	-6.229337713	4.68411E-10	1.29021E-09
ATG7	1763.429946	-0.292452242	0.046926548	-6.232127717	4.60142E-10	1.26761E-09
MTMR4	1968.442755	-0.210068943	0.033701592	-6.233205321	4.56987E-10	1.25909E-09
OR6Y1	1.355183923	-1.09308476	0.175363045	-6.233267456	4.56805E-10	1.25877E-09
MTRR	1334.42388	0.165228541	0.02650619	6.233583215	4.55885E-10	1.25641E-09
TMEM147	582.5843394	-0.391507307	0.062804997	-6.233696736	4.55555E-10	1.25568E-09
TSPAN14	3732.717866	0.279847899	0.044890325	6.23403593	4.54569E-10	1.25314E-09
SH2D1B	25.77629486	0.579289092	0.092919635	6.234302312	4.53796E-10	1.25118E-09
WDR37	2045.772645	0.199758121	0.032039662	6.234713816	4.52605E-10	1.24807E-09
KIF5C	76.51428149	0.464184812	0.074438279	6.235834853	4.49375E-10	1.23934E-09
TEAD4	32.31070821	-0.699491644	0.112166246	-6.236204468	4.48315E-10	1.23659E-09
CALU	1983.833044	0.201947281	0.032380916	6.236614274	4.47143E-10	1.23353E-09
GCAT	218.1432596	-0.374626751	0.060060813	-6.237457197	4.44741E-10	1.22708E-09
MAGEE2	0.558358926	-1.267561405	0.203201985	-6.237938103	4.43376E-10	1.22349E-09
TMED3	1379.810673	-0.26822535	0.042997305	-6.238189816	4.42663E-10	1.22169E-09
PLEKHA3	1749.305046	0.196547851	0.031501864	6.239245034	4.39688E-10	1.21365E-09
MTRNR2L10	25.48901256	-1.033233378	0.165593282	-6.239585135	4.38733E-10	1.21119E-09
ORC6	441.3766169	-0.240583095	0.038551119	-6.240625496	4.35825E-10	1.20333E-09

COG3	1521.023966	0.17513925	0.028063333	6.240857128	4.3518E-10	1.20172E-09
WDR49	875.9413262	-0.531734316	0.085196385	-6.241277912	4.3401E-10	1.19866E-09
ELL	2212.01208	0.26299875	0.04213845	6.241300936	4.33946E-10	1.19865E-09
ZNF224	328.6234209	-0.207828805	0.033297465	-6.241580364	4.33172E-10	1.19668E-09
BHLHA9	1.354922201	-0.65751155	0.105327878	-6.242521561	4.30572E-10	1.18967E-09
FBXO3	2436.913908	0.272142759	0.04359191	6.242964781	4.29354E-10	1.18647E-09
JMJD7-PLA2G4B	204.902874	-0.282004287	0.045162514	-6.244211416	4.25944E-10	1.17721E-09
DENND2D	818.0189961	-0.266333265	0.042649202	-6.244742003	4.245E-10	1.17339E-09
ASCC2	2610.967901	-0.203115418	0.032525104	-6.244881515	4.24121E-10	1.1725E-09
NCAN	7.153698287	-0.554671138	0.088787608	-6.247168393	4.1796E-10	1.15563E-09
MDFI	107.5689254	-0.745875296	0.119384666	-6.24766416	4.16636E-10	1.15214E-09
C22orf31	1.021683055	-0.928962088	0.148683836	-6.247902336	4.16002E-10	1.15054E-09
CBLN1	1.235171797	-0.799671392	0.127988404	-6.247998789	4.15745E-10	1.15E-09
NBPF11	618.7923991	-0.227869012	0.036464657	-6.249037599	4.12989E-10	1.14254E-09
LGALS2	257.1462	0.832444035	0.133209615	6.249128742	4.12749E-10	1.14203E-09
TEN1-CDK3	27.7865729	-0.294761533	0.04716332	-6.249804524	4.10967E-10	1.13726E-09
SLC25A44	2025.05864	0.219590562	0.035132614	6.250333783	4.09576E-10	1.13357E-09
FRMPD4	1.5580408	-0.876540458	0.140229478	-6.250757453	4.08467E-10	1.13066E-09
GALR3	10.25011515	-0.49248607	0.078780995	-6.251330937	4.06969E-10	1.12668E-09
KCND2	1.146599325	-1.444947719	0.231142235	-6.251335769	4.06957E-10	1.12668E-09
SUPT5H	5826.594862	0.174596894	0.027918386	6.253831853	4.00502E-10	1.10909E-09
NDUFAF6	684.3791168	-0.180199876	0.028810578	-6.254642874	3.98427E-10	1.1035E-09
NPAP1	1.330252358	-0.907025918	0.145014518	-6.254724926	3.98217E-10	1.10307E-09
SLC26A8	217.1820713	-0.445901182	0.071273261	-6.256219736	3.94421E-10	1.09271E-09
LYNX1	20.75002662	-0.657172992	0.105020927	-6.257543211	3.91089E-10	1.08363E-09
RHCE	398.3497426	-0.606207678	0.096872445	-6.257792682	3.90464E-10	1.08206E-09
KRTAP10-2	1.37987074	-1.155599529	0.184665498	-6.257798778	3.90449E-10	1.08206E-09
SLC12A8	29.75472661	-0.539134243	0.086147827	-6.258245444	3.89333E-10	1.07923E-09
CEP78	601.9219052	-0.219388979	0.03505389	-6.258619982	3.88399E-10	1.07679E-09
NT5DC4	18.87467111	-0.567519944	0.090661041	-6.259799523	3.85473E-10	1.06883E-09
THSD8	4.019771462	-0.612553164	0.097845671	-6.260401274	3.83988E-10	1.06486E-09
ST8SIA5	10.03584706	-0.519926384	0.08301964	-6.262691356	3.78389E-10	1.04948E-09
HDAC1	2797.165127	-0.148130262	0.023651439	-6.263054899	3.77508E-10	1.04719E-09
SAMD3	46.51494751	0.473737645	0.075634297	6.263529432	3.7636E-10	1.04415E-09
ZBTB6	233.0030109	0.265979269	0.042453437	6.265199928	3.72347E-10	1.03317E-09
NTNG1	35.00369391	-0.773597871	0.123454024	-6.266283157	3.69768E-10	1.02615E-09
ZNF222	165.2534878	0.325332882	0.051913657	6.266807224	3.68526E-10	1.02285E-09

BCAS2	814.6625925	0.265635203	0.042387643	6.266807649	3.68525E-10	1.02285E-09
LMNA	8001.345834	0.451169031	0.071990743	6.267042273	3.6797E-10	1.0216E-09
MCIDAS	0.940016138	-0.920090793	0.14678518	-6.268281265	3.65055E-10	1.01365E-09
CFAP206	4.711480998	-0.578546302	0.092297307	-6.268290147	3.65034E-10	1.01365E-09
TCHP	750.3797309	-0.154319677	0.024613964	-6.269598747	3.6198E-10	1.0054E-09
ELOVL3	9.457892422	-0.645507765	0.102944235	-6.270460558	3.59982E-10	9.99989E-10
PYDC1	19.94920633	-0.678790739	0.108250005	-6.270583888	3.59697E-10	9.99339E-10
SGMS2	310.5712059	0.549127688	0.087571781	6.270600912	3.59657E-10	9.99339E-10
HPD	58.31328263	-0.425147815	0.067794345	-6.271139813	3.58415E-10	9.9606E-10
ANKRD30B	1.844340344	-1.234098077	0.196778331	-6.271514114	3.57554E-10	9.93809E-10
OR1J2	1.137983872	-1.120604229	0.178675718	-6.271720883	3.57079E-10	9.92631E-10
UCMA	0.800065721	-0.957766446	0.152709815	-6.271806713	3.56882E-10	9.92224E-10
ISCU	2372.222225	0.22287122	0.035533818	6.272087587	3.56239E-10	9.90576E-10
MYH8	1.800007563	-1.056721884	0.168456697	-6.272958594	3.54251E-10	9.85189E-10
KIF23	546.428175	-0.341510278	0.054431014	-6.274185464	3.51469E-10	9.77591E-10
TTC19	2428.964032	0.164566105	0.026228817	6.274248016	3.51328E-10	9.77337E-10
CIC	6149.77628	0.230211067	0.036683655	6.275576105	3.48342E-10	9.69168E-10
RBP7	33.52383624	0.657963892	0.104840005	6.275885713	3.47649E-10	9.67379E-10
BFSP1	19.47197623	-0.521900764	0.083129192	-6.278188803	3.4254E-10	9.53296E-10
BTF3L4	1808.388115	0.166885874	0.02658091	6.278410816	3.42051E-10	9.52072E-10
CLEC3A	0.898981415	-0.955667036	0.152211368	-6.278552315	3.4174E-10	9.51341E-10
DENND4C	2481.975317	0.265049283	0.04221073	6.279192065	3.40337E-10	9.4757E-10
SEC22A	164.6821004	-0.188126436	0.02995913	-6.279435809	3.39804E-10	9.4622E-10
COX5B	2489.584224	-0.370526752	0.059001755	-6.279927686	3.38731E-10	9.43366E-10
CLEC4E	167.3980049	0.691241493	0.110026154	6.282519795	3.33129E-10	9.27897E-10
TMEM275	0.974909465	-0.783518666	0.12469451	-6.283505705	3.31022E-10	9.2216E-10
PSMA5	1263.393965	-0.229374554	0.036504191	-6.283512857	3.31007E-10	9.2216E-10
B3GALT1	13.04755819	-0.647473872	0.103025064	-6.28462482	3.28646E-10	9.15802E-10
FANCI	2040.081973	-0.225741848	0.035917285	-6.285047593	3.27753E-10	9.13444E-10
OR5AU1	24.89245383	-0.783494929	0.124656982	-6.285206963	3.27417E-10	9.12637E-10
LHPP	435.1705271	-0.291028449	0.046300221	-6.285681637	3.26418E-10	9.09982E-10
ABCA9	35.02238572	-0.589512295	0.093782851	-6.285928501	3.259E-10	9.08667E-10
SRCAP	491.0059263	0.190925177	0.030369993	6.286638744	3.24413E-10	9.0465E-10
CDC123	1388.808088	0.19414956	0.030881204	6.286981668	3.23698E-10	9.02783E-10
PLAC1	2.029342923	-0.661526588	0.105200933	-6.288219799	3.21127E-10	8.95742E-10
CCDC50	1129.225546	0.51211638	0.081437473	6.288461107	3.20628E-10	8.94478E-10
GTF2H3	458.8225119	-0.195291459	0.031054388	-6.28869128	3.20153E-10	8.93281E-10

UBE2C	445.3686088	-0.388644301	0.06178758	-6.290006857	3.17452E-10	8.85869E-10
ZFYVE16	1066.586241	0.276259511	0.0439187	6.290247902	3.16959E-10	8.84621E-10
POLB	1170.725016	0.194761975	0.030962169	6.290320797	3.16811E-10	8.84332E-10
SMC3	2366.935162	0.220492244	0.03504967	6.290850768	3.15731E-10	8.81443E-10
MED26	745.2203631	0.262257491	0.041687634	6.291014019	3.15399E-10	8.80642E-10
MORC3	2799.88458	0.207846249	0.033037831	6.291159013	3.15104E-10	8.79945E-10
OR14K1	1.895033282	-0.844003933	0.134131936	-6.292341375	3.12713E-10	8.73392E-10
CLIC6	7.237966036	-0.607518155	0.096532203	-6.293424749	3.10537E-10	8.67439E-10
EFL1	643.9017806	-0.160510171	0.025501772	-6.294079056	3.0923E-10	8.63911E-10
SLAIN2	1737.542663	0.15091172	0.023976228	6.294222741	3.08944E-10	8.63235E-10
RHD	452.8615509	-0.597416346	0.094913332	-6.294335384	3.0872E-10	8.62731E-10
CHRM2	1.113709735	-1.020656756	0.162129346	-6.295323951	3.06759E-10	8.57373E-10
DNAH12	57.85725898	-0.412997371	0.065594755	-6.296195026	3.05041E-10	8.52693E-10
BET1	154.1931185	-0.254650886	0.04044335	-6.296483489	3.04474E-10	8.5123E-10
DDX60	627.6093752	-0.37559948	0.05963549	-6.2982543	3.01016E-10	8.41685E-10
KIFC2	521.5439603	-0.36018609	0.057186812	-6.298411801	3.00711E-10	8.4095E-10
LARP4	1316.355542	-0.182557532	0.028984582	-6.298435823	3.00664E-10	8.4094E-10
HLA-F	1072.673103	0.362137438	0.057488204	6.299334726	2.98926E-10	8.36198E-10
PTPRZ1	1.511369225	-1.244514849	0.197535598	-6.300205436	2.97251E-10	8.31633E-10
NFYC	1040.599969	-0.163317104	0.025920598	-6.300668712	2.96364E-10	8.29269E-10
RHBDL2	24.20199464	-0.380579778	0.060386883	-6.302358428	2.9315E-10	8.20393E-10
OR1L4	2.279204818	-0.615122635	0.097600257	-6.302469413	2.9294E-10	8.19923E-10
SCN10A	1.327607585	-0.855092476	0.13567573	-6.302471893	2.92936E-10	8.19923E-10
LRRC34	117.5650785	-0.280577815	0.044514114	-6.303120255	2.91712E-10	8.1672E-10
TFAP2B	1.54396603	-1.532129478	0.24306553	-6.303359754	2.91262E-10	8.15575E-10
PRRC2C	19924.19408	0.179988913	0.02855321	6.30363139	2.90752E-10	8.14262E-10
XYLT1	2890.933992	-0.330211209	0.05238178	-6.303932578	2.90187E-10	8.12797E-10
MOV10L1	20.61265486	-0.569903196	0.09039969	-6.3042605	2.89573E-10	8.11194E-10
OTULINL	925.4599634	0.416289196	0.066031398	6.304412891	2.89288E-10	8.10513E-10
OR1D2	5.164537618	-0.659073401	0.104535704	-6.304768386	2.88625E-10	8.0877E-10
TNNT2	1.827172807	-0.687530738	0.109026012	-6.306116565	2.86124E-10	8.01875E-10
ATP2A1	222.2329701	-0.287785917	0.045629719	-6.30698415	2.84525E-10	7.97509E-10
MKI67	5013.229876	-0.384999271	0.061042005	-6.307120366	2.84275E-10	7.96922E-10
ANXA10	4.009029825	-0.691563361	0.109630918	-6.30810515	2.82472E-10	7.91983E-10
FAM110C	1.926463697	-0.932626903	0.147825123	-6.308987839	2.80866E-10	7.87592E-10
UFD1	2061.839278	0.185510142	0.02940048	6.309765769	2.79458E-10	7.83756E-10
ZDHHC18	2547.987416	0.333237486	0.052810489	6.310062504	2.78923E-10	7.82367E-10

LIN9	185.3954257	-0.212843	0.033728667	-6.31044795	2.78229E-10	7.80533E-10
TUBB6	1850.498065	0.404711221	0.064131649	6.310631717	2.77899E-10	7.79718E-10
NOXA1	165.3199267	-0.404159093	0.064038717	-6.311167895	2.76938E-10	7.77132E-10
AC005551.1	50.51541084	-0.450048581	0.07130766	-6.311363791	2.76587E-10	7.7626E-10
ZNF814	1336.393465	-0.253104556	0.04009663	-6.312364743	2.74804E-10	7.71365E-10
MRPL41	469.2860115	-0.358532791	0.056791027	-6.313194352	2.73334E-10	7.6735E-10
MMS22L	969.7144043	-0.250748137	0.039716167	-6.31350285	2.72789E-10	7.65931E-10
SLC10A6	7.954151816	-0.49417441	0.078272283	-6.313530031	2.72741E-10	7.65906E-10
SLC4A3	67.74827158	-0.690147649	0.109304395	-6.313997245	2.71919E-10	7.63706E-10
RBBP4	4187.144358	-0.146172685	0.023146101	-6.315218343	2.6978E-10	7.57809E-10
STMN1	2897.920251	-0.338419372	0.053581347	-6.315992217	2.68434E-10	7.54134E-10
PPIE	847.9258052	-0.221355914	0.035038527	-6.3175006	2.65827E-10	7.46919E-10
DMAP1	1020.755053	-0.163158718	0.025822461	-6.318480449	2.64148E-10	7.42306E-10
RRAGD	989.6526623	0.319242674	0.050516491	6.319573445	2.62286E-10	7.37181E-10
DDIT3	1169.019147	0.348020518	0.055066902	6.319958224	2.61634E-10	7.35453E-10
ZNF831	177.5354679	0.465297075	0.073622998	6.319996341	2.61569E-10	7.35378E-10
CFHR3	0.778477846	-1.894710821	0.299717336	-6.321659097	2.5877E-10	7.27611E-10
ULBP2	21.45258431	0.617803366	0.097725236	6.321840628	2.58466E-10	7.26861E-10
KPNA6	4754.407681	0.178766462	0.02827717	6.321936037	2.58306E-10	7.26517E-10
C1orf198	225.479304	-0.392319863	0.062052808	-6.32235473	2.57607E-10	7.24654E-10
MRPL14	316.3737177	-0.350108933	0.055374599	-6.322554743	2.57274E-10	7.23821E-10
ALMS1	2642.063842	-0.260273685	0.041159406	-6.323552983	2.55617E-10	7.19261E-10
ATP13A2	1102.724258	-0.332983353	0.052656868	-6.323645261	2.55464E-10	7.18935E-10
PTH2R	384.1545856	-0.512650838	0.081067256	-6.323771904	2.55254E-10	7.18449E-10
TMEM54	3.724205841	-0.611913489	0.096751705	-6.324575769	2.53929E-10	7.14822E-10
FBXO44	228.0715559	-0.33513191	0.052982621	-6.325317698	2.52712E-10	7.11498E-10
MPRIP	5717.652066	-0.208337705	0.032936217	-6.325489908	2.5243E-10	7.10807E-10
OR14A2	1.008608083	-1.049856411	0.165960154	-6.325954682	2.51672E-10	7.08773E-10
COX15	612.4123991	-0.198810635	0.031424367	-6.32663924	2.50558E-10	7.05738E-10
C11orf87	3.884854613	-1.151569306	0.181978115	-6.32806482	2.48255E-10	6.99351E-10
OR2C1	2.244733339	-0.515933662	0.081530315	-6.328120575	2.48165E-10	6.99199E-10
VCPIP1	3551.37588	0.196003039	0.030972171	6.328359716	2.47781E-10	6.98217E-10
AC017083.3	2.9654224	-0.545519951	0.086192458	-6.329091457	2.46609E-10	6.95015E-10
STIL	852.2969119	-0.265073527	0.041881075	-6.329195846	2.46442E-10	6.94645E-10
CYP2C18	0.840384577	-1.100821885	0.17388637	-6.330696782	2.44057E-10	6.88019E-10
FGF22	12.36241372	-0.430998881	0.068074093	-6.331320225	2.43072E-10	6.85343E-10
UTP11	834.26509	0.195511792	0.030873003	6.332775289	2.4079E-10	6.79007E-10

CDHR4	44.56439821	-0.37258954	0.058817965	-6.334621415	2.37925E-10	6.71023E-10
MMADHC	1808.164742	0.218312453	0.034461781	6.334915032	2.37472E-10	6.69843E-10
SUMO1	1866.738802	0.260530051	0.041119471	6.33592913	2.35915E-10	6.65547E-10
C1QTNF2	4.595699886	-0.687252246	0.108459628	-6.33647981	2.35074E-10	6.6327E-10
ABCA1	4323.922043	0.458521223	0.072344161	6.338054294	2.32685E-10	6.56623E-10
RANBP3L	15.09451759	-0.64796388	0.102222347	-6.338769342	2.31608E-10	6.53678E-10
WDR19	679.219624	-0.241812074	0.038146439	-6.339047141	2.3119E-10	6.52595E-10
ANKS1B	13.21147518	-0.467053241	0.073665502	-6.340189452	2.29483E-10	6.47868E-10
DNAJC18	270.2458737	-0.245958639	0.038792846	-6.340309222	2.29304E-10	6.47458E-10
HAVCR1	56.89875149	0.564929564	0.08909526	6.340736469	2.28669E-10	6.45758E-10
KSR2	10.48245131	-0.576687002	0.09094266	-6.341215464	2.27959E-10	6.43846E-10
AC073111.3	214.2986628	-0.279496619	0.044075758	-6.341277609	2.27868E-10	6.43679E-10
LSM2	855.8200495	-0.343404693	0.054153589	-6.341309999	2.2782E-10	6.43636E-10
SULF1	11.78209428	-0.799778097	0.126116959	-6.341558707	2.27452E-10	6.42691E-10
RGPD4	31.1689224	0.37746718	0.059516793	6.342196251	2.26513E-10	6.40128E-10
PARD6B	99.23160922	-0.384201723	0.060578436	-6.342219231	2.26479E-10	6.40125E-10
HAUS7	34.02647974	-0.266763125	0.04204973	-6.343991403	2.23887E-10	6.32892E-10
ITPKA	109.5277824	-0.512185399	0.080731123	-6.344336356	2.23386E-10	6.31567E-10
PTPDC1	119.0668504	-0.349472691	0.055081521	-6.344644908	2.22939E-10	6.30394E-10
C3orf22	8.522616907	-0.575549889	0.090697314	-6.345831713	2.21227E-10	6.25643E-10
HSPA14	642.5917754	0.243320419	0.038338971	6.346555739	2.20189E-10	6.22797E-10
OR1N1	1.316940792	-1.057184322	0.166553162	-6.347428725	2.18943E-10	6.19364E-10
RBFOX2	141.6883375	-0.630597288	0.099332795	-6.348329248	2.17666E-10	6.15839E-10
RGCC	3637.987461	0.486730182	0.076657353	6.349425901	2.1612E-10	6.11553E-10
COA7	263.58094	-0.249279482	0.039250188	-6.351039209	2.13865E-10	6.05261E-10
BCKDK	1139.729866	-0.261884429	0.041229539	-6.351864187	2.12721E-10	6.0211E-10
GATAD1	1112.339192	-0.184884178	0.029106298	-6.352033374	2.12487E-10	6.01535E-10
RAB25	5.774711034	-0.444671572	0.070000951	-6.352364747	2.1203E-10	6.00327E-10
GALNT15	9.816987953	-0.562757883	0.08859008	-6.352380315	2.12008E-10	6.00327E-10
ADAMTSL5	7.567064985	-0.485075032	0.076342008	-6.353972669	2.09824E-10	5.94254E-10
AC008397.1	6.331458981	-0.671790405	0.105707882	-6.355159064	2.08211E-10	5.89771E-10
NOL8	1132.159621	-0.198178528	0.03118377	-6.355181915	2.0818E-10	5.89768E-10
TRAPPC3	775.4062268	-0.177752136	0.02796096	-6.357154282	2.05526E-10	5.82332E-10
METTL21A	549.708548	-0.179128527	0.028176545	-6.357362983	2.05247E-10	5.81626E-10
ZCCHC7	1183.054203	-0.225107023	0.035407316	-6.357641613	2.04875E-10	5.80657E-10
ANXA11	5872.669602	0.180941485	0.028460012	6.35774461	2.04737E-10	5.80351E-10
ARRB2	4990.56552	0.315786754	0.049668091	6.357940182	2.04477E-10	5.79697E-10

STK33	28.77971427	-0.639388134	0.100559916	-6.358280329	2.04025E-10	5.78499E-10
TOR3A	1055.210557	0.204161224	0.032102357	6.359695844	2.02154E-10	5.73276E-10
OR5A1	1.626194177	-0.920191355	0.144673993	-6.360447629	2.01167E-10	5.7056E-10
NATD1	1902.656028	-0.443725802	0.069757665	-6.360961198	2.00495E-10	5.68738E-10
AJUBA	37.27826784	-0.48527817	0.076265698	-6.362993877	1.97859E-10	5.6134E-10
CTXND2	1.646711005	-0.755881015	0.118736498	-6.366037648	1.93974E-10	5.50399E-10
ZNF24	2190.367993	0.204468917	0.032106736	6.368411771	1.90996E-10	5.42026E-10
CFAP20DC	20.33061602	-0.536029028	0.08416482	-6.36880146	1.90511E-10	5.4073E-10
SPIN3	82.01433967	-0.479485699	0.075283237	-6.369089867	1.90153E-10	5.39792E-10
GALNT1	2764.908316	0.219349963	0.034434709	6.37002525	1.88997E-10	5.36588E-10
TYMS	446.9243373	-0.269192317	0.042254614	-6.370720114	1.88143E-10	5.3424E-10
TMEM139	6.567791381	-0.419916806	0.065898588	-6.372166991	1.86376E-10	5.293E-10
TTC30A	53.78443672	-0.340645207	0.053458299	-6.372167018	1.86376E-10	5.293E-10
SPACA9	134.3172902	-0.441394582	0.069260202	-6.372990132	1.85378E-10	5.26618E-10
ANXA7	3051.415809	0.159501959	0.025027086	6.373173314	1.85156E-10	5.26066E-10
C1orf53	18.38115656	-0.53365763	0.083728874	-6.373639165	1.84595E-10	5.24546E-10
SENP8	55.38144907	-0.299923076	0.047053004	-6.374153567	1.83976E-10	5.22864E-10
FLI1	2572.607246	-0.262219162	0.041133402	-6.374847351	1.83145E-10	5.20578E-10
H2AZ2	8181.739495	0.154207487	0.024187779	6.375429736	1.82451E-10	5.18679E-10
TSNAX	823.1947	0.226439433	0.035516476	6.375616648	1.82228E-10	5.18122E-10
ACTR5	455.4202373	-0.209966466	0.032926103	-6.376900034	1.80708E-10	5.13875E-10
YWHAQ	6976.559409	0.203807786	0.031960169	6.376930827	1.80672E-10	5.13847E-10
NUTM2A	64.69676504	-0.385320066	0.060419116	-6.377452903	1.80057E-10	5.12173E-10
UMAD1	163.0350827	-0.238254405	0.037357631	-6.377663661	1.7981E-10	5.11543E-10
ZNF385A	2303.910571	0.46195471	0.072430808	6.377875976	1.79561E-10	5.10909E-10
H4C1	360.1753193	-0.484892297	0.076019029	-6.378564758	1.78755E-10	5.08691E-10
TBKBP1	752.12238	-0.311157067	0.048780297	-6.378744786	1.78545E-10	5.08167E-10
GGPS1	125.2227857	-0.209766088	0.032883017	-6.379161914	1.7806E-10	5.06859E-10
LRRC8B	1152.222372	0.31825146	0.049885514	6.379636748	1.77509E-10	5.05364E-10
NAA10	478.5504385	-0.210120169	0.032932403	-6.380347386	1.76687E-10	5.03097E-10
ZFYVE28	155.2014785	0.42662669	0.066858488	6.381040019	1.75889E-10	5.00899E-10
SLC44A3	9.944586257	-0.740593447	0.116059059	-6.381177441	1.75732E-10	5.00523E-10
ACAP3	1881.304143	-0.264361729	0.041426728	-6.381429074	1.75443E-10	4.99774E-10
PRRG1	20.67151142	-1.072140077	0.168008166	-6.381475975	1.75389E-10	4.99693E-10
ZBTB9	182.6422505	-0.243141728	0.03810037	-6.381610732	1.75235E-10	4.99326E-10
CHST8	2.394643114	-0.764291953	0.11975242	-6.382267313	1.74485E-10	4.97262E-10
FAM209A	48.75142812	0.294547601	0.046138699	6.38395976	1.72566E-10	4.91866E-10

EML1	8.466174051	-0.619377561	0.096997567	-6.38549586	1.70843E-10	4.87024E-10
ASB10	1.516826745	-1.034194633	0.161947394	-6.385991216	1.70291E-10	4.85521E-10
PAPSS2	194.9307657	0.37619564	0.058908999	6.386047084	1.70229E-10	4.85414E-10
IL13	23.63525016	-0.477140497	0.074715584	-6.386090678	1.7018E-10	4.85347E-10
LGR4	276.7966385	-0.691015237	0.108201666	-6.386364138	1.69876E-10	4.84551E-10
USP7	6574.38742	0.190131995	0.029765608	6.387640281	1.68465E-10	4.80596E-10
ZNF117	686.0394244	-0.316674469	0.049573883	-6.387929511	1.68147E-10	4.79758E-10
ZFP3	108.7642272	-0.39459616	0.061764773	-6.38869283	1.6731E-10	4.77439E-10
NDUFAF4	205.7347784	-0.3272591	0.051214782	-6.389934431	1.65957E-10	4.73647E-10
ERICH2	0.828544665	-1.185084133	0.185459658	-6.389983375	1.65904E-10	4.73565E-10
RRP7A	1118.223042	-0.205738381	0.032195143	-6.39035464	1.65501E-10	4.72485E-10
TMEM130	13.63771594	-0.460621208	0.072079646	-6.390447668	1.65401E-10	4.72267E-10
GRIN2C	520.7603915	0.381240699	0.059649052	6.391395757	1.64378E-10	4.69416E-10
TRMT61B	395.7431564	-0.148541441	0.023237042	-6.392441906	1.63257E-10	4.66283E-10
NUDT14	111.2387767	-0.450809788	0.070504733	-6.394035799	1.61564E-10	4.61513E-10
QRICH2	391.5028395	-0.415459444	0.064974508	-6.394191506	1.61399E-10	4.6111E-10
PYGM	424.3337639	-0.480886755	0.075205398	-6.394311691	1.61272E-10	4.60815E-10
DNASE2B	1.214391584	-0.736407341	0.115123038	-6.396698304	1.58773E-10	4.53739E-10
SPA17	24.34050647	-0.347993899	0.054399876	-6.396961253	1.585E-10	4.53024E-10
IFNW1	2.487035933	-0.563088087	0.088002789	-6.398525473	1.56885E-10	4.48474E-10
IFFO2	1714.755937	0.382811644	0.059816022	6.39981788	1.55562E-10	4.44759E-10
TEX11	1.577929362	-0.746976325	0.116714255	-6.400043619	1.55333E-10	4.44167E-10
SEC22C	1642.333802	0.190797265	0.029809877	6.400471348	1.54898E-10	4.42989E-10
CDHR2	72.81947944	-0.405287451	0.063319612	-6.400662288	1.54704E-10	4.425E-10
TRAF3	1948.906888	0.240618752	0.037591543	6.400874537	1.54489E-10	4.4195E-10
PRELID3B	1311.126275	0.238287666	0.03721841	6.402413834	1.52939E-10	4.3758E-10
PSMD11	3150.990995	0.19684975	0.030740254	6.403647457	1.51708E-10	4.34121E-10
OGFR	1748.613721	0.221183837	0.034539723	6.403752467	1.51604E-10	4.33885E-10
ARPP21	257.145811	-1.013839549	0.158312441	-6.404042197	1.51316E-10	4.33126E-10
LRRC41	2465.889551	-0.136132962	0.021253259	-6.405274709	1.50099E-10	4.29704E-10
SLC15A1	3.297186184	-0.543198532	0.084798716	-6.405740089	1.49642E-10	4.28458E-10
EPHB3	107.606236	-0.723355403	0.112909535	-6.406504119	1.48894E-10	4.2638E-10
B4GALT4	620.327748	0.21138129	0.032993935	6.406671009	1.48731E-10	4.25976E-10
PHF3	6476.908998	0.233812358	0.036494584	6.406768729	1.48636E-10	4.25765E-10
GJC1	32.52402075	-0.531787199	0.083003421	-6.406810608	1.48595E-10	4.2571E-10
EPS8L2	63.82299375	-0.305994461	0.0477599	-6.406932552	1.48476E-10	4.25433E-10
SPR	28.75104042	-0.56857807	0.088741726	-6.407110828	1.48303E-10	4.24998E-10

BATF3	22.70160392	0.489921069	0.076464476	6.407172279	1.48243E-10	4.24889E-10
ATP6V1H	1645.458896	0.172500013	0.02692221	6.407349547	1.48071E-10	4.24457E-10
C19orf54	518.4774472	-0.246391718	0.038448845	-6.408299548	1.47152E-10	4.21883E-10
AIP	1058.786713	-0.283110929	0.044167202	-6.40998114	1.45538E-10	4.17317E-10
MT-ATP8	5887.582624	-0.746254201	0.116398347	-6.411209636	1.4437E-10	4.14028E-10
VAT1L	35.3398557	-0.941733093	0.14688737	-6.41125982	1.44322E-10	4.13953E-10
TNNI3K	1.677075405	-0.682527871	0.106445166	-6.412013774	1.4361E-10	4.11971E-10
IL11RA	438.511774	-0.252114043	0.039315215	-6.412632956	1.43028E-10	4.1036E-10
RRH	18.48037326	-0.444428842	0.069294758	-6.413599768	1.42123E-10	4.07825E-10
C16orf92	1.663661342	-0.58500674	0.091211966	-6.413706078	1.42024E-10	4.076E-10
CC2D2A	280.1722732	-0.421274795	0.065683175	-6.413739814	1.41992E-10	4.0757E-10
SBK1	110.0269398	0.463995567	0.07233158	6.41484074	1.4097E-10	4.04695E-10
TMEM232	20.19266389	-0.425842098	0.066379702	-6.415245752	1.40596E-10	4.03679E-10
TMEM240	14.9968541	-0.483166277	0.075312217	-6.415509981	1.40352E-10	4.03039E-10
MT-CO3	392272.7949	-0.682338724	0.106352119	-6.415845104	1.40044E-10	4.02212E-10
ZNF140	338.5533396	-0.215504047	0.033582821	-6.417091948	1.38902E-10	3.98992E-10
OVOL3	5.809900633	-0.444080559	0.06919927	-6.417416798	1.38606E-10	3.982E-10
RFX1	743.0564464	-0.202700788	0.031584799	-6.417669005	1.38377E-10	3.97599E-10
PCSK4	133.2100525	-0.395142978	0.061567142	-6.418082233	1.38002E-10	3.9658E-10
CD33	1496.118454	-0.41190967	0.064165401	-6.419498069	1.36724E-10	3.92967E-10
SIAH3	26.39938182	0.799418041	0.124517028	6.420150333	1.3614E-10	3.91345E-10
TMPRSS4	16.83221213	-0.554506679	0.086369384	-6.420176408	1.36117E-10	3.91335E-10
CIDEC	4.12457554	-0.658955779	0.102613599	-6.421719785	1.34743E-10	3.87444E-10
UBR2	3740.214713	-0.248837314	0.038742272	-6.422889027	1.33712E-10	3.84535E-10
C3orf49	24.86118749	-0.292783272	0.045581867	-6.42324003	1.33404E-10	3.83705E-10
NXPE2	0.656588936	-1.025410267	0.159628954	-6.42371101	1.32992E-10	3.82576E-10
CARMIL2	1138.986611	-0.325466704	0.050664982	-6.423898558	1.32828E-10	3.8216E-10
MGMT	381.9695601	-0.36893956	0.057428808	-6.42429419	1.32483E-10	3.81224E-10
ITLN2	2.249125594	-0.816774018	0.127133918	-6.424516997	1.32289E-10	3.80722E-10
MXRA5	19.64287546	-0.828347812	0.128924535	-6.425059518	1.31818E-10	3.79422E-10
TASOR	2182.882276	-0.217980948	0.033925773	-6.425231533	1.31669E-10	3.79049E-10
INHA	5.053866686	-0.706896673	0.110018237	-6.425268167	1.31637E-10	3.79014E-10
EFNB1	250.0743222	0.48330419	0.075210088	6.426055348	1.30958E-10	3.77113E-10
LIPG	6.943165331	-0.591928614	0.092110313	-6.426301216	1.30746E-10	3.76559E-10
RIPK1	2041.594804	0.192948688	0.030020329	6.427267573	1.29918E-10	3.74229E-10
FAM228A	13.74031753	-0.442544543	0.06885367	-6.427319606	1.29874E-10	3.74156E-10
PDE7B	69.41297288	-0.440568461	0.068537884	-6.428101312	1.29208E-10	3.72292E-10

UBE3C	2293.25706	-0.189999332	0.029556997	-6.428235416	1.29094E-10	3.72019E-10
PAX1	1.162386839	-1.04071639	0.161877187	-6.429049137	1.28405E-10	3.70087E-10
SENP3	205.8238089	-0.149373184	0.023231791	-6.429688667	1.27866E-10	3.68588E-10
AMN	292.4329991	-0.452139355	0.07030802	-6.430836135	1.26904E-10	3.6587E-10
WNT11	60.96830783	0.593607548	0.092281587	6.432567614	1.25466E-10	3.61778E-10
RPEL1	2.224281754	-0.4825483	0.07501493	-6.432696753	1.2536E-10	3.61524E-10
CD79A	693.462391	0.501047855	0.077880973	6.433507902	1.24692E-10	3.59652E-10
IMMP1L	154.1002749	-0.26965976	0.041911433	-6.434038234	1.24258E-10	3.58452E-10
MIGA1	762.0112206	0.184231637	0.028632812	6.434283824	1.24057E-10	3.57925E-10
DTX4	843.0116872	0.48703286	0.075685988	6.434914498	1.23543E-10	3.56495E-10
EGFR	6.01224301	-0.711797363	0.110610212	-6.435186703	1.23322E-10	3.55909E-10
VWA3A	12.99507645	-0.371416881	0.057711336	-6.435769932	1.22849E-10	3.54597E-10
TIGD7	24.76215676	-0.297583556	0.046217334	-6.438786766	1.20432E-10	3.47673E-10
LMAN1	2547.3764	0.193959685	0.030114295	6.440784576	1.18857E-10	3.43177E-10
SLC14A2	22.78277899	-0.402547276	0.062491751	-6.44160657	1.18215E-10	3.41374E-10
POM121	4194.144394	0.212803121	0.033034428	6.441858868	1.18019E-10	3.40857E-10
PDIA6	2995.344778	0.242818712	0.037687105	6.443018479	1.1712E-10	3.38311E-10
FOXN4	4.382241753	-0.631800178	0.098026206	-6.445217094	1.15435E-10	3.33492E-10
IL17RB	94.71960755	-0.410486593	0.063683835	-6.445695301	1.15072E-10	3.32491E-10
KAZALD1	19.88976158	-0.519989234	0.080668556	-6.445996535	1.14843E-10	3.31881E-10
KCNJ6	18.79271943	-0.732560088	0.113638366	-6.446415187	1.14527E-10	3.31015E-10
ZNF473	258.262767	-0.261013638	0.040489336	-6.446478664	1.14479E-10	3.30925E-10
CLEC1B	158.7264581	-0.495321194	0.076822475	-6.447607878	1.13629E-10	3.28518E-10
RIMBP3B	3.340563349	-0.558203592	0.086561594	-6.448628851	1.12867E-10	3.26361E-10
CCN3	124.4369856	0.760150423	0.117860423	6.449581692	1.12159E-10	3.24364E-10
TM7SF3	2980.335748	-0.332628482	0.051573563	-6.449592827	1.12151E-10	3.24364E-10
APOB	6.708784324	-0.902582448	0.139934308	-6.450044041	1.11818E-10	3.23471E-10
TM4SF18	3.072655534	-0.893994437	0.138594715	-6.450422288	1.11539E-10	3.22713E-10
HDAC9	1196.029957	-0.46647167	0.072305491	-6.451400392	1.10821E-10	3.20684E-10
GYG2	54.46172092	-0.620494484	0.096159821	-6.45274165	1.09845E-10	3.17904E-10
TCF7L2	1112.85516	0.355594062	0.055104871	6.453042276	1.09627E-10	3.17321E-10
C22orf15	68.38454355	-0.339994117	0.052679801	-6.453974969	1.08954E-10	3.1542E-10
LYPD5	9.207726171	-0.501131873	0.0776439	-6.45423365	1.08768E-10	3.14928E-10
FIZ1	315.7858967	-0.194807773	0.030182214	-6.454389753	1.08656E-10	3.1465E-10
MOCS1	23.04026217	-0.830236541	0.128616228	-6.455146073	1.08115E-10	3.13129E-10
PAX2	2.622595241	-0.709804448	0.109954092	-6.455461872	1.07889E-10	3.12523E-10
C5	488.5118934	-0.322228469	0.049913143	-6.455784007	1.0766E-10	3.11905E-10

SPTB	3501.94111	-0.695968031	0.107783453	-6.457095306	1.06732E-10	3.09261E-10
EAF2	1008.507057	-0.317434416	0.04915925	-6.457267265	1.06611E-10	3.08956E-10
TJAP1	948.8343157	-0.152833707	0.023667444	-6.457550023	1.06412E-10	3.08425E-10
KDM4C	2677.030212	-0.204823904	0.031718269	-6.457600403	1.06376E-10	3.08368E-10
BAMBI	197.6637399	-0.695530558	0.107703418	-6.457831783	1.06214E-10	3.07943E-10
USP21	884.1355154	-0.154651966	0.023945907	-6.458388388	1.05824E-10	3.06858E-10
ABCG4	8.44503036	-0.606012649	0.093818695	-6.45940184	1.05118E-10	3.04855E-10
RBM17	2684.882981	0.184964401	0.028633881	6.459634389	1.04956E-10	3.04432E-10
FAM155A	2.300249975	-1.120254735	0.173387982	-6.460971048	1.04033E-10	3.018E-10
NMRK2	28.80972737	-0.626587261	0.096974552	-6.461357617	1.03768E-10	3.01074E-10
ATP5IF1	1092.932382	-0.407121232	0.063002547	-6.461980485	1.03341E-10	2.99882E-10
MYNN	630.7039259	0.22041787	0.03410282	6.463332707	1.02422E-10	2.97257E-10
PRR23C	1.105026566	-0.734737987	0.113651522	-6.464831922	1.01411E-10	2.94368E-10
SMIM24	1624.561558	-0.544085071	0.084144329	-6.46609314	1.00569E-10	2.91967E-10
MTRNR2L12	230.3130667	-1.104073857	0.170715767	-6.467322109	9.97549E-11	2.89646E-10
RGS3	944.9857235	-0.395950532	0.061218827	-6.467790269	9.94464E-11	2.88793E-10
ERF	1576.11438	0.282749771	0.043700726	6.470138981	9.79128E-11	2.84382E-10
RFPL2	13.63064269	-0.631224233	0.097556984	-6.470313126	9.78E-11	2.84096E-10
SGMS1	719.7816177	-0.343515871	0.053073594	-6.472444063	9.64302E-11	2.80159E-10
TMEM184B	1158.022102	0.244361344	0.037752842	6.472660896	9.62919E-11	2.79798E-10
ZER1	2069.12866	-0.258390101	0.039915965	-6.473352148	9.58522E-11	2.78562E-10
BRDT	2.901679668	-0.663318933	0.102462018	-6.473803129	9.55664E-11	2.77773E-10
LPAR4	84.22278914	-0.520862388	0.08045689	-6.473807106	9.55639E-11	2.77773E-10
UQCRC2	4547.184131	0.143317388	0.022136106	6.474372107	9.5207E-11	2.7681E-10
CNTN1	14.33768727	-1.057089503	0.163207442	-6.476968772	9.35836E-11	2.72131E-10
OR1K1	4.983838167	-0.521307697	0.080476279	-6.477780802	9.30814E-11	2.70711E-10
SLC6A12	51.27819205	0.598371186	0.092364857	6.478342566	9.27356E-11	2.69745E-10
CCDC65	165.6033706	-0.306022185	0.04722944	-6.479479454	9.20396E-11	2.6776E-10
NPAS4	2.360307613	-0.590828995	0.091180822	-6.4797507	9.18743E-11	2.67319E-10
TMEM183A	2515.83384	0.168231421	0.02595869	6.48073611	9.12762E-11	2.65618E-10
CCDC127	163.1342909	-0.22493994	0.034708372	-6.480855336	9.12041E-11	2.65448E-10
PCDH11Y	1.307395488	-0.757809239	0.116916851	-6.481608357	9.075E-11	2.64166E-10
ITGAD	30.48884114	-0.496664629	0.076610408	-6.482991584	8.99215E-11	2.61793E-10
TRIM13	1413.944452	0.195432084	0.030142491	6.483607599	8.9555E-11	2.60765E-10
GPAT3	357.398374	0.521874513	0.080490651	6.483666231	8.95201E-11	2.60702E-10
RNF145	1798.004564	0.290390355	0.044779766	6.484856516	8.88163E-11	2.58691E-10
ZBTB7A	3432.172995	0.249137742	0.038416882	6.485111008	8.86665E-11	2.58293E-10

NT5C	256.7344756	-0.351668279	0.054226258	-6.485202725	8.86126E-11	2.58174E-10
AGO3	2804.384751	-0.209465152	0.032295302	-6.485932549	8.81846E-11	2.56966E-10
NIBAN3	534.3234249	-0.527954565	0.08139652	-6.486205605	8.8025E-11	2.56539E-10
PRAM1	3729.99374	-0.381840417	0.058868894	-6.486284849	8.79788E-11	2.56442E-10
NAA80	527.2736428	-0.297336579	0.045839379	-6.486487924	8.78603E-11	2.56135E-10
PATE3	0.920510546	-1.02295306	0.157693934	-6.486952499	8.75899E-11	2.55385E-10
CFAP61	6.413153823	-0.617440548	0.095170341	-6.487741247	8.71328E-11	2.5409E-10
NID2	144.7328796	-0.42466443	0.065455474	-6.487836713	8.70776E-11	2.53967E-10
WFDC5	7.862111473	-0.891772165	0.137440654	-6.488416175	8.67434E-11	2.5303E-10
FBXW10	5.158459178	-0.567899018	0.087503609	-6.490006768	8.58325E-11	2.5041E-10
NIT2	1052.493624	-0.186551645	0.02874432	-6.490035057	8.58164E-11	2.504E-10
NPM3	429.0187138	-0.301284016	0.046417834	-6.490695297	8.54411E-11	2.49342E-10
CTRB2	1.311317675	-0.820347215	0.12637642	-6.4912997	8.5099E-11	2.48381E-10
CDH15	11.37892078	-0.577113476	0.088891395	-6.492343551	8.45113E-11	2.46702E-10
CBS	5.305877967	-0.53396578	0.08222416	-6.49402536	8.35727E-11	2.43999E-10
KBTBD7	222.8554066	-0.337112395	0.051909969	-6.494174498	8.34899E-11	2.43794E-10
CER1	1.344648068	-2.593860566	0.399334131	-6.495464242	8.27777E-11	2.4175E-10
NDOR1	880.2292823	-0.197670364	0.030427627	-6.496410815	8.22588E-11	2.4027E-10
B3GALT5	1.853558595	-0.740641788	0.114002551	-6.496712431	8.20942E-11	2.39825E-10
RIMBP3C	1.846097041	-0.56140795	0.086399462	-6.497817642	8.14935E-11	2.38106E-10
ACOT9	878.6397064	0.237110474	0.03649028	6.497907718	8.14447E-11	2.37999E-10
RPA2	872.4076692	-0.238928683	0.036768552	-6.498180458	8.12973E-11	2.37604E-10
CREB5	518.9823635	0.476161327	0.07326798	6.498900694	8.0909E-11	2.36504E-10
ZNF587	1316.300119	-0.19834873	0.030519341	-6.499115733	8.07935E-11	2.36202E-10
DSG3	1.971095834	-0.697389297	0.107293194	-6.499846552	8.0402E-11	2.35092E-10
LRRC32	79.80773183	0.624436242	0.096064042	6.500207901	8.02091E-11	2.34563E-10
LPCAT3	464.7948977	-0.195203688	0.030027689	-6.500789549	7.98995E-11	2.33693E-10
BBOF1	104.2675139	-0.367141842	0.056467124	-6.501868955	7.93282E-11	2.32057E-10
RELA	4746.682067	0.229702656	0.035328654	6.501879587	7.93226E-11	2.32057E-10
REEP6	84.31194288	-0.476557535	0.073289926	-6.502360733	7.90692E-11	2.31368E-10
PMP22	123.2507252	0.643932832	0.099024106	6.502788664	7.88445E-11	2.30745E-10
PDPN	6.944825873	1.057873067	0.162661064	6.503542021	7.84505E-11	2.29626E-10
TPH2	7.680704472	-0.739378228	0.113668034	-6.504715525	7.78405E-11	2.27875E-10
APOBEC3H	51.29987962	-0.416542983	0.064035744	-6.50485115	7.77703E-11	2.27704E-10
PPIAL4C	0.978761162	-0.677134613	0.104092123	-6.505147496	7.76172E-11	2.27289E-10
TXN2	1333.989061	-0.312610183	0.048051906	-6.505677058	7.73442E-11	2.26524E-10
SH2B2	346.2031089	0.38393657	0.059014578	6.505791988	7.72851E-11	2.26385E-10

LMOD1	12.44848848	-0.673596802	0.103529553	-6.506323894	7.70121E-11	2.25619E-10
CNNM2	1312.678003	-0.24590487	0.037794257	-6.506408362	7.69688E-11	2.25526E-10
MRPS7	559.2917441	-0.194221547	0.029849922	-6.506601523	7.687E-11	2.2527E-10
CSRNP3	6.304179393	-0.860245385	0.132173061	-6.508477421	7.59163E-11	2.22508E-10
LRRC8E	5.964615111	-0.552125445	0.084819422	-6.509422375	7.54403E-11	2.21146E-10
U2AF2	3869.320384	-0.173696281	0.026682909	-6.509645714	7.53283E-11	2.20851E-10
CDH7	1.99785861	-0.839185999	0.128882969	-6.511224944	7.45404E-11	2.18574E-10
TUT7	2935.229617	0.246248268	0.037817507	6.511488574	7.44097E-11	2.18223E-10
ZNF391	68.10901758	-0.476291844	0.073142288	-6.511853252	7.42292E-11	2.17726E-10
VSIG4	404.8653809	0.651223474	0.100004313	6.511953884	7.41795E-11	2.17613E-10
SETX	6842.388557	0.209591328	0.032177618	6.513574918	7.3383E-11	2.15309E-10
TTC34	72.01964867	-0.310646354	0.047687983	-6.514143237	7.31057E-11	2.14527E-10
HOOK3	2985.254342	0.201199149	0.030883377	6.514804045	7.27846E-11	2.13617E-10
ARPC5L	1878.256683	0.288474737	0.044271892	6.515979393	7.22169E-11	2.11983E-10
TMEM129	728.0397754	-0.208654397	0.032021779	-6.516015092	7.21997E-11	2.11964E-10
CCDC92	342.946529	-0.369011275	0.056629142	-6.516278762	7.2073E-11	2.11624E-10
TMEM70	535.7925777	0.297834588	0.045704854	6.516476034	7.19783E-11	2.11378E-10
IKZF3	977.9436629	0.460321195	0.070634268	6.516967053	7.17432E-11	2.10719E-10
IGFBP5	121.7691181	-0.832336408	0.127712676	-6.517257603	7.16044E-11	2.10343E-10
LRP4	159.4887717	0.659065064	0.101120641	6.517611608	7.14357E-11	2.09878E-10
SLIRP	558.852618	-0.369647164	0.056708784	-6.5183405	7.10895E-11	2.08893E-10
USP47	3151.929344	0.249530193	0.038280397	6.518485066	7.1021E-11	2.08723E-10
SOX6	635.2351567	-0.519862421	0.079746857	-6.518907969	7.08211E-11	2.08167E-10
SELENOH	348.2911859	-0.200232795	0.030714286	-6.519207181	7.068E-11	2.07783E-10
NELL1	1.480168589	-1.325714417	0.203328777	-6.520053076	7.02825E-11	2.06646E-10
EDEM3	1701.147028	-0.293774218	0.045055865	-6.520221493	7.02036E-11	2.06445E-10
TESMIN	758.6208436	-0.286930848	0.044000671	-6.521056179	6.9814E-11	2.0533E-10
HPN	11.83268241	-0.63083862	0.096736931	-6.521176673	6.9758E-11	2.05196E-10
LAT2	4151.734894	-0.397978025	0.061018232	-6.522280465	6.92464E-11	2.03722E-10
CSN1S1	8.9857181	-0.920981036	0.141198012	-6.522620423	6.90896E-11	2.03291E-10
SEMA6A	218.8500943	-0.370289124	0.056768917	-6.522744156	6.90326E-11	2.03154E-10
NKAPD1	905.2452085	0.180607479	0.027688594	6.522811419	6.90016E-11	2.03093E-10
VEGFA	2627.336977	0.460497311	0.070594954	6.523091041	6.8873E-11	2.02745E-10
FAM83E	29.2057587	-0.462628374	0.07091529	-6.523675939	6.86049E-11	2.01986E-10
CEBPD	2296.154926	0.594770467	0.091166989	6.523967428	6.84716E-11	2.01624E-10
NDST3	86.51786249	-0.896078157	0.137342647	-6.524398494	6.8275E-11	2.01075E-10
ABCG2	99.08245813	-0.635723957	0.097406793	-6.526484854	6.73311E-11	1.98325E-10

PTX3	928.5417325	0.532000198	0.081512659	6.52659601	6.72812E-11	1.98208E-10
OR11L1	1.23643184	-0.927965683	0.14217713	-6.526828075	6.71771E-11	1.97931E-10
CTSE	185.1939369	-0.734481382	0.112529651	-6.527003106	6.70987E-11	1.9773E-10
FAM243B	0.696496752	1.110161437	0.170077998	6.527366567	6.69361E-11	1.97281E-10
TBPL2	2.305709259	-0.593991501	0.090992631	-6.527907729	6.66948E-11	1.96599E-10
SEZ6L2	32.32287809	-0.577480601	0.088441027	-6.529555593	6.59651E-11	1.94477E-10
DOCK8	10503.66033	-0.259059355	0.039669812	-6.530390255	6.55985E-11	1.93426E-10
FOXJ2	1579.329622	0.185092693	0.028342247	6.530628744	6.54942E-11	1.93147E-10
KIR2DL3	4.354504568	0.630746848	0.096569777	6.531513954	6.51082E-11	1.92038E-10
PROKR2	9.340251491	-0.672293519	0.102930372	-6.531536879	6.50982E-11	1.92037E-10
FSD1	18.59751549	-0.501555212	0.07678545	-6.531904319	6.49386E-11	1.91595E-10
PRDM16	440.1167091	-1.015443372	0.155420716	-6.533513668	6.42443E-11	1.89575E-10
LRRC74A	5.245631665	-0.766558421	0.117318158	-6.534013428	6.40302E-11	1.88972E-10
UBN2	2240.682196	-0.249131643	0.038127309	-6.534204745	6.39484E-11	1.88759E-10
SHPRH	1416.431472	-0.274138998	0.041953625	-6.534333966	6.38932E-11	1.88625E-10
THBS2	8.894101612	-0.694047614	0.106150907	-6.538310712	6.22176E-11	1.83706E-10
RLN1	3.40348415	-0.55533388	0.084934818	-6.538353672	6.21997E-11	1.83681E-10
ANKRD40CL	38.96919931	-0.59743518	0.091334019	-6.54121196	6.10223E-11	1.80231E-10
INTS4	1297.46114	-0.178864568	0.027344007	-6.54127123	6.09981E-11	1.80186E-10
CHMP1A	2148.302282	0.161795114	0.024733729	6.541476671	6.09143E-11	1.79966E-10
PCSK2	3.729271016	-0.899227042	0.137463033	-6.541591735	6.08675E-11	1.79855E-10
CCL14	1.185665897	-0.948958136	0.145044171	-6.542545824	6.04803E-11	1.78738E-10
B4GALT2	332.0838559	-0.312349686	0.047741116	-6.542571958	6.04697E-11	1.78734E-10
ATXN1	2210.308837	0.417432584	0.063799622	6.542869228	6.03496E-11	1.78405E-10
IRF2BPL	1106.378419	0.271875901	0.041542371	6.544544618	5.96769E-11	1.76444E-10
GRM4	7.062917056	-0.465397706	0.071103386	-6.545366317	5.93497E-11	1.75503E-10
GJB2	8.330858217	0.893836792	0.136542694	6.546207366	5.90166E-11	1.74544E-10
XXYLT1	471.5076253	-0.29310494	0.04476866	-6.547100961	5.86647E-11	1.73529E-10
EPX	6305.003622	0.846139628	0.129237079	6.547189367	5.863E-11	1.73453E-10
SQSTM1	8311.591509	0.297819416	0.045484531	6.547707724	5.84269E-11	1.72878E-10
BARX2	2.549600607	-0.715090496	0.10921147	-6.547760022	5.84065E-11	1.72844E-10
TEX33	0.978532116	-0.863903511	0.131934948	-6.547950511	5.8332E-11	1.7265E-10
MROH7-TTC4	3.1502368	-0.506265923	0.077306259	-6.548834868	5.79877E-11	1.71657E-10
PPIP5K2	3740.283376	-0.220307675	0.033639254	-6.549124863	5.78752E-11	1.7135E-10
PLA2G1B	7.51810643	-0.619933754	0.094654739	-6.54942121	5.77605E-11	1.71036E-10
ATCAY	37.21894783	-0.620073653	0.094671996	-6.549705115	5.76508E-11	1.70737E-10
ZNF214	2.802345852	-1.056036036	0.161186005	-6.551660815	5.69007E-11	1.68541E-10

MRPL35	270.4355584	-0.226119026	0.034508755	-6.552511842	5.65772E-11	1.67608E-10
ARHGAP9	2496.813139	-0.22686093	0.034619088	-6.55305909	5.63702E-11	1.6702E-10
PPARGC1B	725.3960861	-0.373593842	0.057008435	-6.553308187	5.62762E-11	1.66767E-10
BX255925.3	486.7732439	-0.199209567	0.030398008	-6.553375794	5.62507E-11	1.66716E-10
TCTEX1D2	16.88689937	-0.376177917	0.05739889	-6.553749008	5.61102E-11	1.66325E-10
LHX1	3.521833092	-0.789806704	0.120485117	-6.555222114	5.55591E-11	1.64717E-10
SNRNP48	585.7516085	0.167725344	0.025586505	6.555226713	5.55574E-11	1.64717E-10
PPARD	647.7348652	0.393208964	0.059983034	6.555336406	5.55166E-11	1.6464E-10
SCG5	41.08280705	-0.540355511	0.082410738	-6.55685806	5.49533E-11	1.62994E-10
PLOD2	58.90055307	-0.927633106	0.14146899	-6.557148001	5.48466E-11	1.62703E-10
UTS2R	7.061473215	-0.559865483	0.085381814	-6.557198302	5.48281E-11	1.62672E-10
SPINT2	511.5516972	-0.385815938	0.058836332	-6.557443766	5.47379E-11	1.6243E-10
PILRA	869.446467	0.381014257	0.058103507	6.557508753	5.47141E-11	1.62383E-10
C4orf46	592.1275346	0.229023614	0.03492211	6.558126406	5.4488E-11	1.61737E-10
TRMT10A	73.01118017	-0.245419685	0.037420832	-6.558370595	5.43989E-11	1.61497E-10
OR51B6	1.766943518	-0.808462277	0.12327102	-6.558413115	5.43834E-11	1.61475E-10
PUS7L	642.0377139	-0.257476407	0.039258899	-6.558421465	5.43803E-11	1.61475E-10
INPP4A	1894.066396	-0.326425401	0.049756915	-6.560402764	5.36626E-11	1.59384E-10
OR1J4	1.67802052	-1.084385734	0.165288458	-6.560565353	5.36042E-11	1.59234E-10
RNASE1	107.4413455	-0.734837104	0.112007473	-6.560607849	5.35889E-11	1.59213E-10
PFKL	5174.802281	-0.204421366	0.031157874	-6.560825211	5.35108E-11	1.59005E-10
APOE	246.1040135	-0.452954706	0.069035501	-6.561185153	5.33818E-11	1.58646E-10
TNS1	4207.493544	-0.569532055	0.086789155	-6.562249096	5.30022E-11	1.57542E-10
CCDC33	3.163767686	-0.745522325	0.113603822	-6.562475729	5.29217E-11	1.57326E-10
ATP8B4	9306.535515	-0.451202522	0.068751065	-6.562844118	5.2791E-11	1.56962E-10
COASY	1428.460856	-0.179541177	0.027355693	-6.563210788	5.26613E-11	1.566E-10
SERF2	3704.45569	-0.393344454	0.05993118	-6.563269024	5.26407E-11	1.56562E-10
POLR3C	1229.544392	-0.11950578	0.0182078	-6.563438861	5.25808E-11	1.56408E-10
ADGB	11.12369168	-0.518298158	0.078949589	-6.56492532	5.20589E-11	1.54879E-10
SP6	17.72355318	-0.485778419	0.073992602	-6.565229582	5.19528E-11	1.54587E-10
TMEM268	605.2735479	0.264537156	0.040282167	6.567103474	5.13034E-11	1.52678E-10
MTMR8	97.32333318	-0.427733234	0.065125096	-6.567871072	5.10397E-11	1.51916E-10
WNT5B	29.76931438	-0.5832284	0.088790605	-6.568582346	5.07965E-11	1.51215E-10
NCKAP1	127.4944064	-0.68736626	0.104630235	-6.56948023	5.04912E-11	1.50329E-10
ALDH7A1	161.6735818	-0.489424951	0.074496612	-6.569761211	5.0396E-11	1.50069E-10
ARSL	11.60486977	-0.64325161	0.097893915	-6.570904924	5.00104E-11	1.48943E-10
DNAI4	63.25447519	-0.346893754	0.052789721	-6.571236738	4.9899E-11	1.48634E-10

TIE1	738.626958	-0.49023642	0.074599344	-6.57159156	4.97803E-11	1.48303E-10
OGFOD2	62.20358181	-0.3083598	0.0469206	-6.571949221	4.96608E-11	1.47969E-10
NEDD8	503.3066029	-0.254576881	0.038729236	-6.573248181	4.92293E-11	1.46706E-10
PCDHB8	3.942564708	-1.388094468	0.211161004	-6.57363075	4.91029E-11	1.46352E-10
DHRS3	1112.452321	0.487490027	0.074155583	6.573881677	4.90202E-11	1.46127E-10
SENP2	2181.363364	0.185601894	0.028230188	6.574589372	4.87876E-11	1.45456E-10
NDUFA5	1243.268279	0.229127548	0.034850434	6.574596773	4.87852E-11	1.45456E-10
LRRC6	99.92375887	-0.333111985	0.05065679	-6.575860478	4.83726E-11	1.44263E-10
COA6	131.9705445	-0.339596879	0.051642337	-6.575939447	4.83469E-11	1.44208E-10
ACR	18.73405769	-0.674474233	0.10256496	-6.576068793	4.83049E-11	1.44105E-10
ERC1	1937.981699	-0.179174089	0.027241892	-6.577153021	4.79541E-11	1.4308E-10
RANBP6	2206.203979	0.216348732	0.032891317	6.577685202	4.77829E-11	1.42591E-10
ERN1	1532.132398	0.235316735	0.035771089	6.578405644	4.75519E-11	1.41923E-10
GDF10	7.473583166	-0.890109679	0.135305786	-6.57850416	4.75205E-11	1.41851E-10
NOP9	998.6882406	-0.240978843	0.036630787	-6.578587717	4.74938E-11	1.41793E-10
DDX4	3.228598737	-0.770446179	0.11709299	-6.579780543	4.71143E-11	1.40682E-10
RASSF9	2.229135171	-0.996252741	0.151398202	-6.580347256	4.69351E-11	1.40168E-10
PER1	13213.48424	0.350975476	0.053327984	6.581450345	4.65881E-11	1.39153E-10
KRBOX1	0.882542005	-0.916326222	0.139226727	-6.58153961	4.65601E-11	1.39091E-10
ALG12	413.5885711	-0.173696585	0.026390643	-6.581748785	4.64947E-11	1.38916E-10
TCEANC	168.7921834	-0.316056005	0.048019523	-6.581823131	4.64714E-11	1.38868E-10
CCDC148	6.488948188	-0.493864656	0.075034344	-6.581848108	4.64636E-11	1.38866E-10
CNN2	5153.191419	0.255306958	0.03878918	6.581911638	4.64438E-11	1.38828E-10
AC007040.2	4.820337938	-0.588785639	0.089450105	-6.582280046	4.63288E-11	1.38505E-10
PDCD5	551.1377939	-0.341806739	0.051922354	-6.583036255	4.60937E-11	1.37823E-10
RNF182	175.6836981	-0.905489903	0.137537298	-6.58359526	4.59206E-11	1.37327E-10
MT-ND1	238142.7822	-0.7399892	0.112394672	-6.58384592	4.58432E-11	1.37116E-10
SCLT1	743.8553825	-0.207065623	0.031440167	-6.58602171	4.51768E-11	1.35144E-10
NPRL3	2017.249121	-0.281063811	0.042675056	-6.586138015	4.51414E-11	1.35059E-10
MYBPHL	14.190329	-0.739235258	0.11222577	-6.587036629	4.48692E-11	1.34265E-10
FKBP1B	23.3372249	-0.447969989	0.068006277	-6.587185896	4.48241E-11	1.3415E-10
TPCN1	1385.863245	-0.300784478	0.045660522	-6.587407774	4.47572E-11	1.3397E-10
GPR87	16.02985705	-0.882109376	0.133898812	-6.58788055	4.46149E-11	1.33565E-10
ATP5MD	900.5791033	-0.450971642	0.068452693	-6.588077449	4.45558E-11	1.33408E-10
POLR3B	247.2600059	-0.274793476	0.041696715	-6.590290764	4.38966E-11	1.31455E-10
CPSF7	3608.510499	0.149774755	0.022723617	6.591149337	4.36435E-11	1.30717E-10
DNASE1L3	23.31562552	-0.674188963	0.10228497	-6.591280807	4.36048E-11	1.30621E-10

ATP1A1	3062.364821	0.187906973	0.028508263	6.591315998	4.35945E-11	1.3061E-10
ST20	51.7570055	-0.349521919	0.053024517	-6.591703951	4.34807E-11	1.30289E-10
FAT3	19.72907481	-0.816099236	0.123784333	-6.592912161	4.31282E-11	1.29252E-10
ATP12A	6.893734525	-0.678679142	0.10292687	-6.593799499	4.28711E-11	1.28502E-10
CNBP	6119.563615	0.219801012	0.033334106	6.593877547	4.28485E-11	1.28454E-10
TIMP2	2108.29074	0.307221202	0.046585807	6.594738193	4.26007E-11	1.2773E-10
RAMAC	99.2296948	0.331108081	0.050206875	6.594875273	4.25614E-11	1.27632E-10
CSNK1A1L	15.76560459	-0.695447694	0.105445083	-6.595354403	4.24241E-11	1.2724E-10
TMIE	13.57729083	-0.591197555	0.089623494	-6.596457378	4.21098E-11	1.26317E-10
CENPC	3346.736989	0.245830448	0.037266984	6.596467513	4.2107E-11	1.26317E-10
NTN4	3.983702909	-0.694141738	0.105226221	-6.596661301	4.2052E-11	1.26182E-10
ARAP2	2591.003424	0.385223615	0.05839663	6.596675489	4.2048E-11	1.26182E-10
KATNIP	1133.454695	-0.203329636	0.030822058	-6.596887047	4.1988E-11	1.26029E-10
MAGEE1	32.07022881	0.514048628	0.077911576	6.597846585	4.17173E-11	1.25235E-10
SH2D5	42.61140633	-0.662174134	0.100358495	-6.598087523	4.16495E-11	1.25051E-10
GNPAT	1046.328142	-0.174722449	0.026480001	-6.598279487	4.15957E-11	1.24908E-10
PTGES3L	43.89364575	-0.342544659	0.051912255	-6.598531611	4.1525E-11	1.24715E-10
PPP3CC	694.443482	0.209496631	0.031747875	6.598760691	4.14609E-11	1.24542E-10
PLEKHM1	2769.525398	0.223092373	0.033800275	6.600312271	4.10293E-11	1.23264E-10
PIGP	244.359207	-0.335922848	0.050890412	-6.600906463	4.08651E-11	1.2279E-10
XG	22.07148634	-0.565184832	0.085594591	-6.603043756	4.02801E-11	1.2105E-10
CCL3	171.7070143	0.672595164	0.101824061	6.605463928	3.96274E-11	1.19107E-10
GFRA1	9.995433658	-0.961148952	0.145499741	-6.605846494	3.95252E-11	1.18819E-10
SLC49A4	756.8360355	0.312703068	0.047336462	6.605966252	3.94933E-11	1.18741E-10
PITPNB	1618.941626	0.170400781	0.025792008	6.606727875	3.92907E-11	1.1815E-10
VPS28	2319.285727	-0.236141249	0.035736222	-6.607896201	3.8982E-11	1.17239E-10
BABAM2	1728.074739	-0.460727537	0.069722722	-6.607997012	3.89555E-11	1.17178E-10
SH3BGR	2.125791975	-0.527414698	0.079804647	-6.608821857	3.87391E-11	1.16545E-10
MAT2A	7547.135383	-0.294221599	0.04451474	-6.60953204	3.85537E-11	1.16005E-10
NLRP2B	1.606354187	-0.72341029	0.109448151	-6.60961635	3.85317E-11	1.15957E-10
CD80	66.41915226	0.488046272	0.073838466	6.60964802	3.85235E-11	1.1595E-10
C11orf71	130.7949375	-0.270493758	0.040919788	-6.610341159	3.83435E-11	1.15426E-10
CYP1B1	131.4078604	0.74434963	0.112603152	6.61038004	3.83335E-11	1.15413E-10
BCL2	2608.817867	-0.30397436	0.045974375	-6.611821405	3.7962E-11	1.14312E-10
LRRC18	54.94544046	-0.570714231	0.086316964	-6.611843218	3.79564E-11	1.14312E-10
FAM98C	332.8807976	-0.224095891	0.033892435	-6.611973709	3.79229E-11	1.1423E-10
SERPINB8	1689.214821	0.422987345	0.063969131	6.612366593	3.78224E-11	1.13945E-10

CRYBG1	3067.009017	0.32681849	0.049422237	6.612782183	3.77163E-11	1.13642E-10
HMGN5	85.72156263	-0.557488159	0.084294318	-6.613591229	3.75107E-11	1.1304E-10
ZNF300	70.2786393	-0.501003389	0.075750175	-6.613890826	3.74348E-11	1.12829E-10
SPDYC	8.20526113	-0.684747138	0.103524464	-6.614350927	3.73186E-11	1.12496E-10
RRM2B	755.0550217	0.222872039	0.033686387	6.616086219	3.68833E-11	1.11201E-10
PDGFRL	2.554843033	-0.943925033	0.142667059	-6.616278742	3.68354E-11	1.11074E-10
LRRC69	58.66955511	-0.363135074	0.05487905	-6.617007245	3.66544E-11	1.10545E-10
CBX2	612.4070889	-0.504773689	0.076278313	-6.617525606	3.65261E-11	1.10175E-10
DMBX1	3.560179662	-0.697481636	0.105394672	-6.617807331	3.64566E-11	1.09982E-10
AQP6	9.279733185	-0.598251286	0.090378764	-6.61937892	3.60711E-11	1.08836E-10
SHROOM4	125.0585132	-0.820231396	0.123903239	-6.619935084	3.59357E-11	1.08444E-10
FAM122C	430.9710941	-0.209630662	0.031656772	-6.621984804	3.54407E-11	1.06967E-10
TICAM1	1264.687078	0.397544138	0.060025715	6.622897125	3.52226E-11	1.06325E-10
SCN11A	52.88705098	-0.396365847	0.059845267	-6.623177862	3.51558E-11	1.0614E-10
GPBP1L1	5363.654871	0.134328216	0.020281311	6.623251121	3.51383E-11	1.06103E-10
COX6B1	2749.608724	-0.453685203	0.068496746	-6.623456267	3.50896E-11	1.05973E-10
HIPK3	6970.558349	0.199756198	0.030158034	6.62364783	3.50441E-11	1.05852E-10
MUC22	1.204576474	-1.030152235	0.15552559	-6.623683173	3.50357E-11	1.05843E-10
KCNC1	38.13978452	-0.439643525	0.066357466	-6.625381479	3.46353E-11	1.04649E-10
GRIN2D	94.87018601	-0.629447735	0.095000034	-6.625763287	3.45458E-11	1.04395E-10
FZD9	9.545712818	-0.58896711	0.08888965	-6.625823223	3.45318E-11	1.04369E-10
PTX4	136.8147793	-0.801845616	0.121005688	-6.62651178	3.43712E-11	1.03899E-10
TALDO1	6334.627861	-0.288766196	0.043575214	-6.626845207	3.42937E-11	1.03681E-10
ZNF483	115.7510836	0.49968373	0.075397757	6.627302346	3.41877E-11	1.03376E-10
C4A	28.08385385	-0.361598024	0.054561244	-6.627378621	3.417E-11	1.03339E-10
RAB24	109.4950874	-0.286795728	0.043274325	-6.627387648	3.4168E-11	1.03339E-10
NOTCH3	245.8102073	0.690717359	0.104219242	6.627541548	3.41324E-11	1.03257E-10
RALGPS1	211.6105993	-0.32004535	0.048289654	-6.627617399	3.41148E-11	1.0322E-10
DNA2	481.6080338	-0.247760904	0.037378114	-6.628501964	3.39111E-11	1.02619E-10
EXOC2	874.7901091	-0.201329413	0.030365493	-6.630203928	3.35223E-11	1.01459E-10
EIF3CL	71.52138757	0.426293847	0.064287507	6.631052693	3.33301E-11	1.00892E-10
TRAPPC2L	545.1980286	-0.305803191	0.046111473	-6.631824453	3.31563E-11	1.00382E-10
MAPKAP1	1437.830252	-0.211197407	0.031841282	-6.632817336	3.29339E-11	9.9724E-11
OLFML2A	327.5222612	-0.730623197	0.110151638	-6.632885448	3.29187E-11	9.96934E-11
DRAXIN	36.61436711	-0.4094023	0.061712871	-6.633985601	3.26742E-11	9.89681E-11
SPOUT1	10.28426593	-0.301947579	0.04550337	-6.635719029	3.22924E-11	9.7827E-11
FRMD4B	746.980374	-0.38449131	0.057938229	-6.636228195	3.21812E-11	9.75049E-11

CYP21A2	6.429589191	-0.605075639	0.091166169	-6.637063335	3.19994E-11	9.69693E-11
TMEM8B	208.7779	-0.409939029	0.061749004	-6.638795811	3.16256E-11	9.58514E-11
SPDYE4	7.162264341	-0.493031104	0.074242769	-6.640796305	3.11993E-11	9.45739E-11
SCD5	107.7096898	-0.526731948	0.079284702	-6.643550807	3.06215E-11	9.28368E-11
AP1S1	185.7237592	-0.396963357	0.059749344	-6.643811109	3.05674E-11	9.26873E-11
FHDC1	793.9606931	-0.699174908	0.105231122	-6.644183756	3.04902E-11	9.24674E-11
PCDH7	8.069224557	-1.363575695	0.205206569	-6.64489299	3.03437E-11	9.20375E-11
ANO1	4.266991575	-0.870591376	0.131014378	-6.645006355	3.03204E-11	9.1981E-11
POLR2L	1034.502924	-0.522560247	0.07861758	-6.64686253	2.99406E-11	9.0843E-11
SYT16	5.755734364	-0.767321453	0.115432313	-6.647371379	2.98374E-11	9.05436E-11
SLC38A2	10195.81522	0.251380662	0.037813538	6.647901125	2.97302E-11	9.02324E-11
AL121753.1	30.18648208	-0.26799377	0.040310547	-6.648229561	2.96639E-11	9.00453E-11
IMP4	1130.903083	-0.230643546	0.034689674	-6.648766591	2.95559E-11	8.97314E-11
OPN5	1.588053762	-1.366874333	0.2055515	-6.649790127	2.93511E-11	8.91234E-11
RSBN1L	1162.455797	-0.209910411	0.031561826	-6.650768897	2.91566E-11	8.85464E-11
AGPAT4	951.9972866	-0.380330172	0.057179422	-6.651521857	2.90078E-11	8.81082E-11
MRPL42	1554.986964	-0.188381897	0.028316609	-6.652699597	2.87765E-11	8.74193E-11
DCT	4.159273429	-0.655685495	0.098557446	-6.652825575	2.87519E-11	8.73581E-11
SETD2	9251.167377	0.209060717	0.031419518	6.653848625	2.85526E-11	8.67661E-11
TTC36	18.56868507	-0.505417834	0.075954689	-6.654201829	2.84842E-11	8.65715E-11
PGAP2	848.8292391	-0.180196653	0.027075711	-6.655287858	2.82746E-11	8.5948E-11
DMRTA1	2.924544795	-1.269679916	0.190774924	-6.655381587	2.82566E-11	8.59066E-11
OR1L1	0.86223071	-0.931962823	0.140030067	-6.655447944	2.82439E-11	8.58811E-11
MAPK4	1.521136603	-1.115724462	0.167638933	-6.655521124	2.82298E-11	8.58518E-11
B4GALT6	614.3707951	-0.435318856	0.06540616	-6.655624692	2.82099E-11	8.58046E-11
SGPP1	334.6071299	0.533436113	0.080147634	6.655668904	2.82014E-11	8.57922E-11
TPGS1	237.5223721	-0.23213266	0.03487675	-6.655799666	2.81764E-11	8.57293E-11
C2orf42	231.7409872	-0.18009189	0.027050211	-6.657688894	2.78167E-11	8.4648E-11
ZWINT	476.8576088	-0.356628576	0.053565727	-6.657775277	2.78003E-11	8.46114E-11
FAM237B	1.268854562	-0.906500362	0.136138948	-6.65864084	2.76371E-11	8.41278E-11
NAPRT	793.6536215	-0.270280011	0.04059049	-6.658702791	2.76255E-11	8.41054E-11
ABT1	869.8659253	0.189124986	0.0283989	6.659588319	2.74596E-11	8.36133E-11
NBEA	161.4267007	-0.571023964	0.085740007	-6.659947759	2.73925E-11	8.3422E-11
GABRR3	1.230175898	-1.062538909	0.159535337	-6.660210412	2.73436E-11	8.3286E-11
FECH	3380.239558	-0.505034854	0.075820164	-6.660956976	2.7205E-11	8.28769E-11
ZNF790	158.7100262	-0.280707966	0.042128371	-6.663157346	2.68006E-11	8.16577E-11
WDR97	247.4126214	-0.357763403	0.053690077	-6.663492098	2.67396E-11	8.14845E-11

SLC2A9	432.9755022	-0.466875553	0.070059398	-6.663996064	2.66481E-11	8.12181E-11
RABGAP1L	2569.534463	-0.246475046	0.036985079	-6.664175253	2.66156E-11	8.11317E-11
PCDHGA11	65.68546094	-0.565424571	0.084831931	-6.665232788	2.64246E-11	8.05622E-11
CUL5	1389.063643	0.177595778	0.026640231	6.666450376	2.62064E-11	7.99094E-11
ARID3C	3.160135783	-0.497914435	0.07468664	-6.666713589	2.61595E-11	7.97787E-11
ZNF471	76.50528953	-0.516223055	0.077432245	-6.66677115	2.61492E-11	7.97599E-11
PFDN6	314.9717718	-0.256009694	0.038396682	-6.66749517	2.60206E-11	7.93799E-11
MSANTD4	248.0679033	-0.257930308	0.03868116	-6.668112062	2.59115E-11	7.90593E-11
CD40	235.1300347	0.44157741	0.066217	6.668641093	2.58183E-11	7.87872E-11
CAPN9	1.699862292	-0.957701591	0.143611131	-6.668714196	2.58054E-11	7.87603E-11
POP7	217.0044078	-0.355303855	0.053269254	-6.66996116	2.55871E-11	7.81061E-11
PGP	553.9818518	-0.305948023	0.045867469	-6.670261663	2.55348E-11	7.79585E-11
CRHBP	129.6344928	-0.6060185	0.09085319	-6.670305118	2.55272E-11	7.79476E-11
CCDC179	0.886598374	-0.835757291	0.125275327	-6.671363868	2.53437E-11	7.73993E-11
ALPG	1.383707932	-0.969102113	0.145261442	-6.671433938	2.53316E-11	7.73744E-11
SSNA1	777.2454775	-0.300478129	0.045028973	-6.672995324	2.50634E-11	7.65673E-11
F11R	1472.972802	0.32506943	0.048708032	6.673836205	2.49202E-11	7.61415E-11
KIR2DL1	5.535669283	0.708780597	0.106197127	6.674197491	2.48589E-11	7.59661E-11
ARHGDIG	6.066200212	-0.554451949	0.083071719	-6.674376752	2.48285E-11	7.58851E-11
CCNT2	3811.498676	0.225917305	0.033847889	6.674487293	2.48098E-11	7.58398E-11
DLC1	1233.571017	-0.628669451	0.094188407	-6.674594819	2.47916E-11	7.5796E-11
ITGA10	50.6642076	-0.433633428	0.064963017	-6.67508145	2.47095E-11	7.55568E-11
CBX8	93.68632976	-0.330844612	0.049560336	-6.675592525	2.46235E-11	7.53057E-11
ADA	1695.97149	-0.366769332	0.054938652	-6.675979823	2.45586E-11	7.51188E-11
SOX10	20.71407266	-0.4497234	0.067358671	-6.676547996	2.44636E-11	7.48399E-11
TMEM106A	1082.407751	-0.245130483	0.0367151	-6.676557624	2.4462E-11	7.48399E-11
CDKN2A	230.4982544	-0.58440197	0.087523002	-6.677124409	2.43676E-11	7.45696E-11
ARSF	2.697125045	-0.796286541	0.119240589	-6.677982303	2.42254E-11	7.41461E-11
FAM117A	1574.357533	-0.334871507	0.050140115	-6.678714343	2.41047E-11	7.37883E-11
PLA2G4D	3.695129824	-0.721055776	0.107963085	-6.678725191	2.4103E-11	7.37883E-11
GON7	102.6463029	-0.35651388	0.053378921	-6.678926319	2.40699E-11	7.37047E-11
TMC8	4496.949309	-0.245392174	0.03673925	-6.679291804	2.401E-11	7.35326E-11
TNRC6B	6683.006794	-0.26989556	0.040405183	-6.679726186	2.39389E-11	7.33265E-11
ZNF19	56.72535073	-0.344364067	0.05155085	-6.680085181	2.38803E-11	7.31585E-11
ASS1	105.3862898	0.907163796	0.135771707	6.681537815	2.36448E-11	7.24482E-11
PPP1R3C	3.380243421	-0.93171303	0.13938929	-6.684251233	2.32108E-11	7.11297E-11
CANX	15950.76164	0.200266748	0.029960835	6.684284533	2.32056E-11	7.11247E-11

BRINP3	1.50524079	-2.465402915	0.368831268	-6.684365259	2.31928E-11	7.10966E-11
TMEM132B	2.850278369	-0.769130747	0.115050628	-6.685150369	2.30688E-11	7.07276E-11
KPNA4	4553.940108	0.177005018	0.026467892	6.687537303	2.26957E-11	6.95948E-11
FFAR1	72.23061148	0.588215136	0.08793889	6.688907883	2.24842E-11	6.8957E-11
ATG13	4129.299562	0.175432641	0.026221689	6.690363785	2.22616E-11	6.82851E-11
PLGLB2	12.45550789	-0.545457092	0.081527659	-6.690454516	2.22479E-11	6.82535E-11
GPR150	34.83373065	-0.572617401	0.08557578	-6.69134889	2.21123E-11	6.78482E-11
EXOC6B	314.3714346	-0.412337338	0.061621208	-6.691484197	2.20918E-11	6.77961E-11
CEP85L	855.8959318	0.365920774	0.054679482	6.692103885	2.19985E-11	6.75201E-11
PSMD12	3173.874136	0.240318292	0.035907021	6.692793876	2.18949E-11	6.72129E-11
CACNG2	3.151461685	-0.634062266	0.094737638	-6.692823279	2.18905E-11	6.721E-11
PIP	0.986857795	-0.71212831	0.10640002	-6.692934008	2.1874E-11	6.71697E-11
GPC1	182.3285942	-0.466592519	0.069703055	-6.694003856	2.17146E-11	6.66906E-11
FBRS	11220.95449	0.289551272	0.043247654	6.695190301	2.15391E-11	6.61621E-11
KLF10	8321.818559	0.361084758	0.053931049	6.695303831	2.15224E-11	6.61212E-11
HPS4	3127.683317	-0.196227401	0.029299636	-6.697264084	2.12358E-11	6.52508E-11
CD68	15.75488018	0.559315143	0.083495124	6.698776138	2.10172E-11	6.45894E-11
KCTD7	436.5299816	0.434227943	0.064819052	6.699078914	2.09737E-11	6.44659E-11
TNFRSF10B	2304.395177	0.299639502	0.044727365	6.699243297	2.09502E-11	6.44035E-11
ADAM29	1.07704783	-1.025545662	0.153083386	-6.699261666	2.09475E-11	6.44035E-11
ACER3	931.8614721	0.206363254	0.030802812	6.699494053	2.09142E-11	6.43133E-11
ENGASE	2104.618966	-0.299221516	0.044661192	-6.699810337	2.0869E-11	6.41844E-11
PIMREG	44.05781653	-0.609885289	0.091021246	-6.700471781	2.07748E-11	6.39045E-11
OR9A2	0.795236583	-0.797661745	0.119009005	-6.702532662	2.04838E-11	6.30193E-11
GID4	463.0982289	-0.218476558	0.032593183	-6.703136513	2.03993E-11	6.27692E-11
PIWIL1	1.271607291	-1.064815281	0.158850922	-6.703236412	2.03853E-11	6.27362E-11
INPP4B	572.9654346	-0.602406138	0.089864644	-6.703483285	2.03509E-11	6.26401E-11
DCX	1.87827626	-1.323224375	0.197381806	-6.703882205	2.02954E-11	6.2479E-11
GPR52	83.85725748	-0.523770826	0.078114196	-6.705193888	2.01139E-11	6.19301E-11
S100G	3.8164828	-0.509182003	0.075936649	-6.705352563	2.00921E-11	6.18726E-11
FANCG	51.95146208	-0.220242095	0.032842405	-6.706028276	1.99993E-11	6.15966E-11
PTPN11	3680.668193	0.153869948	0.022943642	6.706430802	1.99442E-11	6.14367E-11
SRL	12.01310476	-0.544310601	0.08115829	-6.706777632	1.98969E-11	6.13005E-11
FRYL	5081.455401	-0.301538612	0.044952469	-6.707943203	1.97387E-11	6.08226E-11
PLD2	211.7340099	0.482371067	0.071903756	6.708565607	1.96547E-11	6.05733E-11
SMIM35	94.13323758	0.47416849	0.070677737	6.708880433	1.96123E-11	6.04523E-11
TBATA	2.14935822	-0.797282122	0.11880115	-6.711064028	1.9321E-11	5.95638E-11

ENY2	2106.801653	0.239899422	0.035738987	6.712541194	1.91264E-11	5.8973E-11
ARID4A	4094.266204	0.26901701	0.040070071	6.713664498	1.89796E-11	5.85298E-11
NDN	77.75018868	-0.988221782	0.147191287	-6.713860592	1.89541E-11	5.84604E-11
THUMPD1	2557.801106	0.271537688	0.040434785	6.71544776	1.8749E-11	5.78366E-11
SLC25A4	54.75599385	-0.402425623	0.059922562	-6.71576133	1.87087E-11	5.77215E-11
ST3GAL6	763.5336426	0.428221818	0.063753203	6.716867536	1.85673E-11	5.72942E-11
UGT8	41.54735652	0.629197823	0.093672302	6.717010396	1.85491E-11	5.72471E-11
VCL	6020.308176	-0.273336075	0.040667395	-6.721258593	1.80162E-11	5.56111E-11
C12orf57	415.8247322	-0.455238702	0.06772008	-6.722359157	1.78806E-11	5.52013E-11
SERPINA5	0.664023084	-1.390021562	0.206746042	-6.723328521	1.77619E-11	5.48438E-11
DIMT1	311.979019	-0.165583779	0.024627726	-6.723470112	1.77447E-11	5.47991E-11
SEMA3D	9.428201712	-0.871429985	0.129606907	-6.723638463	1.77242E-11	5.47444E-11
QRICH1	4307.620403	0.182459257	0.027135471	6.724012873	1.76787E-11	5.46125E-11
ANKRD17	5112.526838	-0.199422799	0.029654999	-6.724761495	1.7588E-11	5.4341E-11
OSM	613.2933427	0.476301234	0.070826904	6.724863138	1.75758E-11	5.43117E-11
XPO4	2445.245175	0.199787218	0.029708357	6.7249501	1.75653E-11	5.42878E-11
RIC3	42.82324914	0.46166038	0.068646386	6.725195671	1.75357E-11	5.42049E-11
GPR62	3.767949834	-0.643668598	0.09570403	-6.725616427	1.74851E-11	5.4057E-11
FABP2	1.61781196	-0.753749966	0.112071407	-6.725622381	1.74843E-11	5.4057E-11
GPD1L	410.6759981	-0.341718172	0.050808162	-6.725655007	1.74804E-11	5.4057E-11
SLC35E1	2174.081228	0.233343852	0.03469214	6.726130285	1.74235E-11	5.38921E-11
ACKR2	53.57520518	-0.513254086	0.076295907	-6.727151004	1.73017E-11	5.3524E-11
OR6K3	5.29788249	-0.79759536	0.118561676	-6.727261156	1.72886E-11	5.3492E-11
ZMYND10	84.58675942	-0.35272761	0.052429933	-6.727599878	1.72484E-11	5.33761E-11
DHFR	546.4068667	-0.324387659	0.048211952	-6.728365947	1.71579E-11	5.31043E-11
SLC29A1	1291.270549	-0.296959122	0.044134929	-6.728437764	1.71494E-11	5.30865E-11
EIF5	13977.66697	0.211076682	0.031364724	6.729747736	1.69957E-11	5.26191E-11
OR1L3	1.001371357	-0.865994382	0.128672128	-6.730240591	1.69383E-11	5.24494E-11
HRAS	265.0134534	-0.24024232	0.035695791	-6.730270238	1.69348E-11	5.24471E-11
ZBTB20	1391.140739	-0.333677235	0.049566097	-6.731965135	1.67387E-11	5.18478E-11
RLN2	2.77906232	-0.797856413	0.118514161	-6.732161013	1.67162E-11	5.17862E-11
DNPEP	1436.354176	-0.163467621	0.02427799	-6.733161127	1.66016E-11	5.14395E-11
NRSN1	1.236801884	-0.804746917	0.119489346	-6.734884265	1.6406E-11	5.08416E-11
ALLC	0.935538607	-1.218054621	0.180854039	-6.735014764	1.63913E-11	5.0804E-11
COL24A1	891.7873131	-0.478438974	0.071037537	-6.735016327	1.63912E-11	5.0804E-11
CDK6	16469.38522	-0.315233245	0.046804882	-6.735050451	1.63873E-11	5.0804E-11
ETDC	3.932159869	-0.491929145	0.072991283	-6.7395602	1.58867E-11	4.92633E-11

PAOX	12.80979372	-0.438724366	0.065093586	-6.739901673	1.58494E-11	4.91554E-11
CALM1	6191.647366	0.242578882	0.035990996	6.739988096	1.584E-11	4.9134E-11
EDIL3	119.5029525	-1.065740521	0.158115662	-6.740259063	1.58104E-11	4.90502E-11
TSTD1	314.8984977	-0.427377904	0.06340333	-6.740622396	1.5771E-11	4.89355E-11
MYLPF	64.17938339	-0.480503821	0.071281801	-6.740904622	1.57404E-11	4.88482E-11
POM121C	3398.996361	0.210661421	0.031250995	6.740950769	1.57354E-11	4.88405E-11
OR51I1	3.110605457	-0.726107486	0.107708071	-6.741439869	1.56825E-11	4.8684E-11
SPINK13	0.735638744	-1.06722723	0.158293688	-6.742070663	1.56145E-11	4.84808E-11
MDFIC2	0.844740968	-1.4516572	0.215311378	-6.74212952	1.56082E-11	4.84688E-11
VAMP8	2918.00979	-0.418777439	0.062108259	-6.742701278	1.55469E-11	4.82861E-11
PHC3	6083.718477	0.208855093	0.030970037	6.743779262	1.54319E-11	4.79366E-11
PLIN3	2883.396061	0.224239821	0.033249884	6.744078304	1.54001E-11	4.78456E-11
APOC3	2.636347058	-1.100044818	0.163086071	-6.745179477	1.52838E-11	4.74916E-11
NTN3	15.76456141	-0.5479434	0.081224546	-6.746032156	1.51943E-11	4.7221E-11
FKBP11	519.2141199	-0.239711631	0.035532775	-6.746212033	1.51755E-11	4.717E-11
CCKAR	1.673006883	-1.170292676	0.173417699	-6.748403899	1.4948E-11	4.64704E-11
SAPCD1	51.90840473	-0.331583255	0.049118481	-6.750682165	1.47152E-11	4.57538E-11
CDC25C	151.8244084	-0.323152709	0.047865375	-6.751283406	1.46543E-11	4.55718E-11
PROP1	1.775273274	-0.782016134	0.115820659	-6.751957202	1.45864E-11	4.53678E-11
KIR3DL1	6.213333273	0.668525757	0.099009726	6.752122057	1.45698E-11	4.53234E-11
SCGB2B2	20.99941524	-0.365144006	0.054077293	-6.75226117	1.45559E-11	4.52872E-11
PDZD4	37.10539032	0.470023675	0.06960233	6.752987698	1.44831E-11	4.50681E-11
TRIM15	31.20481021	-0.796170631	0.117896151	-6.753152053	1.44667E-11	4.50242E-11
PCDHB15	9.049466655	-0.653701603	0.096782654	-6.754326086	1.43501E-11	4.46682E-11
GPR137	396.8485842	-0.221373541	0.0327744	-6.754465136	1.43363E-11	4.46325E-11
VIT	11.22343478	-0.912127521	0.135013174	-6.755840871	1.42009E-11	4.4218E-11
RASGRP1	800.1084597	0.385658272	0.057084938	6.755867462	1.41983E-11	4.4217E-11
ECE1	1613.608989	0.488226519	0.072265294	6.756030371	1.41824E-11	4.41743E-11
VDR	551.5343093	0.484983158	0.071782906	6.756248602	1.4161E-11	4.41149E-11
RGPD8	223.8996511	0.349190617	0.051683242	6.756360543	1.41501E-11	4.40879E-11
PTAFR	1825.092735	0.544873725	0.080645444	6.756410509	1.41452E-11	4.40797E-11
SLC22A16	537.1790948	-0.352140163	0.052095567	-6.759503365	1.38466E-11	4.31558E-11
DYNC2LI1	187.1176328	-0.260689221	0.038564393	-6.759842512	1.38142E-11	4.30618E-11
PLCG2	4055.743999	-0.265629836	0.039293613	-6.760127566	1.3787E-11	4.2984E-11
FAM83A	215.9743618	-0.793676225	0.117385825	-6.761261182	1.36796E-11	4.26558E-11
ALDH4A1	574.7828389	-0.298975374	0.044217957	-6.761401799	1.36663E-11	4.26212E-11
DENND10	1184.919646	0.289683664	0.042842254	6.76163459	1.36443E-11	4.25595E-11

HCRT	4.487914396	-0.576172355	0.085199249	-6.762645927	1.35494E-11	4.22701E-11
BTN2A1	1178.401272	0.182823958	0.027033746	6.762805312	1.35345E-11	4.22303E-11
SEMA4G	32.39591686	-0.371066515	0.054867137	-6.763001274	1.35162E-11	4.218E-11
CARD18	0.738640791	-0.933179479	0.137972284	-6.763528508	1.34671E-11	4.20334E-11
MRPS33	353.3530757	-0.353255888	0.052221436	-6.764576323	1.337E-11	4.1737E-11
TMBIM1	4106.005191	0.271941547	0.040197514	6.765133377	1.33186E-11	4.15833E-11
SIRPA	2634.317277	0.412483857	0.060965777	6.765826265	1.32551E-11	4.13914E-11
NUB1	1846.983955	0.18926891	0.027972747	6.766189509	1.32218E-11	4.12942E-11
RBP4	6.380477666	0.837821159	0.123823998	6.766226022	1.32185E-11	4.12904E-11
CLEC4M	5.857463336	-0.569415368	0.084148407	-6.766799129	1.31663E-11	4.11338E-11
KRBA1	300.7391756	-0.267783802	0.039571459	-6.767094511	1.31394E-11	4.10565E-11
ZNF701	269.1892007	-0.249821477	0.0369063	-6.769074119	1.29609E-11	4.05052E-11
QTRT2	871.8905543	0.184792117	0.027297415	6.769582926	1.29154E-11	4.03694E-11
PRPF38A	2476.990228	0.154098281	0.02276325	6.769608156	1.29132E-11	4.03689E-11
OR51Q1	2.077161114	-0.789619074	0.116628087	-6.770402367	1.28425E-11	4.01543E-11
MBTPS1	4759.095932	0.162946626	0.024064039	6.771374656	1.27564E-11	3.98917E-11
C13orf42	2.02041707	-0.862068867	0.127292695	-6.772335717	1.2672E-11	3.96338E-11
COPS4	1201.28144	0.185082949	0.027328843	6.77243997	1.26628E-11	3.96116E-11
KLHL15	3554.930225	0.271169359	0.040038592	6.772699587	1.26401E-11	3.95469E-11
RASSF10	2.39164709	-1.007622104	0.148775921	-6.772749919	1.26357E-11	3.95394E-11
ADIG	2.66565259	-0.872749002	0.128861655	-6.77275955	1.26349E-11	3.95394E-11
NEIL2	191.6107072	-0.257343	0.037987611	-6.774392758	1.2493E-11	3.91053E-11
GCC1	1165.346465	0.227617503	0.033598033	6.774727105	1.24641E-11	3.90212E-11
FAM89B	45.09336388	-0.427396455	0.06308536	-6.774891284	1.245E-11	3.89831E-11
LPAR2	1427.010245	0.25812359	0.038099626	6.774963949	1.24437E-11	3.89698E-11
WNT3	10.98466008	-0.566651607	0.083635901	-6.775219705	1.24217E-11	3.89071E-11
AC009879.3	4.940586416	-0.451278091	0.066600341	-6.775912588	1.23623E-11	3.87273E-11
MBD3L1	1.011133167	-0.880622969	0.129956861	-6.776271482	1.23317E-11	3.86374E-11
SEC11A	2749.875846	0.261049493	0.038523815	6.776314709	1.2328E-11	3.86321E-11
CAPN12	79.45939835	-0.412186843	0.060818036	-6.777378385	1.22376E-11	3.8355E-11
FLYWCH1	1501.273546	-0.205743775	0.030354687	-6.777990262	1.21859E-11	3.8199E-11
EN2	1.137056198	-1.054916518	0.155602272	-6.779570129	1.20534E-11	3.77897E-11
C4orf51	3.144707674	-0.685763937	0.101151247	-6.779589524	1.20518E-11	3.77897E-11
SGO1	124.7893445	-0.316558313	0.046691832	-6.779736387	1.20395E-11	3.77583E-11
KCNQ3	14.354766	-0.760213671	0.112117211	-6.780526053	1.19739E-11	3.75585E-11
SMARCA2	11069.34835	0.235765336	0.03476997	6.780717318	1.19581E-11	3.75149E-11
NME5	2.545431387	-0.85415633	0.125934197	-6.782560639	1.18064E-11	3.70451E-11

WNK1	14194.19213	-0.307832166	0.045385709	-6.782579164	1.18049E-11	3.70451E-11
NDUFB3	307.4473299	-0.372758685	0.054951277	-6.783439879	1.17348E-11	3.6832E-11
BDNF	23.64471975	-0.698690385	0.102996648	-6.783622588	1.17199E-11	3.67914E-11
UBE2V2	479.6275226	-0.27220109	0.040124557	-6.783902739	1.16972E-11	3.67259E-11
MAFG	3786.876914	0.281703558	0.041520824	6.784633111	1.16382E-11	3.65465E-11
MGAM2	74.91358503	-0.688265277	0.101435838	-6.785227915	1.15903E-11	3.64021E-11
PARD3B	70.47985023	-0.964483765	0.142129408	-6.785955	1.15321E-11	3.6225E-11
AL357556.3	58.76488796	-0.270843108	0.039911184	-6.786145622	1.15169E-11	3.6183E-11
ADGRV1	50.63162407	-0.426043187	0.062779396	-6.786353667	1.15003E-11	3.61367E-11
GTF2H2	109.9138029	-0.357196941	0.052623925	-6.787729051	1.13912E-11	3.57997E-11
ZNF547	30.92132467	-0.35703897	0.052592283	-6.788809156	1.13063E-11	3.55385E-11
UTP6	1249.233361	-0.202852902	0.02987155	-6.790839476	1.11483E-11	3.50475E-11
SRP68	1915.029425	0.143658894	0.021153155	6.791369747	1.11074E-11	3.49245E-11
AGPAT5	932.0108885	-0.278754723	0.041044498	-6.791524753	1.10955E-11	3.48926E-11
KDM2A	9800.521357	0.171479294	0.025247223	6.79200617	1.10585E-11	3.47819E-11
RIPOR1	2144.301763	0.279253729	0.041113471	6.792268396	1.10384E-11	3.47243E-11
ATP10D	615.386498	-0.372652958	0.054857185	-6.793147673	1.09713E-11	3.45188E-11
CCZ1B	405.9815852	0.181577043	0.026725723	6.794092886	1.08996E-11	3.42987E-11
PGGT1B	903.2195343	0.251436946	0.037006217	6.794451469	1.08725E-11	3.4219E-11
ETFA	1262.368641	-0.215489332	0.031709831	-6.795663204	1.07815E-11	3.39381E-11
TRMT11	383.2081807	-0.251557986	0.037015238	-6.796065527	1.07515E-11	3.38489E-11
SLC39A12	2.382146356	-1.198905381	0.176359377	-6.798081275	1.06022E-11	3.33842E-11
C1orf158	1.84673145	-0.679636806	0.099973072	-6.798198652	1.05935E-11	3.33624E-11
PADI6	17.01247884	-0.694525501	0.10216058	-6.798370785	1.05809E-11	3.33279E-11
KRTAP5-11	0.866557573	-1.037923288	0.152661986	-6.798832617	1.0547E-11	3.32266E-11
NOS1	6.693887971	-0.642300921	0.094459751	-6.799731231	1.04815E-11	3.30253E-11
ZNF420	101.6746125	-0.283702825	0.041713201	-6.80127197	1.03699E-11	3.26793E-11
LYSMD1	57.7050433	-0.274939725	0.040415986	-6.802746983	1.02643E-11	3.23515E-11
CR1L	127.9191897	-0.758044993	0.111414207	-6.80384497	1.01863E-11	3.21109E-11
TMEM225B	9.016030993	-0.425810655	0.062572575	-6.805068383	1.01001E-11	3.18444E-11
CRYGN	5.766358983	-0.704918644	0.103572371	-6.80604912	1.00315E-11	3.16332E-11
DLG1	1962.350041	-0.17132767	0.025172764	-6.806073031	1.00299E-11	3.16331E-11
NDUFB6	399.8456596	-0.367960568	0.054055075	-6.807141874	9.95567E-12	3.14041E-11
VSTM2L	9.749929447	-0.648014111	0.095182171	-6.808145968	9.88645E-12	3.11908E-11
TMCC2	2934.627295	-0.720290945	0.105797268	-6.808218787	9.88145E-12	3.118E-11
TMEM26	2.541776005	-1.000284645	0.146922663	-6.808239257	9.88005E-12	3.118E-11
XRRA1	521.9194598	-0.356151141	0.052309618	-6.808521159	9.86071E-12	3.11246E-11

PCP4L1	19.13945292	-0.428728695	0.062960864	-6.809447478	9.79743E-12	3.09299E-11
SGTA	2605.872682	0.240748237	0.035352326	6.809968757	9.76199E-12	3.0823E-11
DPYSL4	32.35064364	-0.526387365	0.077286947	-6.810818499	9.7045E-12	3.06464E-11
FAM120B	1177.742674	-0.232540094	0.034138092	-6.811748376	9.64196E-12	3.04538E-11
PCDH11X	1.114289924	-0.818820237	0.120199327	-6.812186518	9.61264E-12	3.03661E-11
HFM1	14.44750075	-0.951075939	0.139608624	-6.812444054	9.59544E-12	3.03166E-11
CHD3	8089.13762	-0.234552138	0.034423466	-6.81372812	9.51014E-12	3.0052E-11
DGKQ	912.9221844	-0.2752078	0.040389243	-6.813888501	9.49953E-12	3.00233E-11
CES2	662.2886136	-0.182804219	0.026827103	-6.814161745	9.4815E-12	2.99712E-11
BPIFB3	0.931626174	-0.934726017	0.137165884	-6.814566331	9.45485E-12	2.98918E-11
LSM7	1504.0831	-0.40823925	0.05989229	-6.816223743	9.34647E-12	2.95539E-11
SOHLH2	5.216789314	-0.652556092	0.095726886	-6.816852825	9.30565E-12	2.94296E-11
TMEM241	323.462798	-0.240793453	0.035306993	-6.819993178	9.10448E-12	2.8798E-11
PAH	1.432410483	-1.313687629	0.192614714	-6.820286995	9.08588E-12	2.87438E-11
IRF2	628.9626913	-0.311363878	0.045643343	-6.821671188	8.99875E-12	2.84728E-11
RBM46	2.098340595	-2.119896155	0.310731237	-6.82228209	8.96055E-12	2.83565E-11
TMEM155	2.909412861	1.07746549	0.157927877	6.822516121	8.94596E-12	2.83149E-11
NUDT10	25.18350555	0.825240867	0.120931463	6.824037733	8.85166E-12	2.8021E-11
DUSP29	1.869298163	-0.70243061	0.10292085	-6.824959286	8.79503E-12	2.78462E-11
PDLIM3	4.703973108	-0.599395003	0.087823569	-6.824990285	8.79313E-12	2.78447E-11
HOXC12	0.907881732	-1.090853159	0.159810377	-6.825921953	8.73624E-12	2.7669E-11
KLHDC4	1090.398807	-0.20719535	0.030353303	-6.826122068	8.72407E-12	2.76349E-11
STMND1	4.006210088	-0.636263531	0.09320659	-6.826379265	8.70845E-12	2.75899E-11
CCDC137	426.6471016	-0.22595118	0.033098713	-6.826585108	8.69597E-12	2.75548E-11
HSD17B4	1473.861674	-0.25532412	0.037394213	-6.827904559	8.61639E-12	2.73071E-11
HNRNPL	8733.342601	0.149783254	0.021935	6.828504782	8.58042E-12	2.71975E-11
UBXN2A	1091.227859	0.231285405	0.033863527	6.829926654	8.49581E-12	2.69337E-11
PCDHB9	9.159556724	-0.778480638	0.113958167	-6.831284315	8.41578E-12	2.66843E-11
DOCK1	388.5004659	-0.863890811	0.126456102	-6.831547053	8.40037E-12	2.66397E-11
SLC47A2	11.09302227	-0.540700089	0.079145412	-6.831730091	8.38966E-12	2.66101E-11
KLRG1	96.98551069	-0.401842079	0.058818458	-6.831904309	8.37947E-12	2.65821E-11
MCF2L	126.8183522	-0.563268184	0.082444997	-6.832048079	8.37108E-12	2.65598E-11
TSPAN15	62.13130738	-0.744925869	0.109025455	-6.832586674	8.3397E-12	2.64645E-11
IPP	98.25199585	-0.323808182	0.047384312	-6.833658009	8.27762E-12	2.62718E-11
SCAMP3	1089.017326	-0.274059404	0.040101851	-6.834083704	8.25308E-12	2.61981E-11
UBE2R2	3305.041543	0.208884536	0.030560987	6.835006273	8.20014E-12	2.60343E-11
BTN1A1	5.585788266	-0.547619681	0.080111147	-6.835748831	8.15778E-12	2.5904E-11

BMX	68.08057911	-0.775001588	0.113347321	-6.837405428	8.06403E-12	2.56105E-11
FAXC	1.951113321	-0.801487238	0.117209355	-6.83808247	8.02602E-12	2.54939E-11
WAPL	3825.872379	0.19760115	0.02889647	6.838245227	8.01691E-12	2.54691E-11
ARCN1	2514.634422	0.199320334	0.02914106	6.839845111	7.92788E-12	2.51903E-11
PDE1C	6.567811808	-0.708688588	0.103609878	-6.839971236	7.92091E-12	2.51723E-11
BOLL	9.599469453	-0.717735653	0.104922941	-6.840597921	7.88633E-12	2.50664E-11
PRICKLE4	1.692487164	-0.573787566	0.083868945	-6.841478241	7.83801E-12	2.49169E-11
GPR15	22.05274139	0.618331222	0.090371665	6.842091752	7.80451E-12	2.48144E-11
ARHGEF12	3489.674339	-0.59873516	0.087507081	-6.842133836	7.80221E-12	2.48112E-11
TGFBRAP1	4132.904268	0.273865374	0.040024846	6.84238414	7.78859E-12	2.47719E-11
WIPF1	5023.464875	0.23078578	0.033727696	6.842619189	7.77582E-12	2.47353E-11
TOP3A	1561.454017	-0.172751191	0.025245734	-6.84278731	7.76669E-12	2.47103E-11
ZNF582	51.50489394	-0.246682377	0.036036415	-6.845363992	7.62817E-12	2.42735E-11
POU5F2	235.031766	-0.507251893	0.074100469	-6.845461268	7.62298E-12	2.42609E-11
POTEB3	1.058001908	-2.411095092	0.352188197	-6.846041715	7.59213E-12	2.41667E-11
MYH2	1.02502082	-1.240330828	0.181168478	-6.846283858	7.5793E-12	2.41297E-11
FAIM2	2.724962448	-0.6988862	0.102080226	-6.846440585	7.57101E-12	2.41073E-11
EVI5L	379.0866441	-0.269486247	0.039356528	-6.847307373	7.52529E-12	2.39656E-11
ATP1B2	138.6961075	-0.683646166	0.099838225	-6.847539238	7.51311E-12	2.39307E-11
PARVA	13.07872295	-0.71305923	0.104106017	-6.849356539	7.41829E-12	2.36325E-11
NUP42	490.1241181	-0.193967679	0.028314696	-6.850424279	7.36313E-12	2.34606E-11
OAZ2	2809.194879	0.223393892	0.032608419	6.850804204	7.3436E-12	2.34022E-11
ASF1B	346.8982405	-0.367240928	0.053598636	-6.85168424	7.29855E-12	2.32624E-11
MST1R	25.70479224	-0.570866351	0.083316024	-6.851819419	7.29166E-12	2.32442E-11
CREBBP	9946.423337	0.226353812	0.033033575	6.85223486	7.27051E-12	2.31806E-11
TNNT3	172.9352598	-0.441516446	0.064419132	-6.853809288	7.19089E-12	2.29305E-11
ZNF536	11.32808331	-1.047164034	0.152769994	-6.854513819	7.15555E-12	2.28215E-11
ANKS6	217.6346138	-0.417412	0.060874612	-6.856914351	7.03638E-12	2.24451E-11
SLC26A4	11.56627602	-0.439544809	0.064097886	-6.857399504	7.01253E-12	2.23727E-11
LMTK2	1949.023358	0.364878246	0.053207154	6.857691539	6.99822E-12	2.23306E-11
PROSER1	2429.472028	0.253452962	0.036958145	6.857837693	6.99106E-12	2.23114E-11
EPHX2	74.23498536	-0.620060407	0.090416251	-6.857842487	6.99083E-12	2.23114E-11
CCL13	1.637689659	-1.253466615	0.182772137	-6.858083703	6.97904E-12	2.22803E-11
DZIP1	19.1133253	-0.790029576	0.11519162	-6.858394526	6.96387E-12	2.22355E-11
RC3H1	6981.035539	0.245874694	0.035847895	6.858832044	6.94258E-12	2.21712E-11
GSPT1	4277.947336	-0.265989516	0.038772903	-6.860190877	6.87686E-12	2.19649E-11
CMTM3	2695.734651	0.318502065	0.046426636	6.860330535	6.87014E-12	2.1947E-11

MMP7	15.61920554	-0.81106491	0.11820886	-6.861286962	6.82429E-12	2.18041E-11
LYRM9	74.14481426	-0.263540986	0.038407333	-6.861736181	6.80286E-12	2.17392E-11
NFIX	2754.381338	-0.463428465	0.067533179	-6.862233813	6.7792E-12	2.16671E-11
BCL6B	140.7716242	0.689507805	0.100472658	6.862641245	6.75988E-12	2.16089E-11
PRKACA	1578.474983	0.287391957	0.041869263	6.864031877	6.69437E-12	2.1403E-11
CXorf38	1532.265508	0.198170735	0.02886928	6.864415572	6.6764E-12	2.1349E-11
NRIP2	342.5357154	-0.24808664	0.036140385	-6.864526739	6.6712E-12	2.13359E-11
OLFML3	39.16583762	-0.584145612	0.085093457	-6.864753598	6.66061E-12	2.13055E-11
GHDC	722.9303297	-0.287060126	0.041801263	-6.86725958	6.54469E-12	2.09381E-11
NFKB2	4316.983475	0.395818567	0.057632602	6.867962857	6.51252E-12	2.08386E-11
RING1	994.6784912	-0.188234963	0.027407332	-6.868051438	6.50848E-12	2.0829E-11
SKA2	372.2059052	-0.309725908	0.045092458	-6.868685363	6.47962E-12	2.07401E-11
SLC1A7	4.452592316	-0.625228587	0.091025173	-6.868743775	6.47697E-12	2.0735E-11
ECM2	146.6143501	-0.42437086	0.061781422	-6.868907245	6.46956E-12	2.07146E-11
SYBU	18.97551257	-0.680311034	0.099021202	-6.870357252	6.40413E-12	2.05085E-11
C12orf40	5.638388121	-0.669220807	0.097399692	-6.87087192	6.38107E-12	2.0438E-11
MFSD5	533.5396526	0.292632374	0.042587785	6.871274749	6.36307E-12	2.03837E-11
SYVN1	2484.612406	0.25814807	0.037569084	6.871289904	6.36239E-12	2.03837E-11
TAF1A	106.7377004	-0.269427012	0.039210195	-6.871351006	6.35967E-12	2.03795E-11
PCDHGB3	106.8987508	-0.583204885	0.084873762	-6.87143909	6.35574E-12	2.03702E-11
LCK	343.0977033	0.51179554	0.074466762	6.872805101	6.29515E-12	2.01793E-11
CNTN4	53.15578298	-1.087540397	0.158235176	-6.872937026	6.28933E-12	2.0164E-11
ZNF678	382.245576	-0.308332696	0.044856098	-6.873818924	6.25055E-12	2.00429E-11
BPTF	6729.36011	-0.197162563	0.02868251	-6.873964768	6.24416E-12	2.00257E-11
CAPN1	5363.543545	-0.198402891	0.028854281	-6.87602959	6.15437E-12	1.9741E-11
CDCP2	0.849214599	-0.783315781	0.113918143	-6.876128401	6.15011E-12	1.97305E-11
HERC3	268.5972561	0.291878773	0.042447504	6.876229341	6.14575E-12	1.97198E-11
CYP11A1	2.95987275	-0.839872195	0.122136056	-6.876529547	6.13282E-12	1.96815E-11
ZNF609	3742.486892	-0.305786767	0.044467369	-6.876655276	6.12741E-12	1.96674E-11
SLC25A12	635.9942447	-0.214296912	0.031161829	-6.876904236	6.11672E-12	1.96363E-11
TSPO2	171.6043983	-0.588591188	0.085585117	-6.877260975	6.10143E-12	1.95904E-11
MYRFL	8.109957732	-0.618909779	0.08997513	-6.878676161	6.04113E-12	1.94E-11
CRTAM	63.32831756	0.449087791	0.065272127	6.880238367	5.97525E-12	1.91916E-11
GABRG3	1.364329708	-0.841010432	0.122228707	-6.880629406	5.95887E-12	1.91421E-11
PRDM12	2.972846827	-0.635658776	0.092374634	-6.881313093	5.93033E-12	1.90536E-11
MYO3A	4.051480005	-0.98471603	0.143097915	-6.881414221	5.92613E-12	1.90432E-11
NPFFR2	1.494750769	-0.906996941	0.131799483	-6.881642612	5.91663E-12	1.90158E-11

ATXN7L3	2797.090652	0.18616351	0.027050335	6.882114763	5.89705E-12	1.8956E-11
PCDHB14	17.35772749	-0.724037213	0.10520118	-6.88240579	5.88501E-12	1.89204E-11
SC5D	504.827331	0.279532089	0.040595484	6.885792797	5.74666E-12	1.84786E-11
USP43	144.3100503	-0.41299524	0.059973764	-6.886265146	5.72762E-12	1.84204E-11
KIF9	387.4288578	-0.234472164	0.034047924	-6.886533258	5.71684E-12	1.83888E-11
NOSIP	708.9683906	-0.268581307	0.038984398	-6.889456231	5.60061E-12	1.80179E-11
MED15	5796.235931	0.204778953	0.029722176	6.889769809	5.58827E-12	1.79811E-11
ZNF79	229.3080882	-0.254508852	0.036938301	-6.890107155	5.57504E-12	1.79415E-11
ICMT	639.7963986	-0.385831433	0.055996018	-6.890336924	5.56604E-12	1.79155E-11
NEB	348.0829768	-0.390719263	0.056702371	-6.890704157	5.55169E-12	1.78722E-11
RELN	26.46230177	-0.84358968	0.122408553	-6.891590981	5.51718E-12	1.77641E-11
RGS8	1.218715414	-0.88968921	0.129090272	-6.891992662	5.50162E-12	1.77169E-11
MSH4	24.50638715	-0.386896775	0.056135556	-6.892187501	5.49409E-12	1.76955E-11
TSPAN12	10.51873187	-1.074138195	0.155847651	-6.892232165	5.49236E-12	1.76929E-11
MLST8	582.2457578	-0.273290067	0.039649629	-6.892626133	5.47717E-12	1.76469E-11
TTC26	137.1924266	-0.303402717	0.044017295	-6.892806933	5.47021E-12	1.76273E-11
DCHS2	4.673735202	-0.834174679	0.121002089	-6.893886613	5.42883E-12	1.74969E-11
DEPDC7	154.1185503	0.492347201	0.071406162	6.895024028	5.38557E-12	1.73603E-11
SUMF1	767.9173897	-0.239908198	0.034794339	-6.895035369	5.38514E-12	1.73603E-11
FAM204A	1744.001111	0.203705444	0.029543323	6.89514312	5.38106E-12	1.73515E-11
DEDD2	2016.836191	0.334176876	0.048451438	6.89715083	5.30558E-12	1.71109E-11
WDR72	2.767819172	-0.925589656	0.134180654	-6.898085749	5.27079E-12	1.70015E-11
TAF9B	855.291447	0.212696144	0.030825253	6.900061608	5.198E-12	1.67695E-11
CENPE	582.2784369	-0.399151916	0.057845137	-6.900353915	5.18731E-12	1.67378E-11
SOD1	1710.207219	-0.351897545	0.050996891	-6.900372567	5.18663E-12	1.67378E-11
ME3	547.6744391	-0.437352982	0.063375318	-6.900998664	5.16382E-12	1.66675E-11
PYCR1	175.1414145	-0.467077096	0.067679428	-6.901315633	5.15231E-12	1.66331E-11
BTN3A3	468.4072861	-0.329081796	0.047670236	-6.903296897	5.08093E-12	1.64053E-11
ATP1B4	3.778356095	-0.692914446	0.100367929	-6.903743585	5.06497E-12	1.63565E-11
DDX21	7435.68597	0.207762555	0.03009005	6.904692994	5.03121E-12	1.62501E-11
FAM133A	1.430348328	-1.564169081	0.226528024	-6.90496942	5.02142E-12	1.62212E-11
TRIP6	347.1397266	-0.430481625	0.06233831	-6.905570943	5.00019E-12	1.61553E-11
PTPN2	2203.170646	0.171132054	0.024780093	6.906029559	4.98406E-12	1.61058E-11
OR11H4	1.218675824	-0.79779626	0.115520238	-6.906116814	4.981E-12	1.60986E-11
MAEL	14.57229923	-0.641697395	0.092913797	-6.906373614	4.97199E-12	1.60721E-11
RAB10	1991.144011	-0.223093268	0.03230189	-6.90650823	4.96728E-12	1.60596E-11
ERO1B	727.1786847	0.299813251	0.04339559	6.908841369	4.88628E-12	1.58003E-11

CENPJ	904.0690589	-0.288146259	0.041699975	-6.909986332	4.847E-12	1.56759E-11
ZNF223	21.65321772	-0.369333366	0.053448851	-6.910033843	4.84538E-12	1.56732E-11
SGO2	303.3623584	-0.344846582	0.049901592	-6.910532712	4.82837E-12	1.56208E-11
KANK2	787.2511498	-0.415392114	0.060101644	-6.911493378	4.79578E-12	1.55179E-11
VPS18	1378.746215	0.217274262	0.031432693	6.912365522	4.76638E-12	1.54253E-11
ASB18	1.215484063	-1.065402718	0.154103363	-6.913559182	4.72642E-12	1.52985E-11
GRIN2B	5.382240592	-0.67279722	0.09730851	-6.914063513	4.70964E-12	1.52467E-11
TMEM198	10.65255591	-0.654871628	0.094704267	-6.914911527	4.68155E-12	1.51583E-11
PHKA1	79.14814389	-0.59823695	0.08650531	-6.915609594	4.65856E-12	1.50864E-11
SRM	1759.164111	-0.501474537	0.072503654	-6.91654157	4.62803E-12	1.499E-11
TTC1	567.8191295	-0.219253546	0.031698806	-6.916776169	4.62037E-12	1.49677E-11
PLK3	2630.751136	0.390271617	0.056421697	6.917048499	4.6115E-12	1.49414E-11
MPL	611.3129477	-0.611021207	0.088335329	-6.917064916	4.61097E-12	1.49414E-11
UTP20	967.8750543	-0.255873457	0.0369856	-6.918191253	4.57446E-12	1.48263E-11
CARD9	1033.900114	-0.409150222	0.059139223	-6.918424092	4.56695E-12	1.48044E-11
CASP3	1259.722551	0.272882547	0.039426703	6.921262066	4.47637E-12	1.45132E-11
PDX1	1.464515405	-0.963578612	0.139207685	-6.921877983	4.45695E-12	1.44526E-11
ISLR2	3.938470509	-0.579872099	0.083773755	-6.921882663	4.4568E-12	1.44526E-11
SLC22A14	8.565346268	-0.612235122	0.088443611	-6.922321637	4.44301E-12	1.44122E-11
SOBP	55.78487299	-0.789238465	0.11400052	-6.923112833	4.41826E-12	1.43342E-11
CCDC198	7.937771843	-0.521789935	0.075362828	-6.923704287	4.39984E-12	1.42769E-11
SENP1	1013.564868	0.181268209	0.026173759	6.925570414	4.34222E-12	1.40922E-11
PANX2	465.1686646	0.561810174	0.081107501	6.92673511	4.30664E-12	1.39791E-11
PAQR8	211.4267869	-0.547833487	0.079084561	-6.927186322	4.29293E-12	1.39369E-11
PLPBP	710.9001663	-0.282677491	0.040802195	-6.927997211	4.2684E-12	1.38596E-11
ZNF83	1391.735998	-0.26040041	0.037582924	-6.928689536	4.24757E-12	1.37942E-11
UMODL1	373.5919178	-0.734443784	0.105999794	-6.928728379	4.2464E-12	1.37927E-11
MRO	4.800096948	-0.6672657	0.096303934	-6.928748114	4.24581E-12	1.37927E-11
DLX1	9.810695337	-0.808671886	0.116669712	-6.931292388	4.17013E-12	1.35495E-11
TNXB	687.7254893	-0.567695614	0.081888517	-6.932542336	4.13344E-12	1.34325E-11
AGL	780.5990629	-0.252935187	0.036484609	-6.932654392	4.13016E-12	1.34241E-11
TCF15	5.32090998	-0.860200553	0.124076778	-6.932808589	4.12566E-12	1.34117E-11
B2M	63827.55535	0.325061357	0.046877065	6.934336878	4.08131E-12	1.32697E-11
FAM180B	65.4427032	-0.436712468	0.062974749	-6.934723391	4.07017E-12	1.32357E-11
CHRAC1	1117.520446	0.275888742	0.039783298	6.934788077	4.06831E-12	1.32318E-11
CREBRF	3750.686634	0.280994033	0.040517691	6.935094851	4.05949E-12	1.32053E-11
PLEKHA7	98.3362806	-0.44333911	0.063925525	-6.935243978	4.05521E-12	1.31936E-11

NR6A1	362.219596	-0.560825401	0.080861813	-6.935602578	4.04493E-12	1.31623E-11
FNDC1	6.080022431	-0.692999443	0.099918926	-6.93561738	4.04451E-12	1.31623E-11
LNX1	61.63773801	-0.363699407	0.052436519	-6.935994549	4.03373E-12	1.31303E-11
CHMP4C	2.510910134	-0.994916761	0.143437483	-6.936239691	4.02674E-12	1.31097E-11
ATP7A	819.3711641	-0.348235338	0.050195253	-6.937614979	3.98775E-12	1.29849E-11
KCTD6	232.5043005	0.332369614	0.047904543	6.938164819	3.97226E-12	1.29366E-11
NACC2	631.2274011	0.528870324	0.076217005	6.939006946	3.94866E-12	1.28619E-11
FAM118B	265.7429041	-0.23882702	0.034415959	-6.939426644	3.93694E-12	1.28259E-11
CD163L1	47.16418281	-0.697954964	0.100576666	-6.939531742	3.93402E-12	1.28185E-11
PSMD6	3918.718858	0.165934718	0.023909895	6.940002	3.92094E-12	1.2778E-11
TUBA3D	43.85047055	-0.32702489	0.047121038	-6.940103732	3.91812E-12	1.27709E-11
FAM3B	10.31039819	-0.951611633	0.13711045	-6.940474864	3.90784E-12	1.27395E-11
AC108941.3	2.818901984	-0.935467179	0.13478025	-6.940684421	3.90205E-12	1.27228E-11
VPS26C	2160.32317	0.150510123	0.021683156	6.941338254	3.88403E-12	1.26661E-11
PDE4C	9.220883447	-0.681390368	0.098142408	-6.942873984	3.84202E-12	1.25312E-11
EIF4G3	3613.665931	-0.185689303	0.02674012	-6.944220959	3.80554E-12	1.24143E-11
PTPRB	131.3388435	-0.867889218	0.124971152	-6.94471648	3.79221E-12	1.23729E-11
VXN	2.005920997	-0.767585793	0.110521793	-6.945108055	3.78171E-12	1.23407E-11
TMEM59	4441.856625	0.206525386	0.029736376	6.945210406	3.77897E-12	1.23338E-11
GLUD1	2815.29351	0.214819821	0.030928651	6.945657726	3.76701E-12	1.22968E-11
ITGA6	1228.637327	-0.413941996	0.059591548	-6.946320517	3.74936E-12	1.22413E-11
CTTN	204.552681	-0.637211006	0.091728917	-6.946675315	3.73995E-12	1.22126E-11
COMMD10	470.568386	-0.2758267	0.039702368	-6.947361347	3.72182E-12	1.21554E-11
TNFRSF13B	16.84920634	0.518260365	0.074593189	6.947824234	3.70963E-12	1.21176E-11
CCDC167	190.2638332	-0.393797247	0.056664509	-6.949627783	3.66251E-12	1.19657E-11
TAFA4	1.362677936	-1.118579528	0.160944115	-6.950111409	3.64998E-12	1.19267E-11
IARS1	4277.115073	-0.239083168	0.034397482	-6.95060095	3.63733E-12	1.18874E-11
TET2	6283.104105	0.242571518	0.034897483	6.950974701	3.62771E-12	1.18579E-11
ATP2A3	10927.6013	-0.264999759	0.038116655	-6.952335199	3.59288E-12	1.1746E-11
MEIS2	52.90388713	-0.975184368	0.140263369	-6.952523502	3.58809E-12	1.17323E-11
TRPV6	58.2604834	-0.470315499	0.067645774	-6.952622016	3.58558E-12	1.17261E-11
PLEKHF1	94.07212968	0.42555849	0.061204133	6.953100492	3.57344E-12	1.16883E-11
SLC24A2	4.157808026	-0.789228768	0.113476952	-6.954969748	3.52638E-12	1.15363E-11
PRXL2B	314.8826825	-0.324402604	0.046642927	-6.955022518	3.52506E-12	1.15339E-11
MRPS10	771.2128359	0.175261516	0.025198577	6.955214898	3.52025E-12	1.15201E-11
ARHGEF3	999.177326	0.342461134	0.049235923	6.955513623	3.5128E-12	1.14977E-11
ZNF662	154.5750759	-0.594635366	0.085487698	-6.955800424	3.50566E-12	1.14762E-11

OR5C1	4.095991516	-0.602751026	0.086644333	-6.956612256	3.48553E-12	1.14122E-11
AGR2	37.02127399	-0.819511512	0.117789338	-6.957433709	3.46527E-12	1.13478E-11
PACRG	4.193589717	-0.691246952	0.099343613	-6.958141824	3.4479E-12	1.12928E-11
HSPA12B	71.82009074	-0.434662107	0.062466563	-6.958316331	3.44363E-12	1.12807E-11
NKAIN2	47.78224555	-1.187891686	0.170715033	-6.958330898	3.44328E-12	1.12807E-11
NDUFB7	846.7739544	-0.509819686	0.073265604	-6.958513348	3.43882E-12	1.12687E-11
KLK10	3.253968081	-0.60987429	0.087642801	-6.958635289	3.43585E-12	1.12609E-11
SUZ12	2956.414641	0.202645354	0.029121306	6.958663104	3.43517E-12	1.12605E-11
SASH1	72.19805235	0.831013749	0.11941135	6.959252619	3.42083E-12	1.12154E-11
CCDC18	607.2784344	-0.2463212	0.0353928	-6.959641592	3.4114E-12	1.11863E-11
MT-ND4L	33699.4258	-0.786276323	0.112966983	-6.960231199	3.39715E-12	1.11415E-11
FMO1	6.393980752	-0.532082398	0.076440876	-6.96070517	3.38574E-12	1.11059E-11
DNAJC9	521.4196393	-0.230497819	0.033110668	-6.961436602	3.3682E-12	1.10503E-11
TMEM44	576.961973	-0.347404803	0.049887716	-6.963734361	3.31369E-12	1.08732E-11
GDNF	3.779341077	-0.797076511	0.114449438	-6.964442312	3.29707E-12	1.08205E-11
FFAR3	6.69482034	0.78418634	0.112584728	6.965299381	3.27706E-12	1.07567E-11
GNPDA2	339.6150462	0.253079215	0.036330433	6.966039043	3.25989E-12	1.07021E-11
PRPF6	2725.468272	-0.166265703	0.023866599	-6.966459925	3.25015E-12	1.06719E-11
ACTA1	14.67174126	-0.415954814	0.059705459	-6.966780231	3.24277E-12	1.06494E-11
KLHL28	1161.035401	0.227975063	0.032715896	6.968326942	3.20732E-12	1.05348E-11
C17orf100	16.98697146	-0.419922932	0.060260513	-6.968459254	3.20431E-12	1.05267E-11
CEACAM20	2.39296111	-0.854162148	0.122570499	-6.968741692	3.19788E-12	1.05073E-11
ZDHHC5	2602.506626	0.136820122	0.019632874	6.968930061	3.1936E-12	1.0495E-11
SLC46A1	63.07248501	-0.401093652	0.057554471	-6.968939949	3.19338E-12	1.0495E-11
PLPPR1	0.973601187	-1.469911717	0.210921227	-6.969007978	3.19184E-12	1.04927E-11
PDGFD	86.37304048	-0.711553112	0.10209094	-6.969796827	3.17399E-12	1.04358E-11
BBS12	38.32302226	-0.402124131	0.057692162	-6.970169238	3.1656E-12	1.041E-11
ZNHIT6	599.3676007	-0.203423177	0.029181364	-6.97099617	3.14705E-12	1.03507E-11
WIPI2	4410.814577	0.206167866	0.029567809	6.972713703	3.10885E-12	1.02268E-11
ALG1L	6.365148596	-0.528244912	0.075745368	-6.973956635	3.08149E-12	1.01385E-11
HHATL	2.418731177	-0.797607139	0.114356297	-6.974754865	3.06405E-12	1.00828E-11
OR4D9	2.736217669	-0.650200852	0.093219651	-6.974933325	3.06016E-12	1.00717E-11
THOC6	641.2754851	-0.251566229	0.036064366	-6.975479026	3.0483E-12	1.00344E-11
IDI1	1950.425303	0.253100278	0.036273648	6.97752477	3.00426E-12	9.89106E-12
FUT6	32.34669123	-0.60890539	0.087266255	-6.977558373	3.00354E-12	9.89036E-12
AKR1D1	4.264798926	-0.564329523	0.080876717	-6.97765124	3.00156E-12	9.88549E-12
DERL1	2643.395221	0.18707921	0.026809835	6.978006654	2.99397E-12	9.86218E-12

RPL10L	3.33190006	0.826032456	0.11836689	6.978577012	2.98185E-12	9.82389E-12
ARHGEF28	14.49411518	-0.753252077	0.1079294	-6.979118568	2.97038E-12	9.78774E-12
SCAMP5	138.3818581	-0.492748307	0.070597753	-6.979659959	2.95895E-12	9.75174E-12
TCAIM	487.7113696	-0.238593251	0.034183671	-6.979743322	2.9572E-12	9.7476E-12
ELMO1	5558.819437	-0.245839617	0.035209004	-6.982293969	2.90399E-12	9.57381E-12
RNF222	19.9777215	-0.518099423	0.074196876	-6.982765976	2.89424E-12	9.5433E-12
SRA1	1343.489897	0.227374104	0.032556542	6.983975958	2.86941E-12	9.46302E-12
FAM209B	74.9064665	0.394378703	0.056450467	6.986278809	2.82273E-12	9.31063E-12
ANXA5	2223.102467	0.634236298	0.090775534	6.986863843	2.81099E-12	9.27346E-12
CUL4A	2792.051356	-0.1470926	0.021051973	-6.987117018	2.80592E-12	9.25831E-12
PGAP1	316.1587106	0.462285437	0.066161574	6.987219457	2.80387E-12	9.25312E-12
BRD3OS	142.1180194	-0.31383683	0.04490007	-6.989673448	2.75527E-12	9.09424E-12
RNASEH1	957.0620968	0.206859257	0.029591552	6.990483427	2.73941E-12	9.04341E-12
PIK3C2A	2524.493313	0.275430472	0.039396969	6.991158929	2.72625E-12	9.00149E-12
LUC7L2	2218.56836	0.200741376	0.028709371	6.992189974	2.70628E-12	8.93707E-12
C16orf71	56.63921564	-0.361205297	0.051655444	-6.992589089	2.69859E-12	8.91318E-12
CHRNA10	32.54841065	-0.469389546	0.067122828	-6.992994179	2.69081E-12	8.88897E-12
ELK4	2986.680692	0.186163382	0.026620722	6.99317554	2.68733E-12	8.87898E-12
PAFAH1B2	5207.026736	0.232483658	0.033242918	6.993479223	2.68152E-12	8.86127E-12
PROM2	21.66343586	-0.523612628	0.074847274	-6.995747484	2.63848E-12	8.72054E-12
HNF4A	9.76397954	-0.7323924	0.104686788	-6.996034719	2.63308E-12	8.70416E-12
ZBTB49	196.1314703	0.237979733	0.034013491	6.996627654	2.62197E-12	8.66888E-12
CX3CL1	3.463090288	-0.622289715	0.08891809	-6.998460241	2.58791E-12	8.55772E-12
TBCE	160.2893054	-0.28255913	0.040370973	-6.999066569	2.57673E-12	8.52221E-12
IL17RC	210.0382269	-0.371876375	0.053130043	-6.999361446	2.57132E-12	8.50573E-12
TET1	272.9253631	-0.463623342	0.066232007	-6.999989299	2.55982E-12	8.46913E-12
RYBP	2714.568635	0.301821467	0.043111508	7.000949017	2.54234E-12	8.41273E-12
ADGRF2	8.723145205	-0.854754798	0.122079756	-7.00160968	2.53038E-12	8.37456E-12
PDSS1	260.1202368	-0.220199117	0.031449103	-7.001761447	2.52764E-12	8.36691E-12
NME4	1867.634361	-0.34165129	0.04878878	-7.00266112	2.51146E-12	8.31474E-12
PDE10A	6.088040104	-0.899071717	0.128368062	-7.003858301	2.49008E-12	8.24536E-12
AGMO	4.148748576	-0.947098504	0.135209524	-7.004673011	2.47563E-12	8.19891E-12
RESF1	2980.867158	0.301350146	0.043018558	7.005119666	2.46775E-12	8.17418E-12
PLAAT5	23.71294181	0.737915815	0.105337743	7.005236646	2.46569E-12	8.16874E-12
ANKRD54	654.4230432	-0.21668912	0.030931844	-7.005373445	2.46328E-12	8.16214E-12
ZFP28	124.4410678	-0.347125699	0.049541926	-7.006705768	2.43995E-12	8.08619E-12
SLC27A1	676.3991051	-0.301176537	0.04297974	-7.00740708	2.42775E-12	8.04714E-12

ANKRD20A4P	16.46089198	-0.83525452	0.11919211	-7.007632615	2.42384E-12	8.03555E-12
CHST14	393.5171803	-0.316049025	0.045097451	-7.008134947	2.41516E-12	8.00811E-12
TSR3	326.1967477	-0.297598382	0.042463624	-7.008313346	2.41208E-12	7.99927E-12
OR52H1	13.51814258	-0.573162177	0.081781733	-7.008437623	2.40994E-12	7.99352E-12
INSM2	3.518333205	-0.680545707	0.097096983	-7.008927389	2.40152E-12	7.96694E-12
SETDB1	1716.998896	-0.126929361	0.018107577	-7.009737607	2.38765E-12	7.92229E-12
SYT7	4.439910826	-0.647930494	0.092403677	-7.01195578	2.35009E-12	7.79898E-12
KIN	636.474656	0.234729564	0.033473931	7.01230944	2.34416E-12	7.78061E-12
SCFD1	4100.01839	0.266735434	0.038036073	7.012696457	2.33768E-12	7.76042E-12
MYL4	493.3818339	-0.805180961	0.114807731	-7.013299107	2.32763E-12	7.72837E-12
MYH7B	310.2124493	-0.380305107	0.054226025	-7.013331812	2.32709E-12	7.72787E-12
VPS13B	5940.851638	-0.305225036	0.043519602	-7.013507038	2.32417E-12	7.7195E-12
APEX1	4674.370411	-0.298842714	0.042595534	-7.015822669	2.286E-12	7.594E-12
NUP62	1308.386272	-0.221752882	0.031604828	-7.016424198	2.27618E-12	7.56268E-12
CASZ1	728.3074155	0.392902667	0.055994963	7.016750221	2.27088E-12	7.54634E-12
LMO3	13.96984006	-0.709260886	0.101071088	-7.01744582	2.25961E-12	7.51015E-12
OCEL1	180.1641524	-0.339942053	0.048440172	-7.01777141	2.25435E-12	7.49395E-12
FAR2	852.0931695	-0.335579269	0.047814627	-7.018339203	2.24521E-12	7.46484E-12
SLITRK2	1.314435499	-1.353272129	0.192787063	-7.019517336	2.22636E-12	7.40342E-12
GADD45G	333.5162999	-0.646017073	0.092027214	-7.019848232	2.22109E-12	7.38717E-12
SLC31A2	180.8797616	0.41557539	0.05919471	7.020481852	2.21104E-12	7.35499E-12
ITPR3	724.3419878	0.471577252	0.067166403	7.021028844	2.2024E-12	7.32749E-12
RRAS2	240.2555719	0.533945683	0.076046755	7.021281631	2.19842E-12	7.31549E-12
RFESD	88.4869719	-0.557427607	0.079389153	-7.021458044	2.19565E-12	7.3075E-12
PEX5	741.0245279	-0.187797677	0.026741224	-7.022777801	2.175E-12	7.24001E-12
CISD3	145.7534033	-0.439267341	0.062533897	-7.024467663	2.14884E-12	7.15415E-12
ANKRD7	6.296853997	-0.866080443	0.123288246	-7.024841931	2.14309E-12	7.13621E-12
GCLM	477.7057857	-0.435771935	0.062015153	-7.026862209	2.1123E-12	7.03488E-12
DTD1	211.2010268	-0.396599721	0.056438131	-7.027158949	2.10781E-12	7.02114E-12
MYD88	2710.834087	0.318630726	0.045334185	7.028486908	2.08785E-12	6.95583E-12
MGAT3	357.5931042	-0.550203544	0.078278078	-7.028833056	2.08268E-12	6.93978E-12
IFT57	746.3757791	0.265687979	0.03779909	7.028951645	2.08091E-12	6.93507E-12
RTEL1-TNFRSF6B	193.2941807	-0.335747238	0.047765322	-7.029100222	2.0787E-12	6.92887E-12
CELF1	10941.39761	0.246676495	0.035087335	7.030357093	2.06006E-12	6.86791E-12
ANKRD26	418.1371919	-0.28677096	0.040786088	-7.031097558	2.04915E-12	6.83272E-12
HSPA2	265.1881013	-0.713568454	0.101463715	-7.032745184	2.02509E-12	6.75364E-12
GRIN1	20.31363677	-0.667936265	0.094972769	-7.032924019	2.0225E-12	6.74613E-12

STRN	2253.314717	0.200989005	0.028577696	7.033072331	2.02035E-12	6.74011E-12
SYF2	2255.620651	0.320941324	0.045622667	7.034690144	1.99704E-12	6.66351E-12
UBA1	8138.861022	-0.171329318	0.024353927	-7.034977156	1.99294E-12	6.65094E-12
CCSER1	207.1402872	-0.664189876	0.09440841	-7.035282934	1.98857E-12	6.6375E-12
CNOT9	2503.414572	0.172028225	0.024451991	7.035346284	1.98767E-12	6.63562E-12
SIK1B	475.0904384	0.524230093	0.074501193	7.036532846	1.97082E-12	6.5805E-12
TBC1D32	317.8013544	-0.373230443	0.053037994	-7.037039155	1.96368E-12	6.55776E-12
ZSCAN16	102.1906957	0.264558724	0.037594643	7.037138962	1.96227E-12	6.55419E-12
SFRP4	24.10921868	-0.516507509	0.073393492	-7.037511024	1.95704E-12	6.53783E-12
SEC16B	4.889377129	-0.749314264	0.106472359	-7.037641262	1.95521E-12	6.53284E-12
CCDC42	8.172835904	-0.581931776	0.082676337	-7.038673908	1.94078E-12	6.48572E-12
DZANK1	142.2437903	-0.285989001	0.040626753	-7.039425497	1.93034E-12	6.45194E-12
WBP1	283.8007639	-0.194467301	0.027620291	-7.040740509	1.91221E-12	6.39243E-12
MCMDC2	59.37247205	-0.354901111	0.050402527	-7.041335611	1.90406E-12	6.36627E-12
POMT1	943.0059635	-0.229084073	0.032531532	-7.041908487	1.89624E-12	6.34122E-12
ARPC2	15752.44779	0.200459013	0.02845546	7.044659025	1.85916E-12	6.21828E-12
GRHL2	4.754110919	-0.77286205	0.109687332	-7.046046567	1.84073E-12	6.15767E-12
SLC35E2B	2108.857013	-0.239319989	0.033956217	-7.047899089	1.81639E-12	6.07731E-12
LGALS9C	15.08309729	-0.472119777	0.066984977	-7.048144231	1.8132E-12	6.06765E-12
HES5	3.667932437	-0.676955815	0.096045055	-7.048315154	1.81097E-12	6.06124E-12
PIH1D2	16.83241208	-0.333853533	0.047344241	-7.051618688	1.76848E-12	5.92005E-12
MT-ND5	181239.3205	-0.742182695	0.105224186	-7.053346952	1.74665E-12	5.84795E-12
CDH12	2.291921168	-1.070166029	0.151723115	-7.05341458	1.7458E-12	5.84611E-12
PCDHGA6	125.3130171	-0.577878608	0.081907295	-7.05527643	1.72258E-12	5.76934E-12
PAX3	1.422455393	-1.271050315	0.180150875	-7.055476787	1.7201E-12	5.76202E-12
AL031777.2	98.12259024	-0.531355148	0.075297669	-7.056727721	1.70469E-12	5.71139E-12
GPR1	2.488290561	-0.750686463	0.106367023	-7.057511279	1.69511E-12	5.68026E-12
TUBB8	2.526826076	-0.832576117	0.117963451	-7.057915921	1.69018E-12	5.66472E-12
TRIM31	5.303957921	-0.75791904	0.107380784	-7.058237138	1.68628E-12	5.65261E-12
FKBP9	185.8309591	-0.441153691	0.062499032	-7.058568334	1.68227E-12	5.64012E-12
ZNF732	4.614326558	-0.727017508	0.102997197	-7.058614513	1.68171E-12	5.63921E-12
ELN	78.91925937	-0.599078711	0.08486474	-7.059218117	1.67442E-12	5.61574E-12
SLC26A5	2.196353527	-0.829921716	0.117565065	-7.059254503	1.67398E-12	5.61523E-12
USH1G	2.949061292	-0.737866452	0.104517276	-7.059755895	1.66795E-12	5.59596E-12
PPP4C	3183.998237	0.202879146	0.028728538	7.061937725	1.64197E-12	5.50972E-12
MCUR1	671.5703311	-0.244177263	0.034570684	-7.063130811	1.62792E-12	5.46354E-12
ATP23	231.4716921	-0.27638338	0.039127542	-7.0636531	1.62181E-12	5.44396E-12

DMRT2	1.100969376	-1.07465472	0.15213703	-7.063728806	1.62093E-12	5.44193E-12
CCDC22	654.6469851	-0.220984134	0.031283754	-7.063862301	1.61937E-12	5.43763E-12
KIAA0930	2858.670576	-0.399099901	0.05649567	-7.064256443	1.61478E-12	5.42315E-12
NFATC2IP	1913.537732	-0.128206001	0.018146945	-7.064880765	1.60754E-12	5.39976E-12
ZNF681	97.73467603	-0.474260772	0.067128706	-7.064947342	1.60677E-12	5.39809E-12
ARL5A	893.0671505	0.201613027	0.028528215	7.067144763	1.58154E-12	5.31424E-12
FBXO17	4.460762018	-0.670928227	0.094929241	-7.067666614	1.57561E-12	5.29521E-12
STAT5B	3747.643227	-0.238279796	0.033711026	-7.068304435	1.56838E-12	5.27184E-12
CNOT2	4938.788821	0.233776595	0.033069686	7.069211217	1.55817E-12	5.23841E-12
MPZL3	257.2111222	0.375230976	0.053078345	7.069379735	1.55628E-12	5.23295E-12
H2BC14	228.0308627	-0.615387832	0.087047145	-7.069592351	1.55389E-12	5.22584E-12
HNRNPK	15460.44361	0.152257396	0.021535035	7.070218241	1.5469E-12	5.20322E-12
CANT1	1599.489492	0.227356453	0.03215251	7.071188394	1.53612E-12	5.16785E-12
GAMT	390.2269949	-0.459583668	0.06499205	-7.07138282	1.53397E-12	5.1615E-12
PLEKHG7	8.816580222	-0.590142259	0.083451896	-7.071645894	1.53107E-12	5.15261E-12
PKLR	155.5093136	-0.73728389	0.104230483	-7.073591807	1.50974E-12	5.08171E-12
TTC7A	5348.198307	0.246814968	0.034890172	7.074054227	1.50471E-12	5.06566E-12
CTBS	301.7912988	0.222255628	0.031417657	7.074226794	1.50284E-12	5.06024E-12
RNF220	5070.539888	-0.299598366	0.042347019	-7.074839567	1.49622E-12	5.03879E-12
CLCA2	1.783367216	-0.87664083	0.123906046	-7.075044856	1.494E-12	5.03221E-12
AC131160.1	3.683558192	-0.517637559	0.073163328	-7.075095891	1.49345E-12	5.03122E-12
TPRN	310.6681363	-0.288843289	0.040823546	-7.075409184	1.49008E-12	5.02073E-12
RNF20	869.5918569	-0.166982677	0.023598383	-7.076022036	1.48351E-12	4.99945E-12
NBN	2521.851941	0.283215839	0.040023279	7.076277669	1.48078E-12	4.9911E-12
ABCA4	7.218425355	-0.685807662	0.096909944	-7.076752237	1.47572E-12	4.9749E-12
ERVK3-1	458.03032	0.244739355	0.034579214	7.077643681	1.46626E-12	4.94386E-12
YAF2	2069.492763	0.272540556	0.038497177	7.079494647	1.44681E-12	4.87912E-12
EDA	11.73667062	-0.711622683	0.100517559	-7.079585817	1.44586E-12	4.87675E-12
MROH9	1.93639153	-1.014093452	0.143230687	-7.08014096	1.44008E-12	4.85809E-12
DMAC2L	478.043118	-0.218090248	0.030798822	-7.081122994	1.42991E-12	4.82462E-12
CDNF	47.99325077	-0.291246652	0.041126996	-7.0816418	1.42456E-12	4.80742E-12
SYNDIG1	1.401267374	-1.135449151	0.160309946	-7.082836586	1.41233E-12	4.76695E-12
SEPSECS	817.0191749	0.261890883	0.036974045	7.08310061	1.40964E-12	4.7587E-12
PPP1R12C	2216.28854	-0.234451531	0.033097574	-7.083646965	1.40409E-12	4.74078E-12
SMURF1	3583.266301	0.349142535	0.049283847	7.08431978	1.39729E-12	4.71862E-12
GRIK2	3.601258235	-0.971063381	0.13706503	-7.084690965	1.39355E-12	4.70681E-12
CDH16	6.057154562	-0.653429266	0.092223553	-7.085275352	1.38768E-12	4.68779E-12

RGL1	108.0251222	0.635322565	0.089664456	7.085556447	1.38487E-12	4.6791E-12
UBXN4	5723.984342	0.209268614	0.029529752	7.086704012	1.37344E-12	4.64128E-12
NRDC	4344.448626	-0.164241472	0.023175891	-7.086738142	1.3731E-12	4.64094E-12
SPATA3	1.286181381	-0.953370289	0.134523238	-7.087030467	1.3702E-12	4.63195E-12
UBN1	4875.0425	0.225355357	0.031795871	7.087566786	1.3649E-12	4.61483E-12
CDH10	3.015908309	-1.371805724	0.193542119	-7.087892447	1.3617E-12	4.60479E-12
MEP1B	6.565754777	-0.582024314	0.082106364	-7.088662626	1.35414E-12	4.58003E-12
POT1	476.6626533	-0.235628807	0.033237968	-7.08914587	1.34942E-12	4.56485E-12
DGKG	1240.987004	-0.507481104	0.071572915	-7.090407119	1.33718E-12	4.52422E-12
NCAM1	877.1544357	-1.008288593	0.14218356	-7.091456917	1.32707E-12	4.49081E-12
DNAH2	108.6365165	-0.804088915	0.113343991	-7.09423509	1.30069E-12	4.40228E-12
CLEC4C	27.83179893	-0.803581224	0.113260295	-7.094994985	1.29356E-12	4.37891E-12
IQGAP2	3212.755951	-0.349192678	0.049215405	-7.09519061	1.29173E-12	4.37348E-12
SIRPG	44.54467541	0.572096544	0.080614988	7.096652341	1.27815E-12	4.32824E-12
CBX1	560.6034348	-0.260146879	0.036657186	-7.096749918	1.27725E-12	4.32594E-12
HTR6	21.9335726	-0.465261026	0.065553217	-7.097455307	1.27075E-12	4.30467E-12
FIBP	1194.845448	-0.311977453	0.04395385	-7.09784138	1.26721E-12	4.29341E-12
OR2T8	23.03444576	-0.570446825	0.080365148	-7.098186717	1.26404E-12	4.28344E-12
SEPHS1	709.0192085	-0.176808612	0.024905455	-7.099192214	1.25488E-12	4.25313E-12
DKC1	2146.823738	-0.170135748	0.023965099	-7.099313342	1.25378E-12	4.25014E-12
CLCA4	1.201279115	-0.805892016	0.1135143	-7.099475686	1.25231E-12	4.24588E-12
PTGFR	9.459334413	-1.071485351	0.150912491	-7.100044141	1.24717E-12	4.22919E-12
CYP3A43	6.957909542	-0.535315754	0.075369276	-7.102572623	1.22456E-12	4.15322E-12
PSKH2	0.988613088	-1.297628575	0.182684849	-7.103099052	1.2199E-12	4.13814E-12
KIAA1755	18.5189161	-0.780419614	0.109870103	-7.103111723	1.21979E-12	4.13814E-12
CASQ2	1.141719818	-1.021152443	0.143753978	-7.103472577	1.2166E-12	4.1284E-12
ARFRP1	907.5322106	-0.230432078	0.032433593	-7.104734756	1.20554E-12	4.09156E-12
UBE3A	4140.800126	0.211059014	0.029705591	7.105026652	1.20299E-12	4.08363E-12
FAM47E-STBD1	0.637452922	-1.057785877	0.148819205	-7.107858697	1.17857E-12	4.00142E-12
TMEM67	202.2295028	-0.374657662	0.052702008	-7.10898264	1.16901E-12	3.96966E-12
CIART	13.10629533	-0.459522993	0.064636556	-7.109335995	1.16603E-12	3.9602E-12
RBM33	8311.170415	0.223145411	0.03138763	7.109342435	1.16597E-12	3.9602E-12
ACSL6	243.7058323	-0.719901581	0.101248142	-7.110269533	1.15817E-12	3.93487E-12
ASB8	1135.358095	0.185612681	0.026102477	7.110922199	1.1527E-12	3.91698E-12
AADACL2	1.325129934	-0.901215273	0.126731497	-7.111217782	1.15023E-12	3.90928E-12
PNMA3	49.89831798	-0.5203017	0.073160661	-7.111768671	1.14565E-12	3.89438E-12
SPATA9	29.08574611	-0.535691573	0.075319901	-7.112218191	1.14193E-12	3.88239E-12

FLOT1	2389.399464	0.364163787	0.051202162	7.112273624	1.14147E-12	3.8815E-12
ACSM2B	3.338662284	-0.630385767	0.088629036	-7.112632527	1.1385E-12	3.87209E-12
ALPI	1.307022632	-0.927888389	0.130444814	-7.11326393	1.1333E-12	3.85508E-12
ENTPD3	14.30533748	-0.552356368	0.07762777	-7.115448137	1.1155E-12	3.79517E-12
CFAP92	198.2435078	-0.389622859	0.054756518	-7.115552135	1.11466E-12	3.79297E-12
C8orf48	1.623458705	-1.486183743	0.208858963	-7.115728809	1.11323E-12	3.78878E-12
ZNF490	309.2568776	-0.230874362	0.032438968	-7.11719202	1.10148E-12	3.74944E-12
AC011511.1	0.866796614	-0.763138968	0.107221192	-7.11742665	1.09961E-12	3.74371E-12
ACTC1	2.079842686	-0.794508024	0.111618301	-7.118080268	1.09441E-12	3.72666E-12
CTSB	9722.832725	0.374124333	0.052555782	7.118614144	1.09018E-12	3.7129E-12
TRMT10B	297.6720213	-0.208396802	0.029268609	-7.120147182	1.07812E-12	3.67247E-12
PRKCH	1580.493106	0.408809148	0.057415084	7.12023943	1.0774E-12	3.67065E-12
RIOK3	8492.652909	0.333286016	0.046807818	7.120306618	1.07687E-12	3.6695E-12
CD99L2	870.6200371	-0.285265566	0.040060008	-7.120956261	1.07181E-12	3.65288E-12
ZNF765	393.4318464	-0.231173504	0.032459223	-7.121966643	1.06398E-12	3.62683E-12
AVPR2	146.2371948	-0.355673258	0.049938475	-7.122229069	1.06195E-12	3.62056E-12
STON1	64.73366885	-0.379779165	0.053320689	-7.122547969	1.0595E-12	3.61282E-12
SFTPA1	9.740173492	-0.766886236	0.10763901	-7.124612483	1.04374E-12	3.5597E-12
ZSCAN26	221.0176803	-0.224917093	0.031562281	-7.126135663	1.03226E-12	3.52116E-12
NR1H2	3051.889348	0.229094036	0.032140825	7.12782059	1.01971E-12	3.47894E-12
APRT	2205.821968	-0.398306309	0.055876192	-7.128372537	1.01563E-12	3.46563E-12
SSH2	4634.01626	0.26161947	0.036698955	7.12879896	1.01248E-12	3.45551E-12
NDEL1	3777.141438	0.251697653	0.035305492	7.129136036	1.01001E-12	3.44766E-12
PLSCR1	1238.194342	0.371862922	0.052159038	7.129405268	1.00804E-12	3.44153E-12
S100Z	327.6829966	-0.649205799	0.091054892	-7.129828919	1.00494E-12	3.43155E-12
STRN4	3299.148852	0.214693174	0.030110952	7.130069372	1.00318E-12	3.42616E-12
AC097636.1	0.897443249	-1.283666485	0.180021012	-7.130648102	9.98974E-13	3.41238E-12
PCDHB10	4.348633608	-1.019429443	0.142950354	-7.131353049	9.9387E-13	3.39554E-12
NOVA2	6.349999202	-0.569667437	0.079874803	-7.132004328	9.89178E-13	3.38009E-12
AP5S1	349.410753	-0.247516405	0.034703006	-7.132419808	9.86195E-13	3.37049E-12
PRB4	22.89426919	-0.57525119	0.080652692	-7.132448737	9.85988E-13	3.37037E-12
MTF1	1701.103419	0.269178229	0.037738731	7.132678366	9.84344E-13	3.36534E-12
TMEM213	7.673342205	-0.597257773	0.08372361	-7.133683981	9.77175E-13	3.34141E-12
CRYBA4	1.312690942	-0.919147234	0.128846019	-7.133687517	9.7715E-13	3.34141E-12
GRAPL	5.546489252	0.558723489	0.078314347	7.13436954	9.72318E-13	3.32597E-12
ERVH48-1	66.09274671	-0.395712727	0.055460078	-7.135091483	9.67228E-13	3.30913E-12
HAX1	841.3768596	-0.317716929	0.044514422	-7.137393082	9.51176E-13	3.25478E-12

GDF7	63.14217863	-0.478789973	0.067078547	-7.137751113	9.48702E-13	3.24689E-12
TRPM3	6.36186334	-0.663683773	0.092954537	-7.139874956	9.34159E-13	3.19767E-12
ZBTB45	297.3721374	-0.252889979	0.035415732	-7.140611367	9.29167E-13	3.18114E-12
CASP14	1.696144927	-0.840011472	0.117617745	-7.141877065	9.20649E-13	3.15253E-12
MACF1	27939.84046	-0.323221139	0.045256978	-7.141907279	9.20447E-13	3.15239E-12
CSRNP1	12971.41749	0.386070783	0.054038368	7.144382671	9.04012E-13	3.09665E-12
ASB1	564.8729313	-0.272800515	0.038181952	-7.14475035	9.01596E-13	3.08891E-12
PDYN	2.539463126	-0.964225185	0.134951816	-7.144958941	9.00228E-13	3.08476E-12
ISM1	12.90043323	-0.504357862	0.070588637	-7.145029028	8.99769E-13	3.08373E-12
MXRA7	640.0422995	0.501880801	0.070238576	7.145372678	8.9752E-13	3.07656E-12
CLEC19A	1.787350306	-1.01383739	0.141885368	-7.14546824	8.96896E-13	3.07496E-12
YTHDF1	2098.438848	0.207860534	0.029084863	7.146691175	8.88946E-13	3.04824E-12
SLC8A3	128.9690285	-0.818654271	0.114547388	-7.146861075	8.87846E-13	3.045E-12
ZW10	433.3011781	-0.190133175	0.026599157	-7.148090246	8.79935E-13	3.01839E-12
PXDC1	41.85673743	0.64960507	0.090876393	7.14822686	8.7906E-13	3.01592E-12
PLEKHG2	2479.477268	0.361625915	0.050586635	7.148645322	8.76384E-13	3.00727E-12
PSMB11	2.149683415	-0.887797385	0.124170225	-7.149841145	8.68784E-13	2.98171E-12
PICK1	512.1942219	-0.261348381	0.036552196	-7.150004982	8.67748E-13	2.97868E-12
CBR1	137.1265915	-0.541073845	0.075666163	-7.150803283	8.62715E-13	2.96192E-12
HAUS6	1391.978235	0.218218251	0.030511529	7.151993339	8.55267E-13	2.93687E-12
KPNA5	777.7952762	0.273779341	0.038275956	7.15277604	8.50403E-13	2.92068E-12
KCNJ4	3.498612567	-0.857853988	0.119926191	-7.153182954	8.47885E-13	2.91254E-12
CCDC59	1264.450983	0.251068501	0.035098208	7.1533139	8.47076E-13	2.91027E-12
KCNV1	0.798195603	-1.540395033	0.215334352	-7.153503479	8.45906E-13	2.90676E-12
MYCBP2	9216.504007	-0.297390191	0.041568653	-7.154193596	8.41662E-13	2.89269E-12
STRBP	1210.775521	-0.234315273	0.032747117	-7.155294587	8.34934E-13	2.87007E-12
SLC6A4	33.07918246	-0.490333773	0.068513124	-7.156786063	8.25904E-13	2.83953E-12
ECSIT	734.7567623	-0.245894881	0.034354625	-7.157548173	8.21327E-13	2.82429E-12
RFC1	2546.798446	0.210539471	0.029414828	7.157596642	8.21037E-13	2.82378E-12
RAB3B	12.88831422	-0.568662578	0.079436222	-7.158731451	8.1427E-13	2.801E-12
ARSD	1276.852544	-0.398668857	0.055687811	-7.158996708	8.12696E-13	2.79608E-12
SPATC1L	313.4910381	-0.573504837	0.080107184	-7.159218575	8.11382E-13	2.79205E-12
PKD2	1190.870639	-0.32193157	0.044966799	-7.159317015	8.108E-13	2.79054E-12
TBC1D24	646.5664644	-0.293003735	0.040915019	-7.161275749	7.99297E-13	2.75143E-12
SSBP4	1115.614609	-0.297461958	0.041533785	-7.161927464	7.95505E-13	2.73886E-12
CD2	183.6874012	0.531793664	0.074237037	7.16345487	7.86688E-13	2.70898E-12
IL33	5.116034607	-1.0584095	0.147748667	-7.163580717	7.85965E-13	2.70697E-12

PDIA5	371.3993132	-0.373824285	0.052182001	-7.163854939	7.84394E-13	2.70203E-12
ADGRL4	10.07186239	-1.025478033	0.143144669	-7.163927495	7.83979E-13	2.70108E-12
PRPH	2.242336392	-0.942596571	0.131556641	-7.16494858	7.78158E-13	2.68149E-12
ZNF772	230.8812542	-0.376868279	0.052597257	-7.165169846	7.76902E-13	2.67764E-12
IGFBP2	950.2702767	0.788474163	0.110041158	7.165265927	7.76357E-13	2.67623E-12
MNAT1	267.8536544	-0.26657079	0.037201845	-7.165526083	7.74884E-13	2.67162E-12
LXN	283.2166322	-0.389553503	0.054357631	-7.166491533	7.69442E-13	2.65333E-12
ZMYND11	992.0297818	-0.27131055	0.037850857	-7.167883918	7.61658E-13	2.62695E-12
CLIP2	2876.660797	-0.433619195	0.060491225	-7.168299158	7.59352E-13	2.61946E-12
C9orf85	183.0385085	-0.216519946	0.030204933	-7.168363654	7.58995E-13	2.61869E-12
SNTN	1.872412717	-0.796838641	0.111144722	-7.169379048	7.53387E-13	2.5998E-12
CCL25	8.852871784	-0.719021877	0.100282654	-7.169952603	7.50237E-13	2.58938E-12
PTPRO	258.2630097	0.495116306	0.069036679	7.171786301	7.40254E-13	2.55538E-12
TTLL4	896.9784363	-0.319474409	0.04454075	-7.172632075	7.35694E-13	2.54008E-12
GOT2	1044.436127	-0.240002508	0.033455777	-7.173723964	7.29847E-13	2.52034E-12
GPR4	9.521647626	-0.619848833	0.086402357	-7.173980575	7.2848E-13	2.51606E-12
RNF180	5.943907605	-1.108523194	0.154510318	-7.174428289	7.261E-13	2.50829E-12
CCR5	95.00981307	0.693139908	0.096596832	7.175596657	7.19925E-13	2.4874E-12
TRAPPC10	4283.412636	0.213319745	0.02972549	7.176323941	7.16108E-13	2.47464E-12
RABEP2	839.849909	-0.248201841	0.034580463	-7.177516406	7.09891E-13	2.45359E-12
IDUA	734.279217	-0.329492313	0.04590461	-7.177760928	7.08623E-13	2.44964E-12
KLHDC7A	2.514450405	-0.992517439	0.138274795	-7.177862321	7.08098E-13	2.44826E-12
SMC1A	3749.087444	-0.216530461	0.030165105	-7.178176977	7.06471E-13	2.44307E-12
GOLGA6A	6.165325506	-0.673653724	0.093846715	-7.178234426	7.06174E-13	2.44247E-12
CDK13	3963.604613	-0.198246985	0.027617013	-7.178436916	7.05129E-13	2.43929E-12
PPIL2	1269.26488	-0.185062938	0.025778462	-7.178975049	7.0236E-13	2.43014E-12
CCNJ	923.3765673	0.235954318	0.032864574	7.179594594	6.99184E-13	2.41958E-12
ZAR1	12.18608839	-0.637057364	0.088727255	-7.179951213	6.97363E-13	2.4137E-12
GNPTAB	4939.68717	0.282508167	0.039345595	7.180172654	6.96234E-13	2.41022E-12
SLITRK1	2.205230702	-1.650618264	0.22986145	-7.180926861	6.92404E-13	2.39739E-12
SMIM11A	3.199674309	-0.526719075	0.073337277	-7.182146575	6.86253E-13	2.37651E-12
ARMCX2	25.97954525	-0.566481691	0.078868758	-7.182586694	6.84047E-13	2.36929E-12
CRYGD	37.77839065	-1.080917989	0.150472219	-7.183505345	6.79464E-13	2.35383E-12
TLE7	1.500395847	-1.019045862	0.141856122	-7.183657992	6.78705E-13	2.35162E-12
C16orf70	1370.521834	0.282476732	0.039310273	7.185824766	6.68027E-13	2.31503E-12
TIAM2	189.4296437	-0.414778264	0.057719995	-7.18604127	6.66969E-13	2.31177E-12
AC005324.2	2.864650939	-0.629281019	0.087566315	-7.186336614	6.65528E-13	2.30719E-12

PRH1	101.6621698	-0.389092394	0.054141472	-7.186586909	6.6431E-13	2.30337E-12
CDKN1B	2673.513995	0.249285612	0.034686423	7.186835359	6.63103E-13	2.29959E-12
RNF224	10.2366498	-0.492960988	0.068587499	-7.187329945	6.60706E-13	2.29169E-12
HSF4	104.3228599	-0.369126786	0.051343165	-7.189404611	6.50745E-13	2.25754E-12
HOPX	399.07594	-0.772123597	0.107379439	-7.190609357	6.45028E-13	2.2381E-12
ADAMTS3	197.5308644	-0.85943482	0.119501673	-7.191822487	6.39321E-13	2.21869E-12
TREX2	17.6969253	-0.401953906	0.055879748	-7.193194636	6.32926E-13	2.19689E-12
CCDC178	0.896684001	-2.154365613	0.299461092	-7.194141985	6.28548E-13	2.18208E-12
KHDRBS1	6234.970631	0.158110147	0.021975713	7.194767658	6.25672E-13	2.17248E-12
PHF6	973.8116468	-0.252985529	0.035161985	-7.194859081	6.25253E-13	2.17141E-12
RHOA	19136.69246	0.247689824	0.034422636	7.195550646	6.22092E-13	2.16082E-12
GDF11	1078.370396	-0.332808733	0.046249837	-7.19588987	6.20547E-13	2.15583E-12
RPL35	18162.85149	-0.531593467	0.07387064	-7.196275411	6.18796E-13	2.15013E-12
DEPDC1	136.1500492	-0.471473059	0.065514529	-7.196465707	6.17933E-13	2.14752E-12
ZNF84	865.6143886	-0.250393798	0.034789492	-7.197397376	6.13727E-13	2.13328E-12
CDRT1	9.373523913	-0.56041867	0.077859883	-7.197784666	6.11987E-13	2.12761E-12
PPM1H	580.2789634	-0.465672922	0.06468888	-7.198654921	6.08094E-13	2.11445E-12
ESRP2	198.7554773	-0.445244331	0.06184085	-7.199841736	6.02825E-13	2.0965E-12
C17orf97	91.80614133	-0.481991644	0.066940256	-7.200325725	6.00689E-13	2.08944E-12
KCNT1	30.46901486	-0.450151293	0.062515383	-7.20064844	5.99269E-13	2.08487E-12
MAGEH1	82.92745039	-0.402117429	0.055833991	-7.20201833	5.93277E-13	2.06439E-12
SPATA46	6.540055563	-1.091735407	0.151575616	-7.20257938	5.9084E-13	2.05628E-12
SIGIRR	1300.74148	-0.273491097	0.037961974	-7.204343432	5.83242E-13	2.0302E-12
ETV3	2363.438412	0.357732429	0.049654734	7.20439718	5.83012E-13	2.02976E-12
TBK1	2611.161937	0.285194408	0.039580098	7.205500305	5.78311E-13	2.01375E-12
MSI2	7387.390645	-0.412352688	0.057214035	-7.207194705	5.71163E-13	1.98921E-12
LGR6	45.30399599	-0.599084969	0.083121466	-7.207343626	5.70539E-13	1.98739E-12
AQP5	1.656763542	-0.961358461	0.133365847	-7.208430656	5.66004E-13	1.97195E-12
GABRQ	4.288758462	-1.656475469	0.229784837	-7.20881104	5.64425E-13	1.9668E-12
FXN	480.1219976	-0.253777317	0.035192714	-7.211075477	5.55117E-13	1.9347E-12
UBE2L6	1765.423425	-0.338967561	0.046990693	-7.213504244	5.453E-13	1.90083E-12
DGUOK	436.0123889	-0.328035078	0.045473237	-7.21380526	5.44096E-13	1.89697E-12
METTL9	3727.896145	0.283430894	0.039286441	7.214471046	5.4144E-13	1.88805E-12
NIPAL2	556.0522634	-0.402164603	0.055738187	-7.215243753	5.38375E-13	1.87769E-12
TLR9	7.877973866	-0.446614178	0.061891256	-7.216111107	5.34954E-13	1.86609E-12
C5orf24	1037.748264	0.208616233	0.028907741	7.216621758	5.32949E-13	1.85943E-12
CYTH2	1317.639294	-0.232669177	0.032239008	-7.217007883	5.31439E-13	1.85449E-12

RFC4	505.6319826	-0.220733907	0.030583762	-7.217356351	5.30079E-13	1.85008E-12
SLC30A7	1758.169742	0.233079337	0.032287406	7.218893336	5.24123E-13	1.82962E-12
TBC1D31	457.8264482	-0.324974174	0.04501154	-7.219796893	5.20653E-13	1.81783E-12
RBMS2	1990.790507	0.333311482	0.046164784	7.220037748	5.19731E-13	1.81493E-12
OASL	921.6780893	0.679534298	0.094089944	7.222177761	5.11615E-13	1.78691E-12
ASPRV1	43.9690463	-0.357873595	0.049551475	-7.222259185	5.11309E-13	1.78616E-12
HEPACAM2	83.38969277	-0.90838942	0.125767568	-7.22276364	5.09415E-13	1.77986E-12
FAM135B	4.321965904	-1.133931344	0.156981689	-7.2233351	5.07278E-13	1.77271E-12
SLC37A1	1790.981203	-0.309158127	0.04279552	-7.224076822	5.04517E-13	1.76338E-12
USP14	3356.94952	0.206887897	0.028635926	7.224767065	5.01961E-13	1.75476E-12
MEIOB	20.34113496	-0.880858509	0.121915487	-7.225156776	5.00524E-13	1.75005E-12
IL1RAP	2248.603681	0.377784692	0.052283407	7.225709216	4.98493E-13	1.74326E-12
ZNF292	6884.094571	0.313900196	0.043433603	7.227127741	4.93316E-13	1.72546E-12
BAG5	1702.319604	0.195553569	0.027057544	7.227321446	4.92613E-13	1.72331E-12
PRPF4B	5876.436326	0.229455255	0.0317473	7.227551792	4.91779E-13	1.7207E-12
PPFIBP2	586.7463173	0.574654253	0.079506508	7.227763744	4.91012E-13	1.71832E-12
WDFY4	4403.116555	-0.401723199	0.055578075	-7.228087648	4.89843E-13	1.71454E-12
OR13C8	1.01918	-1.111663718	0.153784839	-7.228695126	4.87657E-13	1.70719E-12
APBB1	303.7236432	0.556240671	0.076944317	7.229132627	4.86089E-13	1.70201E-12
CMTM5	54.09852788	-0.983408243	0.136028684	-7.229418196	4.85068E-13	1.69873E-12
PRSS55	3.798178672	-0.849991674	0.117565424	-7.229946075	4.83186E-13	1.69245E-12
SMIM4	156.6413311	-0.527213523	0.07291073	-7.230945637	4.79642E-13	1.68034E-12
SLC25A32	37.0290559	0.333901249	0.046174732	7.231254789	4.78552E-13	1.67682E-12
SAP30	713.6245438	0.531295553	0.073433651	7.235042076	4.65385E-13	1.63097E-12
PPM1A	5024.359052	0.25948296	0.035863224	7.235349549	4.64332E-13	1.62757E-12
GOLGA4	3436.524915	0.30293104	0.041866539	7.235636078	4.63352E-13	1.62443E-12
KCNK9	12.98888648	-0.67777842	0.093670975	-7.235735724	4.63012E-13	1.62353E-12
NRL	58.88513526	-0.27229827	0.037624943	-7.237174209	4.5813E-13	1.6067E-12
POLQ	819.2601893	-0.361125288	0.049894217	-7.237818547	4.55959E-13	1.59937E-12
FBXO27	6.760290321	-0.644840828	0.089089175	-7.23815019	4.54846E-13	1.59575E-12
CFAP58	13.8525013	-0.468264101	0.064688064	-7.23880226	4.52664E-13	1.58838E-12
GPS1	1798.611428	-0.20167474	0.027854911	-7.240186092	4.4807E-13	1.57254E-12
KIF12	3.024529354	-0.882449294	0.121857005	-7.241678856	4.43164E-13	1.5556E-12
PCDHA4	5.217092254	-0.953933995	0.13172814	-7.241687269	4.43137E-13	1.5556E-12
MAB21L2	39.67856489	-0.59402011	0.082020967	-7.242295862	4.41152E-13	1.5491E-12
MELK	256.7620646	-0.34917464	0.04820865	-7.242987292	4.38908E-13	1.54149E-12
OR52D1	4.03757047	-0.765856955	0.105733656	-7.243265643	4.38008E-13	1.53861E-12

NUP37	231.852573	-0.237836933	0.0328325	-7.243948259	4.35808E-13	1.53116E-12
LETM1	2359.602599	-0.218557677	0.030164982	-7.24541041	4.31132E-13	1.515E-12
KBTBD13	8.423073691	-0.813456136	0.112257702	-7.246328045	4.28222E-13	1.50504E-12
SYTL2	321.8193139	0.467476142	0.064507849	7.246810207	4.26701E-13	1.49997E-12
SELENOP	141.2230845	-0.777926947	0.107347131	-7.246835033	4.26623E-13	1.49996E-12
TMOD1	631.6821609	-0.706247277	0.097451349	-7.247178018	4.25545E-13	1.49644E-12
ERBB2	155.5303136	-0.275988782	0.038078736	-7.247845168	4.23454E-13	1.48936E-12
B3GLCT	250.0635758	0.267593132	0.036918966	7.248121022	4.22593E-13	1.48659E-12
HOXC9	2.188513596	-1.524696581	0.210356829	-7.248143969	4.22521E-13	1.48659E-12
CDKL2	18.82577662	-0.730934986	0.100840505	-7.248426438	4.21641E-13	1.48378E-12
ZNF543	296.9241806	-0.293256316	0.040452795	-7.249346233	4.18788E-13	1.474E-12
ZNF208	8.191700732	-0.617472909	0.085155636	-7.251110339	4.13369E-13	1.45519E-12
RNASEH2C	1408.479399	-0.372272005	0.051339581	-7.25116952	4.13188E-13	1.45482E-12
PKD1L3	77.68273434	-0.538075411	0.074195132	-7.252165928	4.10159E-13	1.44441E-12
CLTCL1	1098.163177	-0.647396264	0.089266409	-7.252406258	4.09432E-13	1.44211E-12
ESCO2	216.8792147	-0.418704954	0.057731826	-7.252584578	4.08893E-13	1.44047E-12
VAT1	4864.851327	0.3536501	0.048751216	7.254180118	4.04102E-13	1.42385E-12
MT-ND6	38779.48673	-0.773490083	0.106612028	-7.255185918	4.0111E-13	1.41356E-12
RGL3	12.70419721	-0.666576405	0.09186718	-7.255870999	3.99085E-13	1.40668E-12
SH3BP2	6003.486724	0.327076875	0.045071254	7.256884323	3.96108E-13	1.39644E-12
THBS3	539.2669954	-0.296968146	0.04092194	-7.256941989	3.95939E-13	1.39609E-12
ATOH8	24.41655749	0.696838468	0.096022322	7.257046658	3.95633E-13	1.39527E-12
MND1	9.299338891	-0.512588131	0.070632899	-7.257073356	3.95555E-13	1.39524E-12
IFT81	144.5629593	-0.309434734	0.042638624	-7.257146249	3.95342E-13	1.39474E-12
ZNF541	15.37383733	-0.702618157	0.096797521	-7.258637926	3.91008E-13	1.3797E-12
OR2B11	53.64178114	0.523680382	0.072135369	7.259689516	3.8798E-13	1.36926E-12
RACGAP1	767.9000697	-0.316648788	0.043616512	-7.259837561	3.87555E-13	1.36801E-12
WDR45	642.5173754	-0.182747333	0.025162197	-7.262773302	3.79233E-13	1.33888E-12
EIF4G2	29950.09998	0.214863354	0.029581843	7.263352558	3.77612E-13	1.33339E-12
ZNF674	158.2847927	-0.306735	0.042228876	-7.26363157	3.76834E-13	1.33088E-12
DUS1L	2100.381894	-0.171499367	0.023609019	-7.264146248	3.75402E-13	1.32607E-12
CREM	458.1257048	0.333988164	0.045973376	7.264816991	3.73544E-13	1.31974E-12
CNTD1	86.71034668	0.32013825	0.044066319	7.264919288	3.73261E-13	1.31898E-12
TPTE2	9.825229141	-0.550565217	0.075764107	-7.266834346	3.6801E-13	1.30066E-12
BRD8	1060.650701	-0.240607121	0.033103756	-7.268272488	3.64114E-13	1.28712E-12
CASP6	378.6449457	-0.354957366	0.048826871	-7.269713554	3.60251E-13	1.2737E-12
CDK5RAP3	345.3024484	-0.259071503	0.035634694	-7.270204328	3.58944E-13	1.26931E-12

WTIP	64.15284262	-0.493615163	0.067880296	-7.271847535	3.54604E-13	1.25418E-12
SAP30BP	3454.047614	0.213524212	0.029361449	7.272264098	3.53512E-13	1.25055E-12
CFAP157	46.84256881	-0.383189427	0.052686619	-7.272993282	3.51608E-13	1.24404E-12
TTLL2	1.672938391	-1.054706192	0.145014735	-7.273096721	3.51339E-13	1.24331E-12
MCM6	1914.124136	-0.243578449	0.033489378	-7.27330467	3.50798E-13	1.24162E-12
GUCY1A1	1196.904526	-0.621755062	0.085480673	-7.2736332	3.49945E-13	1.23883E-12
PIEZO1	8501.484182	-0.335101325	0.046059886	-7.275339788	3.4555E-13	1.22349E-12
CORT	8.556749092	-0.355132984	0.048811255	-7.275637184	3.44789E-13	1.22102E-12
LDLRAD1	2.420925088	-0.811048004	0.111452515	-7.277072239	3.41143E-13	1.20832E-12
C9orf57	1.095371172	-0.812731641	0.111657271	-7.278806233	3.36787E-13	1.19311E-12
PRR19	17.39879594	-0.645138424	0.088628572	-7.279124655	3.35993E-13	1.19051E-12
BIN1	787.3339255	0.479658817	0.06589368	7.279284107	3.35596E-13	1.18932E-12
KLF8	101.4455607	-0.537981329	0.073902587	-7.279600803	3.34809E-13	1.18675E-12
SLC34A2	6.409063535	-0.768934929	0.105622006	-7.280063682	3.33663E-13	1.1829E-12
MAPK8	1621.840762	0.216372757	0.029710877	7.282610845	3.27421E-13	1.16098E-12
AGK	677.5438184	-0.186544786	0.025613278	-7.283128276	3.26167E-13	1.15674E-12
BLVRA	493.0024009	0.502910499	0.069050018	7.283278368	3.25804E-13	1.15567E-12
NFIA	1032.576722	-0.777274081	0.106716351	-7.283551929	3.25144E-13	1.15353E-12
POLDIP3	3780.01561	0.174510674	0.023958479	7.283879456	3.24355E-13	1.15094E-12
LRRC40	805.2851965	0.237864401	0.032651984	7.284837674	3.22058E-13	1.143E-12
COL4A3	25.38552507	-0.493865938	0.067783078	-7.28597684	3.19348E-13	1.13359E-12
PPP6R2	2442.062969	-0.181612987	0.024926057	-7.286069527	3.19128E-13	1.13301E-12
WBP2NL	28.94103866	-0.459961048	0.06311645	-7.287498745	3.15762E-13	1.12127E-12
LAMC2	3.036506877	-0.838540202	0.115065533	-7.287501123	3.15757E-13	1.12127E-12
MLANA	96.53947274	-0.362224729	0.049702745	-7.287821399	3.15007E-13	1.11899E-12
ORC2	762.513663	-0.238948389	0.032783698	-7.288634388	3.13112E-13	1.11246E-12
DOK4	202.2901319	-0.448083721	0.061475272	-7.288844824	3.12624E-13	1.11093E-12
ALKBH3	322.9844994	-0.22009364	0.030193813	-7.289362177	3.11426E-13	1.10687E-12
GPBP1	4441.443131	0.232890048	0.031947779	7.289710209	3.10622E-13	1.10422E-12
PNPLA4	141.9458324	-0.386037054	0.052952345	-7.29027305	3.09327E-13	1.09981E-12
EPHA4	124.6941356	0.611172411	0.083824266	7.291115569	3.07399E-13	1.09315E-12
WNT9A	24.1560523	0.965474816	0.132393925	7.292440482	3.0439E-13	1.08265E-12
AC004922.1	84.52361181	0.337880809	0.046332207	7.29256876	3.041E-13	1.08182E-12
MYOC	3.441108515	-1.059429268	0.1452613	-7.293265791	3.0253E-13	1.07643E-12
TTLL6	42.29280778	-0.733758752	0.100604427	-7.293503621	3.01997E-13	1.07472E-12
ZFP36L1	3458.737518	0.564150748	0.0773446	7.293990097	3.00908E-13	1.07104E-12
FAF1	2160.207737	-0.200873594	0.027539411	-7.294041085	3.00794E-13	1.07083E-12

FADS1	1514.235415	-0.337824402	0.046314554	-7.294130447	3.00594E-13	1.07032E-12
TRIM44	2342.147844	-0.257276051	0.035270953	-7.294275498	3.0027E-13	1.06936E-12
NKX6-3	5.014350929	-0.886566558	0.12153627	-7.294666497	2.994E-13	1.06645E-12
SRARP	2.584971264	-0.752325175	0.103128187	-7.295048989	2.9855E-13	1.06362E-12
CSRNP2	484.3585251	-0.261882221	0.035898433	-7.295087891	2.98464E-13	1.06351E-12
QSOX1	6080.830071	0.292321221	0.040070478	7.295176783	2.98267E-13	1.063E-12
CDH2	20.42603356	-1.143089997	0.156684245	-7.295500553	2.97551E-13	1.06064E-12
EXD2	339.2275382	-0.317096301	0.043461339	-7.29605463	2.96329E-13	1.05647E-12
ATXN10	1749.309085	-0.208813462	0.028617656	-7.296665474	2.94987E-13	1.05188E-12
CLCN2	132.2646333	-0.375602642	0.051465011	-7.29821361	2.91613E-13	1.04004E-12
ZSCAN10	9.931482199	-0.444803435	0.060944964	-7.298444411	2.91114E-13	1.03845E-12
MASTL	785.3611816	0.232970857	0.031912194	7.300371061	2.86975E-13	1.02387E-12
MYOCOS	9.954218636	-0.807492295	0.110608258	-7.300470257	2.86764E-13	1.0233E-12
R3HDM2	1085.06342	-0.195610599	0.026791835	-7.301127211	2.85367E-13	1.01851E-12
TGM5	140.3159958	-0.799690949	0.10952362	-7.301538698	2.84495E-13	1.01558E-12
NCOA5	594.5344181	-0.234008414	0.032047392	-7.301948687	2.83629E-13	1.01267E-12
G3BP2	5623.943576	0.158965117	0.021769952	7.302042476	2.83432E-13	1.01215E-12
ZFR	6146.044795	0.214578107	0.029385888	7.302080073	2.83353E-13	1.01205E-12
STOML2	1450.802737	-0.270316506	0.037017173	-7.30246218	2.82549E-13	1.00937E-12
ZNF302	402.5689295	-0.26778221	0.036669939	-7.302499429	2.82471E-13	1.00927E-12
IZUMO1	15.34115919	-0.656333102	0.089873144	-7.30288353	2.81665E-13	1.00658E-12
MATN3	2.832462233	-0.780529022	0.106865707	-7.303830594	2.79688E-13	9.99697E-13
PDP2	284.6542934	-0.368762118	0.050486813	-7.304127516	2.79072E-13	9.97675E-13
SERTAD3	648.9293903	0.305083111	0.041757432	7.306079353	2.7505E-13	9.83476E-13
DPM2	866.7458284	-0.438658242	0.060029045	-7.307433267	2.72293E-13	9.73799E-13
EHD3	369.2029294	-0.522051273	0.071440733	-7.307473654	2.72212E-13	9.73684E-13
ACSM4	5.285469864	-0.949345515	0.129899262	-7.308321056	2.70501E-13	9.67741E-13
SEMA6D	7.442655885	-1.013220679	0.138634645	-7.308567635	2.70005E-13	9.66144E-13
EFCAB9	1.567916321	-0.745297954	0.101965994	-7.309279524	2.68579E-13	9.61216E-13
RALA	1210.062717	0.282913637	0.038705757	7.309342625	2.68452E-13	9.6094E-13
PTPRE	8900.741027	0.382367785	0.052308138	7.309910144	2.67321E-13	9.57065E-13
LDHB	442.1012348	-0.411834268	0.056335295	-7.310412947	2.66323E-13	9.53665E-13
TMEM9B	1040.957355	0.28081141	0.038408457	7.31118691	2.64793E-13	9.48361E-13
GPALPP1	679.7958108	-0.167694925	0.02293422	-7.311995882	2.63203E-13	9.4284E-13
KLHL41	3.11506943	-0.608920079	0.083267687	-7.312801613	2.61629E-13	9.37373E-13
SLC15A4	2023.178073	0.347703909	0.047544869	7.313174258	2.60904E-13	9.34947E-13
BTK	2591.503309	-0.31974321	0.043715601	-7.314167101	2.58983E-13	9.28231E-13

MOB3A	4439.753121	0.318553117	0.043547636	7.315049518	2.57286E-13	9.2232E-13
SLC19A2	313.6845351	0.355933609	0.048656782	7.315190128	2.57017E-13	9.21524E-13
FCAMR	5.428018993	-0.826025059	0.112903859	-7.316180938	2.55128E-13	9.14916E-13
CEACAM19	90.64944474	-0.548379088	0.074948617	-7.316733916	2.54079E-13	9.11323E-13
PCDHGA7	58.86999941	-0.692151171	0.094587158	-7.317601928	2.52441E-13	9.05615E-13
ACTL9	0.998324296	-0.956230099	0.130675195	-7.317609918	2.52426E-13	9.05615E-13
DHDDS	826.5902571	-0.15182619	0.020747905	-7.317663552	2.52326E-13	9.05531E-13
MIIP	889.6655969	-0.264382242	0.036128304	-7.31787036	2.51937E-13	9.04303E-13
GEMIN4	920.4935525	-0.212319402	0.029010149	-7.318797337	2.50203E-13	8.98244E-13
ENG	1227.70149	-0.342697405	0.046805413	-7.321747194	2.44763E-13	8.78875E-13
BMP10	13.2505906	-0.681776715	0.093107623	-7.322458572	2.43468E-13	8.74387E-13
GIPC1	216.8681293	-0.381619386	0.052110263	-7.323305713	2.41936E-13	8.69042E-13
CCDC151	6.868753904	-0.543476296	0.074207341	-7.323753765	2.41129E-13	8.66303E-13
TCTN2	177.1578785	-0.267460082	0.036516956	-7.324271057	2.40201E-13	8.63126E-13
ZNF619	373.9793568	-0.262416425	0.03582222	-7.325521089	2.37972E-13	8.55275E-13
CDC5L	2510.864769	0.226713981	0.030941118	7.327271862	2.34885E-13	8.44335E-13
SMC5	2501.518762	0.241598768	0.032972522	7.327275978	2.34878E-13	8.44335E-13
TBC1D10C	1357.527076	-0.283683404	0.038707338	-7.328930794	2.31996E-13	8.34257E-13
WBP11	4742.938923	0.2910198	0.03970781	7.329031684	2.31822E-13	8.33782E-13
PHYHIP	10.81051757	-0.656854334	0.089622835	-7.329095669	2.31711E-13	8.33538E-13
ENPP5	8.004678448	-0.726716471	0.099154455	-7.329135876	2.31641E-13	8.33441E-13
BHMG1	3.263231557	-0.878054066	0.119800462	-7.32930451	2.3135E-13	8.32546E-13
WDR25	151.9139977	-0.22156056	0.030227643	-7.329733217	2.30611E-13	8.3004E-13
BRIP1	585.8516757	-0.319791047	0.043627755	-7.32999088	2.30168E-13	8.28598E-13
POU1F1	2.807081875	-0.982817373	0.134072282	-7.33050382	2.29289E-13	8.25584E-13
SNCG	6.196657067	-0.74094369	0.101076625	-7.33051473	2.2927E-13	8.25584E-13
SH2B1	1699.967157	-0.172980589	0.023593308	-7.331765036	2.27141E-13	8.18151E-13
SYT6	6.680531734	-0.879924765	0.119976438	-7.334146425	2.23139E-13	8.03884E-13
FAM43B	1.396831575	-0.934015555	0.12729064	-7.337660929	2.17359E-13	7.83205E-13
GORAB	264.4857135	0.294837662	0.040177646	7.33835076	2.16242E-13	7.79323E-13
LTB4R	1197.183568	-0.279308588	0.038060283	-7.338584078	2.15865E-13	7.78109E-13
C1QL4	2.591152055	-0.714558055	0.097368654	-7.33868682	2.157E-13	7.77655E-13
MXD4	3497.534709	0.283944606	0.038688551	7.339241103	2.14808E-13	7.74584E-13
MT-CO2	216793.9221	-0.801695448	0.109232482	-7.339350316	2.14633E-13	7.74095E-13
VPS36	1607.150534	-0.190859213	0.026002482	-7.34003819	2.13533E-13	7.70269E-13
NIBAN1	2963.271577	0.395107213	0.053818724	7.341445211	2.113E-13	7.62354E-13
ANAPC11	1035.410867	-0.453078865	0.06170872	-7.342217885	2.10083E-13	7.58104E-13

CELF5	15.7619417	-0.628221553	0.085560511	-7.342424033	2.0976E-13	7.57076E-13
RELL1	1962.012631	0.323586675	0.044069986	7.342563518	2.09541E-13	7.56427E-13
BBS2	550.9646646	-0.270766215	0.036875125	-7.34278764	2.0919E-13	7.553E-13
APOH	1.994340159	-0.831034323	0.113175127	-7.342906025	2.09005E-13	7.54771E-13
NPR1	10.80424347	-0.656256234	0.089370394	-7.343105531	2.08694E-13	7.53786E-13
ZNF644	4062.035396	0.306574618	0.041746666	7.343690994	2.07783E-13	7.50633E-13
GGACT	48.21415844	-0.407260751	0.055454641	-7.344033679	2.07251E-13	7.4885E-13
NUDT17	143.0896242	-0.217001351	0.029543601	-7.345121922	2.05572E-13	7.4292E-13
POC5	298.0843215	-0.256345314	0.034894383	-7.346320227	2.03738E-13	7.36429E-13
CCDC194	53.75096601	-0.559265734	0.076128271	-7.346360686	2.03676E-13	7.36342E-13
IFIT5	643.6914976	0.393346583	0.0535383	7.347012903	2.02685E-13	7.32895E-13
SCN8A	76.65313777	-0.635465069	0.086491248	-7.347160357	2.02462E-13	7.32222E-13
HPCA	3.200098814	-0.636502851	0.086619248	-7.348284237	2.00767E-13	7.26227E-13
OFD1	1698.227964	-0.276669678	0.037645801	-7.349283894	1.99272E-13	7.2095E-13
TIPIN	146.9186188	-0.261589688	0.035589243	-7.350245972	1.97842E-13	7.15912E-13
PTMS	840.6329235	-0.519565012	0.070685572	-7.350368634	1.97661E-13	7.15388E-13
MBOAT1	813.3664941	-0.349047788	0.047477106	-7.351918009	1.95383E-13	7.07273E-13
TPX2	1533.677497	-0.332636949	0.045244725	-7.351949796	1.95336E-13	7.07235E-13
MCRS1	1243.11558	-0.191238255	0.026011888	-7.351955845	1.95327E-13	7.07235E-13
ROBO3	271.2316336	-0.543789971	0.073948541	-7.353626748	1.929E-13	6.98673E-13
RHBDD1	753.7257235	-0.237464616	0.032285223	-7.355210737	1.90626E-13	6.90565E-13
AL451007.3	1.948427536	-0.786714507	0.106946868	-7.356124766	1.89326E-13	6.85983E-13
COMP	9.513512653	-0.518538887	0.070484486	-7.356780425	1.88399E-13	6.8275E-13
TP53RK	336.96152	-0.280939313	0.038183841	-7.357544597	1.87324E-13	6.7898E-13
DIRAS2	1.861767622	-1.015933186	0.138059742	-7.358649054	1.85781E-13	6.73511E-13
PIGO	922.2513203	-0.167934459	0.022819573	-7.359228757	1.84976E-13	6.70717E-13
GYG1	2044.20781	0.33401391	0.045378425	7.360632573	1.83041E-13	6.63824E-13
PDE1A	6.993583329	-0.873619935	0.118627327	-7.364407162	1.77936E-13	6.4543E-13
NIPAL1	14.10855374	-0.619421722	0.084104258	-7.364927045	1.77244E-13	6.43038E-13
TTC23	75.76147484	-0.531261	0.072112066	-7.367158265	1.74304E-13	6.32488E-13
GRID2	3.980723437	-1.789535066	0.242900156	-7.367368946	1.74028E-13	6.31607E-13
BLACAT1	3.708142837	-0.710309696	0.096389899	-7.369129994	1.71745E-13	6.23436E-13
SLC34A1	7.566477509	-0.735854124	0.099851267	-7.369502118	1.71266E-13	6.21814E-13
GSX1	1.184036869	-1.122004747	0.152243703	-7.369794106	1.70892E-13	6.20569E-13
H2BS1	9.103238703	0.658046746	0.089284118	7.370255323	1.70302E-13	6.1854E-13
WRAP73	697.8304539	-0.24084443	0.032667188	-7.372670908	1.67243E-13	6.07544E-13
PYCARD	757.3208183	-0.520347116	0.070577793	-7.372674797	1.67238E-13	6.07544E-13

UBA2	3613.749265	0.144080757	0.019538662	7.374136281	1.65414E-13	6.01123E-13
MAGEA10	0.894909996	-1.46919386	0.199218705	-7.374778699	1.64618E-13	5.98342E-13
SLC9A9	195.6400933	-0.660770198	0.089580651	-7.376260245	1.62798E-13	5.91835E-13
TLE6	13.28902097	-0.684316126	0.092772273	-7.376300075	1.62749E-13	5.91768E-13
ANAPC4	844.2381828	-0.21909984	0.029698366	-7.377504811	1.61284E-13	5.86548E-13
IL7	28.29555849	-0.776781782	0.105289477	-7.377582298	1.6119E-13	5.86316E-13
ZNF805	1430.521592	0.254257144	0.034462863	7.377713942	1.6103E-13	5.85846E-13
SPARCL1	13.13422755	-0.650157149	0.088118451	-7.378218084	1.60422E-13	5.83741E-13
SMOC2	3.132491234	-1.187589369	0.16093218	-7.379440007	1.58957E-13	5.78516E-13
ZXDA	288.9948883	0.286356053	0.038800424	7.380229998	1.58016E-13	5.75201E-13
TDO2	3.844698199	-0.905530899	0.122696483	-7.380251468	1.57991E-13	5.75201E-13
ZNF32	184.256907	-0.332224824	0.045013001	-7.380641461	1.57529E-13	5.73639E-13
LYPD6	3.973847402	-1.207722281	0.163607211	-7.381840149	1.56117E-13	5.68602E-13
MYPN	3.911238388	-0.681299801	0.092289909	-7.382170011	1.5573E-13	5.67301E-13
HMMR	275.6541617	-0.395657005	0.05359604	-7.382206009	1.55688E-13	5.67253E-13
TLX2	12.81563278	-0.576394762	0.078075203	-7.382558619	1.55276E-13	5.65857E-13
DNAJC12	53.6334097	-0.790891919	0.10712816	-7.382670636	1.55145E-13	5.65487E-13
NRG3	1.404729098	-1.284345497	0.173944586	-7.383647441	1.54011E-13	5.61456E-13
BMP2	81.09753804	-0.932104723	0.126233935	-7.383947297	1.53664E-13	5.60296E-13
EVI2A	657.0164608	0.38083744	0.051558303	7.386539514	1.50699E-13	5.49588E-13
RAD21	6706.955306	0.20969524	0.028380125	7.388805963	1.48153E-13	5.40403E-13
KLF6	29887.44478	0.362417757	0.049043251	7.389758008	1.47096E-13	5.36648E-13
CACNA1I	62.09396828	0.529659762	0.071658175	7.391477119	1.45207E-13	5.29852E-13
CPNE4	8.440479289	-0.82636101	0.11175353	-7.394495843	1.41946E-13	5.18051E-13
CITED1	1.629136973	-0.689862672	0.093284965	-7.395218256	1.41176E-13	5.15338E-13
PLAU	705.4041353	0.522838677	0.070691307	7.396081663	1.40262E-13	5.12095E-13
RPF1	890.8012993	0.273451865	0.036970623	7.396463599	1.39859E-13	5.10721E-13
MINDY1	1065.265087	-0.295953851	0.040012562	-7.396523353	1.39796E-13	5.10586E-13
CUTA	1571.904377	-0.38348546	0.051816421	-7.400848012	1.35318E-13	4.94321E-13
GUCY2C	6.907572907	-0.810051598	0.109446	-7.401381466	1.34775E-13	4.9243E-13
ARHGEF26	45.33492233	-0.601016241	0.081202485	-7.401451328	1.34704E-13	4.92263E-13
KIAA0040	610.2575805	0.334928869	0.045251496	7.401498262	1.34656E-13	4.92181E-13
DAAM1	579.0088634	-0.384178039	0.05190327	-7.401807991	1.34343E-13	4.91126E-13
ADAP2	316.8608651	0.55080315	0.074408053	7.402466877	1.33678E-13	4.88786E-13
RANBP1	1573.688033	-0.361166883	0.048788661	-7.402680759	1.33462E-13	4.8809E-13
SPECC1L	718.297769	-0.193154888	0.026088258	-7.403901211	1.32241E-13	4.83713E-13
WDTC1	2588.154468	-0.262692161	0.035478543	-7.40425447	1.31889E-13	4.82518E-13

AMPD1	9.904658037	-0.763022018	0.103041036	-7.405030568	1.3112E-13	4.79794E-13
PCK1	3.059448835	-1.008464579	0.136175078	-7.405647178	1.30512E-13	4.77659E-13
TTR	2.271645617	-0.951671895	0.128491962	-7.406470286	1.29705E-13	4.74794E-13
RWDD2B	207.7880196	-0.309169267	0.041733602	-7.408161657	1.28062E-13	4.68867E-13
ERVV-2	1.831600538	-0.787952778	0.106359687	-7.40837809	1.27853E-13	4.6819E-13
OXSR1	2296.742525	0.207253025	0.027967093	7.41060311	1.25726E-13	4.60487E-13
GRPEL1	804.4339533	0.291174815	0.039291038	7.410718276	1.25617E-13	4.60173E-13
MRPS23	450.5754893	-0.305754907	0.041253968	-7.41152723	1.24853E-13	4.5746E-13
GAS6	336.7733518	-0.462435959	0.062392753	-7.411693452	1.24697E-13	4.56972E-13
PLA2G4E	3.775279246	-0.720685656	0.097232536	-7.411980419	1.24427E-13	4.5607E-13
IDE	1189.130934	-0.221279261	0.029853322	-7.412215606	1.24207E-13	4.55347E-13
COQ6	196.8894331	-0.193062307	0.026042304	-7.413411163	1.23092E-13	4.51343E-13
MAD2L1BP	663.6159902	0.280033327	0.037770627	7.41405025	1.22499E-13	4.49257E-13
SELENOS	832.4634528	0.270148575	0.03642698	7.41616719	1.20558E-13	4.4222E-13
TNFSF4	180.8714605	-0.555238535	0.074858168	-7.417207119	1.19616E-13	4.38845E-13
CA8	99.54951433	-0.744960207	0.100430375	-7.417678255	1.19191E-13	4.3737E-13
FAM189B	520.3659955	-0.369523907	0.049802923	-7.419723264	1.17365E-13	4.3075E-13
AK2	5016.38925	-0.345795958	0.046604609	-7.419780231	1.17315E-13	4.30646E-13
GRM1	3.87900038	-0.697517955	0.094004862	-7.420020012	1.17103E-13	4.29947E-13
TSPYL6	20.32362803	-0.527764565	0.071124804	-7.420260417	1.1689E-13	4.29248E-13
CYTH3	727.7569151	0.348310738	0.046936641	7.420870635	1.16353E-13	4.27355E-13
ZNF664	1058.525765	-0.296097181	0.039895723	-7.421777572	1.15559E-13	4.24518E-13
ALPK1	1279.750704	-0.33490324	0.0451242	-7.421809991	1.1553E-13	4.24494E-13
RELT	4968.792939	0.369852683	0.049825009	7.42303295	1.14468E-13	4.2067E-13
CHUK	1321.921294	0.220470101	0.029698921	7.423505324	1.14061E-13	4.1925E-13
CEP120	1105.84855	0.212396819	0.028610892	7.423635088	1.13949E-13	4.18918E-13
MKRN3	163.1339401	-0.851924569	0.114752202	-7.424036811	1.13604E-13	4.17727E-13
NDST2	129.0500697	0.321856117	0.043337356	7.426759355	1.11291E-13	4.09299E-13
OPA1	4187.234734	0.25398228	0.034196686	7.427102102	1.11003E-13	4.08317E-13
KIAA1841	290.5494802	-0.301054098	0.040533636	-7.427266021	1.10865E-13	4.07888E-13
KDM3A	6338.74536	0.268413363	0.036131428	7.428805906	1.09582E-13	4.03244E-13
DTWD2	180.3605091	-0.347927796	0.046832818	-7.429144921	1.09302E-13	4.02288E-13
APBA3	410.9596776	-0.220906485	0.029734677	-7.42925468	1.09211E-13	4.0203E-13
VPS8	2182.073361	-0.302093117	0.040651498	-7.431291197	1.07543E-13	3.95962E-13
NUDT1	496.969697	-0.429109797	0.057733335	-7.432617522	1.06469E-13	3.92084E-13
ITGA2	81.52768049	-0.662423951	0.089116962	-7.433197165	1.06004E-13	3.90442E-13
PHTF2	1156.505766	0.247189399	0.033254214	7.433325663	1.05901E-13	3.90136E-13

HSPE1	444.0471559	-0.46749718	0.062890771	-7.433478255	1.05779E-13	3.8976E-13
STX1A	475.9882348	0.604613328	0.081325357	7.434499567	1.04965E-13	3.86833E-13
MPND	244.9892497	-0.282626861	0.038012977	-7.435009859	1.0456E-13	3.85415E-13
HOXB1	2.806678528	-1.093808119	0.147098275	-7.435900392	1.03858E-13	3.829E-13
DHRS4	138.407745	-0.337812519	0.045428184	-7.436188041	1.03632E-13	3.82139E-13
COQ7	279.2257491	-0.251011157	0.033753877	-7.436513279	1.03378E-13	3.81272E-13
TRIM10	568.8954125	-0.850694824	0.114358032	-7.438872533	1.01548E-13	3.74596E-13
DCDC2C	0.827136482	-1.466411757	0.197094816	-7.440133581	1.00584E-13	3.71107E-13
DECR2	274.2649564	-0.277439816	0.03728814	-7.440430495	1.00358E-13	3.70343E-13
PEX7	153.9726932	-0.210625874	0.028307636	-7.440602765	1.00227E-13	3.6993E-13
RABGGTB	3385.074616	0.229682385	0.030866119	7.441245904	9.97401E-14	3.68203E-13
SLC24A1	878.3164082	-0.320607845	0.043081919	-7.441819053	9.93082E-14	3.66678E-13
RND3	14.09049679	-1.066887458	0.143336002	-7.443262302	9.82288E-14	3.62761E-13
RAF1	4363.044088	0.241997865	0.032509151	7.443992122	9.76873E-14	3.60829E-13
FHOD3	35.73465239	-0.578632213	0.077719886	-7.445098584	9.68721E-14	3.57885E-13
DYRK3	808.4000843	-0.531398168	0.071372636	-7.445404823	9.66476E-14	3.57124E-13
NNMT	38.67911623	-0.669800981	0.089955471	-7.445917105	9.62732E-14	3.55807E-13
POLM	1025.758206	-0.248764965	0.03340654	-7.446594663	9.57803E-14	3.54053E-13
GRB2	7206.621243	0.210286823	0.02823881	7.446730939	9.56815E-14	3.53754E-13
ELK3	1599.339988	0.257481751	0.03457451	7.447155448	9.53742E-14	3.52685E-13
ADD2	1107.365484	-0.676655512	0.090858616	-7.447345576	9.52369E-14	3.52243E-13
APOL1	358.2706498	-0.370703861	0.049769565	-7.448404711	9.44756E-14	3.49494E-13
RBM48	774.5258289	0.228760451	0.030711383	7.448718571	9.42512E-14	3.48729E-13
FAM166A	272.0023476	-0.763490328	0.102487298	-7.44960928	9.36171E-14	3.46449E-13
SYT3	7.382202929	-0.833867753	0.111922968	-7.450372075	9.30774E-14	3.44516E-13
KLRB1	115.6965594	0.588376012	0.078946189	7.452874147	9.13284E-14	3.38107E-13
COMTD1	156.8586829	-0.37135682	0.049824206	-7.453341398	9.10054E-14	3.36975E-13
AASDH	361.9030509	-0.210180807	0.028193961	-7.454816551	8.9993E-14	3.33289E-13
C5orf47	6.663347059	-0.890751643	0.119475637	-7.455508618	8.95218E-14	3.31607E-13
CIAO1	2247.910348	-0.116512446	0.015626578	-7.456043542	8.91593E-14	3.30326E-13
ARL16	400.123324	-0.20511742	0.027508604	-7.456482407	8.8863E-14	3.29291E-13
KIAA0408	1.772297013	-0.9088001	0.121875543	-7.456788096	8.86571E-14	3.2859E-13
UBAC1	1478.237452	-0.353477617	0.047402549	-7.456932754	8.85599E-14	3.28292E-13
ZNF546	273.1062933	-0.412976294	0.055375803	-7.457703032	8.80438E-14	3.26441E-13
PIBF1	610.111418	-0.268788056	0.036036201	-7.458834411	8.72912E-14	3.23712E-13
TNFAIP2	5645.481646	0.494516966	0.066290295	7.459869741	8.66081E-14	3.21239E-13
RAB12	457.740752	0.322334959	0.043202794	7.460974858	8.58846E-14	3.18616E-13

AL441992.2	12.85282147	-0.438456321	0.058765748	-7.461086368	8.5812E-14	3.18407E-13
FRS3	144.9816605	-0.281026123	0.037658587	-7.46247126	8.49145E-14	3.15137E-13
CRYAB	6.219091638	-0.700050148	0.093807741	-7.462605302	8.48282E-14	3.14876E-13
GIMAP7	79.24203157	0.615258419	0.082422297	7.464708445	8.34843E-14	3.09946E-13
COQ10A	157.5278222	-0.24061334	0.032230024	-7.465502945	8.2982E-14	3.0814E-13
TMEM254	159.4600913	-0.33536153	0.044918381	-7.466019955	8.26568E-14	3.06991E-13
CYB5R4	2241.73746	0.231409612	0.030992958	7.466522216	8.23421E-14	3.0588E-13
GPC2	185.6987206	-0.455126636	0.060952796	-7.466870502	8.21245E-14	3.0513E-13
FOXE3	2.327210616	-0.821671974	0.110035442	-7.467339228	8.18326E-14	3.04103E-13
PHF1	3216.265905	0.28872317	0.038663376	7.467614076	8.16619E-14	3.03526E-13
ERG28	405.746248	-0.362774235	0.048578387	-7.467811395	8.15396E-14	3.03129E-13
SPACA7	0.789171555	-1.322789091	0.17711435	-7.468559648	8.10774E-14	3.01468E-13
SUPT6H	7585.583177	0.216432893	0.028978163	7.468827137	8.09127E-14	3.00913E-13
PRADC1	151.9763663	-0.414572366	0.055494247	-7.470546784	7.98623E-14	2.97063E-13
OBSL1	623.0124015	-0.658088687	0.088086646	-7.470924593	7.96333E-14	2.96267E-13
TSG101	1194.22611	0.25138344	0.033644024	7.47186013	7.9069E-14	2.94224E-13
TTC25	821.2423891	0.467635957	0.062572964	7.47345057	7.81188E-14	2.90743E-13
CH25H	13.73449369	1.062519982	0.142161971	7.474009899	7.77873E-14	2.89565E-13
FAM111A	1448.806601	-0.334325156	0.044721121	-7.475777611	7.67487E-14	2.85753E-13
CNTNAP3	52.97095418	-0.979928002	0.131073225	-7.476187479	7.65098E-14	2.84917E-13
LILRA5	377.4679358	0.750667223	0.100397965	7.476916738	7.60866E-14	2.83395E-13
SSBP3	1830.676505	-0.296859759	0.039700235	-7.477531468	7.57316E-14	2.82127E-13
PRPSAP2	655.9647102	-0.181129114	0.024221711	-7.47796544	7.5482E-14	2.81251E-13
SCAPER	787.8921418	-0.299315679	0.040024146	-7.478377711	7.52457E-14	2.80423E-13
SPG11	4227.174428	-0.244945281	0.032747735	-7.479762609	7.4457E-14	2.77537E-13
OPRK1	3.562286836	-0.863476992	0.115426994	-7.480719718	7.39167E-14	2.75575E-13
FBXL15	342.2395837	-0.264941597	0.035406302	-7.482893763	7.27037E-14	2.71105E-13
TIMP1	5327.678885	0.645923225	0.086287235	7.485733282	7.11488E-14	2.65357E-13
SLC6A14	1.106571607	-1.685222962	0.225115956	-7.486021829	7.09926E-14	2.64825E-13
GID8	3034.529618	0.214176241	0.02860992	7.486083301	7.09594E-14	2.64752E-13
DNAJC28	33.81100349	-0.36288074	0.048473999	-7.486090512	7.09555E-14	2.64752E-13
SUGP1	795.6684517	-0.145238071	0.019399431	-7.486718104	7.06171E-14	2.63575E-13
STMN4	2.767044868	-0.699447341	0.093392775	-7.48930888	6.92372E-14	2.58474E-13
PSG3	1.280020889	-1.219636735	0.162844199	-7.489592781	6.90876E-14	2.57965E-13
AHCTF1	4897.31218	0.321225642	0.042885815	7.490253826	6.87405E-14	2.56718E-13
SMTN	399.8050069	-0.373798672	0.049904378	-7.490298229	6.87173E-14	2.5668E-13
SKIV2L	2161.119297	-0.205890455	0.027487281	-7.490390104	6.86692E-14	2.56549E-13

THRB	43.276845	-0.68915983	0.091995679	-7.491219572	6.82365E-14	2.54982E-13
RIPPLY3	5.274959757	-0.7357327	0.098209232	-7.491482068	6.81002E-14	2.54521E-13
ANKIB1	1197.421924	-0.23342735	0.031151064	-7.493398983	6.71125E-14	2.50877E-13
ATG3	2107.087744	0.230742496	0.030790941	7.493843586	6.68854E-14	2.50076E-13
DDX19B	561.7702351	0.177818022	0.023727341	7.494224501	6.66915E-14	2.49399E-13
CRYGS	52.77966168	-0.308033852	0.041101496	-7.494468049	6.65678E-14	2.48984E-13
NDUFS8	1331.168586	-0.371469906	0.049544069	-7.497767421	6.49139E-14	2.42844E-13
SLC30A3	26.61725259	-0.639440578	0.08527991	-7.498138487	6.47305E-14	2.42204E-13
DAPK1	2371.774747	-0.450826109	0.060124055	-7.49826523	6.46679E-14	2.42016E-13
CAB39	3024.929461	0.199231015	0.026569989	7.498347727	6.46273E-14	2.4191E-13
DEDD	793.0103472	0.208125038	0.0277521	7.499433769	6.40941E-14	2.39961E-13
TMEM135	513.3899887	-0.265439327	0.035392667	-7.499839681	6.38959E-14	2.39264E-13
DYNLT1	1252.085	0.298499019	0.039800325	7.499914167	6.38596E-14	2.39174E-13
POLE3	2577.263158	0.235719215	0.03142185	7.501761204	6.2966E-14	2.35872E-13
PLS1	73.9153623	-0.641705371	0.085534236	-7.502321874	6.26972E-14	2.3491E-13
PHF12	5995.709895	0.258095091	0.034401207	7.502501084	6.26115E-14	2.34634E-13
TNFSF18	2.363321614	-0.985441579	0.131336455	-7.503183953	6.2286E-14	2.33459E-13
FOXA3	2.679519635	-0.783593723	0.104432061	-7.50338276	6.21916E-14	2.3315E-13
SETD7	1000.332649	0.448153457	0.05972444	7.503686239	6.20477E-14	2.32655E-13
TNFAIP8	1592.430371	0.323947431	0.043168556	7.504245208	6.17836E-14	2.31709E-13
PGK1	11874.68459	0.200819415	0.026758371	7.504919414	6.14664E-14	2.30564E-13
IL17D	17.15746614	-0.438628999	0.058442285	-7.505336265	6.12711E-14	2.29875E-13
EIF1AD	896.1789661	0.229776122	0.030612358	7.505992308	6.0965E-14	2.28771E-13
PPT2	271.4069867	-0.381278422	0.050788442	-7.507188764	6.04106E-14	2.26734E-13
COPS7A	634.9005858	-0.260870806	0.034748831	-7.50732606	6.03473E-14	2.2654E-13
HSD17B11	3535.582458	0.310646137	0.041372675	7.508485737	5.98152E-14	2.24585E-13
JRK	946.0891227	-0.363100033	0.048346023	-7.510442606	5.89278E-14	2.21296E-13
RBM47	1131.630659	0.643614946	0.085693339	7.51067648	5.88226E-14	2.20943E-13
AURKA	273.5075992	-0.38970591	0.051885806	-7.510838493	5.87498E-14	2.20712E-13
USP54	438.416276	-0.363621742	0.048411818	-7.511011973	5.8672E-14	2.20462E-13
PRB1	17.00113756	-0.699417616	0.093103283	-7.51227663	5.81078E-14	2.18384E-13
CGN	7.014791877	-0.667619575	0.088859656	-7.513191097	5.77032E-14	2.16905E-13
SLC16A9	57.83571438	-0.797603323	0.106145907	-7.51421648	5.72528E-14	2.15253E-13
EMC7	954.643971	0.269996248	0.035930526	7.514397269	5.71737E-14	2.14997E-13
ANKRD13B	236.3046549	-0.427461615	0.056880197	-7.515121952	5.68579E-14	2.13851E-13
B4GAT1	64.91614338	-0.487985165	0.064933499	-7.515152794	5.68445E-14	2.13842E-13
ATP6V1E2	110.4955305	-0.272866962	0.036306784	-7.515591608	5.66541E-14	2.13166E-13

USP36	6312.908417	0.299232065	0.03980932	7.516633471	5.62047E-14	2.11516E-13
CD93	3420.827644	0.622859267	0.082845266	7.518344659	5.54741E-14	2.08807E-13
PRR14L	2612.457926	-0.271164122	0.036063619	-7.519049011	5.51761E-14	2.07725E-13
PDE5A	350.249884	-0.491910244	0.06541642	-7.519675419	5.49124E-14	2.06772E-13
ZNF791	2003.977757	0.292047006	0.038835036	7.520194045	5.4695E-14	2.05993E-13
C3orf70	28.95728841	-0.63917344	0.08499187	-7.5204068	5.46061E-14	2.05698E-13
TCF23	29.14136785	-0.932373506	0.123965931	-7.521207613	5.42726E-14	2.04481E-13
AGBL4	12.00424766	-0.675776992	0.089846274	-7.521480478	5.41594E-14	2.04094E-13
VLDLR	702.6019742	0.773353291	0.102818323	7.521551276	5.41301E-14	2.04022E-13
NUP62CL	28.33349802	-0.911262972	0.121142255	-7.522255345	5.38393E-14	2.02965E-13
BHLHE22	2.139007948	-1.231181849	0.163670449	-7.522322205	5.38118E-14	2.02901E-13
RAP1B	5873.22463	0.280112415	0.037234302	7.522966809	5.3547E-14	2.01941E-13
LIPN	83.11893165	0.74493092	0.099014905	7.523422089	5.33608E-14	2.01278E-13
BCL2L10	34.8465392	-0.861413105	0.114471037	-7.525162035	5.2655E-14	1.98654E-13
FAM72B	47.6014269	-0.436470935	0.057999276	-7.52545483	5.25371E-14	1.98247E-13
B3GAT3	849.7155313	-0.189409525	0.025166778	-7.526173037	5.22491E-14	1.97198E-13
SHISA3	4.386008923	-0.827203878	0.109909808	-7.526206195	5.22358E-14	1.97186E-13
SHMT1	428.4375555	-0.27153636	0.03607681	-7.526617774	5.20715E-14	1.96604E-13
WDR1	10209.9415	0.187782927	0.024948942	7.526689023	5.20431E-14	1.96535E-13
OR11G2	2.802289223	-0.901003329	0.119698288	-7.527286659	5.18056E-14	1.95675E-13
METTL4	191.7608312	-0.271113637	0.036017244	-7.527328722	5.17889E-14	1.9565E-13
CSNK1A1	6305.932466	0.202959839	0.026959599	7.528295894	5.14068E-14	1.94244E-13
PEG3	7.053309513	-1.127513254	0.149756027	-7.529000853	5.113E-14	1.93236E-13
IL17RD	7.504616333	-0.53320186	0.070815993	-7.529398902	5.09744E-14	1.92685E-13
TENT4B	915.2885619	0.285552005	0.037923958	7.529594042	5.08983E-14	1.92434E-13
GPR83	22.83746068	-0.585684886	0.077779561	-7.530061634	5.07164E-14	1.91784E-13
PMS2	445.6125489	-0.198964632	0.026421546	-7.530393279	5.05877E-14	1.91334E-13
PCDHB13	26.77804259	-0.783477438	0.10403859	-7.530642584	5.04912E-14	1.91006E-13
ARL6IP1	3517.156908	0.260778082	0.0346275	7.530953242	5.03712E-14	1.90589E-13
MT-ND3	27686.86254	-0.950089025	0.126139476	-7.532051457	4.99493E-14	1.89029E-13
PRR23A	1.239547191	-1.085894307	0.144130573	-7.534101109	4.9171E-14	1.8612E-13
ROCK1	7065.044984	0.230903291	0.030645888	7.53456033	4.89983E-14	1.85502E-13
CLRN1	2.263589632	-0.903288257	0.11988156	-7.53483901	4.88938E-14	1.85142E-13
AQP7	11.13647971	-0.576287999	0.076476414	-7.535499768	4.86468E-14	1.84242E-13
SRC	787.9588339	0.537778963	0.071363928	7.535725345	4.85628E-14	1.8396E-13
GPM6A	8.527893464	-0.780245237	0.103532193	-7.536257199	4.83652E-14	1.83247E-13
AL355312.5	9.967079263	-0.516131315	0.068460899	-7.539067138	4.73345E-14	1.79376E-13

ERN2	7.815290642	-0.697217165	0.092476382	-7.539407873	4.7211E-14	1.78943E-13
UBC	26768.9284	0.295706912	0.039218598	7.539966369	4.70093E-14	1.78213E-13
NFILZ	4.758073361	-0.796553677	0.10564219	-7.540109441	4.69577E-14	1.78052E-13
NBEAL1	526.524516	-0.365283004	0.048434548	-7.541786154	4.63578E-14	1.75811E-13
SORBS2	7.901952186	-0.715249889	0.094831686	-7.542309105	4.61722E-14	1.75141E-13
LAMB3	264.4777753	0.723742236	0.095956176	7.54242478	4.61312E-14	1.7502E-13
OR1N2	1.363684599	-1.18503356	0.157078475	-7.544213568	4.55025E-14	1.72668E-13
CR1	1128.975519	0.670590828	0.088872706	7.545520509	4.50485E-14	1.70978E-13
SLC6A9	400.8525298	-0.786224484	0.104197437	-7.545526157	4.50465E-14	1.70978E-13
GANC	1111.904621	-0.349966244	0.046375108	-7.546424314	4.47371E-14	1.69862E-13
KLF4	3436.20645	0.763317164	0.101125162	7.5482417	4.41174E-14	1.67542E-13
TMEM200A	11.13691604	-0.902099534	0.119466733	-7.551052148	4.31756E-14	1.63997E-13
PEX1	519.2672866	-0.220303639	0.029170861	-7.552181686	4.28027E-14	1.62612E-13
TBC1D22A	1157.938222	-0.218558341	0.028936233	-7.553102727	4.2501E-14	1.61497E-13
AC097625.1	5.158978464	-0.47516955	0.062894766	-7.554993506	4.18881E-14	1.59199E-13
FAM104B	114.7005083	-0.457864406	0.060588034	-7.557010406	4.12439E-14	1.56781E-13
SLC2A10	68.45717574	-0.879355262	0.116356051	-7.557451901	4.11042E-14	1.56281E-13
PPEF2	10.27969824	-0.553219828	0.073193694	-7.558299001	4.08374E-14	1.55297E-13
UBE2L5	18.26553144	-0.530656574	0.070207215	-7.558433564	4.07952E-14	1.55166E-13
ZNF577	374.6342064	-0.258999511	0.034266232	-7.55844735	4.07909E-14	1.55166E-13
SDR39U1	187.4091958	-0.239001704	0.03161991	-7.558582589	4.07485E-14	1.55049E-13
PELI2	1748.036825	0.361972136	0.047886291	7.55899287	4.06202E-14	1.54591E-13
PGF	153.220854	-0.560979252	0.074205521	-7.559804794	4.03675E-14	1.53659E-13
OR51B4	1.702366845	-0.860584898	0.113826517	-7.560495752	4.01536E-14	1.52874E-13
ADAM33	8.022560207	-0.698704295	0.092414349	-7.560560699	4.01336E-14	1.52828E-13
SSR1	4550.956084	0.204275235	0.027015262	7.561475353	3.98523E-14	1.51786E-13
CYP4A11	1.049764649	-1.118937878	0.147970188	-7.561914286	3.9718E-14	1.51304E-13
TANGO6	358.5807762	-0.393538467	0.052032714	-7.563289207	3.93002E-14	1.49742E-13
ANKRD20A1	7.694650015	-0.743837942	0.098338251	-7.564075364	3.90633E-14	1.48868E-13
ARHGAP19-SLIT1	4.255692627	-0.802287503	0.106054881	-7.564833405	3.88362E-14	1.48031E-13
SAMD7	4.127727063	-0.922940787	0.1219769	-7.566521105	3.83352E-14	1.4615E-13
ESCO1	1229.908156	0.235399683	0.031100946	7.568891515	3.76422E-14	1.43536E-13
HMCES	1081.205189	0.243812885	0.032211273	7.569178694	3.75591E-14	1.43247E-13
GPD2	1281.552298	-0.322857765	0.042648885	-7.5701338	3.7284E-14	1.42226E-13
RPAP2	1162.272546	-0.223166447	0.029479534	-7.570216216	3.72604E-14	1.42163E-13
ACTR3	8494.401526	0.180369932	0.0238217	7.57166491	3.68471E-14	1.40614E-13
FOXL2	3.56618357	-0.947851986	0.125178264	-7.572017339	3.67472E-14	1.4026E-13

MAIP1	308.0216972	0.206964587	0.027330561	7.57264306	3.65706E-14	1.39613E-13
BSCL2	123.0946222	-0.310952796	0.041062646	-7.572643961	3.65704E-14	1.39613E-13
RMDN3	903.1544175	-0.222808954	0.029419602	-7.573486262	3.63339E-14	1.38764E-13
MRS2	458.1103238	-0.230816041	0.030476835	-7.573491237	3.63325E-14	1.38764E-13
HECTD4	9448.087527	-0.355613659	0.04694722	-7.574754344	3.59808E-14	1.37469E-13
MAG	6.411254365	-0.689161276	0.09098062	-7.574814055	3.59642E-14	1.37432E-13
FDXACB1	26.6906209	-0.388280705	0.051257206	-7.575143785	3.5873E-14	1.3711E-13
KCNJ1	54.15751608	-0.585899606	0.077338643	-7.575767888	3.5701E-14	1.36479E-13
SHROOM3	17.84581152	-0.93654855	0.123610424	-7.576614622	3.54688E-14	1.35619E-13
ZNF821	149.5323717	0.349336832	0.046107141	7.576631902	3.54641E-14	1.35619E-13
HES1	100.2079618	-0.78500218	0.103606046	-7.57679895	3.54185E-14	1.35479E-13
GLCE	225.2036945	-0.36821579	0.048588853	-7.578194748	3.50396E-14	1.34056E-13
LGR5	11.22974899	-0.901454183	0.118949291	-7.578474598	3.49641E-14	1.33793E-13
AKR1C8P	11.39242777	-0.850866403	0.112260431	-7.579397279	3.47164E-14	1.32871E-13
GPR180	210.6151634	-0.312374456	0.041209617	-7.580134899	3.45196E-14	1.32144E-13
ASTN2	23.7498368	-0.647719767	0.085442292	-7.580786443	3.43467E-14	1.31508E-13
SPSB2	282.4148538	-0.395193222	0.052127567	-7.581271231	3.42185E-14	1.31043E-13
RPA4	39.48509124	-0.477721209	0.063008187	-7.581891007	3.40554E-14	1.30444E-13
ADH6	14.6988925	-0.551253232	0.072695598	-7.583034583	3.37565E-14	1.29324E-13
RASA2	1682.981029	0.294975064	0.038898343	7.583229577	3.37058E-14	1.29155E-13
PSMC1	782.4633826	0.191734258	0.025283861	7.583266658	3.36961E-14	1.29143E-13
IFT122	1605.468341	-0.226011137	0.029800761	-7.584072616	3.34874E-14	1.28368E-13
FAM200A	60.3444623	-0.283678424	0.037395671	-7.585862748	3.30282E-14	1.26633E-13
ADAMTS8	8.646774038	-0.585068294	0.077120898	-7.586378084	3.28972E-14	1.26155E-13
RSPO2	4.734280971	-0.923121641	0.121675139	-7.586772841	3.27971E-14	1.25796E-13
RNF122	848.4260136	0.356594094	0.04699551	7.587833323	3.25299E-14	1.24796E-13
SEC61A1	6246.483983	0.183432014	0.02416924	7.589482239	3.21186E-14	1.23242E-13
INO80B	24.13087527	-0.323876154	0.042666143	-7.5909406	3.17592E-14	1.21887E-13
UNC13D	5771.169822	-0.254057058	0.033466156	-7.591462224	3.16315E-14	1.21421E-13
TRPV3	41.6682985	-0.621320767	0.081844119	-7.591513911	3.16189E-14	1.21396E-13
PCDHB1	3.921410986	-0.702129432	0.092487354	-7.5916263	3.15915E-14	1.21315E-13
MORF4L2	3229.631725	0.215257996	0.02835282	7.592119325	3.14715E-14	1.20877E-13
IFI30	381.7918263	0.629061358	0.08282607	7.594968041	3.07868E-14	1.18271E-13
ASNSD1	248.8580372	0.246943159	0.03250326	7.597488965	3.01932E-14	1.16013E-13
MICU2	958.3005348	0.167967206	0.022107238	7.597837797	3.01119E-14	1.15724E-13
GPR107	3381.969839	0.221445145	0.029128003	7.602482777	2.90503E-14	1.11666E-13
CCL16	3.207528725	-0.605728675	0.079668287	-7.603134177	2.89044E-14	1.11126E-13

ZNF442	69.43087943	-0.504863585	0.06639304	-7.604164309	2.86751E-14	1.10267E-13
KLK2	2.319203308	-0.797333174	0.104850454	-7.604479979	2.86052E-14	1.10019E-13
SMIM3	5088.619141	0.436423015	0.057389945	7.604520543	2.85962E-14	1.10007E-13
MCRIP2	518.810948	-0.334802059	0.044010809	-7.607268887	2.79949E-14	1.07714E-13
ADAM8	5042.527167	0.462891981	0.060848578	7.607276947	2.79931E-14	1.07714E-13
PURA	486.6015742	-0.426013994	0.055996546	-7.607861987	2.78667E-14	1.07264E-13
TSGA10	223.9441929	-0.389053547	0.051137258	-7.60802517	2.78316E-14	1.07149E-13
RFK	482.6821753	0.290798301	0.038222119	7.60811569	2.78121E-14	1.07095E-13
ZNF782	286.0630738	-0.348774978	0.045841121	-7.608343063	2.77632E-14	1.06928E-13
C3AR1	649.0604307	0.579213292	0.076116377	7.609575207	2.74998E-14	1.05935E-13
CCL3L3	606.1718726	0.866351927	0.113838719	7.610345018	2.73365E-14	1.05326E-13
LRRC14B	2.78400303	-1.032451458	0.135659021	-7.610636226	2.7275E-14	1.0511E-13
CTPS1	1492.90035	-0.280876838	0.036896249	-7.612612323	2.68611E-14	1.03535E-13
SPSB1	113.7125117	0.667604936	0.087693404	7.612943536	2.67923E-14	1.0329E-13
ABCA10	30.5680854	-0.581126335	0.076327613	-7.613579352	2.66608E-14	1.02804E-13
CHRNA3	9.000475712	-0.648426373	0.085163607	-7.613890435	2.65966E-14	1.02577E-13
ACRV1	19.87346913	-0.425041253	0.055817355	-7.614858317	2.63981E-14	1.01831E-13
COPS6	1304.022471	-0.331194958	0.043489614	-7.615495425	2.62682E-14	1.0135E-13
C2orf73	1.523260567	-0.913908957	0.120006499	-7.615495542	2.62682E-14	1.0135E-13
RAB5C	1109.278839	0.339371666	0.044561235	7.615849631	2.61963E-14	1.01112E-13
GRIK3	4.624598798	-0.786483221	0.103257534	-7.616715116	2.60213E-14	1.00456E-13
RAB11FIP3	990.6226413	-0.328036152	0.04306613	-7.617033402	2.59572E-14	1.00229E-13
LYZ	66057.0467	0.682022044	0.089519608	7.618688886	2.56265E-14	9.89716E-14
INSC	5.863038196	-0.726126777	0.095294204	-7.61984198	2.53986E-14	9.81109E-14
AP4B1	700.2460673	-0.195098196	0.025603115	-7.620095964	2.53487E-14	9.79373E-14
AL033529.1	1.308483925	-0.837538372	0.109881257	-7.622213258	2.49363E-14	9.63628E-14
ADGRG7	2.884113602	-1.173888041	0.154006671	-7.622319414	2.49158E-14	9.63025E-14
TSHR	78.15888493	-0.577317851	0.07573154	-7.623215541	2.47433E-14	9.5655E-14
OR13C9	1.139771765	-1.1440212	0.150062523	-7.623630301	2.46639E-14	9.53668E-14
PEDS1	1079.898583	0.266753696	0.03498995	7.623723292	2.46461E-14	9.53169E-14
RIPOR3	1561.94729	-0.620960734	0.081449514	-7.623872756	2.46176E-14	9.52254E-14
DNAJC16	734.4953929	-0.227835965	0.029881915	-7.624543615	2.44899E-14	9.47502E-14
GPD1	34.24444013	-0.630909139	0.08273433	-7.625723639	2.42669E-14	9.3906E-14
MRPL27	403.5246429	-0.293603078	0.038496791	-7.626689615	2.40859E-14	9.32237E-14
LRRIQ4	7.588255192	-0.588047116	0.077103361	-7.626737809	2.40769E-14	9.32073E-14
PCDHGB1	8.920941195	-1.000893298	0.131227326	-7.627171339	2.39961E-14	9.29129E-14
FAM8A1	1643.905787	0.307296736	0.040284494	7.62816416	2.3812E-14	9.22186E-14

TSEN54	563.5312691	-0.348991897	0.045748807	-7.628437177	2.37617E-14	9.20417E-14
HPSE	197.0528558	0.616296742	0.080786026	7.628754235	2.37033E-14	9.18339E-14
TMEM258	898.2271437	-0.446765839	0.058548296	-7.630723145	2.33441E-14	9.04599E-14
ARHGAP40	1.597862326	-0.943572467	0.123640105	-7.631605215	2.31849E-14	8.98608E-14
NECAP1	2959.42684	0.274112626	0.035917866	7.631651168	2.31766E-14	8.98466E-14
MROH2A	5.248978144	-0.743651259	0.097420736	-7.633398106	2.28645E-14	8.86545E-14
ZMYM4	2605.618787	-0.229664194	0.03008306	-7.634336192	2.26987E-14	8.80288E-14
ELAVL2	1.451120453	-1.336377227	0.175042853	-7.634571795	2.26572E-14	8.78854E-14
ZNF205	23.6623594	-0.623380281	0.081648919	-7.63488718	2.26018E-14	8.76879E-14
EDNRB	18.93583608	0.850203621	0.11134856	7.635515175	2.24919E-14	8.72788E-14
CYP7B1	223.6828047	-0.980704404	0.128435921	-7.635748593	2.24512E-14	8.71381E-14
PFDN5	357.0929462	0.284056219	0.037199378	7.63604759	2.23992E-14	8.69533E-14
TINAGL1	61.41199038	0.782637193	0.102492221	7.636064358	2.23962E-14	8.69533E-14
SH3GL2	1.565642843	-1.005022623	0.131602108	-7.636827671	2.22639E-14	8.64626E-14
POLI	550.9816	-0.277457208	0.036328126	-7.637531522	2.21426E-14	8.60085E-14
LRTM2	25.63692549	-0.721468533	0.094456028	-7.638141742	2.20379E-14	8.56189E-14
PIAS1	3634.228608	0.227783602	0.02982032	7.63853656	2.19705E-14	8.53738E-14
PLEC	16856.99165	0.4654642	0.060935795	7.638600537	2.19595E-14	8.53483E-14
EEF2K	1687.979222	-0.288161798	0.0377209	-7.639313915	2.18382E-14	8.48937E-14
TOX4	3469.639498	0.220555109	0.028865487	7.640789456	2.15894E-14	8.3943E-14
LRRC7	177.1391839	-0.483737199	0.063298751	-7.642128707	2.13659E-14	8.30907E-14
GUCY2D	31.60152587	-0.736653398	0.096373669	-7.643720601	2.11033E-14	8.20857E-14
FGB	2.198768171	-1.239237062	0.162118762	-7.644007663	2.10563E-14	8.1919E-14
DERL3	69.06308222	-0.378490746	0.04950885	-7.644910942	2.0909E-14	8.13622E-14
SCGB3A1	23.69543455	-0.79202997	0.103595901	-7.645379457	2.0833E-14	8.10826E-14
GTF2IRD2B	287.8458874	-0.257804125	0.033719711	-7.645502142	2.08131E-14	8.10214E-14
CCL1	10.57451179	-1.521760225	0.199012215	-7.646566933	2.06416E-14	8.03696E-14
TIMM21	165.0051812	-0.291211205	0.03808282	-7.646786832	2.06064E-14	8.02483E-14
SSPN	23.65149298	-0.631443929	0.082575905	-7.646830268	2.05994E-14	8.02372E-14
EGLN3	26.36328899	-0.76412403	0.099926155	-7.646887134	2.05903E-14	8.02177E-14
POF1B	12.66715259	-1.238212976	0.161919832	-7.64707423	2.05604E-14	8.01171E-14
AC000093.1	12.35510102	-0.698893139	0.091387586	-7.647571974	2.0481E-14	7.98235E-14
MRPL58	220.9884087	-0.389374406	0.050913361	-7.647784354	2.04472E-14	7.97077E-14
RAI1	2209.273296	-0.280579323	0.03667887	-7.649617379	2.01578E-14	7.85954E-14
GOLGA8Q	52.9377537	-0.642857327	0.084032703	-7.650085082	2.00846E-14	7.83257E-14
DUSP21	1.657417222	-1.101388638	0.143966663	-7.650303328	2.00506E-14	7.82084E-14
RER1	1293.408448	-0.187546329	0.024501367	-7.654525024	1.94028E-14	7.56968E-14

FUT10	249.3453267	-0.360291899	0.047063002	-7.655523033	1.92527E-14	7.51262E-14
POMT2	511.7880834	-0.256276724	0.033474718	-7.6558292	1.92069E-14	7.49624E-14
BAZ2A	15444.73862	0.286373749	0.037404688	7.656092441	1.91676E-14	7.48239E-14
NFYA	1123.431002	-0.278345786	0.036350026	-7.657375258	1.89772E-14	7.40953E-14
USP51	95.65807024	-0.346866661	0.045296074	-7.657764371	1.89198E-14	7.3886E-14
ENC1	617.2615019	0.438537339	0.05726351	7.658233692	1.88508E-14	7.36312E-14
LPGAT1	2436.704173	0.252739878	0.033001971	7.658326787	1.88371E-14	7.35926E-14
WFDC13	0.813323843	-1.025436884	0.13389289	-7.658635849	1.87918E-14	7.34304E-14
PM20D2	1073.936733	-0.316418993	0.041310588	-7.659513251	1.86639E-14	7.2945E-14
SLFN5	483.8250487	0.57184674	0.074652589	7.660105942	1.8578E-14	7.26237E-14
RBM22	3342.708846	0.233046477	0.030422882	7.660236708	1.85591E-14	7.25643E-14
OSBPL3	981.5639817	-0.418002288	0.054564982	-7.660632784	1.85019E-14	7.23553E-14
GLIS2	78.30505864	-0.613399269	0.080061459	-7.661604946	1.83624E-14	7.18239E-14
C14orf28	68.53565669	0.31882599	0.041610567	7.66214005	1.8286E-14	7.15395E-14
GATAD2A	4942.878607	0.224835679	0.029343317	7.66224484	1.82711E-14	7.14954E-14
NUBP1	323.0987613	0.244407384	0.031892744	7.663416626	1.81051E-14	7.086E-14
RNASEH2B	3528.966752	-0.226454151	0.029547833	-7.663985167	1.80251E-14	7.05609E-14
HSPBP1	602.6224987	-0.367093783	0.047890022	-7.665350134	1.78344E-14	6.98285E-14
TAX1BP1	3860.130289	0.248535594	0.032423165	7.66537121	1.78315E-14	6.98285E-14
TCTA	807.7063627	-0.279931802	0.036518301	-7.665520879	1.78107E-14	6.97636E-14
CCDC93	2086.820598	0.263697912	0.034394502	7.666862286	1.76255E-14	6.9052E-14
MCM5	4068.066819	-0.26564388	0.034647275	-7.667092978	1.75938E-14	6.89417E-14
SEC14L4	89.18583022	-0.831317629	0.108410297	-7.668253402	1.74354E-14	6.83347E-14
ZBTB48	1144.990739	-0.213350312	0.027821937	-7.668420561	1.74127E-14	6.82594E-14
KCNB1	45.21008958	-0.580177493	0.075650023	-7.669230854	1.73031E-14	6.78432E-14
YAE1	48.94346361	-0.363985157	0.047460446	-7.669231706	1.7303E-14	6.78432E-14
GREB1	68.11019022	-0.957699023	0.124874377	-7.669299687	1.72938E-14	6.7834E-14
LEPROTL1	1730.76151	0.279678735	0.036462369	7.670339103	1.71542E-14	6.73E-14
C16orf89	5.753415986	-0.594522207	0.07750696	-7.670565448	1.7124E-14	6.71948E-14
RS1	1.523222698	-0.929572481	0.121179328	-7.671048324	1.70596E-14	6.69557E-14
LNP1	32.25379093	-0.404239536	0.052691386	-7.671833478	1.69555E-14	6.65604E-14
EFHC2	184.2756839	-0.652194105	0.085009573	-7.67200781	1.69325E-14	6.64833E-14
TYW5	822.7534936	0.269926036	0.035180056	7.672700647	1.68412E-14	6.61383E-14
FLACC1	116.6004275	-0.474188268	0.06179497	-7.673573884	1.67269E-14	6.57026E-14
ZNF544	545.9163397	-0.460641265	0.060027944	-7.673780495	1.67E-14	6.56099E-14
KCTD2	991.8966651	0.247733426	0.032280787	7.674330336	1.66285E-14	6.53422E-14
CD274	42.92686466	0.599497062	0.078117099	7.674338549	1.66274E-14	6.53422E-14

WNT6	6.664759641	-0.877781352	0.114376658	-7.674479799	1.66091E-14	6.52923E-14
DCSTAMP	17.19126204	0.964861865	0.125714632	7.675016451	1.65397E-14	6.50326E-14
ADRA1A	2.669837708	-0.741593673	0.096622131	-7.675194723	1.65168E-14	6.49553E-14
SLC9C1	17.85414007	-0.81974079	0.106803374	-7.675233055	1.65118E-14	6.49489E-14
AC006978.2	4.952545356	-0.621037956	0.080911481	-7.67552329	1.64745E-14	6.4815E-14
CPLANE1	1439.441886	-0.304155864	0.039624362	-7.675981438	1.64157E-14	6.45968E-14
ZNF730	47.09099692	-0.546850102	0.071221638	-7.678145491	1.61408E-14	6.35279E-14
ADK	1100.313327	-0.232799859	0.030319479	-7.678227524	1.61305E-14	6.35E-14
PITX3	1.875120502	-0.836199913	0.108896246	-7.6788681	1.605E-14	6.3196E-14
LARP6	11.8230598	-0.987986891	0.128654115	-7.679403758	1.59831E-14	6.2945E-14
PDLIM4	158.5443053	0.60896026	0.079293083	7.679866075	1.59255E-14	6.27309E-14
ZNF197	339.2373419	-0.302589676	0.039400306	-7.679881466	1.59236E-14	6.27309E-14
SPAG6	273.0277932	-1.053123638	0.137125391	-7.680004609	1.59083E-14	6.26883E-14
DLGAP5	440.2898785	-0.536182609	0.069813296	-7.680236288	1.58795E-14	6.25877E-14
ATP6V1C2	82.46318445	0.392114001	0.051046461	7.681511948	1.57222E-14	6.19799E-14
BLM	668.3484603	-0.280371752	0.036499513	-7.681520374	1.57211E-14	6.19799E-14
VEGFC	10.66514419	-0.987284345	0.128524666	-7.681672139	1.57025E-14	6.19274E-14
CCDC8	17.7716966	-0.9996241	0.130119015	-7.682382946	1.56156E-14	6.1597E-14
PRDX2	1673.674542	-0.664210447	0.086455309	-7.682702862	1.55767E-14	6.14557E-14
CACUL1	3610.381888	0.2650873	0.034503232	7.68297011	1.55442E-14	6.134E-14
SPATA2	1200.660419	0.301556629	0.039246008	7.683752866	1.54495E-14	6.09785E-14
ANO3	1.215367492	-1.434535418	0.186690928	-7.684012465	1.54182E-14	6.08672E-14
TP53AIP1	6.898160895	-0.876971356	0.114119519	-7.684674496	1.53386E-14	6.05655E-14
ENOX2	258.0345018	-0.376316552	0.048969456	-7.684719819	1.53332E-14	6.05563E-14
ERP44	2345.961284	0.254260098	0.033086435	7.684723203	1.53328E-14	6.05563E-14
ESRRB	13.13084344	-0.620057765	0.080685255	-7.684895604	1.53122E-14	6.04976E-14
KCNK3	2.809619511	-0.733646058	0.095448647	-7.686290832	1.51462E-14	5.98539E-14
STAB2	33.15143632	-0.669453636	0.087090055	-7.686912513	1.50728E-14	5.9576E-14
TOR1AIP2	6887.690152	0.30560062	0.039751197	7.687834422	1.49646E-14	5.91603E-14
KIAA0100	6812.83436	-0.198842981	0.025863865	-7.68806143	1.49381E-14	5.90674E-14
C17orf75	191.6793525	-0.243466807	0.031666021	-7.68858233	1.48774E-14	5.88393E-14
MCTS1	682.5837481	-0.291432836	0.037902439	-7.689025966	1.48259E-14	5.86475E-14
MIA3	2446.638753	0.214255617	0.027856505	7.691403318	1.4553E-14	5.75795E-14
FAM174C	493.403894	-0.43591701	0.056672703	-7.691833727	1.45041E-14	5.73977E-14
CTRB1	2.12530304	-0.849458001	0.11042784	-7.692426105	1.44371E-14	5.7144E-14
PDE12	2031.965915	0.182472075	0.023720275	7.692662594	1.44104E-14	5.705E-14
UBLCP1	489.0090282	0.314704306	0.040906368	7.693283916	1.43406E-14	5.6785E-14

RBM12B	641.2149426	-0.332793732	0.043251525	-7.694381454	1.4218E-14	5.63111E-14
TCF19	571.5920239	-0.387488287	0.050348477	-7.69612729	1.40252E-14	5.55587E-14
C2CD3	2129.098768	-0.23290193	0.030261523	-7.696305578	1.40057E-14	5.54925E-14
C17orf99	69.14056404	-0.671947223	0.087303646	-7.696668512	1.3966E-14	5.53464E-14
RPH3A	31.79751331	0.888178915	0.115348764	7.699943068	1.36127E-14	5.39573E-14
MRPS21	1010.14161	-0.326324341	0.042362994	-7.70305183	1.32854E-14	5.26709E-14
PPP1R12B	2157.41831	-0.320012203	0.0415367	-7.70432425	1.31537E-14	5.21593E-14
HJV	4.002727142	-0.569008994	0.073847453	-7.705194535	1.30644E-14	5.18155E-14
TAS2R31	59.25291255	-0.547019033	0.070991286	-7.70543918	1.30394E-14	5.17269E-14
OR1J1	2.9528908	-1.121467301	0.14553616	-7.705764002	1.30063E-14	5.16059E-14
C18orf25	3620.694199	0.248293715	0.032218531	7.70654977	1.29265E-14	5.12997E-14
OR7A17	4.242209644	-0.600192015	0.077879767	-7.706648854	1.29165E-14	5.12703E-14
MRPL51	507.1362551	-0.405336792	0.052594851	-7.706777022	1.29035E-14	5.12292E-14
FBXL7	2.837633658	-1.129306886	0.146524654	-7.707282379	1.28525E-14	5.10372E-14
PDIA2	25.88081362	-0.584753346	0.075862684	-7.708049793	1.27755E-14	5.07416E-14
ARHGAP4	7502.136678	-0.301287371	0.039085311	-7.708455215	1.2735E-14	5.0591E-14
GCLC	1305.798474	-0.405935221	0.052649147	-7.710195576	1.25625E-14	4.99159E-14
DPM1	618.6831012	0.250124263	0.032438222	7.710788313	1.25043E-14	4.96947E-14
PCDHGA12	46.53091702	-0.658251835	0.085351017	-7.712290496	1.23579E-14	4.91231E-14
FBLN7	34.07888256	0.62552823	0.081098591	7.713182437	1.22718E-14	4.87907E-14
DGKB	1.575304758	-1.36692529	0.177179848	-7.714902719	1.21075E-14	4.81469E-14
FAM181B	2.500362429	-1.187067591	0.153860097	-7.715240099	1.20755E-14	4.80295E-14
TSPAN17	708.3776343	-0.416432034	0.053966725	-7.716459294	1.19606E-14	4.75822E-14
ZNF235	148.24944	-0.358740865	0.04648634	-7.717124365	1.18984E-14	4.73443E-14
SHANK2	26.72899669	-0.749418024	0.097106697	-7.717470069	1.18661E-14	4.72257E-14
AP1M2	9.289557256	-0.704434477	0.091274879	-7.71772568	1.18424E-14	4.71407E-14
MAP6	5.590593828	-0.614910214	0.079662851	-7.71890792	1.17331E-14	4.67151E-14
PHF10	2198.178569	0.239845533	0.031071748	7.719087328	1.17166E-14	4.66589E-14
BRAF	4150.320652	0.286129075	0.037064923	7.719672733	1.16629E-14	4.64545E-14
MYL6B	483.0395779	-0.316629207	0.041015032	-7.719833292	1.16482E-14	4.64055E-14
LAPTM4A	5169.703998	0.339750001	0.044008871	7.720034421	1.16298E-14	4.63417E-14
GALNT13	1.821781138	-1.869741937	0.242139008	-7.721770854	1.14725E-14	4.57239E-14
PTRH1	4.200698267	-0.585257988	0.075789256	-7.722176227	1.1436E-14	4.5588E-14
PPWD1	1274.227048	0.247363264	0.032032837	7.722177751	1.14359E-14	4.5588E-14
FKBP3	519.0735163	-0.300491792	0.038912162	-7.722310319	1.1424E-14	4.55586E-14
CYP2B6	2.679047563	-0.702034741	0.090904935	-7.722735226	1.1386E-14	4.54162E-14
OSBP	3176.8161	0.247115665	0.031994309	7.723738197	1.12967E-14	4.50693E-14

TOX	719.4656195	-0.512364189	0.066302665	-7.727656053	1.09545E-14	4.3713E-14
DCAF11	2345.492084	-0.259347701	0.033559245	-7.728055325	1.09202E-14	4.3585E-14
TOM1L1	59.05570753	-0.982684547	0.127144124	-7.728902541	1.08478E-14	4.33048E-14
ANKFY1	2084.821373	-0.289404222	0.037444135	-7.728959991	1.08429E-14	4.32941E-14
STARD3NL	1426.048923	0.241748003	0.031277889	7.729038356	1.08362E-14	4.32763E-14
SPHK1	353.4205382	0.499901766	0.064675971	7.729327512	1.08116E-14	4.31869E-14
CFAP47	26.13786906	-0.964601114	0.124787275	-7.729963768	1.07577E-14	4.29804E-14
NR2F2	5.277293573	-1.026704752	0.132794291	-7.73154285	1.06251E-14	4.24591E-14
DCTN6	408.3283719	0.294341223	0.038059773	7.733656749	1.04501E-14	4.17682E-14
TCF24	4.394058409	-0.854155713	0.110443541	-7.73386748	1.04328E-14	4.17076E-14
ZNF510	446.9208829	-0.291196781	0.037644235	-7.735494665	1.03002E-14	4.1186E-14
DUT	1299.223646	-0.362882493	0.046905284	-7.736494905	1.02195E-14	4.08718E-14
SS18	2335.305224	0.188608925	0.02437694	7.737186155	1.01641E-14	4.06586E-14
FTCDNL1	155.2802508	-0.508211658	0.065682862	-7.737355579	1.01506E-14	4.06127E-14
HEY2	8.118577133	-1.086199834	0.140360728	-7.738630652	1.00493E-14	4.02158E-14
RARG	261.5563061	0.461357036	0.059614636	7.738989338	1.0021E-14	4.01107E-14
CHRNA5	8.241901771	-0.527900428	0.068208079	-7.739558601	9.97625E-15	3.99397E-14
ING5	1571.597419	-0.255334105	0.032984223	-7.741098142	9.85618E-15	3.94671E-14
IQUB	9.86402056	-0.430433919	0.055575746	-7.744995812	9.5585E-15	3.82829E-14
CSNK2A2	1765.108323	0.198166602	0.025585694	7.745211175	9.54231E-15	3.82259E-14
MARCHF1	660.1600828	0.577689111	0.07458308	7.745578589	9.51476E-15	3.81233E-14
MRRF	1134.646333	-0.172579378	0.022277861	-7.746676277	9.4329E-15	3.78031E-14
PAG1	2677.945054	0.408240184	0.052695054	7.747220079	9.39261E-15	3.76493E-14
DESI2	1136.280206	0.196061799	0.025300897	7.749203526	9.24707E-15	3.70735E-14
MAST1	72.68153431	-0.543604426	0.070148488	-7.749339199	9.2372E-15	3.70415E-14
NUDT12	44.97875114	-0.548779591	0.070815311	-7.749448322	9.22926E-15	3.70173E-14
CWF19L1	1573.97439	0.229390124	0.029600172	7.749621188	9.21671E-15	3.69745E-14
F7	4.270404908	-0.921499048	0.118901941	-7.750075724	9.18378E-15	3.685E-14
SLK	2976.064012	0.262880174	0.033915973	7.750925253	9.12254E-15	3.66117E-14
MAS1	16.8386277	-0.617938191	0.079721802	-7.751181891	9.10412E-15	3.65453E-14
PRKG1	8.940690577	-1.094662184	0.141222776	-7.751314724	9.0946E-15	3.65146E-14
HTR1F	194.3364284	-0.95082701	0.122623286	-7.754049337	8.90077E-15	3.57437E-14
ITIH3	50.50016896	-0.559430834	0.072143577	-7.754409398	8.87556E-15	3.56498E-14
VMAC	378.6064934	-0.258588539	0.033339255	-7.75627816	8.74581E-15	3.51358E-14
CSMD1	119.0560856	-0.938082264	0.12093371	-7.75699567	8.69649E-15	3.49449E-14
TEX52	75.45941897	-0.458812646	0.059145085	-7.757409461	8.66817E-15	3.48382E-14
SDCCAG8	1024.186039	-0.319052023	0.041128332	-7.757475416	8.66367E-15	3.48273E-14

GRIA1	1.412700338	-1.281631896	0.165189834	-7.758539749	8.59128E-15	3.45434E-14
CBSL	2.05503416	-0.868966348	0.111998073	-7.758761579	8.57627E-15	3.44901E-14
THSD4	26.91762574	-0.960529535	0.123789217	-7.759395823	8.53349E-15	3.43251E-14
MRPL36	206.6383839	-0.484446537	0.062427525	-7.760143253	8.48335E-15	3.41304E-14
GOLGA6L2	1.933041972	-1.117344892	0.143940352	-7.762554967	8.32353E-15	3.34943E-14
FAM83C	4.533230606	-0.758319165	0.0976846	-7.762934652	8.29864E-15	3.3401E-14
ZBTB42	179.9915116	-0.421094688	0.05424404	-7.762966869	8.29653E-15	3.33994E-14
DLD	2556.540831	0.24069199	0.031003104	7.763480322	8.26299E-15	3.32713E-14
TRADD	296.5739047	-0.336404814	0.043330204	-7.763748742	8.24552E-15	3.32077E-14
LBP	16.26498953	-0.816840215	0.105207131	-7.764114516	8.22176E-15	3.31188E-14
EIF1	44295.63342	0.341959237	0.044040394	7.76467243	8.18565E-15	3.29802E-14
BX276092.9	3.773678596	-1.028995286	0.132510409	-7.765392102	8.1393E-15	3.28002E-14
MFHAS1	1238.503437	-0.431037269	0.055503247	-7.765982991	8.10144E-15	3.26544E-14
AIPL1	2.049689477	-0.81588532	0.105046946	-7.766863795	8.04533E-15	3.24349E-14
SKA3	258.1656881	-0.353805325	0.045552993	-7.766895291	8.04333E-15	3.24335E-14
DCP2	3513.637804	0.257256712	0.03311477	7.768639588	7.93335E-15	3.19966E-14
SGCD	5.644664064	-0.96765735	0.124520602	-7.771062245	7.78306E-15	3.13969E-14
AVP	8.704245611	0.848008715	0.109121239	7.771252591	7.77138E-15	3.13562E-14
MEF2B	9.910430064	-0.445971922	0.057379748	-7.772287971	7.7081E-15	3.11073E-14
ZSCAN25	1043.991878	0.229562859	0.029535001	7.772569981	7.69095E-15	3.10445E-14
FN3KRP	521.6343986	-0.198143918	0.025491321	-7.772995152	7.66517E-15	3.09468E-14
DISP3	8.361661672	-0.638865152	0.082188339	-7.773184821	7.65369E-15	3.09069E-14
CCDC116	15.38490264	-0.489908027	0.063025034	-7.773229039	7.65102E-15	3.09025E-14
MRPL52	541.1403713	-0.407328263	0.052396645	-7.773937813	7.60831E-15	3.07363E-14
PSEN2	42.95797297	-0.501995463	0.064560872	-7.775537195	7.51279E-15	3.03567E-14
ACLY	3743.472591	-0.216797561	0.027881954	-7.775551198	7.51196E-15	3.03567E-14
DSCAM	2.151443301	-1.161195159	0.149331218	-7.77597058	7.48711E-15	3.02654E-14
ADIPOQ	2.473213369	-0.837283251	0.107664489	-7.776781894	7.43927E-15	3.00783E-14
OR1L6	2.758866528	-0.773083044	0.099397023	-7.777728382	7.38384E-15	2.98603E-14
KRBOX4	259.9794276	-0.227314502	0.029216078	-7.780459053	7.22619E-15	2.92288E-14
GNB3	23.43159815	-0.545081189	0.070054793	-7.780783706	7.20766E-15	2.91599E-14
WASHC3	414.3208487	-0.27398161	0.035211991	-7.780917946	7.20002E-15	2.9135E-14
TNFSF10	448.2657003	0.561399246	0.072150554	7.780941594	7.19867E-15	2.9135E-14
MAPK8IP2	17.95304616	-0.431095505	0.055397008	-7.781927609	7.14277E-15	2.89153E-14
MTMR2	1238.065415	0.236545192	0.030389928	7.783670768	7.04499E-15	2.85254E-14
NSMCE1	823.1974075	-0.368962589	0.047400684	-7.783908555	7.03176E-15	2.84777E-14
DZIP3	324.7391139	-0.34321513	0.044086481	-7.785042516	6.96898E-15	2.82293E-14

ARL10	345.9218082	-0.47312788	0.06076591	-7.786074174	6.91234E-15	2.80056E-14
MUC3A	3.754434472	-0.878912282	0.112865512	-7.787252894	6.84818E-15	2.77514E-14
LDHD	92.61078396	-0.52862509	0.067872061	-7.788552174	6.77814E-15	2.74733E-14
PDZD11	167.9125682	-0.374677205	0.048101925	-7.78923516	6.7416E-15	2.73309E-14
MSANTD2	98.92381552	-0.352126996	0.045197755	-7.790807188	6.65825E-15	2.69985E-14
EXOC8	1022.711815	0.25156629	0.032288325	7.791246272	6.63514E-15	2.69104E-14
RNF8	304.9130611	-0.204896994	0.02629709	-7.791622212	6.61543E-15	2.6836E-14
AREL1	3310.736027	0.239421228	0.030724714	7.792463991	6.57149E-15	2.66633E-14
PJVK	80.94471057	-0.32304814	0.041452879	-7.793141312	6.53634E-15	2.65262E-14
DHRS4L2	150.5071856	-0.36615042	0.046981335	-7.793529408	6.51629E-15	2.64503E-14
BACE1	346.4102924	-0.383008923	0.049130907	-7.795681881	6.40616E-15	2.60087E-14
TJP2	114.6938927	-0.512494713	0.065740164	-7.795762662	6.40206E-15	2.59974E-14
MDC1	1561.430685	-0.196029461	0.025144674	-7.796062867	6.38686E-15	2.59411E-14
HERPUD2	2272.298509	0.18084287	0.023192819	7.797364861	6.32133E-15	2.56802E-14
ZBTB10	4147.84724	0.378619326	0.04855503	7.797736378	6.30275E-15	2.56101E-14
ALOX12B	8.766629733	-0.644930166	0.082692159	-7.799169514	6.23159E-15	2.53262E-14
GIPC2	7.428513403	-0.808971351	0.103718148	-7.799708799	6.20502E-15	2.52234E-14
OR6F1	1.829801965	-1.123510646	0.144034282	-7.800300259	6.176E-15	2.51107E-14
SPATA16	3.112600704	-1.105439961	0.141700113	-7.801263818	6.12902E-15	2.49249E-14
SPATA45	2.935866041	-0.558182851	0.071542558	-7.802109255	6.08809E-15	2.47635E-14
CGREF1	42.06260837	-0.724616185	0.092868944	-7.802567284	6.06603E-15	2.46789E-14
MRPL30	549.5289159	-0.216960373	0.027806019	-7.802640613	6.0625E-15	2.46697E-14
SLURP1	1.269164982	-0.887084028	0.113688255	-7.802776344	6.05598E-15	2.46483E-14
PCDHGA5	41.65164249	-0.786559175	0.10080053	-7.803125415	6.03925E-15	2.45853E-14
ARL15	279.0947178	-0.394985681	0.050604461	-7.805352991	5.93352E-15	2.41599E-14
KLRF2	25.84506987	-1.635112088	0.209391314	-7.808882121	5.76974E-15	2.3498E-14
PCNX4	1670.501082	-0.251338654	0.032177461	-7.811015646	5.6729E-15	2.31083E-14
EXD1	4.880797613	-0.588226486	0.075298221	-7.811957317	5.63066E-15	2.29411E-14
UBALD2	3983.64765	0.353755948	0.045278951	7.812812277	5.59259E-15	2.27907E-14
PLEKHO1	3450.120349	0.326889441	0.041838624	7.81310211	5.57974E-15	2.27431E-14
ACTN2	12.60212836	-0.896510715	0.114729185	-7.814146987	5.53365E-15	2.25599E-14
IQSEC1	3835.860251	0.442114303	0.056578274	7.814206325	5.53104E-15	2.2554E-14
RNF13	937.3674057	0.280790557	0.035930707	7.814779637	5.50593E-15	2.24562E-14
PNLIPRP1	1.731787177	-0.974337578	0.124671364	-7.815247587	5.48551E-15	2.23776E-14
UPRT	432.2452974	0.198347557	0.0253793	7.815327987	5.48201E-15	2.2368E-14
TMEM95	5.462347678	-0.89345146	0.114303806	-7.816462943	5.43283E-15	2.2172E-14
TMEM238L	1.022592278	-0.879735619	0.11250055	-7.819833954	5.28931E-15	2.15907E-14

ZNF341	195.3296743	-0.255546299	0.032677659	-7.82021441	5.27334E-15	2.15301E-14
LAMA4	19.24206762	-0.851078942	0.108828639	-7.82035821	5.26732E-15	2.151E-14
LIMK2	2948.266913	0.420165869	0.053723807	7.820850629	5.24676E-15	2.14305E-14
CLCN3	1136.831144	-0.458569256	0.058607699	-7.82438594	5.10141E-15	2.08411E-14
BHMT2	5.76072537	-0.742773364	0.094927501	-7.824638331	5.09118E-15	2.08037E-14
KIAA0319	14.62418838	-0.680905888	0.087020061	-7.824700234	5.08868E-15	2.07978E-14
INSL6	55.69678241	-0.487744141	0.062332937	-7.824822076	5.08375E-15	2.0782E-14
JHY	77.67052838	-0.578597234	0.07393851	-7.825384013	5.0611E-15	2.06937E-14
EPB42	1288.627826	-0.853313996	0.109032065	-7.826266491	5.02571E-15	2.05533E-14
NKAIN4	2.411407484	-1.057566631	0.135120078	-7.826865165	5.00185E-15	2.046E-14
PCDH8	3.259859622	-1.07479007	0.137291599	-7.828520297	4.93645E-15	2.01967E-14
BPNT2	2299.192912	0.218310432	0.027864623	7.834681111	4.70034E-15	1.92347E-14
TESC	1023.229405	-0.567978188	0.072492427	-7.835000313	4.68842E-15	1.919E-14
RMND5B	616.8541458	-0.373781755	0.047706367	-7.835049612	4.68658E-15	1.91864E-14
LILRB1	397.0265174	0.669553521	0.085453676	7.835280518	4.67797E-15	1.91552E-14
TXNRD1	4920.813857	0.233106826	0.029747827	7.836096009	4.64771E-15	1.90353E-14
OR2T12	1.552910775	-1.012202075	0.129158852	-7.83687731	4.61889E-15	1.89212E-14
DNAJB11	2103.691555	0.291467105	0.037189009	7.837452794	4.59778E-15	1.88387E-14
ZNF850	698.3938879	-0.351721421	0.044876026	-7.837624103	4.59151E-15	1.88169E-14
ARRDC5	139.5917209	-0.38709256	0.049388097	-7.837770325	4.58617E-15	1.8799E-14
DNMT1	6069.559448	-0.235309896	0.030016635	-7.839316192	4.53007E-15	1.85729E-14
MTDH	8017.639099	0.208604994	0.02660975	7.839419628	4.52634E-15	1.85615E-14
NEK10	57.95153075	-0.74431157	0.094939742	-7.839831354	4.51152E-15	1.85046E-14
CILP	19.03476778	-0.465593155	0.059380799	-7.84080311	4.47674E-15	1.83658E-14
CREB1	1575.415826	-0.197147674	0.025139873	-7.842031471	4.43315E-15	1.81908E-14
PACC1	805.8789149	-0.34466656	0.04395064	-7.842128426	4.42973E-15	1.81806E-14
GATM	265.1666274	-0.739981918	0.094353466	-7.842657473	4.4111E-15	1.81079E-14
GLB1L2	42.73112909	-0.759719121	0.09685967	-7.843503063	4.38148E-15	1.79901E-14
TGM2	636.1179977	-0.761812801	0.097120461	-7.843999041	4.3642E-15	1.79229E-14
MEX3C	4969.686665	0.289741887	0.036936777	7.84426547	4.35495E-15	1.78887E-14
KRTAP5-10	4.729018547	-0.665831807	0.084875804	-7.844777642	4.33721E-15	1.78195E-14
SLC5A5	27.01534917	-0.580158891	0.073952167	-7.845055957	4.3276E-15	1.77838E-14
ENDOV	315.8299395	-0.236294958	0.030118042	-7.845628133	4.30792E-15	1.77066E-14
ACADSB	383.183338	-0.327420796	0.041731144	-7.84595783	4.29661E-15	1.76639E-14
ERCC6L	100.3774461	-0.392415441	0.050013713	-7.846156911	4.2898E-15	1.76396E-14
CD44	24637.85546	0.342909061	0.043689569	7.848762803	4.20161E-15	1.72806E-14
LRCH3	1301.706353	-0.274735582	0.035003357	-7.848835161	4.19919E-15	1.72742E-14

PPT2-EGFL8	16.93281032	-0.472817577	0.060237068	-7.849279372	4.18435E-15	1.72168E-14
SPSB3	21.78597005	-0.371843092	0.047372611	-7.849326529	4.18277E-15	1.72139E-14
TENM2	5.048735039	-1.083927587	0.138044412	-7.852020745	4.09387E-15	1.68516E-14
INSRR	20.58025457	-0.657233453	0.083698713	-7.852372242	4.08241E-15	1.68079E-14
GLYATL2	7.682187045	-0.965681401	0.122954984	-7.853942712	4.03158E-15	1.66022E-14
KMT2E	9814.240003	0.279347964	0.035563088	7.854997304	3.99781E-15	1.64666E-14
PLSCR4	39.40333812	-0.930184249	0.118412253	-7.855472959	3.98266E-15	1.64076E-14
LRCOL1	9.565563025	-0.821356082	0.104535062	-7.857230563	3.9272E-15	1.61825E-14
CDK2	642.2100929	-0.232251525	0.029556378	-7.857915668	3.90578E-15	1.60977E-14
USE1	581.217919	-0.33834181	0.0430556	-7.858253275	3.89527E-15	1.60577E-14
MGA	3780.867489	-0.327242664	0.041642811	-7.858323122	3.8931E-15	1.60522E-14
SNX2	2518.814503	0.227526617	0.028952482	7.858622202	3.88382E-15	1.60173E-14
FMO2	7.983953605	-1.097890652	0.139700026	-7.85891518	3.87474E-15	1.59832E-14
ADGRL2	82.00908278	-1.162999782	0.14798152	-7.85908794	3.86941E-15	1.59646E-14
ZNF23	29.26721691	-0.461936732	0.05876691	-7.860490341	3.82633E-15	1.57902E-14
PDHA1	1610.968222	-0.195450684	0.024856022	-7.863313236	3.74104E-15	1.54415E-14
BRI3BP	916.3633203	-0.491265828	0.062474311	-7.863485346	3.7359E-15	1.54235E-14
ADPGK	5107.820926	0.192438703	0.024467431	7.865096311	3.68814E-15	1.52295E-14
CARNMT1	504.6185446	-0.230300098	0.029277734	-7.866049371	3.66016E-15	1.51172E-14
ULK4	810.7477393	-0.325516774	0.04136165	-7.870014251	3.54601E-15	1.46488E-14
DEGS1	1103.751338	0.248669799	0.031589475	7.871919232	3.49242E-15	1.44305E-14
ARF1	12891.25092	0.288565834	0.036641899	7.875296892	3.39935E-15	1.40489E-14
RAC1	4987.861238	0.301680762	0.038303127	7.876139194	3.37652E-15	1.39575E-14
SMAP2	5155.735421	0.327948837	0.041637453	7.876294416	3.37233E-15	1.39431E-14
ERAL1	781.7583073	-0.270057195	0.03428252	-7.877402011	3.34258E-15	1.3823E-14
RIOX1	296.4575803	-0.26695048	0.033886244	-7.877842151	3.33083E-15	1.37773E-14
MLC1	2510.286526	-0.540018866	0.068525033	-7.880607167	3.25794E-15	1.34787E-14
COX11	378.1746932	-0.295165334	0.037453501	-7.880847593	3.25168E-15	1.34556E-14
CTBP2	1605.440889	-0.290211869	0.036821048	-7.881684199	3.22998E-15	1.33686E-14
LDHA	13103.93626	0.331547512	0.042063447	7.882081343	3.21972E-15	1.3329E-14
OR2AT4	4.314719729	-0.833499674	0.105739685	-7.882562513	3.20734E-15	1.32806E-14
FAIM	258.0668891	-0.336867896	0.042732357	-7.883204238	3.19091E-15	1.32153E-14
CYP2F1	22.10098011	-0.566542085	0.071865805	-7.883333198	3.18762E-15	1.32045E-14
CDCA2	284.1489549	-0.435554873	0.055248329	-7.883584519	3.18121E-15	1.31807E-14
PRR27	1.292935047	-1.309468346	0.166072645	-7.884912929	3.14755E-15	1.3044E-14
USP8	2388.307231	0.247981045	0.031449214	7.885126834	3.14216E-15	1.30245E-14
DCDC1	4.986643127	-0.941064999	0.119337616	-7.885736524	3.12686E-15	1.29638E-14

NUP98	10661.64465	0.293083781	0.037165601	7.885888374	3.12306E-15	1.29508E-14
GSTM5	74.52563329	-0.929931104	0.11791675	-7.886335922	3.11188E-15	1.29072E-14
KBTBD8	536.4814921	0.519203214	0.065832511	7.886729567	3.10209E-15	1.28693E-14
TSPYL2	3178.658522	0.467002887	0.059210426	7.887173174	3.09108E-15	1.28263E-14
WFDC3	8.416209649	-0.472595478	0.059915793	-7.887661226	3.07902E-15	1.2779E-14
MAP1S	1714.734926	0.339354785	0.043021903	7.887953836	3.07181E-15	1.27518E-14
MPHOSPH9	2769.650103	-0.263221074	0.033364462	-7.889264788	3.03972E-15	1.26212E-14
KCNA5	5.290807183	-1.101571974	0.139606642	-7.890541278	3.00879E-15	1.24954E-14
TMEM170A	1067.248491	0.226554848	0.028710354	7.891050436	2.99654E-15	1.24472E-14
ANXA1	21220.60291	0.589475116	0.074692572	7.892017922	2.97339E-15	1.23537E-14
PRRG3	46.59689976	-0.8891321	0.112656178	-7.892439754	2.96335E-15	1.23146E-14
NUP43	1241.086114	-0.300269136	0.038044755	-7.892523915	2.96136E-15	1.23089E-14
MLF1	75.63332871	-0.724686145	0.091814754	-7.892916014	2.95206E-15	1.22729E-14
ADM2	105.6543217	0.736826925	0.093351355	7.893050151	2.94889E-15	1.22623E-14
OR51I2	5.982833655	-0.780287774	0.098855929	-7.893181358	2.94579E-15	1.2252E-14
BBS9	405.5863109	-0.354699285	0.044936319	-7.893376535	2.94119E-15	1.22355E-14
CCDC28A	611.3352949	0.253681839	0.032136758	7.893821695	2.93071E-15	1.21945E-14
C6orf58	4.32701358	-0.631213352	0.079954781	-7.894629264	2.91179E-15	1.21183E-14
SULT1C2	8.605903123	-0.587852598	0.074454353	-7.89547653	2.89208E-15	1.20389E-14
TMEM104	1864.168048	0.398540185	0.05047317	7.896079958	2.87812E-15	1.19833E-14
NXPH3	22.39415251	-0.648159777	0.082073865	-7.897273695	2.8507E-15	1.18716E-14
AKAP13	12360.253	0.306609173	0.038822787	7.897660034	2.84188E-15	1.18374E-14
HK2	5601.299269	0.475539321	0.060204792	7.89869549	2.81837E-15	1.1742E-14
CCR1	523.066706	0.75050411	0.095011548	7.899083031	2.80963E-15	1.17081E-14
NAGLU	401.4910224	-0.234446688	0.029674648	-7.900571767	2.77627E-15	1.15715E-14
NUSAP1	51.6179897	-0.360872695	0.045675466	-7.900799375	2.7712E-15	1.15529E-14
AL357673.1	2.330875106	-0.650532085	0.082321121	-7.902371556	2.73646E-15	1.14105E-14
OR10A3	1.052028523	-1.229634654	0.155590786	-7.903004339	2.7226E-15	1.13551E-14
RSL24D1	4494.598652	0.328604496	0.041577537	7.903414283	2.71366E-15	1.13202E-14
PTDSS2	690.313292	-0.22590065	0.028571932	-7.906383428	2.64974E-15	1.10559E-14
NDUFA4L2	19.99267468	-0.506779952	0.064089148	-7.907422181	2.62773E-15	1.09664E-14
WDPCP	611.1073263	-0.254175098	0.032136963	-7.909120121	2.59214E-15	1.08202E-14
NHLH2	1.640364704	-1.208359136	0.152773644	-7.9094738	2.58479E-15	1.07918E-14
RWDD2A	44.32711838	0.509598255	0.06442199	7.91031533	2.56738E-15	1.07214E-14
LRRC10	3.588996967	-0.784105853	0.099123377	-7.910402922	2.56557E-15	1.07162E-14
MED18	160.4707224	-0.290126755	0.036674323	-7.910895916	2.55543E-15	1.06761E-14
SLC35F4	3.944814916	-0.611087308	0.07724102	-7.911434961	2.54439E-15	1.06322E-14

UNK	1412.016394	-0.159882065	0.020207654	-7.911955736	2.53376E-15	1.05901E-14
INKA1	236.4389133	-0.404237591	0.051084452	-7.913123712	2.5101E-15	1.04934E-14
CRTC2	3933.480373	0.342728001	0.043299521	7.915283816	2.4669E-15	1.0315E-14
CFH	183.3134291	-0.963113531	0.121668143	-7.915905571	2.4546E-15	1.02658E-14
ZNF141	902.9071752	-0.364014879	0.045978873	-7.917003027	2.43304E-15	1.01778E-14
HYKK	20.26625442	-0.512653251	0.064749279	-7.917512925	2.42308E-15	1.01383E-14
CSTF2	258.0961546	-0.239608304	0.030260658	-7.91814591	2.41078E-15	1.0089E-14
FGFR1	1941.291757	0.572345425	0.072272163	7.919306682	2.38839E-15	9.99741E-15
MROH7	78.88341692	-0.59233939	0.074790932	-7.919935946	2.37633E-15	9.94907E-15
TECR	1734.648668	-0.436757307	0.055135581	-7.92151448	2.34635E-15	9.82565E-15
PRMT1	2829.87039	-0.284745998	0.03594117	-7.922557726	2.32674E-15	9.74562E-15
PPIL4	1103.297239	0.251328795	0.031719346	7.92351768	2.30884E-15	9.67271E-15
HSD17B1	14.48268586	-0.401704167	0.050695255	-7.923900662	2.30174E-15	9.64501E-15
AHCYL1	2159.405528	-0.210247025	0.026532932	-7.924002765	2.29985E-15	9.63916E-15
F2	18.89594036	-0.574507738	0.072478259	-7.926621634	2.25188E-15	9.44015E-15
C16orf86	88.6201626	-0.308037496	0.038860804	-7.92668877	2.25066E-15	9.43707E-15
KCNJ8	3.949175068	-1.225935051	0.154656478	-7.926826373	2.24817E-15	9.42864E-15
ARL13A	21.40614307	-0.401761894	0.05068382	-7.926827479	2.24815E-15	9.42864E-15
PCLO	55.46197694	-1.353991841	0.170792049	-7.927721754	2.23203E-15	9.36494E-15
HMGXB4	1761.181134	0.251707887	0.031749641	7.927897147	2.22888E-15	9.35374E-15
ATAD3A	534.9331601	-0.302334471	0.03813094	-7.928849214	2.21186E-15	9.28431E-15
KIF5A	49.67511785	-0.474640158	0.059860179	-7.929146988	2.20656E-15	9.26406E-15
SYPL1	2693.25508	0.246144568	0.031039496	7.93004394	2.19068E-15	9.19937E-15
DYDC2	1.897468205	-0.979524444	0.123520398	-7.930062262	2.19036E-15	9.19937E-15
TBC1D23	1854.522855	0.318096962	0.040107407	7.931127563	2.17165E-15	9.12335E-15
PATE2	1.123679615	-1.078910938	0.136032903	-7.931249811	2.16951E-15	9.11633E-15
UBE2G1	2440.000442	0.231262828	0.029154317	7.932369876	2.15003E-15	9.0364E-15
ASCL2	91.00671188	-0.674060805	0.084958047	-7.934043092	2.12124E-15	8.91734E-15
ASCC3	1882.594314	-0.287978981	0.036291568	-7.935148588	2.10243E-15	8.84016E-15
AMFR	3365.859022	0.269153526	0.033918377	7.935330337	2.09936E-15	8.82912E-15
VWC2L	4.820660269	-1.116721144	0.14072082	-7.935720843	2.09276E-15	8.80328E-15
SSBP2	1835.026011	-0.3986373	0.050228021	-7.936551999	2.07879E-15	8.74639E-15
DEFB108B	2.798073681	-0.739520872	0.093168399	-7.937464631	2.06356E-15	8.68416E-15
TMEM229B	169.6810651	-0.521336216	0.065680061	-7.93751113	2.06279E-15	8.68277E-15
MRLN	3.969887607	-0.802318555	0.101072596	-7.938042437	2.05397E-15	8.64753E-15
OR10Z1	97.33583378	-0.88231488	0.111147108	-7.938262148	2.05034E-15	8.63409E-15
ZC3H12D	509.9937288	0.393391957	0.049552007	7.938971188	2.03865E-15	8.58673E-15

EPO	2.164588957	-0.68141145	0.085830643	-7.939023045	2.0378E-15	8.58499E-15
STK32A	1.089511308	-1.314724906	0.165586222	-7.939820657	2.02474E-15	8.5318E-15
LRRC17	21.32419883	-0.597535724	0.075254134	-7.940237862	2.01794E-15	8.50498E-15
DYNC1I2	1324.824508	-0.218529765	0.027517881	-7.941373196	1.99955E-15	8.42929E-15
QDPR	381.3035011	-0.352459446	0.044382522	-7.941401916	1.99909E-15	8.42916E-15
C2orf69	1071.557189	0.247332781	0.031140546	7.942467691	1.98198E-15	8.35882E-15
EFNA2	8.447805157	-0.836127581	0.10526522	-7.943056403	1.97259E-15	8.32102E-15
TUBGCP6	4097.110606	-0.256839142	0.032331794	-7.943856795	1.9599E-15	8.26926E-15
FBXO48	110.1501441	-0.398510739	0.050161741	-7.944515685	1.94951E-15	8.2272E-15
PPRC1	1773.543147	-0.304571325	0.038335383	-7.944914081	1.94325E-15	8.20257E-15
RANGRF	8.836316373	-0.39943226	0.050270997	-7.945580579	1.93283E-15	8.16034E-15
MRPL20	731.3091322	-0.262993239	0.033099201	-7.945606932	1.93242E-15	8.16034E-15
SLC12A3	27.92950986	-0.844384434	0.106260343	-7.946374044	1.9205E-15	8.11177E-15
IMP3	265.8048184	-0.365510903	0.045994855	-7.946778108	1.91425E-15	8.08711E-15
RBPMS2	171.8132113	-0.925126718	0.116402092	-7.947681214	1.90035E-15	8.03012E-15
SPAG16	56.22685926	-0.574527517	0.072283891	-7.948209579	1.89226E-15	7.99768E-15
FAM126B	2450.422448	0.30507445	0.038381025	7.948574891	1.88669E-15	7.97586E-15
PEA15	1183.333079	0.312523165	0.039315118	7.94918541	1.87742E-15	7.93837E-15
PRPF40B	101.9780253	-0.469035088	0.059001872	-7.949495076	1.87273E-15	7.92027E-15
TRAF1	1253.979809	0.516832788	0.064995535	7.951819877	1.83791E-15	7.77469E-15
TRNAU1AP	640.0597054	-0.229543	0.028866351	-7.951922851	1.83639E-15	7.76991E-15
ESPL1	838.4131048	-0.411899099	0.051779851	-7.954814271	1.794E-15	7.59223E-15
LDB1	4490.106876	-0.246570828	0.030993938	-7.955453336	1.78477E-15	7.55477E-15
SMG9	889.6174984	-0.215730579	0.027117036	-7.955536763	1.78357E-15	7.55131E-15
ANKRD2	2.84573926	-0.831833795	0.104550355	-7.95629816	1.77263E-15	7.50664E-15
SLC25A25	850.7926106	0.332243081	0.041754035	7.957148995	1.76049E-15	7.45683E-15
CDK18	230.2002994	-0.537870254	0.06759265	-7.957525753	1.75514E-15	7.43577E-15
FHIT	350.5116534	-0.443801926	0.055766034	-7.958283762	1.74442E-15	7.39197E-15
RBM45	218.4048089	-0.234852329	0.029507913	-7.958960928	1.7349E-15	7.35323E-15
VWA5A	227.6586949	-0.540375933	0.067891359	-7.959421397	1.72846E-15	7.3275E-15
FMO3	6.121016287	-0.929268049	0.116748958	-7.959540387	1.7268E-15	7.32204E-15
GGT6	11.34621001	-0.684611213	0.086004027	-7.96022278	1.7173E-15	7.28335E-15
NINL	54.43447124	-0.782393712	0.098260217	-7.962466799	1.68643E-15	7.15398E-15
TMEM41B	1159.978368	0.3203318	0.040229976	7.962515263	1.68577E-15	7.15273E-15
MAP4K1	998.2972373	-0.352050757	0.044209	-7.963327792	1.67473E-15	7.10744E-15
ASCL4	1.772117443	-1.355657089	0.170233032	-7.96353722	1.6719E-15	7.09695E-15
KHDRBS2	26.15940998	-0.728878133	0.0915215	-7.964009897	1.66552E-15	7.07141E-15

COMT	2279.868166	-0.418677079	0.052552261	-7.966870976	1.62742E-15	6.91117E-15
GATA1	554.9613222	-0.75222901	0.094414299	-7.967320854	1.62151E-15	6.88756E-15
TMEM170B	2073.105437	0.479323274	0.060158859	7.967625719	1.61752E-15	6.87208E-15
GSDMB	359.977282	-0.398169854	0.049972572	-7.967767926	1.61566E-15	6.86567E-15
SDAD1	588.2371727	-0.261520749	0.032821362	-7.968004256	1.61257E-15	6.85405E-15
BEST3	93.68995453	-0.914277637	0.114738221	-7.96837906	1.60769E-15	6.83478E-15
TLE3	8738.865031	0.373031089	0.046802155	7.970382698	1.58184E-15	6.72633E-15
DDX6	11467.88906	0.245685864	0.030821142	7.971342021	1.5696E-15	6.67576E-15
HLCS	401.292703	-0.317476648	0.039813441	-7.974107283	1.53486E-15	6.52942E-15
CLSPN	736.1222964	-0.402797977	0.050507468	-7.975018171	1.52358E-15	6.48285E-15
DPY30	269.5709151	-0.320741115	0.040217884	-7.975086813	1.52274E-15	6.48066E-15
ANKRD36	2305.434665	-0.391532365	0.049091745	-7.975523435	1.51736E-15	6.45919E-15
MVB12A	348.4430771	-0.331281975	0.041535303	-7.975913346	1.51258E-15	6.44023E-15
PCDHGB5	40.30724268	-0.764275561	0.095819467	-7.976203438	1.50903E-15	6.42652E-15
ANTKMT	233.0026836	-0.411872319	0.051631645	-7.977129448	1.49776E-15	6.37989E-15
BUB1B	604.386361	-0.375311347	0.047048346	-7.977142155	1.4976E-15	6.37989E-15
SPC25	104.4580034	-0.433728157	0.054369641	-7.97739601	1.49453E-15	6.36891E-15
CFAP298-TCP10L	28.15004229	-0.514771202	0.064520431	-7.978421598	1.48216E-15	6.31759E-15
CACNA1B	10.87187649	-0.764061638	0.095762014	-7.978754905	1.47817E-15	6.30193E-15
CCDC60	3.182613569	-0.751207961	0.094141744	-7.979541572	1.46878E-15	6.26326E-15
NOTO	2.650542103	-0.678471614	0.08501634	-7.98048483	1.4576E-15	6.21694E-15
SEPTIN4	57.87479667	-0.569286005	0.071332754	-7.980709771	1.45494E-15	6.20697E-15
SCN2B	3.296265453	-0.846596579	0.106076398	-7.98100798	1.45143E-15	6.19334E-15
GFRA4	1.264658365	-0.923183488	0.115669471	-7.981219931	1.44894E-15	6.18406E-15
LAMP1	6532.079057	0.241262031	0.030228033	7.981400232	1.44682E-15	6.17638E-15
LTA	49.04381114	0.615732133	0.077131506	7.982887455	1.42949E-15	6.10372E-15
DBF4	906.7688628	0.294121659	0.036838557	7.98407104	1.41584E-15	6.04677E-15
CCDC117	1145.957096	0.230998132	0.028927156	7.985511431	1.39941E-15	5.97788E-15
FURIN	7803.170842	0.357442267	0.04476072	7.985623637	1.39814E-15	5.97375E-15
RNASE10	3.901288596	-0.655885563	0.082129325	-7.986009382	1.39377E-15	5.95639E-15
SULF2	1267.392273	0.628806697	0.0787278	7.987098509	1.38152E-15	5.90531E-15
ERC2	2.059247633	-1.33577779	0.167216618	-7.988307676	1.36804E-15	5.84897E-15
ADAMTS14	181.7169044	-0.730621859	0.091447653	-7.989509141	1.35477E-15	5.79352E-15
TRMT112	1043.312273	-0.47935816	0.059997017	-7.989699842	1.35268E-15	5.78583E-15
C1QTNF3-AMACR	12.29736709	-0.539542935	0.067516656	-7.991256763	1.3357E-15	5.71446E-15
NOX5	14.9953194	-0.915522874	0.114559062	-7.991710649	1.33079E-15	5.6947E-15
FKBP4	1312.463924	-0.435231609	0.054460156	-7.991743649	1.33043E-15	5.69442E-15

TPM2	306.7625976	-0.452277971	0.056578723	-7.993781903	1.30861E-15	5.60224E-15
ARHGAP35	2442.474049	-0.275519971	0.034460654	-7.995204233	1.29359E-15	5.53916E-15
JPT1	1382.725182	-0.325535664	0.040710603	-7.99633602	1.28176E-15	5.4897E-15
ANK1	5745.824996	-0.85675592	0.107114875	-7.998477545	1.25967E-15	5.39626E-15
ZC3H13	1669.46522	-0.340249975	0.042539193	-7.998505561	1.25938E-15	5.39622E-15
LAMTOR2	553.7458014	-0.551295052	0.068923532	-7.998647755	1.25793E-15	5.39117E-15
ADGRA3	685.1290291	-0.507175748	0.063406139	-7.998842982	1.25594E-15	5.38381E-15
NUTM2D	94.17494038	-0.422938404	0.052867177	-8.000018726	1.244E-15	5.33382E-15
OR1M1	2.420522164	-0.718830053	0.089853128	-8.00005601	1.24363E-15	5.33337E-15
SNX4	941.5568928	0.215983908	0.026995488	8.00074088	1.23673E-15	5.30495E-15
ZNF317	2767.556727	0.23789348	0.029728064	8.002320072	1.22097E-15	5.23848E-15
MS4A10	2.318292527	-0.880069434	0.10996059	-8.003498659	1.20933E-15	5.1897E-15
MICOS13	495.8989786	-0.476302617	0.059511032	-8.003602089	1.20831E-15	5.18648E-15
VPS26A	1930.124292	0.250386743	0.031281562	8.004291517	1.20156E-15	5.15864E-15
CHST3	18.0926944	-0.795643612	0.099399777	-8.004480898	1.19972E-15	5.15183E-15
CGNL1	38.61600626	-0.900528464	0.112491767	-8.00528329	1.19192E-15	5.11947E-15
MRPL43	373.9012811	-0.371764054	0.046433407	-8.006391977	1.18123E-15	5.07467E-15
ST8SIA6	595.1312098	-0.76211996	0.095168819	-8.008084659	1.16509E-15	5.00642E-15
LDLRAD3	320.1370683	-0.85404151	0.106631263	-8.009297542	1.15365E-15	4.95839E-15
CUBN	285.3608025	-0.488244025	0.060958245	-8.009482916	1.15192E-15	4.95201E-15
SP7	27.05589808	-0.863999557	0.107845448	-8.011460583	1.13354E-15	4.87408E-15
TRIM72	166.9504084	-0.699042084	0.087252806	-8.011686026	1.13146E-15	4.86622E-15
WASF3	15.52456207	-0.918019238	0.114574602	-8.012414801	1.12478E-15	4.83852E-15
USP35	797.4307127	0.356765484	0.04452434	8.012819214	1.12108E-15	4.82369E-15
EFCAB13	319.5356482	-0.402966734	0.050284545	-8.013729368	1.11281E-15	4.78916E-15
TXLNB	50.12861643	-0.523804879	0.06536125	-8.013997218	1.11039E-15	4.77979E-15
SMCR8	5268.632232	0.264746159	0.03302468	8.016615517	1.08699E-15	4.68007E-15
POLR1E	604.8563365	-0.218320703	0.02723211	-8.017032249	1.08331E-15	4.66525E-15
CRISP2	13.95247788	-1.311014673	0.163508595	-8.018016853	1.07466E-15	4.62903E-15
METTL17	821.874591	-0.230843499	0.028783747	-8.019925283	1.0581E-15	4.55868E-15
PCDHGB4	39.78458484	-0.788363971	0.098298932	-8.020066483	1.05688E-15	4.55445E-15
NSMCE4A	524.0491499	-0.244111573	0.030437383	-8.020123473	1.05639E-15	4.55334E-15
ROMO1	922.0899709	-0.563362632	0.070237452	-8.020829556	1.05033E-15	4.52823E-15
TLK2	2874.320258	0.272949889	0.034029165	8.021057461	1.04839E-15	4.52084E-15
CFAP69	18.76173393	-0.773500163	0.09642464	-8.021810264	1.04198E-15	4.4942E-15
MYO15B	13686.92259	-0.364037821	0.045380099	-8.0219707	1.04062E-15	4.48932E-15
RGS5	16.24126922	-0.579070833	0.072182058	-8.022365273	1.03728E-15	4.47591E-15

NAV2	71.60697991	-0.667795751	0.083238949	-8.022635566	1.035E-15	4.46705E-15
BPIFB4	1.400628775	-0.91844359	0.114463439	-8.023903484	1.02437E-15	4.42213E-15
OR7G2	2.919610619	-0.658507542	0.082059446	-8.024762209	1.01723E-15	4.39228E-15
SEC23IP	1080.298981	-0.186630075	0.023256626	-8.024812859	1.01681E-15	4.39144E-15
GLDN	95.22739982	-0.894167442	0.111409199	-8.025974978	1.00723E-15	4.35102E-15
ZNF488	7.768598493	-0.931685997	0.116074515	-8.026619765	1.00195E-15	4.32918E-15
CYP3A4	6.382699275	-0.6179309	0.076981893	-8.026964207	9.99142E-16	4.318E-15
WWC1	29.10937779	-0.899695102	0.112079553	-8.027290266	9.96491E-16	4.30749E-15
MRPL2	60.5402452	-0.311233631	0.038769533	-8.027789089	9.92449E-16	4.29097E-15
CDK5R1	176.8246258	0.597664999	0.074444986	8.028277459	9.88507E-16	4.27487E-15
FAM20B	1679.408735	-0.273194509	0.034027192	-8.028711556	9.85016E-16	4.26071E-15
GDF3	1.815694198	-1.34244635	0.167193649	-8.029290338	9.80381E-16	4.2416E-15
CDC42EP4	800.9864419	0.519549498	0.06470037	8.030085483	9.74048E-16	4.21513E-15
RHO	6.43839594	-0.76483694	0.095227149	-8.031710999	9.61226E-16	4.16056E-15
CHKB-CPT1B	9.259864429	-0.466525572	0.058050471	-8.036551075	9.24024E-16	4.00043E-15
JRKL	155.8789159	-0.353749311	0.044012628	-8.037450379	9.1727E-16	3.97206E-15
COG2	515.198467	-0.259714789	0.032311871	-8.037751589	9.15019E-16	3.96319E-15
ANPEP	5051.622217	0.644933513	0.08023342	8.038215447	9.11563E-16	3.9491E-15
PRF1	388.9343045	0.626784972	0.077964384	8.039375676	9.02973E-16	3.91275E-15
NR5A1	35.68693595	-0.820894576	0.102098894	-8.040190713	8.96988E-16	3.88767E-15
PA2G4	1857.089315	-0.291055787	0.036199725	-8.040276223	8.96362E-16	3.88582E-15
GSTM4	370.4278404	-0.425718972	0.052946973	-8.040477942	8.94887E-16	3.88029E-15
CNTLN	452.9887989	-0.405704524	0.050457587	-8.040505805	8.94684E-16	3.88027E-15
MCM4	3259.18954	-0.331559725	0.041229415	-8.041824629	8.85105E-16	3.83957E-15
C1orf35	522.6029172	-0.323356202	0.040207382	-8.04220977	8.82327E-16	3.82837E-15
SPATA48	1.481889148	-1.222271125	0.151952648	-8.04376326	8.71208E-16	3.78096E-15
ANGPTL4	130.9841278	0.578091838	0.0718664	8.043979349	8.69672E-16	3.77514E-15
SLC17A3	2.708186784	-1.174482759	0.145993991	-8.04473357	8.64333E-16	3.75279E-15
RIOK2	391.8913527	0.245598182	0.030524788	8.045860426	8.56416E-16	3.71924E-15
SPIRE2	78.92955895	-0.404433457	0.050265465	-8.045950713	8.55784E-16	3.71732E-15
PUM3	780.252925	-0.268294138	0.033341336	-8.046892263	8.49229E-16	3.68967E-15
ISX	0.846090783	-1.387447358	0.172396856	-8.047985271	8.41681E-16	3.65769E-15
GEMIN7	35.45855104	-0.343233374	0.042647899	-8.048072264	8.41083E-16	3.6559E-15
ANK2	21.10045805	-0.802327197	0.099686066	-8.048539047	8.37882E-16	3.64279E-15
CFTR	1.784428958	-1.421801357	0.176642154	-8.04904902	8.34398E-16	3.62846E-15
OIT3	5.884374244	-0.838401475	0.104156605	-8.049431636	8.31794E-16	3.61793E-15
LRRC2	37.08357672	-0.759302887	0.094318856	-8.050382673	8.25356E-16	3.59073E-15

TEC	1770.441224	-0.344492055	0.042791667	-8.050447149	8.24921E-16	3.58963E-15
SAMD13	55.29406776	-0.463495163	0.05757223	-8.050672427	8.23404E-16	3.58383E-15
AC007906.2	4.078178337	-0.665152978	0.082617759	-8.050968566	8.21414E-16	3.57596E-15
CALHM1	8.002679461	-0.669402699	0.083138048	-8.051700969	8.16512E-16	3.55541E-15
ADAM11	104.2044223	-0.44373699	0.055103812	-8.052745837	8.09569E-16	3.52597E-15
CDIPT	824.1470402	-0.301061453	0.037380659	-8.053936479	8.01728E-16	3.4926E-15
BLNK	168.7200987	-0.655369163	0.081367143	-8.054469426	7.98243E-16	3.47819E-15
F13B	1.299075878	-1.318484399	0.163685683	-8.05497692	7.94938E-16	3.46456E-15
TAX1BP3	11.53916159	-0.61114372	0.075867424	-8.055416734	7.92085E-16	3.45289E-15
NAP1L3	18.99670632	-1.020276003	0.126636221	-8.056747048	7.83516E-16	3.4163E-15
SPNS3	1531.176493	-0.65721263	0.081569897	-8.057048632	7.81586E-16	3.40864E-15
RNF144A	1278.000613	-0.354139335	0.043952036	-8.057404605	7.79314E-16	3.39949E-15
DUS4L-BCAP29	162.6256743	0.318297353	0.039501002	8.057956349	7.75805E-16	3.38494E-15
RGS9	59.87454082	0.638020096	0.079175208	8.058331839	7.73426E-16	3.37531E-15
KCTD12	1779.55717	0.734127942	0.091079087	8.060334868	7.60857E-16	3.3212E-15
LRFN2	4.487539134	-1.176402915	0.145948124	-8.060418171	7.60339E-16	3.31968E-15
MUL1	847.376724	0.253374854	0.031431881	8.061078342	7.56243E-16	3.30253E-15
SFTPA2	1.86234995	-1.159668674	0.143857136	-8.061252341	7.55168E-16	3.29857E-15
ZNF174	145.1139061	-0.248605962	0.03082908	-8.064008601	7.38326E-16	3.22572E-15
PTPRT	1.803919918	-1.125235064	0.139517925	-8.065164843	7.31371E-16	3.19605E-15
RGPD3	31.0447913	0.501717652	0.062205292	8.065513943	7.29284E-16	3.18765E-15
CU639417.1	3.042251989	0.943719831	0.117006509	8.065532773	7.29172E-16	3.18765E-15
APOC1	46.00383903	-0.92606524	0.114807526	-8.066241571	7.24953E-16	3.17013E-15
UTP25	339.1614098	-0.340978251	0.04226722	-8.067203261	7.19267E-16	3.14597E-15
RCL1	850.8675297	-0.352316785	0.043667909	-8.068093752	7.14042E-16	3.12381E-15
FOXD4L4	1.731314455	-0.919508958	0.113946917	-8.06962563	7.0514E-16	3.08556E-15
RBMS3	27.95788733	-0.778694859	0.096495568	-8.069747399	7.04438E-16	3.08317E-15
RERG	1.44465736	-1.015109731	0.125791724	-8.069765606	7.04333E-16	3.08317E-15
TNPO2	2907.217616	-0.218757353	0.027108264	-8.069766303	7.04329E-16	3.08317E-15
PHKG2	1070.73174	-0.217413248	0.026937713	-8.070961523	6.97467E-16	3.05471E-15
VPS4B	1711.584676	0.19845882	0.024586761	8.071775727	6.92831E-16	3.03509E-15
CAMK2G	2028.478592	-0.247978557	0.030719576	-8.07233002	6.89692E-16	3.02201E-15
CBLB	1531.065886	0.299329192	0.037079476	8.072638233	6.87952E-16	3.01507E-15
LUZP1	5512.765046	0.443682424	0.054958429	8.073055116	6.85606E-16	3.00546E-15
FJX1	7.688054699	-0.994472296	0.123142723	-8.075769933	6.70522E-16	2.93999E-15
ZNF519	235.1600074	-0.369632994	0.045767131	-8.076385577	6.67147E-16	2.92585E-15
TRPM1	5.600032888	-0.727213664	0.090037186	-8.076814697	6.64805E-16	2.91623E-15

TRMT12	235.9737536	-0.266919925	0.033047279	-8.076910814	6.64281E-16	2.91459E-15
AC008770.2	7.604577174	-0.494279886	0.061190621	-8.077706678	6.59961E-16	2.89628E-15
CHD2	18498.11204	0.389911678	0.048263703	8.078776727	6.54197E-16	2.87163E-15
SNRNP25	401.8850446	-0.386685284	0.047860156	-8.079482279	6.50423E-16	2.8557E-15
NBR1	1691.528503	-0.285953423	0.035389063	-8.080276759	6.462E-16	2.8378E-15
GIMAP4	119.8432939	0.71128527	0.088023495	8.080629734	6.44332E-16	2.83023E-15
TBRG4	1579.406453	-0.224902514	0.027832021	-8.080710819	6.43903E-16	2.82898E-15
ZBTB34	2470.155559	0.304785966	0.037717082	8.080846934	6.43185E-16	2.82646E-15
TWSG1	1006.180531	0.384429652	0.0475707	8.081227504	6.41181E-16	2.81829E-15
RASAL1	227.245133	1.003455104	0.124166695	8.081515752	6.39667E-16	2.81226E-15
CYP2R1	472.9286365	0.255838017	0.031653635	8.082421375	6.34933E-16	2.79208E-15
SLC17A5	699.7179616	0.342932874	0.042429035	8.082504607	6.34499E-16	2.7908E-15
RHEB	1671.249919	0.238011721	0.029440395	8.084528776	6.2405E-16	2.74546E-15
SIK1	141.625628	0.778073525	0.096240527	8.084676515	6.23294E-16	2.74275E-15
PAPPA2	6.695027197	-0.662008924	0.081883667	-8.084749347	6.22922E-16	2.74172E-15
GPRASP1	262.1684857	0.524533945	0.064870796	8.085825599	6.17445E-16	2.71823E-15
PRORP	255.1939775	-0.263475896	0.032578611	-8.087388811	6.09574E-16	2.68418E-15
PRKAR2A	2073.849882	0.240881598	0.029784365	8.087518433	6.08926E-16	2.68193E-15
ALDH3B2	4.033117586	-0.728976607	0.090123843	-8.088609903	6.03495E-16	2.65861E-15
STX8	496.1549256	-0.354052492	0.043762869	-8.090248631	5.95431E-16	2.62367E-15
RBP2	23.32384262	-0.578221427	0.071468819	-8.090541217	5.94002E-16	2.61797E-15
NEDD1	688.7581608	0.235414683	0.029096086	8.090939887	5.92061E-16	2.61E-15
APEX2	450.9963928	-0.308567343	0.038134695	-8.091512044	5.89286E-16	2.59835E-15
ARID5B	1364.600519	0.503879117	0.062271947	8.091590804	5.88905E-16	2.59726E-15
EIF2B5	1418.999079	-0.162503755	0.020080339	-8.092679871	5.83661E-16	2.57471E-15
RSRP1	5516.515715	-0.331513808	0.040960049	-8.093589091	5.79319E-16	2.55613E-15
TMEM145	12.82417039	-0.716389729	0.088512349	-8.093669845	5.78935E-16	2.55501E-15
NFAT5	9562.187526	0.404484084	0.049974714	8.093774842	5.78436E-16	2.55339E-15
DCAF10	1051.915639	-0.215540224	0.026630152	-8.093841322	5.7812E-16	2.55257E-15
NSG2	5.890149083	-1.105198947	0.136537293	-8.094484093	5.75076E-16	2.5397E-15
GOLGA3	3311.575647	-0.242395396	0.029929288	-8.098936234	5.54418E-16	2.44903E-15
CDC42EP3	2709.968698	0.489242478	0.060398555	8.100234842	5.48532E-16	2.42357E-15
HLA-C	26185.87747	0.368568618	0.04549988	8.100430575	5.4765E-16	2.42022E-15
BTG3	1233.105284	0.466525722	0.057589178	8.100926961	5.4542E-16	2.41091E-15
C14orf132	7.01156192	-0.949617165	0.117213225	-8.101621351	5.42315E-16	2.39773E-15
ZNF778	376.6156066	-0.32519026	0.040123318	-8.10476993	5.28454E-16	2.33697E-15
FIG4	816.1491472	-0.288466042	0.035582999	-8.106850136	5.19489E-16	2.29784E-15

ILVBL	842.7694971	-0.229909377	0.028352004	-8.109104946	5.0994E-16	2.25612E-15
SPAG9	7395.638296	0.368813281	0.045479992	8.109352324	5.08903E-16	2.25204E-15
POLR3D	1079.888745	-0.252141674	0.031086578	-8.110949772	5.02256E-16	2.22313E-15
ZNF687	3584.443759	0.259972366	0.032051912	8.110978473	5.02138E-16	2.2231E-15
MDM1	500.4783934	-0.282011476	0.03476895	-8.111015123	5.01986E-16	2.22294E-15
WNT9B	7.190083062	-0.729361724	0.08991487	-8.111691944	4.99197E-16	2.21109E-15
DARS2	952.2447406	-0.365993652	0.045113867	-8.112664212	4.95218E-16	2.19396E-15
COLEC11	6.162561258	-0.792627371	0.097701574	-8.112738973	4.94913E-16	2.1931E-15
CPOX	624.9130727	-0.535455368	0.065994008	-8.113696758	4.91026E-16	2.17637E-15
NBAS	3338.152869	-0.340628345	0.041981394	-8.113793084	4.90637E-16	2.17514E-15
CCDC168	27.53195954	-0.600536703	0.074014209	-8.113802936	4.90597E-16	2.17514E-15
C2orf83	2.905163031	-1.085883176	0.133825592	-8.114166784	4.89129E-16	2.16944E-15
GP9	89.41199049	-0.882188864	0.108717632	-8.114496651	4.87802E-16	2.16404E-15
CYP4B1	1.587042528	-1.325388115	0.163326994	-8.114936053	4.86041E-16	2.15672E-15
GPR137C	50.13146723	-0.512059249	0.063097937	-8.115308891	4.84551E-16	2.15059E-15
C1orf100	12.25665586	-0.641921198	0.079090971	-8.116238684	4.80855E-16	2.13467E-15
AMER1	447.9356268	-0.309250319	0.038099511	-8.116910384	4.78202E-16	2.12338E-15
ACSBG1	56.12891668	-0.539908233	0.066499738	-8.118952732	4.70224E-16	2.08842E-15
B4GALNT1	3.147001772	-0.778463603	0.095870349	-8.11996215	4.66329E-16	2.0716E-15
ITLN1	52.97368532	-0.983934415	0.121172256	-8.120129568	4.65686E-16	2.06921E-15
ISG20	1066.372019	0.530852197	0.065371882	8.120497345	4.64277E-16	2.06342E-15
ASPM	1062.56975	-0.469518389	0.057817104	-8.120752497	4.63302E-16	2.05955E-15
F2R	486.5170358	-0.612083536	0.075367334	-8.121337249	4.61075E-16	2.05012E-15
DOC2A	93.01002422	-0.585135712	0.072046811	-8.121604596	4.6006E-16	2.04607E-15
ABAT	368.2298878	-0.32718272	0.040282361	-8.122233026	4.57683E-16	2.03596E-15
PLPP4	8.915523573	-0.971382943	0.119577097	-8.123486571	4.52979E-16	2.01549E-15
SORD	461.0317603	-0.365793919	0.045028102	-8.123680604	4.52255E-16	2.01273E-15
TRIM75P	3.122149217	-0.926069849	0.113974869	-8.125210942	4.46585E-16	1.98795E-15
RAPH1	55.29223614	0.629933503	0.077525904	8.125458339	4.45675E-16	1.98435E-15
PXK	3890.136552	0.277519242	0.034151573	8.126104197	4.43308E-16	1.97426E-15
HLA-A	23599.04138	0.30910351	0.038037685	8.126244015	4.42797E-16	1.97243E-15
BAZ2B	3842.097898	-0.318485747	0.039192119	-8.126270088	4.42702E-16	1.97243E-15
ACTG2	4.574683761	-0.684960033	0.08428798	-8.126425978	4.42133E-16	1.97037E-15
ZNF781	10.49658025	-0.653027424	0.080341702	-8.128125362	4.3598E-16	1.94339E-15
SIGLECL1	3.703103771	-0.744382028	0.091575917	-8.128578503	4.34354E-16	1.93658E-15
TIA1	2031.699421	-0.235570523	0.02897887	-8.129044534	4.32687E-16	1.92959E-15
DRD5	8.298849294	-0.897733318	0.110427383	-8.12962591	4.30617E-16	1.9208E-15

PROX2	82.79116094	-0.542494483	0.066706204	-8.132594135	4.202E-16	1.87476E-15
RAMP2	4.534148176	-0.957848421	0.117762114	-8.133757049	4.16186E-16	1.85728E-15
ETNPPL	1.227652969	-1.216272062	0.149516874	-8.134680921	4.13025E-16	1.84359E-15
REEP5	971.4299708	0.432080461	0.053113485	8.135042557	4.11794E-16	1.83851E-15
SEC24D	1612.153356	0.324984672	0.039940045	8.136812813	4.05819E-16	1.81225E-15
SULT1E1	6.156780596	-1.055565991	0.129723773	-8.137028133	4.05099E-16	1.80945E-15
ITPRIPL1	112.1894856	-0.42447508	0.052160435	-8.137874631	4.02277E-16	1.79725E-15
LRRN2	206.6978515	-0.512732423	0.062993828	-8.139407288	3.97218E-16	1.77505E-15
SDHAF1	135.605104	-0.321543663	0.039501901	-8.139954189	3.95427E-16	1.76746E-15
PCGF2	27.51115282	-0.75711047	0.093007735	-8.140295744	3.94314E-16	1.76288E-15
RPRD1B	2800.83528	0.355988349	0.04373053	8.140499296	3.93651E-16	1.76032E-15
OTOGL	3.0888232	-1.111830375	0.136579431	-8.140540376	3.93518E-16	1.76013E-15
RBM10	2316.97311	-0.189291897	0.023250506	-8.14140971	3.90702E-16	1.74793E-15
LGALS3	1577.215065	0.660647347	0.081145149	8.141550757	3.90247E-16	1.7463E-15
MED23	2963.605628	0.303771076	0.037310074	8.141797649	3.89452E-16	1.74314E-15
FAM72C	9.612302958	-0.634851454	0.077962449	-8.143041447	3.8547E-16	1.72571E-15
TMEM30A	3949.004532	0.24994047	0.030692127	8.143471817	3.84102E-16	1.71998E-15
SAG	42.69881518	-0.613893887	0.075379412	-8.144052426	3.82264E-16	1.71214E-15
CLDN4	10.62937808	-0.648696827	0.07964544	-8.14480814	3.79884E-16	1.70187E-15
UBE2T	147.0062972	-0.530035444	0.065072261	-8.145336158	3.7823E-16	1.69485E-15
NOXRED1	40.96839897	-0.33893423	0.041600501	-8.14735935	3.71957E-16	1.66712E-15
C1orf112	307.883603	-0.363652939	0.044632138	-8.147782113	3.70659E-16	1.66169E-15
TMEM39B	623.7822737	-0.244417576	0.029992443	-8.149305443	3.6602E-16	1.64126E-15
TMEM185B	619.1957485	-0.29410199	0.036087591	-8.149670839	3.64916E-16	1.63669E-15
AKR1E2	160.2072637	-0.588363833	0.072193885	-8.14977377	3.64606E-16	1.63567E-15
BECN1	1320.938984	0.173108826	0.02123998	8.150140695	3.63501E-16	1.63109E-15
NFE2L2	2561.734686	0.287271577	0.035246383	8.15038456	3.62769E-16	1.62818E-15
SLC17A4	1.096408906	-1.344713683	0.16492141	-8.153663492	3.53062E-16	1.58497E-15
PP2D1	153.7617801	0.394969914	0.0484325	8.155059378	3.49008E-16	1.56713E-15
SMC2	914.396412	-0.367809307	0.045095078	-8.156307074	3.45424E-16	1.55139E-15
RAB23	29.17287462	-0.603542429	0.073980637	-8.158113489	3.40298E-16	1.52872E-15
G6PC	4.983906159	-0.723719477	0.088674626	-8.161517068	3.30843E-16	1.48659E-15
SLC25A27	11.64821263	-0.896356974	0.109809028	-8.162871406	3.27153E-16	1.47035E-15
LDB3	12.43737119	-0.624258004	0.076474547	-8.162951297	3.26936E-16	1.46971E-15
PTPN23	2207.407822	-0.218704262	0.02679084	-8.163397016	3.25732E-16	1.46463E-15
APLP2	21184.70669	0.382585499	0.046863354	8.163852298	3.24506E-16	1.45946E-15
SPX	25.81726944	-0.855465816	0.104779494	-8.164439277	3.22932E-16	1.45271E-15

POLD4	168.4849921	-0.312432428	0.038262291	-8.165544204	3.19989E-16	1.4398E-15
TNFRSF9	142.8981928	0.626356516	0.076690499	8.167328765	3.15293E-16	1.419E-15
SIX5	11.79689994	-0.708715895	0.086770515	-8.167704182	3.14313E-16	1.41492E-15
UFSP1	20.14613423	-0.504794697	0.061791075	-8.169378756	3.09982E-16	1.39574E-15
GSS	898.8528358	-0.241219647	0.029520165	-8.171351503	3.04954E-16	1.37341E-15
PRDX1	1205.427059	-0.504894434	0.06177989	-8.172472169	3.02133E-16	1.36103E-15
RND2	33.95932947	-0.447134952	0.054707718	-8.173160304	3.00414E-16	1.35359E-15
TEFM	122.6563301	-0.232081762	0.028393359	-8.173804421	2.98814E-16	1.34669E-15
MDN1	6878.337791	-0.320224391	0.03917465	-8.174275745	2.97648E-16	1.34175E-15
APOL3	770.8199109	0.458340029	0.056067754	8.174752753	2.96473E-16	1.33676E-15
RAB43	226.2057979	0.475481407	0.058164482	8.174772458	2.96425E-16	1.33676E-15
TMEM33	2169.465196	0.2424025	0.029651499	8.17505037	2.95742E-16	1.33408E-15
ZRANB1	1261.904422	0.310487437	0.03797932	8.175170974	2.95447E-16	1.33305E-15
PRIM1	255.2467326	-0.348221568	0.042589323	-8.17626443	2.92779E-16	1.32132E-15
HOGA1	3.893303776	-0.62607371	0.076571475	-8.176330849	2.92618E-16	1.3209E-15
LAMTOR3	867.2360813	0.267736142	0.032741891	8.177174058	2.90578E-16	1.31199E-15
TCEA1	2648.136417	0.211642416	0.025880316	8.177737031	2.89224E-16	1.30618E-15
ZNF705E	12.81202529	-0.444064005	0.054298547	-8.178193122	2.88132E-16	1.30155E-15
ZNF30	50.20949391	-0.503327493	0.061537566	-8.179190787	2.85756E-16	1.29111E-15
POU4F2	1.417383011	-1.528518579	0.186876013	-8.179319282	2.85452E-16	1.29004E-15
FBXO28	1035.470777	0.248798936	0.030412115	8.180915258	2.81696E-16	1.27336E-15
MEIG1	4.4552587	-0.651254511	0.079581776	-8.183462865	2.75801E-16	1.247E-15
CCDC163	63.33643603	-0.437923312	0.053499844	-8.185506401	2.71161E-16	1.2263E-15
RIN1	217.4407819	0.574153159	0.070140566	8.185750351	2.70612E-16	1.2241E-15
PRB2	9.442619658	-0.734478001	0.089725662	-8.185818685	2.70459E-16	1.22369E-15
C12orf54	1.689487082	-1.008499261	0.123184696	-8.18688761	2.68068E-16	1.21316E-15
WDR62	907.2479692	-0.344406607	0.042060206	-8.188419393	2.64679E-16	1.1981E-15
STK3	439.9597105	-0.28239805	0.034485595	-8.188869769	2.63691E-16	1.1939E-15
ZPBP	2.805263792	-0.748719674	0.091427906	-8.189181063	2.6301E-16	1.19109E-15
GABRG2	2.242276229	-1.066621304	0.130245651	-8.189304547	2.6274E-16	1.19014E-15
TOMM40	1404.567595	-0.295214851	0.036046289	-8.189881968	2.61482E-16	1.18472E-15
C1orf109	298.6190277	-0.400245579	0.04886834	-8.190283953	2.60611E-16	1.18104E-15
PITX2	1.140207127	-1.187156443	0.144937529	-8.190814672	2.59464E-16	1.17612E-15
CRBN	1858.951591	0.229051965	0.027961555	8.191674936	2.57616E-16	1.16801E-15
NTN5	24.42233646	-0.416084751	0.05078424	-8.193186492	2.544E-16	1.1537E-15
THUMPD2	844.2232802	0.27352481	0.033380589	8.194127813	2.52417E-16	1.14497E-15
DYNC1LI1	1329.873803	0.187311035	0.022855198	8.195555061	2.49439E-16	1.13173E-15

SERPINE3	28.68821511	-0.805867192	0.098317707	-8.196562107	2.4736E-16	1.12255E-15
SIPA1L3	1787.6551	-0.312836463	0.038159167	-8.198199399	2.44014E-16	1.10763E-15
OSER1	2158.008218	0.317091363	0.038675829	8.198696029	2.43009E-16	1.10332E-15
C8orf82	472.2822187	-0.360354349	0.043947766	-8.199605686	2.41177E-16	1.09526E-15
PCDHB16	3.166587972	-1.198433279	0.146153488	-8.199826721	2.40734E-16	1.0935E-15
CLEC18B	17.16037647	-0.616943528	0.075236533	-8.200052605	2.40282E-16	1.0917E-15
MYH7	2.610703081	-0.977792473	0.119224962	-8.201239512	2.37921E-16	1.08122E-15
MCM3AP	3254.843252	-0.255694433	0.03117409	-8.202145842	2.36134E-16	1.07335E-15
DCTN1	2698.982302	-0.225805342	0.027529587	-8.202278681	2.35873E-16	1.07241E-15
NABP2	462.1998287	-0.331997392	0.040474074	-8.202717369	2.35013E-16	1.06875E-15
THAP10	4.980961366	-1.049009755	0.127844147	-8.205379586	2.29863E-16	1.04558E-15
SMPDL3A	82.87329301	0.943073764	0.114901656	8.207660317	2.2554E-16	1.02615E-15
KLHL32	12.86127843	-0.603480943	0.073520709	-8.208312365	2.24319E-16	1.02083E-15
HEATR5B	3465.10321	-0.306184089	0.037297295	-8.209284033	2.22511E-16	1.01284E-15
MXRA8	136.151434	-0.417430575	0.050840293	-8.210624952	2.2004E-16	1.00183E-15
ZFHX4	14.36474507	-0.961147939	0.117035731	-8.212431654	2.16753E-16	9.87091E-16
TEAD3	61.97417821	-0.702959759	0.085596767	-8.212456931	2.16708E-16	9.87091E-16
ZNF135	88.07677102	-0.675041993	0.082190248	-8.213164046	2.15435E-16	9.81543E-16
THY1	8.904804641	-0.816026116	0.099321164	-8.216034594	2.10343E-16	9.58566E-16
CEMIP2	1532.940329	0.46608573	0.056713268	8.218283817	2.06436E-16	9.40981E-16
FNDC8	4.800036935	-0.536849347	0.065318725	-8.218919621	2.05345E-16	9.36224E-16
RHEBL1	79.97361851	-0.52723453	0.064124933	-8.22198963	2.00154E-16	9.12774E-16
SERPINI2	115.1573227	-0.799904814	0.097261409	-8.22427744	1.96371E-16	8.95728E-16
CAPZA2	2924.169591	0.270481043	0.032885105	8.22503211	1.95139E-16	8.90314E-16
FAAP24	70.88463614	-0.313410575	0.038100116	-8.225974247	1.93611E-16	8.83549E-16
CCNG1	4823.4592	0.359655645	0.043721132	8.22612844	1.93362E-16	8.82618E-16
MITD1	247.2827527	-0.315667915	0.038369653	-8.227020249	1.91928E-16	8.76279E-16
CRLF1	14.91839328	-0.399798638	0.04859383	-8.227353999	1.91394E-16	8.74045E-16
ARMC10	588.8616918	-0.307029678	0.037315991	-8.22783128	1.90633E-16	8.70774E-16
FAM72D	16.81966903	-0.52692054	0.064040728	-8.227897359	1.90528E-16	8.70497E-16
SIPA1	4117.035538	-0.307202049	0.03733153	-8.229023811	1.88745E-16	8.62552E-16
MAP7D1	5702.035874	0.316688149	0.03848039	8.229857998	1.87436E-16	8.56767E-16
CEACAM18	2.071949615	-0.950205915	0.115446885	-8.230676107	1.8616E-16	8.51134E-16
SLC9C2	6.425907905	-0.869897672	0.1056535	-8.233496056	1.81827E-16	8.3152E-16
PDGFB	28.65060566	0.816249335	0.099136336	8.23360402	1.81663E-16	8.30965E-16
MEA1	432.3260749	-0.386472048	0.046937595	-8.233741969	1.81454E-16	8.30202E-16
TCAF1	269.8428051	-0.354594257	0.043058862	-8.235105168	1.794E-16	8.20995E-16

CMSS1	320.6506603	-0.336783167	0.040893997	-8.235516105	1.78785E-16	8.18373E-16
ROS1	3.18472597	-1.399910719	0.169979088	-8.235782056	1.78388E-16	8.16748E-16
FTSJ3	1128.133687	-0.239396069	0.029061243	-8.23764043	1.7564E-16	8.04353E-16
SLFN14	306.7974868	-0.747275628	0.09071219	-8.237874433	1.75297E-16	8.02969E-16
CYP2W1	22.78882661	-0.585591085	0.071072553	-8.239342253	1.7316E-16	7.93366E-16
HACD3	1142.834908	-0.256936981	0.031182669	-8.239736588	1.7259E-16	7.90941E-16
SNRPD3	1136.460871	-0.23023766	0.027941704	-8.239929064	1.72313E-16	7.89854E-16
CYP2E1	83.25877935	-0.679680003	0.082468662	-8.241676108	1.69815E-16	7.78586E-16
RTN4	7797.128422	0.224225049	0.027200522	8.243409807	1.67371E-16	7.67562E-16
MCFD2	1989.89789	0.223272689	0.027083625	8.243825877	1.6679E-16	7.65076E-16
MIDEAS	10276.64587	0.35137105	0.042620713	8.244138192	1.66355E-16	7.63259E-16
SMARCD3	323.1290638	0.482884463	0.058571267	8.244391626	1.66003E-16	7.61822E-16
RAB26	144.4453301	-0.776326738	0.094160138	-8.244749349	1.65507E-16	7.59724E-16
RFXANK	569.1770466	-0.322695263	0.039136853	-8.245304391	1.6474E-16	7.56383E-16
AL391650.1	37.59023783	-0.464202315	0.056296971	-8.24560026	1.64333E-16	7.54691E-16
STC2	3.741178552	-1.106460656	0.134179279	-8.246136571	1.63598E-16	7.5149E-16
SLC4A8	291.2085101	-0.476948547	0.05783421	-8.246823967	1.6266E-16	7.47357E-16
ABCA2	3291.120534	-0.449716907	0.05452794	-8.247458178	1.61799E-16	7.43577E-16
ZNF613	100.4776684	-0.341291634	0.041378073	-8.248127741	1.60895E-16	7.39597E-16
RNF149	2665.790328	0.335692212	0.040698826	8.248203778	1.60793E-16	7.393E-16
ITM2B	9201.465144	0.348996017	0.042309698	8.248605648	1.60253E-16	7.36992E-16
SLC5A2	19.10401767	-0.497221145	0.060271871	-8.249638446	1.58874E-16	7.30822E-16
DTWD1	520.481837	-0.286235185	0.034695513	-8.249919476	1.58501E-16	7.29277E-16
DOLPP1	279.0586648	-0.32869306	0.039832705	-8.251838815	1.55976E-16	7.17826E-16
PCBP2	9593.433388	0.203029883	0.024596128	8.25454636	1.5248E-16	7.01904E-16
ATP1A2	7.782136455	-0.814962857	0.098720902	-8.255220961	1.51621E-16	6.98115E-16
G0S2	1425.79589	0.956143142	0.115799023	8.256918919	1.49481E-16	6.88421E-16
TADA3	1240.730977	-0.312164096	0.037796587	-8.259055084	1.4683E-16	6.76373E-16
GJD4	2.567587761	-1.043756531	0.12637465	-8.259223895	1.46623E-16	6.75576E-16
SLC52A2	388.0558932	-0.388498967	0.047037981	-8.259261154	1.46577E-16	6.75524E-16
C1orf127	42.41559627	-0.678060919	0.082086577	-8.260314217	1.45289E-16	6.69748E-16
ACOT12	12.44316381	-0.831635364	0.100647967	-8.262813335	1.42279E-16	6.56024E-16
CTU1	156.6591724	-0.335025958	0.040543645	-8.263340778	1.41651E-16	6.53284E-16
GLIS1	2.079756786	-0.91927862	0.111247006	-8.263401013	1.4158E-16	6.53108E-16
KLC4	485.4235367	-0.251094166	0.030384459	-8.26390113	1.40987E-16	6.5053E-16
B4GALNT2	2.259278572	-0.833563231	0.100863491	-8.264271057	1.40551E-16	6.48669E-16
LLCFC1	28.49609481	-0.917595432	0.111026459	-8.264655505	1.40099E-16	6.46734E-16

SCARA5	8.693617101	-1.260635116	0.152527737	-8.264956553	1.39746E-16	6.45256E-16
NEUROD1	1.677719313	-1.722592285	0.208400782	-8.265766876	1.388E-16	6.41039E-16
CKS1B	414.3245923	-0.421138922	0.050948945	-8.265900706	1.38644E-16	6.40471E-16
SS18L1	555.7556193	-0.293724913	0.035534183	-8.26598197	1.38549E-16	6.40186E-16
PCDHB6	1.620398488	-1.489553447	0.180169021	-8.26753368	1.36759E-16	6.32061E-16
C3orf56	7.723515995	-1.432573892	0.173256813	-8.268499615	1.35655E-16	6.2711E-16
PDE2A	231.9718401	0.638826719	0.077256207	8.268937171	1.35158E-16	6.2496E-16
CYYR1	34.26556439	-1.330643169	0.160911692	-8.269400152	1.34635E-16	6.22685E-16
RANBP2	11982.46982	0.378957923	0.045824105	8.269837891	1.34141E-16	6.2055E-16
SLC6A1	6.334810288	-0.694303258	0.083953516	-8.270091476	1.33856E-16	6.19378E-16
CMAS	580.3623059	-0.375183826	0.045365742	-8.270201501	1.33733E-16	6.18953E-16
RAB38	78.6707996	-0.618325491	0.074764618	-8.270295587	1.33627E-16	6.18611E-16
LOXL1	63.516272	0.778424053	0.094121788	8.270391702	1.3352E-16	6.18259E-16
NUDT9	180.8811418	-0.346670455	0.041912957	-8.271200025	1.32617E-16	6.14226E-16
ZNF706	2892.95091	0.272318515	0.032921002	8.271878098	1.31865E-16	6.10887E-16
TFAP2E	133.2015325	-0.336372035	0.040663392	-8.272109613	1.31609E-16	6.09845E-16
CCT7	3089.663169	-0.360540796	0.043584509	-8.272223444	1.31484E-16	6.09408E-16
SREK1	8609.572209	0.322139492	0.038941508	8.272393798	1.31296E-16	6.08681E-16
TCP10L	5.835045748	-0.592266653	0.071577825	-8.274443238	1.29057E-16	5.98445E-16
SLIT3	6.894549678	-0.793184492	0.09585419	-8.274906799	1.28556E-16	5.96263E-16
MRPS12	375.0955081	-0.458467063	0.055400006	-8.275577823	1.27834E-16	5.93055E-16
GPAA1	2083.561287	-0.311336457	0.03761516	-8.276887782	1.26436E-16	5.86709E-16
IL4I1	182.1931843	0.689987339	0.08336033	8.27716658	1.26141E-16	5.85476E-16
WIZ	1356.018287	-0.23849147	0.028811204	-8.277733517	1.25542E-16	5.82834E-16
CCDC159	543.802371	-0.349171984	0.042169837	-8.280135961	1.23035E-16	5.71331E-16
COL19A1	126.6551283	0.708331784	0.085542799	8.28043732	1.22724E-16	5.70022E-16
HOXC11	1.475928529	-1.197061071	0.144562582	-8.280573378	1.22584E-16	5.69506E-16
MRPS18B	676.207671	-0.321042433	0.038769703	-8.280755593	1.22396E-16	5.6877E-16
CHRNA1	2.813698943	-0.993768977	0.120007438	-8.28089487	1.22253E-16	5.6824E-16
CCDC186	1144.419631	0.346376149	0.041810464	8.284436865	1.18669E-16	5.51713E-16
CTNNA2	3.311333307	-1.476174811	0.17816199	-8.285576564	1.17538E-16	5.46585E-16
NAALADL2	67.69254284	-0.71857689	0.086719744	-8.286197079	1.16927E-16	5.43872E-16
C12orf56	8.359621812	-1.324732897	0.159869976	-8.286314484	1.16812E-16	5.43464E-16
RCC1L	848.4735474	-0.201813993	0.024353445	-8.286876682	1.16261E-16	5.41031E-16
PABPC3	153.9024292	0.448100609	0.054071141	8.287241599	1.15905E-16	5.39503E-16
COMMD2	607.7143522	0.243261215	0.029352794	8.287497664	1.15656E-16	5.38471E-16
CAST	3456.120658	0.389128481	0.046952224	8.28775398	1.15407E-16	5.3744E-16

BRD3	1477.480628	-0.194041881	0.023403082	-8.291295855	1.12021E-16	5.21797E-16
TAPBPL	792.344467	-0.279263066	0.033673309	-8.293306412	1.10143E-16	5.13171E-16
PON1	4.690114273	-1.356420907	0.163515107	-8.295385889	1.08233E-16	5.04393E-16
AGPAT1	380.1293709	-0.41906967	0.0505177	-8.295501863	1.08128E-16	5.04021E-16
RAP1A	1786.503785	0.27728807	0.033425153	8.295790592	1.07865E-16	5.02918E-16
SYS1	1001.01006	0.27327071	0.032928571	8.29889357	1.05085E-16	4.90073E-16
NOD2	774.4522605	0.636504268	0.076686911	8.300037887	1.04078E-16	4.85492E-16
AHSG	3.725606018	-1.027763984	0.123821565	-8.300363351	1.03793E-16	4.84279E-16
BTN3A2	861.4204193	-0.430484305	0.051859327	-8.301000677	1.03238E-16	4.81802E-16
ZNF716	1.442088762	-0.955056925	0.115045135	-8.301584659	1.02732E-16	4.79554E-16
VSIG10	234.3361352	-0.46829197	0.056405762	-8.302200971	1.022E-16	4.77186E-16
RTBDN	3.879544755	-0.811169197	0.097704937	-8.302233472	1.02172E-16	4.77169E-16
RSPH1	23.23585117	-0.634707955	0.076444586	-8.302850362	1.01643E-16	4.7481E-16
SLC13A5	10.30355855	0.782492343	0.094239108	8.303265581	1.01288E-16	4.73266E-16
MTX2	221.0182978	-0.329294203	0.039652738	-8.304450552	1.00282E-16	4.68679E-16
WDR7	1263.72956	-0.353793389	0.042602347	-8.304551657	1.00197E-16	4.68392E-16
ZFAND6	3416.431463	0.324025162	0.039013691	8.305421939	9.94653E-17	4.65082E-16
FBXL8	78.1904121	-0.352972537	0.042494981	-8.306217047	9.88014E-17	4.62089E-16
EBNA1BP2	500.1519007	-0.476621363	0.057373451	-8.307350361	9.78627E-17	4.57807E-16
SNX31	7.104495206	-0.958033156	0.115322945	-8.307394128	9.78266E-17	4.57748E-16
TAT	5.167939456	-0.801413891	0.096463258	-8.30797039	9.73528E-17	4.5564E-16
MAML3	1655.932666	-0.297518469	0.03580728	-8.308882108	9.66078E-17	4.52261E-16
EML2	602.9794203	-0.321075226	0.038636824	-8.310083314	9.56349E-17	4.47814E-16
FAM178B	99.29565572	-0.892940487	0.107451645	-8.310161132	9.55722E-17	4.47627E-16
TRAPPC6B	189.8691936	-0.278427123	0.033500021	-8.311252155	9.46975E-17	4.43637E-16
SMIM10L2A	7.019895636	-0.750090059	0.090242302	-8.31195619	9.41372E-17	4.41117E-16
OR6A2	2.755095753	-1.305057662	0.157004824	-8.312213776	9.3933E-17	4.40266E-16
DGAT1	1372.821562	-0.253106085	0.030443878	-8.313858215	9.26398E-17	4.34309E-16
PRPF40A	6701.868945	0.241006373	0.028988172	8.313955493	9.25639E-17	4.34057E-16
CA6	23.88431494	0.694014593	0.083471363	8.314403488	9.22149E-17	4.32524E-16
CNKSR3	127.765885	-0.644922247	0.077557467	-8.315411384	9.14345E-17	4.28967E-16
TRMT6	438.5284828	0.407331777	0.048981407	8.31604887	9.09443E-17	4.26769E-16
NCKIPSD	431.2179744	-0.318629153	0.038312767	-8.316526879	9.05785E-17	4.25154E-16
ATP11B	5459.942898	0.369658588	0.044445473	8.317125664	9.01222E-17	4.23114E-16
PCDHB4	2.159659836	-1.466314505	0.176292956	-8.31748775	8.98474E-17	4.21925E-16
MRPL4	704.9196964	-0.363758353	0.043730224	-8.318236621	8.92816E-17	4.19369E-16
TEKT4	0.719056124	-1.873167678	0.22511346	-8.320993686	8.72289E-17	4.09825E-16

TMC1	5.087712619	-0.865079995	0.10395583	-8.321611135	8.67756E-17	4.07793E-16
ARHGEF10	26.89839591	-0.791180569	0.095071115	-8.321986846	8.65009E-17	4.066E-16
TMEM101	390.6480311	-0.292235643	0.035111158	-8.323155836	8.56517E-17	4.02705E-16
MARS2	285.0394823	-0.341588821	0.041039473	-8.323421191	8.54601E-17	4.01901E-16
NUP85	1509.032885	-0.222215565	0.026695517	-8.324077963	8.49877E-17	3.99775E-16
OR13G1	4.754232353	-1.062672425	0.127657883	-8.3243776	8.4773E-17	3.98861E-16
OR52B6	10.99615811	-0.653929505	0.078531391	-8.326982339	8.29291E-17	3.90279E-16
YARS2	368.2779354	-0.188118699	0.022586994	-8.32862936	8.17837E-17	3.84981E-16
ADTRP	24.40875658	0.729058442	0.087522611	8.329943891	8.08807E-17	3.80822E-16
NPC1	1712.089154	0.321975585	0.038651107	8.330306925	8.0633E-17	3.79747E-16
C1orf159	236.9296731	-0.26770446	0.032130225	-8.331857488	7.95837E-17	3.74896E-16
SLC15A3	1259.364941	0.669322063	0.080317308	8.333472345	7.85052E-17	3.69904E-16
SH3YL1	372.4735855	-0.309183363	0.0370983	-8.334165288	7.80468E-17	3.67833E-16
NTAQ1	190.3149871	-0.29092796	0.034906831	-8.33441332	7.78834E-17	3.67151E-16
TFDP2	5012.539087	-0.302455228	0.036289881	-8.334423366	7.78768E-17	3.67151E-16
ZNF793	115.7913133	-0.591317332	0.070948388	-8.334471651	7.7845E-17	3.67147E-16
ATF6B	2875.021836	-0.215998994	0.025915235	-8.334826806	7.76117E-17	3.66135E-16
URB2	591.8996296	-0.357013294	0.042828751	-8.335832512	7.69548E-17	3.63123E-16
WRAP53	419.8848757	-0.226367146	0.027153075	-8.33670399	7.639E-17	3.60545E-16
ENAM	6.881547799	-0.888987234	0.106604062	-8.33914974	7.48266E-17	3.53252E-16
MYH13	3.182670646	-1.141995078	0.136911091	-8.341143669	7.35754E-17	3.47429E-16
CPT1A	6874.087689	-0.247642258	0.029689215	-8.34115204	7.35702E-17	3.47429E-16
LASP1	7006.952116	0.276719344	0.033173889	8.34148048	7.33661E-17	3.46608E-16
CCDC171	422.2517736	-0.487408409	0.058417187	-8.343578853	7.20753E-17	3.40592E-16
NASP	3231.335342	-0.237917681	0.028511443	-8.344638234	7.14322E-17	3.37634E-16
CRACD	463.5989284	-0.740231999	0.088700491	-8.345297599	7.10348E-17	3.35837E-16
TIMM10	326.520745	-0.491094198	0.058845852	-8.345434436	7.09526E-17	3.35529E-16
RGN	9.959962451	-0.626717849	0.075093173	-8.345869808	7.06916E-17	3.34376E-16
FMN2	1.672113328	-1.375069005	0.164727395	-8.347542942	6.96977E-17	3.29754E-16
GRIK4	8.365246617	-0.94417791	0.113107386	-8.347623812	6.965E-17	3.29608E-16
MROH1	3101.223194	-0.338226113	0.040513006	-8.348580943	6.9088E-17	3.27028E-16
CCNC	963.6832178	0.28271554	0.033860629	8.349388259	6.86174E-17	3.24879E-16
CDK17	3162.75139	0.335752691	0.040207514	8.350496196	6.79768E-17	3.21923E-16
EHD1	4414.597648	0.425570661	0.050955849	8.351752975	6.72572E-17	3.18593E-16
MIB2	1154.624106	-0.280637943	0.033600989	-8.352073903	6.70747E-17	3.17805E-16
MINAR2	1.189953133	-1.2985443	0.155470462	-8.352353769	6.69159E-17	3.17129E-16
CROCC	503.4590736	-0.433415659	0.05189088	-8.352443866	6.68648E-17	3.16964E-16

MBIP	812.4498619	0.29818838	0.035698262	8.353022335	6.65381E-17	3.15492E-16
HECTD3	1035.894403	-0.270828824	0.03242243	-8.353131516	6.64766E-17	3.15276E-16
NAPG	1448.813081	0.237661581	0.028445604	8.354949478	6.54607E-17	3.10534E-16
MAP1LC3B	7898.444272	0.370287447	0.044316066	8.355602876	6.50994E-17	3.08895E-16
TCF3	3006.024227	-0.277942778	0.033252116	-8.358649245	6.34404E-17	3.01096E-16
DXO	530.1164689	-0.214721652	0.025686327	-8.359375346	6.30512E-17	2.99321E-16
SSUH2	8.10962247	-0.666271141	0.079691788	-8.360599683	6.24002E-17	2.96303E-16
APOBEC3D	39.99346544	-0.433884507	0.051886842	-8.362129811	6.1596E-17	2.92555E-16
ERBB3	45.9749826	-0.638639438	0.076367508	-8.362711509	6.1293E-17	2.91186E-16
ELF5	8.262171884	-0.807025224	0.096482795	-8.364446988	6.03975E-17	2.87002E-16
CNTN5	1.335594483	-2.007608913	0.239964377	-8.36627893	5.94663E-17	2.82646E-16
TAS2R19	47.81005073	-0.592967168	0.070874543	-8.366433683	5.93883E-17	2.82343E-16
LVRN	12.83825087	-0.708586997	0.084689614	-8.366870048	5.91689E-17	2.81368E-16
ARNTL	796.5211003	0.469537273	0.05611611	8.367245631	5.89807E-17	2.80542E-16
SYDE2	69.11569894	-0.930955928	0.111239128	-8.368961021	5.81285E-17	2.76555E-16
CACNA1S	2.163869163	-1.017659612	0.121587789	-8.369751759	5.77397E-17	2.74773E-16
CDC37L1	273.7818901	0.265115925	0.031670702	8.371015081	5.7124E-17	2.71909E-16
UNC13A	12.03164707	-0.581355133	0.069442231	-8.371780745	5.6754E-17	2.70213E-16
UBL7	1096.567347	-0.38883966	0.04644117	-8.372736066	5.62956E-17	2.68096E-16
KLF7	2983.73945	0.318521479	0.038041088	8.373090653	5.61264E-17	2.67355E-16
MAPK10	55.52164853	-0.656266206	0.07837623	-8.373281093	5.60357E-17	2.66988E-16
UBIAD1	609.9266737	-0.232637419	0.027782971	-8.373381613	5.59879E-17	2.66825E-16
CIBAR1	202.4030932	-0.506970717	0.060545091	-8.373440544	5.59599E-17	2.66757E-16
DHX8	3313.594735	0.20963161	0.025031613	8.374674423	5.53767E-17	2.64041E-16
LRRN1	81.99747862	-0.751102819	0.089684386	-8.374956371	5.52443E-17	2.63474E-16
ASB12	5.404971671	-0.637546241	0.07612239	-8.375278847	5.50933E-17	2.62818E-16
KLF3	4420.870857	0.387359047	0.046248801	8.375547853	5.49676E-17	2.62282E-16
RNF5	900.9670631	-0.392801701	0.046890726	-8.376959316	5.43126E-17	2.5922E-16
ARL13B	80.64907697	-0.332799007	0.039726346	-8.377287096	5.41616E-17	2.58562E-16
CHP1	4210.576103	0.259582724	0.030984929	8.377709186	5.39678E-17	2.577E-16
DTD2	60.41901965	-0.388769749	0.046405101	-8.377737416	5.39549E-17	2.577E-16
NSD2	5016.186172	-0.222204221	0.026522433	-8.377972713	5.38471E-17	2.57249E-16
SP8	3.954530581	-2.026759156	0.241896821	-8.378610124	5.35564E-17	2.55923E-16
ZFPM2	27.47574819	-0.985225016	0.117587374	-8.378663289	5.35322E-17	2.55869E-16
MYH1	1.848216196	-1.671960203	0.199500841	-8.38071755	5.2606E-17	2.51504E-16
UBL4B	4.39552327	-1.126854317	0.134455132	-8.380894828	5.25268E-17	2.51187E-16
CHKB	37.89025169	-0.346299126	0.041315597	-8.381801335	5.21238E-17	2.4932E-16

EVI2B	3289.37114	0.652643684	0.077863467	8.381898608	5.20807E-17	2.49175E-16
TRHR	7.255926117	-0.754111817	0.089962795	-8.382485415	5.18216E-17	2.47996E-16
FAM180A	1.144963918	-1.026732108	0.122477599	-8.38301955	5.15869E-17	2.46933E-16
ENPP1	7.409948175	-0.805066344	0.096027624	-8.383695332	5.12914E-17	2.45579E-16
MCL1	38740.14808	0.354562836	0.042288606	8.38435866	5.10031E-17	2.44258E-16
TMC5	47.71227039	-0.765508951	0.091301652	-8.384393171	5.09881E-17	2.44246E-16
PLCE1	130.2281108	-0.529313524	0.06312924	-8.384601517	5.08979E-17	2.43874E-16
ADH1B	1.720963138	-1.004553186	0.119795945	-8.385535783	5.04952E-17	2.42004E-16
ATP5F1D	2012.101115	-0.555706806	0.066260554	-8.386691186	5.00016E-17	2.39697E-16
SH2B3	6886.861038	0.353561095	0.042144074	8.389343117	4.88866E-17	2.34409E-16
PROCA1	332.0226522	-0.25176257	0.030001059	-8.391789428	4.78798E-17	2.29638E-16
SAMD10	189.0491399	-0.373873689	0.044543275	-8.393493473	4.71906E-17	2.26388E-16
HSD17B14	9.337756288	-0.758902405	0.090415347	-8.393513203	4.71827E-17	2.26388E-16
RNMT	2638.040499	0.298152359	0.035516805	8.394684226	4.67148E-17	2.24215E-16
SLC35G5	6.971274119	-0.649397685	0.077347954	-8.395796591	4.62746E-17	2.22157E-16
SF3B1	20991.62201	0.223708068	0.026644483	8.39603698	4.618E-17	2.21757E-16
CALCR	1.355601158	-1.248216109	0.148642361	-8.397445381	4.56297E-17	2.19168E-16
ZNF596	82.83420515	-0.265479871	0.031612235	-8.398010184	4.54108E-17	2.1817E-16
MOGAT3	3.800720769	-0.730482819	0.086972998	-8.398960982	4.50446E-17	2.16464E-16
BAK1	1199.945904	0.261194421	0.031090981	8.400970647	4.42803E-17	2.12843E-16
PPFIA2	1.888829385	-1.3269861	0.157952149	-8.401190532	4.41975E-17	2.12497E-16
SLC1A5	1822.414108	-0.479288026	0.057048766	-8.401374075	4.41284E-17	2.12218E-16
TRAF3IP1	162.0695673	-0.309117835	0.036791799	-8.401813616	4.39635E-17	2.11477E-16
HMBS	2459.167599	-0.683159929	0.081309357	-8.401984188	4.38997E-17	2.11221E-16
TLN2	374.5038084	-0.602088566	0.071649631	-8.403233321	4.3435E-17	2.09037E-16
CSNK1E	1934.060617	0.320558255	0.038132925	8.406338038	4.23011E-17	2.0363E-16
AL590560.2	3.033237845	-0.692024414	0.082319655	-8.406551403	4.22242E-17	2.0331E-16
EAPP	1349.978969	0.267117565	0.031772641	8.407156426	4.20071E-17	2.02314E-16
RYR1	154.7824744	-0.687163033	0.081734835	-8.407223603	4.1983E-17	2.02248E-16
FDX1	620.6149946	0.326034823	0.038770189	8.409420606	4.12041E-17	1.98545E-16
GNG7	539.4691719	-0.437912554	0.052069382	-8.410173831	4.09404E-17	1.97322E-16
TRPC7	1.570043475	-1.212572952	0.14417214	-8.410591344	4.07949E-17	1.96669E-16
OR1Q1	1.83435321	-1.056608353	0.125618113	-8.411273886	4.05581E-17	1.95576E-16
LRIT3	10.35824884	-0.598011114	0.071096246	-8.411289538	4.05527E-17	1.95576E-16
BRCC3	450.9703998	-0.226531498	0.026931611	-8.411360717	4.05281E-17	1.95528E-16
C19orf18	31.34462338	-0.506332273	0.060194544	-8.411597525	4.04464E-17	1.95182E-16
VOPP1	1159.461494	0.540885982	0.064296146	8.4124168	4.01648E-17	1.93871E-16

TMEM120B	796.286232	-0.259158359	0.030805528	-8.41272251	4.00602E-17	1.93413E-16
POLN	14.41038686	-0.592092807	0.070366387	-8.414426683	3.94821E-17	1.90669E-16
TESPA1	1140.665686	-0.493850993	0.05868917	-8.414686999	3.93945E-17	1.90294E-16
ARL14EPL	17.82240639	0.876407444	0.104151228	8.414758606	3.93705E-17	1.90224E-16
SLC39A14	258.4376152	-0.513968747	0.061073835	-8.415530963	3.91119E-17	1.89022E-16
POU6F2	3.842895658	-1.113997375	0.132365839	-8.416048926	3.89395E-17	1.88235E-16
FCSK	552.479757	-0.245740561	0.029193531	-8.417637556	3.84152E-17	1.85746E-16
SETSIP	9.65072941	0.531084491	0.063090037	8.417882077	3.83351E-17	1.85405E-16
SATL1	3.330377679	-0.770739402	0.091552056	-8.418591939	3.81036E-17	1.84331E-16
ZNF251	468.9186983	-0.239138915	0.028404678	-8.418997659	3.79719E-17	1.83739E-16
PNMA8C	4.435031516	-0.718664288	0.08535796	-8.419417333	3.78361E-17	1.83127E-16
MTOR	3539.505221	-0.243923553	0.028969804	-8.419924066	3.76728E-17	1.82382E-16
SMYD5	597.5941888	-0.316004565	0.037527961	-8.420509928	3.74849E-17	1.81517E-16
PPIAL4G	1.536734951	-1.089678826	0.129394994	-8.421336816	3.72212E-17	1.80285E-16
FBXO8	286.6894011	0.249259454	0.029595327	8.422257216	3.69299E-17	1.78918E-16
DMKN	4.673165842	-0.808160027	0.095949566	-8.422758553	3.67722E-17	1.78198E-16
DDX17	21857.37547	-0.269856164	0.032036152	-8.423488731	3.65436E-17	1.77134E-16
NECTIN3	23.29559793	-0.871275178	0.103431954	-8.423655779	3.64915E-17	1.76926E-16
SLC25A36	5890.199486	0.289568833	0.034370323	8.42496695	3.60852E-17	1.74999E-16
FXYD4	3.186860159	-0.980683474	0.116398432	-8.425229272	3.60045E-17	1.74651E-16
HENMT1	636.3798395	-0.440256785	0.052245273	-8.426729512	3.5546E-17	1.7247E-16
ARMC2	149.2169898	-0.305411438	0.036240621	-8.427323578	3.53661E-17	1.71639E-16
ZNF99	5.476626121	-0.839655918	0.099608958	-8.429522144	3.4708E-17	1.68487E-16
KLHL5	971.2737751	0.318338257	0.037758526	8.430897333	3.43025E-17	1.6656E-16
VARS1	2840.22385	-0.259977442	0.030836034	-8.430962312	3.42834E-17	1.66509E-16
THYN1	490.3291159	-0.36318979	0.043074085	-8.431747088	3.40542E-17	1.65436E-16
ADAD1	1.920335701	-1.359063646	0.16117215	-8.432372729	3.38726E-17	1.64595E-16
ACOT11	646.7180646	-0.574043082	0.068073991	-8.432634519	3.37969E-17	1.64268E-16
CHRNB4	6.628667408	-0.797283937	0.09454062	-8.433242099	3.36218E-17	1.63457E-16
SNRNP70	5771.545739	-0.226330937	0.026836639	-8.433654238	3.35036E-17	1.62923E-16
ADAT2	674.7103973	-0.319564999	0.037891198	-8.433752846	3.34753E-17	1.62826E-16
GSTT2B	2.355498504	-1.123346041	0.133193165	-8.433961608	3.34156E-17	1.62576E-16
VTI1A	1722.748456	0.162815227	0.01930422	8.434178061	3.33539E-17	1.62316E-16
C5AR2	387.7357977	0.72363767	0.0857843	8.435549055	3.29652E-17	1.60464E-16
TMEM256	102.9551039	-0.581108545	0.06888095	-8.436418873	3.27209E-17	1.59315E-16
GTPBP10	301.2742903	-0.277733419	0.032919883	-8.436646693	3.26572E-17	1.59044E-16
CCDC196	15.61716817	-0.782896826	0.092772624	-8.438877696	3.20399E-17	1.56077E-16

C1RL	805.8982423	-0.346915731	0.041105964	-8.439547467	3.18569E-17	1.55224E-16
GDPD3	58.22884162	-0.415897797	0.049279383	-8.439590195	3.18452E-17	1.55206E-16
GFRA2	24.54413373	-0.771410769	0.091402774	-8.439686599	3.1819E-17	1.55116E-16
FIGN	10.15336684	-2.126372492	0.251943394	-8.4398819	3.17658E-17	1.54896E-16
ERICH3	3.505634839	-1.208965378	0.143242873	-8.43996879	3.17422E-17	1.5482E-16
SLC2A2	1.610778311	-1.341283549	0.158894654	-8.441338448	3.13724E-17	1.53054E-16
NXN	99.89830373	-0.631996283	0.074864992	-8.441813247	3.12452E-17	1.52471E-16
SUPT20H	1387.283849	-0.235770184	0.027927592	-8.442195148	3.11433E-17	1.52012E-16
CABCOCO1	2.555365753	-1.056629162	0.12513956	-8.443606196	3.07695E-17	1.50225E-16
CRACR2A	1001.119957	-0.322712216	0.038217899	-8.444007194	3.0664E-17	1.49747E-16
DIAPH1	13137.83619	0.267070542	0.031625397	8.444812268	3.04534E-17	1.48756E-16
ATP5ME	255.6215329	-0.639436517	0.07569959	-8.447027443	2.98814E-17	1.45998E-16
CISD1	109.7406476	-0.420360443	0.049749443	-8.449550807	2.92426E-17	1.42913E-16
PARP3	204.8283146	-0.456931332	0.05407702	-8.449639629	2.92203E-17	1.4284E-16
ADGRG1	1102.080626	-0.726313613	0.085955317	-8.449897395	2.91559E-17	1.4256E-16
KIF7	147.6584729	-0.656876543	0.077737212	-8.44996275	2.91396E-17	1.42516E-16
SPACA4	4.242106632	-0.82068956	0.097106617	-8.451427775	2.87762E-17	1.40774E-16
REXO2	382.5338165	-0.462030613	0.054661693	-8.452548475	2.85012E-17	1.39464E-16
DLG2	66.00259485	-0.519744975	0.061489101	-8.45263574	2.84799E-17	1.39394E-16
CDIP1	858.8409314	-0.363019648	0.042938404	-8.454428059	2.80459E-17	1.37304E-16
DNAJC24	161.7535351	-0.306601062	0.036264643	-8.45454522	2.80177E-17	1.37201E-16
GPRASP2	34.18677024	-0.527927076	0.06243415	-8.455742127	2.77318E-17	1.35834E-16
RGS2	2104.379386	0.596689158	0.070556584	8.456888459	2.74606E-17	1.3454E-16
FOCAD	1385.496942	-0.407329088	0.048163846	-8.457154504	2.73981E-17	1.34267E-16
UBE2A	2509.156092	0.248709515	0.029405481	8.457930414	2.72164E-17	1.3341E-16
MAST2	1046.167482	-0.305626763	0.036131194	-8.45880614	2.70129E-17	1.32446E-16
CPLX2	7.924824743	-0.821136409	0.09706861	-8.459340324	2.68894E-17	1.31873E-16
FAM13C	1.830410259	-1.414137758	0.167167474	-8.459407352	2.6874E-17	1.31831E-16
FZD8	78.17720503	-0.862514149	0.101957661	-8.459532501	2.68452E-17	1.31722E-16
CFDP1	444.1072272	-0.236795129	0.027989026	-8.46028467	2.66726E-17	1.30908E-16
LCNL1	10.12359291	1.030277203	0.121771254	8.460758723	2.65644E-17	1.3041E-16
C20orf203	63.74375484	-0.681780173	0.080566026	-8.462378048	2.6198E-17	1.28643E-16
CBY3	8.528963505	-0.505862866	0.059761064	-8.464756723	2.56688E-17	1.26077E-16
GALK1	368.8899071	-0.416557313	0.049192871	-8.46783895	2.49988E-17	1.22816E-16
C17orf98	5.179278599	-0.833200174	0.09838969	-8.468368721	2.48853E-17	1.2229E-16
ATP8A2	25.85638617	-0.862157664	0.101795935	-8.469470452	2.46511E-17	1.21169E-16
NEK11	97.81704808	-0.480863574	0.056773956	-8.469791504	2.45833E-17	1.20866E-16

FAM107A	4.432729788	-0.786513428	0.092836426	-8.472034779	2.41143E-17	1.1859E-16
C1orf105	10.12924504	-0.713951343	0.08426988	-8.472200802	2.408E-17	1.18451E-16
SETDB2	995.1518239	-0.323342597	0.038164419	-8.472357369	2.40476E-17	1.18322E-16
AQP10	13.48512177	-0.892251614	0.105306256	-8.472921253	2.39314E-17	1.1778E-16
FANCD2	1225.726411	-0.33055663	0.039013115	-8.472961613	2.39231E-17	1.17769E-16
BMPER	10.52054473	-0.857764936	0.101232063	-8.473253552	2.38632E-17	1.17503E-16
OR2G2	0.963805351	-1.384951289	0.163434539	-8.474042841	2.3702E-17	1.16739E-16
UBQLN3	7.531130428	-0.799452259	0.09434039	-8.474124998	2.36853E-17	1.16686E-16
ABHD17C	575.5151097	0.737056892	0.08697412	8.474439226	2.36215E-17	1.16401E-16
EPC2	1763.326044	0.266391449	0.031433651	8.474721863	2.35642E-17	1.16148E-16
TCAF2C	1.974043706	0.87581147	0.103320549	8.476643561	2.31784E-17	1.14275E-16
CALML4	9.350175185	-0.503874797	0.059441022	-8.476886437	2.31301E-17	1.14065E-16
CCSAP	1134.390151	0.302150161	0.035643886	8.47691401	2.31246E-17	1.14065E-16
TDRD12	10.43184781	-0.660660716	0.077935457	-8.477023731	2.31028E-17	1.13988E-16
SHOC1	57.65851175	-0.70356406	0.082993015	-8.477388847	2.30304E-17	1.1366E-16
CUL3	3747.279129	0.291635647	0.034399852	8.477816949	2.29459E-17	1.13271E-16
ERCC1	1181.393301	-0.312798951	0.036893972	-8.478321417	2.28466E-17	1.12809E-16
ARF6	4518.849332	0.298466353	0.035197138	8.479847289	2.2549E-17	1.11368E-16
C2orf92	223.4139737	-0.388563113	0.045819294	-8.48033837	2.2454E-17	1.10927E-16
R3HCC1L	1053.453712	0.261775719	0.030865178	8.481263917	2.22761E-17	1.10076E-16
PSD4	3303.956415	-0.324401641	0.038246733	-8.481813216	2.21711E-17	1.09585E-16
OR51B5	5.561836847	-0.829031644	0.097740622	-8.481955878	2.2144E-17	1.09478E-16
ZSWIM7	388.5220075	-0.326908167	0.038541012	-8.482085679	2.21193E-17	1.09384E-16
MRPL48	423.3338871	-0.309610799	0.036491172	-8.484539662	2.16574E-17	1.07127E-16
TOP2A	1738.608144	-0.511405064	0.060269589	-8.485292045	2.15178E-17	1.06463E-16
MINAR1	39.88303991	-0.541995399	0.063865702	-8.486486208	2.12979E-17	1.05402E-16
RXFP3	3.557264372	-1.194308416	0.140708389	-8.487826652	2.10537E-17	1.0422E-16
PLEKHG4B	21.31862105	-1.094950683	0.128995098	-8.488312348	2.09659E-17	1.03811E-16
MAPT	57.03701662	-0.66192261	0.077958721	-8.490680749	2.0543E-17	1.01743E-16
UBE4B	2835.683433	-0.185560397	0.021853777	-8.490998798	2.04869E-17	1.01491E-16
CGRRF1	255.5138325	0.329495592	0.038804148	8.491246583	2.04432E-17	1.013E-16
ARMC3	4.030656459	-0.954192836	0.112365521	-8.491865005	2.03347E-17	1.00788E-16
ZNF226	272.9085399	-0.312534759	0.036803255	-8.492041166	2.03039E-17	1.00661E-16
NSUN5	747.6226396	-0.292000241	0.03438409	-8.492306784	2.02575E-17	1.00456E-16
ODF4	2.045409632	-0.934679488	0.110060544	-8.492412021	2.02392E-17	1.00391E-16
RGS17	22.88797178	-0.559302385	0.065853549	-8.493124411	2.01155E-17	9.98023E-17
GUCY1B1	293.5305761	-0.685813096	0.08074853	-8.493196073	2.01031E-17	9.97661E-17

CDC25A	357.6548994	-0.422707861	0.049770153	-8.493199966	2.01024E-17	9.97661E-17
AAMP	1439.887822	-0.200400747	0.023591923	-8.494464198	1.98848E-17	9.8733E-17
SPPL3	1962.360278	0.208096308	0.024493248	8.496068195	1.96121E-17	9.74035E-17
TEK	31.89055013	-0.899383757	0.10584644	-8.497061961	1.9445E-17	9.6598E-17
AGMAT	83.11574069	-0.424370302	0.049940836	-8.49746098	1.93783E-17	9.62911E-17
SMCHD1	11560.4434	0.367380422	0.043215456	8.501134929	1.87746E-17	9.33152E-17
C9orf40	119.1455609	-0.445818663	0.052437492	-8.501906681	1.86502E-17	9.27203E-17
TMEM250	515.2049376	-0.411608437	0.048412074	-8.502185512	1.86054E-17	9.25213E-17
SYNJ2BP	340.6414649	-0.384368596	0.045206825	-8.502446261	1.85637E-17	9.23371E-17
GRM6	6.239058178	-0.677089295	0.079625014	-8.503474703	1.83999E-17	9.15456E-17
APLNR	9.193502714	-1.034320847	0.121621318	-8.504437056	1.82479E-17	9.08126E-17
C2orf80	1.337745019	-0.907733557	0.106734871	-8.504564144	1.82279E-17	9.07362E-17
SEMA5B	12.43195638	-0.877402041	0.103167009	-8.504676562	1.82103E-17	9.06714E-17
TMEM106C	527.2554844	-0.454260161	0.05340972	-8.505196473	1.81288E-17	9.0289E-17
TSHB	1.952895756	-0.981658603	0.115400709	-8.506521414	1.7923E-17	8.92863E-17
PPAT	410.7013412	-0.291928409	0.034317266	-8.50675015	1.78876E-17	8.91331E-17
FP565260.3	120.6534036	0.708578326	0.083288303	8.507537037	1.77667E-17	8.8553E-17
MT-CO1	745039.9348	-0.763498477	0.089739201	-8.507970536	1.77004E-17	8.82451E-17
DPPA4	146.8811295	-1.100238438	0.129273893	-8.510909773	1.72574E-17	8.60583E-17
CHAF1B	441.1713193	-0.268592868	0.031558291	-8.511007993	1.72428E-17	8.60073E-17
DRC7	47.10054023	-0.553379506	0.065012183	-8.511935483	1.71054E-17	8.53437E-17
TRAT1	205.7579975	0.668490259	0.078532866	8.512235607	1.70611E-17	8.51448E-17
FGF12	3.472781621	-1.522712266	0.178883006	-8.512336072	1.70464E-17	8.50927E-17
PRKCQ	648.5061643	-0.462042492	0.054276759	-8.512713414	1.6991E-17	8.48378E-17
OR2T33	6.920716732	-0.719267633	0.084485337	-8.513520325	1.68731E-17	8.42707E-17
MRPS9	170.793451	-0.260447333	0.030592079	-8.513554491	1.68681E-17	8.42674E-17
TMEM14A	77.19565574	-0.625143945	0.073424948	-8.514053646	1.67956E-17	8.39267E-17
KCNA7	5.078977776	-0.679795008	0.079840524	-8.514410643	1.6744E-17	8.36899E-17
SHB	228.0628819	0.54007806	0.06342683	8.514978003	1.66622E-17	8.33025E-17
CACNG5	7.770172596	-0.714719621	0.083931347	-8.515526635	1.65835E-17	8.29302E-17
PDK1	3380.754666	0.379671955	0.044584093	8.515861264	1.65357E-17	8.27122E-17
DCLRE1A	241.7629656	-0.360458084	0.042325989	-8.516235448	1.64824E-17	8.24666E-17
SAXO2	19.71829795	-0.718867471	0.084409862	-8.516391983	1.64601E-17	8.23763E-17
P2RY4	7.677253954	-0.508478655	0.059700667	-8.517135312	1.63548E-17	8.18704E-17
SYNPO2L	26.65722457	-0.58108923	0.068212556	-8.518801627	1.61213E-17	8.07218E-17
ADAMTS10	662.9125961	-0.711784178	0.083549312	-8.519330243	1.60479E-17	8.03748E-17
FGGY	265.0576594	-0.330836589	0.038833303	-8.519403819	1.60377E-17	8.03444E-17

MAGEA8	2.840731197	-0.74169021	0.087051437	-8.520137456	1.59364E-17	7.98575E-17
AMMECR1	618.9567002	-0.503394789	0.059074393	-8.521370548	1.57676E-17	7.90319E-17
C17orf80	275.5639582	-0.302525574	0.035496975	-8.522573318	1.56047E-17	7.82352E-17
XPNPEP3	819.128228	-0.193261411	0.022675382	-8.522961614	1.55524E-17	7.79933E-17
ADAM30	3.10741895	-1.126365284	0.132149831	-8.52339556	1.54942E-17	7.77214E-17
NR1H3	319.0138893	-0.439992094	0.051614479	-8.524586549	1.53357E-17	7.69456E-17
ARFIP1	286.2209819	-0.411707235	0.048292312	-8.525316367	1.52393E-17	7.64816E-17
XKR5	26.28887182	-0.740128824	0.086802719	-8.526562676	1.50761E-17	7.56819E-17
PCDHGA4	16.67185678	-0.945514966	0.110878793	-8.527464486	1.4959E-17	7.51136E-17
SLC16A13	50.85246495	-0.464595392	0.05447752	-8.528203717	1.48638E-17	7.46545E-17
SYP	39.12486382	-0.466968986	0.05474745	-8.529511236	1.46967E-17	7.38345E-17
AC008581.2	1.473373483	-0.874944292	0.102574388	-8.529851446	1.46536E-17	7.36366E-17
FOXJ3	4085.357375	0.245265416	0.028745022	8.532448283	1.43283E-17	7.20203E-17
CPZ	1.392090228	-1.972751308	0.23118178	-8.533333831	1.4219E-17	7.14893E-17
SULT2A1	2.650346629	-0.837257236	0.098105406	-8.534261951	1.41053E-17	7.0936E-17
CDC7	353.2356706	-0.340203513	0.039860372	-8.534880449	1.403E-17	7.05757E-17
VWF	875.2437004	-0.923400133	0.108187276	-8.535200902	1.39912E-17	7.03984E-17
ELMO2	2567.150269	0.294275861	0.034477167	8.535384142	1.39691E-17	7.0305E-17
RAB27B	471.2018028	-0.752293883	0.088127683	-8.536408243	1.38459E-17	6.97029E-17
SLC17A7	7.954134816	-0.705304595	0.082619276	-8.536804363	1.37985E-17	6.94823E-17
SLC17A2	1.408863504	-1.343282064	0.157350999	-8.53685119	1.37929E-17	6.94721E-17
PLA2G4F	7.467542342	-0.925148603	0.108367307	-8.537156001	1.37566E-17	6.93069E-17
ERCC4	149.1009279	-0.319108112	0.037373549	-8.538341133	1.36162E-17	6.86176E-17
CHPT1	1315.483433	0.358291279	0.041958489	8.539184593	1.35172E-17	6.81362E-17
NOS2	3.330967055	-0.853788632	0.099954998	-8.541730298	1.32227E-17	6.66686E-17
MAN1B1	1567.401568	-0.222830342	0.026084409	-8.542664015	1.31162E-17	6.61489E-17
LCP1	27933.04592	0.359368779	0.042065763	8.543023021	1.30755E-17	6.59607E-17
VANGL2	130.8972243	-0.882164383	0.103256366	-8.543438219	1.30286E-17	6.5741E-17
PTGS1	1554.596134	-0.530644898	0.062106097	-8.544167567	1.29466E-17	6.5344E-17
IRS2	7210.410889	0.485405534	0.05680765	8.544721238	1.28847E-17	6.50482E-17
OR2AG2	4.073253988	-0.994688492	0.116408391	-8.544817784	1.28739E-17	6.50107E-17
FAM102B	1520.773758	0.346134492	0.040504152	8.545654624	1.2781E-17	6.45579E-17
ABRAXAS2	645.3275871	0.256776479	0.03004529	8.546313789	1.27082E-17	6.4207E-17
C1GALT1	1466.123088	0.301845367	0.035318729	8.546325858	1.27069E-17	6.4207E-17
ICAM2	368.3758691	-0.371063405	0.04341697	-8.546506326	1.2687E-17	6.41332E-17
DAPL1	17.26233676	-1.07502603	0.125769988	-8.547556139	1.25722E-17	6.35691E-17
BRSK2	45.24551649	-0.602708649	0.070510449	-8.547791904	1.25466E-17	6.34558E-17

CALCRL	131.8835712	-0.863685711	0.101039634	-8.547989323	1.25251E-17	6.33638E-17
CIBAR2	3.041865761	-0.987879426	0.115552022	-8.549218018	1.23925E-17	6.27091E-17
OTUD4	5075.580348	0.257553531	0.030124508	8.549634558	1.23479E-17	6.24994E-17
ZNF655	3197.903226	0.245488849	0.02870749	8.551386675	1.21618E-17	6.15737E-17
MORC1	9.397919259	-1.006126759	0.11764478	-8.552243098	1.20719E-17	6.11342E-17
METTL26	883.5764884	-0.551620563	0.064493364	-8.553136738	1.19788E-17	6.06783E-17
FAM53C	5056.97933	0.336018422	0.039285295	8.5532874	1.19632E-17	6.06148E-17
CARMIL1	195.378892	-0.703988149	0.082305857	-8.553317718	1.196E-17	6.06145E-17
SCRN3	195.3969461	-0.285557889	0.0333832	-8.553939982	1.18957E-17	6.03041E-17
EBF2	2.50663785	-1.018661028	0.119075141	-8.554774917	1.18099E-17	5.98847E-17
RSPH3	235.3521331	-0.316182105	0.03695729	-8.555337956	1.17524E-17	5.96086E-17
GABRR1	11.06645348	-0.851914339	0.099575281	-8.555480144	1.17379E-17	5.95506E-17
ZNF658	36.78036865	-0.573593199	0.067043537	-8.555533142	1.17325E-17	5.95387E-17
ALDH6A1	552.2166087	-0.359783049	0.042052627	-8.555542867	1.17315E-17	5.95387E-17
FAH	1111.509709	-0.326976725	0.038215693	-8.556085206	1.16765E-17	5.92851E-17
CDRT15	7.551449204	-0.635942501	0.074317327	-8.557122882	1.15719E-17	5.87694E-17
MEI1	442.1324075	0.378849038	0.044272598	8.55719004	1.15652E-17	5.87504E-17
MICB	571.2978524	0.288573229	0.033720043	8.557913919	1.14928E-17	5.83979E-17
SCX	41.49751649	-0.493676503	0.057685835	-8.558019487	1.14823E-17	5.83596E-17
NAA50	2525.818529	0.254630495	0.029750223	8.558943993	1.13906E-17	5.79086E-17
HPS1	3318.849621	-0.292497801	0.034169076	-8.560307523	1.12567E-17	5.72427E-17
OR7D4	4.084651009	-0.704272016	0.082270101	-8.560485642	1.12393E-17	5.71692E-17
LZIC	802.3986511	-0.257861461	0.030110392	-8.563869369	1.09141E-17	5.55297E-17
RBM41	325.5575022	-0.297944237	0.034789579	-8.564180617	1.08847E-17	5.53943E-17
KAT6A	10211.62804	0.332351076	0.038798118	8.566164937	1.06989E-17	5.44626E-17
TRARG1	5.713771481	-0.882659678	0.103025525	-8.567388271	1.05858E-17	5.39014E-17
TRIM67	7.775840453	-0.666370722	0.077770286	-8.568448918	1.04888E-17	5.34212E-17
YBEY	167.4972513	-0.342875806	0.040010019	-8.569748591	1.03711E-17	5.28355E-17
TRIM28	10389.45376	-0.270645329	0.031581415	-8.569765642	1.03696E-17	5.28355E-17
CLNS1A	2054.902402	-0.266843301	0.031136612	-8.570081491	1.03412E-17	5.27104E-17
GLRB	9.485806534	-1.342985115	0.156689262	-8.571009248	1.02582E-17	5.23011E-17
IMPG1	3.311383439	-1.77515396	0.207066563	-8.572866309	1.00941E-17	5.14776E-17
PPP1R3E	498.5513833	-0.276656007	0.032268515	-8.57355864	1.00335E-17	5.11823E-17
LRRIQ1	1.136455547	-1.250802502	0.145848303	-8.57605109	9.81859E-18	5.00989E-17
FTCD	19.02353067	-0.696598352	0.081206565	-8.578103942	9.64498E-18	4.92259E-17
CD248	72.04531202	0.709871894	0.08275274	8.57822829	9.63456E-18	4.91855E-17
CCNG2	1704.6664	0.334616596	0.039006421	8.578500322	9.6118E-18	4.90821E-17

TPGS2	2044.555368	-0.293746746	0.034241051	-8.578788809	9.58773E-18	4.8972E-17
DNAJC22	5.853174001	-0.617983431	0.072025826	-8.580025567	9.48521E-18	4.8461E-17
LRRTM3	7.039889627	-1.659922816	0.19344085	-8.581035564	9.40228E-18	4.80498E-17
MPPED1	7.66968428	-0.901871955	0.105095945	-8.58141537	9.37128E-18	4.79039E-17
PDE3B	3557.742167	0.392448563	0.045726254	8.582565279	9.27804E-18	4.74397E-17
CD48	484.7575197	0.60858606	0.070902092	8.583471085	9.20525E-18	4.70798E-17
EPB41L4A	46.69678839	-0.706955024	0.082352975	-8.584450384	9.12717E-18	4.66927E-17
ARHGEF25	2.942156884	-0.994528896	0.115840996	-8.585293005	9.06052E-18	4.63638E-17
CRTC1	688.8032999	-0.314068573	0.036580085	-8.585780236	9.0222E-18	4.61798E-17
ENPP7	8.526576058	-0.784941037	0.091422405	-8.585871683	9.01502E-18	4.61551E-17
MMEL1	21.49292621	-0.673994607	0.078496381	-8.586314394	8.98037E-18	4.59897E-17
SPAAR	2.767560151	-0.770968292	0.089789161	-8.586429395	8.97139E-18	4.59558E-17
SRD5A3	297.3263916	-0.412391035	0.048007991	-8.590049897	8.69316E-18	4.45422E-17
MFRP	4.985999006	-0.855728701	0.099602775	-8.591414211	8.59053E-18	4.40279E-17
OR13C4	2.02787704	-1.154376715	0.134363952	-8.591416809	8.59034E-18	4.40279E-17
FEM1A	225.9533776	-0.38071482	0.044287336	-8.596471501	8.22045E-18	4.21532E-17
CNBD1	1.247539319	-2.179473463	0.253531103	-8.596473726	8.22029E-18	4.21532E-17
C10orf90	1.233014758	-1.463387364	0.170208505	-8.597616012	8.13891E-18	4.1757E-17
WDR48	4011.017346	0.331100086	0.038509829	8.597807239	8.12536E-18	4.16984E-17
IFI27L1	194.0522657	-0.381998438	0.0444241	-8.598901096	8.0483E-18	4.13138E-17
VSIG1	13.50149001	0.649150655	0.075490574	8.599095479	8.03468E-18	4.12547E-17
KEL	989.6288006	-0.923738869	0.107415863	-8.599650399	7.99593E-18	4.10665E-17
PCDHA10	3.592665957	-0.910348722	0.105847117	-8.600600033	7.93004E-18	4.07388E-17
AC092718.3	13.88194185	-0.418051232	0.04860555	-8.600895062	7.90968E-18	4.06448E-17
ZBTB40	1958.715584	-0.27574535	0.032059934	-8.600933079	7.90706E-18	4.0642E-17
C5orf34	72.88447509	-0.381850927	0.044387729	-8.602623605	7.79142E-18	4.00582E-17
CYP2J2	5.877018212	-1.053606936	0.122464573	-8.603361069	7.74149E-18	3.98119E-17
SPINK5	1.713505972	-1.477027326	0.171662011	-8.604276022	7.68E-18	3.95061E-17
NPIPB6	17.22939584	-0.535258219	0.06220741	-8.604412571	7.67086E-18	3.94694E-17
RNF11	3879.563817	0.380941669	0.044266407	8.605660375	7.58786E-18	3.90527E-17
CLINT1	7649.002612	0.26670274	0.030988511	8.606503905	7.53226E-18	3.87767E-17
PAXIP1	523.0802344	-0.238036134	0.027655826	-8.607088373	7.49397E-18	3.85897E-17
ST6GALNAC1	84.56878828	-0.93953247	0.109154877	-8.607333861	7.47794E-18	3.85173E-17
PCDHGA8	45.87671848	-0.821227014	0.095393166	-8.608866348	7.37866E-18	3.80159E-17
SSR3	2797.136843	0.241602114	0.028061954	8.609597087	7.33178E-18	3.77843E-17
FCGR2A	1352.912573	0.582586145	0.067645113	8.612390706	7.15525E-18	3.68843E-17
FAM214A	1931.075572	0.34610256	0.040184821	8.612768405	7.13171E-18	3.67726E-17

SLC19A1	819.2438077	-0.371885193	0.043149231	-8.618582137	6.77882E-18	3.49623E-17
ZNHIT2	97.46635802	-0.341371992	0.03960128	-8.620226193	6.6822E-18	3.4473E-17
FGF18	16.23507546	-0.752422102	0.087281081	-8.620678116	6.65588E-18	3.43463E-17
SYTL1	2193.9127	-0.432761852	0.050193303	-8.621904232	6.58498E-18	3.39894E-17
HDHD3	273.684199	-0.351821008	0.040796755	-8.6237498	6.47966E-18	3.34546E-17
SLC30A10	16.52695785	-0.937156431	0.108652372	-8.625273543	6.39396E-18	3.30209E-17
CACNG4	24.68630462	-1.162754125	0.134802188	-8.625632454	6.37394E-18	3.29261E-17
RAB3GAP1	1730.298336	0.218886337	0.025374672	8.626173873	6.34386E-18	3.27794E-17
PTH2	2.852772856	-1.772225379	0.20544603	-8.626233272	6.34056E-18	3.2771E-17
MYO9A	1553.877111	-0.35249136	0.040860989	-8.626598872	6.32034E-18	3.26751E-17
C8orf34	1.829743693	-1.372309924	0.159075891	-8.626762479	6.31131E-18	3.2637E-17
ZNF217	3210.135201	0.281097673	0.032583814	8.626911255	6.3031E-18	3.26032E-17
PLEKHA1	277.6538333	0.577025409	0.066876372	8.628240283	6.23031E-18	3.22352E-17
HAPLN1	1.461045446	-1.928371065	0.223478886	-8.628873626	6.19591E-18	3.20657E-17
PLEKHA4	46.36655	-0.599090301	0.069420092	-8.629926684	6.13913E-18	3.17803E-17
ACD	489.5292186	-0.32979476	0.038215033	-8.629974423	6.13657E-18	3.17754E-17
BCL11B	787.6120884	0.714527475	0.082790996	8.630497354	6.10858E-18	3.16388E-17
ISYNA1	1473.803012	-0.423693352	0.049091953	-8.630606951	6.10273E-18	3.16169E-17
SERPINC1	6.099092561	-0.655247697	0.075911314	-8.631752767	6.04189E-18	3.131E-17
SLC17A8	2.68659378	-1.302673717	0.150888694	-8.633342104	5.95849E-18	3.0886E-17
AKIP1	196.6912881	-0.339109609	0.039271568	-8.634990244	5.8732E-18	3.0452E-17
TEP1	2994.690793	-0.392780682	0.045469362	-8.638359296	5.7026E-18	2.95753E-17
OR3A3	2.520871265	-0.958152852	0.11090538	-8.639372195	5.65228E-18	2.9322E-17
COL6A3	167.3326948	-0.755230702	0.087409527	-8.640141761	5.61433E-18	2.91329E-17
MUC16	14.5344104	-0.765195754	0.088561878	-8.640238533	5.60958E-18	2.9116E-17
BTBD2	1969.857247	-0.300992827	0.034820305	-8.644175441	5.41952E-18	2.8137E-17
UQCRQ	783.849804	-0.489486687	0.05661257	-8.646254534	5.32173E-18	2.76366E-17
CD55	4806.814453	0.343727534	0.039751785	8.646845174	5.29427E-18	2.75013E-17
TUSC3	13.36366151	-1.41894474	0.164095205	-8.647082296	5.28329E-18	2.74515E-17
NEUROD2	32.40380737	-0.714037209	0.08257275	-8.647371105	5.26994E-18	2.73894E-17
ADAMDEC1	35.25391157	0.764842193	0.088440131	8.648134992	5.23479E-18	2.72139E-17
RABL3	555.3445094	0.230897614	0.026697962	8.648510988	5.21757E-18	2.71317E-17
PYGO2	714.9141556	-0.275217941	0.031815827	-8.650346911	5.13431E-18	2.67058E-17
GGNBP2	5604.694046	0.295918472	0.03420446	8.651458784	5.08453E-18	2.64539E-17
ACER2	57.27481043	-0.418090995	0.048323493	-8.651919954	5.06402E-18	2.63542E-17
DLST	4917.357738	0.266722122	0.030821961	8.653639051	4.98828E-18	2.59669E-17
JARID2	9748.232731	0.310986398	0.035935898	8.653920386	4.97599E-18	2.59098E-17

ZNF75D	400.3838277	-0.302086428	0.034903983	-8.654783738	4.93847E-18	2.57213E-17
FAXDC2	1057.78494	-0.643169763	0.074294137	-8.657072992	4.84033E-18	2.52169E-17
HCN1	1.516689775	-1.819551256	0.210166148	-8.65767999	4.81463E-18	2.50897E-17
LRRD1	9.348040446	-0.695766769	0.080360821	-8.658034553	4.79968E-18	2.50184E-17
ZNF653	101.4006774	-0.344948551	0.039839842	-8.658381547	4.7851E-18	2.4949E-17
NRIP3	927.8175759	0.546829672	0.063145896	8.659781649	4.72669E-18	2.46511E-17
CSKMT	317.7669555	-0.623382973	0.071983236	-8.660113264	4.71296E-18	2.4586E-17
STK4	7214.142948	0.267184239	0.030851355	8.660372835	4.70224E-18	2.45366E-17
CROT	124.0772699	-0.408209266	0.047133147	-8.660768373	4.68595E-18	2.44582E-17
DGAT2L6	2.00175978	-0.713987704	0.08243677	-8.66103442	4.67502E-18	2.44076E-17
SPINK2	1292.254547	-1.169681628	0.135029482	-8.662416596	4.61867E-18	2.41199E-17
MPP4	13.97585323	-0.700108451	0.080819201	-8.662650009	4.60922E-18	2.40769E-17
KLK14	4.884280842	-0.623038931	0.071916308	-8.663388771	4.57943E-18	2.39277E-17
BAZ1A	3794.019158	-0.364107247	0.042024829	-8.664098317	4.55101E-18	2.37855E-17
CENPO	425.7047214	-0.266511174	0.030755229	-8.665556416	4.49313E-18	2.34893E-17
ZNF181	147.3250412	-0.287237252	0.033139856	-8.667425976	4.41999E-18	2.31131E-17
ZNF48	180.9309081	-0.372689566	0.042992606	-8.668689795	4.37121E-18	2.28642E-17
DNMBP	1023.365716	0.408426893	0.047114485	8.668817976	4.36629E-18	2.28445E-17
PPME1	926.2003687	-0.364939277	0.042096844	-8.669041274	4.35774E-18	2.28059E-17
SIGLEC6	175.3576276	-0.813195873	0.093793296	-8.670085293	4.31797E-18	2.26038E-17
GNAI3	2631.882387	0.252085393	0.029072381	8.670957952	4.285E-18	2.24372E-17
KANK4	7.449640249	-0.95419709	0.110038848	-8.671456567	4.26627E-18	2.23451E-17
GTF2H2C	124.6378402	-0.312636458	0.036043922	-8.673763618	4.18068E-18	2.19027E-17
ATP2A2	10528.02791	0.298926636	0.034461566	8.674203391	4.16455E-18	2.1824E-17
XBP1	8451.980785	0.338597334	0.039034922	8.674215817	4.1641E-18	2.1824E-17
SLCO1A2	1.686613569	-1.214702736	0.140014081	-8.675575523	4.11464E-18	2.1574E-17
CCNO	1.449747098	-1.112058351	0.128171186	-8.676352212	4.08665E-18	2.1433E-17
DNAAF2	473.7906845	-0.350474942	0.040391685	-8.676908189	4.06673E-18	2.13343E-17
SLC48A1	706.6157953	-0.342592716	0.039481095	-8.677386478	4.04967E-18	2.12505E-17
AFF3	1866.182508	-0.570844188	0.065779979	-8.678084113	4.02491E-18	2.11262E-17
SPTLC3	88.81602309	-0.798153549	0.091939601	-8.681281372	3.91335E-18	2.05461E-17
TTC30B	109.8868181	-0.37668419	0.043388066	-8.681746472	3.89737E-18	2.04677E-17
SLC14A1	476.8070265	-0.782619839	0.090130713	-8.683164854	3.84906E-18	2.02194E-17
ADAMTSL3	3.9588736	-0.927984309	0.106868127	-8.683452516	3.83933E-18	2.01738E-17
MRPL34	476.0521399	-0.430518521	0.049567437	-8.685510972	3.77044E-18	1.98171E-17
ZNF112	14.78638162	-1.022516955	0.117719752	-8.686027116	3.75335E-18	1.97326E-17
H4C3	2555.563452	-0.834158909	0.096025644	-8.686834833	3.72677E-18	1.95981E-17

STPG3	20.17463503	-0.791564627	0.091120154	-8.687042275	3.71998E-18	1.95676E-17
POLA1	1393.619083	-0.324980912	0.037407764	-8.687525829	3.70418E-18	1.94898E-17
RB1CC1	5859.023273	0.423429588	0.048739608	8.687587087	3.70219E-18	1.94845E-17
EXOC4	2615.598929	-0.315643481	0.036328224	-8.688657169	3.66749E-18	1.93071E-17
LSM4	1000.540565	-0.430654018	0.049557472	-8.689991648	3.62466E-18	1.90868E-17
HTR4	2.654608128	-0.92587154	0.10651812	-8.692150564	3.55642E-18	1.87325E-17
UCP1	2.102120058	-0.908859566	0.104547376	-8.693279548	3.52125E-18	1.85522E-17
IQGAP3	1207.55203	-0.461254286	0.053052538	-8.694292514	3.48998E-18	1.83924E-17
SLBP	1975.365081	0.289838777	0.033336569	8.694319348	3.48915E-18	1.83924E-17
SIK2	1046.461372	-0.252866171	0.02908236	-8.694829878	3.4735E-18	1.83154E-17
PES1	1225.442967	-0.251394597	0.028911054	-8.695449164	3.4546E-18	1.82207E-17
ANGPT2	196.3718451	-0.57370745	0.065973555	-8.696021462	3.43723E-18	1.8134E-17
ZNF638	3313.023622	-0.341308026	0.039247789	-8.696235832	3.43075E-18	1.81046E-17
STEAP2	2.801923798	-0.993031039	0.114190527	-8.696264642	3.42988E-18	1.81046E-17
CCZ1	399.3844675	0.237230148	0.027276495	8.697237326	3.40062E-18	1.79553E-17
ARHGAP39	26.71765077	-0.633648469	0.072847875	-8.698242342	3.37064E-18	1.78018E-17
LFNG	1231.979656	0.652682217	0.075010695	8.701188792	3.28425E-18	1.73503E-17
DEPTOR	534.7014932	-0.684157452	0.078620789	-8.701991682	3.26109E-18	1.72326E-17
ATPAF1	619.1897319	-0.288434343	0.033144916	-8.702219763	3.25455E-18	1.72026E-17
OR13F1	1.676869112	-1.159970247	0.133295703	-8.702232854	3.25417E-18	1.72026E-17
TBR1	8.238735155	-0.906438128	0.104160034	-8.702360152	3.25052E-18	1.71906E-17
PIGQ	1183.412644	-0.404184437	0.04643237	-8.704798716	3.18139E-18	1.68295E-17
DPP3	940.5864962	-0.32727917	0.03759498	-8.705395521	3.16469E-18	1.67457E-17
EPOR	526.5586796	-0.642702492	0.073824742	-8.705787167	3.15378E-18	1.66925E-17
ANAPC7	1206.162082	-0.230224912	0.026440456	-8.707297205	3.11206E-18	1.64762E-17
ALG2	760.6715602	0.307235885	0.035275807	8.709535265	3.05123E-18	1.61585E-17
ARGFX	5.669557759	-0.798731763	0.09170381	-8.709908207	3.04121E-18	1.61098E-17
GALR1	2.277223442	-0.858267704	0.098534224	-8.710351324	3.02934E-18	1.60513E-17
LYSMD3	723.735745	0.362863823	0.041653014	8.711586294	2.99652E-18	1.58816E-17
UBXN7	5686.401824	0.340598671	0.039082541	8.714854868	2.91131E-18	1.54342E-17
RHOT2	2084.63444	-0.247461025	0.028395208	-8.714886822	2.91049E-18	1.5434E-17
TENM3	8.167369589	-1.006893938	0.115531972	-8.715283904	2.90031E-18	1.53842E-17
GNB1L	256.5825774	-0.467281154	0.053611434	-8.716072726	2.88018E-18	1.52816E-17
ANKRD24	40.68659291	-0.510023088	0.058500387	-8.718285703	2.82446E-18	1.499E-17
IKBKE	725.4422695	-0.405629558	0.046522396	-8.719016838	2.80628E-18	1.48976E-17
ERI2	288.5757068	-0.374373503	0.042935128	-8.719515323	2.79395E-18	1.48361E-17
ZFP14	308.6628827	-0.368354491	0.042242865	-8.719922122	2.78393E-18	1.47869E-17

ALG3	755.4012042	-0.504447448	0.057849777	-8.719954993	2.78312E-18	1.47867E-17
ACCS	1387.707521	-0.501708434	0.057534545	-8.720125145	2.77894E-18	1.47685E-17
ERICH5	1.089912205	-1.362229451	0.156180918	-8.722124764	2.73029E-18	1.45139E-17
MYOG	4.76330628	-1.002935198	0.114986615	-8.722190803	2.7287E-18	1.45093E-17
DR1	3334.849291	0.223303781	0.025595327	8.724396488	2.67604E-18	1.42332E-17
RAB2A	2340.271829	0.287647851	0.032969178	8.724750404	2.66768E-18	1.41926E-17
COX10	446.6919152	-0.261683743	0.029992795	-8.724886942	2.66447E-18	1.41793E-17
C8orf44-SGK3	1.669228501	-0.795462276	0.091162583	-8.72575404	2.64413E-18	1.40749E-17
CNTNAP5	3.898315119	-2.239905364	0.256698009	-8.725838468	2.64216E-18	1.40683E-17
IGFL4	3.879608286	-0.942359427	0.107983312	-8.726898711	2.61751E-18	1.39408E-17
ANKRD31	15.81364218	-0.719978297	0.082489943	-8.72807368	2.59047E-18	1.38006E-17
FIS1	1483.869713	-0.47163348	0.054023177	-8.730206278	2.54209E-18	1.35465E-17
GTF2H4	2.592846237	-0.649055943	0.074345066	-8.730316307	2.53961E-18	1.3537E-17
INO80D	4576.899766	0.28652722	0.032819791	8.730318193	2.53957E-18	1.3537E-17
TCEA2	467.4219027	-0.324107554	0.037121583	-8.730973385	2.5249E-18	1.34659E-17
KDR	29.8021793	-1.0765603	0.12329987	-8.731236264	2.51904E-18	1.34383E-17
AQP11	95.34508422	-0.40010121	0.045818106	-8.732382198	2.49364E-18	1.33064E-17
RMI2	173.3990001	-0.440408464	0.050433765	-8.732412926	2.49296E-18	1.33064E-17
DDI1	7.820035693	-1.031793147	0.118146582	-8.733161224	2.47652E-18	1.32223E-17
IRF6	40.17627117	-0.861000869	0.098589707	-8.733171987	2.47628E-18	1.32223E-17
KCNH2	855.8137645	-0.869306101	0.099536502	-8.733540759	2.46822E-18	1.31852E-17
MSL3	1760.842482	0.245843406	0.028145733	8.734660048	2.4439E-18	1.30588E-17
DNAH7	52.56126571	-0.504818065	0.057789105	-8.735523153	2.42531E-18	1.2963E-17
EFR3B	67.12030243	-0.626399127	0.071688586	-8.73778048	2.37735E-18	1.27101E-17
STAMBPL1	202.6189788	-0.430266775	0.049233243	-8.739354715	2.34446E-18	1.25377E-17
KLF1	597.9225221	-0.888361227	0.101599547	-8.743751812	2.25495E-18	1.20623E-17
ATP10B	17.52974316	-0.815458036	0.093252482	-8.7446255	2.23757E-18	1.19726E-17
CDYL2	1366.446105	0.552289538	0.063145427	8.746310886	2.20441E-18	1.17985E-17
FUT7	149.3968008	-0.666667833	0.076183732	-8.750789895	2.11865E-18	1.13425E-17
PRKAA2	1.985749568	-1.488604167	0.170088986	-8.751913925	2.09764E-18	1.12332E-17
PCDHB3	2.484683355	-1.811401168	0.206896958	-8.755088434	2.03944E-18	1.09244E-17
ZSCAN4	4.65245329	-1.127262805	0.12875116	-8.755360394	2.03452E-18	1.09011E-17
TRMO	508.5092587	0.2564337	0.029287469	8.755748052	2.02754E-18	1.08667E-17
NME3	420.9604117	-0.442400496	0.050514703	-8.757856003	1.98999E-18	1.06683E-17
MYO16	36.46376582	-1.04349694	0.119143229	-8.758340287	1.98146E-18	1.06255E-17
DPYSL5	4.51731091	-0.88599314	0.101154592	-8.758802968	1.97335E-18	1.05849E-17
FAM110D	8.242735462	-0.722594958	0.082499203	-8.75881136	1.9732E-18	1.05849E-17

UPF3A	1032.449062	-0.259780836	0.029656829	-8.759562034	1.9601E-18	1.05196E-17
PAXX	580.332637	-0.430251927	0.049117427	-8.759659357	1.95841E-18	1.05134E-17
MECOM	398.3790049	-1.668214289	0.190416978	-8.760848454	1.93786E-18	1.0406E-17
JSRP1	37.23444897	-0.637796925	0.072797879	-8.761201998	1.93179E-18	1.03762E-17
SZRD1	3280.678277	0.245144211	0.027980123	8.761370093	1.92891E-18	1.03636E-17
METTL1	217.6935097	-0.37625615	0.042943973	-8.761558878	1.92569E-18	1.03491E-17
RASL11B	4.804008012	-0.785932855	0.089688722	-8.762895004	1.90299E-18	1.02299E-17
OSBPL7	894.381326	-0.263775669	0.030100114	-8.76327821	1.89653E-18	1.0198E-17
PRPF19	2521.432434	-0.24339629	0.027774556	-8.763282924	1.89645E-18	1.0198E-17
PARG	472.9717848	-0.323539019	0.036908087	-8.766073906	1.85005E-18	9.95353E-18
RFC2	781.4397362	-0.247049042	0.028180716	-8.766599034	1.84144E-18	9.90996E-18
BCL6	2816.014097	0.567285985	0.064708997	8.766725031	1.83938E-18	9.90161E-18
MAP2K6	379.1501948	-0.539184677	0.061500918	-8.767099585	1.83328E-18	9.87145E-18
TAF3	639.0639558	-0.305540241	0.034848818	-8.767592765	1.82527E-18	9.83102E-18
ADAMTS13	121.5181764	-0.392557712	0.044772182	-8.76789317	1.8204E-18	9.80753E-18
BCL2L11	1161.209628	0.557490126	0.063581708	8.768089885	1.81723E-18	9.79311E-18
GORASP2	2650.751782	0.299676587	0.034175262	8.768815989	1.80555E-18	9.73286E-18
TAL1	1721.670609	-0.846365978	0.096512188	-8.769524296	1.79423E-18	9.67449E-18
MLH1	1216.857342	-0.183233054	0.020893243	-8.769967263	1.78718E-18	9.63916E-18
SCNN1D	204.4882919	-0.415989416	0.047419803	-8.772483031	1.74769E-18	9.42875E-18
B4GALNT4	22.91410825	-0.995900605	0.113495057	-8.774836779	1.71152E-18	9.23616E-18
TIFAB	204.9322185	-1.047870317	0.119404649	-8.77579164	1.69706E-18	9.16065E-18
B3GALNT1	234.7965257	-0.489955399	0.055828068	-8.776148237	1.69169E-18	9.13418E-18
RPS6KB1	1790.60585	0.297661398	0.033916368	8.776334666	1.68889E-18	9.12158E-18
GFOD2	680.3492136	-0.337728658	0.03848132	-8.776431241	1.68744E-18	9.11627E-18
KPTN	186.2001084	-0.358064782	0.040796321	-8.776889021	1.68059E-18	9.08176E-18
UMPS	523.7071975	-0.292958448	0.033375151	-8.777741625	1.6679E-18	9.01568E-18
PIEZO2	320.9967944	-1.17194902	0.133490561	-8.779265076	1.64546E-18	8.89686E-18
MARVELD3	8.462652478	-0.743015643	0.08463076	-8.779498647	1.64205E-18	8.88086E-18
ANKRD27	2548.81538	-0.390914402	0.044522477	-8.780158478	1.63244E-18	8.83135E-18
ADAM7	1.498419973	-1.502001441	0.171057634	-8.780674702	1.62497E-18	8.79334E-18
CRB1	18.65925693	-0.797298436	0.090795292	-8.781275069	1.61632E-18	8.74894E-18
OR51M1	7.100349592	-0.786880228	0.089604802	-8.781674795	1.61058E-18	8.72031E-18
LRIG3	4.827155623	-1.303409833	0.148410642	-8.78245531	1.59944E-18	8.66239E-18
ZNF699	273.1792381	0.333183661	0.037929691	8.784244039	1.5742E-18	8.52802E-18
EXOC6	913.9457263	-0.268896755	0.030610714	-8.784400003	1.57201E-18	8.51856E-18
GJB4	1.348007027	-1.184735803	0.134844226	-8.785958708	1.55037E-18	8.40357E-18

NAPSA	41.52519428	-0.660235024	0.075145408	-8.786099355	1.54843E-18	8.39539E-18
OR2AJ1	6.516681292	-0.880366032	0.100199466	-8.786134934	1.54794E-18	8.39506E-18
HIGD1C	1.969701375	-0.796661464	0.090668763	-8.786504148	1.54286E-18	8.36984E-18
MGST2	519.7938459	-0.498842434	0.056769466	-8.78716087	1.53387E-18	8.32338E-18
ZNF468	514.9826475	0.316279238	0.035992275	8.787419962	1.53034E-18	8.30652E-18
LGI1	2.802598014	-0.936559521	0.106578992	-8.787468338	1.52968E-18	8.30525E-18
VANGL1	424.951499	-0.575295764	0.065464222	-8.787941696	1.52325E-18	8.27263E-18
PARVG	3715.023652	-0.372227299	0.042354998	-8.78827331	1.51876E-18	8.25055E-18
CUEDC2	831.1565222	-0.39897502	0.045392471	-8.789453565	1.50289E-18	8.1666E-18
TEX9	57.91226775	-0.525016769	0.059723607	-8.790774635	1.48532E-18	8.07337E-18
SIGMAR1	777.7313002	-0.585535934	0.06660701	-8.790905505	1.48359E-18	8.06621E-18
ZNF717	198.0152809	-0.56696201	0.06448964	-8.791520815	1.47549E-18	8.02438E-18
NCAM2	24.29870747	-1.108700582	0.126107641	-8.791700291	1.47313E-18	8.0138E-18
TLCD1	53.66029684	-0.457521879	0.052028291	-8.793713336	1.44696E-18	7.87363E-18
TRIB3	560.8598209	0.58278314	0.066269935	8.794080395	1.44224E-18	7.85012E-18
C9orf24	12.68787367	-0.566175077	0.064359397	-8.797084908	1.40416E-18	7.64496E-18
DNAH1	4142.358405	-0.398112679	0.045254353	-8.797223933	1.40242E-18	7.63762E-18
ZNF445	2422.498949	-0.374984131	0.042623234	-8.797646252	1.39716E-18	7.61106E-18
RNF185	420.1507965	0.249042254	0.028306348	8.798106089	1.39144E-18	7.58206E-18
WNT2B	90.83915891	-0.491227145	0.055829814	-8.79865275	1.38468E-18	7.54732E-18
STYK1	51.17208694	-0.64573856	0.073351153	-8.803386654	1.32748E-18	7.23755E-18
ACVR2B	251.1562386	-0.686517229	0.077952222	-8.806897529	1.28657E-18	7.01646E-18
GSTM3	132.914467	-0.741211234	0.084161948	-8.806963843	1.28581E-18	7.01427E-18
TMEM191C	26.88890638	-0.399983323	0.045413434	-8.807599082	1.27855E-18	6.97659E-18
PLAUR	5018.256573	0.614172717	0.069723355	8.808708617	1.26596E-18	6.90981E-18
AHSA1	1154.233842	-0.333010353	0.037801894	-8.80935635	1.25866E-18	6.87192E-18
CHST5	3.366071152	-0.762232461	0.086516561	-8.810249221	1.24868E-18	6.81931E-18
CASP8AP2	664.3693569	-0.36049728	0.040916793	-8.810496936	1.24592E-18	6.80615E-18
BCL7B	1987.976509	0.360140388	0.040865087	8.812911286	1.21937E-18	6.66296E-18
MUC4	42.25115746	-0.831183864	0.094311074	-8.813215934	1.21606E-18	6.64673E-18
RAB37	3047.536832	-0.439618687	0.04988097	-8.813354828	1.21455E-18	6.64035E-18
HAS1	92.64685155	1.110145431	0.125954606	8.81385342	1.20916E-18	6.61272E-18
FBXO32	363.3345648	0.55083197	0.062495635	8.813927141	1.20837E-18	6.61021E-18
STKLD1	87.2526158	-0.400803652	0.045460233	-8.816577076	1.18012E-18	6.4575E-18
CNFN	17.6532998	-0.93668659	0.106240629	-8.816651372	1.17934E-18	6.45502E-18
ADAMTSL1	4.440963499	-0.907961887	0.102952913	-8.81919572	1.15285E-18	6.3118E-18
ATP4B	2.95371165	-0.993325412	0.112622845	-8.81992828	1.14533E-18	6.2724E-18

ART4	80.51914432	-0.929612327	0.105389658	-8.820716817	1.13729E-18	6.23012E-18
NES	10.20703747	-0.81934622	0.092882909	-8.82128075	1.13158E-18	6.20056E-18
PISD	1424.798578	-0.197488123	0.022384844	-8.822403191	1.12029E-18	6.14042E-18
TNFRSF12A	461.9033803	0.61433524	0.069630379	8.822804825	1.11628E-18	6.12014E-18
ZNF250	323.8374458	-0.331408562	0.037558813	-8.823722922	1.10716E-18	6.07185E-18
SUN3	4.291313313	-0.605259313	0.068591881	-8.824066448	1.10377E-18	6.05494E-18
CALR	32435.50495	0.382585059	0.043355833	8.824304166	1.10142E-18	6.04379E-18
PHKB	2655.146045	-0.270104818	0.030599536	-8.827088673	1.07436E-18	5.89691E-18
SNX30	1879.347756	0.371386562	0.042072681	8.827261658	1.0727E-18	5.88945E-18
PKDCC	19.7314474	-0.982519456	0.111297772	-8.827844764	1.06712E-18	5.86048E-18
AZI2	452.8460546	0.312494741	0.035397708	8.82810666	1.06463E-18	5.84842E-18
FAF2	2221.091372	0.255012189	0.028886312	8.828132421	1.06438E-18	5.84842E-18
METTL5	325.6819565	-0.333761363	0.037806178	-8.828222838	1.06352E-18	5.84563E-18
SEPTIN3	14.99625641	-0.861736113	0.09760184	-8.829097005	1.05524E-18	5.80175E-18
C3orf80	490.5719647	-1.222254626	0.138430576	-8.829368945	1.05268E-18	5.78929E-18
TRIM16	169.5872992	-0.414799299	0.046977179	-8.829804264	1.04859E-18	5.76843E-18
ZNF326	4108.012547	0.319860372	0.036220507	8.830919241	1.03819E-18	5.71281E-18
ZDHHC15	28.65509401	-0.825718895	0.093501648	-8.831062448	1.03686E-18	5.70711E-18
OR13C5	2.999424562	-0.899312245	0.101799648	-8.834139049	1.00872E-18	5.55378E-18
NDUFB2	562.4007135	-0.493269933	0.055833975	-8.834583805	1.00472E-18	5.53329E-18
SV2B	42.10979939	-0.874693189	0.098996909	-8.835560571	9.95976E-19	5.4867E-18
PSPN	128.3230808	-0.331556414	0.037517584	-8.837360527	9.80065E-19	5.40057E-18
PCDHB12	6.776959796	-1.230332941	0.139201395	-8.838510137	9.70034E-19	5.3468E-18
MTR	2404.899995	-0.339378258	0.038395976	-8.838901817	9.6664E-19	5.32959E-18
FGFBP3	77.98704411	-0.527895383	0.059723181	-8.839036674	9.65474E-19	5.32466E-18
SCML1	245.3114886	0.763176802	0.08632208	8.841038126	9.48333E-19	5.2316E-18
UQCR10	394.4679843	-0.664168133	0.0751217	-8.841228732	9.46716E-19	5.22415E-18
CEP63	719.051436	-0.248649526	0.028123052	-8.841484334	9.44553E-19	5.21368E-18
KLHDC3	1611.252257	-0.309158743	0.034961234	-8.842901297	9.32646E-19	5.14942E-18
NAB2	856.7290226	0.490385781	0.055450876	8.843607387	9.26768E-19	5.11841E-18
PTPN12	2671.420434	0.334672881	0.037836557	8.84522551	9.13437E-19	5.0462E-18
S1PR3	319.9205629	0.71606685	0.080948033	8.846006775	9.07068E-19	5.01243E-18
ARTN	243.65315	-0.761019595	0.086025692	-8.846422226	9.03699E-19	4.99523E-18
RGS14	830.5467841	-0.438802778	0.049594287	-8.847849343	8.9222E-19	4.93317E-18
IGIP	41.43249445	-0.639938754	0.072323306	-8.848306211	8.88576E-19	4.91441E-18
IFTAP	184.8977222	-0.505216264	0.05709003	-8.849465657	8.79393E-19	4.865E-18
GTF2H5	175.2438958	-0.411422116	0.046457058	-8.855965786	8.29623E-19	4.59096E-18

COMMD1	304.5564497	-0.37583282	0.04243334	-8.857017238	8.21838E-19	4.54916E-18
PRPF31	548.7803478	-0.239800079	0.02707289	-8.857572384	8.17757E-19	4.52785E-18
INCENP	1074.816369	-0.365910609	0.041308744	-8.857945573	8.15025E-19	4.514E-18
SIX1	2.760967764	-1.10189922	0.124391142	-8.858341563	8.12136E-19	4.49927E-18
ATG12	1389.123891	0.20538985	0.023184997	8.858739408	8.09243E-19	4.48452E-18
DLX6	2.086739519	-1.294051126	0.146060061	-8.859719211	8.02162E-19	4.44654E-18
ZNF320	511.8137305	-0.35348219	0.039893629	-8.860617567	7.95724E-19	4.4121E-18
SNTG1	2.263723164	-1.459319573	0.164624952	-8.864510224	7.68411E-19	4.26186E-18
STAG2	6511.506965	0.275471233	0.031073452	8.865163413	7.63919E-19	4.23815E-18
TSPAN5	451.7026776	-0.819916581	0.092476571	-8.866208751	7.56785E-19	4.19976E-18
CKAP5	3368.591809	-0.303513672	0.034229951	-8.866903603	7.5208E-19	4.17483E-18
DGAT2	185.7021835	0.73724836	0.083130908	8.868522873	7.41225E-19	4.11575E-18
ONECUT1	2.343304991	-1.078369994	0.121590849	-8.868841707	7.39107E-19	4.10515E-18
CCN2	172.9321851	-1.194639656	0.134697611	-8.869048563	7.37735E-19	4.09869E-18
SLC38A7	285.4198492	-0.285398885	0.03217753	-8.869508696	7.34693E-19	4.08295E-18
ZSCAN21	96.61566772	-0.244630478	0.027580829	-8.869584034	7.34196E-19	4.08135E-18
COL12A1	24.80744155	-0.89146657	0.100500193	-8.870297089	7.2951E-19	4.05645E-18
SRSF10	9099.582675	0.25886924	0.029177047	8.872359027	7.16125E-19	3.98315E-18
ADAM21	7.81088852	-0.771177217	0.086912841	-8.872995203	7.12044E-19	3.96158E-18
RHPN1	886.4940647	-0.615892002	0.069398966	-8.874656814	7.01494E-19	3.90399E-18
WASF1	619.4335314	-0.750299719	0.084541077	-8.874972287	6.99508E-19	3.89405E-18
CCDC125	978.7723431	-0.367041908	0.04135687	-8.874992376	6.99382E-19	3.89405E-18
PHOSPHO2	44.23211071	-0.399162007	0.04497184	-8.875821059	6.94194E-19	3.86666E-18
MSRB2	253.1735268	-0.537218332	0.060519556	-8.876772494	6.88284E-19	3.83484E-18
PPP1R2B	6.903436093	0.721896967	0.081319894	8.877249271	6.85341E-19	3.81953E-18
ENAH	153.377278	-0.847804194	0.095484686	-8.87895465	6.74916E-19	3.7625E-18
CHMP7	1022.659583	0.428218661	0.048227408	8.879155551	6.73698E-19	3.75678E-18
SPRN	22.28143554	-0.603976618	0.06801038	-8.8806535	6.64687E-19	3.70759E-18
NPIPB11	127.5435193	-0.359352459	0.040463067	-8.880998948	6.62626E-19	3.69714E-18
SLC35B1	1613.563828	0.276338258	0.031110342	8.882520878	6.5362E-19	3.64793E-18
FRMD4A	333.2794578	-0.670214965	0.075444393	-8.883562287	6.47527E-19	3.61496E-18
ADGRF3	113.1022971	-0.411092196	0.046275009	-8.883676267	6.46864E-19	3.61229E-18
SAMD5	4.070594181	-0.987409356	0.111133124	-8.884923964	6.39645E-19	3.57299E-18
PON3	3.794063772	-1.111200879	0.125022304	-8.888021132	6.22069E-19	3.47581E-18
SLC38A5	764.1572937	-0.500027843	0.056257563	-8.8881888	6.21131E-19	3.47156E-18
FPGT	120.0089395	-0.438276916	0.049299664	-8.890058917	6.10765E-19	3.4146E-18
NLRP5	3.373401111	-1.135149558	0.127641631	-8.893254845	5.93445E-19	3.31871E-18

LAMP2	5870.811487	0.261115041	0.029358775	8.893935051	5.89822E-19	3.29939E-18
ACACA	2750.380582	-0.328592022	0.036942061	-8.894793919	5.85278E-19	3.27491E-18
XK	1054.26805	-0.903182449	0.101539475	-8.894889924	5.84772E-19	3.27302E-18
ARMH3	1409.811209	-0.168272872	0.018916291	-8.895658639	5.80738E-19	3.25137E-18
AC087289.3	2.717774301	-0.649686315	0.073033252	-8.895760492	5.80206E-19	3.24932E-18
GOLT1A	2.538311417	-1.128943731	0.12689519	-8.896662919	5.7551E-19	3.22394E-18
GPR45	4.884284226	-0.693667585	0.077953293	-8.898502681	5.66051E-19	3.17186E-18
NHLRC4	98.57565206	-0.528207233	0.059353687	-8.899316268	5.61918E-19	3.1496E-18
SERTAD2	7750.067076	0.318483124	0.035782128	8.900619944	5.55356E-19	3.11372E-18
FOXD4L1	17.23961408	-0.823193636	0.092464941	-8.902764976	5.44724E-19	3.05498E-18
RIOX2	215.6407743	-0.463742253	0.052085775	-8.903433888	5.4145E-19	3.03749E-18
PRRT2	14.47762423	-0.766250896	0.08606118	-8.903560174	5.40834E-19	3.03491E-18
NALCN	5.684433882	-0.893096716	0.100305228	-8.903790344	5.39713E-19	3.02948E-18
ZNF444	336.518012	-0.327285018	0.036756148	-8.904225056	5.37603E-19	3.0185E-18
DDB2	1251.090369	-0.253459479	0.028461735	-8.905271542	5.32555E-19	2.99102E-18
TAS2R20	27.99973817	-0.655451376	0.073578199	-8.908228032	5.18547E-19	2.91318E-18
CCDC68	32.36719719	-0.995194752	0.111704063	-8.909208167	5.13983E-19	2.88837E-18
TMC7	5.160673926	-0.749527326	0.084095983	-8.912760159	4.97776E-19	2.79809E-18
WNT7A	10.36479639	0.735093936	0.082474038	8.913034398	4.96546E-19	2.79198E-18
PGR	2.063753935	-1.340352871	0.150356403	-8.914504777	4.90001E-19	2.75598E-18
PIGU	365.0151906	-0.392774307	0.044056467	-8.915247481	4.86728E-19	2.73835E-18
ZNF253	203.0571631	-0.392426476	0.044017187	-8.915301085	4.86493E-19	2.73781E-18
ASAH2	7.40018679	-1.004084275	0.112618702	-8.915786234	4.84367E-19	2.72664E-18
CHM	315.528375	-0.42320019	0.047459505	-8.917079654	4.78745E-19	2.69577E-18
ANKRD12	3592.243819	0.327321532	0.03669599	8.919817572	4.67057E-19	2.63071E-18
RYK	1256.137131	0.366581622	0.04109669	8.919979317	4.66375E-19	2.62762E-18
PAX7	1.456231178	-1.305131195	0.146314144	-8.920061714	4.66028E-19	2.62643E-18
ARMCX4	235.3397366	-0.358908493	0.040226497	-8.92219101	4.57153E-19	2.57715E-18
HSF2	1142.418432	0.392528542	0.04398658	8.923824908	4.50456E-19	2.54012E-18
CDKL1	233.1638585	-0.319542154	0.035807577	-8.923869862	4.50273E-19	2.53982E-18
VHLL	2.234859068	-0.935146811	0.104768956	-8.925800602	4.42487E-19	2.49663E-18
MALL	2.251047099	-0.928946174	0.104071712	-8.926019896	4.41611E-19	2.4924E-18
COTL1	8920.360584	0.556389466	0.062328351	8.926747783	4.38716E-19	2.47678E-18
SHLD1	128.5633304	-0.37095284	0.041552817	-8.927261044	4.36686E-19	2.46603E-18
TMCO6	335.0655475	-0.265243972	0.029706834	-8.928718895	4.3097E-19	2.43445E-18
ADH1A	1.865173105	-1.137283427	0.127361207	-8.929590549	4.27588E-19	2.41605E-18
PPP1R3F	93.28172948	-0.331564156	0.037129216	-8.930006988	4.25982E-19	2.40766E-18

KCTD13	700.0944576	-0.222120246	0.024871542	-8.9306987	4.23327E-19	2.39335E-18
RRP1B	1254.329526	-0.289354875	0.032388617	-8.933844773	4.11455E-19	2.3269E-18
FAM160B1	3685.383172	0.348793503	0.039036205	8.935128398	4.06706E-19	2.30071E-18
CLPB	1596.35378	-0.350780949	0.039257445	-8.935399292	4.0571E-19	2.29574E-18
ST8SIA2	2.711119522	-1.297342611	0.145187734	-8.935621339	4.04896E-19	2.2918E-18
MS4A1	420.4160486	0.713433622	0.079840305	8.935757735	4.04397E-19	2.28963E-18
MINPP1	506.8003653	-0.663872714	0.074290642	-8.936155361	4.02945E-19	2.28207E-18
ECH1	1298.891936	-0.405840902	0.045415395	-8.936196614	4.02795E-19	2.28188E-18
GALNT3	862.0509366	0.566782111	0.06342477	8.936289545	4.02457E-19	2.28063E-18
PDXP	79.06154962	-0.397747599	0.04450779	-8.936583879	4.01386E-19	2.27522E-18
SLC23A3	11.40419856	-0.650132453	0.072741201	-8.937609578	3.97679E-19	2.25486E-18
RIDA	62.8231022	-0.543463136	0.060793782	-8.939452672	3.91103E-19	2.21821E-18
FBXL4	847.346855	-0.324642224	0.036310101	-8.940823984	3.86279E-19	2.19149E-18
OPRM1	8.960599406	-0.89547411	0.100138936	-8.942317014	3.81095E-19	2.1627E-18
AC013477.1	3.790688657	-0.814489523	0.091074735	-8.943089666	3.78438E-19	2.14825E-18
RTN1	70.76320794	-1.153498698	0.128972665	-8.94374556	3.76198E-19	2.13615E-18
AP3M2	509.8418157	0.359255205	0.040167384	8.943953343	3.75491E-19	2.13276E-18
YIF1A	581.7069077	-0.389480113	0.043545227	-8.94426642	3.74428E-19	2.12734E-18
NRXN1	11.53331737	-1.055427128	0.117973372	-8.946316493	3.67542E-19	2.08882E-18
GPRC5A	72.39667985	1.041172596	0.116377203	8.946533903	3.66819E-19	2.08532E-18
CLIP1	3125.46101	0.262500617	0.029340706	8.946635975	3.6648E-19	2.084E-18
EFS	5.278520913	-1.209354692	0.135169312	-8.946961958	3.654E-19	2.07846E-18
P2RX2	20.97957293	-0.648783012	0.072500721	-8.948642231	3.59882E-19	2.04766E-18
RAB7B	362.7529195	-0.873788364	0.097644585	-8.948661772	3.59818E-19	2.04766E-18
DUSP6	3277.498383	0.732309404	0.081796429	8.952828581	3.46488E-19	1.9726E-18
CNOT6L	4153.049097	0.324470601	0.036230291	8.955782274	3.37335E-19	1.92105E-18
ITGB1	4513.918035	0.3086769	0.034464208	8.956448368	3.35304E-19	1.91004E-18
ATF7-NPFF	10.13453338	-0.465623234	0.051985669	-8.956761363	3.34354E-19	1.90519E-18
ARR3	33.85631341	-0.448390173	0.050060593	-8.956948878	3.33786E-19	1.9025E-18
KIAA0753	482.7323281	-0.263090209	0.029371265	-8.957401304	3.3242E-19	1.89527E-18
SLC9B2	449.5977543	-0.369292351	0.041227433	-8.95744233	3.32296E-19	1.89512E-18
CRYZ	134.4923064	-0.518138456	0.057840105	-8.958117516	3.30268E-19	1.8841E-18
GRIN2A	4.360193003	-0.950529483	0.10610261	-8.958587222	3.28865E-19	1.87664E-18
TBL1X	1386.615835	0.393579638	0.043930192	8.959205965	3.27025E-19	1.86668E-18
CCDC43	332.7685318	0.270765075	0.030218316	8.960296738	3.23806E-19	1.84885E-18
AMOT	120.3769373	-0.626462435	0.069905499	-8.961561597	3.20112E-19	1.82829E-18
NPHP4	691.5558042	-0.359901764	0.040157477	-8.962260387	3.1809E-19	1.81727E-18

IMPG2	71.52868076	-0.576338332	0.064295427	-8.963908595	3.13369E-19	1.79082E-18
TAS2R50	16.22471009	-0.761228796	0.084919972	-8.964072569	3.12903E-19	1.78868E-18
CLDN20	63.32424146	-0.605896438	0.067575109	-8.966266514	3.06735E-19	1.75394E-18
CACNA1A	159.4551732	-0.568774171	0.063433656	-8.966441554	3.06248E-19	1.75167E-18
ADAM17	3831.162833	0.355021423	0.039592506	8.966884421	3.0502E-19	1.74515E-18
CENPK	332.388091	-0.39181073	0.043695132	-8.966919474	3.04923E-19	1.7451E-18
TF	80.56033067	-0.739267528	0.082443684	-8.966939481	3.04868E-19	1.7451E-18
EFR3A	2240.320431	0.356040452	0.039704956	8.967153892	3.04275E-19	1.74241E-18
STAMBP	903.7738906	-0.220523675	0.024591823	-8.967357706	3.03712E-19	1.7397E-18
SLC19A3	4.350425055	-0.869187327	0.096926134	-8.967522917	3.03257E-19	1.7376E-18
CFAP57	6.329323073	-0.884091738	0.098582327	-8.968055105	3.01796E-19	1.72974E-18
PPM1B	1112.088399	0.325728785	0.036319504	8.968425987	3.00782E-19	1.72443E-18
MBD5	1212.504957	-0.308019271	0.034343515	-8.968775403	2.99829E-19	1.71947E-18
SCGN	1.97637963	-1.48364125	0.165421864	-8.968834048	2.9967E-19	1.71906E-18
DUSP23	340.1556471	-0.714042342	0.079590696	-8.971429806	2.9269E-19	1.67951E-18
LMBR1L	2874.488273	0.35755167	0.03984192	8.974258001	2.85268E-19	1.6374E-18
ZNF660	287.3081397	-0.444365227	0.049504063	-8.976338477	2.79927E-19	1.60722E-18
WDR88	96.42986342	-0.427914045	0.047669472	-8.976689361	2.79036E-19	1.60258E-18
SMPD3	169.9123762	-0.525918065	0.058586308	-8.976808522	2.78734E-19	1.60131E-18
LYPLA1	2362.082295	0.324621156	0.036157809	8.977898867	2.75987E-19	1.58599E-18
NUDT7	34.13212376	-0.686504688	0.076460868	-8.978510244	2.74458E-19	1.57767E-18
MTIF3	445.9830683	-0.322719964	0.035941419	-8.979054651	2.73103E-19	1.57034E-18
NXPE4	1.222789441	-1.175395071	0.130880702	-8.980659921	2.69148E-19	1.54805E-18
NOP16	204.4253128	-0.387203859	0.043113441	-8.981047514	2.68201E-19	1.54306E-18
NUP188	3043.532868	-0.211399469	0.023538137	-8.981147123	2.67958E-19	1.54212E-18
CCN1	51.50119039	-0.702302444	0.078197408	-8.98114737	2.67958E-19	1.54212E-18
P2RX6	20.76352689	-0.645136166	0.071818034	-8.982927179	2.63657E-19	1.51826E-18
ORAI3	284.5960076	-0.348138668	0.038752273	-8.983696736	2.61819E-19	1.50812E-18
SMAD7	930.1753045	0.674708902	0.075100936	8.984027842	2.61032E-19	1.50402E-18
PIP4K2A	3696.83324	-0.429878599	0.047840843	-8.985598294	2.5733E-19	1.48314E-18
KIAA1614	96.49266048	-0.667784039	0.074315914	-8.985747567	2.56981E-19	1.48156E-18
STK11IP	1112.085935	-0.288982732	0.03215897	-8.986069385	2.5623E-19	1.47766E-18
CFAP70	140.1268723	-0.494477756	0.055024682	-8.98647188	2.55294E-19	1.4727E-18
TRMT9B	118.206591	-0.870212574	0.096833881	-8.986653851	2.54872E-19	1.4707E-18
COG7	617.1225303	-0.230922612	0.025694484	-8.987244552	2.53506E-19	1.46325E-18
NHSL1	212.6174554	-0.579097195	0.064429438	-8.988083862	2.51578E-19	1.45255E-18
KLHL24	6488.305319	0.407592126	0.045343149	8.989056522	2.49362E-19	1.44018E-18

CAP2	2.493518882	-1.298865478	0.14448191	-8.989813863	2.47649E-19	1.43071E-18
LDB2	13.40536307	-0.979643272	0.108971431	-8.989909245	2.47435E-19	1.42989E-18
PPP1R36	14.78825798	-0.825783456	0.091844195	-8.991133898	2.44693E-19	1.41446E-18
SSH3	918.4204055	-0.340396788	0.037855808	-8.991930205	2.42926E-19	1.40466E-18
NHP2	494.914239	-0.607549303	0.067546567	-8.994525204	2.37255E-19	1.37228E-18
UQCR11	330.1330125	-0.539215308	0.059943151	-8.995444878	2.35277E-19	1.36124E-18
PRKDC	10887.1552	-0.355325689	0.039500074	-8.995570289	2.35009E-19	1.36009E-18
RRP1	657.5806248	-0.318491065	0.035405312	-8.995572919	2.35003E-19	1.36009E-18
INSYN2A	28.55204045	-1.257135685	0.139746085	-8.995856197	2.34398E-19	1.35736E-18
NFKB1	6760.056478	0.422725931	0.046980847	8.997835433	2.30212E-19	1.33351E-18
LIN7C	1381.497353	0.273847216	0.030433362	8.99825706	2.29329E-19	1.32879E-18
SP1	6792.628943	0.240271743	0.02668939	9.002519001	2.20597E-19	1.27857E-18
LTC4S	532.1270115	-0.677356875	0.075219649	-9.005052315	2.15563E-19	1.24977E-18
SESN2	1757.506033	0.521621195	0.057923676	9.005319272	2.15039E-19	1.2471E-18
HERPUD1	4080.478857	0.3928887	0.043624514	9.006145007	2.13427E-19	1.23812E-18
VKORC1	65.4666801	-0.552029884	0.061292432	-9.006493462	2.1275E-19	1.23456E-18
SIN3B	1435.400861	-0.273211999	0.030333427	-9.006961137	2.11845E-19	1.22967E-18
SCARB2	2722.402099	0.273672851	0.030381836	9.007778714	2.10272E-19	1.2209E-18
TMEM98	25.03662906	-1.183562915	0.131379725	-9.008718147	2.08479E-19	1.21085E-18
APOO	114.8121483	-0.389942824	0.043283478	-9.009045609	2.07857E-19	1.2076E-18
ENKD1	190.7191691	-0.354179465	0.039307964	-9.010374246	2.05355E-19	1.19341E-18
FAM71E1	11.98651586	-0.631695183	0.070095467	-9.011926269	2.02468E-19	1.17699E-18
RSPO4	4.605558434	-0.85448971	0.094809243	-9.012725839	2.00997E-19	1.16878E-18
MTFMT	195.3008556	-0.265043929	0.029407212	-9.012888847	2.00699E-19	1.16739E-18
ZNF518A	1140.259503	-0.425095763	0.047164767	-9.012994023	2.00506E-19	1.16662E-18
NR2F1	6.244764672	-1.093191855	0.121276571	-9.014039932	1.98602E-19	1.15589E-18
UBE2Z	2723.45681	0.204213592	0.022654083	9.014427612	1.97901E-19	1.15215E-18
GPR137B	237.5750092	0.424032051	0.047039088	9.014461611	1.9784E-19	1.15213E-18
MAGOHB	227.6547561	-0.341990749	0.037933009	-9.015650326	1.95706E-19	1.14004E-18
RHOBTB2	273.2977046	-0.402926349	0.044691497	-9.015727324	1.95568E-19	1.13958E-18
ZNF747	180.9209419	-0.303119731	0.033620886	-9.01581628	1.9541E-19	1.139E-18
TBX19	131.5024151	-0.410911483	0.045574179	-9.016322179	1.9451E-19	1.13409E-18
KDM4D	10.48784824	-0.539135234	0.059789496	-9.017223272	1.92917E-19	1.12514E-18
IST1	9302.207429	0.325559528	0.036104146	9.01723379	1.92898E-19	1.12514E-18
CDC42BPA	392.0348374	-1.036417864	0.114937083	-9.017262635	1.92848E-19	1.12514E-18
PITPNA	1968.272999	0.271076105	0.03006171	9.017321647	1.92744E-19	1.12513E-18
GFPT1	1947.256356	0.298870513	0.033138121	9.018933727	1.89929E-19	1.10903E-18

SYT10	0.975989393	-1.897070905	0.210321066	-9.019880621	1.88294E-19	1.09982E-18
FANCF	209.0627	-0.357492492	0.039616967	-9.02372199	1.81805E-19	1.06223E-18
SHISAL2B	9.60993341	1.256044487	0.139189392	9.023995788	1.81351E-19	1.05989E-18
MYO5B	18.63139589	-0.680215157	0.075375461	-9.024358116	1.80752E-19	1.05671E-18
CATSPERE	71.62410527	-0.431435673	0.047806238	-9.02467317	1.80233E-19	1.05399E-18
ELF4	5854.232814	0.376072336	0.041669739	9.025070642	1.79579E-19	1.05048E-18
NDC1	699.0608014	-0.283350589	0.031392068	-9.026184327	1.77762E-19	1.04016E-18
NDUFAF8	391.736893	-0.464556955	0.05145754	-9.027966646	1.74891E-19	1.02367E-18
GLIPR1L2	6.665596967	-0.614568043	0.068071645	-9.02825312	1.74434E-19	1.0213E-18
TMEM199	188.0936404	0.27208991	0.030136957	9.028446657	1.74126E-19	1.0198E-18
CEBPG	1653.132072	0.280137891	0.031027045	9.028829121	1.73518E-19	1.01654E-18
MRPL57	990.1392178	-0.588344767	0.065159533	-9.029296904	1.72778E-19	1.01251E-18
ATL1	122.1420595	-0.491459912	0.054428784	-9.029411926	1.72597E-19	1.01175E-18
PABPN1L	54.8649822	-0.603866686	0.066875284	-9.029743823	1.72074E-19	1.00899E-18
ARRDC3	4702.209352	0.576967218	0.0638934	9.030153637	1.71431E-19	1.00552E-18
LMNB2	1924.292924	-0.305781244	0.033859664	-9.030841119	1.70357E-19	9.99521E-19
NDUFB1	658.6644674	-0.639581791	0.07081889	-9.031231538	1.69751E-19	9.9626E-19
PARPBP	279.985341	-0.326143065	0.036112065	-9.031415627	1.69465E-19	9.94883E-19
CRY2	2272.170156	0.33885483	0.037505167	9.034884033	1.64176E-19	9.64124E-19
GOT1	287.8247335	-0.365603428	0.040457353	-9.036760901	1.61383E-19	9.48003E-19
UNCX	26.50986038	2.048489643	0.226674574	9.03713906	1.60826E-19	9.45014E-19
SARDH	134.2581013	-0.661036975	0.073146434	-9.037172903	1.60776E-19	9.45005E-19
PIP4P1	1797.634882	0.250821239	0.027753065	9.037605075	1.60142E-19	9.4156E-19
EPHB1	31.94744854	-0.758540362	0.083930816	-9.037686059	1.60023E-19	9.41146E-19
FOXD1	3.865413232	-1.191680523	0.131855951	-9.037745488	1.59936E-19	9.40917E-19
CACNA2D1	7.662854634	-1.236081857	0.136735578	-9.039943202	1.56753E-19	9.22469E-19
WDR61	619.9694188	-0.317447465	0.035110089	-9.041488489	1.54553E-19	9.09793E-19
CCR4	64.30605106	0.663623132	0.073394573	9.041855579	1.54035E-19	9.07015E-19
MIER3	1157.935497	0.396842155	0.043886643	9.042435983	1.53219E-19	9.02482E-19
DNAJC19	326.2191115	-0.33830934	0.037411675	-9.042881494	1.52595E-19	8.99081E-19
RFFL	1780.895059	0.289845718	0.032051252	9.043194811	1.52158E-19	8.96777E-19
IL37	1.846797678	-0.963184363	0.106508624	-9.043252379	1.52078E-19	8.96574E-19
TP73	86.37461257	-0.738000135	0.08159512	-9.044660256	1.50131E-19	8.85363E-19
MKRN2	2052.358067	0.203623689	0.022511996	9.045119298	1.49502E-19	8.81917E-19
COG6	394.8751548	-0.34833881	0.038509361	-9.045562012	1.48897E-19	8.78615E-19
STAT3	8991.902773	0.262095335	0.02897401	9.045877059	1.48468E-19	8.76349E-19
KLF15	3.634561475	-0.856937948	0.094723331	-9.046746315	1.47292E-19	8.69665E-19

ATRX	3277.977601	-0.332406391	0.036741179	-9.04724336	1.46623E-19	8.65978E-19
TRAF3IP2	1177.495783	0.365135266	0.040358729	9.047244017	1.46622E-19	8.65978E-19
BCAR1	23.89957548	-0.880842696	0.097317863	-9.051192304	1.41416E-19	8.35727E-19
IRAK2	822.026211	0.701683617	0.077522856	9.05131283	1.4126E-19	8.35057E-19
MSN	30888.8951	0.297820899	0.03290344	9.051360675	1.41198E-19	8.34944E-19
STN1	742.8439148	-0.337839183	0.037322918	-9.051788996	1.40645E-19	8.31926E-19
WDR54	226.2033798	-0.454934555	0.050257995	-9.051983697	1.40394E-19	8.30694E-19
NDUFAF5	719.7073109	0.314893748	0.034785238	9.052510997	1.39718E-19	8.26942E-19
SOCS5	1160.507508	0.310975343	0.034352144	9.052574529	1.39636E-19	8.26711E-19
TNFRSF4	160.2937902	0.83525104	0.092254854	9.053735457	1.38159E-19	8.18212E-19
FYN	1688.467791	0.465751801	0.051441636	9.053984911	1.37844E-19	8.16591E-19
OR1B1	2.171307241	-0.992901446	0.109661812	-9.054213346	1.37556E-19	8.15131E-19
ARRB1	3182.206622	-0.359992772	0.039757567	-9.05469823	1.36946E-19	8.11763E-19
EPC1	2605.309004	0.246519761	0.027221981	9.055908329	1.35436E-19	8.03055E-19
ZNF280B	85.50239455	-0.508687514	0.056164833	-9.057046742	1.3403E-19	7.94961E-19
SRRM3	12.35905139	-0.803076189	0.088668629	-9.0570498	1.34026E-19	7.94961E-19
LRRC74B	7.973064177	-0.682217068	0.075299118	-9.060093749	1.30338E-19	7.73534E-19
VCAN	11095.86391	1.04703138	0.115550343	9.061257258	1.28955E-19	7.65558E-19
ATP6V0D1	3679.341068	0.312515377	0.034478667	9.064021537	1.25728E-19	7.46622E-19
LUZP2	1.984523758	-1.78023755	0.196385249	-9.06502683	1.24574E-19	7.39994E-19
SMIM8	265.3659263	-0.386894181	0.042670112	-9.067100139	1.22227E-19	7.26272E-19
CPT1C	30.08531315	-0.739902319	0.08159948	-9.067488141	1.21792E-19	7.23911E-19
NOC4L	504.4429496	-0.317011071	0.034961271	-9.067492633	1.21787E-19	7.23911E-19
LMO7	207.9980494	-0.460841544	0.050815513	-9.0689145	1.20209E-19	7.14932E-19
NUP160	2028.24819	-0.262795695	0.02897139	-9.070869284	1.18071E-19	7.02434E-19
TLR8	274.8802073	0.879788661	0.096983305	9.071547564	1.17338E-19	6.98287E-19
ZNF497	72.08173645	-0.446275104	0.049188205	-9.07280732	1.15989E-19	6.90468E-19
PIGV	597.8054512	-0.345192254	0.038044405	-9.073403967	1.15356E-19	6.86905E-19
ZNFX1	5747.75278	0.389927761	0.042973468	9.073686033	1.15057E-19	6.85337E-19
SHPK	58.42283018	-0.321318391	0.035407547	-9.074856062	1.13828E-19	6.7822E-19
B4GALNT3	49.01041472	-0.794063392	0.087491179	-9.075925116	1.12716E-19	6.71799E-19
WBP1L	817.7044136	-0.467840334	0.051541586	-9.076948667	1.11661E-19	6.65716E-19
MYORG	68.53781257	-0.44388879	0.048894197	-9.078557799	1.10023E-19	6.56149E-19
C1QL3	28.9222423	-0.64689322	0.071253618	-9.078742102	1.09837E-19	6.55238E-19
CCNB3	13.81763509	-0.644053033	0.07094064	-9.078759853	1.09819E-19	6.55238E-19
RRS1	158.6844703	-0.438440244	0.048289529	-9.079406024	1.09169E-19	6.51651E-19
SALL4	67.17748208	-0.98760017	0.108766318	-9.080018422	1.08556E-19	6.48193E-19

FOXL2NB	3.5583887	-1.145755466	0.126167144	-9.081250676	1.07334E-19	6.41091E-19
CSAD	599.6907118	-0.342314172	0.037692083	-9.081858588	1.06736E-19	6.37714E-19
CENPBD1	120.0359772	-0.418274979	0.046038003	-9.085428434	1.03291E-19	6.17318E-19
PPP1R18	5725.674086	0.394974561	0.043471876	9.085749219	1.02987E-19	6.15688E-19
MRPL17	287.1765382	-0.383181151	0.042173589	-9.085808525	1.02931E-19	6.15541E-19
RLIM	6542.18718	0.335263819	0.036897745	9.086295733	1.02471E-19	6.12977E-19
ACOT4	37.05996232	-0.697889685	0.076802267	-9.086837057	1.01962E-19	6.1012E-19
GCC2	2168.232451	0.333825194	0.036734042	9.087624917	1.01226E-19	6.05901E-19
AC006254.1	72.46182677	-0.442921836	0.048726523	-9.08995359	9.90813E-20	5.93245E-19
USP17L7	2.216317246	-1.197032506	0.131664154	-9.091559641	9.76284E-20	5.84725E-19
ERMAP	847.1826879	-0.541118065	0.059507771	-9.093233648	9.61364E-20	5.75965E-19
P3H2	17.541867	-0.988804818	0.108732391	-9.093930634	9.55218E-20	5.72458E-19
MED6	749.0305935	0.260397374	0.028630477	9.095111143	9.44898E-20	5.66447E-19
SUGCT	9.644752209	-0.590638333	0.064934947	-9.095846907	9.38521E-20	5.62797E-19
SEC31B	1488.084352	-0.381050295	0.041892552	-9.095895895	9.38098E-20	5.62715E-19
ZNF92	762.6564045	0.380907765	0.041876588	9.095959866	9.37546E-20	5.62556E-19
SPTBN2	493.411068	-0.640457852	0.070394742	-9.09809215	9.19325E-20	5.51792E-19
ASB9	54.21727559	-0.830796261	0.09128821	-9.100805723	8.96641E-20	5.38342E-19
KLHL26	382.1832016	0.349749364	0.038420224	9.103262035	8.76586E-20	5.26463E-19
TIMM8B	349.2705496	-0.58286721	0.064022228	-9.104138118	8.69541E-20	5.22392E-19
AP1G1	8019.847221	0.362964705	0.03986311	9.105278097	8.60457E-20	5.17093E-19
GRIA4	5.842388522	-0.814136426	0.089406513	-9.106008053	8.5469E-20	5.13785E-19
AMIGO2	100.7384365	0.651127165	0.071501345	9.106502272	8.50807E-20	5.11608E-19
CCDC82	1600.118488	0.331228583	0.036372011	9.106688757	8.49346E-20	5.10887E-19
RIMS1	3.471904189	-1.196092244	0.131322538	-9.108050001	8.38759E-20	5.04674E-19
ANKRD18B	139.3713632	-1.25315778	0.137544034	-9.110957008	8.16584E-20	4.91482E-19
MELTF	405.3558934	0.561842599	0.061662041	9.111644514	8.11425E-20	4.88528E-19
SLIT1	128.9563662	-0.553647063	0.06076166	-9.11178302	8.1039E-20	4.88054E-19
VPS52	1102.786928	-0.254362678	0.027914347	-9.1122562	8.06862E-20	4.86079E-19
CAPS2	69.51290291	-0.552995476	0.060678973	-9.113461367	7.97946E-20	4.80856E-19
GNS	3962.487323	0.434907579	0.047720974	9.113551964	7.9728E-20	4.80603E-19
USP31	536.1350166	-0.466216077	0.051151148	-9.114479327	7.90491E-20	4.76657E-19
MTA1	2432.787562	-0.294936312	0.032350811	-9.116813616	7.73655E-20	4.66649E-19
CYP2C9	2.703508235	-1.377326222	0.151071429	-9.117052991	7.71948E-20	4.65763E-19
RAD9A	487.2850824	-0.274276799	0.030081584	-9.11776459	7.66898E-20	4.62858E-19
FANCL	395.6282539	-0.338640896	0.037138031	-9.118439739	7.62136E-20	4.60126E-19
PHB	878.2487036	-0.43612956	0.047818988	-9.120426434	7.48293E-20	4.51908E-19

PRDM6	2.851097725	-1.108496964	0.121531681	-9.121053532	7.43975E-20	4.49439E-19
MARCHF10	4.787405333	-1.053275459	0.115464413	-9.12207865	7.3697E-20	4.45345E-19
GPR35	463.2614875	0.570390846	0.062523901	9.12276481	7.32318E-20	4.4267E-19
NLRP1	4225.891006	0.475355949	0.052103972	9.123219058	7.29254E-20	4.40954E-19
APPL1	3506.877873	0.271947744	0.029806918	9.12364526	7.2639E-20	4.39358E-19
C2orf81	4.652495333	-0.647408433	0.070954523	-9.124272965	7.22194E-20	4.36955E-19
TRDN	2.3769603	-1.507174696	0.165178415	-9.124525736	7.2051E-20	4.36071E-19
POLG2	1323.40474	-0.301515298	0.033039497	-9.125904642	7.11396E-20	4.30688E-19
RMC1	1315.606313	0.256666208	0.028123146	9.126511186	7.07423E-20	4.28416E-19
UCKL1	1156.508949	-0.262478357	0.028756227	-9.127705018	6.99667E-20	4.2385E-19
MYO6	248.390205	-1.16424955	0.127547393	-9.127976065	6.97918E-20	4.22921E-19
ACSL5	788.525873	-0.32557637	0.035665991	-9.128482378	6.94662E-20	4.21078E-19
VEPH1	23.62521842	-0.802180588	0.087871108	-9.129059673	6.90968E-20	4.18969E-19
SEMA3E	2.647083829	-1.305859709	0.143043752	-9.129092961	6.90756E-20	4.18969E-19
NAXE	755.2881854	-0.513829106	0.056279137	-9.130010404	6.84927E-20	4.15564E-19
TGFBI	687.5792719	1.044951022	0.11444998	9.130198358	6.83739E-20	4.14971E-19
ORC3	466.5263388	-0.261352377	0.02862374	-9.1306158	6.81108E-20	4.13502E-19
ZNF138	220.3452498	-0.354515171	0.038808377	-9.135016766	6.53966E-20	3.97148E-19
CAMKMT	174.9952059	-0.371102972	0.04061496	-9.137100588	6.41491E-20	3.89693E-19
TVP23B	486.881881	0.263719492	0.028862013	9.137252147	6.40593E-20	3.89268E-19
TRIM9	42.7455744	-0.656249738	0.071817531	-9.137737323	6.37726E-20	3.87646E-19
BCORL1	894.2999207	-0.332131495	0.036345133	-9.138266129	6.34616E-20	3.85876E-19
PFKFB3	5205.361632	0.554984927	0.060719712	9.140111259	6.23881E-20	3.79466E-19
MSH5-SAPCD1	27.74388992	-0.40034915	0.043790847	-9.142301941	6.11369E-20	3.71971E-19
CD163	725.2765064	1.072256628	0.117247172	9.145266482	5.9483E-20	3.62022E-19
HIVEP1	2976.683853	0.383097836	0.041887986	9.145768743	5.92072E-20	3.60455E-19
THEMIS	91.16282014	0.771924028	0.084382531	9.147912685	5.80442E-20	3.53484E-19
TMEFF2	7.438636963	-1.375441563	0.150297587	-9.151454727	5.6172E-20	3.42189E-19
SLC25A42	422.4866133	-0.352489504	0.038514469	-9.152131877	5.58209E-20	3.40156E-19
SSC4D	26.56494929	-0.521977231	0.057031214	-9.152483311	5.56395E-20	3.39157E-19
TRPT1	293.5843138	-0.34000634	0.037143584	-9.153837686	5.49461E-20	3.35034E-19
NOP14	853.4026697	-0.189266935	0.02067253	-9.155480087	5.41166E-20	3.30079E-19
KLHL3	239.725788	-0.497226117	0.054303496	-9.156429295	5.36429E-20	3.27292E-19
IQCN	1037.533054	-0.450144532	0.049159966	-9.156729899	5.34938E-20	3.26484E-19
BCL3	3260.902655	0.55892378	0.061038125	9.156961747	5.3379E-20	3.25885E-19
AC009690.1	35.77454959	-0.482132019	0.052632441	-9.16035833	5.17253E-20	3.15887E-19
WFDC2	4.798135231	-0.824013133	0.089948569	-9.160936591	5.14488E-20	3.14297E-19

MYOM2	72.49160631	-0.672616099	0.073407504	-9.162770335	5.05818E-20	3.09097E-19
P4HB	16444.57285	0.384772189	0.041989643	9.1635022	5.02398E-20	3.07103E-19
PCDHGA9	37.02374168	-0.866181661	0.094519721	-9.164031117	4.9994E-20	3.05696E-19
NRBF2	466.4616291	0.353099048	0.038526934	9.164992217	4.95505E-20	3.03079E-19
PDF	78.96362299	-0.353108884	0.038519836	-9.166936355	4.86653E-20	2.97757E-19
PXN	1735.615386	0.477851042	0.052121735	9.167980429	4.81963E-20	2.9498E-19
PNMA2	10.49823818	-0.873177762	0.095227666	-9.169370577	4.75789E-20	2.91292E-19
PLEKHA2	3599.709106	0.268026999	0.029228539	9.170044357	4.72824E-20	2.89568E-19
SLC38A10	3152.629344	-0.443341387	0.048343691	-9.170615095	4.70328E-20	2.88129E-19
GAS2L2	8.772223715	-0.663328474	0.072320401	-9.172079627	4.6398E-20	2.84329E-19
ARFGEF2	7069.954314	0.375244199	0.040903353	9.173922741	4.56112E-20	2.79595E-19
HMCN1	21.80643155	-0.904255749	0.098556162	-9.175030109	4.51448E-20	2.76823E-19
HSD17B2	8.43261284	-0.984167163	0.107259972	-9.175530664	4.49356E-20	2.75626E-19
WHAMM	4315.701346	0.440102339	0.047962584	9.175951373	4.47604E-20	2.74638E-19
NOVA1	5.297628003	-1.877435551	0.204439531	-9.183329362	4.17965E-20	2.56532E-19
DIAPH3	292.6634915	-0.4653714	0.050673587	-9.183707448	4.16499E-20	2.55713E-19
HOXC10	1.55109663	-1.518454739	0.165336261	-9.184039413	4.15217E-20	2.55006E-19
NELFB	1491.17579	-0.271891822	0.029603012	-9.184599927	4.1306E-20	2.53761E-19
ITIH4	25.69882947	-0.43694769	0.047569119	-9.185532492	4.09496E-20	2.51651E-19
FEN1	610.4975169	-0.360261044	0.039216204	-9.186535352	4.05698E-20	2.49395E-19
CDC45	508.3260515	-0.394326164	0.042920015	-9.18746573	4.02205E-20	2.47325E-19
TRAF6	2765.688494	0.321804944	0.035026068	9.187584079	4.01763E-20	2.47131E-19
ACADL	2.196748081	-1.0536898	0.11468221	-9.187909765	4.00549E-20	2.46461E-19
CAPRIN2	596.0504124	-0.594982378	0.064751832	-9.188657111	3.97776E-20	2.44832E-19
ZBTB5	1475.194989	0.305429588	0.033229587	9.191495221	3.87418E-20	2.38532E-19
SLC22A11	9.987592669	-0.88647854	0.096441439	-9.191884181	3.8602E-20	2.37746E-19
EPB41L3	190.536021	1.024519489	0.11145893	9.191901375	3.85958E-20	2.37746E-19
LATS2	3401.930669	0.391574168	0.042592934	9.193406823	3.80592E-20	2.34551E-19
NOX1	3.249579766	-0.696289608	0.075737909	-9.193409435	3.80583E-20	2.34551E-19
CLEC16A	2121.448067	-0.283729042	0.030861929	-9.19349655	3.80275E-20	2.34503E-19
SYT5	17.76287676	-0.778643688	0.08469157	-9.193874784	3.7894E-20	2.33753E-19
ADH4	7.412187555	-0.860310544	0.093569281	-9.194369494	3.772E-20	2.32753E-19
CBLN2	2.311680214	-1.659443794	0.180481584	-9.194532519	3.76629E-20	2.32474E-19
PIGF	226.4670956	-0.338580593	0.036824084	-9.194542229	3.76595E-20	2.32474E-19
CFAP126	96.64968644	-0.354831396	0.038584291	-9.19626575	3.70605E-20	2.28901E-19
GOLGA8T	20.38785254	-0.683395663	0.074307896	-9.196810781	3.68731E-20	2.27815E-19
PNPLA7	411.2958309	-0.449461462	0.048863087	-9.198384425	3.63372E-20	2.24575E-19

TMEM231	67.78155501	-0.505786647	0.054983288	-9.19891598	3.61579E-20	2.23537E-19
NAA30	1219.818446	0.337639599	0.036703089	9.199214793	3.60575E-20	2.22987E-19
LMBRD2	401.7374296	-0.355216774	0.03861363	-9.199258877	3.60427E-20	2.22966E-19
SMIM6	46.74129354	-0.361796193	0.039318576	-9.201660616	3.5246E-20	2.18106E-19
BSND	11.05057701	-1.056860391	0.114846423	-9.202379672	3.50109E-20	2.1672E-19
PSME3IP1	1869.33258	-0.21844818	0.023731746	-9.204892854	3.42012E-20	2.11775E-19
PPP3R1	1933.750834	0.266009936	0.02889576	9.205846805	3.38988E-20	2.09969E-19
SCN7A	4.483839902	-1.185275945	0.128750117	-9.206018409	3.38446E-20	2.097E-19
SHCBP1L	3.013766105	-0.973945438	0.105777823	-9.207463423	3.33922E-20	2.06962E-19
CEND1	10.85149026	-0.598458916	0.064985545	-9.209108258	3.28845E-20	2.0388E-19
NR1D2	1806.822938	0.421007103	0.045710666	9.210259722	3.25337E-20	2.01769E-19
CEACAM5	3.828650883	-0.997166735	0.1082628	-9.210612861	3.24268E-20	2.0117E-19
CNPY2	125.6799556	-0.325860025	0.035378081	-9.210788571	3.23738E-20	2.00904E-19
RANBP9	3173.622284	0.29057616	0.031541239	9.21257923	3.18381E-20	1.97643E-19
RAB40AL	2.267176753	-0.683517899	0.074164964	-9.216183199	3.07864E-20	1.91175E-19
GOLGA8R	245.6513146	-0.66074122	0.071682709	-9.217581579	3.03876E-20	1.88758E-19
AKR7A2	1087.49732	-0.490171575	0.053176661	-9.217795263	3.03272E-20	1.88442E-19
PLEK	15769.41627	0.396304932	0.042978426	9.221020178	2.94287E-20	1.82918E-19
WDR5	979.9402671	-0.260642932	0.028261902	-9.222412905	2.90488E-20	1.80614E-19
COL5A2	31.46522576	-0.650376435	0.070518042	-9.22283745	2.8934E-20	1.79957E-19
NR2F6	254.3331375	-0.418903682	0.045404005	-9.22613944	2.80562E-20	1.74553E-19
DSEL	43.14746904	-0.971554675	0.105287151	-9.227666068	2.76593E-20	1.72138E-19
IMPDH2	6112.463005	-0.333924927	0.036184723	-9.228340049	2.74858E-20	1.71113E-19
ANGPT1	969.4744181	-0.931481632	0.100923075	-9.229620006	2.71594E-20	1.69135E-19
ANTXR1	24.10593331	-0.812821385	0.088063371	-9.229959931	2.70733E-20	1.68652E-19
BOLA1	115.4553941	-0.484033809	0.052440934	-9.230076007	2.7044E-20	1.68523E-19
FAM240C	12.65864304	-0.716776508	0.077648626	-9.231026333	2.68051E-20	1.67088E-19
CLDN16	1.521584169	-1.534672623	0.166217067	-9.232942505	2.63297E-20	1.64177E-19
DPM3	388.3234273	-0.649073953	0.070295046	-9.23356603	2.61769E-20	1.63276E-19
TRAPPC6A	525.7957661	-0.493303272	0.053424866	-9.233589296	2.61712E-20	1.63276E-19
SNX18	2438.297363	0.501888786	0.05435428	9.233657123	2.61546E-20	1.63241E-19
MAP1B	119.6453874	-0.684042651	0.074079173	-9.233940116	2.60856E-20	1.62862E-19
PROS1	106.7625862	-1.067761573	0.115623912	-9.234781572	2.58814E-20	1.61639E-19
TMEM141	125.1008943	-0.441010402	0.04775483	-9.234885734	2.58562E-20	1.61533E-19
AC010197.2	5.449344797	-0.715680204	0.077496995	-9.234941377	2.58427E-20	1.61501E-19
XPNPEP1	3863.052858	0.274244908	0.029695921	9.235103594	2.58036E-20	1.61308E-19
GYS1	1686.655805	-0.346018017	0.037465596	-9.235620177	2.56794E-20	1.60582E-19

SHD	123.0852278	-1.101239421	0.119222121	-9.236871599	2.53809E-20	1.58767E-19
LRRC28	786.6229279	-0.530358283	0.057409971	-9.238086544	2.50945E-20	1.57025E-19
RELB	3273.061977	0.494186762	0.053492537	9.238424406	2.50154E-20	1.5658E-19
POLR3H	488.1605333	-0.269088892	0.029126027	-9.238777707	2.49329E-20	1.56114E-19
NADK2	485.969698	-0.306838394	0.033211728	-9.23885658	2.49145E-20	1.56049E-19
BCL11A	2717.122011	-0.499683822	0.054078404	-9.239988256	2.46524E-20	1.54456E-19
DHRSX	503.2247029	-0.319642946	0.034589771	-9.240967479	2.44278E-20	1.53098E-19
RGS11	19.56397449	-0.661539342	0.071579691	-9.241997703	2.41937E-20	1.5168E-19
TMEM201	857.4564512	-0.326386307	0.035312307	-9.242848595	2.4002E-20	1.50526E-19
ZNF429	287.2500894	-0.458686075	0.049615944	-9.244731461	2.35832E-20	1.47947E-19
EFHD2	5542.845349	0.401091211	0.043384348	9.24506723	2.35093E-20	1.4753E-19
CDHR1	44.08929429	-0.943069553	0.10200395	-9.245421907	2.34314E-20	1.47089E-19
CYB5A	231.4113044	-0.453800222	0.049081992	-9.245757975	2.33579E-20	1.46675E-19
IER3	686.3864836	0.806412583	0.087178875	9.250091601	2.243E-20	1.40893E-19
PSRC1	105.946196	-0.638734147	0.069048538	-9.250509335	2.23425E-20	1.40389E-19
TFE3	2418.928493	0.305675774	0.033040191	9.251634457	2.21086E-20	1.38963E-19
SLC6A6	5269.803255	0.407920407	0.044090843	9.251817012	2.20708E-20	1.3877E-19
BBS5	42.87245466	-0.429966438	0.046463642	-9.253825491	2.16599E-20	1.3623E-19
SSH1	3268.929344	0.328756358	0.035523579	9.254595621	2.15043E-20	1.35295E-19
AFTPH	2875.678973	0.295803524	0.031961536	9.254984711	2.14261E-20	1.34847E-19
LIME1	31.19818825	-0.50928561	0.055025387	-9.255466257	2.13297E-20	1.34283E-19
DUSP11	674.4593265	0.273561258	0.02955615	9.255645924	2.12939E-20	1.34101E-19
CEBPB	2437.151637	0.62665568	0.067683755	9.258583296	2.07163E-20	1.30505E-19
SFXN4	575.1393973	-0.345914395	0.037357351	-9.259607178	2.05186E-20	1.29301E-19
RHOXF2	8.192257183	-0.950651379	0.102645661	-9.261486236	2.01606E-20	1.27087E-19
EXOSC2	987.650923	-0.251100719	0.027107443	-9.26316522	1.9846E-20	1.25144E-19
ACSF2	453.3656792	-0.397195597	0.042876689	-9.263672368	1.97519E-20	1.24591E-19
CXXC4	4.45376539	-1.605218558	0.173280739	-9.263687169	1.97492E-20	1.24591E-19
VSTM4	20.50078221	-0.912968131	0.098550093	-9.264000687	1.96913E-20	1.24288E-19
URAD	18.35026266	-0.761184281	0.082162472	-9.264378969	1.96216E-20	1.23888E-19
MPPED2	51.89798829	-1.253221519	0.135269001	-9.264661594	1.95697E-20	1.236E-19
SEMA3F	388.6695154	0.857736825	0.09258079	9.264738658	1.95556E-20	1.23551E-19
DDX55	608.6698306	-0.242529865	0.026177394	-9.264859036	1.95335E-20	1.23452E-19
GMPPA	542.8699496	-0.364093893	0.039291435	-9.266495113	1.92363E-20	1.21613E-19
METTL2A	342.8257193	-0.256100338	0.027637154	-9.266523433	1.92312E-20	1.21613E-19
KCTD20	4600.340707	0.224196341	0.02419331	9.266873507	1.91682E-20	1.2126E-19
KLLN	107.8943566	-0.48350602	0.052171635	-9.267603456	1.90375E-20	1.20472E-19

ARMC4	11.23508072	-0.964858862	0.10410135	-9.268456793	1.88858E-20	1.19551E-19
RHAG	1959.262895	-1.100533294	0.118739594	-9.268461015	1.88851E-20	1.19551E-19
ALDH1A1	266.9369424	-1.097384058	0.118378742	-9.270110861	1.85953E-20	1.17788E-19
KIF6	30.97550041	-0.846481155	0.091302115	-9.271210795	1.84045E-20	1.16617E-19
TFG	3861.950629	0.288987704	0.031163623	9.273238442	1.80578E-20	1.14458E-19
TNS4	11.28045618	0.869162881	0.093715849	9.274449241	1.78539E-20	1.13202E-19
PPP2R3C	666.1856592	0.240393874	0.02591588	9.27592927	1.76077E-20	1.11677E-19
EML4	2773.412586	0.447679061	0.048260389	9.276325101	1.75425E-20	1.11299E-19
ZNF750	35.59694114	-0.624891326	0.067363981	-9.276341938	1.75397E-20	1.11299E-19
OR10A2	6.087803355	-1.130864825	0.121900845	-9.276923628	1.74442E-20	1.10748E-19
C2CD2	659.9845793	-0.445859565	0.04804796	-9.279469218	1.70325E-20	1.08169E-19
BMT2	484.9507451	0.322044384	0.034704501	9.279614359	1.70093E-20	1.08057E-19
ZNF768	691.250538	-0.331710025	0.035744507	-9.280028055	1.69434E-20	1.07673E-19
PIGL	418.4959895	-0.293000067	0.031572765	-9.280152122	1.69237E-20	1.07583E-19
SLC40A1	1680.997027	-0.813944097	0.087692275	-9.28182208	1.66605E-20	1.05944E-19
UNC13B	321.7286295	-0.909401013	0.097959917	-9.283399152	1.64156E-20	1.04421E-19
LYRM7	531.2658274	-0.381605606	0.041103993	-9.283905959	1.63377E-20	1.03959E-19
GLI2	13.45064074	-1.421023216	0.153053847	-9.284465869	1.6252E-20	1.03447E-19
MOAP1	1459.975817	0.274880492	0.029605979	9.284627561	1.62274E-20	1.03324E-19
COL18A1	515.6694525	-0.639031926	0.068813599	-9.286419172	1.59566E-20	1.01633E-19
MAP2K5	779.5527912	-0.253246685	0.027270473	-9.286479368	1.59476E-20	1.01609E-19
SPIN2A	36.02955037	-0.63571817	0.068454882	-9.286673972	1.59185E-20	1.01456E-19
CLBA1	136.4315692	-0.378229928	0.040724658	-9.287491855	1.57967E-20	1.00713E-19
HAGH	755.4842701	-0.527271981	0.05675707	-9.289978922	1.54319E-20	9.84191E-20
POLR2F	420.0743088	-0.501976787	0.054033261	-9.290144193	1.54079E-20	9.82985E-20
PRDM14	5.846344026	-1.207290054	0.129924345	-9.292254287	1.51055E-20	9.64001E-20
LIPC	49.11078627	-0.70624065	0.075989988	-9.293864474	1.48786E-20	9.49832E-20
EME1	170.0739057	-0.377337385	0.040595944	-9.294952822	1.47271E-20	9.40471E-20
RHOXF1	5.385048783	-0.791289295	0.085128909	-9.295188951	1.46945E-20	9.38692E-20
RLF	4386.692244	0.420907128	0.045269649	9.297777661	1.43412E-20	9.16421E-20
DEPDC4	31.28641058	-0.383789571	0.041277139	-9.297872395	1.43284E-20	9.15904E-20
UCN3	2.007303791	-1.660644239	0.178572745	-9.29953921	1.41056E-20	9.01954E-20
FES	4223.935384	-0.414736235	0.044596361	-9.299777436	1.4074E-20	9.0023E-20
UBA6	3262.562863	0.3609774	0.038809387	9.301290926	1.38751E-20	8.87796E-20
DGCR8	1115.149179	-0.23995162	0.025796307	-9.301781689	1.38112E-20	8.83996E-20
HBP1	3778.240495	0.356874646	0.038365503	9.301967076	1.37871E-20	8.82744E-20
PEX10	232.5578315	-0.301012637	0.032350601	-9.304699983	1.34371E-20	8.60617E-20

PDE4D	2923.783081	0.466530156	0.050137781	9.30496223	1.3404E-20	8.58777E-20
CLCNKB	5.516851914	-0.916654188	0.098465073	-9.309434938	1.28514E-20	8.23644E-20
IDO2	3.354000327	-1.035974259	0.111237271	-9.313193777	1.24045E-20	7.95262E-20
ECI1	591.7166567	-0.343005772	0.036826317	-9.314148051	1.22935E-20	7.88404E-20
CPD	7588.693011	0.478250891	0.051325142	9.318062607	1.18484E-20	7.60107E-20
LBR	6376.725086	0.357975926	0.038414687	9.318725448	1.17746E-20	7.55622E-20
ADARB1	689.3833036	-0.510427909	0.05476444	-9.320425971	1.15874E-20	7.43852E-20
SOGA1	2069.383567	-0.415975618	0.044629647	-9.320611787	1.15671E-20	7.42794E-20
KIF15	456.1068355	-0.488394274	0.052397417	-9.320960845	1.15291E-20	7.40597E-20
B3GALT6	340.1375986	-0.452849693	0.048577457	-9.322219072	1.13932E-20	7.32105E-20
ASAP2	286.5524532	-0.73904473	0.079269803	-9.323155849	1.1293E-20	7.25906E-20
DZIP1L	88.72713115	-0.784332783	0.084122188	-9.323732511	1.12318E-20	7.22208E-20
TEX35	1.630916168	-1.160760494	0.124469889	-9.325632934	1.10323E-20	7.09614E-20
FBXL5	4376.593841	0.306860633	0.032899788	9.327130986	1.08775E-20	6.99888E-20
RNLS	91.87754715	-0.390259575	0.041835571	-9.328415189	1.07466E-20	6.91689E-20
CNIH1	2052.085467	0.32237856	0.034557292	9.328814269	1.07062E-20	6.89317E-20
TRMT5	116.7589097	-0.413483088	0.044318093	-9.329893491	1.05977E-20	6.82558E-20
RHOJ	5.661205625	-1.228964416	0.131720942	-9.330060915	1.0581E-20	6.81705E-20
FBXW9	212.4309144	-0.307837036	0.032990085	-9.331198764	1.0468E-20	6.74648E-20
PTP4A3	353.4117385	-0.669869639	0.071776059	-9.332772602	1.03137E-20	6.64921E-20
ASB16	98.91501175	-0.425832725	0.045626691	-9.332974116	1.02941E-20	6.63876E-20
ZNF639	1748.748919	0.294617077	0.031565439	9.333533414	1.02399E-20	6.60599E-20
MOBP	9.685493027	-0.958001555	0.102629334	-9.334578351	1.01394E-20	6.54331E-20
SLC22A23	498.4301629	-0.813056667	0.087098496	-9.33491056	1.01077E-20	6.52497E-20
RNF215	297.074914	-0.328371832	0.035174429	-9.33552704	1.0049E-20	6.48925E-20
ARHGAP42	90.88011178	-0.91951426	0.098492711	-9.335860964	1.00174E-20	6.47096E-20
SPRTN	669.8370988	0.316851698	0.033935658	9.336836602	9.92553E-21	6.41375E-20
KBTBD2	2687.982332	0.274087879	0.029344574	9.3403257	9.60382E-21	6.20791E-20
GTPBP4	2806.215038	0.298370976	0.031941951	9.341038039	9.53942E-21	6.16832E-20
CHN1	25.13997384	-0.538408755	0.057638667	-9.341103447	9.53353E-21	6.16655E-20
PRR5L	501.8624	-0.65595705	0.070206529	-9.343248505	9.34229E-21	6.04484E-20
MAP7	511.8143703	-0.777115095	0.083168964	-9.343811123	9.29276E-21	6.01479E-20
ERG	1803.63022	-0.636961557	0.068150391	-9.346410948	9.06724E-21	5.87076E-20
UTP14A	484.4174098	-0.25881479	0.027691197	-9.346464395	9.06266E-21	5.86973E-20
HLA-B	42424.8265	0.389118241	0.041618523	9.349640691	8.7946E-21	5.698E-20
S100A1	24.83244841	-0.470123789	0.050282424	-9.34966434	8.79263E-21	5.698E-20
MOCOS	16.50596475	-0.897605191	0.095970961	-9.352883231	8.52904E-21	5.52961E-20

CDC20	585.3373461	-0.479680828	0.051283287	-9.353550747	8.47536E-21	5.49663E-20
SDSL	137.1882046	-0.618458484	0.066115913	-9.3541548	8.42708E-21	5.46713E-20
PGBD4	231.2576635	-0.371072231	0.039668811	-9.354256422	8.41898E-21	5.46368E-20
HACD1	314.2949967	-0.570586596	0.060996907	-9.354352978	8.41129E-21	5.46051E-20
ZNF648	5.781899855	-1.311206728	0.140170514	-9.354369109	8.41001E-21	5.46051E-20
PTCH2	147.7901819	-0.433705031	0.04636282	-9.354586961	8.3927E-21	5.45205E-20
AWAT1	3.881003183	-0.751870252	0.080370014	-9.35510916	8.35134E-21	5.42699E-20
IL6ST	3611.528427	0.545715305	0.05832849	9.355896319	8.28939E-21	5.38851E-20
VIRMA	2493.084759	0.258640619	0.027644638	9.355905328	8.28868E-21	5.38851E-20
PAK5	2.361494239	-1.947794499	0.208116599	-9.359150155	8.03806E-21	5.22861E-20
PPP1R9A	93.648177	-1.275430925	0.136248514	-9.361063012	7.89384E-21	5.13651E-20
AKAP3	9.5795672	-0.603164891	0.064431588	-9.361322746	7.87446E-21	5.1256E-20
CNTNAP3B	5.250708156	-1.149786446	0.12281288	-9.362099835	7.81675E-21	5.08973E-20
TLL2	18.02902044	-0.901362193	0.096266645	-9.363182751	7.73702E-21	5.03949E-20
WIPF2	1775.126519	0.250990091	0.026804833	9.363613154	7.70556E-21	5.02067E-20
YTHDF3	6214.080587	0.333570984	0.035623976	9.363665088	7.70177E-21	5.01987E-20
FAM234B	331.3424189	-0.413978593	0.04420518	-9.364933947	7.60979E-21	4.96157E-20
PTPRR	3.57254876	-1.241744365	0.132582191	-9.365845841	7.54436E-21	4.92055E-20
UFL1	1121.189717	0.304421585	0.032501763	9.366309787	7.51128E-21	4.90061E-20
ANKDD1B	30.2981841	-0.448875647	0.047914898	-9.368185305	7.37903E-21	4.81593E-20
FGF7	26.98944795	-1.212334436	0.129409623	-9.368193863	7.37843E-21	4.81593E-20
MARCHF5	1886.797747	0.320946292	0.034254748	9.369395802	7.29489E-21	4.76419E-20
GMDS	716.4009523	-0.386229227	0.041214715	-9.371148896	7.17472E-21	4.68728E-20
APC2	218.4829885	-0.57488601	0.061331459	-9.373427905	7.02143E-21	4.58866E-20
TRPA1	2.800918673	-1.4912081	0.159074913	-9.374250595	6.96689E-21	4.55454E-20
CAPZA1	6315.483363	0.283808141	0.03027446	9.374507017	6.94998E-21	4.545E-20
TIPARP	3470.577304	0.433594659	0.046252013	9.374611626	6.94309E-21	4.54202E-20
HMGA2	122.882613	-1.250350522	0.13336656	-9.375292579	6.89842E-21	4.5143E-20
CDK19	1339.228227	-0.288536817	0.030773677	-9.37609163	6.84636E-21	4.48173E-20
STK31	17.11003783	-0.808075502	0.086182664	-9.376311449	6.83211E-21	4.4739E-20
C3orf18	56.07325311	-0.501888234	0.053514931	-9.378471112	6.69363E-21	4.38468E-20
NRXN2	656.6860853	-0.736821796	0.078563217	-9.378712104	6.67835E-21	4.37614E-20
TNFAIP8L2	155.962555	-0.574852377	0.061283816	-9.380166137	6.58689E-21	4.31765E-20
IL23R	14.33988543	-1.134135038	0.120898285	-9.380902583	6.54104E-21	4.28904E-20
ABCC9	14.45560844	-1.172654259	0.125001138	-9.381148688	6.52578E-21	4.28047E-20
ARL9	29.46812859	-0.647407079	0.06900384	-9.382189102	6.4617E-21	4.23985E-20
RIN2	299.8936486	0.74169384	0.079048079	9.382819278	6.42318E-21	4.21599E-20

DYTN	66.38539309	-0.958952087	0.102197416	-9.383330043	6.39213E-21	4.19702E-20
MSI1	4.620746311	-0.929712749	0.09905488	-9.38583488	6.24199E-21	4.09982E-20
FAM91A1	5661.12984	0.36883372	0.039289641	9.387556371	6.14084E-21	4.03473E-20
CEP290	657.4055184	-0.385178481	0.041028132	-9.388155438	6.10602E-21	4.0132E-20
AP006621.6	14.26833886	-0.359320678	0.038265831	-9.390118198	5.9933E-21	3.94044E-20
WDHD1	461.1249035	-0.382339601	0.040708492	-9.392133693	5.87969E-21	3.86704E-20
ANXA8L1	11.89767164	1.384610769	0.147365623	9.3957515	5.68108E-21	3.73767E-20
ERICH1	997.4029187	-0.301484923	0.032086945	-9.395875026	5.67442E-21	3.73455E-20
SRPK3	88.30581517	-0.513789181	0.05467713	-9.396784041	5.62562E-21	3.70368E-20
TTLL7	49.02032928	-0.949381163	0.101026168	-9.397378732	5.59393E-21	3.68405E-20
PKD1L1	17.84307968	-0.64460498	0.068578612	-9.399504554	5.48206E-21	3.61159E-20
STRAP	3770.794492	0.307682919	0.032729428	9.400803521	5.4148E-21	3.56848E-20
SPTA1	5090.399854	-1.01802235	0.108282584	-9.40153357	5.37735E-21	3.54499E-20
PCNP	3353.350107	0.312125768	0.033195484	9.402657423	5.32021E-21	3.5085E-20
NAA16	1718.317119	0.308981635	0.032860415	9.40285238	5.31036E-21	3.50319E-20
SLC9A6	1045.840759	0.282645583	0.030053998	9.404591839	5.22325E-21	3.44689E-20
TLR4	1094.447968	0.724680536	0.077055775	9.404623269	5.22169E-21	3.44689E-20
RIC1	2994.173656	0.283557554	0.030148661	9.405311556	5.18763E-21	3.42569E-20
SYNGAP1	1609.456576	-0.392703846	0.041750544	-9.405957552	5.15586E-21	3.40586E-20
HUNK	7.065961305	-1.101601091	0.117114763	-9.406167582	5.14557E-21	3.40021E-20
FAM90A1	19.97793174	-0.67716313	0.071987936	-9.406619641	5.12349E-21	3.38677E-20
COL16A1	18.81601252	-0.724034312	0.076934385	-9.411062578	4.91146E-21	3.2477E-20
STPG1	120.806113	-0.336039422	0.035695243	-9.414123298	4.77047E-21	3.15554E-20
MEX3B	770.7318099	-0.767582527	0.081534209	-9.414238998	4.76522E-21	3.15313E-20
SH3GLB2	1295.405987	-0.365355083	0.038802512	-9.415758527	4.69679E-21	3.1089E-20
ABCC4	1543.696072	-0.585967251	0.062228688	-9.416352328	4.67031E-21	3.09242E-20
LACTB2	55.39528952	-0.481658818	0.051150843	-9.416439458	4.66644E-21	3.0909E-20
PCSK1	12.98104214	-0.936437799	0.099436065	-9.417486539	4.62015E-21	3.06128E-20
KREMEN2	30.40910332	-0.551204995	0.05852477	-9.4183197	4.58364E-21	3.03812E-20
ASCL3	3.587387178	-0.707883216	0.075157072	-9.418717306	4.56632E-21	3.02766E-20
GLTPD2	36.02363854	0.629016526	0.066781609	9.419008241	4.55369E-21	3.02031E-20
KCNJ16	2.860912941	-1.437608192	0.152626604	-9.41911933	4.54887E-21	3.01814E-20
TM9SF2	3959.161004	0.195424174	0.020743621	9.420928858	4.47115E-21	2.96758E-20
SZT2	3839.306068	-0.364662289	0.038703336	-9.421986057	4.42635E-21	2.93884E-20
NR2E1	3.876286512	-1.022427237	0.108511917	-9.422257586	4.41492E-21	2.93224E-20
KIF4B	2.906025018	-0.811044029	0.086072233	-9.42283014	4.3909E-21	2.91728E-20
SIRT3	501.6831822	-0.23812353	0.025266085	-9.424630983	4.31621E-21	2.86863E-20

PGLYRP4	3.926790059	-1.167731976	0.123898928	-9.424875538	4.30616E-21	2.86292E-20
JDP2	1315.956569	0.599824839	0.063640442	9.425214798	4.29226E-21	2.85465E-20
C5AR1	2379.431219	0.896323012	0.095079958	9.427044686	4.21806E-21	2.80626E-20
NOM1	762.5372584	-0.314889426	0.033398517	-9.428245632	4.17005E-21	2.77526E-20
ZFPM1	662.5650649	-0.578817011	0.061382395	-9.429690962	4.11299E-21	2.73822E-20
KCNQ2	9.678294205	-0.920888527	0.097641411	-9.431331609	4.04915E-21	2.69663E-20
GPR63	9.707738161	-1.230242698	0.130438769	-9.431572403	4.03987E-21	2.69137E-20
MMRN2	15.69840949	-0.870540455	0.092295493	-9.432101513	4.01954E-21	2.67873E-20
JCAD	138.1703311	-1.002072218	0.106235328	-9.4325705	4.0016E-21	2.66769E-20
UACA	84.59721439	-0.802678491	0.085074647	-9.43499056	3.91029E-21	2.60771E-20
AASS	71.38465192	-1.092205175	0.115753929	-9.435577552	3.88846E-21	2.59403E-20
CIR1	1358.025785	0.388184337	0.041139151	9.435885953	3.87704E-21	2.58729E-20
GARNL3	53.59621368	-0.678540939	0.071907273	-9.436332549	3.86056E-21	2.57717E-20
WDR82	4781.653592	0.225037229	0.023844846	9.437562555	3.81552E-21	2.54797E-20
NIBAN2	924.5972791	0.731347202	0.077492029	9.43770877	3.8102E-21	2.54529E-20
CNOT1	7385.611271	-0.245811045	0.026037628	-9.440608332	3.70621E-21	2.47667E-20
GZMM	108.1434861	0.526011489	0.055705187	9.442774007	3.63038E-21	2.42683E-20
PIP5K1B	587.8555624	-0.567750762	0.060122439	-9.443242328	3.61419E-21	2.41683E-20
MCCC1	1028.38898	-0.256490903	0.027157955	-9.444411589	3.57407E-21	2.39081E-20
VWA8	928.7239232	-0.350217651	0.037079397	-9.445074118	3.55153E-21	2.37655E-20
SLC9A8	2461.567866	0.355453411	0.037630738	9.445826114	3.52612E-21	2.36035E-20
ME1	78.70250091	0.996138909	0.105449856	9.446564944	3.50133E-21	2.34456E-20
SPOCK3	14.12821944	-1.689364364	0.178815092	-9.447549112	3.46857E-21	2.32342E-20
TIGD5	238.9665883	-0.40088672	0.042432107	-9.447721346	3.46287E-21	2.32039E-20
P4HA3	21.97744081	-0.592826075	0.06274736	-9.447825014	3.45944E-21	2.31889E-20
MYSM1	2757.198084	0.283428599	0.029997982	9.44825543	3.44525E-21	2.31017E-20
DCAF6	1462.97499	-0.346099707	0.036629045	-9.448777864	3.4281E-21	2.29946E-20
PODXL2	248.831493	-0.662445122	0.07010629	-9.449153882	3.41581E-21	2.292E-20
BICC1	27.51609699	-1.176688515	0.124521512	-9.449680594	3.39866E-21	2.28128E-20
PIK3R5	3842.013499	0.540635721	0.057211005	9.449855344	3.39299E-21	2.27825E-20
ZC3H8	285.9126312	-0.316956568	0.03354007	-9.450086704	3.3855E-21	2.274E-20
LCMT2	159.4790856	-0.443796261	0.046961014	-9.450312663	3.3782E-21	2.26988E-20
VPS35L	1408.215957	-0.291607873	0.03084961	-9.452562621	3.30635E-21	2.22236E-20
MESD	631.3545892	-0.331637726	0.035083754	-9.452743412	3.30064E-21	2.21929E-20
IHH	3.957751681	-0.919059694	0.097225987	-9.452819336	3.29825E-21	2.21844E-20
ZNF560	2.915309685	-1.275617862	0.134933922	-9.453648424	3.27222E-21	2.20169E-20
USPL1	1698.789722	0.312600187	0.033061721	9.455048933	3.22871E-21	2.17316E-20

HDGFL2	759.8833317	-0.282555	0.029881883	-9.455729258	3.20778E-21	2.15982E-20
WSB2	709.447341	0.334218027	0.035344806	9.4559303	3.20162E-21	2.15641E-20
SLC2A3	15137.00743	0.623307244	0.065904567	9.457724575	3.14717E-21	2.12047E-20
KRCC1	243.1446236	-0.467225226	0.049396325	-9.458704153	3.11783E-21	2.10142E-20
DPY19L4	496.4466752	-0.35712517	0.037736587	-9.463631919	2.97429E-21	2.00537E-20
CHST4	5.212985007	-1.169119409	0.123518547	-9.465132452	2.93189E-21	1.97746E-20
FXR1	5701.05082	0.264559176	0.027949553	9.465595891	2.91892E-21	1.96939E-20
ASXL2	1734.1822	-0.376430663	0.039768062	-9.465652691	2.91733E-21	1.969E-20
ALAD	1872.552848	-0.452188826	0.047770361	-9.465886718	2.91081E-21	1.96527E-20
TMEM178A	12.61706308	-0.727412245	0.076842437	-9.466282918	2.89979E-21	1.95851E-20
GOLGA8S	17.74392959	-0.883417664	0.093316895	-9.466856636	2.88391E-21	1.94846E-20
PIN4	152.5998416	-0.566453108	0.059833575	-9.467144628	2.87598E-21	1.94377E-20
TSPYL5	126.889414	-1.018510924	0.107570524	-9.4683087	2.84411E-21	1.9229E-20
MMD	436.0028207	0.421330793	0.044496478	9.468857178	2.82922E-21	1.91349E-20
CTTNBP2	3.519261877	-1.435909374	0.151611299	-9.470991819	2.77199E-21	1.87543E-20
PAIP1	1449.050024	0.249455215	0.02633844	9.471146205	2.7679E-21	1.87331E-20
SUSD2	58.96530487	-0.588234295	0.062101548	-9.472135774	2.74179E-21	1.85628E-20
TRMT2B	818.1959346	-0.227875668	0.024054565	-9.473281787	2.71187E-21	1.83666E-20
GLTP	914.2696228	0.369866005	0.039041791	9.473592259	2.70382E-21	1.83184E-20
FAM3A	761.222839	-0.233013526	0.024595258	-9.473920773	2.69532E-21	1.82672E-20
NAA60	1794.861376	0.270495608	0.028545976	9.475787823	2.64755E-21	1.79496E-20
SMPDL3B	94.38504435	-0.580879588	0.061291143	-9.477382165	2.60742E-21	1.76837E-20
ACOT6	3.2876767	-0.827334624	0.087292051	-9.477777361	2.59757E-21	1.76229E-20
ACSM5	4.134127513	-1.072139475	0.113074634	-9.481697488	2.5018E-21	1.69791E-20
PPIL1	180.1104098	-0.447072558	0.047148829	-9.48215622	2.49082E-21	1.69104E-20
LRRC4C	4.076280687	-1.519762049	0.160261973	-9.482986038	2.47109E-21	1.67823E-20
ATP13A5	1.735262404	-1.266094077	0.133510755	-9.483086796	2.4687E-21	1.67719E-20
AC010255.2	3.537197524	-0.930747104	0.098145404	-9.483348811	2.46251E-21	1.67356E-20
ATP1A3	13.81688074	-0.720992364	0.075986471	-9.488430639	2.34539E-21	1.59452E-20
RPUSD4	683.5194424	-0.261912163	0.027591143	-9.492617235	2.25305E-21	1.53228E-20
SATB2	82.79884496	-0.985469532	0.10381371	-9.492672373	2.25186E-21	1.532E-20
NADSYN1	4329.954625	-0.267042738	0.028131421	-9.492685713	2.25157E-21	1.532E-20
C1QTNF6	148.3756873	-0.426744126	0.044952601	-9.493202048	2.24044E-21	1.52529E-20
ZNF862	339.1771946	-0.412843696	0.043486979	-9.493501355	2.23402E-21	1.52145E-20
BOLA3	80.7078099	-0.519515832	0.05472077	-9.493942293	2.22458E-21	1.51555E-20
UBE2G2	2770.188387	-0.242775456	0.025555803	-9.499817339	2.10259E-21	1.43294E-20
CD53	6234.929679	0.358456155	0.03771312	9.504812957	2.00408E-21	1.36628E-20

HTR1D	2.908802893	-1.136223722	0.119534394	-9.505412525	1.99257E-21	1.3589E-20
FAM151A	28.91207299	-0.689612179	0.072547936	-9.505607163	1.98884E-21	1.35684E-20
PREX2	55.3989223	-1.3695328	0.144053446	-9.507115853	1.96022E-21	1.33777E-20
SULT4A1	18.65220156	-1.420524058	0.149393272	-9.508621373	1.93206E-21	1.31902E-20
KLHL23	211.8124188	-0.55882391	0.058768636	-9.508879979	1.92726E-21	1.3162E-20
IGLL1	1538.903383	-1.151541225	0.121069576	-9.511400487	1.88113E-21	1.28514E-20
ZNF347	179.8977506	-0.530559169	0.055781192	-9.511434796	1.88051E-21	1.28514E-20
TMEM87A	1723.917368	0.29392703	0.030902485	9.511436711	1.88047E-21	1.28514E-20
LYG1	46.78396324	-0.44021798	0.046267689	-9.514587585	1.82435E-21	1.24766E-20
TMPRSS9	274.1032995	-0.539632207	0.056711556	-9.515383609	1.81044E-21	1.23858E-20
PIM3	6164.669047	0.528331296	0.055519703	9.516104508	1.79793E-21	1.23045E-20
ACYP2	296.6322808	-0.343116608	0.036054048	-9.516729155	1.78716E-21	1.2235E-20
AKR1A1	1797.228858	-0.416594235	0.043770898	-9.517607618	1.77212E-21	1.21363E-20
PIGZ	141.0619006	-0.397283382	0.041738006	-9.518504118	1.7569E-21	1.20363E-20
CDS2	5801.624579	0.32433747	0.034069755	9.519806482	1.73502E-21	1.18906E-20
CCDC169	49.94852955	-0.653146465	0.068594738	-9.521815907	1.70179E-21	1.16669E-20
SERPINF2	65.3327579	-0.485510411	0.050985365	-9.522544571	1.6899E-21	1.15895E-20
KIF27	271.5128182	-0.322374972	0.033851415	-9.523234861	1.6787E-21	1.15167E-20
UNC80	18.29707618	-0.802978319	0.084301545	-9.525072428	1.64927E-21	1.13188E-20
LDLRAD2	137.7488283	-0.717462371	0.075288482	-9.529510381	1.58027E-21	1.0849E-20
RNF167	1776.838388	-0.335496107	0.035204922	-9.5298069	1.57576E-21	1.08219E-20
PHGR1	32.1261851	-0.750125001	0.07870491	-9.530853896	1.55995E-21	1.0717E-20
CCDC150	123.1138442	-0.422673625	0.044346621	-9.53113493	1.55573E-21	1.06918E-20
ZNF566	188.0082662	-0.368076433	0.038614857	-9.53199004	1.54297E-21	1.06078E-20
PRMT3	529.5452979	-0.301311444	0.031608695	-9.532549297	1.53467E-21	1.05545E-20
GNE	1488.99451	0.243141884	0.025501923	9.5342568	1.50963E-21	1.03859E-20
RCHY1	910.691181	0.292422188	0.030669381	9.534662237	1.50374E-21	1.03491E-20
SMIM1	127.3865523	-0.797854592	0.083671207	-9.535593246	1.49031E-21	1.02602E-20
GABRG1	2.678010327	-2.452641058	0.257197718	-9.536014069	1.48428E-21	1.02223E-20
TRPC1	47.60602927	-0.898609412	0.094231758	-9.536163069	1.48215E-21	1.02112E-20
CCNI	18472.75714	0.284240576	0.029798066	9.538893421	1.44364E-21	9.94946E-21
SLC6A17	4.844425931	-0.898224066	0.094163639	-9.538969304	1.44259E-21	9.94568E-21
STYXL1	491.632585	-0.413853381	0.043378946	-9.540420371	1.42255E-21	9.81095E-21
OTUD1	2112.053973	0.468927779	0.049149584	9.540829078	1.41695E-21	9.7758E-21
C19orf73	15.38854911	-0.590557752	0.061890928	-9.541911446	1.40224E-21	9.6777E-21
BAHCC1	6603.789696	-0.679772852	0.071222801	-9.544315082	1.3701E-21	9.45924E-21
HSP90B1	12056.52574	0.39354261	0.041229912	9.545075172	1.36009E-21	9.39345E-21

NVL	744.9654801	-0.257606799	0.026986631	-9.545719177	1.35166E-21	9.33856E-21
GLDC	61.51174201	-0.640617583	0.067110213	-9.545753926	1.35121E-21	9.33856E-21
SVOPL	58.5692713	-0.813318479	0.085177449	-9.548518909	1.31564E-21	9.09606E-21
RICTOR	7686.444732	0.490526512	0.051368142	9.54923611	1.30656E-21	9.03651E-21
LIMA1	393.6834864	-0.422243332	0.044215949	-9.54957078	1.30235E-21	9.01055E-21
THOC5	1343.900631	-0.198225642	0.02075536	-9.55057573	1.28978E-21	8.92672E-21
CCDC27	12.55578774	-0.740254065	0.077507896	-9.550692258	1.28833E-21	8.91984E-21
ITCH	4946.313074	0.334798978	0.035046466	9.553002663	1.25991E-21	8.72617E-21
A2ML1	28.04134563	-0.724489705	0.075791573	-9.558974388	1.18929E-21	8.24002E-21
METTL2B	428.1291887	-0.25763858	0.026950376	-9.559739855	1.18053E-21	8.1822E-21
KRT72	22.45246812	-0.970931527	0.101550149	-9.561103944	1.16507E-21	8.07792E-21
SLC26A11	392.2907264	0.739813714	0.077373624	9.561575042	1.15978E-21	8.04407E-21
MIGA2	1148.580617	-0.337136124	0.035258989	-9.561707165	1.1583E-21	8.03666E-21
SLC34A3	20.93713527	-0.633929421	0.06628313	-9.563963282	1.13332E-21	7.8661E-21
GLOD5	23.61816018	-0.91071254	0.095215977	-9.564702965	1.12524E-21	7.81282E-21
HPDL	95.52276804	-0.383568009	0.040097661	-9.565844906	1.11289E-21	7.72979E-21
TRIM35	558.6102218	-0.360562137	0.037692028	-9.566005184	1.11116E-21	7.72056E-21
LMLN	127.288892	-0.458658909	0.047944795	-9.566396313	1.10697E-21	7.69415E-21
ALDH9A1	1181.108812	0.276038187	0.028853232	9.566976194	1.10078E-21	7.65385E-21
CEP43	431.5664457	-0.381137738	0.039834926	-9.567928754	1.09069E-21	7.58636E-21
TNIP1	6567.300351	0.383748877	0.040106905	9.568149844	1.08836E-21	7.57285E-21
USP17L2	1.704961516	-1.313644143	0.137292337	-9.568226273	1.08755E-21	7.56995E-21
ANKEF1	22.01371366	-0.827079632	0.086436842	-9.56860075	1.08362E-21	7.54527E-21
MTMR14	3964.982778	0.286883351	0.029980019	9.569151843	1.07786E-21	7.50783E-21
NRG2	6.265335571	-0.852462269	0.089071034	-9.570589147	1.06298E-21	7.40682E-21
LRBA	5370.872663	-0.480283342	0.05017475	-9.572211889	1.04643E-21	7.29405E-21
ZNF761	396.0742549	-0.285994638	0.02987042	-9.574510134	1.02341E-21	7.13618E-21
OR2AG1	4.800098996	-1.193523684	0.124647125	-9.575220315	1.0164E-21	7.08983E-21
ZNF891	402.4075892	-0.478921127	0.050016716	-9.575221301	1.01639E-21	7.08983E-21
LRRCC1	202.4418515	-0.622288695	0.064984163	-9.576005381	1.00871E-21	7.04119E-21
PKHD1	2.973914815	-1.359513406	0.141954691	-9.577093886	9.98139E-22	6.96988E-21
CCDC106	163.898521	-0.476921946	0.049783564	-9.57990775	9.71317E-22	6.785E-21
TMEM243	495.9995624	0.417200802	0.043548745	9.58008779	9.69625E-22	6.7756E-21
TUBA8	15.73323149	-0.61818422	0.064520972	-9.581136178	9.59832E-22	6.70957E-21
RASSF3	1276.511183	0.382224271	0.039883753	9.583457892	9.38491E-22	6.56273E-21
SPTY2D1	2505.764496	0.406728075	0.042434952	9.58474227	9.26888E-22	6.48391E-21
SEL1L	2881.608821	0.30529769	0.031844778	9.587056697	9.06336E-22	6.34241E-21

GAB4	4.745841197	-1.572581875	0.164002502	-9.588767584	8.91434E-22	6.24036E-21
FASLG	10.88281252	0.814978765	0.084985809	9.589586512	8.84387E-22	6.19324E-21
LIMS2	61.70312807	-0.607306941	0.063322772	-9.590656335	8.75264E-22	6.13155E-21
MAVS	4822.709301	-0.357284099	0.03725172	-9.591076591	8.71706E-22	6.10881E-21
TNKS2	4647.943476	0.319449827	0.033299822	9.593139221	8.54449E-22	5.99002E-21
NEFH	61.25379825	-0.676763196	0.070545816	-9.593243566	8.53585E-22	5.98611E-21
TAGAP	2773.751464	0.451669485	0.047076701	9.594331696	8.44627E-22	5.92541E-21
GPR65	175.6330916	0.623051455	0.064924455	9.596560427	8.26568E-22	5.80079E-21
ZSCAN29	836.5228067	-0.290282248	0.030235834	-9.600603187	7.94779E-22	5.5797E-21
PNPLA8	3106.649144	0.486334803	0.050642846	9.603228112	7.7479E-22	5.44132E-21
FBXL16	38.54815375	-0.558287182	0.058135101	-9.603271867	7.74461E-22	5.44097E-21
ZFP69	104.6266897	-0.413448587	0.043048656	-9.604216009	7.67397E-22	5.39327E-21
VWC2	1.784923976	-1.286401724	0.133906415	-9.606722135	7.48953E-22	5.26554E-21
PCCB	1157.248349	-0.301102003	0.031342393	-9.606860709	7.47946E-22	5.26035E-21
KLF13	10403.06961	0.355901223	0.037045121	9.607236104	7.45225E-22	5.24309E-21
LAS1L	1753.811059	-0.203724198	0.021204362	-9.607655122	7.42199E-22	5.22368E-21
PAICS	2635.471897	-0.342601147	0.035652763	-9.609385617	7.29832E-22	5.13849E-21
AKIRIN2	3489.006219	0.30400319	0.031633635	9.610124957	7.2461E-22	5.10356E-21
GPCPD1	7816.858468	0.417926022	0.043486812	9.610408278	7.22619E-22	5.09137E-21
HSD17B6	8.392595304	-0.611271734	0.063604108	-9.610570091	7.21484E-22	5.0852E-21
FBXL19	992.4059512	-0.395900217	0.041185526	-9.612605516	7.0736E-22	4.98745E-21
SLC7A14	3.410856434	-0.939781224	0.09775623	-9.613517486	7.0112E-22	4.94523E-21
FAHD2A	491.3450406	-0.257810388	0.026816529	-9.613861089	6.98784E-22	4.93053E-21
OSBPL8	5191.879346	0.320110729	0.033289532	9.615957527	6.84693E-22	4.83285E-21
GTF2I	1462.931319	-0.281974734	0.0293233	-9.616064115	6.83984E-22	4.82959E-21
DCC	2.10102666	-1.385667629	0.144069177	-9.618071408	6.7077E-22	4.73799E-21
HMCN2	8.588812327	-0.804176076	0.083610913	-9.618075491	6.70743E-22	4.73799E-21
CPSF4	717.1084714	-0.208971888	0.021725472	-9.618749978	6.6636E-22	4.71024E-21
BLOC1S6	2500.913747	0.285179524	0.029647158	9.6191185	6.63977E-22	4.6951E-21
LAMA3	79.39959381	-0.896965742	0.093247859	-9.619156381	6.63733E-22	4.69506E-21
ADCY6	512.2515363	-0.713439758	0.074161961	-9.620022828	6.58166E-22	4.65737E-21
PCDHA9	3.083477296	-1.012144891	0.105200567	-9.621097313	6.51326E-22	4.61063E-21
CRISPLD2	584.2022607	0.76912967	0.079936443	9.621765066	6.47111E-22	4.58245E-21
TNFAIP3	14793.27765	0.575853435	0.059843102	9.622720277	6.41128E-22	4.54173E-21
MRPS31	295.0560289	-0.307319011	0.03193547	-9.62312466	6.38611E-22	4.52554E-21
CYP4X1	3.039780723	-1.203169117	0.125017187	-9.62402965	6.33015E-22	4.48751E-21
PCDHGA10	70.33745033	-0.815887296	0.084775328	-9.624112497	6.32506E-22	4.48552E-21

FBXO33	1505.569402	0.329073169	0.034190206	9.624778776	6.2842E-22	4.45816E-21
AARS1	1238.903739	-0.437699831	0.045468986	-9.626338045	6.1896E-22	4.39264E-21
TAL2	2.278034537	-1.005600155	0.104449187	-9.627649467	6.11114E-22	4.33853E-21
NPIPB2	33.90290225	-0.522296881	0.054249526	-9.627676423	6.10953E-22	4.33853E-21
STK32C	280.2056831	-0.489752848	0.050868372	-9.62784594	6.09946E-22	4.33339E-21
ZNF517	206.4442193	-0.319267291	0.033160375	-9.627975844	6.09176E-22	4.32949E-21
DCXR	756.3969745	-0.532474395	0.05530491	-9.627976847	6.0917E-22	4.32949E-21
FLCN	917.4510583	-0.450510559	0.046789662	-9.628420946	6.06543E-22	4.31391E-21
TMEM97	307.4732045	-0.420679157	0.043687629	-9.629251357	6.01662E-22	4.28075E-21
COL22A1	2.074941808	-1.6700717	0.173413317	-9.630585062	5.93904E-22	4.22708E-21
FARSB	990.1155181	-0.278118623	0.028876395	-9.63134854	5.89507E-22	4.19731E-21
ZNF462	70.65760968	-1.035260023	0.107485581	-9.631617677	5.87965E-22	4.18786E-21
SLC6A3	3.352290056	-1.095463135	0.113733773	-9.631819171	5.86813E-22	4.18117E-21
RBM18	914.0734434	0.274596213	0.028505358	9.633143866	5.79294E-22	4.12911E-21
PDE7A	3206.532328	-0.434893613	0.045139317	-9.634474875	5.71836E-22	4.07743E-21
EIF4EBP1	859.8012093	-0.701246282	0.072783029	-9.63474993	5.70307E-22	4.06801E-21
ZFAND3	3095.468616	0.30582031	0.031741184	9.634811106	5.69967E-22	4.06707E-21
PRELP	4.460423393	-0.840836292	0.087255607	-9.636472856	5.60819E-22	4.00325E-21
NEK1	493.0829415	-0.364786428	0.037847374	-9.638355003	5.50633E-22	3.93197E-21
TBCEL-TECTA	6.004234957	-0.918763442	0.095319855	-9.638741495	5.48564E-22	3.91863E-21
AGBL2	64.80770408	-0.449010854	0.046574327	-9.640737354	5.38001E-22	3.84458E-21
ARHGAP6	84.42698988	-0.940047885	0.097496872	-9.641826068	5.32325E-22	3.8054E-21
GLRA1	4.612769435	-0.831474418	0.086234355	-9.642032133	5.31257E-22	3.79916E-21
ASIP	12.41338921	-0.590157787	0.061202284	-9.642741206	5.27599E-22	3.77438E-21
PLCXD2	402.783656	0.624137919	0.064721332	9.643465298	5.23889E-22	3.74921E-21
FMNL1	11858.1242	0.373201256	0.038699272	9.643624674	5.23076E-22	3.74476E-21
CHSY3	15.56243494	-1.301122331	0.134914772	-9.644031627	5.21006E-22	3.7313E-21
PSORS1C1	1.955928699	-1.041558439	0.107934174	-9.64994125	4.91839E-22	3.52371E-21
AXIN2	39.18306204	0.79882895	0.082773258	9.650809602	4.87692E-22	3.49527E-21
DDX20	985.9545917	0.264464581	0.02739822	9.652619152	4.7916E-22	3.43538E-21
CACNG8	11.8690293	-0.821699558	0.085120622	-9.65335468	4.75734E-22	3.41207E-21
MAP2	10.24108496	-1.092871228	0.113200485	-9.654298075	4.71376E-22	3.38206E-21
GAL3ST1	4.445080704	-0.803197503	0.083188771	-9.655119205	4.67615E-22	3.3563E-21
C14orf119	271.0155536	-0.440122982	0.045579237	-9.656216461	4.62636E-22	3.32178E-21
CCDC175	18.86991138	-1.367356183	0.141582734	-9.657647836	4.56218E-22	3.2769E-21
POGLUT2	92.00425108	-0.942332678	0.097573076	-9.657712065	4.55933E-22	3.27605E-21
OR2A5	2.517133081	-1.025270679	0.106156062	-9.658145338	4.54009E-22	3.26343E-21

ZMYND15	42.57071264	0.830257301	0.085962825	9.658329629	4.53193E-22	3.25876E-21
PREB	653.0106891	-0.433255504	0.044836013	-9.663114042	4.32513E-22	3.1112E-21
TSR1	947.3754397	-0.236933128	0.024518404	-9.663480873	4.30966E-22	3.10121E-21
ZC4H2	145.8711931	-0.504345653	0.052183707	-9.664810741	4.25405E-22	3.06233E-21
SERPINB2	733.5141619	1.117911242	0.115642938	9.666921803	4.16724E-22	3.00093E-21
VCPKMT	619.3094826	0.299357663	0.030966958	9.667002618	4.16395E-22	2.99967E-21
TMEM167A	2113.275085	0.292714983	0.030278632	9.667378139	4.1487E-22	2.98978E-21
CLDN15	565.4831304	-0.376908505	0.038985447	-9.667928241	4.12647E-22	2.97486E-21
LEPR	432.0275665	-0.658378938	0.068097995	-9.668110417	4.11913E-22	2.97066E-21
PAIP2B	83.28024379	-0.727786672	0.075259273	-9.670391984	4.02832E-22	2.90624E-21
DEFB131B	5.064279988	-0.685158553	0.070849064	-9.670679017	4.01704E-22	2.89917E-21
PPFIA4	204.9158494	-0.727828374	0.075261086	-9.670713147	4.0157E-22	2.89917E-21
ACAP1	3609.020018	-0.326933548	0.033806169	-9.670825026	4.01131E-22	2.89717E-21
RXYLT1	155.3840482	-0.304098334	0.031444024	-9.671100967	4.00051E-22	2.89044E-21
DCP1A	4794.932371	0.43257849	0.044728878	9.67112319	3.99964E-22	2.89044E-21
CBX5	4667.149215	-0.337174461	0.034851533	-9.674594794	3.8662E-22	2.79546E-21
SOX11	41.66998715	-2.019094985	0.208689225	-9.675128108	3.8461E-22	2.78195E-21
MPLKIP	413.5639872	-0.312699673	0.032317202	-9.67595118	3.81527E-22	2.76068E-21
FAM114A2	342.8500076	-0.344286349	0.035579679	-9.676488413	3.79528E-22	2.74723E-21
PEMT	216.5316432	-0.544494896	0.056267384	-9.676918592	3.77935E-22	2.73671E-21
PPP1R21	861.8765072	-0.352540069	0.036430205	-9.677136618	3.7713E-22	2.73189E-21
FASTKD5	1774.280655	0.312423696	0.032280485	9.678407887	3.72471E-22	2.69914E-21
AURKB	398.5264009	-0.496541353	0.05130294	-9.678613983	3.71721E-22	2.6947E-21
PLGLB1	10.5753298	-0.631128532	0.06520423	-9.679257476	3.69389E-22	2.67879E-21
ITIH5	10.55051741	-0.90358747	0.093344511	-9.680135003	3.66232E-22	2.65688E-21
ALPP	3.731689176	-0.955325079	0.098667212	-9.682295243	3.58574E-22	2.60229E-21
CLDN18	14.58729978	-1.125558977	0.116247679	-9.682421077	3.58133E-22	2.60005E-21
VWA2	11.91219394	-0.993499941	0.102601402	-9.683103013	3.55751E-22	2.58372E-21
ADAMTS12	7.996498967	-1.199504849	0.123868682	-9.683681389	3.53744E-22	2.57009E-21
STK38L	1209.42499	0.323572163	0.033409816	9.684942989	3.49403E-22	2.5395E-21
PACS2	1715.544848	-0.308829157	0.031879652	-9.687343959	3.41289E-22	2.48144E-21
LAPTM5	23479.92792	0.452820011	0.046726851	9.690788019	3.29973E-22	2.40006E-21
PALMD	11.63490768	-0.957544492	0.098783896	-9.693325802	3.21873E-22	2.34202E-21
NAT10	2475.122378	-0.292703605	0.030186386	-9.696543506	3.11886E-22	2.27019E-21
ARHGEF37	156.0302346	-0.867403133	0.089449882	-9.697085321	3.10234E-22	2.25901E-21
CEP162	242.1906496	-0.415538538	0.042849936	-9.697530018	3.08886E-22	2.25002E-21
ZNF106	5950.42671	0.299737134	0.030904766	9.698734912	3.0526E-22	2.22444E-21

ADAMTS18	48.88370794	-1.515845642	0.156261596	-9.700692161	2.9946E-22	2.18299E-21
CTU2	225.342275	-0.323182988	0.033311876	-9.701734758	2.96415E-22	2.1616E-21
TPM1	422.7416963	-0.745189799	0.076809502	-9.701791881	2.96249E-22	2.1612E-21
CMC1	249.9639744	-0.436330361	0.044960075	-9.704840504	2.87526E-22	2.09834E-21
PSMG3	481.2108195	-0.362854751	0.037379246	-9.707385378	2.80438E-22	2.04738E-21
TRIM16L	85.86632956	-0.402256742	0.041438173	-9.707395699	2.8041E-22	2.04738E-21
LRRC8C	1744.643128	0.425460467	0.043823489	9.708502783	2.77382E-22	2.02657E-21
PCDHGB6	48.67474404	-0.872645544	0.089872163	-9.709853586	2.7373E-22	2.00064E-21
TMEM175	717.1888508	-0.327657318	0.033741157	-9.710909328	2.70909E-22	1.98077E-21
AFF4	9977.850992	0.402303772	0.04142737	9.711062385	2.70503E-22	1.97853E-21
DCTN4	6510.998533	0.327766942	0.033738177	9.715016235	2.60208E-22	1.90394E-21
NLK	1162.930987	-0.424571331	0.043700782	-9.715417167	2.59186E-22	1.89717E-21
SLC22A5	247.2868945	-0.523975714	0.053931916	-9.715503387	2.58966E-22	1.89628E-21
CPSF2	1193.921982	-0.339970746	0.034991869	-9.715707098	2.58449E-22	1.8932E-21
CCDC86	204.1110938	-0.443714331	0.045665816	-9.716553169	2.56311E-22	1.87824E-21
THTPA	120.3352908	-0.351366577	0.036159253	-9.717196763	2.54697E-22	1.86711E-21
KCNAB3	130.9352592	-0.403022242	0.041474319	-9.717392685	2.54207E-22	1.86422E-21
CXADR	73.42417121	-0.963386084	0.099127689	-9.718637587	2.51119E-22	1.84226E-21
RTEL1	67.61039892	-0.454423214	0.046751223	-9.720028347	2.47713E-22	1.81795E-21
NOTUM	13.89780401	-0.748573809	0.077005052	-9.721099909	2.45119E-22	1.79959E-21
CDR2	729.2265315	0.414579818	0.042644346	9.721800434	2.43438E-22	1.78792E-21
EPS8L3	7.54147089	-1.076051299	0.110684175	-9.721817004	2.43399E-22	1.78792E-21
PSMC2	1974.394037	0.213841115	0.021989533	9.724677531	2.36654E-22	1.7394E-21
BICDL1	307.8509141	0.701858873	0.072172344	9.724762071	2.36458E-22	1.73861E-21
SLC35B3	200.8910854	-0.371931647	0.038242424	-9.725629489	2.34451E-22	1.7245E-21
SERPINA1	2845.443755	0.988817118	0.101659872	9.726720123	2.31951E-22	1.70676E-21
RHBG	4.908255572	-0.846270449	0.08698023	-9.729457429	2.25794E-22	1.66208E-21
ROCK2	1187.450997	-0.416044638	0.042752663	-9.731432071	2.21453E-22	1.63074E-21
CFAP46	44.64958064	-1.013264639	0.104113481	-9.732309676	2.1955E-22	1.61733E-21
DHX40	1675.607837	0.26463558	0.027190999	9.732469739	2.19205E-22	1.6154E-21
ZNF568	118.0439124	-0.463563385	0.047629659	-9.732662212	2.18791E-22	1.61295E-21
SCN2A	17.22677879	-1.250266751	0.12843402	-9.734700734	2.14448E-22	1.58153E-21
CSNK1G3	1025.948287	0.296217751	0.030426376	9.73555807	2.12647E-22	1.56884E-21
PHLDA1	912.8091809	0.895091566	0.091931933	9.736459796	2.10769E-22	1.55558E-21
MACROH2A2	201.4072644	-0.818710206	0.084086192	-9.736559484	2.10563E-22	1.55464E-21
DNAI3	4.088970661	-1.239825184	0.127336634	-9.736594618	2.1049E-22	1.55464E-21
LRRC49	13.67191688	-1.022476267	0.105004796	-9.737424475	2.08779E-22	1.54263E-21

IGFN1	5.95919424	1.35076808	0.138717213	9.737566485	2.08487E-22	1.54106E-21
SBDS	2300.148959	0.380779096	0.039103963	9.737608814	2.084E-22	1.541E-21
GP1BA	364.1401816	-0.609513183	0.062576634	-9.740267924	2.03018E-22	1.50177E-21
FANCE	201.6499158	-0.460958112	0.047313438	-9.742646686	1.98321E-22	1.46757E-21
CHAF1A	1589.493584	-0.293095089	0.030083633	-9.742676043	1.98263E-22	1.46757E-21
METRNL	1392.95102	0.580408427	0.059572316	9.742921951	1.97784E-22	1.46471E-21
SUSD3	267.3612461	-0.651949827	0.066877751	-9.748381412	1.87433E-22	1.38858E-21
BLOC1S2	982.2772368	0.366176029	0.037556228	9.750074711	1.84333E-22	1.36613E-21
NEDD9	1198.61841	0.491393381	0.050398844	9.750092314	1.84301E-22	1.36613E-21
UHMK1	5207.030485	0.292737664	0.030021404	9.750965219	1.82723E-22	1.35522E-21
RNF7	1601.253617	0.345650708	0.035444729	9.751822545	1.81186E-22	1.34433E-21
WDR45B	3321.743191	0.348577181	0.035744016	9.752042875	1.80793E-22	1.34193E-21
CCDC146	143.5359522	-0.414786399	0.042525246	-9.753885918	1.77539E-22	1.31828E-21
ZSCAN5B	1.80899618	-0.848646659	0.087003814	-9.754131707	1.7711E-22	1.31559E-21
MROH8	95.47339261	-0.350575114	0.035941114	-9.754152825	1.77073E-22	1.31559E-21
MAGIX	89.12432743	-0.441182956	0.04522321	-9.755675267	1.74436E-22	1.29671E-21
CD3E	486.8048816	0.735149653	0.07534476	9.757143751	1.71929E-22	1.27856E-21
HIC1	752.1699016	0.651807515	0.066801103	9.757436428	1.71434E-22	1.27536E-21
CHIC2	906.1066158	0.274465194	0.028125982	9.75842184	1.69777E-22	1.26351E-21
AMH	71.01483515	-0.708283161	0.072569901	-9.760012831	1.67134E-22	1.24432E-21
FANCC	265.7595607	-0.434510423	0.044518268	-9.760272351	1.66707E-22	1.24161E-21
CRTC3	1907.687308	0.370005171	0.037904243	9.761576699	1.64576E-22	1.22621E-21
CNTRL	4157.441116	-0.353060012	0.036166028	-9.76220028	1.63567E-22	1.21916E-21
MACROD1	210.3301946	-0.458055614	0.046920509	-9.762375215	1.63285E-22	1.21752E-21
NDUFA3	793.9978189	-0.776905467	0.079572482	-9.763494233	1.61493E-22	1.20461E-21
JAM2	18.44818424	-0.628508934	0.064366037	-9.764605051	1.59733E-22	1.19194E-21
CCT3	2929.236359	-0.368245533	0.037709821	-9.765242106	1.58732E-22	1.18492E-21
SYAP1	2104.247664	0.377464776	0.038649782	9.76628486	1.57108E-22	1.17324E-21
RASGRP2	5485.090226	-0.348838348	0.035716112	-9.766974165	1.56043E-22	1.16574E-21
LZTS3	365.7438903	0.702690507	0.071940766	9.767626199	1.55042E-22	1.1587E-21
ARMH4	45.28393133	-0.968277433	0.099126745	-9.768074545	1.54358E-22	1.15403E-21
CXXC1	1773.423436	-0.276124311	0.028267959	-9.768102269	1.54315E-22	1.15403E-21
SERHL2	173.6530039	-0.380848828	0.038986392	-9.768763233	1.53312E-22	1.14709E-21
VIPAS39	609.6902768	-0.222401949	0.022762652	-9.770476261	1.50742E-22	1.12829E-21
SNAPIN	310.821858	-0.422749667	0.043253295	-9.773814146	1.45855E-22	1.09213E-21
IFT74	342.8870997	-0.321607375	0.032900716	-9.775087442	1.44033E-22	1.0789E-21
ENTPD4	3284.715962	0.381036618	0.038977217	9.775880633	1.42909E-22	1.07089E-21

TDP2	761.7617619	0.226060916	0.023121573	9.777056061	1.4126E-22	1.05893E-21
GCM2	3.847853837	-0.923300171	0.094434865	-9.777111183	1.41183E-22	1.05876E-21
VASH2	74.09839648	-0.798122967	0.081627366	-9.777639606	1.40448E-22	1.05365E-21
NUP58	3096.341031	0.333959863	0.034137469	9.782795087	1.33473E-22	1.00171E-21
SECISBP2L	3614.316114	0.412279276	0.042140453	9.783456156	1.32603E-22	9.95569E-22
MED24	2445.534682	-0.219840823	0.022470478	-9.783540019	1.32494E-22	9.95125E-22
SAC3D1	141.9999905	-0.565937677	0.057835318	-9.785330016	1.3017E-22	9.78048E-22
KISS1	2.395580355	-1.531427033	0.156494578	-9.785815264	1.29547E-22	9.73742E-22
OSBPL11	851.5260159	0.372636742	0.03807161	9.787785192	1.27048E-22	9.55327E-22
UBE2O	2213.733708	-0.514453258	0.052551096	-9.789581909	1.24811E-22	9.38864E-22
MINDY4	22.43155367	-0.661110407	0.06750464	-9.793554981	1.20001E-22	9.03029E-22
BICD1	437.5291094	-0.44000488	0.044926068	-9.793977086	1.19501E-22	8.99611E-22
DIS3L	1066.976235	-0.285904853	0.0291834	-9.796831463	1.16173E-22	8.74893E-22
GPR161	68.41224951	-0.687641637	0.070186238	-9.797385548	1.15537E-22	8.70444E-22
DNAJC5G	3.335872941	-1.092706339	0.111514891	-9.7987482	1.13989E-22	8.59113E-22
RAP2B	3031.44209	0.388994913	0.039697066	9.799084785	1.1361E-22	8.56585E-22
EMCN	9.255151699	-1.27104689	0.12970811	-9.799286191	1.13384E-22	8.55208E-22
KIFC1	645.5466863	-0.513852344	0.052436286	-9.799556489	1.13081E-22	8.53252E-22
SLC29A3	187.9477443	-0.447903587	0.045706354	-9.799591317	1.13042E-22	8.53252E-22
NOC3L	338.1621953	-0.43195518	0.044073833	-9.80071739	1.11789E-22	8.44152E-22
TUBG1	605.7596401	-0.586569544	0.059847688	-9.801039388	1.11433E-22	8.4179E-22
SEPTIN5	24.06024024	-0.748527032	0.07636907	-9.801442321	1.10989E-22	8.38762E-22
MCM2	1830.855081	-0.44059165	0.044947018	-9.802466832	1.09869E-22	8.30617E-22
CLCNKA	17.23662935	-0.875929671	0.089355127	-9.802791408	1.09517E-22	8.28272E-22
SLC35B4	502.1801785	-0.403054246	0.041098593	-9.807008469	1.05036E-22	7.94695E-22
TAS2R7	1.097307046	-1.382702949	0.140974038	-9.808209832	1.03794E-22	7.85596E-22
AC027796.3	9.224266995	-0.627575293	0.063973001	-9.810002459	1.01966E-22	7.72063E-22
CMPK1	2986.50877	0.329897638	0.033625901	9.810819371	1.01144E-22	7.66134E-22
PDHX	390.8643996	-0.278959874	0.028433492	-9.810960754	1.01002E-22	7.65357E-22
SLC20A2	826.4263001	-0.258399174	0.026337546	-9.811057381	1.00906E-22	7.64921E-22
TRIM56	2138.811174	-0.337996487	0.034441916	-9.813521545	9.84708E-23	7.46753E-22
TNFRSF10A	491.1207358	0.470980501	0.047992297	9.81366871	9.83273E-23	7.45953E-22
RASSF2	6598.620476	0.398788205	0.040634169	9.814110006	9.7898E-23	7.42985E-22
DAZAP2	10914.5595	0.425506482	0.043356485	9.814137007	9.78718E-23	7.42985E-22
OR10H5	8.112225801	-0.725948881	0.073950285	-9.816715102	9.54017E-23	7.24601E-22
CYP2C19	2.552982843	-1.234510972	0.12575184	-9.817040982	9.50939E-23	7.22544E-22
PDE6D	316.5178937	-0.433165175	0.044122656	-9.817295919	9.48537E-23	7.20999E-22

PCSK9	11.15647896	-1.112606847	0.113282254	-9.821545841	9.09382E-23	6.91505E-22
DSG2	89.45031148	-1.347187183	0.13714428	-9.823137978	8.95129E-23	6.80931E-22
MTMR10	4095.859712	0.388314278	0.039511445	9.827893597	8.5386E-23	6.4979E-22
UBQLN1	7618.010904	0.287638204	0.029257102	9.831397506	8.24663E-23	6.27816E-22
NDUFV1	2510.886725	-0.260500005	0.026495211	-9.83196574	8.20023E-23	6.24525E-22
NDUFV2	1881.584764	0.487422981	0.049551138	9.836766552	7.81832E-23	5.95672E-22
VMO1	8.558713413	1.016200983	0.103302228	9.837164232	7.78749E-23	5.93554E-22
RPP25	48.59479136	-0.847972083	0.086184809	-9.838997064	7.64693E-23	5.83067E-22
GFUS	1002.78873	-0.512293683	0.052066086	-9.839297036	7.62417E-23	5.81558E-22
ATG16L2	5835.847576	-0.431319991	0.043832447	-9.840198849	7.55613E-23	5.76593E-22
NPTN	1410.226452	0.283147322	0.028773999	9.840388413	7.54191E-23	5.75732E-22
PNRC2	3150.008241	0.251860754	0.025591649	9.841521218	7.45746E-23	5.69508E-22
RIPK2	3880.526392	0.495598339	0.05035392	9.84229899	7.40002E-23	5.65342E-22
CEP128	659.7622765	-0.420242517	0.042691861	-9.84362145	7.30336E-23	5.58175E-22
MPV17L	140.9216707	-0.853892994	0.086740288	-9.844249045	7.25792E-23	5.54919E-22
SCYL3	349.4558233	-0.307473843	0.031232793	-9.844583674	7.23381E-23	5.53292E-22
CXCR3	85.73685436	0.792098754	0.0804593	9.844713494	7.22448E-23	5.52794E-22
EGFL8	59.27265613	-0.577828535	0.058670317	-9.848737257	6.94105E-23	5.31315E-22
CENPF	1744.543236	-0.601924348	0.061115047	-9.849036781	6.9204E-23	5.29941E-22
INSIG1	2323.347855	0.456046954	0.046298371	9.850172879	6.84262E-23	5.2419E-22
MFSD2B	410.5495554	-0.753440928	0.076486877	-9.850590763	6.81423E-23	5.22219E-22
FBXO16	2.756606013	-1.339643759	0.135995859	-9.850621706	6.81213E-23	5.22219E-22
ZNF37A	540.1759781	-0.36782619	0.037335107	-9.852019252	6.71805E-23	5.15252E-22
SOX21	1.78485969	-1.553698945	0.157677636	-9.853641803	6.61044E-23	5.07197E-22
C7	28.68712445	-1.206747454	0.122466857	-9.853665609	6.60887E-23	5.07197E-22
NLN	464.2104347	-0.501487795	0.050893251	-9.853719044	6.60536E-23	5.07197E-22
ZNF589	518.8140513	-0.352722122	0.035795718	-9.85375173	6.60321E-23	5.07197E-22
LIN7B	41.36485132	-0.461714308	0.046854165	-9.854285355	6.56823E-23	5.0475E-22
B3GNTL1	1149.358469	-0.450151932	0.045665647	-9.857561746	6.35744E-23	4.88744E-22
KIF22	1587.375277	-0.303994987	0.030838139	-9.8577603	6.34489E-23	4.8797E-22
RAB21	2759.029895	0.326298925	0.033100382	9.857859827	6.3386E-23	4.87679E-22
AGFG2	234.3657833	-0.425023351	0.043098049	-9.86177709	6.09608E-23	4.69204E-22
SOGA3	3.301772762	-0.916308624	0.092906452	-9.862701751	6.04018E-23	4.65084E-22
FGF8	5.889649163	-0.77788425	0.078857069	-9.864483455	5.93391E-23	4.57081E-22
CLEC3B	5.671612644	-0.895510878	0.090777705	-9.864876837	5.91069E-23	4.55473E-22
ARMC7	246.1367075	-0.315160191	0.031947376	-9.864979028	5.90468E-23	4.55188E-22
DYRK4	167.1874807	-0.442170091	0.044820946	-9.865255744	5.88842E-23	4.54114E-22

OR2G3	0.826764145	-1.645471784	0.166722374	-9.869531863	5.64273E-23	4.35338E-22
KIF5B	6150.647917	0.268821754	0.027237368	9.869593628	5.63926E-23	4.35242E-22
LYL1	2100.96912	-0.469410327	0.047554256	-9.871047579	5.55811E-23	4.29147E-22
CCDC102A	127.4566595	-0.61314614	0.062107913	-9.872270835	5.49073E-23	4.24112E-22
THUMPD3	854.6795565	0.24366	0.024680898	9.87241231	5.48299E-23	4.23681E-22
CCNB1	546.0356077	-0.470621147	0.047666939	-9.873114603	5.44472E-23	4.20891E-22
SPC24	332.4853441	-0.514572494	0.052105532	-9.875582755	5.31233E-23	4.10819E-22
DUSP4	332.5867663	0.918683384	0.093019636	9.876230683	5.2781E-23	4.08333E-22
MTRNR2L6	18.79539719	-1.133902175	0.114803588	-9.876887979	5.24361E-23	4.05825E-22
NKAIN3	13.89401409	-1.039736867	0.105268695	-9.876980642	5.23876E-23	4.0561E-22
C9orf64	101.7571432	-0.554716557	0.056156233	-9.878094114	5.18089E-23	4.01288E-22
SIL1	715.5122903	-0.327642035	0.033161047	-9.880328367	5.06666E-23	3.92596E-22
SPIN2B	51.75614793	-0.431806719	0.043701648	-9.880787902	5.04348E-23	3.90954E-22
CLNK	44.12499917	-1.036763582	0.10492091	-9.881381881	5.01367E-23	3.88798E-22
OR52A1	4.936193212	-1.185095689	0.119928094	-9.881718715	4.99684E-23	3.87646E-22
KIF20A	448.9342236	-0.576640673	0.058319482	-9.887616536	4.71111E-23	3.65624E-22
NUP50	4291.939646	0.305623088	0.030909162	9.887783032	4.70328E-23	3.65162E-22
GZMH	39.69353834	0.918458316	0.092849537	9.891899796	4.51379E-23	3.50589E-22
KNCN	2.792320518	-1.137775392	0.114932916	-9.89947378	4.18471E-23	3.25158E-22
SECTM1	208.5014388	0.916826204	0.092611494	9.899702137	4.17517E-23	3.24545E-22
BSN	66.73992675	-0.551999719	0.055757662	-9.899979682	4.1636E-23	3.23775E-22
PRSS27	55.39598889	-0.473802224	0.047855971	-9.900587512	4.13837E-23	3.21941E-22
GTF2B	1274.375352	0.343918099	0.034737067	9.900608437	4.1375E-23	3.21941E-22
DGKI	10.92458475	-1.137996254	0.114937014	-9.901042465	4.11958E-23	3.20734E-22
PCED1B	125.2256258	0.657874543	0.066444351	9.901135841	4.11574E-23	3.20562E-22
RCOR1	4136.740191	0.277664919	0.028043388	9.901261621	4.11056E-23	3.20287E-22
NCF4	2342.736242	-0.457503647	0.046185959	-9.905686918	3.93258E-23	3.06541E-22
PNRC1	13960.88071	0.332798176	0.033588513	9.908094844	3.83897E-23	2.99363E-22
KIAA0825	198.7352277	-0.640520291	0.064639824	-9.909066061	3.80183E-23	2.96585E-22
TMCC3	300.6466645	0.809609795	0.081701978	9.909304676	3.79276E-23	2.95996E-22
LRRC47	1038.755943	-0.244585796	0.024678818	-9.910758262	3.73798E-23	2.91837E-22
CCL20	13.27938996	1.221703344	0.123199612	9.916454513	3.53076E-23	2.75768E-22
TLR1	810.2674252	0.482638996	0.048666634	9.917246278	3.50287E-23	2.73699E-22
CCDC34	109.5806593	-0.629982395	0.063522292	-9.91750102	3.49394E-23	2.73111E-22
ATP8B1	166.0027432	-0.600919904	0.060587995	-9.918134895	3.47183E-23	2.71491E-22
PRMT2	1898.061801	-0.30993356	0.031247069	-9.918804294	3.44863E-23	2.69784E-22
SLC6A18	1.372721156	-1.504460421	0.151637841	-9.921404917	3.35993E-23	2.62951E-22

EHD4	784.4431929	0.587561753	0.059221251	9.921468109	3.3578E-23	2.62889E-22
CETN3	82.12799759	-0.49190074	0.049577722	-9.921810132	3.34632E-23	2.62095E-22
EFHC1	694.7098728	-0.354575883	0.035735002	-9.922369202	3.32762E-23	2.60735E-22
ZDBF2	205.5111742	0.885798928	0.089243012	9.925695083	3.21852E-23	2.52287E-22
GAS7	6033.184002	0.55504705	0.05591732	9.92620985	3.20196E-23	2.51089E-22
KIAA0895L	554.3790155	-0.439648987	0.044285661	-9.927569824	3.1586E-23	2.47788E-22
SEC24B	3181.407086	0.320178656	0.032246423	9.929121694	3.10983E-23	2.4406E-22
GBP1	318.3322292	0.847342314	0.085335142	9.929582309	3.09549E-23	2.43033E-22
IRAK1BP1	174.3819622	-0.467036884	0.047033813	-9.929811219	3.0884E-23	2.42573E-22
AC055811.2	6.545710736	-0.561014472	0.056497844	-9.929838662	3.08755E-23	2.42573E-22
ICE2	1142.724044	-0.288207132	0.029021128	-9.930941818	3.05358E-23	2.40031E-22
SH3GL3	4.909973259	-1.520562848	0.153112379	-9.93102492	3.05103E-23	2.39927E-22
SCAF8	3950.958052	0.287400474	0.028937785	9.931668166	3.03141E-23	2.3848E-22
MLPH	5.378350432	-1.082646533	0.108971561	-9.935129136	2.92796E-23	2.30434E-22
KIR3DL2	11.83569687	0.762443972	0.076739589	9.935471151	2.91793E-23	2.29737E-22
NUTM1	4.775878636	-0.818442501	0.082374892	-9.935582062	2.91469E-23	2.29574E-22
PGLYRP2	10.90566025	-0.846375361	0.08517618	-9.936761254	2.8804E-23	2.26965E-22
KRTAP5-9	14.53993497	-0.61333257	0.06171363	-9.938364884	2.83441E-23	2.23431E-22
PSMB5	704.2041216	-0.41313836	0.041563247	-9.939992358	2.78849E-23	2.19899E-22
TAS2R3	16.07221949	-0.805106734	0.080986354	-9.941264122	2.75311E-23	2.17197E-22
ADAMTS19	2.28437397	-2.641762611	0.265712822	-9.942172117	2.72812E-23	2.15313E-22
MDH1B	7.51667672	-0.796264019	0.080070148	-9.944580376	2.66294E-23	2.10253E-22
DAGLA	123.9226752	0.914727785	0.091978908	9.944973262	2.65245E-23	2.09509E-22
FLRT1	30.20059595	-0.745891054	0.074995685	-9.945786241	2.63088E-23	2.0789E-22
GZMK	20.36495763	1.005272485	0.101047179	9.948545797	2.55895E-23	2.02287E-22
WDR90	1232.943588	-0.381738615	0.038366801	-9.949711942	2.52914E-23	2.00012E-22
ZNF572	36.10676977	-0.549316329	0.055208763	-9.949803175	2.52682E-23	1.99909E-22
ZSCAN22	154.8176226	-0.389723351	0.039162889	-9.951343297	2.48802E-23	1.96919E-22
NMRAL1	732.75629	-0.360835815	0.0362574	-9.952059729	2.47017E-23	1.95585E-22
STARD4	829.1189834	0.618599351	0.062152097	9.952992497	2.44712E-23	1.93838E-22
ZNF554	185.9578824	-0.389172014	0.039085181	-9.95702209	2.34996E-23	1.86218E-22
AGRN	1406.28734	0.804948465	0.080833471	9.958108365	2.32443E-23	1.84269E-22
TTC27	643.5324801	-0.505628375	0.050771019	-9.958996036	2.30377E-23	1.82706E-22
MESP1	15.97867462	-0.709492067	0.071239427	-9.959261242	2.29764E-23	1.82293E-22
ZBTB1	2050.427548	0.322576304	0.032388593	9.959565179	2.29062E-23	1.8181E-22
SCRIB	1880.007489	-0.40622605	0.040785382	-9.960089274	2.27858E-23	1.80928E-22
PPM1E	18.67879789	-0.853731425	0.085715114	-9.960103761	2.27825E-23	1.80928E-22

PLCXD1	2092.35337	-0.358545647	0.035993253	-9.961468157	2.24719E-23	1.7858E-22
ICOSLG	171.0933346	0.823983056	0.082689615	9.964770779	2.17375E-23	1.72814E-22
MTSS1	1359.033167	0.713902523	0.071635908	9.965707706	2.15335E-23	1.71262E-22
ADAM9	3057.404894	0.615393482	0.061741607	9.967241036	2.12037E-23	1.68707E-22
CGA	3.508049369	-2.110333367	0.211719457	-9.967592945	2.11287E-23	1.68179E-22
SELP	255.5327639	-1.051412364	0.105470188	-9.968810926	2.08713E-23	1.66197E-22
GALNT16	13.25041758	-0.652179572	0.065411639	-9.970390301	2.0542E-23	1.63642E-22
COX4I2	2.480617994	-1.166622472	0.116981487	-9.972710192	2.00677E-23	1.59929E-22
STK26	2963.107692	0.339781948	0.03406644	9.974096086	1.97895E-23	1.57776E-22
CORIN	13.97599403	-0.762494894	0.076440616	-9.974996678	1.96108E-23	1.56415E-22
STPG2	24.19507054	-0.700467909	0.070218431	-9.975556244	1.95006E-23	1.55599E-22
ILDR1	23.60167615	-0.706751786	0.070847625	-9.975659606	1.94803E-23	1.55501E-22
MAGI2	31.22533045	-0.719008151	0.07207582	-9.97571932	1.94686E-23	1.5547E-22
SCART1	103.9577078	-0.607456973	0.06088971	-9.976348621	1.93455E-23	1.54551E-22
SUSD6	4932.969752	0.373889597	0.037469717	9.978447232	1.89408E-23	1.51379E-22
ZNF786	140.6958732	-0.512994019	0.051405859	-9.979290806	1.87804E-23	1.50159E-22
UROD	1962.725389	-0.566402924	0.056752221	-9.980277625	1.85946E-23	1.48734E-22
ACSL4	2096.786787	0.359546792	0.036020567	9.981708356	1.83284E-23	1.46664E-22
KASH5	11.65339335	-0.90959019	0.09112383	-9.981913485	1.82905E-23	1.46421E-22
AGTR1	31.94538973	-1.283864093	0.128617792	-9.98201003	1.82727E-23	1.46339E-22
ANKRD36B	861.793501	-0.501118535	0.050187825	-9.984862642	1.77547E-23	1.42248E-22
CLVS1	6.346675328	-0.863919037	0.086507873	-9.986594384	1.74473E-23	1.39843E-22
PATE4	4.632398908	-1.228408308	0.122999982	-9.987060886	1.73654E-23	1.39244E-22
RBM4B	793.7956764	-0.395028444	0.039539938	-9.990618682	1.67533E-23	1.3439E-22
SLC28A2	8.108535864	-0.765138542	0.076572915	-9.992286921	1.64737E-23	1.32201E-22
HSPA9	7972.022063	0.219373795	0.021953989	9.992434515	1.64491E-23	1.32059E-22
KRR1	2265.175291	0.333484209	0.033360338	9.996427903	1.57994E-23	1.26894E-22
SOS2	4000.046907	0.325646272	0.032569969	9.998359996	1.54942E-23	1.24494E-22
TMSB15A	4.823115677	-1.835458944	0.183567681	-9.998813145	1.54234E-23	1.23977E-22
CCDC158	3.573901833	-0.878625768	0.087845296	-10.00196719	1.49399E-23	1.20139E-22
NINJ2	239.4807249	-0.659181402	0.065900542	-10.00267041	1.48342E-23	1.19338E-22
CACNA1F	118.1897189	-0.578547718	0.057836585	-10.00314459	1.47633E-23	1.18817E-22
C1S	81.38201597	-0.551922331	0.055172459	-10.00358397	1.46979E-23	1.18339E-22
FAAH	637.417606	-0.608096784	0.060787743	-10.00360849	1.46943E-23	1.18339E-22
OTULIN	2602.367838	0.357369672	0.035722232	10.00412509	1.46178E-23	1.17791E-22
PPP1R16B	4242.279816	0.826211341	0.082582337	10.00469803	1.45334E-23	1.17159E-22
GRWD1	855.6689139	-0.324619114	0.032444487	-10.00537058	1.4435E-23	1.16414E-22

VPS16	777.5820071	-0.270201905	0.027000536	-10.00727912	1.41593E-23	1.14238E-22
INTU	105.0084769	-1.020987339	0.102023323	-10.00739158	1.41432E-23	1.14155E-22
LRRTM4	1.692106015	-1.94807264	0.194614198	-10.00992044	1.37863E-23	1.1132E-22
ITGBL1	3.403024419	-1.65953132	0.165788237	-10.0099461	1.37828E-23	1.1132E-22
KALRN	151.7414559	-1.212264785	0.121102327	-10.01025178	1.37402E-23	1.1104E-22
PTER	274.3848264	-0.394362662	0.039395599	-10.01032286	1.37304E-23	1.11006E-22
ADHFE1	44.96959467	-0.481958271	0.048145023	-10.0105524	1.36986E-23	1.10794E-22
TRMT61A	366.6139434	-0.337931579	0.033757274	-10.01062994	1.36878E-23	1.10753E-22
GZMA	60.53146553	1.002420261	0.100108581	10.01332999	1.33192E-23	1.07815E-22
TRIM54	10.60497587	-0.762153127	0.076109773	-10.01386682	1.32471E-23	1.07276E-22
UCP3	100.3084248	-0.450018914	0.044924714	-10.01717921	1.28107E-23	1.03785E-22
TDRKH	67.39166802	-0.668755971	0.06675954	-10.01738442	1.27841E-23	1.03612E-22
DNAH6	52.19269711	-0.780875442	0.077944142	-10.01839808	1.26537E-23	1.02598E-22
ENPP6	3.627598558	-1.249974969	0.124764762	-10.01865389	1.2621E-23	1.02375E-22
SMN1	221.5616496	0.355504143	0.035482956	10.01901152	1.25754E-23	1.02048E-22
PIK3C2G	1.57078776	-1.922797267	0.191867754	-10.02147172	1.22663E-23	9.95801E-23
ZNF599	57.97444975	-0.525242835	0.052398477	-10.0240096	1.19552E-23	9.70952E-23
POLR3F	316.8436247	0.27189818	0.027124264	10.02416807	1.19361E-23	9.69798E-23
VPREB1	43.38200811	-1.218754156	0.121580355	-10.02426875	1.19239E-23	9.69213E-23
EGF	53.00110913	-1.101329998	0.109851132	-10.02565905	1.17573E-23	9.56065E-23
ECHS1	1299.309591	-0.481464055	0.048022253	-10.0258531	1.17342E-23	9.54585E-23
SMAD2	2962.411171	-0.303234616	0.030241904	-10.02696856	1.16024E-23	9.44258E-23
DUSP8	1087.309573	0.863066017	0.086072873	10.02715472	1.15806E-23	9.42872E-23
TMEM86B	148.1882819	-0.378113756	0.037704266	-10.02840779	1.14346E-23	9.31372E-23
ANKRD65	16.95818972	-1.163337954	0.11599296	-10.02938417	1.13221E-23	9.22592E-23
C12orf50	2.889532495	-0.949099426	0.094580171	-10.03486693	1.07104E-23	8.73112E-23
HLA-E	22176.917	0.373935347	0.037260319	10.03575258	1.06147E-23	8.65672E-23
MGAT1	3738.320521	0.351993314	0.035067146	10.03769488	1.04078E-23	8.49152E-23
EXOC3L1	26.81118661	-0.617626145	0.061507891	-10.04141311	1.00228E-23	8.18082E-23
TRO	153.9989264	-1.12713996	0.112238701	-10.04234683	9.92835E-24	8.10711E-23
UBQLN4	832.4248475	-0.3500474	0.034856968	-10.04239376	9.92362E-24	8.10664E-23
ALG1	234.9913408	-0.403895644	0.040209288	-10.04483465	9.68099E-24	7.91173E-23
PTBP3	7609.496339	0.253740262	0.025257227	10.0462437	9.54361E-24	7.80272E-23
UHRF1	1525.312182	-0.521201472	0.05187401	-10.04744907	9.42762E-24	7.71111E-23
ALG6	264.9408752	-0.378752652	0.037691651	-10.04871493	9.30732E-24	7.61589E-23
GPR17	8.871432519	-0.920746074	0.091599712	-10.05184468	9.01636E-24	7.38089E-23
RGPD5	17.05517014	0.843141096	0.083879035	10.05186926	9.01411E-24	7.38089E-23

FOXP2	6.562960422	-1.626723856	0.161824062	-10.0524226	8.96363E-24	7.34387E-23
SMOX	1698.271875	0.787163408	0.078303908	10.05267076	8.94108E-24	7.32846E-23
TAF13	383.5211902	0.411464182	0.040925262	10.05403894	8.81776E-24	7.23041E-23
PIWIL3	4.497675162	-0.998866977	0.099348687	-10.05415375	8.80749E-24	7.22502E-23
AKR1C1	225.0461106	-0.873069749	0.086807442	-10.0575449	8.50939E-24	6.98341E-23
XYLT2	668.7144782	-0.363030993	0.036094837	-10.05769856	8.49612E-24	6.97545E-23
SAMM50	828.9236279	-0.306288503	0.030451164	-10.05835129	8.43999E-24	6.93227E-23
KNTC1	2807.108428	-0.459088538	0.045638337	-10.0592741	8.36126E-24	6.87048E-23
BOK	16.50594697	-0.785024315	0.078019297	-10.06192506	8.1391E-24	6.69075E-23
GLRA2	2.64039432	-0.964349624	0.095828883	-10.06324597	8.0306E-24	6.60433E-23
OR1I1	8.704910449	-0.791065869	0.078608351	-10.06338205	8.0195E-24	6.59797E-23
MAL	81.50386041	0.80647239	0.080127376	10.06487954	7.8984E-24	6.50106E-23
COL8A1	8.552406059	-1.095981363	0.108876754	-10.06625681	7.78861E-24	6.4134E-23
GPR153	349.0401705	1.049165312	0.104225749	10.0662775	7.78697E-24	6.4134E-23
MORF4L1	8182.138656	0.227151151	0.022563086	10.06737969	7.70022E-24	6.34596E-23
FAM160B2	1403.267346	-0.280244888	0.0278353	-10.06796716	7.65438E-24	6.31083E-23
CARF	507.4797995	-0.383460469	0.038084755	-10.06860798	7.60467E-24	6.27249E-23
IL24	62.12592514	0.882058103	0.087599094	10.06926062	7.55438E-24	6.23364E-23
PRKAG2	1265.006355	0.366037074	0.036341273	10.07221399	7.3309E-24	6.05178E-23
TMEM116	194.6681767	-0.376146964	0.037336938	-10.07439227	7.17027E-24	5.92167E-23
RBCK1	1655.225679	-0.306529534	0.030421811	-10.07597929	7.05544E-24	5.8293E-23
YPEL1	221.1348051	-0.430521134	0.042726608	-10.07618321	7.04082E-24	5.81968E-23
SWI5	151.9774404	-0.453601275	0.045011284	-10.07750125	6.94703E-24	5.74458E-23
C1QTNF3	104.9638296	-0.529236886	0.052515764	-10.07767667	6.93464E-24	5.73676E-23
KRTAP5-7	7.228000962	-0.773529904	0.076756252	-10.07774464	6.92985E-24	5.73522E-23
ADCY2	40.84093191	-1.383092132	0.137224983	-10.07901112	6.84111E-24	5.66418E-23
TMEM218	248.8715672	-0.468400353	0.046468097	-10.0800416	6.76975E-24	5.60746E-23
NDUFAF2	202.5915389	-0.550434125	0.054595126	-10.08211111	6.62864E-24	5.4929E-23
TTYH1	91.67748731	-0.52214636	0.051785692	-10.08283061	6.58026E-24	5.45513E-23
CDH18	2.326480505	-1.195634577	0.118572415	-10.08358122	6.53017E-24	5.41589E-23
C18orf54	143.0921955	-0.515175959	0.051089306	-10.08383161	6.51355E-24	5.40439E-23
VN1R1	28.72769222	-0.524601737	0.052020954	-10.08443139	6.47389E-24	5.37377E-23
IL17B	6.952044336	-0.772755475	0.076609173	-10.08698363	6.30781E-24	5.23813E-23
RAB22A	1106.483971	0.306265709	0.030359612	10.08793223	6.24716E-24	5.18997E-23
NPIPA1	150.6880956	-0.425600166	0.042187305	-10.08834679	6.22084E-24	5.17029E-23
BPIFB1	4.91927956	-1.140920651	0.113090329	-10.08857843	6.20618E-24	5.1603E-23
ATP5MF	657.2541817	-0.607121509	0.060169422	-10.09020018	6.10449E-24	5.07791E-23

C16orf72	6020.100454	0.380393944	0.037693786	10.09168848	6.01263E-24	5.00362E-23
HEATR5A	231.8677581	-0.541019784	0.053579897	-10.09743974	5.67032E-24	4.72077E-23
EIF2A	3757.291247	0.323941984	0.032077677	10.09867333	5.59946E-24	4.66375E-23
HBEGF	1285.073426	0.846052786	0.083776643	10.09891008	5.58596E-24	4.65449E-23
INVS	1042.815974	-0.308582152	0.030555042	-10.09922216	5.56821E-24	4.64168E-23
HAPLN3	206.4313634	0.69062986	0.06836786	10.10167434	5.43069E-24	4.52897E-23
TMEM158	81.07262143	1.006953031	0.099672235	10.10264329	5.37729E-24	4.48635E-23
MLIP	4.913918122	-0.810522708	0.080218913	-10.10388544	5.30959E-24	4.43175E-23
KLHL13	193.9162756	-0.849823784	0.084097805	-10.10518383	5.23972E-24	4.3753E-23
GINS2	304.6303392	-0.473934921	0.046899048	-10.10542725	5.22672E-24	4.36631E-23
PLIN1	8.1367414	-0.65780975	0.065087046	-10.10661554	5.16374E-24	4.31553E-23
AC104389.5	42.15688212	-0.596392158	0.05900931	-10.10674691	5.15682E-24	4.31159E-23
SMIM17	14.43087369	-0.618654457	0.061210831	-10.10694431	5.14644E-24	4.30476E-23
ADAT1	814.3929	-0.26019922	0.025741096	-10.10831952	5.07472E-24	4.24658E-23
CDC40	1991.816296	0.338221173	0.033459152	10.10848004	5.06642E-24	4.24145E-23
THEGL	6.892480578	-1.075918027	0.106419957	-10.11011523	4.98256E-24	4.17303E-23
CEP250	1236.96251	-0.278844817	0.02757551	-10.11204577	4.88533E-24	4.09335E-23
SPOP	940.9986111	-0.27826215	0.027512456	-10.11404258	4.78674E-24	4.01246E-23
ERVMER34-1	3.969675523	-1.138143594	0.112489176	-10.1178054	4.60628E-24	3.86284E-23
STX17	863.3379889	-0.353951038	0.034978045	-10.11923459	4.53952E-24	3.80848E-23
MTLN	78.22437253	-0.704777553	0.069594316	-10.12694132	4.19569E-24	3.52154E-23
ISOC2	436.9649675	-0.556084146	0.05490838	-10.12749142	4.17216E-24	3.50329E-23
CHST15	679.9406279	0.92035461	0.090868276	10.12844804	4.13155E-24	3.47068E-23
POLE2	238.6618854	-0.384506394	0.037959415	-10.12940773	4.0912E-24	3.43826E-23
DNAH11	79.30924105	-0.636575299	0.062838805	-10.13028973	4.05446E-24	3.40885E-23
NPAS1	55.71282756	-0.510710891	0.050410121	-10.13111814	4.02026E-24	3.38154E-23
XRN2	4445.660046	0.202533655	0.019990819	10.13133327	4.01142E-24	3.37556E-23
AMT	43.61297896	-0.564551304	0.055713049	-10.13319714	3.93566E-24	3.31324E-23
WAC	9127.028185	0.325299282	0.032097376	10.13476239	3.87314E-24	3.262E-23
XIRP2	19.12549907	-1.516229485	0.149602685	-10.13504194	3.86208E-24	3.25409E-23
TTBK1	7.504821794	-0.946733112	0.093409755	-10.13527026	3.85307E-24	3.24789E-23
TLCD3B	29.17644407	-0.540914822	0.053368584	-10.13545387	3.84583E-24	3.24319E-23
RUSC1	565.1797119	-0.334376531	0.032990473	-10.13554816	3.84213E-24	3.24146E-23
CNKSR1	47.0119047	-0.499810859	0.049306921	-10.1367282	3.79601E-24	3.20394E-23
CHAD	21.03510945	-0.645133095	0.063618829	-10.14059995	3.64852E-24	3.08078E-23
PLVAP	108.1069325	-0.946252681	0.093313027	-10.14062787	3.64748E-24	3.08078E-23
GLRA4	5.26897826	-0.882287739	0.086991976	-10.14217377	3.5902E-24	3.03415E-23

FCN3	11.27830099	-0.974993347	0.096118172	-10.14369421	3.53474E-24	2.98857E-23
SYT2	97.67825205	-0.859142029	0.084697135	-10.1436965	3.53466E-24	2.98857E-23
DCDC2	2.417393233	-1.565112891	0.154288374	-10.14407537	3.52097E-24	2.9795E-23
ERMP1	1328.293715	-0.49587532	0.048878454	-10.14506975	3.48529E-24	2.95059E-23
LRP11	10.06941762	-0.7179654	0.070764991	-10.1457711	3.46035E-24	2.93074E-23
PRSS51	46.58774477	-0.695850396	0.068583219	-10.14607369	3.44964E-24	2.92293E-23
MTCH1	5378.104343	0.363752031	0.035849902	10.14652783	3.43363E-24	2.91063E-23
POLD1	1522.885619	-0.383797177	0.037824422	-10.14680885	3.42376E-24	2.90352E-23
SNW1	1726.768679	0.263037032	0.025920376	10.14788657	3.38617E-24	2.87289E-23
SMIM13	334.4772245	0.415376379	0.040931787	10.14801474	3.38173E-24	2.87036E-23
RAB9A	556.3580695	0.355923809	0.035072049	10.14836081	3.36976E-24	2.86144E-23
ZNF213	354.8668371	-0.373600987	0.036813804	-10.14839389	3.36861E-24	2.86144E-23
SERTAD4	2.673851322	-1.612517825	0.158873585	-10.14969118	3.32414E-24	2.82516E-23
CXCR4	11896.92931	0.577787903	0.056920788	10.15073611	3.28873E-24	2.79628E-23
CLDN11	5.391740653	-1.066567181	0.105065935	-10.1514081	3.26616E-24	2.7783E-23
TDRP	9.829367903	-1.032719042	0.101727318	-10.15183595	3.25187E-24	2.76734E-23
BMPR1B	14.53524884	-1.404518694	0.138336359	-10.15292514	3.21577E-24	2.73781E-23
TBCEL	370.2412888	-0.636704107	0.062701505	-10.15452673	3.16341E-24	2.69441E-23
ACSL3	1472.965355	0.371287812	0.036553602	10.15735214	3.07309E-24	2.61862E-23
HLF	33.38286277	-1.182009642	0.116325472	-10.161228	2.95333E-24	2.51766E-23
FILIP1	12.91690993	-0.687002278	0.067605127	-10.16198482	2.93049E-24	2.49928E-23
ZFYVE21	280.3295626	-0.383058655	0.03769504	-10.16204412	2.92871E-24	2.49885E-23
SHARPIN	978.025926	-0.328927503	0.032367755	-10.16219688	2.92412E-24	2.49602E-23
SFRP5	46.00744503	-0.991641099	0.097577431	-10.16260715	2.91184E-24	2.48663E-23
REXO5	598.4159444	-0.429167542	0.042227653	-10.16318715	2.89456E-24	2.47295E-23
ZNF774	27.36591	-0.571009628	0.056175705	-10.16470779	2.84975E-24	2.43573E-23
CERT1	2882.869114	0.333772879	0.032834597	10.16528006	2.83307E-24	2.42253E-23
NPHP1	15.86555935	-0.748130109	0.073581444	-10.16737467	2.77281E-24	2.37204E-23
DERL2	1041.836706	0.335776766	0.033020348	10.16878349	2.73301E-24	2.33901E-23
LRCH2	4.010744812	-2.321101284	0.228129317	-10.17449804	2.57725E-24	2.20667E-23
SEC63	3481.959353	0.252561804	0.024820847	10.17539026	2.55374E-24	2.1875E-23
LIN28A	12.33532978	-0.649379037	0.063816427	-10.17573482	2.54472E-24	2.18073E-23
FPGT-TNNI3K	16.56463167	-0.763132341	0.074993234	-10.17601592	2.53738E-24	2.17539E-23
USP3	3178.645	0.391653468	0.038487021	10.17624791	2.53134E-24	2.17116E-23
KIF4A	347.800879	-0.577095017	0.056702494	-10.17759492	2.49655E-24	2.14226E-23
CDH11	37.28328121	-1.488824396	0.146276209	-10.1781719	2.4818E-24	2.13054E-23
ZDHHC13	322.2261734	-0.467303836	0.045912252	-10.17819459	2.48122E-24	2.13054E-23

CTNNB1	16200.29085	0.376390285	0.036978608	10.17859535	2.47102E-24	2.12315E-23
NRBP1	4102.987693	0.287278933	0.028223107	10.17885558	2.46443E-24	2.11841E-23
STOML1	133.9229041	-0.371022521	0.036446513	-10.17991823	2.43766E-24	2.09632E-23
PLPP7	43.21414321	-0.952884148	0.093600789	-10.18030036	2.42811E-24	2.08903E-23
CD14	1077.854452	1.182786203	0.116176309	10.1809587	2.41173E-24	2.07585E-23
GSDMC	20.86879127	-1.05108827	0.103233255	-10.18168292	2.39385E-24	2.06136E-23
FAM189A1	37.03156047	-1.21119292	0.118929501	-10.18412511	2.3345E-24	2.01115E-23
LRP1	5275.416427	0.956027406	0.093859026	10.18578016	2.29511E-24	1.97808E-23
KCNS3	2.070192042	-1.442492568	0.141617548	-10.18583214	2.29389E-24	1.9779E-23
TMEM121	34.70599878	-0.80698928	0.079221265	-10.18652358	2.27764E-24	1.96475E-23
ADCY10	43.67621787	-0.675978485	0.066355769	-10.18718489	2.2622E-24	1.9523E-23
TAS2R8	1.338168756	-1.282337474	0.125856284	-10.18890304	2.22258E-24	1.91895E-23
SEMA3C	43.04409515	-1.291431787	0.126747945	-10.18897617	2.22091E-24	1.91835E-23
OTX1	5.78335721	-1.275400344	0.125140717	-10.19172959	2.15889E-24	1.8656E-23
PSD2	20.48999	-0.858160757	0.08419431	-10.19262178	2.13916E-24	1.84937E-23
NHLRC1	23.99387654	-0.697530316	0.068422415	-10.1944708	2.09884E-24	1.81532E-23
ZDHHC23	289.1217333	-0.597926633	0.058651561	-10.19455626	2.097E-24	1.81453E-23
TTC24	220.0494495	-0.687960973	0.067482651	-10.19463468	2.09531E-24	1.81386E-23
FUT8	559.3640453	-0.508960246	0.049918202	-10.19588497	2.06852E-24	1.79147E-23
RAD51C	231.9845201	-0.413935673	0.040589853	-10.19800873	2.02379E-24	1.7535E-23
G6PC3	738.7957951	-0.523965978	0.051379193	-10.19801884	2.02358E-24	1.7535E-23
MYOZ3	69.75080231	-0.650625557	0.063795278	-10.19864754	2.01053E-24	1.74355E-23
TRIM29	5.858561693	-1.218732363	0.119494979	-10.19902574	2.00271E-24	1.73755E-23
PAFAH1B3	306.0106065	-0.52804845	0.051769594	-10.19997283	1.98328E-24	1.72145E-23
TULP1	5.138468657	-0.943064678	0.092438869	-10.20203612	1.94159E-24	1.68601E-23
ARSK	79.2392452	-0.422035282	0.041365653	-10.20255338	1.93127E-24	1.6778E-23
C2orf49	987.4558376	0.305343926	0.029924542	10.2037961	1.90671E-24	1.6572E-23
TNC	144.8898978	-0.991448678	0.097158284	-10.20446884	1.89354E-24	1.64648E-23
SLC39A3	987.7959861	-0.440244784	0.043136046	-10.20596059	1.86467E-24	1.6221E-23
PRIMPOL	236.3100087	-0.39766407	0.038962622	-10.20629651	1.85823E-24	1.61721E-23
CUL2	1310.408796	0.259155322	0.025390749	10.20668277	1.85085E-24	1.6115E-23
ISM2	10.40676258	-0.938299403	0.09192789	-10.20690673	1.84658E-24	1.60851E-23
MAP3K3	3474.431598	0.315033457	0.03086213	10.20776793	1.83027E-24	1.59501E-23
KLF17	2.620527969	-0.925165094	0.090626821	-10.20851311	1.81627E-24	1.58351E-23
AHCYL2	522.7947911	-0.417688562	0.040913716	-10.20901068	1.80698E-24	1.57611E-23
VPS41	1528.317065	-0.380157594	0.037213037	-10.21570999	1.6864E-24	1.4716E-23
KCNIP1	7.144871064	-1.072846448	0.104995948	-10.2179795	1.64739E-24	1.43819E-23

AIMP2	206.9316422	-0.405761772	0.039701644	-10.22027627	1.60882E-24	1.40515E-23
COX6B2	33.72467252	0.863058829	0.084439733	10.22100378	1.59679E-24	1.39526E-23
P2RY12	74.37787797	-1.108634129	0.108456497	-10.22192457	1.58169E-24	1.38269E-23
FAM234A	1477.048914	-0.387764593	0.037921758	-10.22538559	1.52619E-24	1.33477E-23
OR1A1	4.189438872	-0.975261388	0.095375166	-10.22552753	1.52396E-24	1.33341E-23
PDK2	295.3496675	-0.504108264	0.049296728	-10.22599839	1.51657E-24	1.32754E-23
TNFRSF18	86.20101696	0.984502152	0.096267576	10.22672632	1.50522E-24	1.31819E-23
WDR87	6.674962778	-1.035058418	0.101211039	-10.22673447	1.50509E-24	1.31819E-23
CFAP65	5.589546044	-1.05317391	0.102982412	-10.22673574	1.50507E-24	1.31819E-23
BCLAF1	11278.19456	0.311027467	0.030411125	10.22742397	1.49442E-24	1.31049E-23
PIRT	2.913124566	-1.064080107	0.104040506	-10.2275561	1.49238E-24	1.30929E-23
CHID1	1208.143247	-0.397433853	0.038852469	-10.22930754	1.46564E-24	1.28641E-23
PKP4	1049.243642	-0.465786335	0.045533997	-10.22941902	1.46395E-24	1.2855E-23
EHMT1	3397.813898	-0.259631768	0.025378104	-10.23054245	1.44707E-24	1.27125E-23
RASD2	45.89958598	1.169324294	0.114287591	10.23141953	1.43402E-24	1.26035E-23
DMAC1	405.4547895	-0.570766139	0.055762604	-10.2356435	1.37279E-24	1.20709E-23
RASSF7	212.0992251	-0.460140933	0.044950726	-10.23656288	1.35982E-24	1.19621E-23
ALB	5.420854977	-0.948256415	0.092624758	-10.23761293	1.34514E-24	1.18383E-23
PSG2	2.439811915	-1.456845201	0.142294404	-10.23824665	1.33636E-24	1.17664E-23
SV2C	21.90855136	-0.759919411	0.074221541	-10.23852908	1.33247E-24	1.17373E-23
DEPDC1B	219.7084876	-0.534422696	0.052193186	-10.23931924	1.32163E-24	1.16471E-23
DKKL1	3.164632168	-1.500330604	0.146512089	-10.24031953	1.30804E-24	1.15325E-23
CCDC71L	769.5069364	0.485068371	0.047346295	10.24511783	1.24473E-24	1.09793E-23
EPM2A	379.4209262	-0.3805119	0.037138825	-10.24566343	1.23773E-24	1.09225E-23
CRYM	25.11143087	-1.085128118	0.105908654	-10.24588712	1.23487E-24	1.09022E-23
FITM2	105.1545842	-0.508311486	0.049603248	-10.2475443	1.21388E-24	1.07217E-23
NFATC4	29.37898669	-1.199915851	0.117089046	-10.24789163	1.20953E-24	1.06881E-23
PRR3	354.3874255	-0.287516512	0.028052684	-10.24916227	1.19374E-24	1.05533E-23
SHC4	8.459344714	-0.753384432	0.073495685	-10.25073018	1.17453E-24	1.03882E-23
RAB30	521.9448679	0.720533846	0.070288111	10.2511482	1.16946E-24	1.0348E-23
ZNF559-ZNF177	9.639692876	-0.666502093	0.065009987	-10.25230315	1.15557E-24	1.02297E-23
TRIM65	684.2778178	-0.352919961	0.034419769	-10.25340874	1.14242E-24	1.01179E-23
KLHDC1	146.7621629	-0.407671801	0.039757276	-10.25401741	1.13525E-24	1.00589E-23
LINGO1	4.62225662	-0.956117966	0.093215076	-10.25711723	1.0994E-24	9.74571E-24
FN3K	206.9717353	-0.83401665	0.08130096	-10.25838625	1.08505E-24	9.62286E-24
SLC45A2	12.38941363	-0.97186944	0.094736783	-10.25862821	1.08233E-24	9.60313E-24
XPO7	5123.595238	-0.53707643	0.052351983	-10.25895094	1.07872E-24	9.57543E-24

SAR1B	1612.633353	0.315487092	0.030751676	10.25918372	1.07613E-24	9.55672E-24
TLR2	2583.560589	0.629304683	0.061324382	10.26190014	1.04627E-24	9.29583E-24
TRIP13	178.8055606	-0.534040113	0.052017962	-10.2664558	9.98041E-25	8.87133E-24
SPATA25	44.52990419	-0.444193296	0.043265377	-10.26671511	9.95363E-25	8.85154E-24
IPO11	580.8489365	-0.350008749	0.034090416	-10.26707177	9.91691E-25	8.82289E-24
HRG	2.664784338	-0.982158637	0.095659481	-10.26723775	9.89987E-25	8.81174E-24
FAM221B	6.23643461	-0.850488028	0.082830715	-10.2677857	9.84381E-25	8.76583E-24
ELMOD3	538.7063775	-0.314955018	0.030657578	-10.273317	9.29526E-25	8.28112E-24
NUP210	8224.996009	-0.524616001	0.051060398	-10.27442044	9.18951E-25	8.19063E-24
STAP2	44.00847093	-0.722265066	0.070292447	-10.27514475	9.12074E-25	8.13304E-24
FGFBP2	50.86043114	0.852674974	0.082973507	10.27647263	8.99599E-25	8.02546E-24
TMEM53	183.752487	-0.401649219	0.03907845	-10.27802319	8.85246E-25	7.90101E-24
ATP7B	434.884042	-0.862070775	0.083863499	-10.27945151	8.72226E-25	7.78835E-24
CATSPERG	216.7187776	-0.460584063	0.04479787	-10.28138311	8.54918E-25	7.6373E-24
TMEM187	76.81959517	-0.445377724	0.043318363	-10.28149947	8.53887E-25	7.63156E-24
PNKP	1238.91331	-0.286699721	0.027884626	-10.28164127	8.52631E-25	7.62382E-24
DTYMK	618.9665373	-0.407224294	0.039603981	-10.28240795	8.45874E-25	7.56686E-24
DUSP5	1830.85043	0.722540713	0.070264029	10.28322347	8.38745E-25	7.50652E-24
TAS2R13	19.28307359	-0.870313832	0.084625958	-10.28424206	8.29925E-25	7.43098E-24
CHRDL1	47.56806781	-1.513729131	0.147185228	-10.28451801	8.27551E-25	7.41311E-24
AP3S1	1488.292814	0.38660748	0.037584578	10.28633289	8.12106E-25	7.27809E-24
DPCD	172.7165661	-0.384132022	0.037338303	-10.28788118	7.99155E-25	7.16531E-24
MRPL12	74.20643203	-0.547697414	0.053236448	-10.28801565	7.9804E-25	7.15859E-24
NOLC1	2179.165985	-0.385183854	0.037438072	-10.28856001	7.93542E-25	7.1215E-24
C9orf72	1468.352494	0.486371139	0.047267253	10.28981182	7.83293E-25	7.03275E-24
PDE4A	537.696764	0.784642734	0.07624294	10.29134934	7.70884E-25	6.92451E-24
BCAN	11.3485483	-0.778454709	0.075615406	-10.29492203	7.42797E-25	6.67529E-24
LMNTD1	1.444453967	-1.435868318	0.139373451	-10.30230868	6.87902E-25	6.1848E-24
SLC31A1	956.7848506	0.408062695	0.039607321	10.30270882	6.85046E-25	6.16195E-24
MIOS	1186.592815	0.24926741	0.024186758	10.30594528	6.62372E-25	5.96074E-24
FKBP7	76.3861004	-0.534594387	0.051870279	-10.30637197	6.59439E-25	5.93708E-24
CSRP3	2.585139494	-1.162791818	0.112811633	-10.30737514	6.52594E-25	5.87815E-24
CIB3	20.53911091	-0.581988302	0.056459904	-10.30799309	6.48412E-25	5.84317E-24
MEX3A	134.6413575	-0.931831287	0.090396264	-10.30829423	6.46384E-25	5.82758E-24
TP53BP2	5461.799551	0.385936083	0.037435612	10.30933001	6.39455E-25	5.76777E-24
MBLAC2	153.5687071	-0.46999259	0.04558639	-10.30993223	6.35461E-25	5.73439E-24
ZNF71	285.1236536	-0.404167492	0.039199304	-10.31057833	6.31203E-25	5.69859E-24

CLUAP1	241.9636511	-0.385816465	0.037415684	-10.31162388	6.24373E-25	5.63952E-24
COL5A1	257.8640028	-1.166525426	0.11312616	-10.31172127	6.2374E-25	5.63641E-24
PRKAR1B	237.4990687	-0.480186491	0.046562133	-10.3128112	6.16705E-25	5.57541E-24
AKR1C2	63.88068984	-1.037258762	0.100569892	-10.31381001	6.10327E-25	5.5203E-24
GSTZ1	391.1816126	-0.391393502	0.037945831	-10.31453222	6.05756E-25	5.48149E-24
EYA2	3.411890322	-1.090063906	0.105675005	-10.3152482	6.01258E-25	5.4433E-24
OR2M3	4.380855245	-1.182955782	0.114668314	-10.31632661	5.94545E-25	5.38502E-24
PRSS37	4.930483339	-1.018959526	0.098749614	-10.31861779	5.80528E-25	5.2605E-24
REL	6367.056942	0.431445356	0.041811169	10.31890196	5.78813E-25	5.24739E-24
ANKRD45	4.253467405	-1.042809555	0.101024871	-10.32230524	5.58655E-25	5.06698E-24
SYMPK	3705.420859	-0.25140818	0.024354202	-10.32299005	5.54683E-25	5.0333E-24
COL25A1	12.80418584	-1.011270431	0.097953164	-10.32402008	5.48763E-25	4.98188E-24
DDX51	1003.787304	-0.307310051	0.029762199	-10.32551572	5.40277E-25	4.90712E-24
TES	2713.094544	0.587515311	0.056893718	10.32654106	5.34535E-25	4.85722E-24
TBX21	65.91620894	0.679633434	0.065812986	10.3267376	5.33441E-25	4.84953E-24
ZNF430	1155.751546	0.34420514	0.033322394	10.32954402	5.18063E-25	4.71191E-24
SWAP70	2134.7357	0.399827235	0.038693397	10.33321611	4.98603E-25	4.53703E-24
PPP4R1	2835.43735	0.228614861	0.022120502	10.33497621	4.89534E-25	4.45658E-24
ZNF232	83.19249787	-0.512295367	0.049562861	-10.33627503	4.82946E-25	4.39865E-24
TSNARE1	533.6028214	-0.508658404	0.049202531	-10.33805371	4.74068E-25	4.3198E-24
AGAP4	192.5271874	-0.341132041	0.032989209	-10.34071591	4.6108E-25	4.20341E-24
TMF1	2608.083964	0.337775608	0.032663726	10.34100039	4.59713E-25	4.1929E-24
XIAP	1487.638544	0.311750934	0.030142585	10.34254132	4.52379E-25	4.12793E-24
PEX11A	40.39065428	-0.534357991	0.051665612	-10.34262394	4.51989E-25	4.1263E-24
PRSS36	77.35732491	-0.626247485	0.060549218	-10.34278397	4.51235E-25	4.12133E-24
MYB	5624.551178	-0.445432692	0.043060109	-10.34443947	4.43504E-25	4.05262E-24
PKNOX1	511.5858901	-0.313805377	0.030334867	-10.34470907	4.42257E-25	4.04311E-24
ACBD5	1601.758513	0.265077684	0.025622398	10.34554532	4.38413E-25	4.00984E-24
CLUH	2091.38776	-0.372271313	0.03597896	-10.34691704	4.32179E-25	3.95467E-24
BRI3	2307.391079	0.523477625	0.050586522	10.34816402	4.26588E-25	3.90533E-24
HAUS2	965.1524277	0.304429538	0.029416866	10.34880922	4.23723E-25	3.88092E-24
NIPSNAP1	618.9397857	-0.448085475	0.043294051	-10.34981633	4.19289E-25	3.84211E-24
DNAH5	5.319889367	-1.127233448	0.108896916	-10.35138086	4.12493E-25	3.7816E-24
GAS8	60.75460272	-0.518737621	0.050107137	-10.35256952	4.07402E-25	3.73669E-24
RBM6	2887.449138	-0.252026866	0.02434252	-10.35335989	4.04052E-25	3.70769E-24
SYTL4	125.3343245	-1.180301089	0.113998849	-10.35362289	4.02943E-25	3.69925E-24
ZDHHC14	481.4131171	-0.48265947	0.046605982	-10.3561699	3.9236E-25	3.60378E-24

TRAIP	191.0688353	-0.332260754	0.032082901	-10.35631893	3.9175E-25	3.59987E-24
ZC3H15	2968.668876	0.314423003	0.030359337	10.35671507	3.90131E-25	3.58668E-24
B3GNT3	10.25635784	-1.140545841	0.110119045	-10.35738946	3.87391E-25	3.56316E-24
YPEL5	8502.929164	0.390842237	0.037734217	10.35776737	3.85864E-25	3.55078E-24
EXOC3L2	129.9144295	-1.119344136	0.108063868	-10.35817208	3.84235E-25	3.53745E-24
TMC2	3.613924259	-0.836880833	0.080779771	-10.36002977	3.76845E-25	3.47105E-24
RAB33B	774.2525863	0.386260292	0.037282398	10.36039304	3.75416E-25	3.45952E-24
TBX10	13.88672973	-0.970232887	0.093623993	-10.36307951	3.65017E-25	3.36528E-24
AKAP6	45.07880809	-0.845507325	0.081570462	-10.36536146	3.56408E-25	3.28746E-24
DOK5	4.079742306	-2.302945156	0.222159111	-10.36619723	3.53306E-25	3.26038E-24
EXPH5	60.81014642	-0.985920679	0.095102996	-10.36687299	3.50817E-25	3.23894E-24
PPP1CB	5829.530299	0.3798907	0.036644079	10.36704188	3.50198E-25	3.23474E-24
MED16	1499.397096	-0.346920448	0.033461774	-10.36766441	3.47925E-25	3.21526E-24
CBR4	373.0305703	-0.411636927	0.039703243	-10.3678415	3.4728E-25	3.21083E-24
LCN12	14.18022656	-0.577922432	0.055736932	-10.36875213	3.43987E-25	3.18188E-24
RGS6	56.43646015	-1.102849054	0.106358047	-10.36921124	3.42339E-25	3.16813E-24
SOCS6	695.8622248	0.56543432	0.05452516	10.37015428	3.38977E-25	3.1385E-24
PUSL1	136.3307462	-0.370402916	0.035715576	-10.37090697	3.36317E-25	3.11535E-24
FSCN2	42.435167	-0.659909812	0.063616668	-10.37322191	3.28266E-25	3.04221E-24
SLC10A4	17.53961729	-0.972976274	0.093787308	-10.37428512	3.24633E-25	3.00997E-24
IDNK	167.0387977	-0.392063704	0.037788992	-10.37507698	3.21953E-25	2.98653E-24
ZNF697	165.0197176	0.785179283	0.075672053	10.37608007	3.18589E-25	2.95673E-24
SYT14	2.902740475	-1.635030182	0.157574667	-10.37622487	3.18106E-25	2.95365E-24
DENND6A	1550.771567	0.288649751	0.027816644	10.37687182	3.15959E-25	2.9351E-24
C11orf16	11.37716679	-0.622478841	0.059972717	-10.37936693	3.0781E-25	2.86076E-24
PELI1	3984.395393	0.665719458	0.064137006	10.37964654	3.06909E-25	2.85375E-24
CUL9	2459.66457	-0.317518588	0.030583122	-10.38215078	2.98963E-25	2.78118E-24
CCDC136	179.5019119	-0.830217512	0.079963534	-10.3824515	2.98022E-25	2.77375E-24
TSACC	13.23048597	-0.62685416	0.060374873	-10.38269937	2.97249E-25	2.76787E-24
C6orf141	3.945929569	-1.623780324	0.156384395	-10.38326314	2.95499E-25	2.75288E-24
GNL3L	3798.418156	-0.282362481	0.027191527	-10.38420833	2.92587E-25	2.72705E-24
PCDHGA1	7.061980425	-0.938606626	0.090374404	-10.38575731	2.87875E-25	2.68442E-24
THEMIS2	4576.642628	0.555317567	0.053468928	10.38579956	2.87748E-25	2.68442E-24
SLF2	982.3653424	-0.385528425	0.03712033	-10.38591035	2.87414E-25	2.68267E-24
ARL8A	2561.48281	0.446549508	0.042991132	10.3870145	2.84107E-25	2.65307E-24
FOXN2	2256.623586	0.312751514	0.030103915	10.38906459	2.78067E-25	2.5979E-24
IFFO1	891.0069779	-0.414585024	0.039905131	-10.38926609	2.7748E-25	2.59366E-24

TST	319.959175	-0.543548625	0.052301247	-10.39265129	2.67803E-25	2.5044E-24
TRPV5	17.58685045	-0.865249855	0.083255607	-10.39269172	2.6769E-25	2.5044E-24
MYT1	31.07934779	-1.056037482	0.101613354	-10.39270367	2.67656E-25	2.5044E-24
KDM8	134.6256579	-0.366300314	0.035242564	-10.39369074	2.649E-25	2.48081E-24
EFCC1	93.01603107	-1.04771997	0.100797895	-10.39426436	2.63311E-25	2.46711E-24
GPR171	108.3085133	-0.971558518	0.093455732	-10.39592214	2.58772E-25	2.42574E-24
POLH	755.9941232	-0.393424239	0.037832697	-10.39905345	2.50408E-25	2.34846E-24
PRTG	9.214076173	-1.181643492	0.11361774	-10.40016722	2.47498E-25	2.32229E-24
P2RX4	3052.793252	0.408689677	0.039294181	10.40076853	2.45942E-25	2.30878E-24
MIDN	7352.345849	0.458534097	0.044083632	10.4014592	2.44165E-25	2.29321E-24
MRPS14	310.2837123	-0.346950538	0.033342936	-10.40551846	2.33979E-25	2.19859E-24
HEATR4	85.40395904	-0.477155221	0.045851927	-10.40643776	2.31731E-25	2.17852E-24
CCDC154	118.0854887	-0.551039424	0.052944002	-10.40796701	2.28039E-25	2.14484E-24
HIBADH	327.0856282	-0.410333792	0.039422221	-10.4086928	2.26308E-25	2.12958E-24
SGSH	920.78068	0.773423479	0.074304459	10.40884344	2.2595E-25	2.12723E-24
AC093512.2	27.07572909	-0.458150409	0.044002602	-10.41189346	2.18825E-25	2.06115E-24
USP30	930.3659279	0.381021619	0.036593891	10.41216473	2.18203E-25	2.05627E-24
UBAP2	2421.917136	-0.199008602	0.019106007	-10.41602257	2.09534E-25	1.97553E-24
RINL	250.8510132	-0.52913932	0.050794496	-10.41725705	2.06832E-25	1.951E-24
ABHD11	197.3217781	-0.469835293	0.045097199	-10.418281	2.04618E-25	1.93105E-24
TTC5	509.464552	-0.38498384	0.036941772	-10.42136894	1.98081E-25	1.87026E-24
GALNT18	12.50920252	-1.36813539	0.131273778	-10.42200056	1.9677E-25	1.85877E-24
SPATC1	50.42432549	-0.92525676	0.088777724	-10.42217255	1.96414E-25	1.85631E-24
TMCO4	502.6104855	-0.416280162	0.039925367	-10.42645809	1.87757E-25	1.77535E-24
DNAL4	132.6538638	-0.432099661	0.041422538	-10.43151111	1.78034E-25	1.68423E-24
HEXB	1828.326257	0.473650923	0.045398254	10.43324106	1.74821E-25	1.65463E-24
CLDN9	39.54490594	-0.792225143	0.075907379	-10.43673427	1.68508E-25	1.59565E-24
DNAJC25	336.7692016	0.445330611	0.04265258	10.44088322	1.61303E-25	1.52816E-24
CPT1B	24.95452605	-0.560901893	0.053706291	-10.44387691	1.56294E-25	1.48143E-24
PCID2	865.050887	-0.25475803	0.024391631	-10.44448516	1.55296E-25	1.47268E-24
SF3B3	5225.35973	-0.2524009	0.024162985	-10.44576632	1.53213E-25	1.45363E-24
RALYL	2.101201235	-2.364794141	0.226273962	-10.45102196	1.44955E-25	1.37595E-24
ARL6	35.84731303	-0.564250685	0.053989448	-10.45112902	1.44791E-25	1.37506E-24
TREH	5.981044151	-1.218062467	0.11654254	-10.45165536	1.4399E-25	1.36811E-24
EOMES	33.16646859	0.754347412	0.072170989	10.45222502	1.43127E-25	1.36058E-24
TRAK2	2771.697613	-0.749939525	0.071747588	-10.45247018	1.42758E-25	1.35772E-24
OR14L1P	6.579692059	-1.323016574	0.126493881	-10.45913496	1.33065E-25	1.26615E-24

POLD2	1380.166254	-0.460757074	0.044052414	-10.45929239	1.32844E-25	1.26467E-24
DNAH3	41.50072571	-0.722950661	0.069098304	-10.4626398	1.28232E-25	1.22136E-24
SLC25A48	5.291187227	-1.335139259	0.127594976	-10.46388581	1.26557E-25	1.20598E-24
LRTOMT	135.2059262	-0.393980539	0.037650504	-10.46415051	1.26203E-25	1.2032E-24
FMR1	2056.049924	0.361734651	0.034567472	10.46459651	1.25611E-25	1.19814E-24
GABRB2	8.288580825	-1.768185984	0.168962801	-10.46494245	1.25153E-25	1.19435E-24
CFAP221	2.847682932	-1.079649318	0.103154444	-10.46633837	1.23321E-25	1.17745E-24
DOCK4	490.6201757	0.803474534	0.076760315	10.46731674	1.22054E-25	1.16591E-24
TAF11	853.7057126	0.258819637	0.024725994	10.4675121	1.21802E-25	1.16408E-24
DNAAF4	7.542481507	-0.873384156	0.083433011	-10.46808868	1.21063E-25	1.15758E-24
HRC	24.36126785	-1.619947003	0.154749514	-10.46818801	1.20936E-25	1.15693E-24
FAM76B	1826.020898	0.334224604	0.031911075	10.47362423	1.14186E-25	1.09289E-24
SCAI	759.3722759	-0.417729662	0.039883919	-10.47363643	1.14171E-25	1.09289E-24
AGAP5	2.880314408	-0.819530109	0.078232033	-10.47563352	1.11787E-25	1.07097E-24
MAGEF1	160.9952291	-0.687714238	0.0656439	-10.47643783	1.1084E-25	1.06243E-24
LRRC29	119.4887052	-0.330835474	0.031574519	-10.47792594	1.0911E-25	1.04636E-24
CDH17	2.254420629	-1.320428622	0.126006764	-10.4790297	1.07844E-25	1.03473E-24
ABHD14A	81.76157881	-0.655098506	0.062512216	-10.47952781	1.07278E-25	1.0298E-24
CENPM	254.5470549	-0.522040232	0.049814452	-10.47969435	1.07089E-25	1.02849E-24
KCNQ4	32.95499902	-0.705180299	0.067285768	-10.48037819	1.06318E-25	1.02158E-24
PXMP2	45.67326157	-0.492254876	0.046968504	-10.48053127	1.06146E-25	1.02043E-24
ABCB9	163.7881621	-0.500756639	0.047771267	-10.48238138	1.04089E-25	1.00115E-24
TEKT3	4.21713727	-1.004712372	0.095820359	-10.48537474	1.00845E-25	9.70423E-25
DCPS	600.109147	-0.402851859	0.038419768	-10.4855359	1.00673E-25	9.69247E-25
SLC35F6	675.6409282	0.501962932	0.047862744	10.48755029	9.85504E-26	9.49275E-25
POP5	622.4502266	-0.362633364	0.034571317	-10.48942869	9.66108E-26	9.3105E-25
TNIP2	1058.26383	0.346142423	0.032988115	10.49294326	9.30828E-26	8.97492E-25
PI4K2A	715.6874421	0.690410947	0.065794846	10.49338957	9.2644E-26	8.93702E-25
PLCD3	433.0378574	0.61376965	0.058466454	10.49780862	8.84087E-26	8.53266E-25
SPDL1	232.4799648	-0.364325026	0.034704513	-10.49791498	8.83091E-26	8.52725E-25
RAPGEF3	278.435283	-0.58785072	0.055990965	-10.49902813	8.7274E-26	8.43146E-25
CCDC24	99.49957151	-0.507479335	0.04833432	-10.49935803	8.69696E-26	8.4062E-25
UCK2	700.2927064	-0.445394457	0.042411739	-10.50167885	8.48573E-26	8.20609E-25
TMEM108	9.804764989	-1.171528957	0.111470477	-10.50976895	7.78841E-26	7.53547E-25
AK7	22.45567443	-0.90509097	0.086113874	-10.51039662	7.73675E-26	7.48919E-25
LCLAT1	421.1025479	-0.408966775	0.038888963	-10.51626855	7.26958E-26	7.04045E-25
CFL2	704.5847231	0.388966119	0.036982552	10.51755755	7.17084E-26	6.94826E-25

ANKRD49	752.8024174	0.350348484	0.033306919	10.51879	7.07767E-26	6.86137E-25
RBM7	1228.834282	0.337868664	0.032119892	10.51898494	7.06304E-26	6.85059E-25
SNIP1	1356.710926	0.396921884	0.037728766	10.52040475	6.95741E-26	6.75148E-25
EMG1	533.3664445	-0.257790099	0.024503663	-10.52047176	6.95246E-26	6.75002E-25
FBXO15	18.17201754	-0.559960117	0.053190344	-10.52747696	6.45412E-26	6.26931E-25
ASXL3	3.213723398	-1.714083787	0.16280257	-10.52860399	6.37732E-26	6.19778E-25
RCAN3	818.6522075	0.466306842	0.044289068	10.52871201	6.37001E-26	6.19375E-25
DSCC1	140.8410913	-0.427739477	0.040613859	-10.53185994	6.1605E-26	5.99302E-25
SPSB4	14.87976712	-1.18214906	0.112237968	-10.53252367	6.11721E-26	5.95386E-25
MRPL44	838.0287372	0.327024219	0.031044864	10.5339234	6.02689E-26	5.86887E-25
DNPH1	353.5467559	-0.711279021	0.067522591	-10.53394147	6.02574E-26	5.86887E-25
WRNIP1	2092.323297	-0.313683671	0.029776717	-10.53452837	5.98827E-26	5.83707E-25
OPRL1	26.42023866	-0.632187626	0.060008523	-10.53496391	5.96061E-26	5.81301E-25
ZNF688	193.0338053	-0.331081135	0.031417043	-10.53826519	5.75507E-26	5.61535E-25
AQP2	2.252902302	-1.549586696	0.146997653	-10.54157443	5.55608E-26	5.4239E-25
POLR2M	500.1087311	0.305329359	0.028955275	10.54486124	5.3652E-26	5.24017E-25
ZNF157	8.330741605	-0.647207561	0.061366184	-10.54664839	5.26415E-26	5.14405E-25
ESYT3	98.5638738	-0.881530577	0.08358016	-10.54712716	5.2374E-26	5.12047E-25
SPN	12357.82832	-0.502601427	0.047649117	-10.54796936	5.19068E-26	5.07732E-25
ABCB8	609.6878112	-0.361658929	0.034276418	-10.55124644	5.01276E-26	4.90574E-25
RAD51	311.8461464	-0.393464271	0.037278336	-10.55477012	4.8282E-26	4.72748E-25
HABP4	62.29231734	0.680326652	0.064455293	10.55501611	4.81557E-26	4.71747E-25
CDH3	9.965789395	-0.730292991	0.069184267	-10.55576691	4.77722E-26	4.68225E-25
HOXC6	4.873595293	-1.673184199	0.158445426	-10.56000315	4.56646E-26	4.47793E-25
CPA1	2.459817794	-1.067836043	0.101114428	-10.56066942	4.53417E-26	4.44848E-25
CEP72	239.3051182	-0.434138986	0.041101877	-10.56250997	4.44612E-26	4.36428E-25
DRICH1	26.62025888	-0.594095122	0.056244827	-10.56266253	4.43889E-26	4.35938E-25
ZNF41	219.1970064	-0.401996334	0.038050457	-10.56482281	4.33787E-26	4.26231E-25
ZMYND12	27.86220357	-0.567765782	0.053740526	-10.5649465	4.33216E-26	4.25883E-25
GCSAM	77.63203405	-0.690505361	0.065351194	-10.56607107	4.28054E-26	4.2102E-25
UBA7	4487.809782	-0.42107232	0.039838546	-10.56947021	4.1282E-26	4.06241E-25
JAKMIP2	69.76923769	-1.246511682	0.117909667	-10.57175134	4.02899E-26	3.96678E-25
ZNF385D	63.66687308	-1.503352597	0.142186543	-10.57310044	3.97144E-26	3.91208E-25
COPB1	4206.458839	0.288062365	0.027244821	10.57310557	3.97122E-26	3.91208E-25
SLC2A6	439.1280462	0.772597435	0.073033052	10.5787368	3.73966E-26	3.68747E-25
COG8	277.5375329	-0.357020613	0.0337389	-10.58186885	3.61671E-26	3.56804E-25
ATP8B3	439.847832	-0.53442147	0.050496618	-10.58331213	3.56141E-26	3.51526E-25

MADCAM1	14.70627629	-0.470401273	0.044440048	-10.5850757	3.49498E-26	3.45143E-25
OR2AK2	10.28827481	-0.891220056	0.084194704	-10.58522705	3.48934E-26	3.44759E-25
PRPSAP1	602.0047469	-0.351015392	0.0331505	-10.58854001	3.36804E-26	3.32942E-25
PROKR1	2.388492489	-0.972765092	0.091862945	-10.58930879	3.34049E-26	3.30386E-25
TTC9C	252.7317287	-0.335054584	0.031631065	-10.59257996	3.22576E-26	3.19201E-25
C2orf88	349.5114843	-0.830804639	0.078432152	-10.59265391	3.22322E-26	3.1911E-25
SLC25A15	360.7226308	-0.423964089	0.040015185	-10.59508018	3.14072E-26	3.11099E-25
LRRC25	1315.045804	0.769766415	0.072640528	10.59692757	3.07931E-26	3.05171E-25
LSS	941.3820139	-0.384133409	0.036245129	-10.5982078	3.03745E-26	3.01175E-25
FGF2	10.29023755	-1.338277913	0.126249276	-10.6002819	2.97083E-26	2.94719E-25
ZNRF3	90.21462901	-0.601859233	0.056769761	-10.60175739	2.92433E-26	2.90253E-25
MLYCD	746.7343574	-0.500673574	0.047218082	-10.60342877	2.87252E-26	2.85255E-25
MAP3K14	707.935162	0.395605009	0.037306659	10.60413932	2.85077E-26	2.83239E-25
ATP6AP2	3015.294686	0.384207912	0.036229152	10.60493818	2.82651E-26	2.80971E-25
TIMELESS	1047.225449	-0.513312009	0.048402219	-10.60513378	2.8206E-26	2.80526E-25
PLK5	6.334206174	-0.912955822	0.086081927	-10.605662	2.80471E-26	2.79088E-25
HLTF	621.5943519	-0.482318868	0.04547444	-10.60637297	2.78345E-26	2.77114E-25
BPNT1	223.1819538	-0.525287805	0.049524561	-10.60661204	2.77634E-26	2.76546E-25
DNAJC6	433.862226	-1.024449152	0.096574761	-10.60783526	2.74024E-26	2.73089E-25
VILL	669.3554778	-0.433848264	0.040889353	-10.61029904	2.66893E-26	2.66119E-25
CXCR5	23.40238704	0.849439887	0.08000184	10.61775437	2.46418E-26	2.45827E-25
ATXN7L1	707.6933074	-0.416037568	0.039181751	-10.61814648	2.45385E-26	2.44922E-25
SLC25A35	224.2529895	-0.444781861	0.04187537	-10.62156256	2.36568E-26	2.36242E-25
ELOA2	4.01838002	-0.995734715	0.093738939	-10.62242355	2.34395E-26	2.34192E-25
SLCO1C1	3.316793707	-1.691062861	0.159186998	-10.62312177	2.32648E-26	2.32565E-25
EDEM1	7431.365898	0.382153608	0.035965079	10.62568515	2.26344E-26	2.26379E-25
CLCN4	142.480391	-1.036426985	0.097533544	-10.6263644	2.24702E-26	2.24851E-25
TACC1	8807.249145	0.327806676	0.030843698	10.62799527	2.20808E-26	2.21068E-25
TRAPPC13	578.325396	0.344380134	0.032401302	10.62858936	2.19406E-26	2.19776E-25
IKZF5	1742.576911	0.39579971	0.037236897	10.62923462	2.17893E-26	2.18373E-25
PDCD2L	38.91054699	-0.489654546	0.046063404	-10.63001213	2.16084E-26	2.16671E-25
DBNDD1	73.81437807	0.992561298	0.09333156	10.63478744	2.05296E-26	2.05959E-25
PDE6B	253.3057802	-0.463267123	0.043559287	-10.63532372	2.04119E-26	2.04883E-25
SLX4IP	456.5525453	-0.552084821	0.051881504	-10.64126478	1.91512E-26	1.92327E-25
ACSL1	7329.362872	0.63399243	0.059571944	10.64246676	1.89057E-26	1.89959E-25
RSRC1	736.907975	-0.377903147	0.035506997	-10.64306126	1.87854E-26	1.88848E-25
L2HGDH	189.825871	-0.375131943	0.035243564	-10.64398421	1.86002E-26	1.87082E-25

CCL4	183.6239276	1.040406798	0.09773588	10.64508552	1.83815E-26	1.84978E-25
CD300LD	4.41367645	1.1990392	0.112610359	10.64768118	1.78762E-26	1.79985E-25
NR3C1	4823.092332	0.308333494	0.028952223	10.64973455	1.74863E-26	1.7615E-25
D2HGDH	1151.211299	-0.434646794	0.040810096	-10.65047224	1.73483E-26	1.7485E-25
GPC5	13.29538173	-1.534733742	0.144094978	-10.65084822	1.72783E-26	1.74234E-25
ZSCAN5C	2.075563273	-0.968131697	0.090892486	-10.65139421	1.71773E-26	1.73305E-25
MCM7	5016.191411	-0.313091381	0.02939122	-10.65254812	1.69656E-26	1.71258E-25
ACY1	7.150868502	-0.589869563	0.055368434	-10.65353523	1.67866E-26	1.69538E-25
NTAN1	646.11456	0.395859505	0.037155702	10.65407135	1.66902E-26	1.68651E-25
LPIN3	39.03659978	-1.323542397	0.124208579	-10.65580499	1.63821E-26	1.65624E-25
MKNK2	9181.435827	0.476379005	0.044702371	10.65668323	1.62282E-26	1.64152E-25
OR2L8	9.345607496	-1.026107725	0.096275657	-10.65801843	1.59969E-26	1.61897E-25
FANK1	33.99660088	-0.703362131	0.065979327	-10.66034105	1.56024E-26	1.57986E-25
EEFSEC	492.9179326	-0.405392823	0.038027576	-10.66049614	1.55764E-26	1.57804E-25
GIMAP5	58.73261655	0.832695791	0.078084239	10.66406998	1.49891E-26	1.51933E-25
SWSAP1	95.73937573	-0.412279047	0.038657302	-10.66497215	1.48444E-26	1.50543E-25
WDCP	139.7863402	-0.47296672	0.044340941	-10.66659186	1.45879E-26	1.48019E-25
RAB11FIP2	946.4890636	0.311034355	0.029156048	10.66791882	1.43811E-26	1.45997E-25
POLA2	137.9356872	-0.380603149	0.035677151	-10.66798033	1.43716E-26	1.45976E-25
APOBEC3F	432.2613159	-0.39180371	0.036720544	-10.66987758	1.40812E-26	1.431E-25
TEX264	690.6662287	-0.415993911	0.038987396	-10.66995888	1.40689E-26	1.43049E-25
FOXH1	3.970377654	-0.773909059	0.072531269	-10.67000572	1.40618E-26	1.43049E-25
SPATA24	84.37761836	-0.404801117	0.037937234	-10.67028539	1.40195E-26	1.42696E-25
BBS7	133.8570577	-0.504745803	0.047280144	-10.67564008	1.32342E-26	1.34773E-25
TKFC	815.3086961	-0.36371898	0.034058966	-10.67909624	1.27507E-26	1.29916E-25
ZNF501	32.63898816	-0.677179288	0.063408102	-10.67969648	1.26685E-26	1.29146E-25
BMP2K	1298.689336	-0.545670201	0.051079389	-10.68278638	1.22537E-26	1.24983E-25
TTPA	1.762609451	-1.746604278	0.163454354	-10.68557816	1.18906E-26	1.21342E-25
TGIF1	1268.199269	0.495792315	0.046392197	10.68697646	1.17127E-26	1.1959E-25
SARS2	73.24806903	-0.416797009	0.038999995	-10.68710425	1.16966E-26	1.19487E-25
ADGRG6	314.2597582	-1.733943618	0.162209387	-10.68953933	1.13935E-26	1.16452E-25
BORCS8	230.678912	-0.396386338	0.037078725	-10.69039841	1.12885E-26	1.15439E-25
MYLK	222.5687646	-0.878657888	0.082184826	-10.69124225	1.11863E-26	1.14453E-25
TELO2	1300.183455	-0.500984886	0.046858956	-10.69133685	1.11748E-26	1.14396E-25
GTF2H1	653.0228973	-0.350293275	0.032762557	-10.69187836	1.11098E-26	1.1379E-25
ZNF225	100.371148	-0.458968346	0.042919805	-10.6936261	1.09023E-26	1.11723E-25
LRRC56	118.2696056	-0.532805403	0.049820984	-10.69439747	1.0812E-26	1.10856E-25

CERKL	25.93115353	-0.829193593	0.077534778	-10.69447298	1.08032E-26	1.10823E-25
SNAP47	343.5877933	-0.344346735	0.032196146	-10.69527809	1.07098E-26	1.09923E-25
HPF1	161.809319	-0.590448481	0.055205334	-10.69549688	1.06845E-26	1.09721E-25
PDGFRA	14.31433597	-1.11162411	0.103933796	-10.69550184	1.0684E-26	1.09721E-25
BUB1	735.9158256	-0.476835479	0.044581326	-10.69585692	1.06431E-26	1.09411E-25
STARD9	1033.07435	-0.695107391	0.064986873	-10.69611991	1.0613E-26	1.09158E-25
SLC43A1	986.9721073	-0.427604922	0.039977006	-10.69627181	1.05956E-26	1.09037E-25
KIF3C	295.5716049	-0.504717036	0.047183469	-10.69690402	1.05236E-26	1.08352E-25
NCAPD3	1724.052764	-0.338845888	0.031676302	-10.69714172	1.04966E-26	1.08132E-25
MALRD1	58.85676419	-1.353528619	0.126514205	-10.69862958	1.03295E-26	1.06466E-25
NLGN4X	4.479946815	-1.386734649	0.129574967	-10.70218024	9.94112E-27	1.02517E-25
DYNC2I2	587.3500819	-0.493324223	0.046090593	-10.70336038	9.81528E-27	1.01273E-25
FMO5	216.6849589	-0.450131732	0.042054484	-10.70353726	9.79656E-27	1.01133E-25
SLC30A6	445.2246704	-0.419565555	0.039197761	-10.70381422	9.76731E-27	1.00884E-25
SCN9A	143.4964994	-1.519775554	0.141973832	-10.70461743	9.68298E-27	1.00066E-25
KCMF1	2467.53286	0.313501804	0.029284294	10.70545879	9.59542E-27	9.92135E-26
TNNI3	4.565167017	-1.389246248	0.129764584	-10.7058968	9.55014E-27	9.87975E-26
CCDC14	2140.516751	-0.415847803	0.038839845	-10.70673184	9.46442E-27	9.79625E-26
SLC25A41	7.035370284	-0.722153855	0.067434442	-10.70897645	9.23775E-27	9.56669E-26
UNC13C	5.31488826	-1.393242213	0.130027555	-10.71497659	8.65792E-27	8.97096E-26
SLAMF7	174.2076323	0.799644655	0.074626444	10.71529899	8.6278E-27	8.94449E-26
NME6	437.9479254	-0.378772631	0.035347854	-10.71557647	8.60197E-27	8.92243E-26
ZNF512	508.8161052	-0.367635158	0.034307574	-10.71585989	8.57566E-27	8.89986E-26
BBS10	131.3782136	-0.527909312	0.049264052	-10.71591342	8.5707E-27	8.89943E-26
NCAPD2	2670.976558	-0.405358293	0.037827247	-10.71604015	8.55897E-27	8.89197E-26
CCDC83	10.88811824	-1.01726178	0.094901724	-10.71910751	8.27984E-27	8.60655E-26
ATP5MC1	740.2212398	-0.711413366	0.066368589	-10.71912755	8.27805E-27	8.60655E-26
TMPRSS11A	5.034656237	-1.123307028	0.104781527	-10.72046817	8.15893E-27	8.48988E-26
PID1	61.90900823	1.269702422	0.118427304	10.72136557	8.08014E-27	8.41238E-26
FAM47E	19.14275617	-0.724259019	0.067548711	-10.72202575	8.02267E-27	8.35698E-26
COL21A1	3.722103445	-1.550481441	0.144598948	-10.72263291	7.97016E-27	8.30671E-26
TRABD2A	257.3705719	0.690885141	0.06442485	10.72389212	7.86236E-27	8.19872E-26
DAXX	721.4399164	-0.31351874	0.029235281	-10.72398601	7.85438E-27	8.19476E-26
RNF227	94.51221583	-0.430639106	0.04015015	-10.72571591	7.70878E-27	8.04714E-26
KCNIP4	6.141826061	-0.743673492	0.069320763	-10.72800495	7.52022E-27	7.8545E-26
FAM171A2	69.56625451	-0.906773773	0.084519018	-10.7286359	7.46906E-27	7.80523E-26
ANKFN1	19.83986969	-1.341287495	0.124989744	-10.73118047	7.26619E-27	7.59729E-26

ARMC9	130.2191673	-0.641019681	0.059713763	-10.73487336	6.98146E-27	7.30348E-26
GBA2	1632.909952	-0.312631439	0.029119521	-10.73614631	6.8859E-27	7.20737E-26
CCNY	3194.361395	0.294776993	0.027455647	10.73647964	6.86109E-27	7.18524E-26
ZC3H4	1560.98085	-0.351200386	0.032709513	-10.73694941	6.82628E-27	7.15261E-26
PCYOX1L	619.4124901	-0.516490046	0.048094382	-10.73909314	6.66963E-27	6.99221E-26
IGLON5	156.4883983	1.322598575	0.123145361	10.7401413	6.59434E-27	6.91699E-26
CYP4Z1	6.942790724	-1.090417721	0.101515834	-10.74135604	6.50814E-27	6.83023E-26
MCCC2	935.6356972	-0.381866362	0.035547825	-10.74232703	6.44003E-27	6.76238E-26
UBQLNL	27.64818909	-0.644409465	0.059986782	-10.7425244	6.42628E-27	6.75156E-26
SHOX	5.728191917	-0.914392162	0.085096729	-10.74532677	6.23408E-27	6.55315E-26
SMYD3	1421.702982	-0.629696931	0.058601085	-10.74548237	6.22357E-27	6.54562E-26
SHLD3	218.0620625	0.38564728	0.03587441	10.7499268	5.93087E-27	6.24112E-26
PLCB2	5975.622004	-0.515228263	0.047919224	-10.75201602	5.79803E-27	6.10462E-26
ZFAND2B	816.2636177	-0.482412651	0.044866316	-10.75222342	5.785E-27	6.09418E-26
CIT	1349.812515	-0.554046765	0.051506475	-10.75683708	5.50265E-27	5.79986E-26
SPECC1	2898.777788	-0.517858268	0.048140521	-10.75722183	5.47973E-27	5.77881E-26
LINC00514	26.10889128	-0.799648173	0.074329986	-10.75808322	5.42876E-27	5.72814E-26
FNDC5	20.58242353	-0.723808835	0.067272353	-10.7593803	5.35289E-27	5.65114E-26
CAV3	6.402810753	-0.804792953	0.074783721	-10.76160614	5.22514E-27	5.51925E-26
CCL4L2	148.6000817	1.245278536	0.115685586	10.76433616	5.07258E-27	5.36099E-26
LCAT	276.7108011	-0.504545548	0.046858712	-10.76737984	4.90769E-27	5.18953E-26
RMI1	188.1352748	-0.495897831	0.046055261	-10.76745246	4.90382E-27	5.18825E-26
ASPA	8.859990421	-0.871726974	0.080954665	-10.76808822	4.87009E-27	5.15534E-26
POGLUT1	525.4866188	-0.389812764	0.036189305	-10.77149086	4.69339E-27	4.97098E-26
MAPK6	3442.776918	0.352946067	0.032759229	10.773943	4.57001E-27	4.84292E-26
ZBTB21	2312.115613	0.471879432	0.04379473	10.77479947	4.52768E-27	4.80066E-26
CCDC122	36.3790228	-0.590793375	0.054804829	-10.77995107	4.28113E-27	4.54172E-26
ADGRB3	7.341959187	-1.793846803	0.166398104	-10.78045219	4.25787E-27	4.51949E-26
CCDC189	68.08865315	-0.495546172	0.045955333	-10.78321368	4.13193E-27	4.38818E-26
ABCG8	25.17103024	-0.708174644	0.065673302	-10.78329577	4.12824E-27	4.38665E-26
FBXL6	355.483011	-0.39481263	0.036602417	-10.78651787	3.98608E-27	4.23789E-26
MYL3	7.892976846	-0.932483375	0.086412215	-10.79110601	3.79198E-27	4.03372E-26
ST3GAL3	868.121247	-0.634917095	0.058829135	-10.7925622	3.73236E-27	3.97246E-26
ASAH1	5483.659819	0.396548351	0.036739172	10.79361144	3.68998E-27	3.92949E-26
MRPS16	1035.010363	-0.279961035	0.025935723	-10.79441814	3.65772E-27	3.89725E-26
ARHGEF19	178.7207795	-0.442552687	0.040997326	-10.79467188	3.64763E-27	3.88862E-26
CLIC4	2611.489807	0.558184878	0.051708389	10.79486122	3.64012E-27	3.88273E-26

CCDC57	2221.044517	-0.497036	0.046043125	-10.79501003	3.63423E-27	3.87856E-26
SCARB1	1133.513656	-0.523087833	0.048438148	-10.79908826	3.47638E-27	3.71213E-26
ZNF585A	177.5159366	-0.392759315	0.036368776	-10.79935493	3.4663E-27	3.70338E-26
CCER2	42.18086109	-0.517379398	0.04790536	-10.80003148	3.44086E-27	3.6782E-26
CLCF1	173.7408775	0.605072102	0.05600948	10.80303011	3.33028E-27	3.56195E-26
ACP6	305.065122	-0.719500144	0.066596837	-10.80381863	3.3018E-27	3.53341E-26
NUBPL	239.4618303	-0.370267192	0.034271578	-10.80391439	3.29835E-27	3.53166E-26
APOL4	81.63151587	-1.065777067	0.098633417	-10.8054359	3.24412E-27	3.47549E-26
CCDC9B	238.1938226	-0.537874157	0.049764644	-10.80835931	3.1424E-27	3.36836E-26
RNF212B	6.693856515	-0.793193096	0.073373867	-10.81029432	3.07681E-27	3.29986E-26
KCNS2	8.859214838	-0.79899352	0.073910333	-10.8103088	3.07633E-27	3.29986E-26
SUOX	460.220547	-0.34207459	0.031636572	-10.81263131	2.99941E-27	3.22038E-26
ENO3	266.3564297	-0.398837308	0.036884447	-10.81315673	2.98227E-27	3.20374E-26
SMIM5	207.2057153	-0.644107394	0.059564501	-10.81361186	2.96751E-27	3.18963E-26
WDR18	570.5506262	-0.492028984	0.045490159	-10.81616323	2.88608E-27	3.1038E-26
EXOSC4	186.2918391	-0.494544208	0.045703217	-10.82077451	2.74448E-27	2.95315E-26
INTS1	5247.518862	-0.374485903	0.034607116	-10.82106661	2.73575E-27	2.94537E-26
PGAP3	394.4753156	-0.331868519	0.030668501	-10.82115231	2.73319E-27	2.94424E-26
SRPRA	5480.319644	0.245129501	0.022646398	10.82421593	2.64332E-27	2.84899E-26
PSPH	236.885723	-0.454164459	0.041951738	-10.82587937	2.59575E-27	2.79927E-26
CYP4A22	16.67184782	-1.086118624	0.100311083	-10.82750368	2.55013E-27	2.75158E-26
PCYT2	504.125895	-0.368563607	0.034032638	-10.82971021	2.48942E-27	2.68756E-26
GABRB3	2.658267727	-1.327858162	0.122605173	-10.83035999	2.47182E-27	2.67003E-26
DPH7	703.3865226	-0.2659012	0.024551362	-10.8304052	2.4706E-27	2.67003E-26
TMEM91	261.577573	-0.389003417	0.035917393	-10.83050264	2.46797E-27	2.66881E-26
CLUL1	4.469123885	-0.950074946	0.087703125	-10.83285167	2.40545E-27	2.60265E-26
VAPA	7275.659461	0.297394379	0.027450783	10.83373036	2.38247E-27	2.57921E-26
CA13	69.63720063	-0.787071361	0.072649662	-10.83379251	2.38086E-27	2.57889E-26
ACADM	681.470383	-0.471006548	0.043475188	-10.83391632	2.37764E-27	2.57683E-26
THBD	661.3227116	1.03617263	0.095624592	10.83583843	2.32823E-27	2.52468E-26
GDPD4	5.114304599	-1.18778693	0.109606175	-10.83686142	2.30235E-27	2.498E-26
AC005041.1	4.338051254	-0.806740062	0.074433111	-10.83845687	2.26255E-27	2.45618E-26
RIMBP3	21.84874931	-0.968591695	0.089356007	-10.8396931	2.23219E-27	2.42456E-26
MTFR1L	707.1576927	-0.275007149	0.0253701	-10.83981338	2.22925E-27	2.42272E-26
CAPN2	4105.890158	0.601346552	0.055472276	10.84048809	2.21287E-27	2.40625E-26
ZNF281	2971.065965	0.342527527	0.031582808	10.84537921	2.09764E-27	2.28222E-26
MYCBPAP	128.9589698	-0.730531516	0.067350344	-10.84673764	2.06671E-27	2.24981E-26

OIP5	41.0644221	-0.409006832	0.03770685	-10.84701668	2.06041E-27	2.24421E-26
DPH1	636.2163328	-0.286053718	0.026371085	-10.84724862	2.05519E-27	2.23977E-26
SYTL3	654.4020489	0.696159299	0.06417697	10.84749397	2.04968E-27	2.23501E-26
RNF139	2827.052226	0.363469012	0.033502977	10.84885731	2.01934E-27	2.20315E-26
TAF6	491.4211635	-0.340307363	0.031361477	-10.85112669	1.96982E-27	2.15032E-26
FBXO11	3182.168531	0.331113597	0.030510617	10.85240598	1.94244E-27	2.12161E-26
SCN4A	39.80610504	-0.755414554	0.069607703	-10.85245623	1.94137E-27	2.12161E-26
WDR77	552.3412549	-0.272815743	0.02513337	-10.85472183	1.89383E-27	2.07083E-26
MORN1	248.681213	-0.480341904	0.044240937	-10.85740797	1.83895E-27	2.01195E-26
PRKN	54.24476637	-1.11665751	0.102843699	-10.85781161	1.83084E-27	2.0042E-26
SURF6	695.3002768	-0.284634263	0.026213115	-10.85846789	1.81773E-27	1.99096E-26
MARK1	5.873338039	-1.060380868	0.097649946	-10.85900113	1.80715E-27	1.98048E-26
EYA4	4.655136311	-2.074924972	0.190973672	-10.86497916	1.69262E-27	1.856E-26
LSM11	232.3052776	-0.416481882	0.038329138	-10.86593386	1.67501E-27	1.83772E-26
INPP5E	584.3759545	-0.315348834	0.029012762	-10.86931458	1.61409E-27	1.77188E-26
OR13D1	6.082215866	-1.127016193	0.103681137	-10.8700215	1.60163E-27	1.75919E-26
TMED2	4132.71718	0.372295677	0.034246733	10.87098376	1.58483E-27	1.74171E-26
CCDC182	2.053368969	-1.544748293	0.14204659	-10.87494106	1.51754E-27	1.6687E-26
MCOLN3	6.560909359	-1.386317872	0.127471749	-10.87549107	1.50841E-27	1.65959E-26
ANAPC5	4522.15767	-0.281776251	0.025906431	-10.87669122	1.48869E-27	1.63882E-26
ITGB3BP	352.2893669	-0.418191706	0.038447435	-10.8769729	1.4841E-27	1.63468E-26
CTBP1	2254.527185	-0.280439981	0.025780753	-10.87788186	1.46938E-27	1.61938E-26
ANKRD62	4.179747031	-0.915204335	0.084133136	-10.87804849	1.46669E-27	1.61733E-26
AGTPBP1	4094.01164	0.465622529	0.042793299	10.88073469	1.4241E-27	1.57125E-26
KRT17	497.5106026	1.640920209	0.150807666	10.88088062	1.42183E-27	1.56962E-26
NEK5	61.67940005	-0.548914123	0.050443642	-10.88173053	1.40863E-27	1.55593E-26
ABCB11	15.92878124	-0.979433299	0.089994868	-10.88321282	1.3859E-27	1.53169E-26
PCDHGA3	5.747065065	-1.472032847	0.135247692	-10.88397755	1.37432E-27	1.51975E-26
NANOG	11.1667125	-0.796534655	0.073181546	-10.88436501	1.36849E-27	1.51416E-26
DCTPP1	403.5087249	-0.594550044	0.054623044	-10.884601	1.36495E-27	1.5111E-26
SLC52A3	46.96405896	-1.263923538	0.116091442	-10.88731017	1.32496E-27	1.46765E-26
HEYL	39.78092552	-0.760875503	0.069877269	-10.88874126	1.30431E-27	1.44559E-26
SNX11	978.653746	0.306189404	0.028116322	10.89009469	1.28507E-27	1.42508E-26
CPNE1	2740.004677	-0.375051536	0.034438022	-10.89062368	1.27762E-27	1.41763E-26
BTBD1	1892.602545	0.279298774	0.025644382	10.89122636	1.2692E-27	1.40907E-26
FN1	451.6067144	0.931041652	0.085480552	10.89185351	1.26048E-27	1.4002E-26
PKDREJ	59.91788068	-0.629522823	0.057791212	-10.89305455	1.24397E-27	1.38263E-26

KNSTRN	287.9627288	-0.384167416	0.035266348	-10.89331445	1.24042E-27	1.37947E-26
TRIP11	802.9335402	-0.334123815	0.030671826	-10.89350924	1.23777E-27	1.37731E-26
SNX9	1579.560069	0.724918101	0.066541338	10.89425192	1.22771E-27	1.3669E-26
ARX	2.096714927	-1.428410216	0.131087657	-10.89660349	1.1964E-27	1.33279E-26
TNFRSF10D	884.6826381	0.691682581	0.06346673	10.89834908	1.17367E-27	1.30822E-26
ASB7	799.4375103	0.373254135	0.034248508	10.89840578	1.17294E-27	1.30815E-26
MTHFD1	1592.9742	-0.358154696	0.032855853	-10.90078818	1.14263E-27	1.27507E-26
ACY3	391.7316464	-1.090959854	0.100016272	-10.90782362	1.05758E-27	1.18083E-26
GCSH	19.831358	-0.503326285	0.046124552	-10.91232891	1.00644E-27	1.12438E-26
OR1L8	4.537864403	-1.058264133	0.096959633	-10.91448159	9.82884E-28	1.09869E-26
POU3F2	2.371029018	-1.30505136	0.11955916	-10.91552804	9.7163E-28	1.08673E-26
TREM1	2724.816729	0.950647189	0.087076737	10.91734965	9.52343E-28	1.06576E-26
EIF2B4	633.0351545	-0.298147869	0.027302512	-10.92016265	9.23303E-28	1.03386E-26
FRG2C	1.878383325	-1.353648328	0.123957578	-10.92025474	9.22367E-28	1.0334E-26
ANXA2	5246.451869	0.747478612	0.068445135	10.92084357	9.16407E-28	1.02731E-26
TAS2R9	1.465020165	-1.356578904	0.124202355	-10.92232837	9.01545E-28	1.01123E-26
TMEM266	73.94386645	-0.556407343	0.050937524	-10.92332922	8.91663E-28	1.00072E-26
PELP1	1251.142175	-0.326762602	0.029910474	-10.92468838	8.78414E-28	9.86414E-27
CD164L2	6.988501803	-1.139891698	0.10433779	-10.92501291	8.7528E-28	9.83458E-27
GTF3C5	2123.198133	-0.422097101	0.038632859	-10.92585728	8.67176E-28	9.74913E-27
ALDH1A3	12.65092192	-0.93848856	0.085890155	-10.92661387	8.59978E-28	9.67377E-27
CCBE1	11.79633856	-0.82571668	0.075545059	-10.93012161	8.27375E-28	9.31237E-27
GRHL3	7.751889569	-0.918884883	0.084055545	-10.93187708	8.11521E-28	9.13919E-27
HSH2D	4809.495274	-0.656534262	0.060040893	-10.93478511	7.8592E-28	8.85596E-27
NUP54	1757.114542	0.302621829	0.027669658	10.93695593	7.67332E-28	8.65149E-27
SURF4	7497.432672	0.412129184	0.03767725	10.93840929	7.55132E-28	8.51884E-27
RIMKLA	25.42677258	-0.80189951	0.073290368	-10.94140378	7.30598E-28	8.24683E-27
TARBP1	4152.922623	-0.480004865	0.043860828	-10.94381681	7.11405E-28	8.03481E-27
CSMD2	6.903667009	-1.084937231	0.099127402	-10.94487705	7.03131E-28	7.94594E-27
GJB3	6.043408177	-1.09901638	0.100407311	-10.94558126	6.97688E-28	7.88899E-27
EPDR1	233.8939265	-0.698914885	0.063843192	-10.94736747	6.84069E-28	7.73947E-27
SLC29A2	75.86805611	-0.67525286	0.061667702	-10.94986253	6.65485E-28	7.53357E-27
HHIP	51.33053504	-1.103390983	0.100767251	-10.94989664	6.65235E-28	7.53357E-27
ABCA8	12.94336304	-1.042538619	0.095191406	-10.9520246	6.49788E-28	7.36439E-27
C21orf62	25.91013528	-0.830582601	0.075833232	-10.95275225	6.44588E-28	7.30968E-27
LUC7L3	6673.030138	-0.479264643	0.043754016	-10.95361481	6.38477E-28	7.24458E-27
USF3	4468.339264	0.395752474	0.036125837	10.95483197	6.29952E-28	7.152E-27

PIGA	2085.830789	0.506657957	0.046248707	10.95507279	6.28278E-28	7.13714E-27
DLK2	88.37891087	-0.453688527	0.041413053	-10.95520592	6.27355E-28	7.13079E-27
LRSAM1	1195.213319	-0.34627802	0.031598707	-10.95861367	6.04178E-28	6.87134E-27
KRT80	30.62489397	1.319316691	0.120379668	10.95963054	5.97428E-28	6.79852E-27
EME2	633.0400218	-0.385774711	0.035193882	-10.96141399	5.85769E-28	6.66972E-27
PTPRC	16042.38601	0.404868443	0.036931796	10.96259816	5.78153E-28	6.58684E-27
TANK	2513.907833	0.394233921	0.035949965	10.96618384	5.55685E-28	6.33455E-27
C14orf39	1.544238252	-2.099025164	0.191379958	-10.96784213	5.45589E-28	6.22309E-27
TJP1	15.35704606	-1.204494564	0.109800979	-10.96979805	5.33915E-28	6.09348E-27
NMD3	2081.199225	0.337580242	0.030768754	10.97152772	5.23798E-28	5.9815E-27
OR2L3	8.405948818	-1.243964668	0.113360832	-10.97349627	5.12515E-28	5.85607E-27
MYCT1	80.33485447	-1.652280912	0.15055902	-10.97430705	5.07938E-28	5.80717E-27
PCDHB11	11.30151601	-1.291463089	0.117602317	-10.98161262	4.68485E-28	5.35924E-27
SGF29	310.8497796	-0.390078049	0.035520876	-10.98165619	4.68259E-28	5.35924E-27
ANKRD46	184.4969232	-0.383411175	0.034911044	-10.98251826	4.63811E-28	5.31198E-27
CADPS2	5.74040565	-1.499079753	0.136479027	-10.98395688	4.56481E-28	5.23109E-27
RABL2B	364.5888298	-0.426547724	0.038831429	-10.98460015	4.53241E-28	5.19701E-27
PLEKHJ1	666.3386995	-0.407964499	0.037139094	-10.98477246	4.52377E-28	5.19014E-27
MAML2	514.7873657	0.61673854	0.056143749	10.98499024	4.51287E-28	5.18067E-27
ANKRD63	14.79340998	-0.879388349	0.080044815	-10.98619996	4.45281E-28	5.11473E-27
PCDHB2	4.591351021	-1.415145298	0.128803762	-10.98683203	4.42175E-28	5.08203E-27
OXLD1	454.3298074	-0.481753501	0.043840836	-10.98869341	4.33151E-28	4.98124E-27
CHODL	4.823964698	-0.952657243	0.08668802	-10.98949134	4.29339E-28	4.9403E-27
ZXDB	885.3433069	0.380729485	0.034641229	10.99064614	4.23881E-28	4.88036E-27
ZNF70	840.458103	-0.498749001	0.045378826	-10.99078669	4.23221E-28	4.87564E-27
ZNF439	620.699312	-0.60207435	0.054779827	-10.99080415	4.23139E-28	4.87564E-27
RORA	746.1559521	0.778864954	0.070865031	10.99082221	4.23054E-28	4.87564E-27
SLU7	2237.393057	0.349530675	0.031792935	10.99397333	4.08536E-28	4.71479E-27
CALCOCO1	2381.002157	-0.403091597	0.036656047	-10.99659217	3.96847E-28	4.58259E-27
SLC22A12	28.84150472	-1.095902616	0.099652176	-10.99727733	3.93844E-28	4.5506E-27
PPP1R16A	205.9347622	-0.413804131	0.03762496	-10.99812812	3.90146E-28	4.51054E-27
GEMIN8	300.3773292	-0.297467628	0.027040632	-11.00076451	3.78906E-28	4.38317E-27
MRPS34	458.7100759	-0.588382282	0.053484237	-11.00104087	3.77746E-28	4.37234E-27
NIPAL4	106.526201	0.874544806	0.079492634	11.00158291	3.75482E-28	4.34871E-27
PNPLA3	13.38150108	-1.143523952	0.103871581	-11.0090165	3.45757E-28	4.00681E-27
DHRS7B	460.0535293	-0.319682559	0.029035174	-11.01018225	3.41313E-28	3.95765E-27
SLC27A4	324.0266912	-0.574062667	0.052138885	-11.01025982	3.41019E-28	3.95659E-27

PDP1	631.7854521	0.455044347	0.041329003	11.01029096	3.40901E-28	3.95659E-27
VEGFD	1.238711926	-1.734209351	0.15750178	-11.01072856	3.39249E-28	3.94073E-27
NDRG4	79.63462857	-0.840285401	0.076283396	-11.01531186	3.22418E-28	3.74744E-27
MAST4	1352.112582	-0.581852514	0.05282204	-11.01533596	3.22332E-28	3.74744E-27
ALDH18A1	1260.756051	-0.520091673	0.047214201	-11.01557723	3.21469E-28	3.74086E-27
C3orf52	57.89346055	-1.039339145	0.094350476	-11.01572764	3.20933E-28	3.73683E-27
KCNT2	11.16223557	-1.888630001	0.171431361	-11.0168291	3.17031E-28	3.6936E-27
NME7	194.4258107	-0.45404625	0.041213358	-11.01696814	3.16542E-28	3.6901E-27
NAT8	2.887398027	-0.972835501	0.088298256	-11.01760715	3.14303E-28	3.66618E-27
MPDZ	22.05553664	-1.663587525	0.150963838	-11.01977497	3.06825E-28	3.58109E-27
LARP4B	2834.178152	-0.458004418	0.041545615	-11.02413378	2.92319E-28	3.41382E-27
C18orf21	305.0337699	0.364447787	0.033056641	11.02494916	2.89682E-28	3.38504E-27
VPS50	537.3221992	-0.548692428	0.049759663	-11.02685179	2.8362E-28	3.31619E-27
INTS9	593.7979256	-0.290789475	0.026370399	-11.02711718	2.82785E-28	3.3084E-27
PLEKHG4	406.5775869	-0.721571437	0.065433469	-11.027559	2.81399E-28	3.29416E-27
GOLPH3L	390.2522089	-0.448297944	0.040652238	-11.02763256	2.81169E-28	3.29343E-27
TMEM249	3.126523668	-0.905946918	0.08213885	-11.02945702	2.75523E-28	3.22923E-27
FLYWCH2	163.9222968	-0.465976028	0.042214315	-11.03834153	2.49597E-28	2.92713E-27
SLC38A4	43.17905136	-1.023952596	0.092738767	-11.04125735	2.41628E-28	2.83536E-27
OGFOD3	405.7155171	-0.361820034	0.03276743	-11.04206311	2.39471E-28	2.81173E-27
LRRC20	122.0292568	-0.721658904	0.06534395	-11.04400499	2.34349E-28	2.75325E-27
FAM168B	5815.501273	0.380530825	0.034446798	11.04691423	2.2688E-28	2.6671E-27
DIS3	2233.273303	0.310989123	0.028143615	11.05007725	2.19026E-28	2.57632E-27
ZC3HAV1L	286.2382942	-0.569098543	0.051500727	-11.05030121	2.18481E-28	2.57145E-27
CSF3	29.26189065	1.242546468	0.112423256	11.05239714	2.13438E-28	2.51361E-27
PRKACG	2.523522519	-0.995922487	0.090103711	-11.05306841	2.11848E-28	2.49639E-27
TMA16	291.769223	-0.471041287	0.042605093	-11.05598533	2.05073E-28	2.418E-27
SLC22A17	42.34444539	-1.086226681	0.098239537	-11.05691979	2.02948E-28	2.39439E-27
BANF1	1285.340442	-0.657625438	0.059465996	-11.05884846	1.98631E-28	2.34488E-27
DNAH10	123.6080577	-0.669421204	0.060522606	-11.06068048	1.94615E-28	2.29886E-27
ADAMTS16	4.361606479	-0.945845943	0.085492671	-11.063474	1.88646E-28	2.2297E-27
PFKFB1	11.722855	-0.646130801	0.058378768	-11.06790745	1.79544E-28	2.12339E-27
TOB1	2431.949274	0.655604208	0.0592316	11.06848724	1.78386E-28	2.11098E-27
TCN2	373.1078981	0.861984326	0.077862972	11.07052948	1.74367E-28	2.06467E-27
CCDC28B	224.95351	-0.364374054	0.032913313	-11.07071952	1.73998E-28	2.06154E-27
SGSM3	53.66448004	-0.453474566	0.040950703	-11.07366993	1.68361E-28	1.99597E-27
MTRNR2L8	245.1190078	-0.909056357	0.082088526	-11.07409765	1.6756E-28	1.98766E-27

MPHOSPH8	1125.781719	-0.345741125	0.03122061	-11.0741311	1.67497E-28	1.98766E-27
CRX	9.668668393	-0.935240079	0.084441463	-11.07560248	1.64768E-28	1.95692E-27
REXO4	478.5312847	-0.366397212	0.033067781	-11.08018738	1.56545E-28	1.86038E-27
MCM8	197.248833	-0.423971336	0.038261509	-11.08088367	1.55332E-28	1.84709E-27
RAB4B	40.32015996	-0.518762061	0.046814105	-11.08131973	1.54577E-28	1.83923E-27
LRRIQ3	10.53441893	-0.810937966	0.073167451	-11.08331579	1.51168E-28	1.79977E-27
PLAA	1866.621416	0.276110188	0.024907766	11.085305	1.47846E-28	1.76128E-27
EDDM13	120.9931548	-0.584475825	0.052719886	-11.08643951	1.45983E-28	1.74015E-27
LAGE3	282.30553	-0.602082814	0.054305456	-11.08696739	1.45125E-28	1.73097E-27
HNRNPH2	1351.655242	0.367160577	0.033115681	11.08721199	1.44728E-28	1.7273E-27
PIH1D1	1019.043452	-0.301560558	0.027197927	-11.08763041	1.44053E-28	1.72029E-27
TMTC4	719.8946949	-0.426413308	0.038457058	-11.08803755	1.43399E-28	1.71352E-27
NHEJ1	149.4731675	-0.41899479	0.037779362	-11.09057356	1.39391E-28	1.66665E-27
SIRT4	11.32872563	-0.630016899	0.056802768	-11.09130637	1.38254E-28	1.65406E-27
STOX1	26.39878518	-0.74594653	0.067247524	-11.09255005	1.36345E-28	1.63222E-27
TRAPPC9	2600.462584	-0.400328849	0.03608503	-11.09404243	1.34088E-28	1.60619E-27
YDJC	683.3228414	-0.423140944	0.038135709	-11.09566219	1.31681E-28	1.57832E-27
SULT2B1	17.5446642	-0.892077316	0.080398329	-11.09571955	1.31597E-28	1.57827E-27
STK17A	1644.465147	0.456755106	0.041163989	11.09598757	1.31203E-28	1.57451E-27
PHPT1	862.9808541	-0.635146645	0.057239105	-11.09637632	1.30634E-28	1.56864E-27
L3MBTL1	366.9599992	-0.465391374	0.041939645	-11.09669316	1.30171E-28	1.56405E-27
FRRS1L	6.347842968	-0.984038673	0.088676632	-11.09693334	1.29822E-28	1.56082E-27
GOPC	488.3074091	-0.385915335	0.03476849	-11.09957132	1.26047E-28	1.51636E-27
RNF19B	2451.354505	0.521215995	0.046950277	11.10144667	1.2343E-28	1.48579E-27
DOHH	315.0588268	-0.354290869	0.031912466	-11.10195823	1.22725E-28	1.47821E-27
FAM149B1	558.9923849	-0.298539909	0.026886858	-11.10356233	1.20542E-28	1.45281E-27
CAMK4	592.9117319	0.960129853	0.086462204	11.10461922	1.19124E-28	1.43661E-27
DNAJB14	1909.907893	0.401934417	0.036192458	11.10547432	1.17989E-28	1.4238E-27
SEMA4F	57.4762393	-0.705871605	0.063552335	-11.10693416	1.16077E-28	1.40158E-27
PRIM2	464.7938224	-0.388646144	0.03498299	-11.10957469	1.12695E-28	1.36159E-27
PTPN21	12.07300778	-0.862728847	0.077655076	-11.10975473	1.12468E-28	1.35969E-27
PPP4R3A	5554.13529	0.327886324	0.029507506	11.11196337	1.0972E-28	1.32729E-27
RGS22	10.65266189	-1.200592696	0.108007379	-11.1158396	1.05058E-28	1.27167E-27
PAN2	1474.676102	-0.404162583	0.036350203	-11.11857829	1.01883E-28	1.234E-27
ELP4	426.879605	-0.459515844	0.041301217	-11.12596382	9.37867E-29	1.13665E-27
CYP2D6	110.3079171	-0.52767585	0.047393331	-11.13396844	8.57318E-29	1.03967E-27
SSTR1	2.148037655	-2.208624204	0.198363558	-11.13422356	8.54867E-29	1.03734E-27

DPF1	36.54065628	-0.656807182	0.058984539	-11.13524319	8.4514E-29	1.02617E-27
LIMCH1	14.99838069	-1.294687495	0.116258048	-11.13632578	8.34933E-29	1.01441E-27
ALDH1B1	181.1904127	-0.756440676	0.067921686	-11.13695384	8.29067E-29	1.00791E-27
SYNPO2	47.41029688	-0.996310866	0.089431424	-11.14050099	7.96699E-29	9.6916E-28
LRRFIP1	11299.13072	0.324218164	0.029099993	11.14152035	7.87631E-29	9.58726E-28
HHIPL2	10.33514158	-0.744621691	0.066830177	-11.14199783	7.83419E-29	9.54192E-28
INCA1	62.3986032	-0.569223956	0.051063318	-11.14741431	7.37174E-29	8.98426E-28
MYRF	581.5167087	0.902905235	0.080993215	11.14791204	7.33063E-29	8.93972E-28
LINC00634	4.418148922	-0.94525863	0.084788478	-11.14843256	7.28788E-29	8.89313E-28
SAR1A	3766.478328	0.351297252	0.031505267	11.15042915	7.12618E-29	8.70124E-28
TRIM41	1057.091342	-0.255067489	0.02287504	-11.15047179	7.12277E-29	8.70124E-28
MOSMO	104.5279126	-0.488351876	0.043796304	-11.15052722	7.11833E-29	8.70124E-28
ARHGAP33	970.184796	-0.462687762	0.041493473	-11.15085639	7.09205E-29	8.67579E-28
BRAP	1712.585374	0.315956983	0.02832221	11.15580267	6.70846E-29	8.21168E-28
KLF9	2219.760049	0.766353809	0.068678618	11.15855026	6.50436E-29	7.96682E-28
XYLB	124.0374588	-0.738570773	0.066188622	-11.15857613	6.50246E-29	7.96682E-28
ZNRF1	1167.757367	-0.500781232	0.044878394	-11.15862644	6.49879E-29	7.96682E-28
GFI1B	788.9189031	-0.973014928	0.087178509	-11.1611788	6.31486E-29	7.74927E-28
FAM86B1	11.56930328	-0.810301635	0.072592834	-11.16228127	6.23703E-29	7.65855E-28
TSPAN6	22.21430133	-1.274103836	0.114114854	-11.16510071	6.04227E-29	7.42406E-28
OSCP1	110.6287381	-0.515733514	0.04618111	-11.16762926	5.87274E-29	7.2203E-28
GDAP1L1	7.185464316	-0.982636288	0.087973268	-11.16971449	5.7365E-29	7.05723E-28
MMP11	39.70250506	-0.757212338	0.067770722	-11.17314842	5.51893E-29	6.79384E-28
DAND5	12.80478271	-0.879331652	0.07867025	-11.17743563	5.25876E-29	6.47764E-28
FRS2	1340.685018	0.353595435	0.031621265	11.18220395	4.98367E-29	6.14266E-28
TUBG2	154.5699241	-0.681964472	0.060986028	-11.1823067	4.9779E-29	6.13941E-28
ZAN	17.45232391	-0.936475592	0.083741914	-11.18287777	4.94597E-29	6.10387E-28
EPHA10	10.6270754	-0.976738347	0.087332347	-11.18415325	4.87537E-29	6.02054E-28
DNAAF3	11.92403379	-1.039658186	0.092955098	-11.1845204	4.85523E-29	5.99946E-28
OPALIN	12.93988177	-1.628805133	0.14559186	-11.18747387	4.69623E-29	5.80665E-28
TXNIP	30727.04838	0.584576968	0.052241016	11.18999996	4.56434E-29	5.64714E-28
THOP1	1089.570349	-0.359140726	0.032091386	-11.19118759	4.50361E-29	5.57553E-28
CDH6	9.280072535	-1.148469411	0.102614122	-11.19211849	4.45657E-29	5.52078E-28
ANTXRL	12.34133305	-1.148912558	0.102646613	-11.19289302	4.4178E-29	5.47622E-28
LRP5	812.5976901	-0.621737341	0.055540314	-11.19434329	4.34611E-29	5.39077E-28
XKR3	4.971391438	-1.281381982	0.114463746	-11.19465354	4.33092E-29	5.37534E-28
THSD1	80.59991514	-0.792773134	0.070800369	-11.19730234	4.20339E-29	5.22036E-28

FAM189A2	7.102583337	-1.269803722	0.113401798	-11.19738612	4.19942E-29	5.21874E-28
RNASEH2A	4.688916631	-0.817443452	0.072996471	-11.19839688	4.15178E-29	5.16283E-28
CTDSPL2	3274.433253	0.365682405	0.032644737	11.2018793	3.99175E-29	4.96697E-28
MYH14	9.295918559	-1.21964008	0.108866642	-11.20306509	3.93866E-29	4.90403E-28
ATP2B4	5156.801792	-0.538043387	0.048026179	-11.20312705	3.93591E-29	4.90372E-28
ETS1	2259.457361	0.727346408	0.064921542	11.20346777	3.92079E-29	4.888E-28
USP11	1770.502741	-0.33703609	0.030078676	-11.20515028	3.847E-29	4.79907E-28
NPM2	86.95756197	-0.778036016	0.069422988	-11.20718136	3.75976E-29	4.69323E-28
AP002990.1	39.12972197	-0.549982714	0.049061969	-11.20996013	3.64357E-29	4.5511E-28
NFIL3	3554.993801	0.596445281	0.053200156	11.21134457	3.58703E-29	4.48332E-28
SLC25A22	354.8279175	-0.421473577	0.037587458	-11.21314404	3.51482E-29	4.39589E-28
MZB1	585.2147677	-0.871459595	0.077674056	-11.21944238	3.27328E-29	4.09641E-28
SUPT3H	398.7292134	-0.54620887	0.048681976	-11.21994034	3.2549E-29	4.07602E-28
COLQ	105.673035	-0.536812435	0.047844425	-11.21995797	3.25425E-29	4.07602E-28
MCM10	339.6457184	-0.581260145	0.051803094	-11.22056813	3.23187E-29	4.05237E-28
NAA38	1063.721492	-0.685497947	0.061083694	-11.22227385	3.17013E-29	3.9775E-28
CZIB	511.269292	-0.448553147	0.039969231	-11.22246125	3.16342E-29	3.97162E-28
RBMS1	2232.463284	0.322887772	0.028770601	11.22283733	3.14999E-29	3.9573E-28
GPR78	3.683061722	-1.572519939	0.14011222	-11.223289	3.13394E-29	3.93966E-28
AKIRIN1	3533.789683	0.408460165	0.036390036	11.22450565	3.0911E-29	3.88831E-28
UQCC3	134.099475	-0.518650505	0.04620509	-11.22496477	3.07509E-29	3.87066E-28
TXNRD3	115.4657533	-0.73373863	0.065359267	-11.22623709	3.03114E-29	3.8178E-28
IL1RAPL1	5.910685426	-2.316422101	0.206290961	-11.22890739	2.94093E-29	3.70655E-28
DAB1	9.805525147	-1.123665645	0.100054465	-11.23053979	2.88709E-29	3.64105E-28
NUAK2	493.628214	0.771470457	0.068692259	11.23082092	2.87792E-29	3.63182E-28
ABCG5	3.796260227	-1.102619915	0.098176199	-11.23103076	2.87109E-29	3.62554E-28
TMEM80	280.5549632	-0.388210221	0.034551789	-11.23560398	2.72623E-29	3.44484E-28
KCNJ12	25.89049934	-1.189367586	0.105851055	-11.23623741	2.70675E-29	3.42243E-28
SHOC2	3543.741401	0.348148264	0.030969605	11.24161144	2.5469E-29	3.2224E-28
SPRYD4	484.2523629	-0.366385174	0.032588362	-11.2428225	2.5122E-29	3.18054E-28
CYRIB	3868.823572	0.31147163	0.027700326	11.24433101	2.46962E-29	3.12866E-28
TMEM25	158.1516152	-0.654497204	0.058196437	-11.24634491	2.41389E-29	3.06005E-28
RPP25L	150.4082858	-0.520926998	0.046304524	-11.2500239	2.31529E-29	2.93696E-28
ZNF177	3.616959864	-0.915085697	0.081335229	-11.25079138	2.29523E-29	2.9134E-28
GPATCH4	410.727997	-0.500551358	0.044466235	-11.25688635	2.14193E-29	2.72058E-28
SLIT2	27.48006408	-1.202202278	0.106759972	-11.26079611	2.04899E-29	2.60422E-28
MTRNR2L4	11.13968751	-0.755272142	0.067069713	-11.2610015	2.04422E-29	2.59984E-28

PCDH17	7.522746647	-1.502793338	0.133439596	-11.26197459	2.02177E-29	2.57296E-28
SLC35E4	117.9469061	0.852380517	0.075681044	11.26280079	2.0029E-29	2.5506E-28
TM9SF3	5817.67656	0.338211751	0.030025974	11.26397254	1.97644E-29	2.51854E-28
NLRP11	5.876022646	-1.414326508	0.12554115	-11.26584002	1.93498E-29	2.46732E-28
FAM186B	50.62578659	-0.737217736	0.065433417	-11.26668564	1.91649E-29	2.44534E-28
UBE2D3	15184.12636	0.284400245	0.025240351	11.26768176	1.89493E-29	2.41941E-28
CFAP251	91.01009286	-0.658451795	0.058435783	-11.26795531	1.88906E-29	2.41348E-28
RGL2	1789.9189	-0.373833433	0.033161473	-11.27312507	1.78133E-29	2.27733E-28
KIAA1210	12.60292428	-1.287532466	0.114203084	-11.27406043	1.7625E-29	2.25473E-28
CHD1	9642.37593	0.525765727	0.046634413	11.27420056	1.7597E-29	2.25262E-28
RBM3	6935.866969	0.395035844	0.035032531	11.27625754	1.71905E-29	2.20202E-28
LMAN2L	262.1682648	-0.45765207	0.040582521	-11.27707334	1.70318E-29	2.18313E-28
CLEC7A	945.9284369	1.066815167	0.094590726	11.27822163	1.6811E-29	2.15624E-28
LAPTM4B	804.5278354	-1.045443415	0.09267753	-11.2804411	1.63922E-29	2.1039E-28
TTF2	1420.667612	-0.336973557	0.029870508	-11.28114571	1.62614E-29	2.08848E-28
NDUFAF3	1219.613705	-0.388470921	0.034432346	-11.28215094	1.60766E-29	2.06611E-28
TRPM2	1572.011337	-0.712147446	0.063120577	-11.28233429	1.60432E-29	2.06316E-28
GABPB2	771.9905436	-0.403116328	0.03572851	-11.28276361	1.5965E-29	2.05446E-28
OR2M4	3.068701064	-1.206746148	0.106940065	-11.28432217	1.56846E-29	2.0197E-28
BCL9L	1271.647923	0.564604308	0.050028144	11.28573363	1.54348E-29	1.98885E-28
POLR2I	85.53208432	-0.94224477	0.083480996	-11.2869373	1.5225E-29	1.9631E-28
FAM3C	291.805312	0.459933117	0.040748509	11.28711515	1.51942E-29	1.96042E-28
CYBC1	1649.753977	-0.483615442	0.042840237	-11.28881346	1.49035E-29	1.92418E-28
SLC26A1	247.918939	-0.587107893	0.052007877	-11.28882646	1.49013E-29	1.92418E-28
NTPCR	538.0935597	-0.527388503	0.046714922	-11.28950834	1.47861E-29	1.91155E-28
ACSS1	1850.578986	-0.452047385	0.040036041	-11.29101102	1.45355E-29	1.88039E-28
GRK5	397.684485	0.684974313	0.060646553	11.29452992	1.39649E-29	1.80777E-28
AFG1L	213.731044	-0.411164665	0.036400568	-11.29555635	1.38027E-29	1.78796E-28
ESPNL	19.93686033	-1.066677795	0.094425403	-11.29651307	1.36532E-29	1.76976E-28
SLC45A1	7.89029964	-1.046405105	0.092627407	-11.29692753	1.3589E-29	1.7626E-28
TMEM123	8115.024906	0.3523577	0.031188346	11.29773591	1.34645E-29	1.74761E-28
BMF	2314.726491	0.67047511	0.059333145	11.30017815	1.30952E-29	1.70081E-28
ESRP1	2.687585442	-1.33937085	0.118468692	-11.30569461	1.22977E-29	1.59829E-28
TBCCD1	371.5646033	-0.31065829	0.027477025	-11.30611095	1.22395E-29	1.59179E-28
IFNG	19.85743743	0.948541272	0.083889153	11.30707894	1.21053E-29	1.57538E-28
CEP164	896.6734894	-0.372968786	0.032978629	-11.30940855	1.17881E-29	1.53513E-28
SHQ1	510.1806275	-0.416010363	0.036783314	-11.30975745	1.17414E-29	1.53005E-28

IFT80	221.3048106	-0.533078897	0.047121942	-11.31275317	1.13472E-29	1.47967E-28
CATSPER2	172.4857489	-0.573827739	0.050721558	-11.31329084	1.12779E-29	1.47161E-28
DDX56	1369.044912	-0.274332702	0.024240003	-11.31735453	1.07672E-29	1.40592E-28
PRMT8	4.111507942	-1.284979398	0.11346567	-11.32482979	9.88707E-30	1.29185E-28
ZMYM1	314.6945225	-0.494048719	0.043621316	-11.32585544	9.77202E-30	1.27767E-28
VAMP3	2331.136395	0.327210315	0.028885666	11.32777474	9.56028E-30	1.25082E-28
DPF3	71.66653509	-1.016444731	0.089725659	-11.32836182	9.49642E-30	1.2433E-28
IL21R	264.0800007	0.833904155	0.073597898	11.33054309	9.26286E-30	1.21353E-28
TIMM13	902.1449384	-0.64316385	0.056761748	-11.3309381	9.22118E-30	1.20888E-28
MIS18A	169.9635598	-0.491334281	0.043340776	-11.33653637	8.65007E-30	1.13477E-28
GTSE1	307.5161178	-0.532598211	0.046977977	-11.3371892	8.5858E-30	1.12709E-28
IQCG	266.471746	-0.420767454	0.037090024	-11.34449126	7.89843E-30	1.03756E-28
JAK1	7596.52163	0.314275539	0.027694642	11.34788238	7.59806E-30	9.9877E-29
PRR22	33.17825767	-0.499891477	0.044027213	-11.35414753	7.07264E-30	9.30327E-29
CYB5D2	316.1235767	-0.363164653	0.031980617	-11.35577387	6.94224E-30	9.1379E-29
PRICKLE2	42.29630975	-1.428467306	0.12578906	-11.3560536	6.92006E-30	9.11483E-29
DPAGT1	656.7942497	-0.488803354	0.043034871	-11.35830873	6.74375E-30	8.88859E-29
MRPS11	284.002299	-0.502372017	0.044214149	-11.36224545	6.4466E-30	8.50265E-29
GPR37	4.451916739	-2.168355007	0.190773493	-11.36612311	6.16662E-30	8.13886E-29
ENDOG	91.37130953	-0.602473726	0.053004992	-11.36635824	6.15003E-30	8.12244E-29
NUBP2	702.5026301	-0.38707239	0.034054135	-11.36638444	6.14819E-30	8.12244E-29
FEM1B	2792.074541	0.312724384	0.027510871	11.36730229	6.08389E-30	8.04595E-29
F2RL2	102.8955973	-0.915892614	0.080566631	-11.36813838	6.02591E-30	7.97465E-29
PPFIA3	73.28888827	-0.874225058	0.076887096	-11.37024422	5.88228E-30	7.78984E-29
DBT	419.503504	-0.394714285	0.034711577	-11.37125779	5.81436E-30	7.70511E-29
FOXO1	1067.124418	0.72266549	0.063548479	11.37187711	5.77325E-30	7.65581E-29
AMACR	24.94180459	-0.533152921	0.046880677	-11.37255157	5.7288E-30	7.60202E-29
RPA1	1643.598879	-0.401806637	0.035326228	-11.37417323	5.62332E-30	7.46711E-29
SLC4A4	72.10094053	-1.269410838	0.111577418	-11.3769512	5.44709E-30	7.238E-29
PTGDS	20.13558844	1.183118823	0.103907844	11.3862321	4.89711E-30	6.51163E-29
XKR4	8.979253342	-1.001790453	0.087962595	-11.38882335	4.75366E-30	6.32517E-29
LIAS	268.6199415	-0.323957901	0.028444638	-11.38906744	4.74036E-30	6.31177E-29
INTS11	2301.292821	-0.239947283	0.021064499	-11.39107465	4.63241E-30	6.17223E-29
HTR2A	10.98213739	-1.328890571	0.116616295	-11.39541068	4.40747E-30	5.87652E-29
ZNF454	5.171780754	-0.941357427	0.082594896	-11.39728331	4.31371E-30	5.75543E-29
TSC2	2951.361056	-0.31884008	0.027971828	-11.39861452	4.24826E-30	5.67198E-29
HMGCL	413.682458	-0.388791009	0.034106403	-11.39935535	4.21227E-30	5.62776E-29

PRDM15	651.8803672	-0.3953165	0.034678747	-11.39938831	4.21067E-30	5.62776E-29
CENPX	500.0451313	-0.722946747	0.063387495	-11.40519509	3.93893E-30	5.26977E-29
FAM102A	1479.621487	0.759316323	0.066571871	11.40596337	3.90431E-30	5.22702E-29
PPIP5K1	336.201831	-0.445858434	0.039085218	-11.40734169	3.84295E-30	5.14839E-29
DHFR2	69.1386086	-0.478638681	0.041957451	-11.40771594	3.82645E-30	5.1298E-29
ROBO2	52.42653343	-2.390625341	0.209516576	-11.41019668	3.71887E-30	4.989E-29
EIF4H	10389.24419	0.287493069	0.025190072	11.41295163	3.60292E-30	4.83676E-29
HDHD2	80.9576595	-0.523260316	0.045847763	-11.41299555	3.6011E-30	4.83676E-29
FAM229B	48.19807832	-0.771022298	0.067551134	-11.41390599	3.5636E-30	4.79054E-29
SETD6	353.8422984	-0.328866694	0.02880936	-11.41527235	3.50803E-30	4.71909E-29
ANKLE1	446.6936498	-0.590210525	0.051691777	-11.41788031	3.40436E-30	4.58277E-29
NAMPT	11230.01992	0.724436564	0.063444138	11.41849481	3.38038E-30	4.55362E-29
S100A13	30.58765834	-0.620880981	0.054366756	-11.42023233	3.31347E-30	4.46657E-29
ABCB5	13.1382631	-1.354663951	0.118617938	-11.4203971	3.30719E-30	4.46118E-29
CCNF	377.400293	-0.682002474	0.059716857	-11.42060236	3.29939E-30	4.45372E-29
ABCA7	3004.779471	-0.628364957	0.055020182	-11.42062657	3.29847E-30	4.45372E-29
NUP35	164.3294753	-0.456725928	0.039991237	-11.42065028	3.29757E-30	4.45372E-29
UBE3D	162.902513	-0.489277867	0.042829097	-11.42395948	3.17435E-30	4.29382E-29
C8G	20.43895741	-0.485183575	0.042468708	-11.42449583	3.15481E-30	4.27034E-29
TSPEAR	5.335724124	-1.532531747	0.134137294	-11.42509814	3.13301E-30	4.24377E-29
CPEB1	9.297463723	-1.078538423	0.094379089	-11.4277266	3.03963E-30	4.12013E-29
CCL7	20.48269373	2.360350353	0.206530116	11.42860131	3.00917E-30	4.08167E-29
LPCAT4	591.4509404	-0.470565762	0.041172273	-11.42919071	2.98881E-30	4.05687E-29
BTRC	656.9772763	-0.44482542	0.038913149	-11.43123677	2.91921E-30	3.96514E-29
ABI1	4016.157325	0.339646426	0.029710192	11.43198343	2.89421E-30	3.93392E-29
CLP1	464.6065283	0.401214263	0.035092016	11.43320647	2.85373E-30	3.88158E-29
ACTL6A	333.5212742	-0.402643756	0.035209923	-11.43551931	2.77869E-30	3.78215E-29
RAD23B	4511.160423	0.318371342	0.027839816	11.43582797	2.76883E-30	3.77135E-29
MAPK12	663.2598169	-0.650229523	0.056841768	-11.43929103	2.66051E-30	3.62633E-29
STAT4	471.7013055	0.804261443	0.070306341	11.43938706	2.65756E-30	3.62484E-29
C1orf226	64.86210418	-1.442843693	0.126086478	-11.44328649	2.54075E-30	3.46793E-29
PRMT6	221.2541216	-0.490410282	0.042849339	-11.44499065	2.49131E-30	3.40282E-29
TOM1L2	953.1551601	-0.428550152	0.037440915	-11.44603856	2.46139E-30	3.3643E-29
IQANK1	14.11680009	-0.842390797	0.073586424	-11.44763872	2.41638E-30	3.30509E-29
DNASE1L2	32.20559758	-0.642525248	0.056124464	-11.44822061	2.40022E-30	3.28528E-29
EEF1AKMT4	28.51511917	-0.768153152	0.067090738	-11.44946649	2.36597E-30	3.24067E-29
RGS9BP	14.6809199	-1.179115854	0.102981167	-11.4498203	2.35633E-30	3.22973E-29

RUVBL2	1640.000512	-0.49920903	0.043595552	-11.45091647	2.32672E-30	3.19138E-29
CD247	456.9178082	0.823361469	0.071898923	11.45165233	2.30705E-30	3.16662E-29
PLEKHM2	6331.750538	0.44649313	0.038986838	11.45240677	2.28706E-30	3.14138E-29
AOPEP	410.1872914	-0.571397654	0.049892412	-11.45259624	2.28206E-30	3.13672E-29
SMNDC1	1657.141775	0.295826972	0.025824721	11.4551858	2.21488E-30	3.04651E-29
ZNF630	66.57724726	-0.572723927	0.049987348	-11.45737761	2.15955E-30	2.97249E-29
REX1BD	870.9706987	-0.708849913	0.061867294	-11.4575872	2.15433E-30	2.9674E-29
TNFRSF13C	481.6141558	0.737428301	0.064335343	11.46225795	2.04123E-30	2.81359E-29
NXPE1	3.938432656	-1.2445879	0.108559268	-11.46459365	1.9869E-30	2.74063E-29
RASGEF1B	1904.784291	0.664703287	0.057954901	11.46931962	1.88133E-30	2.59684E-29
SNUPN	220.2360245	-0.291729838	0.025433069	-11.47049302	1.85599E-30	2.56367E-29
EDNRA	4.00738511	-1.405712902	0.122545999	-11.4709	1.84728E-30	2.55345E-29
PRDM4	1043.430811	0.315505733	0.027498473	11.47357286	1.79108E-30	2.47752E-29
CD83	4243.958498	0.702221923	0.061174608	11.47897706	1.68259E-30	2.3291E-29
C2orf16	99.69087319	-0.738512402	0.064334591	-11.47924302	1.67743E-30	2.32359E-29
NLRP3	3570.716641	0.754637754	0.065719568	11.4826949	1.61177E-30	2.23422E-29
CEP83	492.5693471	-0.462289784	0.040256231	-11.48368261	1.59346E-30	2.2104E-29
HAS2	5.014081769	-1.177627886	0.102541957	-11.48435162	1.58117E-30	2.19491E-29
C15orf48	32.71766777	1.271419752	0.110672106	11.48816805	1.51286E-30	2.10158E-29
ITPA	605.8044034	-0.54099036	0.047091075	-11.48817191	1.51279E-30	2.10158E-29
CCDC183	25.65141667	-0.43275329	0.037669143	-11.48827009	1.51108E-30	2.10158E-29
TACR1	27.37883081	-0.963308184	0.083848714	-11.48864593	1.50452E-30	2.09445E-29
OR2L2	18.30867996	-1.003193423	0.087304075	-11.49079722	1.46751E-30	2.04439E-29
NCOA3	3987.296594	0.35110631	0.030552211	11.49200994	1.44705E-30	2.01732E-29
LRAT	3.048330752	-1.843061396	0.16037369	-11.49229276	1.44232E-30	2.01216E-29
ADSS1	88.3718103	-0.68710989	0.059783416	-11.49331941	1.42528E-30	1.9898E-29
IGSF11	10.97752451	-1.398586798	0.121619821	-11.49966167	1.32434E-30	1.85019E-29
DNAJA2	2413.696187	0.262760766	0.02284553	11.50162726	1.29452E-30	1.80983E-29
OR4K2	2.723261721	-1.871093921	0.162664185	-11.50280205	1.27701E-30	1.78663E-29
TOM1	3477.576756	0.474371562	0.041235689	11.50390784	1.26075E-30	1.76514E-29
PHF7	460.3192059	-0.355784052	0.030927072	-11.50396805	1.25987E-30	1.76514E-29
TANGO2	716.5160817	-0.524215059	0.04555903	-11.50628231	1.22653E-30	1.71969E-29
PLEKHB2	6544.942185	0.407879205	0.035441585	11.50849209	1.1955E-30	1.67739E-29
PRDM11	528.6108165	-0.59296698	0.051505397	-11.51271536	1.13837E-30	1.59837E-29
ST3GAL5	740.8116739	0.561109366	0.048735601	11.51333644	1.1302E-30	1.58803E-29
TFR2	1205.872243	-0.973369929	0.084541286	-11.51354537	1.12746E-30	1.58533E-29
PPP2R5C	4990.976161	0.345118158	0.02997291	11.5143362	1.11716E-30	1.57198E-29

NIF3L1	254.4080335	-0.395103363	0.034301597	-11.51851222	1.06433E-30	1.49871E-29
NTHL1	213.9299947	-0.701833756	0.06092602	-11.51944212	1.0529E-30	1.48369E-29
ZBBX	4.023376136	-1.236843121	0.107349856	-11.52160952	1.02675E-30	1.44788E-29
OSGEP	1393.355443	-0.264672081	0.022971147	-11.52193571	1.02287E-30	1.44345E-29
THAP12	1589.134445	0.362096187	0.031425343	11.52242575	1.01707E-30	1.4363E-29
RHBDD2	1535.949459	0.643473812	0.055835853	11.52438401	9.94207E-31	1.40503E-29
BIVM	242.5770581	-0.69381335	0.060196654	-11.5257794	9.78229E-31	1.38345E-29
DHTKD1	1197.156842	-0.485236971	0.042100076	-11.52579802	9.78018E-31	1.38345E-29
ATIC	1422.789015	-0.43176693	0.037455128	-11.52757866	9.58003E-31	1.3568E-29
ANAPC1	1481.801554	-0.362775163	0.031463714	-11.52995359	9.31939E-31	1.32084E-29
ARID5A	3556.568395	0.557241458	0.048320147	11.53227976	9.07094E-31	1.28656E-29
ETF1	9754.763233	0.400236673	0.034700871	11.53390869	8.90088E-31	1.26336E-29
RIMS2	11.12802529	-1.227068224	0.106380702	-11.53468816	8.82062E-31	1.25287E-29
GJA5	2.525254471	-2.416060016	0.209323139	-11.54225009	8.0784E-31	1.14828E-29
STK17B	11140.61374	0.490028231	0.04245319	11.54278934	8.0279E-31	1.14193E-29
KIF24	391.0997953	-0.581750228	0.050397975	-11.54312714	7.99643E-31	1.13828E-29
PMVK	409.629878	-0.596230142	0.051650556	-11.54353783	7.95833E-31	1.13369E-29
C10orf143	59.14872257	-0.614166337	0.053174328	-11.5500536	7.37744E-31	1.0517E-29
ABCF2	218.1320914	-0.388830715	0.033660951	-11.55138821	7.26376E-31	1.03625E-29
ARL4C	1368.207847	0.826786493	0.071565015	11.55294235	7.13356E-31	1.01842E-29
OR2L13	7.376703789	-1.299563391	0.112452983	-11.55650435	6.84384E-31	9.7777E-30
OGFRL1	2165.370985	0.512837953	0.044373673	11.55725716	6.78412E-31	9.69946E-30
TENT2	3560.177357	0.326745698	0.028270371	11.55788505	6.73471E-31	9.63585E-30
PAPPA	43.5653535	-1.461633477	0.126459342	-11.55812973	6.71555E-31	9.61547E-30
FOXRED2	416.5840349	-0.64416001	0.05573123	-11.55833112	6.69982E-31	9.59997E-30
SLC16A8	82.54526095	-0.761555947	0.065881263	-11.55952263	6.60751E-31	9.47463E-30
TLNRD1	884.1255084	0.560360567	0.048471358	11.56065342	6.52107E-31	9.35754E-30
RRAGB	439.0816996	-0.414491173	0.035851556	-11.56131606	6.47094E-31	9.29242E-30
PSMD5	1098.434241	0.331277426	0.028653754	11.56139696	6.46484E-31	9.29048E-30
DYNC2I1	563.0506575	-0.412725851	0.03569613	-11.56220172	6.40453E-31	9.21057E-30
RAD51B	363.1128883	-0.575637047	0.049778016	-11.56408185	6.26579E-31	9.01767E-30
CCDC69	2881.394951	0.464337729	0.040152986	11.56421423	6.25614E-31	9.0104E-30
TAMM41	318.9376599	-0.399087581	0.03450413	-11.56637135	6.10087E-31	8.79325E-30
FAM83B	3.667891026	-1.803102453	0.155842403	-11.57003758	5.84571E-31	8.43169E-30
TAS2R4	52.79595346	-0.837821615	0.07240168	-11.57185317	5.72329E-31	8.26121E-30
METTL27	10.13818815	-1.189323329	0.102763446	-11.57340836	5.62047E-31	8.11877E-30
HTRA2	485.5525647	-0.292843775	0.02530297	-11.57349399	5.61486E-31	8.11666E-30

SMARCA4	5592.776438	-0.282719554	0.024427101	-11.57401181	5.58106E-31	8.07377E-30
SLC12A5	29.4344701	-0.978091511	0.084506852	-11.57410889	5.57475E-31	8.0706E-30
ZNF514	506.0550954	-0.520165051	0.044930349	-11.57714239	5.381E-31	7.79588E-30
TMEM221	19.78398587	-0.635066814	0.054851884	-11.57784873	5.33685E-31	7.73765E-30
SOX12	860.1102637	-0.435864327	0.03763735	-11.58063263	5.16634E-31	7.49598E-30
OGG1	386.6180436	-0.540108156	0.046636381	-11.58126227	5.12853E-31	7.44664E-30
ATP13A1	2454.341564	-0.329017116	0.028402879	-11.58393526	4.97105E-31	7.22334E-30
ZBTB18	1386.871668	0.409550772	0.035354808	11.58401928	4.96618E-31	7.22162E-30
KCNK12	84.86357417	-0.830782117	0.071679034	-11.59030857	4.61467E-31	6.71546E-30
STK11	2178.753184	-0.444698756	0.038347985	-11.59640474	4.29756E-31	6.25864E-30
OR2L5	7.22022675	-1.304239849	0.112459793	-11.597388	4.24848E-31	6.19177E-30
SEPTIN7	4842.670104	0.313593967	0.027038917	11.59787447	4.2244E-31	6.16127E-30
SRPX2	20.66163309	-1.305037268	0.11250921	-11.59938166	4.15066E-31	6.05824E-30
ZNF311	14.77030468	-1.021982619	0.088092309	-11.60126955	4.0601E-31	5.93047E-30
ZNF605	268.9372812	-0.475257972	0.040961126	-11.60265891	3.99471E-31	5.83931E-30
NDUFA8	239.677719	-0.729219506	0.062839699	-11.60443984	3.91241E-31	5.72329E-30
SUFU	597.1220814	-0.401256244	0.034574062	-11.60570166	3.85512E-31	5.64371E-30
KANTR	374.9397976	-0.4697482	0.040474746	-11.60595803	3.84359E-31	5.63103E-30
ANKRD23	41.32274702	-0.532550655	0.045885244	-11.60614197	3.83533E-31	5.62314E-30
HTR3E	2.738374932	-1.106714057	0.095348522	-11.60703944	3.7953E-31	5.56862E-30
SGK1	2340.062941	0.919854483	0.079246198	11.60755354	3.77255E-31	5.5394E-30
CACNG1	5.418215373	-1.110094506	0.095625993	-11.60871084	3.72184E-31	5.46904E-30
P2RY14	180.8931891	-1.073916674	0.09250102	-11.60978199	3.67552E-31	5.40502E-30
ALG14	49.74631021	-0.563474874	0.048525793	-11.61186326	3.58713E-31	5.27901E-30
GNRHR	31.33366993	-0.911742958	0.07850381	-11.61399629	3.49873E-31	5.15279E-30
JOSD1	4263.313389	0.402921266	0.034687738	11.61566844	3.43095E-31	5.05677E-30
ZBTB43	4809.611739	0.608790254	0.052408086	11.61634217	3.40401E-31	5.02084E-30
PCDH10	52.4175526	-2.296063663	0.197555425	-11.62237719	3.17186E-31	4.68196E-30
NCK1	733.6284994	0.333011448	0.028652117	11.62257723	3.16444E-31	4.67453E-30
NUMB	3350.04596	0.331370522	0.028510736	11.62265761	3.16146E-31	4.67366E-30
PTGES	156.7460196	1.413380627	0.12160107	11.62309365	3.14536E-31	4.65338E-30
FAHD2B	129.0405266	-0.564030314	0.048517765	-11.62523285	3.06756E-31	4.5417E-30
LOX	68.66828315	-1.028959642	0.088510404	-11.62529591	3.06529E-31	4.5417E-30
RASL10B	18.37292888	-0.806501072	0.069368651	-11.62630476	3.02929E-31	4.49184E-30
MIER1	4420.786576	0.369746644	0.031799706	11.62736041	2.99207E-31	4.44002E-30
TRIM26	1478.848767	0.304685213	0.026202693	11.62801136	2.96935E-31	4.40964E-30
DRAM1	1389.519945	0.546505581	0.046993843	11.62930174	2.9248E-31	4.34678E-30

IFT43	129.0786795	-0.468772869	0.040306171	-11.63030021	2.89079E-31	4.2995E-30
C2orf74	11.97309642	-0.658106683	0.056555393	-11.63649741	2.6883E-31	4.00138E-30
KCTD19	26.67019397	-0.785523137	0.067466105	-11.64322647	2.48436E-31	3.70064E-30
ERMARD	540.3514883	-0.319251431	0.02740937	-11.64752887	2.36209E-31	3.52119E-30
MAPKBP1	1138.692901	-0.488638151	0.041938437	-11.65132008	2.25932E-31	3.37055E-30
ZKSCAN1	2440.135231	-0.437204927	0.037522348	-11.65185403	2.2452E-31	3.35205E-30
PEX6	1018.615276	-0.38521113	0.033058786	-11.65230733	2.23329E-31	3.33681E-30
MIPEP	210.7797309	-0.480209482	0.04120582	-11.65392367	2.19132E-31	3.2766E-30
ARHGEF39	164.3130247	-0.440009027	0.037754974	-11.65433273	2.18082E-31	3.26339E-30
CPB1	33.322364	-1.307805063	0.112214536	-11.65450674	2.17637E-31	3.25923E-30
TRPC4	3.561513189	-2.379078647	0.204101631	-11.65634314	2.12995E-31	3.19215E-30
SLC9A2	9.744839147	-1.14660271	0.098318043	-11.66217991	1.98884E-31	2.98296E-30
TBC1D19	147.3781468	-0.547822984	0.046963972	-11.66474976	1.9297E-31	2.89647E-30
LRRC23	223.5424208	-0.379315242	0.032515046	-11.66583733	1.90519E-31	2.86189E-30
ZNF835	8.958318025	-1.121512158	0.096124222	-11.66732107	1.87226E-31	2.81458E-30
ADGRG5	847.452784	-0.893908651	0.076611913	-11.66801113	1.85714E-31	2.79399E-30
PPARA	721.9303093	-0.438291098	0.037562275	-11.66838533	1.84899E-31	2.78387E-30
RHBDD3	505.3384944	-0.330079701	0.02828019	-11.67176394	1.777E-31	2.67754E-30
TCHH	5.587249128	-1.295193326	0.110926097	-11.67618224	1.68704E-31	2.54394E-30
S100PBP	1442.55809	-0.386401564	0.033090837	-11.6769957	1.67098E-31	2.52167E-30
LARP1	7843.985254	-0.314652699	0.026941625	-11.67905416	1.631E-31	2.46324E-30
CDKN1A	2923.161896	0.808947365	0.069233179	11.68438862	1.53178E-31	2.31517E-30
DNAJC17	279.1326316	-0.332338135	0.028442196	-11.68468618	1.52642E-31	2.30886E-30
NPNT	3.627498451	-1.207549277	0.103335645	-11.68569936	1.50833E-31	2.28326E-30
MSR1	144.8351366	1.315282011	0.112538318	11.68741485	1.47818E-31	2.23935E-30
PRKG2	36.38881129	-1.255112586	0.107382602	-11.68823034	1.46405E-31	2.21967E-30
TWF1	1104.720566	0.380144192	0.032513456	11.69190343	1.40209E-31	2.12737E-30
CEP19	77.36973199	-0.471693635	0.040340896	-11.69269095	1.38914E-31	2.10936E-30
TP53INP2	3239.023135	0.718516782	0.06145006	11.69269459	1.38908E-31	2.10936E-30
CTNNA3	31.33123061	-1.975855775	0.168911452	-11.69758325	1.31135E-31	1.99433E-30
MEGF8	1418.049586	-0.504449573	0.043122351	-11.69809983	1.30339E-31	1.98377E-30
KHDC1	51.15806785	-0.666612435	0.056954963	-11.7042028	1.21294E-31	1.84754E-30
DLX3	8.108154952	-1.79019113	0.152919315	-11.70676924	1.17679E-31	1.79387E-30
DDX10	792.009131	-0.435143465	0.037165172	-11.70836678	1.15483E-31	1.76176E-30
ATG10	313.2765549	-0.352625811	0.03010589	-11.71285125	1.09533E-31	1.6723E-30
DEAF1	1041.591095	-0.382183065	0.032623738	-11.71487675	1.06947E-31	1.63409E-30
P3H1	1330.12716	-0.365983721	0.031232351	-11.71809703	1.02959E-31	1.57438E-30

CHCHD4	109.1340403	-0.609573471	0.052017015	-11.71873236	1.0219E-31	1.56384E-30
NEO1	709.1462406	-1.425744595	0.121654858	-11.71958624	1.01165E-31	1.54937E-30
KLRD1	172.645639	0.832444493	0.07102259	11.72084117	9.96773E-32	1.52779E-30
HOXC8	2.704521748	-1.901381117	0.162197478	-11.72263058	9.75937E-32	1.49702E-30
C2CD6	19.83589316	-0.931504854	0.079459717	-11.7229823	9.71892E-32	1.49199E-30
ABCC10	942.4592911	-0.473648903	0.04039627	-11.72506536	9.48278E-32	1.45688E-30
SLC25A45	401.9140357	-0.429252038	0.036599699	-11.72829428	9.12796E-32	1.40347E-30
CD86	626.2839817	1.026450324	0.087497507	11.73119508	8.82044E-32	1.35725E-30
MIPOL1	10.37376451	-1.406899918	0.119918689	-11.73211558	8.72503E-32	1.34363E-30
RNF170	275.6687636	-0.467576137	0.03983595	-11.7375419	8.18303E-32	1.26115E-30
PPP5C	730.407713	-0.50973824	0.043421483	-11.73930977	8.01378E-32	1.23604E-30
TOR1AIP1	3523.300377	0.320171704	0.027269038	11.74121723	7.83506E-32	1.20943E-30
HIRIP3	290.4408756	-0.623625531	0.053108077	-11.74257419	7.71033E-32	1.19112E-30
USH1C	3.306112375	-2.035903834	0.173352811	-11.74427935	7.55639E-32	1.16826E-30
MTAP	634.2772961	-0.531737952	0.045275998	-11.74436725	7.54854E-32	1.16796E-30
SOS1	1727.255581	-0.426721472	0.036323836	-11.74769853	7.25684E-32	1.12372E-30
NSUN6	556.4222737	-0.433919848	0.03693129	-11.7493823	7.11369E-32	1.10243E-30
TAFA5	15.08331485	2.065028865	0.175685609	11.75411505	6.72617E-32	1.0432E-30
ELOVL6	342.8651787	-0.794211909	0.067545226	-11.75822416	6.40676E-32	9.94446E-31
HPCAL4	50.54881297	0.907211353	0.077138005	11.76088693	6.20787E-32	9.64339E-31
CXCL5	9.789276025	1.695981619	0.144192431	11.76193233	6.13147E-32	9.53227E-31
GABRB1	10.36206294	-1.427225779	0.121342578	-11.76195366	6.12992E-32	9.53227E-31
MAP2K1	1264.321287	0.520658825	0.044259304	11.76382754	5.99534E-32	9.33547E-31
CBFA2T2	1135.684254	-0.377481075	0.032083544	-11.76556662	5.87307E-32	9.15236E-31
PROZ	5.894192016	-0.882219279	0.07498016	-11.76603628	5.84048E-32	9.10882E-31
ABHD18	579.5449919	-0.382507013	0.032509062	-11.76616579	5.83152E-32	9.1021E-31
TADA2B	927.6816245	0.504069306	0.04283425	11.76790304	5.71269E-32	8.92374E-31
DIS3L2	1042.827964	-0.36549692	0.031056358	-11.76882755	5.65043E-32	8.83354E-31
EIF2AK3	1678.607336	0.587498465	0.049891551	11.77551019	5.22003E-32	8.1672E-31
PACRGL	215.6816255	-0.42219772	0.035850779	-11.77652837	5.15738E-32	8.07563E-31
HHAT	212.0630686	-0.674571512	0.057269153	-11.77896779	5.01029E-32	7.85159E-31
RAD54L	362.0666382	-0.430544985	0.036546655	-11.78069476	4.90868E-32	7.69852E-31
MAP3K2	5735.789796	0.455395429	0.038648918	11.78287646	4.78324E-32	7.50781E-31
TPD52L1	8.168301014	-1.232597259	0.104585938	-11.78549704	4.63677E-32	7.28374E-31
FYTTD1	2502.516286	0.353973928	0.030027363	11.78837861	4.48084E-32	7.04446E-31
CALD1	114.8572971	-1.245504224	0.105654294	-11.78848658	4.4751E-32	7.04109E-31
ZNF783	560.9922375	-0.429003398	0.036382448	-11.79149361	4.31814E-32	6.7996E-31

NME2	48.7627379	-0.542083077	0.045970732	-11.79191735	4.29647E-32	6.77091E-31
SLC25A19	199.6979616	-0.548541856	0.046506165	-11.79503527	4.14027E-32	6.53002E-31
HNRNPAB	3107.092905	-0.349078794	0.02959516	-11.79513101	4.13557E-32	6.52786E-31
ANKRD39	114.7149816	-0.44801212	0.037965132	-11.80062051	3.87444E-32	6.12061E-31
COMMD4	674.6941828	-0.468962764	0.039731836	-11.80319898	3.7575E-32	5.94068E-31
CCL2	69.61943184	1.794067698	0.151998185	11.80321793	3.75666E-32	5.94068E-31
RAD50	104.8328729	-0.442483485	0.037476831	-11.80685443	3.59771E-32	5.69724E-31
RIBC2	36.54612878	-0.827803835	0.070098839	-11.80909479	3.50312E-32	5.55196E-31
ATG4C	224.594723	-0.536317066	0.045413513	-11.80963631	3.48064E-32	5.52078E-31
TMPRSS11D	12.63208573	-1.038190334	0.087897329	-11.81139792	3.40847E-32	5.4107E-31
DALRD3	674.4252171	-0.399841738	0.033832464	-11.81828612	3.14026E-32	4.98898E-31
MGAT5B	38.43575615	-1.247332398	0.105470882	-11.82632001	2.85381E-32	4.53758E-31
RPL3L	16.49593758	-0.943781213	0.079801475	-11.8266137	2.84385E-32	4.52541E-31
ACE2	17.26029813	-1.470012749	0.124295103	-11.82679536	2.8377E-32	4.51931E-31
GAD1	213.5993862	-1.467291639	0.124059809	-11.82729241	2.82095E-32	4.49629E-31
DENND5A	10123.18781	0.46131109	0.03900269	11.8276738	2.80816E-32	4.47956E-31
CACNB3	188.0276885	-0.672884876	0.05688913	-11.82800426	2.79713E-32	4.4656E-31
ANO4	3.702505678	-2.045207174	0.172883132	-11.82999842	2.73147E-32	4.36433E-31
DCP1B	229.5377583	-0.456688054	0.038602871	-11.83041684	2.71789E-32	4.34618E-31
ADCY5	12.38470173	-0.999494071	0.084473705	-11.8320142	2.66665E-32	4.26773E-31
GAS2	33.77137964	-0.736529714	0.06219141	-11.84294932	2.34074E-32	3.74921E-31
ZNF382	189.8811378	-0.621011902	0.052426868	-11.84529848	2.27607E-32	3.6486E-31
FLRT2	14.08362767	-1.377026987	0.116239113	-11.84650289	2.2436E-32	3.5995E-31
ATRIP	306.8019561	-0.306055111	0.025832806	-11.8475365	2.2161E-32	3.55831E-31
CELF4	8.69349305	-1.082929776	0.091403737	-11.84776253	2.21013E-32	3.55164E-31
TMEM39A	1767.774305	0.368812089	0.031106423	11.856461	1.99219E-32	3.20403E-31
TBC1D15	2515.637068	0.401565599	0.033863741	11.85827623	1.94947E-32	3.13791E-31
NEPRO	1277.825408	0.30561686	0.025771036	11.85892804	1.93436E-32	3.11615E-31
PNPO	199.2882818	-0.556508778	0.046923161	-11.86000178	1.90971E-32	3.07898E-31
GSTCD	297.5580332	-0.375963481	0.031692288	-11.8629329	1.84401E-32	2.97549E-31
HAVCR2	817.6881868	0.765764113	0.064548885	11.86332062	1.83549E-32	2.96419E-31
APOA1	32.09380911	-1.129926136	0.095221878	-11.86624504	1.77246E-32	2.86477E-31
TAS2R14	49.28404897	-0.765356885	0.064485957	-11.86858223	1.72364E-32	2.78816E-31
VPS45	505.881647	-0.48939322	0.04122806	-11.87039167	1.68676E-32	2.73077E-31
DSE	884.3294292	0.706597232	0.059525837	11.87042918	1.686E-32	2.73077E-31
NOS1AP	77.07600276	-1.401810443	0.118006113	-11.87913416	1.51933E-32	2.46378E-31
GJB5	4.513437818	-1.280422193	0.107769561	-11.88111167	1.48381E-32	2.40817E-31

BRICD5	70.31099421	-0.55362479	0.046586687	-11.88375537	1.43761E-32	2.33512E-31
FSIP2	81.96582952	-1.120134526	0.094233313	-11.88682105	1.38581E-32	2.25287E-31
TBCK	778.1281345	-0.556110445	0.046767813	-11.89087986	1.32009E-32	2.1478E-31
TRIM45	180.3746608	-0.496392193	0.041721213	-11.89783693	1.21455E-32	1.97773E-31
QRFPR	2.326732118	-2.235084756	0.187767091	-11.90349568	1.13491E-32	1.8496E-31
ZC3H7B	2426.87334	-0.361726536	0.030384653	-11.90490944	1.11584E-32	1.82003E-31
TBC1D8B	28.27266091	-1.246379543	0.104651117	-11.90985422	1.05161E-32	1.71669E-31
ERLEC1	1012.209796	0.34976096	0.029366388	11.91024784	1.04666E-32	1.71003E-31
RNF150	56.49930855	-1.489003154	0.124994819	-11.91251897	1.01853E-32	1.66547E-31
NARS2	351.9862734	-0.47511792	0.039880558	-11.91352224	1.00635E-32	1.64692E-31
CFAP52	4.634945892	-0.940187891	0.078897938	-11.91650779	9.70937E-33	1.5903E-31
TAB2	5140.110754	0.431053999	0.036172143	11.9167392	9.68244E-33	1.58722E-31
GRIFIN	15.27389989	-1.0500089	0.088111563	-11.91681164	9.67403E-33	1.58717E-31
C17orf67	112.8180514	-0.552789204	0.046360814	-11.92363035	8.91379E-33	1.46367E-31
MKKS	148.6010476	-0.644076132	0.054006984	-11.92579346	8.68525E-33	1.42734E-31
FAM78B	4.772129956	-1.243113011	0.104209671	-11.92896012	8.36114E-33	1.37523E-31
OSGIN2	1282.454351	0.477839697	0.040056054	11.92927524	8.32955E-33	1.37119E-31
HSD17B8	186.7650326	-0.532847722	0.044664414	-11.93002821	8.25455E-33	1.35999E-31
THADA	1790.657751	-0.48841919	0.040932495	-11.93230933	8.03141E-33	1.32434E-31
FBLL1	2.75639601	-1.172515669	0.098249732	-11.9340343	7.86665E-33	1.29826E-31
ZSWIM9	217.6473043	-0.426496439	0.035737586	-11.93411426	7.8591E-33	1.29811E-31
MRPS22	623.1842393	0.306462973	0.025670298	11.93842661	7.46214E-33	1.23358E-31
SRGAP3	561.1878462	-1.025215418	0.085851989	-11.94166179	7.17747E-33	1.18753E-31
SIPA1L1	1666.070971	0.596843676	0.049968174	11.94447632	6.9386E-33	1.14898E-31
CHTF18	898.954699	-0.483739377	0.040496204	-11.9453017	6.87006E-33	1.13859E-31
CCDC191	176.8791564	-0.483344365	0.040458252	-11.94674352	6.75194E-33	1.11996E-31
GALK2	537.2684405	-0.534250016	0.04470051	-11.95176557	6.35604E-33	1.05519E-31
SEC24A	2413.709547	0.514853322	0.043063268	11.95574201	6.059E-33	1.00673E-31
NR2C2AP	102.943481	-0.43824267	0.036655368	-11.95575685	6.05791E-33	1.00673E-31
RAB35	1742.109008	0.340571655	0.028485407	11.95600448	6.03988E-33	1.00526E-31
RXRB	973.8281042	-0.318276217	0.026612665	-11.95957691	5.78559E-33	9.63757E-32
MYLK2	15.51933171	-0.810128735	0.067732142	-11.96077244	5.70289E-33	9.5079E-32
ENTPD5	569.4327351	-0.385576845	0.032236715	-11.96079829	5.70112E-33	9.5079E-32
NFE2	2369.20512	-0.725721428	0.060669322	-11.96191752	5.62478E-33	9.39368E-32
FDPS	644.881981	-0.342475159	0.028629304	-11.96239904	5.59226E-33	9.34733E-32
CAPS	214.0830918	-0.37473954	0.03132208	-11.96406959	5.48085E-33	9.16894E-32
SEC23A	1616.641269	0.327183258	0.027338256	11.96796392	5.22962E-33	8.75615E-32

BCAT2	265.376223	-0.568671784	0.047492452	-11.97394022	4.86616E-33	8.15456E-32
C17orf50	8.270055355	-0.927739385	0.077465849	-11.97610814	4.74061E-33	7.95098E-32
IKBKB	2073.788322	-0.429873545	0.035894238	-11.97611574	4.74017E-33	7.95098E-32
CYP17A1	2.631759265	-1.153820575	0.096340039	-11.97654248	4.71584E-33	7.92302E-32
TUBAL3	17.15307164	-1.869273172	0.156010068	-11.98174706	4.42891E-33	7.44734E-32
TRAP1	675.2548627	-0.460990473	0.038472828	-11.98223508	4.40291E-33	7.40998E-32
OXTR	23.96063598	-0.694985176	0.057988292	-11.98492232	4.26244E-33	7.17975E-32
ACSBG2	15.80302762	-0.772495648	0.064449583	-11.98604564	4.20505E-33	7.08918E-32
JMJD4	90.61481227	-0.466306641	0.038887825	-11.99107014	3.95759E-33	6.67775E-32
MET	47.62220613	1.554129595	0.129598576	11.9918725	3.91944E-33	6.61908E-32
C3orf38	1173.403751	0.322788679	0.026913606	11.99351288	3.84257E-33	6.49486E-32
PDSS2	343.8330689	-0.479115735	0.039940313	-11.99579329	3.73818E-33	6.3239E-32
EDA2R	66.35070483	-1.153043387	0.096095787	-11.99889634	3.60066E-33	6.09651E-32
CASP8	3800.489571	0.463345217	0.038612989	11.99972407	3.56483E-33	6.04107E-32
P4HTM	826.6248369	-0.403687384	0.033618038	-12.00805917	3.22321E-33	5.46689E-32
SCN1A	3.26537465	-1.611729858	0.134212899	-12.00875527	3.1962E-33	5.42577E-32
KLHL2	1131.027317	0.483076765	0.0402269	12.00879933	3.19449E-33	5.42577E-32
KIF18B	425.1855727	-0.704684121	0.058671772	-12.01061605	3.12508E-33	5.31427E-32
GABPB1	1606.653198	0.494646739	0.041167259	12.0155373	2.9445E-33	5.01153E-32
AC096887.1	22.88507085	-0.602561384	0.050123055	-12.02164123	2.73485E-33	4.65876E-32
ANO2	15.96576728	-1.511150572	0.125637103	-12.02790045	2.53526E-33	4.32253E-32
UROS	991.6669419	-0.487189072	0.040493147	-12.03139559	2.43018E-33	4.14698E-32
PCYT1B	78.23048954	-1.243616459	0.103349417	-12.03312504	2.37979E-33	4.06455E-32
PLA2R1	20.22581121	-0.918896307	0.076348149	-12.03560695	2.30929E-33	3.94758E-32
SDHAF4	86.73384644	-0.597533421	0.049618353	-12.04258874	2.12191E-33	3.63043E-32
FKTN	338.9012439	-0.623012573	0.051731105	-12.04328749	2.10401E-33	3.60295E-32
SLX4	589.6578603	-0.40049999	0.03324905	-12.04545657	2.04939E-33	3.51249E-32
CALB1	11.67002211	-0.975875144	0.080997573	-12.04820222	1.98226E-33	3.40042E-32
MORN4	18.65736293	-0.889365633	0.073811827	-12.04909382	1.96094E-33	3.36679E-32
GULP1	9.385204545	-1.679022088	0.139344392	-12.04944138	1.95269E-33	3.35557E-32
ALDH16A1	800.2224648	-0.424766032	0.035248616	-12.05057325	1.92606E-33	3.31272E-32
TRIM27	1230.96292	-0.393251176	0.032633076	-12.05069278	1.92327E-33	3.31083E-32
CYTH1	7320.67039	0.497187354	0.041256287	12.05118992	1.9117E-33	3.29381E-32
MOB4	755.3376129	0.412554569	0.0342313	12.05196922	1.89371E-33	3.26569E-32
YKT6	2311.239922	0.357982305	0.029700282	12.0531617	1.8665E-33	3.22161E-32
EMILIN2	4189.810588	0.673446855	0.055871075	12.05358684	1.8569E-33	3.20786E-32
ANKS3	546.7215413	-0.461588813	0.03828597	-12.05634373	1.7958E-33	3.10505E-32

GABARAPL1	2811.441525	0.67621111	0.056077507	12.05850867	1.74922E-33	3.02718E-32
PIK3R6	748.4602409	-0.625224596	0.051842087	-12.06017409	1.7142E-33	2.96921E-32
CENPP	326.6966107	-0.394561083	0.032710723	-12.06213287	1.67391E-33	2.90199E-32
TRIM68	210.7764384	-0.461960442	0.038288964	-12.06510685	1.61453E-33	2.80152E-32
MMS19	1985.727756	-0.253270132	0.020987649	-12.06757966	1.56675E-33	2.72103E-32
MBD2	1630.956264	0.407410051	0.033759416	12.068042	1.55797E-33	2.70819E-32
BCKDHB	328.3135175	-0.564072704	0.046730317	-12.07080852	1.50647E-33	2.62099E-32
PPP4R2	2853.632177	0.415965515	0.034458315	12.07155687	1.49283E-33	2.59957E-32
TECTA	51.18669333	-0.957919083	0.079334191	-12.07447978	1.44072E-33	2.51107E-32
MFSD6L	38.64331935	-0.807971187	0.066901783	-12.07697544	1.39767E-33	2.4382E-32
FAAP100	1203.913244	-0.449789949	0.037242971	-12.07717701	1.39425E-33	2.4344E-32
IMMP2L	229.2460605	-0.545801022	0.045188984	-12.07818744	1.37722E-33	2.40682E-32
MYLK4	187.6197895	-0.899939708	0.074467294	-12.08503299	1.26719E-33	2.21651E-32
SFT2D2	7999.151975	0.419949235	0.034732084	12.09110398	1.17694E-33	2.0605E-32
TEAD2	43.89410437	-1.089440697	0.090085686	-12.09338292	1.14474E-33	2.00591E-32
CCS	695.6357087	-0.531292947	0.043924877	-12.0954909	1.11573E-33	1.95683E-32
ZNF81	502.3305172	-0.521073941	0.043048196	-12.10443156	1.0006E-33	1.75648E-32
RTN4IP1	109.0025956	-0.417334583	0.034469089	-12.10750262	9.63842E-34	1.69347E-32
IL6	43.05836918	1.326809689	0.109566299	12.10965143	9.38923E-34	1.65117E-32
TAS2R5	19.56322313	-0.806844923	0.066614157	-12.11221399	9.10041E-34	1.60182E-32
COQ5	301.1978008	-0.417534437	0.034466696	-12.11414164	8.88898E-34	1.56601E-32
SPPL2A	1564.414982	0.353591341	0.029185488	12.11531377	8.76281E-34	1.54518E-32
HINFP	576.1340107	-0.318724553	0.026307403	-12.11539396	8.75425E-34	1.54506E-32
AL365205.1	7.653140631	-0.798797841	0.065916759	-12.11828152	8.45126E-34	1.49293E-32
PDE3A	131.8177493	-1.459882404	0.120458852	-12.11934516	8.3423E-34	1.47501E-32
KAZN	71.53567566	-0.928250838	0.076585607	-12.12043461	8.23214E-34	1.45685E-32
CSPG5	39.39043174	-1.083603209	0.089392468	-12.12186251	8.08995E-34	1.43298E-32
GZMB	97.16138167	1.07299086	0.088508759	12.1229907	7.97933E-34	1.41467E-32
LKAAEAR1	31.61436852	-0.730592488	0.0602628	-12.12344075	7.93562E-34	1.40819E-32
FOXD4	8.182656529	-1.004343737	0.08283742	-12.12427588	7.85514E-34	1.39518E-32
ZNF763	47.71383881	-0.524806522	0.043281952	-12.12529708	7.75784E-34	1.37915E-32
INO80E	615.5487535	-0.450318353	0.037094394	-12.13979548	6.49883E-34	1.15638E-32
PMFBP1	136.4175582	-0.604467944	0.049792134	-12.13982809	6.49624E-34	1.15638E-32
FGF17	29.69644738	-0.872683185	0.071885931	-12.13983278	6.49587E-34	1.15638E-32
ANGEL1	695.7491913	-0.396961072	0.032696581	-12.14075177	6.42332E-34	1.14607E-32
CABIN1	4702.746689	-0.335896886	0.02765855	-12.14441418	6.14209E-34	1.09689E-32
SLC44A4	52.87603597	-1.034403212	0.085164042	-12.14600889	6.0235E-34	1.07669E-32

FER1L5	17.20778179	-0.875279036	0.072060393	-12.14646493	5.99E-34	1.07168E-32
FMC1	27.30436217	-0.756397414	0.062271735	-12.14672128	5.97125E-34	1.06931E-32
ACSM6	25.49495872	-0.971605185	0.079987063	-12.14702918	5.94882E-34	1.06627E-32
C17orf113	91.66117893	-0.62365694	0.051331226	-12.14965987	5.76047E-34	1.03345E-32
TGFBR1	1910.756889	0.469708354	0.038656625	12.15078534	5.68172E-34	1.02026E-32
EXOC3L4	37.20236758	-1.197139421	0.098520829	-12.15113018	5.6578E-34	1.0169E-32
NUDT22	350.7851579	-0.440788361	0.036258992	-12.15666326	5.28746E-34	9.51208E-33
GJA4	22.26713603	-1.277636444	0.105066474	-12.16026767	5.05926E-34	9.10993E-33
NEK9	2061.079541	-0.467702882	0.038459625	-12.16087998	5.02148E-34	9.05023E-33
GPLD1	136.847261	-0.748354332	0.061520002	-12.1644068	4.80926E-34	8.67573E-33
SNX15	21.40730091	-0.659092572	0.054179932	-12.16488371	4.78126E-34	8.63317E-33
PHYKPL	3135.479051	-0.370562063	0.030461427	-12.16496082	4.77674E-34	8.63298E-33
FLVCR2	131.7641247	0.833648317	0.068524497	12.16569767	4.73383E-34	8.56334E-33
PCDHGA2	6.784042761	-1.317106745	0.108232004	-12.16929098	4.53E-34	8.2022E-33
QKI	6066.267847	0.390476092	0.032080991	12.17157195	4.40516E-34	7.98354E-33
TMPRSS12	4.983312689	-1.042696501	0.085652552	-12.17356021	4.29913E-34	7.79861E-33
ZNF233	74.57223526	-0.671726896	0.05517672	-12.17409971	4.2708E-34	7.7544E-33
KCNH1	10.31947527	-1.096222144	0.090016978	-12.1779488	4.07399E-34	7.40392E-33
BHLHE40	4047.730766	0.741386403	0.060848322	12.1841717	3.77469E-34	6.86636E-33
ZBTB3	174.4324622	-0.427093648	0.035048818	-12.18567902	3.70554E-34	6.74684E-33
ENOSF1	1417.754548	-0.483497283	0.039665354	-12.18941043	3.53973E-34	6.45094E-33
KIAA0586	956.5650697	-0.426829647	0.035008741	-12.19208797	3.42531E-34	6.24824E-33
DCAF4	389.7598502	-0.405611567	0.033262259	-12.19434803	3.3316E-34	6.08296E-33
ATG5	896.9982973	0.261271593	0.021419834	12.19764775	3.19933E-34	5.84692E-33
HADH	1049.051271	-0.410332878	0.033634792	-12.19965565	3.12141E-34	5.70985E-33
RAB20	304.7868581	0.859391941	0.070429722	12.20212028	3.02834E-34	5.54478E-33
TGFBR3	176.8024657	0.80565697	0.066002255	12.20650669	2.86947E-34	5.25881E-33
RP2	2098.755794	0.427775726	0.03503564	12.20973068	2.75801E-34	5.05927E-33
ALG8	524.6212867	-0.494658279	0.040507205	-12.2116123	2.69495E-34	4.94824E-33
MGAT4C	3.218896144	-1.615965403	0.132312637	-12.21323556	2.64171E-34	4.85503E-33
PDE11A	7.378177753	-1.22589494	0.100320281	-12.21981165	2.43649E-34	4.48208E-33
DYRK2	1526.183645	0.501858769	0.041064908	12.22111033	2.39787E-34	4.4152E-33
DHX16	786.2679117	-0.285479011	0.023356309	-12.22277949	2.34913E-34	4.32953E-33
ACBD3	1266.90324	0.404391148	0.033075849	12.22617574	2.25298E-34	4.15623E-33
STX12	1030.726251	0.418487521	0.034217849	12.23009446	2.14689E-34	3.96425E-33
ATP13A3	6015.37237	0.546792872	0.044705072	12.23111481	2.12009E-34	3.91846E-33
TRMT2A	906.516277	-0.341349634	0.027903722	-12.23312201	2.06833E-34	3.82642E-33

RBPJ	4510.686954	0.42324676	0.034595096	12.23429945	2.03856E-34	3.7749E-33
SPATA17	4.815935421	-1.061318951	0.086727814	-12.23735394	1.96329E-34	3.63897E-33
RBM27	907.2498676	0.427076663	0.03489681	12.23827234	1.94121E-34	3.60145E-33
ZNF491	58.00442229	-0.600664421	0.04907046	-12.2408557	1.8804E-34	3.49194E-33
POLR1B	698.1308589	-0.435150459	0.035528339	-12.24798207	1.72228E-34	3.20135E-33
PHLDB1	255.9709974	-1.027744271	0.083887882	-12.25140329	1.65114E-34	3.07203E-33
SPICE1	106.9373556	-0.586635646	0.047876901	-12.25299952	1.61895E-34	3.01501E-33
SCAP	2852.371279	-0.352365001	0.028752566	-12.25508033	1.57793E-34	2.94141E-33
KRBA2	483.4949835	-0.662929466	0.054079099	-12.25851529	1.51246E-34	2.82205E-33
NBPF1	424.8540413	-0.560923452	0.045754447	-12.25943036	1.49547E-34	2.79303E-33
EMP1	2467.338219	1.164830719	0.094954419	12.26726187	1.35767E-34	2.53808E-33
AL358113.1	80.26449723	-0.486930242	0.039680295	-12.27133617	1.29105E-34	2.41584E-33
DYNLL1	1123.726995	-0.715382429	0.058287523	-12.27333732	1.25953E-34	2.35911E-33
NDRG1	7732.180761	0.611715512	0.049821634	12.27811019	1.1874E-34	2.22614E-33
UNG	513.8034441	-0.650442075	0.052962743	-12.28112516	1.14396E-34	2.14676E-33
BRCA1	773.8499085	-0.64581589	0.05255825	-12.28762167	1.05566E-34	1.98297E-33
BIRC3	1384.492363	0.680542083	0.055381846	12.28817986	1.0484E-34	1.97121E-33
GBGT1	351.8123117	-0.673802078	0.054815676	-12.29214201	9.98253E-35	1.87873E-33
MAPKAPK2	9329.594598	0.478535477	0.038893146	12.30385104	8.63565E-35	1.62681E-33
HDAC8	486.7537332	-0.534323366	0.04342003	-12.30591873	8.41731E-35	1.58721E-33
LANCL3	107.0710596	-1.038810635	0.084379155	-12.31122347	7.88192E-35	1.48768E-33
USP5	1497.562247	-0.401537129	0.032614094	-12.31176727	7.82899E-35	1.47912E-33
TMEM223	255.2437649	-0.414812268	0.033691233	-12.31217219	7.7898E-35	1.47314E-33
USP40	648.6986854	-0.528696594	0.042939509	-12.3125907	7.7495E-35	1.46693E-33
GINS1	200.6005665	-0.686668894	0.0557387	-12.31942791	7.11976E-35	1.34903E-33
NR2E3	17.38071397	-0.988548555	0.080242933	-12.31944686	7.11808E-35	1.34903E-33
RBP1	8.769181141	-0.846300495	0.068651134	-12.32755303	6.43719E-35	1.22207E-33
MKS1	266.1416313	-0.521344213	0.042285599	-12.3291197	6.31326E-35	1.1997E-33
TSPAN10	17.49622573	-0.715519833	0.058034716	-12.32916923	6.30939E-35	1.1997E-33
RNASEL	227.3122003	-0.701659054	0.056890176	-12.3335715	5.9739E-35	1.13742E-33
APOL6	3139.044835	0.524687005	0.042532046	12.3362748	5.77673E-35	1.10095E-33
KRTAP5-8	8.124465475	-0.859910472	0.069697288	-12.33778958	5.66909E-35	1.08149E-33
MAPK7	769.6094309	0.736375408	0.059679803	12.33877079	5.60044E-35	1.06944E-33
CXCL17	11.15714205	-0.956602861	0.077509525	-12.34174593	5.39727E-35	1.03165E-33
IZUMO2	5.140939447	-1.326859596	0.107499665	-12.34291845	5.31922E-35	1.01772E-33
FOSL2	16580.8423	0.608290136	0.049256637	12.34940452	4.90733E-35	9.39836E-34
NUDT18	299.8178366	-0.49291398	0.03991113	-12.35028869	4.85369E-35	9.30474E-34

PEX2	983.1345739	0.379401224	0.030717077	12.35147534	4.78262E-35	9.17748E-34
SLC25A11	940.4282676	-0.397696904	0.032181988	-12.35774811	4.42376E-35	8.49718E-34
CDC42SE1	6672.111978	0.380569717	0.030793691	12.35869108	4.37218E-35	8.40635E-34
TRHDE	13.42313488	-2.331989359	0.188626739	-12.36298401	4.1448E-35	7.97701E-34
PATL1	3399.985511	0.432985158	0.034975463	12.3796835	3.36668E-35	6.48583E-34
GINM1	960.8300236	0.370340111	0.029909866	12.38187119	3.27614E-35	6.31763E-34
CDCA3	191.2888229	-0.625589133	0.050505236	-12.38661932	3.08788E-35	5.96047E-34
FLG2	2.792890514	-1.561778809	0.126071315	-12.38805839	3.03298E-35	5.86027E-34
LITAF	4134.101671	0.626707838	0.050586913	12.38873462	3.00751E-35	5.81681E-34
NREP	392.5127339	-0.576353583	0.046521596	-12.38894687	2.99957E-35	5.80717E-34
PCNT	3528.705041	-0.42784174	0.034532817	-12.3894247	2.98175E-35	5.77839E-34
SOD2	7599.733378	0.711891591	0.057438475	12.39398491	2.8169E-35	5.46434E-34
AZIN1	7208.507056	0.365266714	0.029471193	12.39402522	2.81549E-35	5.46434E-34
PLP1	5.918245898	-1.640225648	0.132313525	-12.39650785	2.72962E-35	5.30555E-34
ZBTB8B	12.28856129	-0.879886907	0.070971898	-12.39768037	2.68998E-35	5.2337E-34
ZNF623	517.6592969	-0.550007716	0.044361695	-12.39825737	2.67069E-35	5.20133E-34
TMLHE	411.9108029	-0.387745845	0.031267987	-12.40072936	2.58956E-35	5.04835E-34
SPOPL	1795.039402	0.429813214	0.034655658	12.40239663	2.53623E-35	4.94931E-34
LCT	52.32110856	-0.950021681	0.076521487	-12.41509705	2.16424E-35	4.22762E-34
KIAA1328	376.4610387	-0.409830832	0.033006244	-12.41676686	2.11955E-35	4.14446E-34
ZNF470	77.71687296	-0.587048911	0.047276817	-12.417268	2.10632E-35	4.1227E-34
MAGT1	2315.865847	0.320703926	0.025824468	12.4186071	2.07136E-35	4.05834E-34
NELL2	327.8857457	1.050045242	0.084513122	12.42464145	1.92086E-35	3.76723E-34
PSKH1	705.3045036	-0.384138855	0.030904693	-12.42979055	1.80106E-35	3.53581E-34
UBXN8	174.3975399	-0.453676309	0.03648774	-12.43366418	1.71585E-35	3.37192E-34
RHOQ	1352.388178	0.396503368	0.031887238	12.43454737	1.69699E-35	3.33822E-34
ITK	750.5884348	1.01425292	0.081540056	12.43870773	1.61089E-35	3.17203E-34
ARV1	149.7966682	-0.610469058	0.049074189	-12.4397176	1.59065E-35	3.13533E-34
FAM199X	2041.445073	0.403353357	0.032421871	12.44077987	1.56964E-35	3.09703E-34
HIKESHI	397.3274552	-0.572417562	0.046010232	-12.44109261	1.5635E-35	3.08804E-34
AP4M1	296.8220456	-0.334747319	0.026900375	-12.44396493	1.50827E-35	2.98196E-34
COG4	1116.96099	-0.448572298	0.036039795	-12.44658286	1.45962E-35	2.88868E-34
DDX11	1118.226885	-0.479888003	0.038550239	-12.44837943	1.42714E-35	2.82726E-34
CTSS	8048.374261	0.779967022	0.062648594	12.44987266	1.40068E-35	2.77767E-34
CCDC15	163.3511631	-0.681136798	0.0547074	-12.45054233	1.38898E-35	2.75725E-34
SIGLEC8	17.27615075	-1.30288797	0.104592503	-12.45680077	1.2842E-35	2.55184E-34
SPATA33	127.609191	-0.535837044	0.042983977	-12.46597181	1.14468E-35	2.27691E-34

PTGR2	72.11530275	-0.538398386	0.043185724	-12.46704543	1.12936E-35	2.24873E-34
RITA1	180.2545751	-0.480750501	0.038553641	-12.46965249	1.09302E-35	2.17858E-34
PSAP	29520.93479	0.643052466	0.0515614	12.4715867	1.0668E-35	2.1285E-34
KRT16	12.83726313	2.182747925	0.175000628	12.4728005	1.05067E-35	2.09845E-34
RAB9B	24.86199265	-1.064272028	0.085314563	-12.47468185	1.02615E-35	2.05157E-34
C11orf21	1466.561001	-0.788116679	0.06315439	-12.47920653	9.69479E-36	1.94025E-34
ZBTB12	126.7718642	-0.623403287	0.049953091	-12.47977404	9.62594E-36	1.92844E-34
CEP41	411.3942046	-0.669081148	0.053608977	-12.48076689	9.50666E-36	1.90649E-34
CCDC114	48.25414368	-0.689592992	0.055226678	-12.48659193	8.83582E-36	1.77378E-34
RAB14	4403.851233	0.361874379	0.028980129	12.48698303	8.7925E-36	1.7669E-34
USP16	2432.548089	0.412408904	0.033019934	12.48969515	8.49786E-36	1.70944E-34
PHC2	3063.470346	0.460900049	0.036901407	12.49004002	8.46111E-36	1.7038E-34
AC112128.1	25.64150991	-0.659376935	0.052781062	-12.49268022	8.1849E-36	1.64988E-34
LGALS8	2747.743507	0.323962018	0.025931987	12.49275703	8.177E-36	1.64988E-34
CTSL	708.7588635	1.284397299	0.102803691	12.49368858	8.0818E-36	1.63246E-34
CYLD	8130.176776	0.50124614	0.040117546	12.49443686	8.00612E-36	1.61884E-34
ATXN1L	4571.490212	0.435956116	0.034884005	12.49730691	7.72232E-36	1.56307E-34
ACSF3	1491.659848	-0.4106559	0.032858192	-12.49782393	7.67227E-36	1.55455E-34
MSX1	17.71850698	-1.456524032	0.116536252	-12.49846295	7.61086E-36	1.5437E-34
EMX2	14.98004676	-1.918799927	0.153507595	-12.49970679	7.49271E-36	1.52132E-34
DDX31	509.2404111	-0.452665136	0.03621111	-12.50072508	7.39735E-36	1.50351E-34
AL139353.1	8.226322467	-0.673505697	0.053868446	-12.50278676	7.20795E-36	1.46654E-34
THOC3	158.142485	-0.452462824	0.036183075	-12.50481963	7.02591E-36	1.43099E-34
PDCD4	3725.283821	0.368747872	0.029486614	12.50560254	6.95703E-36	1.41844E-34
PIGN	941.3703972	-0.505114999	0.040377596	-12.50978392	6.60035E-36	1.34712E-34
LHX4	124.5340901	-0.65033409	0.051985331	-12.50995371	6.58626E-36	1.34565E-34
MALT1	1481.378681	0.595954803	0.047621132	12.51450311	6.21959E-36	1.27206E-34
CYP2C8	22.06544779	-1.006258	0.080400893	-12.51550769	6.14139E-36	1.25738E-34
DMRTA2	9.004213231	-1.377386719	0.110045488	-12.51652152	6.06347E-36	1.24273E-34
STXBP6	33.0135056	-1.764726775	0.140960531	-12.5192972	5.85513E-36	1.20128E-34
GAB3	1310.528152	-0.510982853	0.040798926	-12.52441917	5.48917E-36	1.12738E-34
DFFB	365.9736701	-0.552678348	0.044117988	-12.52727913	5.29482E-36	1.08861E-34
MMP19	186.9543535	1.012718492	0.080835065	12.52820782	5.23319E-36	1.07707E-34
ATPSCKMT	158.5267315	-0.710078841	0.056667433	-12.53063371	5.07555E-36	1.04572E-34
IL7R	1001.625667	0.999484687	0.079693153	12.54166317	4.41628E-36	9.10853E-35
GTF3C2	981.989812	-0.257337083	0.020517533	-12.5423013	4.38086E-36	9.045E-35
FOLR1	6.006509783	-1.625214732	0.12956594	-12.54353369	4.31324E-36	8.9148E-35

ZBED8	34.47886935	-0.706867272	0.056348488	-12.54456494	4.25746E-36	8.8088E-35
TROAP	363.3953939	-0.723863898	0.057699286	-12.54545669	4.2098E-36	8.71941E-35
C10orf88	111.926005	-0.47792782	0.038091076	-12.54697622	4.12981E-36	8.5628E-35
PPP1R13L	84.87192073	-0.607157469	0.048376958	-12.55055072	3.94755E-36	8.19357E-35
MSS51	138.9529031	-0.643236503	0.051241434	-12.55305426	3.82468E-36	7.94696E-35
ACBD4	267.6325454	-0.464338034	0.03698938	-12.55327982	3.8138E-36	7.93278E-35
NPRL2	630.3694018	-0.373390682	0.029732176	-12.55847151	3.57166E-36	7.43703E-35
ZNF267	1747.304053	0.499364573	0.03975766	12.5602104	3.49402E-36	7.28312E-35
NR1I2	96.43786317	-0.83262212	0.066284935	-12.56125717	3.4481E-36	7.19506E-35
SMOC1	7.781006423	-1.343331338	0.106893354	-12.56702395	3.20565E-36	6.69629E-35
MYO18A	6610.958179	-0.477518489	0.037997248	-12.56718608	3.19909E-36	6.68971E-35
CDK4	770.2158755	-0.518477141	0.041249471	-12.56930399	3.11454E-36	6.51987E-35
GRM7	4.833817071	-1.316326435	0.104692271	-12.57329146	2.96133E-36	6.2058E-35
DNMT3A	2355.159078	-0.446631729	0.035520328	-12.5739755	2.93581E-36	6.15892E-35
ZNF800	3076.353423	0.382268801	0.030396144	12.5762266	2.85337E-36	5.99238E-35
SELENOT	2114.773684	0.368428479	0.029293766	12.57702677	2.82462E-36	5.93838E-35
HRNR	29.54276508	-1.466563446	0.116593554	-12.57842647	2.77502E-36	5.84038E-35
APOLD1	190.645362	-0.679474123	0.054010649	-12.58037312	2.70748E-36	5.70436E-35
EYA3	812.5539479	-0.456953962	0.036320243	-12.58124736	2.67768E-36	5.64766E-35
EXOC7	2337.919846	-0.289659787	0.023019758	-12.58309419	2.6158E-36	5.52309E-35
TMEM161A	352.1841508	-0.543175143	0.043159874	-12.58518822	2.54735E-36	5.38437E-35
P3H4	67.13464344	-0.842627903	0.066941395	-12.58754621	2.4724E-36	5.2316E-35
GMEB2	1521.5354	0.453185847	0.036001852	12.5878483	2.46296E-36	5.21726E-35
PARP1	4262.358585	-0.370139954	0.02940187	-12.5889938	2.42748E-36	5.14768E-35
ZNF33B	661.7108457	-0.459662993	0.036512451	-12.58921207	2.42078E-36	5.13903E-35
CTLA4	40.29986651	1.10903753	0.088087762	12.5901431	2.39239E-36	5.08429E-35
CCR7	760.9308534	1.033748823	0.082093089	12.59239773	2.32502E-36	4.94648E-35
TAMALIN	2392.483464	0.950917571	0.075504026	12.5942631	2.27071E-36	4.83618E-35
POC1A	235.1988125	-0.561929454	0.044616832	-12.5945618	2.26213E-36	4.82316E-35
CPSF1	4629.975543	-0.442247352	0.03510523	-12.59776255	2.17219E-36	4.63646E-35
CHMP2B	1240.43477	0.473705508	0.037601079	12.59818921	2.16048E-36	4.61649E-35
NEURL2	18.78152673	-0.50024032	0.039679069	-12.60715882	1.9282E-36	4.12465E-35
MRAP	5.728154245	-1.110521666	0.088073226	-12.60907217	1.88196E-36	4.03014E-35
DHX33	695.0609214	-0.449433143	0.035641782	-12.60972698	1.86638E-36	4.00118E-35
RSAD1	692.9303823	-0.445203057	0.035291554	-12.61500294	1.74551E-36	3.74615E-35
PIK3R1	9003.730883	0.478687502	0.037943523	12.61578949	1.72817E-36	3.71301E-35
DNAI2	6.524980098	-0.967909015	0.076709688	-12.61781967	1.6842E-36	3.62251E-35

ARFGAP3	1460.48779	0.364126841	0.028838154	12.62656572	1.50714E-36	3.24523E-35
FEM1C	3494.451024	0.524087222	0.041499482	12.62876529	1.4656E-36	3.15927E-35
TAS2R10	5.534462557	-1.193994312	0.094502953	-12.63446562	1.36317E-36	2.94171E-35
SAMD14	130.9493469	-1.027551911	0.081315201	-12.63665218	1.3258E-36	2.86422E-35
YIPF5	516.3888846	0.380848816	0.030138025	12.63682042	1.32296E-36	2.86126E-35
LRRC45	346.2520967	-0.6185925	0.048933595	-12.64146843	1.24703E-36	2.70001E-35
LRRC9	8.171058194	-1.136339677	0.089884922	-12.64216124	1.23609E-36	2.67929E-35
COPS2	3503.675276	0.427268572	0.033796804	12.64227734	1.23426E-36	2.6783E-35
GLE1	909.859061	-0.337631869	0.026703219	-12.64386409	1.2096E-36	2.62769E-35
ADCK5	195.6453713	-0.58515223	0.046273928	-12.64539794	1.18622E-36	2.57977E-35
RRAGC	417.7866218	0.427327344	0.033792166	12.64575176	1.1809E-36	2.57104E-35
GABPA	1140.408059	0.373174657	0.02950512	12.64779325	1.15061E-36	2.5079E-35
COBL	13.20374376	-1.490550201	0.11781332	-12.65179696	1.09345E-36	2.38596E-35
PPP2CA	5036.186965	0.353981116	0.027974455	12.65372714	1.06691E-36	2.33064E-35
TIMMDC1	584.044756	-0.502613712	0.039711866	-12.65651202	1.02974E-36	2.25196E-35
SAMSN1	2023.981876	0.763787908	0.060322312	12.66178111	9.62896E-37	2.10814E-35
IL4R	2425.510542	0.611965879	0.048312067	12.66693648	9.01675E-37	1.97631E-35
ZNF780B	357.4931807	-0.696104589	0.054939521	-12.67037957	8.62958E-37	1.89357E-35
FAHD1	184.6560367	-0.574831569	0.045354731	-12.67412597	8.22706E-37	1.80728E-35
ATP13A4	46.526821	-1.04084593	0.082094058	-12.67869997	7.76084E-37	1.70677E-35
CAPN14	33.2279073	-0.974274363	0.076791224	-12.68731383	6.95298E-37	1.53083E-35
CDPF1	91.24573218	-0.561985717	0.044294362	-12.68752249	6.93448E-37	1.52847E-35
ZNF620	133.8099535	-0.515558679	0.040630466	-12.68896784	6.8077E-37	1.50222E-35
WARS1	3503.670997	0.592674434	0.046694659	12.69255305	6.50308E-37	1.43662E-35
ZNF583	62.02162599	-0.520634563	0.04101464	-12.69387143	6.39451E-37	1.41423E-35
CENPI	164.0042437	-0.649926906	0.051186621	-12.69720288	6.1281E-37	1.35685E-35
RAPGEF1	5741.237285	0.510278721	0.040183874	12.69859448	6.02012E-37	1.33445E-35
RNF123	1801.010406	-0.590556496	0.046502301	-12.69951116	5.95002E-37	1.32041E-35
CTNS	417.9845194	-0.377401552	0.029715146	-12.70064606	5.86436E-37	1.30287E-35
PYROXD2	294.1438398	-0.46093906	0.036292393	-12.7007072	5.85978E-37	1.30287E-35
CLHC1	158.6631004	-0.63651732	0.050103395	-12.70407574	5.61286E-37	1.24984E-35
SIM2	6.272618821	-0.984550536	0.077497772	-12.70424318	5.60086E-37	1.24858E-35
TAB1	848.1923358	-0.381592099	0.0300364	-12.70432226	5.59521E-37	1.24858E-35
IL1B	2422.553173	1.173575275	0.092360131	12.70651376	5.44063E-37	1.21563E-35
FREM3	11.11968639	-1.254272474	0.098710133	-12.70662333	5.43302E-37	1.21532E-35
CYP26A1	2.896232923	-1.479993221	0.116455818	-12.70862418	5.2958E-37	1.18598E-35
ELF1	12219.32268	0.405842126	0.031933664	12.70891221	5.27633E-37	1.18297E-35

ACBD6	927.4611919	-0.326802218	0.025701643	-12.71522689	4.86695E-37	1.09244E-35
N4BP1	1851.91965	0.441015307	0.034679503	12.71688663	4.76469E-37	1.07072E-35
NMNAT1	135.5415578	-0.590567363	0.046431666	-12.71906474	4.63373E-37	1.04248E-35
ALKBH2	105.803561	-0.558989062	0.043933773	-12.72344776	4.38095E-37	9.86746E-36
TMEM163	95.44939513	-1.082797154	0.085094117	-12.72470054	4.31125E-37	9.72166E-36
SYN1	122.6799374	1.206360077	0.094791928	12.72640089	4.2184E-37	9.52328E-36
ARHGEF33	13.69178046	-0.984239507	0.07726992	-12.73767983	3.65091E-37	8.25166E-36
ELMO3	131.8994432	-0.553106446	0.043408146	-12.74199657	3.4544E-37	7.81652E-36
MAGI1	25.42254813	-1.668990278	0.13094674	-12.74556572	3.29988E-37	7.47553E-36
PYGO1	12.44323198	-1.444064089	0.113266225	-12.74929127	3.14592E-37	7.13501E-36
ARL2	354.4463364	-0.828268942	0.064953932	-12.75163672	3.05268E-37	6.93157E-36
CLEC9A	45.44931808	-1.521389633	0.119303478	-12.75226557	3.02815E-37	6.88385E-36
LMF1	339.0645979	-0.468020289	0.036699241	-12.75286023	3.00514E-37	6.83948E-36
SEMA3G	63.16820868	-1.304422097	0.102239415	-12.7585051	2.79514E-37	6.36894E-36
NME1	323.2239735	-0.756543599	0.059290216	-12.76000749	2.74175E-37	6.25457E-36
POLR2H	510.045945	-0.458131286	0.035884082	-12.7669782	2.507E-37	5.72572E-36
BPHL	119.4515565	-0.444508197	0.0348117	-12.76893092	2.44489E-37	5.59041E-36
DEGS2	116.7422469	-0.725986835	0.056842466	-12.77190946	2.3531E-37	5.38681E-36
KIF3B	1138.04649	0.378300835	0.029619073	12.7722038	2.34422E-37	5.37276E-36
P2RY10	281.1910869	0.850909004	0.066620498	12.77248039	2.3359E-37	5.35998E-36
RARA	1990.236024	0.654911024	0.051274792	12.77257301	2.33312E-37	5.35988E-36
CHST6	13.49385086	-1.145300138	0.089660988	-12.7736729	2.30038E-37	5.29087E-36
TEX22	59.58688443	-0.596290762	0.046678639	-12.77438199	2.27951E-37	5.24904E-36
TIMM8A	206.4315873	-0.409053035	0.03201925	-12.77522207	2.25503E-37	5.19879E-36
KATNBL1	552.8663345	-0.490147238	0.03836498	-12.77590252	2.2354E-37	5.1596E-36
TUT1	161.7269219	-0.423938802	0.033178969	-12.77733516	2.19461E-37	5.07143E-36
ZNF474	6.170091957	-1.129669039	0.08840851	-12.7778315	2.18065E-37	5.04514E-36
DOK6	70.97747722	-1.483793023	0.116104069	-12.77985372	2.12469E-37	4.92148E-36
PABPC1L	1057.027193	-0.546380575	0.042751709	-12.7803212	2.11196E-37	4.89778E-36
TPD52L2	3339.281172	0.440158737	0.03443659	12.78171654	2.0744E-37	4.8164E-36
RIT1	3701.666681	0.509222395	0.039839347	12.78189597	2.06962E-37	4.81101E-36
AMOTL2	28.83927404	-1.57400623	0.123138731	-12.78238145	2.05674E-37	4.78675E-36
EXTL2	195.6560591	-0.686754829	0.053715628	-12.78500977	1.98838E-37	4.63316E-36
ZNF425	38.654595	-0.625172147	0.048897043	-12.78547962	1.9764E-37	4.61073E-36
CDC42	17573.84589	0.324928494	0.025407804	12.78853128	1.90031E-37	4.43852E-36
CHST7	251.0674559	0.787140823	0.06151591	12.79572754	1.73223E-37	4.05076E-36
MFSD4B	1101.789417	-0.568532322	0.044425164	-12.79752897	1.69252E-37	3.96263E-36

AHNAK2	263.0423603	-0.968023239	0.075640197	-12.79773556	1.68802E-37	3.95684E-36
ODF2	1242.675535	-0.385062668	0.030087089	-12.79826918	1.67646E-37	3.93446E-36
TFCP2	844.7121907	-0.443258519	0.034625557	-12.80148412	1.60847E-37	3.77943E-36
GTPBP6	1570.168647	-0.360179898	0.028134068	-12.80226881	1.5923E-37	3.74593E-36
AQP9	706.8555123	1.351882652	0.105571102	12.80542334	1.5289E-37	3.6011E-36
FBXO43	55.39975093	-0.733582116	0.057272497	-12.80862805	1.46705E-37	3.4596E-36
ABO	493.0474718	-1.491910757	0.11645872	-12.81064013	1.4295E-37	3.37511E-36
PPP6C	2644.225427	0.373856373	0.029167129	12.81772941	1.30465E-37	3.08406E-36
BOP1	1226.466652	-0.494623513	0.038573511	-12.82288029	1.2208E-37	2.88932E-36
LIG1	1401.284619	-0.425048285	0.03314723	-12.82304086	1.21827E-37	2.88683E-36
ITGA8	7.790557562	-1.356123659	0.105728726	-12.82644466	1.16593E-37	2.76615E-36
ASPSCR1	819.2513579	-0.444542065	0.034656362	-12.82714176	1.15549E-37	2.7447E-36
SLAMF1	191.3370389	0.987319365	0.076937588	12.83273096	1.07507E-37	2.55678E-36
KCTD10	1450.283824	0.361327564	0.028156632	12.83276947	1.07453E-37	2.55678E-36
DIP2A	2963.377096	-0.488089081	0.03803422	-12.83289331	1.07282E-37	2.55678E-36
ZNF556	21.2882087	-0.897426457	0.069896419	-12.8393768	9.86655E-38	2.3551E-36
GFY	3.176571834	-2.294629729	0.178683395	-12.84187445	9.5533E-38	2.28311E-36
B3GNT5	1751.0462	0.720775006	0.056126064	12.84207287	9.52884E-38	2.28005E-36
KLHL42	492.5531422	-0.465310762	0.03620826	-12.85095601	8.49538E-38	2.03525E-36
TTC31	740.2616787	-0.34618889	0.026936684	-12.85194885	8.38703E-38	2.01176E-36
UQCC1	692.0448295	-0.401002379	0.03120117	-12.85215829	8.36435E-38	2.00878E-36
KCNIP2	59.57190909	-0.648379065	0.050435087	-12.85571417	7.98846E-38	1.92086E-36
LRRC14	566.9421379	-0.402711069	0.031315681	-12.85972557	7.58455E-38	1.82598E-36
RAB40B	91.8470911	-0.649173089	0.050481013	-12.8597476	7.58239E-38	1.82598E-36
LZTR1	319.9880126	-0.43127576	0.033536707	-12.85981232	7.57604E-38	1.82598E-36
KCNA6	35.65475413	-1.379112556	0.107215121	-12.86304151	7.26603E-38	1.75578E-36
FASTK	1034.726827	-0.365791508	0.028418054	-12.8717998	6.48727E-38	1.56953E-36
ESRRG	7.208571757	-2.175043532	0.168975388	-12.87195465	6.47427E-38	1.56833E-36
MSH2	743.8803549	-0.485721319	0.037731599	-12.87306486	6.38186E-38	1.54786E-36
MRTFB	565.8586612	-0.548026092	0.042561882	-12.87598355	6.14512E-38	1.49229E-36
TRAF2	533.1618931	-0.32566481	0.025285862	-12.87932392	5.88488E-38	1.43087E-36
SLC15A2	820.7949321	-0.85086571	0.066033688	-12.88532776	5.44437E-38	1.32541E-36
PBX4	360.626005	1.032355162	0.080068337	12.89342582	4.9017E-38	1.19478E-36
ALX3	10.44379177	-1.55098392	0.120282444	-12.89451621	4.83286E-38	1.17947E-36
TMEM143	235.7398173	-0.382305628	0.029643638	-12.89671752	4.69681E-38	1.1477E-36
VAMP7	1743.7154	0.370895699	0.028750001	12.90071956	4.45914E-38	1.09098E-36
PLIN2	9139.916756	0.858819957	0.066505943	12.91343182	3.78068E-38	9.26148E-37

YME1L1	5834.403459	0.325627006	0.025211736	12.91569165	3.6713E-38	9.00481E-37
UBE2Q2	2432.867922	0.381267401	0.02951358	12.91837203	3.54564E-38	8.70751E-37
NAA40	1079.888816	-0.396304748	0.030661821	-12.9250231	3.25199E-38	7.99638E-37
ARHGAP28	5.416638382	-1.255486868	0.097116797	-12.9275976	3.14493E-38	7.74286E-37
TMEM150C	34.26641277	-0.99384255	0.076874262	-12.92815724	3.12212E-38	7.69639E-37
RUFY1	1878.414749	-0.362591717	0.028030199	-12.93575242	2.8284E-38	6.98113E-37
CARD8	2104.557097	-0.56947188	0.044022921	-12.9358041	2.8265E-38	6.98113E-37
C1orf141	13.86937372	-2.090899401	0.161636241	-12.93583284	2.82545E-38	6.98113E-37
SGTB	899.8176716	0.657024675	0.050779726	12.93872048	2.72124E-38	6.74213E-37
ZMYND19	277.1128747	-0.41512188	0.032076812	-12.94149419	2.62474E-38	6.51129E-37
SPATA5	583.8124698	-0.612018451	0.047280667	-12.94437005	2.52829E-38	6.27997E-37
CLYBL	62.71254451	-0.716234224	0.055316903	-12.94783663	2.41669E-38	6.0104E-37
NYNRIN	620.7315093	-1.028358512	0.079410416	-12.94991966	2.352E-38	5.85696E-37
CD8B	94.83108011	0.921589007	0.071117958	12.95859771	2.10052E-38	5.23739E-37
AP002495.1	113.0759438	-0.581289569	0.044849272	-12.96095879	2.03686E-38	5.08512E-37
TMEM72	37.36176118	-1.246797264	0.096170183	-12.96448878	1.94523E-38	4.86258E-37
MYO19	1649.034236	-0.359876848	0.027756876	-12.9653225	1.92419E-38	4.81614E-37
ZNF114	35.96576311	-0.739253744	0.05701121	-12.96681372	1.88713E-38	4.72943E-37
WNK3	9.930600705	-1.229617047	0.094818359	-12.96813248	1.85495E-38	4.65473E-37
ABCA12	13.57306623	-1.184622555	0.091341729	-12.96912783	1.83102E-38	4.60058E-37
LINGO2	8.981492646	-2.420282286	0.186601128	-12.97035183	1.80201E-38	4.53352E-37
PTGIS	17.1200642	-1.350118915	0.103962234	-12.98662847	1.45706E-38	3.6704E-37
SERPINB9	1635.102236	0.914888087	0.070434451	12.98921296	1.40868E-38	3.55311E-37
MANEAL	49.55471925	-0.837429539	0.064464233	-12.99060739	1.38325E-38	3.49346E-37
APEH	1739.31834	-0.48670835	0.037466045	-12.99065194	1.38244E-38	3.49346E-37
ZMYM3	2111.339375	-0.4483239	0.034507366	-12.9921216	1.35615E-38	3.43388E-37
TSPAN32	1273.662156	-0.863779075	0.066483494	-12.99238391	1.35151E-38	3.42656E-37
CHMP4B	3064.478058	0.576219706	0.044334852	12.9969917	1.27251E-38	3.23046E-37
EIPR1	575.800496	-0.451668721	0.034737993	-13.00215344	1.18946E-38	3.02355E-37
GPAT4	2145.453057	0.437043071	0.033604228	13.00559753	1.13707E-38	2.89412E-37
SLC37A4	339.2355702	-0.495081755	0.038047165	-13.01231647	1.04137E-38	2.654E-37
LYG2	19.55357684	-0.745370905	0.05727725	-13.013385	1.0269E-38	2.62055E-37
MSTO1	239.0278198	-0.376923364	0.028961457	-13.01465487	1.00998E-38	2.58071E-37
SEMA6C	138.6384413	-0.887858225	0.068202152	-13.01803819	9.66215E-39	2.47212E-37
MAPRE1	6467.742116	0.337485448	0.025924322	13.01810099	9.65421E-39	2.47212E-37
CCL24	28.55374982	1.396567628	0.107212139	13.02620804	8.68161E-39	2.22707E-37
CTPS2	568.1745469	-0.460256598	0.035332614	-13.02639531	8.66033E-39	2.22452E-37

ARHGEF15	7.593656768	-1.070871	0.082181934	-13.03049158	8.20772E-39	2.11103E-37
SETD4	442.6839329	-0.365448194	0.028040774	-13.03274291	7.96905E-39	2.05234E-37
CCDC77	187.5763726	-0.423313901	0.032480142	-13.0330063	7.94158E-39	2.04797E-37
MYLK3	38.88680564	-0.93466531	0.071708792	-13.0341801	7.82031E-39	2.01935E-37
MCUB	491.1357536	0.655486267	0.050289005	13.03438541	7.79929E-39	2.01659E-37
CNRIP1	289.3798869	-1.231193835	0.094456208	-13.03454647	7.78284E-39	2.01499E-37
PANK1	85.93933226	-0.875987359	0.067197	-13.03610819	7.6251E-39	1.97677E-37
FAM207A	170.6476877	-0.588042649	0.045095464	-13.03995112	7.25035E-39	1.88211E-37
ITGA2B	3135.705964	-1.455608713	0.111614011	-13.04145156	7.10906E-39	1.84788E-37
LRRC63	40.49928707	-0.882489246	0.067663428	-13.04233721	7.02694E-39	1.82897E-37
AAAS	857.9982201	-0.483607696	0.037077659	-13.04310239	6.95676E-39	1.81311E-37
RASIP1	40.25298813	-1.116377137	0.085579199	-13.04495898	6.78935E-39	1.77184E-37
METTL8	588.7156262	-0.445682989	0.034154888	-13.04887878	6.44895E-39	1.68525E-37
CBFA2T3	1432.969813	-0.672464206	0.051533004	-13.04919477	6.42226E-39	1.68052E-37
UBL3	1173.992165	0.3995292	0.030612228	13.05129423	6.24769E-39	1.63703E-37
SFXN2	434.3190128	-0.513916176	0.039358284	-13.0573828	5.76768E-39	1.51328E-37
SMARCC2	4460.390784	-0.273221458	0.020922572	-13.05869392	5.66921E-39	1.48944E-37
ACTR3C	104.369025	-0.739111522	0.056534781	-13.07357196	4.66238E-39	1.22657E-37
TFB1M	245.0050158	-0.562418141	0.043013416	-13.0754122	4.5509E-39	1.19885E-37
HECW1	11.73470904	-1.260808151	0.096411142	-13.07741126	4.43281E-39	1.16932E-37
CDC16	1344.513792	-0.382445188	0.029240208	-13.07942779	4.31677E-39	1.14025E-37
CHCHD6	107.432362	-0.617014642	0.04717149	-13.08024503	4.27061E-39	1.12958E-37
RABEPK	412.8934847	-0.408404281	0.03122218	-13.08058194	4.25173E-39	1.1261E-37
SLC43A2	1994.569985	0.840641675	0.064263734	13.08112092	4.22168E-39	1.11966E-37
PCED1A	1230.383602	-0.448241811	0.034262937	-13.08241065	4.15065E-39	1.10232E-37
WDR12	750.5318603	-0.511834928	0.039115462	-13.08523298	3.99932E-39	1.06357E-37
ZNF585B	205.0504399	-0.633735034	0.04842783	-13.08617436	3.95008E-39	1.0519E-37
GPATCH1	277.5927961	-0.42412162	0.032409143	-13.08648068	3.93418E-39	1.0491E-37
CATSPER3	11.08088046	-0.750100121	0.057297038	-13.09142927	3.68606E-39	9.84273E-38
ZNF404	20.67981934	-1.000846366	0.076376434	-13.10412534	3.11836E-39	8.33822E-38
ICAM1	1807.611844	0.928486189	0.07084353	13.10615361	3.0361E-39	8.12936E-38
ZNF285	30.19092346	-1.034706738	0.078929447	-13.10926124	2.91423E-39	7.81374E-38
COL6A5	104.8078449	-2.040671957	0.155654669	-13.11025218	2.8764E-39	7.72289E-38
NIPSNAP2	1831.751436	0.332498628	0.025360748	13.11075784	2.85729E-39	7.6821E-38
MON1A	133.9843275	-0.633345586	0.048290467	-13.11533371	2.68996E-39	7.24216E-38
NFIB	32.09084949	-1.754554104	0.133763342	-13.11685305	2.63658E-39	7.10823E-38
AL360181.3	49.84568737	-0.641921624	0.048935135	-13.11780631	2.60363E-39	7.02908E-38

ZNF500	397.8796673	-0.419178632	0.031948023	-13.12064385	2.50795E-39	6.78013E-38
POLR3K	198.0280556	-0.565331103	0.043069176	-13.12611845	2.33312E-39	6.31622E-38
COQ4	742.0640261	-0.43391141	0.033056378	-13.12640501	2.32431E-39	6.30108E-38
C20orf96	465.6360421	-0.561539689	0.042772277	-13.12859002	2.25822E-39	6.1304E-38
NT5M	337.6030135	-0.632889782	0.048194188	-13.13207675	2.1566E-39	5.86266E-38
VEZF1	2879.751651	0.380889524	0.028994895	13.1364339	2.03598E-39	5.54245E-38
FUZ	271.8096894	-0.414516622	0.031554518	-13.13652222	2.0336E-39	5.54245E-38
MRPL24	324.5253607	-0.858034366	0.065309902	-13.13789094	1.99716E-39	5.45194E-38
LDAH	317.9348843	-0.730978627	0.055632024	-13.13952967	1.95438E-39	5.3426E-38
RCOR2	63.79979753	-1.151589638	0.087627898	-13.14181519	1.89623E-39	5.19089E-38
PPTC7	4884.635085	0.460568829	0.035037328	13.14509009	1.81589E-39	4.97793E-38
AARS2	629.4203791	-0.462435987	0.03517296	-13.14748563	1.75928E-39	4.8295E-38
CMTM1	37.98543991	-0.769589043	0.058516267	-13.15171117	1.66366E-39	4.57344E-38
POLE	4762.810653	-0.559896992	0.042530722	-13.16453076	1.40407E-39	3.86525E-38
NUDCD1	383.7538451	-0.52347765	0.039720833	-13.17891919	1.16041E-39	3.19898E-38
NINJ1	3380.464829	0.927872593	0.070405584	13.17896309	1.15974E-39	3.19898E-38
ZNF749	197.7991209	-0.479933905	0.036404049	-13.18353098	1.0916E-39	3.01777E-38
RPUSD3	653.3456028	-0.575050815	0.043613502	-13.18515572	1.06833E-39	2.95763E-38
FCGR3A	245.5007611	1.311628183	0.09947403	13.18563433	1.06157E-39	2.94308E-38
OCA2	2.855538893	-1.874913255	0.142162725	-13.18850105	1.02197E-39	2.83731E-38
EXD3	357.3441755	-0.532958627	0.040402323	-13.19128686	9.84895E-40	2.73826E-38
UBE2J1	7744.50434	0.500479287	0.037927006	13.19585524	9.26968E-40	2.58087E-38
TRIM66	982.4999101	-0.599212407	0.045407161	-13.19642961	9.19929E-40	2.56493E-38
POLL	632.4864625	-0.370934489	0.028108235	-13.19664813	9.17265E-40	2.56115E-38
CSMD3	5.578677785	-2.021504287	0.153095253	-13.20422575	8.29482E-40	2.31935E-38
C19orf25	304.2295237	-0.446783417	0.033835703	-13.20449623	8.26508E-40	2.31434E-38
FAM227B	71.0534318	-0.58683091	0.04444163	-13.20453159	8.2612E-40	2.31434E-38
TRMT1	1668.080896	-0.459095896	0.034762457	-13.20665846	8.03112E-40	2.25528E-38
CCN5	6.534491698	-1.201196703	0.090945669	-13.20784945	7.90508E-40	2.22308E-38
KCNV2	17.0767595	-1.03125807	0.078077169	-13.2081899	7.86941E-40	2.21623E-38
WDR46	1103.808495	-0.328824071	0.024892741	-13.20963705	7.71957E-40	2.17716E-38
WWOX	426.6569478	-0.526039449	0.039818292	-13.21099992	7.58106E-40	2.14119E-38
ACAD9	869.0241757	-0.37572153	0.028436388	-13.21270217	7.41152E-40	2.09633E-38
IL2RB	291.5481675	0.969237483	0.073332419	13.21703953	6.99639E-40	1.98177E-38
TXNRD2	619.6902612	-0.414754166	0.031365819	-13.22312569	6.45257E-40	1.83038E-38
FADS2	2208.449619	-0.824323861	0.062324024	-13.22642225	6.17579E-40	1.75441E-38
CLDN3	5.503991193	-1.188304529	0.08982711	-13.2287962	5.98381E-40	1.70234E-38

BYSL	336.4455082	-0.443741292	0.033540191	-13.23013618	5.87808E-40	1.6747E-38
GDPGP1	73.44569292	-0.57287993	0.0432671	-13.24054386	5.11769E-40	1.46018E-38
C14orf93	195.0508044	-0.388565283	0.029344156	-13.24165816	5.04231E-40	1.44078E-38
PIF1	116.7599602	-0.775529203	0.0585262	-13.25097492	4.4538E-40	1.27448E-38
GNG2	1717.57096	0.789820555	0.05959265	13.25365714	4.2974E-40	1.23152E-38
PIK3R3	71.07141191	-0.999185262	0.075385818	-13.25428689	4.26148E-40	1.22302E-38
ADNP2	3440.007592	0.551476967	0.04160595	13.25476212	4.23458E-40	1.21708E-38
CFAP54	14.84355653	-0.880642421	0.066425121	-13.25767138	4.07349E-40	1.17251E-38
RAB8B	3984.80057	0.63129484	0.047617093	13.25773594	4.06999E-40	1.17251E-38
HSPA13	1106.109393	0.479539983	0.036139535	13.2691242	3.49643E-40	1.00938E-38
RUSF1	1023.996624	-0.43189942	0.032548062	-13.26958928	3.4748E-40	1.00461E-38
EFCAB6	15.10346824	-0.989848219	0.074574067	-13.27335707	3.30439E-40	9.56758E-39
STX11	2343.978325	0.761557125	0.057369204	13.27466779	3.24708E-40	9.41557E-39
C8orf37	65.76183569	-0.474276387	0.035715179	-13.2794066	3.048E-40	8.85141E-39
KIAA1549L	19.68517365	-1.541396376	0.116055007	-13.28160171	2.95994E-40	8.60846E-39
SNAPC4	707.586873	-0.385009275	0.028981941	-13.28445435	2.84928E-40	8.29895E-39
ZNF397	490.948332	-0.564448837	0.042485095	-13.28580866	2.79819E-40	8.16229E-39
CDX2	10.38267077	-1.26635531	0.095278148	-13.29114115	2.60574E-40	7.61225E-39
PPARG	117.8069033	1.505824058	0.113244736	13.29707779	2.40693E-40	7.04197E-39
CCDC51	66.85083833	-0.652451618	0.04905916	-13.29928238	2.33701E-40	6.84764E-39
TBX2	13.82002909	-1.307493934	0.098311842	-13.29945516	2.33161E-40	6.84207E-39
CNNM1	39.90116339	-1.495847046	0.112430621	-13.30462313	2.17587E-40	6.39465E-39
GSE1	5020.377127	-0.417462616	0.031372814	-13.30650845	2.12167E-40	6.24474E-39
FDXR	265.9368824	-0.602646129	0.045284603	-13.30796972	2.08059E-40	6.13304E-39
ERI3	856.8644029	-0.496856608	0.03732314	-13.31229387	1.96359E-40	5.79689E-39
PIK3CA	2742.377732	0.581051888	0.043646393	13.31271254	1.95262E-40	5.7732E-39
TMEM260	385.9905357	-0.522248353	0.039227838	-13.3132076	1.93972E-40	5.74374E-39
POLR1A	2417.236523	-0.46639932	0.035031413	-13.31374549	1.9258E-40	5.71117E-39
TCAF2	189.3698976	0.952722449	0.071549896	13.31549727	1.88116E-40	5.58724E-39
ITPRID2	9430.394654	0.574818915	0.043151905	13.32082358	1.75164E-40	5.21048E-39
PPIL6	40.60020094	-0.620877814	0.046593889	-13.3253057	1.64956E-40	4.91427E-39
NLE1	477.1737341	-0.466084333	0.034976559	-13.32561998	1.64263E-40	4.90108E-39
SCRN2	473.013297	-0.512716625	0.038475914	-13.32565157	1.64193E-40	4.90108E-39
CEP131	412.529934	-0.443289753	0.033258409	-13.32865179	1.57722E-40	4.72033E-39
KLHDC7B	340.3560163	1.12833771	0.08464134	13.33081102	1.53223E-40	4.5927E-39
PDE4B	3883.982334	0.795669199	0.059682433	13.33171523	1.51377E-40	4.54432E-39
ZNF132	100.1496277	-0.749991166	0.056248619	-13.33350374	1.4779E-40	4.44347E-39

ZNF69	341.154685	-0.606121679	0.04545796	-13.33367545	1.4745E-40	4.44007E-39
ITFG2	851.9948196	-0.374036056	0.028044498	-13.33723469	1.40578E-40	4.23965E-39
FANCA	1193.211014	-0.488160543	0.03659015	-13.34131043	1.33099E-40	4.02029E-39
FGF14	3.340610163	-2.009534169	0.150504346	-13.35200097	1.15309E-40	3.48833E-39
ZNF446	154.4538354	-0.375877585	0.028141795	-13.35656022	1.08462E-40	3.28625E-39
CCSER2	1246.843097	0.512707928	0.038382583	13.35782761	1.06631E-40	3.23579E-39
TTI1	705.1547553	-0.404981432	0.030311484	-13.36065993	1.0265E-40	3.11983E-39
PTGES2	726.3252753	-0.390825116	0.029236241	-13.36783037	9.32205E-41	2.83764E-39
CNNM3	939.3156565	-0.429992644	0.032159735	-13.37052825	8.99001E-41	2.74083E-39
SLC25A20	420.8428566	-0.464736808	0.03475045	-13.37354779	8.63232E-41	2.63589E-39
STK25	1596.753706	-0.403536545	0.030168377	-13.37614361	8.33616E-41	2.54943E-39
WDR31	115.3997933	-0.620120819	0.046356948	-13.37708455	8.23132E-41	2.52131E-39
STAU2	742.3784207	-0.536727416	0.040107709	-13.38215091	7.68896E-41	2.35887E-39
RAB5A	1733.097522	0.387734722	0.02897153	13.38330136	7.57085E-41	2.32628E-39
ASH2L	1435.030374	-0.323926226	0.024197075	-13.3869991	7.20328E-41	2.21682E-39
NANOGP8	5.229993318	-1.015908168	0.075852739	-13.39316395	6.62953E-41	2.04346E-39
CD28	319.3671879	1.076251521	0.080335572	13.39694853	6.30006E-41	1.94497E-39
TRMT13	208.1373769	-0.548342945	0.040926711	-13.39816779	6.19743E-41	1.91631E-39
DPH6	169.6534541	-0.578645363	0.043178611	-13.40120363	5.94905E-41	1.84242E-39
SAMD8	2011.301492	0.547058629	0.040817277	13.40262414	5.83625E-41	1.81035E-39
ARHGAP44	64.99061761	-1.250399687	0.093293397	-13.40287439	5.8166E-41	1.80712E-39
IFT172	911.6481421	-0.488278423	0.036415881	-13.40839257	5.39965E-41	1.68024E-39
TTLL12	1272.905634	-0.661159618	0.049303599	-13.40996668	5.28626E-41	1.64758E-39
S1PR5	58.33214065	1.003208685	0.07479481	13.41281155	5.0873E-41	1.5881E-39
TFAP4	247.4017617	-0.582638657	0.043430987	-13.41527557	4.92101E-41	1.53864E-39
NFASC	36.54661577	-1.015729653	0.075709014	-13.41623138	4.85796E-41	1.52136E-39
MED20	289.9012344	-0.459349648	0.034176924	-13.44034484	3.50798E-41	1.10035E-39
WDR6	3353.4733	-0.410281622	0.03052052	-13.44281243	3.39292E-41	1.06596E-39
ST6GALNAC5	4.915109613	-1.911641267	0.142178151	-13.44539404	3.27656E-41	1.03106E-39
TTLL5	1200.67055	-0.378701231	0.028163295	-13.44662359	3.22254E-41	1.0157E-39
CARS2	1810.870641	-0.304311027	0.022630006	-13.44723583	3.19598E-41	1.00895E-39
CTHRC1	41.71236074	-1.169645926	0.086963678	-13.44982134	3.08617E-41	9.75858E-40
MARCO	96.18086243	1.724792039	0.128224918	13.45130157	3.025E-41	9.58065E-40
HMOX1	790.2336961	1.231460225	0.091479446	13.46160563	2.63135E-41	8.34741E-40
SOCS3	2242.288971	0.984369923	0.073073834	13.47089466	2.32037E-41	7.37283E-40
CFAP44	269.5236653	-0.713904099	0.052981691	-13.47454358	2.20847E-41	7.02867E-40
ENOX1	6.641097136	-1.501105326	0.111371868	-13.47831681	2.09839E-41	6.68923E-40

DUSP19	41.69195147	-1.047654158	0.077705268	-13.48240849	1.98519E-41	6.33868E-40
FAM120C	411.7468136	-0.428999414	0.031816609	-13.48350522	1.95589E-41	6.25534E-40
RNF121	368.23713	-0.431952952	0.032010506	-13.4940996	1.69412E-41	5.42701E-40
TGOLN2	15885.4278	0.253400531	0.018778167	13.49442306	1.6867E-41	5.41211E-40
C19orf57	60.27997371	-0.849859675	0.062961082	-13.49817464	1.60299E-41	5.15193E-40
PARP10	1683.798815	-0.578709075	0.042862451	-13.50153936	1.53143E-41	4.93003E-40
PRKD1	11.01702037	-1.906909087	0.141201676	-13.50486153	1.46389E-41	4.72037E-40
SUV39H2	240.0872758	-0.431136594	0.031920465	-13.50658879	1.42995E-41	4.61856E-40
EEF2KMT	67.20312017	-0.636871476	0.047113861	-13.5177094	1.22945E-41	3.97751E-40
ZRANB3	454.2699333	-0.463644431	0.034290137	-13.52121835	1.17218E-41	3.79853E-40
ZNF334	81.1884395	-1.46011322	0.107946259	-13.52629759	1.09396E-41	3.55093E-40
B3GAT2	36.12133324	-1.079601935	0.079803651	-13.52822744	1.06562E-41	3.46469E-40
UBTF	2528.697258	-0.396673911	0.029302925	-13.53700751	9.45633E-42	3.07968E-40
RAD54B	21.14672885	-0.726226776	0.053637915	-13.53942954	9.14967E-42	2.98477E-40
PPP1R3B	611.8485692	0.711485324	0.052533565	13.54344274	8.66316E-42	2.83079E-40
PUS7	283.5053231	-0.606363479	0.044757865	-13.54764086	8.18176E-42	2.67795E-40
MAT1A	2.408438699	-1.855690026	0.136950959	-13.5500331	7.91944E-42	2.59644E-40
OSGEPL1	124.2002763	-0.522081604	0.038525114	-13.55172114	7.73939E-42	2.54166E-40
LARS1	2788.978438	-0.609332558	0.044962062	-13.5521489	7.69442E-42	2.53114E-40
BDH1	613.973633	-0.606585544	0.044751286	-13.55459471	7.44221E-42	2.45229E-40
LRRC1	88.2674615	-0.677807221	0.050003252	-13.55526291	7.37474E-42	2.43416E-40
AK9	238.1121793	-0.599143646	0.04419001	-13.55835056	7.07083E-42	2.33779E-40
ZNF169	190.728906	-0.57280583	0.042237952	-13.56140171	6.78274E-42	2.24634E-40
PRICKLE3	210.1233842	-0.443367252	0.032688499	-13.5634018	6.60026E-42	2.18961E-40
TRMT1L	425.6433944	-0.605444556	0.044635576	-13.56417036	6.53145E-42	2.17046E-40
PRH2	42.59560774	-1.099685268	0.081018262	-13.57330115	5.76653E-42	1.91953E-40
FLG	26.55595507	-1.693526053	0.124748787	-13.57549118	5.59673E-42	1.86618E-40
AGAP2	877.4555199	-0.555318803	0.040900203	-13.57740958	5.45208E-42	1.82105E-40
POLRMT	1259.697699	-0.409026585	0.030086097	-13.5952025	4.27576E-42	1.43059E-40
SGK2	32.35192928	-0.765020932	0.056148444	-13.62497125	2.84522E-42	9.53588E-41
MYBBP1A	1588.134913	-0.504221708	0.036994254	-13.62973021	2.66564E-42	8.94932E-41
SMYD4	577.7384645	-0.520354851	0.038166913	-13.63366346	2.52576E-42	8.49427E-41
ATP6AP1L	68.13815818	-0.740014479	0.054255776	-13.63936779	2.33577E-42	7.86884E-41
SLC16A6	83.20084979	1.170133547	0.085790151	13.63948588	2.33199E-42	7.86884E-41
HERC2	5211.090611	-0.539594785	0.039545114	-13.64504321	2.16085E-42	7.30473E-41
XPO5	1398.384375	-0.399989383	0.02930109	-13.65100673	1.99109E-42	6.74248E-41
MCM3	2245.218595	-0.583424148	0.042738381	-13.65105858	1.98967E-42	6.74248E-41

MYO3B	16.42123565	-1.5612553	0.11436386	-13.65164918	1.97361E-42	6.7065E-41
PLA2G6	900.7527544	-0.525390299	0.03848117	-13.65317904	1.9326E-42	6.57857E-41
AMHR2	14.8192474	-1.218840625	0.089238375	-13.65825664	1.80247E-42	6.14631E-41
METAP1D	287.4903913	-0.606563057	0.044393836	-13.66322706	1.68354E-42	5.75079E-41
CHST12	1520.540852	-0.898973168	0.065782031	-13.66593811	1.622E-42	5.55024E-41
PHF11	1173.790285	0.408518229	0.029880072	13.67192919	1.49382E-42	5.12057E-41
GPR183	2032.986463	0.984799051	0.072012024	13.67548077	1.42264E-42	4.88514E-41
MED12L	1938.524569	-1.464412621	0.107018204	-13.68377127	1.26935E-42	4.36642E-41
SERPINI1	80.22847242	-1.125722909	0.082258896	-13.68512059	1.246E-42	4.29366E-41
HGH1	369.125296	-0.681925886	0.049827602	-13.68570551	1.23602E-42	4.26676E-41
CTCFL	6.280870444	-1.536697709	0.112253417	-13.68954065	1.17248E-42	4.05459E-41
BCAS3	896.6789439	-0.518664963	0.037879855	-13.69236933	1.12771E-42	3.90667E-41
CLK2	1089.014787	-0.378003604	0.027605633	-13.69298801	1.11815E-42	3.88041E-41
IFNGR2	2277.109548	0.645869035	0.047166363	13.69342465	1.11145E-42	3.86401E-41
ZC3H11B	30.06547551	0.957651645	0.06989023	13.70222486	9.84604E-43	3.42912E-41
TARBP2	483.9893367	-0.452745368	0.033035536	-13.70479864	9.50306E-43	3.31557E-41
RRNAD1	372.4168145	-0.465095723	0.033935388	-13.70533078	9.43364E-43	3.29723E-41
ZSCAN30	478.3605718	-0.552839972	0.040336334	-13.70575658	9.37846E-43	3.2838E-41
ERO1A	1974.849441	0.490225533	0.035759152	13.70909277	8.95708E-43	3.14188E-41
HDAC10	63.95314705	-0.455853032	0.033221711	-13.72153977	7.5446E-43	2.65117E-41
GPT	100.0190294	-0.887505641	0.064666823	-13.72428095	7.26463E-43	2.55738E-41
TTLL1	136.4973094	-0.552344189	0.040234476	-13.72813184	6.88869E-43	2.42941E-41
EHBP1	512.064948	-0.585841488	0.042674305	-13.72820206	6.88202E-43	2.42941E-41
FARS2	471.1630586	-0.61100231	0.044489627	-13.73359039	6.38874E-43	2.26124E-41
KPNA3	2458.806315	0.376986106	0.027447391	13.73486128	6.27762E-43	2.22594E-41
MYRIP	61.34480055	-1.132943024	0.082473651	-13.73703009	6.09241E-43	2.16419E-41
HDAC6	2349.478024	-0.396624895	0.028850074	-13.74779492	5.25056E-43	1.86853E-41
PTCHD4	5.982810625	-2.655705458	0.193022097	-13.75855667	4.5247E-43	1.61315E-41
MAFB	1611.89545	1.415137442	0.102836422	13.76105287	4.37114E-43	1.56125E-41
SNX6	1630.814749	0.317791072	0.023080639	13.76872934	3.93066E-43	1.40649E-41
DNAJB9	600.1924746	0.600582128	0.043614716	13.77017185	3.85296E-43	1.38121E-41
PLEKHO2	2428.663481	0.627426959	0.045525862	13.78177009	3.28127E-43	1.17843E-41
CDIN1	119.5626616	-0.728905511	0.052863763	-13.7883772	2.9942E-43	1.07731E-41
ACVR1B	1110.482472	0.643759784	0.046668192	13.79440153	2.75429E-43	9.92813E-42
UPP1	2316.443021	0.937078804	0.067881608	13.8046052	2.39079E-43	8.63374E-42
ZNF239	27.73164826	-0.957113488	0.0693105	-13.80906909	2.24717E-43	8.13009E-42
ATP2B1	5583.805755	0.712409291	0.051582722	13.81100615	2.18755E-43	7.92905E-42

CHEK2	335.181952	-0.546502965	0.039566311	-13.81233048	2.14769E-43	7.79904E-42
PIP4K2B	1860.173887	-0.489345888	0.035406858	-13.82065261	1.91327E-43	6.96069E-42
TMEM79	88.70632335	-0.61061152	0.044173634	-13.82298598	1.85224E-43	6.7512E-42
IFT140	585.7424282	-0.561218206	0.040584868	-13.82826251	1.72128E-43	6.28556E-42
ZFP64	308.4954103	-0.537509979	0.038861306	-13.83149551	1.64563E-43	6.02055E-42
COQ10B	1149.573938	0.435770767	0.031502868	13.83273319	1.61756E-43	5.92892E-42
METTL6	235.9198284	-0.512851923	0.03702253	-13.85242769	1.22984E-43	4.51627E-42
ZFP2	23.05784033	-0.842438543	0.060807758	-13.85412932	1.20104E-43	4.4188E-42
UFM1	1830.850579	0.49001424	0.035365638	13.85565941	1.17572E-43	4.33379E-42
ARHGAP22	212.7795702	-1.110795309	0.08007918	-13.87121231	9.46632E-44	3.49593E-42
WDFY1	1838.789545	0.592471759	0.042708319	13.87251422	9.29603E-44	3.43953E-42
RYR2	26.34005103	-2.137448273	0.15405163	-13.87488255	8.99403E-44	3.33409E-42
EHHADH	74.16487007	-0.916446183	0.066040458	-13.87704163	8.72723E-44	3.24133E-42
DFFA	954.9276148	-0.407346164	0.029353127	-13.87743666	8.67927E-44	3.22965E-42
RNF38	4535.500587	0.391545483	0.028210298	13.87952306	8.4303E-44	3.14298E-42
BOC	26.07457962	-1.15072303	0.082892166	-13.8821686	8.12481E-44	3.03486E-42
AGFG1	5410.966708	0.54161416	0.039011686	13.88338255	7.98833E-44	2.98959E-42
PRR4	8.875903916	-1.089852442	0.078496654	-13.88406239	7.91291E-44	2.96704E-42
NDUFV3	963.5454859	-0.381639295	0.027484267	-13.88573674	7.73014E-44	2.90407E-42
RAB18	2575.05956	0.446399026	0.032142548	13.88810344	7.47895E-44	2.81511E-42
MRPS26	317.5094909	-0.59127937	0.04255666	-13.89393285	6.89434E-44	2.60006E-42
ZNF616	109.212944	-0.704294349	0.05066753	-13.90030939	6.30682E-44	2.38308E-42
MBD3	954.9154526	-0.410427999	0.029525951	-13.90058544	6.28254E-44	2.37849E-42
NIPAL3	800.7287992	-0.513651103	0.036938052	-13.90574415	5.84557E-44	2.21735E-42
ZNF175	277.6095855	-0.74785764	0.053772028	-13.90793083	5.6696E-44	2.15478E-42
METTL15	266.0891506	-0.583496555	0.041933647	-13.91475814	5.1534E-44	1.9624E-42
UNC45A	1378.204333	-0.387461766	0.027840952	-13.91697281	4.99621E-44	1.90625E-42
SLC7A7	953.2757092	1.097384446	0.078801174	13.92599114	4.4039E-44	1.68354E-42
PCBD2	476.9423654	-0.411445592	0.029543447	-13.92679696	4.3545E-44	1.66792E-42
SLC39A4	365.1900673	-0.839242694	0.060250643	-13.92919064	4.211E-44	1.61612E-42
CD5	626.7403159	0.941021225	0.067502097	13.94062213	3.58799E-44	1.37972E-42
TMEM17	34.16145547	-0.74357987	0.053327033	-13.94376985	3.43316E-44	1.32278E-42
IQCH	43.41152066	-0.657560422	0.047151697	-13.94563627	3.34452E-44	1.29117E-42
ZNF559	225.6887058	-0.659943486	0.047289017	-13.95553411	2.91113E-44	1.12608E-42
C17orf78	10.77478755	-0.910271127	0.065222883	-13.95631548	2.87939E-44	1.11601E-42
GDPD1	96.26268899	-0.790309471	0.056578338	-13.96841084	2.42988E-44	9.43652E-43
KEAP1	1076.050102	-0.363829391	0.026044735	-13.96940276	2.39628E-44	9.32452E-43

PIDD1	1000.882428	-0.409055854	0.02927259	-13.97402346	2.24573E-44	8.7561E-43
WDR70	872.5238653	-0.424058544	0.030344731	-13.97470085	2.22446E-44	8.6905E-43
MRTO4	559.9722514	-0.4602813	0.032930151	-13.97750358	2.13859E-44	8.37172E-43
SDCBP	5272.885677	0.5483057	0.03922745	13.97760237	2.13562E-44	8.37172E-43
HDDC3	135.721484	-0.513296315	0.036713902	-13.98097951	2.03666E-44	8.00471E-43
IL1RN	1002.561368	1.363845414	0.097544239	13.9818141	2.01291E-44	7.9273E-43
RPP40	94.90439608	-0.80951126	0.057886377	-13.98448655	1.93871E-44	7.65048E-43
ATP6V1B2	5195.634013	0.530284094	0.037904917	13.98984991	1.79791E-44	7.10919E-43
PLA2G12A	180.1992308	-0.564018297	0.040314347	-13.99051058	1.78128E-44	7.05771E-43
ABCC5	1215.255209	-0.679079344	0.048527449	-13.99371623	1.70277E-44	6.76029E-43
WDR27	586.0557477	-0.740702305	0.052910953	-13.99903542	1.58001E-44	6.28566E-43
RNF138	2685.025629	0.472490755	0.033736449	14.00534935	1.44567E-44	5.76296E-43
RAB1A	3595.768029	0.447021381	0.031906465	14.01036982	1.34702E-44	5.38065E-43
TYSND1	310.8576648	-0.576640694	0.04114666	-14.01427717	1.2749E-44	5.10298E-43
TIAF1	273.5271727	-0.62785936	0.044772563	-14.02330633	1.1226E-44	4.5026E-43
SH3GLB1	4194.023627	0.405429642	0.028904459	14.02654293	1.07254E-44	4.31064E-43
CLDND1	1458.154617	0.481986069	0.034359558	14.02771447	1.05497E-44	4.24875E-43
KRAS	2345.26901	0.480759632	0.034269345	14.02885366	1.03816E-44	4.18968E-43
NOL6	1406.095128	-0.434531078	0.03096263	-14.03404952	9.64813E-45	3.90171E-43
NFS1	357.142985	-0.445913756	0.031764722	-14.03801855	9.12278E-45	3.6969E-43
PTCD2	284.8731964	-0.586037777	0.041733872	-14.04225747	8.59311E-45	3.48948E-43
HCN4	4.256434268	-1.629551697	0.115954529	-14.0533682	7.34556E-45	2.98908E-43
ZC3H7A	3129.578184	0.374578168	0.026651526	14.05466132	7.21261E-45	2.94109E-43
CKMT2	38.68250065	-0.979293499	0.069661405	-14.05790617	6.88946E-45	2.81518E-43
CYP26C1	12.99681495	-1.271979513	0.090451144	-14.06261392	6.44606E-45	2.63951E-43
RBFOX1	10.50125243	-1.732235621	0.123155604	-14.0654226	6.19517E-45	2.5421E-43
NCDN	541.1052603	-0.631319598	0.044872899	-14.06906193	5.88448E-45	2.41968E-43
AC092835.1	42.33929974	-0.93255612	0.066281056	-14.06972338	5.8297E-45	2.4022E-43
MPV17	1053.93831	-0.495351087	0.035203448	-14.0710957	5.71766E-45	2.36101E-43
RNF6	1428.178577	0.411312102	0.029229615	14.07175935	5.66425E-45	2.3439E-43
TM7SF2	354.5646872	-0.516613439	0.036704258	-14.0750273	5.4084E-45	2.24277E-43
RECQL4	635.8464489	-0.643204421	0.045696966	-14.07542941	5.37772E-45	2.23478E-43
DNM3	254.6778729	-1.274259368	0.090524952	-14.07633292	5.30943E-45	2.21109E-43
TBL3	736.8028586	-0.403514039	0.028662101	-14.07831342	5.16273E-45	2.15458E-43
DNMT3B	746.231068	-0.970512838	0.068916753	-14.08239349	4.87309E-45	2.03806E-43
HOMEZ	220.419575	-0.576039637	0.040901891	-14.08344752	4.80094E-45	2.01218E-43
KATNAL2	91.8753198	-0.651663064	0.046270058	-14.08390418	4.77001E-45	2.0035E-43

ZNF76	981.9309353	-0.364112232	0.025851543	-14.0847387	4.71399E-45	1.98424E-43
PHF14	1531.885846	-0.512493968	0.036383347	-14.08594899	4.63392E-45	1.95473E-43
FMO4	58.13613819	-0.732146259	0.051965476	-14.08908997	4.43236E-45	1.87375E-43
FAM185A	127.9076695	-0.58272795	0.041349606	-14.09270871	4.21095E-45	1.784E-43
UBE2D1	709.7982132	0.792667906	0.056227822	14.09743225	3.93841E-45	1.67216E-43
SLC1A6	8.216658903	-1.515561062	0.107483247	-14.10043988	3.7741E-45	1.60588E-43
ALDH5A1	773.8171408	-0.700123093	0.049652193	-14.10054722	3.76836E-45	1.60588E-43
FBH1	1612.841236	-0.423729281	0.030046477	-14.10246131	3.66752E-45	1.56734E-43
CA7	3.446800308	-1.582461236	0.112194969	-14.10456498	3.55978E-45	1.52463E-43
UQCC2	317.0189772	-0.854721807	0.060576437	-14.10980661	3.30483E-45	1.41854E-43
SLITRK5	90.29365906	-2.136731125	0.151415584	-14.11169888	3.21733E-45	1.38402E-43
MPI	688.4567178	-0.458612118	0.032494086	-14.11371032	3.12684E-45	1.34805E-43
CSDE1	24258.1348	0.374859771	0.026548744	14.11968015	2.87292E-45	1.24132E-43
IPO13	663.1337117	-0.601477898	0.042565958	-14.13049124	2.46419E-45	1.06707E-43
BTBD8	146.3270553	-0.795233446	0.056244835	-14.13878162	2.19044E-45	9.50631E-44
CCDC113	26.30062168	-0.862932296	0.061018696	-14.14209659	2.08965E-45	9.08903E-44
ACOX3	473.7184278	-0.48249188	0.034110214	-14.14508529	2.00274E-45	8.73042E-44
IFT46	302.7715576	-0.384870499	0.027194959	-14.15227343	1.80816E-45	7.8998E-44
SNRK	3085.801828	0.433181315	0.030601821	14.15540965	1.72927E-45	7.57204E-44
CYRIA	1461.941056	0.808296095	0.057077552	14.16136596	1.58876E-45	6.97236E-44
ZFP91	3954.735901	0.501423567	0.035400524	14.16429803	1.52382E-45	6.70241E-44
COA3	234.0220237	-0.934269874	0.065954752	-14.1653155	1.50191E-45	6.62091E-44
ZNF846	284.7140206	-0.50606063	0.035716444	-14.16884145	1.42838E-45	6.31097E-44
WDR24	304.1587715	-0.430400623	0.030369799	-14.17199424	1.36566E-45	6.04755E-44
ZNF202	269.2408458	-0.49076236	0.034626001	-14.17323247	1.34179E-45	5.9553E-44
WDR59	1429.880017	-0.412558885	0.029097763	-14.17837142	1.24707E-45	5.54748E-44
PRDM1	622.3458844	0.949989167	0.066958199	14.18779453	1.09034E-45	4.86134E-44
ZNF607	61.15555843	-0.833885553	0.058742389	-14.19563559	9.74984E-46	4.35693E-44
SPOCK2	2499.440191	1.052615167	0.074134556	14.19871151	9.33123E-46	4.17941E-44
PXDNL	15.97323245	-1.044165906	0.073535881	-14.19940701	9.23909E-46	4.14763E-44
PPOX	710.7055388	-0.692446959	0.048744816	-14.20555087	8.46345E-46	3.80816E-44
RASL10A	91.67988666	1.287238535	0.090610752	14.20624493	8.38001E-46	3.77931E-44
ZNF66	368.2033097	-0.69409882	0.0488568	-14.2068008	8.31377E-46	3.75809E-44
EARS2	378.1791228	-0.565610359	0.039743469	-14.23152983	5.83903E-46	2.64554E-44
FARSA	1342.116754	-0.548144283	0.038508671	-14.23430798	5.61157E-46	2.54838E-44
CNOT8	4050.2609	0.369162782	0.02592795	14.23802451	5.32101E-46	2.42205E-44
PLEKHA6	186.199475	-1.204488974	0.084590446	-14.23906629	5.24228E-46	2.39177E-44

MRPS25	1121.81933	-0.392035075	0.027531903	-14.23930159	5.22466E-46	2.38931E-44
PTP4A2	11352.24488	0.421527321	0.029584838	14.24808621	4.60735E-46	2.11194E-44
ATXN2	2195.482289	-0.369391634	0.025924955	-14.24849685	4.58034E-46	2.10448E-44
SH3TC2	287.3524942	-1.23038852	0.086323061	-14.25330043	4.27586E-46	1.96921E-44
NUDT6	67.52373799	-0.804558559	0.056437995	-14.25561912	4.13618E-46	1.90937E-44
VPS72	427.8398864	-0.652620412	0.045768578	-14.25913681	3.93289E-46	1.81982E-44
TP53	1616.850608	-0.609568483	0.042736237	-14.26350405	3.69429E-46	1.71347E-44
IFRD2	801.0223183	-0.573802987	0.040175467	-14.28242235	2.81639E-46	1.30939E-44
PGAP4	23.4417518	-1.831149235	0.128192705	-14.28434818	2.73961E-46	1.27672E-44
BTG1	12417.49987	0.605602957	0.042392308	14.28568036	2.68772E-46	1.25553E-44
MYO7B	485.5457766	-1.292084677	0.090433364	-14.28769889	2.61095E-46	1.22259E-44
SLC13A4	107.5089647	-0.943813156	0.066049137	-14.28956068	2.54208E-46	1.19319E-44
GATB	396.0604031	-0.494506428	0.034560261	-14.30852698	1.9357E-46	9.10756E-45
PYROXD1	548.1159705	0.445199375	0.031112048	14.30954921	1.90746E-46	8.9963E-45
COQ3	51.77808775	-0.654044463	0.045675347	-14.3194197	1.65499E-46	7.82444E-45
GVQW3	247.2334692	-0.806002927	0.056271592	-14.32344288	1.5619E-46	7.40218E-45
RORB	15.93720139	-1.81317343	0.12658599	-14.3236501	1.55725E-46	7.39805E-45
L3MBTL2	663.1060823	-0.341622899	0.023841989	-14.32862417	1.44964E-46	6.9036E-45
PPP1R2	2956.024814	0.456608687	0.031855693	14.33366027	1.34823E-46	6.43633E-45
MSH5	231.3237388	-0.561146411	0.039139345	-14.33714358	1.28225E-46	6.13632E-45
YPEL2	2936.747205	0.572559323	0.039922357	14.34182171	1.19867E-46	5.75039E-45
SPATA13	1589.283056	0.561036332	0.039113751	14.34371089	1.16648E-46	5.60969E-45
MORC2	945.8936241	-0.47048748	0.032800222	-14.34403357	1.16106E-46	5.59741E-45
KAT2A	1822.148765	-0.617775635	0.043055828	-14.34824642	1.09265E-46	5.28061E-45
ZNF665	77.39265596	-0.621166457	0.043279028	-14.35259724	1.02621E-46	4.97178E-45
SP3	6853.058704	0.387422035	0.026978268	14.36052272	9.15342E-47	4.44565E-45
TNFRSF1B	3573.010548	1.011449007	0.070428367	14.36138657	9.04002E-47	4.4015E-45
MAP3K1	4203.125066	0.52668275	0.036664781	14.36481359	8.60379E-47	4.19955E-45
BZW1	5611.485511	0.404205857	0.028122929	14.37282234	7.66431E-47	3.75034E-45
HIF1A	13407.19681	0.742721328	0.051568741	14.40254915	4.98697E-47	2.44636E-45
CCDC180	62.1660848	-0.92835118	0.064446725	-14.40493949	4.8174E-47	2.36912E-45
FKBP10	68.00901772	-1.488752525	0.103323321	-14.4086786	4.56358E-47	2.24995E-45
FAM161A	82.89782546	-0.666180953	0.046212846	-14.41549284	4.13477E-47	2.04368E-45
CC2D1A	710.8457869	-0.444915018	0.030861115	-14.41668644	4.0639E-47	2.01374E-45
LMBRD1	1340.377279	0.405715378	0.028127044	14.42438743	3.63483E-47	1.8057E-45
CD6	1208.972792	1.111362222	0.077038536	14.42605581	3.54799E-47	1.76704E-45
C12orf66	303.4409127	-0.521546763	0.036142154	-14.43042821	3.33007E-47	1.66274E-45

GSTA4	35.99563655	-1.048654688	0.072669339	-14.43049715	3.32675E-47	1.66274E-45
MYOM1	145.603187	-1.002223715	0.069423954	-14.43628119	3.05908E-47	1.53527E-45
TXNDC11	3106.928355	0.483717106	0.033500473	14.43911257	2.93598E-47	1.47727E-45
ASIC4	19.91600466	-1.348552958	0.093366154	-14.4437025	2.74681E-47	1.38565E-45
SDC4	679.1796478	1.288395612	0.089148762	14.45219865	2.42809E-47	1.22804E-45
ARL8B	4337.430436	0.455793483	0.031523947	14.45864269	2.21115E-47	1.12121E-45
MYH6	6.397433033	-1.875173865	0.129592787	-14.46973949	1.88184E-47	9.56706E-46
ZDHHC16	556.2478644	-0.425463785	0.029403269	-14.46994862	1.87612E-47	9.56285E-46
ZNF221	59.79543579	-0.838672239	0.057953888	-14.47137143	1.83771E-47	9.39152E-46
WDR4	496.7214332	-0.562110157	0.038823716	-14.47852545	1.65612E-47	8.48566E-46
DNAJC3	3973.554549	0.543622198	0.03751667	14.49015049	1.39836E-47	7.18376E-46
TTLL11	84.97719656	-0.722130647	0.049824088	-14.49360491	1.32977E-47	6.84935E-46
DSC3	2.859664751	-2.282214526	0.15743562	-14.49617643	1.28089E-47	6.615E-46
ZNF248	245.4219848	-0.688896238	0.047518381	-14.49746858	1.25701E-47	6.50884E-46
PWWP3A	738.3776	-0.677294573	0.046667423	-14.51322005	9.99178E-48	5.18751E-46
MYH3	167.7374067	-0.595139622	0.04100465	-14.51395429	9.88537E-48	5.14591E-46
CSTF3	397.1229263	-0.529614286	0.036465955	-14.52352724	8.59702E-48	4.48719E-46
DCAF7	2826.862374	-0.530464256	0.036521143	-14.52485358	8.43223E-48	4.41294E-46
GCDH	713.9904793	-0.442280721	0.030421885	-14.53824186	6.9351E-48	3.63916E-46
GLI4	226.6768597	-0.534361512	0.036741746	-14.54371577	6.4021E-48	3.36851E-46
BAIAP2L2	47.84110536	-0.748830127	0.051479378	-14.54621556	6.17246E-48	3.25643E-46
RPAP1	720.1263741	-0.476973708	0.032782342	-14.54971433	5.86474E-48	3.10245E-46
CPS1	14.9402098	-1.561122495	0.107273038	-14.55279468	5.6065E-48	2.97387E-46
KANK1	424.0525055	-1.009809302	0.069382417	-14.55425373	5.48816E-48	2.91901E-46
MMAB	240.5794059	-0.534616723	0.036731742	-14.55462491	5.45845E-48	2.91112E-46
RNF214	548.3541696	-0.37984988	0.026092284	-14.55793898	5.20021E-48	2.78098E-46
ICOS	141.3211566	1.265038628	0.086886488	14.55967043	5.07016E-48	2.71886E-46
S1PR1	729.5680363	1.123494433	0.077124572	14.56726961	4.53664E-48	2.43944E-46
NUDT8	135.5593231	-0.88673811	0.060848982	-14.57276831	4.18579E-48	2.25698E-46
PLD6	75.46055443	-0.664186701	0.045538656	-14.58511876	3.49314E-48	1.88871E-46
PXMP4	163.4801457	-0.668967239	0.045841373	-14.59308892	3.10801E-48	1.68512E-46
AP1G2	1282.560491	-0.462083564	0.031664335	-14.59318692	3.10354E-48	1.68512E-46
ANXA8	37.81197215	1.89008269	0.129486505	14.59675429	2.94538E-48	1.60585E-46
PRMT5	389.430575	-0.457105737	0.031312353	-14.59825595	2.88122E-48	1.57526E-46
FER1L6	20.00795653	-1.674452243	0.114669044	-14.6024785	2.70818E-48	1.4848E-46
POLR3A	786.2345107	-0.505865794	0.034642303	-14.60254503	2.70554E-48	1.4848E-46
PFAS	1581.949437	-0.543674959	0.037218371	-14.60770417	2.50829E-48	1.38295E-46

DROSHA	1558.616874	-0.472904538	0.032359805	-14.6139489	2.28859E-48	1.26538E-46
MTHFSD	387.9426446	-0.481197289	0.032914993	-14.61939506	2.11269E-48	1.17144E-46
ZNF343	111.4192533	-0.543078344	0.037116302	-14.63180102	1.76064E-48	9.79006E-47
FBXW8	599.8643622	-0.503385741	0.034398665	-14.63387452	1.70778E-48	9.5232E-47
FASTKD1	567.5095628	-0.540773899	0.036945376	-14.63711985	1.62821E-48	9.1054E-47
THEM6	231.2253553	-0.705037261	0.048108643	-14.65510595	1.24962E-48	7.00823E-47
GATD1	1100.208657	-0.52809651	0.036034523	-14.65529362	1.24617E-48	7.00823E-47
ZFYVE19	483.2307926	-0.505580758	0.034492248	-14.65780817	1.20087E-48	6.77368E-47
CEP89	346.7590571	-0.482829358	0.03293298	-14.6609677	1.14628E-48	6.48441E-47
TMEM186	61.85745854	-0.535227701	0.036489827	-14.66786094	1.03559E-48	5.87522E-47
ULK3	1035.135421	-0.432303327	0.029462072	-14.67321536	9.57005E-49	5.44519E-47
NPAS3	6.692425373	-1.851485058	0.126152854	-14.67652134	9.11481E-49	5.20129E-47
SLC7A9	7.48147101	-0.997797392	0.067975259	-14.67883188	8.8095E-49	5.04176E-47
LRRC61	79.57651991	-1.011668439	0.068916937	-14.67953291	8.7189E-49	5.00455E-47
STXBP4	239.3819211	-0.730595743	0.049750023	-14.68533477	8.0038E-49	4.6076E-47
C5orf63	126.5973511	-0.788026831	0.053648004	-14.68883789	7.60061E-49	4.3884E-47
CEP104	1006.993258	-0.451571937	0.030739576	-14.69024596	7.44431E-49	4.31087E-47
FLAD1	635.3095459	-0.431160053	0.029346362	-14.69211265	7.24202E-49	4.20617E-47
MTPN	9721.194216	0.406382342	0.027635097	14.70529805	5.96079E-49	3.47234E-47
AFAP1L2	40.82175497	-1.271564135	0.086372018	-14.72194543	4.66057E-49	2.72303E-47
OTOG	5.581339109	-2.00411507	0.136065921	-14.72900092	4.19867E-49	2.4605E-47
TMOD4	47.51619908	-0.635762209	0.043160056	-14.73033779	4.11643E-49	2.41955E-47
ARPP19	5210.695776	0.407516287	0.027653776	14.73637019	3.76483E-49	2.21955E-47
TRAM1	4828.651536	0.51438225	0.034877389	14.74830152	3.15505E-49	1.86568E-47
FREM2	4.7348574	-2.627552842	0.178127555	-14.75096223	3.03309E-49	1.79899E-47
INTS7	738.5873027	-0.653779704	0.044320388	-14.75121796	3.02162E-49	1.79763E-47
RUVBL1	710.0244896	-0.55239476	0.037438638	-14.75467032	2.87091E-49	1.71318E-47
DGLUCY	2094.34759	-0.664959321	0.045049439	-14.76065698	2.62709E-49	1.57248E-47
ELAC2	1731.438585	-0.377266578	0.025552764	-14.76421806	2.49196E-49	1.49617E-47
ZNF837	45.04569375	-0.657392403	0.04449714	-14.77381243	2.16133E-49	1.30165E-47
AKAP1	600.2194366	-0.664129571	0.044935787	-14.77952467	1.98563E-49	1.19953E-47
ZNF324B	120.2413012	-0.629316606	0.04257521	-14.78129204	1.9342E-49	1.17208E-47
AFMID	347.5854209	-0.647203629	0.043777172	-14.78404365	1.85676E-49	1.12864E-47
ADAL	147.5667662	-0.538476646	0.036399062	-14.7936957	1.60873E-49	9.80922E-48
NUDCD3	1430.103016	-0.430813999	0.029090602	-14.80938772	1.27395E-49	7.79222E-48
YWHAZ	20737.61144	0.342861781	0.023144325	14.81407578	1.18812E-49	7.29E-48
INTS3	2263.307366	-0.65516083	0.044180007	-14.82935086	9.46437E-50	5.82535E-48

MRI1	408.3436514	-0.531185731	0.035809348	-14.83371698	8.86833E-50	5.47571E-48
EMILIN3	7.795722634	-2.281595063	0.153809958	-14.83385796	8.84972E-50	5.47571E-48
TADA2A	409.4327955	-0.496093127	0.033433542	-14.83818615	8.29691E-50	5.15541E-48
TRPC3	9.543600287	-1.651063063	0.111187562	-14.8493504	7.0246E-50	4.37874E-48
DRP2	20.69411977	-1.01832888	0.068576661	-14.8494963	7.00933E-50	4.37874E-48
PAX6	6.686330399	-1.28869754	0.086782792	-14.84968975	6.98914E-50	4.37874E-48
LSM14A	4940.005986	0.346289999	0.023318736	14.85029027	6.92681E-50	4.35943E-48
SIRT5	360.2447522	-0.53042082	0.035712643	-14.85246622	6.70557E-50	4.23381E-48
TMX4	3403.161821	0.505855071	0.034037672	14.86162358	5.84903E-50	3.70495E-48
JPT2	546.6921984	-0.654768213	0.044053132	-14.86314784	5.71743E-50	3.63336E-48
ADPRHL1	739.7337367	1.649647106	0.110983324	14.86391874	5.65201E-50	3.60348E-48
RASSF5	4369.858492	0.601477587	0.040462272	14.86514628	5.54936E-50	3.54959E-48
COA4	451.9842874	-0.629300268	0.042308947	-14.87392903	4.86711E-50	3.12341E-48
SMARCAL1	698.2890446	-0.613111013	0.041212487	-14.87682642	4.6609E-50	3.00091E-48
VKORC1L1	474.2485817	-0.644850977	0.043344697	-14.87727505	4.62975E-50	2.9907E-48
PJA2	7104.322814	0.465220913	0.031268329	14.87834266	4.55647E-50	2.95311E-48
UBE3B	1224.049095	-0.505078905	0.033946653	-14.87860671	4.53853E-50	2.95125E-48
EDC3	574.8256141	-0.445555579	0.029942737	-14.88025555	4.42805E-50	2.88901E-48
XRCC2	238.9689489	-0.722233878	0.048530643	-14.88201757	4.31294E-50	2.82332E-48
ING4	519.4484735	-0.539359334	0.036218083	-14.89199032	3.71542E-50	2.44033E-48
DPH2	378.6259944	-0.653769709	0.043895286	-14.8938478	3.6136E-50	2.38145E-48
SPAG17	20.95965494	-1.428023273	0.09586281	-14.89653052	3.47143E-50	2.29549E-48
USH2A	25.97435328	-1.727908641	0.115968505	-14.89980956	3.3052E-50	2.19297E-48
ZNF573	143.5877494	-0.753042586	0.05050133	-14.91134171	2.78107E-50	1.8515E-48
DET1	55.90921275	-0.879126989	0.058944314	-14.91453411	2.65121E-50	1.77106E-48
DDX23	1243.651789	-0.42905078	0.028759116	-14.91877482	2.488E-50	1.66773E-48
SCFD2	608.7766985	-0.696294594	0.046633993	-14.93105245	2.06973E-50	1.39213E-48
PIR	38.83158611	-1.513316794	0.101273427	-14.94288132	1.73316E-50	1.16977E-48
GALT	389.7228456	-0.48648777	0.032524912	-14.95738922	1.39388E-50	9.44028E-49
TWNK	194.9915354	-0.708282034	0.047351816	-14.95786406	1.38397E-50	9.40572E-49
ZNF584	129.5388929	-0.539831573	0.036078952	-14.96250685	1.29071E-50	8.80246E-49
RDM1	14.24310675	-1.036133425	0.069157721	-14.98217994	9.60153E-51	6.57101E-49
MUS81	728.8436027	-0.411497778	0.027453295	-14.98901234	8.66319E-51	5.94964E-49
ZSCAN5A	144.4505714	-0.63438509	0.042313084	-14.99264597	8.20195E-51	5.6527E-49
ELP3	604.9244481	-0.527765172	0.035199939	-14.99335485	8.11486E-51	5.61244E-49
ARHGEF9	451.2023499	-0.5243414	0.034964955	-14.99619823	7.77468E-51	5.39624E-49
CCDC102B	36.55771928	-0.87531718	0.058339042	-15.00396913	6.91571E-51	4.81712E-49

RAD52	593.5189646	-0.526759046	0.03510482	-15.00531955	6.77638E-51	4.73693E-49
IL10RA	4371.172213	0.939712898	0.062607273	15.00964434	6.34872E-51	4.45389E-49
GOLT1B	925.3629543	0.515427354	0.034329698	15.01403697	5.94186E-51	4.18345E-49
COL11A2	50.27920854	-0.943544635	0.062810401	-15.02210824	5.26075E-51	3.71728E-49
CBY1	78.31160614	-0.523867721	0.034866598	-15.02491629	5.0425E-51	3.57597E-49
CCDC142	276.9661118	-0.63033525	0.041938372	-15.0300361	4.66751E-51	3.32208E-49
TERT	73.11795261	-1.369542975	0.091119588	-15.03017086	4.65803E-51	3.32208E-49
BCL10	1194.124326	0.408341364	0.027153815	15.03808459	4.13337E-51	2.96346E-49
EZR	6852.418815	0.668478228	0.044441368	15.04180132	3.9077E-51	2.81197E-49
ZNF284	36.86786926	-0.802814622	0.053344953	-15.0494953	3.47878E-51	2.51255E-49
NEURL4	737.724217	-0.607538003	0.040366704	-15.05047338	3.42773E-51	2.48485E-49
RBMXL1	935.0874347	0.401629028	0.026665922	15.06150899	2.90089E-51	2.11075E-49
RPGRIP1L	97.80024985	-0.715003841	0.047467841	-15.06291052	2.84004E-51	2.07418E-49
LACTB	1096.213192	0.564415342	0.037463115	15.06589469	2.71466E-51	1.99004E-49
MMGT1	1683.376599	0.445037192	0.029536066	15.06758523	2.64609E-51	1.94706E-49
ZMYND8	2562.465028	-0.674017591	0.044700563	-15.07850354	2.24295E-51	1.65665E-49
HIBCH	448.2324622	-0.603906879	0.040030974	-15.08599001	2.00248E-51	1.48464E-49
RFT1	522.4020682	-0.489887926	0.032430761	-15.10565617	1.4862E-51	1.10606E-49
PYCR2	487.2532658	-0.473591787	0.031326661	-15.11785065	1.23508E-51	9.22684E-50
DISP2	117.175613	-0.89913273	0.059453049	-15.12340813	1.13512E-51	8.51256E-50
TMEM127	4509.312909	0.494203983	0.032667465	15.12832382	1.05345E-51	7.93042E-50
TAF6L	256.6002139	-0.47813549	0.031594888	-15.13331789	9.76456E-52	7.37922E-50
DNAAF5	508.5533369	-0.545654944	0.036037557	-15.14128552	8.65061E-52	6.56273E-50
HAUS5	802.5300428	-0.41994442	0.02773296	-15.14243026	8.50132E-52	6.47456E-50
NUS1	1038.108574	0.376902528	0.024878761	15.14957014	7.62638E-52	5.8309E-50
KIAA0319L	1888.687767	-0.439537448	0.029006842	-15.15288865	7.25083E-52	5.56551E-50
SYNE4	13.48955588	-0.749676838	0.049467458	-15.15494968	7.02692E-52	5.41488E-50
TMEM182	75.07249318	-0.810567126	0.05345782	-15.16274198	6.24085E-52	4.82815E-50
GRAMD2A	13.87593244	-1.185848327	0.078199475	-15.16440272	6.08499E-52	4.72625E-50
PMS1	454.3445951	-0.56405507	0.037188301	-15.16754056	5.80101E-52	4.52363E-50
CD300E	808.5418392	1.788210499	0.11770586	15.19219604	3.98348E-52	3.11874E-50
PLAC8L1	10.98750715	-0.861265153	0.056671645	-15.19746172	3.67591E-52	2.8895E-50
PDZRN4	19.60103331	-2.19396487	0.14431275	-15.20284852	3.38573E-52	2.67213E-50
WTAP	4190.762022	0.442195643	0.029081875	15.20519703	3.26647E-52	2.58844E-50
IPO9	2189.243038	-0.382142467	0.025126291	-15.20886909	3.08832E-52	2.45722E-50
RYR3	632.3301423	-1.447887181	0.095022004	-15.23738847	1.99694E-52	1.59535E-50
PLCB4	160.0302492	-2.044782193	0.13414666	-15.24288563	1.8358E-52	1.47263E-50

RASEF	5.557734569	-1.81547135	0.119100487	-15.24318998	1.82727E-52	1.47182E-50
TRAPPC12	1218.489668	-0.530422999	0.034781708	-15.2500563	1.64491E-52	1.33041E-50
URB1	1271.049035	-0.767959484	0.050312775	-15.26370745	1.33446E-52	1.08379E-50
TBCD	1697.556841	-0.523073052	0.034266901	-15.26467341	1.31484E-52	1.07231E-50
SLC27A5	142.9958409	-0.657988589	0.04309767	-15.26738211	1.26136E-52	1.033E-50
EFCAB11	98.96309654	-0.5511295	0.036085923	-15.27270051	1.16257E-52	9.56093E-51
ZKSCAN7	79.43211021	-0.891293743	0.058347577	-15.27559148	1.11214E-52	9.1848E-51
COL11A1	15.34631498	-2.713462923	0.177594536	-15.27897748	1.05584E-52	8.75679E-51
FAM227A	60.48319237	-0.952044504	0.062293383	-15.28323657	9.89041E-53	8.23766E-51
PSMG4	442.4141144	-0.59068671	0.038642947	-15.28575739	9.51503E-53	7.95887E-51
CDV3	7360.59124	0.340223307	0.022253545	15.28849904	9.12286E-53	7.66359E-51
CYTIP	3678.983767	0.776521742	0.050777579	15.29261044	8.56471E-53	7.22574E-51
TMEM42	193.2798164	-0.545683484	0.035676701	-15.29523401	8.22646E-53	6.97041E-51
TNFSF8	425.0912936	0.987323386	0.064533096	15.29948891	7.70594E-53	6.55775E-51
CCDC81	24.4028711	-0.88853947	0.057941068	-15.3352277	4.44711E-53	3.80101E-51
SOAT1	1970.864598	0.535776033	0.034936117	15.33587807	4.40279E-53	3.77964E-51
GEMIN6	97.0165296	-0.790071369	0.051494366	-15.3428701	3.95323E-53	3.40866E-51
CFAP298	90.65112221	-0.779174203	0.050747611	-15.35390894	3.33473E-53	2.88808E-51
HECA	3491.757672	0.646254851	0.042079151	15.35807708	3.12713E-53	2.72033E-51
AAMDC	98.09478036	-0.530644696	0.034550861	-15.35836385	3.11333E-53	2.72033E-51
ACTR3B	171.2794187	-0.566555108	0.036815309	-15.38911725	1.93656E-53	1.69975E-51
GAB2	8000.106318	0.726500166	0.047166309	15.40294713	1.56377E-53	1.37872E-51
NDUFAF1	182.6608128	-0.562709287	0.036530302	-15.40390443	1.54078E-53	1.3646E-51
SARAF	8384.345689	0.509235539	0.033058016	15.40429832	1.53142E-53	1.36248E-51
LYN	8540.396441	0.549705872	0.035636132	15.42552009	1.10263E-53	9.85472E-52
RTKN	138.3736378	-0.870340086	0.056419722	-15.42616755	1.09163E-53	9.80113E-52
CYB5RL	230.7675454	-0.708517601	0.045910423	-15.43260879	9.87946E-54	8.91109E-52
PTPN1	3441.970909	0.472151307	0.030546135	15.45699013	6.76883E-54	6.13362E-52
GEMIN5	558.337904	-0.571999353	0.036977973	-15.46865101	5.64782E-54	5.14162E-52
KRI1	980.3161736	-0.505619609	0.032680484	-15.47160705	5.39434E-54	4.9338E-52
CDK10	1063.737891	-0.52521695	0.033944439	-15.47284225	5.29181E-54	4.86275E-52
KLHL33	78.8572334	-1.4165742	0.091503721	-15.48105562	4.65767E-54	4.30022E-52
FBF1	143.2029904	-0.512770025	0.033120062	-15.48215766	4.57855E-54	4.24721E-52
LIPE	124.2329755	-0.807234669	0.052131897	-15.48446767	4.41703E-54	4.11689E-52
PDIA3	9556.660051	0.535069008	0.034540784	15.49093394	3.99445E-54	3.74084E-52
CAD	2296.269764	-0.735978669	0.047452877	-15.50967435	2.98388E-54	2.80786E-52
POLR1G	25.24686738	-0.808309268	0.052115022	-15.51010125	2.96411E-54	2.80273E-52

TIMP3	391.900207	-1.770812562	0.114135385	-15.51501807	2.74557E-54	2.6087E-52
FBXL3	1341.089724	0.562317282	0.036241602	15.51579532	2.71253E-54	2.58987E-52
NOP56	2269.541871	-0.620634705	0.039986992	-15.52091512	2.50454E-54	2.403E-52
KBTBD12	23.09026993	-1.474315064	0.09496078	-15.52551557	2.33123E-54	2.24774E-52
IGF2BP1	272.9271752	-2.685933014	0.172941236	-15.5308998	2.14351E-54	2.07698E-52
PLEKHF2	1536.141077	0.57214336	0.036795685	15.54919716	1.61113E-54	1.56889E-52
IFT27	364.5375185	-0.681766541	0.043833914	-15.55340327	1.50871E-54	1.4765E-52
MAMSTR	24.29226407	-0.830910184	0.053393973	-15.5618723	1.32175E-54	1.30003E-52
PIGM	393.6011285	-0.687103859	0.044152746	-15.56197338	1.31967E-54	1.30003E-52
SEMA6B	519.0337004	1.428333774	0.091775836	15.56328814	1.29283E-54	1.28449E-52
LRRC46	48.96642493	-0.604741574	0.03885574	-15.5637643	1.28325E-54	1.28148E-52
ERCC2	637.3015944	-0.571704629	0.036731186	-15.56455666	1.26745E-54	1.2722E-52
ZNF594	101.3947356	-0.969415414	0.062257192	-15.57113934	1.14352E-54	1.15372E-52
ZDHHC7	3474.712965	0.495240644	0.031801455	15.5728925	1.1126E-54	1.12834E-52
GMFB	1798.418323	0.479094875	0.030762831	15.57382281	1.09653E-54	1.11783E-52
ZNF843	29.60966342	-0.853916625	0.054821308	-15.57636345	1.05382E-54	1.07992E-52
C12orf45	519.3305061	-0.676738138	0.043411091	-15.58906066	8.63946E-55	8.90001E-53
ADCK1	213.9483451	-0.715022355	0.045838753	-15.59864308	7.43578E-55	7.70056E-53
PBX1	535.7841064	-2.088482567	0.133802873	-15.60865265	6.35652E-55	6.61788E-53
ZSCAN20	54.50054676	-0.919463786	0.058898629	-15.61095396	6.13133E-55	6.41757E-53
RECQL5	811.3946275	-0.518899061	0.033212773	-15.62347893	5.03801E-55	5.30156E-53
ZNF2	41.11891452	-0.914393943	0.058461109	-15.64106394	3.82287E-55	4.0446E-53
MTG1	144.2934244	-0.475659344	0.030389752	-15.65196511	3.22117E-55	3.42652E-53
PIK3IP1	3144.083264	0.843790891	0.053900623	15.65456662	3.09212E-55	3.30722E-53
WDR3	411.8940068	-0.639208437	0.040831788	-15.65467643	3.08679E-55	3.30722E-53
GPRIN3	844.1849014	1.127988987	0.072040384	15.65773138	2.94206E-55	3.18149E-53
ZSWIM3	67.92554069	-0.803706705	0.051315222	-15.66215008	2.74459E-55	2.98444E-53
ELP6	361.7104546	-0.523891307	0.033435491	-15.66871891	2.47518E-55	2.70652E-53
TRPC6	57.85461941	-2.682323094	0.17090693	-15.69464211	1.64568E-55	1.8096E-53
EHMT2	1515.87885	-0.467908896	0.029795427	-15.70405046	1.41886E-55	1.569E-53
CNEP1R1	472.5843023	0.479417149	0.030523248	15.70662312	1.36245E-55	1.51519E-53
TICRR	346.9315335	-0.810614098	0.051601955	-15.70898037	1.31273E-55	1.46824E-53
ZNF610	57.69653366	-0.88426864	0.056262989	-15.7167021	1.16218E-55	1.30731E-53
RIC8B	199.0011277	-0.71536159	0.045501263	-15.72179648	1.07239E-55	1.21329E-53
ZNF771	66.26534723	-0.570453167	0.036281273	-15.72307455	1.05097E-55	1.19597E-53
ENTR1	610.6261927	-0.434746503	0.027586068	-15.75963997	5.8968E-56	6.74959E-54
GBP2	2528.991332	0.885495001	0.056142902	15.77216293	4.83647E-56	5.56848E-54

SERINC1	4660.930758	0.509918022	0.032165693	15.85285356	1.3434E-56	1.55588E-54
MMACHC	156.0926525	-0.707452014	0.044621338	-15.85456754	1.30724E-56	1.52302E-54
PDZD2	248.5437749	-1.050351868	0.066189817	-15.86878343	1.04242E-56	1.22175E-54
LIPH	27.80042535	-1.633794846	0.102854456	-15.88453153	8.11016E-57	9.56266E-55
KAT14	221.3263406	-0.711947204	0.044804085	-15.89022976	7.40556E-57	8.78479E-55
ACTL6B	6.999681833	-1.356627362	0.085284862	-15.90701243	5.66532E-57	6.76142E-55
RBM28	1310.166844	-0.513187119	0.032257475	-15.90909148	5.4803E-57	6.58073E-55
FAT4	66.99398474	-1.934055379	0.121403713	-15.93077617	3.87517E-57	4.68202E-55
SMUG1	154.0508049	-0.743565375	0.046637654	-15.94345587	3.16364E-57	3.84608E-55
ACYP1	128.7689934	-0.847465418	0.05315384	-15.94363475	3.15459E-57	3.84608E-55
KHK	41.69384326	-1.128803418	0.07072231	-15.96106542	2.38623E-57	2.93746E-55
WDR93	10.76826823	-1.083341859	0.067849554	-15.96682349	2.17588E-57	2.69548E-55
POMGNT1	639.9000337	-0.575261101	0.03601785	-15.97155564	2.01692E-57	2.51447E-55
ALG1L2	35.8885915	-0.796647086	0.049861466	-15.97720943	1.8421E-57	2.31124E-55
TRUB2	555.904521	-0.536266918	0.033550059	-15.98408249	1.64978E-57	2.0833E-55
STAC	46.40847017	-2.428045058	0.151775058	-15.9976553	1.32679E-57	1.68631E-55
COQ8B	787.6966115	-0.47259008	0.029524707	-16.00659702	1.14926E-57	1.47023E-55
KNG1	5.794123619	-1.374239048	0.085835613	-16.01012675	1.08588E-57	1.39829E-55
EIF2B3	321.6451008	-0.674293524	0.042116326	-16.01026454	1.08348E-57	1.39829E-55
GART	1588.644477	-0.495365709	0.030932213	-16.01455791	1.01123E-57	1.31952E-55
BNIP2	5534.213514	0.39871379	0.024893354	16.0168769	9.74221E-58	1.27976E-55
TSPAN11	25.68113835	-1.457036113	0.090923549	-16.02484858	8.56994E-58	1.13337E-55
EXOSC5	450.4977593	-0.877156407	0.054703067	-16.03486692	7.29397E-58	9.71189E-56
COQ9	325.1258359	-0.674374736	0.042045283	-16.03924829	6.79721E-58	9.11245E-56
NEK8	173.3912249	-0.632045144	0.039403548	-16.04031057	6.68194E-58	9.01969E-56
EEF1AKMT1	75.92781447	-0.830328507	0.051721379	-16.05387414	5.37046E-58	7.29972E-56
ZNF785	218.120772	-0.700301536	0.043616874	-16.05574799	5.21069E-58	7.13209E-56
C16orf95	28.83219711	-1.086907689	0.067683175	-16.05875733	4.96397E-58	6.84224E-56
TMED5	2948.786063	0.477489205	0.029725757	16.06314707	4.62479E-58	6.41993E-56
CTNND2	14.73084763	-2.407741838	0.149824287	-16.07043753	4.11174E-58	5.74851E-56
ZFP62	342.4838171	-0.578045459	0.035967145	-16.07148593	4.04278E-58	5.69276E-56
TEDC2	97.40069171	-0.839567686	0.05221644	-16.07860837	3.60384E-58	5.11144E-56
CEL	54.465518	-1.056111676	0.065682851	-16.07895614	3.58367E-58	5.11144E-56
CASP7	590.3498485	0.561972894	0.034947573	16.08045558	3.49797E-58	5.03425E-56
PARS2	29.08236713	-1.070875826	0.066534156	-16.0951291	2.75995E-58	4.00152E-56
GPR135	126.0115772	-1.051669218	0.065266577	-16.11344233	2.05272E-58	2.99834E-56
DNASE1	812.2785549	-0.900144659	0.055839921	-16.12009205	1.84334E-58	2.71277E-56

RABL2A	255.4436318	-0.641179331	0.03975543	-16.12809448	1.6194E-58	2.40126E-56
ERMN	385.4595753	1.097674262	0.067998317	16.14266818	1.27891E-58	1.91085E-56
PGBD2	102.4371008	-0.942071607	0.05835291	-16.14438084	1.2439E-58	1.87284E-56
MTG2	322.5332463	-0.485215277	0.030022465	-16.16173987	9.38744E-59	1.42434E-56
CXCL16	876.212948	1.601030684	0.099044087	16.16482858	8.92859E-59	1.36531E-56
DCAKD	520.4621272	-0.508399379	0.031436433	-16.17229862	7.90915E-59	1.21894E-56
IL11	91.72874439	1.893786336	0.117012077	16.18453746	6.48353E-59	1.00716E-56
ZNF283	136.7737612	-0.773802216	0.047739631	-16.20880165	4.37007E-59	6.84283E-57
SPAG8	53.48314991	-0.737818282	0.045455976	-16.23149135	3.02031E-59	4.76746E-57
QTRT1	662.4169589	-0.570149614	0.035081436	-16.25217436	2.15581E-59	3.43054E-57
LBHD1	192.0451966	-0.58152003	0.03575286	-16.2649934	1.74886E-59	2.80577E-57
CFAP161	53.06290534	-1.750449479	0.107563606	-16.27362206	1.519E-59	2.45715E-57
THBS1	5850.468352	1.938227604	0.118911119	16.29980121	9.90136E-60	1.61499E-57
PRSS2	22.34431041	-2.176834508	0.133394882	-16.31872585	7.26359E-60	1.19471E-57
TCEANC2	482.013813	-0.650806211	0.03980004	-16.35189835	4.21637E-60	6.99382E-58
KNOP1	236.8240778	-0.60444839	0.03687897	-16.39005596	2.25237E-60	3.76801E-58
CCHCR1	419.9603764	-0.553272114	0.033727712	-16.40408105	1.78812E-60	3.01714E-58
TONSL	774.82181	-0.716420185	0.043670658	-16.40506976	1.75925E-60	2.99424E-58
CSGALNACT2	4199.208282	0.631277377	0.038462744	16.41269716	1.55157E-60	2.66393E-58
RAB40A	8.303995913	-1.233833172	0.075173652	-16.41310662	1.54114E-60	2.66393E-58
RAD51D	578.2427424	-0.611121371	0.037226953	-16.41609969	1.46699E-60	2.56369E-58
PUS1	513.2428833	-0.532650577	0.032404494	-16.4375525	1.02995E-60	1.81615E-58
THAP8	66.08014687	-0.796507628	0.048449982	-16.43979215	9.92588E-61	1.76617E-58
NRAS	3764.947916	0.505481082	0.030733262	16.44736201	8.76015E-61	1.57305E-58
STK36	458.766877	-0.729869178	0.044349855	-16.45708142	7.46123E-61	1.35221E-58
GRK4	189.7710074	-0.676619599	0.041033231	-16.48955217	4.3617E-61	7.97865E-59
PDCD11	1830.743457	-0.522543924	0.03168064	-16.49410884	4.04483E-61	7.46882E-59
CCDC30	156.0746244	-0.75953617	0.046031317	-16.5004223	3.64336E-61	6.79156E-59
MEN1	724.0020168	-0.616204917	0.037329285	-16.50727873	3.25221E-61	6.12073E-59
MARCHF4	4.942317881	-2.144887827	0.129488911	-16.5642587	1.26327E-61	2.40059E-59
ZNF696	177.2926943	-0.746877612	0.045077433	-16.56876981	1.17199E-61	2.24896E-59
CENPT	766.2313194	-0.519209723	0.0313316	-16.57143999	1.12109E-61	2.17258E-59
HCN3	86.27136184	-0.73178556	0.044091368	-16.59702545	7.32312E-62	1.43335E-59
LRP2	22.00551212	-1.564098663	0.094124458	-16.61734583	5.21927E-62	1.03189E-59
SLC13A3	27.70757543	-1.878061114	0.112910331	-16.63320888	4.00555E-62	8.00007E-60
PAAF1	388.8536895	-0.570134273	0.034222799	-16.65948704	2.5823E-62	5.21065E-60
HEMK1	897.4121705	-0.657444291	0.039441904	-16.66867528	2.21448E-62	4.515E-60

SNN	1947.209907	0.766461709	0.045900132	16.69846415	1.34482E-62	2.77075E-60
FAM216A	224.5325735	-0.649411161	0.038737591	-16.76436611	4.44732E-63	9.26036E-61
FUT2	31.96197497	-0.953248411	0.056852343	-16.76709118	4.24802E-63	8.94048E-61
ACAD10	870.7080296	-0.573145612	0.034179606	-16.76864313	4.13852E-63	8.80469E-61
TSNAXIP1	32.05457109	-0.748858798	0.044643712	-16.77411587	3.77435E-63	8.11817E-61
PYCR3	124.2002072	-0.795617854	0.047405351	-16.78329229	3.23399E-63	7.03321E-61
FOXRED1	525.0696337	-0.404648765	0.024005958	-16.85618041	9.45023E-64	2.07831E-61
UHRF1BP1L	2483.591429	0.56301444	0.033331043	16.89159383	5.18823E-64	1.15397E-61
ZKSCAN3	214.4180212	-0.905417286	0.053564619	-16.90327122	4.25625E-64	9.57559E-62
POP1	124.6006078	-0.736379138	0.043507612	-16.92529414	2.9288E-64	6.66575E-62
RAP2C	2067.497162	0.543149575	0.032015425	16.96524647	1.48475E-64	3.41895E-62
KLHL31	12.64652497	-1.262855703	0.074234569	-17.01169316	6.72654E-65	1.56736E-62
ARMC6	690.4556646	-0.62169426	0.036415855	-17.07207627	2.39528E-65	5.64853E-63
LRRC27	214.1456887	-0.635294294	0.03720644	-17.07484755	2.28422E-65	5.45231E-63
HPSE2	43.28521883	-2.796266682	0.163752894	-17.07613594	2.23434E-65	5.39911E-63
C19orf44	93.40091251	-0.665308439	0.038888539	-17.10808528	1.29177E-65	3.16046E-63
SLC2A11	195.6635183	-0.570836502	0.033336655	-17.12338896	9.93211E-66	2.46077E-63
TSEN2	300.7538439	-0.785661847	0.045868763	-17.12847253	9.1013E-66	2.28384E-63
NAPEPLD	305.59348	-0.710414605	0.041398326	-17.16046715	5.24897E-66	1.33426E-63
MMP16	12.80086529	-2.874642065	0.16748838	-17.16323289	5.00485E-66	1.28895E-63
EAF1	1441.160583	0.62312418	0.036209211	17.2089963	2.27338E-66	5.93291E-64
TRPV1	148.1326772	-0.6701984	0.038939087	-17.21145633	2.17883E-66	5.763E-64
SCLY	103.4745087	-0.71258982	0.041371908	-17.22400175	1.75428E-66	4.70364E-64
N6AMT1	95.69677538	-0.965026826	0.055999953	-17.23263625	1.51105E-66	4.10775E-64
SLC25A17	212.1120293	-0.616438773	0.035716626	-17.25915491	9.54983E-67	2.63266E-64
RRP9	255.2853474	-0.750498831	0.043477121	-17.26192562	9.10242E-67	2.54517E-64
FAM186A	17.40948227	-1.242797842	0.071934334	-17.276838	7.02978E-67	1.99411E-64
IVD	1544.309309	-0.539217183	0.031203153	-17.28085564	6.55681E-67	1.8873E-64
EEF1AKNMT	475.2234549	-0.740529177	0.042714423	-17.33674774	2.484E-67	7.25662E-65
TARS2	642.4948235	-0.441618815	0.025402244	-17.38503139	1.07129E-67	3.17701E-65
AC090360.1	8.941577668	-1.150128488	0.066144202	-17.38819814	1.01371E-67	3.05253E-65
PRNP	3288.560085	0.823502257	0.047353788	17.39042001	9.7517E-68	2.98234E-65
DNAJC11	592.4974871	-0.496826933	0.028541459	-17.40720199	7.27539E-68	2.26034E-65
TGFBR2	4275.493865	0.667875482	0.038345551	17.41728725	6.10016E-68	1.92578E-65
MOB1A	8894.335806	0.456616693	0.026210258	17.42129699	5.68732E-68	1.82488E-65
CCDC61	192.8123339	-0.829477105	0.047485704	-17.467933	2.5144E-68	8.2024E-66
ST6GAL2	38.54124972	-2.534594606	0.145097007	-17.46827624	2.49932E-68	8.2024E-66

RBFA	343.4688975	-0.634879452	0.036342513	-17.46933278	2.45347E-68	8.2024E-66
NMNAT3	334.4993649	-1.04863147	0.059945232	-17.49315902	1.61549E-68	5.54736E-66
BCS1L	414.4135045	-0.769117418	0.043919001	-17.51217916	1.1568E-68	4.04321E-66
ZFP41	161.8980542	-0.726107633	0.041452914	-17.51644385	1.07328E-68	3.81951E-66
ZNF133	162.1287696	-0.899329356	0.051325316	-17.52213981	9.71035E-69	3.51964E-66
ZNF485	38.11070758	-1.055814927	0.060198283	-17.53895408	7.22448E-69	2.66802E-66
ZFHX2	101.5959654	-0.966082826	0.055037513	-17.55317017	5.62508E-69	2.1173E-66
SPCS3	5069.432001	0.424580702	0.02417768	17.56085346	4.91309E-69	1.88557E-66
DNAJA3	630.9653361	-0.527798084	0.029985539	-17.60175383	2.38813E-69	9.34857E-67
THNSL1	45.85684869	-1.234722451	0.070127859	-17.60673245	2.18712E-69	8.73642E-67
CREB3L4	341.3180998	-0.581862283	0.033046369	-17.60744998	2.15957E-69	8.73642E-67
UCN	50.82471019	-0.810064702	0.045929235	-17.63723467	1.27552E-69	5.31187E-67
CUL7	668.1876704	-0.544793867	0.030846807	-17.66127271	8.33391E-70	3.54608E-67
L3HYPDH	206.263665	-0.933270843	0.052722797	-17.70146696	4.08526E-70	1.77691E-67
WDR92	68.08009206	-0.806360848	0.045483238	-17.72874777	2.51575E-70	1.11911E-67
GTPBP3	348.3462256	-0.553904504	0.03118053	-17.76443499	1.33275E-70	6.06651E-68
CD8A	278.104475	1.182876624	0.066581104	17.76595078	1.29723E-70	6.04542E-68
B4GALT1	5650.356409	0.710515516	0.039983867	17.77005501	1.20572E-70	5.75601E-68
ZNF234	138.8949646	-0.858495741	0.048308811	-17.77099706	1.18565E-70	5.75601E-68
MECR	248.9646422	-0.734956686	0.041210255	-17.83431549	3.82708E-71	1.9207E-68
CDC42SE2	5327.301399	0.549816847	0.030822304	17.83827852	3.56511E-71	1.83631E-68
HEXD	697.9093368	-0.591828956	0.03299885	-17.93483595	6.30495E-72	3.33532E-69
MFSD3	202.1465425	-0.994607792	0.055228517	-18.0089535	1.65732E-72	9.01077E-70
IRF3	1179.802323	-0.555319286	0.030631601	-18.12896699	1.88263E-73	1.05282E-70
NPR2	47.22114384	-1.050467701	0.057900559	-18.14261752	1.46867E-73	8.45478E-71
TUBGCP4	559.0714966	-0.503526838	0.027746772	-18.14722245	1.3506E-73	8.01069E-71
FREM1	85.02257787	-3.045141257	0.167447291	-18.18567051	6.70306E-74	4.09997E-71
CACNB2	193.9590218	-1.388234869	0.076271274	-18.20128067	5.04152E-74	3.18315E-71
C21orf58	625.9760889	-0.884400645	0.048580906	-18.20469633	4.73673E-74	3.0904E-71
MID1	29.5234909	-1.734325303	0.09414143	-18.42255099	8.66232E-76	5.84647E-73
COL6A6	174.4692026	-2.138764115	0.115812178	-18.46752352	3.76986E-76	2.63527E-73
ANKMY1	833.8856054	-0.747236486	0.040409525	-18.49159305	2.41319E-76	1.74938E-73
MCAM	478.5717772	1.922418817	0.103688271	18.54036915	9.75498E-77	7.34362E-74
IFNGR1	2945.888853	0.712216584	0.038372138	18.56077415	6.6736E-77	5.2249E-74
TMEM177	46.58791161	-1.06983597	0.057379446	-18.64493375	1.38826E-77	1.13219E-74
ABHD13	868.4539632	0.545176266	0.029238998	18.64551839	1.37317E-77	1.13219E-74
CEP57L1	452.6388762	-0.815759317	0.043731145	-18.65396659	1.17247E-77	1.04313E-74

BACH1	5816.102552	0.616740336	0.032685376	18.86899904	2.05148E-79	1.91207E-76
ZNF691	90.84162492	-0.787086392	0.041693654	-18.87784611	1.73519E-79	1.69815E-76
DBF4B	383.542571	-0.520918025	0.027336254	-19.05594031	5.86532E-81	6.0422E-78
MTMR6	2349.361126	0.668357941	0.034926733	19.13599931	1.26628E-81	1.37694E-78
PPP2R3B	533.5828152	-0.761105416	0.039659729	-19.190888	4.41039E-82	5.07791E-79
ANKRD16	148.4902464	-0.703654392	0.036100925	-19.49131181	1.30103E-84	1.59156E-81
DHODH	348.9311703	-0.734115958	0.037655238	-19.49571952	1.19364E-84	1.55754E-81
FRAS1	84.46645367	-2.879895076	0.147453136	-19.5309178	5.99522E-85	8.38175E-82
TMED7	1717.20747	0.682648144	0.034719741	19.66167141	4.59342E-86	6.91592E-83
ZDHHC20	3732.299228	0.558695797	0.028396415	19.67487098	3.54077E-86	5.77529E-83
HOOK2	298.2217662	-0.726242422	0.03685539	-19.70518897	1.94614E-86	3.4629E-83
ZNF74	352.4081674	-0.862510747	0.043593851	-19.78514672	3.99733E-87	7.82397E-84
RCCD1	419.7071024	-0.688162656	0.03447934	-19.95869563	1.25957E-88	2.73929E-85
PRMT7	1014.755587	-0.677820984	0.033900856	-19.99421429	6.18453E-89	1.51312E-85
PCDH19	12.76242778	-3.376995325	0.168828615	-20.00250566	5.23739E-89	1.46445E-85
GNA13	9413.554519	0.754650729	0.037583623	20.07924368	1.12088E-89	3.65651E-86
ZNF692	723.2409091	-0.775519313	0.038602204	-20.09002669	9.02136E-90	3.5315E-86
MZF1	889.6419872	-0.665045002	0.033027154	-20.13630942	3.5481E-90	1.73617E-86
TMEM167B	2395.11262	0.568772695	0.02819346	20.17392347	1.65937E-90	1.08263E-86
CMTM6	4450.973064	0.653089832	0.031824548	20.52157466	1.38164E-93	1.35214E-89
MRM1	123.5679646	-1.037054438	0.048983502	-21.17150457	1.7488E-99	3.42301E-95

Supplemental Table 2. Panels 1-11: Clinical information, annotated cell populations, and immune gene signatures from bone marrow scRNA-seq data of healthy controls and myelomonocytic (M4) and monocytic (M5) AML patients.

Note: Single-cell RNA-seq data were obtained from the Single Cell Pediatric Cancer Atlas (scPCA), an open-access resource provided by Alex's Lemonade Stand Foundation for Childhood Cancer (https://scpca.alexslemonade.org).

1. Patient data information

scpca_project_id	scpca_sample_id	scpca_library_id	diagnosis	subdiagnosis	disease_timing	age	age_timing	sex	tissue_location	participant_id	submitter_id	organism	mapped_reads
**SCPCP000007**	SCPCS000228	SCPCL000302	Non-cancerous	Healthy control	Healthy control	24	unknown	M	Bone marrow	BM-AML-BM1	BM-AML-BM1	Homo sapiens	77341254
**SCPCP000007**	SCPCS000229	SCPCL000303	Non-cancerous	Healthy control	Healthy control	26	unknown	M	Bone marrow	BM-AML-BM2	BM-AML-BM2	Homo sapiens	94318328
**SCPCP000007**	SCPCS000219	SCPCL000293	Acute myeloid leukemia	M4, Acute myelomonocytic leukemia	Recurrence	3.1	collection	F	Bone marrow	BM-AML-3082	BM-AML-3082	Homo sapiens	147395707
**SCPCP000007**	SCPCS000217	SCPCL000291	Acute myeloid leukemia	M4Eo	Initial diagnosis	22.2	diagnosis	M	Bone marrow	BM-AML-647	BM-AML-647	Homo sapiens	63955396
**SCPCP000007**	SCPCS000216	SCPCL000290	Acute myeloid leukemia	M5a subtype with FLT3 ITD cytogenetics, CNS1	Initial diagnosis	3	diagnosis	M	Bone marrow	BM-AML-335	BM-AML-335	Homo sapiens	117949622
**SCPCP000007**	SCPCS000221	SCPCL000295	Acute myeloid leukemia	M5a	Initial diagnosis	6.5	diagnosis	M	Bone marrow	BM-AML-3210	BM-AML-3210	Homo sapiens	134167187
**SCPCP000007**	SCPCS000236	SCPCL000353	Acute myeloid leukemia	M5a	Initial diagnosis	7.8	diagnosis	F	Bone marrow	BM-AML-4192	BM-AML-4192	Homo sapiens	127494540
**SCPCP000007**	SCPCS000227	SCPCL000301	Acute myeloid leukemia	M5b	Recurrence	20.6	collection	F	Bone marrow	BM-AML-4068	BM-AML-4068	Homo sapiens	163500196
**SCPCP000007**	SCPCS000241	SCPCL000359	Acute myeloid leukemia	M5a	Initial diagnosis	18.8	diagnosis	F	Bone marrow	BM-AML-4239	BM-AML-4239	Homo sapiens	279905137

2. Number of cells in each annotated cell population

scpca_project_id	scpca_sample_id	Early Erythroid	Early GMP	Early Lymphoid	oBasoMast Precurs	HSC MPP	Late Erythroid	Late GMI	LMPP	gakaryocyte Precur	MEP	Monocyte	Naive T	NK	pDC	Plasma Cell	Pre-B	Pro-B	Pro-Monocyte
**SCPCP000007**	SCPCS000228	799	60	13	8	95	86	104	16	5	40	1256	618	708	132	34	75	25	438
**SCPCP000007**	SCPCS000229	348	54	45	13	91	125	128	22	8	40	1592	1511	268	133	94	553	235	483
**SCPCP000007**	SCPCS000219	236	749	100	28	395	21	391	617	1	98	680	80	44	106	1	7	-	254
**SCPCP000007**	SCPCS000217	267	282	158	63	209	41	136	198	28	281	133	3	2	67	2	1	1	145
**SCPCP000007**	SCPCS000216	243	939	137	6	165	14	710	398	-	26	454	11	8	317	8	3	4	378
**SCPCP000007**	SCPCS000221	185	115	23	7	13	14	806	3	-	5	1940	19	11	41	2	-	1	1445
**SCPCP000007**	SCPCS000236	155	198	8	9	1	71	1554	-	-	2	867	185	4	15	7	1	1	2362
**SCPCP000007**	SCPCS000227	221	938	213	2	1005	7	324	846	-	11	248	25	35	315	11	2	-	76
**SCPCP000007**	SCPCS000241	1657	1542	75	61	687	32	758	1185	4	110	1620	147	33	197	6	3	2	890

3. Immune gene signatures (T cell, exhaustion, checkpoint, and ICD/ER stress) in NK/T cells and monocytes, each compared with other cell types
Healthy control_SCPCS000228

diagnosis	subdiagnosis	disease_timing	age	age_timing	sex	tissue_location
Non-cancerous	Healthy control	Healthy control	24	unknown	M	Bone marrow

NK/T vs others	gene	p_val	avg_log2FC	pct.1	pct.2	p_val_adj
T cell	CD3D	5.26E-301	3.23793693	0.389	0.012	1.19E-297
	CD3E	0.00E+00	3.65518073	0.65	0.029	0.00E+00
	CD4	1.10E-08	-0.22114575	0.104	0.172	2.48E-05
	LCK	0.00E+00	3.001658	0.599	0.025	0.00E+00
	ZAP70	0.00E+00	3.01968843	0.573	0.023	0.00E+00
	CD2	3.94E-162	2.36053512	0.259	0.021	8.89E-159
	CD247	0.00E+00	3.12093962	0.773	0.052	0.00E+00
	CD8B	1.10E-106	3.09387693	0.153	0.007	2.48E-103
	IFNG-AS1	2.31E-17	0.93091478	0.054	0.014	5.20E-14
	IL1B	1.38E-63	-2.57137138	0.005	0.163	3.10E-60
	TCF7	4.78E-289	3.14E+00	0.417	0.028	1.08E-285
	SLAMF6	7.93E-84	1.69653	0.139	0.011	1.79E-80
	CX3CR1	6.44E-119	1.827039	0.289	0.059	1.45E-115
	GZMB	1.26E-203	2.333952	0.43	0.074	2.85E-200
	PRF1	0.00E+00	3.308397	0.56	0.055	0.00E+00
	CD244	5.50E-30	0.830937	0.137	0.049	1.24E-26
Exhaustion	TOX	2.93E-62	1.38240308	0.195	0.05	6.61E-59
	TOX2	3.19E-01	0.03071834	0.014	0.011	1.00E+00
	NR4A1	8.80E-52	-1.78316865	0.036	0.195	1.98E-48
	NR4A2	1.29E-02	0.0738085	0.412	0.382	1.00E+00
	NR4A3	2.04E-04	-0.39120697	0.055	0.085	4.61E-01
Checkpoint	LAG3	7.67E-06	0.43072082	0.017	0.005	1.73E-02
	BTLA	7.23E-03	0.39371826	0.03	0.019	1.00E+00
	CD96	2.830996e-320	2.78788246	0.436	0.022	6.383896e-317
	KLRG1	9.97E-22	0.96081739	0.099	0.035	2.25E-18
	ENTPD1	1.10E-38	-1.82100819	0.007	0.109	2.47E-35
	NT5E	2.18E-18	0.7769053	0.047	0.009	4.91E-15
ICD/ER stress	CALR	9.85E-11	-0.16816027	0.545	0.431	2.22E-07
	HMGB1	1.68E-01	-0.69481205	0.513	0.486	1.00E+00
	HSPA1A	8.63E-17	-0.95740871	0.067	0.15	1.95E-13
	HSPA1B	8.89E-09	-0.67930974	0.014	0.045	2.00E-05
	HSPA5	7.49E-32	0.35455267	0.499	0.335	1.69E-28
	HSP90B1	3.66E-06	-0.33018714	0.523	0.421	8.26E-03

Mono vs others	gene	p_val	avg_log2FC	pct.1	pct.2	p_val_adj
T cell	CD3D	1.14E-93	-3.3789716	0.003	0.206	2.58E-90
	CD3E	7.60E-171	-4.1723726	0.01	0.351	1.71E-167
	CD4	1.57E-33	0.75946895	0.231	0.105	3.53E-30
	LCK	4.83E-151	-3.3487777	0.01	0.321	1.09E-147
	ZAP70	1.62E-145	-3.2746713	0.008	0.308	3.66E-142
	CD2	5.33E-57	-2.0514337	0.01	0.147	1.20E-53
	CD247	2.03E-195	-3.406895	0.031	0.426	4.58E-192
	CD8B	1.50E-33	-2.7186071	0.003	0.082	3.39E-30
	IFNG-AS1	4.29E-17	-1.3261095	0.002	0.041	9.66E-14
	IL1B	6.81E-43	1.08678037	0.192	0.068	1.53E-39
	TCF7	2.62E-101	-3.4409261	0.01	0.233	5.90E-98
	SLAMF6	5.55E-33	-1.5459372	0.003	0.08	1.25E-29
	CX3CR1	1.15E-31	-1.8730592	0.062	0.172	2.60E-28
	GZMB	8.39E-100	-3.2930106	0.037	0.274	1.89E-96
	PRF1	2.80E-139	-3.913559	0.025	0.324	6.32E-136
	CD244	5.11E-11	-0.8538584	0.045	0.095	1.15E-07
Exhaustion	TOX	5.37E-53	-2.189875	0.012	0.144	1.21E-49
	TOX2	1.41E-05	-0.5696732	0.003	0.017	3.19E-02
	NR4A1	1.54E-45	1.02979423	0.234	0.093	3.48E-42
	NR4A2	6.39E-30	0.43256539	0.501	0.331	1.44E-26
	NR4A3	2.29E-01	-0.273291	0.069	0.078	1.00E+00
Checkpoint	LAG3	1.59E-05	-0.3996885	0.001	0.012	3.58E-02
	BTLA	2.19E-15	-0.8964302	0.001	0.034	4.93E-12
	CD96	2.52E-109	-3.0085318	0.005	0.239	5.67E-106
	KLRG1	5.80E-25	-1.382544	0.012	0.08	1.31E-21
	ENTPD1	6.92E-66	1.73691442	0.16	0.03	1.56E-62
	NT5E	8.08E-13	-0.8536347	0.002	0.032	1.82E-09
ICD/ER stress	CALR	3.73E-52	-0.7395153	0.338	0.539	8.41E-49
	HMGB1	2.62E-21	-0.8543868	0.433	0.529	5.90E-18
	HSPA1A	1.01E-07	0.26638405	0.156	0.105	2.28E-04
	HSPA1B	3.59E-03	-0.0762296	0.025	0.04	1.00E+00
	HSPA5	7.92E-56	-1.1789792	0.252	0.463	1.79E-52
	HSP90B1	1.14E-39	-0.9666517	0.342	0.515	2.58E-36

4. Immune gene signatures (T cell, exhaustion, checkpoint, and ICD/ER stress) in NK/T cells and monocytes, each compared with other cell types
Healthy control_SCPCS000229

diagnosis	subdiagnosis	disease_timing	age	age_timing	sex	tissue_location							
Non-cancerous	Healthy control	Healthy control	26	unknown	M	Bone marrow							
**NK/T vs others**	**gene**	**p_val**	**avg_log2FC**	**pct.1**	**pct.2**	**p_val_adj**	**Mono vs others**	**gene**	**p_val**	**avg_log2FC**	**pct.1**	**pct.2**	**p_val_adj**
**T cell**	CD3D	0.00E+00	4.380338943	0.589	0.01	0.00E+00	**T cell**	CD3D	2.04E-200	-4.1700718	0.005	0.327	4.59E-197
	CD3E	0.00E+00	4.856950466	0.799	0.019	0.00E+00		CD3E	1.33E-301	-5.0002062	0.008	0.447	2.99E-298
	CD4	5.22E-01	0.612540219	0.117	0.132	1.00E+00		CD4	3.57E-27	0.3193787	0.195	0.1	8.06E-24
	LCK	0.00E+00	3.888107264	0.576	0.024	0.00E+00		LCK	8.18E-207	-4.0740315	0.004	0.332	1.85E-203
	ZAP70	0.00E+00	3.365381515	0.392	0.032	0.00E+00		ZAP70	2.01E-139	-3.4639743	0.002	0.239	4.52E-136
	CD2	1.56E-236	3.143789725	0.244	0.017	3.51E-233		CD2	6.86E-76	-2.6064564	0.004	0.145	1.55E-72
	CD247	0.00E+00	4.062329964	0.498	0.011	0.00E+00		CD247	6.31E-166	-3.9723218	0.004	0.279	1.42E-162
	CD8B	4.97E-230	3.794232005	0.207	0.003	1.12E-226		CD8B	2.02E-62	-3.3180421	0.001	0.115	4.57E-59
	IFNG-AS1	7.69E-06	0.904724432	0.05	0.031	1.73E-02		IFNG-AS1	5.18E-27	-1.6320734	0.001	0.053	1.17E-23
	IL1B	1.29E-113	-2.479920851	0.003	0.156	2.92E-110		IL1B	2.04E-125	1.9166549	0.22	0.048	4.61E-122
	TCF7	0.00E+00	4.34E+00	0.557	0.024	0.00E+00		TCF7	1.14E-193	-4.2950313	0.007	0.32	2.56E-190
	SLAMF6	1.64E-22	0.9848357	0.061	0.02	3.69E-19		SLAMF6	3.50E-26	-1.5108353	0.001	0.05	7.88E-23
	CX3CR1	1.97E-13	1.4071337	0.084	0.047	4.44E-10		CX3CR1	1.15E-02	-0.3857816	0.074	0.057	1.00E+00
	GZMB	1.04E-97	1.6494952	0.151	0.024	2.34E-94		GZMB	4.37E-50	-3.2856001	0.004	0.101	9.85E-47
	PRF1	3.50E-295	3.9465545	0.264	0.005	7.89E-292		PRF1	2.37E-80	-3.505713	0.002	0.147	5.35E-77
	CD244	1.57E-04	0.7427059	0.044	0.029	3.53E-01		CD244	9.25E-01	-0.3239334	0.036	0.035	1.00E+00
**Exhaustion**	TOX	7.62E-02	0.763907143	0.072	0.065	1.00E+00	**Exhaustion**	TOX	1.90E-31	-1.5670226	0.016	0.088	4.28E-28
	TOX2	3.75E-10	-0.561829529	0.009	0.03	8.46E-07		TOX2	8.12E-16	-0.996547	0.001	0.03	1.83E-12
	NR4A1	8.00E-102	-1.674029882	0.024	0.186	1.80E-98		NR4A1	8.56E-85	1.4088317	0.234	0.079	1.93E-81
	NR4A2	7.49E-01	0.34590733	0.347	0.391	1.00E+00		NR4A2	4.91E-58	0.4924961	0.53	0.313	1.11E-54
	NR4A3	2.55E-15	-0.396409054	0.044	0.093	5.75E-12		NR4A3	4.43E-01	-0.1162616	0.078	0.072	1.00E+00
**Checkpoint**	LAG3	7.26E-45	1.509850106	0.051	0.004	1.64E-41	**Checkpoint**	LAG3	1.67E-16	-1.0872298	0	0.03	3.77E-13
	BTLA	5.81E-04	-0.179767465	0.017	0.029	1.00E+00		BTLA	2.03E-17	-0.9610931	0.001	0.033	4.59E-14
	CD96	1.838509e-317	3.214286764	0.319	0.021	4.145838e-314		CD96	2.12E-106	-3.1925216	0.003	0.19	4.77E-103
	KLRG1	8.16E-142	2.679101148	0.182	0.023	1.84E-138		KLRG1	2.27E-53	-2.3413305	0.009	0.115	5.12E-50
	ENTPD1	1.31E-51	-1.306830311	0.005	0.082	2.95E-48		ENTPD1	2.64E-44	1.2156067	0.107	0.031	5.95E-41
	NT5E	2.57E-18	0.953539687	0.039	0.01	5.79E-15		NT5E	7.06E-15	-1.002571	0.001	0.029	1.59E-11
**ICD/ER stress**	CALR	4.63E-01	-0.001793227	0.445	0.491	1.00E+00	**ICD/ER stress**	CALR	2.37E-43	-0.5443735	0.379	0.51	5.34E-40
	HMGB1	1.18E-47	-1.25271685	0.389	0.538	2.65E-44		HMGB1	2.41E-35	-1.1427915	0.41	0.507	5.43E-32
	HSPA1A	5.63E-33	-0.572888026	0.048	0.133	1.27E-29		HSPA1A	2.50E-36	0.8035386	0.167	0.074	5.65E-33
	HSPA1B	1.11E-08	-0.204510585	0.013	0.034	2.51E-05		HSPA1B	5.36E-04	-0.3957356	0.016	0.029	1.00E+00
	HSPA5	2.40E-02	0.177018651	0.345	0.361	1.00E+00		HSPA5	8.32E-52	-1.1526255	0.24	0.399	1.88E-48
	HSP90B1	3.27E-04	-0.367775765	0.357	0.431	7.38E-01		HSP90B1	8.63E-46	-1.1606966	0.3	0.442	1.95E-42

5. Immune gene signatures (T cell, exhaustion, checkpoint, and ICD/ER stress) in NK/T cells and monocytes, each compared with other cell types
AML M4_SCPCS000219

diagnosis	subdiagnosis	disease_timing	age	age_timing	sex	tissue_location
Acute myeloid leukemia	M4, Acute myelomonocytic leukemia	Recurrence	3.1	collection	F	Bone marrow

NK/T vs others	gene	p_val	avg_log2FC	pct.1	pct.2	p_val_adj
T cell	CD3D	1.14E-171	4.239858257	0.624	0.071	2.58E-168
	CD3E	1.235164e-321	5.219177177	0.748	0.044	2.785295e-318
	CD4	2.70E-01	0.700811121	0.094	0.078	1.00E+00
	LCK	0.00E+00	4.671281933	0.678	0.008	0.00E+00
	ZAP70	1.42E-51	2.54689963	0.629	0.277	3.19E-48
	CD2	4.33E-154	2.949729893	0.347	0.017	9.76E-151
	CD247	0.00E+00	4.951880771	0.723	0.015	0.00E+00
	CD8B	7.36E-254	4.29831482	0.322	0.003	1.66E-250
	IFNG-AS1	1.27E-35	1.676043274	0.094	0.006	2.87E-32
	TCF7	2.01E-221	4.70E+00	0.624	0.049	4.53E-218
	SLAMF6	1.03E-01	0.882003	0.109	0.084	1.00E+00
	CX3CR1	2.98E-63	2.3010509	0.163	0.01	6.72E-60
	GZMB	3.68E-213	4.4861082	0.292	0.004	8.30E-210
	PRF1	4.15E-55	3.8836958	0.396	0.088	9.36E-52
	CD244	2.26E-01	0.3250575	0.134	0.183	1.00E+00
Exhaustion	TOX	2.79E-02	0.181815234	0.307	0.456	1.00E+00
	TOX2	1.68E-19	1.15195689	0.04	0.002	3.79E-16
	NR4A1	4.51E-33	-2.703719284	0.069	0.542	1.02E-29
	NR4A2	3.50E-02	-0.230407759	0.594	0.727	1.00E+00
	NR4A3	2.02E-06	-0.818938808	0.178	0.361	4.55E-03
	KZF2	5.29E-02	1.050519823	0.198	0.167	1.00E+00
	BHLHE40	2.83E-01	0.543609159	0.292	0.397	1.00E+00
	ZNF683	1.22E-01	2.024648104	0.124	0.101	1.00E+00
Checkpoint	LAG3	4.50E-67	1.557603871	0.099	0.002	1.01E-63
	BTLA	5.77E-03	-0.248656901	0.025	0.079	1.00E+00
	CD96	4.29E-12	1.005577434	0.619	0.501	9.68E-09
	KLRG1	1.77E-07	1.902434952	0.173	0.08	4.00E-04
	ENTPD1	1.98E-02	0.002100621	0.02	0.06	1.00E+00
	NT5E	7.39E-06	-0.590186167	0.064	0.201	1.67E-02
ICD/ER stress	CALR	5.75E-28	-1.750627779	0.599	0.805	1.30E-24
	HMGB1	3.84E-10	-0.816359954	0.693	0.801	8.65E-07
	HSPA1A	4.03E-08	-0.692802582	0.114	0.323	9.09E-05
	HSPA1B	5.15E-07	-0.793767372	0.04	0.183	1.16E-03
	HSPA5	9.43E-01	-0.172962944	0.554	0.639	1.00E+00
	HSP90B1	5.81E-03	-0.470963761	0.525	0.692	1.00E+00
	IL1B	6.46E-27	-2.584071652	0.02	0.412	1.46E-23

Mono vs others	gene	p_val	avg_log2FC	pct.1	pct.2	p_val_adj
T cell	CD3D	9.89E-21	-1.5606844	0.054	0.134	2.23E-17
	CD3E	3.70E-29	-2.1728262	0.03	0.118	8.34E-26
	CD4	6.24E-07	0.50544565	0.099	0.06	1.41E-03
	LCK	3.48E-24	-1.679446	0.008	0.067	7.85E-21
	ZAP70	1.01E-51	-1.1494544	0.188	0.387	2.29E-48
	CD2	8.16E-17	-0.7668667	0.009	0.053	1.84E-13
	CD247	4.23E-27	-2.0061454	0.011	0.08	9.54E-24
	CD8B	3.56E-12	-1.3780204	0.003	0.03	8.03E-09
	IFNG-AS1	2.41E-06	-0.5148076	0.002	0.016	5.43E-03
	TCF7	1.74E-20	-1.8029598	0.037	0.109	3.93E-17
	SLAMF6	3.02E-29	-0.7609546	0.035	0.129	6.81E-26
	CX3CR1	1.15E-01	-0.3849555	0.014	0.019	1.00E+00
	GZMB	9.24E-10	-1.5358453	0.004	0.028	2.08E-06
	PRF1	2.30E-38	-1.6184268	0.04	0.157	5.19E-35
	CD244	4.66E-22	-0.6544027	0.123	0.233	1.05E-18
Exhaustion	TOX	2.56E-95	-1.4034113	0.295	0.587	5.77E-92
	TOX2	1.09E-01	-0.1224232	0.002	0.005	1.00E+00
	NR4A1	8.77E-48	-1.0405959	0.42	0.61	1.98E-44
	NR4A2	1.26E-63	-0.9635465	0.658	0.778	2.83E-60
	NR4A3	5.43E-67	-1.4480718	0.229	0.464	1.23E-63
Checkpoint	LAG3	3.11E-05	-0.2415182	0.001	0.011	7.01E-02
	BTLA	1.21E-13	-0.5525353	0.046	0.105	2.73E-10
	CD96	5.50E-100	-1.2343413	0.351	0.645	1.24E-96
	KLRG1	5.90E-09	-0.5032159	0.059	0.108	1.33E-05
	ENTPD1	6.83E-17	0.75093633	0.089	0.03	1.54E-13
	NT5E	2.25E-32	-0.9496979	0.122	0.261	5.08E-29
ICD/ER stress	CALR	4.33E-12	0.03961481	0.715	0.869	9.75E-09
	HMGB1	1.16E-57	-0.7108129	0.712	0.872	2.61E-54
	HSPA1A	4.17E-01	0.12387027	0.313	0.313	1.00E+00
	HSPA1B	8.09E-10	-0.3923147	0.138	0.211	1.82E-06
	HSPA5	6.81E-01	0.04894626	0.597	0.67	1.00E+00
	HSP90B1	5.84E-01	0.12935969	0.652	0.713	1.00E+00
	IL1B	7.70E-22	-0.4916697	0.317	0.464	1.74E-18

6. Immune gene signatures (T cell, exhaustion, checkpoint, and ICD/ER stress) in NK/T cells and monocytes, each compared with other cell types AML M4_SCPCS000217

diagnosis	subdiagnosis	disease_timing	age	age_timing	sex	tissue_location								
Acute myeloid leukemia	M4Eo	Initial diagnosis	22.2	diagnosis	M	Bone marrow								
**NK/T vs others**	**gene**	**p_val**	**avg_log2FC**	**pct.1**	**pct.2**	**p_val_adj**		**Mono vs others**	**gene**	**p_val**	**avg_log2FC**	**pct.1**	**pct.2**	**p_val_adj**
**T cell**	CD3D	1.94E-33	3.1008026	0.673	0.137	4.37E-30		**T cell**	CD3D	1.55E-07	-0.8136544	0.089	0.173	3.49E-04
	CD3E	0.00E+00	6.14473877	0.885	0.007	0.00E+00			CD3E	4.80E-06	-1.7104347	0.003	0.036	1.08E-02
	CD4	8.56E-03	-1.18398822	0.019	0.153	1.00E+00			CD4	2.11E-04	-0.1537046	0.103	0.169	4.75E-01
	LCK	1.30E-160	4.49348399	0.788	0.028	2.94E-157			LCK	1.76E-08	-1.2487709	0.007	0.059	3.97E-05
	ZAP70	7.57E-84	3.77318322	0.462	0.019	1.71E-80			ZAP70	2.04E-04	-0.6297545	0.009	0.036	4.61E-01
	CD2	4.17E-222	4.09702138	0.462	0.001	9.40E-219			CD2	4.56E-03	-0.5502009	0.001	0.015	1.00E+00
	CD247	1.09E-182	4.31094561	0.538	0.006	2.45E-179			CD247	1.03E-04	-0.7447261	0.001	0.025	2.33E-01
	CD8B	9.91E-246	4.64847459	0.538	0.002	2.23E-242			CD8B	1.37E-02	-0.7034641	0.004	0.017	1.00E+00
	IFNG-AS1	9.13E-72	3.077384	0.231	0.003	2.06E-68			IFNG-AS1	4.20E-03	-0.4122157	0	0.012	1.00E+00
	TCF7	4.98E-05	1.929129	0.173	0.051	1.12E-01			TCF7	6.69E-06	-0.7172579	0.022	0.067	1.51E-02
	SLAMF6	8.26E-06	1.9703076	0.192	0.055	1.86E-02			SLAMF6	3.10E-03	-0.4945243	0.036	0.067	1.00E+00
	CX3CR1	1.61E-07	1.9703975	0.173	0.036	3.63E-04			CX3CR1	1.03E-03	-0.3765464	0.019	0.047	1.00E+00
	GZMB	2.12E-101	3.6164968	0.212	0	4.79E-98			GZMB	1.15E-01	-0.3465498	0.001	0.006	1.00E+00
	PRF1	1.07E-111	4.4296687	0.538	0.018	2.41E-108			PRF1	1.35E-04	-0.8796061	0.009	0.037	3.04E-01
	CD244	7.28E-01	0.4451902	0.212	0.223	1.00E+00			CD244	1.21E-04	-0.4185011	0.171	0.244	2.72E-01
**Exhaustion**	TOX	3.25E-02	-0.28882155	0.212	0.407	1.00E+00		**Exhaustion**	TOX	2.21E-22	-1.0434347	0.251	0.465	4.99E-19
	TOX2	6.75E-01	-0.05079566	0	0.003	1.00E+00			TOX2	8.09E-01	0.00455912	0.003	0.004	1.00E+00
	NR4A1	1.47E-01	-0.02950731	0.096	0.188	1.00E+00			NR4A1	1.44E-04	-0.3671554	0.135	0.206	3.24E-01
	NR4A2	2.98E-02	-0.207646	0.25	0.47	1.00E+00			NR4A2	7.94E-09	-0.4301876	0.352	0.512	1.79E-05
	NR4A3	3.24E-01	0.00129427	0.019	0.05	1.00E+00			NR4A3	5.03E-01	-0.047676	0.045	0.051	1.00E+00
	IKZF2	3.27E-74	3.14297177	0.173	0.001	7.38E-71								
	BHLHE40	5.12E-02	-0.40348888	0.154	0.304	1.00E+00								
	ZNF683	3.80E-62	2.94448067	0.135	0	8.57E-59								
	LAG3	2.59E-04	1.89991661	0.115	0.029	5.83E-01								
**Checkpoint**	BTLA	3.04E-01	-0.26834845	0	0.02	1.00E+00		**Checkpoint**	LAG3	8.32E-02	-0.2511923	0.022	0.035	1.00E+00
	CD96	9.07E-118	4.10404893	0.5	0.013	2.04E-114			BTLA	6.04E-04	-0.249599	0.004	0.026	1.00E+00
	KLRG1	1.51E-55	3.84847314	0.558	0.051	3.40E-52			CD96	6.36E-04	-0.8125904	0.007	0.03	1.00E+00
	ENTPD1	3.79E-02	-1.11372758	0	0.077	1.00E+00			KLRG1	1.46E-08	-0.9903794	0.019	0.08	3.30E-05
	NT5E	3.53E-01	-0.12211352	0.038	0.074	1.00E+00			ENTPD1	9.76E-01	0.29317153	0.073	0.076	1.00E+00
	CALR	1.01E-04	-1.04911548	0.615	0.789	2.28E-01			NT5E	9.82E-09	-1.1555246	0.026	0.093	2.21E-05
**ICD/ER stress**	HMGB1	4.34E-06	-1.01901236	0.654	0.868	9.78E-03		**ICD/ER stress**	CALR	6.93E-01	0.21459375	0.691	0.823	1.00E+00
	HSPA1A	2.06E-06	-1.32850062	0.173	0.558	4.65E-03			HMGB1	4.02E-22	-0.55922	0.75	0.91	9.05E-19
	HSPA1B	1.97E-07	-3.10192295	0.019	0.389	4.44E-04			HSPA1A	2.87E-08	-0.497816	0.454	0.589	6.48E-05
	HSPA5	7.04E-09	-1.96875779	0.25	0.692	1.59E-05			HSPA1B	1.06E-11	-0.5593375	0.269	0.427	2.40E-08
	HSP90B1	4.00E-02	-0.52341603	0.538	0.757	1.00E+00			HSPA5	4.68E-05	-0.4176842	0.606	0.714	1.06E-01
	IL1B	2.49E-06	-3.38403534	0	0.31	5.60E-03			HSP90B1	1.43E-01	0.1808809	0.688	0.778	1.00E+00
									IL1B	1.40E-15	-1.0813602	0.19	0.35	3.16E-12

7. Immune gene signatures (T cell, exhaustion, checkpoint, and ICD/ER stress) in NK/T cells and monocytes, each compared with other cell types AML M5 with FLT3 ITD_ SCPCS000216

diagnosis	subdiagnosis	disease_timing	age	age_timing	sex	tissue_location								
Acute myeloid leukemia	M5a subtype with FLT3 ITD cytogenetics, CNS1	Initial diagnosis	3	diagnosis	M	Bone marrow								
NK/T vs others	gene	p_val	avg_log2FC	pct.1	pct.2	p_val_adj		Mono vs others	gene	p_val	avg_log2FC	pct.1	pct.2	p_val_adj
T cell	CD3D	2.63E-215	4.41001229	0.535	0.007	5.94E-212		T cell	CD3D	2.18E-04	-0.4940036	0.007	0.019	4.91E-01
	CD3E	0.00E+00	5.63337364	0.744	0.005	0.00E+00			CD3E	3.12E-11	-1.0913066	0.002	0.023	7.05E-08
	CD4	8.54E-01	0.81994967	0.14	0.147	1.00E+00			CD4	1.56E-04	0.39315871	0.163	0.129	3.52E-01
	LCK	0.00E+00	4.87880369	0.651	0.002	0.00E+00			LCK	8.67E-09	-0.6913697	0.001	0.016	1.95E-05
	ZAP70	3.51E-127	4.24821927	0.535	0.016	7.91E-124			ZAP70	2.12E-09	-0.7250762	0.009	0.035	4.78E-06
	CD2	1.61E-186	3.33476353	0.302	0.002	3.62E-183			CD2	5.73E-06	-0.28756	0	0.009	1.29E-02
	CD247	0.00E+00	5.33444363	0.698	0.007	0.00E+00			CD247	5.80E-08	-0.8716182	0.005	0.023	1.31E-04
	CD8B	1.41E-188	3.7569839	0.209	0	3.19E-185			CD8B	5.20E-03	-0.3227784	0	0.004	1.00E+00
	IFNG-AS1	5.45E-18	1.62937929	0.093	0.004	1.23E-14			IFNG-AS1	6.56E-07	-0.4811272	0	0.01	1.48E-03
	TCF7	4.64E-77	4.2112614	0.465	0.022	1.05E-73			TCF7	1.28E-05	-0.5779471	0.017	0.037	2.88E-02
	SLAMF6	1.22E-02	1.0272134	0.163	0.07	1.00E+00			SLAMF6	1.04E-05	-0.3686471	0.056	0.089	2.34E-02
	CX3CR1	4.40E-08	2.4881451	0.256	0.062	9.92E-05			CX3CR1	5.89E-04	-0.2642723	0.052	0.078	1.00E+00
	GZMB	1.08E-215	4.4898757	0.326	0.002	2.43E-212			GZMB	5.71E-06	-0.8598127	0	0.009	1.29E-02
	PRF1	2.65E-257	4.3842404	0.395	0.002	5.98E-254			PRF1	3.07E-08	-0.7205083	0	0.012	6.92E-05
	CD244	2.56E-02	-0.5614536	0.093	0.262	1.00E+00			CD244	3.23E-05	-0.3091986	0.233	0.294	7.28E-02
Exhaustion	TOX	3.03E-01	1.03714497	0.279	0.25	1.00E+00		Exhaustion	TOX	4.20E-61	-1.6935716	0.155	0.361	9.47E-58
	TOX2	8.29E-01	-0.0424984	0	0.001	1.00E+00			TOX2	1.54E-02	-0.0899825	0	0.002	1.00E+00
	NR4A1	2.21E-01	1.26192271	0.093	0.055	1.00E+00			NR4A1	4.61E-01	-0.0712968	0.057	0.052	1.00E+00
	NR4A2	1.22E-01	1.24105887	0.349	0.319	1.00E+00			NR4A2	3.97E-05	0.22772037	0.34	0.295	8.96E-02
	NR4A3	2.08E-14	2.1140938	0.163	0.015	4.68E-11			NR4A3	2.38E-01	-0.2075314	0.015	0.019	1.00E+00
	IKZF2	1.77E-136	3.84799455	0.233	0.002	3.98E-133								
	BHLHE40	9.47E-04	2.00399872	0.256	0.114	1.00E+00								
	ZNF683	1.35E-209	3.76295058	0.209	0	3.04E-206								
Checkpoint	LAG3	2.84E-17	1.31764592	0.093	0.004	6.40E-14		Checkpoint	LAG3	7.34E-04	-0.1028065	0.002	0.009	1.00E+00
	BTLA	5.64E-01	-0.1524475	0	0.008	1.00E+00			BTLA	2.31E-06	-0.1578331	0.002	0.014	5.22E-03
	CD96	6.38E-01	0.34974606	0.442	0.487	1.00E+00			CD96	8.03E-44	-0.9766724	0.405	0.582	1.81E-40
	KLRG1	1.92E-09	2.36923275	0.326	0.086	4.33E-06			KLRG1	2.87E-09	-0.5343637	0.065	0.115	6.48E-06
	ENTPD1	1.58E-01	-0.7992562	0	0.044	1.00E+00			ENTPD1	5.19E-03	0.41112322	0.052	0.035	1.00E+00
	NT5E	2.57E-09	1.0783023	0.047	0.002	5.80E-06			NT5E	1.71E-02	-0.0980378	0.001	0.004	1.00E+00
ICD/ER stress	CALR	1.99E-07	-1.4169907	0.465	0.832	4.48E-04		ICD/ER stress	CALR	1.47E-09	0.21801355	0.833	0.823	3.31E-06
	HMGB1	9.52E-05	-0.8278524	0.558	0.83	2.15E-01			HMGB1	3.73E-07	-0.3263533	0.834	0.82	8.41E-04
	HSPA1A	1.13E-02	-0.9787561	0.116	0.312	1.00E+00			HSPA1A	2.93E-03	0.25287533	0.322	0.296	1.00E+00
	HSPA1B	9.44E-02	0.48174065	0.047	0.143	1.00E+00			HSPA1B	4.41E-01	-0.0522035	0.137	0.148	1.00E+00
	HSPA5	6.20E-01	0.12250197	0.442	0.609	1.00E+00			HSPA5	3.80E-03	-0.1742621	0.59	0.629	1.00E+00
	HSP90B1	8.05E-04	-0.9850471	0.419	0.712	1.00E+00			HSP90B1	5.92E-01	-0.0731686	0.71	0.709	1.00E+00
	IL1B	2.68E-02	-1.3088127	0	0.103	1.00E+00			IL1B	2.24E-07	-0.4155017	0.08	0.128	5.06E-04

8. Immune gene signatures (T cell, exhaustion, checkpoint, and ICD/ER stress)
in NK/T cells and monocytes, each compared with other cell types
AML M5a_ SCPCS000221

diagnosis	subdiagnosis	disease_timing	age	age_timing	sex	tissue_location								
Acute myeloid leukemia	M5a	Initial diagnosis	6.5	diagnosis	M	Bone marrow								
NK/T vs others	gene	p_val	avg_log2FC	pct.1	pct.2	p_val_adj		Mono vs others	gene	p_val	avg_log2FC	pct.1	pct.2	p_val_adj
T cell	CD3D	0.00E+00	4.73751875	0.512	0	0.00E+00		T cell	CD3D	2.03E-21	-1.033874	0	0.021	4.58E-18
	CD3E	0.00E+00	5.44680068	0.561	0	0.00E+00			CD3E	5.29E-22	-1.4525266	0	0.023	1.19E-18
	CD4	1.79E-03	-0.8811768	0.098	0.345	1.00E+00			CD4	2.12E-01	-0.0777909	0.346	0.331	1.00E+00
	LCK	0.00E+00	4.34243068	0.634	0.001	0.00E+00			LCK	5.33E-29	-0.8770555	0	0.029	1.20E-25
	ZAP70	0.00E+00	4.39704662	0.585	0.001	0.00E+00			ZAP70	1.75E-22	-0.877755	0.001	0.026	3.95E-19
	CD2	1.22E-289	2.79566804	0.293	0	2.74E-286			CD2	1.65E-10	-0.3093649	0	0.012	3.71E-07
	CD247	0.00E+00	5.26317596	0.683	0.006	0.00E+00			CD247	2.54E-17	-1.416205	0.005	0.036	5.73E-14
	CD8B	0.00E+00	4.75994328	0.341	0	0.00E+00			CD8B	8.88E-15	-1.0458367	0	0.014	2.00E-11
	IFNG-AS1	2.21E-25	1.53739577	0.098	0.003	4.98E-22			IFNG-AS1	2.57E-16	-0.5187669	0	0.017	5.79E-13
	TCF7	0.00E+00	5.1217582	0.585	0.004	0.00E+00			TCF7	1.15E-16	-1.2745487	0.004	0.031	2.60E-13
	SLAMF6	1.98E-07	0.96573	0.073	0.006	4.46E-04			SLAMF6	2.20E-03	-0.0987839	0.005	0.014	1.00E+00
	CX3CR1	1.24E-01	0.6880311	0.122	0.065	1.00E+00			CX3CR1	8.09E-01	-0.053486	0.066	0.064	1.00E+00
	GZMB	0.00E+00	4.8391177	0.415	0	0.00E+00			GZMB	2.53E-16	-1.0830584	0	0.017	5.70E-13
	PRF1	0.00E+00	4.4579955	0.341	0	0.00E+00			PRF1	8.88E-15	-0.8920595	0	0.014	2.00E-11
	CD244	1.58E-01	0.7963383	0.098	0.051	1.00E+00			CD244	2.00E-04	-0.3923185	0.046	0.075	4.51E-01
Exhaustion	TOX	1.13E-155	2.61066445	0.244	0.002	2.54E-152		Exhaustion	TOX	3.16E-14	-0.5475453	0	0.016	7.13E-11
	TOX2	3.35E-01	-0.5284412	0	0.022	1.00E+00			TOX2	8.27E-01	-0.1366953	0.022	0.023	1.00E+00
	NR4A1	4.56E-01	0.82602838	0.122	0.093	1.00E+00			NR4A1	2.66E-01	-0.0869743	0.095	0.084	1.00E+00
	NR4A2	2.25E-03	0.99517222	0.39	0.207	1.00E+00			NR4A2	2.47E-02	-0.0473292	0.215	0.179	1.00E+00
	NR4A3	1.28E-01	0.24656795	0.024	0.006	1.00E+00			NR4A3	6.68E-01	-0.0185109	0.006	0.007	1.00E+00
	IKZF2	3.46E-03	0.81698188	0.098	0.025	1.00E+00								
	BHLHE40	7.43E-01	-0.3717196	0.171	0.188	1.00E+00								
	ZNF683	8.78E-30	0.59581476	0.024	0	1.98E-26								
Checkpoint	LAG3	2.17E-29	1.70183628	0.049	0	4.90E-26		Checkpoint	LAG3	4.18E-03	-0.148934	0	0.003	1.00E+00
	BTLA	7.51E-01	-0.0632397	0	0.002	1.00E+00			BTLA	8.52E-05	-0.1899818	0.001	0.008	1.92E-01
	CD96	0.00E+00	3.93829136	0.585	0	0.00E+00			CD96	3.29E-25	-0.6815042	0	0.025	7.42E-22
	KLRG1	2.27E-38	2.63815803	0.244	0.012	5.11E-35			KLRG1	4.57E-07	-0.4531229	0.01	0.03	1.03E-03
	ENTPD1	1.42E-02	-1.9580951	0	0.129	1.00E+00			ENTPD1	4.47E-03	-0.6059272	0.122	0.152	1.00E+00
	NT5E	1.33E-01	0.30136043	0.049	0.017	1.00E+00			NT5E	1.92E-08	-0.683999	0.013	0.039	4.32E-05
ICD/ER stress	CALR	6.12E-01	0.1132865	0.537	0.532	1.00E+00		ICD/ER stress	CALR	5.24E-01	0.06159793	0.533	0.526	1.00E+00
	HMGB1	1.71E-01	-0.6774045	0.61	0.629	1.00E+00			HMGB1	1.04E-08	-0.4171053	0.617	0.68	2.34E-05
	HSPA1A	1.15E-01	-1.2560339	0	0.057	1.00E+00			HSPA1A	4.16E-02	-0.2473401	0.054	0.071	1.00E+00
	HSPA1B	2.43E-01	-1.0536728	0	0.032	1.00E+00			HSPA1B	9.78E-01	-0.0945264	0.032	0.032	1.00E+00
	HSPA5	1.90E-03	0.79997191	0.585	0.424	1.00E+00			HSPA5	3.02E-01	-0.0285361	0.428	0.41	1.00E+00
	HSP90B1	8.61E-01	0.06609109	0.488	0.522	1.00E+00			HSP90B1	8.67E-01	-0.0647525	0.52	0.526	1.00E+00
	IL1B	4.48E-02	-1.7770318	0	0.09	1.00E+00			IL1B	7.22E-08	0.58230866	0.099	0.046	1.63E-04

9. Immune gene signatures (T cell, exhaustion, checkpoint, and ICD/ER stress) in NK/T cells and monocytes, each compared with other cell types
AML M5a_ SCPCS000236

diagnosis	subdiagnosis	disease_timing	age	age_timing	sex	tissue_location								
Acute myeloid leukemia	M5a	Initial diagnosis	7.8	diagnosis	F	Bone marrow								
**NK/T vs others**	**gene**	**p_val**	**avg_log2FC**	**pct.1**	**pct.2**	**p_val_adj**		**Mono vs others**	**gene**	**p_val**	**avg_log2FC**	**pct.1**	**pct.2**	**p_val_adj**
**T cell**	CD3D	0.00E+00	4.55582284	0.752	0.006	0.00E+00		**T cell**	CD3D	1.51E-181	-2.9974028	0.004	0.186	3.42E-178
	CD3E	0.00E+00	5.13694024	0.874	0.009	0.00E+00			CD3E	1.24E-198	-3.4801293	0.007	0.215	2.80E-195
	CD4	1.22E-01	-0.5167003	0.252	0.189	1.00E+00			CD4	3.31E-03	0.25043633	0.181	0.24	1.00E+00
	LCK	0.00E+00	4.22141728	0.691	0.005	0.00E+00			LCK	3.22E-166	-2.8145761	0.004	0.169	7.26E-163
	ZAP70	0.00E+00	3.64842449	0.516	0.003	0.00E+00			ZAP70	3.89E-131	-2.2807098	0.002	0.128	8.78E-128
	CD2	0.00E+00	2.78023907	0.325	0.002	0.00E+00			CD2	6.87E-73	-1.2745594	0.002	0.077	1.55E-69
	CD247	0.00E+00	3.81212517	0.63	0.008	0.00E+00			CD247	1.37E-148	-2.8560748	0.005	0.162	3.09E-145
	CD8B	5.75E-121	3.01225153	0.106	0.001	1.30E-117			CD8B	1.51E-26	-1.5019058	0	0.025	3.41E-23
	IFNG-AS1	5.67E-48	1.37962511	0.057	0.001	1.28E-44			IFNG-AS1	1.11E-20	-0.5772146	0	0.019	2.50E-17
	TCF7	0.00E+00	4.0555231	0.74	0.019	0.00E+00			TCF7	2.09E-167	-3.25534	0.013	0.213	4.72E-164
	SLAMF6	2.21E-58	1.2746286	0.089	0.003	4.99E-55			SLAMF6	5.07E-30	-0.8477079	0.001	0.033	1.14E-26
	CX3CR1	1.13E-01	-1.0118684	0.012	0.029	1.00E+00			CX3CR1	2.92E-06	-0.414648	0.024	0.05	6.60E-03
	GZMB	5.91E-60	2.6204883	0.065	0.001	1.33E-56			GZMB	2.26E-13	-1.1248641	0.001	0.016	5.10E-10
	PRF1	1.34E-116	2.3704421	0.106	0.001	3.02E-113			PRF1	2.56E-25	-0.9938998	0.001	0.025	5.77E-22
	CD244	7.61E-16	0.6329442	0.024	0.001	1.72E-12			CD244	8.69E-06	-0.1907842	0.001	0.007	1.96E-02
**Exhaustion**	TOX	7.29E-16	0.81094977	0.102	0.021	1.64E-12		**Exhaustion**	TOX	6.96E-14	-0.5948319	0.017	0.056	1.57E-10
	TOX2	8.44E-01	-0.4867929	0.02	0.022	1.00E+00			TOX2	3.31E-02	-0.0570513	0.02	0.031	1.00E+00
	NR4A1	3.90E-01	-0.4205739	0.049	0.037	1.00E+00			NR4A1	1.12E-01	0.38028557	0.036	0.047	1.00E+00
	NR4A2	2.35E-06	0.28015342	0.289	0.162	5.30E-03			NR4A2	1.40E-14	-0.1647861	0.148	0.258	3.16E-11
	NR4A3	9.93E-06	0.21398918	0.024	0.004	2.24E-02			NR4A3	2.34E-04	-0.0083798	0.003	0.012	5.28E-01
	IKZF2	1.53E-107	1.86492488	0.102	0.001	3.44E-104								
	BHLHE40	5.72E-02	0.27103364	0.146	0.107	1.00E+00								
	ZNF683	3.91E-17	0.85067984	0.012	0	8.82E-14								
**Checkpoint**	LAG3	1.19E-63	1.48854095	0.053	0	2.68E-60		**Checkpoint**	LAG3	6.60E-16	-0.5301287	0	0.013	1.49E-12
	BTLA	1.28E-02	0.05805481	0.016	0.005	1.00E+00			BTLA	7.60E-06	-0.248871	0.003	0.014	1.71E-02
	CD96	0.00E+00	3.11962542	0.423	0.008	0.00E+00			CD96	9.93E-112	-2.1637602	0.004	0.121	2.24E-108
	KLRG1	2.09E-59	2.03090205	0.13	0.008	4.71E-56			KLRG1	6.23E-15	-0.757825	0.008	0.038	1.40E-11
	ENTPD1	1.83E-02	-0.9881134	0.004	0.03	1.00E+00			ENTPD1	4.68E-02	0.11841296	0.027	0.038	1.00E+00
	NT5E	8.01E-15	0.48533112	0.016	0	1.81E-11			NT5E	2.50E-05	-0.1843704	0	0.005	5.64E-02
**ICD/ER stress**	CALR	8.02E-04	-0.2680467	0.476	0.309	1.00E+00		**ICD/ER stress**	CALR	5.50E-02	0.20712831	0.303	0.377	1.00E+00
	HMGB1	1.20E-03	-0.2219766	0.634	0.415	1.00E+00			HMGB1	5.27E-13	-0.2007479	0.395	0.556	1.19E-09
	HSPA1A	8.80E-01	-0.6242121	0.041	0.038	1.00E+00			HSPA1A	1.06E-03	0.06801224	0.034	0.056	1.00E+00
	HSPA1B	9.03E-01	-0.2302131	0.008	0.007	1.00E+00			HSPA1B	4.94E-02	-0.1706409	0.006	0.012	1.00E+00
	HSPA5	3.03E-20	0.51017764	0.447	0.186	6.83E-17			HSPA5	1.90E-18	-0.1905247	0.173	0.309	4.28E-15
	HSP90B1	5.51E-01	-0.6853145	0.415	0.325	1.00E+00			HSP90B1	2.91E-01	0.16603907	0.319	0.376	1.00E+00
	IL1B	3.12E-01	-0.3675667	0	0.004	1.00E+00			IL1B	2.27E-01	0.38144889	0.004	0.002	1.00E+00

10. Immune gene signatures (T cell, exhaustion, checkpoint, and ICD/ER stress) in NK/T cells and monocytes, each compared with other cell types
AML M5b_ SCPCS000227

diagnosis	subdiagnosis	disease_timing	age	age_timing	sex	tissue_location
Acute myeloid leukemia	M5b	Recurrence	20.6	collection	F	Bone marrow

NK/T vs others	gene	p_val	avg_log2FC	pct.1	pct.2	p_val_adj
T cell	CD3D	0.00E+00	5.15207932	0.758	0.004	0.00E+00
	CD3E	0.00E+00	5.78780268	0.862	0.004	0.00E+00
	CD4	1.65E-17	-0.9308953	0.104	0.398	3.71E-14
	LCK	0.00E+00	4.67326382	0.738	0.005	0.00E+00
	ZAP70	0.00E+00	4.493268	0.704	0.004	0.00E+00
	CD2	0.00E+00	3.80640144	0.5	0.006	0.00E+00
	CD247	0.00E+00	4.70728187	0.731	0.011	0.00E+00
	CD8B	0.00E+00	5.12313864	0.546	0.002	0.00E+00
	IFNG-AS1	8.89E-21	0.89455282	0.046	0.003	2.00E-17
	TCF7	1.66E-125	3.1460931	0.315	0.026	3.75E-122
	SLAMF6	4.31E-56	1.9864843	0.204	0.025	9.72E-53
	CX3CR1	1.18E-52	2.4381982	0.369	0.095	2.66E-49
	GZMB	0.00E+00	5.8026706	0.719	0.007	0.00E+00
	PRF1	0.00E+00	6.0432931	0.831	0.004	0.00E+00
	CD244	2.22E-07	-0.4478716	0.173	0.375	5.00E-04
Exhaustion	TOX	3.53E-02	0.06600259	0.304	0.436	1.00E+00
	TOX2	2.01E-01	0.20600364	0.012	0.024	1.00E+00
	NR4A1	5.19E-06	-0.738331	0.027	0.123	1.17E-02
	NR4A2	5.89E-01	0.43316211	0.385	0.462	1.00E+00
	NR4A3	8.01E-01	0.38804292	0.054	0.053	1.00E+00
	IKZF2	9.01E-10	1.28270258	0.077	0.02	2.03E-06
	BHLHE40	5.76E-04	0.99996091	0.377	0.359	1.00E+00
	ZNF683	5.52E-278	3.38249434	0.277	0.002	1.24E-274
Checkpoint	LAG3	8.25E-63	2.53575697	0.327	0.063	1.86E-59
	BTLA	3.64E-01	-0.0540173	0	0.003	1.00E+00
	CD96	4.74E-27	1.44952632	0.5	0.263	1.07E-23
	KLRG1	6.94E-156	3.24689876	0.508	0.063	1.57E-152
	ENTPD1	8.13E-03	-0.4213138	0.008	0.04	1.00E+00
	NT5E	2.33E-08	-1.004036	0.015	0.136	5.26E-05
ICD/ER stress	CALR	4.49E-05	-0.4010442	0.758	0.886	1.01E-01
	HMGB1	2.18E-35	-1.0875591	0.712	0.934	4.92E-32
	HSPA1A	1.00E-14	-1.147812	0.088	0.336	2.26E-11
	HSPA1B	1.26E-11	-1.3639749	0.015	0.181	2.83E-08
	HSPA5	3.44E-04	0.35607443	0.685	0.738	7.75E-01
	HSP90B1	1.28E-07	-0.5211399	0.623	0.831	2.90E-04
	IL1B	2.39E-10	-1.2447617	0.008	0.15	5.39E-07

Mono vs others	gene	p_val	avg_log2FC	pct.1	pct.2	p_val_adj
T cell	CD3D	3.25E-21	-1.8465626	0.001	0.057	7.33E-18
	CD3E	2.27E-24	-2.4431853	0.001	0.065	5.12E-21
	CD4	5.91E-14	0.39486215	0.451	0.354	1.33E-10
	LCK	3.17E-20	-1.5320011	0.003	0.057	7.16E-17
	ZAP70	8.32E-19	-1.3384398	0.003	0.053	1.88E-15
	CD2	1.98E-14	-1.0037777	0.003	0.041	4.46E-11
	CD247	8.07E-16	-1.5165677	0.011	0.061	1.82E-12
	CD8B	1.98E-14	-1.7485376	0.002	0.04	4.46E-11
	IFNG-AS1	5.47E-04	-0.2345875	0	0.007	1.00E+00
	TCF7	4.34E-04	-0.6248582	0.026	0.046	9.79E-01
	SLAMF6	5.18E-05	-0.4088909	0.019	0.041	1.17E-01
	CX3CR1	1.35E-05	-0.4913478	0.08	0.12	3.05E-02
	GZMB	9.59E-16	-2.2809123	0.008	0.056	2.16E-12
	PRF1	9.93E-22	-2.5679047	0.003	0.062	2.24E-18
	CD244	1.47E-11	-0.4841736	0.306	0.39	3.31E-08
Exhaustion	TOX	3.54E-39	-0.9100897	0.305	0.482	7.97E-36
	TOX2	1.87E-02	-0.1280541	0.016	0.027	1.00E+00
	NR4A1	2.05E-04	0.32394278	0.143	0.108	4.62E-01
	NR4A2	2.14E-12	0.33835475	0.526	0.429	4.83E-09
	NR4A3	2.99E-01	0.09051676	0.057	0.051	1.00E+00
Checkpoint	LAG3	4.20E-14	-0.7568345	0.034	0.093	9.47E-11
	BTLA	3.91E-02	-0.0634576	0.001	0.004	1.00E+00
	CD96	2.47E-11	-0.6259589	0.216	0.299	5.57E-08
	KLRG1	3.57E-09	-0.8080312	0.052	0.099	8.06E-06
	ENTPD1	4.91E-02	-0.2303333	0.031	0.042	1.00E+00
	NT5E	8.94E-13	-0.5919431	0.079	0.151	2.02E-09
ICD/ER stress	CALR	7.73E-52	0.47953087	0.927	0.86	1.74E-48
	HMGB1	1.51E-13	0.16179402	0.941	0.916	3.40E-10
	HSPA1A	5.39E-01	-0.0883301	0.317	0.328	1.00E+00
	HSPA1B	1.26E-02	0.19677758	0.191	0.165	1.00E+00
	HSPA5	5.92E-03	0.03249069	0.747	0.731	1.00E+00
	HSP90B1	4.28E-11	0.11323303	0.846	0.81	9.65E-08
	IL1B	3.61E-05	0.27526267	0.173	0.13	8.15E-02

11. Immune gene signatures (T cell, exhaustion, checkpoint, and ICD/ER stress) in NK/T cells and monocytes, each compared with other cell types
AML M5a_SCPCS000241

diagnosis	subdiagnosis	disease_timing	age	age_timing	sex	tissue_location
Acute myeloid leukemia	M5a	Initial diagnosis	18.8	diagnosis	F	Bone marrow

NK/T vs others	gene	p_val	avg_log2FC	pct.1	pct.2	p_val_adj	Mono vs others	gene	p_val	avg_log2FC	pct.1	pct.2	p_val_adj
T cell	CD3D	0.00E+00	4.27337394	0.729	0.056	0.00E+00	T cell	CD3D	5.95E-47	-1.2889756	0.036	0.11	1.34E-43
	CD3E	0.00E+00	5.44091982	0.841	0.018	0.00E+00		CD3E	6.95E-36	-1.8057195	0.016	0.066	1.57E-32
	CD4	2.40E-10	1.4251589	0.217	0.116	5.41E-07		CD4	5.18E-13	0.38796121	0.142	0.099	1.17E-09
	LCK	0.00E+00	4.66666058	0.726	0.007	0.00E+00		LCK	1.00E-30	-1.2049068	0.008	0.045	2.26E-27
	ZAP70	0.00E+00	3.67168222	0.545	0.042	0.00E+00		ZAP70	5.56E-49	-1.0971562	0.021	0.087	1.25E-45
	CD2	1.47E-189	2.8890553	0.382	0.036	3.30E-186		CD2	2.28E-29	-0.7223793	0.022	0.068	5.15E-26
	CD247	0.00E+00	4.47914481	0.643	0.012	0.00E+00		CD247	2.13E-31	-1.3338615	0.009	0.048	4.81E-28
	CD8B	0.00E+00	4.19460115	0.248	0.004	0.00E+00		CD8B	8.95E-09	-1.0436557	0.005	0.017	2.02E-05
	IFNG-AS1	1.70E-159	2.20215914	0.137	0.004	3.84E-156		IFNG-AS1	4.83E-14	-0.5030702	0.001	0.013	1.09E-10
	TCF7	0.00E+00	4.6151224	0.643	0.02	0.00E+00		TCF7	1.91E-31	-1.472829	0.015	0.059	4.31E-28
	SLAMF6	1.11E-07	0.968778	0.175	0.095	2.51E-04		SLAMF6	7.68E-46	-0.7128466	0.052	0.135	1.73E-42
	CX3CR1	4.51E-15	1.7723744	0.102	0.028	1.02E-11		CX3CR1	2.03E-05	0.1090599	0.037	0.023	4.57E-02
	GZMB	5.10E-131	2.9853609	0.076	0.001	1.15E-127		GZMB	2.21E-04	-0.4465242	0.001	0.005	4.99E-01
	PRF1	2.11E-79	3.3561724	0.245	0.036	4.76E-76		PRF1	5.80E-33	-0.9067549	0.016	0.063	1.31E-29
	CD244	2.37E-03	0.2874847	0.064	0.127	1.00E+00		CD244	6.32E-15	-0.4637223	0.098	0.149	1.43E-11
Exhaustion	TOX	3.02E-12	-0.7043196	0.153	0.367	6.81E-09	Exhaustion	TOX	1.41E-121	-1.1007083	0.243	0.461	3.19E-118
	TOX2	1.50E-22	0.69041769	0.032	0.002	3.39E-19		TOX2	8.05E-02	-0.051499	0.002	0.004	1.00E+00
	NR4A1	1.96E-07	-0.7077389	0.038	0.145	4.42E-04		NR4A1	2.09E-09	-0.2874016	0.12	0.161	4.72E-06
	NR4A2	6.99E-10	-0.7213651	0.185	0.38	1.58E-06		NR4A2	8.06E-02	-0.1720402	0.365	0.382	1.00E+00
	NR4A3	8.95E-02	-0.0226976	0.013	0.029	1.00E+00		NR4A3	5.92E-03	-0.1393121	0.024	0.033	1.00E+00
	IKZF2	5.41E-01	0.26662527	0.124	0.144	1.00E+00							
	BHLHE40	3.03E-03	-0.1202009	0.143	0.231	1.00E+00							
	ZNF683	4.02E-10	1.44836601	0.057	0.014	9.07E-07							
Checkpoint	LAG3	9.78E-22	1.03765487	0.054	0.006	2.20E-18	Checkpoint	LAG3	4.86E-09	-0.1390699	0.002	0.012	1.10E-05
	BTLA	7.20E-14	0.57041384	0.054	0.01	1.62E-10		BTLA	3.18E-13	-0.2045998	0.003	0.018	7.16E-10
	CD96	1.11E-56	1.96008616	0.541	0.218	2.51E-53		CD96	7.72E-108	-1.2389382	0.131	0.309	1.74E-104
	KLRG1	4.80E-40	2.40015593	0.242	0.062	1.08E-36		KLRG1	4.94E-20	-0.6539338	0.043	0.089	1.11E-16
	ENTPD1	1.20E-01	-0.1440404	0.029	0.048	1.00E+00		ENTPD1	4.38E-21	0.53529453	0.069	0.03	9.88E-18
	NT5E	2.09E-03	-0.2733159	0.124	0.208	1.00E+00		NT5E	2.74E-47	-0.6001496	0.143	0.258	6.18E-44
ICD/ER stress	CALR	1.51E-53	-2.1860269	0.564	0.798	3.40E-50	ICD/ER stress	CALR	1.20E-15	0.02683146	0.734	0.84	2.71E-12
	HMGB1	5.09E-03	-0.4044681	0.704	0.722	1.00E+00		HMGB1	6.08E-85	-0.6374907	0.667	0.768	1.37E-81
	HSPA1A	6.75E-13	-1.0902122	0.096	0.298	1.52E-09		HSPA1A	8.35E-01	0.00543667	0.287	0.297	1.00E+00
	HSPA1B	1.74E-05	-0.5683235	0.025	0.101	3.93E-02		HSPA1B	4.41E-10	-0.3562518	0.078	0.115	9.94E-07
	HSPA5	7.65E-05	-1.0307983	0.459	0.561	1.73E-01		HSPA5	2.01E-01	0.19155634	0.53	0.583	1.00E+00
	HSP90B1	2.58E-04	-0.7831725	0.51	0.601	5.82E-01		HSP90B1	4.83E-02	0.24226412	0.585	0.609	1.00E+00
	IL1B	2.67E-34	-2.8177298	0.013	0.357	6.02E-31		IL1B	4.39E-36	-0.5728718	0.284	0.401	9.90E-33

Supplemental Table 3. Effect on abscopal tumor volume after intratumoral injection of different regimens into the primary tumor site, compared to the vehicle group.

**Treatment regimen of the primary tumor**	**Tumor volume reduction at the abscopal site (%)**	**Significance** (compared to the vehicle group)
Mitoxantrone (8mg/kg)	23.4	**
OT-55 (80 mg/kg) + A-1331852 (10mg/kg)	15.2	**
Anti-PD-1 (150 μg, injected three times)	6.8	*n.s.*
Anti-Tim-3 (150 μg, injected three times)	9.8	*n.s.*
OT-55 (80 mg/kg) + A-1331852 (10mg/kg) + Anti-PD-1 (150 μg, injected three times)	62.7	****
OT-55 (80 mg/kg) + A-1331852 (10mg/kg) + Anti-Tim-3 (150 μg, injected three times)	65.7	****
OT-55 (80 mg/kg) + A-1331852 (10mg/kg) + Anti-PD-1 and Anti-Tim-3 (150 μg each, injected three times)	74.0	****

Note: Values represent tumor volume reduction. Data are shown as mean ± SEM. Statistical analysis was performed using one-way ANOVA followed by Tukey's multiple comparisons test. The table indicates significance relative to the vehicle group at the end of the experiment on day 16. *n.s.* means not significant, $^{**}P < 0.01$, $^{****}P < 0.0001$.

Supplemental Table 4. Effect on primary tumor volume following injection of various regimens, compared to the vehicle group.

**Treatment regimen of the primary tumor**	**Tumor volume reduction at the primary site (%)**	**Significance** (compared to the vehicle group)
Mitoxantrone (8mg/kg)	62.6	****
OT-55 (80 mg/kg) + A-1331852 (10mg/kg)	40.4	****
Anti-PD-1 (150 μg, injected three times)	3.6	*n.s.*
Anti-Tim-3 (150 μg, injected three times)	0.5	*n.s.*
OT-55 (80 mg/kg) + A-1331852 (10mg/kg) + Anti-PD-1 (150 μg, injected three times)	70.8	****
OT-55 (80 mg/kg) + A-1331852 (10mg/kg) + Anti-Tim-3 (150 μg, injected three times)	72.5	****
OT-55 (80 mg/kg) + A-1331852 (10mg/kg) + Anti-PD-1 and Anti-Tim-3 (150 μg each, injected three times)	78.4	****

Note: Values represent tumor volume reduction. Data are shown as mean ± SEM. Statistical analysis was performed using one-way ANOVA followed by Tukey's multiple comparisons test. The table indicates significance compared to the vehicle group at the end of the experiment on day 16. *n.s.* = not significant, ****$P < 0.0001$.

Supplemental Table 5. Effect on abscopal tumor volume after intratumoral injection of different regimens into the primary tumor site, compared to the group treated with a combination of OT-55 + A-1331852.

**Treatment regimen of the primary tumor**	**Change of the tumor volume at the abscopal site (%)**<br>**+ increase**<br>**- reduction**	**Significance** (compared to OT-55 + A-1331852)
Vehicle	+17.9	*n.s.*
Mitoxantrone (8mg/kg)	-9.7	*n.s.*
Anti-PD-1 (150 µg, injected three times)	+9.8	*n.s.*
Anti-Tim-3 (150 µg, injected three times)	+6.3	*n.s.*
OT-55 (80 mg/kg) + A-1331852 (10mg/kg) + Anti-PD-1 (150 µg, injected three times)	-56.0	****
OT-55 (80 mg/kg) + A-1331852 (10mg/kg) + Anti-Tim-3 (150 µg, injected three times)	-59.6	****
OT-55 (80 mg/kg) + A-1331852 (10mg/kg) + Anti-PD-1 and Anti-Tim-3 (150 µg each, injected three times)	-69.4	****

Note: Data are shown as mean ± SEM. Statistical analysis was conducted using one-way ANOVA followed by Tukey's multiple comparisons test. The table presents the significance levels compared to the OT-55 + A-1331852 group at the experiment's endpoint on day 16. *n.s.* denotes not significant, ****$P < 0.0001$.

Supplemental Table 6. Effect on primary tumor volume after injection of different regimens, compared to the combination of OT-55 + A-1331852 group.

**Treatment regimen of the primary tumor**	**Change of the tumor volume at the abscopal site (%)**<br>**+ increase**<br>**- reduction**	**Significance** (compared to OT-55 + A-1331852)
Vehicle	+67.6	****
Mitoxantrone (8mg/kg)	-37.3	**
Anti-PD-1 (150 µg, injected three times)	+61.7	****
Anti-Tim-3 (150 µg, injected three times)	+68.5	****
OT-55 (80 mg/kg) + A-1331852 (10mg/kg) + Anti-PD-1 (150 µg, injected three times)	-51.1	****
OT-55 (80 mg/kg) + A-1331852 (10mg/kg) + Anti-Tim-3 (150 µg, injected three times)	-53.9	****
OT-55 (80 mg/kg) + A-1331852 (10mg/kg) + Anti-PD-1 and Anti-Tim-3 (150 µg each, injected three times)	-63.8	****

Note: Data are shown as mean ± SEM. Statistical analysis was conducted using one-way ANOVA followed by Tukey’s multiple comparisons test. The table highlights significant differences relative to the OT-55 + A-1331852 group at the experiment's endpoint on day 16. $^{**}P < 0.01$, $^{****}P < 0.0001$.

Supplemental Table 7. Antibodies used for Western blot and FACs analysis.

Western blot antibodies				
**Name**	**Source**	**Dilution**	**Manufacturer**	**Catalogue No.**
Bcl-xL Antibody	Rabbit	1:1000	Cell signaling	2762
Mcl-1 (D35A5) Rabbit mAb	Rabbit	1:1000	Cell signaling	5453
Bcl-2 (D17C4) Rabbit mAb	Rabbit	1:1000	Cell signaling	3498
Anti-Noxa antibody	Mouse	1:1000	Cell signaling	ab13654
Caspase-3	Mouse	1:1000	Santa Cruz	sc-56053
Cleaved Caspase-3 (Asp175)	Rabbit	1:1000	Cell signaling	9661
Anti-β-Actin (ACTB) Antibody	Mouse	1:20000	Sigma-Aldrich	A5441

FACs antibodies		
**Name**	**Manufacturer**	**Catalog No.**
PE-conjugated H-2Kb OVA257–264 (SIINFEKL)	NIH	
BD Horizon™ Fixable Viability Stain 510	BD	564406
FITC anti-mouse CD45 Antibody	BioLegend	103108
BD Pharmingen™ APC-Cy™7 Rat Anti-Mouse CD45	BD	557659
PE/Cyanine7 anti-mouse CD3ε Antibody	BioLegend	100320
Brilliant Violet 605™ anti-mouse CD3 Antibody	BioLegend	100237
PerCP anti-mouse CD8a Antibody	BioLegend	100732
BD Pharmingen™ APC Rat Anti-Mouse CD4	BD	553051
BD Horizon™ BV421 Hamster Anti-Mouse CD279 (PD-1)	BD	562584
PE anti-mouse CD366 (Tim-3) Antibody	BioLegend	119704
BD Horizon™ BV421 Rat Anti-Mouse IFN-γ	BD	563376
Pacific Blue™ anti-human/mouse Granzyme B Recombinant Antibody	BioLegend	372218
PE anti-human/mouse Granzyme B Recombinant Antibody	BioLegend	372208
TOX Monoclonal Antibody (TXRX10), PE, eBioscience™	Invitrogen	12-6502-82
