# Supplementary material for: OT-55 reshapes tolerogenic BH3-mimetic-induced apoptosis toward immunogenic cell death in acute myeloid leukemia, potentiating PD-1/Tim-3 blockade
Source: Cell Death Dis. 2026 Jul 1;17(1):821. doi: 10.1038/s41419-026-09000-9 (PMC13594238; doi:10.1038/s41419-026-09000-9)

Original Western blots

OT-55 reshapes tolerogenic BH3-mimetic-induced apoptosis toward immunogenic cell death in acute myeloid leukemia, potentiating PD-1/Tim-3 blockade

Yejin Lee[1], Eun-Ji Kwon[1], Sruthi Reddy Gajulapalli[1], Ji Yeon Paik[1], Barbora Orlikova-Boyer[2,&], Artur M.S. Silva[3], Hyuk-Jin Cha[1], Claudia Cerella[2,&], and Marc Diederich[1]*

[1] Department of Pharmacy, College of Pharmacy, Seoul National University, 1 Gwanak-ro, Gwanak-gu, Seoul, 08626, Korea

[2] Laboratoire de Biologie Moléculaire du Cancer (LBMCC), BAM3 Pavillon 2, 6A rue Nicolas-Ernest Barblé, L-1210 Luxembourg, Luxembourg

[3] Department of Chemistry & REQUIMTE, University of Aveiro, 3810-193, Aveiro, Portugal

***Corresponding author:**

Marc Diederich, Research Institute of Pharmaceutical Sciences & Natural Products Research Institute, College of Pharmacy, Seoul National University, Seoul 08826, Republic of Korea. Email: marcdiederich@snu.ac.kr

Present address: [&]Department of Cancer Research, Luxembourg Institute of Health (LIH), BAM3 Pavillon 2, 6A rue Nicolas-Ernest Barblé, L-1210 Luxembourg, Luxembourg.

Supplemental Figure

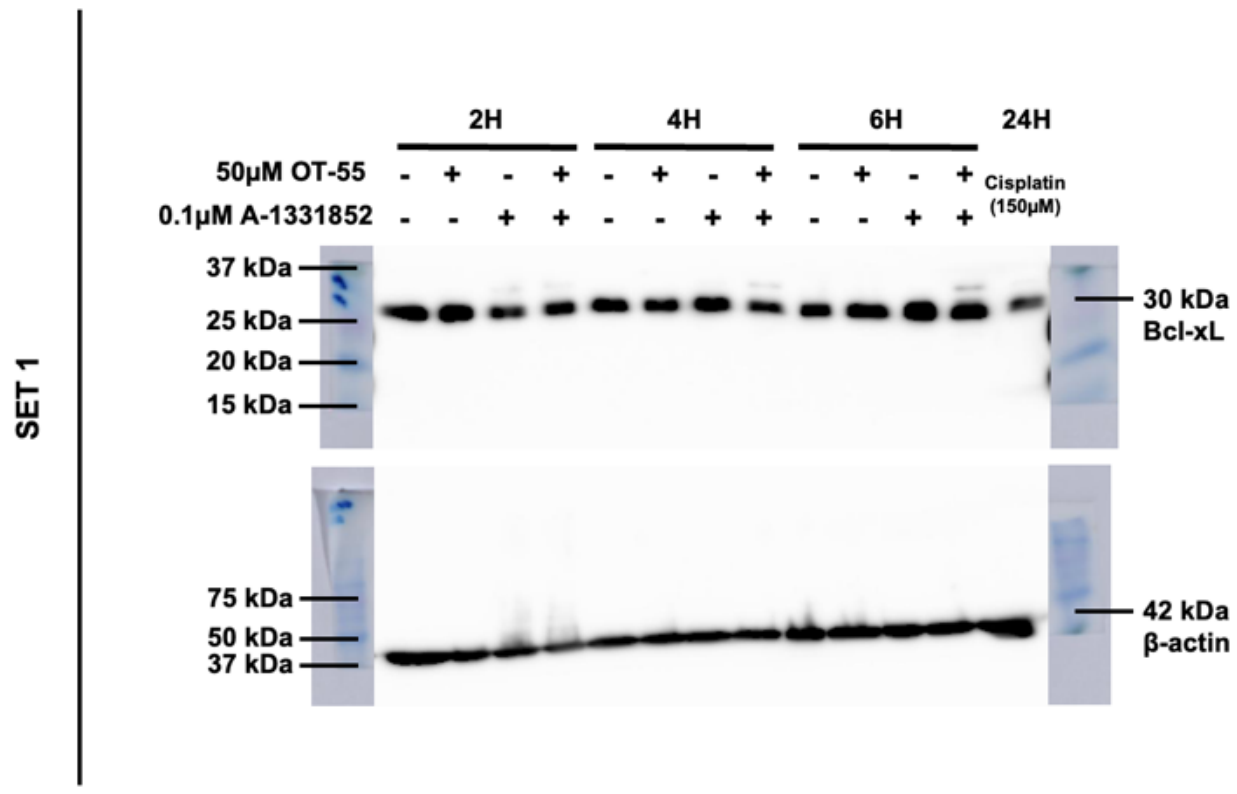

Used in the figure

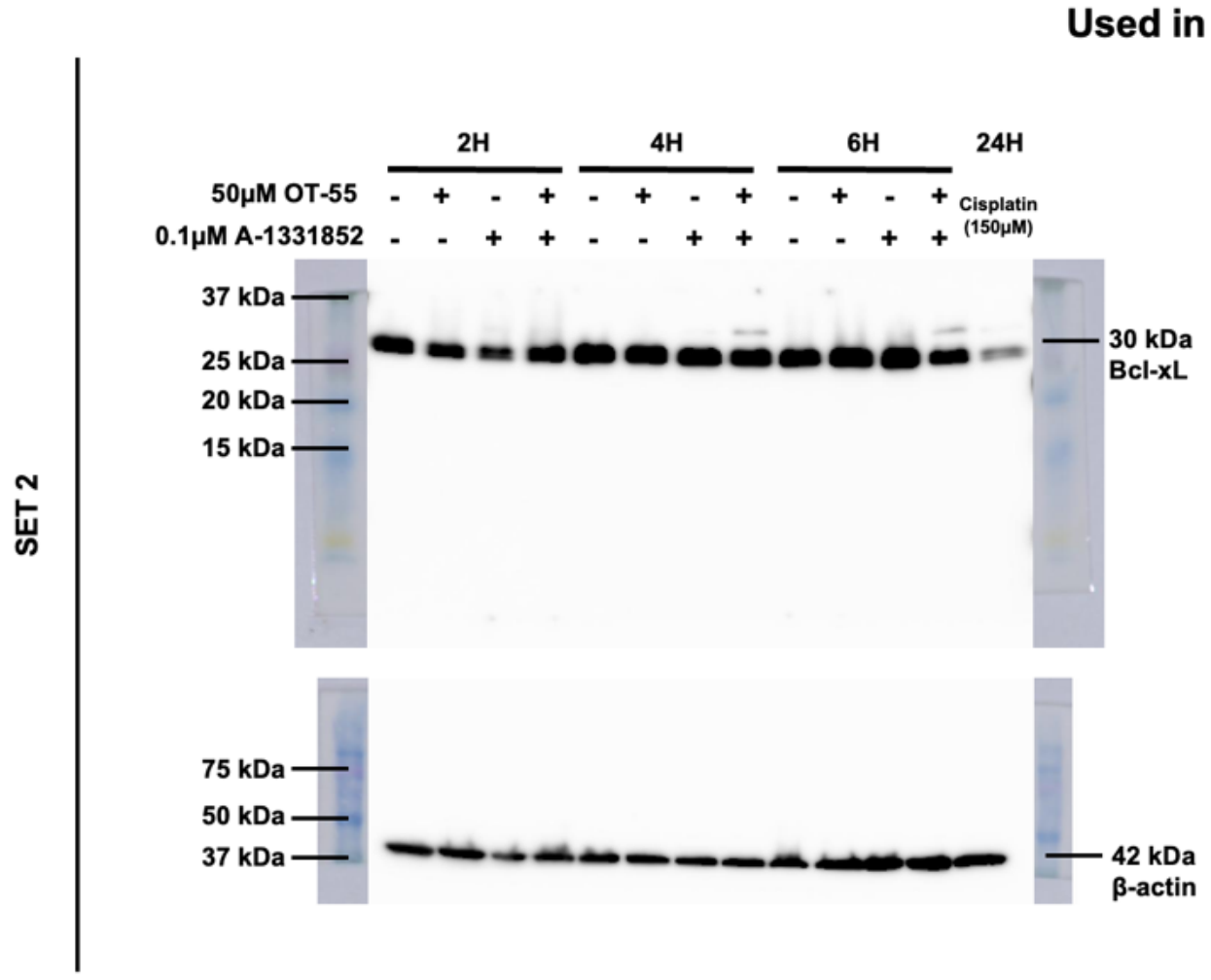

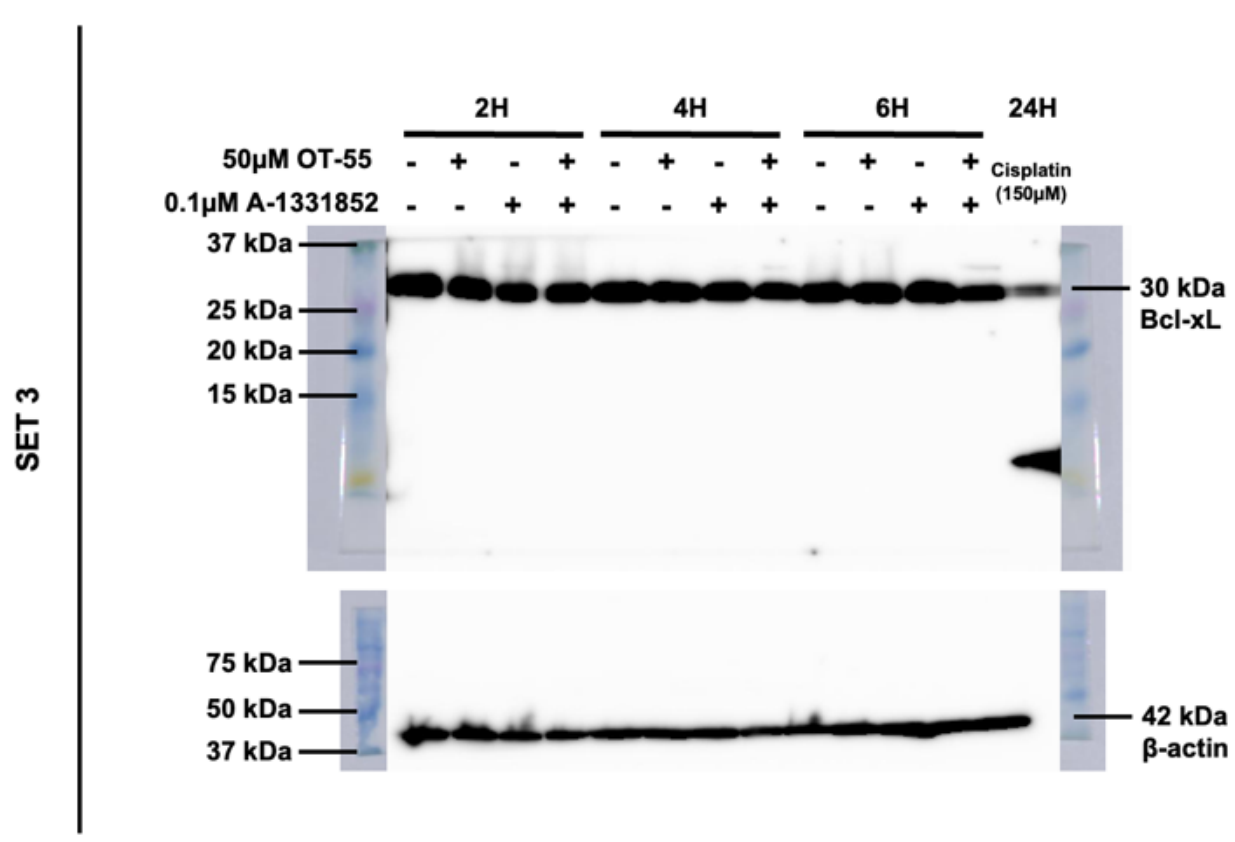

Supplemental Figure

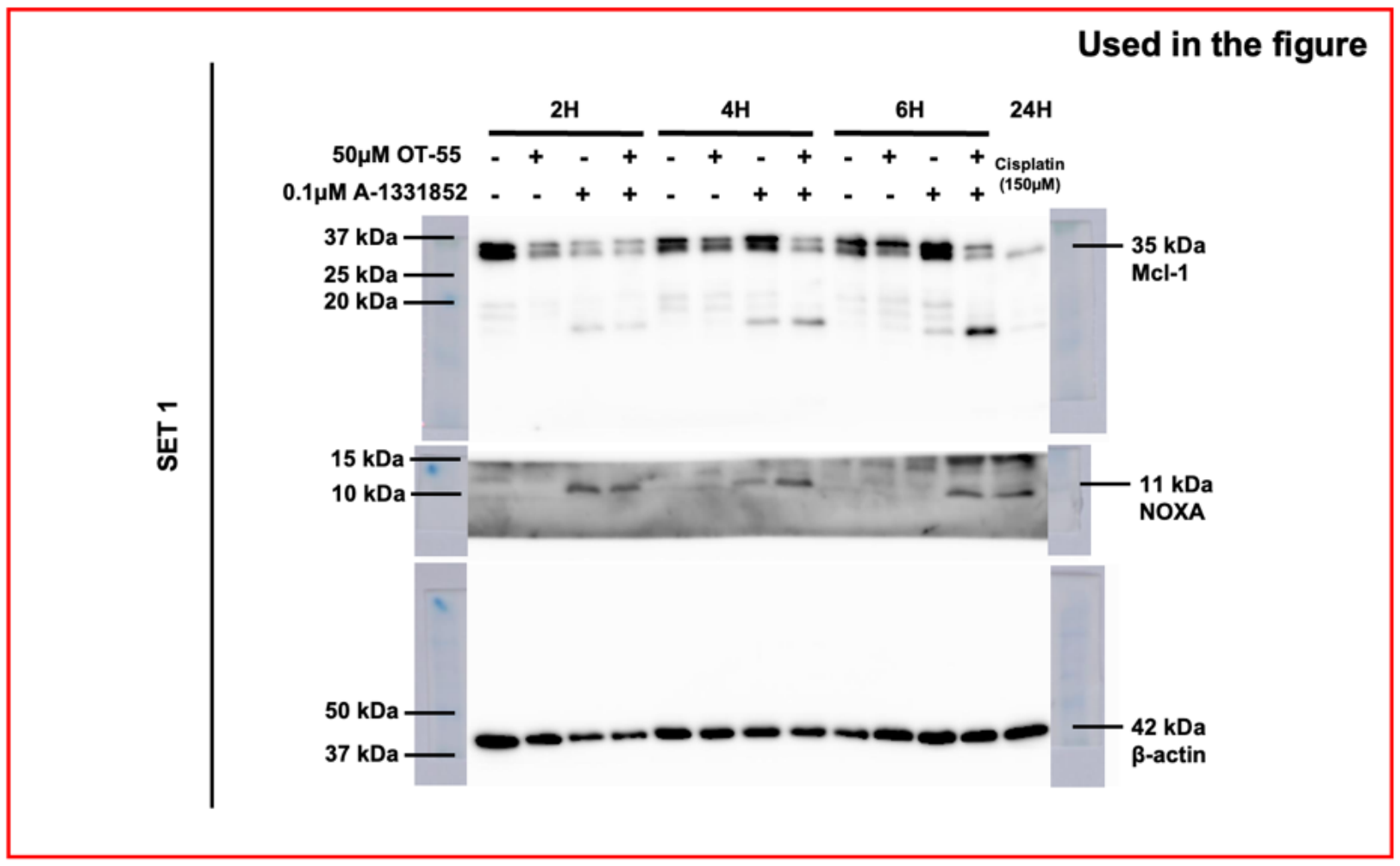

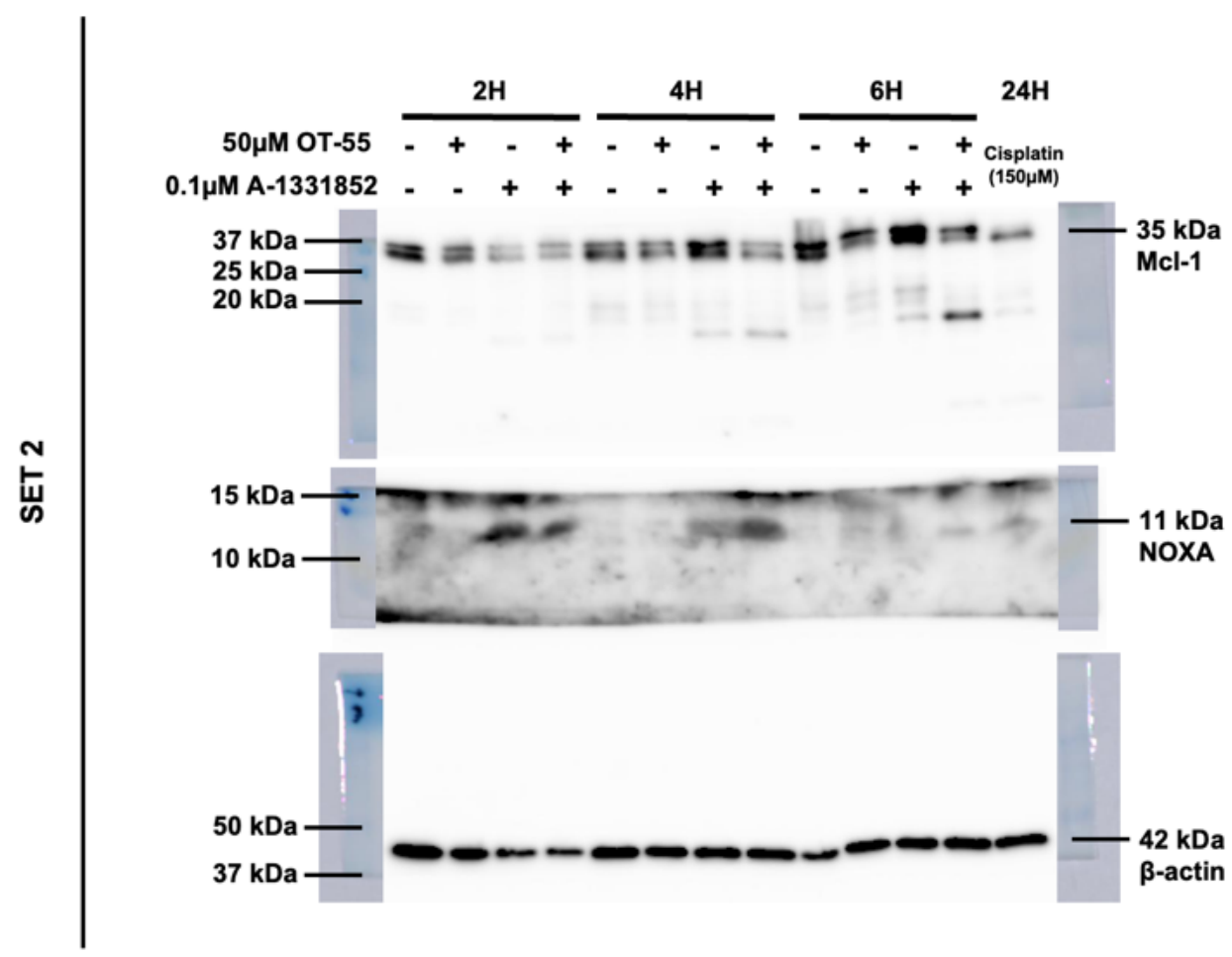

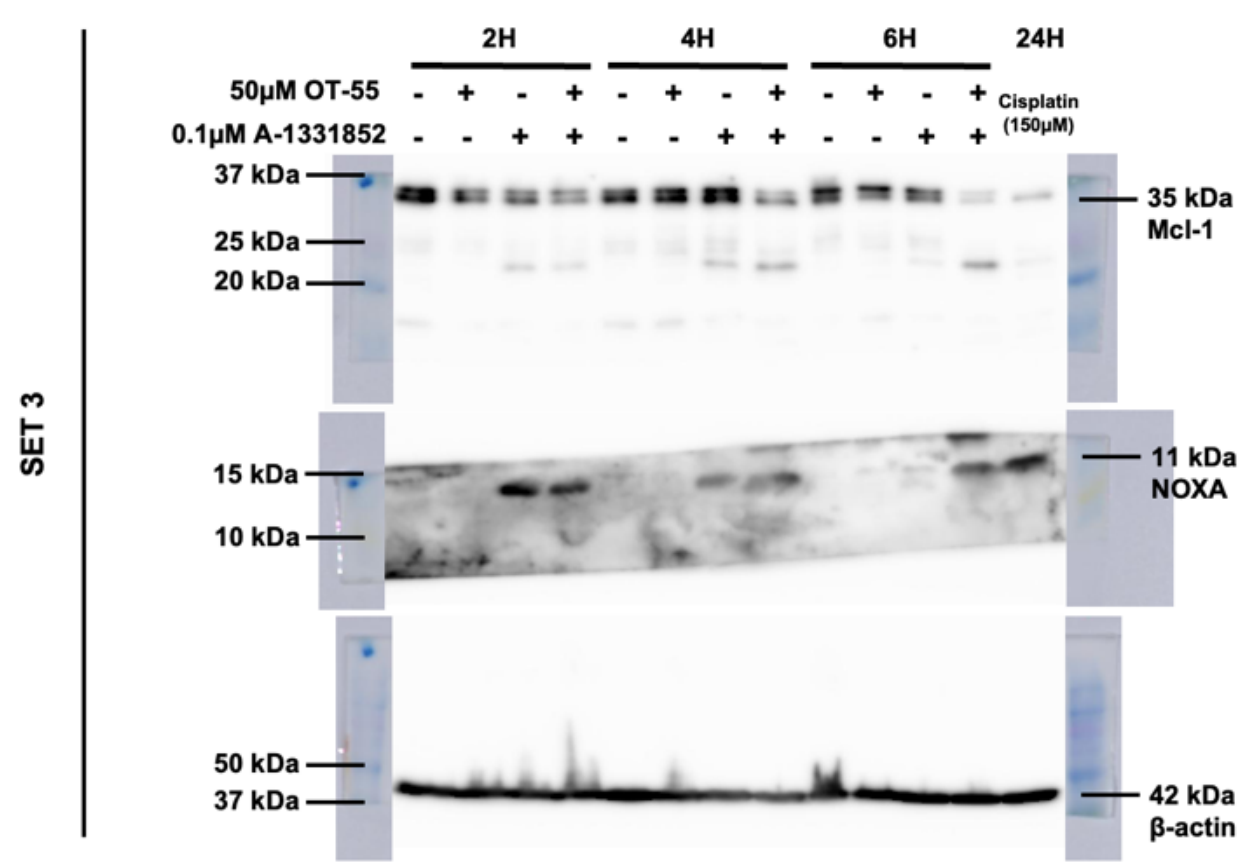

Supplemental Figure

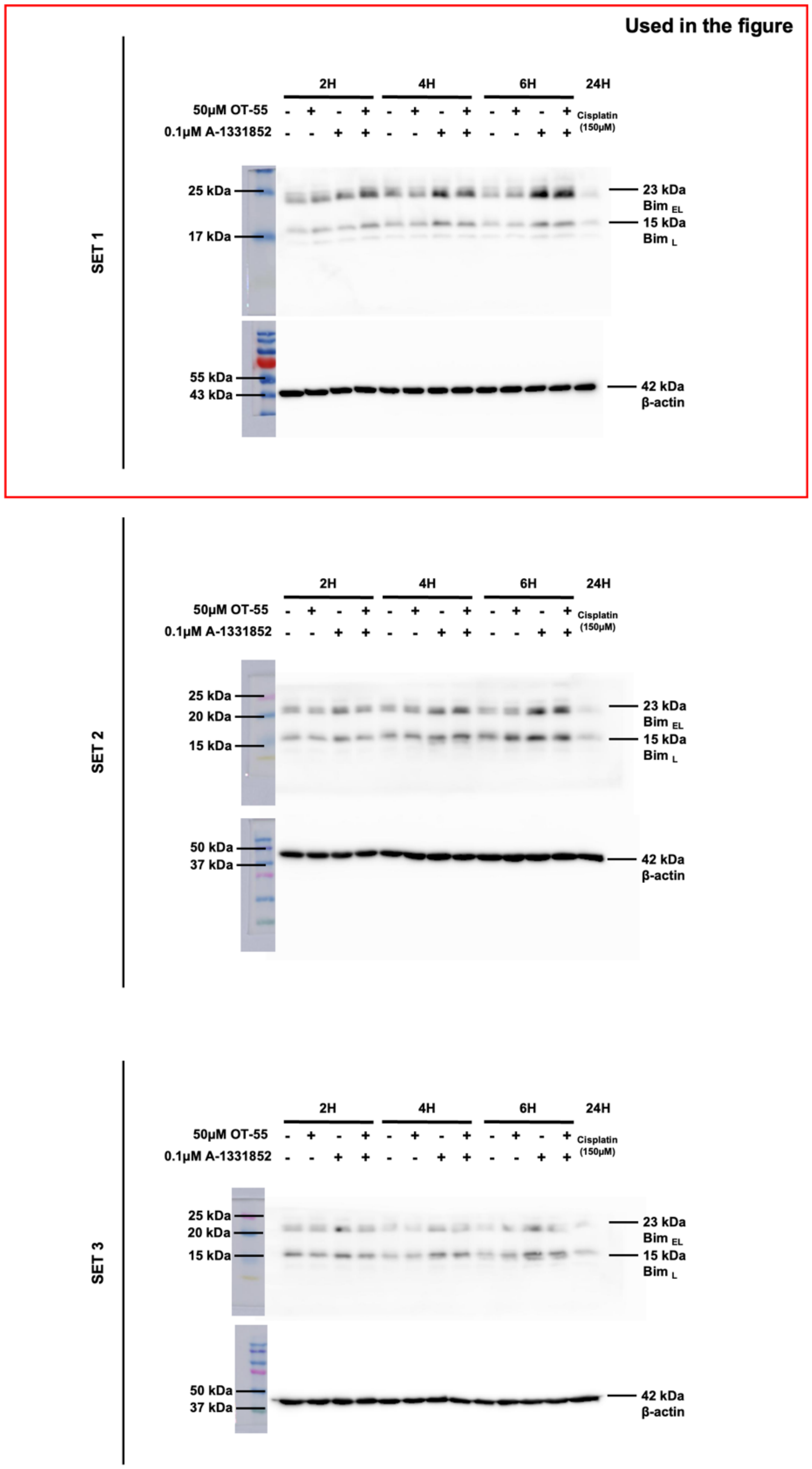

Supplemental Figure

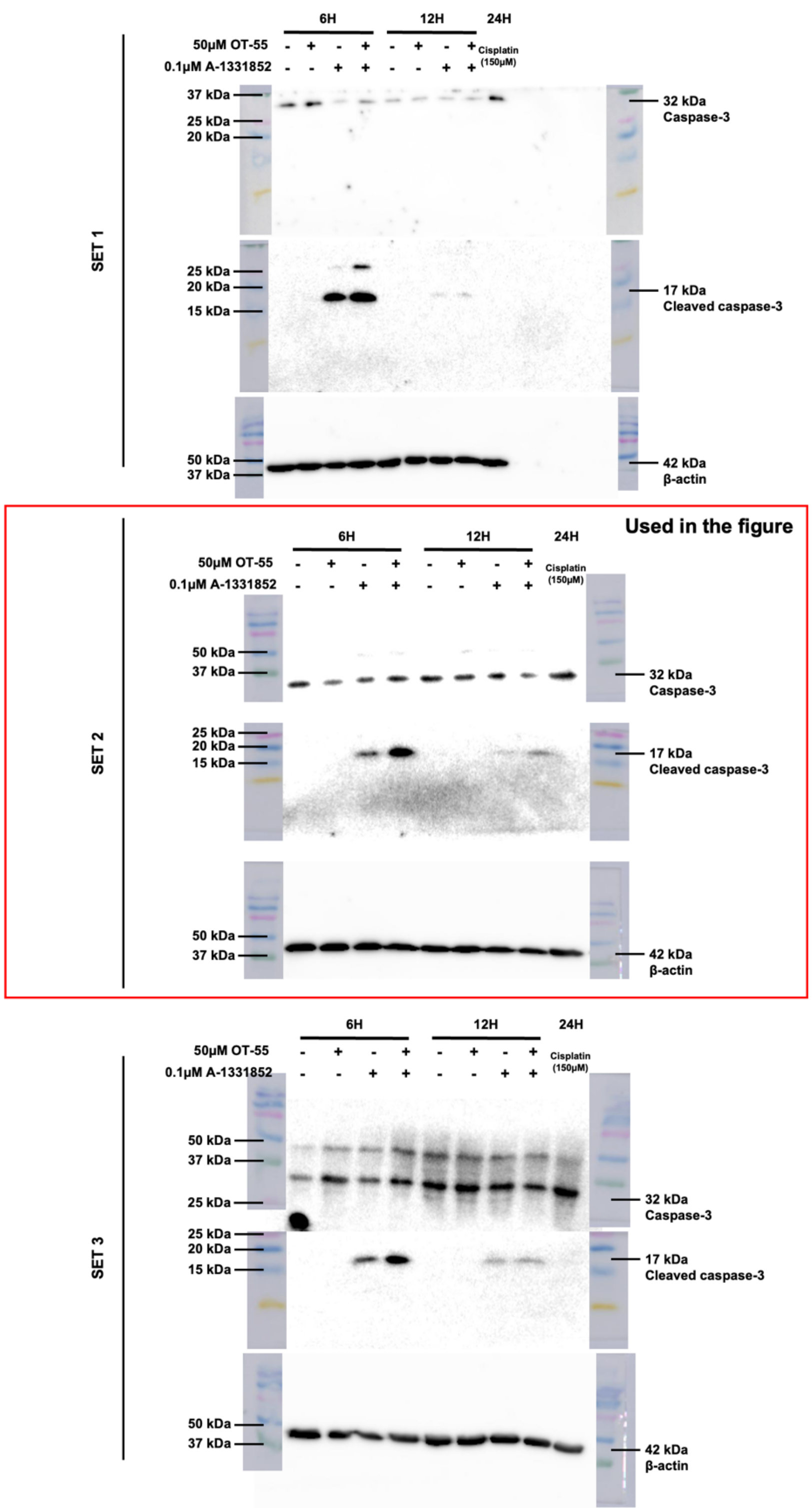

Supplement: Supplementary file 3 [file 41419_2026_9000_MOESM3_ESM.pdf]
